# Molecular mechanisms of processive glycoside hydrolases underline catalytic pragmatism

**DOI:** 10.1042/BST20230136

**Published:** 2023-06-02

**Authors:** Maria Hrmova, Julian G. Schwerdt

**Affiliations:** 1 School of Agriculture, Food and Wine, and Waite Research Institute, Faculty of Sciences, Engineering and Technology, University of Adelaide, Glen Osmond, South Australia 5064, Australia; 2 School of Life Science, Huaiyin Normal University, Huai'an 223300, China

**Keywords:** catalytic mechanisms, evolutionary relationships, GH3 family, large-scale phylogenomics, substrate-product assisted processivity

## Abstract

Processive and distributive catalysis defines the conversion continuum, thus underpinning the transformation of oligo- and polymeric substrates by enzymes. Distributive catalysis follows an association–transformation–dissociation pattern during the formation of enzyme–reactant complexes, whereas during processive catalysis, enzymes partner with substrates and complete multiple catalytic events before dissociation from an enzyme–substrate complex. Here, we focus on processive catalysis in glycoside hydrolases (GHs), which ensures efficient conversions of substrates with high precision, and has the advantage over distributive catalysis in efficiency. The work presented here examines a recent discovery of substrate-product-assisted processive catalysis in the GH3 family enzymes with enclosed pocket-shaped active sites. We detail how GH3 β-d-glucan glucohydrolases exploit a transiently formed lateral pocket for product displacement and reactants sliding (or translocation motion) through the catalytic site without dissociation, including movements during nanoscale binding/unbinding and sliding. The phylogenetic tree of putative 550 Archaean, bacterial, fungal, Viridiplantae, and Metazoan GH3 entries resolved seven lineages that corresponded to major substrate specificity groups. This analysis indicates that two tryptophan residues in plant β-d-glucan glucohydrolases that delineate the catalytic pocket, and infer broad specificity, high catalytic efficiency, and substrate-product-assisted processivity, have evolved through a complex evolutionary process, including horizontal transfer and neo-functionalisation. We conclude that the definition of thermodynamic and mechano-structural properties of processive enzymes is fundamentally important for theoretical and practical applications in bioengineering applicable in various biotechnologies.

## Introduction to processive and distributive catalysis

Enzymes that mediate the degradation or synthesis of polymeric substrates could use either processive (non-dissociative) or distributive (dissociative) catalysis. These transformative processes represent the edges of a catalytic continuum, although ‘semi-processive’ biocatalysts, encompassing processive and distributive features, are also known [1]. Processive catalysis, which requires a single association event, occurs when a catalyst associates with the substrate and accomplishes multiple catalytic rounds before dissociation from an enzyme–substrate complex. Conversely, distributive catalysis follows a typical enzyme-reactant association-transformation-dissociation pattern, meaning that a catalyst associates with a polymeric substrate, transforms it, dissociates from a substrate, and repeats this cycle [2,3]. During processive catalysis, the protein–substrate complex assembles sequentially, while through distributive catalysis this process proceeds in any order [4]. Through processive action, which could be viewed as a directional movement of enzymes along polymers, enzymes could be present at high linear densities, which may lead to reduced catalytic rates [5]. Most enzymes follow distributive catalysis, although processive catalysis is omnipresent in enzymes that degrade, synthesise, and modify biopolymers such as nucleic acids, proteins, and polysaccharides. It is assumed that processive catalysis is advantageous compared with distributive one. Processivity has been observed in nucleases [6,7], aminopeptidases [8], proteases [9,10], proteasomes [11], motor proteins moving along microtubules [12], actin filaments [13], glycosyltransferases [1,14–16], and exo- and endo-acting glycoside hydrolases (GHs) acting on homo- and heteropolymers [17–26].

## Methods of assaying processivity in GHs

There is no flawless approach to deciding whether GHs are processive or not. The most effective approach is to combine a variety of methods to profile reaction products and catalytic rates [27,28]. Targeted site-directed mutagenesis can also be used to examine changes in processive behaviour between wild-type and mutant enzymes, however, there is no convincing correlation between the activities of mutant enzymes and processivity [29]. Additional techniques use X-ray crystallography, 3D protein modelling, and molecular dynamic (MD) simulations, which with experimental data clarify if an enzyme is processive or not.

In cellulose and chitin biopolymers, successive disaccharide moieties are rotated by ∼180° meaning that after the polymer is threaded through the active site, only every second glycosidic bond of a polysaccharide could be hydrolysed. It is assumed that during the processive hydrolytic cleavage of cellulose and chitin, only dimeric products are released, while the shortened substrates continue sliding (or moving along using facilitated linear target allocation) in the enzyme active sites [3,30]. With insoluble cellulose, the processive exo-acting enzymes create more soluble reducing saccharides (the released dimers) than e.g. a distributive endo-acting (1,4)-β-d-glucanase, which will mostly generate insoluble saccharide reducing ends because the higher cellodextrin products have a lower solubility. This soluble-to-insoluble saccharide ratio assay can help to distinguish between processive and non-processive GHs (or estimate the degree of processivity), as any processive enzyme will produce a higher fraction of soluble saccharides when every initial hydrolytic step is followed by a repetitive release of a dimeric product [28]. In practice, processivity in hydrolases acting on cellulose is determined using e.g. a filter paper substrate [28,31,32] or regenerated amorphous cellulose [24]. Here, both the amount of soluble reducing saccharides that cellulolytic GHs produce from the filter paper (or another insoluble substrate) and the amount of insoluble reducing ends, are quantified. This is done by removing the filter paper at the end of incubation and measuring reducing saccharides in both the solution and washed filter paper, using reductometric, chromatographic, or electron [33] and high-speed atomic force microscopy approaches [34]. Alternative techniques include quantifying the hydrolysis of chemically labelled cellulose (at reducing ends) with, e.g. anthranilic acid, under ‘the single-hydrolytic step’ conditions [35]. In this case, processivity in the Cel7A cellobiohydrolase (CBH) from *Trichoderma reesei* was determined by calculating the ratio between the soluble fluorescent (released by initial cuts), and non-fluorescent (released by subsequent processive steps) products.

In chitinases, the diagnostic approach includes the measurement of hydrolytic product ratios that arise from higher oligosaccharides or polysaccharides. While mono- and trimeric hydrolytic products are indicative of initial binding and cleavage by the enzymes, dimeric products are primarily (but not solely) generated by processive catalysis, meaning that the ratio between these products will point to a degree of processivity. This method is considered to be semi-quantitative, as it leads to the overestimation of processivity, because the initial hydrolytic events are undervalued, and the intermediate reaction products are also not reflected upon [27]. However, these relatively simple approaches have limitations as the analysis based on the soluble-to-insoluble reducing saccharide ratios depends on prior knowledge of how a substrate is bound (i.e. via an endo- or exo-mechanism) and a type of substrate used [27]. In chitin**-**degrading GHs, processivity assessments use chitosan with a random distribution of acetylated groups and a chromatographic separation, elegantly demonstrated in *Serratia marcescens* chitinases [36]. Here, the diagnostic dominance of even-numbered products was observed during the early stages of reactions with chitinase A (SmChiA) and chitinase B (SmChiB) — an indication of processivity — as opposed to chitinase C (SmChiC), which is non-processive [17]. This quantitative approach could be combined with viscometry, and the estimations of total amounts of glycosidic linkages cleaved. However, to obtain a comprehensive view of the molecular basis of processivity, structural biology methods are preferred.

In the following sections, we will inspect the underlying molecular mechanisms of processive cellulose-degrading and chitin-degrading enzymes, GH74 xyloglucan-specific (1,4)-β-d-endoglucanases (XEGs), and GH3 exo-hydrolytic β-d-glucan glucohydrolases with tunnel-, saddle- and enclosed pocket-like shaped active sites, respectively.

## Processive GHs with tunnel-shaped active sites

**Figure 1.**
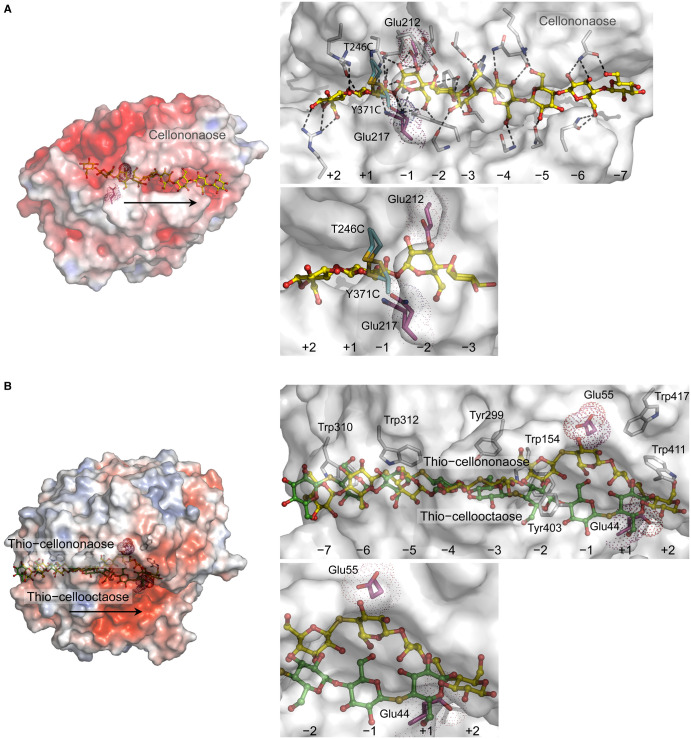
Structural properties of processive GH7 TrCel7A CBH from *Trichoderma reesei*, and GH48 Cel48F cellulase from *Clostridium cellulolyticum*. (**A**) *Left panel:* De-threading mechanism is favoured over the clamshell mechanism in exo-processive TrCel7A (PDB 4C4C) with a closed tunnel-like active site, where the catalytic domain binds cellononaose (yellow cpk ball and sticks). Disposition of catalytic Glu212 and Glu217 (magenta cpk sticks and dots), and a disulfide bridge between T246C and Y371C (cyan cpk sticks, positioned between the +1 and −1 subsites) are indicated. *Right panel top:* The T246C/Y371C mutant of TrCel7A (PDB 6RWF) superposed on WT TrCel7A [Root-Mean-Square Deviation (RMSD) value 0.23 Å]. Dashed lines indicate 2.6 Å to 3.5 Å separations between cellononaose and interacting residues. Modified from [23]. *Right panel bottom:* Detail of the top panel, illustrating the positions of T246C/Y371C residues. (**B**) *Left panel:* Superposition of E44Q (PDB 1G9J) and E55Q (PDB 2QNO) mutant structures of endo-processive Cel48F with a closed tunnel-like active site, in complex with respective thio-cellooctaose (cpk green ball and sticks) and thio-cellononaose (cpk yellow ball and sticks) (RMSD value 0.12 Å), illustrating the binding of substrates that slide during catalysis. Disposition of catalytic Glu55 and Glu44 (magenta cpk sticks and dots) is indicated. *Right panel:* Details of the binding of cello-oligosaccharides with residues (cpk sticks). Modified from [18]. *Right panel bottom:* Detail of the top panel, illustrating the positions of catalytic residues. Arrows, illustrate the forward movements of enzymes along carbohydrate chains during processive catalysis (left panels), and positions of subsites (right panels). Surface morphologies in left panels are coloured by electrostatic potentials: white, neutral; blue, +5 kT e^−1^; red, −5 kT e^−1^.

**Figure 2.**
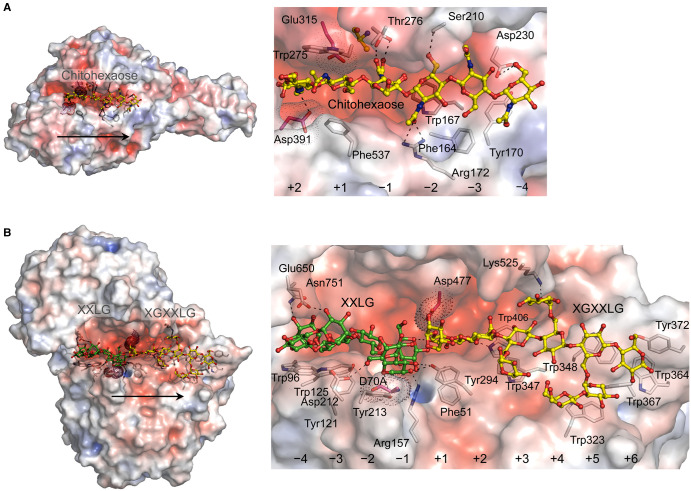
Structural properties of processive GH18 SmChiA from *Serratia marcescens* chitinase A, and GH74 PoXEG74-cat from *Paenibacillus odorifer*. (**A**) *Left panel:* SmChiA exo-processive chitinase A containing mutated D313A, K369M, F396A W359A, and E540M (PDB 5Z7M) in an open-tunnel active site in complex with chitohexaose (in an alternate conformation; yellow cpk ball and sticks). Disposition of catalytic Asp391 and Glu315 (magenta cpk sticks and dots) is indicated. *Right panel:* Details of chitohexaose binding in SmChiA (cpk sticks). Dashed lines indicate 2.6 Å to 3.1 Å separations between chitohexaose and interacting residues. Modified from [21]. (**B**) *Left panel:* Endo-processive PoXEG74-cat with mutated D70A in complex with XXLG (green sticks and balls) and XGXXLG (yellow cpk sticks and balls) in a saddle-shaped active site (PDB 6MGL). Disposition of catalytic D70A and Asp477 (magenta cpk sticks and dots) is indicated. *Right panel:* Details of XXLG and XGXXLG binding in PoXEG74-cat (cpk sticks). Dashed lines indicate 2.5 Å to 3.2 Å separations between oligosaccharides and interacting residues. Modified from [52]. The significance of arrows (left panels), positions of subsites, and surface morphologies are described in the legend of Figure 1.

Processive polysaccharide hydrolases with tunnel-, cleft-, or groove-shaped active sites were described in cellulolytic exo- and endo-acting enzymes, which are classified by Carbohydrate-Active enZymes database (http://www.cazy.org/; [37]) into GH6 [38], GH7 [23,39] (Figure 1A), GH5 [24], and GH48 [18] (Figure 1B) families. Exo-acting chitinases, are catalogued in the GH18 family [17,21,40] (Figure 2A), whereas GH19 chitinases are deemed to be distributive [20]. In processive GHs, the mechanism by which substrates attach to enzymes categorises the two fundamental groups where the substrate is: (i) completely or nearly completely encircled by protein folds (Figure 1), or (ii) enclosed in open or partly open tunnels or saddles (Figure 2). With crystalline cellulose or chitin, processivity is thought to be advantageous for hydrolysis, because the detached single-polymeric chains are prevented from re-associating together [17]. The latter authors showed that processivity comes at high costs in terms of catalytic rates and that from a biotechnology perspective, it is more beneficial to focus on improving substrate accessibility for non-processive enzymes than improving the properties of processive ones.

### Cellulose processive exo- and endo-hydrolytic GHs

We focus on the molecular mechanism of GH7 *Trichoderma reesei* Cel7A CBH [23], which is dependent on the strong affinity of the +1 and +2 product binding subsites that seems to drive processivity [39]. For this enzyme, the Hamiltonian replica-exchange umbrella sampling MD approach was used [23] (Figure 1A) to discuss two opposing de-threading (reversing along the cellulose chain until the enzyme is non-complexed) and clamshell (substrate-enclosing loops open and release the cellulose chain without reversing) mechanisms of substrate binding. The authors calculated catalytic rates and established that the de-threading model, operating from the binding tunnel, was about four orders of magnitude faster than the clamshell model, where substrate-enclosing loops opened and released the substrate without reversing. The X-ray crystallographic Cel7A-cellononaose complexes of wild-type (WT) (Figure 1A, left panel) and the T246C/Y371C disulfide variant with covalently linked substrate-enclosing loops (Figure 1A, right panel) indicate that the T246C and Y371C mutations, located near the +1 and −1 subsites, ceased the mutant's ability to open loops, and caused the enzyme to dissociate via stepwise de-threading. This work demonstrated that the dissociation *k*_off_ rate constant of T246C/Y371C with cellononaose was equivalent to that of WT, implying that de-threading was a dominant (and thermodynamically favoured) model for the processive mechanism. This model fulfilled the Sabatier principle of chemical catalysis, where optimal catalysis occurs when the catalyst-substrate interactions are of intermediary strength [41]. It was also proposed that the two models may overlap, where a cellulose chain could partially de-thread and later unbind via loop unsealing [23]. To understand the mode of action of processive Cel7D CBH in a broader context, it was proposed that its activity could be significantly increased by the addition of a lytic polysaccharide monooxygenase that amorphise crystalline cellulose surfaces, which in turn causes Cel7D to move along the substrate surfaces in more enzyme attached [42].

Next, processivity was examined in the Cel48F polysaccharide hydrolase from *Clostridium cellulolyticum* (GH48), which could be regarded as a prototypical example of an endo-processive enzyme type [18]. Crystallographic data pointed at a two-step mechanism, in which the processive sliding of cellononaose and its cleavage engaged in dissimilar subsites and pathways (Figure 1B). This mechanism is based on the catalytic residue mutants E44Q and E55Q in complex with thio-cellooctaose and thio-cellononaose, respectively (Figure 1B), where these mutations prevented substrate hydrolysis [18]. In the first step, the chain slides up to the leaving (aglycon) group site alongside the first pathway (Figure 1B, right panel; thio-cellooctaose in cpk green sticks), while in the second step, the thio-cellononaose chain adopts a second trajectory in a productive binding mode (Figure 1B, right panel; thio-cellooctaose in cpk yellow sticks), before the substrate could be hydrolysed. Both pathways, one being intermediary, are stabilised by up to seven face-to-face stacking interactions and hydrogen bonds through Trp154, Tyr299, Trp310, Trp403, Trp411, and Trp417 side chains (Figure 1B, right panel). The two separate pathways, in which the interacting residues re-orient, aim to maintain low-energy sliding to reach the −1 to +2 subsites at the substrate-reducing end. Pathways that use thio-analogues demonstrated that endo-processive Cel48F reduces the sliding energy of a chain by adopting two alternate positions. This suggested that sliding of the substrate by one glucose (Glc) moiety leads to non-productive binding, which is disfavoured thermodynamically, as it evokes the re-orientation of side chains.

Special attention was paid to enzyme synergy or cooperative action during cellulose hydrolysis between internally chain-cleaving endo-acting enzymes and end-chain-cleaving exo-acting enzymes that proceed in a processive mode in multilayers [26]. Here, it was demonstrated, based on a single-molecular study via real-time atomic force microscopy, that during cellulose depolymerisation, endo- and exo-acting cellulases engage in the formation of transient clusters of up to four enzymes at the cellulose surface [26]. This leads to cellulose fibre deconstruction, which is dominated by processive steps of fibril dismantling in multilayers, promoted by enzyme clusters. The authors determined that during this endo-exo synergy, endo-acting enzymes prepare substrates for exo-acting enzymes, which together could pass through processive cycles ∼100-fold faster than when acting alone. This synergy represents a paradigm for efficient interfacial catalysis during processive hydrolysis of structurally organised polymers and is significant in the development of industrially important biomaterials.

### Chitin processive exo-hydrolytic GHs

Close attention was paid to processive chitin exo-hydrolytic GHs [20,27,40], in particular to the mechanism of reducing-end exo-processive *Serratia marcescens* SmChiA of the GH18 family [21] (Figure 2A). Other *Serratia marcescens* exo-processive SmChiB [43], distributive endo-acting SmChiC [44], and an endo-processive chitotriosidase (a part of the innate immune system of humans) [45,46] contain carbohydrate-binding modules (CBM) connected to catalytic domains through flexible hinge regions. However, the precise role of CBMs is partially obscure. It was recently shown that the processive Cel9G endo-β-1,4-glucanase from *Clostridium cellulovorans* requires a four-step catalytic cycle (hydrolysis, product release, processive binding, catalytic activation), with aromatic residues in CBM assisting processivity through anchor points [47]. Nevertheless, the possibility remains that these CBM modules participate in substrate binding, specificity, and thermostability.

The combination of X-ray crystallography, single-chain imaging, all-atom MD simulations, and the use of mutants showed SmChiA as a processive enzyme that operates in the form of a linear molecular motor (Brownian monorail) moving on the surface of a crystalline single-chitin chain [48]. Correspondingly, to the processive exo- or endo-hydrolytic cellulases, aromatic residues (Phe164, Trp167, Tyr170, and Phe537) participated via face-to-face stacking interactions and hydrophilic residues (Arg172, Ser210 Thr276, and Asp230) via hydrogen bonds, to ensure chitohexaose substrate binding, sliding and processive hydrolysis (Figure 2A, right panel). The latter authors showed that the de-crystallisation of a single-chitin chain is a rate-limiting step during SmChiA movement along the chain. Furthermore, a much larger forward-step ratio, compared with the backward ratio, occurred during hydrolysis and was explained by the competition between substrate-assisted catalysis and the backward motion of a chain; this suggested the utilisation of a forward unidirectional Brownian motion. As for the hydrolytic mechanism of SmChiA, it uses the so-called substrate-assisted catalysis, where an N-acetyl group of chitin cleaves a glycosidic bond without activating a water molecule, followed by a water molecule approaching the active site, and the oxazoline intermediate being hydrolysed [49].

## Processive GHs with groove-shaped active sites

### Xyloglucan (XG) processive endo-hydrolytic GHs

These polysaccharide hydrolases encompass processive xyloglucan-specific endo-β-d-1,4-glucanases (XEGs) and are classified as members of the GH74 family [50–53]. Dual seven-fold β-propeller scaffolds, approximately in the middle of their structures, enclose long and wide groove (saddle)-shaped active sites that accommodate XG chains (Figure 2B; PDB 6MGL). The mode of action of endo-processive XEGs and their division from the endo-dissociative (*Geotrichum* sp. M128) XEGs, is based on the viscosity rate reduction in XGs and the release of low-molecular-mass XG-oligosaccharides at early stages of hydrolysis (which continue to accumulate) — these characteristics are absent from endo-dissociative XEGs [50–53]. Some processive GH74 XEGs, such as those from *Paenibacillus* sp. strain KM21, *Paenibacillus odorifer*, *Niastella koreensis,* and *Caldicellulosiruptor lactoaceticus* house up to ten subsites in their active sites. These sites are located on a large hydrophobic platform shielding numerous aromatic residues (with mainly tryptophan and tyrosine side chains), which bind glucosyl and xylosyl moieties of XGs, and facilitate processivity [50,52,53]. More specifically, it was shown in bacterial *Paenibacillus* sp. strain KM21 XEG74 through mutagenesis that Trp318 and Trp319 located in positive subsites were responsible for the enzyme's directionality along an XG chain and thus processivity. The replacements of these residues by alanine residues shifted an endo-processive to an endo-dissociative mode of action. Conversely, mutations of Trp61 and Trp64 in the negative subsites of the *Paenibacillus* sp. strain KM21 XEG74 enzyme maintained the efficiency of XG binding and the release of XG oligosaccharide products [50].

Studies of processive GH74 XEGs were extended by the analyses of substrate specificity, kinetics, hydrolytic product profiling, and protein structure in the bacterial *Paenibacillus odorifer* XEG74 catalytic module (PoXEG74-cat) in complex with XG fragments (Figure 2B). These studies showed that PoXEG74-cat had a highly specific endo-processive mode of action and that it hydrolysed branched and unbranched XGs. Site-directed mutagenesis of hydrophobic platform residues, located in the groove of the PoXEG74-cat scaffold at the −4 to +6 subsites, indicated that the conversions of Tyr372, Trp347, Trp348 and Trp406 to alanine residues at the +6, +3, +5, and +3 subsites, respectively, affected processivity and that Trp347 and Trp348 were required. Further analyses of bacterial and fungal XEG74 enzymes provided details on the molecular phylogeny of the GH74 family (sub-divided into groups 1 to 5) and delineated the structure-function relationships of ancestral bacterial *Simiduia agarivorans* GH74, and those of 2- and 5-XEG groups along evolutionary trajectories [53].

## Processive GHs with enclosed pocket-shaped active sites

### Exo-acting β-d-glucan glucanohydrolases and substrate-product-assisted processive catalysis

As referred to above, processivity or distributive catalysis is linked to certain GHs with tunnel-, cleft- or groove-shaped active sites lined with the succession of bulky aromatic residues that mediate the sliding of polysaccharides through enzyme active sites without dissociation. Conversely, exo-acting GH3 β-d-glucan glucanohydrolases [54] and β-d-glucosidases [55] with enclosed pocket-, funnel-, and crater-shaped active sites are deemed to be distributive, as these active sites feature dead ends, i.e. they are completely enclosed from one side [56].

In endo- or exo-acting cellulose- and chitin-hydrolytic GHs, processive catalysis is closely linked to the chemical structure of polymeric substrates, and the formation of productive substrate–enzyme complexes for substrate sliding. However, processive catalysis was surprisingly identified also in exo-acting GH3 enzymes, which do not feature tunnel-, cleft-, or groove-shaped active sites. These enzymes characteristically possess an enclosed pocket-shaped active site [22,25,57,58], and this type of processive catalysis, discovered in barley β-d-glucan glucohydrolase HvExoI, was termed ‘substrate-product-assisted processive catalysis’ [22]. So, how could this occur, when the enclosed pocket in HvExoI generates a physical barrier that precludes the forward threading of substrates through its catalytic site? Considering that HvExoI operates on plant cell wall β-d-glucans, processive catalysis could be expected, as rapid substrate hydrolysis is critical to embryo development during seed germination and other physiological processes [58,59]. We have recently shown [22,25], based on crystallographic and multi-scale MD simulations, that the key role in this mechanism was played by the Glc product, and how it is released from the non-reducing termini of oligo- and polymeric β-d-glucosides by an exo-hydrolytic mechanism.

**Figure 3.**
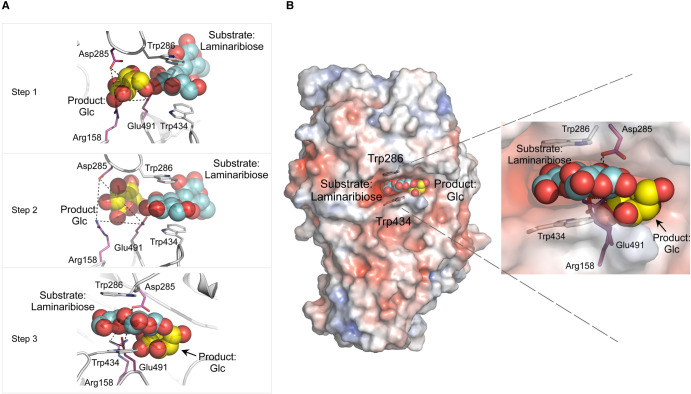
Structural basis of substrate-product-assisted processive catalysis in GH3 β-d-glucan glucohydrolase HvExoI from *Hordeum vulgare*, L. (**A**) Selected steps 1–3 along the Glc displacement trajectory based on enzyme-ligand complexes calculated by GPathFinder. Laminaribiose substrate and Glc product — carbons in respective cyan and yellow cpk sticks and spheres — are bound in the active site. *Top panel (step 1):* Glc (yellow cpk sticks and spheres) is in the −1 subsite; incoming laminaribiose (cyan cpk sticks and sphered) is in the +1 subsite and bulk solvent. *Middle panel (step 2):* Movement of the Arg158–Asp285–Glu491 toll-gate (dashed lines) allows Glc to traverse into the lateral cavity, which is exposed to bulk solvent. *Bottom panel (step 3):* Glc displacement proceeds through the transiently formed aperture in the lateral cavity (arrow). (**B**) *Left and right images:* The substrate (cyan spheres) slides into the active site and locates in the +1 subsite and bulk solvent. After hydrolysis, the Glc product (yellow spheres) egresses from the −1 subsite through the lateral cavity into bulk solvent (arrow). Protein surface morphology is coloured by electrostatic potentials: white, neutral; blue, +5 kT e^−1^; red, −5 kT e^−1^. Modified from [22].

**Figure 4.**
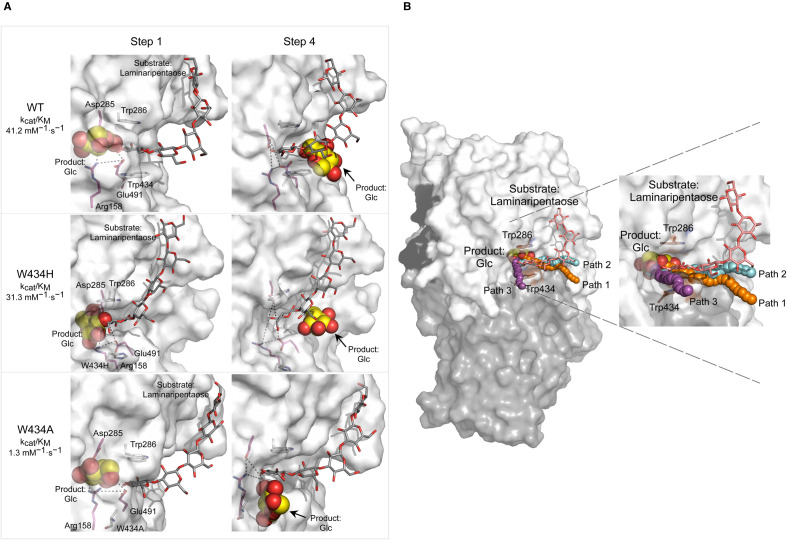
Glc displacement routes in WT, and W434H and W434A mutants of GH3 β-d-glucan glucohydrolases during substrate-product-assisted processive catalysis. (**A**) Selected steps 1 and 4 along Glc displacement routes in wild-type (WT) (top), and W434H (middle) and W434A (bottom) mutants that were calculated by GPathFinder using converged enzyme-ligand complexes with Glc (carbons in cpk yellow spheres in the −1 subsite) and laminaripentaose (carbons in cpk dark-grey sticks in the +1 and +2 subsites and bulk solvent). The Arg158–Asp285–Glu491 (cpk magenta sticks) toll-like barrier is indicated in dashed lines. Dispositions of egressed Glc are indicated by an arrow. Values of *k*_cat_/*K*_M_ for laminaribiose in WT, W434H, and W434A are indicated [25]. (**B**) *Left and right images:* Glc displacement path 1 (in WT and W434H), path 2 (in W434A), and path 3 (in WT, when the ligand is absent from the +1 and +2 subsites) are depicted in orange, cyan, and magenta aligned spheres, respectively. Dispositions of Trp286 and Trp434 (brown cpk sticks) delineating the +1 subsite in WT, and its domains (domain 1, domain 2, and linker in white, grey, and black, respectively), and surface morphology are indicated. Modified from [25].

The structure of HvExoI folds into an (α/β)_8_ barrel (domain 1) and an (α/β)_6_ sandwich (domain 2) that at their interface house an enclosed 13 Å-deep pocket- or crater-shaped active site. This pocket accommodates catalytic Asp285 (nucleophile) and Glu491 (acid/base) residues in the −1 subsite, which in these stereo-chemistry retaining enzymes predominantly provide an anionic environment for the oxocarbenium ion-transition state upon proton transfer [60]. Conversely, the aromatic clamp, composed of the indole moieties of Trp286 and Trp434 delineates the neighbouring +1 subsite (Figures 3 and 4), is fundamentally important for broad substrate specificity, catalytic efficiency, and processivity, as insofar it binds isomeric (1,2)-, (1,3)-, (1,4)- and (1,6)-linked β-d-oligosaccharides [57,58,61,62]. It was further revealed using WT and mutant HvExoI that the processive forward motion of substrates could only be adopted during the hydrolysis of (1,3)-linked substrates, as the Glc moieties would not require rotation during sliding motion, and could be threaded uninterruptedly through the active site [25].

Additional structural analyses of HvExoI combined with multi-scale MD modelling of reactant movements at the nanoscale revealed how each hydrolytic event, including the Glc product displacement route, is coordinated with the incoming substrate association and hydrolysis. This modelling included MD and Gaussian accelerated MD [63], Protein Energy Landscape Exploration (PELE) [64], and GPathFinder [65]. To explain, how these multi-scale modelling tools work, PELE performs local perturbations, senses global motions, and on millisecond time scales captures relevant protein motions, while GPathFinder explores dynamic traits of reactants binding or unbinding, via backbone motions and sidechain optimisations of discrete rotameric states. Using these approaches, it became clear that upon the productive binding of incoming substrates with various glycosidic linkages and subsequent hydrolysis, the released Glc hydrolytic product could alter its binding patterns and instigates the formation of a lateral cavity with a transient life-span (Figure 3). During this process, a series of steps during the Glc displacement event, allow for the next catalytic round (Figure 3, steps 1–3). Such a path enables substrate-product-assisted processive catalysis, which is dictated by the nature of substrates, and where multiple hydrolytic events could take place without HvExoI de-contacting saccharide substrates. These comparative analyses of the structural basis of motion (https://figshare.com/s/28faa2510cb6019a1a8e) suggest that the observed enzyme plasticity may not be restricted to GH3 hydrolases, but could have a broader prevalence among enzymes with enclosed pocket-shaped active sites [25]. Substrate-product-assisted processivity in GH3 enzymes [22,25] could be linked to a ‘multiple attack mechanism’ [66] described in glucoamylases (EC 3.2.1.3); the latter enzymes release the (1,4)-linked α-d-glucosyl residues from non-reducing ends of chains with retention of anomeric configuration. In this sense, mechanistic features in substrate-product-assisted and multiple-attacking enzymes are similar, in that they are non-dissociative, and differ in the degree of continuity or processivity. Active sites of glucoamylases have a similar geometry to pocket-like GH3 hydrolases, with a range of aromatic residues participating in substrate binding [67]. Hence, GH3 enzymes are more similar to glucoamylases than cellulases or chitinases, as they interact with substrates without the requirement of the typical tunnel-, cleft-, saddle-, or groove-shaped active sites.

### Mutations in the β-d-glucan glucanohydrolase (HvExoI) alter substrate-product-assisted processivity

Additional studies were conducted using high-resolution X-ray crystallography, and multi-scale MD simulations with HvExoI mutants [25], where single (and in some instances double) Ala, His, Phe, and Tyr alterations were introduced into the Trp286/Trp434 clamp of HvExoI. Investigations of pre-steady state kinetics and conformational behaviour during the binding of isomeric β-d-glucosides revealed that the W434H mutant retained broad substrate specificity, while W434A behaved as a strict (1,3)-β-d-glucosidase. These findings contrasted with the examinations of the W286F, W286Y, W434F, and W434Y single, and W286F/W434F, and W286F/W434A double mutants, which were not active with at least one substrate. Furthermore, the binding of a series of (1,2)-, (1,3)-, (1,4)- and (1,6)-linked β-d-oligo- and polysaccharides and aryl-glycosides showed that W434A and W434H and other mutants also exhibited lowered *K*_i_ dissociation constants with positionally isomeric (1,2)-, (1,3)-, (1,4)-, and (1,6)-β-d-linked thio-analogues [25]. The high-resolution structures of HvExoI-thio-analogue complexes revealed that in the W434A mutant the reducing-end moiety of (1,6)-β-d-linked thio-analogue was rotated by ∼120° (via x-axis), compared with the disposition in WT, which was presumably due to the presence of a rotatable C1-O-C6 bond. These data supported by enzyme kinetics and the conformational behaviour of S- and O-linked β-d-glucosides, indicate that the tryptophan residue mutations in the aromatic clamp impact the accurate orientations of bound β-d-glucosides that are required for the formation of productive enzyme–substrate complexes [25].

Additional investigations of reactant trajectories via MD simulations in HvExoI showed that natural substrates, such as β-d-gluco-trisaccharide with 1,3- and 1,4-glycosidic linkages [originating from (1,3;1,4)-β-d-glucans] [58], cannot slide through the active site. This process is only reserved for substrates with (1,3)-β-d-linked Glc moieties, as referred to above. Notably, the binding and hydrolysis of the mixed-linkage β-d-gluco-trisaccharide progress in two steps, the hydrolytic Glc product cleavage from the substrate's non-reducing terminus to yield (1,3)-linked disaccharide, followed by Glc egressing through a lateral cavity. In the second step, the (1,3)-linked disaccharide product needs to diffuse out of the active site and re-rebind in a correct orientation to form the productive complex, followed by hydrolysis [25]. Cluster analysis of the exact position of the (1,3)-linked disaccharide in the active site revealed that, in the WT HvExoI, ∼96% of substrate populations locate in the +1 and +2 subsites, and ∼4% to the intermediary (or midway) pose at the −1 and +1 subsites. Conversely, in W434A the (1,3)-linked disaccharide product showed a striking shift in poses to ∼15% of the substrate populations located in the +1 and +2 subsites, and ∼74% to the intermediary positions at the −1 and +1 subsites. These findings reiterated that Trp434 plays a fundamental role in precise orientation and thus sliding of (1,3)-β-d-linked substrates through the catalytic site, and prevents (1,3;1,4)-β-d-glucan-derived substrates from sliding. In this context, the question arises if the processive mechanism of HvExoI with these substrates could be engineered via single or multiple mutagenesis interventions, a proposal that can be tested via molecular modelling prior to experimental evaluations.

Finally, molecular modelling using cMD, GaMD, and GPathFinder of HvExoI W434A and W434H mutants revealed that processivity was sensitive to mutations of the Trp clamp. These mutants were selected based on the hydrolytic rates of poly- and oligosaccharide substrates, where W434H either retained or W434A lost broad substrate specificity [25]. In this case, WT and W434H utilised the lateral cavity for Glc displacement and sliding of (1,3)-linked substrates, such as laminaripentaose, through the catalytic site without dissociation (path 1) (Figure 4A, top, and middle panels). This was consistent with the high hydrolytic rates of WT HvExoI with (1,3)-β-d-glucosides (Figure 4A) compared with those of W434H and W434A, and showed that the processive forward movement of substrates and Glc displacement are linked, i.e. they form a structural continuum. Conversely, processivity in W434A was disturbed, and the Glc product egressed through the W434A-Trp286 clamp (path 2) utilising a gap formed from the W434A mutation (Figure 4A, bottom panel). Thus, W434A did not engage in substrate-product-assisted processivity. The work also unravelled path 3 (vertical) in the WT, W434H, and W434A mutants for Glc displacement, when substrates were absent from the +1 and +2 subsites. Here, Glc displacement proceeded through the space vacated by substrates and exited via the Trp286/Trp434 aromatic clamp (and +2 subsite) into a bulk solvent. These Glc product displacement trajectories 1–3 (Figure 4B), and molecular motions (https://figshare.com/s/faf4d4d8c582ed0d7184) suggested that a variety of Glc egress trajectories could be adopted by HvExoI. This could have significant consequences for the engineering of substrate specificity and catalytic rates.

## Evolutionary relationships in the GH3 family

**Figure 5.**
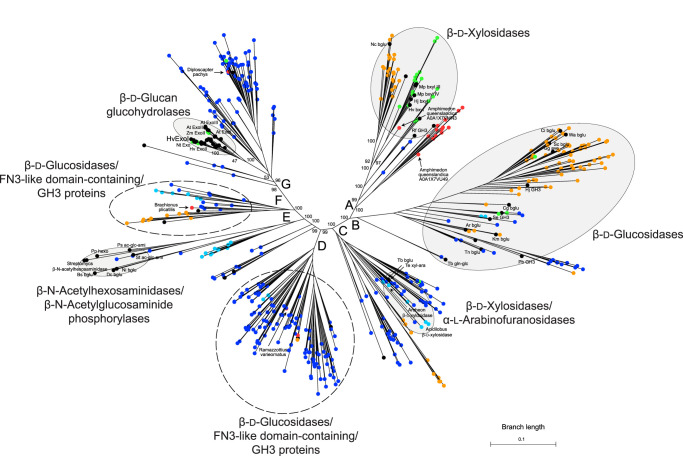
IQ-TREE tree [68] of 550 GH3 entries across Archaea, bacteria, fungi, Viridiplantae, Metazoa (Animalia), and other eukaryotic protists. The tree reveals seven major clades (grey ovals) with the following enzyme activities: β-d-glucan glucohydrolases (EC 3.2.1.-), β-d-glucosidases (EC 3.2.1.21), β-d-xylosidases (EC 3.2.1.37), non-specific β-N-acetylhexosaminidases (EC 3.2.1.52)/β-N-acetylglucosaminide phosphorylases (EC 2.4.1.-), and non-specific β-d-xylosidases/α-l-arabinofuranosidases (EC 3.2.1.55). Two clades containing unannotated β-d-glucosidases/FN3 (Fibronectin 3)-like domain-containing/GH3 proteins (dashed circles) form clades with unknown substrate specificity. In β-d-glucan glucohydrolases, at least three sub-clades were identified with residue variability in an aromatic clamp of Trp286 and Trp434 (numbering corresponds to HvExoI labelled in large types). Annotated entries listed in [25] (*cf*. Figure 1 therein) are indicated by black circles, and Archaean, bacterial, fungal, Viridiplantae, and Metazoan entries are in cyan, blue, orange, green, and red circles, respectively. Five selected Metazoan entries are indicated with arrows. The percentage of replicate trees with taxa clustering together in the bootstrap test (1000 replicates) are shown at selected major deep node bipartitions. Major nodes A–G and branch length (substitutions per site) are indicated. Method descriptions, the list of sequences, and the FASTA alignment used to generate the IQ-TREE tree are included in the Supplementary Dataset.

Large-scale phylogenomics was performed to investigate the evolutionary relationships in the GH3 family and to resolve the precise evolution of β-d-glucan glucanohydrolases (Figure 5 and Supplementary Dataset). The IQ-TREE best-known maximum likelihood tree [68] of 550 GH3 protein sequences resolved seven well-defined (>98%) major nodes (Figure 5, lineages labelled A–G). These clades correspond to major substrate specificity groups [25,38,69], as determined by the presence of sequences from annotated and experimentally determined protein structures included in the data. Four of these seven groups were classified unequivocally — according to the presence of experimentally determined crystal structures — β-d-xylosidases, β-d-glucosidases, β-d-glucan glucohydrolases, and β-N-acetylhexosaminidase/β-N-acetylglucosaminide phosphorylases. Further two clades were classified as non-exclusive β-d-xylosidase/α-l-arabinofuranosidase and β-N-acetylhexosaminidase/β-N-acetylglucosaminide phosphorylase entries. The A and B clades with β-d-glucosidase and β-d-xylosidase substrate specificities are primarily composed of eukaryotic sequences, whereas most of the variation in other lineages (C, D, E, F, and G) is accounted for by bacteria (Supplementary Dataset). Node C represents a major division between bacterial and eukaryotic lineages, both of which have β-d-glucosidase and β-d-xylosidase specificity. Well-resolved evolutionary branches lead from major nodes D, E, and F, except for two clades of primarily bacterial β-d-glucosidases/FN3-like domain-containing/GH3 proteins, which were short in length. Conversely, the tip branches of the former clades were comparatively long. Notably, we could also allocate several Metazoan (Animalia) lineages in GH3 entries, amongst them two entries of Amphimedon, and each of Ramazzottius, Brachionus, and Diploscapter (Figure 5, arrows). The variation is also notable in the predominantly plant β-d-glucan glucohydrolase clade (further splitting into at least three sub-groups), basal to node G (Figure 5). Within this clade, most of the entries carry a tryptophan clamp bordering the active site (Trp286 and Trp434; numbering corresponds to HvExoI) as previously defined [25]. It could be hypothesised that these plant β-d-glucan glucohydrolases evolved from bacterial β-d-glucosidases through, parsimoniously, horizontal transfer or alternatively through parallel neo-functionalisation. These conclusions require subsequent analysis and consideration. Our analysis shows that the unique catalytic mechanism underlying substrate-product-assisted processivity in β-d-glucan glucohydrolases emerged through complex and dynamic evolutionary processes.

## Structural basis of processivity in GHs and its significance

As referred to above, processive GHs feature groove-, saddle-, tunnel- or cleft-like shaped, but also enclosed pocket-shaped active sites to accommodate enzyme–substrate complexes through multiple aromatic and other residues that partially or completely enclose polymeric substrates [20,21,23–26] (Figures 1–4). Although the active site composition and shape criteria are not indicative of processivity in GHs containing saddle-shaped active sites, where e.g. endo-acting *Geotrichum* XEG is a distributive catalyst [50].

The processivity of GHs that act on polysaccharides, is typically linked to the sliding of enzymes along substrates, which reduces a 3D search of an enzyme for a target site to one dimension, and thus lessens the energy barrier for substrate binding [3]. After a catalytic event is completed, the enzymes through sliding must strike an equilibrium amid the strength of binding and the ability to move linearly along polymers. In processive polysaccharide GHs, the problem of sliding is solved by allocating mostly aromatic (Trp, Tyr, Phe, and His) residues in active sites environments (Figures 1–4), which provide stacking interactions with a large interaction surface in a multitude of binding modes – these residues are also shape-complementary to carbohydrate moieties [3,18,21–23,25]. Aromatic residues are often arranged in a parallel fashion and provide large clamp-like geometries, where apolar or hydrophobic (β) or polar (α) faces of carbohydrates are exposed to side chains of a single or pairs of aromatic residues (Figures 1–4). There are up to three or more C–H groups close in space that on an apolar surface of a carbohydrate moiety, could contact aromatic side chains [70], and it is expected that the two faces of carbohydrates provide interaction energies with different strengths [71].

Through stacking interactions, aromatic residues and their clamps (with contributing hydrophilic residues) are thought to be a driving force for the movement of carbohydrates in active sites [25]. Stacking interactions, consisting of non-polar dispersion-driven C–H–π forces (also known as London dispersion forces) [72], are fundamentally different from the standard hydrogen bonds, which are mainly stabilised by electrostatic interactions. Geometries of C–H–π dispersion-driven (stacking) interactions in enzyme-carbohydrate complexes exhibit face-to-face, edge-face, and offset stacked orientations of aromatic moieties [73], and are mediated by Trp residues in 40%, trailed by Tyr, Phe, and His residues in about 39% identified enzyme-carbohydrate complexes [74]. It was estimated that the free energy binding of carbohydrates via aromatic residues could be in a 2–9 kcal/mol range [estimated via isothermal titration calorimetry with endo-(1,4)-β-d-xylanases binding xylo-oligosaccharide] [75]. Likewise, it was shown through X-ray crystallography, isothermal calorimetry, bioinformatics data mining, and quantum mechanical calculations of lectins, that the C–H-π stacking contribution to the overall binding energies of carbohydrates was in the 4–8 kcal/mol range and that the estimated interaction energies almost equalled C–H–π energies [72].

It is now apparent, that dispersive forces must provide moderate binding affinity to avoid tight carbohydrate binding at any given location (subsite) in the enzyme active site [41]. Investigations of cello-oligosaccharides binding in the family GH7 processive cellulases using the free energy perturbation/replica-exchange MD analysis showed that intrinsic processivity (i.e. the probability to act on a substrate without dissociation — which is high for processive enzymes) [76] directly relates to the binding free energy, defined as the free energy change between a polysaccharide alone and the enzyme–substrate complex in the catalytically active complex [77]. In other words, in the case of GH7 hydrolases, the active site architecture is a primary element of processivity, and it is precisely the active site loops and assistant residues enclosed in tunnels, which contribute to association with substrates and mediate processivity.

## Conclusions

Processivity is crucial for the functionality of selected oligo- and polysaccharide GHs and other enzymes. It is beneficial to consider the degree of processivity as a major functional criterion for considerations of the significance of hydrolases with exo- or endo-acting modes of action. Processive catalysis is often associated with GHs containing tunnel-, cleft-, saddle- or groove-shaped catalytic sites, but also with those that contain enclosed pocket-shaped active sites, populated with aromatic residues that allow sliding of polymeric substrates through enzyme active sites without losing contact with substrates. The key question, however, is whether an initial attack in a polysaccharide-hydrolase is made through an endo- or exo-mode of action — by a processive catalysis or not.

It is important to understand processive catalysis at the molecular level and in the context of the evolutionary history of processive enzymes and how they form reaction product trajectories after catalytic events are completed. In a broader context, it is remarkable to observe that the formation of exit routes for hydrolytic products in hydrolases [25] and polysaccharides in GT2 transferases [15] bear some common characteristics in how cavities and channels are formed and how C–H–π dispersive forces are involved in reactant binding.

Based on the analysis of evolutionary relationships in the GH3 family, an argument can be made that β-d-glucosidase and β-d-xylosidase activities existed prior to the evolution of eukaryotic cells but the evolutionary mechanisms such as horizontal transfer add further complication to an already richly diversified GH3 protein superfamily. This diversity is evident in the branch length of tip nodes indicating the significant early evolutionary change. Horizontal transfer is a plausible explanation for the scattered distribution of eukaryotic species in node A, and the emergence of plant β-d-glucan glucohydrolases from bacterial β-d-glucosidases in node G, although a storied history of gene loss and multiple independent origins cannot be ruled out and awaits further analysis.

Although a variety of processive mechanisms exist in polysaccharide-hydrolases, the underlying principle is that evolution has found a way how to implement processive catalysis to safeguard catalytic pragmatism, and how to link it to catalytic efficiency. Further progress in this field, especially in understanding and implementing industrially important GHs, is needed to enhance cost-effective biomass conversions to biochemicals and biofuels [77,78].

## Perspectives

- Structural and biochemical studies emphasised the fundamental importance of thermodynamics and mechanistic knowledge for the understanding of processivity in GHs and their engineering. The findings, unravelled with processive enzymes and molecular motors (in which stimuli trigger the controlled motion of one molecular component relative to another), hold key importance for improvements of heterogeneous but also homogenous catalysis.
- It is now clear that processive enzymes possess a variety of architectures to ensure that a single association binding event takes place permitting a series of enzymatic conversions, which are imperative in understanding the role of C–H–π dispersion-driven (stacking) forces and their contribution to the processivity of GHs. These studies are critical for the future progress in the field of catalytic mechanisms of hydrolases, and other processive enzymes to improve our understanding of e.g. recycling of recalcitrant polysaccharides. In cellulolytic and chitinolytic systems containing non-catalytic carbohydrate-binding modules, the outstanding question is how these modules precisely enhance processivity at the molecular level, and why they are attached only to some GHs.
- This knowledge could encourage improvements in catalytic rates, stability, and product inhibition of processive enzymes, as the new ﬁndings are directly applicable to developing or manufacturing products through bioengineered enzymes that could also be applied outside of biological systems in chemical, food, and feed industries. This includes applications in the design of chemicals (e.g. oligosaccharides used as antibacterial and antifungal agents and drugs), herbicides, and pesticides, where enzymes are seen as environmentally friendly and cost-effective alternatives to current procedures.

## Data Availability

Data contained in the article are available within the submitted material (main manuscript, Figures 1–5, and Supplementary Dataset).

## Open Access

Open access for this article was enabled by the participation of University of Adelaide in an all-inclusive *Read & Publish* agreement with Portland Press and the Biochemical Society under a transformative agreement with CAUL.

## Abbreviations

CBH(s): cellobiohydrolase(s)
CBM(s): carbohydrate-binding module(s)
Cel48F: *Clostridium cellulolyticum* GH48 endo-acting cellulase
cMD: classical molecular dynamics
cpk: Corey–Pauling–Koltun
G or Glc: glucose
GaMD: Gaussian molecular dynamics
GH(s): glycoside hydrolase(s)
kT e^−1^: kilo-tesla electron^−1^
PDB: Protein Data Bank
PoXEG74-cat: catalytic module of *Paenibacillus odorifer* GH74 xyloglucan-specific (1,4)-β-d-endoglucanase
RMSD: Root-Mean-Square-Deviation
SmChiA (SmChiB): *Serratia marcescens* GH18 exo-acting chitinase A (B)
SmChiC: *Serratia marcescens* GH18 endo-acting chitinase C
TrCel7A: *Trichoderma reesei* GH7 cellobiohydrolase
WT: wild-type
XEG: xyloglucan-specific (1,4)-β-d-endoglucanase
XG: xyloglucan
XXLG and XGXXLG: (G: unbranched d-Glc*p* residue, X: branched α-d-Xylp-(1,6)-β-d-Glc*p* residue, L: branched β-d-Gal*p*-(1,2)-α-d-Xyl*p*-(1,6)-β-d-Glc*p* residue)
