# Supplementary material for: Molecular mechanisms of processive glycoside hydrolases underline catalytic pragmatism
Source: Biochem Soc Trans. 2023 Jun 2;51(3):1387–403. doi: 10.1042/BST20230136 (PMC10317168; doi:10.1042/BST20230136)
Supplement: Supplementary Material 1 [file BST-51-1387-s1.pdf]

Evolutionary relationships in the GH3 family

Large-scale phylogenomic analyses of 550 GH3 entries shed light on their complex evolutionary relationships. Putative GH3 protein sequences were identified using the EBI hmmsearch service [1] through UniProt [2] where significant hits to the PF00933 (Glyco_hydro_3) HMM were retrieved for major kingdoms/phyla (Archaea, bacteria, fungi, Viridiplantae, Metazoa and other eukaryotic protists (i.e. SAR clade). Each sequence set was clustered using MMseqs2 (--min-seq-id 0.55 -c 0.8 --cov-mode 1) [3] with cluster representative sequences brought forward for subsequent analysis. Additionally, 62 sequences [4] we included to anchor the functional classification of clades. Amino acid residues were assigned to both the PF00933 and PF01915 (Glyco_hydro_3_C) models using hmmalign [5]. The resulting alignments were stripped of sites below 0.5 posterior probability or those containing only gaps. Given that PF00933 and PF01915 domains are non-overlapping the corresponding alignments were concatenated in preparation for the phylogenetic analysis. Phylogenetic trees for the PF00933/PF01915 multiple sequence alignment were calculated using IQ-TREE version 1.6.12 [6]. Model selection was performed using the IQ-TREE model finder plus function with the BIC score used to determine the best-fit model (LG+F+R10). Both ultrafast (-B 1000 -bnni) [7] and nonparametric (-b 300) bootstrap methods were calculated to determine node support values.

1 Finn, R. D., Clements, J. and Eddy, S. R. (2011) HMMER web server: interactive sequence similarity searching. *Nucleic Acids Res.* **39**, W29–W37 https://doi.org/10.1093/nar/gkr367

2 Bateman, A., and UniProt Consortium (2023) UniProt: The Universal Protein Knowledgebase in 2023. *Nucleic Acids Res.* 51, D523–D531 https://doi.org/10.1093/nar/gkac1052

3 Steinegger, M. and Soeding, J. (2017) MMseqs2 enables sensitive protein sequence searching for the analysis of massive data sets. *Nat. Biotech.* **5**, 1026–1028 https://doi.org/10.1038/nbt.3988

4 Luang, S., Fernández-Luengo, X., Nin Hill, A., Streltsov, V. A., Schwerdt, J. G., Alonso-Gil, S. Ketudat Cairns, J. R., Pradeau, S., Fort, S., Maréchal, J. -D., Masgrau, L., Rovira, C. and Hrmova, M. (2022) The evolutionary advantage of an aromatic clamp in plant family 3 glycoside exo-hydrolases. *Nat. Commun.* **13**, 5577 https://doi.org/10.1038/s41467-022-33180-5

5 Eddy, S. R. (2011) Accelerated profile HMM searches. *PLoS Comp. Biol.* **7**, e1002195 https://doi.org/10.1371/journal.pcbi.1002195

6 Minh, B. Q., Schmidt, H. A., Chernomor, O., Schrempf, D., Woodhams, M. D., von Haeseler, A. and Lanfear, R. (2020) IQ-TREE 2: New Models and Efficient Methods for Phylogenetic Inference in the Genomic Era. *Mol. Biol. Evol.* **37**, 1530–1534 https://doi.org/10.1093/molbev/msaa015

7 Hoang, D. T., Chernomor, O., von Haeseler, A., Minh, B. Q. and Vinh, L. S. (2018) UFBoot2: Improving the ultrafast bootstrap approximation. *Mol. Biol. Evol.* **35**, 518–522 https://doi.org/10.1093/molbev/msx281

>tr|A0A3N7HH59|A0A3N7HH59_9BURK
MKKWLTLGAAGLVLALHGCGGSDKDPEPAKPTALDDWPRVKSSIAQDSSQESQIKSIVANMTLAQKVGQMTQPDIRSITPDQVKQYYIGSVLNGGGAWPGNKKNASVAD
WLALADAYWQASMDSDMAVKIPVIWGTDAVHGHGNVYGATLFPHNIGLGAANDPDLVERIGAAVAKQVVSTGIDWTFAPTLAVVRDDRWGRTYEGFSEDPEIVGSYGGR
YTTGLQGNFAASGRPTVVATAKHFMGDGGTDQGKDQGENKSSLNDMMNIHGAGYYSALAAGAQTVMASFNSWTFKGTTKDGVSLDFDNAKMHGNQYLLTDVLKGKMGFD
GLVVSDWNGIGQVKYKDSTGATKQCTNSSCPPSINAGVDLIMVPDDWKAFITNTIASVNAGEIPMSRIDDAVTRILRVKLRAGLFTVEGGASVSVKPSLRPGAGDATAL
VHRELAREAVRKSLVLLKNDGGVLPLKRGEKILVVGRSADSMSNQTGGWSLTWQGTSNTNADFPNGDTVLAGIKEAAGDANVTYALDASNATLTDYKAVIAVIGETPYA
EGAGDIGKTGTLEHAKRYPADLALLESLKGKGVPVVTVLMSGRPLWVNREINRSDAFVAAFLPGTEGKGVADVLFRKADGTVNNDFQGKLSYSWPKSACQVPLNKGDAS
YDPLFAYGYGLTYATPGTTVGNALDETAPTLGCGQSANTGPAATDPLEVFTQVENPTFPLFIGSSPNWMIPVGSDLNAVLTTQDSTLTLETTQVNVQQDAKKVTWTGLA
EFKAMAADPASTSTKFPQNLESYVHPDAKAVLAFDVVVHQAPAGSVKIRVDCGYPCRGELDGTAVLSALPANTKTTVKIPLSCFADAGADFSAIDTAFLVQTDKPFSAS
FARIRWVVGATGDSDTHTCAEFVPPPPPAIDPLPGPSVTLLGSSGLFADLAAGTWSSNGTHVTANVANGVADLQFAADGGNGIFTLRGSAINLSNYAAGKLQFDVAVSS
YGTNTKGLAIKMESPGDGCRNVDYVIPDAQKPPADGQFHTVTLNVADVAGTKNAACFTLENIVIPFGIFPVWDDQQGVNFQVKNVQLLQ
>tr|A0A4R2CI28|A0A4R2CI28_9ACTN
MPRWPAVPRPDRRRMRPKLRTALVTAAALLTGLLPLGVASQAAADDPVPTVLAAFEGAEPFASPPNAGIFGWGSDADDPPTMELQARADAPVGEKVLHGTYNISGYGGF
SHDVTFDQNPGDWSAYKGIRFWWYGQNTAPLPPGSGKRIFFEIKDGGANAEASELWNTSFTDDWQGWHQVEIPFSELVYRGDYQPVGGIDQVLNLTQMWGYAFTMPVGT
PGEFAIDQVEVFGKADPALKASVVTDAGVYPVKEGDTAQVKLTVATTGSTPLEEPVTVEYRTGTGTAGATDYLPVSGAFTFPAGTASGTSQLVKVVTKKDNSAEVAETI
PLELTVTGAKPPAATPQVVINAHDLPYLNSKLPVKTRVKDLLGRMTLAEKVGQMTQAERNALRSRTDIATYALGSLLSGGGSVPTPNTPAAWAAMIDGFQLNAQATRLQ
VPLIYGVDAVHGHNNVIGATLLPHNIGMGATRDPELSRRAGELTATEVRATGIPWDFAPCVCVVRDDRWGRAYEGFSEDPALAKAMATVITGMQGKADGSQLDQNNHVL
ATAKHFVGDGGTTYGSSTTGAYKIDQGVTEVTPQQLEAIHLDPFKTSVDLGVGTVMPSYSSLDIIGDDQGPVKMHGDAALINGVLKQRMGFDGFVISDWQAIDQLPGDY
PSDIRTSINAGLDMIMVPTNYQAFTQGLTDEVTAGRVTQGRVDDAVGRILTQKFKLGLFEHPFADTSRLSSIGSAAHRVVAREAAAKSQVLLKNQDNVLPLASTAKVYV
AGSNANDLGNQMGGWSITWQGASGNTTTGTTILDGIKQVVPTATFSQDASAPLEGHDVGVVVVGERPYAEGIGDVGNGHDLALSDADKAAVTKVCAAMKCVVLVVSGRP
QVIADQLGDIDALVASWLPGTEGAGVADVLFGKRPFSGRLPVTWAKSEAQQPINVGDASYDPQYPYGWGLTTQAAARQKLTDAKKELLRKDFLSPDVLAAVINIDLALH
VQDWSGPQATTALAALGQAGKYLQRAMVDSFADDDAVVSAARWIAQDQIGQNLDEATSKLTSDADHLALSGDLTGAITKLTAAYKLK
>tr|A0A1L9SYA0|A0A1L9SYA0_9EURO
MAVNSVSARLRISPTLEINELVSKARADGKHVIHLGFGEATFPVQQDVLQAHRDASNETSYMPVAGIEALRDSIASFNSDRLGVKISPDQVVIAPGSKPLLFALFDILD
GAVLLPRPSWVSYEPQVLHAGKKLFWIETDPVDRHSITESSLLSAYRKAISEGCIPRIMLINSPSNPTGHVFSANCVRTIKEFCKSKEISLITDEIYSDICFDETQIGI
SAFDSNTDDSETVILTGGLSKTYSAGGWRVGYAIFPPSQKGETIRKTILAYASECWSAASAPAQLASAKAFSTGEGQAFVINEKYSIDIYIINNPQSGKESEFFADCTR
DPLCSNPVCDPSLSVRERAAALVKVLNVEEKLDTLVDKTPGVPRLGIWPYRWWSEALHGLAYAPGNYFEDSGNFSSATSFPMPIVMAASFNDDLVFAIGETVSIEARAY
ANVGLNGLDFYTPKINLFRDPRWGRGSETPGEDPLLAKNYVASLIHSMEDQSSGYKRIIATCKHYAGNDLESVGNVSRYGFNAVISTQDLSEYFLPPFRTCTAQKQVSS
VMCSYNAVNGVPACADSYLLQDILRDHWRWEAPDHYITTDCGVIGYMVTDHHYASDLGESAALALKAGSDLECNGGPNSWLLSAWNRSLITEREVDRAAQRLYGALVTV
GLFDNPPSLQSLSWDAVNSPSAQQLAYQSAVGGTVLIKNDGALQLSSNQSYALIGPWVAATEQLQGIYYGPAPFLISPLPAAKDLGINYTYVAGTGINETDPSTYDAAI
AAARSADVVIFLGGIDSSFEKETQDRESLEWPTPQMELITALAENAEQLVVVRYGGGQLDDSELLTNENESGLLWGGYPGQSGGQAIMDLLFGKASPAGRLPVTQYAAW
YADVVPPTDMSLRPGKGNSYLGRTYMWYDGEPTAPFGFGLHYTTFDVTIDRTPRWMDHRPIHNGTRVSIQSVKGSADWMELLELPVLQIPVTVTNTGPVTSDYVVLLFL
KSDAGPSPRPLKTLAAYTRIKDIQPGQLVTRDLIVNLDSLVRVDQNGDRVLYPGKFDLFVDLDATATFTFKLHGAPLTVERFPQSK
>tr|A0A0C5V292|A0A0C5V292_9GAMM
MTKFTPLLVASALVLTSGCNENSNDTDTPRSPDTTNDMVYRTDYQDWPELTSTIALDPSIEAQIDTLIMNMTLAEKIGQMVQPEIKQASPQDVIDYHLGSVLNGGGSWP
NNDKNASAADWLAMADSYWEASMNTDDGKAAIPLIWGTDAVHGHSNVMGTVLFPHNIGLGAARDPALIKRIGQATARQVTATGIDWTFAPTLAVVRDDRWGRSYEGYSE
DGEIVFNYGGAMVEGLQGDFGDDHVLATAKHYIGDGGTDQGDDQGDNLSNEHDLINIHGQGYYSALSAGAQTVMASFNSWNGEKLHGHEYLLNTVLKGKMHFDGFIVSD
WNGVGQVTGCTNSHCPQAINAGIDMIMVPDDWKALITNTIQDVNDGAIAMSRIDDAVRRILRVKFRAGLFTKVKPSERQGAGDANNLHSDEMRAVAREAVRKSLVLLKN
NADTLPLSKDARILVTGKTADSMMNQTGGWSLSWQGTGNTNSDFPYGETILSGINSAISSTGEVTYSQDGSAADDSYDVIIAVIGETPYAEGNGDIGKFSTLSFGSAYP
ADAKLLDTLNSNAPGVPVVTVYVGGRPLWMNPELNKSAAFVAAWLPGTEGAGVADVLFGDYPFTGKLSFSWPATDCQVLVNRNDGQTPLFAYGFGLSTSDDGSLSTLSE
ETSNQGCGAGDNSSAGTTDVPLELLSNGNTATDYTLRIGGPSNWSGTEVDIDPSATSTLPGNEITVKTIDGSVQFSAKNIVWSDIAQIYIQSTDANTTRDLAAYGNSET
SISFRVRVHEAPDESVEAVNLSAHCIYPCVGEINIAGLLRSLPLDTWQDIKIPLACLTNTGLDITSVNTPFLLYSTGAMNIDIENIGWQPWTEDEALDCSTLQPPVQAP
TISTTTSVYSDGIDDSSLFKQPAKWATDASTWTETPDYVTLDAAYNDGGNTVIDAQYGNAANHKGIVVLGMVKDTDLSQLPAGSYISFDLNVISLGESNGLTAKMVCNS
DPDNCRSGDLDITTLTDEYSGAIPLQQWHTYHIILDNYADLDKSKITTVLELLPNWADSHNNVHFQIDNVKIVANNAL
>tr|R1EJJ5|R1EJJ5_EMIHU
MAAATLWLTRMSAADKIGQMTQLDISMVLADGCPLRIDDAKLRRQLRVHRLGSLLNSPFSGTDPRCGSSGWNASEWRASVEHVQAAAADEGLPPLIYGIDSVHGASYCR
GATLLPQQLGLAASFDESLAAASGWLTGKDSRAAATPWMFAPILGVATHPLWPRVYETFGEDPLLVARMGAAVVEGMQRRGGEGEIPARAAACMKHFVGYSAPRNGHDR
EGAWLSRRELEQTYLPPFQAAVDAGVQSAMESYQEVNGEPVVGSAALLQGLLRERLGFEGLLVTDWHEVGNLHDFHRVARSPEEAVLLAIGGSSSLDMSMVPTDESFSV
LMERLHASGAIDEAREWLGLLPPPPAVRPAPWSHSEATVRLVESVGSASDAALAHEAARATVTLLKNAPPCAGPPQQQPLPPLSPEPAPPPSSPGGSNGTGEAEGGAAV
GVEECDAYLGEGGGGCGWTERWRRADGRMSSRGVRAARGWPHEFARRQGCARMAAWRTSRGVGGDGTDDDERLPLPPSAPLSPAADSLGRLSGGWTAHWQGTLADDEIR
GSRPGAATPTLLEALRAVDGWEVDHLPGCSLPGTAFAVRSSCAQTNSSAELIAAAAAADVVVACLGEDPHTEKPGDLDDLSLDAGQIDLVRVLASAGKPIALVLVTGRP
RLLHGVEALPQVNAVLQSFLPGPHGGAALADALVGRFEPSGRLPISWPTAASSLLYPHWHPTPRSGLSYSRLQYGPLLLSSGALPRTGAVNASVAVSNPSSLALNHTVL
LFAAADFRRVVPEAQQLKAFRRVLLPAGSSVEVSFTLRADDFRYWGLEERWVVESGDVSLWCDPLLRPKGPLAALGAVTRRRASEGGGMAAARARLYVEGDCRGAGCGA
EGWWERDASGERYEIGKYIAHVPGPSPAGVIATTFLAGWLVGLICAGGAWRRGQASGRGLGEVGKGVGGSSSSLVNTSWSTTLTQRGGEFGSQACMLGAGFKQSHSIGS
SMGSAMDAAFRRTCSSGIALGALAQSGSGASLSSVAEYGRPASISGLRTESNLNNRGDDHEAELHCPPELRIDEDKCL
>tr|A0A7H8R6L8|A0A7H8R6L8_9EURO
MTINDKAVNLISPASGVSGIGLSAYEWWSEGLHGVAGSPGVQFQTPNGSDFSYATSFPLPILMGASFDDDLIQNVARVIGKEARAFANAQKAGFDYWTPNINGFLDPRW
GRGLETPGEDIFHIQNYVKQLVPALQGVDNKEDLRQIIATCKHYAVYDVETNRTGQNYDVTQQDLGDYYLPAFKTCARDALATSIMCSYNAVDGVPSCASEYLLQMVLR
DTWGFTAADNYVVSDCDAVNNIWDSHGFTTDVDSAAAVALNAGTDLNCNGAYKNLVGSVAGNMTTESAMDQSLTRLYHALFKVGYFDGAPDWDGFSWADVSTSDAQTLA
YEAAVEGITLLKNDGLLPLSADTYSSVALIGPWANATSQMQGNYYGVAPYLISPLEAFQASFDKVQYAEGTAISGTTDAGFSEALSAAQSSDAIFYMGGIDTTVEAEGM
DRTSVEWPGNQLDLISQLGALGKPLVVVQFGGGQVDDTVLLQNSSVNAVVWAGYPGQNGGNAVCDVLTGKKAAAGRLPVTQYPADYTNEANIFDPTIRPNITFPGRTYR
WYTGEPVLPFGYGLHYTNFSLSWETQPQSTYSIQNLVDSGGDGSDYLDLAPFVNVSITIKNAGGPANVASDYVGLLFLSSSNAGPEPIPNKTLVSYSRAHDIPVDESQV
LSLPVTLGSLARADENGDLTIYPGDYVFSLDTDAELSAGFQLTGDSAIVDTIPRQAAHTPRSSNPLCQSKYLGVAESQTMSTAMLTVALAGSCLARTSATFNWDSTKHL
VAFGDSYTYVQGTHGYPDYSFIGDYLPGQFAFTPEDLLSNKIVQNFSGTAEGGPNWVEYLTGCALEEGLTSPETCKLQLWDFAFAGADISQEFTPLHHNFTIPLVNQTK
QFLTYAQPVLSKQKSFNLNQALIAIWIGINDIGDSSKYNVSFPNFYEKLWDTVFTESVQPLYHAGYHNFLFVNLPPLDRAPGNVNSVSPLPNKTMIGWFNDEIERHSTA
FGAAHPEVKSMVFDATTLLNYVLDNPSKYNIRNTTSYCSGYTDPDVVVDPGKYGCIPIEEYFWFNTGHM
>tr|A0A1M5IXT7|A0A1M5IXT7_9ACTN
MHRSRLRRPSRLLAGSAGLLLTVGLAAVVTAPAADAQVGPLPVVQDFEGDGVVTTTSPGVFPFGNDATDSPDLTIVSAPGVPGADADNHALDVPYSVTTYGGFSANSAQ
PQDWSAYDSFGFWVKGSATGKEIQFEIKDGGSDGEHAELWESHFTDDSTAWKHVVTPFAQFTKRTSYQPPGAPSNDVLDLTSMWGFAVNLPGGGASGDLQFDDARVSGT
AQPRVSVDDRTVLVDAGHEATVGVTLTTADGGELASPVTVDYATADGTAVAGTDYTATSGTLTFPAGTKSGASQHVTVQTAPADGAAKAKTLTVTFTPTGATLSGGKAL
VVINAHGYAYLDKTRSTSARVKDLLSHMTLADKVGQMTQAERVAVGDGTDVTTYALGSLLSGGGSTPQPNTPAAWAKMIDGYQTQALATPLQIPMIYGIDSVHGDNNLA
GATLFPHNIGLGATRDPALVKQAGAVTATETRATGVPWAFAACVCVVRDDRWGRSYESFGEDPSLVTDMTTVFQGLQGDGKLAARTSVLATAKHFIGDGGTRFGSSQTN
DYTIDQGITYVTQSQLNQLVAPYRAAIKDGVGSVMPSYSSLRIQGKDAAPVKMHGRKDMITGLLKQKLGFKGFVISDYAAIDQLPGDYKSDVQTSINAGLDMIMVPNDY
KTFITDLTDLAGNGVPLSRIDDAVTRILTQKFRLGLFDHPFADTTNIKTIGSSAHRAIARKAAAESQVLLKNSRNVLPLAKRSKVYVAGSNADDEGNQSGGWTLTWQGQ
SGAIPGATSILDGLRKDDPTSTFTYSKTATASAKGYDVGVVVVGETPYAEGQGDIGVNKHTLQLSVADRQAVNRVCGAMKCVVMVVSGRPLDITGVAPQAEAVVASWLP
GSEGTGVADVLTGRRPFTGRLPVTWAKAESQQPINVGDKRYDPLYPYGWGLRTDGTHARLVALRTELRAAGHTTTARELTGALAKANWKGTTLRRADAVLAVLRKAANR
TAGRSFSWTERNTVVSIVRDIAQARVVSGGKAAMSDTAALTADAEHLLMAGHPGAAASSLVAAYRRA
>tr|A0A364LBE3|A0A364LBE3_9EURO
MMAANLRQRMDRFLDFINSGDESIGREVVSESAVFHVPFSEQPLTGLAGYMQILGMMRSSFPDVQWSIDETVIEGDKVVAKFTLSGTHKGEFFGVPPTGRKIQARAMNI
YRFYEDKILEETGLPDIFAIMLQIGAIKPPQPPQCHKVCDTSLSITERVKSLVDSLTLEEKILNVVDASAGSARLGLPPHEWCNEATHGVGSAPGVQFTEKPANFSYAT
SFPAPILTAASFDDGLVRKIAGVIGKEGRAFANNGFSGFDFWAPNINPFRDPRWGRGQETPGEDTFVVQSYIRNFIPGLQGNDPEEKQVIATCKHYAVYDLETGRYGND
YNPSQQDLADYFLAPFKTCVRDTGVGSVMCAYNAVDGIPSCASEYLLEQVLRQQWNFTADYNYVVSDCDAVTDIWRYHNFTDTEEAAASVAMNAGTDLECGSSYLKLNE
SLAASQITARSIDRSLTRLYSALFTVGFFDGGKYSGLDFSDVSTPEAQALAYQAAVEGMTLLKNDQNLLPIRSSHNYKSIALIGPFANATTQMQGDYSGVPPYLISPLQ
AFETHSEWEINYSVGTGINNQSTAGFGPALAAAEKSDLTIYLGGIDNSIEAETLDRTSLTWPGNQLDMVTQLSHLHKPLIVVQFGGGQLDDSSLLQNEGVQALVWAGYP

SQSGGTALLDVLTGKKSIAGRLPVTQYPASYADQVSIFDINLRPALNGSYPGRTYKWYTGKPVIPFGYGLHYTHFDFEWEQTLDHGYNIQNLVASCRSDGPINDTFIWI
IMIFTGIVGTLLFQFAMTVMGEHSSPSTISSGCGKALTLHSGTYTTTVNGKQRQYTLTIPQGYNPSEPYKLMFGYHWLGGTMQDVVSGSYYGIEPLAGNSAVFVAPQGL
NNGWANSGGEDITFTDQMLSTLENALCIDKTQVYSMGWSYGGAMSYALACARPDVFRAVAVMSGANLSGCSPGSQPVAYYAQHGVSDSVLPFTLGEQIRDTFVKDNACT
ATNPPAPAAGSGTHIKTEYSGCSSGHPVWWVAFDGPHEPLATDAGASSSWTPGQIWSFFSQFF
>tr|A0A7J6VWA4|A0A7J6VWA4_THATH
MFIQFRLLLSFGIFIILITLSQAIKTHPHPQYPCKPPDHNSYTFCNTSLPISTRARSLISLLTLPEKITQLCNNASPIARLGIPAYQWWSESLHGIATNGPGVSFNGTI
PSATSFPQVLVTTASFNRSLWFLIGAATAVEARAMYNVGQAGLTFWAPNINIFRDPRWGRGQETPGEDPMTVAAYAVEYVRGFQGENWNVGGGGNRDRFGEKRVLAGSG
ESVGSELMLSACCKHLIAYDLENWKNFTRYDFNAVVSDQDMQDTYMPPFRSCVEDAKASCLMCSYNEINGVPACANKDLLQQARVEWGFKGYIASDCDAVGTVYEYQHY
ARSAADAVADVLKAGTDINCGTYMLLNTQSAIEQGKVQEEDIDGALYNLFSIQLRLGLFNGNPVKGKYGKLGPHNVCTKEHRKLALEAAKQGIVLLKNDRNFLPLKKFS
VASLAVIGPTANGTSNFGGTYTGIPCDAKSFLDGFQAYTQNVHYTAGCVDVACQSTDGFAEAVRIAQMADVVIVVAGLDLSQETEDLDRVSLLLPGKQMDLIAAVVVAS
KRPVVLILTGGGPIDVSFAKEDTRIASILWVGYPGEAGGLALSEIIFGKYNPGGRLPMTWYPESFTTVPMNDMRMRSDLSHGYPGRTYRFYTGETVYEFGHGLSYTNYT
YKFISTHNKISLLGSSTKTDSSSILYHSKEELDYIHIDQVPSCDALNFYTHISVMNNGNMSGSHVVMLFCRQPRMFKDAPQKQLIGFNRVHTESSGATKTRFLIEPCKH
LSTVNKLGLLFTVVMFWGFGLPFSNEMKEMEELPSVEIVRLVLQKQLLKLEPEVITTTNYESCHNVGVQVGLVLGGQALKKGKNVFLLNQVKPSVIYESYTDPDVHRIV
KKKIPWKKFNIGVKEHVGIFIKIDKSESYGKEDHRIFNHLVKSCILECCECVRPLMGKSSYVSELRERRIAAIRLADPVCRCFRFKNPPSKNCPINRAKSTNHMDSWAI
ADEMIARARASMCKRSCSDFGGKNSSTSLGKESCSDSTRKNSSISLDKESCSDYAGKKRKLR
>tr|A0A437A6N7|A0A437A6N7_9PEZI
MSSAYRHEYLEPPHARRPFFHTDVDSSSNTALSTTAVGLSPEGTVYNASSDQLLGQGGNRLPPPQRAVEIPPGEIDLVPPLRTGANSVTPQSERSIGGGVTGGGLGTVG
STNQAAATGGEVGGEKRESEALGHDEKASLFTRFTRRRRNLVLVAILIMLLIGLILGLAIGLTKGQSKGGNNEIVVRSRPYYPSPRGGWVSSWQDAYEEAAKLVRDMEV
PEKVNLTTGVGWSMGPCVGNTGSTKRIGSLCLQDGPAGIRFADDITAFPAGVTVAATWSKYHMYARGKALGAEARAKGVNVILGPCIGPLGRFPVGGRNWEGFGSDPYL
QGIAAGITIDAIQSEGVIATAKHYIGNEQERFRQVPEAHGQGWPNVTEALSSNIGDRTLHELYLWPFAEAVHAGVGAIMCSYNQVNNSYACQNSYLLNGVLKDELGFQG
FVMSDWLAHHSGVASALAGMDMSMPGDAKSFLDGETYWGSFLTESVVNKTLPIERLDDMALRIVATWIKMGQHKDFPEVSFSSWTKLRDGFVYQGTFSGPVDVVNDFVD
VRQNHSVITQEIAAEGIVLLKNFNNSALPLNDEKLPRAIMIFGSDAGPSPNGPNGCIDRGCNQGTLGQGWGSGSVDYGYQITPLEAIQARLLGRRGKKPLVEYSLDDRD
LGRASRLAETPGAKCFVFVSSDSGEAYISSEGHLGDRNDLNLWHGGDDLIKAVAAKCNDTVVVIHSVGAVVMEEWIDNPNVTAVLMAHLPASESGSSLVNVLWGDVPPS
GHLPYTIGKSLADYGPYGDILREPNGPIPQDNFADGLDIDYRYFDANGIEPRFEFGFGLSYTEFEYGNLTVQKVYQGNISSELPPPPDRIPVPRMDTEVPTPQEVTFPE
GFNQLRNYHYPWLTRSQASSVEALLRSGNETEYPYPEGWNAEPRPNPPPGGGGEGGHPALYDVLFHVDIDVKNIGKKPGKTVPQLYVTFPDGVGVYTPIRQLRGFEKVG
VGPGESLTVGFDIRRKDLSVWDEVGGKGWYIPPVKGRAVGEGYTIWVGQSSRRGGIEGKTTE
>tr|A0A4Q2LY88|A0A4Q2LY88_9BACL
MLIKPFRIWLAGFIAFILLAAALAVPLPRTAYANDYQNPGLSIEQRVDDLLSRMTLQEKIGQMLQVERLAATPSQVAQFAIGSVLSGGGSNPTPNNAATWAAMTDSYQQ
AAMSTRLQIPILYGVDAVHGHNNVYGATIYPHNVGLGASGDADLVRRIGDATAREIRATGVNLNFAPCLCVPQDIRWGRTYEGYSENVTLAGKLGTAFVEGLQGNPGDS
GFMKGTKAVASIKHWLGDGNTTGGDDQGNVTLNEQELDPYIQPYRDAIAAGARTVMISLTTWNGQKMHVHQHLITEMLKQDLNFQGIVISDWNGAFSLVNQGVYATYAE
ALRASVNAGIDLFMEPDNWQQFIPTLVNLVNTNQVSQARINDAVSRILRVKFEAGLFEAPYVDNSLIASGLFGGAGHRALGREAVRKSAVLLKNDNHFLPLSKQSRLFV
AGSKAHNIGFQSGGWTISWQGSPGAITPGTTLLQGIQGAVTGGGSVTYSQNGTGAAGHDAAIVVIGEYPSAEMMGDVGPGQPRPNLELSAEDRTLLTNVAGSGVPMVVI
MLSGRPMIVSSDLPGWQAFVAAWLPGTEGGGIADLLFGDDDFTGKLPLTWPRSMAQIPITDKDQNYTPLFPYGYGLSASATHVAEVPGLIEAESAQASFDVRTEATLDT
NGGLNVGYFDSGDWLDYRIFAPRAGTYQLRLRVASQAGAVNAIEVISGESVQFTASVPNTGGWQTWTTISHPVTLEEGVQTLRLRAASGGWNLNWLALTPTAAASPDNL
LVNPGFDSGSAAGWEQWNGGVSAQSVDTNAPYTGTHKLTHWASGDYRQLTRQYVTVPNGQYRFSGWVRTSGGQRALHLYAKSAGRETRAQVSSNATDYWQQYTIDQITV
TNGQLEVGVWSDARGGNWSAFDAFELVQADPLVNAGFERGNLTGWTIWHSGTVAQKVDRDQPAAGSYKLTHWSAAAYQQLTSQTVAVQNGIYRLSVWARAGGAHTALQL
QAKGYGGSERTATITADSGQWARYTIDSLHVTTGMLEMGVWSDAAAGSWSAYDRFELVPM
>tr|F9FY86|F9FY86_FUSOF
MASIRSVLVSGLLAAGVNAQAYDSSDRAEDAFSWVQPKNTTILGQYGHSPHYPANNATGKGWEDAFAKAQDFVSQLTLEEKADMVTGTPGPCVGNIVAIPRLNFNGLCL
HDGPLAIRVADYASVFPAGVSAASSWDKDLLYQRGLAMGQEFKAKGAHILLGPVAGPLGRSAYSGRNWEGFSPDPYLTGIAMEETIMGHQDAGVQATAKHFIGNEQEVM
RNPTFVKDGYVGEVDKEALSSNMDDRTMHELYLWPFANAVHAKASSMMCSYQRLNGSYACQNSKVLNGILRDELGFQGYVMSDWGATHTGVAAINSGLDMDMPGGIGQY
GMYFTKSFFGGNLTRAVNNGTLDETRVNDMITRIMTPYFWLGQDKDYPSVDPSSGDLNTFSPKSTWFREFNLTGERSRDVRGNHGDLIRKHGAESTVLLKNEKNALPLK
KPKSIAVFGNDAGDITEGFYNQKDYEFGTLVAGGGSGTGRLTYLVSPLTAINARAKQDGTLVQQWMNNTLIATTNVTDLWIPATPDVCLVFLKTWAEEAADREHLSVDW
DGNDVVESVAKYCNNTVVVTHSSGINTLPWADHPNVTAILAAHFPGQESGNSLVDLLYGDVNPSGRLPYTIAFNGTDYNAPPTTAVNTTGKEDWQSWFDEKLEIDYRYF
DAHNISVRYEFGFGLSYSTFEISDISAEPLASDITSQPEDLPVQPGGNPALWETIYNVTVSVSNTGKVDGATVPQLYVTFPDSAPAGTPPKQLRGFDKVFLEAGESKSV
SFELMRRDLSYWDIISQKWLIPEGEFTIRVGFSSRDLKEETKVTLVEGCFPDNARESYYPLFISSQYRETYISRLYKHSRRVLEIVLSSLAMSDNPQDIPAMTDSIEQE
FAYGRRKWTSADPVALPHEPVYRFFDSALKAGHFREDVPGNATSAKRKKDADGYLMVKRDGNKAGFLWCDADGKAVDKKYIQMAEGIIIKHLRGPGGDAIIITMARRRI
VKFSALGTAEPPFINNPNRLRQFINEWEKREPFFIDDEDRHEQYMKERVFCSETDPELN
>tr|A8LFQ8|A8LFQ8_FRASN
MQETSPGSLPVYRDPARSTAARVADLIERMSLEEKVAQLRSIWISKYKVVLPDGTFDPARAHVLIPDGIGFVGRPVDAMGMAGFPANWHRSREETIAFVDAVQRYLVEE
TRLGIPALFHDETAHGFVARGATIFPIPPALASTWDEDLVEEVFTVVAREARSVGSTVSLGPVLDLARDPRYGRVEEFFGEDPYLVGRMGVAAVRGLQGRSRPLAADRM
FATLKHFLHASPEGGINAAPAPAHERSLRETYLAPFVDVVREANPAFIMPSYNEVGGLPSHASRDLLQRLGRALLGFEGVYLSDYDALARLISDHRVAAGLGEAAAIGL
TAGVDVDLPDGEAFSMLAPLVREGLVDETLVDEALARVLALKFEAGLFEQPYGRLEQAEYNSAEAVRLARNSATRALTLLTNDGILPLDPNAEIRLAVVGPNAGELYYG
GYSGENDAGVSVLDGLRAAIVGSAITVEHAEGVRLVGAEEEAAMPGPGRAPVLPVDDAENRRRIKDAVAVVERADVVLLVVGDHPAIARETTRPLFPGDRNELGLYGLQ
EELVEAVVQVGKPVIALLVNGRPIAATRLAAGANALLEGWYLGQETGNAVADVLFGRAEPGGRLPVSVPRASGAVPVYYDRHTSANLYPYVEVDRTPLFPFGHGLGYTT
FDISEPVLDRSSIHVGESVGISVEVSNTGNRAGDEVVQLYVRDDVSSVPRPELQLRGFRRITLEPGRSTTVRFVLEPHQLAFWNIDLTERIVEPGTFTISVGRSSTQLR
SVTLSVAGPGSERARRRRGRPGGMCRYFLGGRAVRVIRPMSWQREEARCQLSLLKRSCSPGVRLVSLSAPTRLRVEHLDEAFGTEVRRPRLSWWLPAGSARQTAHRIST
GEWDSGRIESDRARWWCGGSRCGRISARAVGPRPVPGRWGSGRTSGWRGGSNRSSTRHPCRGTAPPICSGTGSTSTGRWPAPGCTRRRTGSTSSSSTACPERRRQQPVM
MCRTRWRIRTTSSRPCRRSRRSAGGSSPGRLTSVTCPRCGGPSRRASAGWARSTSC
>tr|A0A1F5LSY9|A0A1F5LSY9_9EURO
MRFLAFSACIASVTADVITSDSHFYGQSPPVYPSPSGTGTGDWAESYTKAKAFVAQLSLEEKSNLTFGASTTENGCSGFIPAISRLGFPGLCLSDAGNGLRSTDLVNGY
PAGLSVGASWNRKLTTQRAHFMAGEFKAKGVNIALGPVVGPLGRVARNGRNWEGFSNDPYLSGVLAADTVTAFNKRGVMTSLKHYIMNEQETNRNPVTGSTPQTEAVSS
NVDDKTIHELYLWPFQDAIKAGSVNIMCSYNRINNSYGCANSKTLNGLLKTELGFQGFVVTDWTAQHSGVASALAGLDMVMPDTKYWGDNLTTAINNGSVPESRLNDMA
TRILAAWYYLGQDSSIPEPGAGMPYSVITPHKRVDARDPAARPVILQGAVEGHVLVKNNHALPLKKPQVLSVFGYSAATYKASVPSTSGFGSWGLGYESTDVQQIMAAI
GLNEGQSVELQSIAINGTIIMGGGSGATTPTYVSGPLDALTNRAMKDNSALFWDVESPAPEGVPASDACLVFINAWASEGYDRPGVYDDYSDNLVLSVADQCGNTIVVI
HNAGVRLVDNFADHPNVTAIVYAHTPGQDSGAATVALLYGDENFSGKMPYSVPKNISDYGALLDPSLPEGDYVNYPQSDFSEGIFIDYRDFESRNITPRYEFGFGLSYT
SFEYGSLSVTKTKKANNAARYPSGAIEQGGQVDLWDVLARVKFTVKNTGPVAGKEAAQLYIDTPSGVKQLRGFEKVSLNAGEKDSVTLALTRRDLSEWDVVAQKWRLVS
GANRGIGYEVAKLLLSSQDNYYILLGSRDAGRGAKAAAELDPTGASVEPVTIDVSDDASIELAAQHIASKHGHLDVLVNNAGINTELDVLLQQQKKQEQGDLGATPDFA
KLREEIKSQGTEKSNLAQLRKLYRDAYEVNVFGAAATTEAFKPLLAKAVASPPRIVFVSSHTGSMGLRSEPSSGIWEKLRSPSFPTYRSTKAALNMLTLHYAALFEEQG
WKVNASAPNLTATHFSRGIGRPASESAVNIVRLATLSVDGETGTYSDENGTVPW
>tr|A0A540MGK2|A0A540MGK2_MALBA
MAYNIAKLLSLFSLLFLFSSLCNIAVVHARPPFACDPRNPITRTLKFCRVRVPIHDRVHDLIGRLTLQEKIRLLVNNAIDVPRLGIQGYEWWSEALHGVSNVGPGTKFG
GTFLGATSFPQVITTAASFNESLWEEIGRVVSDEARAMYNGGAAGLTFWSPNVNIFRDPRWGRGQETPGEDPVLAAKYGARYVKGLQGDGAGNRLKVAACCKHYTAYDL
DNWNGVDRFHFNARVSKQDLEDTYDVPFRAFVHARPPFACDPRNPITRTLKFCRVRVPIHDRVHDLIGRLTLQEKIRLLVNNAIDVPRLGIQGYEWWSEALHGVSNVGP
GTKFGGTFLGATSFPQVITTAASFNESLWEEIGRVVSDEARAMYNGGAAGLTFWSPNVNIFRDPRWGRGQETPGEDPVLAAKYGARYVKGLQGDGAGNRLKVAACCKHY
TAYDLDNWNGVDRFHFNARVSKQDLEDTYDVPFRACVVDGNVASVMCSYNQVNGKPTCADPNLLKGTIRGQWRLNGYIVSDCDSVGVYYDDQHYTKTPEEAAADAIKAG
LDLDCGPFLAIHTEAAVKTGLVNEIDINYALGLDLDCGPFLAIHTEAAVKTGLVNEIDINYALGNTITVQMRLGMFDGEPSTQRYGNLGPADVCKMSSNELALEAARQG
IVLLENRGNSLPLSTTRHRTVAVIGPNSDVTETMIGNYAGVACGYTTPLQGIARYTRTIHQDGCSNVHCNGNQLIGAAEAAARQADATVIVIGLDQSIEAEFRDRTNLL
LPGHQQELVSRVARASRGPTVLVIMSGSPIDVTFAKNDPRIGAIIWVGYPGQAGGTAIADVLFGTTNPSGKLPMTWYPQNYVAKLPMTDMAMRANPARGYPGRTYRFYK
GPVVFPFGLGLSYTRFSHTLAQGPTLVSVPLTSLVAAKNTTMLNNNGIRVSHTNCDSLSLDIHVDVKNTGTMDGTHTLLVFVTPPAEKWAPIKQLVGFHKVHIVAGSER
RVRFGVHVCKHLSVVDKFGIRRIPLGEHKLEIGDLKHHVSVEANLGEIKF
>tr|L0K850|L0K850_HALHC
MKKLWIISFLLLSVLVLGGCKAPVDQSQITNLAIKVRDTNNQQVAANIKVLHAGEKVASKQGSEVKFELLKNKNYKLEVDKEGYLAKTVNLPLEEDSNLTVKLIKVANL
VGNGDFTTPISNANPKANGELDSGDSWVYHQNSNGQGSVTIEGGEAKVKVNNPGNNPWSVQLLQGPITLEKSAYYKISFEAHADQKAKLHLKLGANGNRGWVGYEERDV

NLTTTPQTYQFKFVMEEETDQQARFELWFLNQTDYTIDNIKLIKIKSGTATEQKDEVKIESGEIITNGSFANKTVGWGSDGNIELTNQAGKLKAKIESIGDNSYTPQVN
QKGIKMVKDVTYTVSFTARANKARKMNVAIGKPLNQAPWYIDYIGEVKTFDLTTEMKNYKFRFTMKEESYDDAKLTFELGQITDGSAATTVYLDNVRITPDLGFYTDSS
LTIDKRVSRIISLMTLDEKIGQMTQGERRHVSPKQVRKYHLGSILSGGGSTPGNNTPQDWIDMYNNFQEEALSGRLELPLIYGVDAVHGHNNLKGATIFPHNIGLGAMG
KGLMEVNKSKQAQKWIEKIARISAQETAATGMDWDFAPAVSVVRDERWGRSYESFGETAELQKLLAGPYVKGLQGTKDILSKERGHVVATAKHFIGDGATKWETGDAGY
QIDRGNVNIDLNKLKKLHGQGYLEAIDENVGTIMISYNSYQGTKMHAHQELIQNYLKAPQKEGGLGFDGFVISDWAAIHEIDAPTHYAKVVKSVNAGIDMFMEPSDWHK
FMIDLKTAVKNGDVKESRINDAVKRILKIKFKAGLFKKALTDNDSIDTIGSQEHRAVAREAVRKSLVLLKNQNQILPLSKDNKFYITGSNADNLGHQCGGWTIKWQGFS
GNQATTGTTIKEGIANLLQGQKGQIVNDLNQADVAIAVVGEKAYAEGKGDDADLELSVSDKRELQRIEESGKPMVVILVSGRPMIVSPRIENWDVFVAAWLPGTAGGGV
ADVIFGDYNFTGKLPVSWPRSVEQLPLNVGDKNYNPLFNYGYGLKMNLEN
>tr|A0A0P7C2D2|A0A0P7C2D2_9HYPO
MKSPGILLLLLLAFPAIGGASELASSPEVLPSPQGSGKGIWKSAYAKAQKLVGEFTLEEKVNVTRGFTADNVCAGNTGTVLRLGWPGMCLHDAGNGVRATDLVNSYPSAL
HVGASWDKNLTYQRGLYMAKEFKAKGVNVLLGPNAGPLGRTPLGGRNWEGFSVDPYLSGQLCAETIIGHQDAGVIANVKHFIGNEQETFRRPYFGVEAASSNIDDKTLH
EFYLWPFVDSVKAGVASVMCSYNRINNTYGCENSKLMNGVLKSELSFDGFVLLDWNAHHTLESANAGLDLVMPQGGSFGENLTQAVGNGTVSEARVTDMATRIIAAWYL
TGQDADFPSPGIGMQNLTLPHKQVEGRIPESRPVILEGAIAGHVLVKNENNALPFRKNPKMLSVFGYDATVPATKNTDKLFELGYTSSPAMGQAVLGTEEHFDQAAKGG
TIVSGGRAAANSPPYMSDPLSAIQHRAAKDGSWVNWDLSSFDPGVNAASDVCLVFINAIATEGWDRDGLHDDFSDGLVLNVASKCANTIVVIHAAGIRLVDQWIEHPNV
TATIIAHLPGQDSGRALVKLLYGEASFSGKLPYTLAKNETDYVPYAPCGRAQGDTDPQCDFTEGVYVDYRAFDERDITPRYEFGFGLSYTTFEYSALAVKVERASSNDL
WQTLATVKAKVRNTGVVLGEEVAQLYVGIPNSPPKQLRGFEKVALDEGEAAEIQFELTRRDLSVWDVVQQQWVLQSGNYTIFVESQPIIVTFATIKMPGWTSFLNGLLT
LCITLLVAAELPANATELDLVFPRSDGRYAETDQGIPVLLSLQHPQLAYHYGWGFRWTISLDKSLHLAASGTLGAVIHNDTEYSANSTHLEVAFTDHLAPGAYTFEWLF
GMGPWCEYLPGSAAYDYNPGLSEGSFGFAIEEGAPTPTFTGTCPTPVGAISFTGLTTWHGLYASLDSPTATTMFCAMTAPVTHEPEPCLATVDAVQERSISSRLQWGSF
APAATANASSESKAVRLQVPGMAVFWLAGIAGMLASLFMS
>tr|A0A364MTF0|A0A364MTF0_9PLEO
MPETSKTDHGISENNGVGISANGAPLSRQVTVALTPEQYERLFFQPSAPRRGDLAKKFANPTLLGLIGFLVPYTSTILILCGFQGAVPPQSLVGIGADYYFFGALAMNL
AGVAEFILGNTFPMAIFIVYGSHWGSLAYQQDPIHRTTDAFSELGGQYGAAYNASQGFHNVSMVLASFVFLIGTIRVNFLFTLTFFGLVMLFAFIAAADFNLAFNGVEA
DIEHIDMLLRVAGGFGFLGLISGWYLAILTACEAVGIPCPLPVFDLSSKVFPTNETTVSTSQTIGPDCQNGPLSNNLICDVYASPPERAAALVAAMETQEKLDNLVSKS
KGVPRLGLPAYNWWGEALHGVAGAPGINFTEPFSNATSFPMPLLMSAAFDDDLIYQIANVIGNEARAFGNGGVASVDYWTPDINPFRDPRWGRGSETPGEDILRIKGYT
KHLLAGLEGNQTQRKIIATCKHYVGYDMEAWGGTNRHRFDAKTTMQDLVEYYMPPFQQCARDSKVGSIMCSYNAVNGIPTCADTYVLQTILREHWNWTGSNNYITSDCE
AVADISENHNYTETLAEGTALAFANGMDLSCEYSGSSDIPGAWKQGLLNTSVVNRALTRQYEGLVHAGYFDGTAATYSDLGIEDINTPKAQQLALQIASEGLVMLKNDH
TLPLPLSNGSNVAMIGFWANDSSKLSGIYSGPPPYLHTPVWAGERLGLNMSIASGPILQNSSVADNWTTKALNAAQRSDYILYFGGLDTSAAAEGFDRTDISWPSAQVD
LITKLSQLGKSLVVIALGDMVDNSPLLSMEGVNSVIWANWPGQDGGSAVMQVVSGAYSVAGRLPITQYPADYTSLSMLDMNLRPDAISPGRTYRWYNESVQPFGFGLHY
TSFDAMFSASKGLIYNVQNIISNCTYKYQDLCEVAPIEIAVTNKGNRTSDFVALAFIKGEVGPEPFPLKTLVSYARLRDIDGGVTNMASLPLKLGTLARVDESGNTVIY
PGEYTLLLDEPTQAELKLTITGDATVLDKWPQPTTHI
>tr|A0A2Z7CXI4|A0A2Z7CXI4_9LAMI
MVARSFIILSSLAATFLFTVISARFNGLPTQLKDTTSTVNGNYTHVCDALRFFELGLNVKDFAYCDTSLPYEVRVKDLIDRMTLTEKVNQIGDTAYGVPRIGLPQYEWW
SEALHGVSDVGQWNSKATFFDDAVPGATSFPTVITTAASFNQSLWKKIGQVVSTEARAMHNLGHAGLTFWSPNINVVRDPRWGRALETPGEDPYVVGEYAVNYVRGLQD
VEGTENVTDLNSRPLKVAACCKHYAAYDVDNWLGIERYNFDARVTEQDMLETFLKPFEMCVKEGDVSSVMCSYNKINGIPACADPRLLRGKIRGEWDLHGYIVSDCDSI
EVMINHQKWLKDEPEDAVAQALKAGLDLDCGNYYTNYARNSVGKGKVSEKDIDVALKNLYIVLMRLGFFDGSPQFEKLDVTDVCSDEHIELATEAAREGIVLLKNDNQT
LPWSTDEIKTIAVVGPHANATSAMIGNYAGVPCQYTSPIDGFSKYGKVIYEMGCVDVACKNDSIIFPAVRAAKKADATVVMVGIDLSVEAESLDREDLNLPGYQNQLIN
LVASQSKGPVVVVIMSAGGVDISFARDSSKVHSILWAGYPGEEGGKAIADVVFGKYNPGGRLPLTWHENGYVEMLPMTSMPLRPIDHLGYPGRTYKFYNSSTVYPFGYG
LSYTNFTYSLVSSTKYLQVELNKFQHCRELNYTEGLYKPACPAILIDDLQCNELQNVELNLEVKNVGEKDGSEVVIVYWSPPDGIVDAPIKQVVAFKRVFVAAGGSEKV
SFVLNACKSLGVVDYKGYNLMPSGGGQFIIGSDLSASGKEKLLAGNKRARIRPVSKEERRNQDVFSSQRSPKTEEPSLERWSPAEIPNLLATLQIDRFKPRYYIGAASD
KMGLEKAKVVENSLLEKGVAVETTDIEALFLPKHRYILWLLAHKKLLTKDSQPYIEDKKCVMCNCDSESVDHLFFKCNLSKQLWNRVLHWVGIHGQLDAYCVFSGDIFA
GTNQASSVNRSCGSEIDFSIPGHCLQRNNGNS
>tr|A9URG4|A9URG4_MONBE
MAERRAWLACVVGILVGLVVVSSGEPQPWMNVEEDPTTRAEKLMQEMTLAEKIAMLHGYSGTSQNYNYTGFVVPNDRLKIPALQLNDGPQGFRATNNGYDRTTTAWPSG
LTMAASWDVAMLSKWGEGMGAEFAAKGANVQLGPGLCVARVPVNGRNFEYLSGEDPFLGYTLVQPVIQGIQSQGVIANAKHYVNNNQETKRTTVSENVDERTRFEIYYP
PFEGAINADVGSFMCSYNKINSAWSCENNETLNTDLKHRLNPDNKRFWVMSDWGATHSTSIDKGLDQEMPGDSHMGDTLADMVSNGTVPMALINASVLNILTPMFSVGL
FDKPNTGAFSNNVTSDAHNQLARELAGNCHVLLQNENKVLPLNAGTANTTYLIVGAQAWNQTIVAGGGSGHVDIPYMVTPLDAFKTALGFGPTRNNQAGQKDCVVENQC
VIYLDGSDTDMVASTAAEVDYVLAFVATTSHEGADRANLSLAGGQDEFIIAAASHSDKVVVIVTTPGAILMPWSADVASIVVNFMPGQEGANAAADVLFGTVNPSGKLP
LTMPNVENEVGFTERQYPGLDDAAEAYYDERLLIGYRWYDAHGIEPRFPFGHGLSYAAFKYDNLTVSKDEVAFKLTNIAEDYGGEVVQLYLGFPSSAGEPPKQLKGFTK
VFLHAFAEAEVKLPLRSRDRSVWDASTHSWSEVSGEFQLACRGAKVTLIERTGVACAASGKAGGFLARDWCNGGPLESLARRSFDLHEQLAAAHGNPWEYRRLRTVGIN
VLKSAGRPSPAVPWLDGGADGKTENMGSTENTAQVTPGLFTRKMVELAQEHGARVRVETVIGLSYREGLAEVCGVQLATGEAVPADAVLLCMGPWSGQCREWVRAITAP
RIGGEKAHSIVVQPGPRADIDGTAVFINYTRRGKMQNPEIYPRPDGSVYMCGGTHAHTPLPANSADVVPTEGIGAELKEIMDACSSALQDAEVTVVQACYLPMPDSGVP
FIGPLQKGLFMAAGHTCWGILNGPATGEAMA
>tr|A0A2P6TID9|A0A2P6TID9_CHLSO
MPPLALLARPAAACHPAPAARAPDAASPVQPQRVAVPRRRRFRHAAVAAAAPATSAAAAARPSRRELLAGALRFGAAGVSLMASVDDVAEVAALPRSLSSALPQSPRAA
GSLERSAAREFEVHMPLPLKPISPHVPRPDGTPASALPAQATCTQAPAPTAAEDELEVPSHARRLLVCGFAVNACPFQALLQPSRPDAAFMQLADVAADMGAERLLSAL
PCGALPIGLLLVAVSFIVVALHATSAAGSLGAAGGDRRLGRRLQQGQPWFDTTKTVDQRVDALLNSMTLAQMKAQMESTDAGAIPSLGVTAFKYQRECLHGMVGDNGES
VMYPMPIAWGATFDDGLVWQAALEIGDAMRAFSNREMYYGRGPSFTHCYGPHPAIVRDPRWGRSAEVYSEDPKVASNMANMFIQGLQGGWSAQGASTPLKVSATCKHLI
GNDLENWYGITRYNLNALIDYRDLRDTFWLPFESCVRAGAAAIMCSYNKVNNEPACLSKTLLTTVLRQQLGFKGFVATDCDALEGYAKPAPEGMGYGDLRTISVQALRA
GSDQACKKWTGLNVNDVTPAEIREAARRILRVRVRLGHFDPPRAQQFSYIPWWVIGSANHLATARKMVQESIVLLKNTQNALPLARTALRRVHLVGPWADNGVYQLGSY
YAIPAANNVTPRQALQTALPGVTVTYSATAATGYTAQNVATDAQQCQLADVCILFLGSRMHRYLNHNINDRPTYAYDRIMEGEGRDRTSLKLNPNQEALWKDLVARTSK
KIVVVLMHGGGLDISDMMRTAVRTPGARISAVMTTWFPGQGAPGIADVLLGNVAPAGRLPTTWYTESYTALNMADMRMRSSTNYPGRTYRYWRGAAPLFPFGYGLSYST
WAQQAPALVSGRGDGQPGARASITLRNTGTVTSDHVVILFMAYEGPNVIAGRAPNVTLSRTKCAATGRTDLVQSQVGYQRAKGLAPGASQRLTFGLSYVNDFSNSWAGF
GDPVAPCGVYALRFNVGQPIRLRIRLA
>tr|A0A1W2TH17|A0A1W2TH17_ROSNE
MHYISRVALVAASLPWSTLAAATPSVRQRDACTSEPGPDYTAELTFAGCYTDDGDDGRALSLTASLLWPDLTPQLCGNLCGAAGYTYAGVEYGRECYCGSAVRQSAVLQ
DPSACTMQCSGDPSQTCGGYYLIDIHKISNPSSDPVWWSLPDCTKAPLCSNPICNTTLSNEERVAGLIANMTLAEKVGNMQYRAPGVPRLNLPNYNWWNEALHGVANSP
GVSFELGNSTPWSYATSFPMPIHIGASFDDDLVYRIAQTVGKESRAFANNAHAGFDFWTPNINPFRDPRWGRGQETPGEDPLHLSNYVYNLITGLQGGVDPEEMLIIAT
CKHFAVYDVETGRHENNLNPTPQDLTDYYMPPFKACARDAKVGAVMCAYNSVDGTPACANRYLMQTVLREHWGWSQPYQWVTSDCAAIEDIHQNHKYADDAPGAAAAAV
NAGTDLACEGSIYNQLVEAVALNLTTEATIDKSLSRLYLSLLRLGYFDLKSSKYASLGWGDVNKPEAQELAYTAAVEGITLLKNDGALPLPRGVGNVAVIGPWGNATGI
MQGNYQGVAPYLISPLEAMDGKWENVQYALGTSMNTGSTANFAAALDLAAKSDYIIYCGGIDISIEAESRDRVNIEWPGNQPDLITQLAELGKTLIVVQFSGGQVDDSA
ILSNPGVNAIVWAGLPGQSGGTAVADVLDGTKSPAGRLPITQYPANYTSQLVPVDPGLQPNETTGTPGRTYKWYSTPVLPFGYGLHYTDFNVSWAGAPKPTYDIGAIVS
AADGPVDRETFATVSLDITNAGGATSAASDFVALLFLSTTDAGPAPYPIKSLVTYGRAFGIETGETQTLQLSIPVGAVARADETGNLVVFPGTYTLAVDYDGKIPVSFE
LVGEATVIDIVPVPPTDPVAISYLGCYGDEGGSLLDGPSFDLPDTNSAQACADECAGAGYGLSGLQSESQCICGSSLETDAAVLPDSKCDVPCAGAPLETCGGTSSLNV
YSVTPVVHIEPPTYQG
>tr|A0A3E2GT90|A0A3E2GT90_SCYLI
MGRDTSSPPTPPSPMQAFDPESVLSKLSIQEKIELLAGIDFWHTKSIPRLGVPSIRLSDGPNGVRGTRFFNGAPAACFPCGTALAATWNVPLLEEAGRLMGEEAIVKGS
HVLLGPTVNMQRSPLGGRGFESFSEDPILAGMCAAAIVRGVQSTHVVATIKHFVANDQEHERMAVDSIITQRALREIYLLPFQIAVRDAQPGAFMTSYNKVNGIHVNDN
PKFLQDILRGEWGWEGLIMSDWYGTYSTVAAMKAGLDLEMPGPSKWRGQLITHSLMAKTLSPITLDDRVRNILKLISRVGKTGVPQNAPEGSRDVPETAALLRKIAGES
IVLLKNERNVLPLDASKTVAIIGPNAKMAAYCGGGSASLLPYYTVTPFDGISSKATPSNVKYSVGCYSHLMLPLLGPNLRTADGKVGVTFKAFTDPATVPDRAPVDELH
LSDTYMYLVDYYHPKLTEDLYWAEVEGYFTAEEDGEYEFGLTVFGTGKLYLDEEMLIDNESVQRSGGSFFNVGTVEETGVKRLEKGRRYKVKVEFASGVTSKLTDADGV
VSFGGGGIRIGGARVIDAQEEIERAVQLAKEVDQVVLCVGLNSPPSAAANPNTVVVVQSGTPVSMPWASQVAGLLHAWYGGNETGNAIADALYGTINPSGKLTLSFPIQ
VEDNPAFLNYRSERGRVLYGEDVYVGYRFYEATKRATLFPFGHGLSYTRFQMSNLSVSVDAGSESGKLNVTLDVENTGSRDGAEVVQVYISQRAPSVKRPMKELKGFVK
VYLKAGEKRTVAVPGIDVRYATSFWDEAREMWICERDTFDVLVGESSAHTPLKGSFEVGETRWWSGNPTKPPEPERQQKDQQEALTFLPHGQNSQDDLIIAQACREQVA
IRRRHRYEDRQRIEHSITPSLNALVARNIPEPPPSSVTSSANNIPVLESCPVITPQSQLETVILGSTIPSSTHIPSVSSTMPILVVIPEIIPQNEQHAVSEDD

>tr|A0A0D3EGP3|A0A0D3EGP3_BRAOL
MSYPMQTKGNRALSSVSTLLLSILFFISKPSNAQSSSPTFACDVTKNPSLAGYGFCNTGLNAEARVTDLVGRLTLEEKIGFLVSKATGVSRLGIPDYNWWSEALHGVSD
VGGGSNFTGPVPGATSFPQVILTAASFNVSLFQAIGKVVSTEARAMYNVGAAGLTFWSPNVNIFRDPRWGRGQETPGEDPTLVSKYAVAYVKGLQGTDGGDPNLLKVAA
CCKHYTAYDVDNWKGVHRYTFNSVVNQQDMDDTFQPPFKSCVVDGNVASVMCSYNQVNGKPTCADPDLLSGVIRGQWKLNGYIVSDCDSVEVIYASQHYTKTPEEAVAK
SMLAGLDLNCDHFTGQHAMSAVKAGLVNETDVDTAISNNFATLMRLGFFNGDPKKQPYGNLGPQDVCTAENQELAREAARQGIVLLKNSPGSLPFSPSAIKTLAVIGPN
ANVTDTMIGNYHGVPCKYTTPLQGLVETVWAKYQMACPNVACTEADIDSATSLAASADAVVLVMGTDLSIERE DHDRVDLFLPGKQQQLVTEVAKVAKGPVVLVIMSGG
GLDVTFAKNDPKITSIMWVGFPGQAGGLAIADVIFGRHNPSGKLPMTWYPQSYVENLPMSNMNMRADNSTGYPGRSYRFYTGETVYAFGDGISYTHFNHRLIKPQRLVS
LGLAKSHPCRTSKCQSVDVTGPYCGKAIEVELRVRNAGEREGTDTVFLFTTPPAVHRSPVKHLLAFEKVRLGKKEKAVVRFNVDVCRDLSVVDETGKRKIALGLKALFL
QLKMDTTKDDDFTFSKAVQPDSEVVLDAKDLASNVDAIALKDGLDPHLNQVSPKENTVGSLSFTVTDSSSEMILKARKPITRTKVPFEKGYSQMDWLKLTRTHPDLAGL
KGESNRRLISMDEVKKHKSGDSMWTVLKGRVYNISPYMNFHPGGVDMLMKAVGRDGTFLFNKYHAWVNFDVLLEKCLVGVLDDSKVMSKC
>tr|A0A1M6SRG1|A0A1M6SRG1_9FIRM
MTQKKTGIPLEGFAEFSRKAAAEGAVLLQNEGMVLPVKPGERVSIFGRIQKNYYRSGTGSGGSVNVAYTTNLLDGMRSKKDIIVNEELAAVYEKWLIDNPFDNGGGVWA
GEPWNQKEMPISEEIAGAARKQSDKAIIVIGRTAGEDKDNDDTPGSCRLTEEEQAMIKAVTGYFEETAVVLNVSNIIDMSWLDSAEYKYPIKSVIYSWQGGMEGGNAIA
DVLAGEVTPSGKLTDTIAYSIQDYPSTKNYGGEDKNLYQEDIYVGYRYFETFCPEKVHFEFGYGISYTDFSSTVLKAEKVRENGGDYFVFQVKVKNEGGTYSGKEVVEV
YYEAPQGKLGRPVRALGAFGKTALLKPGKEEVLIIKMPAAAMAAYDDSGVTGNPYSYVLEAGEYRLYVGSSIKKASLVKIDGKESSQVEKLTVIEKLQQAMAPTEDFTR
LKPGKKKENGSYEITYENVPKLAYSIEERIKNNLPEDIEDTGNVKYLQDKKHLQNEKYSRDMKQPQDKGYLLKDVAEKKVTMEEFISQLSNEDLAALVRGEGMCHPSVT
PGTASAFGGVTDRLLSFGIPLACTSDGPSGIRMDGGYKATQVPIGTLLAATWNPELVEELYTLEGKELLSNSIDMLLGPGLNIRRSPQNGRNFEYFSEDPLITGKFASA
VVKGIMKGGSNATLKHFACNNQEKFRAKVDAVVSERALREIYLKGFEIAVKEGGANGVMTSYNPVNGHWAASNYDLTTTILREEWGFQGIVMTDWWAIMNDVVSGGAAD
RKNTHFMVRAQNDLYMVVSNFGAEVNAYRDLTEEALKEGTLTRGELQRCAKNICEFLLKSPSFKREESFSEEAVFVEPGNFDREAGNILEGEDTFVPVHKTPQSVIEVK
EAGFFRLMVKIRSFGDELAQTSCSLLLNDTQVATIQIGGTDGHWIKQKLVKVELKEGMYSVELKDVKQGLEIEWVEFKRIS
>tr|A0A1X6N6V9|A0A1X6N6V9_9APHY
MTVLLALSCLALAGQAFGAFPDCANGPLTTNTVCDTSATPLERATALISLFTLEEKINNTGNTAPGVPRLGLPAYQWWQEALHGVAESPGVIFAPSGEYSYATSFPQPI
LMGAAFDDALINHVATIVSTEARAFNNANRSGIDFWTPNINPFKDPRWGRGQETPGEDPFHLQSYVYNLITGLQGGLDPEYKRIVATCKHFAAYDLENWEGNVRYGFDA
LVSLQDLSEFYTRSFRTCARDANVGSFMCSYNAVNGVPSCANSYLLQDILRDHWGWTNEDQYITSDCDAIQNIYEPHYYTATRAETVADALNAGTDLDCGEYYPENLGA
AYDQGLFTESTLNRALIRQYAALVKLGYFDPADIQPYRQIGWANVSTPEAEELAYTAAVEGITLLKNDGTLPLSPSIKTIALIGPWANATTQMQGNYYGVAPYLISPLM
AAEELGFTVYYSAGPGVDDPTTSSFPAAFAAAEAADAIIYAGGIDITVEAEAMDRYTLDWPGVQPDFIDQLSLLGKPLIVLQFGGGQIDDSALLPNPGVNALVWGGYPG
QSGGKAIMDIIVGNAAPAGRLPITQYPLDYVYQVAMTDMSLRPSPTNPGRTYMWYTGTPIVEFGFGLHYTTFTASLSQPSAPSYDIATLVSLCSGVAHPDLCPFASYTA
NVTNTGSSVTSDFVSLLFLAGEHGPAPYPNKVLVAYDRLHAIAPLASQTTTLNLTLGSLSRVDDYGNTILYPGEYTLIFDVDAKSTRRFLTTGPNRNNARPLRGGDNGY
EVMRLSPTFIIPFLAPLRATQHSPLNRLASTLPTSVTAAVQVKLRYFHYSRPAMSGPSKTKNDTEWKAILSPEQFRILRQKGTEPAGSGKYENFKGEGIFACAGCGTPL
YKSTTKFDSGCGWPAFFDAIPGAVNRHEDRSFGMTRTEITCTACGGHLGHVFKGEGYKTPTDERHCVNSISLNFVDEPKA
>tr|A0A166DFG8|A0A166DFG8_9AGAM
MPRQRQFIARGISLILISTFLYSTSFISLPSTLFSSSDVISSTLNHALERRQSPGSPTGTALSTGPTSPLILPSPNVTATGPWAKSIIRAKQFTSSLTLPEKINLTTGI
DVNGRCVGNSGSIPRLNFAGFCLEDSPLGVRLTDYNSAFPAGINAAMSWDKDLILQRGQAMGAEHRGKGVNVQLGPMMNLGRDAAAGRNWEGFGADPFLAGVASALTIQ
GIQSQGVIACAKHYIGNEQEHFRSGSGGGENSVSYSSDIDDRTLHEVYAWPFAESIKAGVGSVMCAYNRINGTYACENSKIINGIAKEELDFKGFLLSDWAALESGAAS
ALAGTDMNQPGFIGYGIGPQNEPNPSTANNSFWGEALAQMVANGTVPEWRVDDMVVRIMSAYYEMGQDKDYPAVNFNQLGPTQPGNEHVNVQGNHSILIREIGAKSTVL
LKNLNGTLPLNEKKLPRNIAIFGSDAGPNPDGPNGCGDRGCDQGTLAMGWGSGTANFPYLIDPLSAIANWVHENSPDTMIEYVTNDYNYNQVTSLATQADLCMVFANAD
SGEGYITVDGNAGDRNNLTLWHSGDTLVTTASSHCPNTIVVLHTVGPVILEKWIESENVTAVLFAGLPGQESGNSIVDVLSGAVNPSARLPFTIAESAFDYSANVVYSN
PTNEIIIHIPYDEALEIDYRHFDANGIVPRFEFGFGLSYTTFAYSGMSITKASTFGKRYEPHYIFERDAGDSTTTSWVTTTETTTITGSSTPSSALSTASVSLAPPKPS
PSSASSSSSSTTSASSSSISTNSSSSAPISSSSTNSSTILSSSLPPTPTSAPVNASLSISGTGPAVTPTGGIGGPTSLYDTAFTISFTIHNTGSFDGDEVAQLYVGFPD
GSGEPPKVLRGFENVWVGKGGRCNVQIVLRNKDISVWDVVKQAWVILSGRFTFFVGASSRDIRMSQSVTVTKLVTLEGP
>tr|A0A5C8M3W2|A0A5C8M3W2_9BACL
MHRNSFTQNAINNIWEDLKQKMKKTTSYLASLLMLSLLPFNVANVSAADLEIYKNPNMPVEQRVQDLLGKMTLDEKIGQMVQAERASLGTVSDIKTYFIGSILSGGGSV
PAGNTAAAWKTMVDNYQNQALSTRLGIPMIYGVDAVHGHNNLKDATMFPHNVGLGAANDADLVRRIGSATAEEVRATGVHWTFAPVISAVQNIRWGRTYEGFSEDPQIV
AKLGVAAVQGLQGNPEDPGFLKGKKIVADIKHYIGDGLTDNGVNTGNITVPVPDGKTLNQAKDEYLAPYLAYYKKAIDAGARTVMVTYSGINGLKTHADTHLVTDVLKG
QLGFTGFVVSDYNAIQQITVDDQGNNISGAANYKKQIKASVNAGVDLFMLSDKGNTTSSSGAGNGWIGFINNLKSLVGTGEVPMARIDDAVTRNLRVKFENGLFEQPLS
DSVTNPDSVIYNQDHAALAREAARKSLVLLKNSPNVLPLKKDGSQKIVVIGDKANHIGYQLGGWTISWQGAGNTVTKGTNILDGIKQVAGPGVTVDWDQNGATATAGHD
IAIAVIGEDPYAEGNGDRTGTSLTYGTGGGASSDTSIVANLKAAKAANPNLKVVIILVSGRPLVITNDLANWDAVVEAWLPGSEGAGVADVVFGDNDFQGKLPYTWPRT
FDEINAKDKSNPLFPLGYGLTYAETKSGTVLSGTDTAIPGQEFDLTYSVRNVAQQVYAQDVTVTYDPQQFEYVSASPTKDGLKIVDKKATSGQVRFILANVGTNHDASG
DWLKLKFRAKPVQSATSAINITNVVIADAKGVETQLSGTSHSIQFIIVDKTALSTLIANAQSKHDTSVEGTRPGQYPAAARAALQTAIDTANVVLNNAAATQADVTQAV
TNLNAAIQTFTSSVVNRVPSDTSGDGVISIGDLAIVAAAYGKTSADTDWDLYKVADVNNDGKVDITDLAIVANSILQ
>tr|R5HNG1|R5HNG1_9FIRM
MSMSTIGVPLEGFGVYCRAAAAEGAVLLKNEGHMFPLKKEEMVSVFGRCQFETYRSGTGSGGAVNVPYAVNIYDGMRESGSFTLNEELADIYRAWLKDHPFDNGGGGWA
AEPWHQKEMVITEEIAAAAAEKSEKAIFIIGRTAGEDKDYANEAGSYLLTPEELENLKVLTAHFEQVAVLLNVSNIIDMSWLKNPVYKDHIRSVLYIWQGGMESGNAVA
DVLSGKVSPSGKLTDTIACSLADYPAANDFGDTVRNFYTEDIYVGYRYFETFCPEKVMYEFGFGLSYSKFDIEILKAETVTEESEVGGAWGSHGVSIDNGTCAGKEVQI
TICVKNTGDCRGKEVVQVYVQAPQGKLGKPARELKAFAKTKELAPGEKQKMTLHIPVKNLASYDDSGVTGHKSCYVSEAGAYVFHVGNSVRNTKIADVDGKGAYIVKEL
CVTEALQEALAPTEAFERIRPGQSREDGSYQQEKEAVPQQTIRLQERIEAHLPEQLAITGDKGIRFSDVAEGKADLDTFIAQLTKEELATIVRGEGMSSPKVTPGTAST
FGGVSDRLYEYGIPAACCADGPSGIRMESGLKATQLPIGTLLACSFNIPMMEELYVMEGKELVANEVDTLLGPGINIHRYPLNGRNFEYFSEDPYVTGCFAAAVTRGIK
KGGSFATVKHFAANNQETARHTVDSVVSERALREIYLKGFEIAVKEGEASSIMTSYNPINGHWTSSNYDLNTTILRGEWGYEGIVMTDWWASVNDVVKGGKQDHHTLSS
MVRSQNDLYMVVNNNGAEINAMGDDILEALDNGKLTIGELQRSAKNICRFILNAPVMKRPLRPLEEVKAYEPLQGAGSEGATWESDISIVPEDKVSQKIYVNQSGVYNV
VVKFISPQSNLAQAAANLYLNDELFFTLQTSGTEGRPNTQKICRVELKQGYYEIKTEVVKPKLEMEWIQLWK
>tr|Q0UXS2|Q0UXS2_PHANO
MGFTNAEFNGCNGKSGGVPRLGFPGYCLNNAENGVGGAEGVNAYPAALHVGASWNRELAYARGMHMGREFRRKGVNMALGPSIGPLGRSPKGGRCRNWEAPSNDPYLTG
MLIHDTTAGMQKYVIANIKHIVGNEQEASRKYPRFLPAPNHNASISSNIDDKTMHELYLWPFMDALRAGGASVMCSYNRVNNSDACQNSKVLNGLLKQELGFQGFVVSD
WFMQQSGVASALAGLDMVMPIAPYWADGNLTQMVNNGSVSMTRLNDMVTRILAPWYKIGSQSEEDGRPPGHGIPASLVQPHEFVDARSPDSKPVILQSAIEGHVLVKNV
NKALPLRKPKFISIFGYDAAGQFMNTAEAAGFNLWKMGMRNALQFQNGTTFTAAALDLLFGSSLEQTTTGPEIALNGTLFTGGGSGAVVGSIDAPLDAFKRQAYDDGTY
LAWDIQTPNPTVNAGSEACLVFINALGSESWDRRNLSDAYSDGIITSVASQCNNTMVVIHSVGVRLVDEWFDHPNITAVILGHLPGQDSGRALVELMYGRQSFSGRLPY
TVAKQEADYGHVLDPVQPSDKTPYYPQVNFTEGVYIDYKHFIKEGIEPRFAFGYGLTYTDFEYSNLQIDVDANATSLRLPPNPEHILQGGIDSLWDEVARVRCTIENIG
DVAAKEVAQLYIGIPGGPEKVLRGFEKQLLVPGERGVFEFGLTRRDLSTWNVMEQSWELQRGDYGVFVGKSVLDVPLTGRFEIGDVTQCYLASNRTLGEDAFASQGTTQ
NFQGQQGYLGGAISIVAWCRGVGRALLPARQARRRQRKAYRHSVPTTQFHLFPSNFAYRTRTTANKLAYDYQLCPPASSPSDQSHFDILSLVPHALLAKHHQPIMSDYV
NEFDLTDFMDTPPTSPGFEQPTTSECLDQIDWENPNYKDLASVDWNALHEEIIGVVLGEPADEQLQPDDR
>tr|A0A3N4HIL4|A0A3N4HIL4_ASCIM
MTDSTPSLGPKSTLLANQEPSSDTPNLPQTGAAGPPTSTGSKRKFVYIGLGLAAILAIALGVGLGVGLKKKGSGSDGEKSSGDERKTNLPALELVKGPEGDWKAVLGGL
DPEWAPHYAKAAALVKTWTTLEKVNVTTGIGWSQGPCVGNTGTAPGLGSICLQDGPLGIRFAPLVTVFPAALTTAATFNKDLLFLRGQALGREAREKGVNVLLGPCIGP
LGLFPEGGRNWESFGADPYLQGVGGRLTVRGIQGEGVIANAKHYVGNEQELFRRRDEGRNAGWGLDGPISSVLGSADLRETWAWPFQEVVEEGVGSIMCAYNSVNGSQS
CSNSYLLNEVLKGEMGFQGFVVSDWLAQDTEAEVSANAGLDMTMPGEKHSFTAGDSFFGAELTAAVVKGEVSQARLNDMVLRIVATHFKLSQDSPSYPRPNFSSWFRRS
HGPAYVGAGDNAPGQIILPLNANIDVRGNHSSLARQIASEGTVLLRNENSPNSTLGLPLGKKKVLNLALFGSAAGPNPNGPNSCTDRACNRGALGQGWGSGSVDYTRFI
TPLESIQSRLYAPLSSDPPAGLFDFTLDDTNRDLLRQKAVAMQEVNGTCLVFVTSDSGEGYGSSEGHAGDRNDLELWHGGAEMVLTVATHCRDTVVVIHAVGAVNLEPF
EGHPNVTAILHAHLPGQEHGSSLTPLLFGDEEPSGRLPYTIFKKEGDYPRIEKEANGRVPQSDLRGMAAFDYRNVTSEEQEKTVRYPFGFGLDYHPDPITISDVEVKEE
FKPIRFWANGGNSSTASPVTIPDRDLRKEPFTAPKGWRFIKNYIYPYLPAAPKREDITTGPSANLTTRALPVKPRKVLEVTATLKNPSSKARMAVPQLYVTFPGGKGRN
LRGFEKVRVEAGGEEVVKFGVSEREVSRWNVGRNQWEVREGRYRVEVMMDSLGRGGRKEVEWRGEY
>tr|A0A1G4RFG7|A0A1G4RFG7_9FIRM
MPEYILDWNKYTKAAMDVATEGAVLLENRDNVLPLADGARVALFGRMQTHYYKSGTGSGGMVNVEHVVDIREGLKDSGRVVLDEELMGIYDKWDEENPVDPGIGWGNER
WSQEEMPVTEELVKSVSMRNDTAVIVIARTAGEDRDNSYSKGSLSLQDGEEELIRLVTEAFAKTVVLLNTGNIIDMSFVKKYRPQAVMYIWQCGMIGGTAAAKLLTGEE
NPSGLLTDTIARTFDDYPSTPYFGDQDSVRDQYTEDIFVGYRYFTTFNRAAVMYPFGYGLSYTTFDISGAQFVMKDGKVIVAARVTNLGEVPGKKTVLLFADPPAGALA

KPSRVLVGFTKTYEIMPGGTCPIEIEADMKSFASFDDDGRAGCGTGWILEKGIYDLHIGGDSLNSDIVLSFTLDETRNIEAVEPVMAPVEEFERFTVGDDGKLAFEKVP
TRTKDYYSERFSLVPPEIPQTGDKGIKLIDVKNGKASMDDFIAQISDEDLCLIIRGEGMSCPKVTTGTAGGFAGVDPVLNAMGVPACCCSDGPSGMRIDSGKKAFSIPN
GTCIASTFNLKAVESLFGWFGIEMISNRVDTILGPGMNIHRHPLNGRNFEYFSEDPLLSGKMASAQIKALEQNGVTATIKHFCVNNRETRRRDMDSVVSERALREIYLR
GFEIAVREGGARSIMTSYNKINGTYAASHYELNTLLLREQWGYKGITMTDWWAYIVGTPPANMHHELRDHAAMARAQNDLYMVCSSVDPAHLADSDCYDELMAGNITRA
ELQRNARNILNFAMNTPAMDRLNGDKAVVNGIDCPFHDESVDVDANYFYDIEANPVINVLEQVDTSTGKDMIIGISCETNGVYEMEFTASSELNELAQIPMTLYFTSIP
FVVLTWNGTEGRDDTRTAKVIVNSRHVVLRAHLGGPGVRLKTLKFKFVSAVTQEIMDEILRKGEQ
>tr|A0A369K5A1|A0A369K5A1_HYPMA
MGCIFDRCYRSANSYLPSRPANPSSLAARSLSRENSAFRDSIPILRYTDNPVLSLSDIGGRTKYIHTSLQVYYPIFKNPILQKRLLPGQHSHRQRTYQASNTYSREAPP
LHLRVHVLQWKRDISSSSSKLGRLRPLPQPVTSTETACHSLFCSAQQGPSPRLYKSGCSHPMDRNSFGWEIALFGSRALVTAWAMEGRQQTFISLLRLHCALKHLRLED
AFALRTWEEARGLANTVVAQMTLDEKLGIVRGTGQLNSMRRCVGDTTAVPRLGIPSICFNDGPAGLRLTKGVTGFPSGINAAATFSRRLMRARGKALGEEFRGKGVHVF
LGPALDIMRNPKAGRGWESFGPDPYLNGEGAFETVTGVQSVGVMACAKHLIANNQEHWRYGLSANVDDRTLHEIYWYPFLRSIEAGVASVMCSYNRLNGTSSCHHAGLL
GPNGLLQKDGFQGFVVSDWGATHDSASDNANAGLDMEQPGDFIVIGGGAFIGLKSNVNSGMVSVTRLNEMVSRILSPWYRLGQDSEYPSVNFDSQKPDGSGPLNLGVSV
RSDAHTALVREIASASAVLLKNNRTITTGTPSGNTVRGLPLNASRIKSIAVIGQDAKMPNLMCNELNECNDGTMSVGWGSGSNSLEFIVPPIDAITSFAGSSATITSSL
SNDLDAGVKAARGKDVALVFVNAMSGELGFYTVVVGNQGDRNDLDLWWKGGSLVERVAAVCNNTIVVVHSVGPIHMGWSIHPNITAIIYAGAPGEQTGPSIVDILYGKY
NPSGRLPFSIADKEDDYGTSIVYNSLGFPDVSYTEKLLLDYRYLDSKSITPRFEFGFGLSYTTFSYSSLTIIDSDTSPTVKFTVTNTGAFAGAEIAQLYLVYPTSAGEP
KKVLRGFEEVVLNVGASSSVTITLSQRDISIWDTPTQSWVRPGGFFRVYVGASIKDIRLAGGF
>tr|A0A1Y2GSV0|A0A1Y2GSV0_9FUNG
MIGDHSTKASQHVVSDGRCVRAGSKSLHIKLLIILLSVNFLVAQRVGAVAESRVAASGSVKSPGLIFAIEEYQQMYNKSIDWNQNQSFNPQQTSNVDQIVDQNTFGGRS
PDASYPIHDSDGCSSSGCSSGKTQEGKDSLRPTTEVDPDIRELVESMSLKELAGQMTQIQIGMLLDSKGELDVAKAQYWIGEWGVGSFLDTPTNHGGKYISYSPKRFSK
IVDDIQKVALSTGKGIPVIYGLDSVHGANYVDGAVIFPQQIGLAATFNTTLAYEAGRITAKDTRAAGIPWVFAPILDIAVHKLWPRVYETFGEDPHVSSTMGAAIIRGL
QGNYKKDRTRVAGCMKHFIGYSASRNGQDKASAWIPDNLLMDYFVPPFRAAVNAGVATAMETYIDVNGQPVVGSHFYLTELLRNQLKFEGMLVTDWQELDRLYTEHRSV
PSLKEAALQCLKQTSMNMIMVPESKSFSENAGLLVQEGKLGRERLVSSVAKVLQLKKDLGLFDQPFSDPKLLSYVGSKQDIDAAKDAVRESITVLRNNKGALPLRKNQT
KKIAITGPAANSLRALSGGWSIKWQGAETDEWFQGRGETILSGLIKEFGEGHVAYAESIDFDGNSISNSTASAPLDDAIQGADSVILCLGEGPYAEIVGNINDLDLPNG
QLDLVRKVSERIKGTSTKLILILVEGRPRGLQDVVEKVDAIVLAYLPGPWGGHPIAEILSGAVNPSGRIPMTYPNGSSDMTTNYYRMGVDPYKPLFPFSAGISYSLVEY
SDLILTSNVMYLNASSHNSTATQKSSLENGFKTSTIVNSNDINDPYHQNNYPSNRHHETNRTSNMGKSTSPFIFAKINIHNKSSYPVKETIFWYVTQDYRSDIMPEAFL
LKYFQKVMLKPYQSMEVQFKITPNTLTYHGRDLERKVERGSFTLTVNAMRPEAKSTKFNLV
>tr|Q1YR72|Q1YR72_9GAMM
MTVIDQNQGLVLKGFQMRSSQLHNNNKSITRLKTFGRGVAVLFIALSLSACGGGGSSAGASVTPPVTPPVTPPTPPADNIPVAVADSFNTDQNATLNANLAANDSGLGD
SPITFSIDTAPSNGTLTLTESGSATYIPTPGFSGNDSFGYTIVDGDGDQSTAIVSITVVTNNEPFSWPQISSDVTDNVDTLVSLLLAEMTTAEKVGQMVQAEISNVNAA
QVRDFNLGSVLNGGGTWPNGKNSSIADWVALADSFYEASTDISDGGVGIPAIWGTDAVHGHNNVIGATIFPHNIGLGAMNNAPLMRQIGETTALEVAVTGIDWVFAPTL
AVVRNDSWGRTYESYSEDPEIVRAYAGEVVSGLQGDDSDRFGAAHVIATAKHFIGDGGTQNGIDQGNTVVTEVELRDIHAQGYLSALAAGAQTVMASYNSWNGSKLHGD
EYLLTEVLKQKMGFDGFVIGDWNGHGQVPGCSDGQCAQAIMAGVDMMMVPADWQAFIQNTIAQVQNGTIPMSRIDDAVTRILRVKMRAGFQDKVKPSSRLHANNSSLIG
STAHRDIARQAVRESLVLLKNSDSILPLAANSNVLVAGSGANNIGMQSGGWTLSWQGTGNSNSDFPGATSIYSGIESLVNAAGGTTRLSANGSFSSSNRPDVAIVVFGE
SPYAEGVGDLNNIEYQAGNKSDLALLESLRGQNIPVVSIFLTGRPLWVNKELNASNAFVAAWLPGSEGAGVAEVIFKTASGEINYDFKGKLSFSWPKRAEQTVINRNDS
NYDPLFAYGFGLTYQDSDTLGDDLDTADSGTGEVEILFSVPGTIEAEQYIAMNGVRTEVSSDSGGGTGGGVNVGYIDIGDWLEYSIDVQSSGSYLIEYRMASNVGSSGF
KTLVDGEEIDQQAVPNTGGWQNWITQSETVELLAGTHTLRINAIGPEWNLNWIRLNLID
>tr|A0A1U9NNJ8|A0A1U9NNJ8_9BACT
MAKRVFFVVFAAIFFSVQFCYSQSQSGKEKMPAYKNANLPVNVRVKDLLSRMTLEEKVRQTWEQHTGGLEIVDGEIAQKDIDKLFEGYSYGTLQARFGGPIKEQAIINR
DVQKYALEKTRLGIPVLPMHETLHGILATGATIYPQTIAQGATWNPELIKEMSSAIAVEGAAAGISQSLSPMLTLARDHRWGRVEECFGECPKLVAEMAIAYIKGMQGE
DAEKGIEPGKMACMLKVMAGYEIPSGGINIAATSLGEREFRSLYLYPYEQAVKRAYPYSVMPSYNVVDGLPAHANHWLLTKVLRDEWGFEGYTYSDWGGVPFNHSLHKI
TANAKESAIVCLKAGNDLEAPGPSCYKYLVELVKDGRIDEKYVDQAAGRMLKVKFLCGLFDGVPEPVDVDKLDEKIHTAEHVALARRVAEESIILLKNEGNMLPLKKDK
MKSIALIGPNADQVQFGDYSPTKSNHMGVTVLEGVKGFLAGTDVKISYAKGCGITDPDRSGFDEAVETAEQSDVAVVVVGDTSMIIGGGVGGVIGNESEQKYLATAGEG
YDRTTLTIPGVQEDLVKAVVATGKPVVVVLVHGRPFAMPWLKDNAQVILDVFYPGEQGGNAVADVLFGKVNPSGKLPVTLPRSVGHLPQTYDYLPCGRGYYGVPGTVEK
PGRDYVFSSPEPLWPFGFGLSYTTFAYSDLVIKNAVAGMSDKIRFSFTVKNTGSRAGKEVTQVYYNDVVSSTVTATKRLIRFKKIELGPGESRRLSFEIDPDELAIWNM
DMERVVEPGAFDLMAGSSSDDIFLKSSFSLMTPVMIERDKAKFGLAYGKKAWASSELAAYPAVNVTTGSESTRWAAGQGGEQWITVDLEQPQVIDNVKLYWETAYGKEY
KIQLSSDNRTWETVSHVKNNDGGLDEISFSPQKARYVRMYGIKLATRWGYSLYGISVN
>tr|R9L210|R9L210_9FIRM
MNRYMLDMGRYQALARQAAAESCVLLENENNALPLKNGEKIAVYGRSAFHYYKSGLGSGGLVNTAYTVGILEALKDSSDVLVHQELLDIYEDWIAQNPYDEGEGWGKVP
WSQEEMPVTDRMREIAKEADASLVFIGRTAGEDQDNFAEAGSYLLTEQESRMLEEICKASKRAIVILNVGNIIDMSWVADYHPQAVLCVWQGGQEGGNGVLDVLLGKVN
ACGKLTDTIAKSITDYPSTANFGNESKNYYVEDVYVGYRYFETFAKDCVLYPFGYGLSYTTFSVQCEVAGVTQKHCMVRGSVKNTGGCAGKETVQVYIKVPQGKLGNPV
RKLAGFFKTRILNPQEEQAFELVIDKYDFASYDDSGVTGHPFCYVLEEGTYQFYAGTDVRSAALIGAYEQPFAVVDQLTQACAPTEAFRRMKPVMQSGTASEAEIFENE
PEAVPLRAASYHRMSRDKGTAEIAFSGDKGYQLVDVLDQKVSLDEFVAQMTDDDLIHIFRGEGMCSPRVTAGTAAAFGGVTWHLQDLGIPAACCSDGPSGIRMDCGTKA
FSLPNGTALGCTFNTELVEALFELLGQELRMNKIDSLLGPGINIHRNPLNGRNFEYLSEDPYLTGKIAAAQIRAMESSQIAGTIKHFCANNQEKGRRTSDAVVSERALR
EIYLKGFEMVVRDKNARSVMTTYGAVNGIWTAGNYELCTQILREEWGFDGIVMSDWWAEANYEGAMPARTVKAPMAAAQNDLYMCVCDAAQNPENDDMEQQLASGYITR
GELQRNAKNILRFILKSPSILWASGRITQEEQEAVSKAAVQQDMVTDMVYYAADEVTQEIVIDGADLHPKAGMSDVFGIQVNKPGTYSISFVLKSDLGRLAQLPLSVFY
DNQLKMTVSVQGTNGETVTHRRELECFFGNNHYMKFYYGANGLEIDKVIFKFERGCE
>tr|I0X956|I0X956_9SPIR
MTFSETICRAAEEGAVLLKNEECLLPFTSQDNVAVFGRCQKDWYRSGTGSGGSVHVSYTTTLIDSLLELSLSDGSMPHIDVMLAKTYSNWIQENPYDNGGGAWGAEPWC
QKEMPLSKELLEETGANNSKAVYVIGRTAGEDKDNMAEKGSWYLNDDEKAALKAICGTFEDVCVVLNVSNIIDMSWINSPEFKGHIKAVLIAWQGGQEGGRAAARVLCG
LANPSGKLSDTIAMSIDDYPSTNHFGSKGDIFYKEDIYVGYRYFSTFAPERILFPFGFGLSYTSFNTSIKKSEFKNGKITVQVEVKNSGNFAGKEVLQAYISAPQRKLG
KPARVLADFKKSSELKAGESQTLELSFSLSDFASYDDSGLTGYQFAWVLEEGLYRVFVGSDSLSATEVFFDGKSGINLSKTEVVEQCRQALAPESDFMRLHPIACADGT
FVAKEEKVPKMKVDLAERIKAGLPEEYPFTGDWGIRFSDVLSDNSKLDAFIAQLDDKELAAMVRGEGMMSQKVTVGIAAAYGGITQSLRNFGIPAAGCSDGPSGIRLDT
GKEANLMPIGTLLACTWNPNIIEDLYTFEGKELVQYEIDSLLGPGANIHRSPLNGRNFEYYSEDPLLSGMIAKAALRGLNIGGASGTIKHFAANNQETWRRTSNSIVSE
RALREIYLKPFEIAVKSGEVKSLMTSYNAINGHWTASNYDLVTTILRREWKYQGLVMTDWWATMNDCVKGGSESIKNTASMIRSGNDVYMVVDNDGAEKNMYEDNTLEA
LKNGSLTRAELHAAAKNILNFIMISPVAKRPLRPLKIFKSFQSVLKEQPSDALLVDEGKDFLPEGESFYLNAQHDAMYNISGTYSKEGDDLSQSVTNILIDGSPAASLE
CRSTAGIETTVNAAQVKLEKGFYKITLEHTKPGITVKKLNISSQVITPVSLGVISV
>tr|K0K350|K0K350_SACES
MLRPVCRSASCGGFVRRPEQRPDLHGSALTKVLTGPPPPGQDRREPAAALRRRTDQQMSRRHLGTALAVLLATGLTTGIAGADPSPTRPVVVGEPRTDHGCARIEPTLP
TYADWPRVRSRVHGDPADERRIAKIVGSLTLAEKVGHMTQPEIAAITPDEVRQYGIGSVLNGGGSWPDRDKHAAVRDWLSLADAYWDASKATRAKIPVLWGIDAVHGNN
NVYGATVFPHNIGLGAAHDPCLVRDVSEATAEQIRATGQDWAFAPTLAVVRDDRWGRTYEGFSEDPRITRAYGYEAVRGLQGDGRRRVDVLATAKHFIGDGGTLGGKDQ
GVTPSSTAEMINLHGQGYYGALAAGAQTVMVSFNSWTNADLGIKEGKLHGSKLAVNDILKGKIGFDGLVVSDWNGIGQVPGCTNAGCPQAINAGIDVVMVPNDWKAFIA
NTVAQVESGQIPLSRIDDAVTRILRVKLRAGVLDGAKPSAREHAGSAKALEARRLAREAVRKSQVLVKNDNRVLPLKPRSKVLVVGKSADSLQNQTGGWTLSWQGTGNT
NADFPNGTTILGGLREALGAQNVVFSETGDVDPAGFDAVIAVIGETPYAEGTGDLGRRSLEAAKLYPRDLAVLDKVRGRGAPVVTVYVSGRPLHVNKELNRSDAFVASW
LPGTEGGGVADLLVRGRHTYPGFTGTLSYSWPRSACQTPLNPGQAGYDPLFKPGYGLRSWQTGRVGKLDETAPASCSGDGGGGTATEDLELFNRQDIAPYRGFIGSPDN
WSGTEIGPDGEAAHSTITVRPADVNVQGDALRTTWTGTGQLYLQNPAGGNDLRGYLNADAALVFDVVVHRPPAARTVVSAHCVYPCSAEVDATTLFGGLPAGSKATVKI
PVACFASRGLDLENVNTPFLVYTEGAFQASFANVRWVPKAARDPDARTCSELT
>tr|A0A1G5C4C7|A0A1G5C4C7_9FIRM
MSKIWDYKKFQEVIRAAAAEGIVLLENEGNVLPLKKNEKIAVFGRSQMNYYKSGTGSGGMVNVDYVVGIFEALENSRYFKINKAVRKAYEEFVKENPFDTGIGWAAEPW
FQKEMPLTEDFVEEAAKESGTAIFIIGRTAGEDQDNKNEKGSFLLTDEEKDALSKVCRAFDKTVVLLNVGNIIDMKWVKEIKPSSVLYVWQGGQEGGNAVFDVLSGRVN
PSGKLPDTIAANISDYSSTENFGSDKENYQTEDIYVGYRYFETFDKESVLYPFGYGLSYTSFDIAPGAFSFDKKDGISLEVLVRNTGDVDGKEAVQVYVEAPMGKLGKA
SRALCGFNKTDLLRKGDSQRISFEIPLYYLASYDDSGVTGHKSCYVLEDGIYNIYIGSDIRSAKLAGSFTVEKTEVVCSCEEAYAPIYDFKRLKAVCMDGSVEKTYEKV
PVRTVDPNKRSKDRLPKALPFTGDKGYKLVDVENKKVSMDEFIAQLSDEDLCCMMRGEGMCSPKVTAGIAGAFGGVTDKLAAFGIPVAGCSDGPSGIRMDCGTHAFSLP
NGTCLACTFNEELNEELFNWEGLDLRRYRIDALLGPGMNIHRNPMNGRNFEYFSEDPFLTGKLVAAQLRGLHKYDVTGVIKHFAGNTQEFKRHNVNNIVSERALREIYL
KGFEIAVREGNARAIMSTYGPVNGIWTASSYDLLTTILRGEWGFDGIVMTDWWAMGNNFAGDAGDYKNVSSQVRAQNDLNMVNGNAETNSNGDDLEKALQDGRLSRAEL

ARCAENILRFLLKSPAYRHLLGEESELDEELKSAVTAEDVRLQEIIDVKMPGNEVSLDTSLIKTGRGDETTFLLTEVKRSVVELELVLKADDQPDVAQLPISIFQDKGL
VKTITLTGMDRDWKTVKVTLFPSYIGTFYLKFFLTQGGLCIKSAKLRVVEEL
>tr|C4L039|C4L039_EXISA
MKQYTLDWTRYEELARQAVAEGVVLVKNEQKTLPLQPETKLAVFGRSQFHYYKSGTGSGGMVNVGHVTTPLEALRERPDIHINESLYATYEEWVKENPFDQGVGWAGEP
WSQQEMDVTDELVEQAAQKSDVALIMIGRTAGEDRDNTADPGSYLLTDIEHAMIERVTKRFDKTVVVLNVGNIIDMKWANEPSAILYAWQGGMEGGKGLVDVLVGDVSP
SGKLTDTIARSIEDYPSTKNFGHADKGIYQEDIYVGYRYFETFAKDDVLYPFGFGLSYTTFNTKVKRTAEVDGVLTVEVEVTNTGECDGKEVVQLYVEKPQGKLGNPLR
SLVAFEKTLLLEPGEQQTLSFNVHMTDFASYDDSGVTGHESSFVLEAGTYRLYAGTDVRSATFALEVEQQEQRVLETLSENMAPVLPFERMKPIQGERGLEVGYESTPL
RAVDVETRYLEERPETRPFTDDQGIKLSDVYHGKASLDAFLDQLTDEDLAAIVRGQGMNSPRVTPGTAAAFGGVSDRLNELGIPAACCADGPSGIRMDIGTKAFALPNG
TLLASTFNVDLVEDLFEMTGLEMRKNRVDTLLGPGMNIHRNPLNGRNFEYFSEDPHVTGKMAVAQLNGMHRVGVTGTLKHFSANNQEAHRHDIDSVVSERALREIYLKG
FEMAVKEGRASSIMTTYGAVNGIWTAGLYDQNTRILRDEWGFDGIVMTDWWAKVNFREDESANRQNTAAMVRSQNDLYMVVDRPDLNSFEDNTMASLEEGVVTRGELLR
SARNICAFILKSPAMERLLGIHDGSVEVLGFEDETNQQDAFELDYQHLENGEHVDLSGIDTSTGNTHVFAISVDQTGTYDITLTARSNAGELAQMPVTLFANNIPGATF
TFNGTDGEWVTQTKQVFFLNQHNYLQLYFALSGLEVKDVSFKLADEFSMKNG
>tr|A0A1K1LXU2|A0A1K1LXU2_RUMFL
MKRTLDWNKYLEKAAETVAEGIVMLKNDNNALPLDTSKTVSVFGRIQLHYYKSGTGSGGMVNVSKVTGIVDGLVDAGVKINEKLFDIYRQWDEENPFDQGDGWGGEPWN
QKEMPLDESVVEEAASLSDTAIVIIGRTAGEEQDARLEEGSYLLTSTELDMLKKVRKHFKKVVVLLNVGGLIDLEQILECSPDSLLYVWQGGMTGGTGTAAVLTGKVSP
SGKLPDTIAYKISDYPSDKYFGDKNKAVYKEDIFVGYRWFETFAKDKVLYPFGFGLSYTTFDIELTDVKNTENETIISVIVTNTGKFKGKEVVQVYCEAPQGRLGKPQM
VLCAFEKTRELAPFEAHSMEIRVKHNDLASYDDSDGKNCWILEKGKYIFHIGNSVRNCTLQCSYEIENDKIIEQCSQALAPHAEFDRIRAVSSANGVSYVMEKAPLSKI
NEEKCRLDNLPAEIAYTGDKGIKLADVKNGKYTMEEFVAQLSDHDLSCIIRGEGMGSPRVTAGTASAFGGVSDALVKFGVPAGCCSDGPSGMRLDCGTKAFSLPNGTLI
ASTFNRKLVEELFEFMGTEMIANKVDCLLGPGMNIHRHPLNGRNFEYFSEDPFLTGHMASAELNGLHKIGVTGTIKHFCANNQETNRHFLDSIVSERALREIYLKSFEI
AVKVGKAKTIMTTYGSINGLWTAGSFDLNTTILRDEWGFKGFTMTDWWANVNFRGHEPKRNYYVPMARAQNDVYMVCSDSSCIDEDIEAALKDGTLTRAELQRNAMNIL
GFIIDTHAMKRIMGEEDTVEIINRSEGDDASDEPVVFYEIEDNFKLDLSGVKSEKGKNHSFALIISKQGWYDVTITASSTQSPLAQIPVTIFAMGTASGTLTWNGTDGK
PVALNCEIPFFSRFTAIRLYFAQNGLDLHSIEFRLVREADDMDLAFAEDN
>tr|A0A316Z1G0|A0A316Z1G0_9BASI
MKLSSILATIILAILSGEAVTDAKIVVRRDPNQRRGDQLALKHPSASQHYSSKGAADTSSRYSHSHGILDQGFIPPLDWIRGDHDGANETCSKVGREGYHHFSQHVLPA
ALKTDSGADRLEKQAVYSPKEYEAEEETKSDAVSVDKTKGAKPAEVNSQATNDSVYYSPPFYPTPQTTGAGTGLNSGWKSATDKARALLKQFTLEEKVALVTGAGWELG
DKPRCVGTISPNKRVGFPGLCLEDSPLGVRFADGVTVWPAGVTTAATFSSKLAYERGKAMGEEFRAKGVNIALGPGMNMARTPAGGRNWEMASADPYLTGESAFHTTKG
MQDAGVQACAKHYIANEQEINRNTYSSNIDGRTEREIYLHPFMRSIQAGVSSVMCSYNLNNNSWSCQNSELLNNRLKTELGFNGFVMSDWGAQHSGVASANAGLDMTMP
GQVECCSKDNKGSFWGQNLTMAVQNKSVEASRLDDMGTRILAAWYLLGQDSKDYPRPNFDAFDPTYGEHKQVTSHKHASVAREVAAAGTVVLKNTHGALPLQGEKLKKL
AVVGSDAGPLRQGANFYSDRGGPMIGTLDGTIGIGWGSGTADYSYFITPYEALQARARKDNTGFYWTFDNFNLDQAKKISDDQIGVGAALVFVYADSGENYIVVDANAG
DRNNLTLWGNGVELIKSVASVQKNTIVVINAPGQVDLEEFIDNPNVTAVVHAHFPGAEAGNAITDILYGDVNPSGRLPYTIAKKRSDYSADVQYFTEPDTATSQIDYIE
KLNIDYRHFDSAGIEPRYAFGHGLSYTKFHYAGASGKWLAGGGSDWNDRKWSLPLPDWLFEDVYELTFNVKNVGQVDGFEVPQVYLGFSSSAGEPPKVLRKFDRFAVKT
GATQKIKFRFNRYDLSVWNSEKQRWWKPEGDVKVYIGASSRDIRLTLDKM
>tr|A0A0S7DD26|A0A0S7DD26_9EURO
MINSRHILLVIQVFVLSLVTTSADQGVNFTSLELFWSYGRSPAVYPSPPGKGLGDWAPAYRKAKAAVKKLSNEEKNNITFGYNSYALANFSGCAGLSLPLPRIGYPGMC
LADASNGLRGTDFVNAYPAGIHAGASWNRSLVYHRGLYMGEEFKAKGVNVINGPVIGPLGRTARGGRNWEGFSADPYLAGVLVAETIQGLQKSVIASVKHFIAYEQETA
RGPEGNNASYSSNLDDKTMHELYLWPFANAVHAGVGSVMCSYNRVNNSYACQNSKILNGLLKTELGFQGFVVSDWNAQLTGISSANAGLDMAMPDSPYWQGNLSLAVAN
GTMSQERLDDMATRILAAYYKLAPHNHPGSGMPPVIINSPVPTVDARNPESRPTIFQGAVEGQVLVKNINHALPLLKPRSISVFGYDAGLPPKTNPAFSLKWYLGYEAL
DLADSVELTNLSHLATFPEAATLGTLIGGGGSGASVPSYISTPFAALVEQATVDGTYISWDLESFSPTVPVSSDACLVFVNEFATESRDRPGLADPQSDRLIMSVASQC
PNTIVVIHNAGVRIVDAWIENPNITALIFSHLPGQDSGKAVTEILYGRQSPSGRLPYTVARKPSDYGPLLDPTGPESVSDYYIQANYTEGVNIDYRHFLAHNITPRFEF
GYGLTYTTFRYSALQLSRAEEHCFSTRPPGTEIAEGGLPSLWANIATVKVQVMNTGWGDGFLATLADGSIGTNFAHSGATTASFVAGGYWTKVLDAVKKNKSNYHPYVT
IQFGHNDQKSTSGVSISQFMANLEKMVADVRSAGGTPILVTSLSRRSFDSSGHVVPSLANVVAATKAAAKATNCEYVDLNGASTKYLNSVGAKNAAKYNLTPKDYTHLD
KAGMIIFGNMMGLLLRTSITNSSQIASYIHPRSDVVAAINTGKFIYPS
>tr|A0A084GEZ5|A0A084GEZ5_PSEDA
MHGRWTPNPKSQLKTLKDWLFRPDGGNGFLEGSEKLMWNDDDLRRYVCLADPGIETDPFTTFIRAVMSGVFHKLLGRRSKKRIIDEESGMVSYDDEAMMKASNAIVVVV
ASAVPVLTIFVLNSLQTTAQRIGFTVLFTSIFAALLSIFSSAKRAEIFAATATFAAVEVSTFEFPDCVNGPLADNLVCDVTVSHAERAAALVGAMTIDEKLANLVNASP
GAPRLGLPAYEWWNEALHGVGYSPGVNFQDKGEFASATSFANPILLSAAFDDSLVHEIATVISTETRAFSNAGHAGLDYWTPNINPYRDPRWGRGMETPGEDPRRIKGY
VKALLSGLEGDDPSKKKIIATCKHYAGNDIDRWEDVLRFNFSAVISLQDLVEYYLPPFQQCARDSRVGSIMCAYNAVNGTPACANTYLMQTVLRDHWGWNDENQYITSD
CNAVGNFYADHHWVETAAEAAAKAYAAGTDTVCEVNMATDVIGAWNQSLLTEETIDRALNRLYHGLVRVGYFDPPDSSEYRSLTWEDVNTEHAQQLALQSAVDGIVLLK
NDDAVLPLEYDANTSVAVIGHWAEAPVQLLGGYAGTAPYYITPRSVAAAIHNSTHYANGPIAQDLDADDTWSEKALEAANNADIVFYFGGLRMTIEREDRDRTSIGWPA
AQLSLIQKLCALGKPCIVIQMGDQIDDAPLLENKNVSAILWAGYPGQAGGAAVFDILYGKSAPAGRLPVTQYPSSYTEEVPMTDMTLRPSEVSPGRTYKWYSDAVLPFG
YGLHYTMFEVAFSFEPAVSYDIQGLIDGCQKDHLDLCPFDTISVSVENTGDVLSDFVALVFVNGTYGPSPHPLKELVGYHRFRGVEVGESREAEISLTLGDIARVDESG
NTILYRGTYTLQVDVPAQHTVSFKLTGDDAMLDEWPQPPEDLGAEIE
>tr|A0A1V8V7T9|A0A1V8V7T9_9PEZI
MFWRSSCLAHEHRYTNMAPTMMIIKSLAFFAVSGALAQSSDFVCTASAPNATQYNASTTYLGCWTDATVRTLDRPQINNAQLSPQYCANRCGNAGYIYSGVEYTTQCFC
GTAINPVASQTNESACTYICPGDTSAKCGGTYLMNLYKINNPNPNPPPRQTKFQPACLTNPFCSQKACDTSLSIAARVAALISNMTLIEKAQNLVDAANGVERIGLPSY
EWWSEALHGVASSPGVTFNSPNGSDFSYATSFPTPILLGAAFDDPLIYQIASTVGKEARAFGNYMQSGFDFWTPNINTFLDPRWGRGLEVPTEDSFHAQSYVSQLIPGL
QGGLTAGPSKQIIATCKHYAVYDVEELRNSENYNPTQQDLGEYYLTPFKTCVRDVDVGSVMCSYNAVDGVPACASEYLLQDVLRDAYGFDNEPYRYVTSDCGAVDDIYN
QHDFTDSFAAAAAVAINAGTDTNCGSTYLQLNTSVARGFTTNATLDRALTRLYTALFTVGYFDGQPKYDQLSFSDVSTGAAQSLAYTSAWEGMTLLKNNGVLPLKKYNK
VAMIGPWANATGQMQGNYQGIAPYLISPLQAAQAQWGAGNVVYAQGTAINSTNTTGFAAALSAAKSADLVVFLGGIDTSIESEGHDRTNIDWPGNQLDLVAQLSQLRKP
LVVAQFGGGQVDDTALLQNRNVHALIWGGYPGQAGGAALIDVLIGKQAPAGRLAITQYAGSYINQVSKFDPNLRPGPNNVGRTYKWLTTKAVLPFGYGLHYTSFKASWS
KKLQRQYNIADLVSSGHGGYGKSTKNDASQFATVTATVRNTGRIASDYVALLFLSSSNAGPTPRPKKSLVSYNRAHNIAPRGQQQVSLPLTLGSIARADVNGDLTIYPG
DYQLALDFDGQASFSFSLRGRPTIIDPLPRQRTSYNYTVPVHPQA
>tr|A0A5J5B1L3|A0A5J5B1L3_9ASTE
MVSSSVRSRAAPMVSALLWFYILNSIFYHFCLSSARVFAQTSPVFACDVANNPGLADLKFCDSSKGVAERVDDLVKRLTLQEKIGFLVNTAGNVSRLGIPKYEWWSEAL
HGVSFVGGGSHFSDAVPGATSFPQVILTAASFNVSLFKAIGQVVSTEARAMYNIGLAGLTFWSPNINIFRDPRWGRGQETPGEDPLLASKYGSAYVKGLQQRDDGDKDR
LKVAACCKHYTAYDVDNWKGVDRYHFNAVVTQQDMEDTFQPPFKSCVLDGNAASIMCSYNQVNGTPTCADPNLLAGVIRGKWKLNGYIVSDCDSLDVFYNSQHYTKTPE
EAAAKAILAGLDLDCGSFLAQHTEAAVTGGLVTESSIDRAISNNFATLMRLGFFDGDPSMQLYGKLGSLPLSPTEIKTLAVIGPNANVTETMIGNYEGTPCKYTTPLQG
LTTSVSTTYVPGCSNVACSTAQIDDAKNIAASADATVLVMGADLSIEAESLDRVDLNLPGQQQLLITEVANASKGPVILVIMSGGGMDIQFAKDNDNITSILWVGYPGE
AGGAALADVIFGYYNPSGRLPMTWYPQSYADNVPMTNMNMRPDPSTGYPGRTYRFYSGETVYEFGDGLSYSQFKHHLVKAPMLVSIPLEEGHVCHSSWYLPILPEFPLN
DITVNETLGIFSKEQQLDFVLRLHTFNKNGQVRHIRLCEWWRTWSAQHLEMDLNKDGMFSCDGELMKNNNCGDTILRLDCFGYGGRKTAGFGCTQTNHGDRPIPSIPPD
DGCRLVLGLGPTPSTFSNDFYSVGVQKHKGSSTLLQQGLSYEGDSILKLGLSGGGEELSSVLEYSVSTQRDLNTPHHPNQLNVDGNRLLIPVLDEGSTSAKKSGGYMPS
LLLAPRMDSSTILHPTQELSAGCGQVPLSSFSVEL
>tr|Q6CEN3|Q6CEN3_YARLI
MAPPPPPHYPYYYPNRHGAYRRTRQAAGRSPVGLRNDRRPKARLDNHKKKTAQHHDGEKKAKPKSDTIIIRIASNVGGDIPKLSDPEPFDSNPFVNLTNYMHSPTYYPT
PMAGRINDHCWRSAFLKAKAFVTQLTNEEKANLTTDFSTADSPWYGETGEIPRLNLSSLRLQWGLHGVGGDSHFTTFLPAGITTASTFNKGLMYSRGAIIGKEARKKGM
DIVLGPLIDPTGRSAAGGRNWEGFGPDPYMAGVVATESVTGIQDQGVVATVMHYVGYSQEHFRNLEEWQAHGYNNLTSSGSSFIDDRTMNEVYIWPFANAVKANAGAIM
CAPQKLNNTQGCKNSYMMNYKLKRELGFQGFVLSAGMSQNEPPAALAGMDMSMPRLKASENKLREMMLRNYDKDGFPQSRLDDMATRVLTSYYYVTQHRNGHQSFVGDE
ENLSNKSAYDNILVDVRDGFHYRVALEIALEGIVMLKNDDSALPIEGMRTIGVLGAAANLGPSGSKCDDKFGCHGDGAIFQGWGDGGVNPPFVVTPYEAVNARAARDRIV
VRSNFDSWDLNQAEAVASSTDANIIFVAANSGEGNHVVDDNKGDRKNFTLWHNGDELIKKAVEVNDHNIVVVTAVGPVDMEKWIEHPHVKAVLFTGPGGEEAGAALAHV
LFGDFNPSGKLPFTIARNVNHYIPIETEVPRDGIPKAYFGEMSLTDYKWFDQNLISPRFEFGFGMSFSEFSYSDMKITTQRNPSLTLGAPPDYWGKDGNATTAKAFQQE
FKAASGDFQFPSGYNDDHNDDPVLNGGAVGGNPMLWDVMYQVAVSVTNHGPFDGAVVSQLYVSFPQDDQALRTAPKQLRGFSKTSLVVGETANVLFDLMWRDLAVWDVV
KQTWVVQRGDYDVFIGGSSRQLETLGRITIA
>tr|A6WB17|A6WB17_KINRD

MPYRDPSLPAGERVADLLSRMTLEEKVAQLAAPFGSAVDVHTPPATGWGCVVAGLCTLGLPPRETAERANELQRKHVEQTRLGIPVLLAEEALLGLKVRDATTFPDAIA
QAATWDPQLIEQVGRTIGVQMTRLGVRQALSPLADVARDPRWGRVEETYGEEPQLVGSMAAAFVRGLQGADETTPLIATLKHFLGYSASAGGRNTEPAPLGPREVREVH
ALPFEMAIREGGAKGVMPSYNDIDGEPVTGSRAYLNDLLRGELGFDGLVISDLAAVGQLHSKHHVAATAPEALARAVSAGVDLDLDNRVSSQALQEAVRSGLLPSADLD
RAVSTILRAKVELGLFERPYVDLDAVPESLDTAPERALARTVAEKSVVLLQNDPVDRFRAGPAAGPAAGPAAGLANGAPLLPLDPGVGTIAVIGPNADRPLGQLGHYSY
HVLDSITRRFALAADPTARAEDVEGLAGRTGADDARLLVESVPVVTFLEGIRARAEGEVLYEAGCPVVRDDRSGFAAAVAAATRADVAVLVVGDQAGINGFGNVGEGLD
SSTCELPPVQQALVEAVVATGTPTAVVLSHGRPYTLKWLAQSVPAVVTCFFGGEEAGNALASVLFGDVNPAGRLPIAFLESVGSAPLPYWRTLQAPPSYVEGPARAVFG
FGHGLSYTSFEYRDLEVDQEATTGATLNLSFTVANVGQRAGEEVVQVYGQDVVARTARPARKLLAFQRLHLEAGAATRVSVQVPASLFALWDLQEGWVVEPGTVKFFVG
GSSVKTPLRASALLTGPVDRPGPDRALSCTVSVADIDPDIDSDVAARAARAGGGGALRRPVPVITAEDTVLEWLEHPVGGGLLRALLGLGEEGEDSDGVLTDAYGLTLA
QMAGYSGGRFTEAMVADLVERTRAGGTARA
>tr|A0A1U7LRJ0|A0A1U7LRJ0_NEOID
MGDQRPKQNIRHPWSWSSFPHIGRTAAWFLFAAVMAAIFALLLGLLLHHHTAAQQSRAVTDPWYPSPLASGLDDWGDAYSRARNFVAGLTLLEKVNITTGTGWQSDRCV
GNTGSLPRLGLGSLCLQDSPLGVRFTDYVSVFPSGVQTASTWDKEYALSNMLNDLTYSLIYNRGLALGQEFRGKGVNVALGPVAGPLGRTPAAGRNWEGFSPDPYLTGV
AMYQTILGTQSTGVIACAKHLIANEQEHYRQAAESNGIKTHRHTTESLSSNVDDRTLHELYLWPFADAVRAGVGSFMTSYNQINNSYASQNSKIINGLLKDELGFQGFV
MTDWAAQHSGVESSLSGLDMTMPGTGIPSDGTSYWGSNLTLAVLNGSLPEWRLDDQVTRIFAALYKIQNKSNSTFPEVNFSSWTSDDEGDAIFAPGEQYQRINDHVDVR
RSHSSLIREINANSIVLLKNINSTLPLKSPKQIAIIGSNAGPNLAGPNGCPDRGCDNGTLALGWGSGTADFSYFITPLEAIQSRAISEGSGVQYVLEDYNYAQINATAR
RATTCLAFINSNSGEHYISVDLNEGDRNNITAWHDGDQLVSFTAANCNNTIVVVNSVGPIILEPWINHPNVTALVWAGLPGEDSGSSLVQVLFGDVNPSGKLPYTIAKS
ASDYGTNVMYEATEDVPQQDFTEGLFIDYRHFDQYKIEPRFEFGFGLSYSTFSFSSLLIEKASDSPYKANPYVPLTPINATPTRGKVSDYTFPSTFSRVKNYIYPYIPA
DSNIDGGSYPYPPHAVDTTPHPPSPAGGGPGGNPSLYDVLYRVSATITNNGPHPGKEVAQLYINLPSSANAPPKQLRGFEKVSLDVNASATVTFNLTRRDLSIWDVKVQ
NWVIPTGPITVFVGRSSRDLELVNIL
>tr|F4QPK5|F4QPK5_9CAUL
MNPSARFKRLLKLGTAFGLALSLSVTGGVTAAQAGDVEAVLAQMPAKAAPAAIDLAAKKKKAKAKKRAPRKATKKAAAKKPAAPKADAIIPVDPAWPVVPDAGIADAAT
EARITEIMSQMTLEEKVGQTVQADINFITPEELKTYPLGSILAGGNSSPGQNERATPDAWLQLADDYWRASLEYPSKVKIPVLFGIDAVHGHSNLVGAVIFPHNVGLGA
AHNPELIRKIGEATAKEMAVAGVDWTFAPTVAVARDKRWGRAYESYSENPADVAAYSGYMVEGLQGAEGARTGIKPGNIMSTAKHYLGDGGTTGGKDQGDAEMSESDLA
RIHNAGYPPAIEAGTLSVMISFSSWNGQKLAGSKKLITGALKQRMGFDGFAITDWNAHRQIPGCEQDDCPQAINAGVDMYMAPDTWKAVYTHLLADVKSGEVPMARLDD
AVRRILRAKIKGGLFELGAPKDRAMSGQWQVLGSPEHRAIARQAVRESLVLIKNNNRILPLRGSDHILVTGSGAHDVGKQSGGWTITWQGTGNSREDFPNAQSIWEGIA
ETTERYGGTASLSVDGVYKEKPDVAVVVIGEDPYAEFQGDRPNLDYQPGDATDLALIKKLKDAGIPVVTVFLSGRPMWTNPEINASDAFVAAWLPGTEGGGVADVIVAD
ADKRPRNDFKGKLTFSWPKAANQQPLNVGTMDYDPQFAYGYGLTYLNDGYVPVLSEESGLAEEAVVNVDTYFASGRIKAPWSLNLIDVSGSTSGDQGVFSSPGGAVSQS
AVDAGKQEAGRSLTFSGSGRGQAAISGAPVDLSRQTTGKMTLGVTYRLDAPVQGPVLLGMGVDASSTHPVNIASALTAPVGEWATVKVTLDCFVAAGVDVSKVGVPFSL
TSDAPLKISYSSIKIASDEGDATCPK
>tr|H3NUY6|H3NUY6_9GAMM
MSLSLPLLCAAVLTVCSPGDDPNADRTPPTISLNTAGTVTLFEGDEYVEAGAIAIDDRDGELTVVISGSVGSEPGTYTITYTATDSAGNTTQISLEVIVIDGDFDLFAA
STARIVAEMTLEQKVGQMIQPEIAYITLEEISQYGIGSVLNGGGSHPYGNRAATPEAWLQFARELREASLKRSNSSLGIPLIWGTDAVHGHNNLRGATIFPHNIGLGAI
NDPDLIGEIATATAREVAATGIDWTFAPTLAQAKDYRWGRTYESYSDDPAIVEAYGRVMVERIEAEGIAATAKHFIGDGGTQAGIDQGNTLVSSSAQLMAEHGSGYIGA
FEADVDTVMATFNSINGEKVHGSKSLLTSLLRDELNFNGMVISDWNGIGQVSGCSNASCAQAINAGIDMIMVPTEWLAFRNNLIQQVRRGDVDESRIDQAVTRIIDLKQ
KLGLVSRTFDPSRQPISVVGSPEHRAIAREAVRRSQVLLKNNNATLPLNPSQRILLVGAAADSIPLQAGGWSVTWQGTGTTNADFPGASTIRDAFTEVVESAGGTLEYS
PAGNYSSVPDAVVVVLSEQPYAEGNGDLQNLDWSASSVLQQVQTLRDAGVPITTLLMSGRPMFVNPELNRSDAFVASWLPGTEASGIADVLFTDSQGNVAFDMTGKLSF
SWPGGAINPSNASSPVAANLFERGYGLSYQDTTEFPQLTESLTNNQSGIVEDTGSGGGNTGGGGGSGSGNPDADPFWVFQDGGFTTTFDLGTRAFDSAINFELCVDDNG
AACPSISWSYLTDTERGTVLEILHEPAAPFAALFTESSTGIDLSDYQSGNFVLDLKHIEGPNDYRMKLDCFYPCESAHIDLNVQPGNDWQTVKVPVSAFTSSGLDITKV
NTGIVIWARDHNGTRFRIDNVRFEAN
>tr|A0A5C3F0Z8|A0A5C3F0Z8_9BASI
MKGSTRAVLFLGVYLSALLCSLTSAASIEHAQYLLNARQAAEHNTTVSNSPATEGTAVSSSDTVSPDSPYNAAGFWRGGPELADVNSPFHPITGNGGWEWAVERAKSWV
SQMTIDEKLNLTAGLAGQGRCEGTLGRVDRFGIPELCFQDGPAGVRTSDFVTVFPPGLTTAATWNRDLIYQRAAALAEEVKGKGINVHLGPATGGPLGRGPWQGRNWEG
YGPDPYLQGEAGYHTIKGTQSNGVIATAKHFLAYEQETFRQLYAADDPWTLNPQNNTRNTYSANLDDRTMHELYLWPFMNAVRAGSGAVMCVYNRINGTQGCENSKVLN
TILKDELDFQGFVVTDWSAAFNTSDTYNGGSDVVMPGGNTGGYRNLVGGKNLAKALQDGTVKQERVDDGIVRLLTQYILRGQDKDWPKVNYKDGYMNTYLNGTLVNEHK
NVQADHWKVAKKVAEEAITLIYNKRQGKGKGGAEAGAQGIEGRAPVGLPLAKKARVAVFGSDAGPNPYGINGCQGWLGRGSQLCPGNHTSNGTNAIGWGSGAGYFPYLI
DPLAGISAKAREYGGTVESNLIDEVDEGGQNRKLVQSTASIADASLVFVQARSGEDSDRSTLALEANGDEMIKAVAAASNNTIVVVHSVGQIYMDEWFDHPNITALVFA
HLPGQESGSTIAEMLYGETNPSGRMPFSILAKRDAKHYPKIINGPVDDPQVDFEEGLYIDYRQWDKLNLEPLVRFGHGISYTEFDYSDLEISQTGDDDFYPTEVPTQSG
KDKHPGGSARLFQYLAKVCAKVKNVGHVSGHEVAQLYLGYPEAAEAPIKQLRGFDKLMDVAPGSTKTAEFKLTRRDFSVWSVEKQAYTIVDGTYKVWVGKSSDPKRLTL
KSSITMKDGKIADMNEAKAPTSSGK
>tr|A0A176WBW1|A0A176WBW1_MARPO
MIECTYDNERKKHPAPATHGDFPRTDARRDGLDQHSCTSLGHEGALGVWKKFELRATFGVTFVSQDGDGDLLVDCPESFRNSWKGAPDPDSLTNMTSAGSLLRLAIAIV
VAASIISGQAAAAADGPDARRPRVPHPCQEPAAYPFCDVNLSNEDRVRDLVSRFTLEEKARLLVNMGSGSNITRLGLPAYEWWQEALHGVADSPGARFKGRVKSATSFP
QPILTAASFNKELFNKIGQVISTEARAMHNENQAGLTFWAPNINIFRDPRWGRGQETPGEDPYLTSIYAEYFVRGMQEDDEYEGQNLRTSGKGPAQLKTSACCKHFTAY
DIDQWYDVDRYDFDAKVTQQDLLDTYNPPFQSCIEDGKASSLMCSYNRVNGVPTCADYNLLTKLARGTWGFDGYIVSDCDAVQVMYANSRYAQTPEEAVAYALKAGMDL
NCGDTASNFTVEAVHSGLLNTSDIDKALHNSFTVLYRLGYFDGDPLNDPKYGKLDHSNICSPEHQDLALEAALQGIVLLKNDQNTLPLSADKIRSLAVLGPNANDTVNT
MLGNYAGPPCVYVTPYLGLAQYVPDASYHPGCDNGTTCEGRSDFIRGAAKVATWTDAVVVVVGLSQDQEREAFDRTSLRLPGQQEELITTVSRVAKGPVILVLMTGGPV
DIGFIKDDPKIQSILWVGYPGQAGGQALAQVIFGDRNPGGKLPMSWYPESYTEVPMTDMHMRPDESTGYPGRTYRFYSGDVIYRFGEGMSYTTFSSSFVSAPSVVTASA
NSRQSCSQRQRDSPNIPCSSADNALVCNSMKIEIVVSVKNDGPVAGTNVVLLYHTSPTAGKDGSPLQKLVGFERLYLESMQEQKALFKVNLCRQLTEAQTDGTWSNLIE
GVHTFSLGNSNDPKHEMKLIYEHPS
>tr|A0A6G9QL90|A0A6G9QL90_9GAMM
MLNFTRNINNGGDAISHKVIPFKFAPLSASLLIGLGLLGCQPSTPSKNQQQIDLAQTVAEQKELVPESTSTGVKADINVWPQQQAAIAVDPILEQKIAEILAQMTLEQK
VAQMIQPEIRDITVEDMRQYGFGSYLNGGGAYPNNDKHATPADWINLAEAMYQASVDDSLDGSRIPTMWGTDAVHGHNNVIGATLFPHNIGLGAANNPKLIEQIAAITA
KEVMVTGIDWVFAPTVAVVRDDRWGRTYEGYSEDPRIVRDYAFAIVEGLQGAVDGEFLSDQHVLSTVKHFLGDGGTEKGIDQGDNLASEQDLYAIHAQGYVGGLNAGAQ
SVMASFNSWHGDKIHGNKYLLTDVLKGRFAFDGFVVGDWNGHGQVAGCSNESCPQAANAGLDIFMVPTAAWKPLYENTIAQVKSGEISQARIDDAVSRILRVKLRAGLF
EKPSPAKRALSGKTELIGQASHREVAKQAVRESLVLLKNNQALLPLSPKMNVLVAGDAADNIGKQSGGWTITWQGTDNQNSDFPGASSIYQGIASAVDQAGGQVQLSVN
GQFDANNKPDVAIVVFGEEPYAEGNGDIDNLEYQRGDKRDLALLKSLQAQGIKVVSVFISGRPMWVNPEMNASDAFVAAWLPGSEGQGVAEVLFTDAQGQVQHDFVGKL
SFSWPSTPQQSAVNVNDEDYQPLLPYGFGLKYGDKSTLANDLSEDNFAEQTEIEDLALFERAVKSPWNMYIATVALDNNGLPQIKDRQTLASSMAENSALHIRTIDKVV
QEDARQVTFKGEQMGLVGLNSNFPRDFRAYQDNSVLSVQIQVQDKLEQPLMIGMVCEGACLGQFDISEQVNAKVNTWQTIQIPLSCFAKQGFDFAKVIVPFYIATQANT
TVSFSDVVITNVSASDNSQVIDCK
>tr|A0A316WGQ4|A0A316WGQ4_9BASI
MPPPRNAVLALLGCVLVAAHAQAGKRHSAELHRRSTEDVSLTSLFDKLGIKIPFLGAVPSGGDRDSLSFNSDNSNAADVAKVARDALRKAEEALEGAEKELNDSLHLPL
RKLSASHRIDASALSLLHARQHQQSIVAPFADGGAGWVESFRIAQDLVGKMTLEEKANMTAGANGPCIGSTGSVPRLGIPSLCFSDGPTGVRQALNVSQFPAEVTVGAT
WDLDLVGKRATAMAEEFRDIGINVMFAPVTGGPLGRSPQGGRNWEGFATDEYLTGRASYVSVKDAQAAGIVAGAKHFIFYEQETSRNVRTILPVLQQPIDSVVDDKTAH
ELYMVPFAEAIRAGAGQIMCSYNKINGTHACESSKSLAGLLKTELNFQGHVVSDYGGAWSDSTVGLDVLMPGDGLYGVQPNFFGSHGSKLIENVENGKLTETRLDDMVI
RLLTPILQYQGGDLSKLLPNPDQLNVKPTGNKNVQGDHHQIIRQIGTESLTLLKNTVDGKTGSGGATGLPLDLNKIKKIAVSGQDAVSSLDNRKTCNNLGECVLKDFQG
VVTTGTGSGSTTPPYIIDPLAAIRSYIAKADKDRAVTVTSSPNPFVFLGAVWEAKNADVALVFVSATAGEASDRNADLKLDHNGEDLIKAVAAANNNTIVIIHGPGPVI
VEDWIDLPNIRAVLYAYYPGQEAGSSLTPVLFGDESPSGKLPFVMAKKTSDWPANTLVKTLSLNPQATFAEKLLIDWRWLDAKNIAPRFPFGFGLSYTTFSYGELQVKK
QFKADSTSIQRTKEPFVSSVSPGDSLYDELFSVNLPITNTGDRTGKEVVQLYLSYPASATQQPPHALRGFAKVQIAAGQTRTVSMQLTRKDLSIWDVVQQKWVIPRGTF
TLFAAKSSADWTNAKKANFTV
>tr|A0A2B8AYN4|A0A2B8AYN4_9ACTN
MQHETSGPWQDTSLPAATRVNDLISRMTLEEKLAQLYGIWPGSDADGEDVAPMQHEVTASSASTDTDTLLTHGLGQLTRTFGTAPLGGAEGAEALARTQERIVASNRFG
IPALAHEECLTGFTTWGATIFPTPLAWGASFDPALVGRAAELIGASMRAVGIHQALSPVLDVVRDPRWGRTEESISEDPYLVATVGTAYVRGLEAGGVIATLKHFAGYA
ASRGGRNHGPVSAGPRELADIVLPPFELALRDGGARSVMAAYNEIDGVPSTANVWLLTTLLREEWGFTGTVVADYFGIGFLEMAHKVAGSRAESAGLALTAGVDVELPG
VDCYGTPLRDAIRDGDVPESLVDRALTRVLRQKCELGLLDPDWSPQPDALAGPPAAAGATPDAAASSGAAGPAPDEADASADGEILAPLTAARERTLRTRSTSGAVDLD

PEEMRTLARQLAEESVVLLSDDAGVLPLATSPGTRIAVVGPMADDPAAMLGCYTFPRHVGVEHPDLPMGVEVPTLLDSLRSAFPGAEISHAVGCPPPRGTAEKDHDTAA
AADEADALTEALALTAGADVCVAVVGDRSGLFGRGTSGEGCDSPDLELPGGQGALLDALLSTGTPVVLVVLSGRPYALGRYAGDRRPEGAPSAAAVVQAFFPGEEGGPA
VAGVLSGQVNPSGRLPVSVPYGSGGQPWTYLSPPLGYEAGPSSLDPTPLYSFGHGLSYTGFSWEDAGLVTAGPDGDGADGTGPALLRTDGEATVRLTVRNTGDRAGTEV
VQLYLNDPVARTTRPVNRLIGYTRVPLGPGESAEVRFGFHPDLAAYTDAGGRRVVEPGDLELRLSASSTDVRHRLAIRLTGPERTAGHSRRMVCAAMVHRLGAPGQVPA
QTPEQPQSGAAPLAPSGRPS
>tr|Q5H0B1|Q5H0B1_XANOR
MACGATHRRGRTTLENLILDKLLRRAAAPVARRALCLATAAAVLMLAACQGKDTAGEANKTAPAAAPAASSTTIHPDQWPSPKWPFPQDAALEQRITDVMAKMSVEEKV
AQTIQGDIASMTPDDVRKYRIGSVLAGGNSDPGGKYNASPADWLKLADAFYEASMDTSKGGNAIPIIFGIDAVHGQSNIVGATLFPHNIGLGATRNPDLIKKIGEITAA
ETRVTGMEWTFAPTVAVPQDDRWGRTYEGYSESPDVVASFAGKMVEGVQGTPGTPQFLDGSHVISSVKHFVGDGGTTDGKDQGDTKVSEATMRDIHAAGYPPAIAAGAQ
SVMASFNSFNGEKMHGNKVMLTDVLKGRMNFGGFVVGDWNGHGQVKGCTNQNCPASFIAGVDMAMAADSWKGIYETELAAVKSGQISAERLDDAVRRILRVKLRLGLFE
AGKPSKRPLGGKYELLGAPEHRAIARQAVRESLVLLKNQAGILPLDPTKRVLVLGDGANDMGKQSGGWTLNWQGTGTKRSDYPNGTTIWEGLDKQITAAGGSAELAVDG
AYKTRPDVAVVVFGENPYAEFQGDIATLLYKPGDESELALIKKLKAEGIPVVAVFLSGRPLWMNQYINASDAFVAAWLPGSEGEGIADVLLRKADGTVQHDFKGKLSFS
WPKTAVQFANNVGQKDYDPQFKFGFGLTYADKGDLAALPEASGVSGEQSVSGLYFVRGKPALGIAMQMSGVAQKHVPATTLPVGLSDDSLKITAVDHKVQEDARRLVWS
GSSVASVLLVSGKPVDVSRESNGDVQLQLTLRRDSAVTAPVWLGVGCGEKCGGRVDVQKTLAALPQGQWKVVGVPLKCFAVAGADVTKLSEVASIESAAALTLSISKVA
LGALNEAEVGVDCPVK
>tr|A0A445LR21|A0A445LR21_GLYSO
MVRGSGYHSGPQFQIHLTLLEFVAMEFVTWTKFRISAVQQTNYYFSKFLVIANYSDISNVSKYGDACLSDCECVASVYGLNEESVHRKRTLKREMESSFILSGAPMSFT
YRNLQIRTCNFSQLQGTGGFGSVYKGSLGDGTLVAVKKLDRVLPHGEKEFITEVNTIGSMHHMNLVRLCGYCSEGSHSTLVPSRGVYVGFSLIGGSVGLSEQFPAVLKT
MWDNVGVEKAVSGRFQAEGEREERVQGFRVLSAGTFKMGIYSMPFVGVLLLFCLVSSSEAEYLKYKDPKVPPNVRISDLLKRMSLEEKIGQMTQIERSVATPDVMKKYF
IGSVLSGGGSVPATKASAETWQQMVNQLQKAALSTCHGIPMIYGVTRKVYGYARIERVIMKSHLITLIGMIWDPVLIKKIGEATALEVRATGIPYVFAPCIAVCRDPRW
GRCYESYSEDPKIVKTMTEIIPGLQGDIPGNSIKGVPFVAGKNKVAACVKHYLGDGGTNKGINENNTLISYNGLLSIHMPAYYDSIIKGVSTVMVSYSSWNGMKMHANR
KLITGYLKNKLHFKGLVISDWQGIDRITSPPHANYSYSVQASVSAGIDMIMVPYNYTEFIDELTHQVKNNIISMSRIDDAVARILRVKFVMGLFENPYADPSLVNQLGS
KEHREIAREAVRKSLVLLKNGKSYKKPLLPLPKKSAKILVAGSHANNLGYQCGGWTITWQGLGGNDLTSSTTILDAVKQTVDPTTEVVFNENPDRNFVKSFKFDYALVV
VGEHTYAETFGDSLNLTIADPGPSTITNVCGAIRCIVVLVTGRPVVIKPYLSKIDALVAAWLPGTEGQGVADVLYGDYEFTGKLARTWFKTVDQLPMNIGDKHYDPLYS
FGFGLTTNITKY
>tr|V4GUY9|V4GUY9_9EURY
MAATAAFAEGASAAESDPGEFSQRVRNLLSEMTLEEKVGQMTQMAVSSFDPEEVGDLFTEHHVGSILSGGAAPPSFEASEVAAGVNRLQEWAMENTRLGVPFVYGVDAV
HGNDLVYDAPIFPHNLGVGATWDPSTAREMAALTGESVRAMGAHWTFSPANDIQRDPRWGRFYEGFTESVRLAERMGRAKVEGYERATDQYDKAVGACQKHFAGYSQPL
NGNDRTAAQLPARYLRQFHLPPHEAGIDAGAETVMVNSSSVNGVPAHASKWLLTDLLRGEWGYEGMVVSDWKDFKRMVTIHNYAADLKEATMLGVNAGVDMYMNPDDVG
EFTSILVELVEEGKVSRDRIDQAVARVLRFKENVGLFDQPVAETESVSETISAGLDDARETATKSMTLLQNADDTLPLDPDLGSVLVAGPRIDNPLMQMGGWTLGWQGL
TLDSVDLSRVPATTVVEGVENAVSSSTEVVTMPIEHTWDVYGGLDEYSFDNPGEVETAADEADAAVVVVGEGPYSEGPGDANHLHLHPAQQELVDTVAGTDTPLIGVVI
AGRPRGTEVFDRFDASVMAYQPGSAGGDAIADVLFGEVNPGGHLPFRWPVTVGQVPNVHNALHPVDDPPRFEFGHGMSYTSFEYTDLQVSTSGGSLTASVSVTNAGDRA
GDDVVQVSATRDSAPVIYPQRTLVGFERVSLDPGETRRVEITSPLSTLAVVPGDMTDTGERVVPAGEFRLFVDGLETTFTLDAPADGDGGDGTGDDGTGDGDHQYYQVD
FVAGQPIEELGEEGLYAEQDRLMRFARGTAAGGILDKGTAWPSAEIRECLDYGHIGVEDGTASIRFTVADGCERTVSLAVYETPNESFSAAGADEQEYLRASTETFGPG
EHTVSVDVPSEE
>tr|A0A2P6TNG4|A0A2P6TNG4_CHLSO
MQAVRCVQLAIAILAALAAFCSPAVARSLQQDDVTLRAGSGNTSHPWLDATLPLEQRLAALLAALTPQQKAAQLQTDAGGAVPELGLPPFTWQAECGHGYKVGGAGEQL
PAGKYGAATIYPQNMVMGATFNDALIERMASEISDEMRAHRALAAAAGDVLGHSNCFSPHITLARDARWGRMAEVYGEDPLLLSRMAAAYVRGLQGPPDAPYVTVGATC
KHFLGHILEGNWNGTTIRNLDARLEDRDVRDSLLPAFQACAAQAAAVMCSYNAWRGSPSCASKELLIDLLRGQMGFRGFVVTDKFAVDYSAAKPPDGFSLAGGDLRKAT
ALAIQAGTDVLMKNTSQLHAEDLSAAELDAAVRRVLRARFRLGDFDPAASLPWAGLNASVIGSPEHAATARQIAAEGTVLLKNERGMLPLRPEAVKKLAVLGPFADQAD
HILGTYVGAASGNITTPLAALHAVLPGKDQVSWDTSTADKFSAKNAKADAKRCEAADVCVLFLGSRLSRMKPEDYEESHEAAYLTSDEGEGRDRKTLRLQPNQRKLWEA
VAKATTKPLIVVLFHGGPLDVSDMLESPRVAAILSAGMPGQHGAAAIADIIVGATAPSGRVPVSWHRESYMNELDATDQRMRAAPDGSFPGRTYRYWRGQPPLFPFGYG
LSYVQWHLSGAAIGAAGNGSSSGGSGGADASASGAPDTLAVAEGGSSPAEPGISVDLEGDSVALQVSVTVTNPAGGSASNGSSRSNNGSSGGLTASTSILLFMRLLDAS
SSSSGGSSGGSNSSGVELSSQLPLATIGSSGCSWAANSTDVVQQLVGYARTGQLAPGASARLVFRLQLAPPPEGLSTSTSIGSGSSWAGFGDPEPPCGLYGLRFGHDQP
DAALVLLD
>tr|A0A1I1CDP7|A0A1I1CDP7_9BACL
MLGLWHAFIETPRLKLKLQFKVAKKAVYALDMAMEPFPLPVRFDRVEAEGGTLRATGSAYWKPDEEASVELRFAEDAFEGSLRLPSFGAFALAGIRGRGPSLYASLADE
IAACRKADVPARGDAEIREAVETLLGKLSLADKVSQMCQCMASNFSFGGAVASDPPEKLVAEGRAGSVLGAFDLGRVFELQRIAVERSPHGIPLLFNNDIIHGAQTIFP
VPLAWACSWDMDAIREACAVAGREAAASGIVYNHGPMVDISRDPRWGRVVEGAGEDPYLGALIAKAQVEGFQGGTGGQGSLRDPDTIVACLKHFVGYGAAEGGRDYNTV
DMSEATLRNVYLPPFQAGIESGAGSVMNAFNIYQGVPVAASVPLLKELLRDELGFEGVLISDYGAVDEIVQHGHARNAKEAAKKAVDATMDIEMVTRAYDHLPQLIAEG
KLSEAQLDDAVRRILTLKYRLGLMDDPYRYIRPEREAELHFSEPHLAASRALAAKSIVLLKNDGALPLGGASGASGRLEGAGDAASLPSAASRDCGPGGRGKLALIGPF
ADSKDLLGPWQFSRYVHETVTLYEGLVAAGYSEDRLLYAQGSGVGAPIDGGIEAAVDRAVQADIVLLALGEDSGMSGEAASRMRIALPDAQLRLAEAVAATGKPVVLVL
TNGRPLDLTWFDAHMNAIVETWFLGSQAGRAIADVLTGACNPSGRLTMSFPLHEGQVPVYYNHFRTGRPLTEANAGEKFISKYLDGPNEPLYPFGYGLSYARFEYGELC
LGADTLKPGGKLEARVTVTNALGPAGVETVQLYIQDVCGSIVRPVKELKGFAQVALAPGESREIVFAIGESDLVFWSPAAGYAAEPGEFRVMVGPNSRDVRTASFELEE
P
>tr|A0A1Q2M357|A0A1Q2M357_9GAMM
MNNKIGRFGAGRAMHLASCIALAVGLSACGEAEKPNTSTASSTSQTVLQDSAASNSIDVWPALKPALPRNEQVESHIADLLARMTLEEKVGQMMQAEIKFITPEEVKQY
HIGSILNGGGTFPNNDKFATPDDWRQLADDYFAASMDTSDGGVAIPIIWGSDAVHGHNNVIGATLFPHNIGLGAARDPELIRRIGEATAREVAVTGVDWTFAPTIAVVR
DDRWGRTFESYSEDPEIVAAYAREMIKGIQGDKSSEEFLDGHHLVSAAKHFVGDGGTTRGIDRGDTEVSEKELAEIHAAGYITALESGVQSVMASFNSWNGKRLHGHKY
LLTDVLKERLGFDGFVVGDWNGHRFVDGCTVDSCAQAVNAGLDIFMITAEWKALLKNTIAQAQSGEIPMNRIDDAVSRILRVKIRAGLFERDRPLAGKNGILGSDEHRA
IAREAVRKSLVLLKNNDQLLPVDAGKNILVAGDGADNISKQSGGWTISWQGTGNTAEDFPGATSIYTGIKQAVDAAGGEAVLSVDGEIKEDTFSNNAKPDVAIVIYGEE
PYAEWHGDIASIEYQLGSKADQELLQKLKAQNIPVVSVFLSGRPLWVNKELNLSDAFVAAWLPGSEGAGVADVLLTDADGNKRHDFTGQLSFTWPGQVHQTVINRNDVE
YQPLFAYGYGLDYTSDTQVANNLNEDGESYVDGKLEDAWLMVSRPRAPWKLYIEEEGSDLQWVNGNLASSADDNIVVASIDMLSQEDARALTWKGLRAGSVVLHTQKPQ
DLSRYLAEASALTMEVRVDEMPTASVSAQINCDGSDCSGALPLQNTLSKLPVGEWAEVSIDLQCFAEAGADFTKVSQGLSLHSKGKLTMAVANIKFVPGAREQAQVRCG
S
>tr|W7QE10|W7QE10_9ALTE
MFSTSERRLALKAQATNILLFIIASGLLSACSESANQTVSSTTSTEKQALSLEGRDAKVYWPNANTKLKRDPAVEKQVADILASMTLEQKVAQMIQPEIGYLTLEQMRK
YGFGSYLNGGNTAPYGNKTATPQEWLKFADEMYEASTDDSLDGSKIPAIWGTDAMHGHSNVSGTTLFPHNIGLGAMNNPEHIRKIGDATAKEVAVTGIEWSFAPTVAVV
QDDRWGRTYESYSEKPEIVKAYATAMVEGIQGEIGVDFLQGYRRIATAKHFVGDGGTLNGIDRGDTIASEEAMRDIHAAGYFTAIEAGVQSVMASFNSWKGKRLHGHKY
LLTDVLKEQMGFDGFVVSDWNGHKFVDGCDLEQCANAINAGVDVLMVPEHYEAFYHNTVAQVKSGEIPMSRIDDAVTRFLRAKVRWGLFEKGKPSTRILANDLSVFGAK
PHRELARQAVRESLVLLKNNQQILPLNPKQKILIAGDAADNMAKQAGGWSVSWQGTDNTNEDFPGATTVYAGLKNAIEQAGGKVEFDVAGNYQTKPDVAVVVIGEPPYA
EWFGDIQYIEYQKQNKADLALLKKLKADGIPVVTVFISGRPLWMNKEINMSDAFVAAWLPGSEGQGVADVILAKANGDINYDFKGKLSFSWPKYDNQVILNPHDENYDP
LFAYGFGLTYQDKVELAKLDETSRVKLQKSTNMGYALFYRQLAPELNWVLGDKTNWSVIVNGPAGTTENSDNLTVQGINLAYQEDARRFVWSGGADASAKLAYNGQQDM
TEFVEADSVFNFDLRIDKAASDKVTMGLFCQAEQTGCAGEVDITDFVNQQKVGHWVNVSVDVSCLAGTDKVSSVKTAWKLQTAGELGLAVSNLAIQKTDGKKAQFSVCN
>tr|A0A3N4W8V7|A0A3N4W8V7_9GAMM
MIENAPRRRSVPSDLRVKPRAGAGAVAAAVVTLLFCSAPAPAQAPAAANGAAAGVAHPELWPEAASRGLEDARTEAFVEALLAKMSLEEKVGQMIQGDILSVRPEDLRR
YPLGSILAGGSSPPLDAPDRSPAGPWIDTARAFRAVSLERRGDHEPIPVMFGIDAVHGNNNVVGATIFPHNIGLGAANDPKLIRRIGEATAVETAAAGFDWAFGPTLAV
PQNDGWGRAYEGYSEDPDLVRRYAGEMVTGLQGEPGLRGPLQRGRVAASAKHFLGDGGTEGGVDQGDNRASEEQLVRVHNAGYPKAIEAGAMTVMASFSSWQGVKMHGN
RSLLTDVLKGRMGFDGFVVGDWNGHGQVPGCTPTDCAAAFNAGLDMAMAPDSWKGLYENTLAQARSGAIPIARIDDAVRRILRVKHRLGLFDPARPFEGRTELIGAPEH
RALAREAVRKTLVLLKNNGGVLPIRPDAKVLVAGPGADDVGMQSGGWTLSWQGTGNRRSDFPNAQSIYEGLREALQAGGGRAELSVAGEFTERPDVAVVVFGEAPYAEF
LGDVQTLEHQPGDKRDLALLKKLKAQGIPVVSVFLSGRPLWVNPELNASDAFVAAWWPGSEGGGIADVLVAGRDGKPRHDFQGRLSFSWPRTAAQLELNKGQPGYDPLF
PLGYGLTYASRDTVPALPEISGVNAATTNAGNYYLNGRTPPPWRMVLREGGRTVAVETNAAASASGALSLRAVDAAGRQEGGRQLRWSGQREASVAIVGGPEAKPLDLE
RQANGDVSLQLQYRVDAAPQGKVTMALGCGPACRPVDVTSLLAAAPAGEWRTAKIRLACFRSAPDAKLAGILEPFALSADAPLQLSIAEVRLMADPSGATCPDRTEGK
>tr|A0A0Q4GIV2|A0A0Q4GIV2_9SPHN

MSTGFSTLTLALLLQSAATVPPPPTATAPTAAPVRATPSAPGVRTPAQPGPLSAVAHPDLWPAVAARPSRDPAVEARIDAVIKRMSVADKVGQLIQVDIASITPDDLRT
YKLGSILNGGNSGPGGDDLAPPAAWLKLADAFYDASMARSDAGPSIPVIWGTDAVHGNNNIPGATLFPHNIGLGAAHDRDLMREIGHVTAIETAAAGIDWTFAPTLAVV
RDDRWGRTYESYSEEPAIPADYAGAVIEGVQGIVGTKSFLAPDHVIATTKHFLGDGGTGGRDQGDTRVPETVLRDVHLGGYPAAIEAGTQSVMASFSSWNGAKMHGNQS
LLTSVLKDRMRFDGFVVGDWNGHGQVDGCTNESCAAAINAGLDMFMYSGDAWKTLYANTLKQAQSGEIPAARLDDAVRRILRVKFRAGTFDRGRPSSRALAGKMTLIGA
ADHRAIARRAVRESLVLLKNEGGLLPLKPSATILVAGGGADNIPQQAGGWSLTWQGTGTTKANFPNAESIWSGIDTAVRAAGGTATLSADGSFTKKPDAAIVVFGEEPY
AEFKGDRPTLEYSPEDKSDLALLRKLKAAGVPVVAVFLSGRPMWVNAELNASDAFVAAFLPGSEGGGVADLVLRKRDGTVANDFRGRLSFSWPRRPDQYVLNRSDPGYD
PLFALGYGLRYAKPGSVPRLDETRPAGLAASANGTVFGKGRVPEGWSLTLVEPGQAKVRMSGATATTVAKRLTVTAVDRRRQEDARRFSWTGGPAATRTDARIESDRPL
DLTRESNGELSLVVDVRVDRPATGAVSLGMASGDAAPVTVPITKMLAGAKPGAWQQVIVPLQCFAGRGVDMAHVTAPFVIAADATLDLAVSDVKIDSAPVPMTRCGD
>tr|A0A1Y0FUP0|A0A1Y0FUP0_9GAMM
MKNKTNIRFLFAGAVSAVLLGFLSGCDNTTTTPNETPSASPVTTSSISSEKVEWPAISSAVKKDPALESRIDELLARMTLEEKIGQLVQPEIKQVTPEDIKKYHVGSVL
NGGGTTPGNNKYAKLEDWVALADSFYHASVDKSDGRVGIPIMWGTDAVHGLGNVIGATLFPHNIGLGATHNPELIKQVGAVTAREIAATGLDWNFSPTVAVARDDRWGR
AYEAYSEDPEIVRAYAGKMVEGLQGIGGSDAFLTESHVIATAKHFIGDGGTLNGVDRGATQGDEKHLRDIHGAGYFSALEAGVQVVMASFTSWQDTRMHGHKYLLTDVL
KGQMGFDGLVVGDWSGHGFIPGCTALNCPDSINAGLDIYMVPEPEWKELYNNLLEQAKTGVIPMARVDDAVRRILRVKMRAGLFERGAPSTRELAGKESVLGAPEHRAV
ARQAVRESLVMLKNKNGLLPLARNQKVLVAGDGADNIGKQAGGWSISWQGTGNINSDFPGASSIFAGISEVVTSAGGTAILSVDGSFSEKPDVAIVVFGEDPYAEMQGD
VGNLAYKPRDASDLELLKKLRSQDVPVVSLFISGRPLWVNKELNASDAFVAIWQPGTEGAGIADVIFKNAEGEINHDMKGRLTFSWPKRPDQGPLNRGDENYDPLFPYG
FGLSYADKDTLGDDLPEEGVQLAEALDVLEIFNRRPLDPWSLEIIGNQNDRMTMTSNTMKASSLMIEAVDRNEQQDARRVVWNGSGSGQVALSAENRQDFINYLKSNSA
LVFDIKVDAAPGDTTYLRLGCGSYCASDIDFTTRLKEFTGKGWQTVTIALACFPASGANFGVAQPPSEFFTQVLQPFSLVTSGSLDITFANVRVVKGAAEGEAGKCN
>tr|Q6CEN5|Q6CEN5_YARLI
MLAFVLLLTMLLAAALADPFSDKDAYKHSPPYYPAPEIGRVPTDLRWRAALKVAQGMVANMTLLEKVNITTGTGWEMGPCVGNTGTVERLGIKSLCLQDGPLGIRFADL
ITTFPAGITIASTFSRQLVRERGAAMGRENRRKGVDITLSPVVGPLGRHANGGRIWEGFSADPYLAGKLAAEAVTGIQGQNVMAVVKHMVGNEQEHFRQLGEWQGFGFK
DLKQPLSSNIDDRTLNEAYLWPFADAVRANVGSVMCSYQQINGSQGCQNAHILNGKLKEEMGFQGFVMSDWLAQRSGVASVLAGLDMSMPGDGLVWADGVPLMGYELTR
SVLNGTIDESRVDDMVTRILTPILYLSITPTDPNFSSWTNDTTSYKYYGAKAGGNVTVNRHIDVRDQYTTKAALDGANAALVLLKNEKKTLPLNPTNIGNLNIFGIGSK
TGPLGAVCGENMQCSDGALIEGWGSGSVYPTDYQSPYDAIKERASKDNITIGGTTQSWGNLSNVEILSAAADASVVFVLSDSGESTGIVDGNIGDRNNLTLWHNGDEVV
KAVASKNPNTIVVVTTVGPVNLEKWIDNPNVTAVLLTGAPGDFGGRAAASILFGDIAPSGKLPFTIAKNDTDYIPLTTKIPEDGLPQDYFTEGTLLDYKRFDENQVTPR
FEFGYGLSYSNITVENLEARYAFPSIPEFLPTPFAPSNPNKPKNAFTPHANESVFPSDIDPLNKYVYPYLNDTSEIFSNETHYPYPEGYSSEQSNSTNINGGAVGGNPA
LWLSAVYIVHSVSNYGPYDTGVVTQMYIAFPQDNDDLKTAPRQLRGFERSELKVGERQGILYDVQWRDLAVWDVKLQSWRVQRGEYKVYVGHSSRDFVLTTSFTLK
>tr|A0A1Y2F7W9|A0A1Y2F7W9_PROLT
MTFIAVLVLAIALPLTRAQDSTTFSEDVGQNYVSPPVYPSPLAADGPDGSWSAAYAAAADFVSKLTLVEKVNITTGTGWTQGPCVGQTGSVPRLGFPGLCLQDAPLGVR
FADYVTAFPALSQVAKTWDRSLFRAHAVAMGEEFKGKGANVQLGPVCGPLGTFAEGGRNWEGYAVDSYLCGQGMYEGVKGIQSTGVTACAKHFLGNEQEHFRQAPESNG
FKTHKINVTQSLSANMDDRVTHELYLSPFADAVRAGVGSIMCSYQQINNSYACQNSKLINGLLKDELAFAGFVVSDWGAVHSGVATYLAGTDMLMPGDGNSFSDGLSWF
GPNLTISVLNGTLPETRLNDMAIRIMATYFQHNQNSTSYPKPNFDSFNNDRVGYVYEGAAQTVLNERVDVRGNHAKIARQVATESIILLKNERGALPLKNIKSLAIAGM
DAGPDAYGPNGWNDRGGLNGTLAMGWGSGSCQFPYLITPLEAISARARQEGSLVQDVLDNYAYDKINQTVKYANAALVFINSDSGEGYISVDGNEGDRNNLTAWHGGDK
LVRTVAANCNNTIVIVHSVGAIIMEPWINHENVTAVVFAGLPGQESGNSLTSVLYGDSNPSGKLTFTVAKKASDYTTDGPLLYEPNGPVPQQKFSTLNIDYKHFDAKK
IEPRFHFGYGLSYTTFSITGGQGVRVSSAPYAPRSSFKQGAPPALRIPTGNLNAQDAVYPADFVRPNKLHIYPWITSAELAKPGASYPYPAQNATPPSPAGGGQGGNPS
LWDVLYRVTATVTNTGSIAGAEVAQLYVAFPSSKEFPSPPRQLRGFSKVYLQPGQAAQVTFDVTRKEISVWNTVTQNWQLVSGTYQFLVGDSSRSYSLASESQFA
>tr|A0A1E3QEV4|A0A1E3QEV4_LIPST
MKIRTSLLTAVVLVNAVLAASSTDEDYGYSLPVYPSPMGGRISGDWSDAYTKARAFVSQLTLLEKVNLTTGTGWEMGRCVGNTGEIPRIGFPSLCLQDSPLGIRFTDLN
SVFPAGLATAATFNKNLMYLRGKAMGMEFKGKGINAILGPCMGPIGRAPEGGRNWEAFGADPYLQGVASYESVLGIQEEGVIATAKHYLLNEQEHFRQYAEWVGLYTNL
SAPYSANVDERALREIYAWPFADAIRAGAASIMCSYNQANNSQACQNSYLLNGILKDELGFQGFVMSDWLGQRSGVASVLAGLDMTMPGDGLVWSDGVSLLGSNLTTAV
LNGSVPLWRLDDMATRIMAAYYKVGQNPLTYPEPNFSSWTLSDYGLEIAGSLEGPTGLVNKHVDVRTALSRQVTRQIAQESMVLVKNTNNALPLNNVRRLGLFGSAAGP
PPLGPNGCSDRGCDNGTLGIGWGSGTANYPYLLTPLEVINAMAIVQGTSVQYNLDDYDLDNVATLAGVVDTAIVFITADSGEGYLDVEGNIGDRNNLSAWHGGDNLVTA
VASENSNTIVVVESVGGIDLEAWIEHVNVTAVLFSLVPGQDVGVGIADILYGTVSPSGKLPFTIAKKTSDYPATVIYDVIEPVPQINFSESIFVDYRHFDKYDIEPRYE
FGFGLSYTTFQISGLSVTRLNGVVDSEVPPEPPSYSSPLYLNGSVPSPSSAIYPGDIPRYSHYIYPYIEKDIAESITYNPDDYPYPEGYSDIQPSKPSPAGGASGGNPA
LWDVLVQVKASITNNGTTGGSEVLQLYVSYPAVDGVDFPVRALRGFEKLSASPGETSTANFDLLRRDLSYYDVASRNWLLPRGTYTVSIGTSSRNLVEAGTFSF
>tr|A0A2P7QKD4|A0A2P7QKD4_9SPHN
MKISANVRPAQGQAWTRALALALLLGTAACTAATTESGVETAPAAPSAPADAPAQQSASADHVRWPSVKSGVARDPAVEARVEKILAGMSVEEKVAQTIQPDIASVTPA
DMRRYKFGSILNGGNSAPGNNETAPAREWLALADAFWDAAMSAEWAGEKIPPIWGSDAVHGHTNVVGATIFPHNIGLGAMRNPDVIQKIGEVTAAEMALTGIDWDFSPT
LAVVRDDRWGRSYEGFSEDPEIVRSYAGRMVEGLQGRPGTPGFLGAGKVISSAKHFVGDGGTSAGKDQGDNPSTPEELRDIHGAGYPPAIEAGVQAIMASFSSVRGEKT
TGDRDLLTVALKEDMNFDGFVVGDWNAHGQVPGCSNTSCSAAMNAGLDMYMAPDSWRGLYDSTLAQVRSGEIAQARLDEAVRRILRVKIRAGMFEKPKPSARPMAGEFN
RLGSPEHRAVARQAVRESLVLLKNQNKLLPLSPKRNVLVAGDGADNIAKQSGGWTITWQGTGISNKDFPNGQSIFGGIAETVKAAGGTATLSTDGSYSEKPDAAIVVFG
EEPYAEFVGDRPTIEYSPADKKDLELLQKLKKAGIPVVAVFLSGRPMWVNPEINASDAFVAAFLPGSEGGGVADVLFADAAGKPRNDFKGKLSYSWPKRVDQSPLNRGD
TNYDPLFAYGFGLTYADAGNLAPLSEERPAGARTGADGVLFGRGTVPGGWTLGLRDEGGPASPVAGNAGATGTGRLRVAGVDRRAQEDARRFTWSGQGWAAAQILAAQP
IDLTREANGELSLILDYRVDAAPSAPVTIGMDNATLPIGGLLRAAPVGQWASVAIPLRCFATAGVDMSRIAIPFSFATAGRLTLSISDVRVASAAGPQGRCGQE
>tr|A0A2M8H3D1|A0A2M8H3D1_9VIBR
MTTDNTHIPYFSEWPKINSAIPSDPTIEKRIARIVSLMTIKEKVGQMIQPNLRDVTPQELREYKLGSILNGGGTWPNENKHAGAREWAEKADEFWYATEQAFEHRPFRI
PFIWATDAVHGHNNVFGATIFPHNIGLGAARDPELIRRIGEVTAREVCATGLDWTFAPTVATPRNLRWGRTYEGYSEDPEITYLYASAMVEGLQGDAATLKSDQKVISN
VKHWVGDGGTGQGTDRGNNTYSEDLLRNIHAMGYFSGLTAGAQVVMSSFNRWENPSNYDHSPEYGTTVYNEKLHGSRYLLNDVLKQQMGFDGVVVTDWHGHSEVSKCSD
GNATYAINAGNDILMVPVRQHWKAVYEQTLRDIENGHISIARIDDAVTRILRIKMRAGLWDKPTPSKRLLGGNNAIVGADKHRMIAREAVRKSLVLLKNNHNLLPLANT
TKILLTGSGADNIQKQCGGWNLTWQGDENTRKDFPGSCTVKDALTQEIGSQRLLFDPELTSDLSVAADVIDVAIVVFGEDPYAEMMGDIKEWQTLEFSALKRSYRRDVA
KINRLREMGIKVVSVFFSGRPLFLNQEISASDAFVAAFLPGSEGRGITDVLIGDENAQPRYDFNGRLSYSWPNKKMSAAVNRIPPHIPGYQVPDSEQDPNSEHAPLFPY
GYGLCYRTAEATHMDLDHLPLDLSSAQEGSTILELYGVRATIGDYLLSARSPAREEGFEISRNNPIQTPWLKTIPYNYQLQQDAVELECYGEDSVLYLQTSDKEDDDLN
FFVDNHGVITFSVQVLQIPDGKVLLSTGPDCIVQQHISDSCGAFDITRQLQEASPGEWTEITVSFAELEKAGAECRHTDIPFVFRISGRLHMVLGNVRLTTVS
>tr|A0A2P8A4S3|A0A2P8A4S3_9PEZI
MANRLLRLLVALLFTCLVTTGLGAPTTSSAVPSSTAPSASQASAVLSQNAVSGSNNPVALAISGLVKDAASLARVVAWAAAWAQFALIKLFTPELIYSYGKSPPVYPSP
QGAGDDGGWASAYSQAKALVAQMTDFEKANITFGISENKGCSGFTGSVPRLGFPGICLNDAESGVRTGKLVSGYPAQLHVGASWNRKLAGDRATAIGKEFKKKGINVLL
GPVVGPLGRVAKGGRNWEGFTNDPYLAGSLVEPTITGMQRSVVACVKHFIGNEQETNRSPFLQGFIPGLANESVSSNIDDRTMHEAYLWPFYDAVRAEAASIMCSYNKI
NGSYGCANSKTLNGLLKTELGFEGFVVSDWYAQHTGIASNNAGLDMVMPSSQFLNPNSLAGAVANGSVSADRLNDQATRILAAWYRFAKVDAPGQDLYADVDARDPADE
AIIFQSAVEGHVLVKNNGVLPLNKPATLNLFGYDAIGGSNTSGLDSTLYGFGQSNTRTYTNGLPYTGLQALLLFASVLPAGSAGPEVALQGTIITGGGSGAITPVLSYA
PYDAFVQQAAQDGTTLFTDFSSQRPVVRAPNDPCVIFINAASSEAYDRTTLADEYSDTLVTNVANSCKNTMVVIHNAGIRLVDRWIDHPNVTAVIYGHLPGQDSGLALT
EIIYGRQSPSGRLPYTVGRTETDYGSLLNPSFADSKNPLYSQSTFSEGLYIDYKSFIQRGVAPRFPFGYGLTYSNFSYSALSISKTSGVNTSPLPSDRLSGGSTPAPQGG
LASLYESIATVSVKVSNTGRVAAAEVAQLYLGIPNSGLPKALRGFDKQIVQPGQSAQFSFPLRRRDLSIWDANQQQWVLQAGSYNVQVGKSSVENIVLTGSLTI
>tr|A0A0R1J4Q7|A0A0R1J4Q7_9LACO
MENTNSNGISNTVLSKKIAQEGMVLLQNNHHVLPLKTKTVAVYGSGAFATVKGGTGSGDVNQRTTSIVEGLENSGFTVTTKAWLNRFQRYYQAKEAEYSKDTNILSPAF
SMDDPEISDFNEAPVGIYVISRSSGEGFDRKNEAGDFQLSENELSNIQRLSEYYENSVLLLNVGGIIDTSFVDSCPLLDSILLISQPGMTAGDAVTEILDGTKTPSGKL
SDTWADYQKYPAINNFGVKNPEYSEGIYVGYRYFDSFQIKSRYEFGFGLSYAQFDIKTKRVSVNEKEISIKVEVRNDSEKYRGQEVVQVYISNPQSDIPTPYQVLRAFG
KTKILKPHESQELDLTIKTEDLVIFDERNAAFVLTPGIYILRVGNSSRNTKVVAEMKLDRKTIIKKVEHKVCPQFDPTKLRKNKVKLQRTSGVPFFLLKSENFDQGDPI
KYHDFSEVVTYTVDDKKLPGKYFNEKTITKTKVNNLKLSDVYNKKVSIETFVANLDNKELVDLVEGSLEESNDSIIGNASSLVKGAAGQTVENKKRGIPETVNADGPAG
LRLDKSYNNTAWPIGTLIAQTWNTKLGQEMGSAIGEEMKRCGVTLWLAPGMNIHRDPLGGRNFEYFSEDPLLSGIMAASETKGVQSHSGLGVTIKHFLGNNQESYRNTG
NSIIGEQALREIYLKNFEIAIEEEQPLAIMSSYNMVNNYFSGANFEALTNILRDEWNFEGLVMTDWFSAADPRESMHAGNDLIMPGASKDTLFAAIDDLKPKFNEDGSI
ATKQVLNPAEMKLDTIELWNDFIPDVDGNVLIKVKVSSDKISEKVKELFYSGSIQVLDDSHIIIQGKWKDNNDLNLGDIQKSAIHILETIMKTDKFKSLMA
>tr|A0A495JDW0|A0A495JDW0_9ACTN
MPAINVRAGGPHHVAPVRSVSRAQAILVVAASLPPSARTRTGVEASRPMRPKAGCGGDWGDQMTTVKPETGATYVDATAPVEERVSDLLARMTREEKLAQLGSTWAFAV
LEGGRFSVRRARPILAQGLGHVTRVAGATSLKAAQVAQVANAIQRYLVETETRLGIPAIVHEEVCSGVMAREATIFPQAIGVASTWSPELNGQLADAVRAQMRAMGSHQG
LSPVLDVVRDPRWGRTEETYGEDPYLVARMGVAFVKGLQGADLTNGIIATAKHFVGYGASEGGLNWAPAHLPPRLLREVYLYPFEAAVREGGLQSVMAGYHELDGIPCH

ANRELLVDILRRQWGFGGSVVSDYFAVNDLHSYHHFGQDQQQAATLGLGAGVDVELPATDAYADALTRALDAGEVSEARLDEAVSRVLRHKFELGLFEAPYVDEGAVVA
AVNAERHRDVALEIARRSLVLLKNDGVLPLLPEAATVALIGPNADDARHLLGDYSFAAHIEALTEARERRGLLGEMMTIPDDLEIDESTDGVPTVRDELMARLGDRLSY
VPGCDVVDPSTDGFDEAVAVAAADVAVLVLGDRSGLTPRATTGESRDRSSLDLPGVQEDLVRAVVATGTPVVAVLVAGRPVGSDFLHEQCAAVLMAWLPGQTGAQAIA
EVLLGEVNPSGRLPISYPRSVGQIPVFYGHKVSGGRSHWHGDYVDSPVAPRYCFGHGLGYASFSIDEATVRHAEVRVGDSVSVEVVVTNTGHRAGEEVVQLYVRDPQAT
VTRPVLELKGFARVSAAAGQRVAVRFDLPTGQLGFYDRDMNYTVEPGQIEVYAGFSAHDRTLAGSVVISADADRSPTPKVYTGTAQVRVLPNGASLPTLD
>tr|A0A1E5LIC4|A0A1E5LIC4_9BACI
MNKRTVWNKLFTIVLSISMIVGTFAAFFNTSNVASAEEMPLYKDANASTPDRVADLLSRMTLDEKIGQMTQVERNSIGADSNLATFNIGSVLSGGGSAPSPNTPEAWAN
MYDGYQRAALQSNLQIPIIYGIDAVHGNNNAYGATIFPHNIGLGAARNPELLREIGRATAEEVAGTGVDWTFAPCLCVSRDERWGRTYESYGEDPEIASSYVTIIEGLQ
GDLRDSGTILANAKHWVGDGGTLGGDDQGDTVLSEQELREIHIPPFIDAIEAGVGSVMASFSSWNGEKLHGHKYLLTDVLKDELGFEGFVVSDWAGIDQLPGDYASDVR
NSINAGIDMVMVPQDYRTFINTLRIEVNEGRVPMSRIDDAVSRILTQKFELGLFEQPFTDRTYTSTIGSQEHRDIARDAVRQSLVLLKNEGDILPLDQGLDKVFVAGKN
ANDIGNQSGGWTITWQGQSGDITPGTTILEGIQSTVSPSTEVTFNESGDGINSSYDVAVVVVGETPYAEGEGDRPNDLRLDQTDLATLQTIQSTGVPTVVILVSGRPMV
VTDELANWDAFVAAWLPGTEGDGVAEVLFGVDGYEFTGKLPFTWPRSESQIPMNVGDASYDPLFEYGFGLEYGDVVIPPVVDPIDAFERIEAESFNSMSGVQTEETTDV
DGGENVGWLDNEDYLVFNKVDFDDGATGVEARVASQSAGGTIEFRLDSTNGELIGTVDLTNTGGWQDWVTNTAAVSNATGIHDLYVVFKGETQEGIGNLNWFVFTKEMG
TTPPTGEDKIETDEYTIEMVNDDPTSVTFIFTPTLETSSFVDLHYKVNDGVQQNVKATENGDVWEYTVTGLQEGDSLSFYYTYEKEGLAYDTPEYDYTN
>tr|A0A3N4LVN3|A0A3N4LVN3_9PEZI
MYGKSLTLLVLALSMATASPVEFVERQSEDPFHSPPYYPAPKGGWAPTWAASYKKAQKLVEQMTLAEKVNVTTAVGWMMGRCVGNTGPVERLGFPSLCAQDGPLGIRYN
DHNTAFPAGITTGATWDRDLMYERGKGMGAEAKGKGIHILLGPAVGPLGRQPRGGRNWEGFSADSFLLSEASYHTVKGIQDAGTQATIKHYIANEQEHFRGDGGSNDTI
SSDIDDRTMHEVYLPPFAAAVRAGVASVMCSYNTPQINASYACENSKLLNGILKDELGFQGYVMSDWLAQRSGVGSALAGLDMVQPGDGNLWADGNSLWGPELSRSILN
SSIPVDRLNDAVTRIVAAWYQLGQDKDFPHVSFSSWTFNDTDIEFRGADMGRVMLVNEHVDVRANHGDIADTVARDAITLLKNENNVLPLSKSDTIRVFGQGAGNNPEG
PNACPDRGCNIGVLTQGWGSGTSQLPEDLIAPIDAIKEIAHDVEWYKYDNVTAEVKKMASARNAKCIVSITADSGEIFITVENNVGDRKDLKAWHNGAALVKAVADSCK
NTIVLIHSVGPIFMEEWINHKNVKAVILAYLPGQGTGYPLTDVLFGKVSPSGHLPYTIGKREEDWGDVGIVTEGKGVIRDQFKEGLYVDYKWFDKQGISPRFEFGYGLS
YTKFQFSDLEIEAVRPLTEMPAPPPARGWTPVYDTSIPPASEVAWPKSITTRIPKYIYPYLGNATDVQPGEYEYPVGYQTDPQPAPIAGGAEGGNPALYDCMFRVRVTV
KNTGKMTGKAVPQLYLEFPKGIVFDTPIRQLKGFDKIQLRPGEKKVVTMELLRKDISVWDVVRQQWILPQGGRNGYTVHIGHSSRDLVLKKETPKLA
>tr|A0A5E8B4B1|A0A5E8B4B1_9ASCO
MKFQIITSLTALYLTVLTVAQFTSPQYLKSPIGGRLTTDDKWSKAYDRARSVVSQMTIPEKVNLTTGSGWEADLCVGNTGSVPRFNIPSLCMQDGPLGVRFTDFISAFP
AGITAGASFNKELIAFRGKALALEHKAKGVHVVLGPAVGPLGRNALGGRTWEGFGADPYLQGVAARATTREIQKAGLVATIKHFIGNEQEHFRQVGEYYGSGPISQSLS
SNIDDRTMHEIYMWPFADVVNEGVGSVMCSYNTINNTYACENSYLINKLLKEELGFQGFVMSDWWAMKTGIFSAQAGLDMAMPGDSGYFTSPDTFWGNRLTRAVLDGSL
GDWRLDDMAVRILAAYYYVGLDSINTTQSGPNFSSWTKDTWGFEHPLGNVGWREVNKHVVASNNDLSKSAALQVATEGIVLLKNSGNVLPLVGGSLNPRKINIFGKAAG
PDPRGANCKPDFACSDGALGQGWGSGAVDFQHFVTPFEAVNDAARKIGMTVDYDFTAFNLDGFLSKVSIADANIIFALTNAGEGFASFDSNYGDRRNASLWHNADEVIL
RAAEKNKNNIVVISSVGPTNLERWIDHPNIKAVLFALPGGQDAGIAVANVLFGLSNPSGKLPFTIARDDKDYIPIVTSPGNGKPQADFTEGIYVDYRLFDKNGLTPRYE
FGYGLSYSSFGLSDFKIESGTAPSEQLPQPPSLKPVSALACNKPTNADAYTFPPGFAAVPRYIYPWLSNSGDVGNVKGSCINVTDAISSAPLAGGGLGGNPALFETAYT
LSVTAVNNGPFPGSQAFQLYVEYPQGGKYDVPPRQLRGFEKPYLNVGQSAKLTFKVQRRDLSVWDVESQSWVVPRGKYRFLVGNSSRDLPLSAEITI
>tr|D2RV76|D2RV76_HALTV
MDGMSTDNNHDGVDESRRTFMKATGAATAAAGLGATSTAAGDEKRSKRLEDLIDGMTIEQKVGQMAQVAIDNLGEGFGPDTAFNDHDDAGTLGKLFSELHVGSILNGGA
TGPTFDGEEFVEGLNGLQEYNLEVNEPAIPFVWGCDALHGNCLLDGCTSFPQRLNMGATRDVDLVEAAATHTGDSVAAIGGHWNFGPTLDVLRDMRWGRYFEGHSEDAM
LLGEMGRARARGFQRNERVAATVKHFAGYGTPNTGSDRTHARTSMRDLRTRQFEPYRRGLEEAKTVMVNSGAVNGKPAHASSWLLTTVLRDRFGFDGVVLTDWDDFERM
LSNHEYLPDTDDGWREAVRQGIEAGVDMHMCGGETAPTEFIDTVIDLVESGDLSEARIDESVRRILELKADLGLFADPLAPEDEIGDIVGGAADISEQLAKESLVLLQN
EDDALPLALEDVDDLLLTGPGVHEGTPNRFLMQHGGWTLGWQGIEDGNLTEDGPRPRQNTIEGELTARLGDGLTHVPTEYEPAVYESLYENFDNGFFDVTDEQAAAISE
AAPGSDAVVVVLGEGTHNEGFGDRDKMRFLEAQRELVELVDSETGDDVPIIGVILAGSPRGTAETFQHLDAVLFAGQPGSDTGVAVVDTLFGDYNPSGKLPFTWESHVG
HVPQIYDEYPPRHPDGAGDQMVQFEFGHGLSYTDWEYVDLSLATDSVKNPASRPTVTAHVTVENAGETAGEHVVEIYNTESYGSVLQPHRRLMGFERIALEPGERETVA
VDLDLSTLEVVPGDVPGWGPRVVEAGEYELAVGADWGVNASDDADDGATATLTVGKTASITDPEPTPGRYDIDGDGDEDFEDVMALHRRLKHRKWRR
>tr|A0A0N1ANH4|A0A0N1ANH4_9SPHN
MPEMPVNRFRLSTYLSTAAALAFGLGLSWGGAACAQTAPSEAALPAAAASAVAHPALWPQARWPWRQDAAMEARIDALIKRMTLEEKVGQILQADLSTVTPDDVHRYHL
GSVLNGGNAGPGGNDYAPAPEWLKLADAFYDASVDKRDGGVAIPVMWGTDAVHGHSNIVGATLFPHNSALGATHDPALLQRIAAVTATEVRVTGIDWTFAPTITVPQDT
RWGRAFEGYSEDPALVASYARPFIEGLQGKPNSGPLLAGPHVIATAKHFLADGGTFEGRDQGDAVIDEATLIKVHAQPYVSAIDAGVLSIMASFSSWHGVKMTGNKSLL
TDVLKKRMGFAGLVVSDWNAHGQVAGCSNASCPQAVNAGIDMLMAPDSWKALYHSLIAQVQDGTVPMARLDEAVGAVLRVKMRAGLFGAGRPSSRPYAGQFDLLGSAPH
RAIARQAVRESLVLLKNEAGILPLSPKARILVAGDGANDIARQSGGWTLSWQGTGLDPKLFPGATTLWQGIDTAVRAAGGQATLSPDGSFAGQKPDAAIVVFGETPYAE
FQGDLRSLQLKPDLRAPLETMRKLKAAGVPVVALMLTGRPLYTNPFLNLADAFVVAWLPGSEGEGVADVLLRGSDGSVAHDFTGRLPFRWPMTAVPGGATLYPLGHGLS
LKAKAPRWVPLPEQSGVDDSAGSTTEYFQKGVPTPGWSLEIQNAGTGGVTRITSVPATVADSGVAITASDYQVQEGARTFAFSGSKGTAALALTTHAPADLSRETNGEM
MLVTTIRVDTAPTAPVTLAMDSAGKATSLSLGKLDMLPRGTWTTLGIPLQCLRTAGARMDAVDAPLILSTGGAMTISLARVKLGMDAQVRLPCATH
>tr|A0A545SLY5|A0A545SLY5_9GAMM
MTHLRTALFLGLLLTILHGCSAPPPSGIDQPAAVAGNLERWPQLAARPADPAIERRIQALLAGMTLEQKVGQLMQPEIRHVSNDDVRRYYLGSVLNGGGSFPQRNKHAS
LDDWLSLADGFYHASMSVDADVKIPIVWGTDAVHGHNNVIGATLFPHNIALGAIDNPALIEAIGAATAREMAATGISWSFAPTVAVARDDRWGRTYESYSEAPALVGRY
ARAQVLGLQGRRDNGTFMGEGRVIATAKHFIGDGGTAGGEDRGDTRLTEEELIRIHAQGYFRAIEAGVQVVMASFTSWNGDKLHGHRYLLTDVLKNRLGFDGFVVGDWA
GHQFVPGCTVTRCPAAINAGLDMFMAPDDNWRELYTNTLADVKAGRISMARLDDANARILRVKLRAGLFEKGAPSSYPLAARQDIVGHPEHRELARQAVRESLVLLKNR
GQLLPLSPKAHVLVAGNGAHNIGKQSGGWSITWQGTGNTNADFPGATSVYDGIKAQVEAAGGRVSLSENGDYSEKPDLAIVVYGEDPYAEMQGDTSHLGYHNADDLALL
RKLRDAGIKVVSLFVTGRPLAINPYLNASDAFVVIWLPGTEGKAVAEPLFTKPNGDVQAAFTGRLSFSWPQFADQAPLNLDDPLAQPLFAYGYGLANGDTDTLPAALPE
PTAGTLAATPGPLTIFHRRTLSPWRLDLAAKGEQPGTMSTNSLTVGAINAQTRDRFMQEDTLEITWTGPAQAGFYHPSQINLSDFAGGSLVFEARLKNAADIQVGLSCQ
NTCRSTHLSEFVNPGVETWQSVRIPLACLSDDLQAVASPMQLSGRQGAEIALYNLRIERGNQTCPNPSDTPLSNDVLINRSAPYNLNNGAGKPALR
>tr|A1S5F5|A1S5F5_SHEAM
MKFALSKTTLALACVLGGVMSGKVSAAESSSLTERDVNRWPEAVYHTPIQADVEAKVHKLLAAMTLEQKVAQIIQPEIRDFGVEDMRRYGFGSFLNGGGSFPGNNNRAK
AADWVALADQMYHAAMDDSIDGIAIPPMWGTDAVHGHGNVFGATLFPHNIGLGATQNPQLIKAIAAATAKEVRATGIDWVFAPTVALVDNLRWGRTYEGYARDPELIER
YAEAFVDGMQGEGKSWLGDDYTLATAKHFIGDGGTDNGDDRGDTRVDENTLIARHGQGYVGALGHGVQTVMASFNSWNGEKLHGSKYLLTDVLKERMGFDGVVVGDWLG
HGFVPGCSYEHCAEAVNAGVDILMAPGDSWKALYANTIADVKSGVLPLSRLDDAVKRVLRVKLRAGLFDNKAPSANPYAGKQEWIGHPEHRAIARQAVAESLVLLKNNR
PANGARPVLPIAANARVLVVGEGADSIPQQSGGWSMTWQGTEVTNADFPGATSIFAGIKAALNAAGGDALLSSDGTIPVGFKPDVVIAVYGEQPYAEGNGDLDNLEYQR
GDKRSLAMLSALKATGLPLVSVVLSGRPLWMNPEINVSDAFVAAWLPGSEGAGVADVLIGDKNAQPRADFKGRMPFPWPATPSADGFVSDTGSAGQDQPKPLFSLWQGF
DYRSDATLAALSEDNGSSEADNRLAIFDKAIKAPWHLAVGDDKGLHRVGPGLWQQGPWAVRSVNRIVQEDARRFEFGAAGTLSFRDDFTLDLRRFAPDSSLLSFDIALT
ALPGKLQLSMVCEGGCRQAVELSGQLKADGQWQRVEVPLSCFGVTADELARTFSPMTMSLPQGGTLTLANVSLEGMVQRDASAKAVECGLPPVSDK
>tr|A0A1C7D6Z0|A0A1C7D6Z0_9SPHN
MTRRAIRFALSTTAATLLAACATNAAMDGPMAERSYALPAADVALDLDKWPVIDSAIPAAADAATEARIDAVLSRMTVEEKVGQILQPEITNITPDEVREYNIGSVLNG
GNGRPNKDMYAPASEWLKLADAYWTASTDKSDGGVGIPIIWGIDSVHGNNSYFGGTIFPHNIGLGAANDPDLMRRIGRVTAIETAAIGTDWTFAPALSIPRDDRWGRTY
EGYSEGPEIAGRMGASLTLGLQGDPAGDFFPSGSIIATAKHMVADGGTAGGRDQGDAQVSEDVLRDIHWAPYAPTLEAGAQTVMASYSKWNGVRMHGHGPLLTTMMKDH
AGFDGFVIGDFNGHALIPGCTAGDCPEALLAGVDMYMIPADWKELYNNLVVQVKDGTIPMSRLDDAVRRVLRVKFRAGLFDMPKPSARPYAGAEHIGTDEHKALAREAA
RKSAVLLKDDPSILPFPANSRVLVAGQGADNVPMLVGGWSMNWQGTGLTNKDFPGSTTVYSGLRQAMAPHGGTAELSVDGQYETKPDYAVVVFGETPYAEYQGDRESVI
YAAPDSDSDLALLERLKADGIPVIAVFLSGRPLWVNPHLNASDAFVAAWLPGTQGGAGLADLLVSDGAGKARHDFTGRLSFSWPKLASQTALNQGNANYDPLFPVGYGL
SLNSRRDVGMLSVDPGLSAEKLAAASNSVFFSDGRAIAPWRVYLGDAQNSRLKTEGDRFVTSPTGAVSFDSADRNRQEDTKIGRWSGTGEGSVFVLGYDTVDFGKRAEN
GEAVVVSMALLERPTANVTASFGTTRKSGTVDLTPALRRMAPGKYTEVSIPLACFADDSDLTTVDMPLRIATNGKLAVQLAGISIAPPRGITVEC
>tr|A0A6S7CJ32|A0A6S7CJ32_9BURK
MLHRNANAGFPLTLRLTQKNGREDRQFRNRGGACFRYACPSSGPASLLAAIARAVRRPFEDFLLAMSHPYQDPSRPNSERVSDLLARMTIEEKIAQMHAFWLILSESGD
HRTRSDSFTGASDQAQLQNRLKLGLGQITRPLGSHSVDPVSGVRALNHLQKFLREETRLGIPALSHEECLVGVMTRGGTMFPSALNYGTTWNPELIEKVATAIGTEARS
LGCHQGLAPVLDVSRDVRWGRTEETFGEDPYLIGVLATRYVRGLQGDKRDLLATLKHYVGHSFSEGARNHAPVHLGPRELNDIFLLPFEMAVKQANAGSVMPAYHDVDN
EPAHSSHYLLTEVLRNQWGFDGLIVADYVGVSLLYQHHGVAADPAEAAALSFNAGLDVELPGDDCAAHLREALARGQITEAKIDEIVSRVLTEKFRLGLFENPFVDENK
IDLQNDNAKALALQVAEESFVLLENDGILPLAPDSGKKIAVIGPTADDPLAMVGGYSFPVHLIISDMTSETSEIVTPLQGLRKVYGDAAIRHAPGCVILAQRKAGSPVF
PGDVTDGSTLNLESPVSRDTSLIDAAVQAARESDVAIVFVGDLAGLFQTGTVGEGSDTDSLNLPGVQQQLLEAVVATGKPVIAVITGGRPYILNGLEHKLAALAMAFAP

GQRAGDAIANVLSGTASPSGRLVVSVPKSVGAMPYFYNHKLKSAGTPIAFHFGSRYPFGHGIGYTSFSYSGLSVEQSELDIETGEIVASFDIRNEGGRAGVEVAQLYVR
DELASAVRPVKELKAFARVALDAGETARVTFRVPVDMLNLTNAAHRRIVEPGVFELQIGPSSADVPLQAKVNVTGQVRELGRLWRMESTSSINRR
>tr|A0A176VUW0|A0A176VUW0_MARPO
MAAIKACSIWRGFTVSGSPPASRVHEYLAVAGAHDMTAPLRLCCQLNSKPSHWYFVWTFKQRPALTERRMKSHVLVTAVLLLIQHLLAQKTNGDSSRSSYACTSRESKS
YSFCDKALSIDERVADLISRLSLEEKISQLGNGADAISSVDVPAYQWWGEALHGVAYCPSVHWDGPVKGATSFPMPISLAASFNKSLWNKIGQVISTEGRAMHNLGQSG
LTFWSPVINLVRDPRWGRVQETPGEDPYLIGQYSVYFVRGMQEAKDYDPSNEDDNENENFALKTSACCKHYTAYDLDNWQGVERYDFDAKVTIQDLADTYNPPFQSCVE
EGRASCLMCSYNKVNGVPTCADPELLKGTVREKWGLRGYIVTDCDSLLVMYDESRYSKGPAEAIATAMLAGLDLNCGATIKTYGASAVQQRLISEADIDRALNNTFTVL
MRLGIFDGNPLDQPYGRLGDDHICSEEHQDLATEAAVQGMVLLKNDEDTLPLSSRHIKTLAIIGPNADDTKYTMLGNYAGRPCTYITPLQGLSTYEINNVIFEPGCSSI
DCSSDEKIEDALSAAKKADVVILVMGLDQDLERETFDRNSLKLPGKQELLVSSVANVSNGPVVLVLICGGPLDISWAKNDSRIQSILWMGYPGQAGGLALAQIVFGDRN
PVGRLPVTWYPESITDWPMTCMNMRPDPHTGYPGRTHRFYNGPTVYEFGYGMTYSKSTTMEVTGPTNFTAPALFQQACYHKRFTAEQNDSTGCSPSDLVACLEASLPIN
ITVKNRGPRAVTEVALVYHVPPRAGENGTQLKELIAFERTEVQPFSSKKIHLIINLCHHTSTVKSDGKRSLQLGTHKVVVSPGSGTSAEHRFYVH
>tr|A0A1V3QPG0|A0A1V3QPG0_9GAMM
MLECAASVLAADTAAAKDVPSLAAPSASDGVPERTRYTDWPAIKSRVSTDPALEARVRHIVASMSLAQKIGQMTQAEIKSITPAEVTKFYIGSVLNGGGSWPGGSKHAG
IADWLALSDRYYDASMATDAAIKLPVIWGIDAVHGDNNVFGATVFPHNIGLGAAHDPALIEEIGAATARAVRATGVEWAFAPTLAVAQNARWGRTYESFSSEGPLVRAY
ARAYVTGLQGGFGDHNVMATAKHFIGDGATWNGTDQGDARVSLSDMINVHGSGYFGALEAGVQSVMASYSSWDDVGAGINYGKMSGAHALLTDALKEKMGFDGFVVSDW
NAIGQLPGCSNASCPQAINAGIDMVMVPDDWKAFIANTIRQVEDGQIPMARIDDAVSRIVRAKLRMGLFGTRPSQRAGAGAAGSLQSRELARRAVRESLVLLKNNHDVL
PLKPGRKILVVGKNADSLPNQTGGWSLTWQGTGNGNADFPHGETILDGLRQAAGPANVTYSETAQGVDLQPFDAIVVVIGETPYAETLGDIMPSATLRHSDRHPEDLAV
LQTVAKGHKPVVTVFVAGRPLYVNNLLNLSDAFVAAWLPGTEGAGVADVLFAGHAGAGSYNFSGTLAMPWPGVPCPDASNGSAKATRWLFAPGYGLRYPAKHAMGMLPT
DPAVDACAEASVLSVFHTLAVPPFSMYLAGANPEQPAHDIGSDLNAVIEWPASHPLLRLRTVQVNTQQDAKSVSWLGAGRFFARSPQPRNLMPLLATHAALQFDVVIAT
PAKGPLKVYMGCGDHCTTSVDLAQTFGRYANGTRHTVSIPLACFARNGADLSHVDVPFGVLASPPFSAAFADVKIVAGTAAGRTDLPCASLGAP
>tr|A0A401KYP2|A0A401KYP2_ASPAW
MVRLFVHALLLLVGSSLAIAQRPEIPDDIPLDKGTRDYLESLPPDELAAVLEVYQEAFAGQVPAPPELITQQELSLYGKSPAVYPSPLAKGTGTWADAYAHVKALVNQM
TNEEKANTTSLSDSGVPAIPRLGFPGLRLHDGPNGLNALEEVTAYASGITVGASWNKDLAHARGQSMGKGARRKGATCWDRLLVRLAGQRPGGRNWESFSVDPYLCGQM
GAKTVLGIQENVIATAKHFVLNEQETDRNPSMFGFSNASVSSNIDDKTMHEYYLWPFQDAVKAGVGSVMCSYQRVNGSHSCQNSWTQNGLLKTELGFQGYIISDYNARW
GGIASTQAGMDLVTPPSAVWPQNLTIAVENGTLAESRLDDMVIRILTSWFKFAQFDPANPLGLPPNISKAHEGSSSIDPSPEAKYIIRQGAVEGAVLVKNVNNTLPLKK
PKILSLFGYDAHAPLVNSPTGVNSKYSIGYQSVNRTKVQVQGLFVGVGECPSAATLGTLVGGGGSGSVTPQYINSPFDAFQQRAYEDGTFLYWDFDSEDPSYSESDACL
VFINEFASEIVDRVSLADQYSDNLVMNVAKKCRNTIVSIHNAGVRLVDRWIDHPNVTAVIFAHLPGQDAGPALVDIMYGEQAPSGKLPYTVAKNESDYSEELLWPVRPD
NSSNYYTHADFTEGSYIDYRRFDALNIEPRFEFGFGLTYTTFNYSDLEVRHTEANSSFYPPPGEVMEGGLESLWDVVAIVTARVSNTGEVTASEIPQLYVGIPGAPIKQ
LRGFEKIPLHPKEGKSVSFPLTRRDLSLWDTESQSWYLERGEYKLYVDLLALADGDQILGIVAGSNVYQDENCTPITVPNAPSLSDLFCDKIS
>tr|Q0AL31|Q0AL31_MARMM
MTTKTQKLASVSMIATLISIGLAGCSDGTDTAATERPAASSSPAVYTPPADAMADPDAWPQIAPLPLDPEVEARIDSIMAMMTVEQKVGQTIQADSGSVTPEDVRNYRL
GSVLSGGNSAPGPRAYADAQTWLDAADAYFNASIDPDGVEIAIPIIWGIDAVHGHANLRGAVVFPHNIGLGAMNNPDLIEDIYRVTARELSVSGHDWTFAPTLAVPRDD
RWGRTYEGFSEDPAIVAAYGERIVWGLQGRPGTDEFMGEGRVISSAKHFLADGGTLDGRDQGDAQISEAELRDIHAAGYYTAIPAGVLTVMASFSSWNDARMHGNESLL
EDVLRDRMGFTGFVVGDWNGHGLIPGCVSTDCPESFNAGVDMFMAPDSWRELYHNTLAQVRSGEISMERLDQAVRRILRVKIEAGLFEQVAPSERPLANSDTVLAAPEH
RAIARQAVRESLVLLKNVDQTLPLDPSLTILVVGAGADHIGKAAGGWTLSWQGGEFPNEEFPAGDSILAGIQAAVDTAGGQVIYDPDGQSDIDADVVIAVYGENPYAEF
QGDRDHLDFVSPDYDTSLLEQYQARDIPVVSVFLSGRPLWTNPEINDSDAFIAAWLPGTEGGGVADLIFRTDDDYDFTGRLSFSWPSLASGEPLNVGDADYDPLFTYGY
GLSYADADPGTPLSEESGLSTDDLAEGGSYYARGQVTDPWALQIANGQDWEAIEDRMASSDRVTLGAMDRVAQEDSLRIIWSSAGNLVSIRGTDAIDLSRETNGSMELT
FRARGVNSQPAQVEIGMACDTGPDCVHYVPVDIRQGPWSEYRISLSCFADAGVDMSRLDTPFLIRARDETGEIGIGDVTLGSDIDATRTCGNE
>tr|A0A162J2L5|A0A162J2L5_9PEZI
MPLSLRKTAIAAVPFLATAPAPAQAMMTFPDCVNGPLANNTVCNPRAAPAARAAALVKAMTLNEKLVNLVNTSPGAPRIGLPPYEWWSEALHGVAASPGVSFNRSGAVA
IGNGSKSPSPFSYATSFPGPITMSAAFDDELIERVATIVSTEARAFSNAGHAGLDFWTPNINPFKDPRWGRGAETPGEDPFRNKGYVRALLRGLEGPYDRGTANSTRHR
AHNGTTHHGANGTHHHHGTHGGGHTHDDPSIRKVIATCKHFAAYDLERWNGTVRYGFDAIVSLQDLSEYYLPPFQECARDSRVGSIMCSYNAVNGTPACASSYLMNDIL
RKHWGWTEDHQYVTSDCNAILDFDHEHKFSVDGAHAAAAAYGAGTDTVCEVPSYEGTDVRGAYNQSLLSEAVLDRALTRLYEGLVRAGYFDPASASPYRALGWSNVATP
EARALARRSAADGFVLKKNDVAGPHALPWANLTGRRVALIGHWADATRQMLGGYSGIPPFYHNPVAAARARNWSYVAYGGLDGAPVNPAPGATDTWTEPALAAARQADA
VLYFGGNDLSIEAEDKDRINITWPQAQLDLLTALAALGKPTAVVQLGGGQNDDTQLLRNPNISAVLWAGYPGQDGGNAALDIITGVHAPAGRLPVTQYPGSYIDAVPMT
NMALRPGPGNPGRTYRWYDHAVLPFGHGLHYTNFTAGFASAAPDRAIAKTQLVSNGTYALADLVSAESGCTATHRDLCHFAYVNFTVANTGSAAAGAATSDFVALAFLA
GSYGPAPYPRKTLVAYRRLRNIAPGQTASGVLSLTLGSLARTDAAGNLVVYPGTYSLLLDEPTQSTTQFTLTGEGVVLDEFPQPGSNITSKA
>tr|K6YCB7|K6YCB7_9ALTE
MINIKYKQILTSTALILPLLASLVVYSDTSHEQTSEKLKPVFAEKITAITVTPENWPKQTSPIKRSPEQEAKIVEILAKMTLEEKVGQVIQGDIASVTPQEAGEYNLGS
VLNGGSSAPNGDIHAPPEEWLQLADEFYQESTDTSDGGVGIPLLWGIDAVHGNNNVVGATLFPHNIGLGAANDPNLMRKIGEITAKEILVVGIDWTFAPTLAVVQNDKW
GRTYESYSENPEIVASFAGPLVEGIQGKVNSDNFLSKNHLLANVKHFLGDGGTKDGKDQGDTLVSEAIMRDIHGAGYPPAIQHGALVVMASFNSWHGRKMHGSREMLND
ILVERLGFDGVVVGDWNGHGQVAGCSNVSCPQAFNAGLDMFMAPDSWKELYKNTLKQVKSGEISLARLDEAVSRILRVKLRAGLFDAGLPSKRPFASKFELLSSSEHKA
VAREAVRKSLVLLKNNNQLLPLQANSKVLVAGGGANNIAQQSGGWTLSWQGTGNNNSHFPNAESIYQGIEKAVQQAGGQVELNENGDFETKPDVAIVVFGEQPYAEFQG
DVTDLDYKPNADLALLTSLKEQGIPTVTVFLSGRGMWVNPELNVSDAFVAAWLPGSEGGGVADLLFKNAQGSVQYDFTGRLSFSWPATPLDVEVNIGDENYQPLFAYGY
GLSVKDNQNLALLSEEVDPSVSQINDSRFLFAGDPVQPWRLVLNDAGGNTQVSASSQASSLSKLLVEAKDNKAQEDAMQATWSGEANLLIQGNPVDLTTKMAAGMALAF
NYNVLEQGASKIILSTGCGLDCGGELDITTSLKSKTGQGWQQATIALSCFAKAGSNMQQVDVPFILASDAGLTIQLSEIKIVAAENIANCDL
>tr|A0A2S7KAH9|A0A2S7KAH9_9PROT
MIGRGEMRRACFCAAAVALFLISACADRRDEAADGKTSGAQAGESQYNVTLWPEIETPDLTNAETEARIDALLASMTLEQKVGQIIQADSSSVTPEEVRQYRLGSVLSG
GNSAPGGKAYATADEWLAMADAFYDASVDPEGVETAIPILLGIDAVHGHNNVIGGTVFPHNIGLGAARDPDLVRRIAEATAKELRVTGHDWTFAPTVAVPQDDRWGRTY
EGFSEDPDIVASYAGVIVEGLQGELDSEAFMGPDKVVSTAKHYLGDGGTEGGRDQGDAQASEADLARIHGAGYPPAIRAGALSVMASFSSWKGEALHAHRYLLTDVLKD
RMGFDGFVVGDWNGHAKVEGCTNEDCPAAVNAGLDMFMAPDSWKGLYENTLNEARAGEISEARLDDAVRRILRAKLRFGVFEQGRPSGRPLSGDETVLGAPEHKALARE
AVRKSLVLLKNNEGVLPLRRDLKVLVAGDGADDISKQSGGWTLTWQGGGLDKALFPNGESVFDGLKAAVESGGGQIALSENGAYDEKPDVAIVVFGEDSYAEFQGDLDN
VAFNDPSGEALAIMRKLKADGVPVISVFLSGRPLWVNPELNASDAFVAAWLPGTAGGGVADVLFRDSDGAVNYDFTGRLSYSWPKNAAQTPLNKGDEGYDPLFALGYGL
TYEDDGSLPALSEDSGLDESDKGTPGVYFAGGEFAPSIKLMTGADVSSLSEDGGAVSVRRVDHQAQEDAIELSFPESGGAAALASGEAIDLSRETVGQLELSFAFRAEM
FGEGAMDIGVLCGEGCAGMIDLGDVLREGEGAGWRRMRVTLACFADAGADMSRLSAPFAIETHGAARIAIADVHLVQDANGVKDCGLVEAN
>tr|Q5V5G3|Q5V5G3_HALMA
MAHDTTDDGMQASRRTFLKATGVATAAAATGAGVGTAAAQPDVDSLVGDLTLEQKAAQMTQVAISSFEAEPEESNVPDSFGVDTVGEYFSELGVGSILSGGAEPPSFDG
ETVVQGINALQEYNLENADHDIPFLYGVDATHGNGLLAGATVFPQRLNMGATRDLSLIEAAERHTSDATASMGAHWTFAPTTDLQRDPRWGRFFEGISEDPKLEADVSR
VRARALEDDDRLTACVKHFAAYSIPNNGNDRAPASTSLRDLRTNILPPYREALKSEPGTVMVNSGSINGVPAHASHWLLTTLLRDTYGYEGMVISDWDDLNRMITNHDY
APDFETATEMAINAGVDMYMIGNGGDAPGPVQFIDTVVSLVEDGAIPMERIDEAVRRILELKADLGLFEQPTVDESRIETVLGGAQETAETMAKESMVLLKNTDDTLPL
SGDESVLLTGPGVDSNGNNTRALMQHGGWTLGWQGASAGGPFPRQNLLEAELRARVGSLTHVPTSYENTTWWAGEGDGGNQQSDENGNFDFTAEQRSRVESAGPESDVV
VVVLGEGTHNEGFGDRDELVLDESQQALLDTVVESTDDSAPIIGVMLAGSPRGSPETFSQLDALLFAGQPGSDGGVAIAETLVGEYNPSGKLPFSWPENVGTTPVQYTR
YDPTSTGGTDNTAIYEYGHGLSYTDFEYGSVSVTQPTVGNPANQSEVTVSVEVTNTGRMAGEHIVEVFNTQSYGSVLQPRRRLLGYERVSLGPGELTTVDVTVDLTALE
VVPGDVLGLGPKVVEAGEYELTVGQDGPTATLTVQNTASITDPDPVPGLPDGSGSTPGRGNNGNNGNGNNTSRDPTMEDVVDLLDEVRGRS
>tr|A0A2T9Z6K8|A0A2T9Z6K8_9FUNG
MMNLGLVSIGFLCILNSSTLGDISGSANQSNNLRYKGQRRSVPKFPLINVDGVKSNNGVVCLPDNYPFQDYDPMELKSVKTYSLKQYDESVPQGPSLIPEVDTVRILKK
LSLQEKVGQMVHVHIGKFLGCDGLLNVTLAESIFEKYKIGGVYGSPVDMGGRWNIASPQRWANLTNTLQKIAMEKGSKLPFIYSLESPKGAGNIKGSILFPAPVNIGAT
FNLMHAYIPSQIVAKDIRAAGAHMIHSPSANLNVNRLWKHNYESFGEDPYLAGEMVYTTVRGLQGNYKVDRNRVAACVKHYIGYSGSKNGDDKEPRNIPYHKITEYHLY
PFLRGFNAGATTAMLSPNTLNGEAVSSSRYLKNDILRNFLRFSGVVVSDWSEIKSMVKFGTAISPNWGIYQSFEDGVIDVSMSSDGFDFIDGIFDMLNRNAFSKDKIYT
SVGRILQLKNDLGLFDNPYSNLGLIETVGSNQDIEKSQIAARESIILLKNENRVLPFHKNDKVVFIGPIFNSTRYMCGGWSVHRKEKSDLEGDSPYYGYGDTLEQAIEK
IIGRKPTIFTGFDIDGNLVDDYTELGRHVHRADKVVIGFGEKSAGEANMLDLTESQISLVEYLAKLTSTPIVFLLMQNTPRIIDGIIKYADGILNVNLPGAYGGLPIAE
AIYGSFSPSGRMPYTYPKSNLQTDLTYYTPVTSEYDPTFAFGTGFGYNEMVYSSIRVSNDVLFPGIPIKVTVSVTNNGNGPQLEPVLLYASQHVRNRMTPERYRLKDFK
KKLINPKETVEYVFHLKAEQFQFRASDSQLYIEGGKITLTINAFTKNVVTKDVLLKIDLDFTDNQQITTGRDNKQSPQGRISNKRPTPHY
>tr|A0A383V2S3|A0A383V2S3_TETOB

MLGNLNEAKAASKPGHAKSGVATVTGIKLAKQQAGPAAAAGAAASAGQTQVAAPLKQQQQQQQQQQQQRPPAGASSLRMRGVGALEASQQQQQEQQQQQQPAWRDQQLP
LNDRLADLVGSLTTEEKLSLMNNYQQPVERLGVGGYQFWTECLHGHQERGAGAVPTIFPQPLLLASSFDDALAWQVFNAISDELRAKDNAEFAKSGNRKGLVCWTPHVN
IFKDPRWGRGSETFGEDPVLTSRMALRVVQGLQGSDPDYKKVVATCKHFLGYGLEGAEGHSRYSHNIDISPQDLTDTDLPPFRACVQGAGALGIMCAYNAVNGTASCAS
APLLQKLLRGNMGFDGYVVSDCNAIQALEWGHEAYPSMRDASAAAVKAGVDLFCDKADQLKPAIEAGLLSSADIDAAVNRTLAVRFATGQFDAPQANPWRELPLSTVNG
RMSRLLARSVVQKGIVLLKNKAPAGADKPLLPLDKASLKKLCVLGPLANSAEHMMGNYYGNFDADIAATPLQGIQEELAGSGVKVEYAQGMDLMGQIHYDWPLETALKA
CNGADAAIVVIGSSMIENEKSADGSLRPAHEGEGLDRNNLDLPGRQADLVRALARRAPGLPVAVVLFNGGGLDVGWIDRMPAVSAMMAAGFPGQEGGRGIADVLFGRVS
PSGRLPITWHHSNYTYRVSPLDLRMRPDAASGHPGRSYRFVSDPSFVLYPFGYGLSYSSFAYSDLALLKNEENGCVNVGTEPAFCFRVTVTNSGSAAGEHSVPLFLSHE
LARGKDGYPLQSLRAFKRTKLLEPGASETLEMLLSQSDFVMPRPSDGVNALHLGPWKFTVGSFEGAAGTGGQVGSMPSASLSAEVTLPY
>tr|A0A1D1VK38|A0A1D1VK38_RAMVA
MDFAMRIFIVGTISRLFCGLPCLECKLSPEEIETRINELVGNMTLAEKLGQLQQLDGDYVGTFQPYMIDMVRNGLLGSTINVRGANQTNTLQRYAVESRLKIPVLFGFD
VIHGYRTLFPIPLGETASWDLDAVEQASAIAAAETRSVGLHWTFAPMIDITRDPRWGRVMEGQGEDVYLGNQMAKARVRGFQGDDYSRPDKVLACAKHFAGYGAAEGGR
DYNTVDMSERRLRETYLPPFKAAIDAGVGSFMTAFNDLSGVPATANEFLLREILRDEWKFDGLVVSDYDAVVELTNHQLSANDSEAAMYALNAGTDMEMISRTYNTHGP
QLVQSGKVSTATIDGAVRNVLRIKFRLGLFDNPFVDETLEDKIVFKKEHRDAARNITARTLVLVKNENNTLPIDKNIGKIAVIGGLAADRIETLDHWAGDAKWENSTTM
LEGIMEKLGVTDTTGRVTYAEGCNAYCRTSAGFDAAVRVAEAADFVVLVVGEPREYSGEGGSKTDLDLPGYQLDLVKRIRATGKPFVAVVVNGRPMTLEELHHSAPAVL
ITWRSGTMGGHAIADVLFGDVNPSGKLPMSFPRNVGQIPLYYDYKNTGRPVDRETNSPDIRDKSKYLDVLNSPLYPFGFGLSYTNFVFGNLRLNATSITAEQNVTVSVD
VRNLGTREGEEVVQLYIRDVAASITRPTRQLKAFRKLRIPVDQSERVDFVLMPQRDFSFLDDQWHWLVEEGQFEIYVGNSSDTNMYVNLTVTTTAHEPVEHLTTPSMDL
STTPSFSSTGQTLGSSATTQPASGTTTILLPTSTSPLTPAQTSTISRQTDGTITNSTATAQTTPSGAAKSDPPALQPMLLLFCLVLFR
>tr|A0A5J5C326|A0A5J5C326_9ASTE
MGSLEDTNWLYKDPKEPIEARVKDLLSRMTLKEKLGSLLSCCDSRPYEKTLSSEWAEIVDAFQTSVLESRLGIPLIFAIDAVHGNNNVYGATIFPHNVGLGATRDPYLA
IRIGAATALEVRACGFQYNFAPCVAVCRDLRWGRCYECYSEDTEIVQKMTSIVTGLQGQPPKGHPKGYPFVAGRNNVVACAKHFVGDGGTENGTNEGNTVSSYDDLEKI
HMAPYLDCISQGVCTVMASYSSWNGSKLHSDHFLLTKVLKENLGFKGFVISDSEALERLSEPRGSNYRNCILSAINAGIDMVMVPCRYELFLEDLTYLVEAGEIPMTRI
DDAVERILRVKFVAGLFEYPLTDRSLLDTVGCKLHRDLARVAVRKSLVLLKNGKDPKQPFLPLDKNAKRILVTGTHADNLGYQCGGWTATWQGTSGRITTGTTILEAIK
TAVGDETEVIYEQDPSPDTFAGQDFSFAIVVVGEFPYAETAGDNSELIIPSNGIEVISSVADRVPTLVMLISGRPLVLESWLLEKTGAIVAAWLPGSEGGGIADAVFGD
YEFQGRLPMTCFNAILKLYYFKTEVIYKQDPSPDTFAGQDFSFAIVVVGEAPYAETAGENSELIIPFNGTEVISSVSDRVPTLVILISGRPLVLETWLLEKIDAIVAAW
LPGSEGGGIADVVLGDYEFQGRLPMTWFKRVEQLPMHAEENSYDPLFALGFGLTTPPLTPSLASLSPKPPAQPRHCRLHSLSSSLRSAYKAEVGLSLLVTSPSSPPQSL
EEYSNCGADGFSGGGFSGWSNGDLATPICGSRSMLPRCRSMTRGEYHKIELELKLKPQTAKRQIRYAEIPYGEDAKRVAVKTLKLLER
>tr|A0A545SY60|A0A545SY60_9GAMM
MSNRPTSLSYTLRGLALVALTLLAAGCEQPHLERSTTDSAVWPVLRPPLDPDPALEKRVDTLLAQMTLEQKVGQIIQAEIGFVTPEDVRRYHLGSVLNGGGTYANGNRY
ASVAEWVALADAFYLASMDTAGGGLAIPMLWGTDAVHGHNKIFGATLFPHNIGLGAAGDPALVRDIARVTARAVRATGLDWTFAPSLSVVRDDRWGRTYEGYAEDPQLV
ERLAGAAVAGFQGALDSPSFLDPTRVLATAKHYLGDGGTENGIDRGDTTVTEAVLRDIHAPGYFAALDAGVQTVMASHSSWQGERMHGHKYLLTDVLKQQLGFDGFVIG
DWNSHGLVPGCSNASCPQAINAGVDMIMVIEDWREFWYNTLAQVKRGDISQARLDDAVRRILRVKFRAGLFDRGKPSTRVLANDAGIVGAAAHRALARRAVRQSLVLLK
NKNGLLPLRPQARVLVAGDGADNIGKQAGGWSISWQGDDNRNADFPGATSIYTGIRAAVTAAGGSVEFNVDGAYRQRPDVAIVVYGENPYAEWQGDLQSPAYQPYSHRD
ARLLEKLQSDGIPVVSIFISGRPLWVNRALNASDAFVAAWLPGTEGAGIADVIFTDAGGEVQHDFTGRLSFSWPRLASQVRLNRGDADYEPLFPYGYGLGVNDEDRLAD
KLPITDDFPIARAAGSTLPLFSGRPAAPWMFGLSDGREQKNLTGASGGIDGGAIAEADKDTQGDARDFIWHGRGRTTISLHAGHERPDLSNFLASQAALQLDIRLDRAP
SEAVFAAVRCGGQCGGDLDITAPLAALPAGQWRRLSIDLQCFAQRGADFSRVNAPFAISTAGALKLRVANIKLTPDAASAADIRCPG
>tr|A0A559QPW8|A0A559QPW8_9ALTE
MASYPCARLIAIITLIVILISCGKPDPLPQHAAPQQPGWPALKPVIGPNAAIEQRIKALLENMPLEQKIGQMMQPSIAHVSPEEVKRYYIGSVLNGGGMFPNGNRYATP
QEWQALADAYYQASMAVPDGVPAIPVIWATDAVHGHNNVVGATLFPHNSALGAANNPELVRLIGAATAQQMVATGLDWNFAPTVAVAKNARWGRTYESFSENPGIVAAL
SAAYVAGLQGYPNDKSFLNTDKVIATAKHFIGDGATTRGDDQGDADLNEQQLIAEHALGYFSTLSMAVQTVMISYSSVQGLPMHGNKHLITDILKNQLHFDGIVVSDWN
ALGHVPGCTRDNCAAAINAGIDVLMVPYKPDWPNMIANTIAQVKSGEISMQRIDDAVSRILRVKLRSGVFNKPKPSQRSEFTYDTYLSTPEHKALARRAVRESLVLLKN
NDGVLPIAPNKNILIAGDAANNVSRQTGGWTISWQGDDTTPKDFPVATTVFQGFESAVQAAGGTVHYSATGEYEVIPDVAVVVFGERPAAEMSGDIENLNTLEFNHEDP
EPLKVLQKLKAAGIPTVAVFIAGRPRLINKELNQSSAFVMAWFLGSEGGGIADLLLANQNSGKSFDFQGKLPFVWPATPCHVLGSQPQFNLGYGLSYAQPVNLSTVPED
YPPRTYGCDLPEKFVPSSAKPIDLSGSDFQLHLELKSLESKIISDSETWHGLRGAVQRKPAGAISQIDLEWQDAPRNNAVLQDGKVRSLLANLAAGHALQFEIKVNQKP
SAKVWAKMECGHLCAADTLEITQKISTLPISHWQQVYMPLACIAKNRSDLFKINSGLRIDSSGYFSMSLRHLKVAALNDDDAINLCE
>tr|A0A369QD44|A0A369QD44_9SPHN
MKRVFGRNVLAGMGTCVSAIGLAGCATMSNPAMDAADAAATTARADAVVTMVTPERARANPGNWPNADSPDALTDTATEARITGIIAGLTLEQKVGQLIQADIGSISPA
DLKTFPLGSLLAGGNSGPYGDERGSAADWDRMVREFRAAGLSYDGGGTPVPLIFGIDAVHGHNNIPNATIFPHNIGLGAARDTDMIRRIGAATAAEVAASGIEWTFAPT
LAVPQDLRWGRAYEGYSSDPAIVAAYSAAMVEGLQGQIESRAMLPANKVAATAKHFLADGGTAGGKDQGDAQMDEAELVRIHNAGYPPAIEAGVLTAMASFSSWNGVKH
HGNKTLLTDVLKDRMGFDGFLVGDWNGHGQVRGCEATDCAQAINAGLDMFMAPDSWKDLYANTLEQAQNGTIPVARLDDAVRRILRVKAKIGLLDNPMRDTAPYATVGK
QEHLDLAREAVAKSLVLLKNNGSVLPIKPGARVMVTGPGANSMAMQSGGWTVSWQGADVTSEDFTNGQTIWQALDAATGAAGGTAVLSEDGSFKQKPDVAIVVYGETPY
AEFQGDVATLDYQPASATDLASLEAIRAKGVPTVSVFLSGRPMFTNPEMNASDAFVAAWLPGTQANGIADVLIARRDGKTPRGFTGRLSFPWPNSCGGRGTPLFPLGTG
YTYANVPQVGTLPDTCSDAVAGAAGEVRLFDRGLDQLATATVIDGGVTSALVNLVGSSGGGNFVAAGFDRTAQEDARRLTWTGPATLQLDFAGVEDTADQALEMLVKLD
ARPEGAVILKSGREGATGIDLAPTLALAAQKGFRTISVPLRCFDTGTQSDSGLDRIMLESSTPLVLELASARIVSQPPAAGCNGPF
>tr|M2N8C7|M2N8C7_BAUPA
MLRTALTQAVLLGLASAQLQSGIYTHYPDCVNGPALLTSNLVCDTNATPYQRASAIINAMNITEKLANLLDVSYGSARLGLPPYEWWSEALHGVAGSPGVNFTSSGNYS
YATSFPMPITFSSAFDDPSVQNIASVISTEARAYSNAARGGLDYFTPNINPFKDPRWGRGSETPGEDPLRIQGYVKNLLIGLEGTDDGYFNTSHSGYKKMIATCKHFAG
YDLEDWDGYIRYGYDAEITTQDLAEYYLPPFQTCARDQNVASIMCSYNSVNSVPACANSYLQETILREHWGWTIDNNYITSDCNAISDIYYNHNYSVNNAAAAGLSLSN
GMDTACIVANTGVMTDVNGSYYGGYVTEATITTALIRQYEALVIAGYFDPASSNPYRSIGWSSVNTPAAQTLARQAATEGTTLLKNTGLLPYKFTSQTKVAMIGMWANG
TSQMQGGYSGPAPYLHSPLYAASQLGLSYNYANGPINQTTLTSNYSQNATAAAQNADVILFFGGIDWSVEAEAMDRYQIAWPGAQQALIAQLAALGKPMIVLQMGSMLD
ATPILSNNNISALVWVGYPGQDGGVAAFDILTGAVAPAGRLPVTMYPADYVNQVPMTNMSLRPGPGNPGRTYKWYNNAVLPFAYGLHYTTFKATFNGGPPGPGSPWSPP
WNAPWSAKVRRGWGWGNWGPPNWGWTQPSQVAPGNGGLSSSYNIQSLLSSCTAAHPDLCAFPSVAISVQNAGQTTSDFVALVFSNTTAGPAPYPYKSLASYTRLHSVAA
GQTVTASLNMTLGVLARRDDQGNQILYPGTYNLLLDVPTQATMSFQLTGQAAVLDQWPQPPANQTYDATINCPLYGQCKSAGQPVA
>tr|F7NRC8|F7NRC8_9GAMM
MFRSKPLALLCSAALITTLSGCGQQNTSTETAAEVKTTGADSLALWPELKSPLANSDTEKAVQQLLATMAIEQKVAQLIQPDIRWMTVKDMRQYGFGSFLNGGGSYPND
NKNSTAADWLALAQAYYDAGVDTSIDGSSIPPIWGTDAVHGHNNVVGATVFPHNIGLGAANNAQLVEAIGRTTAVEVAATGINWIFAPTVAVARDDRWGRTYESYSEDP
TIVKELGAALVKGIQGNVGADFMQSGRLIATAKHYLGDGGTENGKDQGNNLDNEADLVRLHAQGYISSLNAGVQTVMATFNSWHGEKIHGSHYLLTTVLKERMGFDGLV
VGDWNGHGQIPGCTNTNCAAAINAGVDILMAPEDWKLLYQNTLAQAKAGEISAARLDDAVSRILRVKIRAGLFERGNPAQSEFAGKTELIGHADHQALAAQAVRESLVL
LKNNDQLLPLAPKQKVLVAGDGADNIGKQSGGWTLTWQGTGNTNADFPNGRSIYSGIQQQVEAAQGSVELSADGSYQQKPDVAIVVIGENPYAEFDGDISTLDYQAGKN
TDLDLLKKLKADGIPVVTVFLTGRPLWVNPELNQSDAFVAAWLPGTAGQAVADVLFKTQAGEIQYDFKGKLPFSWPKTAGQSPLNQGDSNYDPLFAFGYGLTYQNNTTI
ANDLIEQINSATSASNKSLSLFERRPASGYSLILQDEQGVVTVTGNKAKSADQSLNLAAVNWQKQEDALSLDWAANSKSRLVLQAAVPLNASGYGQLVVDMKWNTAPTA
AVTLALSCGSGCGGSLDLAELMNNKTAGQWHKMVIDLSCFAAKGAALNHLQQPFVLHSEAAYQLSIANIRFTPATTKADLSCPAT
>tr|U9VW20|U9VW20_9CYAN
MPHGELLGLVLAMNINLVSLLIGLVLIGGLIAYLATAVKRISHRSIPPTITPENWPITSGGAPLEPEIETRIDGLMAAMTLEEMVGQTIQADISHVTPEDIRHYRLGAI
LNGGNSAPGDNVRAATEDWLPLADAFYEASMDTSNGDQAIPILWGTDAVHGHNNIISATIFPHNIGLGATRNPALMEQIGAITAREVIVTGMDWTFAPTLAVAQDSRWG
RTYESYSENPDLVAQYAKAIVTGLQGELGTDTFLSRQHILATAKHFIGDGGTQGGKDQGNNIDSEIKLRDRHGAGYPPAIEAGVQTVMASFSAWHGQRLHGHQPLLTDV
LKDRMGFNGFVIGDWNGHAQLPGCSTTSCPAAFNAGIDMFMAPDSWRQLHKNTVAQVQSGDIARSRLEDAVRRILRVKFRLGLFEKPKPSERPLAGDYSLLAHPNHRAV
ARRAVRETLVLLKNQDNLLPLAANQTLLVTGDGANDIGQQSGGWTLSWQGTGNTNAQFPNGTSIWAGIRHAVETSGGQAILSTDGSYSQIPDAAIVVFGEGPYAEFRGD
IDTLNCLPNTPLQQLQELRAAGIPTVAVLLSGRPLWVTPEINAAGAFVAAWLPGSEGAGVADVLIRQPNGDINADFKGKLPFSWPRTATQTRVDQGKNNYEPLFPYGYG
LTYADSGNLPQLPEISGLDGTENTQNEALFALGQPMAPWSLQASVGNESSVVIDERIQLENALTIRSIDRAAQEDAQQFTWSGQGSASVAITGPAQDYMVPLHYDMALE
IQYRIDTVPEGQVKLVIGYGDDAKSDLDITRPLVGCPPGKWLQTHIALNRLAATGADLSQLTILALETAAPFSLSISDIRLVAKD
>tr|A0A2R4X4C0|A0A2R4X4C0_9EURY
MKAAGAATAAATIGAGAETAVAQDLPIPALVNRMSVREKAAQMIQPVIGSLDPTTESSHDNVETVGDIVGEIGAGSVLSGGAMPPTTDPEALVAGINELQEYAIENSPH
GIPFFYGIDGTHGAAYVDGATALPQRHNMGATRDPDLIERAEEHTAAMIAATGIHETYAPTIELQRDPRWGRYFEGISESTKVLGDISRARNRALEGHDRVTATPKHFA
GYEIPTNGNDRSAVNTSMRDLRETLLPPFEVTLAEGAGMVMVNSGSVNGVPAHVSQWLLTDLLRGEYGFEGVILTDWNDLYRLIGIHDLFPDTRAGKKMAVRAAIAAGV

DMAMLGGGNEGRPLDPREFVTYVDELVESGDLTEQRIDASVRRILELKQDLGLFDDPYADESLVTDLVGNDESVATSTEIARESLVLLKNEPVTEGGDPVLPLAGDEDL
LVTGPGVDPETGIENRILMQYGGWTLGWQGIEAGSLSEGGPRPAGDSMIAALRDHHDGAITHVPTDFDRTEWWQGEWHPEEGAPNHSSENGDYGFTDDQRSAVESAAPE
ADAVVVVIGEGPHNEGFGDRDSLELPETQREIIATVEAATDDETPIVAVEYAGSPRGNQESFGPLDAVLYAGQPATGGGTAIAETLLGAYNPSGRLGFSWPQQVGHGPL
HHNAWPGNGHDPTYPYGHGLSYTTFEYANLSVSPATVDDPARETVTLGVDVTNTGDMAGEHVVEVYNTTAYGTVMHRDTRVVGYERVSLDPGETTRAEVEIDLAALEVV
AGDVPGLGPKHVEATDYVLTVDPESDLSTTLTVTEPGSVLEGITPPDGSDTGPGQGRGPPRGRGNGRARGRRSSLIDRLAGRDP
>tr|A0A373NUB2|A0A373NUB2_9FIRM
MTVYDNPVFFRRGCAIVNSEKWSETETTEREILQEMKYQNIIENMTLKEKAAFLSGKSEWQTREFPRLDIPAIFCSDGPNGVRKQAGAGDHLGINPSVPATCFPTAATM
ANSWDEELEQKVGVALGEESMEEEVNVLLGPGLNIKRNPLCGRNFEYFSEDPYLSGKMAAAFIRGVQSTGAAACAKHFAVNSQELRRMAMNAVVDERTLREIYLTGFEI
AIKEGNAKTVMSSYNEVNGVYANENEHLLKDILRDEWGFDGSVITDWGASNDHSLGVKNGSTLEMPTPGLDAARELIASVESGKISEKDIDERVDELLDLVLTTTENAK
HYKKAADKKALHEEHHKLARRACEESAVLLKNEGGILPVGEKVQAAVIGDFAFEPRYQGAGSSMVNSTKVDSIKDMLESSGISVTGIVHGYQRDGKEDEAMKKEAVDLA
RRSDVVLFFFGLNEKSETEGLDRKHLRIPQNQINLIQELAKANANMIGIISAGSVIEMPWHHHFKALLHTALMGQAGAGAVLDILSGKINPSGKLAETYIKKYEDTPSY
NYYPSQERNSEYREGIYVGYRYFDTAGVPVNYPFGYGLSYTTFEYENLQVKPDGVTFTIRNTGKYDGAEIAQLYVGLPGAKVFRPVKELKGFTKVFLKTGEEKTVTIKF
DDKTFRYWNMKTNKWEVEGGVYSIMIGASCADIRLQNALAIIGTTEIMPYYTNRVASYMDGKIQKVGNEEFETILGHSIPDGSWSGELGMNDALCQMYYAKSGIARFIC
KKLGEKLKKSEAAGVPDLNTLFQYNMPFRAIAKMTGGMVSMEMVEGLVRVVNGHFSGLGKVISGFFRNQKLNKQYEKDLEEQK
>tr|A0A316W4J3|A0A316W4J3_9BASI
MRLLFGAAQAVAVVLGGSVLSSAPSQAVPFDDTDSDLLRPASRGGMDLLGFDAVEQTPELISRRTAQLNARQLSGDNKAFPPPPTKGGIWRKGYAHAKSLVDQMTLEEV
VNVTTGYSGTCVGFSGEVPRLGLKAACLQDGPIGPRPVRRVSQFPAAITTSATWDRSLFYARGEAMGRQFKAKGVDMALSPVTGGPIGRSPLSGRIFEGFGPDVYLHGA
ASYETVVGLQKNGLMACSKHWIAYEQETFRNQYNNTEPYSVFPKNEQSPIDSVLDDRTTHQLYMWPFAEAVRAGSAAVMCSYNEVNGSHACGNAQILNGLLKTELGFAG
PVVSDWGGTWDNINSWPNGLDVTMPGSAYDGQFGYFGGSGLIDAVKNGTIPMDRVKDAAVRLLTPWFDHQGNNLTWPEVSFDVRDLTVPTKGVRKPSDADLIRRIGEES
ITLLKNVKSTNDSRGLPLKDPNELSSIAILGQDSSANPSGWTACLPDGGKCPTPYNNGTLPAGGGSGWANPSYVIDLYASMQHHARSAAYDVNHDNSRGAADLTNFRNQ
ANVSEVAVVSVSAWTSEGYDRANLTLSGEGEELIKAAAEMNNNTIVVIHAPGPLLVEDWIDHPNVTAVLFAYYPGEAGGSSIPPILFGDKSPSGKLPFTIGKALEDYLP
KSIVDDHVVDPVSNFTEGILGGDYAWFEHANHTPRYAFGHGLSYANFSFSDLDVQAKHAADNTSIAPTNERWSSELGAQSESLYDQLLLLTVDITNTAPKSHHGYSGPA
AEVAQLYVSWPEESSDNPRALRGFEKVWLGAGESKTASFSLRRKDLSTWNTEKQEWELSRGKYTFNVGSSSSDLRSTASYSF
>tr|A0A2N5Y2F0|A0A2N5Y2F0_9GAMM
MPWRTRVPSAFCFSWMDSIMKVLKALVAPVGAKYTWTLLKISPWLAGCALLSGMAFAEPQSPESRIDKLLSQMSLEQKIGQMMQGEIKTITPKEVSRFGIGSVLNGGGS
FPSKNKGASIDDWIDLAEAYYQSSPTLADGTRIPAIWGTDAVHGHNNVMGATLFPHNIGLGAAGSSDLVEAISRATAIEVKATGIDWVFAPTVAVAKDFRWGRTYESYS
SDPQLVSRLGVAAVKGFEAEGIVATAKHFIGDGATQRGRDQGDVRSDLETLLEGHGAGYVSTIKAGVPTIMASFNSWNGRKVHGSRQLLTEVLRDQLGFGGMVVSDWNG
VGQVAGCNNASCAQAVNAGIDMLMTPTDWRLLQRRVVSQVESGEIPMARIDEAVRRVLAVKANYGILDQGSPRDRVTQELKAKVGSEEHRALARKAVRESLVLLKNNGG
ILPIKGNAKVVIAGTGADDLSMQSGGWTLTWQGTENSNSDFPGATSIYTGFNDALAAMGGEAILAADTSQDADVALVVFGETPYAEGQGDIENLQYKPDDHVDLELMRA
FKAKDIPVVAVFITGRPLWMNRELNVADAFVVAWLPGSEGAGLADVLVGNVQGLPRHDFVGRLPFAWPALDVNEIDDSLPVDRFVWPVGYGLTYAEPATVGSLDEKAAG
SILSADAVIFRGGTQSPWANYIGDESNWMTPVSGSYGKSAFGEFEVKSVDVRLQEDARQLTWSGAGERDTQFYWMTANGRGRDFSELREKNGALTMVVMMTEHPEGTTY
LRMDCQWPCSGQVDVTRLFKVLPLNQWVRLSFPLACFAEAGTEMDRVNTPFLLVTNHRMSMTVLDVAVVETPDQDSLVKCR
>tr|A0A6S6FXH3|A0A6S6FXH3_9SPHN
MRKATLVRRLPLLALCGILTVGSAAPPSPNAIAPSAAQTAPSDLDARAEAILAGMSLDQKIAQVIMPDTASFSAADMAQYRFGSYLSGGNSGPGGDERAPPARWLALAD
ANWDAAMAARADGRTPIPTLWAVDALHGHNNIVGATLFPHNIGLGATRDPELVRAIGAATAAEIAVTGIDWTFAPTLAVVRDPRWGRTYESYGERPDVVASMAGAMVEG
LQGTPGTPGFLGPDKVIATAKHFLGDGGTGGKDQGNTVASEADLRRIHAAGYIPAIAADVQTVMASFSSVNGVKQHGDKAHLTDFLRGPVGPKGLGFNGLVVGDWNGHG
QVPGCSVESCAASFNAGLDIFMVPEAWKALLASTTAQVKSSEISMARLDEAVRRILRVKLAAGVFEKGRPSSRPLAGKVELLASAEHRTLARRAVRESLVLLKNNGNLL
PLKPAQRVLVAGDGADNIAMQAGGWTISWQGGGDLTRSDFPRAQSIYDGIAETVRAAGGTAMLSSDGSFTAKPDVAIVVFGEKPYAEFMGDVDTLEFHDDYKSYKTMQR
LRAAGVPVVAVFLSGRPMWVNPEINAADAFVAAFLPGGEGGGIADMLFAKPDGGVAHDFRGTLSFTWPNGPMPPKLDTPRDPGTLFPFGYGLRFADHTLVGPLRETAPA
GQIISSRIAVLGRTGLPAGWNMSLADAGAPPVPISSNAGQSPSGALVLRGLDRDRQEDARLAIWRGGTMARVAVSAPRLLDMSGPGQALVIDLRVVAKPTGKVTIGVKP
VAAGGKASDALLVDATALLSGGVGTWRTVSIPLRCLGSRTALRAVDTPFVIETAGRLSLAMSDIRIAPTTGRTVQCPRKAR
>tr|Q6C1I2|Q6C1I2_YARLI
MIFSLQLLLTTALAASSPDWYPTPEIGSITSDWADALGESMDILAQLTLPEKVNITTGTGWRGGQCVGNTGAVPRLGIKGLCLQDGPLGVRFADFVNVFPCQNAMAATF
DRILVHQRGTAIGRQSRLKGVDVHLGPVVGPLGRHATGGRNWEGFSPDPYLSGKLAFEAILGVQEEGVLATIKHFIGNEQEHYRRAEEWRDGFGFKDLKDAVSSNIEDR
ALHELYMWPFADAVRANVGSVMCSYNYVNGTQACQNSDLLNGKLKSELGFQGFVMSDWFAQGSGVSNALAGMDMSMPGNDVDELETVFWGEQLTRMVANGTLPEARLDD
MVLRILTPLIYFGIDDRTPNFSSFVDTTVGSPHPAAKHSKKVKDVITNYHLDVRDQFAANVALDSARGAVVLLFNDGILPLKNISAIGVFGVGSRLGPNGAVCGENMQC
SDGALIEGWGSGTAYPTEYESPYEALHKKASLLEVSVTGTTESWDMRLPLELAGDTDVNIVYVLANSGESTANVDKNLGDRRNVSLWHNGDELINTVASQGQTVVVVTT
VGQVDMTAWLNHPNISAVLLTAPAGDYGGKAIADVLFGEVNPSGKLPYTIAANTSDYIPIVTKIPRDGAPQSDFVEGIYLDYKWYDKFERTPLYEFGYGLSYTTYSFSN
LHLDVKEISEFLPPRPVPVQVTKPKMTNIDIEDLYVPNDFKMIDGLVYPWILNASAPLADSQTQFPFANGAGHVSDASGGVGGHPWLWSNAVTVTHNTTNCGDVAGRVV
SQLYVAFPETLIDSPPVQLRGFDKSKLLNPGESQMTEYNLNWRDLAIWDVELQSWRVQRGEYSVYIGHSSREFELCETFTL
>tr|A0A1X7EQQ9|A0A1X7EQQ9_CELCE
MTDVVPAVPTAPTGAPEARAPRVVPPQQPTVSERVRALHARMTLEEKLAQLVGYWLDQNGTVAPMQSEMTAGQQDGGRLAEITRHGMGHYTRVYGTRPVDPAERAAWLW
AEQRRLQRETRLGIPAIVHEECLTGLAAWQAATYPTPLAWGASFDPELVEEMAREVGESMRALGVHQGLAPVLDVVRDPRWGRVDECVGEDPYLVGTVGTAYVRGLQDA
GVHATLKHFVAYSGSHAGRNHAPVSAGPREVADVYLPPFEMAVRDGGARSVMASYVDIDGIPLHASEEYLTEVLRERWGFDGVVVADYFGVAFLEVMQRVAADRGEAAA
QALQAGLDVELPTGDAYLEPLAERVRSGALDEAWVDRAVLRLLGQKEELGLLDPDAYADEPPARIELDTPRQRATARRLAQESLVLLANDGVLPLVRPRADGAAGAPRR
IAVVGPNADSSEALMGCYSFVNHVLAHHPGTPAGIELPTVLESLRDALAGAGTEVTHAVGCAVDGDDVSGIPAAVDAARGADVAVVVVGDEAGLFGRGTVGEGNDVESL
ELPGVQRRLVEEVVATGTPVVLVLLTGRPYAIGWAIEDGAAPARPAAVLQGFFPGEEGGRAVADVLLGAVNPSGRLPVTLPRAAGAQPYSYLQPVLGGPSDVTSADPTP
VRPFGFGLSYTRFAYADLEVDARVAAGGTFAAAVTVTNTGDVAGTDVVQLYARDVAGSVPRPVAQLLGYARVELAPGESRRVTFRVPTTRLAFTDRRLVRVVEPGDVQV
WVGAHAGAVDAAPATDDLGSTTGGAIVNESAPAQRRVVPGAATPRATLAVTGPVHEVGAADERIVAVEVSALADAGERA
>tr|A0A0A0B4Q4|A0A0A0B4Q4_9CELL
MPRSALLEGPADRPAADPARTSPRRRRGAALLLAVALTASGLGVAQTASTPTTAPAQAAVLPYLDPALPVATRVDDLLGRMTLDDKIGQMTQAERAVVTPADMTTYRLG
SVLSGGGSAPSPNTTAGWADLYDRLQRGALATPLQIPMLYGIDAVHGNNNALGATLFPHNIGLGATRDPQLAQDVARAVAEEVSATGVDWTFAPCLCVVRDDRWGRTYE
SFGEDPALVSSMATTVTGFQGERLDGPTSVLATAKHFIGDGGTVGGDDQGDARITEAELRAVHLPPFEAAIDRGVGSVMVSYSSWNGVKMHGNRYLLTDVLKGELGFDG
FVVSDWAAIDQLDGARGFTAQEVATSINAGIDMVMVPNDYRTFLTHLRQNVQSGAIPVARVDDAVRRILTKKVELGLFERPLADRSSASTVGSAAHRALARTAVQRSQV
VLKNTGVLPLARTGGKVCVAGKNAHDIGNQSGGWTLSWQGASGATVPGTTILQGVQEVLAGGRTTTHARDASNVDASCSVAVAVLGETPYAEGEGDRPGGLSLDAADTA
VLERIRSTGVPTVVVLVSGRPLDVSVLLPWVQALDVAWLPGSEGAGVADVLFGKVAPTGTLPVSWPASATQQPVNAGDGRTPLFPLGAGLTWTPGDGGVVVQPPSAPTG
LTAGPTTTSTVPLTWSAPAGSSAVTGYEVHRGGLRVATTASMRFTDAGLTAGTAYTYTVRAVDAAGNVSPPSAAVTATTQPATPGPASGCTATWRTENSWGSGFTASVT
VTATGTTALRSWRVLWTWPDGVQLAGGWNAAFSRTGATQTAASLAYNGALAPAASTSFGLQGTTTSGVPAPVLTCVAG
>tr|A0A0F4GVV8|A0A0F4GVV8_9PEZI
MEKPAAVTFATEHTPFLAGPNPSRAARFCRQHKRVVIAAAIMLVLLPLLGLLALLPRHGKSHFNSPVVLPSPQGYGAGNWSQAYDKARTMVGKMTLEEMNNLTVGQSTA
HTGCVGLSGSAPRVGFPGMCLHDAGNGVRDTDGVNAYASAVHIGASWNSTLAYQRARFMGAEFKRKGVNVALGPVVGPIGRIAQGGRNWEGFGADAYLDGILGAQSVKG
LQESVIASIKHWIANEQETNRNPIEDDDGTILSSSSNLDDRTMHELYMWPFQDALYAGVGSVMCSYNRINGTYACENSKAMNSLLKGELNFQGFVVSDWVAQHSGIESA
NGGLDMAMPLSSYWDDDQLAKAVQGNELNETRLTDMATRIVATWYQFGQDDPDFPTTGVGMPPDLLQTHEYIDAKDPASKSSLLQQAIEGHVLVKNKDNALPLGKLRLM
SIFGYDAVEQTSFNPGQSIFAQNWENIGLQQPQSQEIGSNEPLTNAPQTALGTLIVGGGSGSNSPAYISAPYDALQNRAYQDDTSIYYDFHSSAPGVVAGSEACLVFIN
EYASEAWDRPGLADGESDELVKNVAAQCNNTVVVIHNAGVRLVDAWIDNDNVTAVIFGHLPGQDSGRSVVQILYGDVSPSGRLPYTVAKSPSDYGKLQGPCKDDSQSPQ
CDFDEGVNIDYRGFLARGVTPRFEFGYGLTYTTFEYSGLNIDMNATSTAGAPSTAPVYENGTTIQNVDRNDISAGGLHSLFENVGFVSAVVKNTGSVAAAEVAQLYIQI
PVSKADQGNNANTRTLRGFQKVYIEPGESAKVNFSLRRKDISYWNTRNQTWTTPSGDFEVYVGKSVLDLPLNGSFSLY
>tr|A0A0J8GN22|A0A0J8GN22_9ALTE
MKLPSNKILIALCMSGALASCSQSNQANQTVNPIDIWPSVKSAVTKDAAVEQKVASLLAKMTLEQKVAQMIQPELRFVSVEDMRKYGFGSILNGGGSFPDGNKQANVKD
WVDLADSFYLAALDDSQDGIAIPSIWGTDGVHGHNNVYGATLFPHNIGLGAANNPELIKKVAEVIAKEVAETGIGWNFSPTVAVARDDRWGRAYESFSEDPAIVKSYAG
KYVEGMQGEAGKNFLASDKVVSTAKHFLGDGGTVEGVDQGNNIDSEAELFRLHAQGYVTAIEAGVQTVMASFNTWHGKKMHGNKYLLTDVLKEKMGFDGLVVGDWNGHG
QVKGCSNDKCAQAINAGVDILMVPEDWKALYFNTIAQVQSGEIALSRIDDAVTRILRVKYRMGAFDAKPSDRLHVPNKGAIGSISHKEIAQQAVRESLVLLKNNHNILP
LKPNLNVLVAGDGADNIGKQNGGWTLTWQGTANVNSDFPNATSIYHGIKQTLEANGGQANLSLDASYTEKPDVAIVVFGENPYAEMQGDIKDGNLEYQAGVKTDLKLLR
KLKSEGIPVVSVFLTGRPLWVNKELNASDAFVAAWLPGTEGGSIADVLFSELNGQIKHDFKGKLSFSWPAKPDQTILNVNDQNYKPLFAYGYGLDYNSNQMIPDLNEDL

TIKSAAVADELTLFNQAPTLGFDFNLYSQNTKVAVNSNTVSLDAANSVTYKSFNRFVQEDAFELTWSGSQSAGIQLVNPAGFDFSEYAKANSSVSFDLRLQTPVSGQVV
FNLATTQGQAFSVDLAKYINSSALGEWQTLNINLSCLNENSETFKQVSNIWSIESDQPNKLSVSKIRIVSNQASQDCP
>tr|A0A0K6GG86|A0A0K6GG86_9AGAM
MRSSLFAASLALYFDASLALSGGRRQATNSTWSGPLADGGSAWKDAFARAKEVVSQMTLEEKVSITTGVGSGSNLCAGNTGAVPRLNIPAFCLQDGPAGVRPADFASQF
PAQVTVAATWDRELIYERAAAIADEFKGKGVHVALAPVTGGPLGRSPLGGRNWEGFSADPYLSSIGSYITVKGIQDRGVVATSKHYSLYEQETYRNQRLFPPRYNGTYH
PLPISSDVDDATFHETYLLSFVEAVRAGTGSIMCSYNRVNGSHACEDDYTLNQILKGELDYQGYVMSDWYAQWTNVGAALGGMDMTMPGTGFWGTDLVALVNNGTVPVN
RLDDMVHRILMPYYALGQDKGFPSVQYNTSGIGSQTYLALNPGNTNVNVQGDHYKLIRKIGEESTTLLKNVRSNGGGIPLKKSKFVAVFGQDAGPNPDGLLYCGSDNRC
TAGHTNNGTNSVGGGSGAAYAPYIITPLEGIQSRTAANETQVNWMLNDWDLETARTNALIADTSIVFTYAYQTESLDRDNLTAWSNGDALIKTVAAECNNTIVVIHSGQ
QILMDDWVEHPNITAVIFAYYPGQETGNAIASILYGEVNPSGKLPFTIAKSASDYPPNGIFTENVTDPHIVFEEGNLVDYRWFDAKNITPRFEFGFGLSLTTFDYDNVK
IQSTPGKPTDGIQLTKEPFDGNGTLYDVAYTVTASIKNSGSINGCEVAQLYLSYPASQTTQPVRSLRGFDKQCLNKGETKTVTFKFDAKNVTPRFELGLGMSYRTFNYS
NIELQSTCGKPTDGVQLTKKPFDGNATLYDIAYTITTSITNTGSVPGYEVVQLYLSYPAQTNQPLRSLRGFDKLYLK
>tr|A0A1Y2DWN1|A0A1Y2DWN1_9FUNG
MKFFNLIVFSLLFYNLILASPIKKKCYAKNKKNAAENSENSEVSEKMNQYEIDHINTLDSYLSECTILLRKNGDFPIGKSTKQVYLYGNGVRKTIKGGTGSGDVYSRSF
DTIEQAFTKAGFEILSKDYLDEYDKIYTKAKDEFRKKVQSEVTDPMSLMGVIMPEPDYDIPYAKEGDLAIYVLARISGEGSDRQAVKGDVYLTETERKMILDLAKGFKK
FMLVLNTGGAVDLSGIDEVENILLLSQLGVNTSKTLVDIITGDKYPSGKLTTTWTKYEDYPSNDNFGDNDDTDYKEGIYVGYRYFDTENIDVMYPFGFGLGYTDFEFDV
KSAKLSGEKFEVKASVTNVGKYKGKEVLEVYLSKPGTTIDEPYQILVGFAKSKELSPKKKDDLKINFKLSDFASYDAKTATYFLDEGEYVVRIGNSSRNTVPCGVIKVD
KRINVKKVENKMGKPEFEDYVPEGKRKQEDLSGVKVFKLKASSIKEKVVKYNDKYEISEGVKALSNEEKAKLVIGAFQDGPASVIGSASITAAGAAGETAKVADLKPVI
MADGPAGLRLAKDYFIDEYGAHSTSGSMAESMVEFVPDEIKAMLISKPEIPEGTEILHQYTTAIPIGTAIAQSWNRKFAELCGDIVGSEMELFKVQLWLAPALNIHRSI
LCGRNFEYYSEDPLISGAFAASITKGVQKHNNTFVTIKHYAANNQETNRYLNSSNVSERALREIYLKGFEIAVRESQPKAVMTSYNLINGVHTSESKELTNDILRNEFG
FEGIVMTDWVVGLGGNHKYSGPTPYNVIKATGDIYMPGSKADYEAVLKALKEGTLSIEELEMSASRIYNLSKEIFN
>tr|A0A2A2W855|A0A2A2W855_9BACT
MRHSSLFLACFLVASPLGVSRGQDSVGLAQDSVVHPERWPAARSPIGLDDAIETRIGELIRKMTLRQKVGQVIQADIGSIQPDDLKTYPLGSILNGGNSAPGGNNRATA
KDWLLLADAFYNASRECDLQGGPFIPLLWGTDAVHGHNNIVGATYFPHNIGLGATRNPALLRKIGEITAREIRVTGQEWTFAPTIAVVRDDRWGRTYEGYAEHPEVTAS
YTGELIEGIQGVAGDPSFLQGEHVIATAKHFVGDGGTTGGKDQGNNQMSEEDLRDLQAVGYPVAINHGVQCVMASFSEWQGKRLHGHKGLLTDVLKGRMGFDGFIVGDW
NAHAQVPGCTATDALPVLDAGLDMYMAADSWRGLFETMLRQAESGDVDMARLDDAVRRILRVKLRAGLFEAGSPSSRPLAGRFDELGKPEHRAVARQAVRESLVLLKNN
GGVLPLRPNATIVIAGDGADNIGKQSGGWTLSWQGTGNSNEDFPGGTSIYEGIRRQVRAAGGRAILVGDGKPVADSSAMKPDAAIIVFGEDPYAEFQGDVETLAYKPGD
DKDYRLLKSYSDRGVPTVAVFLTGRPLWVNREINASDAFVAAWLPGSEGIGVADVLLTDRRGQVQHDFKGKLSYSWPRTPMQTPLNVGQAEYDPLFPYGYGLTYSDSTT
LPRLSEQASGGETGLNLTEYFDRGRAIAPWRIVLTSEAERMVLTETRGGTANDAIRVVSADESAQEDIRTITWTGKGQASFALAAENPVDLSVPEFASASIKIRLRVDT
PPSSGVTFALATSDGSKSISLGERISGQQPGVWQEVSIPVANLATGDALKSVTAAMVIQTSGTFKMSLAKVQLDLL
>tr|A0A5C6EBX2|A0A5C6EBX2_9BACT
MCDEADVDSGPFTVGPSRWARHDGVAWGTDDLTCEKIARECIVRKMKLLIVFACLLSLADWTKADGIYHENWIDRNKNGEMDPYENPELPIDERVADLLDRMTMDEKTA
QMGTIYGHKRVLRTTHPTADWKNRVWKDGIANIDEHCNGVREVMDMTGHREHADLLNLIQRWFIEETRLGIPVDFTNEGIRGLCHTRASNFPSQLGIGATWDRDLVRHI
GEITGKEAKALGYSNIYSPILDVVRDPRWGRTIECYGESPFLVGELGTQQTLGLQSQGVGSTVKHFAAYSTPHGGRDGRARTDPQIPFRDMQEILLHPFQKVFALAHPK
GAMSSYNTYDGIPVTGSRYFLTELLRGDYGFKGYVVSDSGAVSRLHEQHEVAVDFEDAIAQAVNAGMNVRTTFKRAEDFILPLRKVVKDGRISEDTINSRVGDVLRVKF
ELGLFDKPFVDPDAAPSIVHCKAHEEMTLRAAREAMVLLKNEDVLPVDPKTCGTIFVTGPAADDVIPMISRYGPGTSDVITPLAGIKAFLGEQASVIYSQGVDYYDATF
PGSGILPQPPNEEEQAKLDKAIEAAKSADIIIAVMGDNDDTVGESRSRTDLNLPGHQDLLVQEMVKTGKPVIVVLMIGRAASINWIDRNADGILVSWHGGEKVGQAVAE
TIFGANNPGGKLPITFPKTVGQIPLAVPHRNGAWAKQEARHDPNGWGTTRVLGPLYYFGYGLSYTSFEYENLKISPQELDADGEIHVTCDVTNTGDYDGDAVVQLYVKD
VVASVAPFEQLLRGFERVSIKAGETKEVSFKLLPKRDFRMLNRDNQWVVEPGQFDIMVSDCSGVEGVKLKGSVTIK
>tr|G4U891|G4U891_NEUT9
MKPPTHSLLPLLASVLTANAFQFPDCVNGPLAKSKACDVTGTAPERAASLVDQLTIDEKLVNLVDQALGASRIGLPKYAWWSEGLHGVAGSPGVTFNTTGYPFSYATSF
ANAINLGASFDDDLVYEVGTAISTEARAFANFGFGGLDYWTPNVNPYKDPRWGRGAETPGEDPLHIKGYVKAMLAGLEGNETVRKVIATCKHYAAYDLERWHGLTRYEF
EAIVTLQDLSEYYLPPFQQCARDSKVGSIMCSYNALTIRDMAGGSKPDEIINLTTAQPACANTYLMTILRDHWNWTEHNNYITSDCNAILDFLPDNHNFSQTPAEAAAA
AYKAGTDTVCEVSGSPLTDVVGAYNQSLLPEAVIDTALRRLYEGLIRAGYLDHGRSAVAGGDGGSFSSPAYDALNWNDVNTPSTQELALRSATEGIVLLKNSGSLLPLD
FSGKKVALIGHWANATGTMRGPYSGIPPFYHNPLYAAQQLNLSLSYANGPVVNASDPDTWTAPALAAAEGADVVLYFGGTDTTVASEDLDRESIAWPEAQMKLLSELAG
LGKPVVVIQLGDQVDDSSLLNNGNVSSILWVGYPGQSGGTAVFDVLTGKKAPAGRLPVTQYPEGYVDEVPLTEMALRPFNHSSSNLEEEVSVQGGASLTIQARSTPGNK
TLSSPGRTYKWYSTPVLPFGYGLHYTTFNVSLSLSSNASSPSFSIPSLLTPCTATHLDLCPFSPSANSALSVSITNTGTHTSDYVALLFLSGEFGPEPYPLKTLVSYKR
VKDIKPGETVTVKDVPVSLGAISRVDGDGNTVLYPGTYRFVVDVDGSQGVERGKEVVVELVGDEAVLDEFPQPPKE
>tr|A0A1U8N6R3|A0A1U8N6R3_GOSHI
MMRLKIWYLTASLIGLCTTSAVPARADAPAAAEQGARVRPEIWPQPRWPYARDAAMEQRIDGLLERMTLEEKIGQLVQADIASVTPDDVRRYRLGSVLNGGNSAPGGNE
FAPPAEWLALADAFYDASMDRRNGGVGIPILWGTDAVHGHSNIVGATLFPHNVGLGAMRDPALMRRIAEATAVEIRVTGQEWTFAPTITVPQDLRWGRAYEGYSSDPEL
VASYVGEFIRGLQGEPGGDPILTGPHVIASTKHFLADGGTTDGRDQGDARMSEEELRDIHGRPYVEAIEAGVETVMVSFSSWNGVKMTGHRGLLTDVLKDRMGFGGFLV
SDWNAHGQVEGCTNARCPQAANAGLDMYMAPDSWRQLHASLLEDARAGTIPAGRVDDAVRNILRVKMRAGLFEAGRPSTRPLSGEVERLGSADHRAIAREAVRKSLVLL
KNEGSVLPIRPGTRILVAGDAADDVARQSGGWTLTWQGTGIPPGMFPGATSIWSGIEQAATAGGGTAVLSPDGRFSGARPDVAIVVFGETPYAEFQGDIPNLLLRPELT
GPLDTIRRLKAQGIPVVAVMLTGRPLWTNEWLNAADGFVVAWLPGSEGGGIADLLLAGRDGRAALDFTGRLPFAWPATANAKGPTLYPLGHGLSYTSGSPFTRLPEDSM
VADAVASGVFMSRGLPATGWSLETVDAGGTTRVTAVPLSTAGGQITVTATDHGVQEGARRFAVVGSGPSTIRLSTQAPVDLTRETNGDLQLVVAIRVDEPPTAPAHLTM
RSGEARAVVPLGRLEQLPTGHWTTFGIPLKCFEAAGGDMEAVDVPFALDVQGAITVSLADVRLQSSAETSLACGT
>tr|A0A316YAZ4|A0A316YAZ4_9BASI
MKALLLLLLLLWQAAASSDLAEAFRKAGHSLATLGDAHRIPSGGSRDTLGYETRDARPSRRRHFNSRHRLHNRQTNATATSATLANGSGSWHAAFVRAKSLVGQMTLDE
KANITTGTGDTVCVGGTGAVPRLGLPALCLQDGPTGVRSSLNVSQFPAEVSAAATWDVELIAERGRAIGQEFLDRGAAFMFGPVTGGPLGRSPLGGRNWEGFGSDEYLS
GQASYASVRASQETGIVACSKHWLAYEQETSRNIHLIFGNLQQPISSDVDDLATHQLYAWSFAEAVRAGTGMVMCSYNRVNGSHACESDRLLNGLLKHELNFQGSVVSD
YGAAYSGVDSVNGGLDLLMPGAGLFGVFPNYFGSHGSKLADAVRDGKVAEARLDDMVIRILTPVLHYQLHDTGNVDEIKQPNFDANTFPSDAPDVQRDHYKVIRRLGTE
SVTLLKNSNNTLPLQVNRISKLAVIGQDAVASSDDQASCNALGSCLVSDQGTRTNGEGSGFTTPPYVIDPLSAIRAYTLEHNPRIKITSSPQTLFSTDPAKAAEDADVA
LVFVSALGQEGQDRSDLKLDRNGDELIKRVAKVNNNTVVVVHAPGPVLMEEWIDSVAAVLFAYYPGQESGASLTPVLFGDESPSGKLPFVIGKSLDDWPPNTIVNRPFD
YTPSANFSEGLLTDWKWFDAHGIEPRFAFGHGLSYSAFHYSPVAQAARVFEADDGSLQPTKETFAKSGPFTEGSSLYDVVLHVSARLTNTGGRTAKEVAQLYVALPDSA
GAEQPRHLLRGFRKVQLDAGQTKTVTFKLRRKDISVWNVVQQQWAVPTGRFRFFVQGSSGQWDAGQGAKGSFTV
>tr|W5IHG2|W5IHG2_SCAIO
MKIEDVNSVKKTRAAGSEPYWDPSLSVDDRVNDLISRMSLPEKIGQMMQFDARSDLENAIENKHAGSILHTSPEDLPRANALVQKTRLRIPLLVGDDLIHGYSFYPGAT
IFPEQLGMACSWDPEKIKAAARVTAREAVTTGVQWTFSPVLCIARDTRWGRVGETFGEDPYLIGEMAAAMVKGYQGDSALEGQLPRDAILATAKHFAGYSETQGGRDAS
EADLSHRKLASWFLPPFERVAREGVATFMLGYESIDGIPVTLNRWLLHDVLRGQWGYKGTLITDWANVSRLVWEQHVQPDPVSAAVAAVRAGNDLIMTSPEFYQAAYQA
IDQGLLAESELDQPVKRILALKFRLGLFEDPRLPEPAAAKKLIGCPEHGQVNLDLTRESVVLLRNNGLLPLLHNSPAADADSASAEAGPASANAGLAPANAGLAPTDAE
PSGGPAPRRIALIGPLIDDAQTQLGDWAGSSGQVTWMPEGHPRETITTIQDGLQKLVPQGWTVTSSRGADILHLMPDPEGEFYPDGQPRPLVGVPAQTDSATLEEALKN
ARESDCIVAVVGDLIQLMGEGCSTATLELQGAQNHLLDALKETGKPLVVVLMSSKPQVLPASALTADALLWAPSPGMKGGQAIAEILLGLTNPSGRLPITFPRHAGQLP
VYYNQIRGQHGTRYADLTEDPAFAFGEGLGYTTFVYGDVKLSGSGEEAKSVETENADSAWAPVSDRTSSGDKSNRLPLYHRGDSIEASIDLTNTGHRPGTEIVQLYIGD
LFTSVSWTDRELKAFRRVTLQPGEKQTVTFTIQVNQCTIVNADAQRIVEDGTFEVLIGHSSRRKDLKVARFIVS
>tr|A0A2G3DFA6|A0A2G3DFA6_CAPCH
MVMWRTGTSGSQVWKKILSARDLMEHLILRKMRSGNADSLLDNWTSQGELYSLIEGNSEFSDQYKQVKQLVKDGGWDEQTLIRLFKPNIVEHIMDKVRPPTSVGNDVPV
WTLEAQERFTILEELDRIKCKMRVIIVQWEMPEQGWVKYNIDGAFRETEKKSSYAYCLRDHTGELIYAHANTHTHADSLQAEANAILQAAKHYRNTQFDKIIFQMDSLL
MQKSLTGRWQSPWSISKAEYMKYKDPKQKLGVRIKDLMKRMTLEEKIGQMTQIERKVASAEVMKKYFIGSLLSGGGSVPKPNATAEDWVNMVNEFQKAALSSRLGIPMI
YGIDAVHGHNNVYKATIFPHNVGLGVTRQRIGAATALEVRATGIPYAFSPCIAICRNPRWGRCYESYSEDLNIVRSMTEIIPGLQGDLPANMSKGTPFVGGKTKVAACA
KHFVADGGTVNGIDENNTVIDSKGLFSIHMPAYYDSIIKGVATVMASYSSLNGVRMHANTHLLTGFLKKRLNFRGFVISDWEGINRITSPPNANYTYSVEASVMAGVDM
IMVPENYTDFIGNLTYLVNKNAIPMSRIDDAVKRILRVKFVMGLFEEPLADLSLAQHLGSQEHRELAREAVRKSLVLLKNGKKPEQPLLPLPKKARKILVAGAHGDNLG
YQCGGWTITWQGVPRNNLTIGTTILKAIKNTVDPTTQVVYNEEPDTNFVKSNNFDYAIVVVGEPTYAEVYGDSSNLTILDPGPSNIQNICGSVKCVVVVVISGRPVVIEP
YVEKMDALVAAWLPGTEGQGVADVLFGDYGFTGKLARNWFKRVDQLPMNVGDPHYDPLFPIGFGLTTQAVKGN
>tr|A0A1V4IRS6|A0A1V4IRS6_9CLOT

MNGRFVIKTKTKNGELSILFTILNDELKIEILEIDMKIKPLKVEFSNGGLIKTKTNFYRAPGIIHEMQLIFENDHYNVSGSLAIAGELEGKAEVFLGKTKQDIMLEELP
SYRGKIIKRRSDKEIELEVEKLLGKMTLEEKIGQMYQASGTDTSAVGSNSEVIPIEKLIQEGKTGSLIFIGNNPGKIFYLQKLAVEKSRLGIPLMFAQDVIHGYQTVFP
IPLAWSCSFNPELIKEAVRISAKEASAVGLMYAFSPMVDISRDPRWGRVSEGAGEDPFLGAQIAKAQVEGYQGKDFYEKDSMLACLKHFIGYAAAEGGREYNTVEISEA
TLRNTYVPPFKAGIDAGAASVMNSFNVINGIPVAGNKAILKDLLRGELGFKGITISDFAAVDEIYIHGAAEDKKDAAMKAVTATMDIEMITTSFSTYLPELVKEGKVSE
EIVNDAVRRILSSKFKLGIMDDPFKYIRPEDADKLIFSKEHLEKSKELARESIVLLKNNGILPLKKNSKIALIGPCANSKDMLGPWQFSDKGDETVTILEGLTSKEVEI
AYAQGCNIDSKIENGFEEALAACKKADIVILALGESSKMSGEAASRQNINLPEVQLELANKIKELGKPIILLLTNGRPLIIKWFDDNMDGIIETWFLGSQAGNAIADVI
VGDYNPSGRLTMTFPLNQGQIPIYYNSFNTGRPYTEGNYNPFLSKYIDGPNTPLYPFGYGLSYTSFEYSDMNISSNKMTAEEGIEVSVTVTNTGVFAGTEIVQMYIRDL
YGSIVRPVKELKGFKKIFLVAGESKAVSFFIKEESLKFYNSKNEYKAEKGEFEVFVGRSSQETTGMKFEFI
>tr|A9V0V7|A9V0V7_MONBE
MGRCRALLGALLLLVGGIGMAGAAATTTAAPPEYPFRNPDLPWAARLDDLVGRLTLEEKLQQLQHGGAAQMTPAPAVERLGIGPFVWGSECVTGLGTDGNDPHGTAFPQ
PLGMAATFDPALLKRAAGTIALELRAQRNFDRENGVVKFHHGLSCWSPVVNINRHPLWGRNDETFGECPVLSSFMARSFVEGIQGNHTRYYAAAAACKHLDVYGGPDNL
RYVFDADVSQADLTGTFLMAFEECAAAGVMGYMCSYNSIRGVPACANYRTMTFFAREQWGFEGYVVSDQGAVFRITESHNYTANQTLGAVAALNAGCDMEDSDDAQHVA
YYNLSLALDLKLTDMATIDASVSRLFYVRMRLGEFDPPENDPWRSLNMSIVSSPAHVEMARDVATASIVLLKNQNETLPLSAAAKNASYCLLGPFADNADLMMGKYSPH
GSTNVTVTYRAGLAAALQNASQTASFQYLEGCTGPFCDGLDTAAVTTFIQQGCDTVLLAVGTSYHVESESLDRSNMSFPGAQPTLVQTVLEALGTKQRLVLLVSTAGPV
DLAALEQDTRVAAILDLIYLGQTAGTALADILLGETSPSGRLPFSWPNKVSDVPPIDDYTMQGRTYRFAQADVLFPFGYGLSYTQFNLSHLAAPYILPVCQALRLSVNV
TNTGRLSGAIPLQVYVEWPNAVGGPIRQLATTTRVFVDAASSKTVQLSIRPRELARASDLSCFAPKAERLEGNVLQNVSNVQNHGQCCTACQQFGDRCEGYRFATDSGT
CELLNELRGPVPGTVTSNYESAHVASRLELQTGPLEIFVSDQQPTGPRTTGTNVLHTRVQLSGSAFVSECL
>tr|A9VCI2|A9VCI2_MONBE
MRQGLLLVVLATVLVLARAATQQCTSWENVVIGGSDLQHAELVASPGQCCDLCYANADCTIYTWNATSKTCILKNKQSDSTSAYGVTSGTTNDRTIWSPCMGASSSYPF
CDTKLSVDDRLKDLVSRVSTADAATQLRARESAQIDNIGLPAYYWGTNAIHGMQNTACLADGQCPTSFPAPNGLSATFNYSLVKDMGRIIGRELRAYYNTKFHNGLDTW
SPTINPSRDPRWGRNVESPGESPFVCGQYGAAYTEGLQNGDDKDYTQAVVTLKHWVAYSVEDYDNVTRYEYNAIVSEYDLMDTYFPGWEYVVKNAKPLGVMCSYNSLNG
VPTCGNPALTAYLREDWGFEGYITSDSDSIHCIWADHHYESNAVLATRDGLLGGCDIDSGDTYADNLEAAVNQSLVNRSAVDAALTNSYRMRFNLGLFDPNVTNAYDRI
SADEVGMSSSQETSLLAARKSMTLLKNDGQTLPFATGKKVAVIGKSSNSAEDILGNYVGPICPSGAFDCVQTLYQGVAAANQGGATTLSDDVADINTAIQLAMDADQVV
LTISNYGQAGEGKDRTYIGLDTDQQELVAAVLKVGKPTAIVMLNGGLISLDWIKDEAQAILVAFAPGVHGGQAVAETIFGANNPGGKLPVTMYASDYVNDVDFLNMSMQ
AVAVLHLMNVNGERDDTGPGRSYKYYTGEPLYPFAYGLSYTTFNLSWSPAPPMTFTSTLRSTTYTATVTNTGSVGGDEVVFAFYKPKSESLKTLPVGNPVPIKEIFGF
QRVALGPGQSTQVTFELNAETLAQVTLDGHRELHSGEFEIELTRGHGEVLTGTAVVDVAQPERLATFRKWW
>tr|J9A271|J9A271_9PROT
MFDNRATRKIIFFIAIALIGLQLNACSEDRANQAELILSQMTLEEKIGQVIQADIASVTPAEVKEYNLGSILNGGNSAPGGGKSAAWQDWVALADAYWLASTDTSDGGL
GIPAIWGTDAVHGHNNLQSATIFPHNIGLGATGDTDLLERIGAVTAHEVRATGLDWVFAPTVAVARDYRWGRTYESYSENPQLVSDLGAALVLGLQGKPGSETFLGDKK
TIATAKHFVGDGGTQYGIDKGDTIVTEQELRDIHAYPYKQAFKNDVQTVMASFSSVNGTKMHESKTYLTGLLRDEMNFKGFVIGDWNGHAEIPGCTATNCPDALLAGVD
MYMAPESWKGIYESLKSQVESGAVPMARLDEAVLRILQTKLNAGVFEAGLPSKRPATQQQSLGSAEHRDVAREAVRKSLVLLKNNNNTLPVKPGSNVLVVGAAANSMKD
QTGGWTLSWQGNNNSNEEFETGETIYEGLETAITQSGGTISWSKDGRYQQKPDTEPDIETDMAPDIAIVVFGEEPYAEFHGDRMDLIYEFEGDPNLAILKQLKAKGIPV
ISVFISGRPLWVNSHINLSDSFVAAWLPGTEAGGIADVLIADANGKPRFDFVGKLPFAWPVGATGFAKDRKDAGTTFQFGYGLNYGDDVQVATLPVSEILKKPEEIFSGE
IIAKGKAVDPLEFYLGDLTNSNTPAPMLNLTSLGGSLVSEGTDYQAQEDSRKLSWVNANRAEASARLARPFDLTQIEDYERLTLSLTLKVSATGDDNIILGMACGEGCL
SALNMSSTSLSDLPRNQWNKLAIPLACFVNKGLDPTKVEVPLFVQAKQGWELSLHQASLSNAKTEELTDCPQ
>tr|Q1YRG2|Q1YRG2_9GAMM
MLTVRRYALAKGLVAVVAGLALSMMPVKLVAQDLNAPDKAAIVEKRVATILASMSVEQKVGQMIQPEIKFISPSEVKEYHIGSILNGGGSFPGERKNSAIQDWIDLADD
YYNASVDLSNGGTGIPVIWGTDAVHGHNNVIGATLFPHNIGLGAANDPQLLRQIGEVTAKEVAATGIDWVFAPTVAVVKDLRWGRTYEGYSSDAALVKAYAGEIVRGIQ
GEPGQLGSDSSKVVATAKHWIGDGGTYRGMDQGNTILEFDQLLELHGQGYLSALDADVQSVMVSFNSWNGRKIHGHKELLTDVLKGQLGFDGLVVSDWDGVGQVEGCTT
ESCPLAINAGIDLIMVPKGWKNLISNTLAQVQSGVIPMARIDDAVTRILRIKVRAGLFDKGAPSTRALVGDSKILGSAEHRAIAREAVRKSLVLLKNKNQLLPLKGEQH
ILVTGDGADNIGKQNGGWTITWQGTENKNSDFPGATSIYTGLKQAVGSNGGSVELSADDSWVKKPDVAVVVFGEEPYAEGVGDVESLMYRDGYRADLDLLQSLKGKNIP
VVAVFLTGRPLWVNAEINSSDAFVVAWLPGSEGVGIADVLVADKAGKPRFDFTGRLSFDWPNRELNAVNKNLEVRDNLLVRGQGLSYGDGEILAGKLSETPMLDVSKVA
RVIFNGSVKPPYKTFVGDSSNWQKLVEGHKSSSAFGDLTVTKVDGAVQEDSRLVEWKKGRESQFYWQSETSMDFSALLEEDASLVLAFTVNKHPQGPVTLRMDCGWPCS
GGFDFTSRLRKIPEGQAVRMGVRLSCFENVGVNLSRVNSPFVLSSSDVFAITLSDALITSDISDKSTINCE
>tr|A0A1K1NI46|A0A1K1NI46_RUMFL
MRKTIALLLPLALLASTVSCAEKNTPPSSSDTATESEIEIASDSTVKDHGPKYQRYASMTPEEIVGELTLEQKANQMVQPAVYNITTDDMKANDYGSILSTVGCINSDA
WCETVDGFQNAAIESEAGIPYIYGQDDVHGVNYCRDAVYFPHNIGQGAANDEELAYQVGLITADEAKLCHMLWNFSPCVAQSVDPRWGRTYESYGSDLDTITKLSTAYT
KGLQDGGLVACAKHFFADGNVVYGTGEEGTPLKRIDRGDAQLTSAEIEELLKVYQAQIDAGVQTIMISHSSLNGVKMHENKEYIMKLKDEMGFEGFIVSDWGSIEHTSG
ATYKEQVINGVNAGIDMLMETDKYDEAKQIIVDAVGSGDITEERVNDAVTRIIKVKKEAGIFDDPLCENLQTKQKETGSLEYRKIAEKLVEESLVLLKNDNKVLPIKEG
TKVYITGPAANNGQVQCGGWTMDWNGASTKDVPGVTTIREAFERYAESYGIEVITDKEEAEKADVVLLCLGEQAYAEWNGDTEDMSLFGALGLDGNSKARQEAKELGKP
TVACIVAGRQVLINPRIYDQWDSVVMCYLPGSEGKGISDVLCGCADFKGKLPAPWYGSLDQIGTDKCWLERGYGLSYGGNFKAKAEPETILDPPTVVAEDPATEGTNYT
KGLFKDGVYVNDYAGIKFNVPGNLNYFSAYNSELKERQIEELTDETELAILKATIYEANFGNMNEATTITFYNIPMLLDSVMSDREDITEEDALDYHKKIVIDNSEQQG
LKIDFKKREKVTLGGQEYMRDIWYYNGDENNYEAFWIRKIDDDLICELWYTAHESKKTPEYFESLFEEIK
>tr|H3NUN5|H3NUN5_9GAMM
MRLLRLSISTSAFILLLSACQLEKPSTANSVDSSCAATAFSMDEAKSCASAITAQMTIEQKVGQMIQGEIRDVTPEDVRVYGLGSVLNGGGSFPQENKYATVEDWVALA
DAYYTASMDTSSGGAGIPIVWGTDAVHGHNNVMGATLFPHNIGLGATRDTKLVSQIIGATAREVKATGIDWIFAPTVAVAKDARWGRTYESFSSDPQIAASFVAPIVDA
MQAEGIASTAKHFIGDGGTLRGDDRGETSLPLEELVAIHGQGYVEAIDKDVMSVMSSFNSWYGDKIHGSKAILTDLLRGDMGFEGMVVSDWNGVGEVLGCTNDNCAQAV
NAGIDMVMVPADWRSLYNNMLEQVAAGEISEARIDEAVSRILVMKIKAGLFTRGLPSQFAAEYRDQIGHTDHRAIAREAVRKSQVLLKNNQGLLPLPADQHYLVTGPGA
DDIGQQSGGWTISWQGTGNTNADFPGATSILGGLKQQLEAAGGSITTDESAEVDAAIFVFGETPYAEMQGDVYSVAWYDQRAERSRMKALKERGIPVVAVFLTGRPMWV
NDILNLSDAFVVSWLPGSEGQGVADVLLQDADGGVQHDFTGRLPMPWPALDVNAIDRDLPVDTFAFPIGYGLSVTDAFAWSDLSEVLIGADNSLDQEVFNGGPRGSWKL
FSGDSSDWAVEATSSSSQSSTGSVTIEAIDRLVQEDARRLTFTGAEGRLSQVYMQSEYPTDLTALNDAGGALSIDFRIQSPPTSPVSLRMDCKYPCSGAVRFTKVLLDA
PVGEWTKRAVPVACFQEAGLNLAQVNTAFVLATEGDITLDISEIKLTTMPEIRSIASCADLVNDTMLLD
>tr|A0A5N6KX38|A0A5N6KX38_9ROSI
MDGSGDWADAHRRAQDFVRQLTLAEKVNLTTGVGWQGERCVGQTGAIPRLGMRSLCMQDSPVGVRFGDFASVFPAGGTIAASWDRELIYRRANDMGSEHRDKGVDVQLG
PVVGPIGRSPAGGRNWEGFSPDPYLSGVAVAESVKGIQDAGVMACTKHFIGNEQEHFRQVGEANGYGYNITNTLSSNIDDRTMHELYLWPFADAVRAGTASIMCAYTNT
NNSQSCQNSYLLNHLLKGELGFQGFVMSDWQAQHSGVAGALAGLEMAMPGDTLFNTGVAYYGTNLTIAVLNETVPEWRIDDMATRIMAGWFYVGRDQHERPINFDSWTT
DTYGNANFYAKEGYGLINEHVDVRGNHGADIREMAAKSTVLLKNVRNTLPLPAKEKFTGVFGEDAGPNGWGPNGCPDRGCDNGTLGMAWGSGSANFPYLVTPLTAIENE
VSKVGGIVQGVIDNWATDQITSLASQASLALVFVNSDSGEGYINVDGNEGDRQNLTLWQNGETLVKNVSALCNNTVVVIHSTGPVLLDSFYDNDNITAIVWAGVPGQES
GNSIADILYGRVNPGGKLPFTLGRTRRDYGSDLLYKPNNGKDAPQDNFEDGVYIDYRGFDKRNVTPIYEFGYGLSYTTFEYSNIRIVNRNAKPYAPNTGNTAAAPVLGQ
SGQVGDYVWDNSSNRVYNYIYPWLNTTDLAASSGDPYYGANVSLPANAQDGSPQPVQAAGGAPGGNPLLYETLYTVYATIKNTGSVGGDEVAQLYVCLGGPDDPLRVLR
GFDRLSIEPGQSTTVGFDLTYRDLANWDVVKQDWTISSYAKTVYVGASSRNLPLTVPVPQQKAARSEL
>tr|A0A316VLP4|A0A316VLP4_9BASI
MYTSNLVQKLFILISCSLLVQARSLLDLDFGIEFSQRENVEFSPALDLQKRQADADTGHRQGGYTTWTEAESEAKKLIGMMTLDEKVNMTVGIDQEFCTGMTGSVPRLG
IPSFCLSDGPTGISVTYNVSQFASPITLAATWSPQLASQHGLALGQEFHDSGVNFIFAPVAGGPLGRSPLAGRFWESLGADEYLTGVLGSQIVKALQEDANVVACTKHF
IGYEQESDRNMNTLLPQTQKPYSSDIDDFTMHQLYMWSFAETARAGTGQVMCSYNRVNGTQQCQNDASLNGLLKHELNFRGNVVSDYGAAYSNEEATNNGLDLLMPGEG
QFGIYPNYRGAHGEKLKDAVNSGKVKIERIDDIVKRILTPILRFQYTSKFQNFITPKIHNAYGIVGTPVSNDLGSKVQRDHFKVIRKIGSESITMVKNERNVLPLSKAK
LNEKKATIAILGSDAHGSTDRGKSCNPNNECDDILYDQGTQTDGQGSAFAYPPYIINPLDAIKSYVKANAPQVTIQSSRGKHFSISTADRASRAVDLARKADVAIVFGN
ARGKETEDRSNLTLREHADDLIKRVAAVNKQTIVVLHTPGPVLVEEWIDHPNVTSVLFAYYPGQESGASLTPVLFGDESPSGRLPFVIGKKLDDWLPNSISDPHLTIHP
RIPFSEGILIDWKWFDAKGIKPRYPFGHGLSYASFSYSSFEVHKTFLPDKETEFTTNEAFEPHQNVQGSSLYDTVLEASVTVKNAGKMKASEVVQLYISFPKDSTGMTA
SHLLRGFDKKEIGAGESVKFTFSIRRKDISIWDTVHQRWRIPSGEFTFFTGPQGAADWDKKLQARVIF
>tr|A0A0Q7SVZ9|A0A0Q7SVZ9_9CAUL
MLKTAILSLALAGSLAAPAFAAAPAPVVHPDLWPQAHSPVRRDPAMEARIDAMLAKMSVEEKVGQTLQPEMKSIVPDDVRRYHIGSIENGGGSVPGGNKHASVGDWLKM
IDGYYDASVDPANGGVRIPMMWASDAVHGHNNVYRATLFPHNIGLGAARDPDLVRRIGAATAQEVRALGMDWSFAPTLAVVQDDRWGRTYESYSEDPKIVAAYAAAMVR
GLQGEGASFLDKDHVISTAKHFLGDGGTDGGRDQGDNLADEATLRDVHGAGYPAAVDGGVQAVMASFSSWHGVKMHANKDLMTGVLKDRIGFDGLIIGDWNAHGQIPGC

TKGDCPTAFNAGIDVFNVPEDWKALYAAMVREVNDGTIPMARLDDAVRRVLRVKLRAGVFDQPKPSARPHAGDEALLGAPEHRAVAREAVRKSLVLLKNDGGVLPLDPR
KRILVAGDGADSIMKQTGGWTLSWQGNDNVNADFPGATSIWGGIEAAVKAGGGTALLSPDGAYDAKPDAAIVVFGENPYAEFQGDQADVALHAGSAESLALLKRLKAAG
VPTVAVLISGRPLYLNPQINAADAFVAAWLPGSEGQGVADVLIAGPDGKPRHDFTGKLSFSWPKRPDQTPLNVGQKAYDPQFAFGYGLTYAAPHAVGPLPEAEVAIETG
PRGVFFRDGAGANGFAVSIGDAQAPTIAAVAARVATYGAEGLVLRKVDRRRQEDAWSARWSGGEKAWLSLTRPTPLDLSREANGAMALTFELRIDTPPAGPVWLSAGGD
KPVTLPIGAALSGPVGGWRTLRVPLRCFGAAAEAVKVVRLETASALAITVSDVALTEARADDACPAG
>tr|A0A5K7XJN4|A0A5K7XJN4_9BACT
MKASIGFKFRQCCALLATALIGLSNGSPQLLADEALVTAEKTAALQDASAEPRESIAKFNSAAADRAAEVDRLLAAMTLDEKIGQMCQVWPEQGELTPALVESLRQGEV
GSLINCPDSKFIVEVQRIAQTESRLGIPLLIGRDVVHGYRTIFPIPLGQAASWNPELVEQAARIAANEASSEGINWTFAPMVDVGRDPRWGRIAETFGEDPRLSAALSS
AVVHGFQQEDDEGLHGLVACAKHFAAYGLSEGGRDYNRASLSIADLHNIHLPAFKSSLDVGCRTFMTTFSEVNGIPGTAHAYLLQDVLCDSWKFSGVVVSDWNSVIEMV
AHGFSADEAEAAQQAVNAGVHMEMVSPTFHDNLSRLVEQGRVDEAALDNAVRRVLRLKLELARNDSANQSAEKSVATGATAPRPGAKLLHPRSLELARRAARESIVLLK
NAEQTLPLHREELRRVAVIGPLADAPLSQLGCWSVDGSPADAITPLAAIRNAVGDTVEIAYVAGMTSSYANRLTELAKAQKAAEQADVVLLFVGEDAVLSGEARSRMTL
DLPGAQAKLVEAVAASGTPVAMVVLAGRPLAIGAEIDAAAAVLYAWHPGTMGGPAIVDLLLGDAAPTGRLPVTMPKHVGQVPLYYSHSNTGRPSPADFRPLSQSQGKDL
PAEFQYRSHYVDGDPFPLFPFGYGLTYTTFCYDGFELGAAAIGPQQTLAVRARITNTGTRSGVETAQLYVRDLAASIVRPVRELKAFRRVHLRPGESQAVEFALTADDL
RYFDNQGKSVLEPGKFAVWVGGDSTAALGGEFELTTASDPSSQSAPAVARAPKVETTKQPVRADSGT
>tr|A0A7J7GYW5|A0A7J7GYW5_CAMSI
MRRVPLAVTMVRFSVQFVLLWWSCIAMAMADEYVKYKDPKLPVGVRVEDLLRRMTLAEKIGQMVQVDRSVATDEVMRNYSIGSVLSGGGSTPLLHASAKDWVKMVNEFQ
KGSLSSRLGIPMIYGIDAVHGHNNVYEATIFPHNIGLGATRDPELLRRIGDATALEVRATGIPYVFAPCIAVCRDPRWGRCYESYSEDPKIVREMTDIILGLQGENPNS
SLKGVPYVAGRNKVAACAKHFVGDGGTTNGVNENNTVIDMHGLLSIHMPAYTDSISKGVSTIMVSYSSWNGEKMHANGNLITRFLKDSLKFKGFVISDWEGIDRITTPP
HSNYTYSVQAAILAGIDMVMVPYNYTEFIDDLTKLVKHKVIPMDRIDDAVSRILLVKFTMGMFENPLADLSLIDQVGSQAHRNLAREAVRKSLVLLKNGKSPDDPLLPL
PKKAPRILVAGSSADNLGYQCGGWTITWQGFSGNNYTTGTTILNAIASTVDKSSEIIHLENPDVDYVKSGKFSYAIVVVGEYPYAETAGDSPTLTIADPGPTVIGNVCG
AVKCVVVIISGRPLVIEPYISSIDALVAAWLPGTEGQGVTDVLFGDYGFTGKLSRTWFKTVDQLPMNIGDSHYDPLFPFGFGLTTTKSTVTRSISASIDQRPSLLIIKN
RTPGCNRRASEVGWCAPANGWLKINFDGALFKELKAVGVGVVIRNHLGEVMAALSERLPFWVDSDCAEAYAATKAVELARDLGFSDIHLEGDSLRIVKALREEAEFMSE
YGHILLQTVGACKSFSRFHVSHVGRQGNGLAHGLARMARHQNYQQVWMGDLPASLIAIATSEVSSL
>tr|C7Q9F7|C7Q9F7_CATAD
MRARTPWSRPLRSRTQARSTRRVAHVAIAAALTLAAVDAAAATGAAASTAQPRYLDRTAPIAARVNDLLGRMTLPEKAGQMDQQLVDNATAASGGACGAAGFNLPTPAC
MQSALIDQNVGSILAGGTDNPSDTTGSGTSGNTAQDWANDYNTIQQYAIAHSRLHIPLSFGVDAVHGFGHPWQAPLFPQSIGMGATWDPSQAKAGGAMTATALRSTGWT
WAFAPVQDLARDNRWGRTYETWAEEPALSSAMGAANVTGLQTPAPAGGLDVSATVKHFAGYSESVNGHDRDEALLPLNYLQSTILPSYAGAINAGADAVMVDSGSINGV
PATSSHYLLTDILRGQMGFKGVEISDYQDVQALQTTYHIAASLPDAVALAVNAGLDMSMEVNGPDQWQSAIIQDVGNGKIRMSTINDAVRRILTMKFQLGLFDQPCVAD
PGKPCLNAGAADAVVTSGRDQTLKATQESITLLRNQNSVLPLPAGSRVVVTGPSADSMTNQLGGWSVSWQGVAGAGHVCCMGSPDQIPPGTTVQTGVLGADTHATAISD
QAAAVAAAPNTDAYVAVVGEKAYAEGLGDNPAPALPADQQALISALEATGKPVIVVVEAGRPVALGSAEKASAVVMAYQGSTEAGQAVADVLFGKTDPSGHLSISWPSD
APAVGGDFNSTAPSPLGDEPKFFDQLPGTGSGPGNAYNPLYPFGYGLSYTTFSHSALAVTPNASAHGTLTATLTVTNTGTRDGTDVVPLYVAQPVSASAEPPQRLVGFT
RVTLAAGASQTVKVSFPATALARSQGDINASAPPTVEPGGYVLQVDKEADTTPYDVDLSAPFTLR
>tr|R1D6Z6|R1D6Z6_EMIHU
MTVEEKIAQLGYNIRPCATMDVAKDYANGVGGCAVSSIADTIKMRKALQKLTRLGIPPSIHGETTHSGGAANTTVFPMPCLQGATFNTTLVEEIAASNALQLRAAGGDM
GLSPILQVCTDPRFGRLEENFAEDPYLVGAYGVAAVRGLQGRDGCGGADTYLGSPRSRVAAQGKHYAMYGAAGKDGYTPFGGGIAARTLFDVYLRPWREFARAGGRGVM
AAHNMVDWVPMHANRPLLHDALRVRFGLGEGGYIGSDNTNVEGLADYFHGFAANTSDAAAMALAAGIDQDMPGGAFLSGLLPLVASGRVPMAHVDRACGNVLRRKFAAR
LFDEPPDATLARETDSARARALARQAAEEGSVLLINRNAALPLSASSAPTSPTRPLKRVALVGPFAAGSAYTIQAMVGGYAWVPPPRAVVTVGDALRRRGVEVDVAVGS
GAGVGGPAASEADFEAAVALARAPSTDAVIAVLGTTSCGCCVRCGNGEAGDRMSLELEGRQLELLAALVNASHATARQSGRAAAPVIVVLIHGRPVSFEGGGPTAAGLE
RRRSERRSSGDGDDAVGGDSDVLLLGVDALLAAWRPGEEGGSAIVNLLFGDANPSGKLAQAWQRSAGFIHSPTSPWFQPHSSMTPGRYFGNGDGVPLLPLFPFGHGLSY
TKFDFRGLRVDGARGLPPAAAGAQLEQLGLNVSLVVQNNGSRAGATPVIVTYSKVTRGVVRFDRETCAFAKVFLERGGSKRVVARVRISDLARWDPYHGPTAMAQDDGA
PRTIASGAWVVDGGTYTFFAAGCVANSVLRDIHHAGDPSCPFFESAVVGKSVVIGREGEVYGVYM
>tr|A0A1Y6CA28|A0A1Y6CA28_9ALTE
MASLPHTHKTKNSHAAKLWQSPANPLSTQPKLAYKKEIAAILARLTLRQKIGQMVQAQMDAITPEQVRDYHIGSVLNGGNVYPNNDKYASTQEWVTMVDTYYRASMDSS
NERVAIPIMWGTDAVHGQSKIVGATIFPHNIGLGASRNGALVRKTAEITAIEMLISGVDWNFSPSVAVVRDLRWGRTYESYSENPQLVKELSEDYVLGLQGCLSEGTFM
DDSRVMATAKHFIGDGGTEQGIDRGNNIDSEAELYRVHAPGYLSAIKAGVLSIMPSHSMWQGERLHGHHYLLTQVLKKSLGFDGFLIGDWNSHGLLDGCKDSSSPLAVN
AGLDMFMVTHEWQAFIENTATQVEAGQISEARINDAVSRILRAKFRAGLFKKGPPSERKHACKNNLLGSPQHREVARQAVRESSVLLKNNDALLPLNPRLNFLVAGDGA
DNIGKQAGGWTISWSGAGTTNADFPKGTSILSAISKEVNKAGGTVHYDVAGTYTKKPDVAIVAFGEEPYAEWFGDIKHLAYQATDNKDARLLEKLKKAGIPVVALFISG
RPLWVNRIINASDAFVAVWLPGTEANGISDLLFTNTDGEIKYDFTGKLPFSWPKSPLHNQLNSDHKDYDPLFPFGYGLSYMSQSQLAELSVEDNIPESLKDPGVWLFNG
RAIVPWFIGLRDEDGFSMYLGSRVERPNLSIQAIDRHTQGDAVKLCWSGEKESEIVILNEEITQDYLPYLDKDAALVFEINIAGTHANSLKLSLGDMAKAEYIEVADIL
EKDMGWQTLSVPLACFKNKGADLSQINKPFCLRSTGLLNITLSNIRIELNAASTTSITCRNDAN
>tr|A0A1V6Q0R0|A0A1V6Q0R0_9EURO
MWGVYIRLYRHQRHINWFEPAESVQETENITPVLNMKSVLSLLAFAPTALAQAQTSYVDYNKEAQPNLTPQSVATIDLSFPDCSNGPLSKTLVCDCSARPHDRAAALVA
LFTLEELTNSTGNTIPAVRRLGLPPYQVWSEALHGLDRANFTDSGEYSWATSFPSPILSMAALNRTLIHQIGDIISTQGRALNNVGRYGLDVYAPNINTFRHPVWGRGQ
ETPGEDVLLASAYAYEYITGIQGGVDPEKLKLVATAKHFAGYDIENWGGHSRLGNDMDITQQDLAEYYTPQFVTAVRDARVHSVMSSYNAVNGVPSSVNSFLLQTLLRD
TWGFVEDGYVSSDCDAVYNVFNPHEYAGNVSAASADSLRAGTDIDCGTSYQYYFNESIAQGEISRSDIERGVIRLYSNLVSLGYFDGKDSKYRNLDWSDVVKTDALNIS
YEAAVESIVLLKNDGALPLAKNTSSVALIGPWANLTQGLIGNYFDPAHAPYLTTPLAALQKSSLDVNYAFGTNISSETTDGFGAAIAAAKQSDVIVFAGGIDNTVEAEG
MDRMNITWPGNQLDLIKELSQLGKPLIVLQMGGGQVDSSSLKTNKNVNALVWGGYPGQSGGLALLDIITGKRAPAGRLTVTQYPAKYALQFPATNMDLRPGGDNPGQTY
KWYTGKPVYEFGHGLFYTKFKASLARSRSTSNGTSFDIGKLLSRSHAGYNVAEQLPFMNYTVGIKNAGRVVSDYTAMAFVNTTAGPSPYPNKWLVGFDRLGSIKPHDSQ
TLSIPISLDNIARTDEFGDRIVYPGRYELALNNERSAVMSFTLTGNATTIALWPKEEQQISPA
>tr|A0A2X0L6M6|A0A2X0L6M6_9BASI
MNLMIAVTARPAPLASRPAGSSGDQYRTANLMQPPLAMANNVPTPKVDAQPSAEWSSAIRKARAFVNQLDLEELVNLTTGQGAHGRCIGQTGDVTRLNFTGICFQDSPL
GVRSTDGVSAFPAAINVAATWDKDLMYMRGAAMGSEFRGKVSGVNVALSPMMNLARTPEAGRNWEGFGGDPYLAGFGAAMTIKGIQSTGVIACAKHYIANEQEHYRGGS
GAITSSSNIDDRVMHEVYNHPFSESIRAGVGAIMCSYNRINQTHACENSYLMNKLAKQDLAFHGFVVSDWAAQTSGVSSALAGLDMSMPGFRSYTEGLDNQQNPAKSNT
SYWGAELVKAVNNGSVPLARVKDAVTRSMFISSAFPESGEDPRRAVLTPSPALIPCAAMAAYYQLGQDSKDYPDINFSAFTLDTYDSNRRLVNSHVNVSSDHNKLVREI
GAASNILLKNVANALPLKAGQLKSVGIFGADAGPAKNGPNEFPDRSGLDGTLAMGWGSGTVNFPYLVDPLSAIRYKLGQTNPKTSISSVLDNNATSAIQRLARLVEKCL
VFVASDSGEGYLTVNNNQGDRNDLELWQNGNQLISKVASLCSDVIVVIHSVGAIDMESWIDNKNITAVVLAGLPGQESGNSLVDVLFGDVNPSGRLPYTIGKKRSDYAA
DVLYKSSMAVPQITYSEGLFIDYRWFDAKSIAPRFEFGFGLSYTTFRYSALSVTALSQGGSTTKSNLYHDFYCITYQVTNRGPVDGAEVSQVYLAFPPSAQEPPKVLRD
FARTFLKNRTGSTVTIKLNRKAFSIWDVALQSWKVPKGDFTVFVGASSRDIRLRSAIVNPSAS
>tr|A0A507FDU6|A0A507FDU6_9FUNG
MNILAVLAIVTPLAVYADLADVKARTWEESIALAQKTVAKMSVEEKVKLSTGLGWSYTQCVGNTAAIPSINFPGLCLQDSPTGIRFARNVSAFPASLNVASTFDQGLMY
NNGRFMGEELRDKGVNVHLSPVANIMRTPQGGRGWEAQGADPFLTGVSVTAQINGIQSNGVQATLKHYVLNDQEKFRQDRGDVIIDKKTLMDYYIRPFKMAIDHSGPAS
IMCSYNSVNGVNACDNPVLMTILKEELGFKGYVMSDWWATHTELDGLKAGMDMVMPGSKACCDTGNSTVVPAWWGETLVSNIKNATVKMDRVDDFATRILTGWYRQRQD
ENFPSTNFNSWFPEEVQLNVQRNHKAHIREVGAASAILLKNSGVLPLSAKSGNLALIGSDAISPPSGINAGGDHGVNPDGTLAQGWGSGTTNFPYLVAPQEGITAAASP
LGLKVSVSPNNWDIPAVEAAAAAAGTSIVFVNANSGEGYITIDGNEGDRKNLTLWNNGDRLVEAAAAKGPTVVVIHAPGAVDMPWINLPNVKAVIMALLPGQESGNAIA
DVLFGAVNPSGRLPFTIGHNITDYHTQIDMINPIQHYSEGGYFDYRWNQKNNIAPLFAFGHGLSYASFAYANTAVTQVCKGSTKVQVDVSNTGAVDGHEVVQVYLGYPK
GVDQPAKQLKGFSRVMIGAGKTASVSIEITPDFIQIYDDAKNTWFTPEGTYTVFVGASSADLRGEATFVIDSACSTATTTASAEPSGATTASAASSASASASASASAAS
SASAEPSAPASSGYVAPSAAASASYAAAPPASAKPKPTPSNLYSSGEIATVSLFALLYPIFFA
>tr|A0A0D2MXV5|A0A0D2MXV5_9CHLO
MWMALVVVTALLSRSALADVADAGAAEAEAPRGARALSPGFLPINARTPDTVVAAAAASTPKWKDAAVPAAERAADLVEKLTLQEKLTLLSNKQQAVPRLGLKAFDFLT
ECLHGLWETNPYTKQPPTIFPQPLVMASTFDRLLVKRVFDAVSTEMRAKNNFEVKSKGVSRALTCWTPHMNILRDPRWGRGSETWGEDPHLSSVMAAHVVRGLQGEDPN
YVKVAATCKHFYGYSFEMADGQSRYSFNARIDPVDAADTYLPAFDACIRAARTQGVMCAYNAVDGVPMCANKPKLQGLLRDKWGFDGYVVSDCNAVQGLVWGHKTARDE
PAAIAQAIKAGTDLLCDNMDSQKAASQALSQGLLTEADIDSALNHTMGVRIRTGQFDPPSSMPWAGAELDKVNSAGHKELARSVARKGIVLLKNEPALNATSRMLPLAK
DKLKKVCVVGPLADSAEHMMGNYYGAWDKGASTPLAAIKAELAGATVAVTTDNTMRTMAWNYYDWPLESALQSCDGADAALVFLGASIISTPLTPAQKDSRLSYRPVSE
GEGFDREGLGLPGRQLDLVKALARRSPATPIALVVMNGGPVDLSWAQGSPSVAAIMAAGFPGQEGGRAVSDLLFGRASPSGRLPNTWYREAFIAAAPMEEFGMRPDAAK

GYPGRTYRFVEDPKYVLYPFGHGLSYATFSYSAMSVAPTTDAGGKCAGYDHAAYCVSVTVRNTGAMAGEEVVLLYVSHAEKAGTGGYPLRSLRAFTRTRALAPGESQAV
VLPLRTADFTLPRPEDGALVARLGTWRAQISGLTVDVPIARGQLDLSATTAEWPAQAPAGGE
>tr|A0A453D9Y6|A0A453D9Y6_AEGTS
MATAARPPFLAVLLLLVATVMLSWGGNVRVAEAQTPVFACDASNATLAGYGFCNRKASASARAKDLVSRLTLAEKVGFLVNKQAALGRLGIPAYEWWSEALHGVSYVGP
GTRFSPLVPGATSFPQPILTAASFNASLFRAIGEVVSTEARAMHNVGLAGLTFWSPNINIFRDPRWGRGQETPGEDPLLASKYAVGYVKGLQDAGAAGVTDGALKVAAC
CKHYTAYDVDNWKGVERYTFDAKVSQQDLDDTFQPPFKSCVLDGNVASVMCSYNKVNGKPTCADKDLLEGVIRGDWKLNGYIVSDCDSVDVLYTQQHYTKTPEEAAAIT
IKSGLDLNCGNFLAQHTVAAVQAGELSEEDVDRAITNNFIMLMRLGFFDGDPRELAFGSLGPKDVCTSSNRELARETARQGIVLLKNNGALPLSAKSIKSMAVIGPNAN
ASFTMIGNYEGTPCKYTTPLQGLGASVNTVYQPGCTNVGCSGNSLQLSTAVAAAASADVTVLVVGADQSIERESLDRTSLLLPGQQTQLVSAVANASRGPVILVVMSGG
PFDISFAKASDKISAILWVGYPGEAGGAALADIIFGSHNPSKCRCVAASFSLTCSSVVTTLCRAFSDKRALCFGHGRRWEAAGDVVPGVVRRHGQNDGHANAAGHVDGL
PGPDVPVLHGRHGVRLRGRAELHQDVPPARVRAAVVRVHAAGRGPPVPRRGVRVRGGRERPLRGPGLRREAPGAERRRGGRRALGAAVLVAAVGAQRAGEAPARVREGV
AGARGGRHGGVQGGRVQGPERGGRAGRPQGGARRPHAARRRPQAHRGTTGLMESRVIKENST
>tr|A0A2A2K1B1|A0A2A2K1B1_9BILA
MRAGLYMFGALALGAAVPALTQPRAQTRATSAASPLAHPDTWPQLEMRRQRDPKVEARVDSILSRMSIEDKVGQLLQVDIASITPGDLETYKLGSILNGGNSAPNNDEF
APAAEWLKLFDAFYDASVKRSDGRPVIPVIWGTDAVHGANNIVGATLFPHNIGLGAMRDPQLIRKIGAATAAETAATGIDWSFAPTVAVVQDDRWGRTYESYSEDPAVV
ASYAGEMVQGIQGTVGQDFMKPGHVISSVKHFLGDGGTGGRDQGDTRVSEAVLRDVHAAGYTTALPAGALTVMPSFSSWNGEKMTGNKSLLTGVLKERWGFDGFTIGDW
NAHGQVPGCTNEDCPAAINAGLDMFMYSGPGWKQLYDNTLREAKSGAIPADRLDDAVRRILRVKVLAGTFDAGRPSSRPLAGKFDLLGAPEHRAIAREAVRESLVLLKN
NGGVLPIKPSASILVAGRGADDIGMQSGGWSITWQGTGVTNADFPNGQSIYAGIAQAARAAGGSATLATDGRYTRKPDVAVVVFGETPYAEFTGDRATLEYSPEDKSDL
ALLKRLRAAGIPTVAVFLSGRPMWVNPEINAADAFVAAFLPGTEGGGVADVLLAQADGRPAHDFRGKLSFSWPRRLDQYVLNRRDPGYDPLFPFGYGLTYGETRVVPPL
DESKPKAAGVNQNALFVRGRLVPGARLVTSGAVRQTRVDRSGQEDSLRLSFAGAGSASIVEAEPIDITRESNGQLSLLIDYRVTQAAAGDVTLSIAGGRTGSVPIAGAL
RSAAADWRQLAVPLRCFADQGVDMAKVGTPFAIATTGTLGVDISGVQILSAPPGPVTCGNK
>tr|A0A1Y0G0X0|A0A1Y0G0X0_9GAMM
MVMKYVRATFKRLLIIAGGCMSLSISLAPADQPLYKDSTKPIDARVNDLITRMTLEEKVAQLQAVWLKRQELETDAGVFTPAKAKDILGSGIGQIARPAENKAPVSPNK
TPEQTIAFVNAAQRWLIENTRLGIPVIFHEEALHGHAGRDATSFPQAIAMASTWDTQLVESIYKVSAQEIRRRGGTQALTPILDVARDPRWGRIEETMGEDPYLVAALG
VAGVKGFQGDELGRIGPDRVIATLKHLAGHGEPVGGLNTAPAPVGERLLREVFLFPFEAAVKLGGARSVMASYNEIDGVPSHANGKLLNDILRGEWGFEGALVSDYFAI
KELVTRHQLSDSLEDAALLALNAGVDVEMPDGETFPLLVQAVNDGKLGEAVIDQAVARVLHEKFLLGLFENPYTDPAGAEAFIGNDQHRQLAQQAAEKAMVLLKNDNNL
LPLDSRKVKSIALIGPHVDEVLLGGYSDVPEHAVSILQGLQEYLGEDINIRHEKGTLLTMNAWDQGADSVAANSFSKERWHTDEVVLATSKDTAGMIKKAVAAAKKSDV
AIVVVGDNEATSREAWAETHLGDRTSLELVGEQQALVDAVLATGKPTVVLLINGRPLSISKIAREAPAIIEGWYLGQETGHAVARVLFGDVNPGGKLPVSIPRSVGHIP
AYYNHKPSAKRGYAFTETSALYPFGHGLSYTQFTYSDLKVNKASAKAGDVVDISLTLTNSGTRDGDEVVQLYIRDPIASLTRPVKELKGFQRVHVKAKDSVKVTFSLAV
NQLGFYDQHMRYVVEPGKVELMLGSSSGDIRLNSEFTIVGKTVDVSSNKAYLSLSTVSQP
>tr|A0A2P2EA49|A0A2P2EA49_9PROT
MKNRALNPRLGTVVRVGLVGILTACFSLVGCASGPKKDDQVEARITEIISKMSVEQKVAQLIMPDISTITPEDVAKYRFGTILNGGNSGPYGNERAKPEEWLKLADAFW
AASRTPHADGSPVIPVLWATDAVHGHNNVVGATIFPHNIGLGATRDPALIREIGRITAAEIKVTGIDWTFAPTLAVVRDDRWGRTYEGYGEDKQLVSDLGAAMVEGLQG
VGSEFLSQERVIATAKHFFGDGGTGGVDTGDTVGKEADLVALHAFPYRAALQSGAQTVMASFSSINGQKMHGSKSYLTELLKVEMGFDGLVVGDWNGHGRIPGCTNSDC
PESINAGLDIFMVPEDWKALLTNTKAAVDSGKISQARLDDAVRRILRVKMRYGLFDLPAPSARKLGGQFALLGAPEHRAVARKAVSESLVLLKNEGVLPIDPRRTILVA
GEGADNIAKQSGGWTITWQGGGDLSNADFPGAQSIFAGIAQQAEAAGGKAILSVDGSYTQKPDVAIVVFGEKPYAEFMGDVKNLSYTDAKPLTLLKKFQAEKIPTVAVF
LTGRPLWVNKELNAADAFVAAWLPGSEGGGVADVLLKTKDGQIQKDFKGKLSFSWPQACDTYSLNPGEAGYKPLFEYGFGLNYATKAAWTPLGEACALSKPNADNDVAV
FARGQSLAPFALFAADGQGQRIRLASAKGRSADGKVSVVAMDRSAQEDARTISWTAGGAFGFDLAGTLNPAALGQGRALRIEYQVGTRPSGPVTLSMSCGPNCRDEIDI
TASVALAEQKGWLTAVVPLSCFRKGPQIGLPLSIQSSGRFEMSISSVTLVDDGGETSCRF
>tr|A0A165JBY4|A0A165JBY4_9BASI
MSLLFCLVLAALWPVSLADIVSGVPDAAPPGFEEWISPIVVPAKPVSGDGPWASAVARARDLVSQLTLDEKINITTGAGGNLPCVDGTGSLPRFNFSGFCLQDSPLGVR
DTDYASAFPAGINVAATWDKQLMYDRGFAMGAEHRGKGVNMIFGPMTNLGRVAAGGRNWEGFGADPYLAGVATAQTVLGIQDNNVIACVKHFYGNEQEHYRGGGSSSEQ
IYSSNIDDRTAHELYIWPFAEGIRAGAASIMCSYNKVNQTQNCQNSKLLNGIIKEELDFQGFIVSDAAAARSGVDTALAGLDMNIPSFYYFGTGSYNGGASLPNEGNPD
NTTYSWWGSNLITAVMNGSVPEARVDDMVTRAFAALFFTGQDLTRVPNFNYQTEDTYLNGMLVNQHVNVMGNHGQLIREIGAASIVMLKNTGVLPVDVTKYRRYGIFGS
DAGPNPQGPNSCSDRGCDQGTLAMGWGSGSANYPYLIDPSMAITWYVYSNRPDTMIESVLDDYDYASINSVASQADICLAFVNADSGEEYITVDGNEGDRNNLTLWHSG
EAVINASTSYCSNTIVVVHSVGPVLLEEWIDNPNVTAVLWAGLPGQETGNSLVDVLFGAVSPSGRLPYTLARQRGDYPADVMYESNMTTPQITYYERLMIDYRWFDAQG
LVPRYEFGYGLSYTSFEYSTLAIYGQGVYERDEANNLDARWTTDTSMAPTATYNPYINQTFPGGPPSFSDTLYTITFRVTNTGSRDGYEVAQLYLGFPESAMEPPKVLR
EFNRVWIKAGERITVSMTLLRKDISIWDVISQSWIIPSGTFTVYIGKSSRDIQLTGTFEQ
>tr|A0A518K9A0|A0A518K9A0_9BACT
MPIHAIPPSAALCRVCFALALGVTSALVANAEDATTFNPAVINPEVERTLGAEDPLAEPVGLPAVEIDLTDGTIDEIAQRVLDAMSLREKIGQMCQVSSFGTDLPKEVA
DDLRGGRIGSLFYTGTPEQTREAKRVAMEESRLGLPLLTPRDVIHGFETVFPIPLGQAASWNPELIEAASAIAADEARAQGVNWTFAPMLDISRDARWGRIAESVGEDP
LLASAISKAMVRGFQQPVENADGTVAYEGIAACAKHFAAYGLTEGGRDYNRAEVSISELHNAILPPFRAATEAACATFMTGFSAVNGVPVTGHRELITDLLKGRWDFEG
LVVSDWTSVIEMIEHGYAADRREAARLAVSAGLDMEMASTTFRENLVDLVESGEIEESQIDDAVLRIIRVKLQFAMPRDDDPLADQTIESRREVAKRLAQQSCVLLKND
GVALPLKTDKLKRVAVIGPLADAARDQLGCWMLDGKPEDAVTLLSAMKETLGDGIEVVHAAGSESSIDDSTKGFDGALDAAEGADVAIVCVGEGWMLSGEARSRADLHL
PGSQRALVQAIAETGTPVVLVVMAGRPLTIGAEAELADAVLYAWHPGTMGGPAIAELLVGDESPSGKLPATFPKQVGQCPLYYNHPNTGRPALPETKALIGSGRADFPE
DQKYRSHYIDVDPFPLYPFGFGLSYTSFRYDSPELTTDSIKPGQTIGVRVRLTNTGDVAADEVAQLYVQDVAASLVRPVRELKGFRRVRLEPGESQVLEFALSTDDLAY
YDNRGEVVLEPGEFRLGVGGDSTVSLSETFTLRGSEVTDEAKRSRLRGAKANVVAPVASN
>tr|A8AEA3|A8AEA3_CITK8
MSRCRLSVFLILLPAQGTLKKSENNLSFLLLSRLGLWQTAVFVIFLLPRRAKEKERIKMKWLCSVGVAVSLALQPALAEALSGNHPLTPEARDAFVTELLTKMTVDEKI
GQLRLISVGPDNPKEAIREMIKNGQVGAIFNTVTRQDIRKMQDQVMELSRLKIPLFFAYDVLHGQRTVFPISLGLASSFNLEAVKTVGRVSAYEAADDGLNMTWAPMVD
VSRDPRWGRASEGFGEDTYLTAIMGKTMVEAMQGKSPADRYSVMTSVKHFAAYGAVEGGKEYNTVDMSPQRLFNDYMPPYKAGLDAGSGAVMVALNSLNGTPATSDSWL
LKEVLRDEWGFKGITVSDHGAIKELIKHGTASDPEDAVRVALKSGINMSMSDEYYSKYLPGLVKSGKVTMAELDDAARHVLNVKYDMGLFNDPYSHLGAKESDPQDTNA
ESRLHRKDARDVARESLVLLKNRLETLPLNKSGTIAVVGPLADSQRDVMGSWSAAGVASQSVTVLTGIKNALGDKGKVIYAKGANVTNDKGIVDFLNQYEEAVKVAPRS
PEAMIDEAVNAAKQSDVVVAVVGEAQGMAHEASSRTDIQIPQSQRDLIAALKATGKPLVLVLMNGRPLALVKEDQQADAILETWFAGTEGGNAIADVLFGDYNPSGKLP
MSFPRSVGQIPVYYSHLNTGRPYNADKPNKYTSRYFDEANGPLYPFGYGLSYTTFTVSDVTLSAPTMKRDGAVTASVRVTNTGKREGATVIQMYLQDVTASMSRPVKQL
KGFEKVTLKPGETQTVSFPIDIEALKFWNQRMKYDAEPGKFNVFIGVDSARVKQGEFELL
>tr|A0A255Z468|A0A255Z468_9PROT
MIALPSLRQRARLEQRHREENSVTTPKLSRRLFGASLLALTSAAAVPAGIAFARGKEPYRNPALPIDQRVEDLLSRMTLEEKVAQMIGIWQDKKLVNNDKGGFDAKRAA
QAFPNGLGQISRPGDRVGAVDKDGAPIAAGAKANIINREGLEAATYANDAQKWAVEQTRLGIPLIFHEEALHGFVGRGATSFPQSIALASSWDTAMVERIFAIAAREAR
LRGVHLALAPVVDIVRDPRWGRTEETYGEDPWLVSEMGLAAMRGFQGTTLPLAKDKVLVTLKHMTGHGQPESGTNVGPAQISERTLRENFLVPFERAVKNLPIRAIMPS
YNEVDGVPSHGNKWLLDTVLRQEWGYQGALVSDYFAIREMKTVHQMTSGLADGAVRALNAGVDVELPDGEAYPLLPGLVREGKVSEAQIDTAVRRILRMKFEAGLFEAP
YVDAKVADKLTATPDAIALAREAAARSMILLKNANNALPLNPNATGTLLVVGTHARDTPIGGYSEVPRKVVSVVEGLTAAAGKSLKVEYAEGVRLTRQRVWEEDKVEPI
PDAENDVLIAEAVAKAAKADTILMVLGENEQLSREAWAKNHLGDRASLELYGRQSDLAEAMFKTGKKVIILLLNGRPLSVNRLVEKADALIEGWYLGQETGNGVADVVF
GKVSPGGKLPITIPRSVGQLPVFYNAKPSARRGYLFDDITPLFPFGFGLTYSTFEMDAPTLSRGQIGVADSVDISVTLRNTGARTADQVVQIYLRDEEASVTRPLMELK
RFQRVTLAAGASSTVRFTLTPEDLSIWNIDMKRVVEPGVFTISAGFDSVALKSVQLTVV
>tr|A0A2G5C560|A0A2G5C560_AQUCA
MAAASILQLLFSLHFLFVLFAYVKGGPIVVCDTRALAELGYDPIKLHFCDKSLPYQVRAKDLVGRLTLDEKIAQLGDKAEGVKRLHIPAYNWRSEALHGVSDVGFGTHF
DDTIRAATSFPTVLLTAASFNETLWKAVGEAISDEARAMHNVGLSGLTFWSPNVNVATDPRWGRIQETAGEDPFLVSRYGVNYVRGLQDVKGYINVPDKNSRPLKVAAC
CKYYTPYDMEKWTADPTKKTDRYHFDAQVPDRDMLETYNYPFEMCVKEGDVASIMCSYNRVNGIPTCADPKLLNSTVRGLWGLNGYIVDDCDWVVATVLIPERYRNDTP
ADAISQAFRAGVDLDCRGNYSPHYMMDAITQGKLSEAEIDHALINLYVVLMRLGWFDGSPGDYLHLGQNDVCSKENLELAAEAARQGIVLLLNDNSLPLPLNYSTPDGS
GRKPTVAVVGPHANATTAMLGNYALKTGTACRYVTPLDAIQGYANVKYAPGCLDVNCEDTSGFQEAAKATDGTDATFLFLGLDLSYEDEDSDRQNLYLPKNQIALLDAV
FKEPYPSLLVIVVLSGGGVDLSDWGSKVHAILWAGYPGAEGGQAIADILFGKHDPAGRLPISWYKGDYTNKLPMTSMQLRPNPALGYPGRTYKFCTDDGIVFPFGFGMS
YTTFNYTISAPKSVSIELPETQKCIHIANPTEDCPSILVANSKCTEMVKIVVTVMNIGQRNGAHVVVLFSSPPDNIGGTLPRKRVVGFQRVFIKSGESLPVEFNLNVCK
SFSFISEDANELLPAGDHSIAAGNDFTGGLVFTESAVSTKISIMIHQGTSQTRRIDHL
>tr|A0A257JQE1|A0A257JQE1_9PROT

```
MPIGKATAGADQILSPVTNYGERPLTLTEWPKPHTHLTRDPALESEIAQQLAVMTLAEKVAQIIQADIASITPEEAKQHKIGAILNGGNSAPNGRFDASAKEWLALVDS
FWDEASDSKIPIIFGTDAVHGHSNIAGATIFPHNINLGAARDRDLAKAIGVATAKEIAVTGMDWTFSPTVAVAKDLRWGRTYEAFSCAPSLVSDLGAAMIIGLQGVTDT
KDFLGPDKVGCAAKHFLGDGATQDGRDQGETNCSESQLISQHAAPYIAAIEAGATAIMASFSSWGGQGMHGRHDILTTLLKEHWEFDGLVAGDWNGHGQLPGASPNNAP
QALAAGLDMYMAPDSWRALLASTLELVTGDLNFQARLDDAVLRMLRFKARLGLAQKGRPSKRPLAGRFDLLGCPEHRELARRAVRQSAVLLKNENQLLPLSANQHVLVC
GRGADELDLACGGWTLSWQGGGDFKGLFPGSQTLLEGLKANIIPGGGQVTFDPKGQWQDRPDIAIVVLGEEPYAEFRGDCDTLAYRPGDPSDYQLIERLAAAGIAVITV
LFSGRPLWVNPALNKSAAFIAAFLPGTQAGALAELIVRSPSEEQPDFQGRLPFPWPAFSDHFDLCATSPNRSPLFPLGFGLSLLDHVAWQPLHENSDAKKHDPLTIFDR
GIPQGDWTAELSDAAGSNPIKKPNLTGGTASPAGLVTLRAADFGQQENAWAIHWRDEGSLSFLGPTINLAREANADYALYLTAITENPASLLVGLWPQDGPNSPEAEPV
ISLSGVAKHGSAFVIPLKRFQESGVDMNHVSQLVLYGQGTSGLLLVKAYFAPVVAGSA
>tr|A0A517MQR8|A0A517MQR8_9BACT
MIVPSQSSLQLSILFFVLERTLSTMINLKRSGALSVGLLVLALCVVTFLPNRSLQAETATGPTGPTQATGPTTQEKGSRSDAQSKQDLPRVRINREGLSSEQFVARLLA
EMSIEEKIGQLQQIHPEGDLLAEKVKETIATGGAGSLFFTGTEQLVHDAQRVAVEDSRLGIPLIIARDVIHGFRTVFPIPIGQASSWNPELVEQAAKVAANEASRVGIH
WTFAPMVDIARDPRWGRIAESCGEDAVLASALGVAMVKGFQQPDEDGNLQGIAACPKHYVAYGRAEGGRDYNRVMVSRNELRNVYLKPFKGCIDAGAITIMSAFNTVNG
VPATGHERLLREVLKDEWGFDGFVVSDWDSVKEMISHGYSADKRQAATQAMLAGLDMEMVSNCYQENLQQLVEEKTIDIAMVDDAVSRILLAKVKLGLFDKPYADTNQP
ELLCDAHLAVARELAQQSVVLLKNDGVLPLEKAALKKVAVIGPLADSPQDQLGTWTMDGLASDSRTSWAALQEMLHEGVQLTHTPCLQTKYREAPEEIATAVEAAKAAD
VVLLFVGEEEVLSGEAHSRSELSLPGSQGKLVHALAELETPVVMIVQAGRPLTIGKEIEAVDAVLYAWQQGTMAGPALADLLLGVVAPSGKLPVTFPKAVGQIPLYYNH
MRTGRPSAADYTPPTLDKVKAMASSTRYSSHYIDSDPYPLFPFGFGLTYTQFEYRELEISNDSIGKDQVLVARVELKNTGNRAATEVVQLYTRDLVASTVRPVKELKQF
RRVTLEPGEETIVELSLTYDSLGFYSEEEHYQVEPGKFEVSVGGSSATSLSKSFSVK
>tr|A0A4V3SID7|A0A4V3SID7_9PEZI
MTLLEKVNITSGIGWEQGLCVGNTAPAVHVNFPALCLQDGPLGIRFADNATAFPAGITVGATWNRELMRKRGELHALEAKAKGVNVLLGPSMGPIGRTPLGGRNWEGFG
SDPYLQGVAAAETIKGIQGAGVIATAKHFILNEQEHFRQIMEWEKSRDEALSSNVDDRTIHELYLWPFADAVRAGVGAVMCSYNRVNQTYACGNSDILNGLLKSELGFQ
GFVMSDWFAQRTGFHSVLSGLDMDMPGEVTIFERGQTLFASNLTTSVMNSSIPLERLDDMATRIVASWYQMGQDDPKNFPVREPNFSSWSRERTDVQFHGASEGPEVVV
NQYVDVREDHDIVARQVAAEGVVLVKNIDAILPLSRSSLKKAKLGIYGEDAGDENQPGFPNFCTDRGCNRGSLAMGWGSGAVELETFSSPQSAIHKELPNTKITAITDN
TRLEDISKSAEEQDICIVFINSAAGEGFITDPVTGAQGDRIHLYPQKNGDELVKTVAESCEAAIVILHTVGPVILDRWISHPSVKALLIAHLPGQESGSSLTPLLFGDI
SPSGKLPYTIAKQPADYGKAAAILEEPNAIVPQQTFEEGLLIDYRHFDYYNITPQYEFGFGLSYTTFNVKNINIIPVLEKSEFPPPPPALLPAPKLNSTLPKPEEVVFP
KGWRKIRNYFYPYVNSVDDINSPTPLDPPKGYTTKPHPPSPAGGGEGGNPSLWDVMLEVEFTLSNTGDRPGKEVVQLYVEFPALDGEEEVRGNTIGKTPKRVLRGFEKI
ALGPGEQTTVKLPLMRRDLSYWDVEAQNWRMALEGKYRVGVGASSRKFWGWVEY
>tr|A0A5E7Y705|A0A5E7Y705_9SPHN
MGEGIMKGRISTALLLAGAAFTTIMGPLAPSAAAQAFTEQEVAAMDRPMADAPYWDASLPVEARVEDLLSRMTMEEKLAQMISTWTNKGEIQNEENFFDPSLASERYPD
GLGFFTRPADVGGPGSPRVTPFRSIEESVAYINAVQRWAREDTRLGIPVMAHEESLHGLAALDATSFPQAIGLASTWNPDLVREVNDLIAGETRARGIFQVLSPVVDVA
RDPRWGRIEETFGEDPFLVGEMGVAAVEGLQGVGNDRRLGEGQVMATLKHMTGHGQPESGTNVGPAQISERTLREAFFPPFLEVIRRTGIDAVMASYNEIDGIPSHSNV
WLLDDVLRQEWGYQGTVVSDYFAIEEMVTRHRLAADNAGAAEFAINAGVDIDFPDGAGFTNLAALLADGSITEAQIDAAVRRILTMKFNAGLFEAPFVTDLAAAQASNG
PEGVALARRAAEESLILLQNDGTLPLALPAASPQGSADRPTIAVIGPNAAVARLGGYYGIPRATVSPLEGIQALVGDRAEVIYSEGVRITVNDDWWEDEVVLADPEENR
RMIAAAVEAARGADTIVLFIGDTEQTSREGWAETHLGDRTSLDLVGEQNELVAALADLGIPLVTVLVNGRPPSYPLVAERSNAVLETWYAGEQQGNAIADALFGIVNPG
GKLPVTVARDVGQVPVFYNHKPSARRGYLFAEVSPLYPFGHGLSYTTFEFGAPILLDTTIAPGEGVTVRVPVTNTGDREGDEVVQVYLRDVVSSVTRPVKELVGFERVT
IAPGETATVDITIRPEAFMLWNRDMQFVAEPGEFTIMVGPNSVDLTEVSLELAE
>tr|A0A062U1S8|A0A062U1S8_9PROT
MTMRTDHSVGRLPSPQQSETDLNLDIWPKALSPLRHNPETEARIEEILLRMTPAQKIGQILQADIASITPEEVRAHHIGSVLNGGNSSPDGKMRASPSEWLALADAFWE
ASMHTDGPQIPIMWGTDAVHGHNNLAGATIFPHNINLGATRDADLVERIGSATAMEVRVTGMDWTFAPTLAVTRDDRWGRSYESYSECPNLVAELGAAMVRGLQGGANT
SEFLNADHVIATAKHFIGDGGTQDGKDQGETIATEEELRDVHAAGYYTAIREGILSIMASFSSWKGQKMHGHEEFLTRLLKEHWGFDGLVVGDWNGHGQIPGTSTTNCP
DALNAGLDMYMASDSWKGLHASLLQQLQKGVVNEDRLDDAVRRVLRLKFRARLFNQPKPSERKHAGDYALIGCQKHRALAAEAARKSAVLLKNAESTLPLSPGQNILVA
GKAADDLSMLCGGWTLSWQGEGIRKSDYPQAETLLQGIKRLAEAYGGNVEYAPDGAYRESPDVAIYVFGEKPYAEFRGDLTTLDFKPGDRTDLQTIQKLKQSGIPVITV
FLSGRPLWINPELNSSDAFIAAFLPGSQSGAIADLIFQDSNASHDFDFTAKLPFSWPEHADQYLLNHGDADYLPLFPYGFGLSLGDTCTLGRLHENSLATEPDHGTIFD
HGAPQGSWQIVLTDSAGQSHWKGRSIASPAAELHIHPADLGQQENAIRACWNCTNTATLAFLHSPIDMSREANADFYLKVVLANAPNLISTTQMSVITHSGKVRIGNLL
DMHRQEETELTLTVTIPLKKFGVAGADLTTLKGIELCNSEGGQIVVSYIGISTL
>tr|A0A176VUR7|A0A176VUR7_MARPO
MAAWRYLAVFFVFHHIIQIFQTFVVHGTALHGCGSDLDASILAMPFCNISIPIADRTLDLVSRLTLQEKIAQLVNTAPEVTRLGIPRHEWWNEALHGVAYSPGVTFTGP
ASGATSFPQPILTASSFNMTLWTLVGQARGFLIRVPQAISTEGRAMYNLGQSHLTFWAPNINIFRDPRWGRGQETPGEDPLLTSIYASTFVRAMQEIRYSDEGSRYKAP
FGVQMVPSELDTTEERSPAVGRMLKTSVCCKHFTAYDVENWDGIDRYHFNAEVSDQDLEDTYNRPFQSCVQEGQASSMMCSYNRVNGVPTCANYNLLTETARNTWGLNG
YITSDCDAVELIYSAVNYAPSAEDAVAEVLMAGLDLNCGSTAANFGQSAVDQGKVNESTIDRALFDLFSVRMRLGEFDGDPENQIYGSLGLDDICSQYHKELAIEAAGQ
GIVLLKNDNNVLPFLRGHIQKLALTGPNANATSSMLGNYAGVPCEYITPLQGLQTYVDLLLYEPGCWDISCQDGGLIGAAAAIASSADAVVIVAGIDQTQEQETLDRTS
LLLPGQQQSLIETVANSSKGPVILVIMSGGAVDISFAKFNPQIHGILWVGYPGQGGGQALADILFGHRNPGGRLPVTWYPESFTQVSMTDMNMKPNTSSGYPGRSYRFY
TGETVYSFGDGLSYTNFLSTITSAPSRFLIPSLEQQRCYHNFNSTSKCPKGFQNELTCDTASFEVRVSVSNQGVRRGSETLMLYSVPPKSGIGGEPLKQLIAFQRVSLD
AYSSMDVGFEVLPCKHLSSVKYDGTKELVEGTHTLVLGSKDEVSHAVSFVTNFF
>tr|A0A0G9MSF5|A0A0G9MSF5_9SPHN
MRAVISTAALALGLSACAAVPADGPATAAIESAAPTAMSEEDAVIADLVSRMSLERKIAQLIQPQINSFTPEDMERYRFGSYLNGGNGGPYGDEFAPASEWLRYADEMY
DASVRPLPNGEPVIPTMWGTDAVHGHTNVVGATIFPHNIGLGATGDADLVRRIGHATAIEIEVTGIDWNFSPTVAVAQDDRWGRTYESYSEDPDIVARMGAALVEGLQG
NPQADDFLGTGRVIATAKHFFGDGGTEQGVDQGDVNGDIDELLAVHGRPYPAAIDAGVESVMASFNSINGRKMHGNEMLLTDVLRGQMGFDGLVVGDWNGHGQVQGCTV
TDCPQSLLAGLDIYMVPDDWRGLMENLIAQVNDGTIPMARVDEAVTRVLRVKYRAGILGADAARPSERGVGGQFALLGSPQHRALAREAVAASQVILTNDGILPLAPGM
DVLVAGSAADDIGQASGGWTLEWQGGLEGEMGRSAGIADAIFPGATSIWDGLEMAVTTGGGTATLSEDGSFTTRPDVAVVVFGERPYAEFAGDRPDMAFRDEEGLNLLR
GFREQGIPTVAVFLSGRPMWMNREMNAANAFVASWLPGSEGAGVADILTGAVQATGRLSFSWPNDCIGRPLNQAEGALFAVGYGLSTRETGNRGELGESCSLLEERDGA
DLFTSGRLATGVNAYAAGGALTIADDVLIQMRGSSSPAGVTMRGIDRNAQEDAREIAMQPGARFGIRQDGTGSGGYRIAYRVEERPAGPVTLIVGDGDTATRLDVTMQF
QNAAGKGWREMVISEECAPGLGQRIEFATGSQLTFAISSITREELPEGTACSF
>tr|A0A1Q3ESS5|A0A1Q3ESS5_LENED
MAMMNSTNMPTAGTQFADTIKVLVALLAASPVFTYSFERSIETTLGGHLFRRDTVDYGNSTLWPSPAANGGKAWTKAFAKAKAVVAQMTVEELFNLTVGLDGRCSGNTA
PVPRFNIPEFCFNDGPAGVRDVEGVSQFPAELTTAATWDRDLMYRRTKAMGQEFYDQGVHIPLAPVSGGPLGRSPLEGRAWEGSFTDPYACGEASYQYVTGLLDSGVAS
VAKHWILYEQELNRNPHGLQTKSDTFPGGTQLPVSSNVDDKTMHEVYMWAFAEAVRAGTTHFMCSYNEVNNTHSCSNAKSLNGLLKTELNFQGPIMSDWGAHWDTVPSM
YNGMDISMPGSQLNSLLGDFWANLTELVNNGTVKEDVVREKVIRILTPYYHLGQDVKPLPDFVYNLIGPPYVNVTAGYRNVRKSGTADLIKEIGMSSVTLLKNTGGLPL
KNPSRVAVLGNDATDNILGANSCGATNSFCAINNLNGTLTIGFGAGSGYSPYIVTPLDALKARAIQDNHEIQAVVADSTSATATAAIQALLPLSDVTLVFVNRWYGRGM
DAESLNLTGDADDLVAQAVNSSSNVVVVIHATGAVNIEKWADNPNVTAIIAAYLPGQESGAGLVPVLYGDVSPSGKIPWTWGKSLDDYPPNGIVTDLVYSPQSNFTEGS
FIDYRWFDKNNITPRYEFGHGLSYTTFKYSQITISKKTSKDTTSVMETAEPFVGRDSSNSLYDILFTVSAKISNTGQYTGSEVAQLYISIPEEGQPIRVLRGFDKVKDI
KPGASATASFPIRRKDVSAWSTVGQHWYVPNGTFTIFVGASSRNLPLSTTWTP
>tr|A0A5N8W024|A0A5N8W024_9ACTN
MPTPDHAAAPAEQRPATSTPPYRDPTAPVGTRVRDLLSRMTLREKAGQLNQRMYGWDAYRRTPDGDFELTEALYAETERFAGLGALYGLLRADAWSGVDHATGAGAAHG
AALADLVQRHVIASSRLGIPALFVEEVPHGHMALDGTVLPVNLAVGATWDPDLYRRAAAHAAAELRARGGHMALVSALDIARDPRWGRTEECFGEDPYLAARLTEALVH
GMQGEAGAAGKAGAAGKAGAAGKAGAAGKAGAAAEVRDGLFADDKAPVVLKHFAGQGATVGGRNSAESELGLRELHEIHLPAARAGVRAGAAAVMAAYNEVDGMPCCGN
RALLHGLLRERWGFEGLVMADGLAVDRLARITGDKVSAGALALHAGVDLSLWDEGFTHLEEAVGRGLVGEDAVDAAVARVLSLKFRLGLFERPYSADRSAQAQAPSPAH
GRALSTALARGAVTLLHDDGDVLPVRPTVSRIAVLGPHAATTAHQLGDYTAPQRPGTGTSVLEGLRRLAPPGLAVRHARGSALTGDDLSDIPEAVAAASAADLAVLVLG
GSSARTVDTDFEANGAARTAVSTMTCGEGVDLAGLRLGRAQHALLDAVTATGTPTVVVLLQGRPHAVPEAADRAAALLTAWYPGPWGGDAIAEVLFGHAEPGGRLPVSV
PRSVGQLPVHYNHKDTEYGSYADESARPLYSFGHGLTYTTFAYGPPRLVRHTVEADITNTGRRRGRTVAQLYVRRLLTPVWPRTLELRSFQGVELAPGECRTVTFPLGA
GQLAQVGADLETAVLPGTLEIRVAQSSRAALTAVPLVLRTGGQNFTAPDETPL
>tr|J5KAL2|J5KAL2_9GAMM
MKKILILLISFNLSAQIDWSNDNTCNISSNNEMIDLIVSNMTLEQKVGQVIMPDIDDVTPQEAKDYFLGSILNGGGKFPNKNKYSSIDDWKQLSQEFYNASPIVNEKII
PILWGTDAVHGHNNVIGATIFPHNIGLGAANNTDLMEKIGSAVAKEVLSTGIPWTFAPTIAVPQDSRWGRTYEGFSEDPQIVSDLGEATIIGLQGFGDNFLGDYKILAT
AKHFVGDGGTDKGVDQGNTITSEFDLKETHGFPYYAAIDACVQTIMASFNSWNGDKMHGSSYLLNDVLRDQMGFKGLVVGDWNGHGQLPSCTNKSCPEAFNAGVDIFMV
```

PQDWKELYKNTLDDVKNGTISTARLDQAVKRILQVKYNIGLLSGKKHYEFSENFVGDSNHRLIARQAVRESLVLLKNNNKTLPIKSNKHILIIGQASKEIKYQMGGWTV
SWQARDTVNTDYPNTKSIFEELSDSLASIGSTSEYSIDGSYKKKPDAVIFVYGEQPYAEGDGDRENFFYMPEDKNLINTMNNFKASETPTISLFLSGRPLIVNEELNAS
DAFVSLWLPGTAIEGISDVLLSNKDDSINYDFVGKLSYTWPKFNNAEKKNDINLFNFGYGLTYQDNIYISELEVEQKYIKKDEIIAFLGSAYPPSAEVLNINWNYEKVT
SNIVNDPDYGVSLSRFDYKKQDDARKIIFKPSKNYTEWMISSVIEEDISHMEQGQIEIELKVNKSSIEPLLFAMACTKNSIEINKSQSSICKTEIALSSLFKETQEWQK
VYIPLSCFKNKDFNLSKVKERAILRTNGSWAIDIHSIKYLNNKNSQDCNLQKL
>tr|A0A4Y8UPN5|A0A4Y8UPN5_9GAMM
MFCRAPHFFAAVVCSLLPFSPSWAEQNDTELLALIAGMSTEQKVAQMIQAEIKYITPEQVVEFGIGSILNGGGSFPQSNKYATVQDWVELADAFHAASLDRSQGSAGIP
IIWGTDAVHGHNNVIGATIFPHNIGLGAANNPRLVADIAAATAREVAATGIDWIFAPTVAIAKDDRWGRTYEAYSDRSDIVGRYAGPVVRAMQQQNIAATAKHFIGDGG
TFRGIDQGDTRLSLEQLLLEHGIGYSEAIAADVLTVMASFNSWNGEKLHGSYDLITTILKEGLGFDGFVVSDWNGIGQIPGCSNESCAQAINAGIDMIMVPEDWQPFLT
NTLAQIERGEIAMSRIDDAVYRILSVKKQLGLLDGAAPSSRHSAVGSGVIGSAKHRAIARQAVRESLVLLKNSNGLVPLSPQQNVLVTGLADDIGVASGGWTISWQGTG
NSNADFPGGTSIYAGLAAQIEAAGGTASFSANGDYTTKPDVAIVVFGEQPYAEGQGDIDNLQFDAGSKQGLAQLRKFKAEGIPTVAVFLSGRPMWVNAEINAADAFVAA
WLPGTEGAGVADLLLRDRAGQIQHNFTGRLSFDWPNSDVNASDDNLPVDDILFPYGYGLAAAQPHSQPLQALNEQPTVAAVASEVVVFERGTKSPWRPYVGDASDWRKL
YSGGVVASQYGDLQVSAVDIAVQEDGRKLRWSGSGARLSQFYWQADQGVDLTQLSEQRAQLVVDYQLQRAPSQPVALRMDCGYPCSGAVDITQSLNSAAVANWQSLAVP
LSCFAAQGVDLSRVNTPLLFSTSGMLELTLRRVAITAEPPVGEAAQVLSCQ
>tr|A0A316UCK7|A0A316UCK7_9BASI
MRLLTHASLLFLAAGSLATAAPLDDDPTLLLRDWASLSPLRRQQQDNNATAGSYTGPYASGGAWTDAYSQAKALIAQMTIEEKVNITTGHSGPCAGNSGAVPRLGISPL
CLQDGPTGVRPARRTSQFPQEVTMAAAWDRDLAAERAQGIAEEMHDKGVNFWLGPVTGGPLGRSALAGRNFEGFSPDSYLSGENSFASVRAAQEVGVVTVAKHFIAYEQ
ETFRDAYSPNQTVIDASGENYQRVLNQLPIDSQLDDRATHEAYMWSFAEAVRAGTGSVMCSYNRVNDTHSCSNSYAMNELLKGELGFQGSVVSDWGGTWSTIHSALAGL
DLTMPGQGYGGIFGDFFDGSLIDGVQNGAVPESRLDDMVLRILGPAIHFQGNFTESYPAPTFDINDVNGPTNNVRRDHYKVIDRIGQESVTLLKNNRTLGNHYGLPLRN
KKDISSIAILGEDSGPKIHGYTSCGDFGTACAFDTPPGQNGTLSAGGGSGWSYPPYVSDLLAAVSFEARSGGFDINSDTDSYNLAGAAAQAAKSEVAMVSVNAYAREGS
DRQNLTLFGGGDALIEAVAAVNNNTIVVIHAPGPVLLEEWVDHPNITSVLFAYYPGQEAGNSLIPVLWGDVSPSGRLPFTIAKSADDYPDIQRNYSLDPSVYFEESTDI
DYKLFERRGVEPRYHFGHGLSYTTFSFDSLSVQYNYSADSTSMQKTNEHFDATYGQGDSVYDQLLTASVSVTNEGSVAAKAVPQLYVTLDGVHNLRGFEKVLIQPGETV
KVHFSLRRKDVSRWSVKRQEWYIPDGDLLFQVGKSVGHLSTKATFAFTANQ
>tr|A0A420XZJ0|A0A420XZJ0_9PEZI
MMRTTLALASLAVVAMAQKPAPGTDTFPDCKNGPLHNTSVCNMDLDPTTRAQALVNLMTAAEKFNQTGNNSPGVARLGLPPITYWNEILHGLATNGGAIQFNATGNFSY
SSSFPHIVNLGYTFDDELMTAVGNALSQEGRAFSNAGRAGLTFFSPNVNPIRDPRWGRRLEVVSEDAFYTGNYAKAMVSGLQGGVSPKYKRVVATCKHVFGYDQENNAG
NWRYQFDANISTQDTAEYFMTPFRACAEANVGAFMCSYNAVNGVPSCANSYILDDVIRGHFNWTNEDQMVFSDCDAIQNVYSPHLYSPTRPAAAADALKAGVDNNCGEY
YQVHLPEAYAQGLISDKDIDTTLVRTYGTLIRLGYFDDPDAQPLRQLGWSDVNTPDTQLLAYQAAVKSIAMLKNDGTAPVDLKGKQVALIGSWANATALLQGNYFGPPP
FLISPLAAAMNVSGIGGVHYANGPNQGNPTTSGLGKTIKTANMSDIIIYIDGLDPSDETEEYDRNTLSWTGYQLDLIGNLAELGKPMIVVRMGGGQLDDSPLLKNPNIS
ALFTAGYPGQSGGQAIIDVLTGVAAPAGRLPETQYPSSYLNTVRMSDMSLRPSETNPGRTYRWYTGEPILPFGYGLHYTNFSVAVAPQAADAASSKLVAGGASFAIQDL
VKDCDKVQHTTLVTNELLACPFTSLPLNITNTGKVMSDYVALQFVAGDYGPKPRPKKSLVAYGRAHNITGGSSAAVNLPAVTLGNLGRVDENGNTILYPGQYSFMVDIE
PRVTINFTLTGDAVTLDRWPTPPPVGKPSGVEFIDKDYFLGEYGSIRAQPS
>tr|A0A4Q1BWH6|A0A4Q1BWH6_TREME
MWGILNAALYLLPATITKWHTEDQQTHYEPLRPTYRGPLADGGLAWTQAFKMARDVVANMTILEKVNLTTAVVGPCQANSGGVPRLGIPGLCFNDGPSGPRYTDFVTQF
PSPFTAAASFDRDLIEERAIRIGGEFVGKGINVELGPITGGPLGRSPFAGRNWEGFSPDPYLSSTMSFLTVRGMQSSGLITCAKHYFLYEQEPVCTGPVVDGGRTDCHD
VSSEVDDKTVHELYLPSFAEAVRAGTGSVMCSYNRINGTASCQNDDSMNRLLKDELNFQGFDFGAAHSTVPSALAGMDMELPGEFFCKSSGILTRTMLIIQDGRRLYDA
VLLGEVPTSRLDDMAQRILTPYFAQGQYREYPAVNYQKFNLRDSVEVHGMTFRNLHINNKGDNHLLARKIAAESTVMLKNSGVLPLNGVKRIGIFGTDADYPATMSGCG
PDLFCGVASDRWYWNGTVTIGGGSGAAYADYIAAPLEAISLRARREGARVDYVAHDDPAHFGAMSWIASQSEVCLVFVSLFLVESWDRENLRLDKGGEELIQHVEGSCA
GEVVVVLHIGGQVVMEDWIDLPNIGAVLFAGYPVGQETGNAIVDVLWGDVNPSAKLPFTIGRMASDWPTGNIVRDMSQSTKDIPHSPPNGKRETYPRSVFSEGTAIDYK
WFDKWSIEPRFEFGFGMSYTSFELSYLSLSEEYRPQSDTIQNTNERYEGSADLYDILYVARVKVTNIGKVAGAAVPQLYMSFPSSEPDQPPNHLRGFAKPSLIPGQATV
VEFPLRKKDLSVWDVERQLWRIPNGVFIFRVGQSSRDLPLLLESRIKSR
>tr|A0A2V1BG70|A0A2V1BG70_9HELO
MQFLTSLLVLTSTLLAHASVVPRGRASDQYVPSIQEWDDAQAKAAAFVAQLNTTEKTIMVTGTLLDVGTGCIGRIRPVNRLGFPGLCLLDGPNAVNRAHLVSIFPSGIT
IAASWDREMMFDRGLALGREFKAKGAHVILGPTTGPLGRNPLGGRNWEGFSPDPYLSGEAIKFTVIGHQTAGVQTSSKHYIANEQETMRSDTKMPDGTVVDAVSSIIDD
RTLHELYLWPFADALKAGTTSIMCSYNRLNGTYACENEHMLTEVLRGELGFRGFVVSDWFATHSTAKAANAGLDMEQPGDLPPGVTTSHGGGGYFGKSLEAAVTPGNVT
EERLTEMAVRLMTPYYILQQDSPSYPTTDPSMPYLLGISNGGWDSPIVAGLGVAMPGRDVRGNHGELIRKWGASATVLLKNENDLLPLGDKTSCGGKPITNIGVFGNAA
FDLAEGLVNPPNTPQEGPDIGVLTIGGGAGSGRNSYVVSPLEAIKEQAKQNNAIVQYIGENDIIARNDFRGIYPRPDVCLVFLKSWAAETYDRTSFELSYNSTLVVNNV
AKLCQGQTIVITNSGGTNTMPWSNNPNVTAILATHYPGQEVGNSIVDVLWGKTEPSGRLPYTVPKAAADYDIPVVNLTGPDGGVERNSSKWQVDFVEKQLIDYRHFDAK
NIEPLYPFGFGLGYTTFKVEADVEVRKLATGNLTDLPIAAAPIEPGGNSDLWTELLHISARVTNTGRRAGNTVVQLYMSFPESEIMKEAPVRVLRGFEKVNLRAGQSGN
VNMVLKRRDLSYWDVTVQQWRIPSGDFTFSVGLSSRNLPVNTTARILG
>tr|K6FF82|K6FF82_9GAMM
MKKFILLTMFLIQATSLLAEVDWSRENSCKYNNFSNADLIDQLISDMSIEEKVGQVIQGDLDFITPADVKKFKIGSVLNGGNTAPNGDKYSSLSDWKKLSQEFYDASPT
YKGIKIPVLWGTDAVHGHNNVIGATLFPHNIGLGATRNIELVQKIGEAIALEVLSTGVAWTFAPTIAVPQDDRWGRTYEGFSEDPILVSKLGKALILGLQGEGSTLLNE
KHVLATAKHFMGDGGTTNGVDQGNTKISELGLRELHGYPYFDALDACAQTVMASFNSWNGEKLHGSKSLLTGVLKNDMQFDGFVVGDWNGHGQVEDCSNSKCAQSFNAG
VDMFMVPENWKDLLRNTIRQVNSGEISELRLDEAVKNILVVKSRLGLLNGRKPHEFEGNFLGHPDHISLARQAVRESLVLLKNNDGVLPLMPNQHIGVIGDASNKISTQ
TGGWTITWQGRENSNSDFVNTRTIYESIKNYVEVNGGSVEFSSNGKFQNKPDVVIGVFGEEPYAEMLGDLKDVAFTATDPRYLPLLESISATNIPTISIFLSGRPLVVN
EYLNASNAFVAAWLPGTAVEGISDVIFMKNNKINFDFQGKLSYSWPRSKDQSVLNFTDSIYDPLFPYGYGLTYASEDQINNIKIVKSKKLDLVNIFLGAASIPGKEFVV
TDSGPEFVMQDNFLSANKKIKITRFDYQRQDDAKNIIFIKNKGMQAYGISAELPTDLSSMDSPYYEIVMRVNSLSDAKLYFSVGCGNNCMGSIELPVSKMAEWEAINVP
LSCLKKDGLNESKVQIRGLFSSEEQINFDVNSIIIKDGAITGTKVSC
>tr|G3J632|G3J632_CORMM
MLLPLLASLALLHPAVGRQTYLRSEADAYQHSPDVLPSPQISGQNEWADAFVKAKAMVDQMTLEEKVNTRSSLTLLNCTDTVQVTLTGGVDLKSGCSGTIAGIPRLKFP
GLCLNDAGNGVRATDNVSGFASGIGVGASWNKDLARKRGAALGGENRRKGVNVMLGPVVGPAWTVVKGGRNWEGSSADPYLSGVLSAETVRGVQSQNVMTSVKVCWQAL
AVTWEDGALTLLQHYIGNEQELYRAPEGDIAAVSSNIDDKTMHELYLWPFVDLVKAGAANVMCSYNRLNQTYACGNSKTLNGLLKTELGFQGFVVSDWGAQHSFSDAPN
GLDMGMPNSDPFWGANLVNAVKNGTLAESRVTDMAMRIIASWYQLKQDDPSFPEPGIGMPGNLSAPHRIIEGRDPADLPVILQGAIESHVLVKNDHNTLPLKAPKLVSV
FGYSANTPPAWTAATDADGTWRYGLAPVLGLDAATSPIGGRGTMFGGGGSGAITPQTHVSPQDALVARAAKDGFTLRQDLTSATPTTTVDPASDVCLVFGNAWAREGND
RPQLEDAYTDSLVRAVADRCARTVVVLHNAGPRLVGGFADDPNVTAILFAHLPGQQAGDAVVALLWGDANPSGKLPYTVARSAADYGPLLDPQAQGGKSPQAYFPQGVY
TDYKYFERAHVAPQYEFGFGLSYTRFDYADMVVRRAAAPAAEYPSGGVVSGGPSDLWDVVATVKCTLVNGGKVAGAEAVQLYVRFPGMQAKQLRGFEKPVLRPGESTEV
SFELTRRDLSVWNTDVQKWQLQRGSYQVYIGRSSSKLPLMAHLTV
>tr|A0A0D2C7G2|A0A0D2C7G2_9EURO
MLQKELHDNPFFGGAPLPPKTLDVDAVMKELSMQEKIQLLSGVDNWHLKEIKRLGIPAIRVSDGPNGIRGTKMFNGSPAACLPCGTALAATWDMDMIRRGGELQAREAI
AKGVSVILGPTINTQRSPMGGRSFESFSEDPVLAGATAAATIQGIQSKGLSTVIKHFVCNDQEHERMKQDSRVSARALREIYMLPFQIALRLSKPWAFMSSYNRVNGIH
ASEDPWLLGDVLRGEWGFDGLVMSDWTGTYSTAEAIKAGLDLEMPGPSLMRASLVNHALVCGKLSKDDIDICVRRVLQFINRVIPLGIPSHAPERTIDTKETAAELRSL
ASSSIVLLRNEEGISSLSTGRVDYTVGCASGSKPALITNMIPELRMSAYNAPSQDTSRQKLDEMTIKTSDLFFFDYCPPLPMSHHGAWYADITGTLIADVTGEYLFSIS
VAGTARLFVDGTLVVDAASHQTAGGTFFGHGTGEILGRINLVKSQPYKVMVEFGSIATSLLQTHSIESTTGGGIRIGCAYQLNAEEEIRNAVRIAKHVDQVAIIAGLNS
DIESEGYDREEFGLPGHADRLIREVAAANPNTAVVVQSGSPVAMPWMQLAPAIVQAWYGGNETGNAIADILFGDVNPAGKLPLTFPVRLEDTPTYLNFGSERGRTLYGE
DVFVGYRFYEKSNRRVLFPFGHGLSYTEFKIGKLNIYTSEKEDTITVSISVQNLGSRAGAYVAQVYISQRQPSVARPIKELKGFTKVFLESGEEKTGTISMSFKYATSF
WDEQKNAWAMEKDTFDVLIGSSSYDKESLSTSFEVSENKWWSGI
>tr|A0A1M5YBR7|A0A1M5YBR7_9GAMM
MKKKLLSALITTALVAGVAACTPQANAEQQGAKGNVTAAGVALNPHPSPVPASVQHWNDDKAKMDSYIDELMAKMTLKEKIGQLDLQTSFRDLTGPSINENYEQQIKDG
HVGAVFNAYEPDFNRTLQQMAVEETRLGIPMLFGYDVIHGHKTIFPQSLGEVASWDLDVAKEGARVAAREAAADGVMWTFAPMADITRDPRWGRISESAGEDVYLNTLM
SVARVEGFQGDDLAAPDTVLATAKHFVGYGLAQGGRDYHTTDLSEHELWTTQMPPFKAMVDAGVATFMTAFNDLNGIPATGSKFLLTDVLRDQWGFEGFVVTDYTAINE
LVPHGYARDDKHAAEIAFNAGADMDMVGRVYLDYMEELLEEGKVSIEQIDTSVRRILEMKWRLGLFEDPYRYFDNARAEQEILSEQNLAIARDAARKSAVLLKNEDQAL
PLQLDSLESIALIGPLADNKRDMIGNWAAAGDRKTVPVSLKQGLAERLGDEVTIHYAEGATYNFMVDAGFAASRADHMVGYNDLSQQQLDGMIAEAVSAAKQSDVIVLA
IGEDQRMSGEASSRVELTLPGNQRELMTELKKLGKPMIGVVFSGRPNDLSWEQQNLDAILHAWYPGTAGGHAIADLLVGDYSPSGKLPITFPRSVGQVPIFYNMKNTGR

PYNADNANQRQEHYKSRYDDSPNTPLYAFGHGLSYASFEYGELTLSSETLTMEGELTVSVEVTNRGKHAGEEVVQLYTRQLVGSVTRPVQELKGFDKVHFEPGETKTVS
FTLTAEDLAFYRADMSWGAEAGEFQVMVGTASDQVQHKGFTLVE
>tr|A0A1R1Y0L0|A0A1R1Y0L0_9FUNG
MKISILAYCLISVNASVRPITVNPDATMPIIEVVGPSSNTKELCLPTDIPVKEFDPMRPEGVESYEIPKFDESEVASPGPIDDDIKALLDSLTVEEKIGQMAQLTPDMF
IGCDGELNVTAAEYMFNTYKIGSILDSATDHGGRWNTASPQRWANLTNSLQKIALRSGSKIPITWALISIKGANFVKSATMFPAPISVAASFNVEVAYNVSRITAKDTR
AAGIQWALNPLVGINAHRAWSRNFESFGEDPYLVGEMTYSTVRGLQGNYKKDRTRLAATMRDFVGYSAPVSGKDRQYRHIPDNILMEYFVPPFKRAVEAGVATAANGFG
SVNGEAVVSSSFQLQHILREVIDFKGVMLVEWEEIHNQVIRHGTAFNQTDAVHLTLNNTSIDMYMTPFNGSFVTETLYLSNNKMLSVDRIDESVGRILQMKKDLGLFEN
PYSDPDLIKTVSSKQDIDMARNAVRESIVLLQNNNDTLPLSLSENVLFIGPATNSSRYMGGGWNMHFMGPSDLEGDAIYDGYSDTVVSGVEKITGKKASWIQGYDIDGN
QVDSIEDIISAAKSADKIVIGLGERTSAEFPGDVNTLKINPDLIKIIKKIHSEAKKPIVLLLIQGRPYLIEDLPSIADSILNANLPSMYGGLPTAEILYGKASPSGRQP
FSYPKADYQNMINYYTYKGDEYDPEFAFGTGIGYSKIIYSDLTVNCTSFSPGNPISISVNVTNTGTMQQKEPVLFYTSQLIRRFYVPEQYRLRGFKKIDLAPNESQIVE
FVLKAEDMSFYGRDLKSTISEGPVRITISAFNKNSVFTDTYLDI
>tr|R9ACK7|R9ACK7_WALI9
MKLSALITLAVALTVFTDARAVETRQEDGLGYMDGKSSTSSNDDMKNTKSANVSDDMYDAYHQSNDYDKYPTPKADGGKAWEDAFKKAKDVVSQMTVDEKGGIVLSHDG
RCVGTTHGVERLGIPELCMMDGPTGVRPVHGVSSYPVGQAAAATWDRELIYQRSKHMGQEFFDKGVNVALAPVASGPLGRSPLMGRNWEGSFADSYSNGIYTYESVTGL
QDSHVSATIKHFIGYEQETARHPYQTDSSTDQEPISSNIDDRTMHEVYMPSFAEGVRAGSGMVMCSYNNINGTTACGNAHAQNGLLKTELNFEGAIISDWGGVHSDVDS
IMSGLDGSFPGVGYGGGFGSFWEDLPELVKNGSVSESRLDDACHRMLTPYFWLGQDGKHTPPEVKFVGNPYFEDGESTYTNVRDPSSTEVLRDLATDSVTLLKNNGGLP
LNRPQRLAAVGALDGTDVSACSDSGTSCNYTMSTITIGGGSGYTISPYEVTPLDAIKEKVIQQGTELSYALENDEDVINEISSRADATLVFVSEWAEEGEDRDDLDIVE
EQAKILDTAVKASSNVILVLNTPGVVDIEKWADNDNVTAIVETYYPGQESGNGLVPILFGEKSPSGKLPYTWGKKLSDWPENTIVRSDDENPQSYFKDGLFFDYKWFDK
QDIEPRYEFGFGLSYTNFEFSNISVEDNHKKDDKEIQATNEPFASYDGSNSFYDTVKTVKATVTNTGDMDSSEVVQLYVTIPSEGEPIRQLRGFEKVKNLKSGESKQVE
MDLRIKDLSVWDVVQQTWVMPSGEYVFYVGNSSRNLPLKVTCSN
>tr|S8F7W2|S8F7W2_FOMPI
MHVAILPLLAAALAPLSLCDVWRRGDNSSTSFNGTSYNGTSYYSTNNNSSSSFNATGETWSAPMATGGSTWNDAYTRAQSLVAQMTLEEKAKFITGHAGRCAGNTGSVE
RLGIPILCLQDGPAGARPIQGSSQFPAGQAVAATWDRELFYARGHAMGQEFFDQGVHIALSPVTGGPLGRAPRDGRLWEGFFADAYGTGEAAYHTVKGLQDAGTMATAK
HLIGYEQETFRDPYNALLDLQLPISSNIDDEATHELYLWGFAEAVRVGVSFVMCSYNMVNNTHACANSYTQNHLLKEELNFQGGVVSDWGAVWGTADFINSGLDMSMPG
SAELDIFGLFWGDTLVLAVAFGFVEESRVDDAVLRVLTPWIASGQADNPPPPVTWNTASILYAANSSYWHDVRQRSTSQLIRQIGAESATLLKNTGGALPLNEPGVLVM
VGSDAGSNTLSPLDALGLESYLLGSVDGTVTLGGGSGWAIPPYVVTPYEAINYRAREQGAQVYSIFSDTDLIDVAVTAASAEVAIVFVRAFRKEGTDISSLSLDNDGEL
LIDAVTAVCNNTIVVIHAGGPVLVESCIDNPNVTAVVLAHYPGQESGNGLADVLFGDVSPSGKLPYTIAHSADEYPPNTIVDTPVINPQSDFTESTLIDYRWFSAHNTT
PRFEFGFGLSYSTFAYSNVSVESVSIPDNTTVQKTNEPFEGSDGSNSLYDVIAQVTAQVTNTGNVTASEVTQLYATIPGSEGIWLRGFDKLKLLSPGETRTATFQLRRK
DLSLWDTEEQLWYIPDADIQLQVGASSTKLYLVRCIAPSFMDCR
>tr|A0A433PM31|A0A433PM31_9FUNG
MQISTALFICALPALVAAEITVHKFHIPPSLRKPGPEFDSPSPVIDKDIKELLNSLTLKEKIGQISQLNINTISYDGWSQYGLNLTAVEYYAKEWGIGSFLNQAQSNDT
WLLSSKEYASWINQINEVYMKYTKHKIPIIYGLDSVRGAHYIRDATIFPSGISMGATFNPSLVEEANSITAFESAMGNVRWSFAPILDLGINKLWPRLYENFGEDPYLT
SVMAAAAVHGFQGKYKSDPNKVAACLKHYIGYGGTRSGQDHDNAWIPINYLLDYHVPAFRAGVEAGAATVMTSYSAVNGQPISSSKYFLETLLRKDMGFNGMVVTDWGE
GYNVYDHHRSAPDRYHAVIDFMNAGIDMSMTPTDTDFNVWLAQAVTEGKVSIETVNNRVGHVLQLKKDLGVFSRPFIDLDSPKFDEVGSMKSAEVALNAIRESITLLQN
NNTILPAKNPKKIAVVGTFREIKLSHSSSNDKGYQCGGWCRRWQGVDNRSGFQEFGNSGPVIDPFLGKGYSLIEGVREVYPHAEISFIEGTDIWGNETAEWQTAIDYAK
AADLTIIGAGEFHYAERVGRIDDLDLPQGLINFINAVGKVSKASALVLFEGRPRTLQDIPNNVDAILMAYLPGPWGGLPAAEIISGKVNPSGRLPYTYPLANGDFTYNY
WRGYDQTYNEWAVVNNTYDGPDGVQNHVYKEFGDGLSYSTFEYSPIKLSGTHLTTSNTLYATVIVTNLGPYDGKHTVLFYTQQHWRTIEPEYKLLKGFEKVDLKVGHSA
KVTFPIVADFFKYTTFDGRPALDSSNFTILIENQSVNFTFTA
>tr|V5EIV4|V5EIV4_KALBG
MIGLKSPVATALLALAAFIGSSSAAPSTPQFFSTTEDIFARDNGAHSYTGPYTTGGAAWSNGFAKAKSLIDQMTVEEKVNVTTGYTGKCVGFTGTAPRLGLEALCLQDG
PAGVRPARRVSQFPAGVTTAATWDRELFAQRAEALGQEFRDKGVNIWLGPVTGGPLGRAPRGGRNWEGWSPDEFLSGVASYLTVKHAQEQGVVTCAKHYIAYEQETYRN
PYNLSEPYDVFPRLQQSPIDSVLDDRTTHELYLWSFAEAVRAGTGAIMCSYNEVNGTHACQDDFSLNYLLKEELYFQGAVISDWGGTWTNQESALGGLDVSMPGTAYNG
MFGDFYGEALIAAVNNGSVPETRLDDMVLRILTPAFELQDDSYPQPSFDVRDLTLPTNNVRGDHHKIIHEIGAESITLVKNNDSNGRGLPLKSLESLQTIAVIGQDAGD
NLYGATSCGQSGQCNINAFNGTVTIGGGSGWAFPPYVITPSAAIQEYVRAAGPDVNLHLDNWDLEGATARANSSDVALVFANTYATENQDRQNLTLWANADNLIKTVAA
NNNNTIVVIHSPGQVDVEQWIEHPNVTAVVFAYLPGQEGGASLPKMLWGETSPSGKLPFTIAKNESDYPPNTIVADPVLNPTAEFTEKLLIDYKWFDYHNITPRFAFGH
GLSYTSFSYSDSVSVSSTPKTDETSIQPTKEKLINSNAGLYDTALTLETSITNTGSKAASEVVQLYVSFPSSADSHDNPQPLRTLRGFEKVKNVQPGETRQVSFELRNK
DIAVWSTLHQGWKIPEGGKLTFSVGSSSRKIHSKATWVYQRS
>tr|A0A3M9Y202|A0A3M9Y202_9PEZI
MVNVTRKLAVLGGIGVATALTDGGHFETVPTYPAPESIGTGWEAAFQKATDAVAKLNLTQKVALTTGATMGLSCNGNISPIEEIDFSGYCLADGPVSVRIADLATVFPA
GLTAAATWDRDLIYQRGKALGAEFRGKGAQVHLGPASGALGRHPLGGRNWESFSVDPFLTGVAMDFSIRGIQEMGVQTSAKHFIGNEQETQRSNTFTEDGLEVAALSSN
IDDRTMHELYLWPFADAVRAGVASVMCSYNRVNQTYVCENSKILNGLLKDELGFQGYVVSDWFATHSGIASAAAGLDMTMPGAMNSAVTAIFPTPSYFGGNLTAAVLNG
TLTEEKVNDMARRVLTPYFYLNQDEDFPSIDPSTGFVFARTYNYPDIYLTGAGMDPNNPPPARDVRANHADIVRKVAAAGTVLLKNTNNVLPLGKPKSVGVFGNGAADV
TQGLTFTGDDSGPWGPNIGALSVGGGSGAGRHTRLISPLFALRGRIEDYGGRVQYLLDNHMMVEDDFTSIYPTPEVCLVFLKTWAREGTDRLSFENDWNSTVVVENVAR
RCNNTIVVTHSGGVNTMPWADNENVTAILAAHYPGQENGYSISDVLFGDVNPSGRLPYTIPKKESDYDFPIVNITGDAARDSAAWQADFTEGQFIDYRHFDARNITPQY
EFGFGLSYTTFEMGSNATFERKCAGISPLPPRTNGTHPGGNPALWKEIGSVTVTVRNTGDVAGAQVVQLYMAFPQASVPEGTPVSVLRGFEKVSLQPGEAKSVTLPLRR
RDVSFWNVVAQNWEIPSGDFEFRMGFSSRDIKTAVTSSFVV
>tr|A0A3S9PE37|A0A3S9PE37_STRLT
MPPLPRRRHPFPALLPALVPGPVLALAAVAVCLAAALSGAPAGAEAGAGVRANAGGPRYRDAHAPVDERVADLLRRMTLDEKIAQMTQIALVKLQGDCGETGGGELNER
CMAAVLRDRAVGSVLSGGGSGPPVNTPRDWARMVDAVQRYAVEHSRLGVPVLYGVDAVHGHNNVLGATVLPHQIGLAATWDPELVRACAETTARAVAATGADWAFAPVS
DLARDRRWGRYYETFGEDPLLAGTLAAAAVRGTENAGSTDTAGGAKNVAATVKHFAGYSEPSNGHDRVPADVSPRYLQDTLLAPYRAAVEAGAKTVMVNSGALNGVPVT
ASRHLLTTVLREQWGFTGVTVSDWQDVRALWTTYKVAADYPHAAALAVNAGVDMAMEPYEAGEFADALRTAVRAGLVPGRRIDEAAGRVLRLKFTLGLFERPYVDAERA
NTEVLGAGRELARRAAVASQVLLRNDKGVLPFGPSVKKIVVTGPHADDLAAQAGGWTVGWQGLPGGVRIPGTTVLEGIRQAAPKGTRVVHAPAPADAVAQGRDADATVV
VVGERPGAEGGADSPRPELPADQRELVRSLKETGRPVVVVVLAGRPLVLGDADGTEGLLMSWLPGGEGGHAVADVLFGAAGPSGRLPVSWPRRTGNEPLYYQQLPGTNA
GAESGHDVAYDFGDGLSYTAYRVESLALDAPSVPAGADVGMTVRVHDTGPRDGEMILPVYVSRPVGDVLSPPRRLVAFTKVRLKAGEARSVRLTVPSKVLAVTPGDIDG
AGPPRVEPGPYVFRAGARTAALEITQGAGEGARPRGKSVRP
>tr|A0A1Y4NMZ2|A0A1Y4NMZ2_9FIRM
MPAERLPYLNTELSVEERVEDLLGRMTLEEKLREMYMHPAGAFAEENDAACFSEEKAETFFQGIGIGAVEAPKCLPRQNAVFINALQKYLKEHTRLGIPALIVSECLHG
YMTPEATVFPQAIGLASTWNKELMRQTASAIAEEAAACGIRQGLAPDLDLAREPRWGRVEETYGEDPCLCGEMGLAYIKGLQGENGTDERRPAEKQWLEEEQHQAEGQS
QAEGQSQAEKLCQAEDQYLARDKIAATLKHFCAHGSPEGGVNLSPVPAGERQLRELYLPPFAKAVREGGALSIMPAYSEVDGIPCSASEFLLTDLLRDELGFRGYLFSD
YGAVEMLNCFHCTAENRAEAAAQAVRAGMDMEAPTGCCFAHLKELLDDGKIDISHIDQAVRRILRVKFLTGLFEHPYVDPEKISGIVHTEEKRQLARRAAQESVVLLKN
DKILPLSGKQRTIAVIGPNADEAELGDYSLGNMGNSSAVTLLDGIRSHVSAETRVLYAKGCGLYEKDGIRDEKAWKDAMDAAQQADVIIAAMGHSSSVDYGIGWGTDTG
KPVTCGEGYDCADLRLSGAQEALLEELLALSKPLVLVLINGRPVTLPCIDKIPALLETWYAGEEAGNAIADILFGEVNPSGKLPVTFPKSTGQIPLYYNYKPSARGYYH
RPGAPGAPGRDYVFDDTEPMFPFGYGLSYTSFSYSDLSVEPAVIPAGNPVTVAVTVENIGSREGAEVVQIYVNDLYSSTTTPVKALKAFEKILLQPGEKKRVSFELKPE
DMALVDRNCRYAVEEGEFRVMTRGLEAEFRVSGACYAE
>tr|A0A6I5YSQ1|A0A6I5YSQ1_9SPHN
MKTAKAAFVTCTALLAMPLQAQTGDKAVPYKDAKAPVSERVADLLGRMTVEEKVGQLIAQSTLPRFTPNSPVTALGVVKKGVVDPVVAQRSLANGAGAFILFETSPVDA
PTGVVTQNAVQSWVVNNTRLGIPMLMQAEALHGVVVKGATIFPQAIALGSTWNPALVRTMFTAVADEASAAGFHQVLAPVFDLARDPRYGRVEEMYSEDPYLVTQMGLA
AVQGLQGNAPGQAWRNDGRHVIATAKHLIHGQPENGTNVGPSDYSEYTMRDVFLPPFEAAVKIGRIGSVMPSYNENLGGIPSHASSWLMKDVLRGEWGFTGFVNSDWLA
VKQLHDKQFVASSYGDAGVLGFNSGLDLETPVPEGFAALPAAVQSGAVKMADLDAAVGRILTAKFNAGLFERPYADPKRAAAVVGSKANAELARKVADEAMILLKNEGA
LLPLDPGKVRTIAVIGPNADKARLGTYSGTPPYFVTVLDGIRKRVGPQVKVVYAEGARISEPDRSADSNRLSPFVAPSAEKDAALIGEAAVVAKSADVVILVLGGNETV
SREAFEAFMPGGKPSLGDADTLELPGRQNDLVREIAKLGKPTAAVILGGRPYTALQVNESVPAVLEGWYLGQETGNAVAGALFGDVNPSGRLPVTIARNAGQLPVYYYR
KPAARLGYVGSDNRPLYPFGYGLSYTSFEYGAPQLNRTTIPARGHATVKVRVTNTGKRAGDEVVQLYIHPKYSGVVQPVLKLAGFRKIRLAPGKSADVTFDIGPDQLSI
LGKDMKRVVEPGAVDIMIGRNAADTQKVQLVVADTDGI
>tr|C6LHW8|C6LHW8_9FIRM

MFDQSGGLKRRSNGIAERKQMVMTEKKIGELVSMLTLEEKAGLTSGKDNWFTKAVERLGVPQVRTSDGPHGLRTQAGKINSLEENASIPAVCFPAACATAASFDRDLLY
RMGEALGRECQSTGVHVLLGPGVNIKRSPLCGRNFEYFSEDPYLAGELGAAFVKGVQSQGVGTSLKHFFANSQEHRRMDASSEMDERTMREIYLPAFETVVKQAQPWTV
MASYNKIGGVYSTANRKYLTDLLRKEWGFEGVVTSDWGATHDRPAAVAAGCDLTMPAEDTDHLIVEAVRNGTLSEEALDACCIRLLKLAFRAAEQHRENIAFDYEGDHA
LAREIAGQSVVLLKNEDNILPLAEEADVAFIGPFAKEPRYQGGGSSHINSFKVVGALEAAEHRGVRAQYAAGCRPDGETDELLLAQAIEKAKQAKIAVVFAGLTDGMES
EGVDRRHMRLPEGHNMLIEAVCAANPNTVVVLHNGSPVEMPWVDRPKAILECYLAGQAAGEAVTDVLYGDVNPSGHLPETFPKRLEDNPSYLYYFGEGGVVNYNEGLFV
GYRYYESKKQEVLFPFGHGLSYTTFRCSDLQLNKKKLTEGEDLTAAVTVTNIGKRAGKAVVQVYVAPEKVEMIRPVRELKDFVKVKLAPGESKTVTFTLQKRAFAHWNP
TVHQWRTESGKYTIQIGENAHDICLEAEVQMEAEPVPPAGGYHIGIPMGEFAKSPKGRRFLVENIIYMIKGMAAAGFIPKEMAAMLEQLPGGVNLAAIDMLAQRAGNAA
GGTGGVQVLLGQPLGMLDGFLPQEKQEELHKLLAELNQ
>tr|A0A1I0R1D1|A0A1I0R1D1_9FIRM
MKRQLALLLVSSMILSCAACGKTPDASETTTMITDTAEPTETSNEIPGYKYELYATMTPEEIVADLTLEQKAAQMVMGAETDTGFDYMGEYGIGSIYGGYGRSMEDWQE
RCDGYMQAAIESDAGIPFIYGQDDVHGVGYCINAVYFPQNIGLGAANDEDLMYQMGLIVADETLICHMPWNLYPCVAQSNDPRWGRTYESYGTDLDRITALSTAYTRGL
VDGGAVACAKHYFGDGNVIYGTGESSGGFDRLIDRGDTDLTDEEIAELVAVYQAQIDAGVQTIMVSYTSLNGMKMHENPDYIWLLKNEMGFEGFIVSDYDGIQGTSPET
YEEQIILGINCGIDLYMEGTRYDEARQIIIDAVNNGDITMERIDDAVTRIIRVKKDAGLFDDPFFENRDTVQTEVGSMEYRAVAEQLVEKSLVLLKNDNDVLPLTEGTK
VYIMGPACDNPRVQCGGWTIGWVESTTKDIEGVTTILEAFERYSEDYGIEVVTDPEEADVVILCVGERSYAEWYGDTEDLELCGMMGLEENRDAIDEAAALGKPTVTCI
VAGRQVIPDQSDYADWDSVVMCYLPGSEGKGISDVLCGCGEFSGRLPEPWYGSVDQILTDECFLEEGYGLSYPDGFEPRTEPVALPDEPAQYDSYEMFTLGTNYTPGTI
EDGVYSSSYFGFSFQIPDGYNEIDSYRPEDLEGGLKPEGTWEPVSTMAGVQIYCFNTSLYLHDGATMDELMAEFWEADFVSTETVTLAGNEWTRYIDGDTLGDQPYPTY
LYVHQIEDDMFVMVMIFDEPGIEHEIDYYDQYFGEPL
>tr|A0A2K1IG41|A0A2K1IG41_PHYPA
MKSTETVILACILLGVMLLTTQLFEKPRIKVYKLQYACDPDGPADLLFPFCNTSISDDDRVEDLISRLTIQEKIEQLVNTAANVSRLGIPPYQWWGEGLHGVAISPSVY
FGGATPAATSFPLPCLSVCSYNRTLWNKIGQVVSTEGRAMYNQGRSGLTYWSPNINIARDPRWGRTQETPGEDPKLSSGYAVHFVKGLQEGDYDQNQPQAVSRGPRRLK
ISACCKHFTAHDLDRWKDYDRDHFDSKVTQQDLEDTYNPSFKSCVKEGQSSSVMCSYNRLNGIPMCTHYELLTLTVRNQWGFDGYIVSDCDAVALIHDYINYAPTSEDA
VSYVMLAGMDLNCGSTTLVHGLAALDKKLIWEGLIDMHLRNLFRVRMRLGMFDGNPSTLPYGSLGPEDMCTEDNQHLALEAARQSLVLLKNEKNALPWKKTHGLKLAVI
GHHADATREMLGNYEGYPCKFVSPLQGFAKVLSDHSPRISHERGCSDAACEDQFYIYAAKEAAAQADAVVLVLGISQAQEKEGRDRDSLLLPGRQMELVSSVVEASAGR
PVVLVLLSGSPLDVSFANDDPRIQSIIWAGYPGQSGGEAIAEAIFGLVNPGGRLAQSWYYENYTNIDMSNMNMRPNASTGYPGRTYRFFTDTPLWEFGHGLSYSDFKYT
MVSAPQSIMAPHLRYQLCSSDRAVMTSDLNCLHYEKEACKESSFHVRVWVINHGPLSGDHSVLLFSKPPSRGIDGIPLKQLVSFERVHLEAGAGQEILFKVNPCEDLGT
VGDDGIRTVELGEHTLMVGMVQHVLTVENWREGHGEL
>tr|A0A099CWP6|A0A099CWP6_9GAMM
MKTPSPLTARRKASLLCAAMGCALTLSAGALAQAQPSGPTHVAAKPSNAQLASPAIERKVDALLKKMTLREKIGQLMQYNDAGCSAPTTANADNPAMAANPTAQCVDAM
KLAADGGMGSMLNVVGAERTDKFQRAAVEKSRLHIPLLFGADVIHGYRTIYPVPLGLASSWDPQLVKDLSRMAASEATTGGVRWFYSPMVDISRDARWGRSVEGAGEDP
YLGAAMARAYIHGYQGDDLSKPDHVAASVKHFAAYGAAEAGREYNTTDMSDIRLRQVYLPPYKAAIEAGSATVMSAFNALNGVPATADPYLLTDILRKEWGFNGFVVSD
YTAIMELTHHGIALTPAEASRKAIDAGTDVDMMSHYYDTQLPTLLKEHKVSMATIDEAVRRVLRVKFALGLFEHPYARGTEVTHAVAAHRPLVRKAAEESFVLLKNAKV
GATPVLPLSSNTKTVALIGPLSDDSNQMTGAWGGAYRQSDVVTVRQALSARMKKLGGTLLFAKGTDIKGNSTAGFAAARQAAEKADVVVMALGESGDMSGEAGSRAHLD
LPGNQQQLLESIVATGKPVVLLVFSGRPLVLDWAAKHVPAIMETWFPGVEAGNAIANVLFGDVSPSGKLPMSFPRAVGQEPLYYNQFPTGRPPGKADLSQPPKGDTRFI
SRYIDVPNSALYPFGWGLTYSSFAYTDVHVSRATLPLAQANRPGAKNLVTVTATVKNTGSRTATDVAQLYVRNLGASVEQPVRSLKGFQRVTLKPGESRTLTFHLGFPE
LSFWNVRSKQVIEATHYTVWVGDSSLASQQASFIVTP
>tr|A0A5C5FPD7|A0A5C5FPD7_9BASI
MLANGLVLLALSTLSYAAPANHLTTRKSSSSASHGGAKARSALTADGFTSQKWIDAFDKAVSYVEGMTLEQKINFTDFQSVPNGCSGLGNPLPDIGISQGICTADGPTG
INSRYSTQFPAEVTVGATFDHDLIYARAVAMGKEYHTLGAHVPLSICIGPMGRSVYGGRNWETFSPDPYLTGEAARLSVQGFQEQGVVGLVKHFVGNEQEYLRVGTPRG
YFPNLPNQTVDSFIDEASLHELYSWPFAEAIRAGSGSFMCAYNQVNGSFACENDHLLNTVLKKTLNFHGWVITGASHWGAGHDTVNSSLHGTDFIGWAREDGHLFGDAL
APFVENGTVPIEVVDDKIIRILTPYFALDQASLPKTDFTRYVGSDYSTETARKVTEGALTLLKNVRSKDNKHGLPLHKPRDLLLVGSSAAPAQSGILHNLGFDYAGAVN
PDFSGYSSDGYGSGGSPAPYNLDPTAAITARGRKEETPVVVDYYTYDNPTEGQTPAVFTGNGTNYFLDTKLAYSNAAVVFVTAMAREGFDRTDLELLNGGSDLIEYVAD
RHNDTIVVITAPGPVDMSRFVDHKNVTAILYAYFGGQEGATAIASTLFGDVNPSGKLPFTIARNVSDYPDNYYNGSITVNPVANFTEGVFIDYKHFDAQEIEPLFEFGF
GKSYSSFEVYDVAVKAKSKKMPASVRETNEKLFVDDKEVSGLYDVAYEVTAQVKNTGSVAGAEVAQLYLTFPSTTPNAMPPRSLRGFSKPHLAPGACEQVTFHVRKKDL
AVWDVTLGGWTLSHGEYKMAVGTSSRKIAETVSVKL
>tr|N2AFG4|N2AFG4_9FIRM
RAKSSSCGTVGYSSLTKEIRNGSNSGKKVKKNEMKEMKCQKRTRIIIKSRFAANILKYGKGKHMKQLTEKQKEQIEVLLKEMTIEEKLGQMNQISPSIVGGFDVSFEEL
IEMMTDGRISKEEFGRMMAGAKQDYHDDVNRAGGLSSVLISDPEVANQLQKIAVEETRLGIPLLFGFDVIHGFRTVVPIALAEAGTFEMDLIEKTGRIAAKESRAVGIA
WHFAPMVDISRDARWGRISEGPGEDPYLASAFAAARVRGLQNDEEDSNNEVAACLKHYAAYGACESGRDYNTTGMALSMLHNTYLAPFKAAVEEGAVTVMAAFNDLNGI
PCTVNKYLLRDILKDMYGFEGFVVSDANAIKECVVHGIAEDETEAAAKAMAAGMDMDMGTWIYSRKLKEAVETGKADISDIDDAVRRILSIKMRLGLFDHPYVTQEQMH
RYDTLPQEHIAHALETAEKSIVLLKNEGVLPLEKSANISLVGALADMPEEVLGSWAIGGRAKDCVTIYQGLQNAGAAVSYHPCCGPEGELDEAQVRMAVEAGDVIVAVV
GELASMSGEASSRADISLPGRQQEMLKMLLESGKPVVALLMNGRPLALSWEKEHLPAMVECWHLGIQMGNAVAHVLFGEVNPSGKLTCTFPAVTGQCPMYYNAPKTGRP
GSKSKFTSRYLDAPVEPLYPFGYGLSYTEFTYSDMDVQETKEHLQVRVLLKNTGSRDGAEVVQLYMQDVCASIVRPVKELKGFAKQYLKAGESKTVTIMLPKYQMGFYD
DQARYHLEDGRFILYIGGNSKDCLSQEVYVRFEITG
>tr|A0A1Z5SM83|A0A1Z5SM83_HORWE
MAAKSLLAASLLFLSGSDAFPRRGHGDHNRPNWQESQITEGQFSDVKQRWDTAIARADELVQQMTLEEVANVTLGQSDTMGCSGLTGSVSRLGFPGICLADGPAGVRGT
TFVNAYPAGIHAAASFNRKLAYERGLYMGGEFRNKGVDTALSVCVGPVGRVATGGRNWEAFGADPYLQGQLGSQMVSGMQLSVTADVKHFLGNEQELFRNPVTNKNNVT
IPAFDSIITDHDIHELYMWPFQDAVHAGAGSVMGAYQQVNGEYSCESSDLLNDLLKDELNFPGFVVSDWGAQYTGWPSAASGLDVAMPNSRGKWDGGNLEMSVRNGTLP
ESRLRDMGRRIVASWFYVNFDDVEGEPGFGLADDGYNFPHDTVEARDPAAKASIYQAALEGHVLVKNDGALPLKTPKILNLYGYDATLPPVLMPNPSGFNDWNFGTSAL
DLEESDNELRGMLFQEIRYSQAATLGNLWVGGGSGGNSPPYVSTPYSALAAKAEMDGTILEWNFNASETNPSVNAEADACLVFINDYASEGNDRAGVADPASDELVTNV
AANCSNTVVVIHNAGQRTLDFAGHPNITAIIMAHLPGQDSGRALVSLLFGETSPTGRLPYTIPYKAEDFGSLLYPVVTPEAAPSANLSDPSEFDYKYFQSNDIKPRYAF
GYGLTYSSFSYSDVSVSWKDGSAPASAPPSVEVKVPGGVASLFDTVATVSATITNTGSVAAAEVAQLYVRRPNEPEGVTPLRGFEKTAYLQPGESECVKMHLRRKDLST
WSSDDQQWLMMAGDYELMVGASAEDIRLTGSLSLK
>tr|W2RZ60|W2RZ60_9EURO
MKRQTWRYAAALVLLTSTAASELLLHAYPDCSAGPLAANAVCDQSKTITERATALIGALTLEEKVNITGNVSPGVPRVGLPPYHWWNEALHGVALAPGTTFSDEGEFSY
ATCFPQPYTMGAAFDDQLVRDVAGVISTEGRAFSNARRAGVNFWSPNLNPFRDPRWGRGQEVPSEDVYHTKQYGKAFIRGMQGDDPHRWKVMATCKHVAAYDLENWHGR
VRYGFDATVSSQDLAEYYMQPFQMCVRDEKVASLMCSYNAINGTPACADPYLMKDIVRDHWDWKDEGTYMVSDCGAVRYAYSDHHFRGSREEMVTESLKSGIDIDCGYY
YPDYLGSAVRQGLLSEDDINQALIRAYSGLIRTGFFDLDNPYRSVSWADVGTPEADQLALRIAEEGVVLLKNGGILPLQVPQDRNLTIAAFGSWMNATVEMQGNYQGLA
KSIKSPLEALQALDNATILTANWQRSKYEFVQTVKPDVIVIADGNRLQDAMETVDRDLLEWHGEMSDVVSSLSGLGIPIVVINMGEQCDNGPYLRDDGVSAVLWAGYGG
QAGGEALANILTGKAAPAGRLPVTQYPTEYVYSIDMTDMALRPDPRTGKPGRTYRWYDNATLPFGYGLHYTTFSMSLDSPATLPTSFAIADLIARCDTSKPLDLCPFHP
FNNKSDSRSNGLAATVTNTGTTSSDFVALAFIAGEFGPQPYPRKTLVAYDRLHGITAGASQAVDLQVSLGGLARHDESGNQVLFPGKYRLLLDEPSVVAWEYELVGEEA
VLDLWPQPRGAGAGRVPEKSVKQRVSMGEVHGEL
>tr|A0A420WJH9|A0A420WJH9_9PROT
MTLPYKFLTSLAVVLQLSACNNGDAPNTTPKTITSSSYDRVESLMADMTLDEKLAQVSCVWFGKADIYDEDGNFDPIKMKEKFPHGIGCYARPQDTIGMEGPSDEPRDV
NDSTVVRRMSARTPADTVDLVNTIQKWMLEETRLGIPTLFHEEGLHGFQGRYATAFPQSIALAATFDTELAEDIYSVTAREIRARGVHHVLSPVVDVALDPRWGRIEET
FGEDPFLVSRMGVAAVKGFQGDGFPIGDEKVLTTLKHMTGHGQPESGMNVGPAQISERVLREIFFPPFEAAINEGNAATVMASYNEIDGIPSHANDWLLNDVLRGEWGF
DGAVVADYFAISELQVRHGIVNTVPEAGALALKSGVDMELPDGTAFPQLKAMIENGEYDIKYLDQAVRRILELKERGNLYETPYADPAYADSITGNNEARQLALKAAQR
APVLLKNDDKLLPLNIEDYKRIAVIGPNSDITVLGGYSDEPRQTISILDGIKEQVGDRAEIVHAKGVELTQNRSWWDDEVELESVDKNMARIYQAVATAKTADLIILAI
GGDESTSREAWSETHMGDRNDITLIGQQKELIEALAETGVPIATVVISGRPLSLENVEDKLPSILYAWILGQETGTAVADILFGEVNPGGKMPVTVPRNVGQIPSFYNH
KPTARRGYAFGDASPLYPFGFGLSYTTFDISEPILSTSIMGVEDATSVSVNVTNTGDVAGDEVVQLYIRDKVSSVTRPVKELKGFKRVSLAPGESKTVSLSIDKSALHF
FNRSMERVVEAGEFEIMVGNSSDNVKSTTLTVE
>tr|A0A0S6WTQ9|A0A0S6WTQ9_9SPHN
MKANLPTARRTPTRRVLGRLLLMATCLVPAGALVVGGMANRAEARAAASSEDAAVTARAKALLAQMTPEEKAGQLVQYFYLGEASATSSEAMTSAISVEDAVARGEVGS
LLLMTDPVEINRLQKIAVEKTRLGIPLLFGFDVIHGFHTIMPVPLAMAASWDPSVAEKGQAVAAAEARAAGVNWTFAPMVDIARDARWGRIVEGAGEDPVLGAAMAAAQ
VRGFQGEKIGTPGRILAGPKHFAGYGASLGGRDYDEVNLSDNELRNVYLPPFKAAIDAGAGNIMAAYMQLNGVPATTSSWLLNEVLRKEWGFDGFVVSDANNVSSLVRQ

GAAANPEEAAVRAIRSGLDLSMEMPSRTSPMLSLAQSLKDGTLDAKTLDTAVLRLLEAKIRLGLFEQPYADPAKAKSVAAKAEHRDIARMAAERAAVLLENGKGVLPLD
TSKTQSIALIGPFANAPHDQLGPWVFPGPKPTGVSVLEGLKAKLGDTVKVTYAPGVAIPKRINRSFFDDINPPVDLPEFAEADEAKTAAEFDRAVAATKAADVAVLVLG
EAQNMAGEAASRASFELPGQQQRLLEEVIATGKPVVVVLMNARPINLQGAKPDAVLEAWYPGSEGGPAIANLLTGDAVPGGKLPISWVRDGSQAPFSYDRMISHAPWGA
DTRYWDQASNAPLYEFGYGLSYTTFEYGNLTVAKDAVAKGEPVEVSFDLTNTGSRAADEVAQLYIHQRSGTSSRPERQLKKFRRIALQPGETQRVTFTLTADDLRYWSS
VTKDWIQDASVFDVWAGGSSKADLAGEFTVTP
>tr|A0A010QW43|A0A010QW43_9PEZI
MLWTSLLLLAGAQLSLTSQIHHRSSNNGTRDRVTALTLANTLLSHLTLEEKVSMITGNSSAGNCIGVIAPIPRLGFKGICMLDGPSAVNRADLVSVFPSGITAAATWDR
DMVYRRGVALAEEFKGKGGHVILGPSTGPMGRAALGGRNWEGFGPDPYLAGVTIENTITGMQSTGVQTCSKHYIANEQETQRSNTLPTSPDGTAIEAISSNVDDRTIHE
LYLWPFANAIRAGTTSIMCSYNRFNQTYACENSHLLKELLRDELGFQGYTVSDWFATHSTADSINAGLDLEMPGAVPGNYPGAVTFFGDKVLEAVANGTVTDSRIDDMV
RNILTPYYLLGQDAESYPSPDPSLRFVTLFQEVGLQVAKDTGFIPDDFVFTHGRDVRGDHAKLIREMGSAGTVLLKNVNQTLPLSNPKIIGVFGNDAGEMTNGFLRPSE
VPEDTNTGTMIIGGGSGTGRPSYVISPLAAVRAKAAEYGAEVLYVTNNDVLAANDFRGVYPPPEVCLVFQQTFASESFDRTSFELDGNSTAVINNVADFCETTVVVTHS
GGVNTMPWANHTKIGAILAAHYPGQESGNSIVDLLWGDIAPSGRLPYTVPRNEEDAGPAILNLTQPVVDPLAWQADFTEGQMIDYRHYDALDIEPLYEFGFGLTYTTFA
VVDNQIGLSFIGGNSSIAARPDTAIPIQPGGHPQLWEDLISVKASISNKGNRTAFAVPQLYASFPTSAPEGTPKKVLRGFEKIQIEAGGSAEVEFRLMRRDVSLWNASD
KVWVIPKGEFTFRVGFSSRDLPAEKSFSVLA
>tr|A0A420XNN5|A0A420XNN5_9ACTN
MTPRSTPPGGAQPPTDPDDRPRVSRRTLLKGTVGGATLAGAGLTGLVDPLTAAAASTASAGAGSGGSAALADALGSTAVDRTATAVTPEIRRKVDALLARMTLQEKLGQ
LQMQGGDVDGAPNDDLVSLARRGMVGTTLGVRGAANVNKLQRAALASRLGIPLLFAFDVIHGYRTIFPTPLGESASWDPSLARAAARVAAVEATAAGIRWTFAPMADVT
HDARWGRIVEGAGEDPYLGSAFAVARVRGFQGSDISAPQNMAACVKHFAAYGGVESGREYNTVDVSVERLWNTYLPPYHAAVNAGVATVMTSFNDISGVPSTGNPELLQ
DILRGRWAFDGLVVSDYTSVLELVAHGYAADPADAARLALTAGTDIEMVSTTYHDNGRELLLSKEITRRQLDDAVRRVLTLKHLTGMFDRPFATPARESVLLSAANRAV
ARRAVADSVVLLRNEGGALPLSPGIAKIAVIGPLADSGADMIGSWSGDGRAEDSVTVLAGLKAAYPSATVTYTQGCDPVATTAPDLAPAVAAAAAADLVVLVVGEPAAL
SGEASARSDIGLPGHQEQLVETVAAVGKPYVVVLVTGRPLALPWIAEHAPALLVAWHPGTEAGNGIADVLLGKVNPSGKLTAEFPRAVGQLPLYYSHRSTGRPFTDPTQ
KYQSRYLDVDNSPQFPFGFGLSYTTFELSRLSLSRRTIGSGGSVTVTAAVKNTGTSAGAEVVQLYFRDKVASLTQPVRRLAGFHKVQLAPGQSRWVTFTVRGSDLGFFD
NAARKRVEAGEFDIWVGNSSTGGLHTTLHLR
>tr|L7VKQ9|L7VKQ9_THES1
MVIPIVARVSEKVIIYYAGTVYENLNDKGITGVIKMQRDIKKIISQMTLEEKASLCSGLDAWNLKSVERLGIPSIMVSDGPHGLRKETTDPTDPGKKTTVPATCFPTAV
GLASSWNRELVEKVGAALGEECQAEGIAVLLGPGTNIKRSPLCGRNFEYFSEDPYLSSEMAASHIKGVQSRGVGTSLKHFAANNQEHRRMSVDAVIDERTLREIYLASF
EGAVKKAKPWTIMCSYNRVNGEYASENKFLLTDVLRNEWGFEGIVVSDWGAVNERVKGLEAGLDLEMPSSFGIGDQKIVEAVKKGELPEEVLDRTVERILNLIFKAVDN
RKENAGYDREAHHKLAREAARECMVLLKNEDKILPLRKQGTIAVIGEFAKRPRYQGGGSSHVNPTIMDSPYEEIKKSAGNNADVIYAQGYFIEKDEPDEKLLEEAKQAA
LKADVAVIFAGLPEHYECEGYDRQHMRMPESHCTLIEEVAKVQPNVVVVLCNGSPVEMPWIDKVKGLLEAYLGGQAMGGAIADLLFGDANPSGKLAETFPKQLSDNPSY
LNFPGEGDRVEYREGIFVGYRYYDKKNMEPLFPFGYGLSYTTFEYGDLKISRKEISDNETVTVSVKVKNTGDMAGKEIVQLYVRDIESSVIRPEKELKGFEKVELKPGE
EKTVVFELDKRAFAYYNTGIRDWHVETGEFEILIGRSSRDIVLKDKIFVKSTVTIKKPVDRNTLVGDLLSDRVLEPVFREFIINEIKNRYLLDLLENEDKSLLSVWMRY
TPLRSLANSTGGELNEEKLNRLIDTLNANIK
>tr|Q0CE57|Q0CE57_ASPTN
MRLTECLFVCLTGCSLASVVPHGQTVLHLPGEIPPRNPPIVNPIRAPYQDPKQPTWLRVEDLLSRMPMREKMAQLMQGELEKWVDFTTGEFNISGMKDHMFYHSGMLRV
SHPLTTAEISHQGAHLLQDWIMNDTSLSLPAIVQCEGLHGLGVPNATIFTSPIGLGASWNPELVEQAAQIIGQEARALGITQVFAPSADLARDPRHGRVEESMSEDPYL
AGEMVAHFVQGLWKNKVASIVKHLVAYGNSEQGINMGPVHGGERELRTTYLPPFRKAIEAGAFTVMTSYNSYDGIPSVMNKHLLTDILRGEWDFQYWTTTDYGAPNRLC
TAFKMCRDNPIDAEAITMKIFPAGQDTEGGGSFNFDSIPDLVNDGTLDIAIVDEAVRRVLRAKFDMGLFEDPFPAAAPEDWPNLIHTDKALEVAKQLDLESIVLLENHD
NTLPLKDDIGSIAVIGPFADTVNYGDYTMSRPRGVTPLDGIRSRASQGVHVKHAKGCEAWSNDESGIHEATELAKSSDVAVVVVGTWTRSLEGQQEGLNATTGEAYDTN
DLGLVGAQRLLVQKVAETGVPTIVVFSSGKPIVEPWISNATAALLQQFYTSEQGGEALADVLFGKYNPSGRLPLTFPRDVGSLPVVYDYLDSGRADDEPGHINDDGSIE
FGRTYVTGTPLPWYEFGYGQSYSTFEYSKVEVDRKHVRKTDTITVSVQIENTSDRDGVEVVQLYVADPVSSVVTPNKQLRAFQKVFIKARETATVMLDLKVREIGLWDL
DMKYVVEPGEFVAYVGRSAGDLKGGVSFYVD
>tr|A0A3Q9I604|A0A3Q9I604_9BACL
MQVQKVQFSAPRRLDFLNTTSGRRVRKTVIYKDRNYSNSERAQNLLSLMTLEEKVGQLVQPFGWQVYEHRDGNIELTEAFKKQIESGGVGSLYGVLRADPWTGVTLETG
LSPEEGAAAVNEIQRYAIEHSRLGIPILIGEECSHGHMAIGATVFPVPLSLGSTWNVELYREMCRAVARETRSQGGAATYSPVLDVVRDPRWGRTEECFGEDAYLISEL
AVASVEGLQGQSLDQDESVATTLKHFVGYGSSEGGRNAGPVHMGKRELLEVDLLPFKKAVEAGAASIMPAYNEIDGIPCTTNHELLQDILRTEWGFEGMVITDCGAINM
LAAGHDTAEDGMDAAVQAIRAGIDMEMSGEIFGNHLLEAVRQGKLDEVIIDTAVLRVLDLKFKLGLFDAPYADPEHAARVIGSEEHITIARKLAEEGIILLKNEDQTLP
LSKKSGRIAVIGPNADIGYNQLGDYTSPQPPANVTTVLGGIRSKLADEPERVLYAPGCRIKDESREGFDYALECARQADTVVMVVGGSSARDFGEGTIDLKTGASKVTD
RSWSDMDCGEGIDRMSLKLSGVQLELIQEVYKLGKPVIVIYINGRPIAEPWIEDHANAILEAWYPGQEGGHAIADILFGDVNPSGKLTISIPKHVGQLPVYYNGKRSRG
KRYLEDDSQPRYPFGYGLSYTEFSYSNLTVQPQTISAGGTAKVSVEVRNIGDRVGAEVVQMYISDVASTVTRPAKELKGFHKITLAPGESRTVEFTVGAEQLQYIGADY
KPVVEPGEFRVLVGKHVNDTLSAHLLVLED
>tr|A0A433SSR8|A0A433SSR8_ELYCH
MSYIETNYKVLILCLCLIAALDGSDPLYYKQRVFPLMKDKSDSKFGFFDHETESLSHNEFIKTCGPMGQYPFWNVSLSWEERVKDLVARLSLEEIQLQMARGGAGPYST
PAPPISRLGIGPYVWNSNCHRGDQGAKENATAFPQSIGLAASFSHQVLFDVATVASVETRGKHNDFVRRGVYATHTGASCFSPVINVVRDPRWGRIQETYGEDPFMSGE
LAATFLDGLHGDNQRYVRVTGGCLHLDAYSGPENIPVSRLSFDAKVSDYDLYMTYLPGFKRCVQAETFSIMCSYNSVNGIPACVNKRLMTDILRKDWKFQGYVVSDEQA
LEFVISTHKYLQSFEDVAAAAVTAGVNLELSADMPQPVFLSIVQAIEQGKLSEAVVRERVKPLFYTRMRLGQFDPPGANPFSRLSNNDVVTPAHQAMALEVAKKSFVLL
KNSNNFLPLKPSNYKTVSIIGPMADNYDQMFGNLPPMQSRVFAKTPLEGLKEIFPSLRYKQVCEGETKCTNFSREAVQELVNGTDLVLAVFGTGPAVEAENNDRANLDL
PGMQKQVLEEIMEHCGTAKILVVLMNAGPLNVTFLDVDPRVQAILECFFPGQATGDAIKEVLINTGGNSSPAGRLPVTWPLHASQIPPITNYSMVNRTYRYMQSLPLYP
FGYGLSYSQFSYESLTSDANVKAMENLSFEVTVKNIGDYDADEVIQCYISWGRSDLPVPVRQLGHFTRVHLKQGEEKLHIGTISWQRWAFWDKGLWQVKKGPMTLSCGG
QQPDQTKKVPSNVISTRFYIVNSASTTDEL
>tr|A0A540VGT7|A0A540VGT7_9CHLR
MTDHTTATPIYRNPARSPQERARDLLSHMTLEEKVAQLTSVWVYEVLDDLAFSPQKAAAKLGDGIGQITRVAGASNAAPRQTAELANTIQRYLVEQTRLGIPALIHEEC
CSGFMGRGATCFPQIIGVASTWEPELVAQMAAVIRTQMRAVGAHQGLSPVLDIARDPRWGRLEETFGEDPYLTARMGVAYVQALQTDDLRQGIVATGKHFLGYSFTLGG
LNWAPAFIPPRELWEVFMFPFEAAIQEAGLASMMNAYHELDGIPCGASRELLTEILRNQLGFDGLVVSDYMAINQLVDYHHYARDKTHAAHLALQAGIDVELPSRDCYS
DALVEGVRTGAVDPALVDQSVIRVLEMKFALGLFEHPYVDEEAAPQVFETPEQRQLARTIAQKSIILLKNEGDLLPLDRQPGTIAVIGPNGDSIRNMLGDYSYPAHIEL
LAHLQERGGMDTPIPESLTLEDIYPAMKSVLQAIREKVGEGSEVLYAPGCGVNDSDTSGFDEAVAVARRADVAIVVVGDKSGLTPDCTCGEFRDRATLHLPGVQEELVR
AVVETGTPTVVVFINGRPVSSPWIAAHVPAIVEAWFPGEEGGPAVADVLFGDVNPGGKLPVTVARSVGQVPIFYAHRPSGSHSFLYGDYVDETVAPLFPFGHGLSYTRF
EYSDLEITPEPVSTDGTLQVRFTLANVGERLGDEVVQLYIRLRGASVTRPVKELKGFRRVTLEPGRRVAVTFDLPIPALSYLDPAMERVVEPGELEVMVGSSSADIRLT
GRVQMVGEKICLGRKRPFFSTARVSSLDA
>tr|A0A0B7MX61|A0A0B7MX61_9FUNG
MLSIRQSIYLVLLFSVSTCTALATNTEHHKYGTLNSEEYDHPELTIDDDIISMIGQMSVEEKIGQMTQINQDLVLGIDGVLNRTAVEYYAKNYYVGSYLNQLAVNGRNY
DAADYAKIIEEIQEITLSVNSTFKIPIIYGLDHIHGAHYVANTTIFPHGINIAASFNPELAYESASITARDTRASGVQWTFAPVLDIPVNKQWPRVFENFGEDPHLSSV
MGVASIRGYQGKYKTDRSKVAACMKHFIAYGAPYSGQDRDSTVVSERTIYDYFVPGFQAAIDAGVATAMESYIDINGEPVVSSKKYLRQLLRDQMKFGGMLVTDWQEME
NLHTKHMVAATHKEAVRMAIADTSIDMSMVPQDVIFFESMMDLIKEGRVTMERVDESVARLLQLKKDLGLLEPNGWKADPKLQEMIQNDSDIEVAMEAARESITLLKNK
NAALPLTKDKIKRVLVVGPAGDNLGHLAGGWTIQWQGATEDAWHGNVSDEQFYNNGISILDGMQNAVPEGVQVEYIQGFTIDGNDVNMDLVLEKAEDFDAFVVCIGEHI
YSELPGNIHDLTLPQGQINNVEKLASFIGKEKHLITVLLEGRPRVLGSVQDNSDAILQAYLPGPWGGQAIGEVVFGLTNPSGRLPYTYPKYAGDTTLNYWRPANDIWDP
LYEFGHGLSYSSFQYSNITVNGLLSTDEKQYLIAGEVEKEVSVNITNISPVDGKETIMMFVKQPFRIVTPPAKLLKAFQKVMIPAGETVNVRFSVDADLFRYTGLDGIP
QNTIDNGAVKILIGDQEFELQLVNNNGE
>tr|A0A364KTH7|A0A364KTH7_9EURO
MRVLNLSTTAALYGVLSATLAGSQIVVPTIQGFQPTIDDWKAARAKADAFVGKLTLDEKLAMVRGNASAGFCSGTIAAIERLDFPGVCMEDGPNGVNYNDKVNVFPSGV
TVAATWDTEFMYQRGLALGAEFRDKGINVMLGPVGGPLGRSPYDGRTWEGFSPDPYLTGLAMQATIQGVQENGVQTTAKHFIGNEQETQRSNTTLSNGTQVAAISSNIG
DRALHELYLWPFANAVKAGTSAVMCSYNRLNETYACENSDALISILKTELGFEGYVVSDWGATHSAAPSANGGLDLEMPGYTGTTEVSTWFIPYLPQAIADGSVSIDRL
DDMVGRIMTPYYLLNQDAGHLTPDESMIYMLLVATNGLPGAEQLSSLVGVPWQNIAARDVRGIHGAIIREIGAEATVLLKNEGSILPLGSYNEIGVFGSSAADFVDGLA
YWDMGIDYGPQIGPIAVGGGSGSVRFTTVSAPLDAIKAQASTKGARVQYLTNNTAIAANDITFYPVPEICIVFISQFATEASDRDSLLASDNSTAVVENVASLCNNTIV
VINAPGPLVLPWVENPNVKAILAAHFPGDQIGNSIVDVLWGNTEPSGRLPYTIPAKTSDPDIIPVVREPGPDGWQSTFTEGLFIDYRAYDANRSEPAYEFGFGLGYTTF

NITGELAARTTAFNPTATPANSAIAPGGNPSLWDEIVSVKATILNVGKATGKAVPQLYLEFPESSPEGTPVRVLRGFAKLQLDPGKEGDVQFSLMRRDLSYWDIHEQQW
VIPTGAFTVNVGFSSRDLRASTQVTVLK
>tr|A0A0E9NIL3|A0A0E9NIL3_SAICN
MLLQLSTLTLLLTSPILSTASPTINADPSFPLTTGAGGWESAYQQALALVSQMSIPEKVNLTTGIGSLGRCEGNTGSIPRLGIPELCFQDGPAGIRPTDFNSVFPAGIT
AASTWDRELLLQRGLALGAEWRGKGINVALAPVTGGPLGRNAAGGRFWEGFGADPYLHGAAAYETIIGWQSNGVIATAKHWIAYEQETWRNTNSGGPGAGYPFTLTNFN
NTYTLSSNVSDRTMHELYMWPFAEALRAGAGAVMCSYNRINGTQACEDPYSLNHLLKTELDFQGFVMSDWGATYSTIPAVLAGEDVEMPGGDQWFGQGLIESVNNGSVP
ETRLNDMVTRLLTPWIFLGQNNGSYPETNYNGNTLETYNATTGEVINEHVNVQDDHAVIIKKVAEEGTILLKNTDGDRGLPLTNVKKLAMFGTDAGANPKGPNYCQQWI
PGSQLCPVGASNNGTQAVGWGSGASLFPYLVDPLAAINERASQEQTFDVEWVLEDWPVGDSWATINATAHRATHCLVFVQARSGEDSDRQNFTLWDNGDNLINTVAANC
PNTIVVEHVVGPVLMEEWVENENVTAILNAHLPGQESGNSLVSVLFGDVNPSGKLPYTIAKSADDYIPIFGEYVNDPQVYFSEELLLDYRRFDALNITPRYEFGFGLSY
TTFSYSDLAISGSVSSGPAATPGEQNAPGGFPSLFSTALNITATVANTGDCTGAEVAQLYLGFPASANEPPRVLRGFDKISLCAGESGQVTFPVTKKDLSIWDVYSQGW
QIVQGTYDVYVGASSRDIKLVSNFIVA
>tr|A0A1M6FLV9|A0A1M6FLV9_9ACTO
MPAAPTAPDESAGPAGPRYLDPAAPIGERVEDLLGRLSLEEKAGQLIQYFYQRSMIPGLPGSDGADRLAEQDRDVLAQSEMVEGEVRAGRAGSLLFVRRPEAVNALQRI
AVEEGPHGIPLLFGYDVIHGLRTIMPVPIAMAASWDPAMIEEAQAVAAREARAVGLSWTFAPMVDIARDPRWGRIVEGAGEDPCLGAAAAAAQVRGFQGERIGAPGRVI
AGPKHLAGYGAARGGRDYEDAEISDNELWNVYLPPFRAAIGAGAGSIMSAYMDLGGVPASGNAWLLTDVLREEMGFEGFVVSDANAVRSLTAQHFAADLPDAASRALTA
GLDMEMTMEDAAFRRLPEAVRAGRVEEGRLDEAVRRMLTAKFAMGLFESPYADAGTAPAVLADPAHREVARRAAERSLVLLKNAPWQGRSAGEARGGEPEGAPVLPLAE
DTPSIAVIGRLADSPRDTLGPWVFDEDLDEVTTILAGIRARAGGARVDYAVGEWAPPRVFPSMFDDHEAPGSLPARPEGFDDDAALAEAVRTARGAEAAIVVVGQAQNQ
IGEKASTSTLALPGRQLELLRAVAATGTPTVALVMSGRPLDLRWAQENLPAIMQVWYPGTRGGEAVAAALFGDIDPAGRLPFTWPRTVGQVPMIHSHYRTFQPEGAGER
YWDEASAPLYPFGHGGSYTAFSYSEPRLSSPRIAVGQSVTVSVDVANIGPRDGEEVVQLYIHQRHGTSTRPERELKAFARVAIPAGRSRAVELVLGPDELSYWSAVTRS
RVQDATTIDVYVGGSSAAGAHAVLEVV
>tr|A0A1X2HFP3|A0A1X2HFP3_SYNRA
MVFLSLQGAISAFIGASLLASAVHAVPVTEEKRDDGRNPVYLDPSASIEDRVEDLLGRMNNEEKMYQLMQGNIANMIDTDYNLNSSALHQYGTTFAANMDRDPLARLIN
ETQSYMLNDNRLKIPTIMQSEGVHGYLDVNATTFPAALALAGTFNTDLMEKVGDIIGTEAASLGLHNIFAPVLDLAREPRWGRIEENYGEDPYLTGEMGYAYVKGIQGK
AKDGTAKTAKHRIGAMVKHFVGFGSPMGGLNIAPVLGGERDMRTLYLPPFKRTIIDAGALSIMSAYHAYDGIPSAIDKHTLTDILRNEWGYQYFVESDSGAIANLCDTH
YVCDGSFLDPVAAVKAIEAGNDIEMGGRPMHYATIPDQIEKGNLKQSTVDEAVRRVLRAKFALGLFEVPYGTSNYNASIHTKKHLEIELQTEEEAITLLENDGTLPISE
SVESIAVIGPQANVMQYSDYTAHGVFERGVTPLAGIQKLVGDKVKVNYAEGCKLWSLDKSGFDEAVEAAKKSKVAVVMVGTWTRDQTELWSGYNATTGEHVDANDLRLV
GAQMDLIKAVQKTGTPTVVLLITGKPTAEPWLKDNVNAILNAFYPGEQSGTAIANILFGKTNPSGRLPISFPTSVGSLPAFYNYPKSGRPTDPGQIYANGTMDFGYQYI
LGTPVPLWYFGHGKSYTTFEYSNLKLSKTKVHGGKDTVVSVEVTVKNTGEMDGKEVVQVYVDDVISSVVVPNKALKGFKKVSIEAGKQQKVKIAIKLEDLQVWNYDNEW
ELEKGDFNVFVGGSYADLNLNATFTVV
>tr|A0A1B3NKC1|A0A1B3NKC1_9HYPH
MQWASQRGQGFAQLSRHGHSCRAALWIRHLPFVPPAPVSSRKMPCMISRLAAPLLALGLVLPVATTTAQAQAAPSAAATPVAWKTGREAAAIERRIDALLAKMTLEEKV
GQLHLSGRGDGFDIAQVKAGRMGAVMNFVVPAEVLAVQKAVRESRLKIPLIIGLDAVHGFSTYFPLPLGQAASWNPALIEQAAYWTGREAAAAGINWTFAPMVDMSRDP
RWGRVLEGAGEDVHLASVVAAARTRGYQRGGVATSVKHFVGYGAGEAGRDYNSTWIPTSQLFDLHLPPFKASFDAGSMTAMAAFNALNGMPATAHRGMLTDLLRGQWGF
RGFVTSDFGSITELRLHGIAMDDAEAARKALLAGIDMDMMGDVYHKHLAAEVRAGRVPVKALDEAARRVLRVKFHLGLFDKPDVDPAAAPAMMQTQGARDVALRAAQEG
AMLLKNAGDILPIRPSVKSVAVIGAMARPEDERVWTDPAGLGRRVVQPLPEALKERLPADVAVTYEPAFTKACGTEFADREAAIRAAAASDLIIAMLGEDCEFMGEGAS
RTRLDLPGVQQPLLEALVATGKPVVLVLATGRPLVLTWADAHVAAILQTFHGGTEGRTAIADILTGKVNPSGRVPMSFPRSVGQIPVYYDHLPTGRPQKIRQRYESIFI
DEANEPLYPFGHGLSYTRFTYANARVSKPSMRLGGSVEVSVDVTNAGPRDGQEVVQLYVRQPVASRSRPVRQLKAFDKPMLRAGETRTVTFRLEGSRLGAHDDAGRYVV
EPGLVEIYLGGSSQTSVMTQVMLTKS
>tr|A0A2V1D797|A0A2V1D797_9PLEO
MKALKQLISLWPALILAQNASNSHYPSCTTLPLRDNPVCDESLDPAARAAGLVAAMNISEKLVILIDHSPGIPRLGIPAYDWWGEALHGVAYSPAVRFAMTGEFSSATS
FANPITISAAFDDELVERVGKTIGVEARAFANAGRAGLDFWTPNINPFKDPRWGRGLETPGEDAFRVSQYVKHLLRGMEWASEEPSETRSRHIIATCKHFASYDLERWE
GIVRQKFDAQVRMQDLVEYYLPSFRQCARDSNVGSIMCAYNRVNGTPACADSYMMQTVLREHWGWTKHGNYIVSDCNAVKNIWADHNWTSTAAQAAGKAFTAGMDNVCE
VSRGSTDVIGAFNQSLVSEEVIDTSLKRQFEGLVRAGYFQKSPDPTGFRSYGWNSVNTDAAANLAKQSATDGITLLKNDGILPVQFKKNQTVAIIGMWANDTQNRMLGN
YFGRPPYYRSPLWAARQLNISTLYANGPVSNTTFNQSAAIEAARSSDTILYFGGIDGSIEAEDLDRTEITWPAPQLSLLSALSKLGKPIIVVQLGTSLDNSPLLDNPSI
SAIVWAGYPGMYGGPAVFDILTGVKAPAGRLPITQYPAEYAKQVNMTDMTLRPSQKSPGRTYRWYDQAVQEFGYGLGYSNFSVKFGGPWSGDDSKTVAFPSLGNTECGK
EYRDLCAFPQKVPVLVKNEGKITSDYSVLIFAKSEAGPKPWPKKSLVAYQRLRDIKPGEEREVEFELTVGSFSRVLDNGDSILYDGEYCLVWEGGLDGGDGEEQACLEI
AQGEGGGLALVDRWPQPKPANATLGA
>tr|A0A558R463|A0A558R463_9SPHN
MSKPVLRRSLLLASAVMLAVMPTTSIARDPASSPGASGVGVISEKEAVARADRLLAQMTLEEKVGQISQRFDIASLFPTGASAPPGMPAMTPLDDNVRKAELGSLLFVH
EPAVANKYQKIAVEQTRLKIPLLLGYDVIWGMRTMFPVPIGGAASFDPAGVEQARAIAASEARALGIHWTFAPMVDIARDPRWGRIVEGAGEDPYLGAAMAAAQVRGFQ
GDHIGAAGHIVAGPKHFVGYGASVGGRDYDSAYLSDSELYNVYLPPFAAAINAGAGNIMSAYMDLNDVPASANKRLLTDILRGDLGFKGWVVTDASAVHNLVKQGFAAD
GADASVRAITAGVDMEMSTAPNAFSTLVASVRAGKVPMKRIDEAVRRVLMVKYRLGLFENPYVDAAAAERITSDPAHAMAAQVAAERSLVLLKNDGNALPLVAGAQKRV
AVIGAMADSPGDTTLSLAFPQDAVKAVTVFKGISERLSGIATVETTPGVQLSRLVPSPLAMLLGPVTPWTPAQAADELKKAVGLAARSDVVILTLGEKIEMSSEQASRS
DLALPGDQRKLLDAVLATGKPVIVVLMNGRPLDLTGVYDKVPAILEAWYPGSRGGTAVARALFGDVNPGGKTPVTWPRSVGQVPTYYARNLSHDPDGAGRRYWDAPSTP
LIPFGFGLSYTSFTIAPPTVDKAELAPGGKVVVSTMVTNTGARAGDEVVQLYIHQRAGRQSRPIRLLQGFRRVTLAPGESQQVSFTLDEANVRYWNSIERGWVIDPGTF
DAWIGNSSLADAHTSFIVSGAPRAAH
>tr|S0FPI8|S0FPI8_RUMCE
MGNFREEMYAEKYKDNLLSPEERSEDLLSKMTLREKVGQLNQRLYGFDCYNRIDDEVELSAEFKEEVKYWNGLGVLYGLYRADPWSKRDFNTGLEGSLAIKAYNLAQKY
VVRHSRFGIPMLMSSECPHGHQALDGYLLPVNLAMGAAWNPELMASAYGVCARQMKDLGVNLALVSMLDVLRDPRWGRSEECFSEDPYLCSVLAEAAVKGCQERGVPVV
AKHFCAQGEGTGGINASAARIGERELREIHLPPAAAVCKAGVKGIMAAYNEIDGIPCHGNSRLLQDILRREMNFSGVVMADGTAVDRLDILTGDNMRSGALALSSGVDI
SLWDKGFTGLEEAVKKGLVSEKLIDRAVLKVLELKFQLGLFERPYLEEATEKEAPLVEEEKHSCPSRQPYRYSYEQHPQSLELARQSAVLLKNEGILPLDLKKVKSIAV
IGPNADNIYNQLGDYTPPLRKGEGITLLKGLENLCSDTKIRYTPGCSIGGKDETGIKTAVELVSSSDIVILALGGSSSRFAGASFDINGAAVTPQPDVESARNFQMDCG
EGVDCAGLTLPGVQQELARAVFDTGKPVITVLIQGRPYAVEEAAARSRALIAAFYPGPMGGQALAEILLGKVCPSGCLPVSIPRSAGQLPVYYNHKVSYDAMRYSDMAN
TPLFPFGFGLSYTGFKAENFKLTTENISLEKLEKGEKFELAFTVRNAGNLEGHAVLQLFVQDLQASTVRRIRELKGFTKVRLAAGEEKACRLELEYEHLAVWDSGMRYC
VEPGEFLLELRESGRKVWSGLVVCNT
>tr|A0A0R1VY77|A0A0R1VY77_9LACO
MKDEVFLWSGRTKKSFLRETNISVKTYTFLLQLFLHYANMSFVSVSIQRFEFCCLPINWAGLDFEGKAKRFYILLQGGDCLEKRELQSLVDKMSLKEKIGQLTQFTPQY
LGREDGELTGIMGDFAVDQVYLDSLGSVLNATDRNEVIAMQREHLKKDRLKIPLVFMRDIIHGYRTTFPIPLGLGATFAPQLVEEVSHVAGTESAREGIQVTFSPMADL
CRDARWGRVMEGTGEDPVLNAQMAAAMVRGYQGAPGELVKDPTRIAACVKHYAGYGAVLAGRDYNNVDFSRLSLYQDYLPAFQAAIEAGAKLIMPAFTLFEGLPATASE
YLLKKVLREYLKFDGVAISDWGSVGKLLTMHIADSQERASELALNAGLDMDMMAGAFVRGLSDAVAAKKVAVEDVDRAVLRMLNLKNDLGLFENPYRFIDAHETLPPAP
SSESRRYARVAAEKSVVLLKNKNVLPISVKSHIAVTGPLAASQRILGAWSSYGKHDDAVSLYAGLKKQFENVSLIPDPISSDPADYANFDVIIVGLGEREDDSGESACK
TRIELPEDQVALIRRLQHTGKPIIGVIFAGRPLALTNVVPYLDGLLYAWFPGTEGGNALANLISGKAVPEGHLPMTFPRTTGQVPIYYNEPRNGSPADDQHPTKFTSRY
VDCQNSPLYPFGYGLSYGHIEYGKLQVNQPVLTDDQPIQITVAVTNDSDYRSSTLVQCYVGSTITSVVRPVSELKSWVKVVLEPNQTDQVTLTISVDDLAYVHSNLERF
ADKGDFIVRIGENAEEYQELTLTYQ
>tr|A0A1J4K6J3|A0A1J4K6J3_9EUKA
MIFCFLILASAVPIYLDVSKNIDERVDDLMNQMTLEEKIGQLVCPDGRFNFTEIFNEQHIGATFFLFDDDAKHAQEMARNTRLKIPLIMGIDAIHGNSFYNGSTIFPTQ
LAASCSWDVEVMKEMAQITASEMKYTGTFWTFSPVLCLTRDLRWGRVGESFGEDPYLIGVLADAMITAYQDNGIIATAKHFVGYGETIGGRDASEGDLSRRKLLSYFTP
PFEKVSSIVSSVMSSYQALDGTPAVVNHWLLNETLKEKWGFKGFVVSDYDNVGRLINDQQVFDNFVDASLASVKAGNDMFMHTPQFFGAALEAVNQGKLPISYIDESCR
RILRVKFEYGLFEDDRMADKTKVVYGTEENRKYSLKAAEKSAVLLKNNGILPLDESLIQSFAVIGPNADNIIMNGDWSLGSQQKETHGRNCTVTILDGIKNRMKNKEI
NYAGGVKLEDDDPFEEDIHYAVENVQKSDISVVVIGDRLKYSGEGKSTGTLDLMGNQIELLEKIMETGKKFIIDVISTKPVIIPQHIVDNASAIIHQFSPGMLGGTAFS
ELIFGDINPSGKLTVSYPVHVGQQPVWYNQVRGQHGTTYADLTETPMWSFGHGLSYSNFIYSDINISNKDDETNEFLAGDEIIINMKIKNDGKYDGAEIVQIYIMDNVT
SSTWAVRELKAFQRVEIDAGDTKEVSIKLNTYDFSIVNASEERVVEPGDFVIQIGKSSDDIVKNFYITILPPKGDSPHEKVTAIAIIGAVIAVISLIIIVVILVIFIRR
AKKKEPSQFDELLKSDGEPIKFVNG
>tr|A0A2K1IUW5|A0A2K1IUW5_PHYPA

MVPMARKIETLFPLQGRMCWLIIILLLQPFHKGAALEYACNFDKDDNLKSMRFCDTSLSDEIRVFDLVSRLTLEEKVTQLVNTASAIPRLSIPAYEWWQEGLHGVAHVS
FGGSLPRATSFPLPILTTASFNKDLWNQIGQVVSTEARAFYNDGIAGLTYWSPVINIARDPRWGRIQETSGEDPYTTSAYATHFVQGMQEGDANSKRLKLSACCKHFTA
YDVDNWEGIDRYHFDAKVTLQDLADTYNPPFQSCVQEGRSASLMCSYNKVNGVPTCANYDFLENTVRRAWGLNGYIVSDCDSVLVMHESTNYAPTTEDAAADALNAGLD
LNCGDYLASYTEGAVAMGKVNASRVDNAVYNVFLVRMRLGMFDGNPANQEFGNIGVADVCTPAHQELAVEAARQGIVLLKNDGNILPLSKNINTAVIGPNANATHTMLG
NYEGIPCQYITPLQGLVKFGSGDYHKVWFSEGCVNTACQQDDQISSAVSTAAVADAVVLVVGLSQVQESEALDRTSLLLPGYQQTLIDEVAGAAAGRPVVLVLMCAGPV
DINFAKNDKRIQSILWVGYPGQSGGQAIAEVIFGAHNPGGKLPMSWYPEDYTKISMTNMNMRPDSRSNYPGRTYRFYTGEKIYDFGYGLSYTEYKHSFALAPTTVMTPS
IHSQLCDPHQTSAGSSLPCSQAHQETCSSSNFDVHINVENIGAMAGNHTLLLFFTAPSAGKNGTPLKQLAAFDSVYIRSGSQEKVVLTLNPCQHLGTVAEDGTRMLEAG
NHILSVGDAKHSLSVLFSDTSGARY
>tr|G3AHM6|G3AHM6_SPAPN
MLPIKLLATLALAIFANGETPPSSPDYNTEANPQLPPITFEAVHYTFPDCQNGPLKHNAVCNPHLPTEQRAKAVVDLFTVDELIANMGNTSPGVERLGLPPYQWWSEAL
HGIARSNFTASGEYSHATSFPQPILMGGAFNNDLYKQVGNVIGTEARAFNNVGRAGLDFYSPNINPFRDARWGRGQEVASESPVLVGNYALNYVQGLQGGLDSNQNDDT
LQVAATCKHFVGYDMESWNQHSRLGYNAIISDQDLADFYLPTFQSCVRDAKAAGAMCSYNAVNGVPACASEFFLNTVLRDGFDFQNGVIHSDCDAIYNVWNPHLYAQDL
GGAAADAIKAGVDVNCGDTYQNNLGYALGNKTINENQIRTSVTRQYSNLIRLGYFDSPQTNKYRKYDWNDVSTPQANQLAYQAAVEGIALLKNDGTLPFNKQKVRKVAV
IGPWANATTQMLGDYAGTPPYMISPLQGAQSEGFQVEYALGTQINTTDTSGYTAALNAAKGADAIVYFGGIDNSVENEALDRESLAWPGNQLDLVSKLSGLKKPLVVLQ
FGGGQIDDTEIKNNKNVNAIVYAGYPGQSGGTAIWDILSGKYAPAGRLTTTQYPASYADQVPMTDMTLRPRQGYPGRTFMWYNGEPVYEFGYGLHYTTFSASLANAPRG
GHQSFNIEQVVAAAKRSQYVDTGLITTFDVNIKNTGKTTSDYAALLYSKTTAGPGPHPNKILVSFDKLHQIHAGQTQTAKLPVTIGSLLQTDTNGNKWLYPGTYTFFVD
NDKKAQWEITLTGQAELIQKYPSQK
>tr|A0A0Q6UM06|A0A0Q6UM06_9ACTN
MAHTRRRTTTLAAALGLTLALASGALASRSASDDEDALAPTRGRPSNSIERKIDRLVARMTVEEKLQQVQLLSDGQITDADARAGVGSVFSLVDPEKIDHFQRIAVEES
RLGIPVLFAYDTIHGYRTIFPVPLGAASSFDPDVARADAEVGARESAVQGLKQVYSPMVDVSHEPRWGRIVEGAGEDPYLGSVMGAARVKGAQGSDYSAPDKVVSSVKH
YVAYGQPEGGRDYNTTDMSESRLRNLYLPPFKAAIDAGADTVMCSFNSINGVPGCANKYTETDILKKEWGFDGFIESDYTAVAELRACPPVRPDEGPCGHGVAADGPQA
GAAALMAGTDSEMVSTNIRDYGTELLASRQITMRRLDDAVRRILRVKFRAGLFDNPYIDVGAAADPASYGRPEDLEKSRWAAGRSMVLLKNDDGALPLDPERSTALIGP
FGDNVDDVLGPWSGRGSDDLAPDHVPLVAGLRAASSAEVTYTQACNLAHNFDPPNNANPPLTPEEEVCAGPGADGTTIEDAVAAAEAADQVVLALGENAFMSGESNARS
ELDLPGAQEELIDAVAQTGKPLVVVLFNGRPLDLSAVQGKASAILEAWFPGTQGGNAVADVLFGTVNPGGKLPVSFPRRVGTVPYYYNHEPTGRPCDPTFKWNSRYRDL
DTCAPLYPFGYGLSYTTFEVTGLSLSRSTVRRTGSLTASMRVRNTGSVAGDDVVQLYLHDPVASLSQPVRRLRGFQRVSLEPGQSTTVSFTLDKSDFGFWDNQGTYVVE
PGLIEVYGGDSSTATMKQTFTVTR
>tr|A0A5C6CKZ0|A0A5C6CKZ0_9BACT
MKFCTALCNRHDMTPTYFLDSFLCFVCCLAISGSPLLSAQETKTERPWLDKSAPLDQRVDSLLAEMALEEKVGQLTQSNGIGGEATGNTKNLVADSALYELIRSGQLGS
ILNEINVTTVNEFQRLAVEESRLGIPLIIGRDVIHGFRTIFPLPLGQAATWNPEMVEQACAIAAREARSAGVGWTFAPMVDIARDPRWGRIAESFGEDPYLASSLSAAS
VRGYQGDDLSNSDSIAACVKHFAGYGAAEGGRDYNATMISPSTMRNVYLPPFQAAVDAGVATLMCGFHDVNGIPMSVHKQLLSNVLRGEWGFEGFVVSDWDSIFETIEH
GSSKDERAAALAAAQAGVNMEMSSPCYRKNLTELVTSGQVSETTVDELVKPILRVKFQLGLFEQPYTEPDAAKILLSQEHLDAARKVARQSVVMLKNESSVLPLDKTIL
KKIAIIGPLADAKRDQLGTWIPDGKEADSQTPLAAIRESAKNNEIEVLYASGLVDDLDRSTQGFAEAVATAEQADIVVLIVGERANISGEARSRATLDLPGAQNELVST
LAKVGKPIILIVQAGRPLTIGKQIEAVDAVLYSFHAGTMAGPALADLLWGIESPSGKLPVTFPKSVGQIPLYYNHVNTGRPPRPYDFAKDKRVDDDFDVELGYNSNYID
VGPYPLFPFGYGLSYSTFTYGDVELSATKLRSGEILAVRVPVSNSGKVAADEIVQLYVRDLVGSLTRPVRELKGFRRIQLEPGETSVVEFALPVSDLGFFNNDEERVLE
PGEYEIFVGGSSLAPKVGTLEIVE
>tr|A0A5P1FMX0|A0A5P1FMX0_ASPOF
MASSLSSLLPLLLLSLTSFAALSLADYPLPYNFTHVCDAKRYASLNLSIPDFAFCDKSLSYYVRAKNLVDSMNLTEKAKQLGNNNNGEWYAVAAGVPRLGLPAYNWWSE
ALHGVSYIGGGTSFGGPDVKAATSFPLPINSAAAFNATLWREIGKTISTEARAMNNLGFAGLTFWSPNINLVRDPRWGRALETPGECPTTAGIYAINFVRGLQDVEGQE
HSNDPNSRPLKVSACCKHYAAYDVDYWRTTGSMTVDRFHYDANVTERDMVESYLKPFEMCVKEGDVSSVMCSYNKINGVPACADGRRMRGTIRDEWDLHGYVVSDCDSI
KTIFEKQQWLDDTAIEATAQVMRAGLDLDCGWYYAQYLEETVKKGLIKESDVDEALINNYIVLLRLGWFDGNPLYDNLNASNVCSVDNMKLAASAARQGMVLLKNKGDA
LPLSPQKFKRIAIVGPHANATEVMIGNYNGVPCRYISPIDGLKKYAEVDYKCRGCRCAVGLNLTYEREDWDRTGFDLPGYQNHLIETVANVSKGPVVVVIFSSGTINIS
SFVKSGDVDAIIWAGYPGQEGGQAVADIIFGAYNPGGRLPVTWYPSEYTSQIPETSQQFRPNDELGYPGRTYRFYNGTTQFDFGYGLSYTKFSYSFVNPNVLVTKNVNN
ARQCYNFLPFEDKQLTDVPACQSALVSDLDCTAGDVSVQVSVTNVGKLDGSDAVMLYAKQPQGLIGAPIKDLVAFERVFLKAGESKVVSFEIDACKRLAFVTESAQEVL
PQGEHTLLVGDQKLPVYVHLQKEV
>tr|A0A031JUY3|A0A031JUY3_9SPHN
MARARALPQCNKMPYRGLTGASRRTNSQEFMNPDRRTVLSGIIASAIASGFSFRALAAEEVTRVDDLIKKMTIEEKAGQMTCLADAFRPFNPPNPAAGIQDVKKLSEEI
RKGRVGCLFNGIGVAGARKAQEIAVNDSRLGIPLLLAGDVIHGLKTIFPVPLAEASSFDPVLAQRTARAMALEATAAGLHLTFAPMADVARDQRWGRVVEGSGEDVTLT
ALLSAARVRGFQGRDLRRDDSLLACPKHFAAYGAVAGGLEYGSVDISDETLRETHLPPFGSAFAAGALTTMAAFSEINGVPATADRTLLTDILRGEMGFTGFVFSDYTA
DEELVAHGYAEDDRDAARLAVLAGVDMSMQSGLYIRYLPDLVKSGAVPMGTVDVAVRRILYVKMAMGLFDNPWRSLDEAAEKSRIGAPVHRELAREAGARSIVLLQNDG
VLPLDKGKKQKIALIGPFGDDKANLYGPWAFYGDADKGVDVASGLRGAMVDPDQLTVVAGCAIHGPTDGGIEEAVKAAKAADVVILALGESQDMSGEAQSRTVIEIPPA
QQALADAVAAVKKPTVVLLRHGRALAIHDGVANANAVLATWFLGSEAGNAIADVVFGKVDPSGKLPVSFPWESGQEPFFYDRKSTGRPTVPNGSTEYKARYATTDNSAR
YPFGHGLSYTRFTLDKLQVSDPALRWDGTVSITARLTNSGKRRGSEIVQLYTRDRVASRTRPIREFKKMERVTLEPGESRTVRFTLSRTDLEFVGAGNRRLAEPGAFDV
WVGQSSEDGLHTQITLYAAAPASA
>tr|F7PJW4|F7PJW4_9EURY
MTDTDTPPYLDEALPVDRRVEDLLSRMTVEEKVAQLESVPPRWMDVEDESEMTAKAQLLDEDGNINEDNARELLSDSIGHLTRMGGGGSLEPETAARVTERVQEIAMEE
SRFDIAAVPHEECLSGYMGPKGTTYPQGMGIASTWDPELVEGMSTQIREQLRAIGTHHALSPLFDVARDPRWGRVEETFGEDPYLVARMGSAFVDGLQGDDPEEGISAT
LKHFAGHAISEGGKNRSTVQIGEREFREVHLFPFEAAVKETDADSVMNAYHDVDGVPCTADEWLLTDVLRGEWGFDGNIVSDYFSVRLLKDEHQVAPTYYDAAIQALEA
GLDVELPQIKAYQHLPEAVENGDVAEETIDTAVRRVLKQKIEKGLIGDTGVDPDRVEEAFGPEKNRDYARKLARESITLLKNDDELLPLEGEESVAVLGPKADAPSGQL
GDYAYAAHYPEAEVNREIVTPLDSLQDRLGADAVEYVEGCTTTGPETDDIESAAAAAEDADVAVAFVGTRSAIALSDDDEGIEQVPDLPTSGEGADVTDLELPGVQQKL
VEAVNGTDTPLVVVQVSGKPHSIEWIDQHVPAVLHAWLPGEEGGNGIADVLLGEHNPSGRLPISIPKDVGQLPVYYSRRPNSRNERHVYVDSDPLYSFGHGLSYTDFEY
SDVTLESEEIATTGTIEASVTVENAGDVAGHEVVQVYTHQRWPSQARPVQELRAFERVHLEPGESATVNFEISATQLAFHDRDMNLVVEPGEYELRVGASAADIESTAD
FEIVGEPRELARSARAYFSETEVE
>tr|A0A1F5LCV7|A0A1F5LCV7_9EURO
MALYASLVVVFFMHLEGSCGMQFPDCIRGPSLLTENDVCCPQSSSSDRAAALVASMNITEKLGNLIEASTGSSRLGLPPYNWWNEALHGVGFSDGVDFGVNFSIVTENP
IGEFSYATSFATPLLLAAAFDDEMIYQVADTISTEARAFSNAGHAGLDYWTPNVNPYRDPRWGRGSETPGEDPRRIKGYAAHFLRGLGGDQNSTKKTLTNCKHYAGYDL
ETWGGYSRYGFKANITMQDLVEYYLPPFQQCARDSKVDSIMCSYNMVNNTPACTNSYLINRVLRQHWNWTASSQYIVTDCGVVNETVMRTHYTSSLAEFSALMFETGID
LVCTGGSPAAISEAYMKSLLPEKTIDASLRRQYEALVHVGYFGPNWTDPYVSLDWSAVNTPRSQALARRTAAQSMVLLKNNGTLPIPFMTHPRVALIGVYANATWEMLG
SYFGVPPYYHSPLYAAQQRGLKAVYASGPSTSQNATGNWTAQIEAAKNADVVIYFGGIDVSTSSELNDRSSVSWPASQLSLIKEVCALGKPCVVVQLGDQLDDTALLNN
SNVSAILWASYPGQDGGPAIFDVLTGSIPPAGRLPVTQYPTSYVDQIPMTDMGLQPQDDRPGRTYWYYEDDVLPFGYGLHYTNFTALIQGPRSTRQSSSTQEDTLHLGA
YHINELIADCGSQHLDLCPLPPVAVAVTNTGNRRSDFVVLGFIANKLGPPPHPIKKLGAYTRVKAISPNETRSALLNLSLGDMARVTERGDRVLFPGVYKILIDVPTQS
TATLELLGSETTLESWPQRETNV
>tr|A0A5B1CHC4|A0A5B1CHC4_9BACT
MNNTRLPILAALLAGWITVADAQTETATAETATLTYQDPLAYQDPSAPIDERVEDLLGRMTLEEKVAQLNSISIRGSAATQEGFVVMKKTITERLNNGIGQIENTFDPR
PPRKSVEQVNKMQQYLIDNTRLKIPALIGSECLHGHAGYNSTVFPVPLAMASSWNPELVNEAFNAIGIESRVRGSHEAHTPVLDLGRDPRWGRIEESYGEDTYLVSQMA
LAVVSGLQGGKSGDPGRDHIVSAPKHFAGYGQVVGGRNFAATPIETKTLMDDILPPFEVAVKIAGAQGMMASHCDVGGVPAHGNRWLLTELLRDQWGFKGMVVSDYMDI
KRLEEFHHVAATVQDAARMALIAGMDLDLPDGVAYQELTAVIKNEPELESYLDQSVSRILRLKFMMGLFEDPFVDADVAEKIVGQPSHVALAEQLALESITLLRNPENI
LPLQLDSLDQIAVIGPNAASELIGNYTMQNDYVVSLLKGITDFASDSSTVKYQKGCGLGTFDPKSGYRSASLKDELPMIEKAVKLAANSGVAIVCVGGDTKSAREAFYR
PGVRGDRSTLGLLGNQKELVMRVIETGTPTVVVLMGGRPFSIPEIAEQPCAILNTFYLGQTNGTAVAKVLFGEVNPSGKLPLSVPRSVGQLPVYYSQKATSFYKDYWEE
TSRPLFPFGHGLSYTTFETSNLKLASQEFAMDEPVKFSVQVKNTGKVAGAEVVQVYFRDKVASVVRPEKLLVRFQKVFLEPGQTKELSFELSPKIDLSFTGIEMERVTE
PGEFELTVGGSLDSELKETFLVR
>tr|A0A6P2C3P3|A0A6P2C3P3_9ACTN
MRSPLRRPRAAQLAIGAGALTALALLTVPAATAGPTSTNAAARAGGQAALAATQQAGAGLSAATRQRLASISDKVNSLIGKMTLAEKFGQLEMSGPTGPNGTPGQTLLD
EVRAGTVGSVLDLVGVSNINQVQQAALQSRLHIPVIFGLDVIHGYKTIFPVPLGEASSWDPAAISRDESISASEATADGIKWTFNPMVDISRDPRWGRVVEGAGEDPFL
GAAIAAAKVRGYQGSDYSAPDKMAATIKHFGAYGAVQAGREYASTDMSEQQLRNIYLPPYRAAVDAGAATVMSAFTSLNGVPASANPYLLTTILRDEWGFGGTVLSDYQ

AIQELEVFGYATSGAQAAQLALTAGVDIEMGVQVPSQFSTYTLYGPDLVKSGKVSMATINNEVRHVLNLKFLAGMFDHPFTDPNRVKTAELTPANLAAARTMAGKSMVL
LNNNNGALPLSTSLPSIAVVGPLADNPSDQLGPDVPIGYSSSDLNSVVPVLDGIKTAAPNATVSYAQGCDTSCTSTSGFGAAVSAAKASAVTVIVAGEPSADSSEASSR
SDISLPGQQTALIQAIAATGKPYVVVLMNGRPLTLGWVADNAPALLEAWYPGTEGGNAVADVLFGKVNPGGKLPMSFPRNVGQIPISYNELPTGRPADPNNKYTSKYLD
VANTPQYAFGYGLSYTTFALSNLHVSAASVSTKGTLSVSADITNTGTVAGDDVVQPYTHQDGTSILQPVRRLNGFERVTLAPGQTKTITFTLGPSNIGFYNNLGQFEVD
PGTVDVFVGDSSTGGTQGQFTVG
>tr|A0A0H0XV02|A0A0H0XV02_9SPHN
MQVSRRALLAGISGASLTAAFPMPVLAAVNQASDDYRDRSLSPARRAAALANLMTLDEMAAQLNCPRAADVMSDPAGFEADFPYFAHGIGGVYSASLEAGPEDNARAVM
AMQQEVVSRSRFGIPAFVFEECLHGLLADGATQFPQAMAMACAFRPDMVRQVFEATAKEARSRGSQGCFSPNIDICTDPRWGRSEETWGEDPHVVTVSAKAIVEGLQGA
PAEYLPANRIATSVKHFAGYGQGIGGRNFAPSHIGPVEMQNVVLPPFRAAITEAGSIGLMASHGEIDGVPAHADTHLLNDVLRDDWGFEGYVVSDWDDVRRIHSLHGVA
GSEAEAAIMGLRAGVDIELANNGVYLMLPQLVRDGLLEERYVRRAAERILAAKFKCGLFDMPFADPALAGRLARSTEHKLLARRMAEESIVLLQNEGNVLPLQSSAVRK
MLVVGPNAASVHLGGYSPKPFVGVSALEGLQAYAEQAGFEVEYAQGCAITAGDEGNNEIETDASDESVQADPARNRRLIAEAVATAQDCDVIVMCLGGNESTAREAYFA
GDSRGDRDDLELIGEQNELAEALLALGKTTVAVLIHGRPLSPLVLAENCPAILDAFYPGEQGGHAIASILFGDVNPSGKLPVTIVRNVGQLPGYYYQKPTGRFRNYVFS
DSTPLYPFGHGLSYTSFGYGAPQAERASIGLQDRLRVSVSVRNTGDRAGQDVVQLYIRDSIASRARPIKEMRGFQKVLLEPGEVQVVQFELGPEDFGYRDADGKLLVEP
GEIVIMAGPDSQNLQETRITLV
>tr|A0A402AWY6|A0A402AWY6_9CHLR
MATIPLYRDPQASIEQRVEDLLALMTLDEKLAQLGCYWSTAFVSSGTFDPEVAAEKMPHGIGQVTRIGAATGLHPQESAAFMNELQKIALERTRLGIPIIVHEEATGGF
CHRDATVFPQGIGLAATWNPDLVKQVAEVIRAQMLAVGARHALAPVLDVARDPRWGRVEETYGEDPILIGAIGTAYVQGLQGQDLAHGVAATGKHFLGYAMSEGGRNWG
PVQMGPRELREVYAEPFATVIRNTGIATIMNSYASVDGLPCAGSPAILTDLLRTELGFIGPVVADYASVDMLMNYHRVAATRGEAARLALLAGLDMELPAIDCYGEPLK
AEVEAGRLSQEVVNTAVRRVLQLKFQLGLFEHPYVDTSSASAVFQTPDQRSLARQVVAQSTILLTNDGVLPLSPTIKRVAVIGPGADDERLLQGDYHYPSHLEIIYAAP
QNTEATGLTVPQAGGDYAPGPYFTPHVTPLAGLRAALGQDVELHYARGCDILGDDSSGFAQAVEAASAADVAVVVVAGRSGLLRPVTSGEANDATNLALTGVQPELINA
LAASGTPLIVVVLSGRIHTLASVADKANALLQLFPAGEEGGNGLADVLLGKVNPSGRLPVSMPRSVGQIPTHVGHRAGGDRAMFFNDYIDSPTTPLFAFGHGLSYTTFA
YSDLSIEAKDITEPIKIAFKVRNTGEVIGDEIVQLYGRDQVASVARPDNLLLGFARITLAPGQTRSLAFTIHPSRLAFYNPQMHFVTEPGAFTFRIGASSTDIRLTETV
TLNGPIIEYRQRDIVDTTVTID
>tr|D8QYJ3|D8QYJ3_SELML
MGRRRRRDFSRWNLVLVVVVVVVVILSNARVSTAQPRYACDVSSNASLGSFPFCDTKLGVDVRVQDLVSRLTLDEKVDEMVNAAQGIPRLGVPSYQWWQEALHGVASSPG
VQFGGLAPAATSFPMPIAMAASFNSTLFYSIGEAVSSEARALHNLGRAGLTFWSPNVNIFRDPRWGRGQETPGEDPLLASKFASLYVRGLQGGAYGGSASDGFLKVSAC
CKHLTAYDMDNWKGMDRYHFNAEVSEQDLVDTYNPPFQSCIEDGRVSSVMCSYNRVNGVPTCADRSLLTETVRNSWGFNGYIVSDCDALQVLFEDTTYAPSAEDAVADS
ILAGLDLNCGTFLGKHAKSALQAGKVTEADLDHAISNLMRTRMRLGLFDGDLNTRPYSSLGATDICSNDHQQLALDAALQGVVLLKNDGSLPLSTALKTVALIGPNANA
TYTMLGNYEGIPCKYVSPLQGMQIYNNNILYSPGCRDVACSEGDLVASAVEVATKADAVVLVVGLDQSQERETFDRTSLLLPGMQSQLVSNIANAVTCPIVLVIMSAGP
VDISTFKDNSRISSVIWIGYPGQSGGAALAHVVFGAYNPGGRLPNTWYHEEFTNVSMLDMRMRPNPPSGYPGRSYRFYTGTPLYNFGDGLSYSTYLYKFLLAPTRLSFF
KSNTRNSRDCPTVNRSEAEFGCFHLPADDLETCNSILFQVSVEVSNLGPRSGSHSVLIFSAPPPVEGAPLKQLIAFQKVHLESDTTQRLIFGIDPCKHLSSVRRNGKRF
LHSGRHKLLIGNAVHILDVSKV
>tr|F0RST1|F0RST1_SPHGB
MQQNRERAEQLLHSMTIEEKVAQLVSAWLEIQEDGSFSIREYGHKDHRNGNLYQEVLGKGIGQLTRPFGTMANDPHKQAKAINKLQHYLVTETRLKIPAMLHEECLTGA
MVKGATIFPSALNYGSTWDPALIGRAASAIGDELRSLGIHQGLAPVLDVARDARWGRLEETFGEDPYLCGVMGIGYVKGLQGAKQSPLATLKHFVGHSFSEGARNHAPV
HCGMREIRNTFALPFEMVVRNAHPAAVMPAYHDIDGIPCTSNHSLVTDLLKKQWGFDGLIVADYEAIVQLVNDHQVANDMAEAAALAFNAGMDIELPGFTVFKEGLIEA
LYRGLVTDEALDQSVLKILQEKLRLGLFENPYIREEAIDLGSEKNHILAREVAEKSLVLLKNDGTLPLKKGLKVALIGALADHPYAMFGGYAPPVHLQGSHGPEETVPV
LAKTIKTALQDVLGNEQVLFEPGCMLYESNVERAVFFPGDVQEGQGTQVHELSSDVSRIKLACEASRKADVTVLVVGDLAGLFGQGTVGEGSDAASFTLPGVQEQLMQE
VLQTGKPVVVVLVSGRPYTLDKAVTDARAILCTWLPGEGGGEAIARTLVGLNNPSGKTPLSFPKSVGSMPYFYNHTKKAGGLPVQKQFGTLYPFGHGLSYTSFTWSDFQ
VEQSQITNTGEFKISLQVQNSGTVAGDEVVELYIHDKVASLVRPVKELKAFARVSLKPGEKKKVVFSLPAELFSFIGERMERVLESGSYELFVGKSCDDIVFTNELSIL
GEDKVLSNSWRCLSSSSIVSLS
>tr|A0A317XSE7|A0A317XSE7_9BASI
MTGNGGWESHFNKAKHIVSQMTLEEKANMTIGLGLAWRCEGHSGSAERLGIPEFCYQDGPAGVRASDFVTVFPAGVTTGATFNRDLMYRKAKAIGEEFRAKGVHVALEP
VTGGPLGRSPYQGRNWEGPGSDPYLVGEYAYQTVAGTEDAGVIATSKHFILYEQELWRQLDGLDDPWRHVSYQNKRTYSANADDRTLHELYLWPFMNAVRAGTGAIMTV
YNRVNHTQGTESSYLLNDILKEELGFQGFAVSDWYSAYDTVNTFNAGLDVIQPGGIDGGKRPQTAAHTVEAVRNGSMSEARLDDAAIRRLTQFYRFNHDDPDYPTVSFK
DMDLNSYDEDGLFINHNRDVRGDHDQIAFEVAQEGITLVKNTQSEGKLDRHGRPFGLPLTKGQKIGVFGSDAGPNPNGLNACQNWLSIGTQYCHGNSTNNGTLAVGWGS
GGGYFTYLVDPLSALSQRIRSDRVGGIESNLNNAAPHDAHYRKQIANQVDAALVFVQASSGENVDRFDLELFAEGSKLVQEIASWNNNNTIVVMHNTQQVLIDEWFNHPN
VTAVIMPHLPGQESGNSLVPVLYGDVSPSGKMPYSMLKRADAKHYPTIDWSHNPDPHVNFDEGLFIDYRQWDKLDLEPLLEFGFGLSYTTFEMDSLSVTPKKGHYPTRV
PNTQKTVNPPGGPDYLWDYLASVSVKVKNTGCMDAKEVAQLYIRYPSSANTPPKQLRGFDKVEVPKSATRTAKFSLTRRDFSVWDVVQQKWVVEDGEYEILVGNSSRNL
PLSYKLTLEDGNIVWGRHEEL
>tr|E1R2R1|E1R2R1_SEDSS
MQDYHDQALTLCKKMTIHEKLAQMYSVWFNIRPDGSLWLKDHTGMIVTESPVRFEELLKDGVGEITRPLGSQPIDARTAVKALNSIQEFLVKGTRLGIPALAHEECLAG
LMAKGATLFPSGISLGALWDEGLVEKIARAIGDELYSVGSRQGLAPVLDVSRDARWGRTEESMGEDPYLVGTLATAYVRGFQGNDGRLLATLKHYVGHSFSEGARNHAP
VRMGEDELSDVMLLPFEMAVKLAHAASVMPAYHDIDGIPMHASLTYLRDILREKWGFDGIIVSDYSGIGQLCHDHRVAEDLASAACLAIEAGVDVELPGHECYKSGALA
AIERGDLPVALVDGCVTRVLEQKIRIGLFEHPYADVDAISLRSDEHLALAYEAAVKSMVLLKNDGILPLTTGKKIALIGPLADDPLCFFGGYSFPVHHILSSLEDRDEG
AMTLKEVFESVPGALFSYAKGCDILSQRPKDAPVFPGDVHLDGSAQHSYVSHDKSGFPSAIEVARSADVVVLALGDLAGLFLAGTVGEGSDASSLVLPGVQQELLEELL
GLGKPVVLVLLSGRPYSLKIAAERCSAILQAWLPGQKGGQAVVDILYGRQNPSGRLPVSIPKAAGAMPFFYNHKIKSAGTPIQLDFGATYPFGHGLNYTSFSFDAFRLD
DKRVAIEGEITGSFIVRNSGERDGEEVIQIYVRDLYASRVRPVEELKGFKRVHLAKGESALVRFTVPVDMLNFTKGNFVRVVEAGAFEISIGRSSKDILFTDTVQVTGE
DRILPEQWNMQSSITLSPCAF
>tr|A0A0L6JSQ8|A0A0L6JSQ8_9FIRM
MLIGEKSIRKKSKSVLSIFVSAVTVSSLVFVNAPTNVKAANVYQDPSQPVESRVKDLLSRMTLDEKVGQMIQPERHTATADDVKNYYLGSILSGGGNVPLSNTPTGWCD
MTDAYQKAAMSTRLQIPFIYGVDAVHGHNNLYGATIFPHNIGLGAANDEELVYKIGQITAKEVRATGVHWTFAPCIAVPQNEKWGRTYEGFSENTDIVTRLGVAATKGL
QGDNYLTDLTKNDKILGCIKHFIGDGATTNGVDASDSVLTEDQIREKYLPPYIEAIKAGARTLMASFSSINGLECHGNKRLLTDILKNELKFDGFVVSDYEAIKDIDKA
NFRNCVKESVNAGVDMYMEPRMWKTVITHLKDLVGNGEVPMSRIDDAVTRILRVKIQLGLFEKPYADRSLMSQVGSADHRAVAREAVRKSLVLLKNDNKVLPLAKSGKK
IFVAGKNADDLGNQCGGWTITWQGKSGNITQGTTILQGIKSAVNPGTTVTYNLNGYGAQGHDVAVVVVGETPYAEVKGDRTDVALSADDIQTINNVKSAGIPMVVVLVS
GRPMIVTDQIKDSAAFVAAWLPGTEGNGVSDCLFGDYDFSGKLPMSWPSSNAQIPVNEGDGKTPLYQLGYGLKMKADEGYKLSGYVSPDFAVISAGTDLKQGFKVEVVG
KQLNAVTDAKGYFEIKGLPLSTDGYSLKISKTGYLTRTISNILLTADKELSSQSSPVEMWVGDIPKNGIQDDAINMADVISLAKVFNAVSSDAHYESQFDLNLDGSINM
SDIILLAKHFNATAANYPAV
>tr|A0A0S7BST2|A0A0S7BST2_9CHLR
MGITPKQFYKNYVPIETKVSALMEQMTLDEKIAQLGSCWFYELQSNGKLDMQKVQKRFSNGIGQITRLTCTSILPPVQAAQTANLLQKVLLEQTRLGIPAIFHEECNSG
SIALGATIFPQSIGLASTFQPELARLMAAEIQKQLRAIGVHQGLAPELDVARDPRWGRIEETFGEDPLLISQFGMQYTRGLQGADFHHGIIATGKHFVGHSLSQGGLNC
APVQVGKRTLWETYLMPFQAAIRDAGLASIMNAYPELDGEVAAASKNLLTDLLRNQLGFQGLVVSDYEAIIMLHTYHRMASSWAEAAAAAMNAGIDVELPTTKCYAEDL
IHQIEAGIVHMERIDQAVANHLTMKFKLGLFENPFVDEGKVLEVFETQQQRDLAKNIAAKGMVLLTNNGILPLSKNIKTIAVIGPNADDERNLCGAYSYVGMIDNWIYS
KIPDSSFINLDRDSLIKDQPIVITPLNAIKEKLPNSKILYAKGCDVNSQDRSGFPSAIQAAQKADAVILILGDRSGMIPDCTCGETRDSADLKLPGVQTDLAEAIFTVG
KPAVVVLINGRPLAIPDIVKRADAILEAWIPGEEGGTAIADILFGDQNPGGKLPITFPRSVGQVPIFYNNKPAGMKSNWHIDYVSESVKPLFPFGHGLSYSHFRYSDFS
IDKPQAGMDETVIISCTVENIGSIAGDEVVQLYICDEYGSIPRPVKELKGFQRISLAAGESRTIHFLLPVNMLAFYDENFDLVIEPGRILVMIGSSSEDIRMQGAFEIT
GDRKMMIHERIFICPTKVES
>tr|A0A1I4K1N3|A0A1I4K1N3_9FIRM
MTKFDLKGYTAAEKVEILISEMTLEEKVAQLGSVGPDKIMEAGKFSQQKAEKYLKNGIGQITRIAGASALEPEKAAELANQVQKYLAEETRLGIPALIHEECLSGYMGK
GGTTFPQSIGIASSWEPELLKRQTDVIRKQLRSIGAHLALSPVADVARDLRWGRVEETFGEDPYLVAEMVNAYVAGLQGEKLSEGIIATLKHFAGHSYSEGGRNHAPVN
LSERELRETFLFPFEAAIKTAKAGSVMNAYHDIDGIPCAASRQLLTDILRGEWGFDGIVVSDYWSIKMLYNEHKIAVDLQEAGIKALSAGLDIELPETECYGHNLVKAV
KEGLISEKIIDQAVSRHLLTKFKTNIFEKRYVNTDQINSLFETPAQRELARESSRKTMVLLKNESAILPLSKQIESIALIGPSAASTRNLLGDYAYSAHVDSKEDAVDI
VSIMAGIKAKISSETKLNYAAGCNIMDQNKDGFSQAVKAAQASQVAVVVVGGKSGLSGMGENENSEDEVVDFEGGAFLSELQNTTDTTGEHHDRTSLNLPGVQEELVKE
IVKTGTPVIVVFVNGRPLSSQWIAENAAAILEAWLPGEEGGNGVADILFGDYNPGGKLPVSIPKNVGQLPIHYNRRHMSHYRDYVFTGNRPLYPFGFGLSYTEFAYANL

QISPQKFSGNSEITIQAEIENIGQCSGTEVVQLYVQDKIASLTRPLKSLKGFKRVELAAGEKKRISFILKAEQLAFYDKDMNLVVEPGEFEFMLGSSSTDIRLTNLVEL
IGDKIELTAERNFFSKVKLN
>tr|A0A1U7LSJ7|A0A1U7LSJ7_NEOID
MMFLQVINLSLLSLVSSTTHVHHQRELPVYRDPSKSIDQRVEDLLSRMTDQEKLSQLMQGDQINWYNDTSGAFNKTGLIDSMNSKGGSFYVGEYLKTIDMYAEAINYGQ
HVRAVRRHEIDSQNSIDGLGLHGLVMTNATIFTSPIGLGATFNPQLIEKMAEAIAEEAAVVGINNLFSPNVDLARELRFGRTEETFGEDPYLTGEMAYAYVVGLQRSRK
VAAMPKHFAAFSSPQGGLNLAPVPGGERELRTIYLPPFKRAIMDAEATMIMSAYDGIPSIANHHLLTEILRNEWGYKYAVMCDSGATDLLSSQHGLCATGDEDCIATIS
LNSGVDIEMGGGSFNYKQIPRLVSEGKLEQKAFDDAVRRILSAKFKLGLFDTNHKNTSFSNSTGNYFKVINTQMKKDLAKAIDSEAMILLENNGVLPLDSSSLKSIALI
GPFANANNYGDYVVQGSKYRGITPLDGIQNLIKTKFPNIEVKYTEGCKQWSNDQSGFAAAEQLAQEADVAIVFVGTWSRDQSELWAGLNATTGEHVDQSSLDLVGAQAD
LVRAVSAKAKKLVTVLTSGTAITEPWITNVTHALLQTFYPGEQGGAALADILFGLTNPSGKLPISLPRSVGTTPAFYNYLKGARPIFPGFIQTDGALNFGHQYVLNSPV
PWYPFGHGLSYSNFTIKDAVLSATNVPRNGKVEISVTVQNNGIYEGKEVIQAYVIDVVSSVVTSERSLRAFNKVSLKPKESTSVQLTIDVSELAVWSIKNEFIIEPGQF
QILVGTSSAQIFTNSTFYVI
>tr|A0A1C6LN67|A0A1C6LN67_9ACTN
MAENLSRRSVSRASAGAAALATSASVPPLTATPAADAPATGEVAAGGGPAPRAAAALSAESHERTEQRVRELLARMTVEEKFGQLQQLTWNPDTGPGEGQNEQATEAAA
EGRLGSVLNITGAKECNALQRHAVEESRLGIPLIFGLDVIHGYLTTFPVPLAQGASFDPEVVMRDAEVSAREAASWGVHWTFAPMADVSHEPRWGRVAEGNGEDPYLTA
QLAAAKVRGYQGEDYAADGRLAACVKHFVGYGFPEGGRDYNTVDISERRLRDVALPPFKAAVDAGVATVMAAFNTVNGVPAHANPHTLTRILHEELGFDGFVVGDYNGV
QELIPHGVAADGADAARLALGAGVDMEMVSTTYAEHGKELLEAGKIDQRRLDDAVARILRVKVRLGLFENPYTDEDGQIAEPTAAARRHARETAASCAVLLKNDDRTLP
LSKDTPSLAVVGPLGDDTKELHGTWAGPGSRMFPAVSVLEGIEKAAPGAKVTFSRGCDVTGSGTGGLAEAVAAVEASDAAVVVVGEKAGHSGEAAVRSDIGLPGVQEEL
IRRVAETGKPFAVVVLAGRPLALSGVAEEAPALLYAWHPGIEGGNAVADLLFGDVAPGGKLPATLPRAVGQTAVYYGHENTGRPYAPDDPYTSKYLDLPHGPLFPFGYG
LSYTTFRFSGLELSEKSVSAQAIREGRATVTVSVTVENTGDRKGDETAQLYVRDKVASLAQPVRRLRGFERMTLERGERRTVTFELGAADLGFHTNDPRGELLVEPGEF
EIYAGGSSEAELRTTLTLT
>tr|I2FNF0|I2FNF0_USTH4
MLFRRGITLSSLARMHLSFSFPSCAPDSPLSKIPDICDPTIPFYTRATSLVNQFTTEELLNNTINYAPGVPRLGIPNYQWWTEALHGVAKSPGVNFDLSDPHAEFTSAT
QFPQTINLGATFDDDLYQQIASVIASEVRAYNNAGKAGLNLYSPLNINCFRDPRWGRGQETVGEDPLHMSRFAVSIVHGLQGPHAQNEAEGNKLTVAATCKHFLAYDLE
QYDRGERYQFDAIVSKQDLSDFHLPQFRACVRDGGATTLMTSYNAVNNVPPSASKYYLQTLARQAWGLDKTHNYVTSDCDAVANVYDGHRYAQNYVEAAAKSINAGTDL
DCGATYSENLGAALKQKLTDIATIRRAVIRMYASLVRLGYFDDPASQPLRQLTWKDVNSPSSQRLAYTSALSSITLLKNLDSTLPIKQKPTKIAIIGPYTNVSTSFSGN
YAGPAAFNMTMVHAASQVFPDAKIVWVNGTDISGPYIPSDAQDAVKLTSDADSVVFAGGIDASIERESHDRKDIAWPPNQLRLIHELSQSRKKDKKSKLVVVQFGGGQL
DGASLKSDDAVGALVWAGYPGQSASLAVWDILAGKAVPAGRLPVTQYPASYIDGLPESAMSLRPKAGYPGRTYKWYKGVPTYPFGHGLHYTTFSASLAKPQPYAIPTTP
AAKGPEGVHAEHISVADVQANIKNTGKVASDYTALLFARHSNGPAPYPRKTLVGYTKVKNLSAGEESSVTIKITQAALARADEEGNQFLYPGSYQLELDTEEHRLASTT
LVLTGEAVNVIPWVSGDMS
>tr|A0A5C5WAK1|A0A5C5WAK1_9BACT
MKLLIVLILLTTNMSTVATQPAYRNPSLPVDRRVDDLLARMTLEEKVAQMVCVWNEKVTKLLDDQGGFDPARATEHFGHGHGIGQVGRPGDAHGGATPRDFAELTNAIQ
RFFVENSRLGIPVLFHDECLHGLVGRDATSFAQPIGLASTFNPELVRRLYEMTAREARACGVHQALGPVLDVARDARWGRVEETFGEDPHLVGEMGLAAVRGMQGDNQL
RDPDRVIATLKHFAAHGQPESGTNCAPVSISERHLREIFLTPFHKAIAEGGAQSVMASYNEIDGVPSHANSWLLRDVLRQEWGFTGTVVSDYYAISELHHREGLFGHHV
AADAAAAATAAVRAGVNIELPEPDCYLHLVDLVRRGVVDEEEIDELVAPLLAQKFALGLFESPYVDPPAAEAVVGCEEHRALSLEAARQIITLLKNQSDTLPLQADRLK
TIAVIGPNADRVMLGGYSGKPKQFVTVLKAVEQAVAQSVNVLYAEGCAITVGGGWFEDSVVRADEEEDLRKIAEAVSIANQADLVIFVGGGNEQTSREAWSAEHMGDRT
DLQLVGKQDELVEALHTTGKPIVSVLFNGRPLAVKDLADRSAALLECWYLGQESGTAVADVLFGRCNPSGKLPITIPRSVGHVPAFYNHRPSARRGYLFDDISPLFPFG
FGLSYTRFAFDEPSLSDPVIDTDGSTTVRVRVTNTGSYTGAETVQVYVRDLVSSVTRPVIELKNFKRVTIPAGESRDVELTLTPAELAFWNIDKKLVVEPGEFDVLVGP
SSVDLQTVRLRVNPRKR
>tr|A0A137NZN8|A0A137NZN8_CONC2
MLTGIFTQLTLLLITGIRADYPTKPVCDVGSFEAQMNAKVDKILSGMTLEEKVGQLTQININNLYDAKNDKLNTTQLDYYIKQRLAGSFLNNLADSTDLNAAVPATWVR
VMNEIQDYIQKNTRLRIPMIYGLDSVHGANYIRGATLFPQQIGMGATFNRTAARVWGEITAKDTRAVGVHWNFSPLADIAVNKQWGRVYETFGEDPYVAGELGKQVVKG
YQGENVCDLKRADKVAATMKHFIGYSSTKSGHDVDGSWMSKRVLDEYFVPPFQALVDSGVATAMESYSDIDGDHVAKSKKILVDLLRDQMGFKGALVTDYEQIFKLNFQ
HHVAKSLNDSLVMAMKLGTIDVSMVPHNGQFFDMMVSLVKSGRLPESTITKNAKRVLQLKYKMGLLDNNGQVDPNSDLIKTIGSAADRTASLNMARESVVLLENRNKAL
PLNPSSKVFVTGPCADSLNYLTGGWTFAWQGAGKDDFYEGRGTTIAQGLKNIGGSNVNYLPSVDINGKWFNNTSADQIVGAAAASDVNVVCLGETNYAEFIGNVDDMRL
PEGQTDLVKLLSKSNKPLVLVLSQGRPRSFDNVVDLPSAILSSFLVGPEGGQAIAEIIYGKVNPSGKLPIVYTNKASLNTINNYRRFSDSYRVQWDFGHGLSYTTFNYT
NLSLSSSQVQPNGSLEVSVTVTNNGTVAGAESILLYATQIYRTISPEFQRLRGFEKIYLNPNESKTVKFQILPKRDLSFIDVDDKRALEMGEFTVKVGGLTKNFNLTLT
ASPNNPTFLEAADHILV
>tr|A0A231RT81|A0A231RT81_9BACL
MELYREEAASIEARVEDLLGRMTLKEKVGQLNQRMYGWNAYRKTENGFELTEAFMEEVAAGGGMGALYGLFRADPWSKVTYENGITAADSAKVANQIQRYLIEHTRLGI
PVMLSEECPHGHQALDGTLTPTNIGIGATWNPQLAEQAYGQVAAEIRARGAHLGLVSTLDLLRDPRWGRSEECFSEDPHLTAQFTKAAVHGLQGCRQSGGEAAGQDRAE
QDKLAPNKVVAVLKHFCGQGAGEGGMNAFPASIGERELREIHLPGMEAGVEAGALGCMAAYNEIDGIPCHANRKLLTGILREEWGFDGIVMADGTAVDRLLALTGDYES
AAALALIAGVDISLWDKAFSTLEQAVLQGKVQVSFIDRAVRRVLQLKFQLGLFETPYAAEETDAEIVGNETARQINLQLARESVVLLKNENRLLPLGADLRQIAVIGPN
ADALYHQLGDYTAIQRPNSGTTVLEGIRELAGDAKVVYAKGCSVRGDSRDGFAEAVELARQSEVAVLVMGGSSARNFELKFDINGAAIVEDGQPSDMDCGEGVDLADLR
LGGVQSELIRAIAATGTPIVLVLIQGRPHAIADVAPDCQAILSGWYPGPEGGRALGEILFGAVNPSGKLAVTIPYSSMSLPAYYNRKDQGYEPRYADCPRPVLYPFGYG
LSYTEFAYSNMRAASSERTLRQLAAGERFEIAIDVENTGTMAGAEVAQLYIKDMEASVTRRVKELKGFRKIWLEPGEKRTLSFRLTAKELGVWNVDMANVVEAGRIKVM
IGGDSEPSCSLMLRVTE
>tr|A0A1B6Z9V1|A0A1B6Z9V1_9SPHN
MSLEEKVGQILQADIASVTPAEVKSYNLGSVLNGGNSAPGGGKVASVEEWIALADAFWEASTDKTDGGLGVPLLWGTDAVHGHSNIQSAVIFPHNIGLGAARDPDLLGR
IASVTASEVRATGLDWNFAPTLAVAQDDRWGRTYESYSEDPSIVASYSGAIVKGMQGEPGSDGFLKGTKVISTAKHFVGDGGTENGIDKGDTQGSIDELWALHGAGYPP
AISADVQSVMASFSSINGKKMHGYRELLTDKLRGELGFTGFVVGDWDGHAEIPGCTPTDCVAALNAGVDMYMAPDSWRGLYNSLLVKAKSGELDTARLDEAVLRILTVK
VRSGLLDGVKPSLRAASGADKIGTAQNRAVGREAVRKSLVLLKNNKGILPLNPSRNILVTGSGANSIQQQTGGWTLNWQGDGNSNDEFVNAETIYEGISKAFKAHGGEV
SLSSNGTFESRPDVVVVVFGEEPYAEYRGDRSDLVFEGRDGENLALIESFKEQDIPVVAIFLSGRPMWVNPLLNASDAFVAAWLPGTEGGGVADVLVGDAQGNARHDFV
GRLSFSWPSLGDGNPVNGANAKGALFPFGYGLDYTRVSEFATLSEDPGVDLSKSFDGNILNRGDAAGKFSLYLGDNTNANVPAPALISKSLSGAIATSGVDYKAQEDAR
KISFSGAGKANLSIRAPRPLDLSGRESDALAIEWRLDSAPAGQLNIGMSCGEDCSGMVDAKSVFNFSNKNGWREDSISLKCFANAGLDLSKVNAPFVLEADSAARIVVH
RVELVEANATTKSCAN
>tr|A0A1I6LUV7|A0A1I6LUV7_9EURY
MADRGRSGSPELTRRRLLRASGSAAPLLLAGCSDLEFGGDQATDTTTGSATSGPDGTGSTEDEALRRRVEDLSIREKAGQMTFLALDAQVSEDRARDVVADPGIGGILY
GGANPGSFDPEAVASKLNEYQQIATTETDHGLGMVAGIDAVHGNATNEAAVVFPHNVGMGATWRPDLVRKRAAVTSRSLRAMGFQWNFSPVADVLYDPRWGRYYEGFHE
SPSAVSSFVSAAVEGLESSGDGTATVASSVKHFAGYSMPDAGNDRDDARIPLRDLREKVFPPFEAGVEAGAETVMANSGAVNGTPVHASEWLLRTVLRKQMGFEGVVVS
DWADIRNLISEHAFLGHGQFEEGVKVVVNAGVDMYMAAESPGQFVDAVETHVQDGEIPEARVDDAVYRILRLKKRLGLLEDPTVDTNAVTDRVVTQGDFELARETAEQS
MTLLTNDDVLPFDGPDTVLVTGPNADDPKSQHGGWTLGWQGLATETTPPTTTILEGIRERAPPDTSVEHVPTDRGAFSEHDAVADAAAAADVVVAVLGEGAYAETDGDV
ESLALPQGQRELLSVVAEAAAPTVGVLVAGRPRGGDRAFDALDAALMAYYPGSEGGPAVARTLFGDVNPGGRLPFYWPDSTGDVPVASTVRQITPGDADRFPIGHGETY
TEFDLTVPAVEPSQVDPAETDTVRIAYDVENEGDRPGDHLAGALIYRNGSDPTTKPWIRVGPFERKTIAPGERQTVTLDVPLDRLAVVGGDVRGRGDWVVPAGTYLAYI
EGQETQFTVTQTVSLD
>tr|A0A1J0KSY9|A0A1J0KSY9_9GAMM
MKFKKIITVIFMGTAIVSCSNIEDNIFSPNNTTTFDKNYESQIDELLDSMTLDEKLGQLNLLDSGDITTGEGANSNVAKLIEEGKVGGILNLTSAEKINKAQKIAVEQS
RLHIPLLIGLDVIHGYKTTFPIPLGLSSTWNIDLIEKTARIAATEASADGINWVYSPMVDISRDPRWGRVAEGSGEDPYLGSLIAKAMVRGYQDNDLSLNNTVMACFKH
FALYGAVEAGRDYNTVDMSKVRMYNEYFPPYKAAVEAGAGSVMAAFNDINGVPATEDKWLLTDVLRKEWGFNGFVVTDYTAIPEMVNHGVGTDLQDVSALALKAGVDMD
MVGQGFIKTLKKSLKEEKISIDDINLAVKRILQAKFELGLFKDPYKYSNDKNRAEKEIFTKDNRTFARQVGSESAVLLKNQNNLLPLAKKGTIAIIGPLGNNSVNMAGT
WSVSTDQYKSISLLDGVREVIGDKAKVLYSKGSNLSYDEEFEKNVTMFNKPIPRDNRTDKQLLDEAVKIAEKSDVIIAALGESAEMSGESTSRVDIEIPKAQKDLLKAL
LQTGKPVVLVLFTGRPLVLVDEATTVPAILNVWFPGSEAGLSIADVLFGDVNPSGKLTMSWPRDVGQIPIYYNHKNTGRPLSKDDGKFEKFHSNYLDVPNDPLYPFGYG
LSYTTFKISKPELSKNSIGPNDKLVITTEVTNTGNYNGAEVVQLYIHQKVRSITPPVKELKGFKKIFLKKGETKTVKFVISPEDLKFYNNDLEFVYEPGEFELFVSDSS
DHKFTNTFSLTKEEDN
>tr|A0A5E7ZR05|A0A5E7ZR05_9SPHN

MVNGEALRRKHAGISEGSFSMTDIRISRRAALLGAGAIAAWASSPARALFQATDSLAVPAFIDALIAKMTLEEKAGQLQLMASAWGGGVATALNPPSTGPSFEGQIADA
VAGKLTGVFNGNGAEMARRMQTAVMKQSRLKIPLIFAADVIHGHRTIFPVPVGEAASFEPDLAMRTARAAAYEAAGAGIDWTFFPMVDIARDQRWGRTMEGAGEDVLVG
ELFAAARVKGFQGKDLAANDAVMACIKHFAAYGAAESGLDYNSVDLSERTLREIYLPPYEAGFAAGAMSGMASFNEINGIPATANKWLMQEVLRDEWKFPGIIVSDYTG
DEEMIAAGYARDGRDAARLAILAGVDMSMQSNLYTLHLPELVRSGAVPQAVVDRSVRRTLAVKNMLGLFDKPFGRIDLKREKARSRTKASLALSREAGRKAIVMLKNDG
DLLPLDKSAKIALIGPFASGQHDLNGPWVVYGDNAQAIDLATGIRNAGARNVSVTLGSGVEAPLAGGIDAAVAAAQAADIVVLAIGESEGMSGEAQSRADIVLPEPQMA
LAEAVAKTGKPIVVVLKNGRALALHGAVKDAPAILVTWFLGSESGNATADVLFGVYSPAGRLPCSFPYESGQEPYHYDHKATGRPAPDGPRVPYKAQWRSAPNAALYPF
GHGLTYGKVGYGALTVAPTMAWDGRLEVSTTVTNSGKRACEEVAQLYIHDRTASITQPVRKLKAFHKVALAPGASETVKFTLTREMLQFLGEDLKPTVEPGTFDIWIAP
SAQADGAKGSFELVKG
>tr|A8F439|A8F439_PSELT
MPKVPAYKNASLPVDIRVKDLLSRMTLDEKVAQLGSVWSYELLDDQGNFSNEKAEALLKNGIGQITRPGGATNLSAKEVARLINQIQKYLIEQTRLGIPAIMHEECLTG
YMGLGATNFPQAIAMASTWDPELIEKMTSTIREDMRQMGIHQGLAPVLDVVRDPRWGRTEESFGESAYLVAKMGVSYIIGLQGKDIKNGVIATAKHFVGYGASEGGKNW
APTNIPERELREIFMFPFEAAVKEASVMSVMNSYSEIDGIPCASSKELFTGVLRKNWGFSGIVVSDYFAIDMLREYHRLAKDKKEAAKYALQAGIDVELPKADCYTTIR
ELVEQGLISESTVNQATSRVLQIKFMLGLFDKPYVDVEKIELKKHYSIATEIARKSIVLLKNDGILPLKKDAKIALVGPNASEVRNLLGDYAYLAHIKVLLDSVNQTTF
NAPKFNLKNVEKVINESIEKIPSILDSMKAEGVIFTHAIGCDILNSSTEGFSEALHAVKNADIAVVVVGDRSGLTEDCTSGESRDSANLKLPGVQEELVLEIAKCGKPI
VLVLVTGRPYSLKNIVSRVNAIIEMWLPGEVGGMALVDVLFGKVNPGGKLPISFPRSAGQIPVYHDVKPSGGRSHWHKDYVDELVEPLFSFGHGLSYTKFEFSNLVIEP
QKIPSDGQVTIKVDVKNSGEVEGDEVVQLYLTREHASVTRPIKELKGFKRITLKPGESRTTVFKIHTDVLAYYDRGMELVVEPGVFKAMIGSSSTDIRCFGEFEITGKK
RKLGKNRKFFSDVEVI
>tr|D8SVP2|D8SVP2_SELML
MAKASFCSHWILLLLLEVALLTHQVVAGQAPYACDQRNATLLQFGFCNTRLPTSTRVEDLISRMTLQEKIIQLVNNAAGIPRLGLPRYEWWQEALHGVAVSPGVKFGGK
FPGATSFPMPILTAASFDAVSTEARAMHNYQRAGLTYWSPNVNIYRDPRWGRGQETPGEDPLLSSKYATFYVRGLQDTNLGGDKLKVSACCKHMTAYDVDNWKGTTRFK
FNAIVTQQDLSDTYNPPFQSCVEDAKVSSVMCSYNRVNGVPTCADYNLLSATVRSSWNLNGSILLTCEVLLLYLPCSYIVSDCDSLQTFFDNTNYAKTAEDVVADALLA
GLNLDCGPFLAIHTQSAITNGKITEANVNQALRYLYNVQMRLGLYDGNPRSQPYGNLGPQSVCTGENQQLALDAAKEGIVLLKNNGNVLPFSKSNIRTVAAIGPHAKAT
RAMIGNYQGIPCKYTTPHDGLSAYARVVYSAGCSDVACYSDSLIGSAVSTASQADAVVLFVGLDLNQEAEGKDRTSLLLPGKQQELVTEVTKAAKGPAVLVIFSGGSVD
VSFAKYNNKVQGILWAGYPGEAGGAAIAQVLFGDHNPGGRLPVTWYPESFTGITMLDMNMRPDASRGYPGRTYRFYTGQSVYNFGYGKTYSKLSHKFKEAPLSLGFPEA
AAVKRSCDGNLTCFHLNAHDEITCSTLTSKVRILVHNKGDRPSNRAVLLYSSPPNAGRDGAPIRQLAGFGKVSVAPGAVENVEIEIDPCKHLSHAGANGVRILHGGIHT
LAVGNARHPLPILLQS
>tr|A0A1B8G8J9|A0A1B8G8J9_9PEZI
MLFQFFPSCRQLVTGAALASLMGTGHTAASPAQNEPRTNAIYQDASKPVKARVADLLSRMTFLEQLAQTRNVGGILGENASFDNTTVYSFNNGQGGGSISFGNYQNPAY
LAAATLEDVVLQFQKNDRLHVPLINVADSVNGVTLLNTTLFPATLSMGQSWNIDLYGKVVQAMSIENHAVGIHWVLSPELDLAVEPRYGRVGEMYGEDRYHVSRFGVAY
VKNMQDTDSKGFSRVATTVKHWVYGSSLGGINEARILGGINDFYNVHSYPYMAVFEEANPMALMPSYSSYDNVPMTTNIQYTKNVIRDLLKFDGVIISDYAAISQVLST
QHTANNIQAAGLKALAATVDHELGPPNQSGMEALSILSKNPTVAKAVREAARRVLTLKFLTKTFDEPIPDLKKLNSSLRTADAMQRNLDITRESMVLLKNDGILPLKSS
LLLKVAVIGPMADIINPGSYAASDYSTGSTILSGIKKISSDVTFSRGCFRNNDTDFDTMKAEAVANARNAGLAVVALGSVAQIVDTNANDRTDGEGFDHANLDFPGPQN
ELLKAIVETGTPVVLIVSGGQAFSMEYAANATNAIIHTFLQGELGGDVLAEIITGKTNPSGKLTVSIPRLSSAVPIYYNYINSDRKQVGWQQVYTDYQSPVLDRLALYP
FGHGLSYTKFDISGVTVTNNTSTHGQITVKAIVKNTGSVQGKEVVQVYFNQFAPDIERPVKNLIRFTKIDLKPGASTTVSFSIPVSELGYFVNGVKQVDADQYTIFVGS
SSDEADLTATKIVVK
>tr|A0A1M5MU27|A0A1M5MU27_9ACTN
MSDTLDPRPATGPSRRRLLGAAAGATALGAASATGLLGATRADADPAADRGHGGSRGSADPALPADRRRPGHGRPDRATERRIDALLRRMTLAEKLGQLQLVNTADLAK
AGLGTAGGVGGLFSVTDSAVLDAVQHQAVEKTRLGIPLIFGLDVIHGYVTNFPIPLGTASSWDPEVARTDGRISAAEARASGQHWTYAPMMDVTHEPRWGRIAEGNGED
PFLTAAFAAAKVDGYQGSDYSARDRLAACMKHYVAYGGAEGGRDYNTVDVSLQRLHNLYLPPFLAAVRAGVATAMASFNTIAGVPAHGSEYAIREVLKERYDFDGFVVS
DYTGIQELINHGLAGGGADAAAAGLNAGVDMEMVSTNYVDFGVALLAAGRVTRREIDDAVRRLLRIKFRLGLFENPYTDPKRAVTTISAANAKAARAAAARSMVLLRNE
DDVLPFGSGVKTVALVGPLGRATTDLNGTWAGLGPVTPPVTIEAGLQAAGKAVVYVKGCDVNGTDTSGFAAAVAAARKADAVVLAVGETADMSGEAAARSDIGLPGVQS
RLVEAVAAAAKRTAVVLINGRPLTIADVLAAAPAVLEAWAPGSQGGNAIADVLTGAVNPGGKLPVSFPRSVGQVPIYYNHENTGRPADPDNKYTSKYLDLPSGPQLEFG
FGLSYTTFAVADLRLSSHTLHRSGGSVSVTVTVRNTGEVAGDEVVQLYIHDPVASVVQPVRRLRGFQRVTLAAGASRTVRFTLTPSDVGFYDGDARFVVERGTIHVYAG
TSSDDTPLTDELRVV
>tr|A0A267DV12|A0A267DV12_9PLAT
RQLVQSSGVLFIKTQFGHGSRDMAMRLLWLLLLPLLSIVAARHPELPFWDPTLSWDARVRDLLNRLTDSELVEQLSKGGAGTNGGPAPAVPRLGIRPYQWNTECLSGHG
EAGPATSFPQAIGLAATFSPELVEAMARATGLEVRASYNDYSRAGNYSDHHGLTCFAPVINLLRHPYWGRNQETYGEDPHLTGVLSAAFVRGLQGGDLAANRFALAGAG
CKHFAAYSGPEDFPVSRVSFNAIVPEQDLRQSFLPQFRRCLSAGSFSVMCSYNSVNGVPACANKRLLTDVLRRDWNFTGYVVSDEGALEFAVDFHKYFANRSEAAVGAL
QAGVNLELSPPGCPDQACIVFEHLHEAVAAGRVTRQQLLGRAYPLFYTRMRLGEFDPADSNPYSRLRLADIVQTDGQRRLSAGLAAASFVLLKNADDFLPLSVGRLNGK
RVGLIGPFADNMTLMYGSYAADPMPQYQVPLRSGLARVGAVVTVWRYCLDGVLCRRRNESALLEAARMSDLLLVAVGTGQLVEAENTDRRNLSLPSGQDRLVRSLAESP
GRWPPAVLLIFSGGPVDIQLAADPRSGIRAILWCGFPAQEVGVAVAAVLTGEMSPFASLPFTWYSPDQPLSSITNYTLANMTYRFLDEPVGSAPRPLYRFGQGLTFAAC
RVQLSELSAPVRVARGAALDLAFSASIPGSSEYSCLYSAKAYLRPAPGSRIGRNPRVWLVAMRLIAWQRGGRLQQVRMRVLPEDMQLWNGSAMSALAGDYELLIGDYFD
DSCAHCRLRQKFSIV
>tr|L8M232|L8M232_9CYAN
MKWKLTISKRQRAQLYWILTAIAVCLTIVFTHPNLRPEPRANVQLAQLDSPTKPSEIAQKVEDLLAQMTLPEKVGQMTQITLQAVSKTEGKLDQKYEVDLKKLREAIVK
YHVGSILNVHSSALTLGEWQQLITQIQNLATQETRTGIPILYGIDAIHGANYTLEATLFPQNLAIAATRNLSLARESAAITAYEMRASGIPWNFNPVLDVGRHPLWPRL
YETYGEDPYLVSNMGVAYIQGLSGEKQQIIAADKVAGCAKHYLGYSFPLSGKDRTPAWIPERMLRDYFLPPFAEAIAAGVPTVMVNSSEINGIPVHSDRNLLTDVLRGE
LGFQGFVVSDWEDVKNLYQRDRVASSPKEAVYLAVMAGLDMSMVPYDFSFYNYLIELVQEGRIAESRIDESVRRILHVKFMLNLFANPYPDLAMTSQVGSPEFAQVSLQ
AARESLTLLKNDQDLLPLNKNQKILVTGPNANLRSVLNGGWTYTWQGNEESLYPTSQNTILSALQEKLDAANITYIPGTKFDEAVNIPEAVTAARNVDVAVVVLGEKTY
TETPGNIDDLALPAAQLQLASAIANTGTPVVLVLVEGRPRLITPIVEDAEAILMAYLPGAFGGDAIADVLFGDYNPSGKLPMTYPRSPNDLVTYDHKPIETDTPNKLNP
LFSFGFGLSYTYFKYSNLQVNPQQIRPGESTNINVTVKNTGKRTGKEIVELYLSDLYRSVSPPVRQLRRFRGVTLEPGESQTIEFTLEQDDFAFHGRDNQRIVEPGEFK
VAISNLANVFTLLAD
>tr|T1EEJ9|T1EEJ9_HELRO
MLFLRTIALLIFFAFCCESKKTKKIPSICTTTSKSQEKNHDGTEKPGCSEFIYPFWDHTLSIEERLDDLMRRLTLQEMVDQMANGGGVPPDPAPAIERLGIPPYNFDTE
CLRGVADLNSTAFPMPIGLAASFSPDLLYEVASAISTEVRAIHHNATKHGDYGGMKGLNCFAPFMNLAHHPLWGRLQEVFGEDPFLIGQLTKSYVKGLQGNHPKYIKAI
AGCKTVLVHSGPENIPSSRFTFDAKVTWRDLQLTFLPHFKDCIEAGALNLMCSYNSINGVPACANKSLLTDLIRKEYKFEGFIVSDMDAIENIANGHKYAPNYLESAVL
AVNAGVDLELHGTANISYKLLVEAVEQEKVLYSTILNRTRKLFEARMKLGLFDPPSLNPYTDIDINEVIQSDYHRSLAMVAATKSFVLLKNINNRLPLVEGSIDKLAII
GPMSNNMDQIFGGYTPMVVRQFTTTPYDGLKMMVKQAVLVEGCESNACKTYDQPGVIKAASDADVVVVCLGTGQKIEGEDKDMPDMSLPGLQTQLLRDVVSHAKSPIIL
LLFTGCPIDLVEFNYTQNVGAIMQCFYPAQSTGEALKRVLFKNIGDNGDWSMVSPSGRLPYTWPMDLKQVPPMEDYSMVNRTYRYLTSRPLYPFGYGLSYASFFYFDLS
VYPTVINISSGFDDVINVGVSVKNAGPVDAEEVVQVYISWRFLPKEIVMPNVQLVAVSRQIVMLSADRAQFKLQFKSEQLKVFVDDVGFTYIPGKIDVYVGGQQPFDKR
IVNSNVLQSRIEIVI
>tr|A0A179SPW0|A0A179SPW0_9BACI
MEKYKDSHYPIHERLNSLINQMTLKEKVGQLNQKMYGWEAYNKTENGYELTSAFKEQVAKFDSMGALYGLFRADPWSNVNFANGIKVENSAAVTNMIQKYVKENTRLGI
PVLFSEECPHGHQALDSTIFPTHIGSGASWNPQLQQMVSKHVADELHARGGHLGLVSTLDIVRDPRWGRTEECFSEDPFLSSKMTEAVVRGMQGENENTPKVLPVLKHF
AAQGAGVGGHNSGPALIGERELREIFLPPMKAGVQSGVLACMAAYNEIDGVPCHANHHLLTKILREEWDYKGIVMADGTALDRLLLLTGNKELAAAYGLKAGVDLSLWD
DVYMEIETAVKSGKIEEQLVDKAVSRVLYLKFKLGLFDETTTRSEGKASNVIGSRRAIETNVEMARQSIVLVENKNKILPISKRLKKIAVIGPNADNIYNLLGDYTPPQ
RRENVVTILDGIKSMVGKDTEVLYAKGCGIRDKDKSEFTTVKKMAKEADIVVLALGGTSAREFGMEFENNGAAKGFNETEMDCGENIDVANLELGGVQLDLVKEIHSTG
TPIISVLIQGRPYSIPLLTQYCEAVLIGWYPGQQGGRAIAEVLFGDVNPNGKLPVSIPRSSMQLPVYYNYKDSGAKEDYFDMSGRALYPFGYGLSYTSFTYRFLSEENE
TITLKELHDSKQVTFYVEVKNIGEIDGYEVVQLYIKDMEATVTQRKKELKGFQKVWLKRNETKTITFSLGFDELAIWDIDMNFAIEPGSVKLMVGGSSHTVLEKKLIIE
DRSHHMGMKDQMQE
>tr|A0A2T1HU26|A0A2T1HU26_9HYPH
MVWPHAYGLPNRRDRMASFASRPRVLIAAAAATLALVAPGAASERVESLLAKMTLEEKVGQLNLVSGNHAVTGPFAPQDVRDAILKGQAGGLFNVYGAEYTHSLQEMAV
KETRLGIPLLLGFDVLHGYRTILPIPLGQAASWDMKAIEQGERVSATEAAAAGVNWIFAPMVDVARDPRWGRVAEGGGESAWLGAQIATARVRGLEGSNLANTDSVAAC
VKHFAGNGATEAGRDYSGLDLSERALREQQLPPFQAAVASRARCVMAAFNAVDGVPGVANDRLLRDILRREWGFDGIVVSDFGAISELPVHGVAEDGFDAARIAFQAGT

DMDMESRTYVASLPLLVREGAVPGGELDEAVRRILQLKEDLGLFDDPYARSDPRREATLIGSAEHRTAALSLAEKSLVLLKNDRQTLPFRRDARRVAVIGPLGDAGADT
LGPWAAHGDPDSTITLKEGIEAVLGGSAQVSTLAAGAVDGSKPADIAGAAKLAAQSDIVILALGEHATQSGEAASRASLDLPGDQMALARAVLAVGKPTAVVLFNGRPL
TVEDLDREAPAILEAWFPGSEGGLAVARTLFGLNEPTGRLPISFPRSVGQIPIYHDHLPTGRPAAPTYKPYTSAYIDLPSTPLYPFGYGLSYTRFEMGEPRLDRLRMGA
GETVRVSVDVSNVGDRRGTAQAQLYIRHRVARLSRPVRELRGMGRVELEPGQSAQIVFSLTEQDLAYWQPGGRFAPPEGGPIEVHVGPDSGQTRMAVLDYRPRGPSVAH
DPGVRVDTVRPTP
>tr|A0A1I2SBL1|A0A1I2SBL1_9BACL
MNRRKFSLLMLVTALSVALTGTHFAIATAEKPPSKPPYLDPSLPVEKRVKDLLKRMTLEEKVGQMTQINVTRLMGEDEWDRGPLNEEWMKKVFVDNHVGSILSGGGAAP
VPNNPEEWAKMTNALQRYALQHSRLKIPIIYGVDAVHGHNNVLGATIYPHNIGLANSWNPSLVREVYERTAKEVRATGIHWNFAPGADIARDLRWGRYYETFGEDPLLA
SEMVGAAVTGLEGDKLSSPDRVAATAKHYVGYSHPLNGQDRAPAELSLRTLREIFLPSFERAVQDGAETIMVNSGSVNGIPVHASPYLLRDVLRKELGFQGVVVSDWED
IIKLHTVHKIAPSYKEAIRISINAGVDMSMVPLDAEGFTKNLIELVREKKVSEKRIDEAVSRILALKFRLGLFENPYVDEKKAKEIIVDRADRDLARRAATQSITLLKN
EKNLLPLKKDLSTVLVTGPSADNPANQMGGWTIGWQGVENPDEMPPAVTLLEGIKGKGSKNTRVLYEPGVPPEDQDDDPEAVDKAIRKAVNAAKKADVVIAAVGETPYA
EGEGDTTTAALPPSQAKLIRALKDTGKDVVVVLVAGRPLVMTETIESVPAFLMAYLPGTEGGSALADILFGDVSPSGKLASTWPKRIGQLPTFYNRQPGASYDPLFPFG
YGLSYTSFRYENFQVPKSAKPKDTLRVSVTVTNTGDQAGDEIVQVYADRQYQSVLSPVERLVAFQRISLKPGESKRVTLDIPVSRLSVIPGDILGTEKRVVEPGTYQLR
VGNLKKTFTISK
>tr|A0A194XN72|A0A194XN72_9HELO
MSLSSFVNPLAVALIPPFATNTTVLFEVLTEATLIRPLPDCDQDPLKNLTICNTSLSPSERATSLVKLLTLDEKIDASIFYVPAVQRLGIPPYTLWNEALHGLGTSTGV
DFASNGSYSVASNFPGPLLMAAAFDDNLIEQVATAISIEARAFGNVGRAGLDYWAPNVNPFRDPRWGRGQETPGEDAFRVSGYTKAFVTGLEGPSSSKFKRGIATCKHL
AAYDLENYRNVTRFTFDAQVSIQDLADYYTPPFQACARDAKAGSIMCSYNSVNGIPTCLDPYLLQTVLREHWDWDAADHYVTTDCFALDVAFDSHNYTSTPEQTAADAL
KAGTDTDCGIFFSSYLPKALSDGLVAEKDLDRALTRVYAALIKLGYYDPPETQPYRNISYQDVNTPATQNLARQAATSGMTLLKNLNKTLPLFTPSAGNSTLSIALVGE
WSNATTEMLGGYAGAPPFIHSPLYGLQQVSGISVNVVVGVLDSAPVLAAAESSDIILYVGGIDNTIEAEGLDRVNITWNATQTSLITLLAKLGKPLIIAQSGGGQLDDT
DFLANPNISSILWIGYPGEDGGVALADVLFGNVAPAGRLPVTMYPASYESVPPTDMSLRPNEPLNPGRTYKWFDGAVLPFGYGLHYTNFSATASLTFPSSQDTGDIIRR
ANNTPYIDQFNLGDLVINVQNVGDVTSDYAALAFVSGEYGPLPRPKRELVAYSRLVGIEPRCTKTTGLPLKLGALTRWDASGRRVLYPGVYRLVVDTEPELAVVEFEIT
GSEVVIEQFPTA
>tr|A0A261Y1M3|A0A261Y1M3_9FUNG
MKASLGLITLAIAMLAGNVNATYSNKIPPMGYGQGVNFDAPSKNLDADVKTMVKKMSLKQKIGQMSQLNEDKILLPDGTVNVTAVEYYAKTYYIGSYLNNMAGHNGNLN
AAGYATNIETLSDITVKAGGLPILYGLDSVHGAHYVANATIFPHGIAQAASFDPSVAYNAAEITAKDSRGANIPWTFAPILDLGTNKQWPRIYENFGEDPYLQSVMAAA
SVRGFQGNYKTNRTKIAACAKHFLGYGATHSGEDRDSSWIPDRFLYDYYVPPFQAAYDAGAATTMESYIDINGEPVVGSTRYLKTLMRDDMGFNGMLVTDWAEIENLYT
YHQTASSPLDAVFQGIGQTSTDMSMIPEDTSFPELLLQLVQSGKIPESRIDESAGRVLQLKKDLGLLAPNGWKADKTLTASVGQTSDQEVAIDAARQLLTLTKNNNTVL
PIKNANKVLVVGPTANAMSYLAGGWTIYWQGATVDGWQGAVSDEYFYGNGVDILDGIKKAAPSGTTVDYVMGVDIWGNVNTTAADVASQAADADYVILCIGEHPYAEAP
GNIHDLTLPAGILTFADDLKAKLSNQKLVTILTQGRPRVIGDVPTISDAILHTFLPGPWGGIAVGEVLFGITNPSGRLPYSYPQYPGDQTLVYWKDIPDNQHADPLYNF
GDGFGYSPMEYSSITASSKTLTSSKSVSVSVTVTNMGEYDGKEPVLMYVQQPYRKISPPTKLLKAFNKISLKKGQSQKVTFEVTADMFKYTGVNNVPYGSLDSGIVNIF
IGTQNVTLTLQA
>tr|A0A2G1PHN3|A0A2G1PHN3_9ACTO
MTARLFAVLQLNEEPHVTAANASTSTKSPLAQLTAAQREHIEALLASLTWEEKLAQIQVTFKMTQEECLDAARSGIGALFWPGNAADTNAVQRVAVEETAHGIPLLIGL
DVIHGQRTTFPTPLAMGASFSAAVAQSCAVVSAAEARSGGVTWTFSPMVDVSRDPRWGRVAEGFGEDPLLTAELGAAMVSGYQHERLDEPGTMVATAKHFIGYGAAEGG
RDYNTVDMSDQRLHSVYLPPFATCVQAGVGSVMASFNTMNGRPVHANRRLLTGILKEELGFTGAIVGDASGVGNLIPHGVAADLPDAARMSLAAGLDVEMGGHLHDPAA
RPEHPALLDGDDPALVARVDDAVRRVLTLKTALGLFDNPYVDPAAEITAPTAEHLSAARIAAEKCPVLLTNDGTLPIAPTARRILLAGPAATHTDHLGAWVQHFAAPPA
HSLADALTAALADRAAATSTPAPELTVLGGQDPLGVTDAQIAEVAAAAADADLVILALDEPSQLTGEATSRADLHLPGNQAALVHAVAATGTPLAVVLVAGRPLVVEDW
IEEPGAVLMAWHLGTTAPEVIADVLTGAVNPSGRLPMGLPRHSGQLPATYDAHENTGRPATRGGEMIKPAFDMGLDGPANLQEFFTSKYRDLELGPRFRFGHGLSYTSF
TYRDAELNRATITRAELNAGQAVEVSVTVANTGERDGEDVVLLFTRDVLASLAPAVRRLSGFQRVAVPAGTTAQVIFRLERSHLALWDDDGQGWRVEPGDFEIRLGPDP
DAEPLLLTVTD
>tr|A0A4S8PYZ1|A0A4S8PYZ1_9ACTN
MDTDTPSGAAERAAGLVKRMTLEEKCAQLASLWRGVDSDAGDMAPHQSEQSIGHSEDEVIANGLGQLTRPFGSAPVDPEAGAARLAALQEKIKANSRFGIAALVHEECL
AGFMAHGATIFPTPLAWGATFDPELIGAVARQIGATMRQVGVHQGLAPVLDVVADSRWGRTEETMGEDPYLIGTIGSAYVTGLQESGIIATLKHFAGYSASHAARNFGP
VYLGERQLAETYLTPFEMAVRIGRAGSVMASYAANDGIPSHANQRLLTGILRDEWGFEGTVVADYFGVNFLDSLHHIAERRGDAAALALTAGVDVELPNVDCYGDPLID
AVGTGRLEVDVIDRAAERVLTQKIELGLMDELPEAPTEVDFDPPGARALAAEAARRSIVLLANDGILPLAAPARIALVGPVAADKAVMLGCYAFPNHHGRNDADDLDPD
DPYPHVDMGVEIATLAGALAADLPEAVIEHVAAGTVLETTDADIAAAVRAAAAAELAIVAVGDRSGLFGRGTSGEGCDAASHALPGQQGELLDAVLATGTPTVVVVISG
RPYALGAAADSGAEHPAAAIVQAFLPGQEGAGAISAVLTGSAEPEGRLPVGVPARPDSPPATYLGPELARRSNVSTVDPTPLFAFGHGLSYTEFEWDAPRLLSGTEVAP
DGTVEVAVRVRNAGGRSGAEVVQLYLHDPVSTMVRPVQRLVGFAKLRLGPGEAAETRFAMPVDQAALIGADGEWTVEAGELELRLARSASDVAGTVTVSVTESRHLDPG
TRRLETVTEVG
>tr|A0A433D7H7|A0A433D7H7_9FUNG
MMTLLATSLLGEGLISYNSLAPTPWAFAKMSIRILNLSIVLIYLAVQTIHAIDEKPWLDASLPYTKRANLLLYAMNLDEKLTLVHGIGGQYVGNVPNNTRLAIPALHLQ
DGPQGVAGRVTNVTAFPSVLTVTAAWDPSLMELFASAIAVEQRIKGANVMLSPMINIARIPVGGRNFESMGEDPYLAARLVASYVRGVQKNGVMACAKHWANNNQEHSR
MTVGTYIDERTEWEIYYPAFQAAVDADVASVMCAYNLVNGTYACENYKLLTVNLKERMGFKYFVMSDWFATHSTVQSVNAGLDQEMPDRNFFNPGALKQAISQGLVTTS
RIDDMVRRILLPMFKFGLFDRTNEGTINAPAESRSHRQLAHDLAAVGTVLLKNRRGILPLREEKIASIAVIGDAAHLNPVVVGGGSGSVHPPHISTPLDGIRSRAGKSI
NVTYTPGGNGGNMALVEDAARKANVVIVVVGATSAEGVDRPNLLLPLAQDAMVERVAAANPNTVVVVYAPGAVLLPWKRKVAAIVCGFLPGQEAGDAIAEILFGDVNPS
GKLPLTFPLSETQVATSTPIQYPGIDNREEYIEKLLVGYRWYDALKKQPLYPFGHGLSYTTFRYHRPTITLTPDDPHCRATVRIDVRNTGDRAGTEVVQLYLGFPPWTH
EPPRQLKGFQRVFFEPGEIRTIEFMLDDRAFRVWDAGKGRFVVAPGRFRVFVGSSSRDVRAEAHVVIGREGVVNMMQIDLIDFWIRAFPLIGISETILRLKNSANSHNP
KASGCDSRMHA
>tr|A0A1L9T5R3|A0A1L9T5R3_9EURO
MVGLLNPRNLIVLTPLILPLPLQTLAQSNYPDCTTGPLSQLAICDTSLDAHTRAKSLVNALTLVEKINNTGHEAAGSPRLGLPAYNWWNEALHGVAEKHGVSFEADGEF
SYATSFPAPIVLGAAFNDDLVKTVAGIISTEARAFSNSDHAGLDYWTPNINPFKDPRWGRGQETPGEDPLHCSRYVKGFVGGLQGDDPENPKVVAACKHLAAYDLEDWG
GVSRFEFDAQVSAVDLVEYYMPPFRTCAVDAKVGAFMCSYNALNGVPACADRYLLQTVLREHYGWEGPGHWVTGDCGAVERIQTHHHYVDSGPEAAAAALNAGVDLDCG
TWLPTYLGEAQAQGLVSNETLDTALTRLYTSLVQLGYFDPADDQPLRSIGWEDVATSEAEKAAWTVATQGTVLLKNDDRTLPLKRNGTLALIGPYVNLTTELQSNYAGP
AKDIPTMIEAAKHLGYTVLTAQGTEMDSTSEDGFENALNIAAEADAVIFFGGIDNSIEEESLDRTSIDWPGNQQDLILQLAEVGKPLTVVQFGGGQVDDSQLLSADNIG
AIVWVGYPSQAGGTAVFDILTGKVAPAGRLPVTQYPKEYVDEVPMTDMNLRPGTDNPGRTYRWYDEAVLPFGYGLHYTTFNVSWAKKDFGSYDSASLTKGEQPHLNVVD
TFSLAVTNTGGTVSDYVALIFASSADAGPQPAPIKTLVGYTRVSGIKPGETRKVDVEVTVAPLMRGRSDGGVVLYPGKYTLLIDVNEDYPTSGFTIKGQPQVIERLPLS
TNGTTKVRDL
>tr|A0A133UC01|A0A133UC01_9EURY
MRGNSSPLYLNPEASVEARVEDLISRLSLEEKVAQLGSVESDKLLKDGEFSPEKAEKALSNGIGQITRIAGATGLNPKESAMVANQVQEFLVKNAPHSIPAMTHEECLS
GYMGKGGTTYPQSIGMASTWDPNLMEKITKEIKKQLKAIGAHLALSPVSDMARDLRWGRVEETFGEDPYLVARMVTSYVKGLQGPEPKDGIYATLKHFGGHSVPEGGRN
HSPVNISPRELRENFLFPFEATIKKAKAGSVMNAYHDIDGIPCAASEELLTDILRGEWEFDGIVVSDYFSIGMLYTDHKIAEDLQEAGIKAMEAGIDVELPKTNCYGEK
LIEAVENGLISEAVIDEAVRRHLRAKFRKGIFEKRFVETDGVDSSFETEKQRKLAREAVRKSAVLLKNEGDLLPLNKNIDSVGVIGPNADSTRNLLGDYAYNAHLETEE
SAISIISILEGIKKKVSSDTEVMYAQGCSINGKSRDGLEEAVEIAKKSDLAIVVLGGKSGVGLVEPSSREKENAQTTGEGNDRTNLQLPGIQKELIKRMYETDVPVVVI
LVNGRPLATKWIAEHVPAILKTWLPGEEGGNGIADILFGDYCPSGKLPVSIPESVGQLPVHYRRKPISKERNYVFSRNEPLFPFGHGLSYTKFEYDKLKIKPKEINPAS
EFSVKVRIKNSGEREGDEIVQLYIRDEIASLTRPVKELKGFKRISLEPKESKTITFKLSADQLTFYDHNMNLVVEPGSFEVMLGSSSEDIRLSEKFEVTGEKRKVSGSR
KYFTEVKVED
>tr|F2N9A5|F2N9A5_CORGP
MSDFPYTDSSLTIRARVEDLLGRMSLEEKIGQINQHLYGWQCVSQSQTGSWYLTDMFKRHVEWSKGLGALYGAFRADPWSKIDFTNGVRGRDGWRITNLIQDYVKSHSR
WGIPALIVEECPHGHQGLDGISYPTNIGRGCMFNTDLIREGAHLMGRELSCMGVDLALVSTLDLARDPRWGRTEECFGEEPYLSAKYSEAIVEGFQGRLIRHGSSFLDR
PVHTREPQERPVGAVLKHCIAQGDAQGGHNSGTVVIGDREFNDVYMPLMRAGREAVGIMAAYNDIDGVPCHSNTALLTDVLRTNVGFQGIVMADGIALDRLFGPYPTIS
AAAAAALTAGVDMSLWDDAFLHVDSAIKQNLTSELDLNRAVARVLSIKFLLGLFDRPPLTDPGEEYERVLKRSRELNFETARRTMVLVKNNGTLPFDESGKSIAVLGPN
ADSVYSMLGDYTAPQDDDSLAATILHELRKISPSATFTYAKGCEIRQIDGQDEALDDALACAQASDAVVLCLGGSSERNFNMEFLRNGAVSSRGANMDSGENVDVASLS
LGGCQMQMAREVAKLGKPMVSVLVQGRPYDIQELEQLSDAVLIAWYPGQSGGAAVARVLTGADNPSGKLSISYPRNASQLPVYHHQRRSFGNADYLDEPGSPLHPFGFG

LSYTNISYHDLIAHISEDNVEIRVQVRNLGDRGAIETILAYVELIGGGVLQREMLQGFESVKLNPGEVQSVIIRFDRSAFSYMDARRNLATASRARIRVNELETLIDLS
EGTASRMTDA
>tr|W9W491|W9W491_9EURO
MAGFLFCLLALDISTALSAVLLPRQGDPTWQDTTLPAAQRADALLPQLSWEEKIAQMGGIRRLLGANATFNRTAWEALYPLQHGILSYGSQLNQAQDVLPYANMVREEQ
LNSSKVPWITVTDSVNSIYVPGGTLFPATLSLSTSWNLPLYEEIVASIRDENMALGTHWVLSPELDIAKEPRNGRVGEMYGEDVYLVGEFAAQYVKTMQERDEGGHMKV
ATTVKHFLYGQGSGGVNTASMDGGVNHLYNDLAIPYIRVLKEKPASIMISYSSVDRIPMSMNTALIQDMLRSEMGFTGLIMSDAMGILHLYTESNVASSYKDAAIKALR
AGLQLELAPGQPACFPYLVNSSSDQEIVDLVNEAARQHLIIKFDTAMFDLPLPTMENLNRTLRAPQHLDVNRRASREAIVLLSNDGFLPQANFSQVALIGPFGDIIDPG
SYAPTTSANPEYGRTLRGSLEARMGAGNVRYVKGVDIRTTSPNDTAGIQEAVAVAKAAGVAIVSLGSLSVYSQDAAVNQRTDGEFYSHASLAFPGNQQQLLDAVLDTGV
PTILVLNGGQAFVLNNSTMRCNAILHQFLGGEFSADALVEIITGQVNPSGKLTISMPQADGAFPIYYDFLPSDNVGGGGSDPTDSTVCAGDWNLPCLNRDGAPMAFGYG
LSYTTFDISGPQVTNSNSSGGSISIFCTITNTGNVAGKEVVQVYFRQQYSDIELPNKRLVRFQKVELQPGEARQVKFVIDKADLGYYNNAKYQVDSGHYTFWVGSSSRM
ADLKNATISL
>tr|A0A1J4JE96|A0A1J4JE96_9EUKA
MSFEVKSIVSQLTLEEKVNLCGGIDMWHTASVERLGVPNIMMSDGPHGLRKQTVSEGIININDSIDAVCFPAACATSCSFDKDILRKMGQTLADEMIATDISILLGPDV
NIKRSPLCGRNFEYFSEDPFLSTTLSGSLIEGIQSKGVGACIKHFAVNNQEHRRLSVSANVDERALREIYLASFEGAIRNAKPWAIMCSYNRVNGVYASDNEQLLNQIL
RKEWGYEGFVMSDWGAVNDRVAGLKAGLDLQMPGPCKWSIPVVIKAIKDGLLDEKYVDICAERVLNMVSKAHHTFKNLTGEKGCFDLEKHHIIARKVEECIVLLKNEN
NVLPLKKTQKIAFIGGFAKTPRYQGSGSSHINASKVTNAYDSSKVIVGESTGSIEYAEGFRTDIQKEENENEQQIDREKIDQAINLAKSSEVIVIFAGLPDAYESEGYD
RAHINMPSNQNILIEEIAKVNNNVIVILHNGSPIAMPWASSVSGILECFLGGQAVGEATANILFGITNPSGHLSETYPLRLEDNPSYLEFPGHDNEVNYLESIFVGYRY
YEKKHMNVLFPFGHGLSYTTFKYNNLSINKSEFKDNDENIVVSVDVENTGSVAGKTVAQLYVSDHTNIINRPVKELKGFEKVYLEPGEKKTIQFKLCKRSFAYWNTKIH
DWHAESGKYEIHIGQSSQQIEASLAVNVVSSSKIVRHIDMNSTLSEILQIQAIKDFLKSEIAKLPELKGALESSDSFIRILASESPLRSLAFLLPNRDQVENIIKHSNK
LIDDAVNSE
>tr|A0A4V5PN39|A0A4V5PN39_9DELT
MTKRGGGAGPDAGVRAKGGDMQRRTFGGIGLVLLASLLGCGDSAQNGVFPSGSVEERRTRALLDRMTLEEKVGQMNQYIAPIYAQSLDSPDPAGKIDPLLAQGLVGSFL
FVTDASEANALQEKASRARLGIPLIFGIDAVHGLAPVRGATIFPTPIGMAATFDVDLMERFGEVTAHEMRVTGMHWAFSPVLDVARDPRWGRTAETFGEDPFVVAAMGS
ALVRGLQGPDRAHLRVLACLKHFLGPGLPLGGRNMGPIEVSERALRSTFLPPFQAGVEAGALSVMAAYNDVNGVPSHVSEELLTNVLRHEWGFRGFVVSDWEGIEMLHT
THHVAASQKDAIRQAVLAGVDMHMHGEGFAEPLVELVREGAVGAWRIDEAAGRILRVKRALGLFERRYVDVARAGSVLASPGHRKVALEAARKSIVLLRNEQDLLPLRK
NLKKVLVTGPNADNTALLGDWTAPQPAENVITVLEGIRAAVSPQTVVHFVDAGRVFEETNEAIQRAADEARGAEVAIVVLGENETRYDDRGVLDRRRRERTGGEGADRA
DLTLVGRQMDLVRAIVETGTPTVVVLVNGRPLAIPWIAANVPAVLEAFEPGLAGGQAVAEVLFGDVSPSGRLPISIPRSVGQLPVHYDHPRSAESPYVDESFEPLYGFG
HGLSFTRFVYTDLQVPERTRRGEDVVISVVVRNAGARTADEVVLLFVRDVVASVTRPVRALRGFQRVHLAPGERQVVQFRLPFVSLAVHDRRMERVVEPGTFEVSIGGL
RGSWVVERK
>tr|A0A553F9M5|A0A553F9M5_9GAMM
MKYKNALPLTAALASLSMLCMPVVAADLLPYQDKNLSAEERVDDLISRMTLREKAGQMSQFVGLEHIKESESKLTAEQLNSSDAHGFYPGLRPADLLAMVEKSEIGSFL
HVVNIEEANELQKHAMKSRLGIPLIIGIDAIHGNALVRGATVYPSPISAASSFNLDLVKKSSVETAKEMRANGSHWTFTPNVDVVRDPRWGRVGETFGEDPFLVAKMGV
ATVEGLQQTDFTGYDKVIANAKHFVGGGDSINGLNIAPLDVSERTLRQDYFPPFKEVLDAGVFTVMAAHNEVNGVPSHGSKFLLTDVLRGEWDFPGFVVSDWLDVDRLK
TLHKVVPTHKDAVHLTVDSGMDMNMHGPQFAGPIIELVEEGRLTEARIDASVKPILLAKFRLGLFDNPYVDETLRDKVNFNSEHQQTALAMARESIVLLKNDNNVLPLK
NVKNIFVTGPNADAHTTLGDWSLEQPEDNVTTILEGLQQVSGNKIKLDYLDVGKQVKVLSDEQITEAAKRAKSADVSIVVVGENPLRFDNEGKTSGENVARAELDLYGR
QLELIKAVHAAGKPVIVVLINGRPISEPWLSENVDAIVEAWEPGSFGGQAVAEILYGKVNPSAKMPISVPYSVGHIQSIYNHKPSTYFKRYVDSPTKNLYEFGFGLSYS
TFTYSELSLDKNSINKDGSATVTVKVTNTSDVAGDEVVQLYINDNYSQVTRPVKELKGFKRVSLGANESKKVSFTVTPDLLAYYNLAMQWGVESGDFTLMVGSSSRDSD
LKTIQLNVK
>tr|D8RVL3|D8RVL3_SELML
MLALLFFFSIAWAPAESRGGAGAACTDSRSSSFPFCDVSLPVPDRVADLVGRMNLSEKIAQIVSNASGIPRLGIPGYQWWEEALHGVAESPGVKFAAPVPSATSFPQVI
LTVASFNSSLWNKIAQAISIEAIAMYNAGRSGLTFWSPNINIFRDPRWGRGQETPGEDPLLSSKYAAYFVRGLQEGDYDEGTAISTMQRRPTRLKVSSCCKHFTAYDME
KSEGTDCFHFNAQVTVQDLQDTFDPPFRSCIVDGQASGLMCSYNRVNGVPSCADYTFLTETVRNSWGFEGYIVSDCDAVALLYEYINYTTTAEDAVADVLSAGMDLNCG
TFLLRHTAAAIEQGKVTEAAVDRALSNVMTVRMRLGLFDGNSGETYNSIGPDAVCTREHRQLSLEAAEQGIVLLKNSGNVLPFPRNDLMTIAVIGPSGNATETMLGNYA
GVPCQYITPFQGLQEYTKGVVFEPGCKDIMCNDTTLFLAAVRAAENSDAVVIVVGLDKDQEREGLDRTSLLLPGYQQDLVLEVSKVAKGPVILVVMSGGPIDVTFAKGN
CKISSVLWVGYPGEAGGKAIARVIFGDHNPAGRLPMTWYPQAFAEHVSILNMHLRPNTSTGFPGRTYRFYTGENVYEFGHGLSYTNFTYTNFSAPSNITARNTVAIRTP
LREDGARHFPIDYTGCEALAFKVVAYISNTGTRDSDHISLLYAIPPAASSSLSPPRKQLISFKRQHLIAGRCAKVEFDVDTCKDLGLTNEAGTKVLVHGDYKLSLGDIE
HVISLTLLS
>tr|A0A0W0FN73|A0A0W0FN73_9AGAR
MLHRHLLFQTLTTVIFINAQSPSSVTNDVSSSAGATTNSSPPSTANSVMLTSSAMSTSFSTPSTPASFTLVSNSMTLTPSAGIPSQASAFPSSQQASISVPAPPQSPIQ
GLFPSTDPQSPPDVKDSSIIVPDFLPAWEAAYEKARAKVAGSILEELVNIVTGVGALGLQGRCVGNIGSLSSNGGWPGLCLEDSPLGVRFADFVTSFPTGLNTAATFNR
TLIRQRGLFIGLEHRGKGVNVALGPMMNILRVAEAGRNFEGFGADPFLAGEAAYETILGMQQGGVQACAKHFINKYTHFSSRCPTAKRPQSEQETARTTSTSNIDDRTQ
HEIYAHPFLRSVMAGVASVMCSYNQVNGTYACENNSTLNGLLKNEFGFQGFVTSDWGATHSTVASTLSGLDMDMPGSGGPNGSFFGGTLVEAVRNGSVPSSRIQDMATR
VFAAWYFLHQNSPSYPFVNFDVNFLNETTNQHIDVQDNHRHIVREIGAASVVMLKNVNNALPLKKPRSLFLAGSDAGPGIIGPTESLDQKNNDGVLGVGWGSGSTYFTY
LISPYEAIERRAIEDHTSVSWVFDDFNLARAEIMAIGHSAALVFVNADSGEDSDRTNLTLWHQGESLIQAVAAQSNNTIVIVHSVGPLIVESWIDHPNITAVLWAGAPG
QEAGNSIADVLYGDFNPSGRLPFTIAKRAEDYPAHVVNGSGIVSVPYTEGLEIDYRAFDARNITPRFEFGFGLSYSTFEYSDLDVTPVSESSTDEQAIKNWESGKTSRL
EFGSSVAL
>tr|A0A242KB74|A0A242KB74_9ENTE
MSYKNPDLTIHERVEDLLSQMTLEEKVGQVNQHLYGWKAYEKAHTGFQLTDYFKEHVHWGQGMGALYGLFRADPWSKVNHLNGIPAEESWKLANEVQDYVINHSRLGIP
VLLVEECPHGHQGLGSISYPTNIGRGNSFNKELIEETARHMAEELAMKGVHLALVSSLDLSRDPRWGRTEECYGEDPYLAAAFNHAIVNGFQGNMINAQHSFVHQTVAD
IGRKPEQLGVVLKHCIAQGDGLGGHNSGAVNIGEREFMEIYYPLLKSAKNAVGIMAAYNDIDGVPCHTNQRLFEELLRKDIGYQGIVMADGTALDRLKPIYGSDEKAAG
KALQAGIDLSLWDNTYLTIGAGIKQAVVQQAALDRAVYRMLSIKFMLGLFDRPYTEKPEIAYKERFYSYRKRNHQMAAESMTLLKNEGVLPLQDIGEKIAVIGPNAHAL
YNQLGDYTAPQDEDVLDRTIFTSIKQAFSHSDVAYAQGCDIRAEENQVTHMEKAVELAEKSDKIILVLGGSSARNFDMEFFANGAVSSKGVNMDSGENVDVASLALGGK
QLALFDCLSKLGKPIVTLLIQGRPHEIEAICQKSDAVVAAWYPGQEGGPAVAQLLAGVVNPSGKLSISYPRSSGQLPVYHYQRAIAMNENYYDLPGSALYSFGYGKSFT
LFSYEKLTILNPHLTKQDLIDGKKLQLEVTVKNTGQVVGKEAVLLFVKLEGGEVIQRKQLLRGFEKVQIAPNQTEKVSFALGFDELSYFSNQGDFELSEQVIIKIEELE
QSISLGNS
>tr|E4Q6A9|E4Q6A9_CALOW
MSIEKKVNELLQKMTVEEKVYQLTSVLVKDILENNQFSEEKAKKAIPHGIGQITRVAGASNFTPQQALEAANKIQKFLIENTRLKIPAIIHEESCSGFMASKATVFPQS
IGVACTFDNELVKEMAKVIRLQMKAVGAHQALAPLIDVARDARWGRVEETFGEDPYLVANMAVSYVEGIQGKDFEEKVIATGKHFVGYAMSEGGMNWAPVHIPERELRE
VYLYPFEVAVKVAGLKSIMPAYHEIDGIPCHANRKLLTEIARNEWGFDGIFVSDYSGVKNILDYHKSVKTYEEAAYISLWAGLDIELPRIECFTEKFIEALKEGKFDMA
VVDAAVKRVLEMKFRLGLFDNPFVKTENIIELFDNKEQRSLARKVAQESMVLLKNDGILPLKEKDLKKVAVIGPNANSVRNLLGDYSYPAHISTTEMFFMKEEVDLGDE
DAFVKKVVNIKSVYEVIKERIGKHTEVVYAKGCDVNSQDKSSFEEAKKAAQGADVVIVVVGDKAGLKLDCTSGESRDRASLKLPGVQEELIEEIAKVNQNIVVILVNGR
PVALENIWQKSKAILEAWFPGEEGAEAIADVIFGKYNPGGKLAISFPRDVGQVPVYYGHKPSGGKSCWHGDYVEMSSKPFLPFGYGLSYTTFEYKNLTIEKEKITMDES
IKISVEIENTGNYEGDEVVQLYTRKEEFLVTRPVKELKAYKRVHLKPGEKKKVVFEIFPDQFAYYDYDMNRVISPGTVEVMVGASSEDIKFTGTFEIVGEKKDAKEIKN
YLSRAWCE
>tr|W3WW73|W3WW73_PESFW
MRGLVMKALYFALATSLAIADSTLDGSKCNLTVPSDAVYLTASATTDERVNDLLDYMCWSEKIAQLTGIGGLLGSNVTYNNTTLYDQLSSIHQGSISPGSYLNYASDAVP
VIKDVIEEFTNNSRLHIPYVNIADSVNGVTLLGTTFFPATISMSMSWNLDLFKQAVTAIRDEMVACGINWVLSPDLDPARDPRHGRVGETYGEDAFLNGEYGITYVETM
QESDENSFMKIATTVKHFLYPTSVGGINSGSIDTGINNIFNVLAYPYIRVFRKTTPASLMPSYASIDRVPSHANKGLLQDILRDTLGFKGVILSDADGVSGIYNQHKIG
QDVYDAGARALEAGVQSVLAIQFPTGFEEVINTPSLAPQVNEAVTNLLRLKFTLGLFDKSFPDQAQLNSTLRSDEHLAIAQNMSRESIVLLKNDGLLPLPSTTSNVAVI
GPLGDKIIPGTYAAWTNADNHNKTFVDALKGWLGEDQVNFVPGVQVLSSENADIASAVTAATAAEIVILTLGAATVGWDDPLLSQKTDGEGATHGSLTFPGLQEDLLAQ
VLAVGKPTILVISGGQAFELSGTAQGASAIVHSFLAGEYTGQAVVDILRGLVNPSGKLTISFPNASPVNPIYYDLLPSDWSSTAMNWPQLTLPALYPFGFGLSYTNFSI
SSPSADQDEYSQDGTITVSFSIENTGAFAGKQVVQVYFGQSSGASIELPSKRLVGFTKVDLQPGEQRTASIAIPVIELGYFVNGQFTLDKTIYTLYVATSSASSDFVSA
LNVTLV
>tr|A0A4P8XIZ1|A0A4P8XIZ1_9BACL

MESYRNASTPIDARVIDLLQRMTLVEKVVQTLSIGKVGQSFDVELNEDGSLEESCIEEIYKHGAGAIQLPFKTDSIETRIKKLNALQDYYVNKTRLGIPVMAQEECLHG
HLAKDATSFPIPIAMASTWDTELIERVYSAIGKEARVRGGHEAHTPVLDLARDPRWGRTEETYGEDTYLVTRMGVAAVRGLQGTDEIVNHEHVVSAPKHLAGYAQSDGG
RNFAPSNIPTRVLRDQILPPFKAVVQEAGALGMMPSHNEIDGIPCHGNRQLLTEILRDEWGFNGIVVSDYFDASRLDILFHVVNNSKEAAVKALKAGLDMDLPGGGCYT
QLLDAIAEDPELEDVLNVTVARILRVKFLLGLFENPYVDAEHAKQVINCEQHKKLAKEAADKSITLLKNEGGLLPLNRNEIRKLAVIGPNAHPICTGSYSTKPNKGISI
LDGIIAKTNGELEVAYAVGCEIIKGKDDSGETELDRRMNNPQLSTGDKNEAWIVEAVSVAKESDVAILCVGGNTLTSREAIFFNDDRGDRYDLDLPGVQNELVKRIVET
GTPTVVLLINGGPLTINYIAQHVPAILEGWYLGEETGHAVADVLFGDVNPSGKLPITFPRSVGQLPVYYSQKPTGLFKKYLFAEHDEPLFSFGTGLSYTTFQYRHLQLS
SITMAMNGSVTVSVEVSNTGEYAGDEIVQLYIADLVSSVTRPVQELKGFMRITLQPGEAQTVAFRIDPSMLSFTGEDYEITVEPGAFKIMVGSSSKHYESIVLNVIEEE
KTNDA
>tr|A0A3N2DBE8|A0A3N2DBE8_9MICO
MTMTDPARTTQDSPTDRVRELLARMTTREKVGQLNQRLFGWQHVRRTRTGYVLSEELLAHAEHWGGIGAVYGLHRADAWSGQSWSNGIAPEAAAEVTRQLQDAVAAVSR
LAVPALIVEEAPHGHQGLGGTLLPTTIGQAATFDPEWVAERAAAVAAELASVGAHVALVSGLDLARDPRWGRSEECFGEDPLLAAELTGATVAAMQGEGERIDAAHVAV
VVKHLAAQGAAAGGRNAASAVIGRRELHEIHLPPARSAVLADAAGFMSAYNDIDGVPCSANRDLLTGLLRQQWGSRGIVMSDMGALDRLAGPAGGLVEAGALALNAGVD
MSMGDVAFTLLEEALARGLVNEAAIDLACERVLRLKDRLGLLDGTRSVEAPARPRFPVPPVDASALVLVKNDGALPLPPGIERVAVIGPNSQDVGCLLGDYVPPLPAGD
GVSIAEGLVQTLAAEVRCERGSMVRGELPGGIGRAVDLARWAEVVVLALGGSSRRRYDGEFEDNGAAAGAEDLDVSGGEGADLADVRLPGAQRELVEAVAEAVAGSGTR
IVTVMVTGRAQGASPALELSDALLYAWYPGPDGGRVIAETLIGNDEPAGRMPVSVPRSSAELPVTYDRRLEWSPRYLTGPVSPETPFGAGLGYTSWTLGPVEGVPAAAG
IEDVDAGRVGVRVRVTNTGRRAGSQVVQLYGRLMVPGLAPRTASLLGHSRVRLEPGESREVEVRIAPSPPALAAIDGPGRLALVAGLDSSVSFDEAVEIDLVVTAAGAD
SAAL
>tr|A0A4U0TLR0|A0A4U0TLR0_9PEZI
MVKRSAAIALATSSILSPVAGIVTLPESDPWSAAYCQAAALVSNMTLSEMNNVTLGFAVSAGTVATGSACSGISGGVPRLGYPGMCFNDAGNGVRSQDGVSAFSSGVSV
GASWNAMLAYERGLYMGAEFQRKGINVALGPVVGPIGRVAEGGRNWEGFGADPYLDGMLAIPTVQGMQESVIACTKHFVAYEQETNRTQSTLNPLGLATSANVNDKTMH
ELYLWPFEDAVNAGTGSIMCSYNRINGTYSCSNGETLNALLKGELGFNGFVVSDWAGQHDGLPSAQGGLDVVMPSSVYWDNDQLAKAVQNGTLSRERLENMAQRTLAAW
FKLEQNSPALPAVGSGLVANRSLPHSLKDVRDPASAPSILQQAIEGHVLVKNLDGALPLKSPRVMSLFGYDATATPINSGYTIPFSDNSSSDLWATNWQPLGLETIDAY
LKGTAAPNTVPGLLITGGGSGASNAPYISTPFDALSQRAISDGSLLFWNFEQTDPEVEGASDACLVFLNSYSSEGWDRAGLTDEQSDNLVLSVASKCGNTMVFIHNVHI
RLVDAWIDHPNVTAVMYAHLPGQDAGRALASLIYGEVSPSGRMPYTVAKSASDYGDLLHPCRGSINDTNPQCDFTEGVNIDYRSFLARNVTPRYEFGYGLTYSSFEYSD
FSIDSQPNSTAQAALFDTVTTVSIRVANTGNFSTAEVSQLYLQIPGVGTRTLRGFAKTDLAPGASKPVTFSLRKKDLSEWDVVSQQWVQPKGTYEVMIGKSVLDVQLRG
SFKL
>tr|F0YZW8|F0YZW8_9CLOT
MNTTKYMNTSLSVGERAQDLLSRMTLKEKIGQMNQKMHGWNAYKVEGETVELTEAFAEEVAFGDGVGAIYGVFRADGWNSHLTSGIKVRDSVRVANTIQRYIRENTRLG
IPVFLSEECPHGHEALQATTFPTNIGIGASWNTELYEKVCNIIARELRARGGHLGLISALDIAMDPRWGRTEECYSEDPFLAAAFCEKAVLGMQGDKDALTQPDRVISV
LKHFCAQGATIGGHNGKATNIGPRELFEIHLPGMKKGAKAGALGCMAAYNDIDGVPCHINRSLLTGILREQFGFTGFVMSDGRGVDRAKNITGSYESACAAAVHAGVDL
NLWNECFLKLENAVRKNPLLEKDIDAAVLRILEAKFRMGLFENPYVEETPALLNIGSKEAKETALEIARESVVLLENKGDVLPLGKEIKRIAVIGPNGDSVYNQLGDYT
QWKEEGEVVTVLQGLRKQAPSGVTIEFATGCGIRDVSKDGFPSAISLAEDADAVVMVLGGSSTREPGMTFEDNGAVFMNAFTKELNCGEAVDLAELRLGGVQEDLAKEI
KKFGKPLIVVLIEGRPHAVSWMKENADALLCAWYPGERGGDAVGEILFGRTNPSGRLPVSIPKSSAQLPVYYNRKDETDYIDESAKPLYPFGYGQSYTEFVCGDFKLNT
QRLSLKELEQGKRFEMTCSITNKGGRQGKEVLQLYIYDMESCITRRVKELKDFAKVSLEKGEERQVRLSVGDEELRIYDYDMNFVLEKGHVRLMLGKSSADILYDTIVE
IVS
>tr|A0A1S1Y3V4|A0A1S1Y3V4_9GAMM
MFAGRPVYPVTFTKTLNIKAVLVSLSLFVLSESVLSAEPNPSPVEERVQALLAQMTLEEKVGQMTQIDFSVVSVANGQDAENPVDQAKLDDAIFNHHVGSILNTPTTPN
NRAQPIEKWRNITQSIRDTAARSRLKIPVIYGIDAIHGATYTQNSVLFPQAISMAATFNPELSFKEGEIVAREVKASGLDWNFSPVMDIGRQPLWPRLWETYGEDVHLA
QTLGGAFIKGHQGEDMAAADKAPTCLKHYVGYSYPLNGKDRTPAWIGERALREWFLPPFEAGILAGAPSVMINSAEVDGIPGHANYHYLTEILRGEMGFTGFTVSDWED
IIRLHTRDKLAASPREAVKMAVMAGVDMSMVPFNFSFYDLLLDLAKSGEVPLSRIDEAVARILRVKFQTGLFERTEPALPVAGYVATAEAIEINQQAAREAIVLAKNDN
RMLPLSKTASILVTGPTANLLSVMNGGWTITWQGASEELYPKQYPTLLQAIQRKTSGQVTFVGGQRFSDEINIEQAVNEARKHDVVVLALGEKTYTETEGNIDSLALDP
VQLQLARAIFEIGKPVVLVTFGGRPRIITEIAEKARAVVLGFLPGMEGGAALADILFGDVNPSGKLPISYPRAVNDITPYDHKPIEAYETNQYRPLYPFGHGLSYTRFE
TSGLLVKPAQIKAGDSVEVSVKVKNTGTRTGKETVLVYLNDIAASVSRPVKQLKAFEKVELQPGEQKTLHFTLTAQAMSFIGLNMRRVVEPGDFKVMVGDESVPFSVLP
N
>tr|A0A6H0Y5Y3|A0A6H0Y5Y3_9PEZI
MVHSTALALALGLAGVNAISWSEARTQAEALVAQMSNTEKQNITYGFPSNCVGQTGSVDRLKIPGFCLADAENGVRQTDFVNAYPAGISVAASWNKDLAYWRGKYMGAE
FKRKGNHAALGPVVGPIGRMAKDGRAFEGFGSDPFLAGKLAAHTIEGLQENVMAVVKHLIYNEQETMRQEHGATPAISANVDDTTAHELYLWPFVDAVRADVAAVMSSY
NRINGTYASQNNKTLNGLLKGELAFPGFVVTDWNSQHSGSESAVNGLDMAMPNSKGYWQDALPAFVAAGNLTQERFDDMVTRTLTAWFKVIGGPDADFPEAGVGMFADL
LQPHALVDARDPESKDTILQGAIEGHVLVKNTNNALPLNQPKLLSLFGFDATIPKKYYPSTPLGLDYIFTQGFESAGLNLTQLLQLAGSGNATAAPQVADGGILWNGGG
SGANHPSALSETYGAIQQRARRDDTYLYWNFLDPTVEVHPLSSACLVFINELTSEMFDRVTLAPSIADAYVLNVASQCNNTIVVTHNPAIRTVDAWIENPNITAVVYAH
FPGNDAGESLAQVLYGESSPSGRLPYTVAKNDTDYGSLENPCLEGDLTTDAYCNFTEGIYTDYRYFLKNSITPRFAFGYGLTYSTFSYSNFTASWIDGADLSTSPPDPE
DMTPGGIESLFDEVAMAHITVTNTGNVYTAEVPQLYVHFPGEAQQVHFLRGFDKVWLEPGESWEACFELTRRDLSRWNVVSQSWELAQGEVELRVGPNAGEIKFTNTLQ
R
>tr|R4XB27|R4XB27_TAPDE
MLAILSVAIAIGAVQVNAISVAQQNSPAWQRALNLTSLMTNEEKANITTGTGLVARCSGNTSPVARFNIPSLCFQDGPAGVRAVDGTSAFAAQVNAASTWDIDLIYQQA
LAMGAEFRGKGVNVALGPVAGGPLGRTPYNGRNWEGYGSDPYLHGIAAYHGVRGIQDNGVIATPKHFIAYEQETYRWTGVLNAVAGIGGPNPSNNTNAEQISSDLSERT
LRELYLWPFEESLAAKPLAIMCSYNRINGTDACADGTLLNDLLKEELEFPGFVVSDWLSVFMAGSTNRTMNNGLDLDMPGGEGYWGSDLVTQVSNGTIAQSRLDDAVTR
ILYAYLEAGQDVGYPAVNYNSLTPAAVDAVGNVNKYLNVRANHSQIIRKVGEDSAVLLKNVAQNATGGLPLKSGARLAIFGTDAGPRPGGPNYGPTNGYPANSTNNGTV
ALGSGSGSAHFPYLIDPLAAITGAADLQKWQVSPVLLDYPANVTTGAEILNEYDAAMSGADTCLVFVSSFSGEGYDRTTLKFDNRGDQLIYYVASRCNNTVVVSHIVGV
TNFEVPFSHPNVTAILNAGLPGQESGAALVSVLTGNTNPSGKLVYTILQNDNDYIPVNKTASSDPQAVFTEGLLTDYRAADAMNLPVRYPFGYGLSYTTFGYSNIKVTN
ITGYASAVRSQTLMNVTATVTNTGGLAGREVSQLYISFPSGSGEPPKVLRGFTKSSLDPTQSTTVSFPIRKKDIQIWNETAHSWQVPSGQFTISVGASSRNLPLTSTFV
I
>tr|A0A1Q2MA35|A0A1Q2MA35_9GAMM
MAGLLSLAATLPLQAANETVAAAEPQHQQQDGIEARLQNLLAQMSTQEKIGQLALRDWGTFGASDMKAIKQAIREGRVGGFLNVSFSAVDDEAFAELQRIAVEESPLGI
PLLFGQDVIHGYETIFPIPLGQAASWNPELIKNGARVAAQEASADGIRWTFAPMIDISRDPRWGRIAETLGEDPLLTSVLGVAMVEGFQTANLADPSSLAACGKHFAGY
GAAEGGRDYNSAYIPERLLRDIYLPPFKAGIDAGMQSIMSTYSTLNDVPGTGSPFLFKQILRDEWGFDGFVVSDWNAVMEMVPHGFARDAKHAATLAANAGIDMEMHTD
TYEQFFPQLMDEGKFSETQLDTAVANILRVKLRLDLWSTPYPRAKSKAERKQIIRNDQFLSAAKEAAKETFVLLKNDKQLLPLKKGQTVAVIGPLAEAAHEQLGTWIYN
GDKKYSHTLLPALREMVGDEGEILYAAGLDYSRDTRTRGFKSALKAARKADVILFVGGEEAILSGEGHSRGDIRLPGAQADLVAALAETGRPLAMVLLAGRPLQLDDTL
EQADAVMMAWHPGTMAGPALADVLYGETSPSGRLPLSWPVGAGQIPIYYNHLATGRPPTDDNYTRIEKIGREVFQHQPGNSSNLLDYGHKPLFPFGYGLTYSAFEYKDL
ALSDTTLNADGQITVSATLTNTGKTTATETAQLYVRDLVGSVSRPVRELKGFERITLNPGESRLVQFTLSPDQLAFHNADMRQVVEPGEFRVWIAPNAESGLEGGFTLK
>tr|A0A4R5TSP5|A0A4R5TSP5_9GAMM
MTRETDTLSNAVLAGLMAVALTAPAAAGGDASAGTPKAAFSSAGDRAAERAFVDALMAKMTLEEKLGQLNQPPGVGNHTGPEAMAGNEDQIRRGEIGSFFGTHGVELTC
RLQKIAVEETRLGIPLIFAYDVIHGHRTLFPVPLGEAASFDVEEVRIAARHAAVEASAHGIHWVFAPVLDVSRDPRWGRIVEGAGEDPYLGAVLATARVQGFQGDDLAA
PDTVLATAKHFVAYGAADGGRDYDTAEISERTLHEVYLPPFKAAVDAGVQSIMAAFNDVGGVPMHAHGGLINGLLRGQWGWDGVLVSDYTGIMELMPHGVAANRQEAGA
LGINAGVDIDLVSRIYYEDLPAAIADGRVSMEQIDEAVRRMLNAKYRSGLFDDPYRYCKDPAREAALTLNPEQREAARRLAQKSFVLLENDNATLPLSKSLPTLAVIGP
LAEHRQAMLGNWAVAGRQEDVVTPLEGLQAALGEGTRLVVAAGTDIEGGDRSGFDEAIRAAQQADAVVMFLGEHPDMSAEAHNRTSLDLPGAQEDLALAIAATGKPVVV
VLLNGRPLSIGALQGKVGAVLEAWFPGVEGGNAIADVLFGDVNPSGKLPVTFPRNVGQIPIFHSHRNTGRPPSEEEKYTSKYIDVPWTPLYPFGHGLSYTTFAYDAVQV
ASPRVSKDALQQQVSVRVTNTGQRAGEEVVQLYLRDDVASVTQPVRRLRGFQRVALQPGESRTVTFDLGFADMALLDAGLRKVVEPGTFTVFVGGSSTAELEARFEVVR
>tr|A0A5C6CW83|A0A5C6CW83_9BACT
MKTLCTKKRVSISIVLFATIINPSCAAPGAAYQDASLPVEKRLDDLLSRMTLEEKVAQMRMFHANLGIRFSDDDQLILSEDVQNRLAQGIAGIKNPGEHLTPERAALLN
NQLQKYTIENSRLNIPALFVTESYNGVDAHGSTRFGRPINMAATWNVELTRSIWDAIGREARLRGMHMCHSPEADIVRDPRFGRMSEAFGEDTYLTTEMIVAAVTGVQG
GYDGTGRNTTHIGAVTKHFAGYGQVLGGTNFAAIEISPRTLRDEIFPPFQAAVQRAHTLGIMASHGDINGVASHANPWLLTEVLRGEWGFQGYVVSDSNDIARLHSFMK
VAETPEAAVKMAIKAGMNVDLYSDIAFSLLPKMAKQDARLMKYIDRSVSHVLRTKFILGLFDNPYIKLDEVKQGVRASSSLELAKRMDEESIILLKNKKKTLPLIKEKV
GKVALLGPLLNDSTKAAFEAAAGSAVEFSAEKGFKLTNEVRGVPELTKNNEQAIENMVSMAQEADVAVLFLGGDEFTAKEAFFNGALGDRDSIDPVGQQDELMRQVKAV

GKPVVVVLKHRRTLSISVIAEEADAILDCWDLSEFGDEVLAKMMFGEFSPSGKLPVTVPRSIGQLPFHYSQKEINYKKGYLFSKPGPLFAFGHGLSYTSFKYANLKLSD
HELARDGKLVVSVDVSNTGEFAAKEVVQLYVKDLIGSVTRPDKELKGFRKIELKPGETRKVEFTITPDMLAFTGLDMKPVLEAGNYDVMVGTSSAEHQKASFRLKYCFR
>tr|A0A167UWX7|A0A167UWX7_9PEZI
MPLSYSISLAALAGLLPAFAAAASLPIYKNASYTAQERAADLLPRLSWQDKVGQMGGVRRILAANLAFNQTSYDALTEYQNGILGFGNRLNDPARVLQMANQLREDWAN
KSLVPFITVTDTINGPYVEGGTLFPPTLSVAATFNVDLYGDVVAAIRDENMALGTHWVLSPELDVPKDPRYGRVGETYGEDPLVVGRFGLKYVDTMQEADEDGYMKVAC
TIKHFIYGNPNGGINLASQYGGLNYLYNNLFPPFIQVIQEADPPPASVMVSYTSVDRVPMAVNKYLLQDHLRQNIGFRGVLMSDAGEVPNLYTLSLTADSAETAALRAL
RAGMQLELAPSDAAFPRLIDHVNETDVADLINQAVRQILEIKFQTGTFDKPLPTVDNLKATFRAPAHLAINRNFSREAIVLLQNENNTLPLSVNRNGTGKIALLGPFAD
VVVAGTYAASNATNKTFGNALRQSLEAAVGASNVLYEPGVDFVDTSNASGIAAAVAAARDAGLAIVNLGSLAVQVEDPLAKKNTDGELYTHADLGFPGLQQDLLDAVLD
TGVPTVLVITGGQAFALHNRTLSRTGAILHSFLAGEYTADALVEILVGKVNPSGKLPISMPESSGSVPVAYDYLPSDGVLWTYPGTSRAVKYAFGFGLSYTAFDYAQPS
VTKATTAHGEPAVNVSVTVTNKGAMDGQEVVQVYFRQQYTSIETPTKRLIDFAKVGIAAGAAQTVTFTIKVNDLGFYVNGDWTWEAGNYTFYVGSSSRSEDLAPVSIVL
>tr|G4QE37|G4QE37_GLANF
MSTKFIQAFSNLKLNLEYSFSMSNSPAFNLYSADISKKAKQLLSKMTLDEKLGQLSQVCSPGAHIPDYLAESIRQGRISSVINEVDLNVNNALQRIAVEETRLGIPLLI
GRDVIHGFKTIFPIPLGQAATWSPEIVEKGARIAAEESSKAGVNWTFAPMIDIARDPRWGRIAESLGEDPHLCSILGAAMVKGFQTDDLSSIGSIAACAKHFAGYGASE
SGRDYNTANIPENELRNVYLPPFHQAAKVGAASFMASFSDLNGVPATGNRWLLKQVLREEWNYQGVLVSDWESIKELQVHGLSANEKDSAYLAAKAGVDMEMASTCYID
NMAALIAEHQIDEAEVDQMVLNILQMKFALGLFENPFTDPQTLPELVNPSNRNAAKDAAQKSCVLLHNRSRFEQRPVLPIDRESTKRIAVIGPLANDGYEQLGTWIFDG
EEKHSVTCLAGLEALANDSTGIVFEPVFANSRDRDTQHFDKALDLVNSADVAIVYLGEESILSGEAHSRANIDLPGAQPELIDYLSQSNTPIVLVVLAGRPLILESLLD
KVDSILYAWHPGTMGGLAIAELLFGEASPSGKLPVSFPRVLGQIPIYYAQKNSGRPASEDKYVYIDDVPERAPQTSLGMAATHLDTHFSPLYPFGFGLSYARFEYHEVC
ISHSEIKLGSAFEVSLIVENKSDVDADEIVQLYIRDLVGSVTRPVKELKGFKRVTVKANSSVPVTFKMHTDDLAFYDICNQLNAESGDFLLGVGADSSLELNIPFKLVK
>tr|A0A340YCS6|A0A340YCS6_LIPVE
MKLSLLGLAMGLVSQAAVAATSAPPLENKQAFIDHLISQMTEAEKIGQLRLISISPEMPREKIREEIAAGRIGGTFNSRTAPENRPMQDAAMRSRLKIPMFFAYDTVHG
ERTIFPIGLGMAATWDMDAVAKVGRTAAIEAAADALDMTFAPMVDIARDPRWGRTSEGFGEDTYLTAKIGQVMVRAFQGSSPANPDSIMAIVKHFALYGAVEGGRDYNT
VDMSLPKMYNDYLPPYRAAIDAGAGGVMVALNSINGVPATSNTWLMNDVLRKEWGFKGVTISDHGAIQELIRHGVARDGREAAKLAIKAGIDMSMNDTLYGEELPGLLK
AGDVSQAELDQAVREVLGAKYDMGLFKNPYVRIGAAQNDLKDYYGEDRLHRDAARDVARRSLVLLENRKQTLPLSKDATIALVGPLADAPIDMMGSWAADGRPNHSVTV
REGLRRAIEGKGKLVYAKGSNVTGDKAILDYLNFLNFDAPEIVDDPRPPAVLIDEAVKAAQQADVVVAVVGESRGMSHESSSRTSLQVPANQRALIEALKATGKPLVLV
LMNGRPLSIGWEREQADALLETWFAGTEGGNAIADVLFGDYNPSGKLPITFPRSVGQIPMYYNHTRIGRPFTPGKPGNYTSQYFEEPNGPLYPFGYGLSYSTFELSGLA
LSGNKLKRGDTLQASVVVRNTGKVAGETVVQLYVQDVSASMSRPVKELKNFQKLMLKPGETRTVTFSISEEDLKFYNGQLQRVAEPGTFNVQVGLDSEAVQQQGFELL
>tr|B8E050|B8E050_DICTD
MEEKEILKKVRDLISKMTLEEKIAQLQSVFGKELVDESGNFSEEKAEKLLKNGIGQISRVAGEKGMDPERAVELANKIQKFLKEKTRLGIPAIIHEECLSGFMAKGATV
FPQAIGMASTFEPELIRRVSDVIRQHMRAANVHQGLSPVLDIPRDPRWGRTEETFGEDPYLVSRMAAEYVKGLQGEDWREGIIATVKHFTAYGISEGARNLGPAKVGER
ELREVFLFPFEVAIKEGQAGSLMNAYHEIDGVPCASSKFLLTKILRWEWGFKGYVVSDYIAIRMLENFHRVAKDAKEAAVLALEAGIDIELPSVDCYGEPLIQAVKEGL
ISEEVINASVERVLRAKFMLGLFDGDLEKDPKKVYDIFDKPEFRELSREVARRSIVLLKNDGILPLSKNIRTVAVIGPNADNPRNLHGDYSYTAHIPSVSETLEGVKIP
EECAVRTVSILEGIKNKVSAETQVLYAKGCEILSDSKEGFDEAIEIAKRADVIIAVMGEESGLFHRGISGEGNDRTTLELFGIQRDLLRELHKLGKPIVLVLVNGRPQA
LKWEHENLNAILEAWYPGEEGGDAVADVIFGDYNPSGKLPISFPAVTGQVPVYYNRKPSAFTDYVEESAKPLYPFGHGLSYTTFEYSNLKIHPEKVNALEKVEISFTIK
NTGVREGEEVVQLYVHDQVASLERPVKELKGFKKIHLKPGESKRVTFILYPEQLAFYDEFMRFVVEKGIFEIMIGSSSEDIRLTGTFEVLETKVITEKRKFASEIKVE
>tr|W2RR05|W2RR05_9EURO
MASPTLIALVALLSLGVHAQDHPDCESGPLSNNSVCDTSLPPRQRAQALVDAFTLQEKLNLTHNNSPGVPRLGLPTYNWWGEALHGVAAAPGVDFAEDGEWSYATSFPQ
PITMAAAFDDDLIYAIGDVISTEARAFNNANRSGLDYWTPNINPFRDPRWGRGQETPGEDTYVVKRYIDNMVTALQGGRNLEQYKIIATCKHYTAYDMEFWQGNTRYGY
DAKVSPHDMSSYYMQPFVQCARDTKVGSIMCSYNAINGVPACANGYNIETILRGHWNWTADENYITSDCTSIQNMYTDHHAFDSRQQTVAAALNAGVDVDCGYYNPTWL
ASAYSQGLFDEATLDTSLLRLYTALVKSGYFDPPTSPWRSLAWSNVSTPDSEALALKIAEEGIVLLKNDNDILPLTISSDRNYTILMAGGWINATEQMQGIYAGPARTL
VSPWMALQNVSNLVVETFQWYESPLLVAERLQPDLILWIDATNEGAEETEDRNTIKWDLMQVDALEMLALTGIPTVAVHMGEQCDDAAILANDNISALVWAGYPGMLGG
QALVNVLLGDAAPAARMPLTQYPTDYVHLVPMTDMGLRPNNETGNPGRTYKWYDNATIDFGYGLHYTNFSAAISPPSNTTSTFDIASLISSCDQTGLTHVELCPFLPLS
PLQVNVSNTGAVTSDYVALAFIAGEFGPAPHPRKSLVAYQRLFGVAPGSAQMAKLNLTLGSLARHDEMGNQVLYPGRYRVEVDVPVQDVWEFELTGGEVVLDEWPQEQ
>tr|A0A0J1FYP4|A0A0J1FYP4_9FIRM
MAVLQECLQYKMGTIMKQTELEQLLKSMSLEEKINQMLQVTGDFYLGKTVITGPMRENGFTEESVAQAGSVIGLAGADVVKKVQKEYMEKQPHHIPLLFMLDVINGYKT
VFPIPLGQGAAFEPEMSKSCAKAAAKEAAVSGLHITFAPMVDLVRDARWGRVMESTGEDTYLNCCFSKAMVEGFQGSDLKEPYRVAACIKHFAGYGAPDAGRDYNTVEL
SEHTLREFYLPAYQAGIEAGSALVMTSFNTIDGVPATGNKWLMRDILREEMGFDGVLISDWAAMEEIIYHGYCEDRLDAAKRSIEAGVDIDMMTGIYSERLQELIEKGK
VEERLIDEAAMRILELKNKLGLFENPYKDADEEKSREIILCREHRELARECARKSFVLLKNEGALPVEKEQKIAFIGPYTDSREILGAWSFIGKSEDAVSIKDAAMEVL
DQSRTTYCQGCPVLDDTVKLEGFAAGNEAEGKSNNTANPEDEEQKQAEMLNTAIKAAKEADVVIMPLGEHRLQSGEACSRAEIVIPEVQMDLFRKICQVQPNVVVVLFN
GRPLDIREISQKAKAVLEVWMPGTEGGHAIMDVLTGEYNPSGKLTMSFPYSVGQVPVHYNEYSTGRPHVPGKDKDRFRSKYLDIPNAPLYPFGYGLSYTDFEVSKTELD
HREMQPEDTVRASVSIRNTGNIAGTQVLQLYIRDVKASVVRPVKELKGFQKVHLQPGEEKTVTFEIREEMLRFYSENKVFESEAGEFEVYIGVDSSVEECDRFYLKK
>tr|A0A175W7P6|A0A175W7P6_9PEZI
MASLCGFALAFAALIAPASAGVRYPDCANGPLRSNLVCDTSADPAARAGALVAAMNNNEKLANLINNSPGVSRLGLSPYQWWNEALHGVAHNRGITWGGEFSAATQFPQ
AITTSAAFDDPLIERMGVIISTEARAFANNGRAHLDFWTPNVNPFRDPRWGRGHETPGEDAFRNKKWADAFIKGMQGPGPIHRVVATCKHYAAYDLENSGSTTRFNFNA
RVSTQDLSEYYLPPFQQCARDSKVGSIMCAYNAVNGVPACADSYLMDTVLRKHWNWTDDNQYIVSDCDAVYYLGNANGGHRYRPSYAAAIGASMEAGCDNMCWATGGTT
PNAAAAFNARQFSQATLDRAMLRQMQGLVRAGYFDGPNSPYRNLGVRDVNTQAAQDLALKAAEEGIVLLKNDGVLPISLDGSNSQVAMIGFWANAADKMLGGYSGSPPF
RHDPVTAARSMGINVNYVNGPLTQSYADTSAAVNAAQRSSVVIFFGGIDNTVEKESQDRTSISWPSGQLNMIQRLAELGKPVIVVRMGTHVDDTPLLSLPNVKAILWAG
YPGQDGGTAIMNLITGKASPAGRLPITVYPSSYTNQAPYTNMALRPSSSYPGRTYRWYKNAVFPFGHGLHYTNFSISVGDTFPKILSIPDLLLSCNGVAYRDQCPFPSV
PITVTNTGSRASDYVALGFLAGEYGPSPYPVKTLATYKRLFGIQPGQEQTAQLEWKLDSLARVDQNGNTVLYPGKYTLQLDEPMIANITFVLTGDEVVLDNFPQPPA
>tr|A0A428WPZ1|A0A428WPZ1_9ACTN
MTPPYRRAALPVGDRVRDLLGRMTLTEKIGQLNQRLYGWRAWRRAGDGFAATDQLTAEARRYGGIGAIYGLQRADAWSGQHWGTGVTAAEGAELCATIQRAVTAESRLG
IPALFVEEVPHGHQALDGTVLPVALAVASTWDPDLYERACRDVAAEVRARGAHVALVSTLDILRDPRWGRAEETFGEDPYLAAAFTTAAVRGMQGGGADGPIPPDRLAV
VLKHAAGQGATVGGRNWAATELGWRELAEIHLPPVRAAAEAGAAGLMAAYSEVDGLPVAANRRLLTEVIRGDLGFAGLVMADGTALDRLLRLTGDPASAAAMALRAGID
LSLWDEVYPHLAAAVERGLVAETAIDRAVERVLAVKFRLGLFDPLAAADPAPRETTRLAAELAARSVTLLTDDGLLPLTGKRVAVLGPHADTVAHALGDYTAPQRPKTG
VSIAEALCAAGVRVTTAPGADIATDDESGIPAAVAMARDADVAVLCLGGSSARDAETRFDVNGAARPEGPRAQMTAGEGVDLAHLRLGAGQLELLRRTAATGTPVVAVV
VQGRPHVLGEVLATAGACLAVWYPGPTLGTAVAGVLLGRREARGRLPVSLPRDAASLPVHYNHRDHAWHGYLDAPPGPALPFGAGLGAHPLVLSEPRLVPDGPVGVADL
HAGARLVCRVTLRNDGPVPASAVVQLYLHRVTASTWPRARELRGFQHVRVPAGGSMTVELPVGAAQLSTVDDTGRPLLEAGVVEIQTGLSAADVTGVRLTLTGWPR
>tr|A0A512HBQ3|A0A512HBQ3_9PROT
MAIPTIPANTDDAATTSFVDGLMARLSLAEKLGQLNLLTASLPIGKERADEIRAGRVGGLLGGLFPCSFGVGGPRALREVQEIAVHESRLGIPLLLAYDVIHGHQTVFP
IPLALSCAWDMAVVETAARWAAREASAQGLNWVFSPMVDIGRDPRWGRIAEGPGEDPYLGACVARAMVKGLQGDDLRQPDTVMACVKHFVGYGAAEGGRDYNNADMSPA
RLREVYLPPFQAAVAAGAGSAMLSFLALNGMPSHADAALIEECLGGLLTVSDFDGIPEMIAHGLGDEEGRIDEADPLQTLAERSLRAGVHMDMMGHAYATRLEAALAAG
RVTQAHIDAACRAVLRAKVRLGLFDDPFARFDEARAARTVHAPEARAAARAAAAQSCVLLQNKGDLLPLPRQTLNLAVIGPLADDTANILGPWSFQGDPGTAVSVLEGI
RQAAGPGVRVAHARGANITDDPRMLELANFAGPRVVNDPRPPETLIAEAVAVASAADVVVAVMGEASEMSGEAASRQDLGLPGGQRRLLEALHDTGTPIVLVVMNGRPL
TLSWETDHLPALLVAWFGGCEAGAAIAELLFGDRAPSGRLSTTWPRQVGQIPLSYDHASTGRPGDPKADPGKYSTRCYLDGPSTPLFPFGFGLGYTTFAYDDLAVDKAT
AVGADTVMVSVRVTNTGSRAGQETVQLYVGDPVASVVRPVRRLRGFQKIPLEPGEARRVSFEVTTRDLEFFHPTRGWGWEAGAFLLGIGPHAEATQTVRVDWRKEA
>tr|A0A1G7L574|A0A1G7L574_9SPHN
MTAARVLDRRTLLARAGLLAAAAAIPRPAGAATTRINALIARMTIEEKAGQLSCFNDEIRPVGAVFNPVVNAQGAAAQLADIRAGRIGMLFNGYGAQGAIRAQEAALAS
RLRIPLLFAADLIHGCRTIFPIPLGEAAAFDGDLSRRVARAVAVEARAAGIHWTFAPVVDVARDQRWGRVAEGAGEDVALNVALAVARVRGFQGAGLSGADALAATPKH
FAGYAEVRGGMEYGAVDMSDAQLREVVLPPFAAAFRAGAAATMAAFTAFNGIPATANRHLLTDILREDLGFTGVCVSDYDADRELIAHGVAADEADAARLAILAGIDMS
MQSGLFQRHLPALVASGAVPIAVVDRAVARVLALKDAFGLFDDPFRGLRQPAAPPPTAPPTALAREAATRSIVLLRNEGAVLPLAPATRVALIGPFGADRTHLNGPWS
FAGRAEDGVDLATAIRPLVVEPGSGIHHPLPGGIDRAVAAARAADVVLLAIGEGADMSGEGNSRVAITVPAAQQALAEAVAATGKPLVILLRHGRALALEGAVRDAPAI
LATWFLGAQTGHAVADILFGRAEPSGRLPVSFPFATGQQPWSYDRPATGRPAPDDAPLAPGRAHWRDAPDRALFPFGAGLSYTRFALDRVRVPATIPYGAPVPVSVRVR
NIGPRPGLACIRLDIHDRVASRIRPVWQMKRWLRLSLAPRAARHVTFTLTPDDLALITPTGHPSVEPGAFDIRLSEGMEATAFTSFTVTPHDAPILEINGGSSVP
>tr|A0A1J4JN71|A0A1J4JN71_9EUKA

MNSSQIYSMIFYSKIRKKFCFTFFFVSSTTMTEIYLDPSQPVEARIKDLMSKMTIEEKVGQLVQLPGYANPQEGQLMFMHGPCEQAIKDQHVGSFLWVVGENAKIPINL
QKETRLKIPLLFGVDAIHGLSFIDGSTLFPTQLGMAPSWDEDLLKEVASTTAKEMRYNGTHWTFSPVLCIARDLRWGRVGETFGEDPYLIGRFASAMIKGYQGDQISSD
QDKILACAKHYAGYSETIGGRDASEADLTPRKLLSYFLPQFKKACEAGVGTYMTGYQSIDGTPSTANQWLLREILKEEWGFKGFLVTDWYNVGWLVHDQKICKDFEEAA
AVAITCGNDMIMATPEFYEGCLKALKSGKLEEKYVDEACERILRIKFQLGLFEDPRMPDISKVQMRSQEALDLCLRAAQEGAVLLKNDNILPLCASSLKKAAVVGPNAD
HPGAQNGDWAAGTSQTSDVPPPPRENCKTVLDGVKANFQGEVLYCRGASIDPEETSDFSKAIEYVNDADVTIVVVGDRERYWGESKSTCTLELQGDQIDFLNKIVETGK
PFILDIISSKPLVIPANIRNNARAIIQQFSPGQMGGQALANIVFGKYNPSGRLTISIPYHVGQQPIYYNQVRGQHATRYADMTQDPCWAFGYGLSYSKINYESATINQK
EFKVGDNLEVTVTLKNESDRDGIEVVQVYIHDEVTSATWAVQELKGFERVTVKAGETKQVKVTVPISECSIVNTKCQRVVEPGTFQCRVGRSSNDFAFVLPFEVI
>tr|A0A1I2VTE1|A0A1I2VTE1_9EURY
MDSETENGRPRPEVSADDIDELLAEMTLEEKAGQLAGTYVGKLGTEKTLEDAEREITEDHIGAVSPFGIGITTLDDPARAAEVANRLQRHAVEETRLGIPLSVPVDAVH
GHAYINGATVFPHNVGMAATRDPTLVERGGEITGREARATGATLNYGPTLDVARDQRWGRVFETYGESSYLCGELAAAEARGLRGDGDVDETLRVSSANQNAERSGDDP
TIAATAKHFPAYGQPERGEDASVVEISESTFRRTFLPAFERVLDEGVDAVMPCYNSIDGEPAHGSERFLTDLLRGELGFDGFVTSDWGGVDHLHEDHGTSDSRRTSAWQ
AFTAGLDLVSVGGPDYAEEVVELVRKEELSERRLDESVRRILELKARLGLFEDPYVDPDGATDTLANDDHREASLDAARDSMTLLKNDGVLPLSESLDDVLVTGPNADD
LHHQFGGWSVMDDSDLQGVTVREGIEDVVGEESVTYEPGATITEEADVDAAADAAEDADAAVVVLGEDWYIHEFGPQQMNRPTGDFPTRTQLELPEAQQNLLEAVQATE
TPTVLVLVSGRPLSIPWADENVPGILTAYYPGMDGGTAIAETVFGRHNPSGRLPVSVPRSAGHLPTVFDYLPHPTPIGADEHPDSYDPLYPFGHGLSYTDFEVSGLTLS
SESIGPAGEITASVTVENVGDRAGDRSVDLFVEDVVSSRVTPVREHVGFAKASLEPGESAELEMTVPADALAVRGRDYERVEPGEFVFSCEEETTTLTVEPGRN
>tr|A0A1Y1WEX9|A0A1Y1WEX9_9FUNG
MADYSSVAGDASASVPASGTNTAPATQPDSSPYTSNSADSASAVQTTVTPTGTPVPSGTVAVPSAGYSSSGTDELPASTALPSDKCTWDKVQIPDPVMLNPVKSYSYQP
ESSVVQAPGKIDKDIIQMVRGLTLEEKIGQMTQIEVGQIIDCNGKLNRTASPGNHDGIYQWYSAEKFAELTDAVQEIATKKGAKIPVIWGLDSVRGANYVKGAVIFPAG
IATAATFNPQFAYDAGRVAAKDTRAAGAHWAFAPVLDIGVNKFWSRTYENFGEDPFLSSQMALIRSRVAACMKHFIAYGNPFDGSDRADRHVADHELLEYYVPSFKAAI
DAGSATAMESYGAVSGETVMLSNFYLQTLLREHLGFKGFMVTDWGEILSQATIYMTARDPEQAVEGAILRTSTDMSMVADDASFSNITLALSVARILQAKKDLGLFENP
YADKELQKTVGSAQDIESGRDTARESITLLKNENSVLPLKTSESVLFLGPTLNSSRYMGGGWNEGDAVYQGFADTVLTGIKQVTGSEPSYMPAVDIDGTTRYDWDGVIA
AAKKADKVVIGLGERTYAENFGNIDELTLPQAHIDLVSSSLCWSKDAPVSSAASLMTPNAIVNAYLPGSYGGLPIAEILYGKVNPSGRLPYTYPAHSSQASHTIWQASY
VPYKPQWQFGFGLGYSTIKYSNVTVSSTTLEIGSPITVSVSLTNTGTYDQKEPVEVKAGQTKQVELTLKAEDMAFWDRYLRRRIEPSPATVQLEADPQQVLERL
>tr|A0A239A3G8|A0A239A3G8_9PROT
MRIVRLKKIIPPLLLAGLLGGAATVAMAQGNAAAPAATAAAQAQPWLNPALAADERAALALAAMTLDEKKLLVFGHFGSALKYKNYLPPKEVRMGSAGYVAGIKRLGIP
PQYLTDAGVGVATQRESLAPYLERTSLPAGIATAATWNPALAEQGGAMIGREARASGFNVMLAGGINLVREPRNGRNFEYGGEDPWLAATMVAAQIKGIQSNQIISTIK
HFALNAQETGRFVLDARIAEDQARMSDLLAFEIAIEKGQPHSVMCAYNRYNGPYACESDFLLNQVLKKDWAYPGYVMSDWGAVHSTAPAANAGLDQESGYIFDKKPYFG
PELLGKALKDGSIPQARLDDMVRRILRALFASGAVDNPVSIQPIDYGAHALVTQADAEEGIVLLKNDANLLPLAASAKRIAVIGGHADKGVLSGGGSSTVFARGGNPVP
GLGPKDFPGPIVYHPSAPLEAIRRRSGDVRFDPGTDVAAAAKLAAESDLVILFANQWTGEALDFPLTLPDGQDALIAAVAKANPKIVVVLQSGGPVLMPWLAQVPAVLE
AWYPGTRGGEAIARVLYGEVDATGRLPVTFPQSLDQLPRPVLDGTDKKPDVSFPVTYSEGAAVGYKWFDLKGHKPLFAFGHGLSYTSFTYEGLAAEMTADGLKLRARVT
NKGSRFGSAVPQFYIGPAPGVTAGWEAPRRLVGFDKLALAPGESRQVEVTIDPRLLAIYDGAAKGWKISGGTYQVQLARSAVDFVQQADVTLPARQWSAAKGAK
>tr|A0A0W1A3I4|A0A0W1A3I4_9GAMM
MTDYKTWCAVLLYGIISSVHSQIAEQKEAYINNLLARMTLAEKLGQLNLLTVGLTATGPVSNQDIDNKIKNGQVGALMNAYSPKVVKQLQDLAINNSRLKIPLLFGFDV
IHGYRTVFPIPLAQASSWNPELIKKLAHASAAEAAQDGLHWTFSPMVDIVRDPRWGRVMESAGEDPYLAAQIGEAMVKGYQGTDLSQPSSIMACVKHFALYGGAEAGRD
YNTVDMSLPRMYNEYLPPYVAAIKANAKSVMTSFNDVNGIPATGNYWLMNKLLRDTYHFDGLLVTDYTAINEMMAHGVGDDQEVTRLAMNASVDMDMVGEMYVNQLPKL
VNNKSIKEEQIDAAVKRVLRAKWDLGLFESPYHQMDSLAYDKKKHQALAHEAALESMVLLKNNNHILPLNPKQKIAFIGPFVKDANQQLGEWRAIGRPEEATSLWQALQ
KDQDAKKILYAKGCNFSDDKELITYLNQHGGSIKNDDPQKLLNEALSIAKKSDVVIAVLGEPFGMSGEAASRTRIGLLPNQSDLLKELKKLDKPLVLVLMNGRPLTLEW
PHHHVDAIIEAWYGGSEAGPALADILFGRYNPSGKLTMTFPRHVGQIPIYYNAKPTGRPYRQPNLRFGEKYKSKYIDESNLPRYPFGYGLSYTQFEYQDLKLSSSVLHK
NTPITASVTVTNKGSYPGHEIIQLYLHDKIASTTQPEKQLKGFRKIFLRPGESQLIQFTINESMLRFYNARLDYVSEPGEFELYIGPDSQTNKKISFSYSSDG
>tr|A0A553FCL0|A0A553FCL0_9GAMM
MFTKLKLITLSLCLFTVQALAADKATEDKIKKLIASMSIEEKVGQMTQLDVREVSVTGPEGVAVVKAEKLKKIIHKYKAGSILNAAGQPMNIEQWGSLISAIQDEALST
EHQIPILYGIDSIHGVTYTAGSTLFPHNIGLAASLDLELSNQIAKVTAMETRASGIRWNFDPVLDIGRQPLWSRFEETFGEDPYIVEQMAVGMVKAYEQDGLDKPTAVA
STMKHFVGYSAPDNGKDRTPATISDKDLWEYYLPQFQAAVDAGSSTIMINSASINGVPVHASKHLLQDVLRDKMGFKGLIVTDWEDIIRLHTRHRIAATPREAVKIAID
AGIDMSMVPNEMSFFEHLTDLVKSGEISEQRINESVAIILRLKFELGLFDNPYPEQEAIANFGKKQYQDLALESARATMTLLKNETLSKASASILPLTKNDKVLIAGPG
ANNLGPLHGSWSYSWQGSNEERYPESTLTLVEAFQAEVGADNVTSFAFTGYDNLANYDTDGLAKAAENVDYIVLALGEGAYAESPGALDDLTLDKNQIELAKAAALTGK
PVILVLLQGRPRIVKEIEPAMKAILLAYRPGSKGAEAIVDTLYGKHNPSGVLPFSYPQFTGDHSTYDRNFLTNVQQLNVSGVSFNGHKPQWKFGFGLSYSNFTYSKLSL
NKSTYSADDKISVSVQIKNTGDKAGEHAVQLFLSDLYASESPDFKRLKRFSKVKLKPGQSKTVKFELTTKDLSFVNSALERVVEDGEFKIAVGDLTKNFNYKK
>tr|E0STZ1|E0STZ1_IGNAA
MDIDERVRELIGRMSIEEKIAQLISIPLESVLDGKKFSVEKAREVLKYGVGEILRIGGSSARLSPREAVEIYNAIQRFLTRETRLGIPAIVHEESIAGLLAPTATVFPI
PLALASTWDPDLVYRVAVAIRRQIMAIGSRHTLAPVLDLCREPRWGRCEETYGEDSYLAASMGIAYVKGIQGDDIRYGVIATGKHFVGHGVPEGGRNIASIHVGLRELL
EIYMYPFEATVKEANLLSIMPAYHDIDNVPCHANKWLLTDILRGSWGFKGIAVSDYEGVKQLHTIHRVARDCMEAAVKAIKAGVDIEYPSGECFKQLVEAVRKGLIDED
TINRAVERVLKLKFMLGLFENPFIDETKVPTTLDNEADRELAREVARKAIVLLKNDGILPLKRDIKTIAVIGPNANDPWAMLGDYHYDAHIGSFDGTYGKISPSVRIVT
VLEAIKSRVSPSTEVLYAKGCDTIGDDRSGFGEAIEIAKRADIIIAVMGDRSGLFNLKMFTSGEGVDRASLKLPGVQEELLKELASLGKPIILVLINGRPLALSSILPY
VNAIVEAWRPGEEGGNAIADILFGDYSPGGRLPVSLPYDVGQLPIYYSRKPNCFRDYVEYPAKPLFPFGYGLSYTQFAYENLVVESTEVRDPDTVIRVSVDVKNVGSMA
GDEVVQLYISRDYASVTRPVAELKGFKRITLEPGEKKTVVFEIPLELLAYYDMDMNYVVEPGEYTFMINKNAEETILKTKISVIGGTKIYRERRVFTSKAYVR
>tr|F7PQA1|F7PQA1_9EURY
MDENRPTDENPPYRDPNRSTEARVENLLSRMTPAEKVGQLVGTAPTLRPGRETVSGIAEAVTEHHLGAVSPFGHGGSPWETPEECVEVANAIQREARNTTRLGIPVLFY
VDADHGHGFVKGTTVFPHNLGMAATRDPELVERAASVTATEVAATGGHQNLNPVADVGREARWGRIYETFGESPHLCASMSAAAVRGYQGDDVGDADSVIATPKHFPAY
SDPVRGEDGSPVDVSEYTLRRVFRPPFEAALDAGAGSIMPAYNELNGYPVHGSKEYLTGWLREELDFSGYVVSDWNGINMLHHDHRTARSMEEAVWQATTAGVDVASVG
GVEHAERLLDLLESGDIAESRIDESVRRVLGAKFRLGLFEDPYVEADRVEADVGTDDHRAVAREAARESMTLLQNDDDVLPLDPDLDSIAVLGPNADDLRNQFGGWSTI
SEPEPPGTTIREGIERAVPAETTVRYEQGAEMTERVDVDAAREAASASEAAVVVVGETGYRHEFHRSETDRGEFPTRSELELPAAQRELLGAVRETGTPTVAVFVAGRP
LAMGWTVEHVPAILFAYLPGSEGGKAVADVLFGAADPGGSLPVSIPRSSGHLPTHFDYRPHPHPIEGSPREENPRPPEHPETYDPLFPFGHGLSYAAFETSDLSVSPER
VGPEGRLTATVEVENVSDREGSTALHLYGTDEFSSRVTPVRELVGFEKVRLAAGERTEVSFEVDVADLGVLDDPGERRAEPGTLGLSCAGESVEVVIEGRFDR
>tr|R7TKV4|R7TKV4_CAPTE
MRCAFVALSLLVAWVQSYDFPFQDPSLSWDDRADDLVARLTLEEIAPQTQASYGGQHTPAIERLGIKPYVWITECLAGQVNTNATAYPQPIGMAASFSEELLFNVSRDI
SYEVRAHWNANRAVGKYSTKVGLSCFSPVINIMRHPLWGRNQETYGEDPLLSGTLAQSFVRGLQGDDPRYLRANAGCKHFDVHGGPEDIPVSRFSFDAKVNMRDWRMTF
LPQFKMCVDAGSYSLMCSYNRINGIPACANKQLLTDITRDEWGFHGYIVSDSGAISNIKEQHHYTNSTVATVVAAIKAGTNLELGGGSNMYYPKQLDAMKQGLLTEKEI
RDNVRPLLYTRLRLGEFDPEAMVDYNKIGVDVIQSPEHREQAVKAAYMGFVLLKNHNNLLPIKKQYSKLAIVGPFTNATSELFGTYSSEVNLKFTSTIFEGLSPLGGST
RSANGCTNSACSGYVRDDVETAVAGADLVIVALGSGQRFESEGNDRAYLDLHGHQLDILKDAVFFSNGAPVILVLINAGPLDITWAKLDPGVTAILSCGYPAQSTGEAL
RRSLTMSEPQAAPAGRLQATWPLNLDQVPKITDYTMQGRTYRYYVGEPLYPFGFGLSYTSFSYTRLSISPSVITQGDNVTVEVCLKNTGSYDSDEVVQVYMSWPQTPFP
LPKWTLAAFARPFISAGQTICVKSVIRADQMAVWLSDDAGFGFVPGVMTVYAGGQQPNQRTKAPSNVLTSKFEVKDKPWSRTMKMKSDFSARENDVKSRFTKL
>tr|V6DQS2|V6DQS2_9EURY
MDNRPEQPAYQREGAPTDERIDDLLDRMTLAEKAGQVTGTWAGTLRETHDLDDVKEAIDDGHLGFAAPFGWGGALATTPEDAVSAVEELQTYAREETRLGIPLLFSVDA
VHGHAYVSGATVFPNGLGAAATWDPEGVEAAAAVTAREVRATGAHQNYGPTVDVGRDARWGRVFETFGESPHLVGALAAAKVRGYQGKGSDDPDGPAIPTDRVVATAKH
FPAYGEPERGEDASPVDVSEYKLRNTFVRPFERVLDAGVASVMPSYNSINGEPSHGSAAYLDRLLRDELGFDGHVVSDWNGIRHLHEDHRTAVDHADGVRQARTAGVDV
ASVGHTPHADRVVELIEDDRLDEATLERAVRRVLRLKFDLGLFDEEPEDAADETDAVETLGAPAHRERALETARDSMTLLQNDGLLPLSGDEDVFVGGPNADDLVNQLG
GWSVGRDEGVPGKTIREAVADATTGTVSYAPGTTMNEAIDIDEAVRAARDADVAVLALGEGWYIHEFGPEAQAGSETGEWPTRSDLRLPESQRELVRRVHETETPVVGV
LVTGRPLIVDWMAEHVPAILMAYFPGTEGGVAVAETLFGDNDPSGRLPISVPRDEGDLPQHHDALAHPTPIGADEHPDSYDPLFEFGHGLSYTTFETTDLSASVAARPG
GSGEDDAPTDRTVRVSVEVFNVGDRAGTETVQVYARQRHASRVRPVRSLVGFERVTLAAGESERVEVSVSVERLGFHKPREGHVVESGPYRFDVAGASTTLEL
>tr|A0A5C5BFD0|A0A5C5BFD0_9MICO
MPHHGEDPRTRSDRPWADPARPVEERVAALLDAMTLPELVGQLHQPANVDLERDAALLAAGAIGSTLHASGATAGNVRDGGVSRDRVDDLQRAAIESSRLGIPLLIARD
VIHGHRTVAPIPLGQAATFDEQVVHDVAARAALEASADGLTWTFAPMLDVVDDPRWGRVAESFGESPLLTARLGAAAVRGFQASGLVTACAKHYVGYGLSRGGRDYATA
EVGEITLRNRHLVPFRAAVDAGVGTVMAAFCDVDGVPMHSHHHLLREVLKGEWGFDGVVVADWNGIGELVEHGVAADLRDAARLAIEAGVDVDMVSGAYAAHLAALVEG
GEVDLELVRDAARRVLRLKVRLGLLDPDCASLPGRGDLAGSHGLGLDRALAREAAARSFVVLRNDGMLPLRPRPTDTVLLTGAYTRERASLLGTWVLDGDPEQVSAVAP

AVVRALGELAELAELTAGAGDAGGADADGDAVGVVAEPTVRRVLVDDGAFADRTLRLARDADLTVAFVGEHAARSGEDGSTSDVGLPPGQLEVLRGIAALGSRLVVVVL
TGRPLALGEVLDLADAVVLAWHPGTEAGEALADVLVRGVPATGRLPMSLPRSVGHLPITHAERPSGRPLPDEARGRGRYIDAPSSAGVPFGAGASALAYGSLTAASGEV
TRDGEAVVTLEVTNRSDREVREPVLLFLRDEVAEVTRPVRELVDVQVVTVPAGASVPVTFRVPAHAFGYHGRDHAFRVDPGRVVLTVGWPRADASAVVLDVV
>tr|A0A395JNH3|A0A395JNH3_9GAMM
MPHSLPAKLSTALASIMLLASPFTSSASNDIEQRVDELLAKMTLAEKAGQMNQYNGFWEVTGPAPAEGDAKNKYEHIRTGMVGSVLNITGTKDVRATQKLAVEGSRLGI
PLIFALDVIHGHQTVFPIPLAEAASWDLNAIEKSARIGAIEASARGINWTFAPMVDISRDARWGRVMEGAGEDTFLGQKVAVARVRGFQGDDLTDIDTIAATAKHFAGY
GFGEAGREYNRVDVGTNTLYNMILPPFKASVDAGVASVMNAFNTLNGIPATGDVFLQRGILKGAWDFNGLVVSDWGSGKEMMQHGFAKDLKHAAQLGVTAGSDVDMESY
MYVKHLEALVNEGKVDIALVDDAVRRILRLKFKLGLFDDPYKYINEEREATLLGHADHMAAALDVAKRSIVLLKNQGNLLPLSKTSGKIAVIGDFADDKNSPLGSWRFG
SIDNSAVSLLEGLAKYRDDIEFAQGVKVVTGEESFVQEITVNQTDATGIDEAVALAKKSDVVVMLLGEHGFQSGEGRSRTDLGLPGLQQEMLEAVYAVNPNIVLLIASG
RPLALPWAAEHIPSIAATWQLGSQSGNAISEVLFGDYNPSGKLPVSFPYNVGQMPLYYNRMNTGRPIDKDSNVFWSHYSDSPSEALFPFGHGLSYTQFDYSKLSINADD
PAKVDVSVTVKNVGKRAGEEVVQLYMHDKVASVARPIKELKGFEKIELEPGATKTVNFTLDSSKLGFFDREGNWIVEPGEFDILVGSNSREGLTGQFTLSMK
>tr|B8E3C1|B8E3C1_DICTD
MEKEKIEEKIDYLISSMTLEEKIAQLKAKTVSIWQTVGKILLEDVFSDLTPEFKEKIMGFLFGFYRDRELAEEMTKALWKKMWKKLVLEGEEKKYPIGELSCALRSMSP
RESAEFANEIQKYVLENSEIKIPVLIHDEALHGCMAKGSTIFPQAIGMASTWNPELIYQVATAIGKETRSRGIHQVLSPTINIARDPRCGRTEETYGEDPYLASRMAVA
YIKGVQEQGVIATPKHFAANFVGDGGRDSYPIHFSERLLREVYFPAFKASIKEAGALSLMAAYNSLDGIPCSSNKWLLTDVLRKEWGFKGYVVSDYFSVLHLMTKHKVA
ESKAEAARLALEAGLDMELPDSDCFEEMINLVKGGKLSEETINEAVRRILGVKFWAGLFDNPFVDPDYAERVNDCAEHRELALRVARESIVLLKNEGILPLSKDIGSIA
VIGPNAAVPRLGGYSGYGVKIVTPLEGIKNKMENKAKIYFAEGCGLNDTSKSGFDEAIKIAQKSDVAILFVGNSVPETEGEQRDRHNLNLPGVQEELIKEICNTNTPVI
VVLINGSAITMMNWIDKVQAVIEAWYPGEEGGNAIADVLFGDYNPGGKLPITFPKYSSQLPLYYNHKPSGRVDDYVDLRSPQYLFPFGYGLSYTEFRYSNLRITPEEIP
MDGEITITFEVENIGKYKGDEVVQLYLHDEFASVVRPVKELKRFKRITLAVGEKKTVSFKLDRRDLEFLNIDMEPIVEPGRFEVFIGSSSEDIRLKGFFIVK
>tr|F7B5X2|F7B5X2_CIOIN
MANIIKLLSALSWLVLCLGERDVSQILPFRDLTLSREERVEDLVSRLTVKELVLQISRGGAGDNGPAPAITRLGIGPYQWNTECLRGYAMNGDATCFPQPIGLAATFDQ
GLIYKLAKTIALEARAKHNNFTKNGNFGDHTGLSCFSPVINILRHPLWGRNQETFGEDPVMSSLMARAYVTGLQGDEIYYPATANCKHFAAYDGPENIPSSRLSFNANV
SIEDLGRTYFPAFRECVHSGAFGIVCSYNAINGEPACASSYLQTILRDKFNFKGYVSSDESAIEFFDIYFKYTKSNLLSAVAAFDAGVDLELTSYGKNNRYSLLNQAVE
QGLVTEAALRRSAKRLFRTRMALGEFDPQEFNHWLNVPIDVVQSLAHRKQAVEVAAKSFVLLKNDGVLPLNHRYENVAIVGPFINNSEALTGDYHPNYNLKYFSSPLFA
ANSLSSSGVARFTTGCVGTNNQNLPICATYNSTHVKEVVTGSDIVLVTLGTGTGIEAEGRDRLSMELPGKQLDMIKDVVKYANGPVIVVLFNAGPLDVSWVMGNTAAVI
ACHFSAQMTGEAMLEVLTGIVNPAGRLPNTWPASMQQVPPMTDYSMHERTYRYSTSSPLFPFGYGLSYTKFWYLDAVVEPTTIQRCQIPVVRVLIQNTGHLDGEEVVQI
YMTSKKKRDRELLRQLVAFQRVPIKAGEEVSISLPIPHLSRAIWMKHNRSFDYYFDADEFTLYVGGQQPHQVVKVPSNILELSFKYELEFPGQVISLSDCNV
>tr|F8DDY5|F8DDY5_HALXS
MTASPTYEDETAPVNERTADLLGRLTLREKAGQLSGLFASEVQADPYESYTLDDVEHAVREHAIGSVTPFATGFSSHNSPAVVPRIANRLQRIAREETRLGIPLLVPVD
AVHGHANVKGATVFPHNLGMAATWNPALIRRAARATAAEMRATGATMNYSPNADVAREPRWGRTYETYGESPHLVGELAAAEVAGLQNADSNADDTGGDPSSRDPLETT
AVAATVKHFPAYSAPARGEDAAPVDISPSTLHRVFVPPFDRAIDAGVAAVMPTYSAVDGEPAHGSRRYLTSLLRDDLGFDGLVQSDWHGVAFLYDRHRTASSPKEAAAQ
AVGAGLDVASIGGPEYAKHLCELVESGRLSEERLDESVRRVLELKFRLGLFDDPYVDPRRSREVVGRSAHRELSLECARESVVLLDNDDALPFNDPDEVLVTGPNADSL
DALCGGWTVADLAADHGTTILEGLSNATDDDTTVAYEPGATVREEINIEAAADAAVGADAAVVVCGENWYVHEFGPKSMTGPNDAFPNRTQLRLPDAQRRLLERVADTG
TPTVLVVATGRPLAIPDEVQVADATLAAFYPGYEAGQAIGEILIGATNPSGRLPISMPRSISQLPLVHDHRPHPQPLGGDEHPDAYDPLFAFGHGLSYAEFEYESIDCT
ADQIEVGNSLDVTVTVANRGDRAGTEVVQLYVRDIVSSRVTPVRELAGFDRVSIPSGEKQKATMTLKPSDLAVIYSDGSRVVEPGRFEVSSGGLSMSFTVVE
>tr|Q8Y3Q3|Q8Y3Q3_LISMO
MAVYLDKEKPVSERVEDLLSKMTLAEKCGQLNQRMYGWEAFSRDGETFQITEKFKEEVTRFEGIGALYGLFRADPWSKMNKDTGVSRKNAGKVANKIQRYVIENTRLGI
PVLLAEEVPHGHQALDSESYPVNLARAASFNPELQKQVASAITEEISEKGVHLALASALDVLRDPRWGRAEECYGEDPYLAAELTAAITEGFQASGKIAVILKHFAAQG
EPIGGHNSGPVSIGVRELREIFLDPMRAGIRSGALGVMAAYNEIDGVPCHANKELLTTILREEMGFSGIVMADGCALDRLLKLNPDPKKAAKMALEAGVDLSLWDEVFP
FLEESVEKGILDEKIVDDAVSRVLQVKFQLGLFEQPFVEEDIQEPKSDWRQLNLQAAREGICLLKNDFETLPLVGEPKKIAVVGPSIDSLYNQLGDYTAPQNESECVTV
LEGIKNQLPENWEVLSEKGAEIREELPDGIQRAEVVAKEADAIVMVLGGSSARNFDMEFLNNGAVSSKGPNMDAGENVDVADITLPQPQLDLFYAMKRTGKPVIVVMIQ
GRPIAIPEISLAADAILTAWYPGSLGGTAIAEVLFGHYNPSGKLPVSIPRSSGQIPIYYNQKAVEYKEDYFDLTGKPLYPFGYGLSYSTFEYQDLQIKQESIKISELLG
GQSVEVKVTLKNISNVAGEEVVQLYIHDMEASITRRKKELKAFKKVRIAANQTTTVTFKLDKSSFEIWSINNKYEVEPGGIEIFVGGSSDTKLTGRVSIIGG
>tr|A0A0J1G1G5|A0A0J1G1G5_9FIRM
MTNQYLLKYNHSGSVLLLFTELRDNQKNRKEDSMMRAEKVLECRRRAKELVGKMTLEEKVSQTVHSAAGIERLGIKAYNWWNEALHGVARAGVATVFPQAVGMAASFDE
ELVWEVADAISTEGRAKYNMQQEFGDLDIYKGLTFWAPNVNIFRDPRWGRGHETFGEDPYLTSRLGVRFIEGLQGDDSQYMKTAACAKHFAVHSGPEDERHSFDAKVNA
RDLFETYLPAFEACVKEGKVEAVMGAYNRTNGEPCCGSRTLLTEILRKGWGFEGHVTSDCWAVKDFHEYHKVTSSALESAAMAMENGCDLNCGSLYLYLTEAVKRGMVT
EERLDEAVTNLLSARMKLGLFDEAATVYSGITYEENDTAVHGRLNFEASRKSFVLLKNEGNLLPLDKNKINTIGIIGPNANNRKALVGNYEGTASRYYTISEGIQDYVG
DDIRVLYSEGCHLCKTRLSGLGQENDRLAEVRAVCAHSDVVVACLGLDSSLEGEEGDEGNEYASGDKPDLALPGLQQDILRTAWESGKPVVLVLLSGSALAVSWADEHI
PAIIEGWYPGAMGGKAMASVIFGDYSPEGKLPVTFYRTTEELPDFRDYSMEGRTYRYMKQKALYPFGYGLSYTDFAVTKAAVSQDTVQKGTMVTVTAVLTNQGKMGGAE
TLQVYIKAKRPGTPNAQLKALKKVELKPGESKEIRMELADRVFGIRDDKGDLILGEGSYEIYVGTQQPDARSSELTGKHPVCLEIKADKTELLEKACAAVC
>tr|F0Z224|F0Z224_9CLOT
MEVKVVIKKYEDEIQALLEQMTLSEKTGQLRQCGPSIAGAFAMDFEELVNMVFDGRISKEKFQEMTGSANIDFHEEDLRAGKIGSYNGINDAKTANYLQKIAVEETRLG
IPLLFGYDVVHGYRTIFPIPLAESCAWEPALWEKTARAAAKEATAGGNHITFAPVVDVAKDARWGRISEGAGEDAYLTSAFGQAKVKGFQGESLSDEDSMAACIKHFAA
YGAAEAGRDYNRVDMSTQRLFEEYLPPFRACIEAGARVVMPAFNDINGVPCTASQWLMKELLRKQWGFDGMTISDANAIAECVEHGIASDRMDAAWQAICAGVDMDMAS
DCYSQCLEDLIESGKLDSAILDEAVANILRIKFELGLFEQPCQTTDEREAQEMLNPQSRALARECAAKSMVLLKNDGILPLKPDVKLGIVGALADNRSEMMGAWAIRGD
GNDCISIVDACRAQNKTFIYSTGIKKDPPDWKESGTAEVFCDCVDETEVLRIADECDVILAVVGEYKNQSGEAASRADISLSDTHMKLLHVLKGTGKPVVAVLFNGRPL
AIPWLKENLSGILEAWHPGVEAGNAVLDILYGCVNPSGKLTTTFPYTSGQCPVYYSHINTGRPGGKAKFTSKYLDTPVEPVYPFGYGLSYTSYTYTKLMVEQKPQGICA
SVMVKNNGSRDGDEIVQCYFRDLTAKRVRPVKQLAAFKKVSIKAGGEKNVVFEIPYQSMGYYDWRMKFVIESGKYEIFIGGNSEECLTKEVELKDDFTAFL
>tr|A0A416ET44|A0A416ET44_9FIRM
MEIYKDSMQPIQSRVEDLLKRMTLREKVGQLNQKIYGWEGYKITDSGFEITDIFREEVAYGDGVGAIYAPFRADGWNIKEFPGVSAKDSAKVINMLQKYLLEHTRLGIP
ALISEECPHGHEALDGTSIPVNVGIGSTFNPQLYQEAAKCIAAQIRARGCTLGLVSCLDILQEPRWGRSEESFGEDPYLAARMCENVVRGFQGEDPDELKSSNKIIAVL
KHLCGFGNPIGAHSGICSNIGEREMREIHLQGMKAGVRAGALGCMAAYNDIDGVPCHMNEKLLKDIIRGEYGFQGIVMSDGCAIDMSEAQYGGYENAGAAALNAGIDLN
LWNQSFLALQDAVEHGKVKEDDIDTAVRHVLYVKFLLGLFENPYTPEDLAEKVFGDDRMKGINLQLAEESVVLLENKEQLLPLGKDTGTIAVIGPNAHDIYNLLGDFTS
WKKEEDVYTILDGIREVFSDSRIIYEKGCGIRSGTREELEAAVKAAEMSDAVILALGGSSARNPEVAYGKNGAAEIVHYNTEVDTGEGRDIAELNIGQVQRELAEAIFK
VGKPVIVLNISGRPFSLDWAKPANALLQVWYPGEMGGLAVARILAGEVCPSGRLSVSIPKSSGQLPVYYNHKGHRNFTDMSPEPSYSFGYGLSYSTFSYTNLKADHISR
KELERGGKISVTIDVTNTGTVPAKEAVLLYVKDIQSCVTRRVKELKGFEKIYLLPGETKTVNLYLGMEELGVWDLHMNFVIEQGEVLLMAGTEETTVKIK
>tr|A0A0R2ESD2|A0A0R2ESD2_9LACO
MPQWLKYGGMNLEEAKLRQLLKSLTIEEKIGQLVQLSGEFYNASDISMGPQRKLGITQRMVDLSGSVLNVAGAKQIHRLQEAYLAKGGHAIPLLFMSDIVYGYKTVYPI
PLGMGASWDPRLIKRAYENAAEEAYAGGQQVAFAPMVDLVHDARWGRVLESTGEDPYLNAQFAKAMVGGFQSGLKDGKGIVSCVKHFAGYGAVEAGREYNSADMSMSNL
YQNYLPSYKAAIQAGAKMVMTSLTTLNGVPATADKWLLNDLLRKQWGFTGVVISDYASIYELTQHGFAANEQDAAYKALDAGVDIDMKSPCYANGLKPLLEDGRLSEEK
IDAAVWRVLSLKNELGLFEDPFRGSSEALEKANLLTPAKRQLARKVSQEAMVLLKNERHLLPLNRSGHRRVALIGPYADESKMLGLWAIHGYPKDTITFKHGLSEYLDT
SRLTVEKGTDIIRDADFFAESGRPEDQASGQISDSATEKKHHDSAVAAAKRADIIIFAGGEHTLQSGEAGSRTKLRLPDNQQRLLDELATLGKPIISVIISGRPLVLTD
VLADSDAVIQAWFPGIEGGHALADIIFGAYNPSGRLSMSMPAVEGQAPIYYNHLSTGRPEKTSQHVGRFVSRYTDAPTGPLFPFGYGLSYGKVTYHQPTIDQSQISSAQ
SVTIRVPVENTSEIDRTETVQLYLHDEVASIVQPVKRLIDFKRALVPAGETLMVSFVVKADRLGFYNQQGKYVIEPGKFELMIGPNSDETQTVEFSLKD
>tr|A0A5N6EL41|A0A5N6EL41_9EURO
MRGFYLPLLAAIGSSGQTYAALAPAATSLSYVISPPVADGGKAWRDAHAQAAELVAEMTLEEKVSVVTGQTGPCAGNSGNVTRLGIPKMCFQDGPGGVRPSLGNTQFPS
SVTTAATWDVDLIYARSYAMGKEFYDMGVHVAMAMVTGGPLGRSPYGGRNWEGWYADPYGTGIASWYGVKGMMDSGVQTCSKHFGFYEQETYRNPFNFTEPYSIYNASE
QLPVSSNVDDKTSHEIYLWSFAEAVRAGTTHIMCAYNCINGTHACANSETNNGLLKGELNFQGAVISDWGGVWGTQGFAMGGLDVNMPGRGYGNKFGPFFDQELYHLVS
NGTIAEARLNDMVARVVTPAIVTGQLDKPLPSIAINSVGSQYWPVQATYRNVQKESTVELIKTISTDGTVLLKNTGGLPLKSPQNIAVIGQDAGPNILGLQGCGELFRN
CDVHNNNGTLSLGGGSGYAWPLNLITPLDAIQAAGLEARSLVQFVLNNTAASVIAETVTGSALPIGPPDVCLVFADRYMRENMDRNDLSLNIGNWTHSEDIIVQTAATC
NNTVVVLHVGGPVIMEAWIDNPNVTAVVAPLFPGEQTGPGLVDILWGRVSPSAKLPFTIAKQESDYPPNTISYDPSVTPQANFTEKLNIDYRWFDTYNITPRFEFGFGL
TYSTFEYNNIELNDTMRPDEHAIQETKEKFAGQKSDESLYDILATVMAAVTNTGDWTASEVAQLYVEFPAAEDEPPRLLRGFAKLKNMHPGSTVKASFS
>tr|A0A086MCX5|A0A086MCX5_9HYPH

MIVRLSDVQGLAKLALAVVLTASLVPQARAQETAEQSIERRVNELLGQMTLEEKVGQLNLFSHGPPLRWEDISEGKAGALLNFNSAQDVARAQALARQSRLKIPPLFGL
DVLHGFRTQFPLPLGEAAAFSPRISRLASEWGAREAAYVGVNWTFAPMADLSRDSRWGRIVEGFGEDPYLGSVLTAARVEGFRQGGLAASTKHFAGYGAPQGGRDYDTT
YIPRAEMYDTYLPPFRAAVEAGSASFMAAFNALNGEPSTANPWLLTDVLRRQWGFDGFVTSDWVGIGELINHGIAADGAEAARKAILAGVDMDMMGLLYIKHLPDEVRA
GRVPESVIDESVRRVLRTKFRMGLFDRPDIDPSRSDSIFPSAESRQMAREVARETFVLLQNRGGVLPVPASARSIAVIGPLADAPHDQMGPHAARGHKEDSVTILEGIR
RRAQGTGITVRHAPACDLFCRDTSGLQAALDAAKQSDVVIAVFGEPQELSGEAASRVRLDLNGKQIEVLEELAKTGKPVALVLLGGRPQVLGPVAERIPAILMAWYPGT
EAGPAVADVLFGDVSPSGKLPLTWPRATGQLPLYYNRLPTGRPTLENNRFTLQYIDEAIAPLYPFGWGLSYTQFSYSDAAMAQKQLDQGGVLEVTVNVKNAGAREGQEV
VQLYTRDPVASRSRPLRELKAFEKIALKPGETKRVTLRVPVESLGFHLDDGTYLVEAGAIQVFVGGSSLAEPIGEAEIMKTFRIPPMERRASASTSVAQ
>tr|A0A430B320|A0A430B320_9ENTE
MTIKALLSNMTLKEKVGQLNQRLYGWHVYQKKNGEISLTDAFKEEVARWGSIGLIYGVFRADPWSGKNQTTGLTKEEALTVSRMIQNYIKEHTRLGIPVFLSEECPHGH
QGLDSTTTPANISSGASWNPELYQQVQELVAREIREKGAHLGLISTLDIARDPRWGRTEECFSEDPYLAAQFTLAALAGLQGGDTRAVDSNHVLAVLKHFAAQGSGMGG
HNSGPVNIGERELREIHLPPMRAAVAAGAELCMAAYNDIDGIPCHGNARLLSSILREEFGFKGAVMADGCALDRLVELSGSPAATAAWALESGVDISLWDNVFPYLEEA
VLQGLLSEETLDRAVLRVLRLKEKMGLFEDAAPAVEPVSDMQKRTLVTKLAEESVVLIKNKHRQLPLADTAVESIAVIGPNSHLIYNQLGDYTPFKHVGRCVTVLQGLQ
ARLAGTPVQIYHAPGTAVTTHLENGLEEALAAASKAEHLILVLGGSSARDFDTDFDANGAAISGSSEMTSGENIDLADIRLPNVQVELVKKLAQLGKKMTGILIQGRPH
SLCEIEPYLDSLLIAGYPGEFGGDAIASIVFGDANPSGKLAMSIPRSSGQLPVYYNYRDIAFKKDYFDLAGEAAYPFGFGLSYTDFEMTNVSVKDPLPTRAQLQAGQKI
VIQGEIANIGGRKGAEVIQLYVKSNQRNVITRVKELKGFVKVPLNAGETTCFKLTLGASELREYDANMTETIMSEFTAIVETTNNRCELPLVIDGENSR
>tr|A0A1H4CLN6|A0A1H4CLN6_9RHOB
MTADPEGLLAAMTVEEKIGQLNLLPAGEGMATGAGDATPLAERLDAGLVGAVFGVKSRESARAMQERALAGSRHGAPLYFAEDVIHGHRTVFPLPIALACSWDMALIEE
TAAHAAREAAEDGVHQVYAPMVDVSRDPRWGRVAESPGEDPTLAALYAAACVRGLQGADVAAEGRVAACLKHFIAYGAPQSGRDYDNASLGWAELFEVHAPPFRAGVEA
GAASAMVAFNAVNRTPMHAHAPLVEGWLRGRLGFEGLVVSDYTGVRELQAHGLGDRAATVARALHAGVDMDMVGEDYLAALPALARDGIDCAESGVRFTAAEIVALIDR
ACLRVLRFKQRLGLLADPFRSLRGEGPPRAPSATGRALARRAAGASAVLLKNDGVLPLRSGPLTAALVGPLADDRANLIGTWSVAGDAAQVRTLREGLEGREGLTLICA
PGANLVEEPWLVDRLNCHGPTVRPDPRPEAEMIAEAVAAARLADIVIAAVGEAKEHAGEASSRLSPDLPAPQRRLIAALAKTGRPLVVAVCAGRPLALAEVAEAADALV
MAWHGGVEAGPGLADILTGAAEPSARLAAALPAHPGQTPLSHAAEPTGRPNPGRFRKFLTGWLDLPDGPGQTDGLYPFGFGLAYTTFRHGAPQADRREGLGEEDRLAVR
IDVTNAGDRPGVETVQLYLSDPVARSTRPARALKAFRRLSLAPGETGRATFEIDVEMLRYPLADGPEDERRVWDPGAFTLWLGPNSRDAQALEIAWRA
>tr|E3EFF8|E3EFF8_PAEPS
MDNKTLLEKLNSLSIEEKIGQLIQLTGDFFEGDMDTVVTGPLKKLGLNTDYNIYNTGFILNITNPEKIIRLQTDYLEKSVHKIPLLFMADIIYGYRTIFPIPIAQACSW
NYEEIENAASIAAQECYDEGIHVTFSPMVDMVRDPRWGRVMESPGEDTLLAKKYAQSVVLGIQGSNLESTAVPANKIAACVKHFAAYGAPVAGREYNAVDLSEHALREY
YLPGYQAAIEAGAKLVMTAFNTLNGIPATGNEWLNRDVLRQEMNFDGVLISDYAAIEELIMHGYAEDEASAARLALLAGVDVDMKTAVYANQLKDVISDNSYMLELLNE
AVYRVLKLKNDLGLFEDPYRGLQEIVKRSTSSILSDEHKQAALSLAEESVVLLKNENNVLPLSKETKVALMGPYAEENSTLGMWAIKGEQTDTINLKTGLLQLVGSDNL
SVCRGSYLLPPDARETFDKYADKLAVETGSEEVLLQEAIRNANEAEVIVLALGESIYQSGEGGSRTNPTLPEPQLRLLHELSLLGKRIVLIVYSGRPLILTDVAGKVDA
IVQAWYPGTMGGEALANILYGEVNPSGKLAMTFPRSVGQIPVYYNELNTGRPNLKENGSYRFASRYIDEVNEPLYPFGYGLSYTSFEYSLLTVSQKEMKTNESVDIRIS
VSNTGTYAGKETIQLYIRDKFASVARPVKELKDFKKVFLQSGETTIVEFSVDEDMLKFYGREMEYKSEPGEFEVYIGSSSQDILLSFSFTLLPEDKHE
>tr|V4A1Z7|V4A1Z7_LOTGI
MALQMAYGGDTFSPTPAISRLGIQPYQWGTECLHGMDYPFRNVSLSWNGRVDDLVGRLTLEEMALQMAYGGDTFSPTPAISRLGIQPYQWKTNSLHGIMGINATSFPQS
VGLAATWSTDAVFQMAEATSEELRAYYNDGIKERRYSRGLSCFAPVINIFRDPRWGRNQETYGEDPFLSGMLAQAYVKGLQGDHPRYVRASAGCKHFDAYAGPENIPIH
REYFSANVTTRDWRTTFLPAFRYCVEAGTYSLMCSYNSINGVPSCANKELLTNVLRGEWGFTGYVVSDGRAIELIESAHHFFNNSIDTVAGAVNAGVSLELCGVRKTVN
YFTQIPDAVKAGKLTEDLVRERVKPLFYTRMRLGEFDPPEMNPYASITTDVIENAAHRDLASELAMKSFVLLKNDGTLPLRKSVYDKIALIGPMADNPKLLSGDYAAQP
DLKFVVYTPKFALGPLANTLQYASGCKDTACKDYDSHSVQKAVQDVDIVILCIGLGYVLETEGHDRENMELPGKQNQLIQDSIKYSGSAKVILISFNAGPVNITWADMNP
RVSAIIAAFYPGQATGVALYNVLKSSNHSQFGRLPYTWYHTCDQVPPMIDYTMQGRTYKYIKQEPLYPFGYGLTYSSFEYLELYLDQYVNAGDSILGVITVQNNGQQPA
DEIIQVYVSWNSPSVETPQIQLVNFTRVRIESYHVTSFRFFISPQNMAVYTDDKGWVVEEGTMTVYAGGELPGQAKSGDTGHMKGEFTIKGTKILGFY
>tr|A0A1H8FTK1|A0A1H8FTK1_9BURK
MRSHLPAAALLAAVMLPVAAVAAEATARLPALSAEDSRRVGALMARMSVDEKVGQLNQVAGADATGPVSPAKAEDLAAAVRSGRIGSMLNIRGAAATRQYQQWAMRSRL
KIPLLFALDVIHGYQTVFPVPLGETASWDLGLIEQSARIAAIEASASGIHWTFAPMVDVARDPRWGRVMEGAGEDPWLGAAVARARVLGFQKRRLGDTDAVMATAKHFA
GYGAALAGRDYNTVDMSLQQLHDVYLPPFKAAADAGVASFMNAFNTLNGVPATGSRYLQTTLLRRQWGYDGIVVSDWGSVGEMITHGFARDAAHAAPLALNAGNDIDME
SHVYDKALAAAVRSGAVATPTLDAAVRRVLEMKARLGLFKDPYRFSDERRERQALSNPAHRLVALEAARKSAVLLKNHERLLPIPPRVRHIAVIGPLARARRDLEGGWL
VNSSPQHVVTIAEAIKARAPAGTQVTVLDGCDVDCASDAGFAAAEAAALQADLVVLAVGEGWDMTGEDRSRADIGLPGRQNELVQRVARAGKPVAAVVLGGRPLILNTV
AEHAGAILLAWLPGSEGGNAIGDLLFGHVNPSAKLPITFPRSVGQIPISYAHYMTGRPAGAVRAPYRSQYIDSPNTPLYAFGHGLSYTTFEYTAPALSAAAIKADGKAT
LRFTLTNSGAVYGEEVVQLYVHDEVASLARPVRLLRGFRKVGLQPGASAQVEFTIDRATLSFIDASLASVVEPGSFRLMVGAASDDLRLEARLEVLP
>tr|A0A2Y9BML5|A0A2Y9BML5_9FIRM
MEDKMESKLQELTIEEKVGLLSGADFWTTKEVKRVNLPSFMMTDGPNGLRKQVTSSDHLGMNESLPATCFPMACALACSWDRELLYEIGKALSEECIAADVDILLGPGV
NLKRSPLGGRNFEYFSEDPYLTSQLAAKYIKGVQENGTGTSLKHFAANNQETRRMVSDSLADERTLRELYLACFESVVKEAEPWTVMCSYNKLNGTYTSEHDWLLNQVL
KKEWQYDGAVVSDWGAVNEKVKSVQAGLDLEMPGNQGESDRILLEAVKNGEISEDKIDEAVTRILRLSDYILEARAQKNVPVSAEINWENHHKLARESAGESMVLLKNE
NKVLPLASGEKIAVLGELASDIRYQGTGSCKVHPYHVDSPFEEIRVYEPSAVFAKGYLLDGGDDGLLLEEAEAVVNGCDKVIIFAGYPERAENEGEDKVDMKLPYNQTE
LIHRIARTHPAVIVVLCNGSPVEMPFSNEAEAILETYLAGEAAGGAAADILFGKKNPSGKLSESFPVKLEDNPSYLNFPGMQNKTRYGEGIFVGYRYYDKKNIEPLYPF
GHGLSYTEFTYSNLSVSAGKENTADVTLEVQNTGDRYGAEVVQIYVQKKGTEIIRAVQELKGFEKVRLEAGEKKQITIHLPKEAFRYYDAENGSWQVEPGEYEILAGAS
SRDIRLKGSIVLEGDELPPYKCDQNSTIAEISRSPEGKAVLEKWMKWCSDKGYPIFTEEPEFIQNAYETTPLRWVKMLSGGCFTEEMIEDVLSHCNR
>tr|A0A5R8Q824|A0A5R8Q824_9FIRM
MELYKQKEAPISARVEDLLSRMTLDEKVGQINQRLYGWKSYQKVDDDYELTDYFKDEVARFGGIGCLYGVFRADPWSRVNHETGIPHADSLKVARMLQAYVREHTRLGI
PLLLSEEVPHGHQALDSVLYPTNIGMGSTWNPELQMQVSAAVAAELHYKGVQLGLVSALDVLREPRWGRSEECFSEDPYLASIFTTAVVAGLQRKIDSTKTVAVLKHFC
AQGEPMGGHNTAAAVIGERELREIFLLPMVAGIGAGAKATMAAYNEIDGMPCHANKWLLTDVLRDELGFDGFVMADGVALDRLRNDDVDGKQAAAFGLEAGVDLSLWDD
AYTHAGESVTSGLLDEAVLDKAVTRILTLKFELGLFDEELEVVEFDKTGAEAVSLQAAREAIVLLQNNEQLPLSKDIKHIAVIGPNANHLYNQLGDYTSPQRDDYGVTM
FQGIAAAVDGQVSYQPGCGVKSYIDGGIAAAAELAAKADQVVLVLGGSSTRNFNMQFDSNGAVIGSSRGADMNCGENMDVADLCLPQPQLDLLDAVVAVSKQPVVSVII
QGRPHALSEVSEVSGSVVCAWYPGPFGGQAVAEVLFGDVNPSGKLPASLPRSSAQLPVYYNYKDNGALVDYSDMSGSPLFVFGHGLSYAQFVYKDMRFVDGRVEVTVEN
VSAIAGFETVQLYVKANSRIVTRRVKELKDFRKLWFEAGEERVVRFEVDSDAYFGYWNERMQFEVADGRLQVLAGGSSATALAMKLEISDGKVMRID
>tr|A0A0R1NB30|A0A0R1NB30_9LACO
MLPYQNKTLTINERTSDLLGRMTLKEKVGQVNQHLYGWKCYKKDQDGVKLTDYFKQHVKWGGGIGALYGVLRADPWSGVNFDNGVTGQEAHDLIQLMRDYVVNHSRLGI
PPLFSEECPHGHQGLNGVSVYPTNIGKGNSFDTALMTEMAGLQAKELRNLGINLALVSTLDLAKDPRWGRTEECFGEDPMLSAQFSQAIVNGFQGHLIQENVDFGAAPVP
STTEQDGHIGVVLKHFIAQGEVLGGHNSGSVSMGAREFKEVYTPLFNAVKNAAGVMVAYNDIDGVPCHANASLLTSLRHRYQFQGLLMSDGTALDRLAQLYDSPKQAII
AALNAGVDLSLWDNVYTQIADNFDTNMSAALDRAVSHVLRIKFLLGLFDNPFPSFHPTQDETQKLNHQISTESITLLKNDPAVLPLNRQKKVLVVGPNANAFYNLLGDY
TAPQTVEFMTKTPLASIRAAFDTADYALGCEIRDRSNSAGLIQEAVTKAANADVIIAFLGGSSTREFNLKFLDNGAVSSKGINMDSGENVDLASLALGGAQNQLIQALE
KTGKPIVTVLIEGRPHSLKYVLPASAAVITAWYPGQQGGNAIVDVLTGKVDPSGRLSMTYPKNSGQLPVYYYQRDAPKNEDYYDLSGSPEFPFGYGLHYGKINYSNLTV
SLSANTILGSVQVSNRSNHVIHESTLIFVKLFGGTVIQRRDMLKAFVRNTLPANSTQEVVFSLPLGQVKYIDLNGDWTLPDALELRVDNLTKTVQL
>tr|A0A2R4X227|A0A2R4X227_9EURY
MHAADAAPAIDALLDRMTLAEQCAQLVGVRPCDLADSEGRFDPERAAEAIPHGVGHVARIGGWAGREPRDAAAFANSVQRFCREETRHGIPAIPHEECLCGYMGPGGTV
VPQAIGMAATFDPDLLRATTDAIGAELAGIGCRQALSPVLDVGRDPRFGRIEETFGESPALIEVMATAFVDGIQDGDGPALGATVKHFIGHGTPSGGRSRASVSVGPRD
LRAVHARPFETVIAAADPDGAMNAYNDVDGRPCGGAPELLEGLLRDDLGFDGTVVSDYFSVPQLASVHGVASDDREAARQALTAGIDVELPEADAYRALEASVESGDLD
RSVVRRSCRRVLEQKAALGLFADPTIDAERASDRVGAERTRQLAQRAARESVTIVESGPLPIDDPDRIAVVGPQADATVGLLGDYAYPVHMEDPDACAVVSPVEGLRRT
VDARIDHAPGCPLLDDDRSGIEEARSVAADADLAIAVVGARSGLFAAETEDGEYTGLASAGEGISRSGLGLPGVQPELLDALAAVDTPTVAVVVAGRPLRVDAAVDGLV
YAWLPGERGGGLGLAEALAGTDPGGRLPVSIPRSAGHVPAHHARRPQSGPAGYRFGPGDPAHAFGAGESYATIEYGPLEAPDASETTAPLELATTVTNVSDRPGVAVPQV
YATDLPGDRTSPERELCGFARVDLDPGASKRVSWSIPAVALADYADVERVLHPGPATIALGRSSADLVDRQSFDLTGDPARLPPGRDHRATVTIE
>tr|A0A5D0CN98|A0A5D0CN98_9BACL
MDNQQLLTMVTQMTLEEKVAQLMQLAAPFFEGAEGEGQITGPMKSMGVTEEMVRNSGSVLGYAGAEGTIAVQKAHLKKNRLGIPLLFMADIVHGFKTIFPIPLAIGCSW
DLPLAERSAEIAAREAAVSGVHVTYAPMVDLVRDARWGRVMESTGEDPYLNSELARAFVRGFQGQNLREDVQRVAACVKHFAAYGLSEGGRDYNTVDLSEWQLREYYLP
AYRAALDEGCEMVMTSFNTVDGVPASGNTRLMRKLLREEWGFDGVLISDWGAVKELIPHGVAEDEAEAAYKAIQAGVDIEMMTSCYVHHLPRLVREGRVEEEIIDEAVL
RILQLKERLGLFERPHRGADPEAEKEIVFCAEHRAAARELAEKSCVLLKNERGVLPLARGQRVALVGPFAQSGDILGPWSWTGSRKDAVRLDTAMKAAAAEPGDISVAE

GSGIEAMTEAQWAEAARAAADVDVIVLALGESSEMSGEAGSRADITLPAAQLELLRRMKALGKPVAVVLFNGRPLDLRGVYDTADAVLEAWFPGSEGGAAIAALLYGDA
NPSGRLSMSFPAAVGQIPVYYNHFSTGRPLGAPDAQERYVSHYLDLPNEPLLPFGFGLSYTTFIYGKPQLSGKIMTPDRPLELSVVLTNAGPAEGEETVQLYIRDWVGD
TVRPVKELKAFAKVRLAAGESSEVRFTVTEQMLRYTHQNLEFASDAGQFSVFVGPNSRDAEELRFRLEKGPVTSGIRLQGASLGNNQHEQMGSEMR
>tr|A0A267EI12|A0A267EI12_9PLAT
MLRLLLLLLSLAAACQADEDFPFRNVSLPWPDRADDLVGRLTAQELVQQLAKGGVGPFGGPAPAIARLGIGPYQWNTECLRGAVKSGQATSFPQAVGLAATFDPDILSQ
VGRATGLEVRAKHNDFVAKGDYGDHTGLTCFAPVINILRHPLWGRNQETYGEDPHLSGVLGAAYVGGLQGNATNRYLLTGAGCKHFAAYDGPDSYPESRFGFNALVSQQ
DLEQTFLPQFRRCVEAGSVSVMCSYNAINGVPACANAQLLRGVLRERWNFTGYVVSDAGALEFAILFHKYFADRLSSAIGSLRAGVNLDLSACPYESCVVFEQLSVALN
TSRISEAELRAAAWPLFLARLRLGEFDPPALNPYSSLRPDQFIQSAAHRALSLTAAQTSLVLLKNLGGFLPDGLIKRPGLRLGVVGPLSDDMSLVYGDYSPNRMPQFEQ
PLLPALKERFGADKVSHATYCTDGGVYCTRQDPAAVSSLIAQSDVLLAVVGTSVDIETEALDRRQLTLPDGQLALLRLLTASGRPVVLAVVSGAPVGLDEAIGNDSVRA
VVWCGYPAQTFGPALINLLTGRYSPSAALPFTWPSERAQLPSITDYDLAARNQTYRFSEPWSGSPPPLFRFGQGLSYCRFSLTHLTAPASISAGQDLHLSLRVAANAST
SGISECPIVLKAYLTPPTGVGRNPTIWLASFRKFRLQSGSSASTVSLSVLARDMQLWDGSAGLMRQLPGNYGLLVGDYFDESCADCRVRTSFKVVS
>tr|M1MJF4|M1MJF4_9CLOT
MINRESKQKEFIKQYYEKRIDDLIGKMTLEEKVGQLVQISPSIFGAFGLTFDETIEKLINGEITPAEFEALERNYREDEIRQGTLGCMGGVHGAEKSNELQKIAVEESR
LGIPILFGLDVIHGYRTIFPIPLAEACSFDIEKIKESARIAAKEASAAGLHWTFAPMVDISRDPRWGRVAEGAGEDPYLGSVIAKARVEGFQGESLDNPESILACAKHF
AGYGAPDGGRDYNTVDMSLQTLHDVYLPPFKAAAEAGVGTFMSAFNDLNGIPCTVNKYLLTDVLREKFGFNGFVVSDANSIPEVVVHGYAEDNKAASKKALNAGLDMDM
SQGTYRNELPELVKEGDILEEVLDEAVRRVLRVKFLLGLFDNPYRTDAKKEEKTLLCKEHLEAARDISRRSIVLLKNENNALPLKKDLKKIAVVGPLAENAAEMLGTWS
HTGNPSDVVTIISGIKAAVSTETEILYAEGCKITGEECIDFEGAVRVAKESDVIIAVVGENSDMSGEAASRIDINLPGKQEELLKELRKIGKPLIVVLINGRPLTIPWE
AENVDALVEAWQLGTQSGNAIADVLFGDYNPSGKLVATFPYSVGQVPIYYNNPMTGRPAGKIKFTSKYIDGPAEPLYPFGFGLSYTTFKYENLSILSAENKIGDTVAVK
VYVTNTGEVSGEEVVQLYVSDVVASRVRPVKELKSFEKVLLQPKECKTIIFKLNTKDLGFHDENMNYVVEPGLFKVYVGTNSKECLEGEFTIVALD
>tr|Q0AT45|Q0AT45_MARMM
MNNEKAVMAKVQEINASTSKDRVEVRVRDLLDRMSLEEKIGQLNQVEASADNVLDLLGDDIRAGQVGSIINQVDRDTVLELQRIAREESRLGIPLLVGRDVIHGFKTVV
PLPIGQAASWNPQLVEACARLASEEASTVGVNWTFAPMIDVCRDPRWGRIAECLGEDPVLTSVLGAAMVRGFQGASLDDPSSLAACAKHFAGYGASESGRDYNTTNLPE
NELRNVHFPPFRAAVEAGVASLMTSFSDIDGVPATANSFLLRDVLREEWRYDGLVVSDWDAIQQLCVHGLTETRDEAAFQAASAGVDMDMVAGAYLQHLAGLVASGRIE
LETVDRMVANVLRLKFRLGLFDSRPVLADEPARMTSRSLAKEAALQSCVLLKNEGRALPLDPACLDHLAVIGPLANEPAEQLGTWVFDGDPERSVTPLAAIESLAADAG
MSVSHARAMPTTRSLDETAFAEAEAIARNADVVVVFLGEEAILSGEAHCRADIDLPGAQVSLVKRLKAVGKPVIAVIQAGRPLTLTSVIDDLDAILFAWHPGSLGGAAI
ADLLFGRACPSGKLPVSFPKMVGQIPVYYGHKNTGRPPTPDSIVLIDDIASGAAQTSLGMTAFHLDAGYEPLYRFGFGLSYTEFAYSELSLSAVRITPSETLTVAVNVT
NSGEVEGDEIVQLYLRDRFGSVTRPVRELKAFQRVTLAPGETREVRFSLTVEDLKFYKRDQTRGAEAGKFDVWIGGDSAAGRKAEFTLTQDSPAMA
>tr|R7TQQ4|R7TQQ4_CAPTE
MRALRVTVVCLTLISTASFSQDNVLRFAPSSHALDSFPFRNVSLPIETRLNDLISRLTIEDAINQTVARYGKFTPGIERLGIKPIEYITECLRGVRRENATGFPQALGL
AASFSRDLMQRVATAVSVEVRAFYNHDIQRETYGAHGITCFSPVINILRHPLWGRNQETYGEDPYLSGELASQYVSGLQGDDPRYLRVSAGCKHFDAHGGPDTIPVRKF
GFDAKIEERDLQMTFLPAFKKCIAAKPYNVMCSFNSINGVPSCANKRLLTDVLRAQWGYEGFVVSDDAAVEYIFTEHHYNSSFETAAVEAIKSGCNMELVGKFDPSYWQ
LTKALNEHLITKDELMENVRPVFLTRFLLGEFDPPALNPFNQITKDVVLSAEHQRLALEAAVKSFVLLKNDRNFLPLLKNSLKTVAVVGPMSNYTDGLIGDYSTDTDPS
LILTPLHGIKKLAPNVQFASGCSNSTCTDYRATDVAAAVDGAQVVFVALGTGFIVEAENNDRSDIVLPGAQLQLLKDAVYHANGRPVVLLLFNGGPLDVTFAQLTSGIV
SIVECFFPAMMTGEAIYRMLINNEGISSPAGRLPLTWPAYLNQVPNITDYTMKGRTYRYYTEDPLYPFGYGLSYTQFKYSDLKVTPLEVTKGQEIRVKVKVTNIGLYDA
DEVRIIVVQAYVSWPKTEIPVPRWQLVAFDRIHIASGKSETVELTIEASLLEVWQNPETGFDILEGEMTLYIGGQQPNQRVNVGSNVISRNLSLQH
>tr|A0A3N4IQ33|A0A3N4IQ33_ASCIM
MLSLVTLALSANHEDLSSQFGPAQPQRTWEESYALAKTLIGKLNSIEKIRLTTGVGFGLGKCAGNIGKIDKVDFPGMCLQDGPVGVRKTQNATVYPAGVTTAATFDREL
MYMRSRAMGLEYRTKGINVALAPAIGALGRSPNAGRNWEGFGPDPYLCGIGGAESVRGLQDEGVIATAKHWVANEQERYRLVIENIIGFNSGPILYSISSNVPPRALRE
LYEWPFIDIIEAGAASVMCAYQRVNGTYSCESEEIIKGHLKSDKNGNLGFKGFVVTDWFAASLASKASTAGTDVVMPGDIGLMGAAAGTQAASDGALTGEKGATRLDEA
ALRVVAAWYKTGQDKGFPSLDLERDATTEFHAQIAQRINTDGTVLLKNTNKALPLTKKIKKIGVFGSDAGPIPKGPNSCGEFHTCRDGTLAVGWGSGGGTFEYLIDPLQ
AIEERAAAEGATVESVLNDFDYKKIREVAADKDVCLAFIQSRSGEGLAAVDGNVGDRNNLTAWHGGDALIATLTPICDNIVVVAHTVGPIVVEPWIEHPNVKAVLFPHL
PGQESGSSLVQTLWGDVNPSGKLPYTLGKKQDDYCCKLISDWAGFAPEQDFTEGTNIDYRWFAAKGITPRFEFGFGLSYTTFAYDASTLSISTPKVLPDVGLEDVLFSV
EIEIENTGDVEGKEAAQLYITYPSEAKDSGRWLRGFDKVKLAPGEKKKAIFEVRKRDVSYWKEGSGWAQVTGKDYEIQVGASERDVKAKGSVRFA
>tr|A0A161XRQ5|A0A161XRQ5_9EURY
MQASGGITLATALAGYLGESAVATKQREPSRHVTELVDEMTLEEKVGQMTQMAAGEISTDDPAETLRKYKPGSLMYLTSFDPEEVARTSNKLQKAMVNETRLGVPFVYG
IDSVRGDNNVAGATLFPHNHGVGATWDADKAEEMATVTSKVMRTTGTHWNFSPVCDIQRDPRWGRFYEGFSEDPFLASQMVASKVRGYEEETNGYKRTGASVKHFAGYS
APANGNDRTAALLPYRTFVSSILPSYAAGINAGAETVMVNSGSLNGLPAHASKELLTDILRDQLGFDGMVVTDWHDFYRMIKVHGFAEDLKEATKLGINAGIDMYMVPA
AGIEGDDAAGYQQRLIELVKEGSVSRDRIDDAVTNILAFKEHVGLFEDPYGTPSKVSDVISDGRDLAEEVATESMTLLTNDGTLPLDSGSSILVTGPSADSVRNQMGGW
ALGWQGVGDTEPPATTVLEGLTDAADSSASVTHVPTGLHEFTNEDDVRRAAECADVVVAVLGEGPYAEEKGDTDTLALPEAQQRLVEVVSGTDTPTVGVIMAGRPRGTD
VFDDLSASMMAYLPGTAAGPAVAATLFGDANPSGRLPFTWPKGTGQIPNLHNNYPPDEFNKSTNVNPPQSHETPLFEFGHGLSYTEFEYADLRVAPTKLQQKNATNDIT
VSVAVKNTGDEAGETVVPVYGGRDLGRVMYPQEVVVGFERVALDPGETKRVEIRSTILPLGTVLGDIFSRGELVIDTGEYWLSVGDLTETVSVV
>tr|A0A318XPS1|A0A318XPS1_9FIRM
MKSPCRYKMDFYIRIQGMAMLMEFIYQNPALSPQERTNNLLSLMTLEEKVGQMMQISYNTLSAEEAEDWASNKKAGSFLHVLGKKAEHLQELALSTRLGIPIIFGIDAI
HGHGLMNGSTIFPSQLGMSCSWNPELLEKAGRVTAREAAADGIHWTFSPVLCIGRDLRWGRINETFGEDSYLIGVLASAIIKGYQGKSLSDADSILACAKHYLGYGEST
GGKDAYDTEVTCRKIREVFLPPFKKAIEAGCATFMTGYQSIDGTPMAINKKLLYDILKQELGFKGFVVTDYNNTGSLVTLQMVSPDLDDASKKTVEAGNDMIMATNDFY
ESAIRLVKSGLLSESLIDEAAGRILSIKFSSGLFDVKKKSGYASRSVISCKEHLQVNMELTRQSIVLLKNDKNILPLNKSTNIKKLAVIGPNADNLKAQLGDWTFFTHP
DPNPEAVPAFEYYTMLRGISERSRLKGIETVYHKGCDILDDENQNIEDAVSAAAACDAIIAVIGDCVEQNGEFKDRANLDLSGAQQKLLKALKALGKPLIAVLVNGKPL
CVPWLQKNADAVVESFNSGILGGKAVAELLYGDFVPSGKLTVSFPYHSGQIPVYYNQLSGWHSPKYMDLAERNALYPFGFGLSYTKYQYSNLVLSSAVCGKEDTVAVQV
DITNTGEYAGTEIVQLYINDKVSSVMTPEKELKGFERVSLKAGETKTVTMSLQISALAIVTPDERYVVEPGEFEIMVGPDSKASSLLKKTLTVC
>tr|A0A0P6W8Z2|A0A0P6W8Z2_9HYPH
MRITIFFAAIASLLAPIATAGADPAPSERVRRLLAEMTLEEKIGQLDMPSHGGHYDPARVAAGRTGGVLNFGSPQEIAAIRALNAQSRLKIPLIFGLDVIHGYRTLFPM
PLAEAASFDPAVAEQSTHWAAVESRAAGLNLTFSPVADLARDPRWGRIVEGGGEDPMLAGLFAAARVRGFHSGGIGATLKHFAGYGAVEGGRDYAPADISEGTLRDRYL
PPYRMAVEAGVDVVMTSYISLNGTPTTASRRLMSDILRGEWGFPGYVVSDMNAIPEIALLGVADGNGEAAKLALEAGVDQDMDGEVYIAELAGLVRDGTLPEAALDRAV
ARVLATKERLGLFDEKPFDPAAASAVLGRADIRAASRALAAETFVLLQNKDDVLPIRPETRSIAVVGALAASQADTLGNNAARAVPAETVTVLDGLRARAPAGTRIDFV
EGCDTACTSDSNFQAAAAKAAEADLIVAVLGEPQDHSGEGASRASLELPGRQTDLLRRLAATGRPVVVVLLAGRPLALSEAVDPAAAILLVWYPGSEGGHAVAETLFGD
RDPAGRLPVTFPRHVGQVPITYDALPTARPGKVEDRYTSRYLDVALGPLFPFGHGLGYTRFTQSDARIAARKLGPDDRLTVAVTVANAGTRPGREVVQLYVRDVVASRA
RPVRELKAFGKLLLEPGEAREVVLSVPVRSLGFHRDDGSYVVEPGRFDVYLGSSAAAPLIGSVVVSPGYEAPPAGLSAERFEAGHMTVIGKSP
>tr|A0A512INI3|A0A512INI3_9HYPH
MTMRGKAARFGGRVLASVLLAGCSMAARSETVDDRVEALLAKMTLEEKAGQLNLVSLEPGFDPESVRRGEVGAVINFSNQNLASNIDGYARTSRLGIPLLISLDIVHGY
RTVFPLPIGMAASFDPDLVRRASAAAARETLAAGINWSFSPMADVARDLRWGRVVEGLGEDTWLTGQLAAAQVEGFRDGGVASTLKHFAGYSAVYGGRDYDVTWVSPTE
LYDTHLPPFRAGIRAGADSVMTALTALNAVPTTADGHLMTQILRREMGFKGVVIADWEAVASLIKHGVARDGAEATRKAVAAGVDMDMTSGLFVKHLPEEVRAGRISQA
SVDESVRRVLRLKFGLGLFDRPVLDPATAEGRLLTPETRAVARETARASFVLLKNDRNLLPIDPRTVKRIALVGPFADAKWDQVGPHEGNGQVDDVISIRTGLTERAAK
AGIALDYAAGCDRICSERADFPKAIEAAKAADLVVAVIGEGRDQSGEGSSRAYLGMRGLQQDLVQELAATGKPLLLLVFGGRPVELHEAIDRAQAAMMIWIPGTEGGPA
VAETLFGDFDPSGKMPLSWPRTVGQMPLTYDRRAGGRPHIDGARWTLGYNDETISPRFPFGYGLTYTSFAYGDPEVVTPKVGVGDRYEAVLEVRTSVTNTGTRPGRTVA
QLYLNQPVASRSRPLRLLKGAVPMVLAPGETAVATFRVPSRDLGFHDTDGTLVVEAGDYRVFVGEDSDAPKSAGFSVETGWRSAPGNVEASSR
>tr|E7G6B9|E7G6B9_9FIRM
MSISIDKIDELISMMTLDEKVGQLNQHLYGWQCFCKNKDGDYELTQLFKDHVQKFGGVGAIYGILRADAWSGMNEVNGVKKEESLQVIEMIQNYLKTETRLKIPALITE
ECVHGHQGLHSMMYPANISMGMTWNPDLLKEICQEVSCELASKGGNLALFTGLDVMRDPRWGRSEECFSEDSYLTSEMTKAAVKGFQLNDRNGVGVILKHLCAQGACEG
GHNSGAASIGQRELREVFLPPVKAGVLSGAKGVMAAYNEIDGIPCHVNQALLTQLLRGEYGFNGIVMADGCALDRLSIMNSDIPLMAATALKAGVDLSLWDHVYPLLGD
AVRQGYLDEKVLDRSVKRILKLKFELGLFEERQVTVYPSREKELALKAAQECQVLLKNDGILPLRKDIKSIAVIGPNAHNVMNMLGDYTSFQKAEDVTTLYQGIQQVLG
EEVQVNYALGCHIRDCSKADLAEAVELASQSDVVIMALGGSSARHFKMDFESNGAVTTSYDKNEMNCGENVDKASLDLEGLQVELLRKIKQVNQQIVTVLIQGRPHSIG
NIVNDSRAVLAAWYPGNLGGLAIAQTIFGDHNPSGRLSMSIPQSSMQLPCYYNGKYSGAKEEYIDMSGKPLYPFGYGLSYSQFQYHHIQLSQTEITIQDLEEKGIDISL
DVENVSQRDGYEIVQIYIIDNESTITRRYKELKAFQKIYIEAKSCCSLVIHLDSETFKIWDYQMNHLIESGTVDILIASHSEQFITRKLTILK
>tr|R7N7J7|R7N7J7_9FIRM

MQEQELLDLLKDMSLDEKINQMVQLNADFYDIEENGDSITGPMLEMGLTKDQVWQAGSVLGTVGAAKIKKFQKVYMEHQPHHIPLLFMADVINGYRTIFPMPLAQGCTF
DPELVRKMASVSARETARAGLHLTFSPMSDLARDARWGRVLESTGEDPYLNCCMTSAMVKGYQGEDVLKEDHVAACVKHFAAYGAPLGGRDYNQVELSERTLREDYLPA
YKAGVDAGAQMIMTSFNTLGRVPSTANKALMRGILRDEWGFDGVVISDWAAIWELLKHGIAEEEDEAAALAVKAGVDIDMMTEIYANHLKKLVEEGKVEEKLIDEAAYR
ILRLKNRLGLFENPYKNADEVYDEALEIEEEHRALARVAAAESFVLLKNDGILPLVKKADAAENSQHTGIALIGPYVKEKQICGSWSLFWKQSDLVTVEEGIRNKELDK
NMAYAKGCEILDYQQKICGFGVEFENTKSKEELEAMMQEAVETAKKAETVILFLGEHYQASGESASQTEIQLPAHQLELLDRVYEVNPQIVVVTFSGRPLDLRHVVEKA
KAVLHVWFPGTEGGNAIADVLFGDQEPGGRLAMCFPYTVGQVPVYYSELHTGRRRPEGEITPRGVSGYLDAPNHPLYAFGYGLTYTSFAYSEVSLSSEIMTREENLTAK
IMVQNTGNRAGTEVVQLYIRDVAGSVARPVRELKGFQRIAMQPGESREVTFEITEEMLRFYNIDMEYVAEPGRFEVYIGGSSDTEQKAEFRLV
>tr|A0A5C5W5X1|A0A5C5W5X1_9BACT
MDSRKFSDWHSRLCSLLGGAILMAAVALPPPARADEIDARIDALLAKMSLADKVGQLNQISGGSADADAAVREKLLSEIRAGRVGSVLGATGAEYTNMLQRVAVEESPH
KIPLLIANDVIHGYRTIFPIPLGEAASWNPALIKTCCQVAAREARAGGTHWTFAPMVDVCRDPRWGRIAETAGEDTYLGSVIAAARVRGFQQAADGPHGGVMACAKHFA
AYGGAEGGRDYNTVDISRRTLHEIYLPTFHAAVKAGAGSFMTSFNEISGIPATANNYILRDVLRRGWRFNGIVISDYNAIGELIPHGFAADGAAAAEKAIRAGVDIDMC
AFLYEKHAAALVESGRLPAALLDEAVRRVLAAKFGLGLFEQPYADAAHESRTLLDRSHRVAALAAAAGSLVLLRNEGGLLPIRSGVERIALIGPLAENRADLLGTWACI
GRAEDVVPLSDGLRKALPNAAVKIVRGSEFLKPITGGIDEAVAAAKASQVAILAVGESEDMSGEAHSRAEIDLPAPQLELVKAVHAAGVPTVVVVFTGRPLAIPWLAEN
IPAILVAWHPGIEGGNAVADTLIGTYNPGGKLPATFPRSVGQIPLYYNHKNSGRPPVAGERYTSKYIDIDWTPQFPFGFGLSYTRFEYSAIELSHQKLKSDGVLEVSVD
VRNAGDRRGDEVVQAYFHDCAASVTRPVRQLIAFQRIGLSPGETYTCRFRLSVADMGFYDADMKFVVEPGVMELYVGGSSAGGLKATFEIVE
>tr|E8RPE5|E8RPE5_ASTEC
MRLRWMVATALLLTGVSSVALAQTQAWFDKSKSPDERAKAAVAAMTLDEKLTLILSFTDPDQLAKQPDSIVSPEIKALVNKEHIRGSAGYVPAIPRLGIPAQWQTDASI
GVRVQGMARTALPSSLATAASFDPAVTEAGGRMIAQEARLSGFNTFLAGGANLAREPRNGRNFEYVGEDPWLAGQMASGLINGIQSQHMVSTLKHFAVNDQESQRTTVN
VTISPEAMRQSDLLAFEFILEQSNPGSAMCAYNLVNGRWACENEYLLNKTLKQDWGFKGYVMADWGAVHSTVDSANYGLDQFTGFPCCGDRQPYFAPINVKAAMEKGDI
SQKRLDDMAQRVLWPLFSTGVFDNPPKVGSIDFKANAVVSQKAAEESLVLLKNEGNLLPLSGVKSVAMIGAHADKGVISGGGSSGVTPVGGNPLEGQLEPKSWPGPVTY
MPSSPVEALKADLPKAKIVYAGGEDIAAAVALAKSSEVAIVFVTQWMGEGMDGTLELKGNQDALVAAVAAANPRTIVVVQSGGAIFMPWKDDVGAILQAFYPGIRGGEA
ISRLLTGKVNPSGHLPISFPASNDQLAHPTIPGFGKPDGVAVNITYHEGAAIGYKWYDVKGYTPLFAFGHGLSYTTFAVTDAKATVEKGELTISAAIRNTGKTAGKGVI
QVYVAPTNYKAVGWEAPKRLVAFRKSAVAPGTTSGTFTQKIDPRLLATYEVADNSWRIRAGTYQVLFGQAADDLPQSVMVTLPDLTWSAVHN
>tr|R4W6C6|R4W6C6_9EURY
MSESEARHPYEDPSLSTEARIDDLLDRMTLEEKAGQLVGMPLGNFERGRDVDEAKELVDEHHLGSVAPFGWGGAFYWTPDECAEVARELQEYAREETRLGIPLLFNADA
VHGHAYVKGATTFPNGLGMAATWDTELTKRAGEVTGRELRATGIHQNYGPTCDVGRDPRWGRAHETYGESPYLVKELIGAEVTGLQGEHGNVDDESVIATAKHFPAYGE
PRRGEDAAPVEISNSTLRRVFLPSFEAAVEADVWSVMPCYNSIDGEAVHGSRRFLTELLREELGFDGHVVSDWGGVDHLQNDHKVTPDDEASSRLTRQAGLDVISVGMG
RHAEHTVDLVESGELSEDRIDESVRRVLRAKFELGLFEGETADPEEAEATLRDEEHVQLSRESIQKSITLLQNDGDVLPLDDPDEIFVGGPNADDLIAQIGGWSIMDEE
GVPGKTIREGLESVTDAEITYERGAGVAEYDDVEAAAEKAAEADVAVVAVGEDWYIHEFGPQQDAGHPTGEFPTRTELELPDPQIELVKAIQETGTPTIGLLVTGRPLA
IPWMAEHLDALMMTYFPGSEGGEVVAEALTGEIEPAGRLPVSIPKSSGHLPTYFNYLRHPRPIGDDEHTDSYDPLFEFGHGLGYSEIETTGLSLSADAIGPGETVDVEV
TVENVGDRASSEVVQLYVTDEWSTRVTPIRELKGFQRVEVDAGDTATVTLELSGEELGVVGPDTPHHVEDGVFELRVDDLEAELTVESEYDY
>tr|A0A1W6LPU7|A0A1W6LPU7_9BACT
MGANRNVLKLFSLLLIFTVSLVNAEEKVEKKVDALLSKMTLDEKAGQMTQLTLKAFTNGKSGDKLKLDENKLEKYIVQEKIGSVLNCGGRALSPEKWLEITSLVQKFAK
KTRLQIPVIYGLDSIHGAGYVAGSTLFPHNIAMAAAGSRQLVQQMAEVTALETRAAGIRWNFAPVLGVARHPFWPRHYETFGEDPFIASEFAEAYITGLQGDKLPLAED
KVLACMKHFLGYSYPRSGRDRTPAWIPEIQLRELFVPPFRAAVQAGAVTAMINSSEINGRPVHASPFYLKKLLRKDIGFSGFVVSDWADIDNLYTREMVAENQREAVKI
GVNAGIDMSMTPFKIDFKKHLVSLVEAGEVPVQRVDEAVKNIIKVKFQAGLFNDPFEDDKLSEKIASKEAKKLNERCAEECITLLKNESEILPLKKEEKILVTGPCSNL
KSVLNGGWSRTWQGREEHLYPDSQNTILEAVKAEFGRGNVSFEKGADFDKLLDADKAAEKAENCDVILLCAGENTYTEHSGNIRDYNISLSQRKLAARLSETGKPIITI
LTQGRPRVVREIEKVSDAVVMAYLPGVKGADAIADVVSGDVNPSGRLPFSYPKYAGGFEWYDYKKSANWSSCKVEFQWPFGAGLSYTDFECSGLKLSKNSASKKGFDGL
KVSFDVKNKGKMKGGYIAQVYVSDKVASVTPANKRLKEFKKIYLNPGENQTVNFDLPAEAFSFINAECRRVIEPGKFIIRAGGLKKEFTLE
>tr|A0A172YJV1|A0A172YJV1_9GAMM
MSIHAPSALKVPYLGLAPARLAELERRAEALLARMTLEEKIGQMTQFTSERDVTGPSVRDDYEQDIRAGRVGSVFNSFDADFTRRMQRLAVEGSRLGIPLLFGYDVIHG
FRTIFPIPLAMASSWHLEAIEAASRVAAREAASAGIHWVFTPMVDISRDPRWGRVMEGAGEDPLLGSLVAAAQVRGLQGESIEALDSVAACVKHYAAYGAAEAGRDYNG
VDISERLLRSVYLPPFKAAVDSGVATLMTAFNALDGVPASCNSLLLDQILRREWGFEGLVVTDYTAVMELIHHGVAEDSKDAARLAVNAGVDMDMQDGYFLDHLAELVA
EEAVDGARIDQAVKRILVLKYALGLFDDPYRYSDEQRERTTLLTPEHLETARSIARDSMVLLENDGLLPLSSELERVALIGPLADSHADLLGPWHGDGRAEEVTSVLEA
LGDRLPDALITHSRGVPKTTEHGHRRKVKEAIDAAVREASQAQVAILVLGERESMSGEAASRADIGLPGHQLELALKVIGTGTPTVVVTMSGRPLVLSELAPKAQALLH
AWWPGTEGAAALVELLFGDHAPCARLPMSFPRAVGQLPLYYAQAPTGRPFDADERYTSKYLDVANTPLYPFGHGLGYTRIEYGTPRLDRDLIEGEHDTIELRIELSNQG
ERPGVEVVQLYLRDPVASISRPVRELIDFDRVTLEPGESREVSFVVSLGQLGFLGHDLAWRVEPGRFELHIGPDCQRTQCVGFDYRPAKRR
>tr|A0A397NS57|A0A397NS57_9SPHN
MKRMLFAAAAALSLATLSFAPLPLAGAPALAQAVSPDAAMRATVDRLIARMTLEEKAGQLSILGGDYGDVDDLARRGLLGGTNGVMPGHDVAAYTRHIQDMAMQSRLKI
PVWFMGDVAHGYRIVMPVPLALAATWDTALVERVHRAAAIEATSDGVDWTFSPMLDIARDPRWGRVVEGAGEDPYLGSAMAVAQMKGFQGDDLAAKDTMLATAKHFAGY
GAVTAGRDYNAVDLPPRLFRDVYLPPFKAVADAGIGSVMAAFNTLDGVPATSSRALLTGTLRDDWGWDGLIVSDYDAVKELSVHGVAADAAEAARMALRAGIDIDLHSG
TYLEQLPTLVRDGKVPIAEVDAAVRRVLEAKYKLGLFDDPYRYGDRSGMAGAVLEEHRALARQAARQAMVLLRNTGVLPLKKAGRIAVIGPMADLQRDVQGPMPALGVP
GEVVTMLAGVRGVAGGKAQVSYAPGVSVKGAETDEAGISAAVAAAKAADVAVLVLGDSIDMIGEGNSRASIDLPGRQLDLAKAVAATGTPVVAVVVSGRPLAVPWLATH
ADALVFAWLAGDQAGNALGDLLFGDANFSGRLPITVPRSLGQVPLSYDALPTGRPKAGVYTSRYVDEENTPLFPFGYGLSYTHFSYGAPRLDRTTLTQGGRITITVTVR
NEGTRAGAAVPQLYVHDELASVSRPMRQLKGFRRIDLAPGEARDVTFTLTPDDLAFVRRDMTWGTEPGGFKLWVGDDSTATQSARFALAGQ
>tr|A0A1E3H1U3|A0A1E3H1U3_9HYPH
MSGRIDDLLARMTLAEKIGQLNLVTPGGDTLTGSVANADVAGKVRAGAIGSIFGVKSREAMRAFQDLAMETRLKIPLMFAEDVIHGYRTIFPIPLALAASFDMDLVARA
ARVSAVEASGMGIDQAYAPMIDVCRDPRWGRIAESPGEDPHLAARFSEALVRGFQGDDLACPESVMACLKHFVGYGAATGGRDYDRADMSPTELHDVYLPPFQAGVAAG
AGSIMAAFHALNGVPMHTHAGLLRGVLRHAWGFDGPVVADYTGVMELTYHGIAGDLAAAAVKGLVGGVDMDMVSEAYVRHLADAVAEGRLDPALVDEACRRVLAAKERL
GLLDDPYLRIGAAGRPPVALLSAAHRQAAREAVADSCVLLKNEDGLLPLAQGCNVALSGPLADDSVNMNGTWAVCGDWRDAVPLTAGLGNRCRFTLARGANITDEAWLA
ARLNVHDSGPALSATIDGRSPEQLIAEAVAQARDADVALAVVGEAREYAGESSSRTDLRLAPGQVALLKALKATGKPVVAVVMTGRPLVLTDIVDHCDAILIAWFGGTE
SGHGIADVLFGAAEPGGRLPATFPWHEGQIPVFHARLPTGRPFPGRFQKFRSGYVDVPEGIRHDDGLYPFGFGLSYTQFAFGAPQPEKTQLSGIGDRLRVTVPVTNTGA
RTGSAVVQLYIGDPLASRSRPLRELKGFAKLTLAPGEHADATFELTSDDLSFSVAESVGDVRRAFEPGRFIVGAGPDPWNLSETAIDWLA
>tr|S6CZX7|S6CZX7_9EURY
MGIMELSDERLDQYVDDDRRAEIDERVESSLEEMTVEEKVGQLNQRSVHFITGTEDEGDELEGAIADGEVGSLLNVTDLETKRRLQERAVEESRLGIPLLIGYDVIHGY
RTVFPTPLAQAASWNPELAERAERIAATEASADGHNWTFAPMVDVSRDPRWGRVMEGAGESPVLGSAFARARVRGFQGEDLSAPDTVLACAKHFAGYGAVEGGREYNTV
NVSETALRERHLPPFAASVEEGVGSVMNAFNVHERIPASGNESLVDGILKGEWDFPGLVVSDWASFREQIQHGTASDRREAARKAIQAGSDIDMASEVVVDELADLVRD
GDVSEDRLDDAVRRVLTVKGLLGLFEDPYRYFDEQRREEAMLTAQHRDAAREAARQSFVLLENEDDRLPLDPADDVAVIGALADSAEDALGAWSFEGRSEDVITIRDGL
ESFLDREVAYAPGYSLPSEVTDESMPGEVTDESLEAAVDAATDADVAVAVVGEPADITGEAASRSQIDLPGDQRRLLEALGETDTPVVAVLMNGRPLAIEWLAESVPAI
LDVWHPGVEGGPAVAETLFGEAVPGGHLPMSVPYNEGQIPVAHDRLPTGRPPGDGVRDYASKYLDVPNEPLYAFGHGESYTDFAYTDLTLSSNALSPGETLEATVTVEN
IGDIAGRDVVQLYVQDLVGSRSRPEKELVGFETVDLEPGETATVTVELGERDLAFWTADEEWAAEPGEFELMVGHAADDIVHTERFELRE
>tr|A0A0A2VY79|A0A0A2VY79_BEABA
MFGPHPLTPEARDAYVTDLLKKMTTDEKIGQLRLISVGPDNPKEAIREMIKAGQVGAIFNTVTRQDIRTMQDQVMQLSRLKIPLFFAYDVVHGQRTVFPISLGLASSFN
LDAVKTVGRISAYEAADDGLNMTWAPMVDVSRDPRWGRVSEGFGEDTYLTSIMGRTMVEAMQGKSPADRYSVMTSVKHFAAYGAVEGGKEYNTVDMSPQRLFNDYMPPY
KAALDAGSGGVMVALNSLNGTPASADSWLLKDLLRDEWKFKGITISDHGAIKELIKHGVASDPKDAVRIALNSGINMSMSDEYYSKYLPELVKSGAVPMKELDDATRHV
LNVKYDMGLFNDPYSHLGPKDSDPVDTNAESRLHRKEAREVARESMVLLKNRLQTLPLKKTATIAVIGPLADSKRDAMGSWSAAGVAAQSVTPLEGIKNAVGQQGKVVF
AKGANVTDEKDIVEFLNQYEPAVTVDPRTPKAMLDEAVNTAKNADVVVAVVGEAQGMAHEASSRTNLTLPQSQRDLIAALKATGKPLVLVLMNGRPLALVKEDQQADAL
LEAWYGGTEGGNAIADILFGDYNPSGKLPMSFPRSVGQIPTYYSHLNTGRPYDPEKPNKYTSRYFDEANGPLYPFGYGLSYTTFKVGDVKLSAPTMKRDGKVEASVTVT
NTGDREGETVVQMYLQDVTASMSRPVKELKGFKKVALKAGESQTVSFPIDISALKFWNAKMKYDAEPGKFNVFIGLDSARVKQGEFELK
>tr|A0A511YX56|A0A511YX56_9CELL
MSTDDTRRWLDPDVPAGERVAALLAAMTLEEKVGQLVQMANPDVERHADLLTAGAVGSAIVASGAYAGNVRDAGTQASHANALQRAALASRLGIPLLVARDVVHGHRTV
FPIPLGQAASFDPDLVRACARVTAREARADGVRWTFAPMVDVALDPRWGRVAEGYGQDAWLTSLLGAAAVEGFQGGPDGAGLAEEESLAACAKHYVGYGMAQGGRDYAE
VDLGPVTLQNRHLRPFRAAVDAGVATVMSAFHTVDRVPMTAHEPLLRDVLRGDLGFAGPVVSDWDAVGEIVRHGLAMDLTGAATLALRAGVDVDMVTGAYATHAADLVR
SGAVDEALLDDAVRRVLALKVRLGLFERPFADEGRAARVHLAPEHRALARRAAASAVVLLRNDGVLPLRPVGRVHVTGPFATATEELLGTWTLDGRGEDVVTIADALRE

RLAADGVEVTVDDGRFPDASHLAAREADLVIACVGEHPLRTGEANSVASLELPPGQAEVLEGLARVSRTLVVVVVTGRPLALERLAHLGSALVLAFHPGTEGGHGIVDV
LTGDVPPSGRLPASMPWVTGQVPMPHDHLPSGRPLDPAGPLGRYRDHSDAPQYPFGFGLGYSAVEYGPVRLSAERLGAGEVVRASVILRNAGEHPAVETAQLYVQAHLA
SISRPVSELVGVQKVALAPGEEREVTFDVDHALLAHRDARGRLRHEGGPFTVRIAPFAGVGGGAGPSAQLDVDPLPDAADAADDGDDQT
>tr|D7UX19|D7UX19_LISGR
MKKEKLEALFQTLTLEEKIGQVVQVSGNVYMEDKQTIDTGPLQDIGFHSDFQLDLIGSVYNVNDWRQIKKIQTEYLHKHPKKIPLLIMADVIYGFRTINPIPLAQAGSF
DFELIQKGATETAKESYRNGLHVLFSPMLDLVRDPRWGRVMESPGEDVYVASQYANSIVTGYQGELNEEKTIEEDHVAAGIKHFAAYGAPEAGREYNSVDMSLLKLRQT
YLPPYEAALKANAKLVMTAFNLLNGIPATANKFLNRKILREENNFDGVLISDFAAIEELVDHGYAKDKQTAAQKALEAGVDIDMMTSCYANELQSLIESDQLPMELLDE
AVRRILVLKNELGLFENPYRGLEAETAGEILTATAKQTAIELVEKSSVLLKNDQQLPLEKPEKLAVIGPYGTSKLTLGFWASVTGSPLDTISLAEGLQQHFPNQQLLLS
KGYNLFDDYEAFGPLKEAVIQLNGPIDAENELVQEAVEKAKQADKILFTFGENFMESGEGASKAHLDLPEKQLHLLRELKKTGKPIVGILYTGRPLVLTEVATLFDSLL
LVWYPGTMGGIGISNLLTGKANPSGRLAMTFPRAEGQIPIYYAHHSTGRPMGTSNQQPRFTSRYTDEANEPLYPFGYGLSYSSFQITTDSAAVHCRAGDTFSFKLAIQN
TSQVDGEEVIQFYIQAAASEIVRPVQELVGSKRIFVRAKSVEEVEIEVDSNSLGYVNESGEHVLEKGSYRYLIATSSDNVHKSISIVIE
>tr|D9PYZ6|D9PYZ6_ACIS3
MLELRPEHFLRAREIVSRLSLEEAIAQLEAIPFTDLLDEDGRLSEEKARELVGSGVGIVKRVYGNGVVRDLAAAARELNRLQRFLMERVGIPAIPEEEGAGGLMVPQAT
AFPSPLAMASTFDPGLMLRVAQAIGRQASRLGVRQLFSPVLDLCLDPRWGRCEETYGEDPKLAAAMGVSYVRGVQSQGVAATLKHFVAYGASEGGRNGANAPIGYLDLR
NLVMVPFEAAVKESSPLSVMPSYNDVDGVPSHANGLLIDGVLRGEWGFGGFTASDAEALSMLIDTQGVAQGREEAALLAITSGVDVENGYAPMRERLYWALADMARSGR
APESVIRRAAERVVAAKVALGLFENPYVSYSGEPLEEPGDRELAREAAERSLVLLKNEGALPLRRDARVLLTGPSAADGRALLFDYHLQAHRGLREEVTRVVSVLEGLR
GLGLNVDFVRVSSWTRCSDEEVRAAAEATPGHDVVVAVVGEVAGGFWLADPEVTSGEGIDRDPVLPECQARLLRELASAGVKLVTVIVSGRPLAIPDDVVRSSVAILYS
FYPGEEGGNAIARALAGLVNPSGRLPVTIPASVGDLPVRYNLRRSSYSRITRRRPSAAFPFGHGLSYSRVEYSDLELITYDNGVRASFTVRNLGPLDVEEVSQLYMSAP
SQAYERPKVELVGFARVPLKVNEARRVTFSVSYEALSVYLPGDRLVVPGGTYSFYVGPSSADLRLSGSLRLDEVRLGGRGGGLWAAVSY
>tr|V3ZQT9|V3ZQT9_LOTGI
MWYFLSLLLALSVSCENFPFMNPSLPWEERVEDLLGRLTLDEIQDQLATGSGPSPNISRLGIGPFQWWSNCGRGDVRAGNATSFPESLGLAAAFSPELLFDVTKVTALE
TRAKNNYFEKHGKFGIHQGLSCFSPNLDIYRDARWGRNEETWGEDPYMAEIYARAFVTSLQGDDPRYALVTATCKHYTASGGPDSYPENRFTFDAKVSMRDLQMTFLPG
FKGCIDAGSYGLLCAYNKINGIPACANKETLNDILRTDWGFKGYVISDVAAIENMIFTQHYYNNTVDTVAGSINGGCNINAASNSQPNHTNAYYSMVQAVREGKLTEQT
VRESMKQTLYTRFRLGEFDPPGMNPYKKLDPSIIQSPDHQLLSVEAALKTFVLLKNRGNFLPMKQTKYDNVAIIGPMINNPKAQTGNYSPTIMPTYTTTPLETLSKYLA
HSVTSTNGCLGDNNCLQYNQTAVIQAVQNSDAIIVCLGLGNDVEQEGNDRRNISLPGHQLDILKDAVAHSPASTPIVLIMFNGGAVDLRWPDGEDRVTSILEVFYPSQA
TGKAVYNALVMGGGSLSVPAGRLPITWPMSDDQIPPITNFTMEGRTYRYSPPEPLYPFGYGLSYTTFHYSNLTYSDSIAAGVNFTGSVQVQNTGNIDADEVIQVYFSWN
RPSVPAPKIQLGNFTRKLIPAGSTVTVQFTIPARIMALWVDDSTGWKIEYGTYNFYVGGQQPNQKKNVGSNVLKGFFTIFADKYLGRSR
>tr|A0A1X7VU49|A0A1X7VU49_AMPQE
MRVFAVPLLFACCLAISDHVEALFPFQNTSLSIEDRVKDIVDNLTLEELVEQMAHGGATLNGPAPGIPRLHINPYQWGTECLSGNVSAGDATSFPMPIGMAASFNYDLL
KRVTNATAYEVRAKHAAAVKDGSYAFHTGLSCWSPVLNIMRDPRWGRNQETYGEDPYLSGYLGQAYVNGLQGNNSRYIIANAGCKHFDVHGGPENIPTSRFSFDAKVSM
RDWRMTFLPQFKACVEAGALSLMCSYNRINGVPACANKALLTDILRNEWDFKGYVVSDQGALEFIVIEHHYAPDFMKAAADAANAGTCLEDGNIGRKFFNVFEHLVDAV
KNNLVSVDTLKNAVSRLFYVRMKLGEFDPPDNNPYANIPLSVIQSDAHINLSLQAAMESIVLMKNDDGFRSPFLPITNEVKKACMVGPFSDDPEVLFGDYSPTLMRDYV
ITSLAGLKNANIGTDTLNYAVGCEDGPACRNYDSAKVRSACDGVELIIVTAGLSKHLESEGKDLSDINLPGHQLDLMQDAEAASKNASVILILFNASPLDIRYAKTDPR
IVGILEAYYPGQTAGKAIANVLTGEYNPSGRLPNTWPASLDQVPGITNYTMKERTYRYFTQEPLYPFGYGLSYTTFHYSNLNISSTATASGAGMIAVSVLVTNTGSMDG
TEVTQVYVWCNISYAPKLQLVGVNKDFISKGKTLEVSFSIKPEQLQVWTDDDKWVIPVSTYDLSVSGQQPNQTVLASSNVLKGSFKIQ
>tr|A0A4D7QK13|A0A4D7QK13_9HYPH
MRLVLSRAALPILMVLGLALPALAQGEATRAPLSVAWKTGAEAEAIEQRIEALLAQMTLAEKVGQLHLTGRGDSFKPEWVAEGRTGVLMNFTQPREVRAVQAQLARSRL
RIPLLFGLDAINGFATYFPQPLGQAATFNARLMEVSAYWAAREARAVGVNWTFAPMVDITRDARWGRVMEGAGEDVHLGVISAAARVAGYHRGGLATSAKHFIGYGEAE
AGRDYNSVWIPLSKLWDIHIPPFRAAIEAGAFTVMTSLSAMNGIPSSADRAMMTDTLKVKLGLRGFIVSDFESIKEMLAHGVGADGPEVVRKAMLAGVDVDMMAGLYDR
HLADEVRAGRVPQSAVDDAVRRVLRVKFHMGLFEAEPDHPAMAVAQLALPEARAAALEVAREAIILLKNDQILPLGRQTRSVAVIGGLATHQEDWQYSDNAGLPRIRPA
TLPAELTRFLAPNVRVTAERGLATHCSLAPGNTAAALRAAHAADVVVVMLGEDCEQYGEGTSRAHLELPAPQKALLDELIATRKPVIVIMHTARPFVLTGFVDRVKAVL
QTFHLGTEGRTALAEVMTGRVNPSGKLPMTFPRATGQVPIYYDQLPTGRPRLTNARYESGYLDEKIEPLFPFGFGLTYSRFTLDRLALDATSLPLTGTIAGTVRLANGA
GPAGQEVVQVYVRQRVGSRSRPVRQLKFFEKVAVAAGGEASVAFRIPVQALGFHDDQGRYSVEPGLYDVFVGTDSRASLTATVTVTAP
>tr|A0A176U903|A0A176U903_9FIRM
MTEQQLKDLLADMSLEEKIGQMVQLTANFYGTDMLITGPMGSAKIEPEDMKLCGTILGTSGAAKLKEIQDNAMAAQPHHIPMLFMLDVINGFQTIFPIPLAQGCSFEPE
IARKGAQIAAKEAAASGLHVTFAPMADLVRDARWGRVMESPGEDPYMNYCFAKAMTEGFQGEAPENLKEKGNISACLKHFACYGYPEGGREYDNVELSERTLRQDYLSG
YQGAVDGGCRMAMTSFNTLNRVPSTANKWLMRKVLREDLGFDGVLISDYSAVEELIPHGIAENKREAAKLAIEAGVDIDMMSDVYLHYLKELVTSGEVDETLVDEAVLR
ILKLKNDLGLFENPYKDASEEDEKNLILCDAHRAAAREAASKTFVLLKNENKTLPLSEQEAADVLFAGPYVDTRAICGAWSFPATYDNIKTVQECIQEHIGTACNFMKG
CSMFYDKEVIRDYVEEGMTPDEAEASIEQTVQAAKSVSKVVLFLGESFRQTGEGASRTCITLPEIQMELLKRVSAVNNNVIVVLFAGRPIEVALIEDLAKAVLYVWMPG
TEGAAAITDVLFGVKEPTGRLAMSFPNVSSQEPLYYNRFQGGRPRNPGDPKAFCIGYIDTEYRPLHSFGYGLSYTTFSYSPVTVIKRETNTKANDAEAVWVASVMVTNT
GDREGTEVVQLYIRDVSGSVVRPVRELKGFEKITLKPGESREVSFEIIEPMLRFWRIDMTYGSEPGKFEVYIGGDSTTENKAEFDGV
>tr|A0A498G1Y2|A0A498G1Y2_9EURY
MTAPPDLDTLVGEMALESKVAQLGTVRIGSLLEDGAFSPRLAREVIPHGVGRVTRVGRESGLPPRALAAVVADLQSFLRTETPHGIPAFVREESLCGYAGRKGATVPQA
IGIASSWDPTLAREVASAVGDQLRAVGCQLTLSPVADVGVEPRWGRIEETFGEDPALAATMTRHVVDGLADANVDATLKHFVGHGRPVGGRNRARPTASLDEMRDADLV
PFRAGIAAGAASVMAAYNTVDGVPCHANERLLTDLLREQWGFRGTVVSDGRGIEMLADDYEVASTRREAGVRALTAGVDVELPETECFGDRLVAAVRDGDVDESFVDRA
LRRHLRQKVDAGLFTDDSPAPASVTDAFETDAVRRLTRRAARRSQVLLDNDGALPVSADATVALVGPNADAPRNLLGNYSYAGAENADGGVDIVTPRAALRDRVAAVKY
APGCGVRATAEDDIDAAVTAARDADVAVACVGGQSGIDVERDSPGTAGEALDRAELGLPGRQTELVRRVAATGTPVVAVLVSGRPLAVPEVVDCAAATIAAWLPGQEGG
AAIADVLLGTDPGGRLPVSVPRTVGQLPVAYRQDSVSRGEYVFTPGDPLFPFGHGQSYADFEYESLTVSAPTLSPDSSITASVGVQNIADRKGTEVVQLYTRHRGGHTV
RPTRELVGFARVDLDADDRATVSFELSAATLGAHDAEGRFVLEPGRVELAVGRSVTDLRTRESIEVTGETTVLRRQSPLADASVAFP
>tr|A0A660KS62|A0A660KS62_9ROSI
MPAMAMVESGGGHDFPGKLTSQVVVCSIIAAFGGLMFGYDIGISGKKFGRRPTIQAASVFFVTGAILNTTAINIEMLIVGRLFLGAGVGFGNQSIMGAILQVHLKATNT
MPMSDYCSSEECGEAEEELDRNERSGSGHGLHLQRPQRTRRGSGQRPSLSNDIGREGSIFSACGSGPKENASAEDWANMVDGFQSKARESRLGIPLIYGIDAIHGHNSI
CNNGVYDNSIRGATIFPHNVGLGATRDADLVRKIGEATALEVRATGMHYTFAPCVAVCRDPRWGRCYESYSEDTEIVRKMTSIVTGLQGKPPDKHQKGYPFVAGRNNVI
ACAKHFVGDGGTENGKNEGNTISSYEDLERIHMAPYLDCISQGVSTIMASYSSWNGRKLHSDHFLLTEILKEKLGFKGFVVSDWAGLDRLNVPRGENYRNCISSAVLAG
IDMVMVPYRYELFLKDLISLVKEGRIPIERIDDAVERILRVKFVAGLFELNSSDRPSLDTIGCKLHRDLAREAVRKSLVLLKNGKDPKKKFLPLDRNAKRILIAGTHAD
NLGYQCGGWSFKWLGRSGRITIGTTILDAIKEAMSNETEIIYEEVPTADTLARRDFSFAILVVGEKPYSETFGDNLEGLVIPVKEGVDIISSVTNNIPTLVILISGRPL
VLEPWLLEKIDALIAAWLPGSEGGGIADVVFGDHDFEGQLPVTWFKRVEQLPLHIGVDSYDPLFPLGFGLTYKEKEEKSLNLCRNNL
>tr|G5IKP4|G5IKP4_9CLOT
MERGQIKNIISRMNLKEKISQLCSIPSIEVRMDGSKVEIENLEEKIAWGIGSFQLPARNFPPEISARINNEIQRRVMEKSRFHIPALIQEECLSGQVAKGASMFPKPIG
LACTFHTELVKQIYEAVGRETRARGGHQAFTPVLDLGRDPRFGRLEETFGEDTFLTSQMGCAAVKGLQKNVVSTVKHFAGYGQCSGGRNFAPSFVGRRELLDEILPPFR
AAVVEGGAMGVMPSHCDVDGIPCHQNRELLDDILRKQWGFKGIVVSDYYDIGRLEHLHGTAESPLEAAMLALKAGVDMDIPDGKTYQSLENQLLSPDMMDCIDRAVERV
LCIKNELGLFEQPFVEPAQAKLTVRCESHCDLALEAAAEESLVLLENDGILPLVSERKSLSIAVIGPCAHPVHFSYYSSAPHIGVSILEGIRERWEGNQEIYYEEGCGLT
KEAFTMETEVDATMMREPELCSLKEDRKMIRKAADCAKTADLVVLCLGGSAATSREAIYANNSSGDNDNLNLPGHQEELFKKIRKVNPNIVTVIVSGKPYSNERIYRES
RAVIQCFYAGQETGRAVAALLGGDYNPSGKLCVSVARNVGQLPVYYSQKRTGQFKHYLFSEYGPLYPFGFGRSYTTYTYENLVIEKRAGTVLASVDVKNTGHMAGEEIV
QLYVSDIHASVVRPAKELKGFRRVHLEPGEKKCVSFSLREEDVSFTGMDGKFGFEPGEFEILMGPDSSRGLTDRFIWEKEVEETYER
>tr|A0A1E5KYZ2|A0A1E5KYZ2_9ENTE
MKEKELKKLMNSLSWPEKIGQLIQLSGDFFHSEALAVGPKQKLGITQEIVDRVGSVLNVTGAETTRVIQEKYLAKSRHKIPLLFMADIIYGYRTIFPIPLGLGATWNPE
LVKKAYKVTASEARSAGAHVTFAPMVDLVRDARWGRCLESTGEDPVLNADFSKAMVEGFQQEKDGTIEGIASCVKHFAAYGAGEAGKEYANVDMSERRLRQDYLSGYKA
AVDAGCEMVMTAFNTYDGIPATGSEFLLKEILREEWGFEGVIISDYAAIQELIAHGVAKNDREASLMAINATTDIDMKTSCYAKQLEPLIKDQEINPQLVNEAVWRVLK
LKNKLGLFEDPYRGATVENEKLMLLTEKNRELSRQVASESIVLLQNKDNILPLAPNKQKILLVGPYGDKQDLIGLWAVHGKTSDVVSLKSSLEKEIVEEHFHFEQGCDM
LENYDFLGEFGATKEQIASIAMSGDAKKEFLKKALAAGEKADVIIFAMGEHTMQSGEAGSRTDITLPTIQKEFIKEMVTLGKKNILISFSGRPLVYTDEIKQMDAILQA
WFPGTEGGNALAEILLGKVNPSGRLSLGFPYHVGQLPMYYSEFKTGRPLTSSTHKGRFVSKYLDCPNNPLFPFGYGLSYSEIEYSELALSTDIMTEKLTVSITIKNTSS
IPCLETVQLYLQDVVGSVVRPVKELKKYKKVLLAAKAEQTISFTLTKEDLYYYTRSMDFNVESGEFLVFVGPNSEETLESRFELKL
>tr|A0A1X7VMN3|A0A1X7VMN3_AMPQE

MKLVDLLKVMELLGRSAIAVFLLFLVASRADYCEKPPFNAYLYCNYSASITERVKDLLSRLTVLEKMSQTATNASAIERLDIPAYDWWSECLHGLAQSPGVFFENDLTS
ATSFPQVIGLGATFNMSLVLAMGQVISTEARAFANNGQSGLTFFAPNINIYRDPRWGRGQETPGEDPYLTSQYAANFVKGIQEGSEDRRYLKAIATCKHYAAYNLERYL
DVRRVNFNAIVSDQDLEETYLPAFKACVQEGQVGSIMCSYNAINGVPNCANDFINNKIARDTWGFEGYIVSDCGAILDIQYKHNYTSDTNITVADALKGGCDLNCGHFY
EKYMEDAFDNSTITEEDIDKSLTRLFTSRMRLGMFDPPEIQPFRQYSVKDVNTPEAQDLALNAAREGIVLLQNKGSVLPLDIVKHSNIAAIGPNADATHIMQGNYHGIA
PYLISPLQGFSNLGINATYQIGCPVACNDTEGFPDAVKAVQGVDAVIAVIGLNNTQEGESHDRTSIALPGHQEDLLLELKKNAAKGTPLIVVVMSGGSVDLTGVKDIAD
AILWAGYPGQSGGQAIAEVIYGKVNPSGRLPVTFYPASYINEIPYTNMSMRVPPGRSYKFYTGTPVFPFGFGLSYTTFEIKWKDTSTAKDYYLKTTHDEVVNYEATVTN
SGSRPGSVSVLAFITSSVPGAPMKELFAFKKIYLEPTESVDVSFVAEPKVFTTVDIYGIRKIRPGAYKIIIGDDDHHIKHNVFISA
>tr|A0A2T4A960|A0A2T4A960_TRIHA
MRISSLTTFVDTEARAAALVAELTLDEKAANLVNNAPGVERLGLPPYEWWNEALHGLAGVSPGQGINSTFTQGNVAFNSSTQFPSPMVLGAAFDDQLVHDIATAVSTEA
RAFSNHLKAGLDYWAPNINPYRDPQWGRGQETPGEDPYHVAQYAYNYVVGLQGGIGPAKPKVVSTCKHFAGYDIEDSDGVVRGSYNAIISTQDLAEYYLPSFRSCFRDA
KAGAVMCSYNAVNGHPSCANSYMLETVLRDHWGWNSSAHWVTGDCGAVDGVFNQHHVGQSDAQGVALALNNGTDLDCGTAYSSYIASAIQSNYTTEAQLDQALGRLYGS
LIVLGYFDPPEGQDYRTLGASDVNTPATQKLAYTALVEGITLLKNDDNILPIRPTGQTVLFVGPWANNASVSMFGNYNGVAPYKTIPVPTANSSSYNWKVTYSQGLQYV
LSNDTSQFAAAVSAAQEADVVVYIGGIDEQVEAEAHDRTSIDWPGAQLDLIQQLAAVKPVVVVQVGGGQIDDSSLLQNGNVKGLLWMGYPGQEFGPGLIDILSGVAAPA
GRLPVTQYPADYINQVPMTDQSLRPSSSNPGRTYRWYNGSVIPFGTGLHYTEFNVSWATGGSGKGTYDTADFTDVEDPKDLAEFDTFQINVENIGSTASDYVALLFLKS
TDSGPQPYPLKTLVAYARAHNVQPGATTTLDLKVNVGQIARNDANGNLVLYPGTYTLQVDLENLSGPTAEFQVQGAETVLDQFPQP
>tr|A0A542DYC3|A0A542DYC3_9MICO
MTPTRRTATAPDRPWLDPALDVERRVDLLVDAMTTEEKAAQLTQVDNLEPGRDADLLRRGVGSSLYASGATAGNVRDGGVLASAVDECQRLAVEGSRLGVPVLFGRDVI
HGHRTVAPIPLGLAATFDVDLLRRVSALAAREASTEGVAWTFAPMMDISEEPRWGRVAESLGESPVLAGRLAAAMVEGFQGTARERRDGAREQQGGRPTLGATAKHYVG
YGLVQGGRDYDTVTVGENTLRNLHLRPFRDAVDAGVMAVMAAFNDVDGVPMHAHRHLLRDVLKGEWGFDGVVVADWNGIGQLVNQGVAADLRDAARQALLAGVDLDMCS
GAYLDHLPDLVEDGEVPLDLVDDAVRRVLRMKLRLGLFESPYTDPSRTLAAPGPQERALAREAAARSMVLVKNDGLLPLHANIGKIHLAGPFVHDGDALLGTWVLDGRG
EEVVTPAQAFAERLAAEDLVVSDGRFSDVAMSMVREAEVTVAVVGEHRSRSGEDRCISTLELPAGQLEVLEEMAGLGKPLVVVVHTGRPLELGRVLELADAVLVAWHPG
TEAGHALTDVVFGDVSPSGRLPMTFPRTVGHIPSSSHERPTGRPIDRDRDRQLGRYLNSLVFPELTFGYGLTYTHMEYGDLEASRTTLPVKGNGSVSVSVEVTNTGVRA
GREVVQLYVRDLVADVTRPLVELADWRVVDLEPGESTKVVFKVTPRMFGYHDRELRWRVDPGEVDVMVGPNAAYFSRVRLTLVDGK
>tr|A0A0R1N9E4|A0A0R1N9E4_9LACO
MILNKTTLKALFDDMTSTEKVAQTIQVNGDLVGEGSLKTGPQVDLGFSRGFDFNTAGSVFNVTKPDQLRALQKKAIEESPHHIPYLFMADVIYGFRTIFPIPLAQAGSF
DFDKLKKAAQVTARESYNNGIHCLFSPMLDLSRDPRWGRVLESPGEDVYTAKAYAKSIVQGYQGDDGDVSMPGRVAACIKHFAGYGAPEGGREYNTVDMSTQRLFNEYL
QPYQAAIDANCQLVMTAFNVLNGTPATGNRWLNRDVLRGRFGFSGPLISDYSAISELQSHGMTTSVEESAKTALIAGVDMDMMTSVYANALPQLIRQPEFAKLLDEAVW
RLLVLKNKLGLFEDPYRGLTTTNTGEVLRPEDRELATRLVEESVVLLKNEDVLPLRKGKSVALIGPYADSRLTLGFWASVTGNPMDTVTLREGLKQSYSDEQLTVAQGF
NLFESYSNLGAYQHILEKVNGPLQDEQKLIQLALTAARESDVIVLTLGEQFMESGEAASKAHLALPMREKRLLKALADLHKPIVGILYTGRPLVLTDIVPYLNALLVAW
YPGTMGGVGIANVLTGVASPSARLSTSFPRSEGQIPVYYAQNTTGRPMGQNNHSERFISRYIDEANQPLFGFGAGLSYASSVAHWQTPSITDNGVLLPYVVTNNSDRSA
AVVVHIYVHQPASPIVPPEKRLVDSEFLILAARETVETQAIINQSDLSFFDNKGVSHFQSGNYEFILDVQGQEEKTNIELGGFKQ
>tr|A0A1H7FME3|A0A1H7FME3_9FIRM
MKNEDLKKLLDDMSLKEKVLQLVQLPGQAFEENAAVTGLSDEGASEEEKSLVGSTLGIWGAEKIIEIQKKYMDKHPHHIPLLFMLDVIHGHKTVFPCPLGQGATFDPEI
AKTGAEVQAREAAAEGVHVTFSPMGDLVRDARWGRVLESTGEDPYLNGKMVSAMVEGYQGKDLKDINHIAACVKHFAAYGAATSGRDYNSVELSESMLRDQYLPAYAEG
IKKGARLVMTSFNTLNGIPSSGNTWLMRDILRNEMEFDGVLISDWAAVAEMATWGFAKDLKEAAELAMKAGVDIDMCTESYGRNLEELLDEGKISKDLLDEAVMRVLTL
KNDLGLFEDPFRGASATREQEECLSDKNREIARDAVRKSLVLLKNDYNALPLVEKKIAFIGPYSDEKNLHSSWAISCDSKDAVSVKKAAKELYDDTDTRIKVAKGCLIA
SNGDEFMNGTFSDEKEKEKNKKLFKKAMEVAEWADEIVFCLGEHIGQSGESTSKTDLRIPRAQKKLFEKICKKFRNDKHIITLIFCGRPLELENIPEKSDALMICFRPG
TEGGHGIMDVLGGNYSPSGKLSMSFPYSAAQAPLNYNAFSTGRPKPSEGLSIFTTRYLDCPNEARYPFGFGLTYSQFKISDVILNKNTLNKADEDDFIRATVTVRNTGF
HEATETVQLYIRDMVGSRVRPIKELKGFRKVSLLPGESKEISFDISEDMLRFWTIENKNDSEPGDFKVFVGFDSTTDNSEDFELI
>tr|A0A1J4JGT0|A0A1J4JGT0_9EUKA
MLFTYLFCISLVEKTPLYLDPSAPINDRIQDLMDRMAIEEIVGQLQQVDGHFDFIGPMKDQHPGSMFSLLGFTAGHVIELARESRLKIPVLMGIDAIHGHAFYSGGTVF
PTQLGVAQSWDEELIESQGNVTAFEIRYTGPSWTFSPVLCISRDARWGRVGETFGEDPFLIGKFATALIKGLQGPDGVTNDPNKVAACAKHFVGYSETRGGRDSTEADL
SYRKLQSYFLPPFEKAVKEAKVATFMSGYHAIDGTPMTLNDFLLNDVLRGKWGFDGFVVTDYSNIGYLISNQYIFDNYVDASAAAVKSGNDMSMAVTDFYQAALDAVTS
GKLDMEFVNQSCRRILELKFKLGLFEDDRYPDANKAAERNGSPENRQVALQACRESIVMLKNDGFLPLNPTNIQKLAVIGPNADNPLSQNGDWSLGTGQVDFFGKHPRN
CTITYVDGFTNRLKEANVTVKYALGCGIEPGEKADLEEAIKIVEDSDVTVVVVGDRLVYYGEMRSTGTLELMGGQLELLRRIIETKKKFIIVLMSYKPLIIPDDIIEHA
SAVLLQFSPGMMGGQALAEAVFGDFSPNGRLTISIPRHAGQIPVYYNKVRFSHQNNYVDLSTTPRFAFGYGLGYSEIEYIDAKINKNEFQKDEVIKISVVLRNTGKFDQ
YEVVQAYLHDILTSMTWADIELKGYSRVLVKAGEEIETTIEIPVNECSIVNAKGERVVEPGDFQVRIGKASDNIIFTLDFNVKDE
>tr|B8CZK9|B8CZK9_HALOH
MVVMRRYTPFSVAIIILLLFIMGSGSVYSESIDRKVNDMLSQMSLEEKIGQMFVVEVSTVMERRGPFGTSGRIDTRKLKGLTNVGSIFSGGSLRANPVPNNPATWTEMI
NNIKQVFNDNSRIPVIYALDAIHGNNKVIGAPISPHNLGLASTWNPELVERVYGYTSDSLEAIGVSWNYAPVLDVARDPRWGRTYETFGEDPFLVSVMGAASVRGIQKS
GRVCATAKHYIGYSGSENGMDRDPSYIPKRELYEVYYPPFKKAVAEGVKTIMVNSGEVNGIPVHVSKWLLNDLLRMELGFSGVIISDYADISKLHDYHMVAKDYEEAII
RAVNAGVDMFMEPDNYPGFYRFLIEAVKEGTVSEERINQSVSRILKLKMELGLFNEELKDPANAEKIISNNSEAVELFRQAARESIVLLQNKDNVLPLSREIKSVLVVG
NCAESMGNLCGGWTINWQGPEETDLTTGKTILEAIKEKVAPGTRVDYIKYRLESIDIYVKKVLEAASDAEAIIVAIGEEPYAEMMGDVQNIQLPADQIKLIKALGNTGK
PVITVLITGRPLAVGPILNSTPGLLLSFLPGTEGGNAVADVLFGDYNPSGKLPITIPKYTGQLPLYYNHKPGVDYDPQFPFGYGLSYTSFEYSGLTVKKQHDSLQISVD
VKNIGDVGGTEVVQVYINDVYSSVTTPVRELKDFKKVYLRPGETRRVEFVIPFTKLTLYNENFVEVVEPGTFEIQIAGFSRRVKI
>tr|L0M3W6|L0M3W6_ENTBF
MSRITTLLAQMTLEEKVGQLVQLSGEFFVDEPQLTVGPKAKLGISQQQVQCAGSVLNVSGAKKTRHIQEQHLQNSRLKIPLLFMADIVYGYRTVFPIPLALGCSWSPAL
IERCCQVAAKEAVSAGSHVTFAPMADLVRDARWGRCMESTGEDPLLNSRIVSACVRGFQHNLEHGEGLASCVKHFAGYGAPVAGRDYNTVELSRQSLFEDYLPPYLAAV
QAGCKLVMSAFNTIDGVPATANRWLLQDVLRDRWHFDGVTIADYAAVKELIPHGVAEDERHASQLAFNAGLDIDMKSPCYVNHLAALVNQGVIPEEALNQAVMRVLQLK
ETLGLFDDPFRGASEEKERAWFYHPDHLALARETAARSLVLLKNDNALLPLKPGTKVALIGPYADNPDIIGLWAVYGETSHSVTLKQALDEVLAPDELLTTQGCDLLLN
YDFLGEFGATTQVDAQSCMSEEQRQTEQARALQYAREADVVLLAMGEHMMQSGEAASRTDIHLPQHQVEFIQAIARVAKKSVLILFNGRPLVLSDVVEHVDAVIEAWFP
GSEGGHAIADVLYGKRNPSGRLSMSFPRANGQVPIYYNALSTGRPADKSDHSPRFISKYLDCPTTPLFPFGFGLSWHRVELSELTLAAQFCAGETLTVGITVRNLTPHA
GIETVQLYLQDCVASVARPVMMLKEFQQVMLKADETRRVIFTVNEEMLTFFNAEGEKVLEPGRFNIFVGTSSVEVLEGEFIYRAA
>tr|M0MRD9|M0MRD9_HALMO
MGDGPASRGDGRREADGTTDPVGAIGSDRSIEALLNELTLSEKVGQLNQLNGTDETGPSVGTVDLDAEIAAGRVGSMLNTDGMAARERYQRLAVEESRLGIPLLFGFDV
VHGYETVFPTPLAEAASWDRETVRANAAAAAAETAAAGITWTFSPPCDVARDARWGRSMESSGEDPYLAAELTHERVRGYQGTDLAAPDTMLACVKHFVGYGDVLAGRE
YNTVDVSETALRERHLPPFEAAVEAGVGSVMTAFTDFERVPVGAHKGLLRDLLKDEWGFDGVVVSDWNSVRELLPHGIAVDEREAARAALEAGTDVDMVGHIYSKELEE
LVAEGTVAESLLDDAVRRVLETKRRLGLFEDPYRYFDDARRESVVGSERHREAAREGARESMVLLENDGMLPLTDAGTVAVGGALATEGTDVLGEWRARGDGDDAVPIL
AGIENAVDAARIEFVEGYDRFGETTPEKRDELVETVAGADVAVLAVGEPWELSGECSSRTDISLPGDQRAVLEAALETGTPVVTVILSGRPLAIPWTAENVPAVLEAWH
PGSEGGNAVADVLFGDHSPSGKLPMSFPRTVGQVPLQYDELPTGRPEATAEPGWSSSYVDCPNDPLYAFGHGLSYTEFAYDDLAIGAQTISPTDHLDIEATVENIGERA
GREVVQLYVRDVVGSRSRPTIELAGFEKISLEPGERRRVAFELGPKTLATWTADGERRVESGEFEVFVGRASDDLRLSNGFTVAE
>tr|R9NAL5|R9NAL5_9FIRM
MDTRDLKRLLSGMSLREKIGQMIQLTGMYFDEEAVLTGTVGEVKPEEWVIRYAGSVLGMDGADKIRAVQEKYMEEHPHHIPLLFMADIIHGYKTIAPIPLGQACSFHPE
LVHEAAEYAAAEASSEGIKATFSPMVDVSKDPRWGRIMESFGEDPFLNGVMGIAMLEGYQGYGSIGGARIAGCLKHFAGYGAVNAGKEYNDVELSRRTFMEQYMKPFRM
AVYADPAMVMTAFHAVDRTPVSKNRELLEEILRGELGFGGTIISDWGAIGQLEEQQVASGQKEAACAAAEAGVDIDMMSPAYMSHLEELVEEGRILESAIDRSVWRILQ
MKNRLGLFEDPFAGMKRSVCLSPAAKRGALRLACESCVLVKNEGILPLDPGQKVFWAGPYIDSREFLSRWAIFGEHADVETIETVLKGRKIPVKLIPGCEILSEEEMRI
WCAGDGKPVEEGIQGDRKDVDWQKQYAGVEEIREDDTVVLVLGEHEAQSGEAASRAFLDLPERQAELFDMVARRTSHIVTVMISGRPLDLRRIAERSEAILLAWRPGTM
GAQAIVRLIYGEESPSGKLAAGIPWCTGQIPVSYWDMTTGHRMSEADPGNRFCSRYMDIPNTPLYPFGFGLSYTEFEITPPQMRKEGAGEEERICVSCRVKNTGDSSGA
EVVQCYVETICAPVVRPAKELIRFRKVFLEPGREREIEFAIERKEFVFYGEDMEPIDGGIPLRITVGNSSYNAAGSVDFTVGGSV
>tr|R9PTK2|R9PTK2_AGAAL
MSQPYTNPSLSVAERVENLLSLMSVEEKVGQMLQLPANMPENLDKLESWNVGSYLHCTGTMVEELQQRAAKTRLGIPLIFGIDAIHGHCFENNGTVFPTQLALSCAWSK
QLSQSMARITAIETRACGLHWTFSPVLCVGRDSRWGRINETFGEDPWLIGELAAAAVYGYQGDNLASHDTILACAKHYVAYGEATGGRDAYEAEVSPRKLLSLFLPPFE
KAVKEANVATLMVGYQAIDGVPCSANSWLLREVPKNQWGMDGFIVTDWDNIGSLHDKQRVAEDLRHAAKIAVESGNDMIMTTPSFYQHTIDLVKDGELDIALIDDAVSR
ILKYKFELGLFEDFRYTDLSKKEQILGNADHWQAALEASRQSLTLLQNNGILPLSDASKPKILLCGANADDVVAQLGDWSFGSMQAGAADDSYHQKDAITLRQGLQAAA

DAGRCELSYVRGAAPAEAEFGEISSAVNAANNSDIIVACVGDTLSQHGEFHDRADLDLSGQQQAMLEALKATGKPLIVVFMASKPLTIGWVKQHADAIVCAFNPGAKGG
QALSELLFGELNPSGKLTISFPQHVGQSPVYYNKYEGWHARLSERTDNQERYIDMPLLPLFSFGEGISYSEFSYSDLAVSTPCLAANKDLSQGEALKVSVNISNISQRS
GVEIAQLYIHDCAASVTVPVLQLRGFERVELAPGETQRVEFSVPFADLALINTQLQKVVEPGAFKVFVGPSSKAEDLLSADFKVA
>tr|A0A0W1RCA5|A0A0W1RCA5_9EURY
MTTHDTQITQTDETDEHVESLLDEMTLHEKAAQLAGTYVGTMGETRTVEDAAEMVRDHGIGFVTPFGYGASPHRDSEEVVEIANELQRIAREETRLGIPILIPIDAIHG
NAYVEDTTVFPHNMAVAAARDRTLAEQIGTVTATEVAATGSSLTYGPTCDVARDPRWGRAFETFGESPYLVGELASAKARGIRDAPVDVAAMAKHFPAYGEPERGEDAS
PVDKSLSSLYRDFLPPFEKVLDEGVEGIMPSYNSINGEPSHGSHYWLTEVLREQLGFDGYVASDWNGINMLYENHRVADTRREAIRQSFTAGVDVHSLGEQGHVEHIVS
LVEDGAIDEAAVDSSVARVLRLKSDLGLFDDPFVDSETATETLGAETHRGISLDAARKSLTLLENDDDCLPFDSNVDNLLVAGPNADELTSQVGGWSLKETDELDGITI
KDGIEAQVSDETTVQYERGSGIAEPDDVDAAVAAAKEADAAVVVLGENWYLHEFGPQDVNGPTERFPNRADLSLPDAQRDLLERILETGTPTALVLVSGRPLALPWAAE
NADAILHAYYPGADGGTAVAETLFGDNNPSGKLPISVPRSEGQLPVRHNYLPHPYPIGEDEHLPSYDPLWPFGHGLSYTTFEYRSLSVSNETVGEGDSVTVSATLANTG
DHAGEEVVELFGGREYSSVVTPVEELVEFERVALEPGEERTVEFEVPVAAFDVVRPDGTSRFEAGTLSLRCEGLEATLEAVSQR
>tr|A0A1I6LBI5|A0A1I6LBI5_9EURY
MPTVPYRDPTEDVETRVADLLDRMTVEEKVGQLLVEPATGSVDEVVDTVTSNVDRHHLGQASPFGREDAPDTPEAMVEVANTIQAYAVEETRLGIPLLLISNAVHGNAY
AKGAAVAPHNLGMAATRDPELLEEMASVTATELAVTGAHQNYDPICDVGREPRWGRVFETFGESPRLCGAMGAAKVRGYQGDGIAGEETVLATVKHFPAYGEPVRGEDG
AVNELSESTFRRMHLRAHAAAIEAGGGAEAVMPSYAAVDGFPVHGTRKYLGDLLDGLEFDGFVVSDHHGVRMLHDDHQVTGSLRESTLAAVGAGMDAFLDQPAAGTYAD
ALVGLVESGEVDEERLDESVAPVLEAKFESGLFEDPSVDLERAKETLRTDDHREVALDAARESMTLLQNEGEVLPFDPTVSEVFVAGPNADDLDHQFGGWSRGDNEDGV
SIRDGVAAVTGDETSVTFEQGATMTESRDLDAVARKAASADVAVVAVGESQYIHEFALAEKSGETGEFPTRSQLHLPDAQRELVEAVVDSGTPTAVVLVTGRPLAVADV
ADAAPALLMAYYPGSEGGTAVAETLFGEHNPSGTLPISMPRSAGHLPTRFNRSRDTDVVGPHEHPASYDPLFPFGHGLSYIDFTVEDLSLSAETVAPDETVEATVTVRN
AGQRPGATAVDCYVSDVQSSRVTPVRELADFARVELDAGDAAEVTLGVDVSDFGVRQPDGERVVEPGAFEVQIGDETARFAVAE
>tr|K9ZYC0|K9ZYC0_DEIPD
MRIPVLLASLTVTLAAQSTSAQSTTSPAAAPPLPYQDRAQPVEARVADLLARMTLTEKIGQLTQINVTRLMGQNEWDRGPLSERWLDVVLGEHQVGSLLSGGGSAPVPN
TPEAWARMTNDLQRYTLTHSRLKIPLIYGVDAVHGHNNVKGAPLFPHNIGLAATFDVNLTRDINALTARALRATGISWNFAPVADVGRDPRWGRFYETFGEDPTLTAQL
VVASVQGLQGEKLGPATVAATLKHFIGYSVPQNGRDRQPAQISRESLQRVHLPPFQAGMKAGAATVMINSGALNGEPAHSSRGLLTELLREELKFPGLAVSDWEDIARL
QTVHKTASTYQDAVRQALGAGIDMSMVPNDAPAFTSAVKTLVENGSLPLARVDEAVRRVLALKFELGLFEQPYVDPAAAGSAVTAGQDLALRAARQSMTLLKNDSELLP
LKGRRSVVVVGRRAVDPRSQLGGWSIGWQGLPENEDIPAVTVLDGMKQVLPKGTRLTYSEDLSAALPANTDAIVAVVGEAPGAEGEADNPGLTLPQEDVALLRRALGSG
RPVVAVLLAGRPLLLPDDVQRGLRALVMAYLPGSEGGRAVADVLYGNTSPSGRLPFTWPKSVSALPMIEGATPGQNAQALYPFGTGLSYTRFQYAGLSVQGAGARQTAQ
VRVTNSGQVAGGHTVLAFARPASGGTAGGERLLVGFTRVTLKPGETRTVALPLDLTSLRVAAPGQAVQSAELSVGELRTALPTP
>tr|A0A5C5WWZ1|A0A5C5WWZ1_9BACT
MNIDDLIAQMTLTEKIGQLNLVNPGGQTLTGSVANSDVHRKLVDGQVGMMFGTASLESRCEIQSISVNQTRLGIPMLFACDVIHGYRTALPLPIALSCTWNLAMIERAA
HLSATEARADGIDLTFGPMADLTRDPRWGRVAEGNGESATLSSWITAAVIRGYQGDGETSQPSDIDRMLACVKHFAGYGAVDGGREYASVNLGPIELHESHLPPFEAAT
CAKVAAVMPGFHALDRIPVTAHRDLLIGVLRERWNFEGAVISDYTAINELEHHGLGDLSSAAVAAINAGVDVDMVGESYITTLEQSVASGRVDVARIDAACRSVLKLKR
DAGLFDDPFRALDPERAKQVLGCDAHRNEARQMAAASCVLLKNKSETLPIGKECRRIALIGPLADDRSNLPGTWSVSAIAEECVSLADGLRSTVCDQTKVVVARGCNLV
DDPVQAERLNVFGTTVTLDSRDAQAMIDEAVAVARDSDVVVLALGEAKEHAGECSSRTDLQLPPPQRLLIDAVADLGVPIVLLVFAGRPLVLTGIADRVNAMLYAWYGG
SMAGPGIADVLTGSHVPEGKLTMSLPRSVGQIPVHHDALPTGRPLPPDCEFMKFKSCYLDEQNDPLYPFGFGLSYTTFAYRSLSARTDSGRVIVSVEVKNEGQRRGVEI
VQCYQLPGVCSVSQPLLSLKSFERVQLEPGETRLVEFVIDKQTASSLEGTSLTNVSRGFIPGLFRFAVGPNSQDLQHVQIDWR
>tr|A0A0N0RTV2|A0A0N0RTV2_9HYPO
MNIYQIKNPDSIFTNNPGVPKCDRDPLCSTGVCNTDLSIGDRAAAFVKNMTLAEKIQNLDNVAPGVPRLGLPPYNWWSEALHGVAGGNGVSIQPPPGNFSAATAFPAPI
HMSAAFDFDLVFSIGEIISTEARAFYDFGFAGASYFTPNINPFRDPRWGRGQETPGEDPFVGSQYALAMVTGLQGQDPDYLRVIATCKHYAAYDVENTRFQNDVHPSMQ
DMSEYFLPAFKKCVADAHAASVMCSYNGVDGTPSCANSWLLETVLREHFGFSEAYNYVVTDCDAVDSLVTGLHFAPNLSAGAADALNGGTDLDCGSTFGSNLNSAVQAG
LTTEAAITKAVTRLVAALIKVGYFDQPDQFAHFSWADVNTTSSRNAAYQAATEGMVLLKNDGTLPITKAPGSVALIGPMCNATTQILGNYAGTPAVVASPLDAFKSKWQ
NVQFQQGTGISSDDTSGFAAAIAAAKNADVIFYCGGLDKSVEDEARDRDNIDWPGNQKDLITQLAGLGKKLVVLQFGGGQVDDSDFLKSSGVNSILWTGYPGQEGGRAI
FDTVTGASSPAGRLPVTQYPSSYTDSVDMLDMTLRPGSNPGRTYWWYNDAVVPFGYGLHYTQFEVTPTGTAFPSTDISLLPGNVTVSFNVKNVGSRTSDYVALLFVSSC
SGPTPCPLKKLVAFTRLHDIAPGQTQKADLVIDTDLLARADDKGTQSIYPGMYFLMLDVVNPTGTSFTLTGSTKQVSTLPSQ
>tr|A0A2Z4UDZ3|A0A2Z4UDZ3_9FIRM
MTEKDLKALLSDMSLKEKIGQLTQLDASCFSEEEGPVTGAETELGFSAEDFPYAGSILGVVGAEQIEKLQKKCMEKQPHHIPVLFMADIINGYRTVFPIPLALGCSFEP
KEAKEVGDVMAKESAAAGLHVTFAPMVDLVRDARWGRVMESTGEDPYLNAQMAAAMVQGIQGEAADYDSHLGACTKHFAAYGAPTAGREYNTVELSERTLREDYLPSYQ
AAIDAGSAMVMTSFNTLDRIPATANQWLMKDVLREEMGFQGVLISDWNAIGELITNGVAEGKKEAARQAIQAGTDMDMMSGCYMSQLENLVQEGTISEDSIDTAVLRVL
ELKNKMGLFENPYRSAGTDSEEKNILTEENREAARKVAADTMVLLENKDHFLPLKKEEKVAFIGPYAEEKRMLGAWSFFANPEDTVTCREALEEKTENGVFAKGCGILN
PGQTVYGFRFNMTNEDSEEDTEKMIQEAAEEAAKCEKAVLFLGESCLQSGEGGSRGDITIPEVQKKLLRAVAQANPNLAVVVLAGRPLDIREIKEHAKAIVYAWFPGTE
GGHAIADILYGDRNPQARLAMSLPWCVSQVPVFYGEFPTGRHVEDGEQIENRFLSRYTDMPNQPLYPFGYGLSYTEYSYGEVQADKQEFSAGESVQVWTTVKNTGNREG
TETVQLYITDECGSVARPLRELKGFQKVTLKPGEEKKVCFTIEESMLRFYRADMKFAAEPGTFTVRIGADSRTENRVKIYLK
>tr|A0A3N1KR67|A0A3N1KR67_9PROT
MKRAAILLALLCLAAPARANEAISARVEALLGRMTIEEKAGQLNLETADWSQARDLYLSDAAEARLRAGRIGGFFNMHERALARRIQEIAVHGSRLGIPVLLGYDVVHG
YRTIFPMPLAQAASFDLARIERSERIAASEAIGDGVNITFSPMLDTSRDPRWGRTAESAGESPWWSAQVAVARLRGFQGERLAATDSLAACPKHLGANGATRAGLDYTG
AEIGERELREVHLPPFRAVVATAPCFMAAFNTYDQVPAAASPFLLRRILRDEWRSGAVVMSDFGALLELERHGVASDDAMAARLAIAGGLQLDMASAVYRDQLPALVRA
GRVPEAAVDEAVRHVLRLKEQMGLLDDPFARFTASEGMASPEALGHREEALAFAERSLVLLQNRGDRLPLRTDVRRVAVIGPHADAPQEMLGSWVGRGAYSRPVSLAAG
LRQVLDPGAEVVAVPVGDFAGATPAEQADALAAARAADSVVLALGEPASMSGEASSRTEIDLPGAQNDLAEAVLAVGRPTVAVLFAGRPLAIERLSQRADAILLAWFPG
TMGGTAVARMLFGLAEPVGRMPMTTPRAVGQIPLHHERLPSGRPAVKWPDVYSANYVDRPTTPLYPFGHGLAYTRFAFQPPVLDRTTIGNGETVNVSVTVRNVGPRAGT
AMVQLYLRNRVAPISRPVQMFRDFERVTLAPEETATVTFQVKAEDFAYWQAGDRFAAPAGPIDVMTGPSAGEVQTVRLEYAP
>tr|D7BB48|D7BB48_MEISD
MIKRWRFTLACAVLLGSCPALAQPLYKDPAQPVEKRVADLLARMTLEEKLGQMTQVAVSKLMSDGWGLGPLKGELLERYLVQRGIGSVLSGGGMGPVPNTPRAWAEMTN
AIQRAAVEKGRLGIPLLYGVDAVHGHNNVVGATLYPHSLGLAATWNPALVEQVARRVGQELRATGTLWNFAPVADLGRDPRWGRFYETFGEDPLLAGSLVAATVRGLQA
GRVAATLKHFTGYGQPLGGTDRSPAFLAPRTLQEVWLPPFRAGLEAGALTVMANSGSLNGVPVHASRYLLTDVLRGQMGFKGVVISDWNDIDKLVGDHKAAAGFADAVA
MSINAGVDVYMVPMEVDRYLQTLKELVEAGRISRARVDEAAGRVLWLKFQLGLFERPYVEAAEAEKVVEAQRPLAKQAALESITLLENAAFTLPLTNVKTLLVTGPAAT
DKTMQMGGWSIDWQGKEGAKAPGATVLEGLQKGAPQGVKVAYADPKDARALAAAVRASDAVVVALGEKPYAENEGNNLTGELPAEQYKLLRDLKALGKPVVLVLLAGRP
LAFPDDAWLVPKAILMAYLPGSEAGSALADVLFGRHNPSGRLPFTWPKLFGQVPFTYDRYPDIYPKAEPLYPFGYGLSYTDFTYRLQAARATANAVEVDVEVRNTGKRA
GSDVVQLYTRFPPLGLLAPRERLVAFTKVNLEPGQTRTLKLTAPLDRFALVPGDVFSLELPRVLPGRYVLRVGEQTVGVEVR
>tr|A0A4Q9VLD7|A0A4Q9VLD7_9HYPH
MVRFLRPLSLLLALLPAIAGAGEAPVAPPRDARIEAEIAALLARMTLEEKVGQMYMGGWSPDFDLSEVTSSSIGALSGPPDAPTIASIAATARRTRLGIPLLFTQDMIH
GYRTLFPMPLGLAASFDPRVIAAAAEGTAREAAAQGMHLALGPMVDLSRDPRWGRVVEGPGEDVFLSRLFAEATVRGLAKGGLAATLKHFVGYGAAEAGREYAATWIPD
VQLRDVYLPAFHAGLSAGAPMVMAAFNALNGIPVTANRFALTTLLKGEWGFDGFVVSDWDSVSELKAHGLAATDSDAVALAVNAGLDVEMAGRLFPRLLPDLVRAGRVP
MARIDDAVTRVLRVKHRLGLFDATRPAIDPAAATAALATPATREIAREVARRSVVLLKNDADRLPLVAPPPRIAVIGAAAADAGDHMGAWGAAGRREDVPLLLDQLRDR
LAGRSEVTFSAPCDDACRSTDGFDAAVATAAASDLVIAVLGEPWWMTAEATSRTRLGLPNHQEELLTRLAATGKPIVLVVIAGRPMVLTETLPKVAAILWTFSPGTMGG
AALADLLLGEASPSARLPMSLPRAVGQVPISYDQLPSGRPTGPDLLTSRYLDEEASPLFPFGYGLTYTRFAHRDLRVVADRLRLADTLAVAVTVANVGSRPGREIVQLY
VHDLVASRSRPLRQLAALAIVDLAPGESREVTLSVPVADLAFHDDAGRRIVEPGVFRVFVGGSSDATLSADVTVVEGTPSP
>tr|A0A4R2H0Z2|A0A4R2H0Z2_9HYPH
MPHKPGRSASPLAGLAPEDILTRMTLDEKIGQLHMVSADAVITGPSGPPGGLRDLDQGRIGSVLNVWGDAAAGLQRKAVEQTRLGLPLLFCLDVVHGFRTVFPIPLAEA
ASFSDGIWRETAAAAAREAAEAGVALTFAPMADVSRDPRWGRICEGGGEDPLVNARMAQAKIAGFQGDDLRDEARLAATVKHFAAYGAGVGGREYDSADVSDYAMAADY
LPPFRAAVAAGVAALMPAFLDIAGAPMTANRALLTDVLRKTWGFGGVTITDYNAVAELIAHGAAEDGLAAATLAFNAGNDVDMVSGLYHRHLAEAAALGLVALADIDAA
ALRVLRLKQALGLFENPWRVCPPATPVQVASRREAARRAARESLTLLRDPQGALPLPRQLARLAVIGALATSAADMRGAWAETGDVEAIVTIADGVRAAWPQADVAVCG
DDPQEAAAAARAAGVAILCLGETAAMSGEAAARANPVLPDGQQALLDAVLATGARVIVLLACGRPLVAPQLFVADCAVLVIWGLGSEAGRAIADVLSGDASPSGRLPVS
WPRAIGQIPVFYARRPTGRPHEAGARYSNGYADLPIAPQFPFGHGLGYATFSWSDLQVSAMDRAGATSPLTISLAVRHETGAGGWPQDGRTAAIETVFLFARVRVPGRT
RPLLLLRDFTRVALRPGETRRVSFEVSRDWLTREGLAPAPAAVSFLEIIVAASAEPEVDPARCLRRVLVAQKGAEHGRAAG
>tr|A0A542LUL6|A0A542LUL6_9BURK

MQGKNTTRIRTAVLLATALWAGQAGAAGYPFQDTALAPEARIANMLSLMTVDEKIDALSTDSGVARLGIPSFGSTEGIHGVVQRGDQDHQKARITTTQFPQPPGMGASW
DPALVRRAGAVQSTEARYITQSAAYQKPGMQPGVQPMLMQWGPQADLARDPRWGRSEEVYGEDPFLAGTMAVAFTRGIQGDDPTYWRGAALLKHFLANSNENGRGNSSS
DFDERLFREYYAEPFRMGFEEGGARGVMAAYNAWNGTPMGVHPLLNSLVIKQWGVDVVSSDGGAIGNLVKLYKRYPSQKEAAVAGLKAGINQYLDTYKDELRAAFKEGA
ITEAELDAALARKFRITLKLGLLDPAQGNPYAAIKDGAAPWDSASHKAVSLQMALESIVLMKNEQHTLPLSKTGIKRIAVIGPHADSVHWDWYGGIPPYKVTALDGIRA
ALGPGVQIDYVPDNGGGAAERAARAADVAVVLVGNDPTCGPNMGKEWTDAGTKPCADPGDGREGRDRETLTLAQEALVRQVLAANPKTVMVLMSSFPYTINWSKQHVPA
IVQMAHSSQDQGSALAQVLFGDYNPGGKLVATWPASMDQLPPMMDYDIRHGRTYMYARGEPLFPFGHGLSYTSFRYTRLRTDAAALPRDGALNVEVEVANTGKVAGDSV
VQLYAAWPKSAVSRPQKMLVGFQRVSLKPGETRTVRIPLEARRLAWWNAARGAYEVERVPVRLMLGESSADIRLTRTVEVR
>tr|D3R0E2|D3R0E2_MAGIU
MNYKKKPKNDREIQQAVQELLAKMTLSEKLGQMTQAVGAEISYIGSTGKGEPVEQRIREGKIGSMILVDEPTALAAKIKHFQKMAVEESRLGIPLLFAQDVIHGFETVF
PIPLAWSSSFDLELIQGAAEIAAKEAAACGINYVYSPMVDLVRDPRWGRVAESAGEDPFLGSAIAAALVRGYQGEKIGCTSHSVAACLKHFLGYGAAEAGRDYNTVDFS
PTTMFNHYLLPFKAGIDAGAASIMAAFNVVDGVPVTANYKLLTEILRNKLNFAGVVISDYNGVLELIAHGVAVNEKEAAAKALKAGLDIEMTTNFFTRFGEELCRASQA
ITDNVDRAVTRILTLKYQLGLMDDPFRSLNSPEKIKSTVFCREYLAYSRKLAENSVVLLKNDSILPIKAEQKVALIGPFAESKDLCGCWSFSTRKNETTDLKSGFELLG
WKVMTEAGCAVNEKINGGLQRAKLLAEKSDLVILALGENDVMSGEACSRMSISLPAVQQELAESLAEIGVPLVLCLMNGRPLLLNWYAEHCQAILQCYQLGSQAGLAIA
KILIGQVNPSGKLTMSIPYAQGQIPVYYNHLSTGRPLLDESSEEHFLSRYLDGPNHPLYPFGFGLSYTEFRMQEIELDKPTIAVNEEAEVAVTLANIGKYEGSEVVQLY
IRDPSASIARPVKELKGFKKISLLPGQKKQVNFKVNREMLSFYDSDGELRCEPGLFILTLATSSDDAHSLNINLEVVADEK
>tr|A0A0P7B566|A0A0P7B566_9HYPO
MIYYLLVLVLFTIASVCTADNSADGPLCSKALTKSATYLNPKAPRDKRVKDLLSRMCWDEKVTQLGGVGGILGQNNTLDVQDYRKKARLHNGTICKLFAPKTSLQRLTS
PGSYLNYAEDAVPVLSDLIVASKKEHRLGIPCVHIADAVNGPTFRGTTLFPSTLSMAMSWNLPLYTEVAEVLRDELMACGVNWVLSPEVDVARDPRNGRVGEMYGEDPW
MNGEYAAAYISTMQEEDSNGFMKVATTIKHYLYCTSTGGINQASMLGGVNHIYNTLALPYINVMKKVAPASVMPSYSTIDGIPAHSNSYLLQDILRQQFGYDGVIVSDA
DAIAMLYLTHKTAANLNDAGIKSLAAGVQLELAIGFPTAFESLTGNQAVEGVARNVNEAVSRILNLKFKLGLFDKTLEADAKALKSLRSKKHTKVNNTLLDAIKTEFGA
ENVHHVRGVDTIDTDDTEIDTAVAAAKNAGLAIVALGSIAVGWEDSLVGDRTDGEGNTHASLKLPGRQEELLEAITATGVPTILVLTGGQAFELHGAAQDAQAILHGFH
SGEETGRALVDIITGRVNPSGKLTITFPARSEVNPVYYNREQSDWQSAGAIQFPYLEKNYIYSFGYGLSFTKFEFSDARLDKPAYGPKDSVKVAVKVSNTGKVDGKEVV
QVYFKQKVAPLSLPVKRLVGFSKVLVSAGSSKTVEIIIPVQDLGYWLDGRYRVDVGTYEFFVGNSSADSSLSKALTAVVA
>tr|A0A1S3JUY6|A0A1S3JUY6_LINUN
MAVCGEAAVVFWFLFSSILPLIADLPFRNTSLSWEDRVNDLVDRLSIDELTLQMASGGRESSGQAPGIPRLGIHPYMWTQECLHGVVHARRSTSFPQSLGLAAAFSPEL
IYNVSRAIGMEVRAIHNNHTKYGQYGDDSGLSCFSPVVNIMRHPLWGRNQETYGEDPYLTSVYTDHYVRGLQGDHPRYVLASAGCKHFDAYSGPENSSLYDSEISERDW
HLTYLPAFKQCVKSGSLGFMCSYNKVNGIPSCVNSKMLDNILRQQWGYKGYVISDMGALEFIISVFSYYNNTVDTASASVMAGTNLELPHRLAQDGVFMSIPKAVSAGK
LTFDIVKEKAKPLFYTRMRLGEFDPPELNPYSKVDISVVQSLAHRELAVQAAVKSFVLLKNTDKFLPFQRTFESIAVIGPMANNTDQLFGDYSGNIDTSVALNPLQALR
TKALYTKFTAGCKDTGCEEYNNETILETVSKTQIIFLCLGTGTMVETEGKDRTDLQLPGNQPQLLKDVMQAANGIPIVLLLFNAGPLEISAASLNDRVPVILECFFPGQ
ATGEALRRVLWLEDPKANPAGRLPNSWPTYLSQVPSIYNYSMAGRTYRYFEGSLQYPFGYGLSYTTFSYTAIDVWPQTVLAGGSVSVTVGLTNTGMYDGDEVIQLYISW
PDTKEKMPRVQLVGFKRIFIKSLQEIKYKFTITADEMAVWNETFGFLVEPGIIQVYAGGQQPMQSSASNNILSASFKVVK
>tr|A0A1U7P1R4|A0A1U7P1R4_9DEIO
MKLFLLGAALLVASAGAQAVTGAQTVPRLPQAEAETRARTVLEGMTLDEKIGQVSMAHFFRFLEGGKSGPIAADAGDVFARLLPGATLNGGGDAPQPNTPRGWADALRG
LETLGRQNLPGGVPAVFGTDAVHGVNNVPGAILYPHNLGLGAAFNPALTQELARATAADMRAMNMDWDFSPVADLGRDPRWGRFYETFGESPWLVADQITASVTGLQSG
GVAATLKHFVAYGSPGLGRDRANAEISLRALHELYLPPFKAGIRAGAMSVMANSGSVNGVPVHASQNILTDLLRSELGFKGLVVSDWNDIDRLVNTFKTHADLVRATAA
SVNAGIDLYMVPNDVEKYEAALKEAVEGGLVSQERLDEAALRMLTFKAQLGLMDARPSGSGVITNGRALAKRAAAATLTLLENDGALPIKTGRVLVTGPAMDSAAIQLG
GWSVNWQGVGKGNVSYMPKVSTLATALKASAPAGVTVSALPDSKREALLKAAANADTIVVALGEAPAAESQANNPNLSLPSGQIGLLRDLLGTGKPVVLVLMAGRPILL
PEDLQNRLPAFVMAYLPGSEGGAALADALYGRAGFPGRLPFTWPGSLGEVGLTADRPPEGAGDAPLPLYPLGFGLDYTTFRTSDGQAARTADGVKISVTVTNTGEKAGT
ATFIVNASLPPAGNLEAVSRPVGVVRAELKKGEARTLSLDIPAERLEAFTGDAFRTVRGEVLPGQYTFKTGDELVRLSLP
>tr|D3EFJ4|D3EFJ4_GEOS4
MMHTDSTIQTKVEELLSQMTLAEKIGQMYQTDPGTVLRSYHSKIEVSTPMTGPVSERTLSKELISNLGSILSATDAETAYEVQKVFLEHNRLKIPLLFMFDIIHGFRTV
FPVPLGLASSWEPKLAEDTSRVAAAEGAASGINVTFAPMADLVRDPRWGRVMESPGEDPYLNGLMAAAMVRGFQGDDLKALDTVAACVKHFAAYGAAEGGRDYNTVDMS
EAALRNYYLPAYKAGIDAGAELIMTSFNVYDGVPATTNSFLLRKVLREEWGFEGVVISDYTSLWETIFHMSSKHGEDAAKQGLEAGLDIEMISTEYISHLEQLVERGEV
DVALIDEAVRRILTLKFKLGLFDDPYRYLNIEREKKAHLKPEYRQLAREAAQKSMVLLKNDDILPLKKDIKSVAVIGPFADNGRILGPWSGLGKPDEAVTVKQGLIKKL
GNAVKVTAAAGCDYSSNDVSAFEAALEAAASSEVVILAMGEEDHMSGEAGSRAYLTLPGVQSKLVEEVLKLGKPTVLVLFNGRPLELKWYHEHVPAILEAWFPGTEGGN
AVADLLFGDANPSAKLTMSFPYTVGQVPVYYNCLNTGRPKGHEDNDFRFLSKYLDIPNAPFYPFGYGLSYTEYTYSDAALIGDTLTPGGCVKAKVTVQNTGNYAGEEIV
QMYVRDLWGSTSRPLLELKGFKKIHLAPGEKATVEFVIEPEMLSYFTAKKQYEVEAGDFKVFVGKNARDLIECGTFAYSV
>tr|E7G5H3|E7G5H3_9FIRM
MKQEQLDELLESLTLDEKIGQLVQLNGNFFDSNEKVATGPVQKIGIDKDKIHLTGSILNTLGAKELKTLQNAYLEKSEKKIPMLFMADIINGFKTIFPIPLGLGCSFDA
ELVKKTAQIAAKEAAVSGIHITFSPMVDMVRDARWGRVMESYGEDNFLNCEYAKAMVEGYQGNNDPHSTIASCVKHFAGYGAPLAGKEYNTVELGERSLREAYLPAYKA
AIDAGCHLVMTSFNTIDGIPVTANKWILKDVLRDEWGFDEVVISDHSAVKELVPHGIAAHYEEAAKLAIEAGCDIDMMTATYSNHLKDLVESGQIDIQLINESVKRVLK
LKNDLGLFENPYRGANELEEQQYVMCDEFRKVAREVVSKTCVLLKNDSVLPINQNQRVAVVGPYASNKNLSGMWSINVDPDKVVTIYDGLKKQGKHVKCAEGYPMMDDD
SLFESFGYGKKETNLTGNKDEKIQEAIDIAKESDVVIVAIGEHSHQSGEGGARGDITIPKVQLELLKAIKKLGKPVVTLVFSGRPLALKDVAENSDAILQCWFPGTEGG
DGIADLVYGKVNPSARLSMTFPYSVGQCPIYYNHMSTGRPALESTHSKRFTSRYIDIPNDPYYCFGYGLSYSEFEYSDITLDKTTLTNDSTITASVTVSNTSDISGEEV
VQLYIRDIAGSVVRPVKELKGVQKVYLAPHTSQTVTFEIKEEMLRFITKDMSFASEPGEFHIFIGHDSNTQNKQIFTLES
>tr|A0A0B2A325|A0A0B2A325_9MICO
MSIDIEELLGRMTWPQKLLQLQIVWRQDAAERDALVRRGLGSTFWPPSAAEADALQRIAVEETELGIPLLIGLDVVHGQFTIFPTPLAQAASFDPQTAVDDARVSAIEA
RSHGVNWTFSPMVDVTRDPRWGRVVEGFGEDVHLSSVFTAAKVRGYQGDGPAAGDSVAACLKHFVAYGAAEAGRDYNTADVSDRRLRETYLETFRIGVEAGAVTVMASF
NSLNGHPMHGNHALLTGVLKREWGFDGVVVGDADGVAQLVDHGVAPDERAAVALALESGIDIVMGGTLLVDGAGIPLLTPADVSPERIDDAVRRVLRLKDRLGLFAAPS
ADPASAVGTTPAHRELARRAAERCPVLLTNGGSLPLPATGRILLAGPYARSLDHLGAWVQHFATPADQSLAEALARRLPDVAWDVADGPDFFTVSDGQIRDLVARADAA
DLVVLALGEPSRLSGEAASRADITLPDAQRRVIHALADAGARVVVVLATGRPLVVEDWVDRVDAVLCTWHLGVEGPAAIAATLSGAVNPGGRVPMTFPRAVGQVPIHHD
HERTGRPPRTGGGLVGSPASFVDPVTGFAGPNNTDDHYTSKFLDLPLGPRFDFGHGLSYTTFALDDLTVSADAITIGELREGSAEVAVTVRNTGDRAGDDVLMLFVTDE
VASVAQPVRRLRGFRRIPLAAGGSARVAFRLDATDLEFWADGSRRLLEPGDFTLTVSDGTSSLTTRLELTACARSEASA
>tr|A0A0P6VM49|A0A0P6VM49_9HYPH
MTRTLSAVSAAVAALVAVALPAAADPAIEARIDALIARMTLAEKIGQLTVESHGPFGTRERIATGEIGNVINYANAGETGDVQAEAAKSRLGIPLLTGLDVLHGYRTLF
MVPLGQAASFDPELNRRAAEAIARETTLQGVNWTYAPMVDIGRDPRWGRVVEGAGEDPWLAARMAAARVTGYRAGGLVVTPKHFVGYGAPAAGLDYAPADMSEATLRDV
YLPPFRAAVEAGAQAIMPALSALNGIPASVDSRLLTGILRGEWGFRGFVVSDWGAIDQLGNHGIDPDRAVMARKTFAAGVDMDMASGTYRTRFPEEVAAGRVSEAAIDA
AVRRVLRVKFEAGLDTRKPIPWKEAEAAMLAPATRDLAVEVARRSIVLLENRRETVPIEGRRKVLLVGALADIAREHVGPHAAQYREQDVVTIRRALAERAARAGIAFD
FQAGCDAFCTDERGFGAAVKAARAADVIVAVLGEPGDIAGEAASRADPNLPGRQQALLERLVATGKPVVLVVMAGRPLIVPWAAEHVSALLTTWYPGTYGSRALAEILF
GDTAPSGRLPMTWPRHPGQIPITYDRLRSGRPAERDQKFTSKYVDVDWKPQFAFGHGLSTTRFAYDGLRLDRTEVATDGRLTATVTVTNAGRRAGREVVQLYVRDVQSS
RVRPDRQLKGVASVDLAPGEAKTVSIPIDVADLGFHDETGTYRVEAGRFEVFVGGSSEATLSAAFTATGPDLVRPPGGR
>tr|A0A1I5UPQ6|A0A1I5UPQ6_9FIRM
MSDNAKQNENGGIPIYLDPDQPLEKRVDDLVSRLTLEEKVSQMIYASSAIPRLGIPEYNWWNECLHGVARAGIATVFPQAIGMAASFNDKLLYRVACAISDEARAKHHE
FVRQGDRGIYKGLTFWSPNINIFRDPRWGRGQETYGEDPYLTGRMGVAFVKGIQGDHPKYLKAIATPKHYAVHSGPEPLRHSFDARVSQKDLRETYLPAFKECVKEGKA
ASIMGAYNRTNGEPCCASPTLLQKILRDEWGFDGYVVSDCGAIYDIHAHHKVTNTPEESAALAVNAGCDLNCGRVFESLVNAVKQGLVSEETIDRAVKRLFKARFKLGM
FDPPERVPYAQIPYEVNDCEEHRRLALEMARESMVLLKNEGGLLPLRKDLKAIAVIGPNADNKKVLLGNYNGTPSKYVTALEGIRAKVSPQTKVYYAEGCDLTSTQSSY
WGQEATAGFAEALAAAQRADVVIMCLGLSPELEGEEGDAARSTAGGDKTSLDLPGMQEELLKAVCATGKPVVLVLFSGSPVSINWAQENVPAILQAWYPGEEGGTAIAD
VLFGDYNPGGRLPVTFVKSLDQVPPFTDYSMKGRTYRYMEDEPLYPFGYGLSYTTFEYSNLKLSAEVIEAGQSITISVDVKNTGNMAGDEVVQLYLKDLEASVDVPIHE
LKGFSRIKLQPGETTTVTFTLTPRQMALIDNDGRCILEPGRFRVMVGGRQPDKRSEALAKTPILVGEFEVRGEPMELEY
>tr|A0A1J4K0C2|A0A1J4K0C2_9EUKA
MSAKQYRTEAHTDEEIRVAVEELLAKMTLKEKLGQMTQSPGLNVSAIGAEVKGNSLYDLIRNGDIGSQILIHPVNHMIENAKLHQKIAVEESRLGIPLIFCMDVIHGYQ
TIFPIPLAWSCSWNPEQIKKAQQIAAKEAATVGISLAFSPMVDIAHDARWGRVAESAGEDPFLGSAIARAHVEGLQGNSLADPDTVCACVKHYLGYAAAEAGRDYNTCE
FSETALRNTYLPPFKAAIDAGCGSLMTAFNTINGVPAVGNKWLCKTLLRDELKYDGVVISDYAAVDELIVHGNAEDGKEAAKLSVDATLDIEMSTVNILNNGEQLLKEG
EITLDQIDDAVRRILTYKYKIGLMENPYIYMKEDKLKETIQCQAHLDASRDLARRSIVLLKNEENALPLKKSQKVAVIGPFGESKDLLGTWQFSDFKDVPTIVDGLKAK

GIEVISEKGCAIHEKIENGFESALEAAKQADVIILALGESSDMNGEAACRLDISVPAVQFELSQLIVKEVRKPVILVLTNGRPLLLKWYQENVTAIVEAWNLGSMTGHA
VADVLVGDYNPSGKLTLTFPQFLGQVPLYYNHYNTGRPCIDHDPQKFVSKYLDGWNDPLYTFGYGLSYTKFEFKNLKLSQDKINFHETLKVSVDVQNVGDVAGEETVQL
YIHDVTGSIVRPVKELKGFKKIVLEPTASTTVEFEINEKLLRFYNITNQLVAEPGKFEVFVGNSSDDKDLLKSEFRLTK
>tr|A0A2K9E9P1|A0A2K9E9P1_9FIRM
MSAIYTDPSKSPKERTKDLISKMTLEEKIGQMCQIDGNENAEFWIKERNIGSFLHVRGEKAINLQKMATEETRLGIPLLFGIDAIHGHAFHSGATVFPSQLALSSSWNP
QLVEKVGNITAKEVSVTGLHWTFSPVICLARDIRWGRVDETFGEDPYLAGKLAAAMIRGYQGEDLSHPESILACAKHFIAYGETQGARDASESDVSERKLRSIFLPPFK
DAVDAGCATFMVAYQAIDGVPCSANKWLLKDVLKDELGFEGIVITDWDNVGHMHNLQKIASTMKESCEIAIKAGNDMIMSTPDFFENAVGLVKEGVIPESLIDEACSRI
LEIKFKLGLFDHKRYPDSDKYKIYMGCSEHIQASYESALESIVLLKNNDNILPISDKIKKIAVIGPNADDVQAQLGDWSFGPRYHPEIPTFDYHPDYDISKIVTILEGI
KKRAGEDFEVYYEKGCDVIDPEIENIKCAVKIAEKSDIVIAVVGDTIVLNGETRDRSNLDLTGAQEKLLKALKDTGKPLVVVLINGKPLTIPWIKENADAIVEAWNPGM
EGGNAVASILFGDYNPCGKLSISFPKTVGQQPVYYNQLPGWHGGKYADVDAEPLFNFGYGLSYTTFEYSDLKASKPVIDCDENLIVSVQVKNTGKMAGSEIVQLYINDI
YTSVTTPVKELKEFKKVFLNPGETKTVEFELTFMSFSFVNANCRRCVEPGEFEIMVGSSSKDEDLLKIVVEYKGKVKEF
>tr|A0A4Q7P3A6|A0A4Q7P3A6_9FIRM
MTNSELKNLLDSLSLEDKIGQLVQLTGECFAGGEIQTGPAEELGIPEDMVQRTGSILNVTGAEKLKGVQKRYLEQNPSGIPMLFMADVINGFKTVFPIPLGQGCSFDPG
LVEEAAAVSARESSAAGLHVTFSPMVDLVRDARWGRVMESTGEDVCLNGVMGAAMVRGYQGKSLSEPGTIAACVKHFAAYGAPEAGREYNMVDLSEKRLREEYLQPYHA
AAAEAGAALVMTSFNTVNGVPATGNRWLLDEILRKEWNYKGVVISDYSAIAELIVHGVVADGREASRLALECGVDIDMVTDMYAGHLQDMVESGVISEELLDASVMRVL
ELKNELGLFENPYRFADESKEKELGHCQAHRETARKLAEESCVLLENDGILPLKKEGQKIALIGPAADSAEICGSWSLFWNSADVTTLRTGMEEKTQLSACVKGCDLLD
AEPVYIGFRGQELRGRKDQSDLIAEAVEAAKKADVVVMTLGESPQQSGEGAARAFLDLPECQKKLFDAVYEVNSRIAVLVFSGRPLDLRYIVNRAAALMLVWQPGTEGG
AALANLLFGDAVPSGKLSMSVPYCVGQVPVYYARYNTGRPAKKGTRNRFLSAYQDIPNEPLYPFGYGKSYTEFSYGKPVADRGEVGTGETVTVVSEVQNTGMTAGTEVV
QCYVQDLVSSTSRPVRELKAFARVFLRPGEKKQVAFTLDEKDFSFLRADKTWGPERGRFRVFVGGDSNAEQETEIYYCG
>tr|E0IBJ9|E0IBJ9_9BACL
MSVSVMAAAPSKKQWITTEIREQAEQLLSKMTLEDKVGQMTQFDWGYNPINPETGESEHDLIIELIRQGKVGSIFNLSGAAEANELQGLIEQHTELKIPMVIGRDVIHG
YRTVFPIPLAMAAAWNPEVARQTSAAASTEALTDGVTWVFAPMIDVSRDPRWGRIAESIGEDPYLTAAYGRAWVEGSQIDNGPGRATASCPKHFAGYGMAEAGRDYNTV
DLSDRELRDIILPPFQDAVEAGALSIMASFNEINGIPACANEYLLKTILRDEWGFEGVVASDYNALVELIVHGVAANEEEACEMTVLAGCDMDMHSGIFTRQLPKLVRA
GRVPESVVDDSVRRILAMKIKLGLLEQSKSDVSQSAATQPLKSEYVELAREAARQSIVLLQNKEQVLPLSKAGASIAVIGPLADNATDPLGCWALDGRSDEVVTALEGI
RQAAAEGTSIRYAQGCDIDSDSEEGFEAALEAARSSDVVVMLLGESATMSGESRSRAALDLPGKQRALVEAVAKLGKPIVAVILSGRPLTFAWLPEQASAIVQAWHLGV
QSGNAIADVLFGDFNPSGRLPVTFPQNVGQIPIYHYRKKTGRPPAGAYSSYYIDSTTEPLYPFGYGLTYTEFEYGAIQTSKSSIGADEQLDVTVSIRNVGNLAGEEVVQ
CYVRDEVASVTQPLKRLVAFRKVKVAAGESVDVTFTIGAAELAILDKHMKRTVEPGDFTLWIGPSAGSGESVSITVEGR
>tr|A0A0R2BYY5|A0A0R2BYY5_9LACO
MNQDKLADLFHNLSWAEKIGQLVQLSGDFFGAKNQSIGPAKELGISPTAVKLVGSVLNVAGTQETVRIQKKHLSQHPHQIPLLFMADILYGYKTIFPSPLGLGATWNPT
LIQQAFQAIGQEAQTGGINVAFGPMVDLVRDARWGRVMESTGEDVYLNSQFARSMVTGLQHGGVAACIKHFAGYGAVEGGREYNSTNLSPVELYQHYLPAYRAGVKAGA
YLVMTSLTALNGRPCTANEWLLKKILREKWGFKGVIISDFNSIKELIAHGLAADEATAAKLALQAGVDIDMKSPCYAKQLEPLVKNQQLDPQLIDQAAWRVLCLKNKLG
LFEDPYRHLQAATEPAEVLSPDKQTLARKVAQAAVVMLKNEGDILPLHSQQRVAVIGPYADNRMALGMWAVHADYQTTSTLKEGLEKYFATANLKIAPGTPITLNETQL
LAKLGIKLPSNLTSKKLEQMSTQAYKQAAADVIIFAGGESSLQSGEAGSRTDLHLPANQLKLLQKLAKLDKPIILINYSGRPLVLTKLPVQVKAILQVWYPGTRGGQA
IADILVGQVNPSGRLSISFPAAVGQCPIYYNHLATGRPKEVTGDTRFTSGYLDAPAEPLYPFGYGLSYGQVVYHQPQIDRTTLTAGQQLRLTIKVTNNSKKFARLETVQ
LYLHDIAASIVQPVKQLIDFRQVALKAQETVQLIFTINEQQLGFYDETGERISEPGDFELMVGPNSAATQKVRFTFLK
>tr|D9STW6|D9STW6_CLOC7
MLSNNKDIEESNFIDSLIGKMTIREKLGQMYQICIEANNENVAFNLEDDRSLVKKGFAGSVINLVDRNLIQEYQKIAVEESRLGIPLIFARDIIHGFNLIFPIPLAQAA
SWNLELAEKAAAITAKEATICGIRWTFSPMIDVSRNPKWGRVAEGYGEDAFLVSSFAQAVIRGYQGENLSDPSSLAACSKHFVGYSATEDGRDYNVTPIPPRELNDVYL
PPFLSSVKAGVATIMAGFNDLDGIPMSGNKELLKKLLRDEWNFDGVVVSDFNSITEMIFQGFCQDGKEAALKSIESEIDIEMVSLNYMNFIEKLIEEGYIDTELVNKCV
RRILNLKHKLGLFKNPYSNNNFTEINSDFDNNVALELAEQSLILLKNNASFLPLKDDVQKITVIGPLADSKIDQMGCWSMDGDIDRVVTFYESIKSYCLEQNIELYYDS
ILPDCRVSFETIDDVTVEHIRRSDVVIVVVGEDSLMNGEKHCRAYLSLPLGQSKMLEKIAEINKNVVTVVYAGRPLIIKDILSNSTSVLYAWHPGSMGGKAVTNILFGK
SSPSGRLPISFPRAEGQIPIYYSHKNYGRPPLERPISVNDGDFHNSIGWTSEYLDINHKPLFPFGYGMSYTKFEYSEIKLNKIILDIDDSLEVYINVKNVGSVDSYEVI
QVYITDVVGSVTRPQKELKAFEKVFIKQEEIVQLKFSIPVKSFKFTDINGDFVVEPGQFKVWVGSNSEECLNMEFYVK
>tr|R6B9T8|R6B9T8_9FIRM
MERDSRKRAEALLAQMTATEKVGQLNQHLYGFRIYHVEDGKIIFTQELKDEVKKWGGIGTIYGLYRADPWSEKNYETGLAGGLSMQAYNQLQKYVIEHSRLGIPFLLSS
ECPHGHQALDGYLLPVNLAVGASFDPALYERAGQVCGRQLKQMGVDFALVSALDILRDPRWGRSEECYGEDPYLSAELARAIVTGIQKEGVAVVAKHFCAQGETTGGVN
ASAARIGERELWEIHLQAAKACCEAGVKGIMAAYNEIDGKFCHANRHLLQDILREQLGFDGVVMADGIAIDQLDIMTGDNIRSAALALKAGVDISLWDEGYTKLEEALK
QGFITEKELDQAVLRVLTLKFEQGLMDKPYIDENGNRTASYSENGAVSFPTEAYQESEKLARESVVLLKNENVLPIGRAEKIALIGPNADDIYRQIGDYSPPMDRAGYE
TLKSGMEKEFGADRVRCYNGHEVGTAVSLAENADIIVLALGGSSSRFKGALFDENGAAKVQGVLEMDCGEGMDTARLQLPGNQNELFTAVCALKKPVISVVIAGRPYAI
PEIAQDSDALLYAFYPGPMGGKAIAELLSGKYAPSGRLPVSLPRNCGQLPVYYNHTVSYPAMHYCDMEQGVLYTFGEGMGYSHMEYVDICLKKQQDVWIISGIVKNKGN
IDDTAVLQCYRKVLSGELVPRERELVAFSRIKVEKGSHKAFEIVMDADKFRYFNESGAKIYKSKILLMDSGKIIFEE
>tr|R7UZ23|R7UZ23_CAPTE
MRGVLICCLAVLCGVANAKFPFEDVTLSWDKRVDDLVQRLTIEEVVNISVAQYGKSTIPVDRLGVKPYQFINECITGVRWENSTAFPQAIGLGASFSPDLAFNMSQAIA
RELRGFYNTEVKSQIYGHRGVNCFTPVINIMRHPLWGRNQETYGEDPWLSGQLSVGFVKGLQGDHPRYIQASGGCKHFDVHNGPENIPVSRFGFDAKVSERDWRMTFLP
QFKTCVEAGSINIMCSYNRINGVPACANKKLLTDILRKEWGFNGYVISDSGAIENIVYHHKYTKTLAEAAADSVKAGCNVELTGATGSGVAYFNLLNAVKQNLISEEEL
RENLKKPMYSRMRQGEFDPVDMNPFTKIDMSVVLSQEHQDLAVKASAMSFVLMKNLNRVLPLKKRFDRLAIIGPFADNAETLFGDYIPNWDPKFVSTPYEGLKSLGDDV
RYASGCDDPSCTNYDPKAIEKAVKGAQFVFVCLGVGSNLEREGHDRADLDLPGYQLQILKDAEFFSREAPLVLVLFNAGPVDLTWPKLSPEVDGIIECFYPAMGTGKAL
YQVVTATGDDGVPAARLPSTWPAQLHQVPSITDYNMTGHTYRYFDGGDPLYPFGYGLSYTSFHYQTVSVSPTSVRAGGNVTVTVQVLNRGPYNADEVTQVYMSWMEATV
PVPRWTLVGFKRHRHTVNQSSSLSFVVSAEQMAVWVDEATGFQVQPGKMLIYAGGQQPYQRTDTDTNVIGTSFLVTA
>tr|A0A2K4ZN91|A0A2K4ZN91_9FIRM
MNRDNLLVKQPFDPGCYTEQVNQLLERMTVREKVGQLNLDHVSDSGDLTDCNLGERSSMEAEAEKVRRGLVGTMLMKGREEANYFQKVAVEESRLGIPLLFGFDVIHGH
KTVFPVPLGGASTWDPQMLEEAEEYAAREAYADGINWIYAPMIDLCRDPRWGRVAEGAGEDPLLGSLIAQAKVRGLQTVNPETGYPYVAACFKHFCGYGLSQGGRDYEE
CETSPRTLFMDYMKPYQAAVDAGALSTMSAFSVLNGQAVTGSRYYLTEVLRKQFGFRGFVCSDYGAVKELVHHRVAADRKEAAAQALLAGVDEDMGSHAFADYCEALYE
EDQRFAEAVDEAVRRILSVKFAVGLFEFPYFPEEAEKYMLAPEAREAARNVGRHSIVLLKNQEKILPLSPSKKYFLTGPLSDNVEEMPGAWAQYHPGTNVISVKMALEE
AGLDFLQSDGCPFTGKYCFDSQDDSGFAKALKMAEECDEIIYVCGEKAAWSGENRGRVSIDLPKQQYRYLRELKKTGRKIISVLMCGRAMSCQELAESSDALVLAWHLG
TEAGHSIADVLLGSYSPSGRLPITFPCYTGQVPYYHSWLSSGRSRESLIRYKDGENGPLYPFGYGLSYAEITYDAVHISRNRWKADETIRLEVVLTNHSEMDAYEVVQA
YYQDVVSSLPTPDRKLCGFTKILVPAGSTVKAQMLIPVERLALMTLDLKEVVEPGEFILFVGRDSMCGESARIVVE
>tr|A0A1R0H2K1|A0A1R0H2K1_9FUNG
MRYSLFFYLVVSADLNLTTPQPSLLNKTKAQQPKIDVVGVRTLEGVTCLPNFFPLQDLDPMMPKSVDSYQIPQFDESGYIGPSSNIDSDVLELLASLSIEDKVGQMVQV
PMWNMVGCDGLINMTYENLMVEKYRVGSVLDSPSNNAGRWNLNSPQRFANFTNTFQHIAITKGSKIPIVNGIDTLRGAGFVKGATTFTAPVNIGATFNPIHSYNAGRVG
AKDTRSIGVHWTFGPLADINVQKLWSRNYESFGEDPYLIGEMMHAGIKGVQGNYKKDRTRIASTFKHFIGYSGPLNGRDEEDRYIPYNHLLEYLAPPFQRAIDVGSATG
MEAYGAVNGQDTIASKRLLKLKKDLGLFENPYSDLSLIDTVGSAQDIELARDGARESLILLKNENNALPLSPGENVLFVGPSFNSSSYITGAWSIRMQGSNEYEKDTPY
GGYCDTILEGIEGIIGRTPNYIQGYTIEGDRVSGYDTIVRQARKADKVVFLFGEKPGTETAAIIKTLRMAEDQYNIAKRVIEETITSVILILLQNHPYLLGEPSSISDG
IINANLPGAYGGLPVAEALYGKFSPSGRQPYSYPKMDYQAPVVYYTPIWNEYDPEFAFGQGMGYNNITYSNITVSSVELRPGKPITVSVTAFNNGNMNQLEPVLMFTTQ
NIRREYSPERYRLRAFNKQPIGPGASRIFSFNLTAEEMMFYNIDNNRILSEGPVDITINAFNVNAVAKSIYLYEN
>tr|A0A559QQJ4|A0A559QQJ4_9ALTE
MNGLQSPLYKNPEAPLAARVEDLLMRMTLPEKLGQMMQLPAVDENAEEYIEKYHVGSYLHALDGDITRLKQLNTERSRLGIPLIFGIDAIHGHCFEDGSTVFPVQLALA
CSWDTALLTEVGKITAKEAYGAGIDWTFSPVLCMARDPRWGRTSETFGEDSMLIGELAAAMVDGYQNAGVPFAACAKHYAAYGETLGARDCGDAHVSERNMRNIFLPPF
EKVAALGCKTMMAAYQSLNGVPCSANTWLMNTVLRDEWRYEGVVVTDWNNCGQVHTVQGAAQSMKDAVELCLAASNDIFMTTPTFFECAQQLIAEGAISETRIDESVRR
VLALKFSLGLFDDSEPRSKQQQWRDDRWDVSLQASRQSLTLVKNGGILPLRAKGRQKILLVGDNADNVLSQLGDWSFIPGMHAYSDSITHRSSVITLRQALEADENISL
TFMGSEVCGPLTDTQADKVGLLAAAQSADLVIFCGGDALKQHGEFHDRAELDLPGNQNPVFDVLAGSGVPLISVLIMSKPHAINSVMEHSDAVLIAFNPGAKAGCAIRE
CLFGEFNPSGRLPMSFPRSVGQLPVYYNQAPGWHAYQSPHYDGQNHYIDCDATPLLAFGEGMSYSEIVYGEVQLDQSEVRSNESARLTVQLGNNSDRSAVEVVQLYAQW
RIKGVTSPAKNLIQYQRVELEAGEQQAVTFLIEADAIRVRDRDLNLVHYQGELVLYVGHSSKHDDLQVATLQIVP
>tr|A0A2H3IKM7|A0A2H3IKM7_9EURO

MYGQDGMTYKNSSYSALERAKDLLQRMTWEEKIGLMGGIRVLLGANLTFNETTYNSIHELQNGILGFRYNLNNALDVLPIANKIRAQETSESLLEIPYITVTDSVNSIY
LPGGTLFPSTLSMSAWWNIPLYEQVISAIRDENVATGVRWVLSPELDVARELRNGRVGEMNLTFELGGEKKGKVVLINFHTATTIKHFVYASPTGGINTASQNGGLNHL
FNDLIQPFVTVIKDSQPASVMISYASIDRGPMSANRFMMRTILREKLGFDGVLMSDAEAILHLYTQSKVVKSLENAALRALKAGLQLELSPAQPAAFPTLLSSINSTLV
RERIDDAVLKILVIKFQTGLFDEGLPQVADAKAVLKSDAHLAINHNMSRESIVLLQNDGILPLRKGINGSWPKTAVIGPFADIINPGSYVPNNSIDRSFGKSLYQSMAT
AFGSDRVSYTQGVDIIGNDTSGIKAAVQAAKEAELAVLMLGSLSVVSANPLFDKRTDDLLNEILATGVPTVLILSGGQAFVLDDQTRQSNSILHSFLGGEYTADSLVEI
LIGDVNPSNKLTISVPQSTGAIPIYYNYLPSDNQGGPGVFASGNFSTAWQFPELPRTPPTMAFEYGLGYTTFECSNAQTSSGDSVQVTVDVQNTGNVTGKEVMQLYFRL
GFSVTEFPVKKLIRFEKVELAPGESITVNFTVPTCDLGYYRNMEWEVETGAYNFWVGSSSLDHDLISLNTTVL
>tr|A0A4R5DDH3|A0A4R5DDH3_9ACTN
MSDLPLYRDPAAKPAERVDDLLARMTPAEKGQQLQMLDRSWGDGESFLRDGRFDPATAAANLGPDGHGSLSQRQDDASPHAYAESSNAVQRYLAEHTRLGIPALFIGET
LHGLVGPAGTTVFPQAIGLAASWNPELLEQVGLATAREARAVGTHVGLSPVLDVCRDPRWGRLEETYGEDPFLVSRLGVAMVRGLQGGDGAGPGLVATVKHFAGHGAPQ
GGRDSAPSGHGREFLREFALPPFEAAVREAGAGGVMAAYSDWCYVPCNASHELLTEILRDEWGFGGFVIEDMGAIGFLHTAHAVAADTADAAVQALRAGVDQSFASALG
PPVVDAVRAGVLDEEVLDRAVRRVLRTKVELGLFEDPYVDATTADAVCDSPEHRALAREAARQSVVLLQNDGTLPLRADLGTIAVIGPNAGVAETGDYSGRNPDLVTPL
AGIGARAGTGTAVLYTEGCPVVDQPPDDAGIAAAARIAARADVAVVVVGGSAATSGEIRDAGELDLTGRQDELIRAVHATGTPVVLVHIGGRPNTMEWAFDHVAAAVAA
WNPGEEGGSALADVLFGDVAPSGKLPVQWPAATAQLPSTYDYMHTGRDERGRYVNTPVAPRFPFGHGLSYTTFEYGEVGVEVGQDVTVTVEVTNTGDRDGVEVAQLYLR
DLVASLKRPMRQLKAFRRVTLRAGEARRVVFTLSRDDLAFWNAAGERVVEPGEFAVMVGGDSTTTNEARFDLP
>tr|A0A380PMR6|A0A380PMR6_YERPE
MSTVYRDATLPIEARIDDLLSQMTLAEKVGQLCQQPMLDYQQHRDDYLAGVRAGRWGSRILADTAWAGNAPGENVDPCQLNEIQRAAVEHSRLGIPLLFARDVIYGQAT
VLPIPLAQAASWNPELVQQAYRVIAREAASLGIHWTFAPMLDIARDPRWGRTIETSGEDPWLTAQFAAAVVKGFQGDDLSAADSLMACAKHFVGYAAVEGGRDYDTTEL
SDNTLHNVHLPPFQAAIEAGVGSVMTGFCDFGGTPVTAHGELIRGWLKQQQNFDGLVISDWGSIADLTHFGIAQDALRAAELALQAGVDMAMTHEAYEDKLDQLVLQGR
IKEALLDDAVRRVLRAKFRCGLFERPYVDEEWHKRELRRPEHLALAQRLAEQSIVLLKNRQALLPISRTSVPLTLAVIGPHAHSQRQHLGSWCLDGDADQVMSIYQSLC
AIAGEVKVITEQSCFSDEMIECAHRADIVILCTGESHRRTGEARNIAELQLPPGQEELIAAVGRTGKPLVVIQCTGRPLPSPATEQYADALLYGWQCGSEAGKAIARII
FGEQVPCGKLPMTVPRSTGQIPIYYGRKPLGKMRDYREYQPYKDLADTPLYPFGFGLSYTRFTYSDLQLSHESLELGASLRVTMTVTNDGAVAGDEVVQCYIRQYVAST
TRPQRELKGFQRISLQPGELGEVSFTLGVRELAFYGQHRRYAQEPSRISVFVGGDSTTPHQADFTLFENLYTV
>tr|V4IV34|V4IV34_9EURY
MVTRRFDHQVEELLERMTLEEKVGQMVGTPPHEDVAEMESEIRDHHVGSVHFGGTPHNTPEAKAEVANAAQRVAVEESRLGVPVFLRAMAEHGHAAVAGSTVFPQQLGM
AATRNPDLAREAASVAATEMRATGVQSTSSPIGDVARDQRWGRIAETFGESPYLSARMTAAMVEGYQGDDPSDPESVLAVTKHFPMYSEGVRGEDTAPNEVSTYTMRRV
HVPPYQAGIDAGTGGVMPCYNSINGEPVHGSKRILTGLLREELGFEGFVLADYRGAEDLHRAHDTTADLEQSIWQSVSAGMDLLPSGGSTYTDAIVNLVESGELSERRV
EESARRVLRAKFELGLFDDPYVDVDEATRTLGSDDHREIARRVARETMTLLKNDDDVLPLSPDLGEVLVTGPNADDIAHQHGGWGNVRDPDPMGDTVLDGIEAAVGDDT
TVTHEPGSGINEAGDVDAAADAAADADAAVVVLGEPDYVHEFSRSTLGDTADEFPKRTQLTLPDAQLDLAKAVHETGTPTVVVFVTGRVLATPWVAEHVPGVLMAYQPG
SEGGAVADVLFGAYNPHGKLPISVPRSEGQVPVRFNYLPHPRHHGAEESHVDSYDPLFAYGHGLSYTEFAYDDLTLSAETVGPGDTVDAEVTVTNAGERAGVETVEVFV
RDVLSSRVTPVRELAGFARVELDAGETDVATVPLAVEDAGVIGDDGDRTPEAGTHRVFAGDLSAEFDVERRYS
>tr|A0A428J701|A0A428J701_9BACI
MRKISIILLVFLVLSACTNNSSPQINDEIDQLISDMTIEEKVGQLAQIDIHFLMKNTWSKGGLDKNKLKTVIVDYGVGSILSGGGGSPDINSPKEWAEMVNEVQKMTEQ
TRLNIPVLYGVDAVHGHNNVKGAVIYPHNLAVASTFNVQLAEKEAALTSEELAATGINWNFAPVLDVARDPRWGRTYETFGEDPYLVSMMGKHMINGIEASGKVAATAK
HFIAYSGTNNGQDRQPADISERTLREIYLPPFEAAFNAGVDTVMVNSAEVNGVPVHASKGLLTDLLRDELHFNGVILSDWEDVHKLTDYHQVSRNYKQANALAFEAGID
MSMIPMTLEDIDLLIELVTEGAISEERLDESVQRILTLKKELGLFDERYVIADEAADVFKRQETFETARELARQSIILLKNDDILPLPKDVKSILVVGPTANSISRLAG
GWTIGWQGAEEHDLTTGSTVLDEIKKKVSPQTNISILTGEEERDEIMAVAKDVDVVIAVVGEKPYAEFEGNTTSLALPDGQSELLATLQSVNENVVMVTVSGRPLAIEQ
EASMVKGVLWTFLPGTEGGMAIADVLFGDYNPSGKLPITIPKHEGQLPTYYNDRVTATYEPLFSFGDGLSYTTFSYKDLKVSDLMNNDKTLKVSIIVKNTGAVAGDEVV
LVYAQDFYQGVTPRHKQLTAFERVSLQPNEEKEVQFEIPFNQLKKYDQELQRTLPNQLFIKIDHLREEVTLR
>tr|A0A0S7BI70|A0A0S7BI70_9CHLR
MASPASSIIKKEAQNLPREEFCSNLLSQMTLKEKVGQMVQADLSWSENVKQLLREGRIGSLLTIQDVRSINEHQHIAVEESRLGIPILVGNDIIHGYRSIFPIPLALAS
SWDVDLVEAVAHASISEAIAAGTTWNFAPMVDITRDPRWGRIAESAGEDPLLNSKIGAAWVRGYQGYRSETGRQAAACVKHYAAYGAVESGKDYNTTDMSERRLREEYL
PPYKAAIDAGVKTIMTSFNDLNGLPATINPLLLKQILRKEWGFEGVIISDYDSIGELIFHGVARDHKEAALRSILAGVDIDMMGNAYHYHLADLVRDGQVPEALLDEAV
LRILRLKYDLDLFDHPYAEESRISSSLMRPETLNLAAEAAAKSIILLKNDPRLLPLHLEGKTIALIGPFADERQSLLGCWSFDGKAGDVETLHEAIERNLPPRASLRVC
SGCSFDGDAADLASAVAVARQADVVLLAVGETDRMSGEAHSRAHLGLPGRQQELVDAVVATGKPVIAAVFTGRPLAILKLAQEVPSILLAWHGGTRAAQGLCDVLLGRV
NPSAKLNVSFPRSEGQIPVYYAHKSTGRPAESSGVIQFNEADKSVYLDESNDPLFAFGFGLSYTNFTYSDLRVLTPVVEKDGFLRVSATLTNAGNFTGTEIVQLYVRDL
VGTVTRPVKELKDFQKMTLAPGESCSVEFDLPAGELAFLNVDLNPVLEAGEFKVWIGPDSTRGLEGHFAVR
>tr|A0A1B9GJE5|A0A1B9GJE5_9TREE
MHIASLLVPAIALASLASGQYVVPAADGSDGWADALGKAKALVAQMTQEEKLNITQQNNDFPRVGEIDRLGYTGLFYADGAEGIRGASYSSAFPQALNGAATWDRNLLY
RRAVAIGQEARAKGINVQFGPGVNLMRTPNAGRGFEYNGADPYLAGQAGAQHVQGVQSQGVMSTVRHYIGNDQETGRRWTSSNMGDRTAHELYLWPFQDAVKAGVASTM
CSYNGLNGTYSCADPMSLGKWLHEELKFQGWVLSDFGAITFGEEIDAANAGVDTTRVADPPSAPFPFGPGSALSAAVANNSVSQDRLDDMVHRIVSAWYKLGQDQDYPA
YNTSVNSLSKEHNELIREIGAKSIVLLKNSNSVDLLGAPFGGGAGAGPGGFGPDSDQGNFLGGSGSSYVIPPYQVTLLDALNQQAREQNTQVFAYLDNFNHTVQASQAS
TGNLAWATCLVDVRQTCGEGYDRANLTASWGGDQTILSVASQCANTVVIYSACGPFNTTTWGDHPNVTAILNAGGLGQEAGNSLVDVLYGVVNPSARLPYTLAQDISDY
PALPDLTIETLNTSFVDIDYNEGLFIDYRWFDKNDIAPAFEFGFAGLSYTTYKYSNLSVQPSTATLQKTVAASSDEVFDCLAIITVDITNSGQIGGSEIPQLYIGSPAD
GAPIKVLRGFDTVGLDAGAKKTVEFQLTRRDLSFWDTYNEGWTLPEGDHQVYIGASSRDIRETGKMKFTLA
>tr|A0A2P6VDP7|A0A2P6VDP7_9CHLO
MTTDQKVRQLGSDATTGAVPSLGVPSFIWQYECLHGMKNMGLGVMYPAPIAWAATFNHQLTSKAATEIGDEMRAFNNQEMRRGRPPAASHCFGPHVGIVRDPRWGRLLE
TYGEDPRLSADMAYAFVTGLQGGPANGTSPVKVAATCKHFVGNDMENWKGFTRYNFDAKLSADDMRDTFLPPFEACVKANSLAMMCSYNGVNGAPSCVNKPLLDGTLRR
DMGFKGFVLTDCTALNRIGGPRPYGFAYTNSRQEASVLALKAGTDMACHNYYETLDPDAVAKADIDRAARRVLTARVRLGHFDPANRQPWSHLKPTEMGSPEHRQTARQ
ILAQGIVLLKNTGGSLPLKVAELGKVAVFGPFADDEQSILGNYYSYPNHGLTTPLEALRAALPPRVKVTHDKRTMYAYSPQNAAADVERCKGADACILFMGTRIKKGTD
DGHGIPVKARYEPMSEGENMDRTSLLLNSNQRKLLQAFIKGGIPAKTKIVVVLVHGGPLDVSELQASPRIGAMLTAWVPGEGAAAIADVLFGTVSPSGRLPVTWYRDAY
SRLPMTDTRMRASAGYPGRTHRYYKGSATLYPFGYGLSYTTFSLSAPELVSTKGAGATAYVTITNTGGTAADTSVLLFLRYLGVAAGPVGPQAPEPRASIAASGCKRGG
VRTDLLQALGAYQRSALLAPGKSQRLRFWLPLDGGSASAWAGFGDPAPPCGAYALRLSADQPVAATLVLLA
>tr|A0A2V3TTN3|A0A2V3TTN3_9HYPH
MPGSVETLHVETPRVKTLLAAMTLEEKIGQLTMASGETVGGRLGVTPSVAEAIAAGHVGSLLNLWGPLSVREVQRLALERSRLKIPLFFSFDVLHGHRTVFPIPLGEAA
AFSPDLWERTARAAAREAARDGISLTYAPMLDLARDPRWGRMAEGPGEDPWVGSRFAEAKVRGFQGDDLATAGGVAATAKHIGAYGAVTAGRDYASIDISERSLRELHL
PAFKAAVDAGVAAVMAAFPDIAGVPATGNARLLRDILRGEWGFDGIVVSDYGAVAELVAHGVAADIAEAAALALKAGVDIDMTSKAYPLGLPVALARGLVSMGEIDAAV
SRVLRFKERLGLFEDPYRGLADGAGTVAPVEPPLDRDLAREAARRAIVLLANDGVLPLAREAGRIALIGPFAAAASEMLGPWTGAGHAAESITIADGLSAALPEAAISV
VAGVSIDGPDGAGIAKAVETARSADVVLLCLGEAADMSGEAASRARVDLPGCQRELAEAVLALGKPVVAVLFSGRPLILPWLVARAAAVVAAWFPGHEAGHAVADVLTG
AFNPVGRLPLSWPYDVGQIPIFYAERPTGRPVDAANRYTSKYLDVPTTPQFPFGHGLSYTRFTLGEPRPTSPVMRRGDQIFVEVEVTNEGQVAGEETLFLFIHDVVASI
ARPVLELKDVAKISLLPGASGTVRLALTGEALTFPGLDLAPAFEAGDIVLFVGVSADRAHLRRTTIRLIDP
>tr|A0A150WHT2|A0A150WHT2_BDEBC
MKKMTFEEKVGQLSQIAAGAAITGTSKEADFITDLKKGRVGSFMNVSGTEKTRELQKIAIENTRLKIPLIFGYDVIHGHKTIFPISLAMSCSWDLKAIENSSRIGSVEA
AADGVHWVFAPAVDISRDPRWGRVFEGVGEDPWWGSKVAVAQVKGIQGASPDAVDTTLACVKHFAAYGAPTAGREYAAVNVSKRSLLETYLPTYKAAIEAGAATVMTAF
NDVDGVPATGNKWLFTDLLRKQWKFKGFVVSDFTAVRELIKHGVAANESEAANLAFNAGVDMEMIGGTYLNYFKKLVKEKKIKMSTIDTSVRRVLEAKYRLGLFADPYR
YNDLDRANSVHLSKSHREAARDVARRSMVLLKNDNQVLPLKRGGTIALIGPLIHDKRNILGNWSLQGDSRDTVSVAAGFTALAGKNTKILMAKGCNLLNDEELLIKMNR
DGDEIEHDTRSPEAMLKEAVDIAKKSDVIVLTLGESAHMSGEAASRTSIRIPEHQQNLLKALKATGKPVVVILFNGRPLCLELENSIANALLEAWWPGTEAGHALADVL
FGDYNPSGHLVSTFPRNEGQIPIFYEELPCGRPNNPEDKYTSKYIDSPNDPLFPFGWGLSYTKFDYGDIKLSSKTLKWGKKLSVSIPVSNVGKYDGEEVVQLYIRDLVA
SVSRPVKQLRGVEKIFLKKGETKTVKFELTMNDLSFYNQDYKWVAEPGEFQVFVGTNSQDVKEASFNLTR
>tr|A0A1V2M6D8|A0A1V2M6D8_9FIRM
MTLEEKVAQLRGFWMRDLIENDEISIEKCRELIPDGIGHICQFASTNAYQADKLAKVISELQDYVKNETKSQIPIMFHEEIICGLAAREATVTPQMIGMACSFNPALVS
QNAKNAAESIKNLGGYHVLSPMMDVITNANWARAEEGFGEDSYMVSVFSDAFISAVQENGVAATAKHYAGYGVSNQEEDFFINETLFPFEVAVTKSKVATVMPGYHKFR
DVPASVSTELLTRSLREHLGFDGVIVSDYNAIRNAHTNHKYTKSHEEAALMAIKAGIDVDLPAGFNYAHFVELVKNGQLDESIIDASLERVLKFKEKYIPKNLSTKSNI
NLDEKKYRNDALKSAREAIVLLTNNGILPLQNKETNILVTGPNADSCYSLLGDYSWGGLAEFFRKLPIDRNDPKLYTLLGGLEATVNENFNIEFERGFNWTDEDEKIVA

TKDGDIQEQKANRTPLEQIPVTSFNSALEKAKHSDIIIVGVGETRYLCGEGSNRKGIDLPGNQENYINKLVETGKPVIVVVFGGRPMAISRLAKKCAAILYAWYPGEEG
GNAIAEILLGKTNPTAKLAVTLPNSNQDVPVCLKDTNRPQMFPFGFGLSYTTFEYSNFSKLEQVSTSNTSFDIMFTLKNTSDIDGAEITQFYFNNKQNKKLLGFSKIVL
KAGEEKNVTMRFYLDQFGRYENGEFIIKPNQYTLMIGASSIDIKYECSFDITGENIKKLKRDYFFSENI
>tr|A0A291KE00|A0A291KE00_BROTH
MMENEKLQVLLSKMTVKQKVAQLLQLNPFYFEADRNEEQITGPMTDLHVTEEEIGLVGSILGVPDAENARKIQNQHLTTDPNKIPLLFMIDAIHGYKTIYPIPLAMAGT
FNPAIVEETASMSAYETSTQGHHVTFSPMVDLVRDARWGRVMEASGEDVYVNQEMARAFVRGYQGPANELHGNQEKIAACVKHFAGYGMVEGGREYNTVDLSRKELFQN
HLPAYEAALDEGAKLVMSAFNLFEGVPVSANTYIFRDILRERLAFEGVTISDWGAVKELVYHGVTGGKEGAAELALKAGIDIEMMSSCYIEYLEVLVERGEIETAILDE
AVLRILTLKNDLGLFENPYRGLDLENKELSEEHLDIARRAAEESFVLLKNDEQTLPLNTEAKVALVGPFAESDDLLGGWVIFGKRDETPVLATKFKETFTNATVVPTAD
HQTITDAEIAEAVAAAKLSDSVVLAVGETSDESGEASSRSNIQLAAAQQKLIKAVAEVNENITLVIFNGRPLELTGIEAYAKAILIAWFPGSEGANALTRVLSGKVAPS
AKLTMSFPRAVGQVPIYYNQLPTGRPVDAADADAAFASSYLDVANTPLYPFGYGLSYGELTITDWSIANTQLQKDEAVSIEVTVANTGTTTTSDVVQLYMNDPIAAVSR
PIKELKRFHKLTMAPQTTATITLTLLYKELGYYDSQTNYRIDAGELHFTVGFDSAHVSERKTLTVKEGS
>tr|A0A559J2C5|A0A559J2C5_9BACL
MVNRQRMVDLLQQMTLEEKIAQLLQLAAPFYDGADSDGVITGPMKELGITEETVRQAGSALGLAGAEKIKHIQQMHLKNNRLGIPLLFMADIIHGFKTIFPIPLAIGCS
WDLELAEKSAEVAAKEAAVSGVHVTFAPMVDLVRDPRWGRVMESTGEDPYLNSMFARAFVRGFQGDDLTNDRHRVAACVKHFAAYGLAEGGRDYNTVDLSERQLREYYL
PAYKAALDEGCEMVMTSFNTVDGIPATANSKLMRDLLRDEWGFDGVLISDWAAIKELIPHGVAANEEEAAYKAIQAGVDIEMMTSTYVHHMPELIASGKVDEALIDEAV
LRVLQLKDKLGLFDNPLRGADPEAEREVVFCEEHRNISREMAAKSCVLLKNEEGLLPLQPSSRIALIGPFAKSSDILGNWSWTGVKDDAVRLDQALLAKVPDSLVAIAE
GSGIEMMTEEHYSEALSAAEQADVIVLALGEHSDMSGEGGCRADIKLPQAQLELIARLKQLHKPIVAVLFNGRPLDLHGVYDQVDAVLEAWYPGSEGGAAIADILYGDV
NPSGRLTMSFPYSVGQVPVYYNHFNTGRPHDPKDVDARYVSRYLDVPNAPLLPFGYGLSYTTFVYGELVLSDEIMTVDRPLRVEVTVTNTGELAGEEVVQLYVRDISGD
VVRPLKELKDFAKVSLEPGESRKVTFTIMEEQLRYHHADLQFTSDPGEFVVFVGSNSRDVIECRFHFVR
>tr|A5Z7X3|A5Z7X3_9FIRM
MNIEERIEELLSKMTLHEKVGQLHQVAPSKVGGFEIPEEEAFKLYKSGDMDEKTYDAIINHKMLSNHEDEIRKGEIGSFISVMDAETANHYQKIAVEESRLGIPLIFGL
DVIHGFKTMFPIPLAESCSFDDELFEETARVAAKESAAGGVNWTYAPMVDVARDSRWGRVAEGAGEDTYLASRFSRAKVRGFQGKDLTEEDRIAACVKHFAAYGAVEGG
CDYDTVDMSMPKFFETYYPPYEAAVKEGCASVMMAFNDLSGVPCTTNEWLIQDLLRKNLGFKGVVISDANAIKECVNHGTALDTEDAVKQSIEAGTEMDLGSDLYETLL
EQMVLDGKVEEKYVDEAVRNILRLKFKVGLFEKPYADVTKKECLLCDEHRGIARDAARKSIVLLKNDNKLLPLSKKLKIAVVGSAASDKEQMYGCWSFTGEWENAVTLV
DALKKEGYDYQYGKVCGEKLPFDKEEMMKTVKDADVIIATIEHLNSGEAESLADITIQGQQLEMLSELKKLEKPIVTVLFNGRPLAIPEIVEMSDALVEAWHLGSEAGN
AVADVLFGDYNPSARLTMTFPHKSGEYPIYYNHPNTGRPAGDYFWTNKYMDTPQKPLFPFGYGLGYSEFEYGDLELEKTQEGFKATVEIKNIGEYDGTETVQLYIHRRK
ATRVRPVRELKGYKKVTLKSGESRKVSLCVNREDLGYYDTRMNYIVDESEFDVWMAHDSGCGSWARISL
>tr|Q8Y6F8|Q8Y6F8_LISMO
MKQEKVQDLVNQMTLDEKIAQCLQLSPFLFKGTNKNAELTGPLLQEMKLTDAHTENAGSVLGSSSALDMIGIQEAYLKTNRLGIPLVFMADVIHGYKTVFPIPLALGCS
FDRETVRVMAEVSALEATADGHHVTFSPMLDLVRDPRWGRVMESTGEDPFLNSELGKAMVDGYQGDASKLDGNFDRMAACVKHFAAYGAAEAGLEYNTVNMSTRELYQN
YLPAYHAAIQAGAKLVMTAFNVVDGVPATMNKWLNRDVLRGEMDFDGVLISDWGAVAEVINHGTARNPKEAAQFSMEAGVDLEMMTTCYIHELKGLIEEGKLSESLLDE
AVLRMLHLKNDLGLFEDPYRGLKNNDRTKDILTDDSRGKARAAGVESAVLLENKNRLLPLAKETKIALVGPLASSPDILGGWNVYGEEKDGINVETGLREVFETVEVIS
TEYTEFSDEDKVAVKAAVQNMDVVVLALGEKNEWGGEAGSLATIRLPEAQYELAKFVQTLEKPVVITLFNGRPLEVKELAESSDALLELWFPGTEAGRVTADLLSGASN
PSGKLSMSFPQTTGQIPVYYNHLRTGRPQTPENKGERYVSHYLDIPNEPFYPFGYGKSYSEFELKTSSLPKELNLGEPLHVEVTIKNISDIAGKEVIQVYLQDVTASIS
RPVKELKAFEKVALQAGEEKTVTFELTSEAFSFYNHQLEKVQEPGLHRIFVGTSSEDVDAFELEVGGYV
>tr|A0A2Z2KCG2|A0A2Z2KCG2_9BACL
MTKPFIQDLVKDMTLDEKLAQLTQLGPYYWGLDDTVDLTGPFKELNLKPQVMKNIGSVLNGIGARNVIGLQTRHLQTSRQKIPLLFMADVIHGYRTILPIPLAMGCSFD
LEACERFAEIAAKESAAAGIHVTFSPMTDLVRDPRWGRVMETSGEDPYLNARVTESMVCGYQGKDLKEKGRIAACVKHFAAYGAPEGGREYNTVDMSSGVLRDFYLPAY
KAAVDAGVSMVMVAFNTIDRIPASGNEQLLRGILREEWGFSGVTIADFNSVNELIPHGNAQDGREAAEKSLAAGLDIEMMSTHYLNHGADLVEQGKLDVALIDEAVVRV
LELKDALGLFDNPFKDADPLVDEAAEPNTVHLQAARELGAGSVVLLKNDHETLPLKRGMKIGLAGPFATSVHVLGGWAGTEKDPAVSLHTGISNKTSEGDILTAMTNEL
GSMLEGIFDVEDEVEEAYHRLKDCDVILAAVGENQQDTGEGGSKTSLRLSANQEKLIWRLKDTGKKIVTIVFSGRPLELKPILAASDALLQAWFLGSESGNSLADVLFG
DYNPSGRLSMSFPYTVGQIPVYYNAYQTGRPYDPNYPQVRYVTRYLDCPNDPLFCFGYGLSYSHFAYNSFAVEAAEASDQIIASIEVENTSDIAGKETVQLYIHDVSAS
VVRPVKELKGFRQLSLAAHEKQVVSFEITKDMLMFYGKDDQLVFEPGEFELMIGRNSSDYCSERIWIG
>tr|A0A0R1N2K6|A0A0R1N2K6_9LACO
MISKNIQDLMKQMTIEEKIGQLLQLSADFFQGDNVAITGPLKEAHLDEAALYSAGSVLGISGAKTVRQVQNNYLKHSRLGIPLLFMADVVHGYKTIFPIPLALGASFDP
QVMKTASTIAAKESAAGGIHITFAPMVDLVRDPRWGRVMESTGEDPYLNSVMAKAAVNGFQGTLPLDQEHIAACVKHFAAYGAPEAGREYNTVDISEWRFREQYLPAYA
AAIEAQALLVMTSFNTLFGVPATVNQHLMRDILRNELQFHGVLISDWDAIGEIIHHGVAGDLQHAADLALKAGVDIDMMSFAYAKYLTKAAVLDTQIEQLINESAQRIL
ILKEQLGLFNDPYRGLSDKRETEVTLTSKNLAAAQAAAEQSMVLLKNNHHTLPLNSATAISLVGPAADTGDILGSWSWQGDPEKTETIAAALAKQFSNLTVTPGTQYHH
LQLSKLDDAVNASRQQEAIIAVLGLPASESGEATSVTDIKLPTEQLMLLRRLAKLNKPLITIVITGRPLDLTEVDALSDAVLLAWFPGSRGGAAIANVLSGKVDPSGRL
PMTFPRNVGQVPIYYNAYNTGRPIAGTPADDENKYLSKYIDSANSPLYPFGFGLSYGDFTISDWTLSPQSVSPDQPLMVQATIHNAGNHAGTTVVQLYTHQTIGETVQP
VKQLQAFKKISLAADQTQTITLTVPYDQLSTIHADLHRATDQGNYTAMLGFDSAHTASQPFTVVTHQ
>tr|A9B3W3|A9B3W3_HERA2
MQYEQQIEALLAQMTLAEKIGQMRQLHGTGETQQQLVREGNLGSVLNVIDADAHEIQRIAVEESRLGIPLLIGRDVIHGFRTIFPIPLGQAASFNPQLVREAARIAARE
ASASGINWTFAPMIDISRDPRWGRIAESCGEDAYLSSLMGVAMVEGFQGDDLTAPDAIAACAKHYVGYGASENGRDYNTAWIPEVLLRDVYLAPFKAAADAGVATMMSA
FHDLNGVPTSGNEFTLRQILKGEWNYDGMVVSDWASVAEMIAHGYAADLRDAALKGVTAGVDMEMASTSYAEYLAALVESGALSLDLIDDAVRRVLRIKFRLGLFDQPY
ANAAAADSVVAPDHLALARQIAKESCVLLSNQQTLPLNPQQTRVAIVGPLANHAADQLGCWVFDGKPEDSQTPLQAIRELLGDERVQFAQGLPEARSLDQSLFGEAVAA
AQTADVVIAFLGEDAGLSGEAHSRAFIDLPGAQLALVDALVATGKPVVAVVMAGRSLVLGELQDKVQAILYAWHPGTMAGPALADLLFGLDNPSGRLPISFPRTVGQVP
IYYNRKNTGRPPSEDAPSIPTGTPLDPSGFTSSYLDVDHRPLFAFGYGLSYSTFSYSNLRLSSQKLAVGDTLSITTTVTNTGKYAGAEVVQLYIRDLVGCMTRPIKELK
GFQRIHLEPGQSQTVTFELSSADLSFHNNAMQRIVEPGEFNLWVAPSSIGGLQASFELVAKSKEHRA
>tr|A0A060LXM6|A0A060LXM6_9BACI
MTEEQLQALLQEMTTAEKVGQLTQFAGAFYSEQEDSTPVTGRIEFPVDEEMIKQSGSVLGVAGATKLAAIQREHLKKSRLGIPLLFMADVINGYETIFPIPLGLGATWE
PDLIEKLQTISAKEAASAGLHISFAPMADLVRDPRWGRVMESTGEDPYLNSLFAAATVRGLQGEGSLIAKDRVGACVKHFAAYGLAEGGRDYNTVDLSERELRDKHLPA
YEAALEAGVKLVMTAFNTVHSIPATANSALLRDLLREELQFDGVIISDFGAVQELIPHGVAENEAEAAKKALEAGVDIDMMSLSYAHSLAALLDKHAIDSTLIDEAVLR
VLQLKNELGLFEDPYRGVSLQKEQTIVGHPSHHAFAQTVAEKSMVLLKNNNVLPLTKQATVALIGPYADNQDLLGEWSIFGKTDQTTTLKQAVERYYTHAIYAKGSHLY
ERNDALLTEAIAAMEQSDYLLLALGEGKDRSGEGRSRSSITLAPCQIELARLAKETGKPVIVALFNARPLDLTELEPYADAILECWHPGSEGAQAAVALLSGEVNPSGK
LTMSFPRATGQIPVYYNAYQTGRPLPPGSNERFFSRYIDIPNEPLYPFGYGLSYTSFSYSNRSLSAVEWDGSASLTVSVDVTNTGKIDGVETVQLYIQDLVGEVVRPVK
ELKSFQKITLSPGETRTITFEITHDMLQYYHSDLTYASDSGQFRVYVNGSSVCEQENTLTFSLVKS
>tr|A0A2R4X4H4|A0A2R4X4H4_9EURY
MTTREKVGQLVGTYVGTMWTHKGPDDVADEIREYGVGSAVPFGVSAADTADPLAAAELANELQEVAIEETRLGIPLVIPVDATHGHAYVLESTVLPHGLGMGATRRPAL
ARAGASVTASECRATGASVAYSPTADIAADQRWGRAFETFGETATLAGAFARAKVEGLQGGRVDEDDSHQEGLTKGVAACVKHFPAYGQPVGGEDAAPVDVSETTLRER
FLPPFEDVMAAAPAVMMPCYNAVGVEPAHSSTWMLEDLLRGELGFDGAVFSDWGAIRMLDEDHHVARDHREATYLARQAGVDSASVDGPTHAESLLDLLEAGELSEQRL
DRQVERILALKFELGLFEDPYVEADRVRDQLFTDEHRATALEAARDSMTLLDNDGVLPLDASDDVLVTGPNADSLVGQCGGWTWPDEDGLAGTTVREGLDDALDGDLVY
EPGADYTAERDLEAVETAARDADAAVAVVGEGWYIHEFGGDGSGDAEAWPRRDDLSLPPAQRRLLDTLFETPTPTAVVLVSGRPLAVTEAAERADAVLMAYYPGSTGGT
AVAETLLGDVNPGGALPVSVPRSASRVPERIEDRPHPTPIGSEHHLDSYDPLYPFGHGESYTDFEVGAPEAPATVRPGERVTVEVPVENVGDRAGERVVDVFVRDRVSS
VVTPRHEHVGFDRVAVDPGETETATVEFAASELSVQDGRERRIEPGAFEVIVGEESATIELESDY
>tr|Q5V332|Q5V332_HALMA
MADDDHIEAVLARLSRPQKLALVRGATDPEGTATGYLSGVEEAGIPPFRLVDGPLGIRAEGQRATAFPASIATAATFDPDLARQQGAAMGREAAALGQDALLAPGVNII
RVPHCGRNFEYLSEDPVHAGAVGAGLIDGIQSADVVATVKHFVANNQETHRTTVSTEVDERTLRELYLPPFRSAVDAGVGSVMTAYNRVNGTHMSDHGRLVGDVLKSEW
GFNGYVVSDWYGLESTVGAANAGMDVEMPGVAAPGAAEAANSDTADDAEEFEWPDGIPDATRAGLFGDPLVEAIDSGEVPAERLDDMVRRVLGQMDRFGRLDGSRTAAE
DGASDDQAGELDSQRHRDIAADVAARGTVLLDNDGVLPLADGADVAVIGPNIDEPKLGGGGSSETTPVHSVTPVAGIESRAEGAVTTAFGVPQIESVSLFDLLPFVGED
DDTDGTAADIAQQDPSLDDAVDAAAAADVAVVFVRDATTEARDRDSLALPGQQDDLVSAVAATNENTVVVVRSGGPVELPWREDVAAVLEQWYPGQADGDAAAAVLYGD
RDPSGRLPVTFAPEGEYPTAGAERRYPGVDDEVHYDEDVFIGYRHFDDEDADPTYPFGHGHSYATFEYGDAEITGEWTVAVPVKNAADRPGREVIQAYVRPPSVDGVDR
PRRELAGFEAVQLDADEQKTVALTLDDLAFSRYDPNSGWTVDTGTYTVEIGRSSRDIRTTVPVDR
>tr|A0A1H4ULH1|A0A1H4ULH1_9HYPH

MTTEIARRIENLIAQMTLSEKLGQLTMLAASLVETGPPGPHNPATMVREGRAGSILNTWGAKEIREAQRVALEETRLKIPLFFAVDILHGHRTIYPIPLAEACAFDRAL
WLRTAQEAAEEATRDGIQMTFAPMLDVSRDPRWGRICECAGEDAFINAEYAKAKVIGFQDAPPRPEHRLAAVAKHFVAYGAVTAGRDYAEVDVSQRALHEIYLPPFQAA
VEAGVMGIMPSFTDIAGVAMTAHKPLLHDLLRKRWGFEGVIISDYNAIAELIPHGVAADLTDAATQALKAGVDIDMMAGAYEQGLPVALERGDVSMDEIDRAVRRVLKL
KFALGLFDNPLRGLEDVSPTPDSDPSRRASARDAARRSIVLLQNRDAFLPWSQAPQRIAVVGPLADTPSELMGAWCMAGEIDETVGILGGMRTGFAGSTITHAAGGDFE
ATDAQAIADAVAVAQAADAVVLCIGESRHISGEAASRTRPAPPDSQLELARAILATGKPVLLVLVGGRPFILPQWLVDGAQAIIAAWFPGTEGGNAITDIIKGVWNPSA
RLSISWPVAVGQIPIHYGLRTTGRPHDPKNGFSTGFLDAPITPRWSFGEGLSYTTYELGEARASAPILDRDGAIEVSLDITNAGKKDGETTLFLFIRDPVAQVTRPALE
LKDFQNVVLAAGESKRATFTLRASQLSYPDMEMKPRLDSGRIEIHVGTSARACDLKRLDIEVRA
>tr|A0A1L8QUD7|A0A1L8QUD7_9ENTE
MDIQELLNKMTIKEKIGQLVQVTPSFFSEVDGTGIITGLMGDYEIEGEDIYEVGSVLGSYDREEIISIQKAYLEKNRLGIPLIFMADVIHGIHTIFPIPLALASSWNRQ
VAEEMAELSAKECQISGVHLTFSPMVDMMRDPRWGRVMEGTGEDSYLNSEFSKAFVRGYQGEKGELSENFQRIAACVKHFVGYGAVEAGREYNHVSIDDLELYQHYLPS
FKAAIDQDVKMVMTSFNPIKGIPSTGNEYVLKKLLRKQLEFEGTVIADWGAIAQLVTHGVAENKAQASSMALRAGCDIDMMTNSYYDYLESEISSGNVSMELLDSAVLR
VLSLKKDLGLFLDPYRGALEINDEFIMGKEIQKRALEISEESIVLLKNTDNILPVTKSKKIALIGPKAVTQDVLGAWSGYGDPLQAVSLATGLSKEYEQIEVIPISSVN
HISEEEKKQAVQAAKNNEVVILALGESSEESGEAASKGSIELHRSQIELLQTIEKENNQIVTVLFNGRPLDLREVEKYSKGMIEAWFLGTQAGHAITNILTGHKNPSGK
LPMTFPYSTGQIPITYNHMNTGNPLIPERQNEKYISKYLDIPNDPLYPFGYGLSYTQFSLQSQLEIPERNDDIVATVKVNVTNTGEVSGAEVVQLYIRDLIGQVVRPVK
ELNGFEKVYLKPGEQKTIYFDISPEDLSYYNMELEWVLDDGWFDVFVGNSSQAPKIGGFKYEA
>tr|V4B2C5|V4B2C5_LOTGI
MLTKLTLVFLSFAVQLTLQDYPFRNTSLPWEARVDDLISRITLDEIVQQMYKGGGGPHNGPAPPIKRLGIGPFQWDTECQHGMNEQNGTSFMQDIGFGASFSREMVRKA
AAATSIEVRAINKMYAEKGYYLDHTGISCLNPISSLMRDPRWGRSQETYGEDPFLSGQLGQENIRGQHGEHPRYIRTSTTCMWFDVHGGPENIPESRFSFNAVVSERDW
RTTFLPAFRYCIKGGSDSIMCSYNSINGVPTCANKKLLTDILRNEWGFSGYVITDQNAAENIIATHHYLNNSIDAAVAAVDAGVNLEIVADYIKNPVLKSITDAINQGK
LTEDLVRERVKPMFYTRMRLGEFDPPEMNPFSLINTSVIESANHQALGLEAAMKSFVLLKNDGVLPFGSTKFNAAALVGPLADNKNFRLAADFRFAAGCSTPACGQYNS
SAVKDAVANTDVVFVCFGLGNHLEKENIDRYDLELPGKQQQLLEDVVQYSGSAKIVLITFNANPTNIKWAEENNRISAIIAAFYPAQAAGNALRAVMTSSTTPQFGRLP
YTWYYSADQVPAMTNYSMEGRTYRYFKEEPLYPFGYGLTYTQFFYDYFEAPDIVHAGEELHGVVNVENVGTLPGDEVIQIYLSWNNPSETSPQIQLVAFERFSIDYGAT
KTYKFSISPENMAIWTDDRGWVVEEGNVTIYAGGQQPNQKKSVGSNVLSRTVKILGYKNLGYY
>tr|A0A2R4X4B7|A0A2R4X4B7_9EURY
MTVAELAGQAVGTWAGHFVDHQTVDDVERMVRSHGLGAVATFGWNGASDVLLEDVIETVNRLQRTAIEESRLGIPLLFSVDAVHGHAYVENATVFPNGLGAAATWDPAL
VERSASMTGSEMRATGAVHNYAPVADVARDPRWGRTFETFGESPFLVSEMTAAAVRGYQSGSARGGPVAATAKHFPAYGGSIAAEDAAPADISRDSLQNVYLAPFRRAI
AEGVEAIMPAYSSVEGHPPHGSRWILQDLLREQLGFDGAVVSDWGGVDHLHKDHFTTSDARDSVVRTRRAGMDVESTGGADHADRLVALVEAGVLDVETLRASAERVLR
WKVEYGLFEDPFVEAWHADQIVGHADHHAHAREMATQSLTCLENDGILPVAPTADVLVTGPNADDPVAQLGGWSVTDPDHTDAVTVRAAFESSHEGSVEFVPGASVTDR
VDVDAAARAAADADVAVVVLGEDWYIHEFGMEVITGRATGEFPTRTHLELPEAQRALLEAVAATDTPTVAVVVGGRPLILDREADLADALLWAYYPGSDGGPAIRAALT
GETSPGGRLPITIPRSLGQIPITADRLARPRPIGDDEHYPDYDPLYTLGDGQTYTTFETESVGVSPETVGPRGTITVEATVHNVGTRRGSELIRVEGSDRASDRVTPVR
ELVGFDRVTLGPGESETVSIDVPIARLARYDAEGHREVAPGEYVLAVGDHQRRVTVERQYH
>tr|A0A4R6GAY3|A0A4R6GAY3_9MICO
MTTGRIERLIGDMTLAEKLGQLQIVFRPALEDAAQLVLQGVGSVFWPPSAAATNALQRVAVEQTRLGIPLLVGLDVIHGQRTIAPVPLAQAAAFDPPLVEELASLAAAE
ARSGGVNWTFSPMVDISFDPRWGRVVEGFGEDVHLTATMGRAMVRGYQGAGLSSRRAIAATAKHFVAYGQPEGGRDYDAVDASDHRLRNVHLEPFRAVIEEGVASVMAS
FNTVAGVPMHANRRLLTGVLKHEWGFDGVVVGDADGVRNLLPHRVAETLADAVALAYSAGVDVEMGGAASELGVEERDAIDVARLDDAVERVLRLKEALGLFDDPYVDE
GAEITAPDEGSRRLVRRAAARSSVLLKNDGTLPLRAPRRVLLTGPYADSTDHLGAWTQYFGAGAGTIADALLSRPAIQVEVLPGVGFLSDDGSGIADVVEAAARADVVV
VCAGEPSALSGEAASRSDLRLPGRQAELIRAIAGTGIPYVVVLETGRPLVVADWIDVAPTVLVAWHGGTEAPAAIVDVLLGEADPAGRLPMSWPRSVGQIPMRYAHENT
GRPATTGGVLTAESIDVGLHGPDNVQEKYTSKYLDLDLGPQFAFGHGGGYARFEHGTPRVADTRIPADGGTRVEVEVTNSSDRAGDEVVQVYVEDVVASVAPPVRRLVA
FERRTLEPGETATFSFEIGPRALGFWSTATPAPEFVVEPGLFRLHIGPTLASTQAVELRVR
>tr|A0A4S2DI38|A0A4S2DI38_9CLOT
MKKEEIKKLVSNMTLEEKIGQLTQIRTSYYNNMNTSGTGTKSKLNINNNQKWMIGTVLGKLDAAMMIEIQKEYLKNNRLGIPLLFMHDIIHGFKTIFPIPLALSCSWDE
GLVEKTARISAKEGSSSGYQATFSPMVDIVRDPRWGRVIESYGEDTLLNSLFGAAMVRGYQNNDLKEEDTLISCVKHFAAYGAAEGGRDYNTVDISEYRLRNEYFPPYY
EAIKAGAKLVMASFNVLNGVPSTVNRWLLREVLRNEWMFDGTVISDWGAVKELIPHGVAEDSKDAAELSLKAGIDIEMSTTAYFEALPELCKDKNMEKLLDEAVEKVLL
LKNECGLFEDPYRGVSTEKEKKTLLRSDFRKVAREAASKSMVLLKNSGVLPLESNKKVALVGPYANNKSILGPWSLDGDLNDVVTIYEGIKNKNIIIEAVETTPFSDIN
EEDISKLMNKVTTSDVVILALGEEEKQSGEAGSVSKISLESSQVKLLERMKELNKPVIVLLVNGRPLDLTNIINKADAILECWFPGTEGGNSVADILVGDYNPSGRLTM
SFPNGVGQIPVYYNNLATGRPKDLLVNEKRYKSQYLDIPNEPLFPFGFGIGYSNFEYSGLTFNKNKIKRNEEIKCSIKVTNKGKYRGIETVQLYMRDKVADTSRPVKEL
INFKQVEINPNEIEIVDFVISEKQLRYWNTENQYKSDDGVFEFTIGKDSSSGAMFEVTLIS
>tr|A0A094J2F8|A0A094J2F8_9BACT
MDVKKLLKKMNLEEKIGQMLQIAPHNFISKSDTKIHGFEYDLGLTNKQVFSAGSVLGIGNAKEQQELQRKYLNDSRLKIPLLFMADIVHGYETIFPIPLALSCSWNTDT
AFHSARISAVEASTAGIQVTFSPMADLSREPRWGRVMEGYGEDPYLLSMFVRAMVKGYQQDDISAKGNIASCVKHFAGYGAPIAGLDYNTVDMSRLSFYQTYLGGYEAA
IDEGSKMVMASFNTFDGIPATVNQFLMIDVLRNQLNFNGVTITDYDGLNQVIAHRVAHDQREAAIQGVEAKIDIEMASSCYMKNIEKLIETSIINENDINAAVGRILSF
KNELGLFDNPYGHANIDDEKRLIKSKEHLAKAKEIAQECAVLLKNESNILPLSNNSKVAVVGPYATSKSTNGVWSWKGSINDNISLSEALINQGVDVVYINGENPLPTD
IDMLREIDIVICALGEDIEKTGEAKSIVNLELPLFQVEWIKLAKILKKKVVTVLYNGRPLVLNNIDQSDAILEAWYLGSKSNEALTDLLIGKINPSGKLTMSFPRHEGQ
IPIYYNHLTTGRPFNKDVHNIYTSFYLDCERTPKYPFGYGLSYSKFEYENMIMSDKEIYLDESITASITITNKSNVAGYEIVQLYIQDEVSKIARPEKELKGFKKILIQ
PYETKTVKFNICIKDLSYINSLGETKYEYGRFIIMIGSNSNNVQKKYIFFKENNNEDKKYT
>tr|A0A5R9EFS6|A0A5R9EFS6_9LACT
MKQVDLVALFNEMTLDEKIGQLVQLTPDFFEQGGEITGPVKEWELDSEQLSRVGSILGTQKASQVYNIQKEYLEQSRLKIPLLFMADIIHGYETIFPIPLAMASSFDEA
IIKEAARLSAHEGTNAGIHVTFSPNADYVKDARWGRVMETNGEDPILSAALTRAFIEGYQGTDLAKDKNSLAACVKHFIGYGAAQAGRDYNTVDISDIEMYQNYLPAFK
AAIDAGVKLVMTSFNTIQGIPVSGNKRVIQKTLRQDLNFEGVLISDWASIAELVPHGVAKDGYESAELAFDAGVDIDMMSDSYLSHLNLIVNAENLDKLNESVLRVLNL
KNALGLFEDPYRGLEKDSLDKKTIDGLRDSTRKIAERSIVLLKNDGILPLKNGQNVGLIGPKATSQDILGAWSWMGQNDKAISLADGLASKSINLSTLDFSENENITDE
YIEKAKKLAKELDIVIIAVGEKSDESGEGSSLVNIELSRKQDRLIQEISKINAKTIVIVFSGRPLALSNINEEARAIIQAWFPGSEGGNALANILMGDANPQAKLPMSF
PRSVGQLPNTYAQMSTGRPKKNDNKNEKYISQYLDELNTPLYPFGHGLSYSEFTLEHITLSKETMTRAEAITVGADLKNISHIKGSTVVQLYLQDVVAQVTRPMRELKK
WSVETVNAKDEKEIEFIITEEDLAYVHSDLTQRADVGEFKLYLGFDSQSAMYIGSIHLTD
>tr|A0A242K0Y1|A0A242K0Y1_9ENTE
MKEQELKKLVEDMTLAEKIGQLVQVTPDFFDQSGEITGPAAEWQLPQDKLFQIGSVLGTHTAEQVYTIQKMYLENSRLNIPLLFMADVIHGYETIFPIPLALASSFSSE
LTEEVARLSALEATRAGVHVTFSPMADHVMDARWGRVLESNGEDPTLSAELTRGYVRGYQGTDLEKETERLAACVKHFVGYGAVQSGRDYNHVDLSAIELYQNYLPAFQ
AAIDEGVKLVMTSFNPINGQLMSVNQSLIKETLRQEMGFEGVVISDWNALEELLAHRVAADKYQAASEAFQAGTDIDMMSDCYINHLSEFSELDRQELDGAVLRVLTLK
NELGLFENPFRGIPKTPDSIEQMLRNATKDAALKSFVLLKNEDDILPLQKTQQIALLGPKAASKDILGAWSWIGKTEHAVSLAEGLSSGLPHLTVWDNDGRTVDIAEAY
QKMKDLAHKNDVILLALGEASEETGEAASKAEIQLSEAQIELVKEISAVNENVVVVLFNGRPLDLTAIEPYTKGILEVWFPGSEGGTAIAETLMGEHNPEGKLPMSFPR
AVGQLPLSYQAYSTGRPLTEKNQNEKYVSKYMDIGNDPLYPFGYGLSYSDFSIDGFEQSAKILTENQPLTIQVVIRNTSKRAGVATVQLYVEDVVTKVVRPIKELKQWE
KVTIEAETTEKCTFTLSVDDLAYVHKELACYPDYGQFRFHIGLDSQQTIDGQFDYVSDEE
>tr|A0A328UC58|A0A328UC58_9BACL
MDSKQAKKPFKLDAETESAVEALLAQMTLQEKIGQLTQLGTFDEESDGELIEEGMVGSILGVRGADTVNELQRIAVEESRLGIPLLFADDVIHGYRSTFPIPLAESSSW
NLELLEETAAIAAREASSDGINWILAPMVDIARDARWGRIAEGAGEDTFLGSAVAAARVRGIQRNDWTDRPHIMACPKHFAAYGLAEGGRDYNTVDVSETRMRETYFPP
FQAALDAGAGTIMAAFNEINGVPASGNGWLLKDVLQGEWGFNGVVVSDWESVDELVQHGFAADREQAGKLAANAGMHMDMHSLIFHEHLANLIDKGDVKLEVIDDAVRR
ILRVKHALGLFKHPYVDNKLAGEFMLHEEHVASAREMARQSIVLLKNENGTLPLRQGLKKLAVIGPLADNGEAQLGCWRGQGRPEDTVSVLAGIQSLAHERQAEVVYAP
GSGVHDGTAEALEKAVEAAKASEVAVLVLGETADMSGENNSRVSLELPAAQQRLLEAIHATGVPVVLAVVNGRPLTLEWADAHIPAIVNGWQLGIQAGPAIADILFGEA
SPSGKLTVTFPRHTGQVPIYYNAKKTGRPHMRRYADSDIAPLYPFGHGLTYTSFGYDELQIEQAEIKFGQSVSVSAKVTNTGDRDGVEIVQLYICDEAASVTRPVKELK
GFSRISLKAGESATVSFTLSEEQLGFINQAQQFVVEPGKFKVWIGPSSAEGLEGSFEIIA
>tr|A0A0P6X8D2|A0A0P6X8D2_9CHLR
MTDAQTDTPLYLDTTRPVEERARDLISRMTLEEKVSQMRNSAEAIDRLGIPAYDYWNEGLHGVGRNGRATVFPQAIGMAATWDTDLIYRVATAISTEGRAKYHETLRRC
GNTIIYQGLTFWSPNVNIFRDPRWGRGQETWGEDPFLTGEMGAAFVHGMQGDDPHYLRTAACAKHYAVHSGPEDERHTFDANVTRRELFDTYLPAFKKLVTEANVEIVM
GAYNRLYGIPCCASPLLIQEILRDKWGFKGHFVSDCGALADFHQTHGYTKDVVESAGVALKAGCDLSCVCTYEHLPEAIERGLITEKDIDESLIRTYTTRFKLGMFDPV
EKVPYAAIPMDVVGCEEHRQLAYEAAVKSMVLLKNRNNILPLKKETRDIYVVGPNAANLDCLLGSYFGINDHMVTALEGLSLRAPEGTKVEYKMGSLLAQQSANPFDWS

LEAAPAADVTIACMGLAPVLEGEEGDALLSAKSGDRESLSLPAPQVDYIKKLVVRGARIVLVIFSGSPVVLGELEDMVEAIIQVWYPGEEGGKALADVLFGNATPSGKL
PITFPASLSQLPAFNDYSMNNRTYRYSKETPAFPFGFGLSYTKFTYNQLILHETNVKMGQPLELKFNITNAGDVEAEEVAQIYLTDVKASTIVPQYKLIGFKRIHLQPG
ETRTVPFTVTPEMMMFINDNGDPILEPGDFVVHVGGSSPSQRAVDLGLQANLTASFNLS
>tr|R6MKF1|R6MKF1_9FIRM
MQKYKDKSLNIEERIDDLLSKMTNEEKVGQMLQVSYNTLSKEDYEKYKNLGIGSFLHVLGDEADDIKKRAEKTRLGIPPIFGIDAIHGHCLLNGATVFPSQLAMSSSFN
RKLIHDMGKATAKEVAADGLDWTFSPVLCIARDLRWGRINETFGEDSYVIGELGKAIIEGYEEDNLIIACAKHYIAYGEATGGRDAYDSEVSERKIREVFLPPFEKAAK
VGCGSVMTAYGSVDSVPLNANPRMVREILKDEIGFDGFVVSDYENVLNLVTRQFIAENLKDASKLSIEAGNDMSMNTHEFYDCVSELIENNEIDIKYIDDAVRRILRAK
FRIGLFDGKKTVDRSVINCNEHKKLNHKLAQESAVLLKNDGTLPLKGNKTIAVIGPNADDIRAIYGDWTYFSHPVPKENVTPMGDYYTIRRGMEEVFGKENILYSKGCD
ILGEENYIDDAVKIAEKADVVVCVIGDCLAQNGEYRDRADLELSGYQTELVKRLIDTKKPVIAVLVNCKPLCISYLKENCNAIIEDFNGGDFAGLAIAEMIGGKFNPSG
KLTISFPRHSAQTPCYYNQYEGWHGGQYVDLEKGYVYEFGDGLSYSEFEYSNLRLSQNTIKNEEEITVSVDVTNKGNEDGKETVLMFVNDVISSVLTPTKQLKGFEKVF
IKAGETVTVNLKLNIKGLAIVTRDLEYIVEKGKFEIMVGRNTKEYLKDILSAEENIKF
>tr|A0A7K0C063|A0A7K0C063_9ACTN
MIIVAAAAIPVARSFSDAPRPAGLTGSALSSPCAVRVARPWCDIRLSADARAQLVVKALTQDEKLGLMAGDDLLGPLKAADDARFRAGTVHGVARLGIPELFMVDAGSM
GVKQGPNTALAAGVSLAATFDTGAARRAAAVVADEAAHRGNDVVLGPAVDIMRTPKGGRTFEAYGEDPLLSSRMGVEWVKTVQKAGLMAEVKHFPANNQEANRYRVNAV
IDQRALREVYLAPFEAAVRQGDAATVMCGYNLVNGRPSCSDRTVLGGILRKQWGFKGAVVSDWAMAAKNTDASVTNGLDLEMPVGAHYTPPLLRSVLNSKKITWGDVDQ
RLHARLRVMFAYGMFDKPKRAADGRPDYAGHGKTAQDLAEQGITLLKNSGGVLPLTTGTKVAVIGKAASQFRTGIGSMYVKPTAVTTPLQGITARAGAGNVTYNDGNDL
NAAANAARNAQVVVVVAADARAEDADVSCLTLKCGAAKDTALGDQDALINAVTAANPSTVVVLQTGGPVLTPWAAKTKGIVQAWYAGQHGGAAIARVLYGDTDPGGRLP
VTFPAAEKDAPAAGSSALHPGVNNTVTYREGVNVGYRHYDTGKITPAFPFGHGLSYTTFKLTGLKTGRTGVTVTVTNTGTRRGYAVPQLYLGLTAAKGAAQPPRQLKGF
TKLALDPGRSTTVTFPLGARARAYWDTATSTWKTAPGCAKAMVGFSSRDLPLTGTVC
>tr|A0A0R2CAN8|A0A0R2CAN8_9LACO
MSEFVKELISKMTFAEKVGQLTQLSGDFFVGDNAAITGPLQQENLSSADLYRVGSVLGVSGFEQVKKIQTNYLRKSRLKIPLLFMADVVHGYRTIFPIPLALAASFDPQ
LVRQVSEFFSREAAAGGIHVTFAPMVDLVRDPRWGRVMEANGEDPFLNSQLAKASVEGIQGQQKIPDAEHVAACVKHFAGYGAVEAGREYNTVDVSEWRLRDQYLSGYQ
AAIDAGAPLVMTAFNTFQGQPATGNYHLMRDILRRELGFQGLLISDWDAIGEMVAHGTAADLQDAAQQALKAGVDIDMMSMAYLKLTAQKNPSSKLVKLIDQAAERILN
FKESLGLFTDPFRGLAQRAEKQNLTTKNLQLAQQAAVESAVLLKNKNDLLPLAKDCKIGLVGNYANNPDLLGSWSWQGKTAETPTIKSALQAKFTRVSSVQNQQLQTAD
LQLLKQQDVIVACLGLSAQQSGEATSMTHPQLPEEQLALLRQLQQLNKPVITVLITGRPLVLDPLVKSSSGLLLLWFPGSRGAAALTQILLGEEEPGGRLPMTFPQDLG
QIPLYYNHYRTGRPLTGDQSDLENKYLSKYIDQVNQPAFCFGYGLGFAKTTLHESKLDKKTYQASDTIKVVVKIENHSDIAAKQVIQVYSQQVVGETVRPLWELKAFQK
VKLQAKEIREVEISLPLSRLAYIHRDLKSRLDPGNYRLKVGFDSQSGLIQSFKII
>tr|A0A2N6UWX1|A0A2N6UWX1_9ACTO
MSTKHPWFDASKSPRERAELLVEAMTLEQKIAQLHGAMKTINIYDLVNQGETAEEMEQLVAQIRLERHVKGIEELGIPRFRITNGPVGVGMGDGTPSPPATALPMTIGV
AASFDTELAYKYGDVIGEETATLGQHVLEGPAVNLHRTSIAGRNFEYFSEDPYLSGVMGIEVTKAIQSHDIIAMVKHYVLNDQEDERFRHNIEVDENVFRELYLLPFEM
VVKDSKVASVMSAYNRIRGVYATEYYYSLTTILRDEWGFDGYVQSDFWSTRSAAPSLNAGLDHEMPDAKWLNEENIKAALQDTSLEEKTIDRALIRRYTQMFRFNQFGK
PYNPGEIDAKKNGQISRELGSQMAVLLRNDDGLLPVTEPKGTIAIIGQQTFAGQACQGGGGSSKVDPLYTVDPAPGMEDVLKEMGSDAKVNLVVVANDLSNLDEAKAVA
KEADLVVLMAGLVATEGADMKNANMFNDQNKMLDELLGLNNKTVVVMKDSAPVLMPWQDKAPTILEVWNQGTEDGHVVADLLFGRINPSGKVPTTYPAREEDTIYYNHP
ERHPGVEEDAGYPVIRYSEGLNMGYRWWQSQGIKPLFAFGHGLSYTTFELSDVAVDTKKLTDAPVTVTAKVTNTGDRAGAEVVQVYLGIPEDGQPPKRLVGFAKVELEP
GESKTASIVIDPAATNHPFGVWCNAKHDFVIRDGEYKVYVGTSSEDTPFTETITR
>tr|A0A416EJJ3|A0A416EJJ3_9FIRM
MREDRRITDLIRSMSTEEKAGQLNLMPYMDNTDEILELIREGKAGGILIAATALAGSEIQTAPAAEIWKLKQAALHESRLGIPLLVGKDVLHGHHTVFPIPLAMAAGWN
PELVQRAAETAALEAAADGINWIFAPMLDIARDPRWGRIIEGFGEDPYLASQFAAASVKGFQRQAGAGSPGMAACAKHFIGYGASEGGRDYHSAELSDSTLHSVYLPPF
RAAVMTGVHTVMASFNDINGEPVTASRRLLTGVLREQLGFEGIVVSDWAAVDQLRQQGAAADRMQAAKAAFLSGVELDMSDGCFLECLPTLIRQEKEAAGRSGLEQRLD
QAVYRVLEVKQRLGLFDEHERIQVPVSPPSGLISKDGKNCAFRLACESMVLLKNLDGLLPLRKSGGKIAVVGPMAELKMHHLGSWSLDGREEDVISIAEGIRRTVRETE
LLWPASPLLDDALSAARQADAVVIVLGESRWRTGEAQSTATISLSPDQELWMECIAAVNKNVITVVCAGRPLILKKADQYSKAILYAWHSGIQAGMAAAAILFGEVNPS
GRLPVTFPRNAGQIPIYYNHRSPARSIDGYYGGSEYCAYQDESGAPMYPFGYGLSYSEFIYYGLEIKKEGPYVRVSIWVENRSETAGFEVVQCYVKGTGGTGRCSVSRR
LVDFRKVWIEGWEKKQVVFEWGEETVKEWEVERKCLVGCGGDCLTLEMGEMVVER
>tr|D3EAQ6|D3EAQ6_GEOS4
MDNVEQRAKELLAQMTLAEKIGQTVQYGRCEERELKLVAEGKIGSLLNVHGPKKINELQRLAVEETRLGIPLLIGDDVIHGFRTIFPIPLGEASSWDLEGMEKNARIAA
EEAAAEGIRWTFAPMVDVTRDPRWGRIAESTGEDAYLSSLAAAAKVSGFQSPNGDGNPTVAACVKHFAGYGWIEGGRDYDTTDMSERTLRETVLPPFEHGIRAGALSVM
SAFSELNGVPASGSRYLLRDILKREWGFDGMVVSDWESIEELIYHGYAEDRKDSARKGLNAGVDMDMHSGVYLDHLEALVQDNPELLQLLDDAVLRILRVKIRLGLFEN
PYVSEESEEAGDLLKGRAIPAAHLAQARDSARKSIVLLQNRSGILPLDTGKHKKLALIGPLADDRHNSMGCWAWKGRDEDVVTVRDAFQSEIAPKAEVFYEQGSGILEE
LDGGVERAVQLAKQCDVAVVVVGESEAMTGEHYNVTSITLLPCQERLIRELKRQTDTPIVAVLMNGRPLATPWLHEHADAVVEAWHLGTATGLAIVDVLTGKYNPSGRL
PVTVPRATGQIPMYYNRKNTGRPHLYEDYIDCDDSPLYPFGYGLSYTTFEYTDLQLDRHQIGRDESVRVSVSITNTGSRAGEETAQLYIRDLVASSTRPVKELKGFSKV
QLQPGESREVHFTLGREALGLLDERLEFTVEPGKFHVWVGPHSEEGLQAELEVVE
>tr|R7I7Q9|R7I7Q9_9FIRM
MLSNEAIRQQVENLLSQMTLEEKAAQMVQVPYTVVGREEALRWAKLGAGSFLHVLGDDAREVQQAALHSRLGIPVLFGIDAIHGHGLNDHATIFPSQLSCACAWDKDIA
REMGEVTAREVATDGLHWTFSPVLCLGRDTRWGRVDETFGEDPYLAGELGEAIVRGYQGDDLSDGEHILACAKHYIGYGEAVGARDACDTEMTYRRLRETFLPPFEKAF
RAGCATVMTAYGSIDGTPFTADEKSMKAILRGDAGFDGFSVTDWDNCHSLLTAQHVAADMPEASVLAAKAGNDMMMTSLGFYDAAIDAVRSGKLDEAVLDDAVRHILTV
KCRMNLLAQPEKSGRPGCIGCEEHQQAALRAARKSITLLKNDAHTLPLTSVRRVAVIGANADDIRAQYGDWTYFTHPELIPNRPAVRPYVTIREGIEAIGAQENFEVAY
HRGCGVLPSDADNIPGAVAACRNADAVVLVVGDVYAQYGETKDRADLALSGRQLELYRELRALGIPLVTVLLSSKPLAIPEIAHSTDALVCAFNGGMFGGQAVAEAIFG
RLNPSGRLPISFPHHSGQVPVYYNHLPGWHWGHYSDLPAEPLFTFGEGIGYAPFVYRDLAVDADSLTLSVKVRNAGDIAGEETVQVYLRDCVCEVMQPVKQLIAFRKVL
LQPGEEQEVVFHLDRTAFTYINRAEERVFAPGDFMLMAGHSAKDEDLLKETVWVG
>tr|A0A2A5RJ85|A0A2A5RJ85_9LACT
MELEKLSALFEQMTLEDKFGQMTQTTGEHFLEMPNKEELVETGPSMEDLGFNAENIYQIGSVLGVSSTAVINEIQRTYLKKSRLKIPLLFMHDAIHGYRTTFPIPLALA
SSFDRELVKDVAEAVAEEMRATGLHVNFSPMVDLSRDPRWGRVMEGFGEDSFLAGELGKSMIEGYQGSENGEILNDHVVACLKHFVAYGAPEAGKDYASVDMSEKEFYG
FYARPYEIALSANPRFVMASFNSLNGEPVTASRYLMNDLLREKFGFDGLNISDWGAVGELKNHGVADSDKEAGDLAMSAGIDIEMLSNTFLTYGSKILEEKPELLEQID
LAVWKILCLKNELGLFEQPFASEESEKTVVRQEKFIQLARKSALESTVLLKNNQNILPVNRKDNLILIGPFAKTQDLLGNWACKGKETETISFEQGFKNQNCPIKAYET
LDEVPDKELKYATQVIITIGEASNWSGEGHSSSDLEIADDQKELIRSLKGMGKKIIAIGLSGRPLALQSVIADLDALLWTWYLGNEMGNAVAQLVLGIERPAGRLPMSF
PRVSAQVPIRYNELRSGRPANESTYSSRYQDIEIGPLFPFGFGLQYDEFSIDDVSLSKFEINQDDVIEIHVRISNHSQYDSKFTLNLFFEDPIARIVRPVRELLDFVKL
DLKAKHSEDYSFKFTLADLSYADNKGTKVIENGPIHFYINDLSKARISCNVKLK
>tr|E6LF07|E6LF07_ENTI1
MNEKNLLELMNNLSIEEKVGQLIQITPDFFDSEGEVTGPAQAWNLDKRTLYQVGSVLGTHTREQVEMIQKNYLQRSKHKIPLLFMADVIHGYEHIFPIPLALAASFDEK
VVEKMAHYSAKEAADAGIHVTFSPMADHVTDARWGRVLESSGEDPRLAARLTAAYVRGYQGESLNDPNKIASCVKHFVGYGAAKGGRDYNEANLSDVELYQNYLPAFQQ
AIEAGAKFVMTAFQSVSGVPMTANRPLIQHVLRELLKFEGVVISDWGAVQELCAHRVAENSSEAAKLAFQAGIEIEMMSNCYHEHLEHLVSENGWEELLDQVVWRILTV
KNELNLFENPYRSMNSRVQSNEYRKVNNEIALRTPVLLKNEHQVLPLKKNEKIILLGSKADSQDVLGAWSWIGKQEMAISLKQGLVDGGNDTLTFIKGTPSERLQDAEV
VKAIQEADKIIVAIGETSGETGEAASKVHLRVPFEDQLWLDELHKMGKVTIAVIFAGRPLVLTDIEPKVDGLVMAWFPGSEAGHILAQLLTGDFDFSGRLPMSFPQNEG
QLPLTYQQMETGRPLTQTNYQEKYLSKYLDCSNDPLYEFGFGLQYATVSWKNIQLSTNQLSDECSTRLTMTVSNDTDIQTHSIVQLYMRDEVTTVVRPMIELLDVKVVK
LEPNQKVIVTFTIDKKDLQYVHADLTKQIELGKVTFFIGESSKRIVWSAPCFVG
>tr|R2Q1S1|R2Q1S1_9ENTE
MKEQALREVLDSLTLEEKVGQLVQVTPDFFDASGAITGPMLEWTMSNQERYQSGSVLGTTEASQVREIQAQYLAQSRHKIPLLFMADVIHGYREIFPIPLALASSFDEE
LVQAVARVSAKEAAGEGIHVTFSPMADYVKDPRWGRVLESNGEDPLLSQRLTAAYVKGYQGDNLKDESSLAACVKHFIGYGAAEGGRDYNTVDFSDLVMYQDYLPSFRS
ALEAGAKLVMTAFNSVRGVPISGNQSLIQQVLREDLAFDGVLISDWAAIQELIAHRVAENQKEAATMAFKATVDMDMMTSCYQRELATIVTEQHLEHELDEAVWRVLTL
KNELGLFENPYRGLDKLVATAELQEQAFEAAVKSAVLLKNEQVLPLQKNEKILLVGTKVATGDLLGAWSCVGDTAQTTSLASAFQTEFPQLTSIPLAADASDLAEKWPE
IQTAALAADKVVIAVGEAGDEAGEAASKTSLRLKPQEVDLIQQLSQLNQHCIGVIFSGRPLVLTEVVDHLQGIIAPFFLGSQTSGALAALLSGRRDFSGRLAMGFPRHE
GQLPYSYREMSTGRPETAANHGQRYLSNYLDEENDALYPFGYGLSYNTWEYQDITLSQEPLTKDAPISLRLTVTNQGTQTGSENIQLYFTDEVAQVIRPKIELLDWQLV
SLAPGESREICFVIHSDQLAYVHQDLRRYPDSGRFQLHLGKNSREILASFIVEL
>tr|A0A1I0PAT0|A0A1I0PAT0_9EURY

MQSDSTSLVDDLTLEEKLELVHGTLDPDEKATGYVPGNDRVGVPPLTMVDGPLGVRALGEQATAFPSSIALASSWDPDLAREFGAALGREAAAHDQDVVLGPGVNIIRT
PHSGRNFEYYSEDPHLAGRMGVGTIEGIQSEGVAATVKHYVANNQETNRYEVSADVSERALREIYLPAFRAAVEDADVLSVMTAYNRVNGVHMSDHEHLLSNVLKDEWG
FDGLVVSDWWGTRSAVDAALAGLDLEMPGVDLEAYLPGEADDEMEAPDDEDGELPPLPDVPAYFGEPLREAVESGAVDESVLDEKIERLLRVMEAVGRFEAEGEADARE
GELDTPEHRRLARDIAVEGTVMLTNDGTLPLDESDSIALIGPNADAAKLGGGGSSEVSPVTETSPREGLAERAADLSFERGVSPIAESSFFDDEDEPTAADADGDTDAG
ASIDDAVAAAEAADCAVVVAQDDATEFKDRDHIELPGEQNELISAVADAADRTVVVLRTSGPVELPWLDAVDAVLETWYPGQADGEALAAVLFGDDDPSGRLPVTFGRS
AADYPTADEAAFPGTDGSARYDEGVFVGYRYFDEHDVEPLFPFGHGLSYATFEYGDATVSETDDGFEVAVDLRNVGERPGKEVVQVYARKSSAPVACPDRELVAFDAVT
LEPGEKTTVRVSLDRDDFAYYDEDDGWTAATGTNTILVGRSSRDVRATFDVDV
>tr|A0A6C0G6B7|A0A6C0G6B7_9BACL
MNDNQLQQLLQAMTLEEKIGQLTQITGELYVGKVDAEMVETGPEYASHVLGDGTLYTIGSVIGASSAKFTNLIQSEYLKQSRLKIPLLFMHDAIHGYKTIFPIPLGLSC
SWDENVLETAAAVTASELRASGIHVNFSPMVDLVRDARWGRVMESFGEDHLLSGNLGRAMIKGYQKIQEGRISAAGVAACLKHFAAYGAGIGGKDYYAVDMSLREFFDY
YGKPYEIALQEQPKFVMSSFNSFNGTPVTASKRMMKDVLRDRYGFDELVISDWGAVAELQKHRVAHDGREAAELAMNAGIDIEMVSTLYLEHFETVLAQHPALLADIDA
AVMKVLRLKNELGLFENPYASEEEEPSVILNADFLAAAEDAARKSCVLLKNDGDALPLRAAHKRILLVGPFAGTKELLGNWACKGSFDDVVSLADGLKQADPSLTVDVC
ETLADCPADVLERSDYIIASIGESWTLSGEGHSSVDIGLEASQQALVRAVKATGKPYACVCFAGRPLALQDIADDMPALLWCWYPGTRAGAAIASLLTGQATPSGKLTM
SFPRHSAQNPIHYNEYSSGRPANASSYSSRYQDCELGPLFPFGHGLTYAGATYADFDISSDVITADKPVSVSFTIHNPSGYAYPEIAILYIEDVVSRSVRPVREMKAYQ
VISVPAHGSVRATLTVTLEDLHYLDAELRRTIEPGAFRLYINDPRQPVFTITH
>tr|U4KMV4|U4KMV4_9MOLU
MKDLVSTLTLEEKIGQLIQIAPFFYIEKIDKNVFGPLLDLGISEEEIFLTGSVLGIKDAEEMIEVQKTYLEKSRHKIPLMFMADIIHGYKTIFPVPLAMAAAFNPNLVK
KAARISSIEAQTAGIHVTFSPMADLTRDPRWGRVVEGFGEDPYLNGVLAASMVQGYQHDGIDKEGNLASCVKHFAGYGLSEAGRDYNTTDVSRLNLHQYYLTGYKKALD
AGARLVMTAFNLIEGVPATTNAYLLREVLRDQYQFDGVVISDYDSLKETIEHGTSKDEKDAARQGIIAGLDIEMATAAYFRNLPALIQENKVDIALIDEAVLRVLELKR
DLGLFDNPYKGASVTKAKQLVLSEAHKKASLEVALESAVLLKNDGGTLPLKKEMRVALLGNYATSKDTIGPWSWHGNPSDNNSLDDVLNGYAASLYVNDTLEAHLLDET
SINDSDILVVAIGESRRESGEAHSKTNIKLSNQQERFINDLKQFNKKIVLVLFNGRPLDLSGVIDQSNAILETFFLGTCSSEAIAMLLYNEKNPSGKLPMSFPRNVGQV
PIYYNYLNTGRPYKNDGNEYTSFYLDEKNDPLFSFGYGLSYSQFEFSNLIVKNAVFYKGETFQFSIDVTNTSAFDGYEVVQVYLRDLVAEVSRPVKELKAFNKVFVKKN
ETVTLEFLLDDEAFSYVHRDLSFESDAGDFILYVGNSSTSTLETKITMKEGKL
>tr|A0A3M7P1S5|A0A3M7P1S5_BRAPC
GDQPNYDENPINITKLEYAIKQKHVGSILNAPYNIAQKSSTWQEAIKTAQNVAQQTKNKIPIIYGIDSIHGAHYIQESVLFPHAISLAGSFNLDVAKKIAEIVSIETRA
VGIPWNFNPVLDVGRQPVWPRLFETYGEDPYLVGKMGEAFIQGSQGNDIKNRTKVATCLKHYIGYSLPFNGKDRTPAFIPENMLREVFLPPFEKAIAAGAPTVMVNSGE
VNGIPGHANYHYLTEILKGELNFSGFVVSDWEDIKRLHYRDKLAENDEEAVRIAVMAGLDMSMVPYDYSFADYCVNLAKKDAKFSERVDDATRRILKVKNDLGLFENSQ
PFAEDLNKIGTMESHNLNVEAARETIVLAQNDGTLPLNSNENKKILVAGPTGNVLRVLNGGWSYVWQGSVEELFVNFGTQKKTTIFEEVNKLNSNSEYSQGADFNGLTN
LNETVEKAKNADIIILTIGEDSYCEGFGNIDNLMLSESQQVLADRLLELNKPVVLVYIGGRPRIITNIARRSKAVLIGFLPGEKGSKAIAEIIFGKYNPNAKLSVSYPL
NVNGITTYDYKPMEIYDANKLYTFGHGLSYTKFEYSNLRLSKKSIGLQESLTISVNVKNSGQMDGKESVLLFLNDEYASVTRPVRELKRFEKISLKIGESVDVKFNINS
DDLSFINTNNQRVVEQGKFSIFVEGLSDSFSLIMDKDVTTSQVQTTMTTS
>tr|A0A0P6WDS3|A0A0P6WDS3_9HYPH
MTLAEKLGQLTMISLGGPPTGPVADPATLADVAAGHVGSVLNLVGRDRIATAQTLARSTRLGIPLVFSLDVVHGYRTIFPVPIGEAAAFDPALWQETAQAAAAEARREG
IHLTFAPMLDIARDPRWGRIVEGPGEDPWLAARFARAKVDGFQGADLAAAGTLAATAKHFVAYGAAIAGRDYASAAVSAGSLAEVYLPPFRAAVDAGVAAIMPAFSDID
GIPMTAHAALIAGRLRHDWGFDGVVISDWDAIGQLVAHGVAADLAEAAALALNAGVDIDMVSGAYRCGLPEALERGLVEPETIDAAVLRVLALKERLGLFAAEAEPAAA
AGPPASTDAAGRRALARHAAERGTVLLHDRSGLLPVGSPRKIAVIGPFGRDRGGGADRIGPWAGLGEPDRAPSLPTALRARFPEAEILSKAGVDARRPGRRGLAGAVAA
ARAADLVLLCLGEPADWSGEAASRAEPGLPGDQAALAEAIFALGRPTVLILTGGRPLIAPAVIDRAGAALMAWFGGSEIAAALARILAGDAAAGGRLPVSWPRSPGQLP
VFFGERPSGRPYDPDNPFTSHYLDLPNAPQFPFGHGGTPGAFALSGLSVTPEAVGPTGTVSVAVTVASTAARSDEAVIFVFIQALKALPNRPVLELRAVERVALAPGET
QRLTIPLSAAALDRLDDGAGPEPGRYAVRVGLDAERSRHLVAEVTLGAG
>tr|A0A1Y1WE41|A0A1Y1WE41_9FUNG
MLIGSDGTLNRTAAEYWINKVKVGSIYDTPGNRGGKYAWYAPQTLANITNTIQELALAKGSRVPVLFGMDSVRGANYVKGAAMFPAGIGLAATFQPMYAYEAGRVAAKD
TRASGYQWAFAPSADLNVEKRWSQNYRSFGEDPALLSEMVRYSVRGYQGDYKSDRTRVATCVKHFIGASYPFNGKERSTQFIPDNILFEYYLPGFEAALNSGATTLMES
LSNLNGEALVESSFYLKKLLRDKLQFRGVMLTDYEEVRSQALDFHTAANFTDAVYLTLNNTSVDMSAATSDAEFTLDTLDLVRGGGIHEDRITESVARILQLKKDLGMF
ETPFADPNLFTAVGAKQDIELARNAVRESITLLKNANNVLPLKDDDRVLFIGPHLNSTHLLGGGWNIHRQGPTEIEGDAIYQGLGDTIMSGIESITKHRPVFMEGFSAT
GSELQEDILQSIIDIAKQAEKVVIGLGESNYADDQGDVDDMSLPEPQIELVRRISQAVDRPIVAILVEGRPRLLKDVAELADGIVNAYLPGMYGGVPIAEVLYGKISPS
GRQPFSYPKHEYQARDTIWQGMWNEYAPQYPFGFGLGYSTMVYSNISVDSTDLRPGKPITVRLSIQNTGPFDQREAVLLYTTQSFRTGYQPELFRLRRFNKVEIRSGTL
TEVSFTLTAEELAYYNRDLVKVIDPSPVNITINALTPQERTISIRLSV
>tr|A0A4Q2KED3|A0A4Q2KED3_9FIRM
MKKEYYEKAKQLLFQMTLQEKIGQLCQKNIGDTSGLGEEKNLINEDAYDKIRQGRIGLILQPAWNMIDDIREAQRVAVEESRLKVPLLVHSDIIHGFDTIFPLPIASAC
SFNTELIRRSAAISATEATVAGINVTHAPMLDIARDPRWGRIAEGAGEDAYLAGEIAKAYVKGFQEDNGRSECLSATLKHFAGYGAAEAGRDYNTCELGERTMRNTYLR
PFKAGIDAGAKLVMAGFHTVDSVPMTANAKYLRKILRKEFTFEGVVISDWCAPYELIAHGIAENEREAALAAFQGGIDVEMCSDCYDRFLNELITAGLIDEKTLDAAVL
RILMLKFECGIMEDPYLFMRKSGACELPPDHLQVAEKLADESIVLLKNENILPLKPGEKIIVTGSRCFDANLLGCWQSSRFITDTITYPQGLEKEGFHCHYASTAQELI
SDDCGTVIVFVGEAAENSGEACSEQDIRILEEDTELLRIAKENDKKTVCVVSSGRPMILTETEKYSDAIVYAWYLGHSAGKSLAGILSGRVNPSGKLCVTLPRDMGQIP
IYYNHLPTGRPRSEADDNKFTSRYIDGSSEPLYPFGFGLSYSKFIYENLILDSDVITDVPVKASITVENQSDTAGKETVQLYVHDVCAQISRPVKELAAFQKVYLNGRE
RKTITFKITEEMLSYYHSDNTFCCDPGLFEIYIGGDSETKIFKSLRKI
>tr|A9WCT4|A9WCT4_CHLAA
MNDVEDRVNTLLGQMTLEEKIGQLNQPMIHGLPGLDLLRQGKAGSIINAFGALSGQGFDHLNSAEQCNALQRAALESRLGIPLLFGRDIIHGQRTVFPIPLAQAASFNP
SLVEQINQIAAREASALGIRWTFAPMLDIARDARWGRIAEGYGEDPLLTSRMAAAAVRGFQGDDVSQPDRLVACAKHYVGYGAAEGGRDYEQAEISEPTLRDVYLPPFR
AAVAAGVGTIMSAFLDLNGMPATANRRLLTDVLRNEWGFDGFVVSDWESVGELVQHGIAEDRAHAAALALRAGVDMDMVSGAYLETLAENVRCGRVTLAEIDEAVRRIL
RIKCRAGLFEHPLTDPERAIHDILTPKARELARQAARETMVLLKNERHLLPLRDFRRILVAGPFVHATGELFGTWTMDGRAEDAVPLDQAFQAIAPAGTDLWFAAAPDL
ALSRAHYADAVVLLVGEHPARSGENANVSDLGLPPGQLEWITAMAAIGKPVVLVVFAGRPLAITRAVAQAQAVIYAWHPGLEGAAALAEILFGLATPTGRLPVSMPRTT
GQAPLYYAHKPSGRPLEADGPFRTRYVDIPTAPLFPFGYGLSYTSFSYSDLRLSSAHMRGTLEISALITNTGERTGSEVVQLYVRDLVGSLTRPVRELKDFQRITLQPG
EARRVSFILREEDLAFTRADGSWGVEPGRFKVWIAPHAEAGLEGEFIL
>tr|A0A4R4BP97|A0A4R4BP97_9SPIR
MSEIDFDSILTVLYAMKRRFLMKENRPSLGKNILRFVGGALFFSLLAGGVIGCRTQNAAEGKVPQNAPYKDAGLPVEKRVEDLLSRMTLQEKIGQMTQIERGSLRPGDI
SRYFLGSVLSGGGGAPRPNTVQGWQDMIRRYQEEALATRLQIPIIYGVDAVHGHNNLQNATIFPHNIGLGATGDADLVRRIGKATALEMAATGVYWNFAPCIAVGRDPR
WGRFYESYGESSALVARLGRAYIEGFKDAAALLPVRPVTTAKHFIGDGGTRWGTSKTDTYKIDQGDTVADRTYLEEVLFPPYKAAVEAGVRTIMVSFSSLNGIKMHAHR
ELITDVLKKSWGFTGFVVSDWGGIDQIDPDYSRAVEQGINAGIDMVMVPYDAPRFIDTLEQLVQKRKVPLSRIDDAVRRILRVKFEMGLFDAPLEAVLAAQPASVVRSA
DHRALAREAVAKSVVILKNNGILPLGGSGSGTTGARAEKAGGAEKGGVAGAGTGPRRLFVAGWAADDIGIQCGGWTIDWQGKPGNITAGTTILGALREALPGVEIIYDP
RADFEETPGSGAGSGSGAKNAPQSGAKTASDRGELCIVVAGEFPYAEGKGDTARPELPPREQEALREARTRFKQVILVIISGRPLVLDEESLSCDAILAAWLPGTEGAG
VVDILLGHVPSTGRLPCAWPRSVEQLPLDTLIQGNEKPLFPVGWGVE
>tr|A0A6G7Z9B1|A0A6G7Z9B1_9FIRM
MSKVDIQALLQSLTLEEKVGQLAQITPNFFLEDKGEITGPLHDIGYSIEDLNHIGSVLGTHTKEEVIAIQKAYLEQSRHQIPLLFMADVIHGYKTIYPIPLALSASWNT
DLVERVSQYAAYEAATQGIHVTFSPMVDTVKDARWGRVMESTGEDVYLNRMMSAAMVKGYQGNSKLDENYDAIAACLKHFAGYGYTEAGREYNKVDISHNELHQFVFPP
FKAGIDAGAKVVMTSFNLIDGIPATGNQWLLKSILRDQMAFKGVVISDWGSVGEMIPYGVAADGEAAAHKAINASVDIDMMTNSYLKNLARIASNDNDVAMKIDEAVLR
VLTLKQELGLFEDPYRGLLSRYDYQFKQEEARKLSREAGQSSIVLLENDGSLPLKTQQKIALVGDKVKTQDVLGAWSWVGRTDESISLYDALVAQDIEIVSPVDADVII
AVVGETSEQSGEAASRTSISLSEETEALLDSLRTFKKPVVTVVYSGRPLDLRRAKENSNSLVQAWFLGSEAGNALVDVLYGVYNPSGKLTMSFPYNIGQIPIYYNQTVT
GRPLRPWNEDNKYVSKYLDAPNTPLYPFGYGLSYSEFKLDDIKVPAEFTREGLTLTFTLKNVSERVGTETIQIYIRDEVAEIAQPVKLLKGYVQLTLKPDETKTLHYTI
PCDEFAYINSREQRVVESGSFEIFVGTDSTAPCAGKTIYKGVNYEIK
>tr|A0A6I2GEK1|A0A6I2GEK1_9LACT
MKKEKLDQLLEKLSLEEKVGQLVQLGPMFFSEGGEITGPMQAMDMTKTELYQIGSVLGTHTKEEVKNIQSTYLEHSAHGIPLMFMADVIHGYETIFPIPLALASSFDPS
IVKEMARLSAKEATSAGVHVTFSPMADLVRDPRWGRVLESNGEDPLLNATLIKAYVEGYQGDSLLDPETLAACVKHFIGYGEAEGGRDYNTVDISDLVLYQNHLPAFKA
AIDAGVKLVMTSFNTIRGIPATGNRWLLQEVLRRDLNFDGVIISDWASVHELINHRVAADKRDATFKAANAGVEIDMMTDNYQHHLVDLVKEGLISEEFINQAVMHVLQ
LKNDLGLFEDPYRSLDKHPEKEIIEVELRAESRRIASQSMVLLENQQAILPLQKEDQLALIGPLAESHDLLGAWSWIGKTEQSVTISEGLLSKTKQIEMVKDRHDYVKL

KYVDKVIVALGEHSEETGEGGSKTEITLPQEQIDLLKEVYKWNQNIIVVLINGRPLDLTEIKMYSRAILEAWFPGSEAGNAIADVLYGDVNPSGKLPMSFPRSVGQVPL
TYNMMSTGRAINEYNHTQKYVSKYLDNENTALYPFGYGLTYSNVKLSEVEVIDKGDNVTVNYTLINPSMLDAQEVVQLYVKDMVTEVARPERELKYFEKVFISAGASKK
CSVNLTSEDIAYYHPDLIKKADDGTIELYLGLDSQAPLIGSWHYKA
>tr|A0A7L6N4W5|A0A7L6N4W5_9BACT
MDNKINDLLKTMTLKEKIGQLYQAPYFSDVITGHAFDSSATIQRIKDGRVGSILSVHDEKVLYQLQKTAVEESRLGIPLLFAFDVIHGYKTSFPINLALSNTWDMDLIE
RISKAVAFESTKKGLHLTFSPMVDLVRDPRWGRVMESNGEDPYLSSCLAKAYIKGYQGNDLSHPDTIAACAKHFIGYGLSEAGREYNTVDLSKRVLYNMYLPAFKAAVE
ANVQMVMTSFNTVFDVPSTANKALLKDILREELDFKNVIISDYTSTEEIINHGIARDLKDVAEQCFNAGLEMEMVSESYISHLEELVHEGKVKESDIDDSVSRILTLKY
KLGLFDNPFKYFYENSDQYMLLEKTRELSREAAEKSMVLLKNNQVLPLKKETKIMLCGPMIKSQDLIGEWAALTSKDDVVSIFDAFSKDKTLSIIEESGDEAIKNAEVI
IMALGEPGNQAGEGNSKTHLHLADEQKAYFDKVYQLNQNIVLIVFAGRPLIMTDFANKVKALIYAYQPGLEAGNALKRLMYGDCSFSGKITMTFPYHQGQIPIYYNHYA
TGRPFDPQRPNYRYNTRYIDCPNEPLYPFGYGLSYGKFVYKNFIINQHKLNKHDKLFLQVEIENQSQYPADEIIQCYIQAKNFSVSRPVNELKSFKRVSFKAYETKTID
FTLDINDFRSYNINMEWTAEYRDYFVKVGPNSQDLLSISVNVVD
>tr|F4S421|F4S421_MELLP
MEHIFNQKSRILRLSPGPCTGTTSEVNGRIHFPAICMQDGPAGLRNVDDLVSAFPAGISVAATWNRKLMRARGVAMGEEWRAKGAHVYLGPAVDVTRDPRAGRSWEAFG
ADPYLNGEAAYETVKGVQSQGVQTCVKHLIGYQQEQYRFTMTSQIDDKTLKELYLKPFQRAIDAGVTCVMCSYNKFNGLSACKNPTLTGDEGILREELGFQGYVVSDWG
ATHDGNWNVKRVNETALAGIDVEMPGGFMLIGGGVYDNLEEAVNENYVTDATIDKMATRFISAWYKLGQDQGFPQISFNVHDQSANINVNARSNKHTKLIRKIASASAV
LLKNSDDILPLSIPSTIALIGLDAGPISNTDCEMNACKLGGTIPVGWGSGTNSLKHVVSPAIAIQDMICKDDLPTIVSTSLTDDISSAISVAEAAEIALVFVYTGEIGA
GFTRVEGNLGDRKNLNLLQNGEELIKAVSAVNNKTIVVIHSVGSVVMETWVDLPGVKAIIMAGLPGEQTGPGIADVLFGKVNPSGRLPYTIAKSEDDFGVKVYPEGLLN
GLEPIVDYKEGLFTDYRRFNQLNTTVRYCFGHGLSYTTFRYSQVNNSCAIQTLIGLTKSDHFFQVTNDGPRDGTEVAQLYLTFPSGLGEPPKQLRGFDSVFIPRGETQT
ISLELKTRDVSVFDTNLNKWITPDRGFNVYVGSSLLDIHLEGTI
>tr|A0A369AW13|A0A369AW13_9FIRM
MHITEKDSRPGLAQIEEQISSLISRMTLEEKVSLCHGNTLFKTAGVERLGIPPLVTTDGPHGIRQEFKEHKFEVTGQTDDFVSYFCSLIALAATWNDERAYDFGSGMGQ
EARARGKDVVLGPGINIMRSPLCGRNFEYLSEDPYLISRIAVAYIKGVQQQDAAACVKHFAANNQETNRLSVSVEMDERTLREIYLPGFKACVEEGGVFSVMGAYNKLR
GQYCCHNEYLLKTILKEEWGFDGAVISDWSGTHDTMEAANNGLDIEMGTERPFNEFYLADALVEAVKNGLVEESVVDDKVRRALRLRFKAGMFDENRCKGSYNTAKHQR
ASLDIAREAITLLKNEKGILPLNENSIGSIAVIGDNAVKKHASGGMSSEVKALYEISPLEGLKKKLGGKVKINYARGYSENEAEADILMEEAVKAAAMSDVAVVFAGLN
HDFDTEASDRTDMSLPYRQPELIQRIYDTNPNTIVVMISGLPVEMEPWLKNVPALLQAWYCGMEGGNAMAEVLFGDVNPSGKLPVTFPARLEDCSAHSIGEFPGSDAVE
YKEGIFVGYRHFDTNKIEPLFCFGHGLSYTDFEYSGLKITPPSVERGQSAQVSFCVTNTGERDGGETAQVYIRDTESSLVRPEKELKGFKKVFLKPGEAKELSFVLDEA
SMSFYNDKIGGWTAEPGAFEVLVGSSSRDIRLKGGFELKK
>tr|A0A1W9QH37|A0A1W9QH37_9DELT
MDAPLVGLELNSSSQRESATRRKAGLLSVCLLSFLAGCSDVPGPINGTGGTANPGAGGMPPVGAGGSGGMAPGAGGASGGAAPGAGGIAAVGGFQAAGGAIGVDPDAYE
IPEIAWPSESCVSQVDALLAKMTLEQKAAQMVMGQYGDVSAADVSGEVTGTVFASGSAIPGSSRAGDWASMIDGYITASQSTPNSVPILFGVDAVHGNSKVVGAVIFPH
NIGLGAGKNPKLVERIGEITAFEMMATGATWTYAPVLSVAHDKRWGRTYESFSEDPEDVALLGAASVIGLQGREGLGSGNPGVIACAKHFAGDGQSTYGTSRKEPAGSD
TGGLVDRADVKVDEATMREYGINPYIPSIKAGLGSIMVADTTWNGVNMTGHEQLLTEILKGELGFKGFVSTDWDAAMPDQKGPGVIAAINAGVDMLMAANDWKGQRSAI
INAAGNEISQERIDDAARRVLTAKCEAGLFGWSRDPAHLEKVGSAEHRAVARQAVRESLVLLKHENNVLPLMQGSKAWVAGSGANALGRQTGGWTINWQSGGDMTEGTT
ILQGIAKAATVVSTPEEADVAIVVLSESPYAEWRGDVASINTLPAGDFELLAEARASGKPVVAVILSGRPVLITDHLDKADAWVAAWLPGTEGDGVAEVLFGLYPFKGK
LSHTWPRSEEQVTLKKFDETYDPLFPYGHGLTY
>tr|A0A387BQ62|A0A387BQ62_9LACT
MKKENLDKLLEQMTIQEKIGQLTQLGGEFFQTDNQEITGPIYAENKISEAEVYTLGSVLGVYGVENIRKIQSDYLEKSRLKIPLLFMADVVHGARTIFPIPLGLATSFN
PELARKTAEIAAKEATAAGLHVTFAPMVDLSRDPRWGRVMEGTGEDPFLNGVFAERFVRGFQGDFDEEHLAACVKHFAAYGAVEAGREYNVVDLPENKLRELYLPAYKA
AIDAGVALVMTSFNTINGVPATGNKWLMKDILRDEWGFDGVVITDYGAVMEQVIWGTAKNAEEAAQKALSATVDIEMMTTAYLQTLEKLVKTDKKLSVLLDEAVRRVLE
LKNRLGLFEDPYRGATLEREKSQVFTEENKQVAYDAAVESIVLLKNDKKSLPLKKADKVSFLAPLLDSTDLLGSWSWKGDVTETESILSVLTEKECCPTKEADKLVIFV
GEKSAQTGESKSYTNISLTQEAIDLVGQYADGKQEIILVVFAGRPLDLSTIADRVDSILYAYFPGTMGARAIVDLLYGTQNPSAKLTMSLPRSSGQIPIYYNQYMTGRP
TLTENEEYVSRYQDCLSTPLYPFGHGLSYSDFEYFDPSVSTDENKIKVDISVKNRSTVDGKTTVLIFVRDLVATTARPLKELKAFEKIELNSGEAKRLHFEIDSEQLKF
YNFTNDYVFEHGQFEVFIAKSSEDIIESKIVEF
>tr|I2F843|I2F843_9BACT
MKRLSDMTLEEKIGQLVMYGKYGEAQQKALAEGRIGSFLNNRGEETQMRQNLALDSPTGIPLIMGDDVIHGFRTIFPIPLALSCSFDLGLIEETCALSAREAAMEGINM
IFAPMVDISRDPRWGRVAEGAGEDPYLGSEVARARVRGYQRNDWEDLPKTAACAKHYVAYGAPQGGRDYDGADISERSLREIYLPPFDAAVKAGVMSVMSSFNDLNGIP
VSGNERAIRGILRGELGFGGVVVSDWESVEELVNHSIASDGREAARLGFKAGVDIDMNSGVYERYLKELVREGKLTVEEIDQAAGRVLKLKERLGLFGKKRFRIEDARR
LSLENLAAKELALKAARESIVLLKNRDLLPLRKGTKLAVIGKLADDRPDMLGCWAGQVEVQESVTVLEGLENLGEEFRYERGCEINSDIEGGIDRAREIASKVQVVLMV
LGETSSMSGENRSRADITIPAAQRRLLRSVMEVNENVVLVVVSGRPLVLSWEEANVPAILQLWQPGHQAGNALAEILYGIHNPSGKLTLTFPASVGQIPVYYNRKNTGR
PLFKKYIDIQDEPLFPFGWGLSYTDFDYEDLALSSGSPKMGEELKVSARVTNRGDHDGSETVMLFVRDVTASVTRPVMELKGFEKLFLKAGQSKNVQFDITGEMLSFLD
ENLKPALEKGRFEVFLGRNSVEHLKGYFELI
>tr|A0A6S6QYD9|A0A6S6QYD9_9HYPH
MTLEEKIGQLVMTGIDSPLGPDAIETGRTGSLISFNDAAAIAEAQAKARNSRLGIPLLVGLDLLHGFRTLFPVPLAEAASFDPALAARNAELAAREAVPAGLNWTFAPM
VDVGRDPRWGRIVEGAGEDVRLAMDFAAARARGFRAGGIAPTLKHFAGYGAVAGGRDYDAVSVSDYELQNLHLPPFRASLGPMTTVMSALTVTNGIPASSDVGLLRGLL
RDQWKFNGVIVSDWGAIDGLVKQGTAQDAQDAVRQAMAAGVDIDMASGFYAAHLAGEVKAGRIAMSNLDAAVRRVLALKFELGLFDKPIADPDAAEKAALTPELRTAAR
ETVRKSAVLLKNENQILPLKPDIKKIAVVGPFADNAFEQLGPHEARGRPEDAVTLLKGITERAKGNGIEILHAPGCDVTCYEDKGFRQAIEAAKSSDLVIAVLGEKREF
SGEGASRAFLDFWGRQEDLLEALAATGKPVVLVIVAGRPLDLRRASEIVPSILMAWYPGTEGGNGIADILFGDEAPSAKLPISWPRSVGQSPFSYDTPASGRPYVPDAR
YVLRLIDENPTPLYPFGHGLTYTKFAYSDLRVAPVQSMNSVAQASITLKNAGSRPGTEIVQLYVRDVVASRVRPVRELKQYRRVSLAPGQSETVTFLVTRDELGFRDER
GELIVEPGDFKIGIGADSTTELAAGFTLEK
>tr|A0A518D915|A0A518D915_9BACT
MIDPRRIDGPNHRPLESHVTYESQVTPLNDSLMANQQYNRSRSVRRVPGRMRRTCLPVLACLTLAVTQPPYVGADDSRVVDFTRYDGEVNPLLQRMTLAEKVGQMTQAD
LGGLKDFRDIATLSLGSVLSGGDADPAEGNAREAWDDTYQQCQRQAMASHLGVPILYGVDAVHGHNNVLGGVIFPHNIGLGCANDPDLVEQIARLTALEVRATGIQWTF
APCITIPRDDRWGRTYEGYSEDPRRVAELGAAAVRGLQGGDLRSPTSVLACAKHFVGDGGTSALVGPSRFRKGLGLTLDQGDTRCDEATLRRIHVAPYPPCIAEGVGTI
MPSYSSWNGVKCTMHHPLLTDLLKEELGFDGFLISDYDAIDQCHSDYKTAIGLSINAGIDMAMVSKRYKQYIRLLTELVEEGTVPMARIDDAVRRILRVKASMGLLEAD
YSPQTDPALVAEFGSSQRRSVARDAVRKSLVLIKNNGVLPIRDSVRHVRVAGAKADDMGVQCGGWTIDWQGRKGEVTPGGTTLLQGVREVATGVEVTHTVDGRGVEGAE
VVIVVVGEAPYAEGVGDDAELGLPLEDLALIAEAQKSSAPMVLVLLSGRPIALDDEVIAGADAIVAAWLPGTEGAGVADILFGAASPTGTLSFTWPHSADQHPINVGDE
KYQPRFPFGHGLRYPAPRSTQREATTPIRE
>tr|A0A1Z5SL14|A0A1Z5SL14_HORWE
MCLQDNENGVRGTDQSSGFPSQLSIGASWNRSLALERAQFLGREFKASGANVVLGPVGGPIGRIARGGGIGRAIGFSNDPYLSGQLIAPTVTGIQESVIACVKHWLFNE
QETNRNPFAFGFLGYFGNQAVSSNVDDRTTHELYMWPFQDALAAGAGSVMCSYQRANNSYGCQNSKLMNGLLKGELGFQGFVVSDWYAVHTGIAANEAGLDMVMPSSSF
LTPESLAEAVNNGSVSAERLTDQATRILAAWYRFAELEEPGLNQHEETNAQAEDVESTLLQGAIEGHVLVKNTEGALPLDPSSTHTLNLFGYDAIGGLNTSAEGFLLYD
MGLANTQKYEDGRIFTFIDYYLQAGDILPDPHAGPQIALDGTILSGGGSGAVKVAWSISAHDALASRARTWNQTLHTQFVDQKPTVKAPDAPCIVMINAQSSEGWDRSG
IRDEYSDTLVQNVASQCKNTIVVIHNAGVRLVDSWIENPNVTAVIFAHVPGQMNGEALAAILYGEQSPSGRLPYTHFIQQDITPRFPFGYGLTYTNFTYSNLSIRLAEG
ATTSPLPPAFQPSNPNTTTTLPQGGNPSLFDHLATISVQITNSGSVTAAEVAQLYIEVASSDLPLALRGFEKKLLSPGETETFSFPLRRKDLSVWDVGQQEWVLSKGPH
GVRVGKSVGEILVEGTLELN
>tr|R9MUP5|R9MUP5_9FIRM
MKRWRKVIALFLLAVLVTVGCGSGKEKMETEVQSENEALNQEGRTQPEELNEPEDVPVEGTEVDKSVYMDAGQDVEIRIEALLAQMTLEEKVAQMVQPEQAGITSSDVE
QYGFGSVLSGGGSSPSSGNRPENWQERVNELKAAALNTRLGIPLLYGIDAVHGNNNVYGAVIYPHNIGLGATGDLELVERIGEAAAEEVRAVGIQWTFAPTLGNPQNEC
WGRTYECFSEDLEEVSKYGRAYIRGFQGEAGTEKYLDEYHVLACAKHFVGEGYTDEGVNQGNISMTQEEFDLLLESGVIDPYTAALDEGVRTVMVSFHSIDGVKCHENK
HLITDILKGELGFTGLVVSDYNGIQQLSGVTYKEQVRQGIDAGIDLFMEVYVWEDFIKYAKELVEEGSISKEQIDDAVRRILRVKFEAGLFEEEIGGNTEQEMLNQVGS
IEHREIAREAVRKSLVLLKNDKIGETTALQALQNAQNIRVCGQKAYDLGSQCGGWTISWEGSAGNITKGTTIIEGIASQIMLEKTISHDLKGEVLEENDGVIVVFGEGP
YVESGGDRTAADLKISAADEEMLENLRASLAQKNRQDIPVIGIIIAGRPVNITEYMDIFDAVIMAWLPGTEGEGVADVLFGDFDFTGKLNFTWMKNPEDMDEKFKEGNE
DKILFVRGFGLDKNGDILE
>tr|A0A1G4VAB2|A0A1G4VAB2_9FIRM

MKKKKLIKALGVVLTYSLLFASCGKAEDVSISSDTSVSSTAEVLEEVDEPETVEAADEPAVEAAKKIYLDKELPVAQRVEALLSVMTLEEKAYQCVQGEQVNVSVEDVK
NTGIGSVLSGGGSAPSYGNHPEHWQKRVNELKAAALETRLGIPLLYGIDAVHGNNNIYGATIFPHNIGLGAANDPELMEEIAHVVAREVRAIGVQYSFAPCLANPQNER
WGRTYEGFSEKTADVAKLAGPFVAALQGNVKGDEYMPEDAVIACAKHYIGEGYTTEGVNQGNVEMSAAEFDELLAMGVLDPYKACIDNNVLTVMPSYNSIDGLKCHENK
HLLTDVLKEQLGFKGMVISDYNAIGQCKGTYDEEVANCMNAGVDMFMEAQEWDRCAQTIIKLVKAGKISEERLDDAVSRILRVKFMSNMFDEEVGSAHENELFGEFGSE
ENREVARRAVRESLVLLKNGNVDGKLAMEKIKEATNITVCGSGAFDIGRQCGGWTISWQGEAGNITLGTPIVQGIYDEVKEYAKVSHSVRGELDAASDVIVTVVGENPY
AESDGDVKPSDLKPISGDVKLLENIEKQIVANGNDPLKVLIVIAGRPIDINDYVDKYDAVVMAFLPGTEGEGIADVLFGDYDFTGTLPITWLKDFKKIDEKHELPESEI
LFPYGYGLNKTGELLIRE
>tr|A4BA26|A4BA26_9GAMM
MIKPRYWIALSALLLLTLTVVVSHWPSTMSLGLPNSPYQTVSTWPEFERPVDAALEARIDDILQTMTLRQKVGQMTQGEIQHVRPSQAKEYGLGSVLNGGGSWPGKTPF
HVLEDWLELADAYWLASTESATGIPLLWGTDAVHGHNNLQGATLFPHNIALGATGDLELVRSIAAVTADQVRASGVDWTFAPTVAIADNPAWGRSYESFSQDADAVFHF
AKAVVEGYQQGQNAPGILATAKHFIGDGATRNGVDQGDAWVSEAILRERHAQGFYGALDADVQVIMASFNSWWTKRLHGHEYLLTDVLKKQMGFDGFVISDWNGINDVY
QCLPNSCPQAINAGIDMVMVPTAWKAFIDNTVASVEAGDIPMSRIDDAVRRILRVKLRSGLFEQPRPSERIGAGDESAVNSPELNALARQAVRQSTVLLKNNDQVLPLN
PAGRYLVTGLAHRIAIQAGGWSLNWQGGAYDNDYFGPSATLLDGLREWTGANNGSLQVGLPGVNEKLDADAAIVVLSERSYAEGEGDLTAWQSSAAEKQTGFDGIAQLS
AIQQRYPELPIVTIVIAGRPLWMNPQINVSDAFVMGWLPGTQGAGIADLLFGEHPFTGRLPFNWPADDCEGLPRSTRRAAFAVGYGLTTDQTVIVPELKAQPAREIGTN
GLAPDCVWQRVLLSPFQ
>tr|W4V508|W4V508_9FIRM
MELYKKDLNPKALERVLIEYGVGFIFYGGTGILKDNTIEGWRNVCDELKKYTDKTRLKIPVLQGLDAIHGFNYLSNATIYPHNLGVVSTWDDSLAYRLAKNVEKELRYA
GMNFVFAPNGDIARDPRWSRVYESCGEDPYLAAKMTEQYVKGYQESKLFGACAKHFLAYGESSTGRDREPIDISERTIREIHLPSFRAAVQAGVKMIMLNSSSLNGIPV
HKNKWIMQKLLREELGFQGLIVSDWADLQKIYTRHMTMDSLKSGIIAAINAGIDLNMVPDNLDIIDIIINAVENGLIPMKRINESVERVLRLKAEFNLFNGRKESEVVE
IGSPESVETAYHIVSESAVLLKNDQVLPISDNHKRILVVGEAADTRRHLCGGWTMGWNIINEEDIQSGDTLLTALRKNSKEFSKIDYAKDEIELSKYKETADMAIMVIA
EEPYAEEGCDLPELCVPQEQLDLLKKLRKNQEKVVCVLISGRPLVIANILELADAIVWCCLPGTQGGTGIADILLGKVNPSGKLPISFPRSSSDLPVLYNSRNMTTYNP
LFPFGYGLSYTSFTYENIYVPEELEANQDLIITLDIRNNGDLDGKEVVQVWGRHLYFSCVGLRLELLGYKKLLVRKGQVENVTIVIEKDRLKVLNEENKYVFEEHLLQV
LVGGKQFDIRLHK
>tr|R7IED0|R7IED0_9FIRM
MLTKKWMRRSLAMLGALTLTAGLWAAPAMAEEKTYTQPTLNPHVKSIIEVDGYQFIDLNSNGTLDPYEDWRLDADTRTADLVGQMTVREKIAQMQHPTYLPRADGKIPS
YLNKWCNKEGIGMLLIRELNSVEAAAVSMNTIQEYAEGSRLGVPVLVSMDSVHGLSYVSGATVTGHNLALAATRDEDLVTRLAEIARDEHIAIGVRMTLSPEADIASEP
RWGRVMETFGEDPDLVTKMVTAQVVAFQNGRDGLNTGAIVACMKHFPGAGPQMEGKDTSPIISSAETLQIHLKPYYAALEVNIASIMPYYSVPLELDMENSAIGSKATL
QDLLRDEMGFEGIIQTDWGMIWAIQEGRGTMMGEEVSDEEAILIGITESRVDGIGGESIRLIDQMEELTNAGKIDEDILTAAATRIVKAKFELGVFENPYCDVEYAVQF
VGNEEHQAVNLEAARKAMTLLKNDGILPLKQNEKQTILVCGPRAGDMDSLVGGWSSAQEGLTIADAVAAYAGENATVIYESDNVERIAELAKDADIIIVSVGEPSYQHD
PPWGYDTLEITGSQQEILEAAKASGKPMVTVVTGGRPYILTWCDENTNAILEAYYPGSQGGIAIAETLFGLNNPTGKTPLQFPRDMDSVRNQEGDVSFDLENPLYDYGW
GLSYGE
>tr|A0A194VF47|A0A194VF47_9PEZI
MSQEDRIEALLRALTLEEKCFLVAGKNMWETCDVDRLGIRSLKTTDGPAGVRGATWIDGTHTTFIPCGISLAATFDPALIERVGDILGAETRSKNSNVLLAPTMNISRS
PLGGRNFENFGEDPYLSGIMATSYIKGVQKHGVGACMKHYVANDMETRRFNMNQQIDERTLREIYLKPFQMTLAAQPWTAMAAYPKINGHHADCSTFLLQDILKKEWGF
DGLVMSDWGGTNDTVESILAGTDLEMPGPPIRYGKALVEAVKAGKVSEKDHLNASVMRLLRLLERAGLLDPERKQADQELDSDLPEFRQTAREAASGGIVLLKNSGILP
LMPQKIKKLAIIGPNAKTPTTGGTGSAALRIDEQALFDEAVAVATEAEIVVLVVGHNNDTEKEGIDRTSLSLPRRTNELVEAICRANANTVVVTQSASAIAMPWAGAPP
AIVHAWYQGQENGNALADVLLGHVSPSGKLPVTFPSRLEDHGSHSWFPGDAERDYAEYGEGVLVGYRWFDAKNIQPLWTFGFGLSYTAFRVTDAKVSGTINLDGSAKAI
ISATVTNTGAHDGAEVVQVYVAPSPVIAQKGRDAPPRSLVGFYKVFVRKGKSELVRISLDVSAVSWFDVEAKGGPSSSGRWRADDGKYKCFLGTSSRDIVAEVEVVVE
>tr|A0A1M6SZE5|A0A1M6SZE5_9FIRM
MGKSKISGAALFCLAFICLLTGCTGFQNKETRKQEAKHITEEGPSATTKAAEPTKADSIDRGYLDTTKSIEVRVEILLSQMTLEEKAGQMLQAERNTVKESEVTKLALG
SVLSGGGSYPGKNTLSDWNSMIHNIQAAAMKSRLGIPLLYGVDAVHGQNLIKGAVVYPHNIGLGAANDADLMYQMGAAVAEEMKLTGTLWNFAPCVAVGQDPRWGRTYE
SLSSDPKIVSTLSSAYLRGLQEHGVAGTAKHYVADGGTSYGSGIINGILDRGDTDISKSKLFKIHLAPYKQLVKSGVKTVMASFSSYQGTSMHENKYLLSDVLKGELGF
KGFVVSDWEAVKDLEGDSFEEKIAFAVNAGVDMLMEPYDYAKAIDGIVANVNKGTVAMARIDDAVKRILRVKFEMGLFEDPYMEKLNHQVTELGSDEYKGLAKQLVEKS
LVLLKNKKNILPLKQGTTIFVTGPAADDMGVQCGGWMSTWQGLSDKDGKKAAEGTTILEGLEEYAEQYDLNIITDKDKAGEADVVILAIGEIPYAEYEGDTRDLSITGV
KGLEENKESIKFAKSLGKPTVALIVAGRNVLISNHVKNWDSIVMCYLPGTQGDGIASVLTGETNFTGKLSMPYYKSIKDIGKKDAEYLYPLGYGLTYKSKK
>tr|R1DJC8|R1DJC8_EMIHU
MASPLTSADDEALIEEILARLTLREKLGQMMQPDWRTFRSAPKLSEAGAAVLPAWAGSAVKRALNAPLTDEQCDRSVGRHCLGSVLGGGGACPTPNEPLAWQAQAAAMQ
RAALRASSGLPLLVCNDSANLRDATLFPHHIGQGCMRDEGLVEELGALAARESAACGINWIFSPCAAVALDLRWGRTYESFSEDPALCGRLSAAEVRGIQRCGLPMAAC
AKHWVADGGTALGTGKFDMGGIGKAAVKSFGLDQGNADCDDAELRATHVAPYLPALAEGVLTVMVSYSSLNGVKCHASRALVTGLLKEELGHARLREITRDAPRLGLLK
EELGFRGVVGSEERLFGDLTWRRQLEELEAAVGRGDVPAERVDDAVRRVLRVKVACGLLRRAAASTPAGGDAAAAASRCPLFDAAVPPMGDAACVGCEAHRAAARRAVA
KSCVLLVNEGGLLPLPAGEGGRPAAELLVTGIAADSLGRQCGGWSLEWQGCEGNAFTTGTTIADAIRAVQPGARLAPSAGAASRPVAVVVTGEAPYAEGFGDVEELRLP
PADVRAAVALADAGWAVVLLLVTGRPLLLPPPLLSRVAAVLVAWLPGTEGDGVADVLFGRAPATGRLSFSWPHAMDQAAAAARLKGPLFPFGHGLQTRAVG
>tr|A0A4R3VMD8|A0A4R3VMD8_PELSC
MPRATPILTGTLLLCVATLADAAGTDSIVQAWPTRPSVRDAALEARVEAALKGLSLRQKIGQMTQPEIRAITPDEVRDYAIGTVLNGGGGWPGQDRNATPARWRALSEQ
FQAAARQATPGVPLLWGTDAVHGHNNVRGATLFPHHIALGATRDADLVRDIGRATASAVRASGLHWTFAPTLAVVQDVRWGRTYESFGADPALVRRLGQAEVEGLQDGL
AQGRGVLATAKHFIADGGTRRGIDQGVSHATATELAQVHGAGYLGALDAGVQTVMASFSSWTDSAGYVEHGKMHGNAALLTGVLKDRLAFDGLVVSDWDGIAQLPGCTV
DDCPAAINAGIDIVMVPFHWKPFIANTVKAVEQGRIPMARIDDAVRRILRVKFGVGMQPAVPATVEAMQAPELARRAVRESLVLLKNRPGLLPLRPTARVLVVGAAADS
LPHQSGGWSLTWQGRDTTNADFPQGTTLLAALRQRLGGPQVAFDADGSRSDPARFDAVVAVLAESPYAEGEGDIRITDNLRHSRRQPQDLALLQRVAGRGAPVVTLLYS
GRPLYVNDLANHSDAFIAAWLPGSEGAGLVDALVAPADGRPWPGFTGRLPFPWPAGPCQFSSHEAKPWWPVGGGLSGKETQAGGKLLPEPADPQPICPL
>tr|A0A0M9UBR3|A0A0M9UBR3_9CHLR
MTLRRTLFILLVALGVGACGGQASPPQQSLATETLASPTASPTEMPPTPAPPLYTQPDADIDARVDDLLSRMTLAEKIGQMTLIEKNSISPDEVRELAIGGVLSGGGGY
PKDENSPEAWARMVEAYQQAALETRLGIPILYGVDAVHGHNNVYGATIFPHNVGLGAANDAELVARIGRATAVEMAATGIYWNYAPGVMVPQDIRWGRTYEGYAERPEH
VAALASAFVRGLQSPDIAPPNLVMSTPKHYLGDGGTAWGTGDSGYQIDQGDTQVDEATMRALHLPPYQAAIEAGAKVIMASYSSWNGEKLHASLYWLTDVLKGELGFDG
FVVSDWGAIDQISPDYYEAVVTAINAGIDMNMVPYDARRFITTLTQAVEAGDVPEARIDDAVRRILRVKFHMGLFEHPMAHAELLSEVGSAAHHALAREAAAKSAVLLK
NEGNVLPLSKDVGHLFVGGLAADNMGIQAGGWTIEWQGKTGDIIPGTTILEGIQAAVSPQTVVEYNKFGNFQGDPSADDAVCIAVVGESPYAEGRGDSADLRLPTKEIR
TLNRMEEACARLVVVLVSGRPLIVTDHIDAWDAFVAAWLPGSEGAGVADVLFGERPFVGRLPYTWPRSVEQVPLGRSDEPPLFPYGFGLTTEGE
>tr|A0A2Z7A0Q1|A0A2Z7A0Q1_9LAMI
MQGIERLGIPGIDLADSAVGVRTATFQGRYATLLPSTLGAASSWNPQAAFLYGSVIGRELRAMGFNMSIGGGVDITREPRNGRNFEYAGEDPLLAGTMTGTLMKGVQAQ
HLMGDIKHYAFNDQETGRTTYSADIGKRAARESDLLAFQIAIGIAHPSGVMCSYNRVNATYACENHWLLTDVLKHDFGFKGWVLSDWGGTHSTVKAALAGLDQEMPSDR
YFGEPLKQAVLAGKVPMARLDDMDHRILRSMFAAGVVDDPPTPRSVIDPFRGRDDARHIAEQSMVLLKNEGHLLPLDAASIRSIALIGSHADVGVPSGGGSAQVNAPGG
DAIGPDAREGEAIYFPSSPLKYIRKHAPAAQVGFDPGTDPAAAAALARKAQVAIVFVNQPMREGMDRPTLSLPYGQDALVAAVAAANPHTLVVLETGGPVAMPWAGQVQ
GIVEAWYPGIGGAQALADLLFGKADFSAKLPVTFPRNDDQLPHPVVGGLAAKPSSSFDVDYDKAGAAVGYKWFEEKHLKPLFPFGFGLSYTSYAYSGLTVDRDGRSATL
TVRNTGARAGTEIAQVYAVLPKASGETYKRLVGFTRVELAPGASKTVTVPLNPLCLSIYDTSKDAMLRIPGRYTILAGPSSADTPLEASFVLGH
>tr|B8I1R3|B8I1R3_RUMCH
MSLKGLGRVLLTTLLAMSLCCCSNTGSNTRKVSNKPYISTNSGNNSEVTAIYNKQKSIKDNISAYISKMTLEEKVGQMIMAEKDYISAHDVKTYGVGCVFAEGGSAPKD
NNPDGWRSMIETYKKAANDSRLSIPLLFATDAVHGNNNMKDTIIYPHNISLGATRNGKLTRQIGAAVADELKAIGVDWTFSPCVAVSNDIRWGRDYECFSETPDLVTMM
ATALITELQNKGIIACAKHYVADGAVEFGSGTNGLLDRGNTNISTEELKDKYISVYKDAVKSGVKTIMVSYSSIKGRKNHSERDLIEYKLKQDIGFQGIVISDYEGVEY
LDGNSLYTKVVNAVNAGIDVLVEGKRWKETYKCLLEAASQKRQDVNMDRIDDAVSRVLRVKMETGKFEGKDVTNKNYKLRQTSNVQLAEQAVKESLVLLKNKRKILPLK
KSDKVAVIGPASDNIGVQCGGWTKTWQGGLDDGDEKWMSGTTILDGFKEMADKGGGLIITDPSRVKEADVVLAVLGEHPYAEGKGDEKALGLSDGLAFKENAEVLRIAY
QSNKPIVVILVSGRPRIITNEINKWDAMVEAWLPGTEGRAVAQVIYGENCNFKGRLPVSWPKSVEQLPITIEKLDNNEVYDALFQYGFSLKYSN
>tr|S0FQH7|S0FQH7_RUMCE
MGNKKAVVLVMLLLISIFTGSCSDSGERASVVPGVNVSVPDNGYNELESLYDEKKSVDENISAFLNEMTLEEKVGQMLQVERRSISSKDIQKFFIGSVLAAGGSVPEKN
TMVEWRKLTDEYKSAAVHTRLGIPLLFAIDAVHGNNNLENTVIYPHNIGLGAGGDSELAGKIAGATALELNAAGIDWNFSPCVAVSNDIRWGRSYESFSENPDLVSIMS
IPYITNMQKNGVVACAKHYVADGAVSYGTGDSGYLMDQGNVNINQKDLNDTCLSVYQEAINAGVKSIMVSYSSINNEKNHGNKYLLQDRLKDDMGFNGIVISDFEGIHQ

LKGGSLYDKVVLAVDAGIDVLMEASQWRECYEALIEAVGNGDIRTERINDAVTRVLRVKMEMGKFENVSSIPANEYSLRNIIHKQIAEEAVLKSLVLLKNKDNIIPFGN
NQNIAVIGPAADNIGIQCGGWTKTWQGGMDNEDGRWMSGTTILDGFKELAGKKGHKIITDIAKLEEADIIVAVLGEYPYAEGKGDDDSMDLVNGTALADNEKTLKAAYA
AKKPIVVILVSGRPRLITDEIDRWDGLVQAWLPGTEGGVIARAFYGDAEFTARLPVTWPRNLEQLPITLYKQSDGYNALFPYGYGLNVKN
>tr|A0A4P6PZN7|A0A4P6PZN7_9ACTN
MAGPRAATARSGRSPWFAAACSATVGALLLPLLAAAAPTAAAAPRCDSTEDCLAQMTLEEKAGQMTQVNHPNVMDNKEALAEYGIGSLLSGGGAGPGGEAGGTASEWAD
MYDEYQRAAMKSRLGIPLIYGVDAVHGHSNVEGATIFPHNIGLGATRNPWLVRKAQNITRKEVLGTGIDWNFAPAVSVPRDDRWGRTYEGFGEVPWLASSMGRASVRGF
QGYRLDSESVAATAKHYVADGGTTWGTGEGDYQIDQGNAQMSERELRRIHLPPYRAAIRADVASVMISFSSWNGLKMHQHEYLVNDVLKGELDYDGVVISDWAGVRQVE
GDSYAEKLRKSINAGLDMIMVPNDYKQNIDAIVSEVRAGRISEKRINDAVRRILDLKFDMELFEEPFTDRRYTDDVGSAKHRRVAREAVAQSQVLLKNDGNVLPLSRKS
GKDIVVGGKTADNLGYQMGGWSITWQGSGGDTTEGTTFWEAIQAETEGTRTDVEFVGTETGGDYSGDIGIWVGGETPYAEGFGDDGDLQLSEADTRQLNDICSKTDVCI
AMLVSGRPMIINEELKTADAFVASWLPGTEGAGMTDVVFGHERFRGRLPVTWPSSVDQQPINLGDHRKDPLFRYGYGLRR
>tr|A0A4R4SR02|A0A4R4SR02_9ACTN
MHRPRTRRRLLAGFTAGALAAGALTAATASAAQAAPPDGGHGTGHGTGHGKDPRLMRVVRSMTLEEKAAQLFVLQIHGLSADTSDPAAVAANRRLYGADNAAQVMARYR
PGGFIYYGENVRDPQQVAAFSNGIQRAAAAQPHRIPATIATDQEGGIVARLQPPATQSPGAMALAAGRRTADARALARITGRELRAVGIDQNYAPDADVNVDPANPVIG
VRSFGSDPGLVASMVTAQIRGYRSAGVTATAKHFPGHGDTTTDSHVGVPQIDHTREEWERLDLPPFRAAIAAGVDSIMTAHIVVPSLDPSGDPATLSRPILTGILRERL
GYRGVVVTDALDMDGVRAKYGDERIPVLALKAGADVLLKPPVGEDGTGAFPRQLAAVVEAVRSGELTERRIDESVYRILALKHQRGLFRDPYADPARVGEIVGSPAHLA
AAQRAADRTTTLVRNDAGVLPLRPGARDVLVTGWGVSTTAVLGTEIARRGATTTIRQTGAAPTPAQIDEAVAAAREQDLVVAVTNRAWDAEDEPGHNGPGQMDLVKALL
ATGTPVVVVAVRDPYDIAWFPEADTYLATYSYTAEALRSAAAALFGELNPRGRLPVAIPVRDRPGTALYPFGHGLSYRR
>tr|I5AXQ2|I5AXQ2_EUBCE
MKYRKIAAMTLAFSLVFASCGSTASENSSVSTSVSKEAAKGGIERYEGKTAEEIVASLTTEQKAAQMVEGAFYNVSPEDMKTYDYGSVLSNFSELPNPSADDWMNTVRE
YQEGALSSEAAIPYIYGQDSVHGVNYASGCVIFPHNINMGAANDPELMKKYGSLVGSDIVHTGMLMNFSPCVDAAQDPRWGRTYECYSDDNEMVKNLSVAYAEGLLSEG
VVVCAKHFFGGGYTKYGTGENSDMTERLIDRGDAQMSKEEIDGQLSVYDGLVKAGVQVIMVSHSSLEGTKMHENAKYISYLKDDLGFDGFVLSDWDSIENCSGADLKEN
VILCVNAGIDMLMEADNFEECRGYLVEAVEEEAISRERLDDAVTRIIKVKMDAGLFKDPYLKEVKPTYEYGSEESHKVARELAEKSFVPLKAGEHMTIEKGMKVYVSGP
AADDTGVLCGGWTYLWQGETDANNGERVLPDSPSILDALKASAKEKDFEIITDPKKIDECDLIVLCVGERPYAEWNGDTKDLSIVGELALEGNKKAIKEAAKSGKPTLT
LIVAGRNVIVDDYLKDWDSCIMCYLPGSEGGNAIADVLTGDASPEGKLPMPYYSSVKQIGTGKCWHEAGWSATEA
>tr|K6YRG7|K6YRG7_9ALTE
MIKSKQNINQQIIANVEALMAKMTLAQKIGQMTQAERSTCTAQDVYQYHLGSVLSAAGSVPGNNRLKDWLEMTDAYWLASMQTDADHLAIPVIYGIDAVHGNNNVKDAV
VFPHNIGLGAGADFDLIEQIAEITAKEVCAIGVDWVFSPNLAVAEDYHWGRTYESFSERTDLVCDFAKSMITGLQSALPQSGVLACAKHWIGDGGTLHGVDQGDTILDW
QQLEQIHVRPYYQAIEAGALSIMVSFSSWNGEKCHGNRHLLTDILKGNMQFSGILISDMQGIDDLAEDFYIAVAKGVNAGIDMFMVPGNWKQFIEHLISHVELGTVPIE
RINDAVRRILSVKMAIGLFEKPRPSKRQLANHASFGSKQHRNVARKAVQKSLVLLKNHDHVLPLSKNSRILVTGNSADNIGYQCGGFTISWQGDDGNEEFPAATSIWQG
IQNQATNAQFIGAGEITDIDPNQFDVAIVVVGERPYAEGLGDIRYDDDVMFKSGLQINGQLRMQPASGNSLELQVMYPQALQTIKTLKVKGIPVVTILISGRPLITTSE
ITQSSAFIAAWLPGSEGDGVADVLYAKAAFSGKLGFSWPDNSQSNIDLEKQAFDTIYPVGFGLTYPVKTLAKSAV
>tr|A0A4Q2A2W5|A0A4Q2A2W5_9FIRM
MKKWMTCLTLIAVLLMGGCGTSEVEDEVVPVDDETAQAQEERGKILIESLEDIDRAIKGMTLEEKAGQMIQAERSGIQLSEISKYNIGSVLSGGGSVPTKNTPEGWMQL
SNRMQKVSRNSSSGIPLIYGIDAVHGHNNVLDAVIYPHNIGLGAANNPALMHEIGKAVAKDIKATGIQWNFAPAVSIVQDIRWGRTYESYSEVTGRVSVLGSEYIKGLQ
GEGVVATTKHFIGDGFTTFGTGEGENLIDRGDVTADYQVLLNMYLPAYEQAIASGTKTIMASFNSVNGVKMHGHKTLLTDVLRTQLGFEGVVISDWEAIDGLEGTLEDR
VASAIDAGIDMLMQPFNWKEVYEAILSGVENGKISEDRIDEAVKRILVLKFEAGLFEPFTEKSATDLGTDEAKALARKAVSESVVLLKNEEVLPLDSGLKIYLVGPASD
NVGIQCGGWTLSWQGEMTADLNQGTSIKEAFEAELAKGGGRLVKDPEEADLVILVIGEKPYAEMNGDTADLSLDGPLSLEDNLAAVKEVKKYDLPVVTIMVAGRPLLVK
DHIGGWDAFVMAWLPGTEGAGITDVLFGQSPFKGTLPVTWPIENEQASDSALFSDYDRLEHQYKYGDGIIDN
>tr|A0A1V1PCC3|A0A1V1PCC3_9DELT
MSDIENKVNNLISKMNLDQKVGQVLQVEREFITPEEVKTYHIGSLLSGGGSTPGDNMPEDWIQMNDELWAASMEENERYLAIPVIYGVDAIHGHGNAKGAVIFPHNIGL
GAANDPDLIERIAMTTARETAATGVDWTFAPTLAVVRNDHWGRTYESYAEVPEIVTSYAARFVKGLQGNFGEENVIACAKHFIADGATLHGVNTGDAPINEAELRKIHL
PPYLAALKENVLTVMISFSSWNYIKCHANKYLITHLLKEELGFDGIVITDWDGIDYLSDDYFEAVAIGINAGMDMFMVTERWKLCYHHLKTHIQTGRVAMSRLDDAVRR
ILRVKYKAGIFDKPRPAQRILSQPPTCMGSSQHRETAREAVRKSLVLLKNNKDILPLNKDARIIVAGKSAHSRGIQCGGFTIEWQGVLDNDSIVGGTSIWEGISKAAPN
ASLSENLTGEDAHLNKHDVGIVVIGEKPYAEGFGDIYPLGIGLTKAIRPDHMSSQYASDLFAPLNGEESYGTTLNLCELHPEDLKTIQNISSKGIPVIVILISGRPLVV
NKELDESEAFVAAWLPGSEGGGVADVLFGDYDFQGKLSFTWPTYDDDNLNIGDDNYHPLFAYGYGLSYRRS
>tr|A9NG51|A9NG51_ACHLI
MKNKLITIAIIICFVVMIVSCDNTPPVIHDNDYPDSNLTMNEKINWWLDKLTIAEKAGQMVQGERSNNNGASGVKPTDVRNLNLGSVLNGGGNRPSSNTTFGWVSMYEN
MLNASLESSSKIPIIYGVDAVHGHNNLYGATIFPHNIGLAAANNKELMKEIGMITAYEMGQTGMNMNFSPSIGLIKDKRWGRTYETLGESPDIALNLIPSYIEGIQSYG
VIGSAKHFVGDGYTTFGTGLDNKLDRGNSTISKEDLETIHFPLYEAAIEAGVKSIMVSYSSLNDVRMHENKELITDILKDQMGFKGFVIGDYNGIDDIRANTFYERVIK
GVNAGIDMLMQPHNFKEVIDAIVRGVEEDRIDIDRINDAVSRILSVKYEMGLFDEKTPIESDLRSENALNVARKAVRESMVLLKNNQNLLPFNKDLNLLILGKGSQNIG
IQSGGWTIDWQGSDQLNIPGTTIVDAFKSVTNGQIYTDINDIDKADQIIIVFSEKPSAEMMGDSLALSLTDDTSYASNQTLIDIAKQTNKPVIGLLLSGKPLIIEEVIP
YLDAFVMLFLPGSEGLGITDVLYGDYNFKGKLPFTWPKSISQSSHTVLDENYEPSDYRYPFGYGLNYTILQ
>tr|A0A4R5GYI3|A0A4R5GYI3_9ALTE
MKRLKLTLTACLLIGLYGCNGPQPSTSENVVSQLKLSETTPEKFVQSLVSQMTLAEKVGQMTQAERNNVTPEDIKKYFLGSVLNGGGSVPGENRPEDWRAMIDAYQAAA
LDTRLGIPFIYGTDAVHGHNNVKDATIFPHNVGLGAMRNPDLMEKIGKATAAEVAATGVHWNFAPALCVSRDKRWGRAYECYGEKPEIGVSYSGRYVKGMQESGLVLAT
AKHWVGDGGTTYGTGDHDYVIDRGDTRVSEQELRDIHIAPYLNAFKQDVGSVMYSYSSVNGLKMHENARINNDILKGELGFDGFVISDWQAIEEIAAETNRERIVKAIN
AGLDMAMEPEFWREYITDITAAVNDGEIPMARIDDAVTRILLQKVRLGLFDSPMAADRTTDFDGVLGNQAHRDIAKQAVRESQVVLKNEGILPLNKGSKILVAGSHADD
IGLQSGGWTIEWQGGEDITQGQTILDGIKQHASDVTFSKDGSGAAGHDVAVVVVGEKPYAEGAGDYDVQPCEHCQPLTLSDEQLATIAKVREAGVPVLVVLVSGRPLL
ISDELPEWDGLVAAWLPGSEADGVADVLFGDFKPKGKLPVSWPSTLEDVNKNTGDAGYQPLFDYGYGLTF
>tr|G0GCI8|G0GCI8_SPITZ
MKNHKPVWLLMIVFLLFPTWGCGVKGDPSRGRFRDSSLSPEERARDLLSYMTIEEKIGQMAMVDRGYLKSPHDIAEYGLGAILSGGGSAPRRNTPESWKEMVDGFQREA
LGTRLGIPILYGIDAVHGHNNVHGAVIFPHNIGLGATGDPELVERIGRAVAEEVVATGIHWTFAPCVTVPQDERWGRTYEGFGEDPELVARLGAALIRGFQGVPAPESL
ARPDTILATAKHFVADGGTTGGKDRGDARLTEEELRKVHLRPYVEAVKAGVGSVMVSFSSINGVKMHANRDLIQGVLRGELGFDGLIVSDWAAHTELPGSLEEKLATVI
NAGVDMIMIPDDYRGFVAAVKSLVEEGVVSRKRIDEAVYRILLTKFRLGLFERPIQEDVDFSMVGSEPHRALAREAVRKSVVLLKNDGGVLPLKKEGTRILVLGDKADD
LGVQCGGWTITWQGKRGRVTEGTTILEAIRKAVSDPSLVTHVRRASQLAQVKADVIIVVVGETPYAEMYGDRQDLSLTREDAELIIHASQTGLPVVVVLVSGRPRIITD
LLDSMDALLAVWLPGTEGDGIADVLFGDYAPTGKLPFVWPRSMEVLPLTIEESGHHPEKALFPYGYGLSY
>tr|G4QEA4|G4QEA4_GLANF
MTKFTFKYPIAFGACLVLMQACTLQKTASPALTEKPKVQCIWGSDKFDICRYDSKEAIINALIAEMTVDEKIGQMTQSVWHNSVSPEIIQDRKIGSIIHTEGPTPGPKV
SDWVAKFNTFQAHALKTRLGIPLLIGVDAIHGQNTFEGAVIFPHNIGMGATRNYDLIRRAAEITAIETAGTGFNWTFSPVIAMPEHEHWGRVYEGFSEDANVTTKALIA
SIQGHQGTDLAQAYTIAATAKHYLGDGATVGGREGGNAIISEKALRERFLPPYQAAVNHGISAIMVGFNSVNGTNMHQNTYLVQDVLKGQLGFDGVVITDWLGGTRWGE
PHTVINAGIDIAMQPANHDEFMAKLKETVLDGTVSMERIDDAVRRILGLKFDLGLFNDPFAKKELSALVGSTQHREVARQAVRESLVLLKSEANALPLKANESIAVVGE
HANNSGLQSGGWTMHWQGQTHSYANSTTILDGIQAFAPEVQYQPMGCTSDTQAEKVVAVVGELPYAEFKGDSTNLALTVAQQEMIKHCKALGKKVIVVLISGRAMTVTD
TINQSDAFIAAWLPGSEGMGIADFLFAANGFEPVGKLPTSWPKEYADLPLAQDAENALFPFGFGLSKF
>tr|A0A5B9WCA9|A0A5B9WCA9_9BACT
MTMTARMVTCLLVWAAALLPMVAGVRACAADDDAAAKKADGWLAAMTLDEKIGQMTQVDFKAFKDLGDITRLSLGSVLCGGNSDPDDITPAGWAKAQDDCQALALKARL
KIPLIFGVDAVHGHNNVDGAVIFPHNVGLGASRDPSVVEKAARVTALEMVGTGIRWAFAPCVAVARNERWGRTYESFGERPELAAELGPAAVRGLQGDSLAAADSVLAC
VKHFVGDGGTTNGVDQGNTECDEAALRQIHLPGYVASIKQGAGSIMASYSSWNGKKLHGHKYLVTDLLKGELGFGGFVVSDWAGVDQLSPDFKAAIDQSINAGVDMVMI
PNGPGQKNNYVEFIDKLKELVNEGKVPASRIDDAVRRILLIKARMNLADHPYSDPALKAKVGSAEHREVARDCVRKTLVLLKNDRKVLPLSKSIKKLVVAGPAANDIGI
QCGGWTIAWQGKAGDVIHGGTTVLAALKEALGPGVEVVHSADGSGAAGADAAVVVIGERPYAEMFGDRRGGKDLGLPADDLAALKKVREAGIPVATVVFSGRPVLLGPV
LESSDAILAAWLPGTEGRGIADVLLGDYKPTAKLPHTWPRSMEQIPCNPEDGAASEALFPFGFGLSY
>tr|A0A1E7JN49|A0A1E7JN49_9ACTN
MQQTPPPQSTTRRQILAGAAGAAGIAAATALPSGTAHAATAADDARTRRAARALLGRMSLKEKIGQLFVVEVYGKSADSAHDKNKELYGVSTPAEVVAKYRPGGVIYFD
ARRGPDNLQQPRQIAGLSNGLQRAALRTGARIPLLVSIDQEGGSVVYRMLEPATQLPGNMALAAARSRGDVRRSSEIIGTELAAMGINQNYAPVADVNINPDNPIIGVR
SFGSDPALCSDLVAASVRGYHRGEVSSAAKHFPGHGDTDVDSHTGLPVIKHTREELEKIDLPPFRAAIARGVDTIMTAHIVVPSLDSSGVPATMSKPIVTGLLREELGF

RGLIVTDALDMGGATEDFPPDVAPVRALKAGCDQLVLAPKADTAHAAVLKAVESGDVPEDRVDASVLRILEHKLRGGLFPRPYVDEDRAARVVGSRRHIADAARITDRT
VTLVRNEGGTLPLSPGARTVLVTGWDTDRVGILARTVGERSGQEATALATGATPGATQIEDAVTAAGDHDVTVVLTNAAASAKDKGAAQADLVKALVKTGKPVVAVAVR
NAYDIRRFPGVPACLATFSYGKPSLDSVVRALYGDVNPSGKLPVSIPALDEDEGTLYEFGHGLSY
>HvExoI
DYVLYKDATKPVEDRVADLLGRMTLAEKIGQMTQIERLVATPDVLRDNFIGSLLSGGGSVPRKGATAKEWQDMVDGFQKACMSTRLGIPMIYGIDAVHGQNNVYGATIF
PHNVGLGATRDPYLVKRIGEATALEVRATGIQYAFAPCIAVCRDPRWGRCYESYSEDRRIVQSMTELIPGLQGDVPKDFTSGMPFVAGKNKVAACAKHFVGDGGTVDGI
NENNTIINREGLMNIHMPAYKNAMDKGVSTVMISYSSWNGVKMHANQDLVTGYLKDTLKFKGFVISDWEGIDRITTPAGSDYSYSVKASILAGLDMIMVPNKYQQFISI
LTGHVNGGVIPMSRIDDAVTRILRVKFTMGLFENPYADPAMAEQLGKQEHRDLAREAARKSLVLLKNGKTSTDAPLLPLPKKAPKILVAGSHADNLGYQCGGWTIEWQG
DTGRTTVGTTILEAVKAAVDPSTVVVFAENPDAEFVKSGGFSYAIVAVGEHPYTETKGDNLNLTIPEPGLSTVQAVCGGVRCATVLISGRPVVVQPLLAASDALVAAWL
PGSEGQGVTDALFGDFGFTGRLPRTWFKSVDQLPMNVGDAHYDPLFRLGYGLTTNATKKY
>tr|D3PUC5|D3PUC5_STANL
MPRPRLFTTLALTAVLALTGCAPTASDDDGDTKVDASAKARDWAERQLKSLSLEEKVGQMFTHYAYGQTADTTESADVKRNQKLHGVDNAKQLIEKYHLGGIIYFGWSN
NLANPGQVAGLSNGMQKTAMSQGGEIPLLVSTDQETGTVVRLGPPATEWPGNMALGAGRDRGDARDTAAIAGVELRAVGINQNFAPSGDVNVNPQNPVIGVRSYSSDPK
LVAKFTGEQVKGYQGRHGTSATVKHFPGHGDTHEDSHTDLPRIEHTKEQWKKLDAPPFKAAIKSGVDTVMSAHIQFPALDASEKPATLSKPILTGLLREELGFDGVIVT
DSLGMQGVRELYTDAEIPVMAIKAGVDQLLMPADLKVAYEAVLAAVKSGEITEKRIDKSVKRLLTLKYLRGVVKNPYVDPDKVDDTVGTEDHLATAQAITDKTTTLVKN
DGDALPLSKDTGKVFVAGDGANTTSILAKQIGKLGPATEALNTGADPNAATISGAVEKAKQADVAVVATNTVRAHPAQADLVKALRDSGTKVVVVGVKEPYDINRFPEV
DSYVASYGYNTPVLTAVAKVLFGEVDPRGKLPVTIPKADDPDATLYEFGHGLSYE
>UniRef90_A9NUD1_27_625
YAKYKDPSQPIIARVEDLLARMTVEEKIGQMTQIERSDATADVMKKYYIGSVLSGGGSVPAPKASPATWINMVDDLQKGAMSTRLQIPMMYGIDAVHGHNNAYGATMFP
HNIGLGATRDPDLARRIGAATALEVRATGIQYTFAPCVAVCRDPRWGRCYESYSEDPKIVKAMTQIIFGLQGQPPANSTKGVPFIAGQSNVAACAKHFVGDGGTTNGID
ENNTVIDYKGLVNIHMTPYFDAIAKGVSTIMVSYSSWNGMKMHANRFLVSEVLKKQLGFKGFVISDWQGIDRITSPPGANYSLSVFDGVGAGIDMVMVPENFTNFITEL
TSQVKGGLISMTRINDAVRRILTVKFTMGLFEYPMADPSLANHFGSKEHRELAREAVRKSLVLLKNGKSAGKPLLPLDKNAPKILVAGTHPNNLGYQCGGWTIEWQGLS
GNSTIGTTILQAIKFAVSPSTEVIYQQNPDANYVKGQGFSYAIVVVGEAPYAEMNGDNLNLTIPLGGGDTIKNVCSSLKCLVILISGRPLVIEPYLPLVDAFVAAWLPG
TEGQGVTDVIFGDYGFQGKLPRTWFKSVDQLPMNVGDKHYDPSFPLGFGLTTTV
>UniRef90_W1PPJ1_6_602
IYKDPQQAIEERVKDLLLRMTIAEKVGQMTQIERSVVTLDAMKNQFIGSILNAGGSTPRSGASAEEWADMVDGFQRWALESRLSIPVLYGTDAVHGHNNCYGATIFPHN
IGLGATRDPNLVQKIGEATAIEVRATGIPYTFAPCVAVSRDPRWGRCYESYSEDTETVRKMTTIVQGLQGSPPQSHPKGYPFLASRKNVIACMKHFIGDGGTKGGINEG
NTVGSFDELHSVHLKPFLDCLDQGVCTAMASYSSWNSMMLHSHHFLLTQVLKHQLGFKGFVISDWEGIDRLCQPQGSDYRFCISASINAGIDMVMVPHDFQKFIGDLTF
LVESGEISMTRIDDAVERILRVKFVGGLFEHPFTDRSLLNLVGCKMHRELAREAVRKSLVLLKNGKNPNKPFLPLSKNGGRILVGGEHAHNLGYQCGGWTITWYGIGGQ
ITQGTTILEAIKAAVGMNTAVIYEENPTETSFKTQEFSSAIVVVGEKPYAEFLGDDPKLELSPKAIETIELVCSKVPTLVILLSGRPLIVEPLIEKMEAFVAAWLPGSE
GAGVADVIFGGYEFHGCLPRTWFKRVDQLPMNVGDSNYDPLFPFGFGLKMNL
>UniRef90_W1NE16_9_604
VYKDPKASLNKRIHDLMSQMTLAEKIGQMTQIERQVANYSVMKEYAIGSILSGGGSVPSPQASAAVWVNMVNEFQRGALASRLQIPMIYGIDAVHGHNNVYGSTIFPHN
VGLGVTRDPDLLKRIGAATALEVRATGIPYTFAPCIAVCRDPRWGRCYESYSEDPEIVEAMTEIIPGLQGDAPKKGVPFVAGKTKVAACAKHFVGDGGTHNGINENNTI
IDRHGLLAIHMAGYYHAIIKGVSTVMVSYSSWNGEKMHANRDLVTNFLKKTLHFRGFVISDWQGIDRITSPAGSNYPYSVHAGVNAGIDMIMVPYNFTEFINDLTEQVN
SKSIPMSRIDDAVRRILRVKFQMGLFENPLADLSLADQLGSQEHRELAREAVRKSLVLLKNGKEGDKPLLPLDKKAPKILVAGTHADNLGYQCGGWTIQWQGQSGNTTK
GTTILTAIKSTVSPSTQVVFEENPSASSLKGQDYDYAVVVVGETPYAETNGDSMNLTMPEPGPTIIKNVCGSVKCVVVVISGRPIVLQPFLQYIDALVAAWLPGTEGQG
VADNLFGDYPFTGKLARTWFKSVDQLPMNVGDKHYDPLFPFGFGLTTEKTS
>UniRef90_W1NE06_27_621
TYKDPKAPLQSRIEDLLKQMTLAEKIGQMTQIDRSVATQDVMKNYSIGSVLSGGGSIPKPNATVEDWVNMVNDFQKGALSSRLQIPMIYGIDAVHGHNNVYGATIFPHN
IGLGATRDPELAKRIGVATALEVRATGIPYVFAPCLAVCRDPRWGRCYESFSENPEIVEAMTEVIVGLQGGEPSQKGTPFVTGKQSVAATAKHFVGDGGTVRGIDENNT
VIDYHDLLSIHLRPYYRAIIKGVSSVMVSYSSWNGVKMHSNRKLVTGLLKSTLRFRGFVISDWQGIDRITSPPGSNYMFSVHAGVNAGIDMIMVPYNYTDFINDLTDQV
NSKSIPMSRIDDAVRRILRVKFQMGLFENPMADLSLKDHFGSRKHRELAREAVRKSLVLLKNGKDGDKPLLPVDKKAPKILVAGTHADNLGYQCGGWTITWQGGSGNTT
IGTTILSAIRSTVDPHTEVVYQENPDPSSLKANDYSYGIVVVGELPYAEFDGDSTTLTMIEPGPTIIKNVCASMKCVVVVVSGRPIVLEPYVPYMDALVAAWLPGTEGQ
GLADVLFGFYPFSGKLPRTWFKSVNQLPMNVGDKHYDPLFPFGFSLTTDI
>tr|B9TBT5|B9TBT5_RICCO
MAEGGNDFCRRCDFMSCADGFRNCRPGAVRPPSPLFAEDMQVDRLATIRNRIALLCATAVCCGGALAAEPWMNAQLPADERAALVLREMTQDEKLKLVFGYLGADHEQK
KTKRPEPSHNQSAGFVYGVPRLGIPHLWETDAGLGVASQAGPNVRQATALPSGLNTAATWDVDTAYAGGAMIGAEARARGFNVMLAGGVNLMRDPRNGRNFEYGGEDPL
LAGRMVGAQIRGIQSNHVVSTLKHFALNDQEIGRTTLNVLISEQAAVTSDLLALQIANEEGNPGAVMCAYNRVNGVYSCENSWLLNDVLKGDWGFTGWVMSDWGAVHST
VPAANAGLDQQSGMPFDLADYFGAPLKEAVTNGWVPQARLDDMARRVLRTMFEHGVVDHPVAPAPESIDFRKHAAVSMKDAQEGMVLLKNAAQALPLQRTAKRIAVIGG
HADKGVLAGGGSSLVYPVGGNAVPGIAPTTWPGPVMYYPSAPLEAIRRRAPGATVTYADGADRAAAAALARDSDAVVVFATQWTGEGVDAPDLALPGGQDDLIAAVAAA
NPKTVVVLETGGPVTMPWLPNVAAVLEAWYPGTSGGDAIAGILFGEVNP
>UniRef90_A0A176WE76_43_636
LYKDASQPVELRVQDLLSRMTVQEKIGQMTQIERTVATPEVMTQYFIGSVLSGGGSAPEPNAPAAWQDMIDTMQQAALATRLAIPMIYGIDAVHGHNNLYGATVFPHNI
GLGCSRDPDLVKRIGAATALEVRATGIPYAFAPCIATCRDPRWGRCYESYSEDTAVVKTMTDIILGLQGDPPNLTAGVPFMADKSKVIGCAKHYVGDGGTFKGINENDT
IVDYDTLYKVHMAPYLDAIAKGVSTIMVSYSSWNGERMHANQYLVTQVLKEQLAFRGFIISDWMGVDRLSDPNNPNYTNSVLKSINAGLDMIMVPFDYEAYISGMLSLV
NDGEISMERIDDAVTRILRVKFVMGLFEDPSVDRSLTNHLGSQEHRILAREAVRKSLVLLKNGQQGSQALLPLKKNATSILVAGSHADDIGLQCGGWTISWVGAAGNTT
IGTTVLDAIKAAVSPTTVVTYEKNPAPGFAAQLKPDYAIVVVGEEPYVETYGDNMELTIPLDGIPTIQNVCAEVKCLVIVISGRPLVIEPYMPQIDALVAAWLPGSEGQ
GISDVIFGDYDFVGKLSRTWFRTVDQLPMNFGDAVYDPLFPFDFGLTMG
>UniRef90_A0A2N2MB45_49_637
LYTDSSLSAEERASDLLARMSLDEKIGQMTQVEKNSILPGEVSRYYIGSILSGGGGAPTGDDSLEGWVKMVDGLQAAALETPLAIPLIYGVDAVHGHNNVKGATIFPHN
IGLGATNDPELVEKIGRATAEEMLATGISWDFAPVLAVVQDIRWGRTYESYGENTELVTRLGVAYQNGLQAAGDGDSIFVLATPKHYIGDGGTTWASSTTDNYKLDQGD
TQMDEARLRELFLPPYQAAVEAGAQSVMVSYSSWNGVKMHGHKYLITDVLKGELGFEGFVVSDWAGIDQVDSDYYTAVVTAINAGVDMNMVPQGYPRYLTVMQQAVEKG
DIPMERIDDAVLRILTVKFKLGLFEKPFADPAYLETVGSQAHRDLAREAVAKSLVLLKNDNATLPLAKDAGLIFVAGASANDIGAQCGGWTIEWQGKSGNITTGTTILE
AIEASASAEVRFDRFGKFESEQKADVAVVVIGERPYAEGRGDKENPSLSKSDIELIQRVREQSQRVVVILLSGRPLVITEALPYADAFVAAWLPGTEGSGVADVLFGDK
PFTGKTPFSWPRSADQLPFDFANLPADGCAAPLFPYGYGLDVTS
>UniRef90_A0A2R6W019_43_631
IYEDSNQPVELRVQDLLSRMTLDEKIGQMTQIERYVANFDVMKNFSIGSVLSSGGSTPNSSTTEAWQNMVDDLQAGALATRLGIPMLYGIDAIHGHNNVYGATIFPHNI
GLGCTRDPDLVRRVGAATALELRATGIPYTFAPCIAVCRDPRWGRCYESFSEDTSVVRMMTDVIYGLQGNGTPGIPFVSDRSKVAACAKHYVGDGGTQRGINSNDTILS
YEDLFRIHVAPYVDAIAKGVSTIMLSYSSWNGVKMHMNHRLISTLLKQELGFKGFVISDMEGIDFITDIPDANYTASVLESINAGLDMIMVPFDYEKFISTLRTLVNTG
YISMQRIDDAVTRILRVKFSMGLFEHPLADRSFSPHLGSQDNRMVAREAVRKSLVLLKNGKTESRPFLPLSKNATRVLVAGTHADDVGLQCGGWTISWQGSAGSITKGT
TVLDAVKAAVFTTTQVIHEASPTAEVAAKTKADFAIVVVGEQPYAEGAGDNTNLTIPDEGISTIKNVCSEVKCLVILISGRPLVVEPYLPLMEAFVAAWLPGTEGNGVT
DVIFGDYDFVGSLSRTWFKSADQLPMNFGDPIYDPLFRFAFGLG
>tr|B9TDB0|B9TDB0_RICCO
RSPRCSALASKTEIQDAKGEFVAAKAQKVYPHGLGMIGRPSDRQLGQAAGAGDTGAQANRNALETATYVNAVQKWAVEQTRLGISLFMHEEALHGYVARDATSFPQAIG
IASSFDPDLTTKIFSVAAREMRARGANLALAPVVDVAREPRWGRIEETYGEDPYLCGEIGKAAIIGFAGTDPKLPKDKVLVTLKHMTGHGQPESGTNIGPAEVSERTLR
EEFFPPFEKAIKEANVGCVMPSYNEIGGVPSHANHWLLHKVLREEWGFKGITVSDYFGINELITRHKLAATPKEAALRAIKAGVDVETPDGLAYKTLGELVKEKRVSEA
EIDTVVRRILTLKFQLGLFENPYVDAKAADNLTATPDAVALARLAATRTPVLLKNDKGLLPLDGKKVGKVLLIGTHAKDTPIGGYSDHPRHVVSIHDGLQAEAKAQGFS
LAYSEGVRITESRVWGADEIKFTAPEVNARLIADAVAAAKSADTIIMVLGDNEQTSREAWADNHLGDRESLDLMGQQNDLARAIFALGKPTVVFLLNGRPLSINLLAEK
ADAIIEGWYMGQETGNAAADLLFGRANPGGKLP
>AUC61062.1
MKNLSLKEKIGQLIVVRTTGYLFDHQIRYPAWEANQQQLQTWLSEYNIGGVILLGGSCAEIAQRTKQLNQWAKTPLLVAADIEEGVGQRFTGASWFPPPMALAQIAQDD
LELAKKYAEEMGKITAKEALCIGVNWIFAPVVDVNNNPDNPVINVRAFGDNPEVVKELSSAFIRGTQSYPILNGAKHFPGHGDTSTDSHLDLPVINHSQARLEKIELVP
FQGAIALNVDAIMTAHLLVSAYDNQNPATLSHRILTEELRHNMGFEGLIVTDALIMGGVAKYAPPEKIAVKALQAGADILLMPENPVVAIHSIIEAVEKGEISEHRIDE

SLQRVSKAKEKLFKSQESFSFVDISTNDSQQVINEILVKSNQTSVLKPIVGLDKGINLVVVNNLLNCDFLDRQSPSITIPDSFGYHAQVFDQCNLYIWENQLITEPFIL
QVFVRGNPFRGTAGLSAIALKAYEQILENHHLQGIMVYGSPYVLDWFKNQIPTPIPWGFSYGQMAIAQRTICNKMFQLSTNLDITKGNFL
>AFZ46311.1
MKNLSLQEKIGQLIVVRTTGYLFDHQIRYPAWEANNQQLQTWLSEYNIGGVILLGGSCAEIAQRTKQLNEWAKTPLLVAADIEEGVGQRFTGASWFPPPMALAQIAKKD
LALAKKCAEEMGKITAQEALSIGVNWILAPVVDVNNNPDNPVINVRAFGDNPGVVRELSSAFIRGTQYYPILNGAKHFPGHGDTSTDSHLDLPIINHSRTRLEAIELIP
FQGAIALGIDAIMTAHLLVSAYDNQNPATLSHAILTKQLREKMGFDGLIVTDALIMGGVAKYAPPEKIAVKALQAGADILLMPENPLVAINSIMEAVQRGEITESRIDE
SLQRIAKAKAKLSDGDFSFLQISTSTSQEVVNDILDQSNQIQVFEGIKKVDQGINIVVVDDLLNCDFLDRQTPAITIPATFGYHAQVFDQRNLHIWENQPINQPFILQV
FIRGNPFRGSAGLSAIALTTYTKLLQHPEIQGIIVYGSPYIKDWFTKQLKPNLPWGFSYGQMAIAQYILSQKLFQISESLDISKGNFL
>UniRef90_E0Z601_2_481
VKKYYIGSVLSGGGSVPAPKASPATWINMVDDLQKGAMSTRLQIPMMYGIDAVHGHNNAYGATMFPHNIGLGATRDPDLARRIGAATALEVRATGIQYTFAPCVAVCRD
PRWGRCYESYSEDPKIVKAMTQIIFGLQGQPPANSTKGVPFIAGQSNVAACAKHFVGDGGTTNGIDENNTVIDYKGLVNIHMTPYFDAIAKGVSTIMVSYSSWNGMKMH
ANRFLVSEVLKKQLGFKGFVISDWQGIDRITSPPGANYSLSVFDGVGAGIDMVMVPENFTNFITELTSQVKGGLISMTRINDAVRRILTVKFTMGLFEYPMADPSLANH
FGSKEHRELAREAVRKSLVLLKNGKSAGKPLLPLDKNAPKILVAGTHPNNLGYQCGGWTIEWQGLSGNSTIGTTILQAIKFAVSPSTEVIYQQNPDANYVKGQGFSYAI
VVVGEAPYAEMNGDNLNLTIPLGGGDTIKNVCSSLKCLVILISG
>UniRef90_W1P4N1_9_377
VYKDPKAPLNNRIHDLMSQMTLAEKIGQMTQIERLVANYSVMKEYAIGSILSGGGSVPSPQASAAVWVSMVNEFQRGALASRLQIPMIYGIDAVHGHNNVYGSTIFPHN
VGLGVTRDPDLLKRIGAATALEVRATGIPYTFAPCIAVCRDPRWGRCYESYSEDPEIVKAMTEIIPGLQGDAPKKGVPFVAGKTKVAACAKHFVGDGGTHNGINENNTI
IDRHGLLSIHMGGYYHAIIKGVSTIMVSYSSWNGEKMHANPDLVTNFLKKTLHFRGFVISDWQGIDRITSPAGSNYPYSVYAGVNAGIDMIMVPNNFTEFINDLTEQVN
NKSIPMSRIDDAVRRILRVKFQMGLFENPLADLSLADQLGSQ
>UniRef90_UPI0009BF64A3_53_245
LYKDPKAPLEERVADLLSRMTLAEKIGQMTQIDRSTANPELLMRLNIGSVLSGGGSIPAPKASPAMWADMIDGLQNAALATRLGIPIIYGIDAVHGHNNVYGATIFPHN
IGLGATSDRDLVRRIGKATALEVRATGIPYTFAPCLAVCRDPRWGRCYESYSEDTEVVRSMADIILGLQGVPPPSHPRGYPFMA
>HvExoI
DYVLYKDATKPVEDRVADLLGRMTLAEKIGQMTQIERLVATPDVLRDNFIGSLLSGGGSVPRKGATAKEWQDMVDGFQKACMSTRLGIPMIYGIDAVHGQNNVYGATIF
PHNVGLGATRDPYLVKRIGEATALEVRATGIQYAFAPCIAVCRDPRWGRCYESYSEDRRIVQSMTELIPGLQGDVPKDFTSGMPFVAGKNKVAACAKHFVGDGGTVDGI
NENNTIINREGLMNIHMPAYKNAMDKGVSTVMISYSSWNGVKMHANQDLVTGYLKDTLKFKGFVISDWEGIDRITTPAGSDYSYSVKASILAGLDMIMVPNKYQQFISI
LTGHVNGGVIPMSRIDDAVTRILRVKFTMGLFENPYADPAMAEQLGKQEHRDLAREAARKSLVLLKNGKTSTDAPLLPLPKKAPKILVAGSHADNLGYQCGGWTIEWQG
DTGRTTVGTTILEAVKAAVDPSTVVVFAENPDAEFVKSGGFSYAIVAVGEHPYTETKGDNLNLTIPEPGLSTVQAVCGGVRCATVLISGRPVVVQPLLAASDALVAAWL
PGSEGQGVTDALFGDFGFTGRLPRTWFKSVDQLPMNVGDAHYDPLFRLGYGLTTNATKKY

>Hv_Exo_II
MGNLHKTTFVLLMFCLAALGSADYLKYKDPKQPLGVRIKDLLGRMTLAEKIGQMTQIERENATAEAMSKYFIGSVLSGGGSVPSPQASAAAWQSMVNEMQKGALSTRLG
IPMIYGIDAVHGHNNVYKATIFPHNVGLGATRDPMLVKRIGEATALEVRATGIPYAFAPCIAVCRDPRWGRCYESYSEDPKVVQSMTTLISGLQGDVPAGSEGRPYVGG
SKKVAACAKHYVGDGGTFMGINENDTIIDAHGLMTIHMPAYYNSIIRGVSTVMTSYSSWNGKKMHANHFLVTDFLKNKLKFRGFVISDWQGIDRITSPPGVNYSYSVEA
GVGAGIDMIMVPFAYTEFIDDLTYQVKNNIIPMSRINDAVYRILRVKFTMGLFESPYADPSLVGELGKQEHRDLAREAVRKSLVLLKNGKSASTPLLPLPKKAGKILVA
GSHADDLGNQCGGWTITWQGQTGNDKTAGTTILSAIKSTVDPSTEVVFSENPDSAAVDSGKYDYAIVVVGEPPYAETFGDNLNLTIPAPGPSVIQNVCKSVRCVVVLIS
GRPLVVEPYISAMDAFVAAWLPGSEGQGVADVLFGDYGFSGKLARTWFKSADQLPMNVGDKHYDPLFPFGFGLTTEAKK

>Zm_Exo_II
MALLTAPAVAALLLLFWSSAYGGDAQGEGVPPPYQDASKDVEVRVRDLLARMTLAEKVGQMTQIERIVASPQALRDYYIGSLLSGGGSVPRKQATAAEWVAMVSDFQKA
CLSTRLGIPMIYGIDAVHGHNNVYGATIFPHNVGLGATRDPNLVKRIGAATALEVRATGIQYAFAPCIAVCRDPRWGRCYESYSEDHRIVQAMTELIPGLQGDVPQNFT
SGMPFAAGKDKVAACAKHFVGDGGTQNGINENNTIIDRQGLISIHMPAYLDALRKGFSTVMISYSSWNGLKMHANHNLITGFLKDRLNFQGFTISDWEGIDRVTSPPGA
NYSYSVQASILAGLDMIMVPNNYQNFITILTGHVNSGLIPMSRIDDAVTRILRVKFTMGLFENPMPDPSLADQLGKQEHRDLAREAVRKSLVLLKNGKPGDAPLLPLPK
KAARILVAGSHADNLGYQCGGWTIEWQGDTGRTTVGTTVLDAVKAAVDPSTEVVFAESPDAEFVRSGGFSYAIVAVGEHPYTETKGDSMNLTIPDPGPSTVQTVCAAVR
CVTVLISGRPVVIQPFLGAMDAVVAAWLPGTEGQGVTDVLFGDYGFTGKLPRTWFRSVDQLPMNYGDAHYDPLFPLGFGLTTQGKMYQN

>Nt_Exo
MGRMSIPMMGFVVLCLWAVVAEGEYVKYKDPKQPVGARIKDLMKRMTLEEKIGQMTQIERKVATADVMKQNFIGSVLSGGGSVPAPKASAQVWTNMVDEIQKGSLSTRL
GIPMIYGIDAVHGHNNVYGATIFPHNVGLGVTRDPDLVKRIGAATALEVRATGIPYAFAPCIAVCRNPRWGRCYESYSEDHRIVRSMTEIIPGLQGDLPAKSKNGVPYV
GGKTKVAACAKHFVGDGGTLHGVDESNTVISSNSLFSIHMPAYYDSLRKGVATVMVSYSSWNGRKMHANRDLVTGFLKDKLKFRGFVISDWQGIDRITDPPHANYSYSV
QAGIMAGIDMIMVPENYREFIDTLTSQVKANIIPMSRIDDAVKRILRVKFVMGLFENPMSDPSLANQLGSQEHRELAREAVRKSLVLLKNGKTPSQPLLPLPKKAPKIL
VAGTHADNLGYQCGGWTIEWQGVAGNDLTIGTTILTAIKKTVDPSTQVVYQQNPDANFVKSNKFSYAIVVVGEVPYAEMFGDSSNLTIAEPGPSTISNICGSVKCVVVV
VSGRPVVLEPYVSKMDALVAAWLPGTEGQGVADALFGDYGFTGKLARTWFKRVDQLPMNFDDAHVDPLFPFGFGITTKPVKGY

>Tm_bglu
MGRFLLPILGWFLLLSCLSAFTEAEYMRYKDPKKPLNVRIKDLMSRMTLAEKIGQMTQIERKEATPDVISKYFIGSVLSGGGSVPAPKASPEAWVDLVNGMQKAALSTR
LGIPMIYGIDAVHGHNNVYNATIFPHNVGLGVTRDPALIKRIGEATALECRATGIPYAFAPCIAVCRDPRWGRCYESYSEDHTIVQAMTEIIPGLQGDVPPDVKKGVPF
VGGKTKVAACAKHFVGDGGTTKGIDENNTVIDSRGLFSIHMPAYHDSIKKGVATVMVSYSSWNGLRMHANRDLVTGYLKNKLKFRGFVISDWEGIDRITDPPGRNYSYS
VEAGVGAGIDMIMVPEDFTKFLNELTSQVKKNIIPMSRIDDAVKRILRVKFVMGLFESPLADYSLANQLGSQEHRDLAREAVRKSLVLLKNGESADKPFVPLPKNAKKI
LVAGSHADNLGRQCGGWTIEWQGVNGNDLTTGTTILNAIKKTVDPTTQVIYNENPDSNYVKTNSFDYAIVVVGEPPYAEMQGDSFNLTIPEPGPTTISSVCGAVKCVVV
VISGRPVVLQPYVSYMDALVAAWLPGTEGQGVTDVLFGDYGFTGKLARTWFKTVDQLPMNVGDKHYDPLFPFGFGLTTKPSNRTEFIGLIFGDLEMFSRYYVEGCKDGV

>At_Exo_I
MVVEESSCVYKNGDAPVEARVKDLLSRMTLPEKIGQMTQIERRVASPSAFTDFFIGSVLNAGGSVPFEDAKSSDWADMIDGFQRSALASRLGIPIIYGTDAVHGNNNVY
GATVFPHNIGLGATRDADLVRRIGAATALEVRASGVHWAFSPCVAVLRDPRWGRCYESYGEDPELVCEMTSLVSGLQGVPPEEHPNGYPFVAGRNNVVACVKHFVGDGG
TDKGINEGNTIASYEELEKIHIPPYLKCLAQGVSTVMASYSSWNGTRLHADRFLLTEILKEKLGFKGFLVSDWEGLDRLSEPQGSNYRYCIKTAVNAGIDMVMVPFKYE
QFIQDMTDLVESGEIPMARINDAVERILRVKFVAGLFGHPLTDRSLLPTVGCKEHRELAQEAVRKSLVLLKSGKNADKPFLPLDRNAKRILVTGTHADDLGYQCGGWTK
TWFGLSGRITIGTTLLDAIKEAVGDETEVIYEKTPSKETLASSEGFSYAIVAVGEPPYAETMGDNSELRIPFNGTDIVTAVAEIIPTLVILISGRPVVLEPTVLEKTEA
LVAAWLPGTEGQGVADVVFGDYDFKGKLPVSWFKHVEHLPLDAHANSYDPLFPFGFGLNSKPVSDVIFGDYDFKGKLPVSWFKRVDQLPLNAEANSYDPLFPLGFGLTS
NFG

>At_Exo_II
MEGSNETCVYKNKDAPVEARVKDLLSRMTLPEKIGQMTQIERVVTTPPVITDNFIGSVLNGGGSWPFEDAKTSDWADMIDGYQNAALASRLGIPIIYGIDAVHGNNNVY
GATIFPHNIGLGATSLVMLLHIDLEPKSLGRNKVVVKCDRDADLIRRVGAATALEVRACGAHWAFAPCVAALRDPRWGRSYESYSEDPDIICELSSLVSGLQGEPPKEH
PNGYPFLAGRNNVVACAKHFVGDGGTDKGINEGNTIVSYEELEKIHLAPYLNCLAQGVSTVMASYSSWNGSKLHSDYFLLTELLKQKLGFKGFVISDWEALERLSEPFG
SNYRNCVKISVNAGVDMVMVPFKYEQFIKDLTDLVESGEVTMSRIDDAVERILRVKFVAGLFEHPLTDRSLLGTVGCKEHRELARESVRKSLVLLKNGTNSEKPFLPLD
RNVKRILVTGTHADDLGYQCGGWTKAWFGLSGRITIGTTLLDAIKEAVGDKTEVIYEKTPSEETLASLQRFSYAIVAVGETPYAETLGDNSELTIPLNGNDIVTALAEK
IPTLVVLFSGRPLVLEPLVLEKAEALVAAWLPGTEGQGMTDVIFGDYDFEGKLPVSWFKRVDQLPLTADANSYDPLFPLGFGLNYNSSENV

>At_Exo_III
MAETEESSWVYKNRDAPVEARVKDLLSRMTLPEKIGQMTQIERSVASPQVITNSFIGSVQSGAGSWPLEDAKSSDWADMIDGFQRSALASRLGIPIIYGTDAVHGNNNV
YGATVFPHNIGLGATRDADLVKRIGAATALEIRASGVHWTFAPCVAVLGDPRWGRCYESYSEAAKIVCEMSLLISGLQGEPPEEHPYGYPFLAGRNNVIACAKHFVGDG
GTEKGLSEGNTITSYEDLEKIHVAPYLNCIAQGVSTVMASFSSWNGSRLHSDYFLLTEVLKQKLGFKGFLVSDWDGLETISEPEGSNYRNCVKLGINAGIDMVMVPFKY
EQFIQDMTDLVESGEIPMARVNDAVERILRVKFVAGLFEHPLADRSLLGTVGCKEHREVAREAVRKSLVLLKNGKNADTPFLPLDRNAKRILVVGMHANDLGNQCGGWT

KIKSGQSGRITIGTTLLDSIKAAVGDKTEVIFEKTPTKETLASSDGFSYAIVAVGEPPYAEMKGDNSELTIPFNGNNIITAVAEKIPTLVILFSGRPMVLEPTVLEKTE
ALVAAWFPGTEGQGMSDVIFGDYDFKGKLPVSWFKRVDQLPLNAEANSYDPLFPLGFGLTSNFG

>Dd_bglu
MKTIKSLFLLSLLIVNLLISSTYGSSIRVSIVGGEEAEVIEKPRTFGNKRELKLEYSQIYPKKQLNQENINFMSARDTFVDNLMSKMSITEKIGQMTQLDITTLTSPNT
ITINETTLAYYAKTYYIGSYLNSPVSGGLAGDIHHINSSVWLDMINTIQTIVIEGSPNKIPMIYGLDSVHGANYVHKATLFPHNTGLAATFNIEHATTAAQITSKDTVA
VGIPWVFAPVLGIGVQPLWSRIYETFGEDPYVASMMGAAAVRGFQGGNNSFDGPINAPSAVCTAKHYFGYSNPTSGKDRTAAWIPERMLRRYFLPSFAEAITGAGAGTI
MINSGEVNGVPMHTSYKYLTEVLRGELQFEGVAVTDWQDIEKLVYFHHTAGSAEEAILQALDAGIICLCHDLLSQLFSLEILAAGTVPESRLDLSVRRILNLKYALGLF
SNPYPNPNAAIVDTIGQVQDREAAAATAEESITLLLFKNNILPLNTNTIKNVLLTGPSADSIRNLNGGWSVHWQGAYEDSEFPFGTSILTGLREITNDTADFNIQYTIG
HEIGVPTNQTSIDEAVELAQSSDVVVVVIGELPEAETPGDIYDLSMDPNEVLLLQQLVDTGKPVVLILVEARPRILPPDLVYSCAAVLMAYLPGSEGGKPIANILMGNV
NPSGRLPLTYPGTTGDIGVPYYHKYSENGVTTPLFQFGDGLSYTTFNYTNLACSNCKPISGQSGNYTGLGQSYTFTVTVTVTNNGNVQGKDSVLLYLSDLWAQVTPEVK
MLRGFQKVDLMPAKSQQISFTLNAYEFSFIGVDNKITLESGPFIIMVGNQQLGLYLQ

>Tb_bglu
MSYGIGQITRLGGASNLSPRETVRIANQIQKFLIENTRLGIPALIHEESCSGYMAKGATIFPQTIGVASTWNNEIVEKMASVIREQMKAVGARQALAPLLDITRDPRWG
RTEETFGEDPYLVMRMGVSYIRGLQTESLKEGIVATGKHFVGYGNSEGGMNWAPAHIPERELREVFLYPFEAAVKEAKLSSIMPGYHELDGVPCHKSKKLLNDILRKDW
GFEGIVVSDYFAISQLYEYHHVTSDKKGAAKLALEAGVDVELPSTDYYGLPLRELIESGEIDIDFVNEAVKRVLKIKFELGLFENPYINEEKAVEIFDTNEQRELAYKI
AQESIVLLKNENNLLPLKKDLKSIAVIGPNADSIRNMIGDYAYPCHIESLLEMRETDNVFNTPLPESLEAKDIYVPIVTVLQGIKAKVSSNTEVLYAKGCDVLNNSKDG
FKEAVEIAKQADVAVVVVGDKSGLTDGCTSGESRDRADLNLPGVQEELIKAIYETGTPVIVVLINGRPMSISWIAEKIPAIIEAWLPGEEGGRAVADVIFGDYNPGGKL
PISIPQSVGQLPVYYYHKPSGGRSHWKGDYVELSTKPLYPFGYGLSYTEFSYTNLNISNRKVSLRDRMVEISVDIKNTGTLKGDEVVQLYIHQEALSVTRPVKELKGFK
RITLDAGEEKTVIFKLSIEQLGFYDENMEYVVEPGRVDVMIGSSSEDIRLRDYFEIVGEKEKVAKKFITEVRVENK

>Te_xyl-ara
MKPLYLDSTQSVEKRVEDLLQQMTIEEKVAQLNSIWVYEILDDMKFSFDKAKRLMSYGISQITRLGGASNLSPRETVRIANQIQKFLIENTRLGIPALIHEESCSGYMP
KGATIFPQTIGVASTWNNEIVEKMASVIREQMKAVGARQALAPLLDITRDPRWGRTEETFGEDPYLVMRMGVSYIRGLQTESLREGIVATGKHFVGYGNSEGGMNWAPA
HIPERELREVFLYPFEAAVKEPKLSSIMPGYHELDGVPCHKSKKLLNDILRKDWGFEGIVVSDYFAISQLYEYHHVTSDKKGAAKLALEAGVDVELPSTDYYGLPLREL
IESGEIDIDFVNEAVKRVLKIKFELGLFENPYINEEKAVEIFDTNEQRELAYKIAQESIVLLKNENNLLPLKKDLKSIAVIGPNADSIRNMIGDYAYPCHIESLLEMRE
TDNVFNTPLPESLEAKDIYVPIVTVLQGIKAKASSNTEVLYAKGCDVLNNSKDGFKEAVEIAKQADVAVVVVGDKSGLTDGCTSGESRDRADLNLPGVQEELIKAVYET
GTPVIVVLINGRPMSISWIAEKIPAIIEAWLPGEEGGRAVADVIFGDYNPGGKLPISIPQSVGQLPVYYYHKPSGGRSHWKGDYVELSTKPLYPFGYGLSYTEFSYTNL
NISNRKVSLRDRMVEISVDIKNTGTLKGDEVVQLYIHQEALSVTRPVKELKGFKRITLDAGEEKTVIFKLSIEQLGFYDENMEYVVEPGRVDVMIGSSSEDIRLRDYFE
IVGEKEKVAKKFITEVRVENK

>Tn_bglu
MEKVNEILSQLTLEEKSETCSGGWTSGVVWKSHSGWRCRGETHPVPRVGLPAFVLADGPAGLRINPTRENDENTYYTTAFPVEIMLASTWNRELLEEVGKAMGEEVREY
GVDVLLGPAMNIHRNPLCGRNFEYYSEDPVLSGEMASSFVKGVQSQGVGACIKHFVANNQETNRMVVDTIVIERALREIYLRGFEIAVKKSKPWSVMSAYNKLNGKYCS
QNEWLLKKVLREEWGFEGFVMSDWYAGDNPVEQLKAGNDLIMPGKAYQVNTERRDEIEEIMEALKEGKLSEEVLDECVRNILKVLVNAPSFKNYRYSNKPDLEKHAKVA
YEAGAEGVVLLKNEEALPLSENSKIALFGTGQIETIKGGTGSGDTHPRYAISILEGIKERGLNFDEELAKIYEDYIKKMRETEEYKPRRDSWGTIIKPKLSENFLSEKE
VHKLAKKNDVAVIVISRISGEGYDRKPVKGDFYLSDDETDLIKTVSREFHEQGKKVIVLLNIGSPVEVVSWRDLVDGILLVWQAGQETGRIVADVLTGRINPSGKLPTT
FPRDYSDVPSWTFPGEPKDNPQKVVYEEDIYVGYRYYDTFGVEPAYEFGYGLSYTTFEYSDLNVSFDGETLRVQYRIENTGGRAGKEVSQVYIKAPKGKIDKPFQELKA
FHKTRLLNPGESEEVVLEIPVRDLASFNGEEWVVEAGEYEVRVGASSRNIKLKGTFSVGEERRFKP

>Hv_bxyl
MGRRTHVVLAAAVPALLLVLLLRLHAAVAADPPFSCGAPSSAAFCDRRLPIEQRAADLVSKLTLEEKISQLGDESPAVDRLGVPAYKWWSEALHGVANAGRGVHLDGPL
RAATSFPQVILTAASFNPHLWYRIGQVIGTEARGVYNNGQAEGLTFWAPNINVFRDPRWGRGQETPGEDPTMTGKYAAVFVRGVQGYGMSGAINSSDLEASACCKHFTA
YDLENWKGVTRFAFDAKVTEQDLADTYNPPFKSCVEDGGASGIMCSYNRVNGVPTCADHNLLSKTARGDWSFNGYITSDCDAVAIIHDVQGYAKAPEDAVADVLKAGMD
VNCGGYIQTHGVSAYQQGKITGEDIDRALRNLFAIRMRLGLFDGNPKYNRYGNIGADQVCSKEHQDLALQAARDGIVLLKNDGAALPLSKSKVSSLAVIGPNGNNASLL
LGNYFGPPCISVTPLQALQGYVKDARFVQGCNAAVCNVSNIGEAVHAAGSADYVVLFMGLDQNQEREEVDRLELGLPGMQESLVNSVADAAKKPVILVLLCGGPVDVTF
AKNNPKIGAIVWAGYPGQAGGIAIAQVLFGDHNPGGRLPVTWYPKEFTAVPMTDMRMRADPSTGYPGRTYRFYKGKTVYNFGYGLSYSKYSHRFASKGTKPPSMSGIEG
LKATARASAAGTVSYDVEEMGAEACDRLRFPAVVRVQNHGPMDGGHLVLLFLRWPNATDGRPASQLIGFQSVHLRADEAAHVEFEVSPCKHLSRAAEDGRKVIDQGSHF
VRVGDDEFELSFMA

>Tb_gln-glc
MPLPRPLRAMPLRRPLRRAPRVTLVLALTAAPARADADLPFRDPTLPLAQRIDDLLGRLTLDEKISLLHQYQPPIERLGIKSFKTGTEALHGVAWSTDVTDNGAVVTAN
GTVFPQAVGLASTWDPELNQRVGTVGEEARGFHAQNPVVWGLNLWAPVVNLLRDPRWGRNEEGYSEDPLLTGAIAIAYGSGIQGDDPDHLRAAPTLKHYLANNNEIRRD
TTSSNLPPRVKHEYYEAPFRAAITAGAATGVMTAYNLVNGRPATVNPDLNDTVRTWTDRDLLNVTDAGAPNNLVGSQAYFATLAEADAAALKAGIDSFTTDETNSAPTI
TAIKTALSQGLLTEQDIDTAVRHILGIRFRLGEFDPDGGPYAKITPDVIDSPAHRRLARETAAKAMVLLKNERGTLPLDPGKKVAVVGPLADVLYTDWYSGRPTYQVTP
LDGIRERAASVTSSEGVDRVAFKDLATGQVHHRGERPEGADLRLSATTIGETEQFDVFDWGQGIVTLRSVANGKYVSRANWSTLVNNADQPSGWFVQEQFKLEEQDDGS
YLIRYAGYETAYDWFGPNTYVKAAPDGTLTLTTAGDATRFAKEVVRSGIDDAVAKAKEADVAVVVVGSMPFINGREDHDRTDMNLAEGQEALVKAVFNANPRTVVVLEN
SYPTTINWIDEHVPAILWTTHAGAETGNALADVLYGDVNPAGRLTQTWYLGGRLPDILDYDIVQRDRTYLYFKGTPLYPFGHGLSYTTFRYQGLRVAEKGDAYEVSVRV
TNTGHRAGDEVVQVYTHQRTSRVKQPVKQLRAFQRITLAPGQSKTVTFTIRKADLALWDVTRNKWTVETSAHDVMVGASSADIRQRATIHVKGERIPDRDLSVPTRAID
FDGYSGVELVDETKARGDAVAGSTGDWIVFKDVDLKRRPSRVTAGVASTSGGSIELRLGSPKGKLIATVPVAATGDVYRYETAAARVTGPSGVKDLYLVFQGDVRIKDL
SLTSG

>Nc_bglu
MKSSWASYCLLSCTSALVSAIDLPFQTYPDCVNGPLASLKVCDATLSPPQRAAALVAAMTTEEKLQNLVSKSKGAPRIGLPAYNWWSEALHGVAYAPGTQFRSGDGPFN
SSTSFPMPLLMAATFDDELIEKVGEVIGTEGRAFGNAGFSGFDYWTPNVNPFKDPRWGRGSETPGEDILRIKRYAASMIRGLQGPLPERRVVATCKHYAANDFEDWNGS
TRHDFDAKVTLQDLAEYYLSPFQQCARDSKVGSIMCSYNAVNGVPACANTYLMQTILREHWNWTAPGNYITSDCEAVLDIFANHHYAKTNAEGTALAFEAGTDSSCEYE
SSSDIPGAWTQGLLEQSTVDRALTRLYEGLVRVGYFDGNHSEYASLGWKDVNSPKSQEVALQTAVEGIVLLKNDQTLPLGLKTDPKSKLAMIGFWANDPKTLSGGYSGK
PAFEHSPVYAAEAMGFNVTTAGGPVLQNSTSNDTWTQAALEAAQDANYILYFGGLDTSAAGETKDRTTINWPEAQLQLIKTLTKLGKPLVVVQMGDQLDNTPLLATKTV
NSILWANWPGQDGGTAVMQILTGLKSPAGRLPVTQYPANYTAAVPMTDMNLRPSDRLPGRTYRWYPTAVQPFGFGLHYTTFQAKIAAPLPRLAIQDLLSRCGGDNANAY
PDTCALPPLKVEVTNSGNRSSDYVVLAFLAGDAGPRPYPIKTLVSYTRLRDVSPGHKTTAHLEWTLGDIARYDEQGNTVLYPGTYTVTVDEPAQASASFVVEGEAVVLD
RWPAPSGQVVV

>Rf_GH3
MLVEKYLDEALSDLERAEDITDRLSTEEKAEQQKYDAPAEERLGKDAYNWWSEGLHGVARAGTATMFPQTIGMAAMFDDEAVHRAGETTSREARAKYNEYSAHDDRDIY
KGLTLWSPNVNIFRDPRWGRGQETYGEDPYLTSCLGVAYAKGLQGDGKVLRTAACAKHFAVHSGPEATRHEFDAKANMKDMTETYIAAFEALVKDAKVESVMGAYNRVN
GEPACASDFVMNKLEEWGFDGHFVSDCWAIRDFHTNHGVTKTAPESAALALKKGCDLNCGNTYLHLLAAFNEGLINEEDLRRSCIKLMRTRVRLGMFDKSTEYDGLDYD
IVACDEHKEFSLRCSERSMVLLKNNGILPLDGSKYKTIGVIGPNADSVPALEGNYNGKADEYITFLSGIREAHDGRVLYTEGSHLYKDRCMGLALPDDRLSEAEIITRT
LRCSGSLCWLDATIEGEEGDTGNEFSSGDKNDLRLPESQRKLVKTVMAKGKPVIIVTAAGSAINVEADCDALIQAWYPGQLGGRALANILFGKVSPSGKLPVTFYEDAS
KLPDFSDYSMKNRTYRYSEGNILFPFGYGLTYSETECSELSFENGVATVKVTNTGSRFTEDVVQIYIKGYSENAVPNHSLCGFKRVALDAGESRIVQITLPERAFMAVN
EKGEWIKEGSEFTLYAGTSQPDELSKKLTGKECTTL

>HJ_bxyl

MVNNAALLAALSALLPTALAQNNQTYANYSAQGQPDLYPETLATLTLSFPDCEHGPLKNNLVCDSSAGYVERAQALISLFTLEELILNTQNSGPGVPRLGLPNYQVWNE
ALHGLDRANFATKGGQFEWATSFPMPILTTAALNRTLIHQIADIISTQARAFSNSGRYGLDVYAPNVNGFRSPLWGRGQETPGEDAFFLSSAYTYEYITGIQGGVDPEH
LKVAATVKHFAGYDLENWNNQSRLGFDAIITQQDLSEYYTPQFLAAARYAKSRSLMCAYNSVNGVPSCANSFFLQTLLRESWGFPEWGYVSSDCDAVYNVFNPHDYASN
QSSAAASSLRAGTDIDCGQTYPWHLNESFVAGEVSRGEIERSVTRLYANLVRLGYFDKKNQYRSLGWKDVVKTDAWNISYEAAVEGIVLLKNDGTLPLSKKVRSIALIG
PWANATTQMQGNYYGPAPYLISPLEAAKKAGYHVNFELGTEIAGNSTTGFAKAIAAAKKSDAIIYLGGIDNTIEQEGADRTDIAWPGNQLDLIKQLSEVGKPLVVLQMG
GGQVDSSSLKSNKKVNSLVWGGYPGQSGGVALFDILSGKRAPAGRLVTTQYPAEYVHQFPQNDMNLRPDGKSNPGQTYIWYTGKPVYEFGSGLFYTTFKETLASHPKSL
KFNTSSILSAPHPGYTYSEQIPVFTFEANIKNSGKTESPYTAMLFVRTSNAGPAPYPNKWLVGFDRLADIKPGHSSKLSIPIPVSALARVDSHGNRIVYPGKYELALNT
DESVKLEFELVGEEVTIENWPLEEQQIKDATPDA

>Ci_bglu
MSPTIWIATLLYWFAFQARKSVAAPPGVGALDDRAELPDGFHSPQYYPAPRGLGAGMEEAYSKAHTVVSKMTLAGKVNLTTGTGFLMALVGQTGSALRFGIPRLCLQDG
PLGLRNTDHNTAFPAGISVGATFDKKLMYERGCAMGEEFRGKGANVHLGPSVGPLGRKPRGGRNWEGFGSDPSLQAIAAVETIKGVQSKGVIATIKHLVGNEQEMYRMT
NIVQRAYSANIDDRTMHELYLWPFAESVRAGVGAVMMAYNDVNGSASCQNSKLINGILKDELGFQGFVMTDWYAQIGGVSSALAGLDMSMPGDGSVPLSGTSFWASELS
RSILNGTVALDRLNDMVTRIVATWFKFGQDKDFPLPNFSSYTQNAKGLLYPGALFSPLGVVNQFVNVQADHHKLARVIARESITLLKNEDNLLPLDPNRAIKYSEQMPG
TNPRGINACPDKGCNKGVLTMGWGSGTSNLPYLVTPEDAIRNISKNTEFHITDKFPNNVQPGPDDVAIVFVNADSGENYIIVESNPGDRTVAQMKLWHNGDELIESAAK
KFSNVVVVVVHTVGPIIMEKWIDLLRSRVSCLPDFQDKKLEILLLISCSETSVRVAASIYDTESRIGLSDSVSLINQRFGQIQDTFTEGLFIDYRHFQKENITPRYHFG
YGLSYTTFNFTEPRLESVTTLSEYPPARKPKAGDRHTPTISHLLQKWPGPKTLTGSGAYLYPYLDNPSAIKPKPGYPYPEAIQPNLNLNPRAGGSEAVTRRYGMLRSRF
PLKLLILERNPVRAVAQLYVELPTDDEHPTPKLQLRQFEKTATLEPGQSEVLKMEITRKDVSIWDTMVQDWKVPATGKGIKLWIGASVGDLKAVCETGKGKSCHVLN

>Sc_bglu
MLLILELLVLIIGLGVALPVQTHNLTDNQGFDEESSQWISPHYYPTPQGGRLQGVWQDAYTKAKALVSQMTIVEKVNLTTGTGWQLGPCVGNTGSVPRFGIPNLCLQDG
PLGVRLTDFSTGYPSGMATGATFNKDLFLQRGQALGHEFNSKGVHIALGPAVGPLGVKARGGRNFEAFGSDPYLQGIAAAATIKGLQENNVMACVKHFIGNEQDIYRQP
SNSKVDPEYDPATKESISANIPDRAMHELYLWPFADSIRAGVGSVMCSYNRVNNTYSCENSYMINHLLKEELGFQGFVVSDWAAQMSGAYSAISGLDMSMPGELLGGWN
TGKSYWGQNLTKAVYNETVPIERLDDMATRILAALYATNSFPTKDRLPNFSSFTTKEYGNEFFVDKTSPVVKVNHFVDPSNDFTEDTALKVAEESIVLLKNEKNTLPIS
PNKVRKLLLSGIAAGPDPKGYECSDQSCVDGALFEGWGSGSVGYPKYQVTPFEEISANARKNKMQFDYIRESFDLTQVSTVASDAHMSIVVVSAVSGEGYLIIDGNRGD
KNNVTLWHNSDNLIKAVAENCANTVVVITSTGQVDVESFADHPNVTAIVWAGPLGDRSGTAIANILFGNANPSGHLPFTVAKSNDDYIPIVTYNPPNGEPEDNTLAEHD
LLVDYRYFEEKNIEPRYAFGYGLSYNEYKVSNAKVSAAKKVDEELPQPKLYLAEYSYNKTEEINNPEDAFFPSNARRIQEFLYPYLDSNVTLKDGNYEYPDGYSTEQRT
TPIQPGGGLGGNDALWEVAYKVEVDVQNLGNSTDKFVPQLYLKHPEDGKFETPVQLRGFEKVELSPGEKKTVEFELLRRDLSVWDTTRQSWIVESGTYEALIGVAVNDI
KTSVLFTI

>Gg_ave
MLRSSAFALLAWASLSEAQFGIKHTQYGTSEPVYPSPEISGSGGWEAGLAKAKDFVAQLTPEEKANMVTGTPGPCVGNIAPVPRLNFTGLCLQDGPATLRQATYVTVFP
GGVSAASSWDKDLIYKHGVLMAEEFRDKGSHVILGPVIGPRGRSPYAGRNWEGFSPTSYLAGVMAEQTVKGMQSVGVQACTKHFIGNEQEEQRNPTAVDGKTVEAISSN
IDDRTMHEAYLWPFYNAVRAGTTSIMCSYQRINGSYGCQNSKTLNGLLKTELGFQGFVVSDWAATHSGVASIEAGLDMNMPGPLNFFAPTFESYFGKNITTAVNNGTLS
SRRVDEMIERIMTPYFALGQDKNYPPVDGSTVSVGFSQPGFWSHEFPLGPTVDVRRNHHEHVRELGAAGSVLLKNEKGALPLKKPMNIGVFGNDAADVTRGPYMAGGPF
GGVGGDCDIGTLPLGGGSGTGRYTYVFPPLEDIKARGRSYGALVQYITSNEVITSGGLVTIFPVPEVCLVFLKSWATEGEDRISLEAQWNAAVVVEKTAVLCNNTIVVI
HGGAPVVMPWRNNPNVTAILAAHMPGQENGHSLVDLVWGDVNPSGKLPYTLADQATDYNKNLVNSTELVQSTDPDAWQADFLEGQLIDYKDFDAHNKTPAYEFGFGLSY
TTFELSGVQVEVQASNPSRLPDPSAPIAPGGNVQLWETLATVKATVKNTGDREGATVAQLYLSLPGAEAGKDTPVRNLRGFEKVKLAPGACAEVEFALMRRDLSFWDTT
AQAWRLPEGAIGVDVGFSSRDLKLKSEIKI

>Wa_bglu
MLLPLYGLASFLVLSQAALVNTSAPQASNDDPFNHSPSFYPTPQGGRINDGKWQAAFYRARELVDQMSIAEKVNLTTGVGSASGPCSGNTGSVPRLNISSICVQDGPLS
VRAADLTDVFPCGMAASSSFNKQLIYDRAVAIGSEFKGKGADAILGPVYGPMGVKAAGGRGWEGHGPDPYLEGVIAYLQTIGIQSQGVVSTAKHLIGNEQEHFRFAKKD
KHAGKIDPGMFNTSSSLSSEIDDRAMHEIYLWPFAEAVRGGVSSIMCSYNKLNGSHACQNSYLLNYLLKEELGFQGFVMTDWGALYSGIDAANAGLDMDMPCEAQYFGG
NLTTAVLNGTLPQDRLDDMATRILSALIYSGVHNPDGPNYNAQTFLTEGHEYFKQQEGDIVVLNKHVDVRSDINRAVALRSAVEGVVLLKNEHETLPLGREKVKRISIL
GQAAGDDSKGTSCSLRGCGSGAIGTGYGSGAGTFSYFVTPADGIGARAQQEKISYEFIGDSWNQAAAMDSALYADAAIEVANSVAGEEIGDVDGNYGDLNNLTLWHNAV
PLIKNISSINNNTIVIVTSGQQIDLEPFIDNENVTAVIYSSYLGQDFGTVLAKVLFGDENPSGKLPFTIAKDVNDYIPVIEKVDVPDPVDKFTESIYVDYRYFDKYNKP
VRYEFGYGLSYSNFSLSDIEIQTLQPFSENAEPAANYSETYQYKQSNMDPSEYTVPEGFKELANYTYPYIHDASSIKANSSYDYPEGYSTEQLDGPKSLAAGGLGGNHT
CGMLVTLSLLKSQIKVLMLVGLHLNCMLDIQIMMNSQHLQCNYVDLKRCFWIKIILKLFLLN

>HJ_GH3
MRYRTAAALALATGPFARADSHSTSGASAEAVVPPAGTPWGTAYDKAKAALAKLNLQDKVGIVSGVGWNGGPCVGNTSPASKISYPSLCLQDGPLGVRYSTGSTAFTPG
VQAASTWDVNLIRERGQFIGEEVKASGIHVILGPVAGPLGKTPQGGRNWEGFGVDPYLTGIAMGQTINGIQSVGVQATAKHYILNEQELNRETISSNPDDRTLHELYTW
PFADAVQANVASVMCSYNKVNTTWACEDQYTLQTVLKDQLGFPGYVMTDWNAQHTTVQSANSGLDMSMPGTDFNGNNRLWGPALTNAVNSNQVPTSRVDDMVTRILAAW
YLTGQDQAGYPSFNISRNVQGNHKTNVRAIARDGIVLLKNDANILPLKKPASIAVVGSAAIIGNHARNSPSCNDKGCDDGALGMGWGSGAVNYPYFVAPYDAINTRASS
QGTQVTLSNTDNTSSGASAARGKDVAIVFITADSGEGYITVEGNAGDRNNLDPWHNGNALVQAVAGANSNVIVVVHSVGAIILEQILALPQVKAVVWAGLPSQESGNAL
VDVLWGDVSPSGKLVYTIAKSPNDYNTRIVSGGSDSFSEGLFIDYKHFDDANITPRYEFGYGLSYTKFNYSRLSVLSTAKSGPATGAVVPGGPSDLFQNVATVTVDIAN
SGQVTGAEVAQLYITYPSSAPRTPPKQLRGFAKLNLTPGQSGTATFNIRRRDLSYWDTASQKWVVPSGSFGISVGASSRDIRLTSTLSVA

>Cg_bglu
MHRKNHAAAAARTALVAAIAAAFPLFTPGAFAAPEPAEPAQKPWLDASLDADQRARLAVQAMTQQEKLRWVFGYFGHDFGKSKKHPDALPQSAGYIPGTPRLGLPALFE
TDAGQGVASQSGANVRERTALPSGLSTASTWDPKVAYAGGAMIGSEARASGFNVMLAGGVNLQREPRNGRNFEYAGEDPLLAGTMIGQAIKGVESNRIISTLKHFVLND
QETGRNELDARIDKAALRMSDLLAMELALEQSDAGSVMCAYNRLNGPYTCEHPWLLSEVLKRDWGFRGYVMSDWGATHSTVAAANSGLDQQSGQEFDKSPYFGGALEEA
VKTGAVPQKRLDDMVTRIVRTMFGKGVVDNPLKPGVAIDFAANGGQPPDGEEGMVLLKNEGRLLPLAKTVRTIAVIGGHADAGVLSGGGSSQVYPVGGIAVKGLLPATW
PGPVVYYPSSPLRAIQAQAPNAKVVFDDGRDPARAARVAAGADVALVFANQWIGEANDAQTLALPDGQEELITSVAGANGRTVVVLQTGGPVTMPWLARVPAVLEAWYP
GTSGGEAIANVLFGAVNPSGHLPATFPQSEQQLPRPKLDGDPKNPELQFAVDYHEGAAVGYKWFDLKGHKPLFPFGHGLSYTTFAYSGLSGQLKDGRLHVRFKVTNTGN
VAGKDVPQVYAAPMSTKWEAPKRLAAWSKVALLPGETKEVEVAVEPRVLAMFDEKSRTWRRPKGKIRLTLAEDASAANATSVTVELPASTLDARGRAR

>Se_GH3
MTGGERVKRLVIRIAPLLLVVPLLVAAVSPVRHSQRVDELIGQLTLDEKLSFVYWDYNEKDPLAKLWLPGVPRLGIPQIRGTDGPAGVTIHQPAIAMPAPVALASAFDD
RLAHEYGTVLGREGRAFEQDIILGPMVNNIRVPQAGRNFETFSEDPLVTARTAAAQIRGIHSQGLMTSAKHYAANTQETDRFTIDVDVDQRTLRELELPGFEAAVAAGA
TSVMCAYPKVNGTHACGHRQLLTEILKEQWGFKGWVMSDWTATHATEDLVAGLDQEMGVEVREDGSLFRGKYLGEALKKAIREGRIPESALDASVRRILTQFERFGLLD
ETKPPRPERDVAGGTRIAQEVAESGAVLLRNEGGVLPLDPAAGQDIAVIGPSAQQPKVTGLGSSYVEPDFANAPLDTITQRVGSGGRVGYSVGEELKGAPIPETALQPA
FVPGEVTPPPSGGVIYDGRLKVDADGLYRIAARIDGGNGSLQIDGGAPIGVGDVFGPLTSVPVWLTKGEHTIQMTGAAPVGGGSLDVDLTWVTPGHAQREFDAAVERAR
DSDVAVVFAYDDGAETADRTSLSLPGTQDKLIDAVASVNPNTVVVLNTGSSVTMPWLDKTRAVLDMWYPGQAGAEATTALLFGDAEPGGRLTQTFPVSQERTPVGGDPA
RFPGVDGKVHYSEGIFSGYRWYDREGVDPLFPFGHGLSYTTFERTDPVVERTRDGLDVTVTVRNTGQRRGSDVVQVYLGPSPQVPLDQAPRQLAGYQKVELAPGETKRV
RVHVAERALQHWDEAAGGWKLGGGKRAVEIGSSSRDIDLRADINL

>Ar_bglu
MIDDILDKMTLEEQVSLLSGADFWTTVAIERLGVPKIKVTDGPNGARGGGSLVGGVKSACFPVAIALGATWDPELIERAGVALGGQAKSKGASVLLAPTVNIHRSGLNG
RNFECYSEDPALTAACAVAYINGVQSQGVAATIKHFVANESEIERQTMSSDVDERTLREIYLPPFEEAVKKAGVKAVMSSYNKLNGTYTSENPWLLTKVLREEWGFDGV
VMSDWFGSHSTAETINAGLDLEMPGPWRDRGEKLVAAVREGKVKAETVRASARRILLLLERVGAFEKAPDLAEHALDLPEDRALIRQLGAEGAVLLKNDGVLPLAKSSF
DQIAVIGPNAASARVMGGGSARIAAHYTVSPLEGIRAALSNANSLRHAVGCNNNRLIDVFSGEMTVEYFKGRGFESRPVHVETVEKGEFFWFDLPSGDLDLADFSARMT
ATFVPQETGEHIFGMTNAGLARLFVDGELVVDGYDGWTKGENFFGTANSEQRRAVTLGAARRYRVVVEYEAPKASLDGINICALRFGVEKPLGDAGIAEAVETARKSDI

VLLLVGREGEWDTEGLDLPDMRLPGRQEELIEAVAETNPNVVVVLQTGGPIEMPWLGKVRAVLQMWYPGQELGNALADVLFGDVEPAGRLPQTFPKALTDNSAITDDPS
IYPGQDGHVRYAEGIFVGYRHHDTREIEPLFPFGFGLGYTRFTWGAPQLSGTEMGADGLTVTVDVTNIGDRAGSDVVQLYVHSPNARVERPFKELRAFAKLKLAPGATG
TAVLKIAPRDLAYFDVEAGRFRADAGKYELIVAASAIDIRASVSIHLPVDHVMEP

>Pb_GH3
MNKKIGVFALGICLSGASMAQAPQLRADNIDEIIAAMTLEEKAQLLTGCGNAGFPGSGAAMGHQTKLVAGAAGVTAGIPRLGIPQTVVADGPAGVHIDAYRKGTNQTFF
ATGFPIGTCLASTWNLDLVEKVGKAIGNETLEYGCDAILGPGMNLHRNPLCGRNFEYYSEDPIVTGLIGAAMVKGIQSQGVGVSAKHFAVNSQESDRTRVDERVSQRAL
RELYLKGFEIMVRKSAPWTLMSSYNRINGVYSQGNYDLLTKVLRQDWGFKGIVMTDWIGERADLPISDVVKAGNDLLMPGFPTQVNHIIEGVKSGKIDIKDVDRNVRNM
LEYIVKTPRFKGYKFSNTPNLKAHAAITRQSSTEGMVLLKNGKLNGQHILPMKQMSKVALFGVNSYDFMSGGLGSGCVNVPYVVDMVQGLKNAGISTTAQLTDIYEKYV
AYAKSKLKADKNPEMWFLDQGQPKLDEIEISRRCVEHEVSEAQAAIITIGRQAGEGLDRKIEGEFNLSAQEKDMISNVSAVFHNQGKPVIVVINSGSVMETASWRDLVD
AILVAWQPGEEGGNSVVDVLTGKANPSGRLTMTWPIAAVDHPSTANFPQQPDYYNLTEKLYANNLAGVNYTNHDEDIYVGYRYFDAFDKPVAYPFGYGLSYTTFSMSNF
AVKQIKDKVQVKVTVKNTGKTSGKQVAQIYVQAPKGNYEKPVKELKSFAKTQELAPGQSQILTMTIDRRDLTSFDEANSQWKGDAGEYVFMVGDNAAHMVGTAKLRLVE
YTEKVSEAMKPQHTLNLLRRNK

>Km_bglu
MSKFDVEQLLSELNQDEKISLLSAVDFWHTKKIERLGIPAVRVSDGPNGIRGTKFFDGVPSGCFPNGTGLASTFDRDLLETAGKLMAKESIAKNAAVILGPTTNMQRGP
LGGRGFESFSEDPYLAGMATSSVVKGMQGEGIAATVKHFVCNDLEDQRFSSNSIVSERALREIYLEPFRLAVKHANPVCIMTAYNKVNGDHCSQSKKLLIDILRDEWKW
DGMLMSDWFGTYTTAAAIKNGLDIEFPGPTRWRTRALVSHSLNSREQITTEDVDDRVRQVLKMIKFVVDNLEKTGIVENGPESTSNNTKETSDLLREIAADSIVLLKNK
NNYLTSKERRQYHVIGPNAKAKTSSGGGSASMNSYYVVSPYEGIVNKLGKEVDYTVGAYSHKSIGGLAESSLIDAAKPADAENAGLIAKFYSNPVEERSEDEEPFHVTK
VNRSNVHLFDFKHEKVDPKNPYFFVTLTGQYVPQEDGDYIFSLQVYGSGLFYLNDELIIDQKHNQERGSFCFGAGTKERTKKLTLKKGQVYNVRVEYGSGPTSGLVGEF
GAGGFQAGVIKAIDDDEEIRNAAELAAKHDKAVLIIGLNGEWETEGYDRENMDLPKRTNELVRAVLKANPNTVIVNQSGTPVEFPWLEEANALVQAWYGGNELGNAIAD
VLYGDVVPNGKLSLSWPFKLQDNPAFLNFKTEFGRVVYGEDIFVGYRYYEKLQRKVAFPFGYGLSYTTFELDISDFKVTDDKIDISVDVKNTGDKFAGSEVVQVYFSAL
NSKVSRPVKELKGFEKVHLEPGEKKTVNIELELKDAISYFNEELGKWHVEAGEYLVSVGTSSDDILSVKEFKVEKDLYWKGL

>Pp_hexo
MSFITSAHATAAQVPLTTSQMLGQKLMLDFRYYCGESKKPSGDCRAAMTTLPPELSELISRYDIGGAILFAENVQNTAQIISLTNALQSAAQQSKSQLPLFIAIDQEGG
RVARINREQATSFTGNMSIGATYPKQGDIYATKVASAIGKELNSLGINVNFAPTVDVNSNPNNPVINVRSFSENPTVVTKLGLAQVKAFEAAGVLSALKHFPGHGDTHV
DSHTGLPRVDHDRDKINQQDLLPFAEIIKASPPGMIMTAHIQYPALDNSKVVNSQGESMIRPATMSYQIMTQLLRHELGYQGVTVTDALDMAGISDFFNPVDATIETFN
AGVDIALMPIAIRNRADIKRFEQYMAQLADALETNKLNQEQLSSSMARIAKLKTKLPQSSASLAIANSTLGNPSHRRLEAELALAAITEVKNDGVLPLRDNAQVVHLIM
PDRQKCFALEQALQTYSKNSLTLSCTSLQAYDPDIAHDAIKQADMIIAAHASPPQSAVEIGGMDDVKKLREHGVARNVQPAALKALLQYGQQQGKKQLFISLRAPYEIS
TFGPLSNAVLASYAYNVDVNHDKKVAGPAYTALAKVILGIAKAEGSLPVTVNH

>Dc_bglu
MEKSATRQKALLIALPLLFSPLASAVQQAVLDTRGAPLITVNGLTFKDLNRDGKLNPYEDWRLPAAERAADLVSRMTLAEKAGVMMHGSAPTAGSVTGAGTQYDLNAAK
TMIADRYVNSFITRLSGDNPAQMAEENNKLQQLAEATRLGIPLTISTDPRSSFQSLVGVSVSVGKFSKWPETLGLAAIGDEELVRRFADIVRQEYRAVGITEALSPQAD
LATEPRWPRIDGTFGEDPDLTKKMVRGYVTGMQNGKNGLNAQSVISIVKHWVGYGAAKDGWDSHNVYGKYAQFRQNNLQWHIDPFTGAFEAHAAGIMPTYSILRNASWH
GKPIEQVGAGFNRFLLTDLLRGQYGFDGVILSDWLITNDCKGDCLTGVKPGEKPVPRGMPWGVEKLTPAERFVKAVNAGVDQFGGVTDSALLVQAVQDGKLTEARLDTS
VNRILKQKFQTGLFERPYVNATQANDIVGRADWQQLADDTQARSLVLLQNNNLLPLRKGSRVWLHGIAANAAQEVGFIVVNTPEQADVALIRTHTPYEQPHKNFFFGSR
HHEGSLAFRNDNPDYQAIVRASAKVPTLVTVYMERPAILTNVVDKTRAVVANFGVSDSVLLNRLMSGAAYTAKLPFELPSSMSAVRNQQPDLPYDSAKPLFPFGYGLPH

>Ec_bglu
MKWLCSVGIAVSLALQPALADDLFGNHPLTPEARDAFVTELLKKMTVDEKIGQLRLISVGPDNPKEAIREMIKDGQVGAIFNTVTRQDIRAMQDQVMELSRLKIPLFFA
YDVLHGQRTVFPISLGLASSFNLDAVKTVGRVSAYEAADDGLNMTWAPMVDVSRDPRWGRASEGFGEDTYLTSTMGKTMVEAMQGKSPADRYSVMTSVKHFAAYGAVEG
GKEYNTVDMSPQRLFNDYMPPYKAGLDAGSGAVMVALNSLNGTPATSDSWLLKDVLRDQWGFKGITVSDHGAIKELIKHGTAADPEDAVRVALKSGINMSMSDEYYSKY
LPGLIKSGKVTMAELDDAARHVLNVKYDMGLFNDPYSHLGPKESDPVDTNAESRLHRKEAREVARESLVLLKNRLETLPLKKSATIAVVGPLADSKRDVMGSWSAAGVA
DQSVTVLTGIKNAVGENGKVLYAKGANVTSDKGIIDFLNQYEEAVKVDPRSPQEMIDEAVQTAKQSDVVVAVVGEAQGMAHEASSRTDITIPQSQRDLIAALKATGKPL
VLVLMNGRPLALVKEDQQADAILETWFAGTEGGNAIADVLFGDYNPSGKLPMSFPRSVGQIPVYYSHLNTGRPYNADKPNKYTSRYFDEANGALYPFGYGLSYTTFTVS
DVKLSAPTMKRDGKVTASVQVTNTGKREGATVVQMYLQDVTASMSRPVKQLKGFEKITLKPGETQTVSFPIDIEALKFWNQQMKYDAEPGKFNVFIGTDSARVKKGEFE
LL

>Ni_bglu
MRRLPHLSLLALMLYSGTALAAPQQPALPEGQPLLTVEGLSFRDLNRDGTLNPYEDWRLSPEVRAADLVARMTLAEKAGAGVHGTAPIQGGPMASGPAYDMTAAQAIIR
DQHLNSLITRMAIAPADFAAENNRLQGIAAGTRLGIPLTISTDPRNHFQVLGGASVAASGFSQWPETLGFGALNDPALTRRFADLVRAEYRAVGIQMALSPQADLATEP
RWSRINGTFGEDPARVSAQVKAYVQGMQGADTGLAPGGVATVVKHWVGYGAQIDGYDGHNYYGRFTDFTKGGFDRHVAAFQGAFEAGATGIMPTYTIQKGLSLEGKPVE
PVSGGYNKQMLIDLLRGTHKFKGLILSDWAITNDCNESCRTGNPPQQPKDIATPWGVEDLTQPQRFAKGMLAGIDQFGGVNDGLPLLAAVEQKLLPEARLNEAVATIMT
LKFEQGLFENPFVDPAAAATIVGRADVVAEGRATQAKSLVMLENRLGPAPLPAGGGKRLFIYGVDAANAKAAGFTIAASLDEADIALIRLKAPFQTLHPGFFFGRMQHE
GDLDFKEGDAGLTLVRQAAAKVPVILTIYLDRPAILTNIKPHAATLIGEFGITDAALFDALTGKVAPMGKLPFELPATMAAVRAQSPALPHDSADPLYPVGFGR

>St_ac-glc-ami
MSRHQQREGTGRPGQPPHPAADVPATGAARPTRRTVLAATAGLTAALAVQGTGTAQAASRHDDRSLRALISRMTLQEKVGQLFVMRVYGHSATDPDQADIDANLEEIGV
RTAAELIEKYHVGGIIYFAWAHNTRDPQQIADLSNGIQKAALAQPRGLPLLIATDQEHGIVCRIGKPATLFPGAMAIGAGGSTADARTLGRISGAELRAMGVNQDYSPD
ADVNVNPANPVIGVRSFGADPDAVARMVAAQVKGYQGSGVAATAKHFPGHGDTAVDSHTGFPVITHTREQWETLDAVPFRAAIKAGIDSIMTAHLQFPALDPSGDPATL
SRPILTGILREELGYDGVVITDSLGMEGVRTKYGDDRVPVLALKAGVDQLLNPPSLDVAFHAVLDAVRSGELTEARLDESILRILRLKARLGLFDDPYTTRADVTRTVG
TEAHLRTADALAERTTTLLVNKGNLLPLSRRSHQRILVVGADPDSPSGTTGPTTGVLAAALNELGFTATALPTGTAPSAAVTDQAVAAARDADAVVVATYNVTAGSAQQ
TLVERLAATGRPVIAVAVRNPYDVAQLPAATAVLAAYSWTDVEVRAAARVVAGRVSPRGTVPVPVQRADDPEQTLYPIGYGLSYRRH

>Bs_bglu
MENAARQASVRYAQNGQGPLLGYDESSGVRILRVDGHAFKDLNKDGKLDPYEDWRLPPEERARDLASKMTIEQIAGLMLYSSHQAIPGNMGWFPATYAGGKAFPDSGAA
PSDLSDQQLDFLSNDHIRHILVTRVQSPEVAANWNNNVQAYAERLGLGIPANNSSDPRHGSDTSKEFNAGAGGAISMWPESMGLAATFDPAVAREFGEIASREYRALGL
STALSPQVDLATDPRWFRFGMTFGEDPRLATDMARAYIDGFQTSEGDAEIADGWGSDSVNAMVKHWPGGGSGEAGRDAHFGYGKYAVYPGNNFEEHLRPFTEGAFRLAG
KTGEASAVMPYYTISVGQDPVNGENVGNAYNAYLIRDLLRGKYGYDGVVCTDWGITADEGPDIERLFPGGRCWGVEENHTVAQRHYKLLMAGVDQFGGNDDAGPVIEAY
RIGVEAHGEPFMRARFEQSAVRLLKNMFRLGLFENPYLNPGKSAALVGNPAFMEAGYRAQLRSVVMLKNEGILPLPKRQTVYIPKRKLPADADWMGNPVPPSETYPINL
DVVRKYFDVTDRPADADFALVCIESPRSTKGYSKADAEAGGNGYVPISLQYRPYTADHARETSLAGDPRDVLNRSYKGKTAAVANEGDLDAVLETKRLMNGKPVVVSIA
LSNPAVAAEFEPAADAILAHFGVQDQAILDILTGAFEPQALLPFRMPADMTTVEKQLEDVPHDMDVYVDSAGHAYDFAFGLNWSGVIADARTSRYANKRRTL

>Ps_ac-glc-ami
MTNPVFNGKALTLKQKVGQLVMAGFNGLEASDDARKLITEDHVGGIIYFRRNLAEPAQVAKLSAELQQIAAESDNVPLLISIDQEGGMVTRLENGVTVVPGNMALGAAG
DAELAYEAAHIIGSELRALGINMNFAPSLDINNNPGNPVIGVRSYGGTAELVARLGTEAVRGFQDAGVAATVKHFPGHGDTGEDSHHALPTVPHARERLDRLELAPFRE
AIARGVDAVMTAHVLFPAVEPEKLPATLSSNVIEGLLRGELGYDGVVVTDCLEMNAISKFYGVGEGAVQAVEAGADLILVSHRYERQKAALDALLAAVESGRISEERID
RSVGRLLALKQRRAVDAGAAVTLSSGDTLVTDEKTELVERISEKSITLLRSEGEFTLDKTKPVLVVWPEVRVGSEVDEVLPRKETLGYWLKSAGYDVNEQTIGVQPTEE
EVAHIQELSGQISQVVVVSYNAIFSPDQAALIEALAAKPDVQLIVASARNPFDINALPTVKTFFAAYENTPSAMRALALVLTGQIAVQGTLPAPLTVTV

>Cf_GH3

YLRYKDPKQPMNGRLRDLMGRMTLSEKIGQMVQLDRTVASAEIMRDYSIGSLLSGGGSVPLHKATAQDWTNMVNHFQNGSLSSRLGIPMIYGIDAVHGHNNVYKATIFP
HNIGLGATRDPELVKRIGAATALEVRATGINYVFAPCIVCRDPRWGRCYESFSEDPKLVEAMTDIITGLQGDIPANSRKGVPYVGGKDKVAACAKHFVGDRGTVNGINE
NNTVIDRHGLLSIHMPGYYHSVIKGVSTIMVSYSSFNGKKMHANYDLVTTFLKDTLKFRGFVISDWEGIDRITSPPHSNYSYSVLAGIQAGIDMVMLPYNHTEFIYILT
GFVNNNVIPMSRIDDADGRILRVKFTMGLFENPYADQTFEDQLGNQAHRDLAREAVRKSLVLLKNGENADGALLPLPKKASRILVAGTHAINMGYQCGGWTITQGLDGN
NHTEGTTILNAISAAVDPSTEIVFSENPESDFVNSNNFSYAIVVVGEPPYAETQGDSLNLTMLEPGPSVINNVCGRVKCVVVVVSGRPIVIEPFVSQMDALVAAWLPGT
EGQGVSDVLFGDYGFTGKLPPRTWFKSVDQLPMNVGDAYYDPLFPFDFGLITE

>Aa_bglu
VYKNPNAPVEARVKDLLSRMTLPEKIGQMTQIERTVASPTVITDSFIGSVLNAADSWPFEDAKSSDWADMIDGFQRSALASRLGIPIIYGIDAIHGNNDVYGSTIFPHN
IGLGATRDEDLVRRIGAATALEVRASGAHLTFAPCVAAVRDPRWGRCYESYGEVAKIVCEMTSVVSGLQGEPPEQHPNGYPFVAGRKNVVACAKHFAGDGGTNKGINEG
NTILSYKDLNRIHIASFKKCIAQGISTVMVSYSSWNGDKLHSHYFLLTEFLKQKLGFKGYINSDWEGLDRLSDPPGSNYRNCVKIGINAGIDMVMVPFRYKEFIGDLIN
LVESGEVPMARIDDAVERILRVKFVAGLFEYPLADRSLLPTVGCKEHRELAREAVRKSLVLLKNGNYGQFLPLNCNAEKILVVGTHADDLGYQCGGWTKTMYGQSGKIT
IGTTLLDAIKAAVVESTEVIYEKYPSKETLASGYRFSYAIVAVGEAPYADTKGDNSELIIPFNGSDIITMVAEKIPTLAILFSGRPMVLEPQVLEKTEALVAAWLPGTE
GQE

>Es_bglu
IYKNPKEAVEDRIQDLLQRMTVEEKIGQMTQIHRGVSSAAVIKDFFIGSVCNSAGKSGNDVLSVDWAEMIDGFQTAALETRLAIPIIYGLDAVHGNNKFYGATIFPHNI
GLGATRDEDLARRIGSATALEVRASGAHWAFAPCVAVCKDPRWGRCFESYSEDTDVVCNMTSFVSGLQGTPPQGHPAGYPFLAGRHNVVACAKHFVGDGGTEKGKNEGD
TVASYEDLERIHMSPYLKCLAQGVSTVMPSYSRWNGSKLHADRFLLTEILKEKLGFKGCLVSDWSGIDKMGEPRGSNYRECVEAAINAGIDMVMVPYKYEKFINDLTSL
VQSGEIPMSRIDDAVERILRMKFTAGLFEHPFSDRSLLKFVGCKEHREIAREAVRKSLVLLKNGKDSEKPFLPLDRNAKRVLVAGTHADDLGFQCGGMVQDLVRSEWSD
HNRPGTTVLDAVKAIVGAETVVVYEKSPSEETLSGKDFSYAIVVVGEAPYAESRGDDPEPSIHFDGAEVMRLVAGKIPTVVILMTGRPVILDPTVLDKVEALVAAWLPG
TEGDGIADVVFGDYDFSGKLPISWFRTTEQLPMNKEADGYDPLFPFGFGLK

>Fv_bglu
IYRNPNESIEARVKDLLSRMTLKEKVGQMTQIEREVATPFAIKDLSIGSIISGAGSEPFKKALSADWADMVDGFQRCALETRLRIPLIYGIDAVHGNNSVYGATIFPHN
VGLGATRDADLARRIGEATALEVRASGIHYTFAPCVAVCRDPRWGRCYESYSEDTEIVRKMTSIISGLQGKAPQGHENGFPFIKGRNNTIACAKHFVGDGGTQKGVNEG
NTISSYYDLERIHMAPYLDCISQGVSTIMASYSSWNGNRLHADRFLLTEILKGKLGFKGIVISDWDGHETISEPQGSNYRFSVLSAINAGIDMVMVPFRFKNFVDDLVN
LVEHGEIPMSRIDDAVERILRVKFVAGLFEHPFSDRSLLDKVGCKLHRDLAREAVRKSLVLLKNGKEPRKPFLPLDRKAKRILVTGTHADDLGYQCGGWTVTKAGLSGR
FTIGTTILEAIKKAVGHKTEIIHEQLPSTDTLARNDISFAIVAIGEGSYAECKGDNSELVIPFDGDDIITSVAERIPTLVILISGRPLTVQPSLLDKMDALVSAWLPGS
EGEGIADVIFGDYNFEGKLPVTWFKSVEQLPLDAGSNSYEPLYPLGFGLTCNTEK

>La_unpro
VYKNPNEPIEARVRDLLSRMTLKEKIGQMTQIERTVATPSSLIHLSIGSILSCGGSAPFENAMSSDWADMVDGFQKSALESRLGIPLIYGIDAVHGNNNVYGATIFPHN
IGLGATRDADLARRIGAATALEVKASGVHYTFAPCVAVCKDPRWGRCFESYSEDTEIVRKMTSFVSGLQGQPPEGHKHGYPFVAGRENVIACAKHFVGDGGTYKGMNEG
NTALSYEELEKIHMAPYLDCISQGVSTIMASYSSWNGHKLHANNFLLTEILKEKLGFKGFVISDYEGIDRLCDPRGSDYRYCISSAINAGIDMVMVGVRFEQFIEDLTF
LVESGEVPISRIDDAVERILRVKFVAGLFEIPSSDRSLLDIVGCKLHRDLAREAVQKSLVLLKNGKDCSKPFLPLDRNAKRILVTGTHVDNLGYQCGGWTKTGRGDSGR
ITIGTTILDAIKAAVGVETEVVYEQYPSKDTIENNGIVFAIVAVGEVPYAETSGDNSKLRIPVDGAEIISLVADKIPTLVILISGRPLVLEPWLLEKTEAVVAAWLPGT
EGDGITDVIFGSHDFKGQLPMTWFRRVEQLDHQPSDGVNSSEALFPLGFGLSYN

>Rs_unpro
GAHWAFAPCVAAMRDPRWGRSYESYSEDADTICDLTTLVSGLQGEPPKEHPNGYPFLAGRNNVVACAKHFVGDGGTENGTNEGNTIVSFEELERVHLPPYLNCLAQGVS
TVMASYSSWNESKLHSDYFLLTELLKQKLGFKGFVISDWEALDRLSEPFGSNYRHCVKISINAGIDMVMVPFKYEQFIHDLTDLVQSGEVSMARIDDAVERILRVKFVA
GLFEHPLTDRSLLGTVGCKEHRKLGREAVRKSLVLLKNGKHVDKPFLPLDRNAKRILVTGTHADDLGYQCGGWTKAHSGLSGRITIGTTLLDAIKAIVGDQTDVIYEKN
PSEESLASSEAFSYAIVAVGESPYAETMGDNSELTIPFNGSDIVTTVAERVPTLMILFSGRPMVLEPTVLGKTEAVVSAWLPGSEGQGMADVIFGDYDFEGKLPVSWFK
RVEQLPVNADSDLYDPLFPLGFGLISSG

>At_bglu
IYKDPQQAIEERVKDLLLRMTIAEKVGQMTQIERSVVTLDAMKNQFIGSILNAGGSTPRSGASAEEWADMVDGFQRWALESRLSIPVLYGTDAVHGHNNCYGATIFPHN
IGLGATRDPNLVQKIGEATAIEVRATGIPYTFAPCVAVSRDPRWGRCYESYSEDTETVRKMTTIVQGLQGSPPQSHPKGYPFLASRKNVIACMKHFIGDGGTKGGINEG
NTVGSFDELHSVHLKPFLDCLDQGVCTAMASYSSWNSMMLHSHHFLLTQVLKHQLGFKGFVISDWEGIDRLCQPQGSDYRFCISASINAGIDMVMVPHDFQKFIGDLTF
LVESGEISMTRIDDAVERILRVKFVGGLFEHPFTDRSLLNLVGCKMHRELAREAVRKSLVLLKNGKNPNKPFLPLSKNGGRILVGGEHAHNLGYQCGGWTITWYGIGGQ
ITQGTTILEAIKAAVGMNTAVIYEENPTETSFKTQEFSSAIVVVGEKPYAEFLGDDPKLELSPKAIETIELVCSKVPTLVILLSGRPLIVEPLIEKMEAFVAAWLPGSE
GAGVADVIFGGYEFHGCLPRTWFKRVDQLPMNVGDSNYDPLFPFGFGLKMNL

>At_unpro_I
VYKDPKASLNKRIHDLMSQMTLAEKIGQMTQIERQVANYSVMKEYAIGSILSGGGSVPSPQASAAVWVNMVNEFQRGALASRLQIPMIYGIDAVHGHNNVYGSTIFPHN
VGLGVTRDPDLLKRIGAATALEVRATGIPYTFAPCIAVCRDPRWGRCYESYSEDPEIVEAMTEIIPGLQGDAPKKGVPFVAGKTKVAACAKHFVGDGGTHNGINENNTI
IDRHGLLAIHMAGYYHAIIKGVSTVMVSYSSWNGEKMHANRDLVTNFLKKTLHFRGFVISDWQGIDRITSPAGSNYPYSVHAGVNAGIDMIMVPYNFTEFINDLTEQVN
SKSIPMSRIDDAVRRILRVKFQMGLFENPLADLSLADQLGSQEHRELAREAVRKSLVLLKNGKEGDKPLLPLDKKAPKILVAGTHADNLGYQCGGWTIQWQGQSGNTTK
GTTILTAIKSTVSPSTQVVFEENPSASSLKGQDYDYAVVVVGETPYAETNGDSMNLTMPEPGPTIIKNVCGSVKCVVVVISGRPIVLQPFLQYIDALVAAWLPGTEGQG
VADNLFGDYPFTGKLARTWFKSVDQLPMNVGDKHYDPLFPFGFGLTTEKTS

>At_unpro_II
TYKDPKAPLQSRIEDLLKQMTLAEKIGQMTQIDRSVATQDVMKNYSIGSVLSGGGSIPKPNATVEDWVNMVNDFQKGALSSRLQIPMIYGIDAVHGHNNVYGATIFPHN
IGLGATRDPELAKRIGVATALEVRATGIPYVFAPCLAVCRDPRWGRCYESFSENPEIVEAMTEVIVGLQGGEPSQKGTPFVTGKQSVAATAKHFVGDGGTVRGIDENNT
VIDYHDLLSIHLRPYYRAIIKGVSSVMVSYSSWNGVKMHSNRKLVTGLLKSTLRFRGFVISDWQGIDRITSPPGSNYMFSVHAGVNAGIDMIMVPYNYTDFINDLTDQV
NSKSIPMSRIDDAVRRILRVKFQMGLFENPMADLSLKDHFGSRKHRELAREAVRKSLVLLKNGKDGDKPLLPVDKKAPKILVAGTHADNLGYQCGGWTITWQGGSGNTT
IGTTILSAIRSTVDPHTEVVYQENPDPSSLKANDYSYGIVVVGELPYAEFDGDSTTLTMIEPGPTIIKNVCASMKCVVVVVSGRPIVLEPYVPYMDALVAAWLPGTEGQ
GLADVLFGFYPFSGKLPRTWFKSVNQLPMNVGDKHYDPLFPFGFSLTTDI

>Ps_bglu_I
VKKYYIGSVLSGGGSVPAPKASPATWINMVDDLQKGAMSTRLQIPMMYGIDAVHGHNNAYGATMFPHNIGLGATRDPDLARRIGAATALEVRATGIQYTFAPCVAVCRD
PRWGRCYESYSEDPKIVKAMTQIIFGLQGQPPANSTKGVPFIAGQSNVAACAKHFVGDGGTTNGIDENNTVIDYKGLVNIHMTPYFDAIAKGVSTIMVSYSSWNGMKMH
ANRFLVSEVLKKQLGFKGFVISDWQGIDRITSPPGANYSLSVFDGVGAGIDMVMVPENFTNFITELTSQVKGGLISMTRINDAVRRILTVKFTMGLFEYPMADPSLANH
FGSKEHRELAREAVRKSLVLLKNGKSAGKPLLPLDKNAPKILVAGTHPNNLGYQCGGWTIEWQGLSGNSTIGTTILQAIKFAVSPSTEVIYQQNPDANYVKGQGFSYAI
VVVGEAPYAEMNGDNLNLTIPLGGGDTIKNVCSSLKCLVILISG

>Ps_bglu_II
IYAKYKDPSQPIIARVEDLLARMTVEEKIGQMTQIERSDATADVMKKYYIGSVLSGGGSVPAPKASPATWINMVDDLQKGAMSTRLQIPMMYGIDAVHGHNNAYGATMF
PHNIGLGATRDPDLARRIGAATALEVRATGIQYTFAPCVAVCRDPRWGRCYESYSEDPKIVKAMTQIIFGLQGQPPANSTKGVPFIAGQSNVAACAKHFVGDGGTTNGI
DENNTVIDYKGLVNIHMTPYFDAIAKGVSTIMVSYSSWNGMKMHANRFLVSEVLKKQLGFKGFVISDWQGIDRITSPPGANYSLSVFDGVGAGIDMVMVPENFTNFITE
LTSQVKGGLISMTRINDAVRRILTVKFTMGLFEYPMADPSLANHFGSKEHRELAREAVRKSLVLLKNGKSAGKPLLPLDKNAPKILVAGTHPNNLGYQCGGWTIEWQGL
SGNSTIGTTILQAIKFAVSPSTEVIYQQNPDANYVKGQGFSYAIVVVGEAPYAEMNGDNLNLTIPLGGGDTIKNVCSSLKCLVILISGRPLVIEPYLPLVDAFVAAWLP
GTEGQGVTDVIFGDYGFQGKLPRTWFKSVDQLPMNVGDKHYDPSFPLGFGLTTTV

>Mp_bglu_I
LYKDASQPVELRVQDLLSRMTVQEKIGQMTQIERTVATPEVMTQYFIGSVLSGGGSAPEPNAPAAWQDMIDTMQQAALATRLAIPMIYGIDAVHGHNNLYGATVFPHNI
GLGCSRDPDLVKRIGAATALEVRATGIPYAFAPCIATCRDPRWGRCYESYSEDTAVVKTMTDIILGLQGDPPNLTAGVPFMADKSKVIGCAKHYVGDGGTFKGINENDT
IVDYDTLYKVHMAPYLDAIAKGVSTIMVSYSSWNGERMHANQYLVTQVLKEQLAFRGFIISDWMGVDRLSDPNNPNYTNSVLKSINAGLDMIMVPFDYEAYISGMLSLV
NDGEISMERIDDAVTRILRVKFVMGLFEDPSVDRSLTNHLGSQEHRILAREAVRKSLVLLKNGQQGSQALLPLKKNATSILVAGSHADDIGLQCGGWTISWVGAAGNTT
IGTTVLDAIKAAVSPTTVVTYEKNPAPGFAAQLKPDYAIVVVGEEPYVETYGDNMELTIPLDGIPTIQNVCAEVKCLVIVISGRPLVIEPYMPQIDALVAAWLPGSEGQ
GISDVIFGDYDFVGKLSRTWFRTVDQLPMNFGDAVYDPLFPFDFGLTMG

>Cs_GH3
MKNLSLKEKIGQLIVVRTTGYLFDHQIRYPAWEANQQQLQTWLSEYNIGGVILLGGSCAEIAQRTKQLNQWAKTPLLVAADIEEGVGQRFTGASWFPPPMALAQIAQDD
LELAKKYAEEMGKITAKEALCIGVNWIFAPVVDVNNNPDNPVINVRAFGDNPEVVKELSSAFIRGTQSYPILNGAKHFPGHGDTSTDSHLDLPVINHSQARLEKIELVP
FQGAIALNVDAIMTAHLLVSAYDNQNPATLSHRILTEELRHNMGFEGLIVTDALIMGGVAKYAPPEKIAVKALQAGADILLMPENPVVAIHSIIEAVEKGEISEHRIDE
SLQRVSKAKEKLFKSQESFSFVDISTNDSQQVINEILVKSNQTSVLKPIVGLDKGINLVVVNNLLNCDFLDRQSPSITIPDSFGYHAQVFDQCNLYIWENQLITEPFIL
QVFVRGNPFRGTAGLSAIALKAYEQILENHHLQGIMVYGSPYVLDWFKNQIPTPIPWGFSYGQMAIAQRTICNKMFQLSTNLDITKGNFL

>Cs_bglu
MKNLSLQEKIGQLIVVRTTGYLFDHQIRYPAWEANNQQLQTWLSEYNIGGVILLGGSCAEIAQRTKQLNEWAKTPLLVAADIEEGVGQRFTGASWFPPPMALAQIAKKD
LALAKKCAEEMGKITAQEALSIGVNWILAPVVDVNNNPDNPVINVRAFGDNPGVVRELSSAFIRGTQYYPILNGAKHFPGHGDTSTDSHLDLPIINHSRTRLEAIELIP
FQGAIALGIDAIMTAHLLVSAYDNQNPATLSHAILTKQLREKMGFDGLIVTDALIMGGVAKYAPPEKIAVKALQAGADILLMPENPLVAINSIMEAVQRGEITESRIDE
SLQRIAKAKAKLSDGDFSFLQISTSTSQEVVNDILDQSNQIQVFEGIKKVDQGINIVVVDDLLNCDFLDRQTPAITIPATFGYHAQVFDQRNLHIWENQPINQPFILQV
FIRGNPFRGSAGLSAIALTTYTKLLQHPEIQGIIVYGSPYIKDWFTKQLKPNLPWGFSYGQMAIAQYILSQKLFQISESLDISKGNFL

>Cb_bglu
MKNKFPILSLTLILLISACQPAAVDVNPAPIATTQTTEEMPAEANVAPLYTDSSLSAEERASDLLARMSLDEKIGQMTQVEKNSILPGEVSRYYIGSILSGGGGAPTGD
DSLEGWVKMVDGLQAAALETPLAIPLIYGVDAVHGHNNVKGATIFPHNIGLGATNDPELVEKIGRATAEEMLATGISWDFAPVLAVVQDIRWGRTYESYGENTELVTRL
GVAYQNGLQAAGDGDSIFVLATPKHYIGDGGTTWASSTTDNYKLDQGDTQMDEARLRELFLPPYQAAVEAGAQSVMVSYSSWNGVKMHGHKYLITDVLKGELGFEGFVV
SDWAGIDQVDSDYYTAVVTAINAGVDMNMVPQGYPRYLTVMQQAVEKGDIPMERIDDAVLRILTVKFKLGLFEKPFADPAYLETVGSQAHRDLAREAVAKSLVLLKNDN
ATLPLAKDAGLIFVAGASANDIGAQCGGWTIEWQGKSGNITTGTTILEAIEASASAEVRFDRFGKFESEQKADVAVVVIGERPYAEGRGDKENPSLSKSDIELIQRVRE
QSQRVVVILLSGRPLVITEALPYADAFVAAWLPGTEGSGVADVLFGDKPFTGKTPFSWPRSADQLPFDFANLPADGCAAPLFPYGYGLDVTSSEPVSLPVCP

>Xc_bglu
MLLRRTAAPVARRALFLATAAAVLMLAACQGKDATSTAAADTTAPAATPAETSTTIHPDQWPSPKWPFAQDQALEQRISDVMAKMSVEEKVAQTIQGDIASMTPDDVRK
YRIGSVLAGGNSDPGGKYDAKPAEWLKLADAFYEASMDTSKGGNAIPIIFGIDAVHGQSNIVGATLFPHNIGLGATRNPDLIKKIGEVTAAETRVTGMEWTFAPTVAVP
QDDRWGRSYEGYSESPDVVASFAGKMVEGVQGVPGTPQFLDGSHVISSVKHFVGDGGTTDGKDQGDTKVSEATMRDIHAAGYPPAIAAGAQTVMASFNSFNGEKMHGNK
VMLTDVLKGRMNFGGFVVGDWNGHGQVKGCTNENCPASFIAGVDMAMASDSWKGIYETELAAVKSGQISMERLDDAVRRILRVKLRLGLFEAGKPSKRPLGGKFELLGA
PEHRAIARQAVRESLVLLKNQAGVLPLDPKKRVLVVGDGANDMGKQSGGWTLNWQGTGTKRSDYPNGNTIWEGLNKQITAAGGSAELAVDGAYKTKPDVAVVVFGENPY
AEFQGDIATLLYKPGDDSELALLKKFKAEGIPVVAVFLSGRPLWMNQYINVADAFVAAWLPGSEGEGIADVLLRKSDGSVQNDFKGKLSFSWPKTAVQFANNVGQKDYD
PQFKFGFGLTYADKGDLAALPEESGVSGEQSVGGVYFVRGKPALGIAMQLSNAGQANMPATTLPVGLSDGSLKMSAVDHKAQEDARRLQWSGAKASSVLLVSGKPVDVS
RESNGDVQLQLTLRRDSAVTAQVWLGVGCGEKCGGRVDAQKTLAALPQGQWKVVGIPLKCFAVAGADVTKLTQVASIESAAALDIAVSKIALGALNEAEVTVDCPVK

>Cr_GH3
MTSLLSALGRRASAGTPLGRPAPGGSTLPAVAPAAAASPALPRRTCCPTAALSTTASSACGAAPGISAEPLPQLASCPSRISSSRSGGRRSRVLLAAPPRSTGNGQVGP
PQHRKGVVAPGAGAGAAAASAAAPAAAVAAAVDEEVADDDEDVLMDEDEAGQQLAPFMDAVVKVYCMHTEPNYSLPWQRKRQYSSSSSGFVVSHGGRNWLLTNAHSVDY
HTQVKVKRRGDDRKFLARVISVGVDCDIAALQVDDPDFWAPQSPDSPPPPVLELGPLPRLQDGVAVVGYPIGGDTISVTAGVVSRIEVTDYSHGSTDLLAIQIDAAING
GNSGGPVFNRACQCVGIAFQALVGSDVENVGYVIPTPVVAHFLDDYTRTGGFSGFPQLGIQWQRMESEALRRAYGMTAAAAGAPHALLPHFTPYRRPVPPHSPPATPDL
RESYLPAFRACVVEGRAHSVMCAYNKVNGVPACAHPHLLRATLNDSWGFGSDPANFVVSDCGAVSDLALTHGWNTSLAAAAAEALTAGGLSLFCDNAAAAAVPQAVASG
LLAPAVLRAAVRRMLLARCRLGILGAAQPNTTNTTDSSSSTQLDTSEAPAGASSTSADGTLSQAEAAVDRSGNSGSGGGSGSGSGGGSGSSSGSGSGSGGGSGSSSGSG
SGSGSGRPDVQALSRSHAHTRLAYEAALRSITLLVNRPPPGGSGGRPLLPLQLPPPPPAPANTTDNTTAAPGPLLALLGPHADGALYYLGTYYGTPSHPVVTPLAALRE
ALGPAAVSHTPCLTGVGLEPSDGLHTCTQAAAAAQVAAVVFVGGSSRNFACGDQQCTTITPVSESEGLDRGSLRLPGLQEELVRAVARSGVPVVVVAVAGGPLDLSPLL
GLQGVAAVLAAPYGGQQAGYALASVLLGTSSPSGRLPATWLYDWYTHLSDPTSMAMRAWPGRTYRYLQEELVRAVARSGVPVVVVAVAGGPLDLSPLLGLQGVAAVLAA
PYGGQQGGYPLASVLLGTSSPSGRLPATWLYTHLSDPTSMAMRAWPGRTYRYLQVPVLFPFGFGLSYTHFHTTDMRASPWDCRTRGDTGAGTAAEGRRRRQAAPRGAGG
GGGEEELCFRVEVDVVNAGRRASGHAVLLSLRRSPQPPGAAPAAGGTRTGAGGGSGSGGGGARSDSGDAGGGGASGGAGGRVLAAAGVGGVAAAAASGGEPGAGRAHSG
CRPCAEVLQQQQPWRAQRQQEQGQRDGPQDGEEESEEEEEGGEFAEWGGERYGHPDTLAAHTAAYHRIMADRLERPQGQQRPQAQQGQQGRGARGGSGARGSASAAGAG
SHSDGGGGDELDGQVDGVDDDPPPVRELVAFGRLEDVSPGEVRTLVLQVRLPAAGLEVEVEAEAEVEAEAEVEAEEEQQAGRQGGGPRRRAHRRYVAHLQAGALCCPLP
RPAASRR

>Mp_bxyl_I
MLSEPLRLCCQLNSKPSHWYFVWTFKQRPALTERRMKSHVLVTAVLLLIQHLLAQKTNGDSSRSSYACTSRESKSYSFCDKALSIDERVADLISRLSLEEKISQLGNGA
DAISSVDVPAYQWWGEALHGVAYCPSVHWDGPVKGATSFPMPISLAASFNKSLWNKIGQVISTEGRAMHNLGQSGLTFWSPVINLVRDPRWGRVQETPGEDPYLIGQYS
VYFVRGMQEAKDYDPSNEDDNENENFALKTSACCKHYTAYDLDNWQGVERYDFDAKVTIQDLADTYNPPFQSCVEEGRASCLMCSYNKVNGVPTCADPELLKGTVREKW
GLRGYIVTDCDSLLVMYDESRYSKGPAEAIATAMLAGLDLNCGATIKTYGASAVQQRLISEADIDRALNNTFTVLMRLGIFDGNPLDQPYGRLGDDHICSEEHQDLATE
AAVQGMVLLKNDEDTLPLSSRHIKTLAIIGPNADDTKYTMLGNYAGRPCTYITPLQGLSTYEINNVIFEPGCSSIDCSSDEKIEDALSAAKKADVVILVMGLDQDLERE
TFDRNSLKLPGKQELLVSSVANVSNGPVVLVLICGGPLDISWAKNDSRIQSILWMGYPGQAGGLALAQIVFGDRNPVGRLPVTWYPESITDWPMTCMNMRPDPHTGYPG
RTHRFYNGPTVYEFGYGMTYSKSTTMEVTGPTNFTAPALFQQACYHKRFTAEQNDSTGCSPSDLVACLEASLPINITVKNRGPRAVTEVALVYHVPPRAGENGTQLKEL
IAFERTEVQPFSSKKIHLIINLCHHTSTVKSDGKRSLQLGTHKVVVSPGSGTSAEHRFYVH

>Mp_bxyl_II
MTSAGSLLRLAIAIVVAASIISGQAAAAADGPDARRPRVPHPCQEPAAYPFCDVNLSNEDRVRDLVSRFTLEEKARLLVNMGSGSNITRLGLPAYEWWQEALHGVADSP
GARFKGRVKSATSFPQPILTAASFNKELFNKIGQVISTEARAMHNENQAGLTFWAPNINIFRDPRWGRGQETPGEDPYLTSIYAEYFVRGMQEDDEYEGQNLRTSGKGP
AQLKTSACCKHFTAYDIDQWYDVDRYDFDAKVTQQDLLDTYNPPFQSCIEDGKASSLMCSYNRVNGVPTCADYNLLTKLARGTWGFDGYIVSDCDAVQVMYANSRYAQT
PEEAVAYALKAGMDLNCGDTASNFTVEAVHSGLLNTSDIDKALHNSFTVLYRLGYFDGDPLNDPKYGKLDHSNICSPEHQDLALEAALQGIVLLKNDQNTLPLSADKIR
SLAVLGPNANDTVNTMLGNYAGPPCVYVTPYLGLAQYVPDASYHPGCDNGTTCEGRSDFIRGAAKVATWTDAVVVVVGLSQDQEREAFDRTSLRLPGQQEELITTVSRV
AKGPVILVLMTGGPVDIGFIKDDPKIQSILWVGYPGQAGGQALAQVIFGDRNPGGKLPMSWYPESYTEVPMTDMHMRPDESTGYPGRTYRFYSGDVIYRFGEGMSYTTF
SSSFVSAPSVVTASANSRQSCSQRQRDSPNIPCSSADNALVCNSMKIEIVVSVKNDGPVAGTNVVLLYHTSPTAGKDGSPLQKLVGFERLYLESMQEQKALFKVNLCRQ
LTEAQTDGTWSNLIEGVHTFSLGNSNDPKHEMKLIYEHPS

>Mp_bglu_II
MGKGSCVTLLLFIGLVSSVYSYDLYKDPSQDVEARVQDLLSRMTLAEKVGQMTQIERGVSNNSVVKDLFIGSVLSGGGSVPDAGNTAEAWQDLVDRLQNDALSTRLGIP
MIYGIDAVHGHNNVFGATIFPHNVGLGCTRDPDLVKRIGVATALEVRATGIPYVFAPCIATCRDPRWGRCYESYSESTEVVTSLTDIILGLQGEPANKTAGFPFVADSS
KVAACAKHFVGDGGTTNGINENNTVIDYKGLVDIHMAPYFDSIAKGVSTVMISYTSWNGMKMHANKFLVTQVLKYNLKFKGFVISDWEGIDRITSPAGANYTFSIESSI
NAGLDMIMVPNNYPQFINGLTDLVKGGYINMHRIDDAVTRILRVKFAMGLFEKPLADRSYSKHLGSEEHRSLAREAVRKSLVLLKNGKEAGKPLLPLSKTAAKIIVAGS
HANDLGRQCGGWTITWQGGNGTTTKGTTILDAIKGAVSPTTQIVYEPYPQPGFAQSQGADFAIVVVGEPPYAETFGDNLNLTIPEAGIPTIQNVCGEVRCLVILVSGRP
LVVEPYLPVMDAFVAAWLPGSEGAGVSDVIFGDYDFVGTLARTWFKSVDQLPMNVGDEKYDPLFPFGYGLKMGTPNSSGGSINFMRRWRLLDISCWAVPLIIHMLKL

```
>Mp_bglu_III
MTLDEKIGQMTQIERYVANFDVMKNFSIGSVLSSGGSTPNSSTTEAWQNMVDDLQAGALATRLGIPMLYGIDAIHGHNNVYGATIFPHNIGLGCTRDPDLVRRVGAATA
LELRATGIPYTFAPCIAVCRDPRWGRCYESFSEDTSVVRMMTDVIYGLQGNGTPGIPFVSDRSKVAACAKHYVGDGGTQRGINSNDTILSYEDLFRIHVAPYVDAIAKG
VSTIMLSYSSWNGVKMHMNHRLISTLLKQELGFKGFVISDMEGIDFITDIPDANYTASVLESINAGLDMIMVPFDYEKFISTLRTLVNTGYISMQRIDDAVTRILRVKF
SMGLFEHPLADRSFSPHLGSQDNRMVAREAVRKSLVLLKNGKTESRPFLPLSKNATRVLVAGTHADDVGLQCGGWTISWQGSAGSITKGTTVLDAVKAAVFTTTQVIHE
ASPTAEVAAKTKADFAIVVVGEQPYAEGAGDNTNLTIPDEGISTIKNVCSEVKCLVILISGRPLVVEPYLPLMEAFVAAWLPGTEGNGVTDVIFGDYDFVGSLSRTWFK
SADQLPMNFGDPIYDPLFRFAFGLGMGKLPR

>Mp_bxyl_III
MRVYVLAVLLLIQHLLAQNMSGASSGSSYACTNSESKSWAFCDKALSIEKRVADLISRLSLQEKISQLGNGADDISSIDVPAYQWWGEALHGVASSPSVHWDGPVKGAT
SFPMPISLAASFNKSLWNKIGQVISTEGRAMHNLKQSGLTFWSPVINLVRDPRWGRVQETPGEDPYLIGQYSVYFVRGMQEAKDYDPSNQDDDGNEYFALKTSACCKHY
TAYDLDNWHGIERYYFNAEVTKQDLADTYNPPFQSCVQEGRASCLMCSYNKVNGVPTCGDPDLLKGIVRKAWGLRGYIVTDCDSLLVMYDQSRYSRSPAEAIATAMLAG
LDLNCGSTIKNHGATAVNQSLISEDDIDRALSNTLTVLMRLGIFDGSPLDQPYGSLGEDDICSDEHQDLAREAAVQGIVLLKNEEDSLPLSPSQIKTLAIIGPNADDKK
YTMLGNYAGRPCKYVTPLQGISTYHIDTVVFETGCSSIDCSSDEKIEDAVSAAKKADAVILVMGLDQDLERETFDRNSLKLPGKQELLVSSVANVSSGPVVLVLMSGGP
LDISWAKTDSKIQSILWMGYSGQAGGLALAQIIFGDRDPVGRLPVTWYPETITDWPMTNMNMRPDPDSGYPGRTHRFYNGPTVYEFGYGMSYSKSTTTEVTAPTNFKAP
DLFQQACYHKRLTTEQIDSIGCLQSDLDACMEASLPMSITVKNGGPTAVTEVVLVYHVPPRAGEGGTQLKELIAFERTEVQSFSSKIIDLTINLCYHTSTVKQDGTRAL
QLGIHKFVVSSGSGTPAEHSFSVQ

>Mp_bxyl_IV
MAAWRYLAVFFVFHHIIQIFQTFVVHGTALHGCGSDLDASILAMPFCNISIPIADRTLDLVSRLTLQEKIAQLVNTAPEVTRLGIPRHEWWNEALHGVAYSPGVTFTGP
ASGATSFPQPILTASSFNMTLWTLVGQAISTEGRAMYNLGQSHLTFWAPNINIFRDPRWGRGQETPGEDPLLTSIYASTFVRAMQEIRYSDEGSRYKAPFGVQMVPSEL
DTTEERSPAVGRMLKTSVCCKHFTAYDVENWDGIDRYHFNAEVSDQDLEDTYNRPFQSCVQEGQASSMMCSYNRVNGVPTCANYNLLTETARNTWGLNGYITSDCDAVE
LIYSAVNYAPSAEDAVAEVLMAGLDLNCGSTAANFGQSAVDQGKVNESTIDRALFDLFSVRMRLGEFDGDPENQIYGSLGLDDICSQYHKELAIEAAGQGIVLLKNDNN
VLPFLRGHIQKLALTGPNANATSSMLGNYAGVPCEYITPLQGLQTYVDLLLYEPGCWDISCQDGGLIGAAAAIASSADAVVIVAGIDQTQEQETLDRTSLLLPGQQQSL
IETVANSSKGPVILVIMSGGAVDISFAKFNPQIHGILWVGYPGQGGGQALADILFGHRNPGGRLPVTWYPESFTQVSMTDMNMKPNTSSGYPGRSYRFYTGETVYSFGD
GLSYTNFLSTITSAPSRFLIPSLEQQRCYHNFNSTSKCPKGFQNELTCDTASFEVRVSVSNQGVRRGSETLMLYSVPPKSGIGGEPLKQLIAFQRVSLDAYSSMDVGFE
VLPCKHLSSVKYDGTKELVEGTHTLVLGSKDEVSHAVSFVTNFF

>Sf_bglu
MDRTELLLAISFTLIMLAASSHGGVNAAAAQSPTKLLYKDFNQPVALRVKDLMSRMTLAEKIGQMTQIERSVANASDIEHYLVGSVLSGGGSVPAPNATAFQWNNMTDY
FQRSALKTRLGIPLMYGIDAVHGHNNVYGATIFPHNIGLGATRDPELAQRIGVATALEVRSTGIQYVFAPCLAVCRDPRWGRCYESYSEDPDVVTSMTTIIDGLQGQAP
VGWEGPYVENNYKVAATAKHFVGDGGTTHGIDENNTVISYKGLVDIHMKAYFSAVARGVSTIMVSYSSWNGEKMHANRFLLTDVLKGQLGFKGFLISDWEGLDRITTPY
DANYTYSVFTGIHAGIDMVMVPFDYEGFASNLTKEINIGNIPMSRIDDAVSRILRVKFEMGLFEYPFGEPQLKSSLGAYPHRELAREAVRKSLVLLKNGKTANTPLLPL
DKNAQKILVVGAHANDIGLQCGGWTITWQGSPGNTTLGTTILQGIQQTVGSSSEIVYKAVPDKNFAKGKGFDYAIVVIGEVPYAEFVGDNNNLTIPAPGIETVEYTCKA
VKCVVVIISGRPLVVPPYLLQSMDAFVAAWLPGTEGQGIADVLYGDYDFTGKLSRTWFKSVDQLPMNVGDDYYDPLFPFGFGLKMGLKNR

>Cp_bglu
MRSSLTKPGNKYTKKGNSLTQQLRNSLGRPLTVKFLTRAFAMALTANVLLAMVVAFLAFQKSESAKNHPPARKTYRLYQDMGQPVEERVKDLLSRMTLAEKIGQMTQTE
RSVTNHTNIRDFGIGSILSGGGSGPVENATVLQWGNMTNYFQKAAMNTRLAIPLIYGIDAVHGNNNIYGATIFPHNVGLGCTRDPELVERIGSATALECRATGIQYAFA
PCIAVCRDPRWGRCYESYSEDPEIVRDMTSLIDGLQGRAPAGWDGPYVEDSDKVAACAKHFVGDGGTTRGLNGNNTQVSYRELVNIHMKAYKDAVRKGVATVMASYSSW
NGVKMHANKFLLTRVLKKELGFKGFIISDYMGIDLITDPPGANYTYSVYAGIHAGLDMIMVPFAYEQFIGNLTQMVKSGAIPMSRINDAVTRILRVKFQMGLFESPYSD
TKLVKTVGQESHRELSREAVRKSLVLLKNGKTPLTPLLPLNRNAKRILVTGSHASNIGLQCGGWTIKWQGKSGDITPGTTVLEGIQQAVSPDTEVVYAEKPGKGLFENL
GFDYAIVVVGEPPYAETHGDNLNLTIPLNGPHAITHTCRRVRCVVVLMSGRPLVVAPLLHQMDALVAAWLPGTEAGLGIADVLFGSYDFTGKLARTWFRSVDQLPMNVG
DKHYHPLFPFGYGLTMGLNNSRYIVLLVVLFLHM

>Pp_bglu
MAMALTSRLVLVTLVAFLAFHGTRAKQSSVSRDRYRLYQDAKRPVEDRIQDLLSRMTLLEKIGQMTQTERTVTNHTNIREFGLGSILSGGGSAPAENASVFQWDNMTNY
FQRAAMSSRLQIPINYGIDAVHGNNNIYGATIFPHNIGLGCTRDSDLVERIGTATALESRATGISYVFAPCIAVCRDPRWGRCYESYSEDPEIVRNMTSLIDGLQGRAP
PGWDGPYVESSDRVAACAKHFVGDGGTTDGINGNNTEVSYDELVNIHMKAYKDAIDKGVTTIMASYSSWNGVKMHANHFLLTKVLKEQLGFKGFIISDYMGIDQITDPP
GVNYTYSVYAGIQAGLDMIMVPFAYDQFIGNLTQMVKSGLIPMSRIDDAVTRILRVKFQLGLFERPYSDNKLKLSVGHDWHRQLSREAVRKSLVLLKNGIYPGSRLLPL
NRHAKKILVVGSHANDIGLQCGGWTIHWQGGFGDITPGTTVLQGIQQAVSPTTEVVYSERAKKSLIKDQDFDYAVVVVGEPPYAESQGDNTNLTIPLMGTHAIRNTCRY
VRCVVVIISGRPLVIEPYLPMMDALVAAWLPGTEAGLGIADVLFGAYDFTGKLSRTWFRSVDQLPMNVGDKYYDPLFPFGFGLSMGIDGTRN

>Sm_bglu
MLLLLLLLAFDASLSRAALYKDTSQPIHARVQDLLSRMTLEEKIGQMTQIERENATGSVITKYFIGSVLSGGGSVPAPRASAATWAAFVDGLQDGALATRLGIPIIYGI
DAVHGHNNVYGATIFPHNVGLGSAGDPDLVKRIGAATALEVRATGIQYTFAPCVAVCRDPRWGRCFESYSEHPELVKAMTTIISGLQGETPAKGVPYVGGSSKVAACSK
HYVGDGGTRSGINENNTVGSYKRLVGTHMLPYFDAIDKGVSTVMISYSSWNGIKMHKNRHLITDILKKRLRFKGFVISDWQGIDRITNPAGANYTYSVLVSVTAGIDMI
MVPYEYTKFIDTLTSLVKQGFISLDRIDDAVRRILFVKFTAGLFEHPKSDSSYRSQIGAHRDLAREAVRKTLVLLKNGKNAKYPLLPLSKTASKILVAGSHANNLGNQC
GGWTITWQGASGNTTLGTTILQGISNTVSKNTQVVYEESPSSSSVKGGGYDFAIVVVGEPPYAETQGDNLNLTIPQDGANTIESVCSSVKCLVILISGRPLVVAPHLSS
MDALVAAWLPGSEGQGIADVIFGDYDFQGKSSRTWFKSVEQLPMNYGDVEYDPLFPFGYGLKMGKK

>Cr_bglu
MHRQEKRGKELHCAADDGAMPPIYKDPGRPVEERVEDLLARMTLAEKIGQMTQIERLVADPSVIQNYFIGSILSGGGSAPAPNASPSAWADMVDGFQTAALSTRLGIPI
IYGIDAVHGHNNVYGATIFPHNVGLGATRDPNLVQRIGAATALEVRATGIPYAFAPCIAVCRDPRWGRCYESYSEDPELVQQMTQIVFGLQGTPPEGYPKGAPYVGGGS
NVVACAKHYVGDGGTTGGIDESNTVATFNELRRIHLPPYYNAIAAGVATIMVSYSSWNGVKMSANKFLISTMLKQRLGFQGFTISDWEALDRITSPPDANYNYSVNVAI
NAGLDMVMVPMNYQGFINLMTAQVNAGQISQDRIDDAVRRILRVKFLAGLFEKPMADRSLLQMVGSEEHRNLAREAVRKSLVLLKNGKDSTNPMLPLSKNAPKILVAGA
HANDIGLQCGGWTITWQGSRGNTTVGTTILEGIKNAVSPTTQVDYLVSPDSGNAANGGYSYAVVVVGEPTYTEMFGDNDNLTIPQDGINLIQNVCSVVECAVILISGRP
LVVEPHLPLMSAFVAAWLPGSGGQGVADVLFGDYDFQGKSPRTWFKNVNQLPMNIGDQSYDPLFPYNFGLTTGVGKTG
```

>A0A3N7HH59_9BURK
TLAQKVGQMTQPD-----IRSITPDQVKQYYIGSVLNGGGSVADWLALADAYWQASMDSDM-AVKIPVIWGTDAVHGHGNVYGA-TLFPHNIGLGAANDPDLVERIGAA
VAKQVVSTGIDWTFAPTLAVVRDDRWGRTYEGFSEDPEIVGSYGGRYTTGLQGNTVVATAKHFMGD--GGTDQGKDQGENKSSLNDMMNIHGAGYYSALAAGAQTVMAS
---FNSWTFKKMHGNQYLLTDVLKGKMGFDGLVVSDWNGIGQVKYKDSTNSSCPPSINAGVDLIMVPDDWKAFITNTIASVNAGEIPMSRIDDAVTRILRVLVLLKNDG
GVLPLKR-GEKILVVGRSADMSNQTGGWSLTWDFPNGDTVLAGIKEAAGDTYALDASNATLTDYKAVIAVIGETPYAAGDIGKTGTLEHAPADLALLESLKGKGVPVVT
VLMSGRPLWVNRE-INRSDAFVAAFLPGTE-GKGVADVLFRKADFQGKLSYSWPKSACQVPLNK--G-D---ASYDPLFAYGYGLTY
>A0A4R2CI28_9ACTN
TLAEKVGQMTQAE----RNALRSRTDIATYALGSLLSGGGSVNTPAAWAAMIDGFQLNAQATRLQVPLIYGVDAVHGHNNVIGA-TLLPHNIGMGATRDPELSRRAGEL
TATEVRATGIPWDFAPCVCVVRDDRWGRAYEGFSEDPALAKAMAT-VITGMQGKHVLATAKHFVGDGGTTYGSSTDQGVTEVTPQQLEAIHLDPFKTSVDLGVGTVMPS
---YSSLDIIKMHGDAALINGVLKQRMGFDGFVISDWQAIDQL--PGDYPSDIRTSINAGLDMIMVPTNYQAFTQGLTDEVTAGRVTQGRVDDAVGRILT--VLLKNQD
NVLPLASTA-KVYVAGSNANLGNQMGGWSITWNTTTGTTILDGIKQVVPTATFSQDASAPLEGHDVGVVVVGERPYAIGDVGNGHDLALSDADKAAVTKVCAAMKC-VV
LVVSGRPQVIADQ-LGDIDALVASWLPGTE-GAGVADVLFGKRPFSGRLPVTWAKSEAQQPINVGDA------SYDPQYPYGWGL--
>A0A1L9SYA0_9EURO
---NFSSATSFPMPIVMAASFNDDLVFAIGET
VSIEARAVGLNLDYTPKINLFRDPRWGRGSETPGEDPLLAKNYVASLIHSMEDQRIIATCKHYAGNDLESVGSRYGFNAV-ISTQDLSEYFLPPFRTCTQKQVSSVMCS
---YNAVNGVPACADSYLLQDILRDHWRWDHYITTDCGVIGYMVTDHHLGESAALALKAGSDLECNGGPN----SWLLSAWNRSLITEREVDRAAQRL---TVLIKND-
GALQLSSN-QSYALIGPWVATEQLQGIYYGPAPF--LISPLPAAKDLGINPSTYDAAIAAARSADVVIFLGGIDSSFEKETQDRESLEWPTPQMELITALAENAEQLVV
VRYGGGQLDDSELTNENESGLLWGGYPGQSGGQAIMDLLFGKASPAGRLPVTQYAAWYDVPPDMSLR--PYMYDGEPTAPFGFGLHY
>A0A0C5V292_9GAMM
TLAEKIGQMVQPE-----IKQASPQDVIDYHLGSVLNGGGSAADWLAMADSYWEASMNTDDGKAAIPLIWGTDAVHGHSNVMGT-VLFPHNIGLGAARDPALIKRIGQA
TARQVTATGIDWTFAPTLAVVRDDRWGRSYEGYSEDGEIVFNYGGAMVEGLQGDHVLATAKHYIGD--GGTDQGDDQGDNLSNEHDLINIHGQGYYSALSAGAQTVMAS
---FNSWNGEKLHGHEYLLNTVLKGKMHFDGFIVSDWNGVGQV--TGCTNSHCPQAINAGIDMIMVPDDWKALITNTIQDVNDGAIAMSRIDDAVRRILRVLVLLKNNA
DTLPLSKDA-RILVTGKTADMMNQTGGWSLSWDFPYGETILSGINSAISSVTYSQDGSAADDSYDVIIAVIGETPYAEGNG-DIGKFSTLPADAKLLDTLNSNAVPVVT
VYVGGRPLWMNPE-LNKSAAFVAAWLPGTE-GAGVADVLFGDYPFTGKLSFSWPATDCQVLV--NRN-----DGQTPLFAYGFGLS-
>R1EJJ5_EMIHU
---DKIGQMTQLDISMRIDDAKLRRQLRVHRLGSLLNSPFNASEW---RASVEHVQAAAADE-GLPPLIYGIDSVHGAS-YCRGATLLPQQLGLAASFDESLAAASGWL
TGKDSRAAATPWMFAPILGVATHPLWPRVYETFGEDPLLVARMGAAVVEGMQRRRAAACMKHFVGY--SAPRNGHDREGAWLSRRELEQTYLPPFQAAVDAGVQSAMES
---YQEVNGEPVVGSAALLQGLLRERLGFEGLLVTDWHEVGNLHDFHRPEEAVLLAISSSLDMSMVPTDESF--SV-------------------------------
---------------PAAD---SLGRLSGGWTAHWQGTLDDEIRGSRPGTNSSAELIAAAAAADVVVACLGEDPHTEKPG-DLDDLSLDAGQIDLVRVLASAGKPIAL
VLVTGRPRLLHGVALPQVNAVLQSFLPGPHGGAALADALVGRFEPSGRLPISWPTAASSLPH------------------------
>A0A7H8R6L8_9EURO
---DFSYATSFPLPILMGASFDDDLIQNVARV
IGKEARAFANAQKWTPNINGFLDPRWGRGLETPGEDIFHIQNYVKQLVPALQGVQIIATCKHYAVY---DVETNRTGQNYDVTQQDLGDYYLPAFKTCADALATSIMCS
---YNAVDGVPSCASEYLLQMVLRDTWGFDNYVVSDCDAVNNIWDSHGVDSAAAVALNAGTDLN--CNGAY---KNLVGSVAGNMTTESAMDQSLTRLY--ITLLKND-
GLLPLADTYSSVALIGPWANTSQMQGNYYGV--APYLISPLEAFQASFDKDAGFSEALSAAQSSDAIFYMGGIDTTVEAEGMDRTSVEWPGNQLDLISQLGALGKPLVV
VQFGGGQVDDTVLQNSSVNAVVWAGYPGQNGGNAVCDVLTGKKAAAGRLPVTQYPADYTNEANIFDPTRPYRYTGEPVLPFGYGLHY
>A0A1M5IXT7_9ACTN
TLADKVGQMTQAE----RVAVGDGTDVTTYALGSLLSGGGQPNTPAAWAKMIDGYQTQALATPLQIPMIYGIDSVHGDNNLAGA-TLFPHNIGLGATRDPALVKQAGAV
TATETRATGVPWAFAACVCVVRDDRWGRSYESFGEDPSLVTDMTT-VFQGLQGDSVLATAKHFIGDGGTRFSSQTNDYTIDQGITYVTNQLVAPYRAAIKDGVGSVMPS
---YSSLRIQKMHGRKDMITGLLKQKLGFKGFVISDYAAIDQL--PGDYKSDVQTSINAGLDMIMVPNDYKTFITDLTDLAGNGV-PLSRIDDAVTRILT--VLLKNSR
NVLPLAKRS-KVYVAGSNADEGNQSGGWTLTWAIPGATSILDGLRKDDPTFTYSKTATASAKGYDVGVVVVGETPYAEGQGDNKHTLQLSVADRQAVNRVCGAMKC-VV
MVVSGRPLDITGV-APQAEAVVASWLPGSE-GTGVADVLTGRRPFTGRLPVTWAKAESQQPINVGDK------RYDPLYPYGWGL--
>A0A364LBE3_9EURO
---FSYATSFPAPILTAASFDDGLVRKIAGV
IGKEGRAFANNFFWAPNINPFRDPRWGRGQETPGEDTFVVQSYIRNFIPGLQGNQVIATCKHYAVY---DLETGRYGNDYNPSQQDLADYFLAPFKTCVDTGVGSVMCA
---YNAVDGIPSCASEYLLEQVLRQQWNFTAYVVSDCDAVTDIWRYHNEEAAASVAMNAGTDLECGSSYL-----KLNESLAASQITARSIDRSLTRLYS-MTLLKNDQ
NLLPISHNYKSIALIGPFANTTQMQGDYSGV--PPYLISPLQAFETHS-ETAGFGPALAAAEKSDLTIYLGGIDNSIEAETLDRTSLTWPGNQLDMVTQLSHLHKPLIV
VQFGGGQLDDSSLQNEGVQALVWAGYPSQSGGTALLDVLTGKKSIAGRLPVTQYPASYDINLPALNGSRTYKYTGKPVIPFGYGLHY
>A0A7J6VWA4_THATH
---TIPSATSFPQVLVTTASFNRSLWFLIGAA
TAVEARAMGQATFWAPNINIFRDPRWGRGQETPGEDPMTVAAYAVEYVRGFQGEMLSACCKHLIAYDLENWKNFYDFNAV-VSDQDMQDTYMPPFRSCVDAKASCLMCS
---YNEINGVPACANKDLL-QQARVEWGFKGYIASDCDAVGTVYEYQHAADAVADVLKAGTDINCG--TYM--LLNTQSAIEQGKVQEEDIDGAL------IVLLKNDR
NFLPLKFSVASLAVIGPTANGTSNFGTYTGIPC--DAKSFLDGFQAYTQNTDGFAEAVRIAQMADVVIVVAGLDLSQETEDLDRVSLLLPGKQMDLIAAVVASKRPVVL
ILTGGGPIDVSFAEDTRIASILWVGYPGEAGGLALSEIIFGKYNPGGRLPMTWYPESFTTPMDMRMRSSHYRYTGETVYEFGHGLSY
>A0A437A6N7_9PEZI
---RFADDTAFPAGVTVAATWSKYHMYARGKA
LGAEARAKGVNVILGPCIGLGRFPVGGRNWEGFGSDPYLQGIAAGITIDAIQSEGVIATAKHYIGNEQVPEAHGQGWPNVNIGDRTLHELYLWPFAEAVHAGVGAIMCS
---YNQVNNSYACQNSYLLNGVLKDELGFQGFVMSDWL-----AH-----HSGVASALAGMDMSMPGDAKSFLGSFLTESVVNKTLPIERLDDMALRIV--IVLLKNFN
SALPLEKLPRAIMIFGSDAGGTLGQGWGSGSVDYGYQITPLEAIQARLLGDRDLGRASRLAETPGKCFVFVSSDSGESSEGHDRNDLNLWHGGDDLIKAVAAKCNDTVV
VIHSVGAVVMEEWDNPNVTAVLMAHLPASESGSSLVNVLWGDVPPSGHLPYTIGKSLADYPYDILREP-NYRFDANGIFEFGFGLSY
>A0A4Q2LY88_9BACL
TLQEKIGQMLQVE-----RLAATPSQVAQFAIGSVLSGGGTPNNAATWAAMTDSYQQAAMSTRLQIPILYGVDAVHGHNNVYGA-TIYPHNVGLGASGDADLVRRIGDA
TAREIRATGVNLNFAPCLCVPQDIRWGRTYEGYSENVTLAGKLGTAFVEGLQGNKAVASIKHWLGD--GNTTGGDDQGNVTLNEQEL-DPYIQPYRDAIAAGARTVMIS
---LTTWNGQKMHVHQHLITEMLKQDLNFQGIVISDWNSLVNQGVYATYAEALRASVNAGIDLFMEPDNWQQFIPTLVNLVNTNQVSQARINDAVSRILRV-VLLKNDN
HFLPLSKQ-SRLFVAGSKAH---NIGFQSGGWTISWQGTLLQGIQGAVTGSVTYSQNGTGAAGHDAAIVVIGEYPSAVGPGQPRPNLELSAEDRTLLTNVAGSGVPMVV
IMLSGRPMIVSSD-LPGWQAFVAAWLPGTE-GGGIADLLFGDDDFTGKLPLTWPRSMAQIPI--TDKD----QNYTPLFPYGYGLS-
>F9FY86_FUSOF
---ASVFPAGVSAASSWDKDLLYQRGLA
MGQEFKAKGAHILLGPVAGLGRSAYSGRNWEGFSPDPYLTGIAMEETIMGHQDAGVQATAKHFIGNEQTFGEVDKEALSSNMDDRTMHELYLWPFANAVHAKASSMMCS
---YQRLNGSYACQNSKVLNGILRDELGFQGYVMSDWG----------ATHTGVAAINSGLDMDMPGGIGQYGGGNLTRAVNNGTLDETRVNDMITRIMT-TVLLKNEK
NALPL-KKPKSIAVFGNDAGGTLVAGGGSGTGRLTYLVSPLTAINARAKQATTNVTDLWIPATPDVCLVFLKTW---AEEAADREHLSVDWDGNDVVESVAKYCNNTVV
VTHSSGINTLPWADHPNVTAILAAHFPGQESGNSLVDLLYGDVNPSGRLPYTIAFNGTDYNATAVNT--TYRFDAHNIYEFGFGLSY
>A8LFQ8_FRASN
-LEEKVAQLRSIWISKGTFDPARAHVLIPDGIGFVGRPVDNWHREETIAFVDAVQRYLVEETRLGIPALFHDETAHGFVARGA--TIFPIPPALASTWDEDLVEEVFTV
VAREARSVGGSTVSLGPVLDLARDPRYGRVEEFFGEDPYLVGRMGVAAVRGLQGRRMFATLKHFL-H--ASPEGGINAAPAPAHERSLRETYLAPFVDVVEANPAFIMPS
---YNEVGGLPSHASRDLLQRLGRALLGFEGVYLSDYDALARLISDHRLGEAAAIGLTAGVDVDLPDGEAF---SMLAPLVREGLVDETLVDEALARVLA--TLLTND-
GILPLPNAEIRLAVVGPNAG-ELYYGGYSGENDA--GVSVLDGLRAAIVGRRRIKDAVAVVERADVVLLVVGDHPAIARETTDRNELGLYGLQEELVEAVVQVGKPVIA
LLVNGRPIAATRL-AAGANALLEGWYLGQETGNAVADVLFGRAEPGGRLPVSVPRASGAVPV-YYDRHANYVVDRTPLFPFGHGLGY
>A0A1F5LSY9_9EURO
--SRLGFPGLCLSDAGNGLR--STDLNGYPAGLSVGASWNRKLTTQRAHF
MAGEFKAKGVNIALGPVVGLGRVARNGRNWEGFSNDPYLSGVLAADTVTAFNKRGVMTSLKHYIMNEQTGSTPQTEAVSSNVDDKTIHELYLWPFQDAIKAGSVNIMCS
---YNRINNSYGCANSKTLNGLLKTELGFQGFVVTDWT----------AQHSGVASALAGLDMVMPDTKYW--GDNLTTAINNGSVPESRLNDMATRILA--VLVKNN-

HALPL-KKPQVLSVFGYSAAGTIIMGGGSGATTPTYVSGPLDALTNRAMKWDVESPAPEGVPASDACLVFINAW---ASEGYDRPGVYDD-YSDNLVLSVADQCGNTIV
VIHNAGVRLVDNFDHPNVTAIVYAHTPGQDSGAATVALLYGDENFSGKMPYSVPKNISDYPSDYVNY--PYRFESRNIYEFGFGLSY
>A0A540MGK2_MALBA
--LGATSFPQVITTAASFNESLWEEIGRV
VSDEARAMGL-TFWSPNVNIFRDPRWGRGQETPGEDPVLAAKYGARYVKGLQGDKVAACCKHYTAY-DLDNWNGVDRFHFNVSKQDLEDTYDVPFRACVDGNVASVMCS
---YNQVNGKPTCADPNLLKGTIRGQWRLNGYIVSDCDSVGVYYDDQHPEEAAADAIKAGLDLDCGPFLAI----HTEAAVKTGLVNEIDIN---------IVLLENRG
NSLPLTTRHRTVAVIGPNSDTETMIGNYAGVACG--YTTPLQGIARYTRTNQLIGAAEAAARQADATVIVIGLDQSIEAEFRDRTNLLLPGHQQELVSRVARASGPTVL
VIMSGSPIDVTFANDPRIGAIIWVGYPGQAGGTAIADVLFGTTNPSGKLPMTWYPQNYKLPMDMAMRA-NYRYKGPVVFPFGLGLSY
>L0K850_HALHC
TLDEKIGQMTQ-----GERRHVSPKQVRKYHLGSILSGGGSTPGNNTPQDWIDMYQEEALSGRLELPLIYGVDAVHGHNN-LKGATIFPHNIGLGAMGKGLWIEKIARI
SAQETAATGMDWDFAPAVSVVRDERWGRSYESFGETAELQKLLAGPYVKGLQGTHVVATAKHFIGDGATKWETGYDRGNVNIDLNKLKKLHGQGYLEAIDENVGTIMIS
---YNSYQGTKMHAHQELIQNYLKAGLGFDGFVISDWAAIHEIDAPTHYAK-VVKSVNAGIDMFMEPSDWHKFMIDLKTAVKNGDVKESRINDAVKRILK-LVLLKNQN
QILPLSKDN-KFYITGSNAD---NLGHQCGGWQATTGTTIKEGIANLL--QGQKGQIVNDLNQADVAIAVVGEKAYAEGKG-DDADLELSVSDKRELQRIEESGKPMVV
ILVSGRPMIVSPR-IENWDVFVAAWLPGTA-GGGVADVIFGDYNFTGKLPVSWPRSVEQLPLNVGDK------NYNPLFNYGYGLK-
>A0A0P7C2D2_9HYPO
--RLGWPGMCLHDAGNGVR--ATDLNSYPSALHVGASWDKNLTYQRGLY
MAKEFKAKGVNVLLGPNAGLGRTPLGGRNWEGFSVDPYLSGQLCAETIIGHQDAGVIANVKHFIGNEQETFRRGVEAASSNIDDKTLHEFYLWPFVDSVKAGVASVMCS
---YNRINNTYGCENSKLMNGVLKSELSFDGFVLLDWN-----------AHHTLESANAGLDLVMPQGGSF--GENLTQAVGNGTVSEARVTDMATRII---VLVKNEN
NALPFRKNPKMLSVFGYDATGTIVSGGRAAANSPPYMSDPLSAIQHRAAKWDLSSFDPGVNAASDVCLVFINAI---ATEGWDRDGLHDD-FSDGLVLNVASKCANTIV
VIHAAGIRLVDQWEHPNVTATIIAHLPGQDSGRALVKLLYGEASFSGKLPYTLAKNETDYPY---APCDTYRFDERDIYEFGFGLSY
>A0A364MTF0_9PLEO
---FSNATSFPMPLLMSAAFDDDLIYQIANV
IGNEARAFGASVDWTPDINPFRDPRWGRGSETPGEDILRIKGYTKHLLAGLEGNKIIATCKHYVGY-DMEAWGGTNRHRFDTTMQDLVEYYMPPFQQCADSKVGSIMCS
---YNAVNGIPTCADTYVLQTILREHWNWTGYITSDCEAVADISENHNLAEGTALAFANGMDLSCEYSGS----SDIPGAWKQGLLNTSVVNRALTR----LVMLKND-
HTLPLLSNGSNVAMIGFWANSSKLSGIYSGP--PPYLHTPRLGL-----NDNWTTKALNAAQRSDYILYFGGLDTSAAAEGFDRTDISWPSAQVDLITKLSQLGKS-LV
VIALGDMVDNSPLSMEGVNSVIWANWPGQDGGSAVMQVVSGAYSVAGRLPITQYPADYSL-SDM---NRTYRWYNESVQPFGFGLHY
>A0A2Z7CXI4_9LAMI
---GATSFPTVITTAASFNQSLWKKIGQV
VSTEARAMGL-TFWSPNINVVRDPRWGRALETPGEDPYVVGEYAVNYVRGLQDVKVAACCKHYAAY-DVDNWLGIERYNFDVTEQDMLETFLKPFEMCVEGDVSSVMCS
---YNKINGIPACADPRLLRGKIRGEWDLHGYIVSDCDSIEVMINHQKPEDAVAQALKAGLDLDCGNYYT----NYARNSVGKGKVSEKDIDVALK-----IVLLKNDN
QTLPWTDEIKTIAVVGPHANTSAMIGNYAGVPCQ--YTSPIDGFSKYGKVDSIIFPAVRAAKKADATVVMVGIDLSVEAESLDREDLNLPGYQNQLINLVASQSKPVVV
VIMSAGGVDISFADSSKVHSILWAGYPGEEGGKAIADVVFGKYNPGGRLPLTWHES---MPLR------PYKYNSSTVYPFGYGLSY
>A9URG4_MONBE
------------------------KLMQEMTLAEKIA---MLHGYSGTSQNYNYTGFVVPNDRLKIPALQLNDGPQGFRATNDRTTAWPSGLTMAASWDVAMLSKWGEG
MGAEFAAKGANVQLGPGLCVARVPVNGRNFEYLGEDPFLGYTLVQPVIQGIQSQGVIANAKHYVNN---NQETKRTTVSENVDERTRFEIYYPPFEGAINADVGSFMCS
---YNKINSAWSCENNETLNTDLKHRLNF--WVMSDWG---------A---THSTSIDKGLDQEMPGDSHM--GDTLADMVSNGTVPMALINASVLNILT--VLLQNEN
KVLPLGTANTTYLIVGAQAWQTIVAGGGSGHVDIPYMVTPLDAFKTALGFGSDTDMVASTAAEVDYVLAFVATT---SHEGADRANLSLAGGQDEFIIAAASHSDKVVV
IVTTPGAILM-PW-SADVASIVVNFMPGQEGANAAADVLFGTVNPSGKLPLTMPNVENQYPGAYYD---EYDHGIEPRFPFGHGLSY
>A0A2P6TID9_CHLSO
--QRECLHGMVGDNGESVMYPMPIAWGATFDDGLVWQAALE
IGDAMRAFGFTHCYGPHPAIVRDPRWGRSAEVYSEDPKVASNMANMFIQGLQGGKVSATCKHLIGNDLENWYGITRYNLNAIDYRDLRDTFWLPFESCVRAGAAAIMCS
---YNKVNNEPACLSKTLLTTVLRQQLGFKGFVATDCDALEGYAKPAPLRTISVQALRAGSDQA-------------------------------------IVLLKNTQ
NALPLRTALRRVHLVGPWADGVYQLGSYYAIPAA-NNVTPRQALQTALPGAQNVATDAQQCQLADVCILFLGSRMHREGEGRDRTSLKLNPNQEALWKDLVRTSKKIVV
VLMHGGGLDISDMPGARISAVMTTWFPGQG-APGIADVLLGNVAPAGRLPTTWYTNMADMRMSS-----TYWRGAAPLFPFGYGLSY
>A0A1W2TH17_ROSNE
--GNSTPWSYATSFPMPIHIGASFDDDLVYRIAQT
VGKESRAFANNAHWTPNINPFRDPRWGRGQETPGEDPLHLSNYVYNLITGLQGGLIIATCKHFAVY---DVETGRHENNLNPTPQDLTDYYMPPFKACADAKVGAVMCA
---YNSVDGTPACANRYLMQTVLREHWGWSQWVTSDCAAIEDIHQNHKYADAAAAAVNAGTDLA--CEGSI--YNQLVEAVALNLTTEATIDKSLSRL---ITLLKND-
GALPLPRGVGNVAVIGPWGNTGIMQGNYQGV--APYLISPLEAMDGKWENTANFAAALDLAAKSDYIIYCGGIDISIEAESRDRVNIEWPGNQPDLITQLAELGKTLIV
VQFSGGQVDDSAISNPGVNAIVWAGLPGQSGGTAVADVLDGTKSPAGRLPITQYPANYQLPVPGLQPNETYKWYSTPVLPFGYGLHY
>A0A3E2GT90_SCYLI
--RLGVPSIRLSDGPNGVRTRFFNGACFPCGTALAATWNVPLLEEAGRL
MGEEAIVKGSHVLLGPTVNMQRSPLGGRGFESFSEDPILAGMCAAAIVRGVQSTHVVATIKHFVAN----DQEHERMAVDSITQRALREIYLLPFQIAVDAQPGAFMTS
---YNKVNGIHVNDNPKFLQDILRGEWGWEGLIMSDWY--------GTYS--TVAAMKAGLDLEMPGPSKW-RGQLITHSLMAKTLSPITLDDRVRNILK-IVLLKNER
NVLPLDA-SKTVAIIGPNAKMAAYCGGGSASLLPYYTVTPFDGISSKATPVEFASGVTSKLTDADGVVSFGGGGIRIAQEEIER-AVQLAKEVDQVVLCVGAANPNTVV
VVQSGTPVSM-PW-ASQVAGLLHAWYGGNETGNAIADALYGTINPSGKLTLSFPIQVEDNPANYRSER-GYETKRATLFPFGHGLSY
>A0A0D3EGP3_BRAOL
---GATSFPQVILTAASFNVSLFQAIGKV
VSTEARAMGAATFWSPNVNIFRDPRWGRGQETPGEDPTLVSKYAVAYVKGLQGTKVAACCKHYTAY-DVDNWKGVHRYTFNVNQQDMDDTFQPPFKSCVDGNVASVMCS
---YNQVNGKPTCADPDLLSGVIRGQWKLNGYIVSDCDSVEVIYASQHPEEAVAKSMLAGLDLN--CDHFT--GQHAMSAVKAGLVNETDVDTAI------IVLLKNSP
GSLPFPSAIKTLAVIGPNANTDTMIGNYHGVPCK--YTTPLQGLVETVWAEADIDSATSLAASADAVVLVMGTDLSIEREDHDRVDLFLPGKQQQLVTEVAKVAKPVVL
VIMSGGGLDVTFANDPKITSIMWVGFPGQAGGLAIADVIFGRHNPSGKLPMTWYPQSYNLPMN-MNMRDNYRYTGETVYAFGDGISY
>A0A1M6SRG1_9FIRM
-------------------MEEFISQLSNEDLAALVRGEGMCHPSVTPGTASAFGGVTDRLLSFGIPLACTSDGPSGIRMDGGYKTQVPIGTLLAATWNPELVEELYTL
EGKELLSNSIDMLLGPGLNIRRSPQNGRNFEYFSEDPLITGKFASAVVKGIMKGGSNATLKHFACNNQEKFRAKVDAV---VSERALREIYLKGFEIAVEGGANGVMTS
---YNPVNGHWAASNYDLTTTILREEWGFQGIVMTDWWAIMNDVVSGGDRKNTHFMVRAQNDLYMVVSNFGAEYRDLEEALKEGTLTRGELQRCAKNI----VLLQNEG
MVLPVKPG-ERVSIFGRIQKNYYRSGTGSGGSNVAYTTNLLDGMRSKKDIPISEEIAGAARKQSDKAIIVIGRT---AGEDKDNGSCRLTEEEQAMIKAVTGYFEETAV
VLNVSNIIDMSWLYKYPIKSVIYSWQGGMEGGNAIADVLAGEVTPSGKLTDTIAYSIQDYPSK------NYGGEDKNLYQEDIYVGY
>A0A1X6N6V9_9APHY
---YATSFPQPILMGAAFDDALINHVATI
VSTEARAFGIDF-WTPNINPFKDPRWGRGQETPGEDPFHLQSYVYNLITGLQGGRIVATCKHFAAYDLENWEGNVRYGFDAVSLQDLSEFYTRSFRTCADANVGSFMCS
---YNAVNGVPSCANSYLLQDILRDHWGEDQYITSDCDAIQNIYEPHYRAETVADALNAGTDLDCGEYYP----ENLGAAYDQGLFTESTLNRALI-----ITLLKND-
GTLPLSPSIKTIALIGPWANTTQMQGNYYGV--APYLISPLMAAEELGFTTSSFPAAFAAAEAADAIIYAGGIDITVEAEAMDRYTLDWPGVQPDFIDQLSLLGKPLIV
LQFGGGQIDDSALPNPGVNALVWGGYPGQSGGKAIMDIIVGNAAPAGRLPITQYPAMTDM-------SRPYMYTGTPIVEFGFGLHY
>A0A166DFG8_9AGAM
---LTDYSAFPAGINAAMSWDKDLILQRGQA
MGAEHRGKGVNVQLGPMMNLGRDAAAGRNWEGFGADPFLAGVASALTIQGIQSQGVIACAKHYIGNEQEHFRSGSGGGEVDIDDRTLHEVYAWPFAESIKAGVGSVMCA
---YNRINGTYACENSKIINGIAKEELDFKGFLLSDWAALESGAASALTDMNQPGFIGYGIGPQNEPNPSTANGEALAQMVANGTVPEWRVDDMVVRIMS-TVLLKNLN
GTLPLKKLPRNIAIFGSDAGGTLAMGWGSGTANFPYLIDPLSAIANWVHEDYNYNQVTSLATQADLCMVFANAD---SGEGYDRNNLTLWHSGDTLVTTASSHCPNTIV
VLHTVGPVILEKWESENVTAVLFAGLPGQESGNSIVDVLSGAVNPSARLPFTIAESAFDYSAVVYSNPNEYRFDANGIFEFGFGLSY
>A0A5C8M3W2_9BACL
TLDEKIGQMVQAE----RASLGTVSDIKTYFIGSILSGGGSVNTAAAWKTMVDNYQNQALSTRLGIPMIYGVDAVHGHN-NLKDATMFPHNVGLGAANDADLVRRIGSA
TAEEVRATGVHWTFAPVISAVQNIRWGRTYEGFSEDPQIVAKLGVAAVQGLQGNKIVADIKHYIGD--GLTDNGVNTGNITVPLNQAKDEYLAPYKKAIDAGARTVMVT
---YSGINGLKTHADTHLVTDVLKGQLGFTGFVVSDYNAIQQITVDDQYKKQIKASVNAGVDLFMLSDKGNTTINNLKSLVGTGEVPMARIDDAVTRNLR-LVLLKNSP

NVLPLKDGSQKIVVIGDKANIGYQLGGWTISWTVTKGTNILDGIKQVAGPVDWDQNGATATAGHDIAIAVIGEDPYAEGNG-DRTGTSLTTSIVANLKAAKAANPNVVI
ILVSGRPLVITND-LANWDAVVEAWLPGSE-GAGVADVVFGDNDFQGKLPYTWPRTFDEINA--K-------DKSNPLFPLGYGLTY
>R5HNG1_9FIRM
----------------------FIAQLTKEELATIVRGEGSPKVTPGTASTFGGVSDRLYE--YGIPAACCADGPSGIRMESGLKTQLPIGTLLACSFNIPMMEELYVM
EGKELVANEVDTLLGPGINIHRYPLNGRNFEYFSEDPYVTGCFAAAVTRGIKKGGSFATVKHFAAN-NQETARHT--VDSVVSERALREIYLKGFEIAVEGEASSIMTS
---YNPINGHWTSSNYDLNTTILRGEWGYEGIVMTDWWSVNDVVKGGKQDHHTLSSVRSQNDLYMVVNNNGAEGDDILEALDNGKLTIGELQRSAKNICR--VLLKNEG
HMFPLKKE-EMVSVFGRCQFETYRSGTGSGGANVPYAVNIYDGMRESGSFVITEEIAAAAAEKSEKAIFIIGRT---AGEDKDYGSYLLTPEELENLKVLTAHFEQVAV
LLNVSNIIDMSWLYKDHIRSVLYIWQGGMESGNAVADVLSGKVSPSGKLTDTIACSLADYPADFGDTVRNETCPEKVMYEFGFGLSY
>Q0UXS2_PHANO
--PGYCLNNAENGVG--GAEGNAYPAALHVGASWNRELAYARGMH
MGREFRRKGVNMALGPSIGLGRSPKGGRNWEAPSNDPYLTGMLIHDTTAGMQK-YVIANIKHIVGNEQEASRNHNASISSNIDDKTMHELYLWPFMDALRAGGASVMCS
---YNRVNNSDACQNSKVLNGLLKQELGFQGFVVSDWF---------MQQSGVASAL-AGLDMVMPIAPYW-ADGNLTQMVNNGSVSMTRLNDMVTRIL---VLVKNVN
KALPL-RKPKFISIFGYDAAGTLFTGGGSGAVVG-SIDAPLDAFKRQAYDWDIQTPNPTVNAGSEACLVFINAL---GSESWDRRNLSDA-YSDGIITSVASQCNNTMV
VIHSVGVRLVDEWDHPNITAVILGHLPGQDSGRALVELMYGRQSFSGRLPYTVAKQEADYGHDPVQPSD-----KTPYFTEGVYIDY
>A0A3N4HIL4_ASCIM
--RFAPLTVFPAALTTAATFNKDLLFLRGQA
LGREAREKGVNVLLGPCIGLGLFPEGGRNWESFGADPYLQGVGGRLTVRGIQGEGVIANAKHYVGNEQERRDEGLDGPISSLGSADLRETWAWPFQEVVEEGVGSIMCA
---YNSVNGSQSCSNSYLLNEVLKGEMGFQGFVVSDWL---------AQDTEAEVSANAGLDMTMPGEKHSFTGAELTAAVVKGEVSQARLNDMVLRIV--TVLLRNEN
LGLPLKKKVLNLALFGSAAGGALGQGWGSGSVDYTRFITPLESIQSRLYADLLRQKAVAMQEVNGTCLVFVTSD---SGEGYDRNDLELWHGGAEMVLTVATHCRDTVV
VIHAVGAVNLEPFGHPNVTAILHAHLPGQEHGSSLTPLLFGDEEPSGRLPYTIFKKEGDYPREKEANG-RSEQEK--TYPFGFGLDY
>A0A1G4RFG7_9FIRM
----------------KASMDDFIAQISDEDLCLIIRGEGMSCPKVTTGTAGGFAGVDPVLNAMGVPACCCSDGPSGMRIDSGKKFSIPNGTCIASTFNLKAVESLFGW
FGIEMISNRVDTILGPGMNIHRHPLNGRNFEYFSEDPLLSGKMASAQIKALEQNGVTATIKHFCVN--NRETRRRDMDSVV-SERALREIYLRGFEIAVEGGARSIMTS
---YNKINGTYAASHYELNTLLLREQWGYKGITMTDWWIVGTANMHHELRDHAAMA-RAQNDLYMVCSSVDPADSDCYDELMAGNITRAELQRNARNILN--VLLENRD
NVLPLADGA-RVALFGRMQTHYYKSGTGSGGMNVEHVVDIREGLKDSGRVPVTEELVKSVSMRNDTAVIVIART---AGEDRDNGSLSLQDGEEELIRLVTEAFAKTVV
LLNTGNIIDMSFVKKYRPQAVMYIWQCGMIGGTAAAKLLTGEENPSGLLTDTIARTFDDYPSPYFGDQDQTTNRAAVMYPFGYGLSY
>A0A369K5A1_HYPMA
-------------------------GLANTVVAQMTLDEKIVRGTGQLNSMRRCVGDTTAVPRLGIPSICFNDGPAGLR-LTKGVTGFPSGINAAATFSRRLMRARGKA
LGEEFRGKGVHVFLGPALDIMRNPKAGRGWESFGPDPYLNGEGAFETVTGVQSVGVMACAKHLIANNQEHWRYGLSA---NVDDRTLHEIYWYPFLRSIEAGVASVMCS
---YNRLNGTSSCHHAGLLPNGLLQKDGFQGFVVSDWG-----A----THDSASDNANAGLDMEQPGDFIV-IFIGLKSNVNSGMVSVTRLNEMVSRILS--VLLKNNR
RGLPLASRIKSIAVIGQDAKGTMSVGWGSGSNSLEFIVPPIDAITSFAGSSNDLDAGVKAARGKDVALVFVNAMSGEVGNQGDRNDLDLWWKGGSLVERVAAVCNNTIV
VVHSVGPIHMGWSIHPNITAIIYAGAPGEQTGPSIVDILYGKYNPSGRLPFSIADKEDDYGTIVYNSLDVY-LDSKSIFEFGFGLSY
>A0A1Y2GSV0_9FUNG
----LAGQMTQIQIGMELDVAKAQYWIGEWGVGSFLDTPTISYSPKRFSKIVDDIQKVALSTGKGIPVIYGLDSVHGANY-VDGAVIFPQQIGLAATFNTTLAYEAGRI
TAKDTRAAGIPWVFAPILDIAVHKLWPRVYETFGEDPHVSSTMGAAIIRGLQGNRVAGCMKHFIGY--SASRNGQDKASAWIPDNLLMDYFVPPFRAAVNAGVATAMET
---YIDVNGQPVVGSHFYLTELLRNQLKFEGMLVTDWQELDRLYTEHRLKEAALQCLKTSMNMIMVPESKS-FSENAGLLVQEGKLGRERLVSSVAKVLQLITVLRNNK
GALPLKNQTKKIAITGPAANLRALSGGWSIKWT----DTILSGLIKEFGESTASAPLDDAIQGADSVILCLGEGPYAEIVG-NINDLDLPNGQLDLVRKVSGTSTKLIL
ILVEGRPRGLQDV-VEKVDAIVLAYLPGPWGGHPIAEILSGAVNPSGRIPMTYPNGSSDMTTNYYRMGD----PYKPLFPFSAGISY
>Q1YR72_9GAMM
TTAEKVGQMVQAE-----ISNVNAAQVRDFNLGSVLNGGGSIADWVALADSFYEASTDISDGGVGIPAIWGTDAVHGHNNVIGA-TIFPHNIGLGAMNNAPLMRQIGET
TALEVAVTGIDWVFAPTLAVVRNDSWGRTYESYSEDPEIVRAYAGEVVSGLQGDHVIATAKHFIGD--GGTQNGIDQGNTVVTEVELRDIHAQGYLSALAAGAQTVMAS
---YNSWNGSKLHGDEYLLTEVLKQKMGFDGFVIGDWN--GHGQVPGCSDGQCAQAIMAGVDMMMVPADWQAFIQNTIAQVQNGTIPMSRIDDAVTRILRVLVLLKNSD
SILPLAANS-NVLVAGSGANIGMQSGGWTLSWDFPGATSIYSGIESLVNARLSANGSFSSSNRPDVAIVVFGESPYAEGVG-DLNNIEYQKSDLALLESLRGQNIPVVS
IFLTGRPLWVNKE-LNASNAFVAAWLPGSE-GAGVAEVIFKTADFKGKLSFSWPKRAEQTVI---NRND---SNYDPLFAYGFGLTY
>A0A1U9NNJ8_9BACT
----------------EIAQKDIDKLFEGYSYGTLQRFGGPIKEQAII--NRDVQKYALEKTRLGIPVLPMHETLHGIL--ATGATIYPQTIAQGATWNPELIKEMSSA
IAVEGAAAGISQSLSPMLTLARDHRWGRVEECFGECPKLVAEMAIAYIKGMQGEKMACMLKVMAGY--EIPSGGINIAATSLGEREFRSLYLYPYEQAVRAYPYSVMPS
---YNVVDGLPAHANHWLLTKVLRDEWGFEGYTYSDWGGVPFNHSLHKAKESAIVCLKAGNDLEAPGPSCY---KYLVELVKDGRIDEKYVDQAAGRMLKVIILLKNEG
NMLPLKDKMKSIALIGPNAD-QVQFGDYSPTKSNHMGVTVLEGVKGFLAGRSGFDEAVETAEQSDVAVVVVGDTSMIAGEGYDRTTLTIPGVQEDLVKAVVATGKPVVV
VLVHGRPFAM-PWLKDNAQVILDVFYPGEQGGNAVADVLFGKVNPSGKLPVTLPRSVGHLPQ-TYDYLRGYVSSPEPLWPFGFGLSY
>R9L210_9FIRM
----KGYQLVDVL-DQKVSLDEFVAQMTDDDLIHIFRGEGMCSPRVTAGTAAAFGGVTWHLQDLGIPAACCSDGPSGIRMDCGTKFSLPNGTALGCTFNTELVEALFEL
LGQELRMNKIDSLLGPGINIHRNPLNGRNFEYLSEDPYLTGKIAAAQIRAMESSQIAGTIKHFCAN---NQEKGRRTSDAVVSERALREIYLKGFEMVVDKNARSVMTT
---YGAVNGIWTAGNYELCTQILREEWGFDGIVMSDWWA-EANYEGAMPARTVKAPMAAQNDLYMCVCDAAQNNDDMEQQLASGYITRGELQRNAKNILR--VLLENEN
NALPLKNG-EKIAVYGRSAFHYYKSGLGSGGLNTAYTVGILEALKDSSDVMPVTDRMREIAKEADASLVFIGRT---AGEDQDNGSYLLTEQESRMLEEICKASKRAIV
ILNVGNIIDMSWVADYHPQAVLCVWQGGQEGGNGVLDVLLGKVNACGKLTDTIAKSITDYPSNFGNESKNE---TFAKYPFGYGLSY
>I0X956_9SPIR
------------------------AQLDDKELAAMVRGEGMMSQKVTVGIAAAYGGITQSLRNFGIPAAGCSDGPSGIRLDTGKENLMPIGTLLACTWNPNIIEDLYTF
EGKELVQYEIDSLLGPGANIHRSPLNGRNFEYYSEDPLLSGMIAKAALRGLNIGGASGTIKHFAAN---NQETWRRTSNSIVSERALREIYLKPFEIAVSGEVKSLMTS
---YNAINGHWTASNYDLVTTILRREWKYQGLVMTDWWTMNDCVKGGSSIKNTASMIRSGNDVYMVVDNDGAEEDNTLEALKNGSLTRAELHAAAKNILN--VLLKNEE
CLLPFTSQ-DNVAVFGRCQKDWYRSGTGSGGSHVSYTTTLIDSLLELSLSPLSKELLEETGANNSKAVYVIGRT---AGEDKDNGSWYLNDDEKAALKAICGTFEDVCV
VLNVSNIIDMSWIFKGHIKAVLIAWQGGQEGGRAAARVLCGLANPSGKLSDTIAMSIDDYPSNHFGSKDISTFA---PFPFGFGLSY
>K0K350_SACES
TLAEKVGHMTQPE-----IAAITPDEVRQYGIGSVLNGGGAVRDWL---SLADAYWDASKATRAKIPVLWGIDAVHGNNNVYGA-TVFPHNIGLGAAHDPCLVRDVSEA
TAEQIRATGQDWAFAPTLAVVRDDRWGRTYEGFSEDPRITRAYGYEAVRGLQGDDVLATAKHFIGD--GGTLGGKDQGVTPSSTAEMINLHGQGYYGALAAGAQTVMVS
---FNSWTNAKLHGSKLAVNDILKGKIGFDGLVVSDWNGIGQ--VPGCTNAGCPQAINAGIDVVMVPNDWKAFIANTVAQVESGQIPLSRIDDAVTRILRV-VLVKNDN
RVLPLKPRS-KVLVVGKSADLQNQTGGWTLSWDFPNGTTILGGLREALGANVVFSETDVDPAGFDAVIAVIGETPYAEGTGDSLEAAKLYPRDLAVLDKVRGRGAPVVT
VYVSGRPLHVNKE-LNRSDAFVASWLPGTE-GGGVADLLVRGRGFTGTLSYSWPRSACQTPLPG-------QAGYDPLFKPGYGL--
>A0A1G5C4C7_9FIRM
-------------------MDEFIAQLSDEDLCCMMRGEGSPKVTAGIAGAFGGVTDKLA--AFGIPVAGCSDGPSGIRMDCGTHFSLPNGTCLACTFNEELNEELFNW
EGLDLRRYRIDALLGPGMNIHRNPMNGRNFEYFSEDPFLTGKLVAAQLRGLHKYDVTGVIKHFAGN-TQEFKRHNVNNIV--SERALREIYLKGFEIAVEGNARAIMST
---YGPVNGIWTASSYDLLTTILRGEWGFDGIVMTDWWAMGNNFADAGDYKNVSSQVRAQNDLNMVNGNAETNGDDLEKALQDGRLSRAELARCAENILR-IVLLENEG
NVLPLKK-NEKIAVFGRSQMNYYKSGTGSGGMNVDYVVGIFEALENSR-YPLTEDFVEEAAKESGTAIFIIGRT---AGEDQDNGSFLLTDEEKDALSKVCRAFDKTVV
LLNVGNIIDMKWVKEIKPSSVLYVWQGGQEGGNAVFDVLSGRVNPSGKLPDTIAANISDYSSNFGSDKYQETDKESVLYPFGYGLSY
>C4L039_EXISA
------------------------DQLTDEDLAAIVRGQGSPRVTPGTAAAFGGVSDR--LNELGIPAACCADGPSGIRMDIGTKFALPNGTLLASTFNVDLVEDLFEM
TGLEMRKNRVDTLLGPGMNIHRNPLNGRNFEYFSEDPHVTGKMAVAQLNGMHRVGVTGTLKHFSAN---NQEAHRHDIDSVVSERALREIYLKGFEMAVEGRASSIMTT
---YGAVNGIWTAGLYDQNTRILRDEWGFDGIVMTDWWKVNFREDESANRQNTAAMVRSQNDLYMVVDRPDLNEDNTMASLEEGVVTRGELLRSARNI---VVLVKNEQ
KTLPLQPET-KLAVFGRSQFHYYKSGTGSGGMNVGHVTTPLEALRERPDIDVTDELVEQAAQKSDVALIMIGRT---AGEDRDNGSYLLTDIEHAMIERVTKRFDKTVV
VLNVGNIIDMKWA--NEPSAILYAWQGGMEGGKGLVDVLVGDVSPSGKLTDTIARSIEDYPSNFGHAD-KETAKDDVLYPFGFGLSY
>A0A1K1LXU2_RUMFL
--------LADVKN-GKYTMEEFVAQLSDHDLSCIIRGEGSPRVTAGTASAFGGVSDALVK--FGVPAGCCSDGPSGMRLDCGTKFSLPNGTLIASTFNRKLVEELFEF
MGTEMIANKVDCLLGPGMNIHRHPLNGRNFEYFSEDPFLTGHMASAELNGLHKIGVTGTIKHFCAN-NQETNRHFLDSIVS--ERALREIYLKSFEIAVVGKAKTIMTT
---YGSINGLWTAGSFDLNTTILRDEWGFKGFTMTDWWANVNFRGHEPKRNYYVPMARAQNDVYMVCSDSSCIDEDIEAALKDGTLTRAELQRNAMNIL--IVMLKNDN

NALPLDT-SKTVSVFGRIQLHYYKSGTGSGGMNVSKVTGIVDGLVDAGVKPLDESVVEEAASLSDTAIVIIGRT---AGEEQDAGSYLLTSTELDMLKKVRKHFKKVVV
LLNVGGLIDLEQILECSPDSLLYVWQGGMTGGTGTAAVLTGKVSPSGKLPDTIAYKISDYPSKYFGDKNKETAKDKVLYPFGFGLSY
>A0A316Z1G0_9BASI
--ISPNKRVGFPGLCLEDSPLGVR--FADGTVWPAGVTTAATFSSKLAYERGKA
MGEEFRAKGVNIALGPGMNMARTPAGGRNWEMASADPYLTGESAFHTTKGMQDAGVQACAKHYIAN---EQEINRNTYSSNIDGRTEREIYLHPFMRSIQAGVSSVMCS
---YNLNNNSWSCQNSELLNNRLKTELGFNGFVMSDWG----------AQHSGVASANAGLDMTMPGQVECCSGQNLTMAVQNKSVEASRLDDMGTRILA-TVVLKNTH
GALPLGEKLKKLAVVGSDAGGTIGIGWGSGTADYSYFITPYEALQARARKLDQAKKISDDQIGVGAALVFVYAD---SGENYDRNNLTLWGNGVELIKSVASVQKNTIV
VINAPGQVDLEEFDNPNVTAVVHAHFPGAEAGNAITDILYGDVNPSGRLPYTIAKKRSDYSAQYFTEPDTYRFDSAGIYAFGHGLSY
>A0A0S7DD26_9EURO
---RIGYPGMCLADASNGLR--GTDFNAYPAGIHAGASWNRSLVYHRGLY
MGEEFKAKGVNVINGPVIGLGRTARGGRNWEGFSADPYLAGVLVAETIQGLQK-SVIASVKHFIAYE-QETARGPEGNNSNLDDKTMHELYLWPFANAVHAGVGSVMCS
---YNRVNNSYACQNSKILNGLLKTELGFQGFVVSDWN----------AQLTGISSANAGLDMAMPDSPYW--QGNLSLAVANGTMSQERLDDMATRILA--VLVKNIN
HALPLK--PRSISVFGYDAGGTLIGGGGSGASVPSYISTPFAALVEQATVWDLESFSPTVPVSSDACLVFVNEF---ATESRDRPGLADP-QSDRLIMSVASQCPNTIV
VIHNAGVRIVDAWENPNITALIFSHLPGQDSGKAVTEILYGRQSPSGRLPYTVARKPSDYPTD------YLAHNITPRFEFGYGLTY
>A0A084GEZ5_PSEDA
--FASATSFANPILLSAAFDDSLVHEIATV
ISTETRAFGLD-YWTPNINPYRDPRWGRGMETPGEDPRRIKGYVKALLSGLEGDKIIATCKHYAGNDIDRWEDVLRFNFSAISLQDLVEYYLPPFQQCADSRVGSIMCA
---YNAVNGTPACANTYLMQTVLRDHWGWNQYITSDCNAVGNFYADHHAAEAAAKAYAAGTDTVCEVNMAT----DVIGAWNQSLLTEETIDRALNRL---IVLLKNDD
AVLPLYDANTSVAVIGHWAEPVQLLGGYAGTA--PYYITPAAIHNSTHYDTWSEKALEAANNADIVFYFGGLRMTIEREDRDRTSIGWPAAQLSLIQKLCALGKPCIV
-IQMGDQIDDAPLENKNVSAILWAGYPGQAGGAAVFDILYGKSAPAGRLPVTQYPSSYTEPMD---MTRPYKWYSDAVLPFGYGLHY
>A0A1V8V7T9_9PEZI
--FSYATSFPTPILLGAAFDDPLIYQIAST
VGKEARAFGNYMQWTPNINTFLDPRWGRGLEVPTEDSFHAQSYVSQLIPGLQGGQIIATCKHYAVY---DVEELRNSENYNPTQQDLGEYYLTPFKTCVDVDVGSVMCS
---YNAVDGVPACASEYLLQDVLRDAYGFDNYVTSDCGAVDDIYNQHDFAAAAAVAINAGTDTNCGSTYL-----QLNTSVARGFTTNATLDRALTRLYT-MTLLKNN-
GVLPL-KKYNKVAMIGPWANTGQMQGNYQGI--APYLISPLQAAQAQWGATTGFAAALSAAKSADLVVFLGGIDTSIESEGHDRTNIDWPGNQLDLVAQLSQLRKPLVV
AQFGGGQVDDTALQNRNVHALIWGGYPGQAGGAALIDVLIGKQAPAGRLAITQYAGSYINQVKF-DPNRPYKLTTKAVLPFGYGLHY
>A0A5J5B1L3_9ASTE
--GATSFPQVILTAASFNVSLFKAIGQV
VSTEARAMGLATFWSPNINIFRDPRWGRGQETPGEDPLLASKYGSAYVKGLQQRKVAACCKHYTAY-DVDNWKGVDRYHFNVTQQDMEDTFQPPFKSCVDGNAASIMCS
---YNQVNGTPTCADPNLLAGVIRGKWKLNGYIVSDCDSLDVFYNSQHPEEAAAKAILAGLDLDCGSFL----AQHTEAAVTGGLVTESSIDRAI--------------
GSLPLPTEIKTLAVIGPNANTETMIGNYEGTPCK--YTTPLQGLTTSVSTTAQIDDAKNIAASADATVLVMGADLSIEAESLDRVDLNLPGQQQLLITEVANASKPVIL
VIMSGGGMDIQFADNDNITSILWVGYPGEAGGAALADVIFGYYNPSGRLPMTWYPQSYDNPMTNMNMRDPYRYSGETVYEFGDGLSY
>Q6CEN3_YARLI
--L---HGVGGDSHFTTFLPAGITTASTFNKGLMYSRGAI
IGKEARKKGMDIVLGPLIDPTGRSAAGRNWEGFGPDPYMAGVVATESVTGIQDQGVVATVMHYVGYSQEHFQAHGYNNLTSIDDRTMNEVYIWPFANAVKANAGAIMCA
---PQKLNNTQGCKNSYMMNYKLKRELGFQGFVLSAGM----------SQNEPPAAL-AGMDMSMPRLKASENREMMLRNYDKDGFPQSRLDDMATRVLT-IVMLKNDD
SALPI-EGMRTIGVLGAAANGAIFQGWGDGGVNPPFVVTPYEAVNARAARSWDLNQAEAVASSTDANIIFVAAN---SGEGNDRKNFTLWHNGDELIKKAVEVNDHNIV
VVTAVGPVDMEKWEHPHVKAVLFTGPGGEEAGAALAHVLFGDFNPSGKLPFTIARNVNHYPIETEVPRDGYKFDQNLIFEFGFGMS-
>A6WB17_KINRD
TLEEKVAQLAAPFG-SAVDVHTPPATGWGCVVAGLCT--LGLPPRETAERANELQRKHVEQTRLGIPVLLAEEALLGLK--VRDATTFPDAIAQAATWDPQLIEQVGRT
IGVQMTRLGVRQALSPLADVARDPRWGRVEETYGEEPQLVGSMAAAFVRGLQGAPLIATLKHFLGY--SASAGGRNTEPAPLGPREVREVHALPFEMAIEGGAKGVMPS
---YNDIDGEPVTGSRAYLNDLLRGELGFDGLVISDLAAVGQLHSKHHAPEALARAVSAGVDLDLDNRVSS---QALQEAVRSGLLPSADLDRAVSTILR----LANGA
PLLPLDPGVGTIAVIGPNADRPLQLGHYSYHVESVPVVTFLEGIRARAEGRSGFAAAVAAATRADVAVLVVGDQAGIVGEGLDSSTCELPPVQQALVEAVVATGTPTAV
VLSHGRPYTLKWL-AQSVPAVVTCFFGGEEAGNALASVLFGDVNPAGRLPIAFLESVGSAPLPYWRTLQAYVGPARAVFGFGHGLSY
>A0A1U7LRJ0_NEOID
---RFTDYSVFPSGVQTASTWDKELIYNRGLA
LGQEFRGKGVNVALGPVAGLGRTPAAGRNWEGFSPDPYLTGVAMYQTILGTQSTGVIACAKHLIAHYRQAAESHRHTTESNVDDRTLHELYLWPFADAVRAGVGSFMTS
---YNQINNSYASQNSKIINGLLKDELGFQGFVMTDWA---------AQHSGVESSL-SGLDMTMPGTGIPSDGSNLTLAVLNGSLPEWRLDDQVTRIF--IVLLKNIN
STLPL-KSPKQIAIIGSNAGGTLALGWGSGTADFSYFITPLEAIQSRAISDYNYAQINATARRATTCLAFINSN---SGEHYDRNNITAWHDGDQLVSFTAANCNNTIV
VVNSVGPIILEPWNHPNVTALVWAGLPGEDSGSSLVQVLFGDVNPSGKLPYTIAKSASDYGTM-YEATDVYRFDQYKIFEFGFGLSY
>F4QPK5_9CAUL
TLEEKVGQTVQAD-----INFITPEELKTYPLGSILAGGNTPDAWL---QLADDYWRASLESKVKIPVLFGIDAVHGHSNLVGA-VIFPHNVGLGAAHNPELIRKIGEA
TAKEMAVAGVDWTFAPTVAVARDKRWGRAYESYSENPADVAAYSGYMVEGLQGANIMSTAKHYLGD--GGTTGGKDQGDAEMSESDLARIHNAGYPPAIEAGTLSVMIS
---FSSWNGQKLAGSKKLITGALKQRMGFDGFAITDWN--AHRQIPGCEQDDCPQAINAGVDMYMAPDTWKAVYTHLLADVKSGEVPMARLDDAVRRILR-LVLIKNNN
RILPLRGS-DHILVTGSGAHVGKQSGGWTITWDFPNAQSIWEGIAETTERTASLSVDGVYKEKPDVAVVVIGEDPYAEFQG-DRPNLDYQATDLALIKKLKDAGIPVVT
VFLSGRPMWTNPE-INASDAFVAAWLPGTE-GGVADVIVADADFKGKLTFSWPKAANQQPLNVGT------MDYDPQFAYGYGLTY
>H3NUY6_9GAMM
TLEQKVGQMIQPE-----IAYITLEEISQYGIGSVLNGGGTPEAWLQFARELREASLKRSNSSLGIPLIWGTDAVHGHNN-LRGATIFPHNIGLGAINDPDLIGEIATA
TAREVAATGIDWTFAPTLAQAKDYRWGRTYESYSDDPAIVEAYGRVMVERIEAEGIAATAKHFIGDGGTQAGIDQGNTLVS-SSAQLMAEHGSGYIGAFEADVDTVMAT
---FNSINGEKVHGSKSLLTSLLRDELNFNGMVISDWNGIGQV--SGCSNASCAQAINAGIDMIMVPTEWLAFRNNLIQQVRRGDVDESRIDQAVTRIID--VLLKNNN
ATLPLNPSQ-RILLVGAAADIPLQAGGWSVTWDFPGASTIRDAFTEVVESTLEYSPAGNYSSVPDAVVVVLSEQPYAEGNG-DLQNLDWSSSVLQQVQTLRDAGVPITT
LLMSGRPMFVNPE-LNRSDAFVASWLPGTE-ASGIADVLFTDSDMTGKLSFSWPGGAI-NPSNAS------SPVAANLFERGYGLSY
>A0A5C3F0Z8_9BASI
--DRFGIPELCFQDGPAGVR--TSDFTVFPPGLTTAATWNRDLIYQRAAA
LAEEVKGKGINVHLGPATGLGRGPWQGRNWEGYGPDPYLQGEAGYHTIKGTQSNGVIATAKHFLAYEQETANNTRNTYSANLDDRTMHELYLWPFMNAVRAGSGAVMCV
---YNRINGTQGCENSKVLNTILKDELDFQGFVVTDWSAAFNTSDTYNGSDVVMPGGNTGGYRNLVGG------KNLAKALQDGTVKQERVDDGIVRLLT---------
--LPLAKKA-RVAVFGSDAGGTNAIGWGSGAGYFPYLIDPLAGISAKAREGQNRKLVQSTASIADASLVFVQAR---SGEDSDRSTLALEANGDEMIKAVAAASNNTIV
VVHSVGQIYMDEWDHPNITALVFAHLPGQESGSTIAEMLYGETNPSGRMPFSILAKRDHYPKPV----------DDPQFEEGLYIDY
>A0A176WBW1_MARPO
--GRVKSATSFPQPILTAASFNKELFNKIGQV
ISTEARAMHNETFWAPNINIFRDPRWGRGQETPGEDPYLTSIYAEYFVRGMQEDKTSACCKHFTAY---DIDQWYDVDRAKVTQQDLLDTYNPPFQSCIDGKASSLMCS
---YNRVNGVPTCADYNLLTKLARGTWGFDGYIVSDCDAVQVMYANSRPEEAVAYALKAGMDLNCGDTAS----NFTVEAVHSGLLNTSDIDKAL------IVLLKNDQ
NTLPLADKIRSLAVLGPNANVNTMLGNYAGPPCV--YVTPYLGLAQYVPDSDFIRGAAKVATWTDAVVVVVGLSQDQEREAFDRTSLRLPGQQEELITTVSRVAKPVIL
VLMTGGPVDIGFIDDPKIQSILWVGYPGQAGGQALAQVIFGDRNPGGKLPMSWYPESYTEPMDMHMRPDEYRYSGDVIYRFGEGMSY
>A0A6G9QL90_9GAMM
TLEQKVAQMIQPE-----IRDITVEDMRQYGFGSYLNGGGTPADWINLAEAMYQASVDDSLDGSRIPTMWGTDAVHGHNNVIGA-TLFPHNIGLGAANNPKLIEQIAAI
TAKEVMVTGIDWVFAPTVAVVRDDRWGRTYEGYSEDPRIVRDYAFAIVEGLQGAHVLSTVKHFLGD--GGTEKGIDQGDNLASEQDLYAIHAQGYVGGLNAGAQSVMAS
---FNSWHGDKIHGNKYLLTDVLKGRFAFDGFVVGDWN--GHGQVAGCSNESCPQAANAGLDIFMVPTAAWKPYENTIAQVKSGEISQARIDDAVSRILRVLVLLKNNQ
ALLPLSPKM-NVLVAGDAAD---NIGKQSGGWDFPGASSIYQGIASAVDQQLSVNGQFDANNKPDVAIVVFGEEPYAEGNG-DIDNLEYQKRDLALLKSLQAQGIKVVS
VFISGRPMWVNPE-MNASDAFVAAWLPGSE-GQGVAEVLFTDADFVGKLSFSWPSTPQQSAVNVND------EDYQPLLPYGFGLKY
>A0A316WGQ4_9BASI
--PRLGIPSLCFSDGPTGVRQALNV-SQFPAEVTVGATWDLDLVGKRATA
MAEEFRDIGINVMFAPVTGLGRSPQGGRNWEGFATDEYLTGRASYVSVKDAQAAGIVAGAKHFIFYEQETSRNRTILPVLQQPIDSVHELYMVPFAEAIRAGAGQIMCS
---YNKINGTHACESSKSLAGLLKTELNFQGHVVSDYG--GA----WSDSTVGLDVLMPGDGLYGVQPNFFGSGSKLIENVENGKLTETRLDDMVIRLLT--TLLKNTV

TGLPLLNKIKKIAVSGQDAVGVVTTGTGSGSTTPPYIIDPLAAIRSYIAKPFVFLGAVWEAKNADVALVFVSAT---AGEASDRADLKLDHNGEDLIKAVAAANNNTIV
IIHGPGPVIVEDWDLPNIRAVLYAYYPGQEAGSSLTPVLFGDESPSGKLPFVMAKKTSDWPANTLVKTNPLDKNIAPRFPFGFGLSY
>A0A2B8AYN4_9ACTN
TLEEKLAQLYGIWPGSASSASTDTDTLLTHGLGQLTRTFGPLGGAEGAEALARTQERIVASNRFGIPALAHEECLTGFTTW-G-ATIFPTPLAWGASFDPALVGRAAEL
IGASMRAVGIHQALSPVLDVVRDPRWGRTEESISEDPYLVATVGTAYVRGLEAGGVIATLKHFAGY--AASRGGRNHGPVSAGPRELADIVLPPFELALDGGARSVMAA
---YNEIDGVPSTANVWLLTTLLREEWGFTGTVVADYFGIGFLEMAHKRAESAGLALTAGVDVELPGVDCY--GTPLRDAIRDGDVPESLVDRALTRVLR-VVLLSDDA
GVLPLTSPGTRIAVVGPMADPAAMLGCYTFPRD----LTLLDSLRSAFPGADALTEALALTAGADVCVAVVGDRSGLSGEGCDSPDLELPGGQGALLDALLSTGTPVVL
VVLSGRPYALGRYGAPSAAAVVQAFFPGEEGGPAVAGVLSGQVNPSGRLPVSVPYGSGGQPWTYLSPPYESSLDPTPLYSFGHGLSY
>Q5H0B1_XANOR
-VEEKVAQTIQ-----GDIASMTPDDVRKYRIGSVLAGGNSPADWLKLADAFYEASMDTSKGGNAIPIIFGIDAVHGQSNIVGA-TLFPHNIGLGATRNPDLIKKIGEI
TAAETRVTGMEWTFAPTVAVPQDDRWGRTYEGYSESPDVVASFAGKMVEGVQGTHVISSVKHFVGD--GGTTDGKDQGDTKVSEATMRDIHAAGYPPAIAAGAQSVMAS
---FNSFNGEKMHGNKVMLTDVLKGRMNFGGFVVGDWN--GHGQVKGCTNQNCPASFIAGVDMAMAADSWKGIYETELAAVKSGQISAERLDDAVRRILRVLVLLKNQA
GILPLDPT-KRVLVLGDGAN---DMGKQSGGWDYPNGTTIWEGLDKQITASAELAVDGAYKTRPDVAVVVFGENPYAEFQG-DIATLLYKESELALIKKLKAEGIPVVA
VFLSGRPLWMNQY-INASDAFVAAWLPGSE-GEGIADVLLRKADFKGKLSFSWPKTAVQFANNVGQ------KDYDPQFKFGFGLTY
>A0A445LR21_GLYSO
-LEEKIGQMTQIE-----RSVATPDVMKKYFIGSVLSGGGTKASAETWQQMVNQLQKAALSTCHGIPMIYGVTKVYGYA-RIERVIMKSHLITLGMIWDPVLIKKIGEA
TALEVRATGIPYVFAPCIAVCRDPRWGRCYESYSEDPKIVKTMT-EIIPGLQGDKVAACVKHYLGD--GGTNKGINENNTLISYNGLLSIHMPAYYDSIIKGVSTVMVS
---YSSWNGMKMHANRKLITGYLKNKLHFKGLVISDWQGIDRITSHANYSYSVQASVSAGIDMIMVPYNYTEFIDELTHQVKNNIISMSRIDDAVARILRVLVLLKNGK
PLLPLPKKSAKILVAGSHANLGYQCGGWTITWDLTSSTTILDAVKQTVDPNENPDRNFVKSFKFDYALVVVGEH--TYAETFDSLNLTIADPGPSTITNVCGAI-RCIV
VLVTGRPVVIKPY-LSKIDALVAAWLPGTE-GQGVADVLYGDYEFTGKLARTWFKTVDQLPMNIGD------KHYDPLYSFGFGLT-
>V4GUY9_9EURY
TLEEKVGQMTQMAV-SSFDPEEVGDLFTEHHVGSILSGGASFEASEVAAGVNRLQEWAMENTRLGVPFVYGVDAVHGND-LVYDAPIFPHNLGVGATWDPSTAREMAAL
TGESVRAMGAHWTFSPANDIQRDPRWGRFYEGFTESVRLAERMGRAKVEGYERAAVGACQKHFAGY--SQPLNGNDRTAAQLPARYLRQFHLPPHEAGIDAGAETVMVN
---SSSVNGVPAHASKWLLTDLLRGEWGYEGMVVSDWKDFKRMVTIHNLKEATMLGVNAGVDMYMNPDDVGEFTSILVELVEEGKVSRDRIDQAVARVLR-MTLLQNAD
DTLPLDPDLGSVLVAGPRIDPLMQMGGWTLGWRV-PATTVVEGVENAVSSFDNPGEVETAADEADAAVVVVGEGPYSEGPG-DANHLHLHPAQQELVDTVAGTDTPLIG
VVIAGRPRGTEVF-D-RFDASVMAYQPGSAGGDAIADVLFGEVNPGGHLPFRWPVTVGQVPN-VHNALH--PVDDPPRFEFGHGMSY
>A0A2P6TNG4_CHLSO
---GKYGAATIYPQNMVMGATFNDALIERMASE
ISDEMRALGHSNCFSPHITLARDARWGRMAEVYGEDPLLLSRMAAAYVRGLQGPTVGATCKHFLGH---ILEGNWNGTTIRNLDARLEDDSLLPAFQACAAQAAAVMCS
---YNAWRGSPSCASKELLIDLLRGQMGFRGFVVTDKFAVDYSAAKPPLRKATALAIQAGTDVLMKNTSQ----------LHAEDLSAAELDAAVRRVLR-TVLLKNER
GMLPLPEAVKKLAVLGPFADADHILGTYVGAASG-NITTPLAALHAVLPGAKNAKADAKRCEAADVCVLFLGSRLSREGEGRDRKTLRLQPNQRKLWEAVAKATKPLIV
VLFHGGPLDVSDMESPRVAAILSAGMPGQHGAAAIADIIVGATAPSGRVPVSWHRESYMN-EDATDQRRAYRRGQPPLFPFGYGLSY
>A0A1I1CDP7_9BACL
-LADKVSQMCQCMASNAVASDPPEKLVAEGRAGSVLG-------AFDLGRVFELQRIAVERSPHGIPLLFNNDIIHGAQ------TIFPVPLAWACSWDMDAIREACAV
AGREAAASGIVYNHGPMVDISRDPRWGRVVEGAGEDPYLGALIAKAQVEGFQGGTIVACLKHFVGY--GAAEGGRDYNTVDMSEATLRNVYLPPFQAGIESGAGSVMNA
---FNIYQGVPVAASVPLLKELLRDELGFEGVLISDYGAVDEIVQHGHAKEAAKKAVDATMDIEMVTRAY----DHLPQLIAEGKLSEAQLDDAVRRILTLIVLLKND-
GALPLASG-AKLALIGPFADSKDLLGPWQFSRYVHETVTLYEGLVAAGYSDGGIEAAVDRAVQADIVLLALGEDSGMSGEAASRMRIALPDAQLRLAEAVAATGKPVVL
VLTNGRPLDLTWF-DAHMNAIVETWFLGSQAGRAIADVLTGACNPSGRLTMSFPLHEGQVPV-YYNHFRTYLGPNEPLYPFGYGLSY
>A0A1Q2M357_9GAMM
TLEEKVGQMMQAE-----IKFITPEEVKQYHIGSILNGGGTPDDWRQLADDYFAASMDTSDGGVAIPIIWGSDAVHGHNNVIGA-TLFPHNIGLGAARDPELIRRIGEA
TAREVAVTGVDWTFAPTIAVVRDDRWGRTFESYSEDPEIVAAYAREMIKGIQGDHLVSAAKHFVGD--GGTTRGIDRGDTEVSEKELAEIHAAGYITALESGVQSVMAS
---FNSWNGKRLHGHKYLLTDVLKERLGFDGFVVGDWN--GHRFVDGCTVDSCAQAVNAGLDIFMITAEWKALLKNTIAQAQSGEIPMNRIDDAVSRILRVLVLLKNND
QLLPVDAG-KNILVAGDGADISKQSGGWTISWDFPGATSIYTGIKQAVDAGEIKEDTFSNNAKPDVAIVIYGEEPYAEWHG-DIASIEYQKADQELLQKLKAQNIPVVS
VFLSGRPLWVNKE-LNLSDAFVAAWLPGSE-GAGVADVLLTDADFTGQLSFTWPGQVHQTVI---NRND---VEYQPLFAYGYGLDY
>W7QE10_9ALTE
TLEQKVAQMIQPE-----IGYLTLEQMRKYGFGSYLNGGNTPQEWLKFADEMYEASTDDSLDGSKIPAIWGTDAMHGHSNVSGT-TLFPHNIGLGAMNNPEHIRKIGDA
TAKEVAVTGIEWSFAPTVAVVQDDRWGRTYESYSEKPEIVKAYATAMVEGIQGERRIATAKHFVGD--GGTLNGIDRGDTIASEEAMRDIHAAGYFTAIEAGVQSVMAS
---FNSWKGKRLHGHKYLLTDVLKEQMGFDGFVVSDWN--GHKFVDGCDLEQCANAINAGVDVLMVPEHYEAFYHNTVAQVKSGEIPMSRIDDAVTRFLR-LVLLKNNQ
QILPLNPKQ-KILIAGDAADMAKQAGGWSVSWDFPGATTVYAGLKNAIEQKVEFDVAGNYQTKPDVAVVVIGEPPYAEWFG-DIQYIEYQKADLALLKKLKADGIPVVT
VFISGRPLWMNKE-INMSDAFVAAWLPGSE-GQGVADVILAKADFKGKLSFSWPKYDNQVILNPHD------ENYDPLFAYGFGLTY
>A0A3N4W8V7_9GAMM
-LEEKVGQMIQG-----DILSVRPEDLRRYPLGSILAGGSPAGPWIDTARAFRAVSLERRGDHEPIPVMFGIDAVHGNNNVVGA-TIFPHNIGLGAANDPKLIRRIGEA
TAVETAAAGFDWAFGPTLAVPQNDGWGRAYEGYSEDPDLVRRYAGEMVTGLQGERVAASAKHFLGD--GGTEGGVDQGDNRASEEQLVRVHNAGYPKAIEAGAMTVMAS
---FSSWQGVKMHGNRSLLTDVLKGRMGFDGFVVGDWN--GHGQVPGCTPTDCAAAFNAGLDMAMAPDSWKGLYENTLAQARSGAIPIARIDDAVRRILRVLVLLKNNG
GVLPIRPDA-KVLVAGPGADVGMQSGGWTLSWDFPNAQSIYEGLREALQARAELSVAGEFTERPDVAVVVFGEAPYAEFLG-DVQTLEHQKRDLALLKKLKAQGIPVVS
VFLSGRPLWVNPE-LNASDAFVAAWWPGSE-GGGIADVLVAGRDFQGRLSFSWPRTAAQLEL--NKGQ----PGYDPLFPLGYGLTY
>A0A0Q4GIV2_9SPHN
-VADKVGQLIQVD-----IASITPDDLRTYKLGSILNGGNPPAAWLKLADAFYDASMARSDAGPSIPVIWGTDAVHGNNNIPG-ATLFPHNIGLGAAHDRDLMREIGHV
TAIETAAAGIDWTFAPTLAVVRDDRWGRTYESYSEEPAIPADYAGAVIEGVQGIHVIATTKHFLGD---GGTGGRDQGDTRVPETVLRDVHLGGYPAAIEAGTQSVMAS
---FSSWNGAKMHGNQSLLTSVLKDRMRFDGFVVGDWN--GHGQVDGCTNESCAAAINAGLDMFMYSGDAWKTYANTLKQAQSGEIPAARLDDAVRRILRVLVLLKNEG
GLLPLKPSA-TILVAGGGADIPQQAGGWSLTWNFPNAESIWSGIDTAVRATATLSADGSFTKKPDAAIVVFGEEPYAEFKG-DRPTLEYSKSDLALLRKLKAAGVPVVA
VFLSGRPMWVNAE-LNASDAFVAAFLPGSE-GGGVADLVLRKRDFRGRLSFSWPRRPDQYVL---NRSD---PGYDPLFALGYGLRY
>A0A1Y0FUP0_9GAMM
TLEEKIGQLVQPE-----IKQVTPEDIKKYHVGSVLNGGGKLEDWVALADSFYHASVDKSDGRVGIPIMWGTDAVHGLGNVIGA-TLFPHNIGLGATHNPELIKQVGAV
TAREIAATGLDWNFSPTVAVARDDRWGRAYEAYSEDPEIVRAYAGKMVEGLQGIHVIATAKHFIGD--GGTLNGVDRGATQGDEKHLRDIHGAGYFSALEAGVQVVMAS
---FTSWQDTRMHGHKYLLTDVLKGQMGFDGLVVGDWS--GHGFIPGCTALNCPDSINAGLDIYMVPEPEWKEYNNLLEQAKTGVIPMARVDDAVRRILRVLVMLKNKN
GLLPLARNQ-KVLVAGDGADIGKQAGGWSISWDFPGASSIFAGISEVVTSTAILSVDGSFSEKPDVAIVVFGEDPYAEMQG-DVGNLAYKASDLELLKKLRSQDVPVVS
LFISGRPLWVNKE-LNASDAFVAIWQPGTE-GAGIADVIFKNADMKGRLTFSWPKRPDQGPLNRGD------ENYDPLFPYGFGLSY
>Q6CEN5_YARLI
---RLGIKSLCLQDGPLGIR--FADLTTFPAGITIASTFSRQLVRERGAA
MGRENRRKGVDITLSPVVGLGRHANGGRIWEGFSADPYLAGKLAAEAVTGIQGQNVMAVVKHMVGNGEWQGFGFKDLKSSNIDDRTLNEAYLWPFADAVRANVGSVMCS
---YQQINGSQGCQNAHILNGKLKEEMGFQGFVMSDWL----------AQRSGVASVLAGLDMSMPGDGLVWAGYELTRSVLNGTIDESRVDDMVTRILT-LVLLKNEK
KTLPLPTNIGNLNIFGIGSKGALIEGWGSGSVYPTDYQSPYDAIKERASKWGNLSNVEILSAAADASVVFVLSD---SGESTDRNNLTLWHNGDEVVKAVASKNPNTIV
VVTTVGPVNLEKWDNPNVTAVLLTGPAGDFGGRAAASILFGDIAPSGKLPFTIAKNDTDYPLTKIPED-GYKFDENQVFEFGYGLSY
>A0A1Y2F7W9_PROLT
---RLGFPGLCLQDAPLGVR--FADYTAFPALSQVAKTWDRSLFRAHAVA
MGEEFKGKGANVQLGPVCGLGTFAEGGRNWEGYAVDSYLCGQGMYEGVKGIQSTGVTACAKHFLGNEQQAPESGFKTNVTNMDDRVTHELYLSPFADAVRAGVGSIMCS
---YQQINNSYACQNSKLINGLLKDELAFAGFVVSDWGAVH----------SGVATYLAGTDMLMPGDGNSFSGPNLTISVLNGTLPETRLNDMAIRIM--IILLKNER
GALPL-KNIKSLAIAGMDAGGTLAMGWGSGSCQFPYLITPLEAISARARQNYAYDKINQTVKYANAALVFINSD---SGEGYDRNNLTAWHGGDKLVRTVAANCNNTIV
IVHSVGAIIMEPWNHENVTAVVFAGLPGQESGNSLTSVLYGDSNPSGKLTFTVAKKASDYTTLLYEPN-GYKFDAKKIFHFGYGLSY
>A0A1E3QEV4_LIPST
---RIGFPSLCLQDSPLGIR--FTDLSVFPAGLATAATFNKNLMYLRGKA
MGMEFKGKGINAILGPCMPIGRAPEGGRNWEAFGADPYLQGVASYESVLGIQEEGVIATAKHYLLNEQEHFRQYAEWVSANVDERALREIYAWPFADAIRAGAASIMCS
---YNQANNSQACQNSYLLNGILKDELGFQGFVMSDWL----------GQRSGVASVLAGLDMTMPGDGLVWSGSNLTTAVLNGSVPLWRLDDMATRIM--MVLVKNTN

NALPL-NNVRRLGLFGSAAGGTLGIGWGSGTANYPYLLTPLEVINAMA-IDYDLDNVATLAGVVDTAIVFITAD---SGEGYDRNNLSAWHGGDNLVTAVASENSNTIV
VVESVGGIDLEAWEHVNVTAVLFSLVPGQDVGVGIADILYGTVSPSGKLPFTIAKKTSDYPAVIYDVIEPYRFDKYDIYEFGFGLSY
>A0A2P7QKD4_9SPHN
-VEEKVAQTIQPD-----IASVTPADMRRYKFGSILNGGNPAREWLALADAFWDAAMSAEWAGEKIPPIWGSDAVHGHTNVVGA-TIFPHNIGLGAMRNPDVIQKIGEV
TAAEMALTGIDWDFSPTLAVVRDDRWGRSYEGFSEDPEIVRSYAGRMVEGLQGRKVISSAKHFVGD--GGTSAGKDQGDNPSTPEELRDIHGAGYPPAIEAGVQAIMAS
---FSSVRGEKTTGDRDLLTVALKEDMNFDGFVVGDWN--AHGQVPGCSNTSCSAAMNAGLDMYMAPDSWRGLYDSTLAQVRSGEIAQARLDEAVRRILRVLVLLKNQN
KLLPLSPK-RNVLVAGDGADIAKQSGGWTITWDFPNGQSIFGGIAETVKATATLSTDGSYKSKPDAAIVVFGEEPYAEFVG-DRPTIEYSKKDLELLQKLKKAGIPVVA
VFLSGRPMWVNPE-INASDAFVAAFLPGSE-GGGVADVLFADADFKGKLSYSWPKRVDQSPLNR--G-D---TNYDPLFAYGFGLTY
>A0A2M8H3D1_9VIBR
TIKEKVGQMIQPN-----LRDVTPQELREYKLGSILNGGGAREWAEKADEFWYATEQAFEHRPFRIPFIWATDAVHGHNNVFGA-TIFPHNIGLGAARDPELIRRIGEV
TAREVCATGLDWTFAPTVATPRNLRWGRTYEGYSEDPEITYLYASAMVEGLQGDKVISNVKHWVGD--GGTGQGTDRGNNTYSEDLLRNIHAMGYFSGLTAGAQVVMSS
---FNRWENPKLHGSRYLLNDVLKQQMGFDGVVVTDWH--GHSEVSKCSDGNATYAINAGNDILMVPVRQHWKYEQTLRDIENGHISIARIDDAVTRILR-LVLLKNNH
NLLPLANTT-KILLTGSGADIQKQCGGWNLTWDFPGSCTVKDALTQEIGSPELTSDLSVAADVIDVAIVVFGEDPYAMGDIKEWQTLEFSRRDVAKINRLREMGIKVVS
VFFSGRPLFLNQE-ISASDAFVAAFLPGSE-GRGITDVLIGDEDFNGRLSYSWPNKKMRIPHGYQVPDEQDPSEHAPLFPYGYGLCY
>A0A2P8A4S3_9PEZI
---RLGFPGICLNDAESGVR--TGKLSGYPAQLHVGASWNRKLAGDRATA
IGKEFKKKGINVLLGPVVGLGRVAKGGRNWEGFTNDPYLAGSLVEPTITGMQR-SVVACVKHFIGNEQETNRSANESVSSNIDDRTMHEAYLWPFYDAVRAEAASIMCS
---YNKINGSYGCANSKTLNGLLKTELGFEGFVVSDWY----------AQHTGIASNNAGLDMVMPSSQFL-NPNSLAGAVANGSVSADRLNDQATRILA--VLVKNN-
GVLPLNK-PATLNLFGYDAIGTIITGGGSGAITPVLSYAPYDAFVQQAAQTDFSSQRPVVRAPNDPCVIFINAA---SSEAYDRTTLADE-YSDTLVTNVANSCKNTMV
VIHNAGIRLVDRWDHPNVTAVIYGHLPGQDSGLALTEIIYGRQSPSGRLPYTVGRTETDYGSSFADSKY-----SQSTFSEGLYIDY
>A0A0R1J4Q7_9LACO
---TKVNNLKLSDVYNKKVSETFVANLDNKELVDLVEGSLNDSIINASSLVKGAAGQTVENKKRGIPETVNADGPAGLRLDKSYNTAWPIGTLIAQTWNTKLGQEMGSA
IGEEMKRCGVTLWLAPGMNIHRDPLGGRNFEYFSEDPLLSGIMAASETKGVQSHGLGVTIKHFLGN---NQESYRNTGNSIIGEQALREIYLKNFEIAIEEQPLAIMSS
---YNMVNNYFSGANFEALTNILRDEWNFEGLVMTDWFS----------AADPRESMHAGNDLIMPGASKDTL----------------------------MVLLQNNH
HVLPLKTK--TVAVYGSGAFATVKGGTGSGDVNQ-RTTSIVEGLENSGFTFSMDDPEISDFNEAPVGIYVISRS---SGEGFDRGDFQLSENELSNIQRLSEYYENSVL
LLNVGGIIDTSFVSCPLLDSILLISQPGMTAGDAVTEILDGTKTPSGKLSDTWADYQK-YPANFGVKN-PYRFDSFQIYEFGFGLSY
>A0A495JDW0_9ACTN
--EEKLAQLGSTWAFAGRFSVRRARPILAQGLGHVTRVAGSLKAAQVAQVANAIQRYLVTETRLGIPAIVHEEVCSGVM--AREATIFPQAIGVASTWSPELNGQLADA
VRAQMRAMGSHQGLSPVLDVVRDPRWGRTEETYGEDPYLVARMGVAFVKGLQGAGIIATAKHFVGY--GASEGGLNWAPAHLPPRLLREVYLYPFEAAVEGGLQSVMAG
---YHELDGIPCHANRELLVDILRRQWGFGGSVVSDYFAVNDLHSYHHQQQAATLGLGAGVDVELPATDAY--ADALTRALDAGEVSEARLDEAVSRVLR-LVLLKND-
GVLPLLPEAATVALIGPNADARHLLGDYSFAAHI----EALTEARERRGLTDGFDEAVAVAAAADVAVLVLGDRSGLTGESRDRSSLDLPGVQEDLVRAVVATGTPVVA
VLVAGRPVGSD-FLHEQCAAVLMAWLPGQTGAQAIAEVLLGEVNPSGRLPISYPRSVGQIPV-FYGHKRSYVSPVAPRYCFGHGLGY
>A0A1E5LIC4_9BACI
TLDEKIGQMTQVE----RNSIGADSNLATFNIGSVLSGGGSPNTPEAWANMYDGYQRAALQSNLQIPIIYGIDAVHGNNNAYGA-TIFPHNIGLGAARNPELLREIGRA
TAEEVAGTGVDWTFAPCLCVSRDERWGRTYESYGEDPEIASSYV-TIIEGLQGDTILANAKHWVGD--GGTLGGDDQGDTVLSEQELREIHIPPFIDAIEAGVGSVMAS
---FSSWNGEKLHGHKYLLTDVLKDELGFEGFVVSDWAGIDQL--PGDYASDVRNSINAGIDMVMVPQDYRTFINTLRIEVNEGRVPMSRIDDAVSRILT-LVLLKNEG
DILPLDQGLDKVFVAGKNAN---DIGNQSGGWTITWQGTILEGIQSTVSPVTFNESGDGINSSYDVAVVVVGETPYAEGEGDRPNDLRLDQTDLATLQTIQSTGVPTVV
ILVSGRPMVVTDE-LANWDAFVAAWLPGTE-GDGVAEVLFGVYEFTGKLPFTWPRSESQIPMNVGDA------SYDPLFEYGFGLEY
>A0A3N4LVN3_9PEZI
---ERLGFPSLCAQDGPLGIR--YNDHTAFPAGITTGATWDRDLMYERGKG
MGAEAKGKGIHILLGPAVGLGRQPRGGRNWEGFSADSFLLSEASYHTVKGIQDAGTQATIKHYIANEHFRGDGGSDTISSDIDDRTMHEVYLPPFAAAVRAGVASVMCS
-YNTPQINASYACENSKLLNGILKDELGFQGYVMSDWL---AQRSGVGSALAGLDMVQPG-DGNLWADGNSLWGPELSRSILNSSIPVDRLNDAVTRIV--ITLLKNEN
NVLPLSKS-DTIRVFGQGAGGVLTQGWGSGTSLPEDLIAPIDAIKEIAHDDNVTAEVKKMASANAKCIVSITAD---SGEIFDRKDLKAWHNGAALVKAVADSCKNTIV
LIHSVGPIFMEEWNHKNVKAVILAYLPGQGTGYPLTDVLFGKVSPSGHLPYTIGKREEDWGDIVTEGKDQYKFDKQGIFEFGYGLSY
>A0A5E8B4B1_9ASCO
---RFNIPSLCMQDGPLGVR--FTDFSAFPAGITAGASFNKELIAFRGKA
LALEHKAKGVHVVLGPAVGLGRNALGGRTWEGFGADPYLQGVAARATTREIQKAGLVATIKHFIGNEQEHVGEYYGSGSSNIDDRTMHEIYMWPFADVVNEGVGSVMCS
---YNTINNTYACENSYLINKLLKEELGFQGFVMSDWWAMKTGI----------FSAQAGLDMAMPGDSGYFTGNRLTRAVLDGSLGDWRLDDMAVRIL--IVLLKNSG
NVLPLSLNPRKINIFGKAAGGALGQGWGSGAVDFQHFVTPFEAVNDAARKAFNLDGFLSKVSIADANIIFALTN---AGEGFDRRNASLWHNADEVILRAAEKNKNNIV
VISSVGPTNLERWDHPNIKAVLFALPGGQDAGIAVANVLFGLSNPSGKLPFTIARDDKDYPIP----GKPYRFDKNGLYEFGYGLSY
>D2RV76_HALTV
TIEQKVGQMAQVAIDNHDDAGTLGKLFSELHVGSILNGGATFDGEEFVEGLNGLQEYNLEVNEPAIPFVWGCDALHGNC-LLDGCTSFPQRLNMGATRDVDLVEAAATH
TGDSVAAIGGHWNFGPTLDVLRDMRWGRYFEGHSEDAMLLGEMGRARARGFQRNRVAATVKHFAGY--GTPNTGSDRTHARTSMRDLRTRQFEPYRRGLEEAKT-VMVN
---SGAVNGKPAHASSWLLTTVLRDRFGFDGVVLTDWDDFERMLSNHEWREAVRQGIEAGVDMHMCGGETAPTIDTVIDLVESGDLSEARIDESVRRILELLVLLQNED
DALPLLEDVDDLLLTGPGVHLTARLGDGLTHVPTEYEPAVYESLYENFDNDEQAAAISEAAPGSDAVVVVLGEG--THNEGFDRDKMRFLEAQRELVELVDSETVPIIG
VILAGSPRGTAET-FQHLDAVLFAGQPGSDTGVAVVDTLFGDYNPSGKLPFTWESHVGHVPQDEYPPRHP-DGAGDQMFEFGHGLSY
>A0A0N1ANH4_9SPHN
TLEEKVGQILQAD-----LSTVTPDDVHRYHLGSVLNGGNPAPEWLKLADAFYDASVDKRDGGVAIPVMWGTDAVHGHSNIVGA-TLFPHNSALGATHDPALLQRIAAV
TATEVRVTGIDWTFAPTITVPQDTRWGRAFEGYSEDPALVASYARPFIEGLQGKHVIATAKHFLAD--GGTFEGRDQGDAVIDEATLIKVHAQPYVSAIDAGVLSIMAS
---FSSWHGVKMTGNKSLLTDVLKKRMGFAGLVVSDWN--AHGQVAGCSNASCPQAVNAGIDMLMAPDSWKALYHSLIAQVQDGTVPMARLDEAVGAVLRVLVLLKNEA
GILPLSPKA-RILVAGDGANIARQSGGWTLSWLFPGATTLWQGIDTAVRAATLSPDGSFAGQKPDAAIVVFGETPYAEFQG-DLRSLQLKRAPLETMRKLKAAGVPVVA
LMLTGRPLYTNPF-LNLADAFVVAWLPGSE-GEGVADVLLRGSDFTGRLPFRWPMTAV--PG------------GATLYPLGHGLS-
>A0A545SLY5_9GAMM
TLEQKVGQLMQPE-----IRHVSNDDVRRYYLGSVLNGGGSLDDWLSLADGFYHASMSVDA-DVKIPIVWGTDAVHGHNNVIGA-TLFPHNIALGAIDNPALIEAIGAA
TAREMAATGISWSFAPTVAVARDDRWGRTYESYSEAPALVGRYARAQVLGLQGRRVIATAKHFIGD--GGTAGGEDRGDTRLTEEELIRIHAQGYFRAIEAGVQVVMAS
---FTSWNGDKLHGHRYLLTDVLKNRLGFDGFVVGDWA--GHQFVPGCTVTRCPAAINAGLDMFMAPDDNWREYTNTLADVKAGRISMARLDDANARILRVLVLLKNRG
QLLPLSPKA-HVLVAGNGAHIGKQSGGWSITWDFPGATSVYDGIKAQVEARVSLSENGDYSEKPDLAIVVYGEDPYAEMQG-DTSHLGYHADDLALLRKLRDAGIKVVS
LFVTGRPLAINPY-LNASDAFVVIWLPGTE-GKAVAEPLFTKPAFTGRLSFSWPQFADQAPLNLDD------PLAQPLFAYGYGL--
>A1S5F5_SHEAM
TLEQKVAQIIQPE-----IRDFGVEDMRRYGFGSFLNGGGKAADWVALADQMYHAAMDDSIDGIAIPPMWGTDAVHGHGNVFGA-TLFPHNIGLGATQNPQLIKAIAAA
TAKEVRATGIDWVFAPTVALVDNLRWGRTYEGYARDPELIERYAEAFVDGMQGEYTLATAKHFIGD--GGTDNGDDRGDTRVDENTLIARHGQGYVGALGHGVQTVMAS
---FNSWNGEKLHGSKYLLTDVLKERMGFDGVVVGDWL--GHGFVPGCSYEHCAEAVNAGVDILMAPGDSWKAYANTIADVKSGVLPLSRLDDAVKRVLRVLVLLKNNR
PVLPIAANA-RVLVVGEGADIPQQSGGWSMTWDFPGATSIFAGIKAALNALLSSDGTIPVGFKPDVVIAVYGEQPYAEGNG-DLDNLEYQKRSLAMLSALKATGLPLVS
VVLSGRPLWMNPE-INVSDAFVAAWLPGSE-GAGVADVLIGDKDFKGRMPFPWPATPSAD----------------------------
>A0A1C7D6Z0_9SPHN
TVEEKVGQILQPE-----ITNITPDEVREYNIGSVLNGGNPASEWLKLADAYWTASTDKSDGGVGIPIIWGIDSVHGNNSYFG-GTIFPHNIGLGAANDPDLMRRIGRV
TAIETAAIGTDWTFAPALSIPRDDRWGRTYEGYSEGPEIAGRMGASLTLGLQGDSIIATAKHMVAD--GGTAGGRDQGDAQVSEDVLRDIHWAPYAPTLEAGAQTVMAS
---YSKWNGVRMHGHGPLLTTMMKDHAGFDGFVIGDFN--GHALIPGCTAGDCPEALLAGVDMYMIPADWKELYNNLVVQVKDGTIPMSRLDDAVRRVLRV-VLLKDDP
SILPFPANS-RVLVAGQGADVPMLVGGWSMNWDFPGSTTVYSGLRQAMAPTAELSVDGQYETKPDYAVVVFGETPYAEYQG-DRESVIYADSDLALLERLKADGIPVIA
VFLSGRPLWVNPH-LNASDAFVAAWLPGTQGGAGLADLLVSDGDFTGRLSFSWPKLASQTAL---NQG---NANYDPLFPVGYGLS-
>A0A6S7CJ32_9BURK
TIEEKIAQMHAFWLILASDQAQLQNRL-KLGLGQITRPLGSVDPVSGVRALNHLQKFLREETRLGIPALSHEECLVGVMTRGG--TMFPSALNYGTTWNPELIEKVATA
IGTEARSLGCHQGLAPVLDVSRDVRWGRTEETFGEDPYLIGVLATRYVRGLQGDDLLATLKHYVGH--SFSEGARNHAPVHLGPRELNDIFLLPFEMAVQANAGSVMPA
---YHDVDNEPAHSSHYLLTEVLRNQWGFDGLIVADYVGVSLLYQHHGPAEAAALSFNAGLDVELPGDDCA---AHLREALARGQITEAKIDEIVSRVLT--VLLEND-

GILPLPDSGKKIAVIGPTADPLAMVGGYSFPVETSEIVTPLQGLRKVYGDTSLIDAAVQAARESDVAIVFVGDLAGLVGEGSDTDSLNLPGVQQQLLEAVVATGKPVIA
VITGGRPYILNGL-EHKLAALAMAFAPGQRAGDAIANVLSGTASPSGRLVVSVPKSVGAMPY-FYNHKK---SAGTPIYPFGHGIGY
>A0A176VUW0_MARPO
---KGATSFPMPISLAASFNKSLWNKIGQV
ISTEGRAMGQSTFWSPVINLVRDPRWGRVQETPGEDPYLIGQYSVYFVRGMQEAKTSACCKHYTAY-DLDNWQGVERYDFDVTIQDLADTYNPPFQSCVEGRASCLMCS
---YNKVNGVPTCADPELLKGTVREKWGLRGYIVTDCDSLLVMYDESRPAEAIATAMLAGLDLNCGATIKT----YGASAVQQRLISEADIDRALN-----MVLLKNDE
DTLPLSRHIKTLAIIGPNADKYTMLGNYAGRPCT--YITPLQGLSTYEINDEKIEDALSAAKKADVVILVMGLDQDLERETFDRNSLKLPGKQELLVSSVANVNGPVVL
VLICGGPLDISWANDSRIQSILWMGYPGQAGGLALAQIVFGDRNPVGRLPVTWYPSITDWPMM--NMRHTHRYNGPTVYEFGYGMTY
>A0A1V3QPG0_9GAMM
-LAQKIGQMTQAE-----IKSITPAEVTKFYIGSVLNGGGGIADWLALSDRYYDASMATDA-AIKLPVIWGIDAVHGDNNVFGA-TVFPHNIGLGAAHDPALIEEIGAA
TARAVRATGVEWAFAPTLAVAQNARWGRTYESFSSEGPLVRAYARAYVTGLQGGNVMATAKHFIGD--GATWNGTDQGDARVSLSDMINVHGSGYFGALEAGVQSVMAS
---YSSWDDVKMSGAHALLTDALKEKMGFDGFVVSDWNAIGQL--PGCSNASCPQAINAGIDMVMVPDDWKAFIANTIRQVEDGQIPMARIDDAVSRIVR-LVLLKNNH
DVLPLKPG-RKILVVGKNADLPNQTGGWSLTWDFPHGETILDGLRQAAGPTYSETAQGVDLQPFDAIVVVIGETPYALGDIMPSATLRHSPEDLAVLQTVAKGHKPVVT
VFVAGRPLYVNNL-LNLSDAFVAAWLPGTE-GAGVADVLFGSYNFSGTLAMPWPGVPC--PD-ASNGS---AKATRWLFAPGYGLRY
>A0A401KYP2_ASPAW
--RLGFPGLRLHDGPNGLN-ALEEVTAYASGITVGASWNKDLAHARGQS
MGKGARRKGATCWDRLLVRLAGQRPGGRNWESFSVDPYLCGQMGAKTVLGIQE-NVIATAKHFVLN-EQETDRGNASVSSNIDDKTMHEYYLWPFQDAVKAGVGSVMCS
---YQRVNGSHSCQNSWTQNGLLKTELGFQGYIISDYN----------ARWGGIASTQAGMDLVTPPSAVW--PQNLTIAVENGTLAESRLDDMVIRILT--VLVKNVN
NTLPL-KKPKILSLFGYDAHGTLVGGGGSGSVTPQYINSPFDAFQQRAYEYWDFDSEDPSYSESDACLVFINEF---ASEIVDRVSLADQ-YSDNLVMNVAKKCRNTIV
SIHNAGVRLVDRWDHPNVTAVIFAHLPGQDAGPALVDIMYGEQAPSGKLPYTVAKNESDYPVNYYTHA---------DFTEGSYIDY
>Q0AL31_MARMM
TVEQKVGQTIQAD-----SGSVTPEDVRNYRLGSVLSGGNDAQTWLDAADAYFNASIDPDGVEIAIPIIWGIDAVHGHANL-RGAVVFPHNIGLGAMNNPDLIEDIYRV
TARELSVSGHDWTFAPTLAVPRDDRWGRTYEGFSEDPAIVAAYGERIVWGLQGRRVISSAKHFLAD--GGTLDGRDQGDAQISEAELRDIHAAGYYTAIPAGVLTVMAS
---FSSWNDARMHGNESLLEDVLRDRMGFTGFVVGDWN--GHGLIPGCVSTDCPESFNAGVDMFMAPDSWRELYHNTLAQVRSGEISMERLDQAVRRILRVLVLLKNVD
QTLPLDPSL-TILVVGAGADIGKAAGGWTLSWEFPAGDSILAGIQAAVDTQVIYDPDGQSDIDADVVIAVYGENPYAQGD-RDHLDFVSPDYDTSLLEQYQARDIPVVS
VFLSGRPLWTNPE-INDSDAFIAAWLPGTE-GGGVADLIFRDYDFTGRLSFSWPSLASGEPLNVGDA------DYDPLFTYGYGLSY
>A0A162J2L5_9PEZI
--FSYATSFPGPITMSAAFDDELIERVATI
VSTEARAFGLDF-WTPNINPFKDPRWGRGAETPGEDPFRNKGYVRALLRGLEGPKVIATCKHFAAYDLERWNGTVRYGFDAISLQDLSEYYLPPFQECADSRVGSIMCS
---YNAVNGTPACASSYLMNDILRKHWGWHQYVTSDCNAILDFDHEHKGAHAAAAAYGAGTDTDTVCEVPSY--EGTDVRGAYNQSLLSEAVLDRALTRL----VLKKNDV
HALPWNLTGRRVALIGHWADTRQMLGGYSGIPPF--YHNPVAAARARNWSDTWTEPALAAARQADAVLYFGGNDLSIEAEDKDRINITWPQAQLDLLTALAALGKPTAV
VQLGGGQNDDTQLRNPNISAVLWAGYPGQDGGNAALDIITGVHAPAGRLPVTQYPGSYDAP--MTNMARPYRWYDHAVLPFGHGLHY
>K6YCB7_9ALTE
TLEEKVGQVIQ-----GDIASVTPQEAGEYNLGSVLNGGSPPEEWLQLADEFYQESTDTSDGGVGIPLLWGIDAVHGNNNVVGA-TLFPHNIGLGAANDPNLMRKIGEI
TAKEILVVGIDWTFAPTLAVVQNDKWGRTYESYSENPEIVASFAGPLVEGIQGKHLLANVKHFLGD--GGTKDGKDQGDTLVSEAIMRDIHGAGYPPAIQHGALVVMAS
---FNSWHGRKMHGSREMLNDILVERLGFDGVVVGDWN--GHGQVAGCSNVSCPQAFNAGLDMFMAPDSWKELYKNTLKQVKSGEISLARLDEAVSRILRVLVLLKNNN
QLLPLQAN-SKVLVAGGGANIAQQSGGWTLSWHFPNAESIYQGIEKAVQQQVELNENGDFETKPDVAIVVFGEQPYAEFQG-DVTDLDYKNADLALLTSLKEQGIPTVT
VFLSGRGMWVNPE-LNVSDAFVAAWLPGSE-GGGVADLLFKNADFTGRLSFSWPATPLDVEVNIGD------ENYQPLFAYGYGLS-
>A0A2S7KAH9_9PROT
TLEQKVGQIIQAD-----SSSVTPEEVRQYRLGSVLSGGNTADEWLAMADAFYDASVDPEGVETAIPILLGIDAVHGHNNVIG-GTVFPHNIGLGAARDPDLVRRIAEA
TAKELRVTGHDWTFAPTVAVPQDDRWGRTYEGFSEDPDIVASYAGVIVEGLQGEKVVSTAKHYLGD--GGTEGGRDQGDAQASEADLARIHGAGYPPAIRAGALSVMAS
---FSSWKGEALHAHRYLLTDVLKDRMGFDGFVVGDWN--GHAKVEGCTNEDCPAAVNAGLDMFMAPDSWKGLYENTLNEARAGEISEARLDDAVRRILR-LVLLKNNE
GVLPLRRDL-KVLVAGDGADISKQSGGWTLTWLFPNGESVFDGLKAAVESQIALSENGAYDEKPDVAIVVFGEDSYAQGD-LDNVAFNDPGEALAIMRKLKADGVPVIS
VFLSGRPLWVNPE-LNASDAFVAAWLPGTA-GGGVADVLFRDSDFTGRLSYSWPKNAAQTPLNK--G-D---EGYDPLFALGYGLTY
>Q5V5G3_HALMA
TLEQKAAQMTQVAISSSFGVDTVGEYFSELGVGSILSGGASFDGETVVQGINALQEYNLENADHDIPFLYGVDATHGNG-LLAGATVFPQRLNMGATRDLSLIEAAERH
TSDATASMGAHWTFAPTTDLQRDPRWGRFFEGISEDPKLEADVSRVRARALEDDRLTACVKHFAAY--SIPNNGNDRAPASTSLRDLRTNILPPYREALKSEPGTVMVN
---SGSINGVPAHASHWLLTTLLRDTYGYEGMVISDWDDLNRMITNHDFETATEMAINAGVDMYMIGNGGDAPIDTVVSLVEDGAIPMERIDEAVRRILELMVLLKNTD
DTLPLSGD-ESVLLTGPGVDALMQHGGWTLGWPFPRQNLLEEALRARVGSAEQRSRVESAGPESDVVVVVLGEG--THNEGFDRDELVLDESQQALLDTVVESTAPIIG
VMLAGSPRGSPET-FSQLDALLFAGQPGSDGGVAIAETLVGEYNPSGKLPFSWPENVGTTPVQYTRYDS-TGTDNTAIYEYGHGLSY
>A0A2T9Z6K8_9FUNG
-LQEKVGQMVHVHIGKGLLNVTLAESIEKYKIGGVYGSPVNIASPQRWANLTNTLQKIAMEKGSKLPFIYSLESPKGAGNIKG-SILFPAPVNIGATFNLMHAYIPSQI
VAKDIRAAGAHMIHSPSANLNVNRLWKHNYESFGEDPYLAGEMVYTTVRGLQGNRVAACVKHYIGY--SGSKNGDDKEPRNIPYHKITEYHLYPFLRGFNAGATTAMLS
---PNTLNGEAVSSSRYLKNDILRNFLRFSGVVVSDWSEIKSMVKFGTPNWGIYQSFEDGVDVSMSSDGF-DFIDGIFDMLNRNAFSKDKIYTSVGRILQLIILLKNEN
RVLPFHKN-DKVVFIGPIFNTRYMCGGWSVHRYYGYGDTLEQAIEKIIGRVDDYTELGRHVHRADKVVIGFGEK--SAGEA---NMLDLTESQISLVEYLAKLTTPIVF
LLMQNTPRIIDGI-IKYADGILNVNLPGAYGGLPIAEAIYGSFSPSGRMPYTYPKSNLQTDLTYYTP---VTSEYDPTFAFGTGFGY
>A0A383V2S3_TETOB
--SLTTEEKLSLMNNYQQPVERLGVGG--YQFWTECLHGHQERGAGATIFPQPLLLASSFDDALAWQVFNA
ISDELRAKDNAVCWTPHVNIFKDPRWGRGSETFGEDPVLTSRMALRVVQGLQGSKVVATCKHFLGYGLEGAEGHSRYSNIDISPQDLTDTDLPPFRACVGAGALGIMCA
---YNAVNGTASCASAPLLQKLLRGNMGFDGYVVSDCNAIQALEWGHEMRDASAAAVKAGVDL--FCDKA----DQLKPAIEAGLLSSADIDAAVNRTLA-IVLLKNKA
PLLPLKASLKKLCVLGPLANAEHMMGNYYGNFDADIAATPLQGIQEELAGDWPLETALKACNGADAAIVVIGSSM-IEGEGLDRNNLDLPGRQADLVRALAAPGLPVAV
VLFNGGGLDVGWIRMPAVSAMMAAGFPGQEGGRGIADVLFGRVSPSGRLPITWHHSNYTYRVL---RMRPYRFVSDPSYPFGYGLSY
>A0A1D1VK38_RAMVA
TLAEKLGQLQQLDGDYGTFQPYMIDMVRNGLLGSTIN-------VRGANQTNTLQRYAVES-RLKIPVLFGFDVIHGYR------TLFPIPLGETASWDLDAVEQASAI
AAAETRSVGLHWTFAPMIDITRDPRWGRVMEGQGEDVYLGNQMAKARVRGFQGDKVLACAKHFAGY--GAAEGGRDYNTVDMSERRLRETYLPPFKAAIDAGVGSFMTA
---FNDLSGVPATANEFLLREILRDEWKFDGLVVSDYDAVVELTNHQLDSEAAMYALNAGTDMEMISRTYN---THGPQLVQSGKVSTATIDGAVRNVLR-LVLKNEN
NTLPIDKNIGKIAVIGGLAARIETLDHWAGDAKWENSTTMLEGIMEKLGVSAGFDAAVRVAEAADFVVLVVGEPREYSGEGGSKTDLDLPGYQLDLVKRIRATGKPFVA
VVVNGRPMTLEEL-HHSAPAVLITWRSGTMGGHAIADVLFGDVNPSGKLPMSFPRNVGQIPL-YYDYKRPYLVLNSPLYPFGFGLSY
>A0A5J5C326_9ASTE
------------------------RMTLKEKLGSLLSCCDPYEKTSEWAEIVDAFQTSVLESRLGIPLIFAIDAVHGNNNVYGA-TIFPHNVGLGATRDPYLAIRIGAA
TALEVRACGFQYNFAPCVAVCRDLRWGRCYECYSEDTEIVQKMT-SIVTGLQGQNVVACAKHFVGD--GGTENGTNEGNTVSSYDDLEKIHMAPYLDCISQGVCTVMAS
---YSSWNGSKLHSDHFLLTKVLKENLGFKGFVISDSEALERLSEPRGYRNCILSAINAGIDMVMVPCRYELFLEDLTYLVEAGEIPMTRIDDAVERILRVLVLLKNGK
PFLPLDKNAKRILVTGTHADLGYQCGGWTATWRITTGTTILEAIKTAVGDKQDPSPDTFAGQDFSFAIVVVGEAPYAETAGE-NSELIIPFNGTEVISSVSDR-VPTLV
ILISGRPLVLETWLLEKIDAIVAAWLPGSE-GGGIADVVLGDYEFQGRLPMTWFKRVEQLPM--HAEE----NSYDPLFALGFGLT-
>A0A545SY60_9GAMM
TLEQKVGQIIQAE-----IGFVTPEDVRRYHLGSVLNGGGSVAEWVALADAFYLASMDTAGGGLAIPMLWGTDAVHGHNKIFGA-TLFPHNIGLGAAGDPALVRDIARV
TARAVRATGLDWTFAPSLSVVRDDRWGRTYEGYAEDPQLVERLAGAAVAGFQGARVLATAKHYLGD--GGTENGIDRGDTTVTEAVLRDIHAPGYFAALDAGVQTVMAS
---HSSWQGERMHGHKYLLTDVLKQQLGFDGFVIGDWN--SHGLVPGCSNASCPQAINAGVDMIMVIEDWREFWYNTLAQVKRGDISQARLDDAVRRILRVLVLLKNKN
GLLPLRPQA-RVLVAGDGADIGKQAGGWSISWDFPGATSIYTGIRAAVTASVEFNVDGAYRQRPDVAIVVYGENPYAQGDLQSPAYQPYSHRDARLLEKLQSDGIPVVS
IFISGRPLWVNRA-LNASDAFVAAWLPGTE-GAGIADVIFTDADFTGRLSFSWPRLASQVRL--NRG-D---ADYEPLFPYGYGLG-
>A0A559QPW8_9ALTE
-LEQKIGQMMQPS-----IAHVSPEEVKRYYIGSVLNGGGTPQEWQALADAYYQASMAVPDGVPAIPVIWATDAVHGHNNVVGA-TLFPHNSALGAANNPELVRLIGAA
TAQQMVATGLDWNFAPTVAVAKNARWGRTYESFSENPGIVAALSAAYVAGLQGYKVIATAKHFIGD--GATTRGDDQGDADLNEQQLIAEHALGYFSTLSMAVQTVMIS
---YSSVQGLPMHGNKHLITDILKNQLHFDGIVVSDWNALGH--VPGCTRDNCAAAINAGIDVLMVPYKPDWPIANTIAQVKSGEISMQRIDDAVSRILRVLVLLKNND

GVLPIAPN-KNILIAGDAANVSRQTGGWTISWDFPVATTVFQGFESAVQATVHYSATGEYEVIPDVAVVVFGERPAASGDIENLNTLEFNPEPLKVLQKLKAAGIPTVA
VFIAGRPRLINKE-LNQSSAFVMAWFLGSE-GGGIADLLLANQDFQGKLPFVWPATPCHVLG------------SQPQFNLGYGLSY
>A0A369QD44_9SPHN
TLEQKVGQLIQAD-----IGSISPADLKTFPLGSLLAGGNSAADWDRMVREFRAAGLSYDGGGTPVPLIFGIDAVHGHNNIPN-ATIFPHNIGLGAARDTDMIRRIGAA
TAAEVAASGIEWTFAPTLAVPQDLRWGRAYEGYSSDPAIVAAYSAAMVEGLQGQKVAATAKHFLAD--GGTAGGKDQGDAQMDEAELVRIHNAGYPPAIEAGVLTAMAS
---FSSWNGVKHHGNKTLLTDVLKDRMGFDGFLVGDWN--GHGQVRGCEATDCAQAINAGLDMFMAPDSWKDLYANTLEQAQNGTIPVARLDDAVRRILRVLVLLKNNG
SVLPIKPGA-RVMVTGPGANMAMQSGGWTVSWDFTNGQTIWQALDAATGATAVLSEDGSFKQKPDVAIVVYGETPYAEFQG-DVATLDYQATDLASLEAIRAKGVPTVS
VFLSGRPMFTNPE-MNASDAFVAAWLPGTQ-ANGIADVLIARRGFTGRLSFPWPNSCGG--------------RGTPLFPLGTGYT-
>M2N8C7_BAUPA
---YATSFPMPITFSSAFDDPSVQNIASV
ISTEARAYGLD-YFTPNINPFKDPRWGRGSETPGEDPLRIQGYVKNLLIGLEGTKMIATCKHFAGYDLEDWDGYIRYGYDEITTQDLAEYYLPPFQTCADQNVASIMCS
---YNSVNSVPACANSYLQETILREHWGDNNYITSDCNAISDIYYNHNNAAAAGLSLSNGMDTACIVANT-GVMTDVNGSYYGGYVTEATITTALIR-----TLLKNT-
GLLPYFTSQTKVAMIGMWANTSQMQGGYSGP--APYLHSPLYAASQLGLSSNYSQNATAAAQNADVILFFGGIDWSVEAEAMDRYQIAWPGAQQALIAQLAALGKP-MI
VLQMGSMLDATPISNNNISALVWVGYPGQDGGVAAFDILTGAVAPAGRLPVTMYPADYVNQVPMTNMSRPYKWYNNAVLPFAYGLHY
>F7NRC8_9GAMM
-IEQKVAQLIQPD-----IRWMTVKDMRQYGFGSFLNGGGTAADWLALAQAYYDAGVDTSIDGSSIPPIWGTDAVHGHNNVVGA-TVFPHNIGLGAANNAQLVEAIGRT
TAVEVAATGINWIFAPTVAVARDDRWGRTYESYSEDPTIVKELGAALVKGIQGNRLIATAKHYLGD--GGTENGKDQGNNLDNEADLVRLHAQGYISSLNAGVQTVMAT
---FNSWHGEKIHGSHYLLTTVLKERMGFDGLVVGDWN--GHGQIPGCTNTNCAAAINAGVDILMAPEDWKLLYQNTLAQAKAGEISAARLDDAVSRILRVLVLLKNND
QLLPLAPKQ-KVLVAGDGAD---NIGKQSGGWDFPNGRSIYSGIQQQVEASVELSADGSYQQKPDVAIVVIGENPYAEFDG-DISTLDYQNTDLDLLKKLKADGIPVVT
VFLTGRPLWVNPE-LNQSDAFVAAWLPGTA-GQAVADVLFKTQDFKGKLPFSWPKTAGQSPLNQ--G-D---SNYDPLFAFGYGLTY
>U9VW20_9CYAN
TLEEMVGQTIQA-----DISHVTPEDIRHYRLGAILNGGNATEDWLPLADAFYEASMDTSNGDQAIPILWGTDAVHGHNNIISA-TIFPHNIGLGATRNPALMEQIGAI
TAREVIVTGMDWTFAPTLAVAQDSRWGRTYESYSENPDLVAQYAKAIVTGLQGEHILATAKHFIGD--GGTQGGKDQGNNIDSEIKLRDRHGAGYPPAIEAGVQTVMAS
---FSAWHGQRLHGHQPLLTDVLKDRMGFNGFVIGDWN--GHAQLPGCSTTSCPAAFNAGIDMFMAPDSWRQLHKNTVAQVQSGDIARSRLEDAVRRILRVLVLLKNQD
NLLPLAANQ-TLLVTGDGANIGQQSGGWTLSWQFPNGTSIWAGIRHAVETQAILSTDGSYSQIPDAAIVVFGEGPYAEFRG-DIDTLNLPNTPLQQLQELRAAGIPTVA
VLLSGRPLWVTPE-INAAGAFVAAWLPGSE-GAGVADVLIRQPDFKGKLPFSWPRTATQTRV--DQG----KNNYEPLFPYGYGLTY
>A0A2R4X4C0_9EURY
-VREKAAQMIQPVIGSHDNVETVGDIVGEIGAGSVLSGGATTDPEALVAGINELQEYAIENSPHGIPFFYGIDGTHGAAYV-DGATALPQRHNMGATRDPDLIERAEEH
TAAMIAATGIHETYAPTIELQRDPRWGRYFEGISESTKVLGDISRARNRALEGHRVTATPKHFAGY--EIPTNGNDRSAVNTSMRDLRETLLPPFEVTLAEGAGMVMVN
---SGSVNGVPAHVSQWLLTDLLRGEYGFEGVILTDWNDLYRLIGIHDKKMAVRAAIAAGVDMAMLGGGNEGRVTYVDELVESGDLTEQRIDASVRRILELLVLLKNEP
PVLPLAGD-EDLLVTGPGVDILMQYGGWTLGWPRPAGDSMIAALRDHHDGDDQRSAVESAAPEADAVVVVIGEGP--HNEGFDRDSLELPETQREIIATVEAATTPIVA
VEYAGSPRGNQES-FGPLDAVLYAGQPATGGGTAIAETLLGAYNPSGRLGFSWPQQVGHGPL--HHNA--WPNGHDPTYPYGHGLSY
>A0A373NUB2_9FIRM
--------------------TTEREILQEMKYQNIIE---NMTLKEKAAFLSGKSEQTREFPRLDIPAIFCSDGPNGVRKQAGAATCFPTAATMANSWDEELEQKVGVA
LGEESMEEEVNVLLGPGLNIKRNPLCGRNFEYFSEDPYLSGKMAAAFIRGVQSTGAAACAKHFAVN--SQELRRMAMNAVV-DERTLREIYLTGFEIAIEGNAKTVMSS
---YNEVNGVYANENEHLLKDILRDEWGFDGSVITDWG---ASNDH-------SLGVKNGSTLEMPTPGL-DAARELIASVESGKISEKDIDERVDELL---VLLKNEG
GILPVGEKV-QAAVIGDFAFEPRYQGAGSSMVNS----TKVDSIKDMLESEAMKKEAVDLARRSDVVLFFFGLNEKSETEGLDRKHLRIPQNQINLIQELAKANANMIG
IISAGSVIEM-PW-HHHFKALLHTALMGQAGAGAVLDILSGKINPSGKLAETYIKKYEDTPSNYYPSQRNYRFDTAGVYPFGYGLSY
>A0A316W4J3_9BASI
---RVSQFPAAITTSATWDRSLFYARGEA
MGRQFKAKGVDMALSPVTGIGRSPLSGRIFEGFGPDVYLHGAASYETVVGLQKNGLMACSKHWIAYEQETFRNQYNYSVFPKNEQTTHQLYMWPFAEAVRAGSAAVMCS
---YNEVNGSHACGNAQILNGLLKTELGFAGPVVSDWG---GTWDNI-------NSWPNGLDVTMPGSAYDGQGSGLIDAVKNGTIPMDRVKDAAVRLLT-ITLLKNVK
RGLPLPNELSSIAILGQDSSGTLPAGGGSGWANPSYVIDLYASMQHHARSAADLTNFRNQANVSEVAVVSVSAW---TSEGYDRANLTLSGEGEELIKAAAEMNNNTIV
VIHAPGPLLVEDWDHPNVTAVLFAYYPGEAGGSSIPPILFGDKSPSGKLPFTIGKALEDYPKSIVDDHDPFEANHTPRYAFGHGLSY
>A0A2N5Y2F0_9GAMM
-LEQKIGQMMQ-----GEIKTITPKEVSRFGIGSVLNGGGSIDDWIDLAEAYYQSSPTLAD-GTRIPAIWGTDAVHGHNNVMGA-TLFPHNIGLGAAGSSDLVEAISRA
TAIEVKATGIDWVFAPTVAVAKDFRWGRTYESYSSDPQLVSRLGVAAVKGFEAEGIVATAKHFIGD--GATQRGRDQGDVRSDLETLLEGHGAGYVSTIKAGVPTIMAS
---FNSWNGRKVHGSRQLLTEVLRDQLGFGGMVVSDWNGVGQV--AGCNNASCAQAVNAGIDMLMTPTDWRLLQRRVVSQVESGEIPMARIDEAVRRVLA-LVLLKNNG
GILPIKGN-AKVVIAGTGADLSMQSGGWTLTWDFPGATSIYTGFNDALAAMGGEALAADTSQDADVALVVFGETPYAEGQG-DIENLQYKHVDLELMRAFKAKDIPVVA
VFITGRPLWMNRE-LNVADAFVVAWLPGSE-GAGLADVLVGNVDFVGRLPFAWPALDVNEID----DS---LPVDRFVWPVGYGLTY
>A0A6S6FXH3_9SPHN
-LDQKIAQVIMPDT-----ASFSAADMAQYRFGSYLSGGNPPARWLALADANDAAMAARADGRTPIPTLWAVDALHGHNNIVGA-TLFPHNIGLGATRDPELVRAIGAA
TAAEIAVTGIDWTFAPTLAVVRDPRWGRTYESYGERPDVVASMAGAMVEGLQGTKVIATAKHFLGD---GGTGGKDQGNTVASEADLRRIHAAGYIPAIAADVQTVMAS
---FSSVNGVKQHGDKAHLTDFLRGPVGFNGLVVGDWN--GHGQVPGCSVESCAASFNAGLDIFMVPEAWKALLASTTAQVKSSEISMARLDEAVRRILRVLVLLKNNG
NLLPLKPA-QRVLVAGDGADIAMQAGGWTISWDFPRAQSIYDGIAETVRATAMLSSDGSFTAKPDVAIVVFGEKPYAMGD-VDTLEFHDDYKSYKTMQRLRAAGVPVVA
VFLSGRPMWVNPE-INAADAFVAAFLPGGE-GGGIADMLFAKPDFRGTLSFTWPNGPM--PPKLDTP-----RDPGTLFPFGYGLR-
>Q6C1I2_YARLI
---RFADFNVFPCQNAMAATFDRILVHQRGTA
IGRQSRLKGVDVHLGPVVGLGRHATGGRNWEGFSPDPYLSGKLAFEAILGVQEEGVLATIKHFIGNEQEHAEEWKDAVSSNIEDRALHELYMWPFADAVRANVGSVMCS
---YNYVNGTQACQNSDLLNGKLKSELGFQGFVMSDWF---------AQGSGVSNAL-AGMDMSMPGNDVDELGEQLTRMVANGTLPEARLDDMVLRILT-VVLLFND-
GILPL-KNISAIGVFGVGSRGALIEGWGSGTAYPTEYESPYEALHKKASLSWDMRLPLELAGDTDVNIVYVLAN---SGESTDRRNVSLWHNGDELINTVASQGQT-VV
VVTTVGQVDMTAWNHPNISAVLLTAPAGDYGGKAIADVLFGEVNPSGKLPYTIAANTSDYPITKIPRD-GYDFERTPLYEFGYGLSY
>A0A1X7EQQ9_CELCE
TLEEKLAQLVGYWLDQGQQDGGRLAEITRHGMGHYTRGTRPVDPAERAAWLWAEQRRLQRETRLGIPAIVHEECLTGLA-AWQ-AATYPTPLAWGASFDPELVEEMARE
VGESMRALGVHQGLAPVLDVVRDPRWGRVDECVGEDPYLVGTVGTAYVRGLQDAGVHATLKHFVAY--SGSHAGRNHAPVSAGPREVADVYLPPFEMAVDGGARSVMAS
---YVDIDGIPLHASEEYLTEVLRERWGFDGVVVADYFGVAFLEVMQRRGEAAAQALQAGLDVELPTGDAY--LEPLAERVRSGALDEAWVDRAVLRLL--LVLLAND-
GVLPLRPRADRIAVVGPNADSEALMGCYSFVNH----HTVLESLRDALAGVSGIPAAVDAARGADVAVVVVGDEAGLVGEGNDVESLELPGVQRRLVEEVVATGTPVVL
VLLTGRPYAIGWAAPARPAAVLQGFFPGEEGGRAVADVLLGAVNPSGRLPVTLPRAAGAQPYSYLQPVD-VTADPTPVRPFGFGLSY
>A0A0A0B4Q4_9CELL
TLDDKIGQMTQAE-----RAVVTPADMTTYRLGSVLSGGGSPNTTAGWADLYDRLQRGALATPLQIPMLYGIDAVHGNNNALGA-TLFPHNIGLGATRDPQLAQDVARA
VAEEVSATGVDWTFAPCLCVVRDDRWGRTYESFGEDPALVSSMAT-TVTGFQGESVLATAKHFIGD--GGTVGGDDQGDARITEAELRAVHLPPFEAAIDRGVGSVMVS
---YSSWNGVKMHGNRYLLTDVLKGELGFDGFVVSDWAAIDQLDGARGTAQEVATSINAGIDMVMVPNDYRTFLTHLRQNVQSGAIPVARVDDAVRRILT--VVLKNT-
GVLPLARTGGKVCVAGKNAH---DIGNQSGGWTLSWQGTILQGVQEVLAGTTHARDASNVDASCSVAVAVLGETPYAEGEG-DRGGLSLDAADTAVLERIRSTGVPTVV
VLVSGRPLDVSVL-LPWVQALDVAWLPGSE-GAGVADVLFGKVAPTGTLPVSWPASATQQPVNAG-------DGRTPLFPLGAGLTW
>A0A0F4GVV8_9PEZI
--PRVGFPGMCLHDAGNGVRDTDG-VNAYASAVHIGASWNSTLAYQRARF
MGAEFKRKGVNVALGPVVGIGRIAQGGRNWEGFGADAYLDGILGAQSVKGLQE-SVIASIKHWIANEQETNDGTILSSSSNLDDRTMHELYMWPFQDALYAGVGSVMCS
---YNRINGTYACENSKAMNSLLKGELNFQGFVVSDWV----------AQHSGIESANGGLDMAMPLSSYW-DDDQLAKAVQGNELNETRLTDMATRIV---VLVKNKD
NALPL-GKLRLMSIFGYDAVGTLIVGGGSGSNSPAYISAPYDALQNRAYQYDFHSSAPGVVAGSEACLVFINEY---ASEAWDRPGLADG-ESDELVKNVAAQCNNTVV
VIHNAGVRLVDAWDNDNVTAVIFGHLPGQDSGRSVVQILYGDVSPSGRLPYTVAKSPSDYGKPCKDDS-QLARGVTPRFEFGYGLTY
>A0A0J8GN22_9ALTE
TLEQKVAQMIQPEL-----RFVSVEDMRKYGFGSILNGGGNVKDWVDLADSFYLAALDDSQDGIAIPSIWGTDGVHGHNNVYGA-TLFPHNIGLGAANNPELIKKVAEV
IAKEVAETGIGWNFSPTVAVARDDRWGRAYESFSEDPAIVKSYAGKYVEGMQGEKVVSTAKHFLGDG-GTVEGVDQGNNID-SEAELFRLHAQGYVTAIEAGVQTVMAS
---FNTWHGKKMHGNKYLLTDVLKEKMGFDGLVVGDWN--GHGQVKGCSNDKCAQAINAGVDILMVPEDWKALYFNTIAQVQSGEIALSRIDDAVTRILRVLVLLKNNH

NILPLKPNL-NVLVAGDGADIGKQNGGWTLTWDFPNATSIYHGIKQTLEAQANLSLDASYTEKPDVAIVVFGENPYAQGDIKDG-NLEYQKTDLKLLRKLKSEGIPVVS
VFLTGRPLWVNKE-LNASDAFVAAWLPGTE-GGSIADVLFSELDFKGKLSFSWPAKPDQTILNVND------QNYKPLFAYGYGLDY
>A0A0K6GG86_9AGAM
--RLNIPAFCLQDGPAGVRP-ADFASQFPAQVTVAATWDRELIYERAAA
IADEFKGKGVHVALAPVTGLGRSPLGGRNWEGFSADPYLSSIGSYITVKGIQDRGVVATSKHYSLYEQETYRNQRLFPYSDVDDATFHETYLLSFVEAVRAGTGSIMCS
---YNRVNGSHACEDDYTLNQILKGELDYQGYVMSDWY---------AQWTNVGAAL-GGMDMTMPGTGFW--GTDLVALVNNGTVPVNRLDDMVHRIL---TLLKNVR
GGIPL-KKSKFVAVFGQDAGGTNSVGGGSGAAYAPYIITPLEGIQSRTAADWDLETARTNALIADTSIVFTYAY---QTESLDRDNLTAWSNGDALIKTVAAECNNTIV
VIHSGQQILMDDWEHPNITAVIFAYYPGQETGNAIASILYGEVNPSGKLPFTIAKSASDYPPIFTENVDPYRFDAKNIFEFGFGLS-
>A0A1Y2DWN1_9FUNG
---IKAMLISKPEIPEGTEILHQYTTAIPIGTAIAQSWNRKFAELCGDI
VGSEMELFKVQLWLAPALNIHRSILCGRNFEYYSEDPLISGAFAASITKGVQKHNTFVTIKHYAAN---NQETNRYLNSSNVSERALREIYLKGFEIAVESQPKAVMTS
---YNLINGVHTSESKELTNDILRNEFGFEGIVMTDWVGLGGNHKYSG--PTPYNVIKATGDIYMPGSKAD--YEAVLKALKEGTLSIEELEMSASRIYN--ILLRKN-
GDFPIGKSTKQVYLYGNGVRKTIKGGTGSGDVYS----RSFDTIEQAFTKMPEPDYDIPYAKEGDLAIYVLARI---SGEGSDRGDVYLTETERKMILDLAKGFKKFML
VLNTGGAVDLSGI-D-EVENILLLSQLGVNTSKTLVDIITGDKYPSGKLTTTWTKY-EDYPSNFGDNDDTYRFDTENIYPFGFGLGY
>A0A2A2W855_9BACT
TLRQKVGQVIQAD-----IGSIQPDDLKTYPLGSILNGGNTAKDWLLLADAFYNASRECDLQGGPIPLLWGTDAVHGHNNIVGA-TYFPHNIGLGATRNPALLRKIGEI
TAREIRVTGQEWTFAPTIAVVRDDRWGRTYEGYAEHPEVTASYTGELIEGIQGVHVIATAKHFVGD--GGTTGGKDQGNNQMSEEDLRDLQAVGYPVAINHGVQCVMAS
---FSEWQGKRLHGHKGLLTDVLKGRMGFDGFIVGDWN--AHAQVPGCTATDALPVLDAGLDMYMAADSWRGLFETMLRQAESGDVDMARLDDAVRRILRVLVLLKNNG
GVLPLRPNA-TIVIAGDGAD---NIGKQSGGWTLSWQGSIYEGIRRQVRAGDGKPVADSSAMKPDAAIIVFGEDPYAEFQG-DVETLAYKDKDYRLLKSYSDRGVPTVA
VFLTGRPLWVNRE-INASDAFVAAWLPGSE-GIGVADVLLTDRDFKGKLSYSWPRTPMQTPLNVGQ------AEYDPLFPYGYGLTY
>A0A5C6EBX2_9BACT
TMDEKTAQMGTIYGHKTHPTADWKNRVWKDGIANIDEHCNMTGHREHADLLNLIQRWFIEETRLGIPVDFTNEGIRGLCH-TR-ASNFPSQLGIGATWDRDLVRHIGEI
TGKEAKALGYSNIYSPILDVVRDPRWGRTIECYGESPFLVGELGTQQTLGLQSQGVGSTVKHFAAY--STPHGGRDGRARQIPFRDMQEILLHPFQKVFLAHPKGAMSS
---YNTYDGIPVTGSRYFLTELLRGDYGFKGYVVSDSGAVSRLHEQHEFEDAIAQAVNAGMNVRTTFKRAEDFILPLRKVVKDGRISEDTINSRVGDVLRVMVLLKNE-
DVLPVPKTCGTIFVTGPAADDVIPMISRYGPGTS-DVITPLAGIKAFLGEQAKLDKAIEAAKSADIIIAVMGDNDDTVGESRSRTDLNLPGHQDLLVQEMVKTGKPVIV
VLMIGRAASINWI-DRNADGILVSWHGGEKVGQAVAETIFGANNPGGKLPITFPKTVGQIPLPHR--NKQTTRVLGPLYYFGYGLSY
>G4U891_NEUT9
--FSYATSFANAINLGASFDDDLVYEVGTA
ISTEARAFGLD-YWTPNVNPYKDPRWGRGAETPGEDPLHIKGYVKAMLAGLEGNKVIATCKHYAAY-DLERWHGLTRYEFEVTLQDLSEYYLPPFQQCADSKVGSIMCS
---YNALTIRPACANTYLM-TILRDHWNHNNYITSDCNILDFLPDNHNPAEAAAAAYKAGTDT--VCEVSGSPLTDVVGAYNQSLLPEAVIDTALRRLY--IVLLKNSG
SLLPLDFSGKKVALIGHWANTGTMRGPYSGIPPF--YHNPLYAAQQLNLSDTWTAPALAAAEGADVVLYFGGTDTTVASEDLDRESIAWPEAQMKLLSELAGLGKP-VV
VIQLGDQVDDSSLNNGNVSSILWVGYPGQSGGTAVFDVLTGKKAPAGRLPVTQYPQARSTPGNKTLSSRTYKWYSTPVLPFGYGLHY
>A0A1U8N6R3_GOSHI
TLEEKIGQLVQAD-----IASVTPDDVRRYRLGSVLNGGNPPAEWLALADAFYDASMDRRNGGVGIPILWGTDAVHGHSNIVGA-TLFPHNVGLGAMRDPALMRRIAEA
TAVEIRVTGQEWTFAPTITVVPQDLRWGRAYEGYSSDPELVASYVGEFIRGLQGEHVIASTKHFLAD--GGTTDGRDQGDARMSEEELRDIHGRPYVEAIEAGVETVMVS
---FSSWNGVKMTGHRGLLTDVLKDRMGFGGFLVSDWN--AHGQVEGCTNARCPQAANAGLDMYMAPDSWRQLHASLLEDARAGTIPAGRVDDAVRNILRVLVLLKNEG
SVLPIRPGT-RILVAGDAADVARQSGGWTLTWMFPGATSIWSGIEQAATAAVLSPDGRFSGARPDVAIVVFGETPYAEFQG-DIPNLLLRTGPLDTIRRLKAQGIPVVA
VMLTGRPLWTNEW-LNAADGFVVAWLPGSE-GGGIADLLLAGRDFTGRLPFAWPATANAK--------------GPTLYPLGHGLSY
>A0A316YAZ4_9BASI
--RLGLPALCLQDGPTGVRSSLNV-SQFPAEVSAAATWDVELIAERGRA
IGQEFLDRGAAFMFGPVTGLGRSPLGGRNWEGFGSDEYLSGQASYASVRASQETGIVACSKHWLAYEQETSRNHLIFQPISSDVDDLATHQLYSFAEAVRAGTGMVMCS
---YNRVNGSHACESDRLLNGLLKHELNFQGSVVSDYG----------AAYSGVDSVNGGLDLLMPGAGLFGVGSKLADAVRDGKVAEARLDDMVIRILT-VTLLKNSN
NTLPLVNRISKLAVIGQDAVGTRTNGEGSGFTTPPYVIDPLSAIRAYTLETLFSTDPAKAAEDADVALVFVSAL---GQEGQDRSDLKLDRNGDELIKRVAKVNNNTVV
VVHAPGPVLMEEW-IDSVAAVLFAYYPGQESGASLTPVLFGDESPSGKLPFVIGKSLDDWPPNTIVNRYTF------DFAFGHGLSY
>W5IHG2_SCAIO
-LPEKIGQMMQFD-----ARSDLENAIENKHAGSILHT-----SPEDLPR----ANALVQKTRLRIPLLVGDDLIHGYS--FYPGTIFPEQLGMACSWDPEKIKAAARV
TAREAVTTGVQWTFSPVLCIARDTRWGRVGETFGEDPYLIGEMAAAMVKGYQGDAILATAKHFAGY--SETQGGRDASEADLSHRKLASWFLPPFERVAREGVATFMLG
---YESIDGIPVTLNRWLLHDVLRGQWGYKGTLITDWANVSRLVWEQHPVSAAVAAVRAGNDLIMTSPEFY---QAAYQAIDQGLLAESELDQPVKRILA--VLLRNN-
GLLPLHNSPARIALIGPLIDAQTQLGDWAGSSPRETITTIQDGLQKLVPQSATLEEALKNARESDCIVAVVGDLIQLMGEGCSTATLELQGAQNHLLDALKETGKPLVV
VLMSSKPQVL-PASALTADALLWAPSPGMKGGQAIAEILLGLTNPSGRLPITFPRHAGQLPV-YYNQIQHYALTEDPAFAFGEGLGY
>A0A2G3DFA6_CAPCH
TLEEKIGQMTQIE-----RKVASAEVMKKYFIGSLLSGGGN-ATAEDWVNMVNEFQKAALSSRLGIPMIYGIDAVHGHNNVYKA-TIFPHNVGLGVTRQ-----RIGAA
TALEVRATGIPYAFSPCIAICRNPRWGRCYESYSEDLNIVRSM-TEIIPGLQGDKVAACAKHFVAD--GGTVNGIDENNTVIDSKGLFSIHMPAYYDSIIKGVATVMAS
---YSSLNGVRMHANTHLLTGFLKKRLNFRGFVISDWEGINRITSPPNYTYSVEASVMAGVDMIMVPENYTDFIGNLTYLVNKNAIPMSRIDDAVKRILRVLVLLKNGK
PLLPLPKKARKILVAGAHGDLGYQCGGWTITWNLTIGTTILKAIKNTVDPNEEPDTNFVKSNNFDYAIVVVGEPT--YAEVYDSSNLTILDPGPSNIQNICGSVKC-VV
VVISGRPVVIEPY-VEKMDALVAAWLPGTE-GQGVADVLFGDYGFTGKLARNWFKRVDQLPMNVGDP------HYDPLFPIGFGLT-
>A0A1V4IRS6_9CLOT
TLEEKIGQMYQASGTDNSEVIPIEKLIQEGKTGSLIFI------GNNPGKIFYLQKLAVEKSRLGIPLMFAQDVIHGYQ------TVFPIPLAWSCSFNPELIKEAVRI
SAKEASAVGLMYAFSPMVDISRDPRWGRVSEGAGEDPFLGAQIAKAQVEGYQGKSMLACLKHFIGY--AAAEGGREYNTVEISEATLRNTYVPPFKAGIDAGAASVMNS
---FNVINGIPVAGNKAILKDLLRGELGFKGITISDFAAVDEIYIHGAKKDAAMKAVTATMDIEMITTSFS---TYLPELVKEGKVSEEIVNDAVRRILS-IVLLKNN-
GILPLKKN-SKIALIGPCANSKDMLGPWQFSDKGDETVTILEGLTSKEVEENGFEEALAACKKADIVILALGESSKMSGEAASRQNINLPEVQLELANKIKELGKPIIL
LLTNGRPLIIKWF-DDNMDGIIETWFLGSQAGNAIADVIVGDYNPSGRLTMTFPLNQGQIPI-YYNSFNTYIGPNTPLYPFGYGLSY
>A9V0V7_MONBE
---AVERLGIPFVWGSECVTGLGTDGNDPTAFPQPLGMAATFDPALLKRAAGT
IALELRAQGVVSCWSPVVNINRHPLWGRNDETFGECPVLSSFMARSFVEGIQGNAAAAACKHLDVY--GGPDNLRYVFDADVSQADLTGTFLMAFEECAAAGVMGYMCS
---YNSIRGVPACANYRTMTFFAREQWGFEGYVVSDQGAVFRITESHNQTLGAVAALNAGCDMEDSDDAQHVAYYNLSLALDLKLTDMATIDASVSRLF--IVLLKNQN
ETLPLAAKNASYCLLGPFADADLMMGKYSPHGSTNVTVTYRAGLAAALQNLDTAAVTTFIQQGCDTVLLAVGTSYHVESESLDRSNMSFPGAQPTLVQTVLEALQRLVL
LVSTAGPVDLAALQDTRVAAILDLIYLGQTAGTALADILLGETSPSGRLPFSWPNKVSDVPPDDYTMQRTYRAQADVLFPFGYGLSY
>A9VCI2_MONBE
---ATQLRARESAQIDN--IGLPAYWGTNAIHGMQNTAADGTSFPAPNGLSATFNYSLVKDMGRI
IGRELRAYGLD-TWSPTINPSRDPRWGRNVESPGESPFVCGQYGAAYTEGLQNGQAVVTLKHWVAYSVEDYDNVTRYEYNAISEYDLMDTYFPGWEYVVNAKPLGVMCS
---YNSLNGVPTCGNPA-LTAYLREDWGFEGYITSDSDSIHCIWADHHYESNAVLALLGGCDIDSGDTYA----DNLEAAVNQSLVNRSAVDAALT-----MTLLKNDG
QTLPFATG-KKVAVIGKSSNAEDILGNYVGPIAFDCVQTLYQGVAAANQGVADINTAIQLAMDADQVVLTISNY-GQAGEGKDRTYIGLDTDQQELVAAVLKVGKPTAI
VMLNGGLISLDWI-KDEAQAILVAFAPGVHGGQAVAETIFGANNPGGKLPVTMYASDYVN-DDFLNMSQAYKYTGEPLYPFAYGLSY
>J9A271_9PROT
TLEEKIGQVIQAD-----IASVTPAEVKEYNLGSILNGGNAWQDWVALADAYWLASTDTSDGGLGIPAIWGTDAVHGHNN-LQSATIFPHNIGLGATGDTDLLERIGAV
TAHEVRATGLDWVFAPTVAVARDYRWGRTYESYSENPQLVSDLGAALVLGLQGKKTIATAKHFVGD--GGTQYGIDKGDTIVTEQELRDIHAYPYKQAFKNDVQTVMAS
---FSSVNGTKMHESKTYLTGLLRDEMNFKGFVIGDWN--GHAEIPGCTATNCPDALLAGVDMYMAPESWKGIYESLKSQVESGAVPMARLDEAVLRILQ-LVLLKNNN
NTLPVKPGS-NVLVVGAAANMKDQTGGWTLSWEFETGETIYEGLETAITQKPDTEPDIETDMAPDIAIVVFGEEPYAEFHG-DRMDLIYEDPNLAILKQLKAKGIPVIS
VFISGRPLWVNSH-INLSDSFVAAWLPGTEAG-GIADVLIADADFVGKLPFAWPVGATGELV---------RKDAGTTFQFGYGLNY
>Q1YRG2_9GAMM
-VEQKVGQMIQPE-----IKFISPSEVKEYHIGSILNGGGAIQDWIDLADDYYNASVDLSNGGTGIPVIWGTDAVHGHNNVIGA-TLFPHNIGLGAANDPQLLRQIGEV
TAKEVAATGIDWVFAPTVAVVKDLRWGRTYEGYSSDAALVKAYAGEIVRGIQGEKVVATAKHWIGD--GGTYRGMDQGNTILEFDQLLELHGQGYLSALDADVQSVMVS
---FNSWNGRKIHGHKELLTDVLKGQLGFDGLVVSDWDGVGQV--EGCTTESCPLAINAGIDLIMVPKGWKNLISNTLAQVQSGVIPMARIDDAVTRILR-LVLLKNKN

QLLPLKGE-QHILVTGDGADIGKQNGGWTITWDFPGATSIYTGLKQAVGSSVELSADDSWVKKPDVAVVVFGEEPYAEGVG-DVESLMYRRADLDLLQSLKGKNIPVVA
VFLTGRPLWVNAE-INSSDAFVVAWLPGSE-GVGIADVLVADKDFTGRLSFDWPNRELNA---------------------------
>A0A1K1NI46_RUMFL
TLEQKANQMVQPAV-----YNITTDDMKANDYGSILSTVGCINSDAWCETVDGFQNAAIE-SEAGIPYIYGQDDVHGVN-YCRDAVYFPHNIGQGAANDEELAYQVGLI
TADEAKLCHMLWNFSPCVAQSVDPRWGRTYESYGSDLDTITKLSTAYTKGLQDGGLVACAKHFFADGNGTGEEGTPLKRIDRGDAQLTSAELKVYQAQIDAGVQTIMIS
---HSSLNGVKMHENKEYI-MKLKDEMGFEGFIVSDWGSIEHTSG-ATYKEQVINGVNAGIDMLMETDKYDEAKQIIVDAVGSGDITEERVNDAVTRIIKVLVLLKNDN
KVLPIKEG-TKVYITGPAANGQVQCGGWTMDWDVPGVTTIREAFERYAESGIEVITDKEEAEKADVVLLCLGEQAYANGDTEDMGALGL-DGNSKARQEAKELGKPTVA
CIVAGRQVLINPRIYDQWDSVVMCYLPGSE-GKGISDVLCGCADFKGKLPAPWYGSLDQIGT-------------------------
>H3NUN5_9GAMM
TIEQKVGQMIQ-----GEIRDVTPEDVRVYGLGSVLNGGGTVEDWVALADAYYTASMDTSSGGAGIPIVWGTDAVHGHNNVMGA-TLFPHNIGLGATRDTKLVSQIIGA
TAREVKATGIDWIFAPTVAVAKDARWGRTYESFSSDPQIAASFVAPIVDAMQAEGIASTAKHFIGD--GGTLRGDDRGETSLPLEELVAIHGQGYVEAIDKDVMSVMSS
---FNSWYGDKIHGSKAILTDLLRGDMGFEGMVVSDWNGVGE--VLGCTNDNCAQAVNAGIDMVMVPADWRSLYNNMLEQVAAGEISEARIDEAVSRIL---VLLKNNQ
GLLPLPADQ-HYLVTGPGAD---DIGQQSGGWDFPGATSILGGLKQQL-EAAGGSITTDESAEVDAAIFVFGETPYAQGDVYSV-AWYDQRAERSRMKALKERGIPVVA
VFLTGRPMWVNDI-LNLSDAFVVSWLPGSE-GQGVADVLLQDADFTGRLPMPWPALDVNA----IDRD---LPVDTFAFPIGYGLS-
>A0A5N6KX38_9ROSI
---RLGMRSLCMQDSPVGVR--FGDFSVFPAGGTIAASWDRELIYRRAND
MGSEHRDKGVDVQLGPVVGIGRSPAGGRNWEGFSPDPYLSGVAVAESVKGIQDAGVMACTKHFIGNEQVGEANGYGYNITNIDDRTMHELYLWPFADAVRAGTASIMCA
---YTNTNNSQSCQNSYLLNHLLKGELGFQGFVMSDWQ---------AQHSGVAGAL-AGLEMAMPGDTLFNTGTNLTIAVLNETVPEWRIDDMATRIM--TVLLKNVR
NTLPLPAKEKFTGVFGEDAGGTLGMAWGSGSANFPYLVTPLTAIENEVSKNWATDQITSLASQASLALVFVNSD---SGEGYDRQNLTLWQNGETLVKNVSALCNNTVV
VIHSTGPVLLDSFDNDNITAIVWAGVPGQESGNSIADILYGRVNPGGKLPFTLGRTRRDYGSLLYKPNDAYRFDKRNVYEFGYGLSY
>A0A316VLP4_9BASI
---VP-RLGIPSFCLSDGPTGIS-VTYNVSQFASPITLAATWSPQLASQHGLA
LGQEFHDSGVNFIFAPVAGLGRSPLAGRFWESLGADEYLTGVLGSQIVKALQEDNVVACTKHFIGY-EQESDRNMNTPQTQKPYSTMHQLYMWSFAETARAGTGQVMCS
---YNRVNGTQQCQNDASLNGLLKHELNFRGNVVSDYG------AAYSNE----EATNNGLDLLMPGEGQFGIGEKLKDAVNSGKVKIERIDDIVKRILT-ITMVKNER
NVLPLKAKLNTIAILGSDAHGTQTDGQGSAFAYPPYIINPLDAIKSYVKAADRASRAVDLARKADVAIVFGNAR---GKETEDRSNLTLREHADDLIKRVAAVNKQTIV
VLHTPGPVLVEEWDHPNVTSVLFAYYPGQESGASLTPVLFGDESPSGRLPFVIGKKLDDWPNSISDPHHPFDKGIKPRYPFGHGLSY
>A0A0Q7SVZ9_9CAUL
-VEEKVGQTLQPE-----MKSIVPDDVRRYHIGSIENGGGSVGDWLKMIDGYYDASVDPANGGVRIPMMWASDAVHGHNNVYRA-TLFPHNIGLGAARDPDLVRRIGAA
TAQEVRALGMDWSFAPTLAVVQDDRWGRTYESYSEDPKIVAAYAAAMVRGLQGEHVISTAKHFLGD--GGTDGGRDQGDNLADEATLRDVHGAGYPAAVDGGVQAVMAS
---FSSWHGVKMHANKDLMTGVLKDRIGFDGLIIGDWN--AHGQIPGCTKGDCPTAFNAGIDVFNVPEDWKALYAAMVREVNDGTIPMARLDDAVRRVLRVLVLLKNDG
GVLPLDPR-KRILVAGDGADIMKQTGGWTLSWDFPGATSIWGGIEAAVKATALLSPDGAYDAKPDAAIVVFGENPYAQGDQADVALHAGSAESLALLKRLKAAGVPTVA
VLISGRPLYLNPQ-INAADAFVAAWLPGSE-GQGVADVLIAGPDFTGKLSFSWPKRPDQTPLNVGQK------AYDPQFAFGYGLTY
>A0A5K7XJN4_9BACT
TLDEKIGQMCQVWPEQGELTPALVESLRQGEVGSLIN-------CPDSKFIVEVQRIAQTESRLGIPLLIGRDVVHGYR------TIFPIPLGQAASWNPELVEQAARI
AANEASSEGINWTFAPMVDVGRDPRWGRIAETFGEDPRLSAALSSAVVHGFQQEGLVACAKHFAAY--GLSEGGRDYNRASLSIADLHNIHLPAFKSSLDVGCRTFMTT
---FSEVNGIPGTAHAYLLQDVLCDSWKFSGVVVSDWNSVIEMVAHGFEAEAAQQAVNAGVHMEMVSPTFH---DNLSRLVEQGRVDEAALDNAVRRVLRLIVLLKNAE
QTLPLREELRRVAVIGPLADPLSQLGCWSVDGSPADAITPLAAIRNAVGDLTELAKAQKAAEQADVVLLFVGEDAVLSGEARSRMTLDLPGAQAKLVEAVAASGTPVAM
VVLAGRPLAIGAE-IDAAAAVLYAWHPGTMGGPAIVDLLLGDAAPTGRLPVTMPKHVGQVPL-YYSHSNTYVGDPFPLFPFGYGLTY
>A0A7J7GYW5_CAMSI
TLAEKIGQMVQVD-----RSVATDEVMRNYSIGSVLSGGGLHASAKDWVKMVNEFQKGSLSSRLGIPMIYGIDAVHGHNNVYEA-TIFPHNIGLGATRDPELLRRIGDA
TALEVRATGIPYVFAPCIAVCRDPRWGRCYESYSEDPKIVREMT-DIILGLQGEKVAACAKHFVGD--GGTTNGVNENNTVIDMHGLLSIHMPAYTDSISKGVSTIMVS
---YSSWNGEKMHANGNLITRFLKDSLKFKGFVISDWEGIDRITTPPHYTYSVQAAILAGIDMVMVPYNYTEFIDDLTKLVKHKVIPMDRIDDAVSRIL--LVLLKNGK
PLLPLPKKAPRILVAGSSADLGYQCGGWTITWNYTTGTTILNAIASTVDKLENPDVDYVKSGKFSYAIVVVGEYPYAETAG-DSPTLTIADPGPTVIGNVCGAVKC-VV
VIISGRPLVIEPY-ISSIDALVAAWLPGTE-GQGVTDVLFGDYGFTGKLSRTWFKTVDQLPMNIGD------SHYDPLFPFGFGLT-
>C7Q9F7_CATAD
-LPEKAGQMDQQLVDNLPTPACMQSALIDQNVGSILAGGTN-TAQDWANDYNTIQQYAIAHSRLHIPLSFGVDAVHGFGHPWQA-PLFPQSIGMGATWDPSQAKAGGAM
TATALRSTGWTWAFAPVQDLARDNRWGRTYETWAEEPALSSAMGAANVTGLQTPDVSATVKHFAGY--SESVNGHDRDEALLPLNYLQSTILPSYAGAINAGADAVMVD
---SGSINGVPATSSHYLLTDILRGQMGFKGVEISDYQDVQALQTTYHLPDAVALAVNAGLDMSMEVNGPDQWQSAIIQDVGNGKIRMSTINDAVRRILT-ITLLRNQN
SVLPLPAGS-RVVVTGPSADMTNQLGGWSVSWT---VQTGVLGADTHATAISDQAAAVAAAPNTDAYVAVVGEKAYAEGLG-DNPAPALPADQQALISALEATGKPVIV
VVEAGRPVALGSA--EKASAVVMAYQGSTEAGQAVADVLFGKTDPSGHLSISWPSDAPAVGGDFNSTADEGPNAYNPLYPFGYGLSY
>R1D6Z6_EMIHU
TVEEKIAQLGY-NIRPCATM-DVAKDY-ANGVGGCAV--------SSIADTIKMRKALQKLTRLGIPPSIHGETTHSGG--AANTTVFPMPCLQGATFNTTLVEEIAAS
NALQLRAAGGDMGLSPILQVCTDPRFGRLEENFAEDPYLVGAYGVAAVRGLQGRRVAAQGKHYAMYGAAGKDGYTPFGG-GIAARTLFDVYLRPWREFARAGGRGVMAA
---HNMVDWVPMHANRPLLHDALRVRFGEGGYIGSDNTNVEGLADYFHTSDAAAMALAAGIDQDMPGGAFL---SGLLPLVASGRVPMAHVDRACGNVLR--VLLINRN
AALPLSASSARVALVGPFAAIQAMVGGYAWVPPPRAVVTVGDALRRRGVEDFEAAVALARAPSTDAVIAVLGTTSCGNGEAGDRMSLELEGRQLELLAALVRAAAPVIV
VLIHGRPVG---DLLLGVDALLAAWRPGEEGGSAIVNLLFGDANPSGKLAQAWQRSAGFIPTWFQPHS-SFGGDGVPLFPFGHGLSY
>A0A1Y6CA28_9ALTE
TLRQKIGQMVQA-----QMDAITPEQVRDYHIGSVLNGGNSTQEWVTMVDTYYRASMDSSNERVAIPIMWGTDAVHGQSKIVGA-TIFPHNIGLGASRNGALVRKTAEI
TAIEMLISGVDWNFSPSVAVVRDLRWGRTYESYSENPQLVKELSEDYVLGLQGCRVMATAKHFIGD--GGTEQGIDRGNNIDSEAELYRVHAPGYLSAIKAGVLSIMPS
---HSMWQGERLHGHHYLLTQVLKKSLGFDGFLIGDWN--SHGLLDGCKDSSSPLAVNAGLDMFMVTHEWQAFIENTATQVEAGQISEARINDAVSRILR--VLLKNND
ALLPLNPRL-NFLVAGDGADIGKQAGGWTISWDFPKGTSILSAISKEVNKTVHYDVAGTYTKKPDVAIVAFGEEPYAEWFG-DIKHLAYQNKDARLLEKLKKAGIPVVA
LFISGRPLWVNRI-INASDAFVAVWLPGTE-ANGISDLLFTNTDFTGKLPFSWPKSPLHNQL---NSDH---KDYDPLFPFGYGLSY
>A0A1V6Q0R0_9EURO
---ATSFPSPILSMAALNRTLIHQIGDI
ISTQGRALGLDV-YAPNINTFRHPVWGRGQETPGEDVLLASAYAYEYITGIQGGKLVATAKHFAGYDIENWGGHSRLNDMDITQQDLAEYYTPQFVTAVDARVHSVMSS
---YNAVNGVPSSVNSFLLQTLLRDTWGFDGYVSSDCDAVYNVFNPHEVSAASADSLRAGTDIDCGTSYQY----YFNESIAQGEISRSDIERGVIRLY--IVLLKND-
GALPLAKNTSSVALIGPWANTQGLIGNYFDPAHAPYLTTPLAALQKSSLDTDGFGAAIAAAKQSDVIVFAGGIDNTVEAEGMDRMNITWPGNQLDLIKELSQLGKPLIV
LQMGGGQVDSSSLTNKNVNALVWGGYPGQSGGLALLDIITGKRAPAGRLTVTQYPAKYALQFPATNMDDNYKYTGKPVYEFGHGLFY
>A0A2X0L6M6_9BASI
---STDGSAFPAAINVAATWDKDLMYMRGAA
MGSEFRGSGVNVALSPMMNLARTPEAGRNWEGFGGDPYLAGFGAAMTIKGIQSTGVIACAKHYIANEHYRGGSGAITSSSNIDDRVMHEVYNHPFSESIRAGVGAIMCS
---YNRINQTHACENSYLMNKLAKQDLAFHGFVVSDWAA----------QTSGVSSALAGLDMSMPGFRSYTEGAELVKAVNNGSVPLARVKDAVTR-----ILLKNVA
NALPLAGQLKSVGIFGADAGGTLAMGWGSGTVNFPYLVDPLSAIRYKLGQNNATSAIQRLARLVEKCLVFVASD---SGEGYDRNDLELWQNGNQLISKVASLCSDVIV
VIHSVGAIDMESWDNKNITAVVLAGLPGQESGNSLVDVLFGDVNPSGRLPYTIGKKRSDYAAVLYKSSVPYRFDAKSIFEFGFGLSY
>A0A507FDU6_9FUNG
---FPGLCLQDSPTGIR-FARNVSAFPASLNVASTFDQGLMYNNGRF
MGEELRDKGVNVHLSPVANIMRTPQGGRGWEAQGADPFLTGVSVTAQINGIQSNGVQATLKHYVLN--DQEKFRQDRGDVIIDKKTLMDYYIRPFKMAIHSGPASIMCS
---YNSVNGVNACDNPVLM-TILKEELGFKGYVMSDWWAT----------HTELDGLKAGMDMVMPGSKACCDGETLVSNIKNATVKMDRVDDFATRILT--ILLKNS-
GVLPLSAKSGNLALIGSDAIGTLAQGWGSGTTNFPYLVAPQEGITAAASPNWDIPAVEAAAAAAGTSIVFVNAN---SGEGYDRKNLTLWNNGDRLVEAAAAKGP-TVV
VIHAPGAVDMPWINLPNVKAVIMALLPGQESGNAIADVLFGAVNPSGRLPFTIGHNITDYHTDMINPI-QNQNNIAPLFAFGHGLSY
>A0A0D2MXV5_9CHLO
---KQQAVPRLGLKAFFLTECLHGLWETTKQPTIFPQPLVMASTFDRLLVKRVFDA
VSTEMRAKGVSTCWTPHMNILRDPRWGRGSETWGEDPHLSSVMAAHVVRGLQGEKVAATCKHFYGYSFEMADGQSRYSFNRIDPVDAADTYLPAFDACIAARTQGVMCA
---YNAVDGVPMCANKPKLQGLLRDKWGFDGYVVSDCNAVQGLVWGHKEPAAIAQAIKAGTDLLCDNMDS---QKAASQALSQGLLTEADIDSALNH----IVLLKNEP

RMLPLKDKLKKVCVVGPLADAEHMMGNYYGAWDK-GASTPLAAIKAELAGDWPLESALQSCDGADAALVFLGAS--IEGEGFDREGLGLPGRQLDLVKALARRSTPIAL
VVMNGGPVDLSWAGSPSVAAIMAAGFPGQEGGRAVSDLLFGRASPSGRLPNTWYRA-------------AYRFVEDPKYPFGHGLSY
>A0A453D9Y6_AEGTS
---GATSFPQPILTAASFNASLFRAIGEV
VSTEARAMGLATFWSPNINIFRDPRWGRGQETPGEDPLLASKYAVGYVKGLQDAKVAACCKHYTAY-DVDNWKGVERYTFDVSQQDLDDTFQPPFKSCVDGNVASVMCS
---YNKVNGKPTCADKDLLEGVIRGDWKLNGYIVSDCDSVDVLYTQQHPEEAAAITIKSGLDLN--CGNFL--AQHTVAAVQAGELSEEDVDRAI------IVLLKNN-
GALPLAKSIKSMAVIGPNANSFTMIGNYEGTPCK--YTTPLQGLGASVNTSLQLSTAVAAAASADVTVLVVGADQSIERESLDRTSLLLPGQQTQLVSAVANASGPVIL
VVMSGGPFDISFAASDKISAILWVGYPGEAGGAALADIIFGSHNPS---
>A0A2A2K1B1_9BILA
-IEDKVGQLLQVD-----IASITPGDLETYKLGSILNGGNPAAEWLKLFDAFYDASVKRSDGRPVIPVIWGTDAVHGANNIVGA-TLFPHNIGLGAMRDPQLIRKIGAA
TAAETAATGIDWSFAPTVAVVQDDRWGRTYESYSEDPAVVASYAGEMVQGIQGTHVISSVKHFLGD---GGTGGRDQGDTRVSEAVLRDVHAAGYTTALPAGALTVMPS
---FSSWNGEKMTGNKSLLTGVLKERWGFDGFTIGDWN--AHGQVPGCTNEDCPAAINAGLDMFMYSGPGWKQYDNTLREAKSGAIPADRLDDAVRRILRVLVLLKNNG
GVLPIKPSA-SILVAGRGADIGMQSGGWSITWDFPNGQSIYAGIAQAARASATLATDGRYTRKPDVAVVVFGETPYAEFTG-DRATLEYSKSDLALLKRLRAAGIPTVA
VFLSGRPMWVNPE-INAADAFVAAFLPGTE-GGVADVLLAQADFRGKLSFSWPRRLDQYVL---NRRD---PGYDPLFPFGYGLTY
>A0A1Y0G0X0_9GAMM
TLEEKVAQLQAVWLKRGVFTPAKAKDILGSGIGQIARPAENKTPEQTIAFVNAAQRWLIENTRLGIPVIFHEEALHGHA--GRDATSFPQAIAMASTWDTQLVESIYKV
SAQEIRRRGGTQALTPILDVARDPRWGRIEETMGEDPYLVAALGVAGVKGFQGDRVIATLKHLAGH--GEPVGGLNTAPAPVGERLLREVFLPPFEAAVLGGARSVMAS
---YNEIDGVPSHANGKLLNDILRGEWGFEGALVSDYFAIKELVTRHQLEDAALLALNAGVDVEMPDGETF---PLLVQAVNDGKLGEAVIDQAVARVL--MVLLKNDN
NLLPLSRKVKSIALIGPHVD-EVLLGGYSDVPEH--AVSILQGLQEYLGEAGMIKKAVAAAKKSDVAIVVVGDNEATSREAWDRTSLELVGEQQALVDAVLATGKPTVV
LLINGRPLSISKI-AREAPAIIEGWYLGQETGHAVARVLFGDVNPGGKLPVSIPRSVGHIPA-YYNHKKRYATETSALYPFGHGLSY
>A0A2P2EA49_9PROT
-VEQKVAQLIMPD-----ISTITPEDVAKYRFGTILNGGNKPEEWLKLADAFWASRTPHADGSPVIPVLWATDAVHGHNNVVGA-TIFPHNIGLGATRDPALIREIGRI
TAAEIKVTGIDWTFAPTLAVVRDDRWGRTYEGYGEDKQLVSDLGAAMVEGLQGVRVIATAKHFFGD---GGTGGVDTGDTVGKEADLVALHAFPYRAALQSGAQTVMAS
---FSSINGQKMHGSKSYLTELLKVEMGFDGLVVGDWN--GHGRIPGCTNSDCPESINAGLDIFMVPEDWKALLTNTKAAVDSGKISQARLDDAVRRILRVLVLLKNE-
GVLPIDPR-RTILVAGEGADIAKQSGGWTITWDFPGAQSIFAGIAQQAEAKAILSVDGSYTQKPDVAIVVFGEKPYAEFMG-DVKNLSYTAKPLTLLKKFQAEKIPTVA
VFLTGRPLWVNKE-LNAADAFVAAWLPGSE-GGGVADVLLKTKDFKGKLSFSWPQACDTYSL---NPG---EAGYKPLFEYGFGLNY
>A0A165JBY4_9BASI
--ASAFPAGINVAATWDKQLMYDRGFA
MGAEHRGKGVNMIFGPMTNLGRVAAGGRNWEGFGADPYLAGVATAQTVLGIQDNNVIACVKHFYGHYRGGGSSSEQIYSSNIDDRTAHELYIWPFAEGIRAGAASIMCS
---YNKVNQTQNCQNSKLLNGIIKEELDFQGFIVSDAAAARSGV---------DTAL-AGLDMNIPSFYYFGTGSNLITAVMNGSVPEARVDDMVTR----IVMLKNT-
GVLPVVTKYRRYGIFGSDAGGTLAMGWGSGSANYPYLIDPSMAITWYVYSDYDYASINSVASQADICLAFVNAD---SGEEYDRNNLTLWHSGEAVINASTSYCSNTIV
VVHSVGPVLLEEWDNPNVTAVLWAGLPGQETGNSLVDVLFGAVSPSGRLPYTLARQRGDYPAVMYESNTTYRFDAQGLYEFGYGLSY
>A0A518K9A0_9BACT
-LREKIGQMCQVSSFGTDLPKEVADDLRGGRIGSLFY-------TGTPEQTREAKRVAMEESRLGLPLLTPRDVIHGFE------TVFPIPLGQAASWNPELIEAASAI
AADEARAQGVNWTFAPMLDISRDARWGRIAESVGEDPLLASAISKAMVRGFQQPGIAACAKHFAAY--GLTEGGRDYNRAEVSISELHNAILPPFRAATEAACATFMTG
---FSAVNGVPVTGHRELITDLLKGRWDFEGLVVSDWTSVIEMIEHGYRREAARLAVSAGLDMEMASTTFR---ENLVDLVESGEIEESQIDDAVLRIIRV-VLLKNDG
VALPLTDKLKRVAVIGPLADARDQLGCWMLDGKPEDAVTLLSAMKETLGDTKGFDGALDAAEGADVAIVCVGEGWMLSGEARSRADLHLPGSQRALVQAIAETGTPVVL
VVMAGRPLTIGAE-AELADAVLYAWHPGTMGGPAIAELLVGDESPSGKLPATFPKQVGQCPL-YYNHPNTYIVDPFPLYPFGFGLSY
>A8AEA3_CITK8
TVDEKIGQLRLISVGPDNPKEAIREMIKNGQVGAIFN-------TVTRQDIRKMQDQVMELSRLKIPLFFAYDVLHGQR------TVFPISLGLASSFNLEAVKTVGRV
SAYEAADDGLNMTWAPMVDVSRDPRWGRASEGFGEDTYLTAIMGKTMVEAMQGKSVMTSVKHFAAY--GAVEGGKEYNTVDMSPQRLFNDYMPPYKAGLDAGSGAVMVA
---LNSLNGTPATSDSWLLKEVLRDEWGFKGITVSDHGAIKELIKHGTPEDAVRVALKSGINMSMSDEYYS---KYLPGLVKSGKVTMAELDDAARHVLNVLVLLKNRL
ETLPLNKSG-TIAVVGPLADQRDVMGSWSAAGVASQSVTVLTGIKNALGDEAMIDEAVNAAKQSDVVVAVVGEAQGMAHEASSRTDIQIPQSQRDLIAALKATGKPLVL
VLMNGRPLALVKE-DQQADAILETWFAGTEGGNAIADVLFGDYNPSGKLPMSFPRSVGQIPV-YYSHLRPYFEANGPLYPFGYGLSY
>A0A255Z468_9PROT
TLEEKVAQMIGIWQDKGGFDAKRAAQAFPNGLGQISRPGDNREGLEAATYANDAQKWAVEQTRLGIPLIFHEEALHGFVGRGA--TSFPQSIALASSWDTAMVERIFAI
AAREARLRGVHLALAPVVDIVRDPRWGRTEETYGEDPWLVSEMGLAAMRGFQGTKVLVTLKHMTGH--GQPESGTNVGPAQISERTLRENFLVPFERAVNLPIRAIMPS
---YNEVDGVPSHGNKWLLDTVLRQEWGYQGALVSDYFAIREMKTVHQLADGAVRALNAGVDVELPDGEAY---PLLPGLVREGKVSEAQIDTAVRRILR-MILLKNAN
NALPLPNATGTLLVVGTHAR-DTPIGGYSEVPRK--VVSVVEGLTAAAGKDVLIAEAVAKAKADTILMVLGENEQLSREAWDRASLELYGRQSDLAEAMFKTGKKVII
LLLNGRPLSVNRL-VEKADALIEGWYLGQETGNGVADVVFGKVSPGGKLPITIPRSVGQLPV-FYNAKRRYLDDITPLFPFGFGLTY
>A0A2G5C560_AQUCA
--TIRAATSFPTVLLTAASFNETLWKAVGEA
ISDEARAMGLSTFWSPNVNVATDPRWGRIQETAGEDPFLVSRYGVNYVRGLQDVKVAACCKYYTPYDMWTADPTKKTDRAQVPDRDMLETYNYPFEMCVEGDVASIMCS
---YNRVNGIPTCADPKLLNSTVRGLWGLNGYIVDDCDVVATVLIPERPADAISQAFRAGVDLDCRGNYSP---HYMMDAITQGKLSEAEIDHAL------IVLLLND-
NSLPLPLNYSTVAVVGPHANTTAMLGNYALKTTACRYVTPLDAIQGYANVTSGFQEAAKATDGTDATFLFLGLDLSYEDEDSDRQNLYLPKNQIALLDAVFKEPYPLVI
VVLSGGGVDLSDW-GSKVHAILWAGYPGAEGGQAIADILFGKHDPAGRLPISWYKGDYTNPM--TSMQRPYKTDDGIVFPFGFGMSY
>A0A257JQE1_9PROT
TLAEKVAQIIQAD-----IASITPEEAKQHKIGAILNGGNSAKEW-----LALVDSFWDEASDSKIPIIFGTDAVHGHSNIAGA-TIFPHNINLGAARDRDLAKAIGVA
TAKEIAVTGMDWTFSPTVAVAKDLRWGRTYEAFSCAPSLVSDLGAAMIIGLQGVKVGCAAKHFLGD--GATQDGRDQGETNCSESQLISQHAAPYIAAIEAGATAIMAS
---FSSWGGQGMHGRHDILTTLLKEHWEFDGLVAGDWN--GHGQLPGASPNNAPQALAAGLDMYMAPDSWRALLASTLELVT-GDLNQARLDDAVLRMLR--VLLKNEN
QLLPLSANQ-HVLVCGRGADLDLACGGWTLSWLFPGSQTLLEGLKANIIPQVTFDPKGQWQDRPDIAIVVLGEEPYAEFRG-DCDTLAYRPSDYQLIERLAAAGIAVIT
VLFSGRPLWVNPA-LNKSAAFIAAFLPGTQAG-ALAELIVRSPDFQGRLPFPWPAFSDHFD--LCAT----SPNRSPLFPLGFGLS-
>A0A517MQR8_9BACT
-IEEKIGQLQQIHPEGDLLAEKVKETIATGGAGSLFF-------TGTEQLVHDAQRVAVEDSRLGIPLIIARDVIHGFR------TVFPIPIGQASSWNPELVEQAAKV
AANEASRVGIHWTFAPMVDIARDPRWGRIAESCGEDAVLASALGVAMVKGFQQPGIAACPKHYVAY--GRAEGGRDYNRVMVSRNELRNVYLKPFKGCIDAGAITIMSA
---FNTVNGVPATGHERLLREVLKDEWGFDGFVVSDWDSVKEMISHGYKRQAATQAMLAGLDMEMVSNCYQ---ENLQQLVEEKTIDIAMVDDAVSRIL--VVLLKND-
GVLPLKAALKKVAVIGPLADPQDQLGTWTMDGLASDSRTSWAALQEMLHEPEEIATAVEAAKAADVVLLFVGEEEVLSGEAHSRSELSLPGSQGKLVHALAELETPVVM
IVQAGRPLTIGKE-IEAVDAVLYAWQQGTMAGPALADLLLGVVAPSGKLPVTFPKAVGQIPL-YYNHMRTYISDPYPLFPFGFGLTY
>A0A4V3SID7_9PEZI
--LCLQDGPLGIR--FADNTAFPAGITVGATWNRELMRKRGEL
HALEAKAKGVNVLLGPSMPIGRTPLGGRNWEGFGSDPYLQGVAAAETIKGIQGAGVIATAKHFILNEQEHFKSRDEALSSNVDDRTIHELYLWPFADAVRAGVGAVMCS
---YNRVNQTYACGNSDILNGLLKSELGFQGFVMSDWF----------AQRTGFHSVLSGLDMDMPGEVTIFEASNLTTSVMNSSIPLERLDDMATRIV--VVLVKNID
AILPLSLKKAKLGIYGEDAGGSLAMGWGSGAVELETFSSPQSAIHKELPNNTRLEDISKSAEEQDICIVFINSA---AGEGFDRIHLYPQKNGDELVKTVAESCEAAIV
ILHTVGPVILDRWSHPSVKALLIAHLPGQESGSSLTPLLFGDISPSGKLPYTIAKQPADYGKAILEENAFDYNITPQYEFGFGLSY
>A0A5E7Y705_9SPHN
--EESVAYINAVQRWAREDTRLGIPVMAHEESLHGLA--ALDATSFPQAIGLASTWNPDLVREVNDL
IAGETRARGIFQVLSPVVDVARDPRWGRIEETFGEDPFLVGEMGVAAVEGLQGVQVMATLKHMTGH--GQPESGTNVGPAQISERTLREAFFPPFLEVIRTGIDAVMAS
---YNEIDGIPSHSNVWLLDDVLRQEWGYQGTVVSDYFAIEEMVTRHRNAGAAEFAINAGVDIDFPDGAGF---TNLAALLADGSITEAQIDAAVRRILT--ILLQND-
GTLPLP-AASTIAVIGPNAA-VARLGGYYGIPRA--TVSPLEGIQALVGDRRMIAAAVEAARGADTIVLFIGDTEQTSREGWDRTSLDLVGEQNELVAALADLGIPLVT
VLVNGRPPSY-PLVAERSNAVLETWYAGEQQGNAIADALFGIVNPGGKLPVTVARDVGQVPV-FYNHKRRYLAEVSPLYPFGHGLSY
>A0A062U1S8_9PROT
---QKIGQILQAD-----IASITPEEVRAHHIGSVLNGGNSPSEWLALADAFWEASMHTDG--PQIPIMWGTDAVHGHNNLAGA-TIFPHNINLGATRDADLVERIGSA
TAMEVRVTGMDWTFAPTLAVTRDDRWGRSYESYSECPNLVAELGAAMVRGLQGGHVIATAKHFIGD--GGTQDGKDQGETIATEEELRDVHAAGYYTAIREGILSIMAS
---FSSWKGQKMHGHEEFLTRLLKEHWGFDGLVVGDWN--GHGQIPGTSTTNCPDALNAGLDMYMASDSWKGLHASLLQQLQKGVVNEDRLDDAVRRVLRL-VLLKNAE

STLPLSPGQ-NILVAGKAADLSMLCGGWTLSWDYPQAETLLQGIKRLAEANVEYAPDGAYRESPDVAIYVFGEKPYAEFRG-DLTTLDFKRTDLQTIQKLKQSGIPVIT
VFLSGRPLWINPE-LNSSDAFIAAFLPGSQSG-AIADLIFQDSDFTAKLPFSWPEHADQYLL-NHGD-----ADYLPLFPYGFGLS-
>A0A176VUR7_MARPO
--SGATSFPQPILTASSFNMTRVPQAIST
EGRAMYNLGQSTFWAPNINIFRDPRWGRGQETPGEDPLLTSIYASTFVRAMQEIKTSVCCKHFTAY-DVENWDGIDRYHAEVSDQDLEDTYNRPFQSCVEGQASSMMCS
---YNRVNGVPTCANYNLLTETARNTWGLNGYITSDCDAVELIYSAVNAEDAVAEVLMAGLDLNCGSTAA----NFGQSAVDQGKVNESTIDRAL------IVLLKNDN
NVLPFRGHIQKLALTGPNANTSSMLGNYAGVPCE--YITPLQGLQTYVDLGGLIGAAAAIASSADAVVIVAGIDQTQEQETLDRTSLLLPGQQQSLIETVANSSKPVIL
VIMSGGAVDISFAFNPQIHGILWVGYPGQGGGQALADILFGHRNPGGRLPVTWYPSMTDMNMPN-----TYRYTGETVYSFGDGLSY
>A0A0G9MSF5_9SPHN
-LERKIAQLIQPQ-----INSFTPEDMERYRFGSYLNGGNPASEWLRYADEMYDASVRLPNGEPVIPTMWGTDAVHGHTNVVGA-TIFPHNIGLGATGDADLVRRIGHA
TAIEIEVTGIDWNFSPTVAVAQDDRWGRTYESYSEDPDIVARMGAALVEGLQGNRVIATAKHFFGD--GGTEQGVDQGDVNGDIDELLAVHGRPYPAAIDAGVESVMAS
---FNSINGRKMHGNEMLLTDVLRGQMGFDGLVVGDWN--GHGQVQGCTVTDCPQSLLAGLDIYMVPDDWRGLMENLIAQVNDGTIPMARVDEAVTRVLRV-VILTND-
GILPLAPGM-DVLVAGSAAD---DIGQASGGWIFPGATSIWDGLEMAVTTTATLSEDGSFTTRPDVAVVVFGERPYAEFAG-DRPDMAFREEGLNLLRGFREQGIPTVA
VFLSGRPMWMNRE-MNAANAFVASWLPGSE-GAGVADILTGAVQATGRLSFSWPNDCIGRPL---------NQAEGALFAVGYGLS-
>A0A1Q3ESS5_LENED
--RFNIPEFCFNDGPAGVRDVEG-VSQFPAELTTAATWDRDLMYRRTKA
MGQEFYDQGVHIPLAPVSGLGRSPLEGRAWEGSFTDPYACGEASYQYVTGLLDSGVASVAKHWILYGQTKSDGGTQLPVSNVDDKTMHEVYMWAFAEAVRAGTTHFMCS
---YNEVNNTHSCSNAKSLNGLLKTELNFQGPIMSDWG----------AHWDTVPSMYNGMDISMPGSQLNSLWANLTELVNNGTVKEDVVREKVIRILT-VTLLKNT-
GGLPL-KNPSRVAVLGNDATGTLTIGFGAGSGYSPYIVTPLDALKARAIQATATAAIQALLPLSDVTLVFVNRW---YGRGMDAESLNLTGDADDLVAQAVNSSSNVVV
VIHATGAVNIEKWDNPNVTAIIAAYLPGQESGAGLVPVLYGDVSPSGKIPWTWGKSLDDYPPLVYSPQ-SYRFDKNNIYEFGHGLSY
>A0A5N8W024_9ACTN
---DLVQRHVIASSRLGIPALFVEEVPHGHM--ALDGTVLPVNLAVGATWDPDLYRRAAAH
AAAELRARGGHMALVSALDIARDPRWGRTEECFGEDPYLAARLTEALVHGMQGEKAPVVLKHFAGQ--GATVGGRNSAESELGLRELHEIHLPAARAGVRAGAAAVMAA
---YNEVDGMPCCGNRALLHGLLRERWGFEGLVMADGLAVDRLARITGKVSAGALALHAGVDLSLWDEGFT----HLEEAVGRGLVGEDAVDAAVARVLSLVTLLHDDG
DVLPVRPTVSRIAVLGPHAATAHQLGDYTAPQRPGTGTSVLEGLRRLAPPLSDIPEAVAAASAADLAVLVLGGSSARCGEGVDLAGLRLGRAQHALLDAVTATGTPTVV
VLLQGRPHAVPEA-ADRAAALLTAWYPGPWGGDAIAEVLFGHAEPGGRLPVSVPRSVGQLPV-HYNHKDTYAESARPLYSFGHGLTY
>J5KAL2_9GAMM
TLEQKVGQVIMPD-----IDDVTPQEAKDYFLGSILNGGGSIDDWKQLSQEFYNASPIVNE--KIIPILWGTDAVHGHNNVIGA-TIFPHNIGLGAANNTDLMEKIGSA
VAKEVLSTGIPWTFAPTIAVPQDSRWGRTYEGFSEDPQIVSDLGEATIIGLQGFKILATAKHFVGD--GGTDKGVDQGNTITSEFDLKETHGFPYYAAIDACVQTIMAS
---FNSWNGDKMHGSSYLLNDVLRDQMGFKGLVVGDWN--GHGQLPSCTNKSCPEAFNAGVDIFMVPQDWKELYKNTLDDVKNGTISTARLDQAVKRILQVLVLLKNNN
KTLPI-KSNKHILIIGQASKIKYQMGGWTVSWDYPNTKSIFEELSDSLASTSEYSIDGSYKKKPDAVIFVYGEQPYAEGDG-DRENFFYMPEDKNLINTMNASETPTIS
LFLSGRPLIVNEE-LNASDAFVSLWLPGTA-IEGISDVLLSNKDFVGKLSYTWPKFNNAEKK-----------NDINLFNFGYGLTY
>A0A4Y8UPN5_9GAMM
-TEQKVAQMIQAE-----IKYITPEQVVEFGIGSILNGGGTVQDWVELADAFHAASLDRSQGSAGIPIIWGTDAVHGHNNVIGA-TIFPHNIGLGAANNPRLVADIAAA
TAREVAATGIDWIFAPTVAIAKDDRWGRTYEAYSDRSDIVGRYAGPVVRAMQQQNIAATAKHFIGD--GGTFRGIDQGDTRLSLEQLLLEHGIGYSEAIAADVLTVMAS
---FNSWNGEKLHGSYDLITTILKEGLGFDGFVVSDWNGIGQI--PGCSNESCAQAINAGIDMIMVPEDWQPFLTNTLAQIERGEIAMSRIDDAVYRILSVLVLLKNSN
GLVPLSPQQ-NVLVTGLADDIGVASGGWTISWDFPGGTSIYAGLAAQIEATASFSANGDYTTKPDVAIVVFGEQPYAEGQG-DIDNLQFDAGSKQGLAQLRAEGIPTVA
VFLSGRPMWVNAE-INAADAFVAAWLPGTE-GAGVADLLLRDRNFTGRLSFDWPNSDVNASD----D----NPVDDILFPYGYGL--
>A0A316UCK7_9BASI
---LGISPLCLQDGPTGVRPA-RRTSQFPQEVTMAAAWDRDLAAERAQG
IAEEMHDKGVNFWLGPVTGLGRSALAGRNFEGFSPDSYLSGENSFASVRAAQEVGVVTVAKHFIAYEQETFASGENYQRVQLDDRATHEAYMWSFAEAVRAGTGSVMCS
---YNRVNDTHSCSNSYAMNELLKGELGFQGSVVSDWG---------GTWSTIHSAL-AGLDLTMPGQGYGGIDGSLIDGVQNGAVPESRLDDMVLRIL--VTLLKNNR
YGLPLKKDISSIAILGEDSGGTLSAGGGSGWSYPPYVSDLLAAVSFEARSSYNLAGAAAQAAKSEVAMVSVNAY---AREGSDRQNLTLFGGGDALIEAVAAVNNNTIV
VIHAPGPVLLEEWDHPNITSVLFAYYPGQEAGNSLIPVLWGDVSPSGRLPFTIAKSADDYPDRNYSLD-PYKFERRGVYHFGHGLSY
>A0A420XZJ0_9PEZI
---YSSSFPHIVNLGYTFDDELMTAVGNA
LSQEGRAFGL-TFFSPNVNPIRDPRWGRRLEVVSEDAFYTGNYAKAMVSGLQGGRVVATCKHVFGY-DQENNAGNRYQFANISTQDTAEYFMTPFRACAEANVGAFMCS
---YNAVNGVPSCANSYILDDVIRGHFNEDQMVFSDCDAIQNVYSPHLRPAAAADALKAGVDNNCGEYYQV----HLPEAYAQGLISDKDIDTTLVR-----AMLKND-
GTAPVDLKGKQVALIGSWANTALLQGNYFGPPPF--LISPLAAAMNVSGITSGLGKTIKTANMSDIIIYIDGLDPSDETEEYDRNTLSWTGYQLDLIGNLAELGKPMIV
VRMGGGQLDDSPLKNPNISALFTAGYPGQSGGQAIIDVLTGVAAPAGRLPETQYPSSYLNTVRMSDMSRTYRYTGEPILPFGYGLHY
>A0A4Q1BWH6_TREME
--RLGIPGLCFNDGPSGPR--YTDFTQFPSPFTAAASFDRDLIEERAIR
IGGEFVGKGINVELGPITGLGRSPFAGRNWEGFSPDPYLSSTMSFLTVRGMQSSGLITCAKHYFLYEQPVVDGGRHDVSSEVDDKTVHELYLPSFAEAVRAGTGSVMCS
---YNRINGTASCQNDDSMNRLLKDELNFQGFDFGAAH------------STVPSAL-AGMDMELPGEFFCKSGRRLYDAVLLGEVPTSRLDDMAQRILT-TVMLKNS-
GVLPL-NGVKRIGIFGTDADGTVTIGGGSGAAYADYIAAPLEAISLRARRPAHFGAMSWIASQSEVCLVFVSLF---LVESWDRENLRLDKGGEELIQHVEGSCGEVVV
VLHIGGQVVMEDWDLPNIGAVLFAGYPGQETGNAIVDVLWGDVNPSAKLPFTIGRSTKD----------IYKFDKWSIFEFGFGMSY
>A0A2V1BG70_9HELO
--VNRAHLVSIFPSGITIAASWDREMMFDRGLA
LGREFKAKGAHVILGPTTGLGRNPLGGRNWEGFSPDPYLSGEAIKFTVIGHQTAGVQTSSKHYIANEQETMRSDTKMPVVDIDDRTLHELYLWPFADALKAGTTSIMCS
---YNRLNGTYACENEHMLTEVLRGELGFRGFVVSDWFA----------THSTAKAANAGLDMELPPGVTTSHGKSLEAAVTPGNVTEERLTEMAVRLM--TVLLKNEN
DLLPLGDKTSNIGVFGNAAFGVLTIGGGAGSGRNSYVVSPLEAIKEQAKQIIARNDFRGIYPRPDVCLVFLKSW---AAETYDRTSFELSYNSTLVVNNVAKLCQGTIV
ITNSGGTNTMPWSNNPNVTAILATHYPGQEVGNSIVDVLWGKTEPSGRLPYTVPKAAADYPVNLTGPDERYRFDAKNIYPFGFGLGY
>K6FF82_9GAMM
-IEEKVGQVIQG-----DLDFITPADVKKFKIGSVLNGGNSLSDWKKLSQEFYDASPTYKG--IKIPVLWGTDAVHGHNNVIGA-TLFPHNIGLGATRNIELVQKIGEA
IALEVLSTGVAWTFAPTIAVPQDDRWGRTYEGFSEDPILVSKLGKALILGLQGEHVLATAKHFMGD--GGTTNGVDQGNTKISELGLRELHGYPYFDALDACAQTVMAS
---FNSWNGEKLHGSKSLLTGVLKNDMQFDGFVVGDWNG--HGQVEDCSNSKCAQSFNAGVDMFMVPENWKDLLRNTIRQVNSGEISELRLDEAVKNIL--LVLLKNND
GVLPLMPNQ-HIGVIGDASNISTQTGGWTITWDFVNTRTIYESIKNYVEVSVEFSSNGKFQNKPDVVIGVFGEEPYALGDLKDVAFTATDPRYLPLLESISATNIPTIS
IFLSGRPLVVNEY-LNASNAFVAAWLPGTA-VEGISDVIFMKNDFQGKLSYSWPRSKDQSVLNFTDSI------YDPLFPYGYGLTY
>G3J632_CORMM
---LKFPGLCLNDAGNGVR-ATDNVSGFASGIGVGASWNKDLARKRGAA
LGGENRRKGVNVMLGPVVGPAWTVVKGRNWEGSSADPYLSGVLSAETVRGVQSQGALTLLQHYIGNEQRAPEGDIAAVSSNIDDKTMHELYLWPFVDLVKAGAANVMCS
---YNRLNQTYACGNSKTLNGLLKTELGFQGFVVSDWG------AQHSFSDA-----PNGLDMGMPNSDPF-WGANLVNAVKNGTLAESRVTDMAMRII---VLVKNDH
NTLPL-KAPKLVSVFGYSANGTMFGGGGSGAITPQTHVSPQDALVARAAKLTSATPTTTVDPASDVCLVFGNAW---AREGNDRPQLEDA-YTDSLVRAVADRCARTVV
VLHNAGPRLVGGFDDPNVTAILFAHLPGQQAGDAVVALLWGDANPSGKLPYTVARSAADYPLDPQAQGKSYKFERAHVYEFGFGLSY
>A0A0D2C7G2_9EURO
--KEIKRLGIPAIRVSDGPNGIRGMFNGSACLPCGTALAATWDMDMIRRGGEL
QAREAIAKGVSVILGPTINTQRSPMGGRSFESFSEDPVLAGATAAATIQGIQSKGLSTVIKHFVCN-DQEHERMKQD--SRVSARALREIYMLPFQIALLSKPWAFMSS
---YNRVNGIHASEDPWLLGDVLRGEWGFDGLVMSDWT--------GTY--STAEAIKAGLDLEMPGPSLM-RASLVNHALVCGKLSKDDIDICVRRVLQ---------
---DEEEIRNAVRIAKHVDQVAIIAGLNSDIESEGYDREEFGLPGHADRLIREVAAANPNTAV
VVQSGSPVAM-PW-MQLAPAIVQAWYGGNETGNAIADILFGDVNPAGKLPLTFPVRLEDTPTNFGSERRTYESNRRVLFPFGHGLSY
>A0A1M5YBR7_9GAMM
TLKEKIGQLDLQTSFRPSINENYEQQIKDGHVGAVFNA-------YEPDFNRTLQQMAVEETRLGIPMLFGYDVIHGHK------TIFPQSLGEVASWDLDVAKEGARV
AAREAAADGVMWTFAPMADITRDPRWGRISESAGEDVYLNTLMSVARVEGFQGDTVLATAKHFVGY--GLAQGGRDYHTTDLSEHELWTTQMPPFKAMVDAGVATFMTA
---FNDLNGIPATGSKFLLTDVLRDQWGFEGFVVTDYTAINELVPHGYDKHAAEIAFNAGADMDMVGRVYL---DYMEELLEEGKVSIEQIDTSVRRILE--VLLKNED

QALPLLDSLESIALIGPLADKRDMIGNWAAAGRKTVPVSLKQGLAERLGDDGMIAEAVSAAKQSDVIVLAIGEDQRMSGEASSRVELTLPGNQRELMTELKKLGKPMIG
VVFSGRPNDLSWE-QQNLDAILHAWYPGTAGGHAIADLLVGDYSPSGKLPITFPRSVGQVPI-FYNMKRPYDSPNTPLYAFGHGLSY
>A0A1R1Y0L0_9FUNG
TVEEKIGQMAQLTPDMELNVTAAEYMFNTYKIGSILDSATNTASPQRWANLTNSLQKIALRSGSKIPITWALISIKGAN-FVKSATMFPAPISVAASFNVEVAYNVSRI
TAKDTRAAGIQWALNPLVGINAHRAWSRNFESFGEDPYLVGEMTYSTVRGLQGNRLAATMRDFVGY--SAPVSGKDRQYRHIPDNILMEYFVPPFKRAVEAGVATAANG
---FGSVNGEAVVSSSFQLQHILREVIDFKGVMLVEWEEIHNQVIRHGQTDAVHLTLNTSIDMYMTPFNGSFVT-ETLYLSNNKMLSVDRIDESVGRILQ-IVLLQNNN
DTLPLSLS-ENVLFIGPATNSRYMGGGWNMHFYDGYSDTVVSGVEKITGKVDSIEDIISAAKSADKIVIGLGERT--SAEFPDVNTLKINPDLIKIIKKIHEAKKPIVL
LLIQGRPYLIEDL-PSIADSILNANLPSMYGGLPTAEILYGKASPSGRQPFSYPKADYQNMINYYTYKD----EYDPEFAFGTGIGY
>R9ACK7_WALI9
---ERLGIPELCMMDGPTGVRPVHG-VSSYPVGQAAAATWDRELIYQRSKH
MGQEFFDKGVNVALAPVASLGRSPLMGRNWEGSFADSYSNGIYTYESVTGLQDSHVSATIKHFIGYEQEQTDSSTDQEPINIDDRTMHEVYMPSFAEGVRAGSGMVMCS
---YNNINGTTACGNAHAQNGLLKTELNFEGAIISDWGGVHSDVD----------SIMSGLDGSFPGVGYGGGWEDLPELVKNGSVSESRLDDACHRMLT-VTLLKNN-
GGLPL-NRPQRLAAVGALDGSTITIGGGSGYTISPYEVTPLDAIKEKVIQENDEDVINEISSRADATLVFVSEW---AEEGEDRDDLDIVEEQAKILDTAVKASSNVIL
VLNTPGVVDIEKWDNDNVTAIVETYYPGQESGNGLVPILFGEKSPSGKLPYTWGKKLSDWPET-IVRSDEYKFDKQDIYEFGFGLSY
>S8F7W2_FOMPI
---ERLGIPILCLQDGPAGARPIQG-SSQFPAGQAVAATWDRELFYARGHA
MGQEFFDQGVHIALSPVTGLGRAPRDGRLWEGFFADAYGTGEAAYHTVKGLQDAGTMATAKHLIGYEQETFRDDLQLPISNIDDEATHELYLWGFAEAVRVGVSFVMCS
---YNMVNNTHACANSYTQNHLLKEELNFQGGVVSDWGAVWGTADF----------INSGLDMSMPGSAELDIGDTLVLAVAFGFVEESRVDDAVLRVLT--TLLKNTG
GALPLNE-PGVLVMVGSDAGGTVTLGGGSGWAIPPYVVTPYEAINYRAREDTDLIDVAVTAASAEVAIVFVRAF---RKEGTDISSLSLDNDGELLIDAVTAVCNNTIV
VIHAGGPVLVESCDNPNVTAVVLAHYPGQESGNGLADVLFGDVSPSGKLPYTIAHSADEYPPPVIN---PFSHNTTPRFEFGFGLSY
>A0A433PM31_9FUNG
TLKEKIGQISQLNINTGLNLTAVEYYAKEWGIGSFLNQAQLLSSKEYASWINQINEVYMKYTKHKIPIIYGLDSVRGAH-YIRDATIFPSGISMGATFNPSLVEEANSI
TAFESAMGNVRWSFAPILDLGINKLWPRLYENFGEDPYLTSVMAAAAVHGFQGKKVAACLKHYIGY--GGTRSGQDHDNAWIPINYLLDYHVPAFRAGVEAGAATVMTS
---YSAVNGQPISSSKYFLETLLRKDMGFNGMVVTDWGEGYNVYDHHRRYHAVIDFMNAGIDMSMTPTDTD-FNVWLAQAVTEGKVSIETVNNRVGHVLQLITLLQNNN
TILPA-KNPKKIAVVGTFREKGYQCGGWCRRWQ----ESLIEGVREVYPHTAEWQTAIDYAKAADLTIIGAGEFHYAERVG-RIDDLDLPQGLINFINAVGKVSKASAL
VLFEGRPRTLQDI-PNNVDAILMAYLPGPWGGLPAAEIISGKVNPSGRLPYTYPLANGDFTYRGYDQTYNPDGVQNHVYEFGDGLSY
>V5EIV4_KALBG
--EALCLQDGPAGVR-PARRVSQFPAGVTTAATWDRELFAQRAEA
LGQEFRDKGVNIWLGPVTGLGRAPRGGRNWEGWSPDEFLSGVASYLTVKHAQEQGVVTCAKHYIAYEQETYRNPYNLSEQQSDDRTTHELYLWSFAEAVRAGTGAIMCS
---YNEVNGTHACQDDFSLNYLLKEELYFQGAVISDWG---------GTWTNQESAL-GGLDVSMPGTAYNGMGEALIAAVNNGSVPETRLDDMVLRILT-ITLVKNND
RGLPLLESLQTIAVIGQDAGGTVTIGGGSGWAFPPYVITPSAAIQEYVRANWDLEGATARANSSDVALVFANTY---ATENQDRQNLTLWANADNLIKTVAANNNNTIV
VIHSPGQVDVEQWEHPNVTAVVFAYLPGQEGGASLPKMLWGETSPSGKLPFTIAKNESDYPPNTIVADNPYKFDYHNIFAFGHGLSY
>A0A3M9Y202_9PEZI
--RIADLTVFPAGLTAAATWDRDLIYQRGKA
LGAEFRGKGAQVHLGPASALGRHPLGGRNWESFSVDPFLTGVAMDFSIRGIQEMGVQTSAKHFIGNSNTFTEDGLEVAALNIDDRTMHELYLWPFADAVRAGVASVMCS
---YNRVNQTYVCENSKILNGLLKDELGFQGYVVSDWFATHSIASAAALDMTMPGAMNSAVTAIFPTPSYF--GGNLTAAVLNGTLTEEKVNDMARRVLT-TVLLKNTN
NVLPL-GKPKSVGVFGNGAAGALSVGGGSGAGRHTRLISPLFALRGRIEDMMVEDDFTSIYPTPEVCLVFLKTW---AREGTDRLSFENDWNSTVVVENVARRCNNTIV
VTHSGGVNTMPWADNENVTAILAAHYPGQENGYSISDVLFGDVNPSGRLPYTIPKKESDYPINITGDARDYRFDARNIYEFGFGLSY
>A0A3S9PE37_STRLT
TLDEKIAQMTQIALVKELNERCMAAVLRDRAVGSVLSGGGPVNTPRDARMVDAVQRYAVEHSRLGVPVLYGVDAVHGHNNVLGA-TVLPHQIGLAATWDPELVRACAET
TARAVAATGADWAFAPVSDLARDRRWGRYYETFGEDPLLAGTLAAAAVRGTENANVAATVKHFAGY--SEPSNGHDRVPADVSPRYLQDTLLAPYRAAVEAGAKTVMVN
---SGALNGVPVTASRHLLTTVLREQWGFTGVTVSDWQDVRALWTTYKYPHAAALAVNAGVDMAMEPYEAGEFADALRTAVRAGLVPGRRIDEAAGRVLRL-VLLRNDK
GVLPFGPSVKKIVVTGPHADLAAQAGGWTVGWI--PGTTVLEGIRQAAPKAPAPADAVAQGRDADATVVVVGERPGAEG-GADSPRPELPADQRELVRSLKETGRPVVV
VVLAGRPLVLGDA-D-GTEGLLMSWLPGGEGGHAVADVLFGAAGPSGRLPVSWPRRTGNEPL-YYQQLTNAESGHDVAYDFGDGLSY
>A0A1Y4NMZ2_9FIRM
TLEEKLREMYMHPAGAACFSEEKAETFFQIGIGAVEAP--KCLPRQNAVFINALQKYLKEHTRLGIPALIVSECLHGYM--TPEATVFPQAIGLASTWNKELMRQTASA
IAEEAAACGIRQGLAPDLDLAREPRWGRVEETYGEDPCLCGEMGLAYIKGLQGEKIAATLKHFCAH--GSPEGGVNLSPVPAGERQLRELYLPPFAKAVEGGALSIMPA
---YSEVDGIPCSASEFLLTDLLRDELGFRGYLFSDYGAVEMLNCFHCRAEAAAQAVRAGMDMEAPTGCCF---AHLKELLDDGKIDISHIDQAVRRILRVVVLLKND-
KILPLSGKQRTIAVIGPNAD-EAELGDYSLGNGNSSAVTLLDGIRSHVSAEKAWKDAMDAAQQADVIIAAMGHSSSVCGEGYDCADLRLSGAQEALLEELLALSKPLVL
VLINGRPVTL-PC-IDKIPALLETWYAGEEAGNAIADILFGEVNPSGKLPVTFPKSTGQIPL-YYNYKARYVDDTEPMFPFGYGLSY
>A0A6I5YSQ1_9SPHN
TVEEKVGQLIAQSTLPGVVDPVVAQRSLANGAGAFILFTSPVDAPTGVVTQNAVQSWVVNNTRLGIPMLMQAEALHGVV--VKGATIFPQAIALGSTWNPALVRTMFTA
VADEASAAGFHQVLAPVFDLARDPRYGRVEEMYSEDPYLVTQMGLAAVQGLQGNHVIATAKHLI-H--GQPENGTNVGPSDYSEYTMRDVFLPPFEAAVIGRIGSVMPS
---YNELGGIPSHASSWLMKDVLRGEWGFTGFVNSDWLAVKQLHDKQFYGDAGVLGFNSGLDLETPVPEGF---AALPAAVQSGAVKMADLDAAVGRILT-MILLKNEG
ALLPLPGKVRTIAVIGPNAD-KARLGTYSGT--PPYFVTVLDGIRKRVGPAALIGEAAVVAKSADVVILVLGGNETVSREAFDADTLELPGRQNDLVREIAKLGKPTAA
VILGGRPYTALQV-NESVPAVLEGWYLGQETGNAVAGALFGDVNPSGRLPVTIARNAGQLPV-YYYRKARYVSDNRPLYPFGYGLSY
>C6LHW8_9FIRM
-------------------AERKQMVMTEKKIGELVS-MLTLEEKAGLTSGKDNWFTKAVE-RLGVPQVRTSDGPHGLRTQAGKAVCFPAACATAASFDRDLLYRMGEA
LGRECQSTGVHVLLGPGVNIKRSPLCGRNFEYFSEDPYLAGELGAAFVKGVQSQGVGTSLKHFFAN---SQEHRRMDASSEMDERTMREIYLPAFETVVQAQPWTVMAS
---YNKIGGVYSTANRKYLTDLLRKEWGFEGVVTSDWG----------ATHDRPAAVAAGCDLTMPAEDTD---HLIVEAVRNGTLSEEALDACCIRLLK-VVLLKNED
NILPLAEEA-DVAFIGPFAKEPRYQGGGSSHINSFKVVGALEAAEHRGVRELLLAQAIEKAKQAKIAVVFAGLTDGMESEGVDRRHMRLPEGHNMLIEAVCAANPNTVV
VLHNGSPVEM-PW-VDRPKAILECYLAGQAAGEAVTDVLYGDVNPSGHLPETFPKRLEDNPSLYYFGEVNYEKKQEVLFPFGHGLSY
>A0A1I0R1D1_9FIRM
TLEQKAAQMVM-----GAETDTGFDYMGEYGIGSIYGGGRSMEDWQE---RCDGYMQAAIESDAGIPFIYGQDDVHGVGYCINA-VYFPQNIGLGAANDEDLMYQMGLI
VADETLICHMPWNLYPCVAQSNDPRWGRTYESYGTDLDRITALSTAYTRGLVDGGAVACAKHYFGDGNGESSGGFD-RLIDRGDTDLTDEEIAVYQAQIDAGVQTIMVS
---YTSLNGMKMHENPDYI-WLLKNEMGFEGFIVSDYDGIQGTS-PETYEEQIILGINCGIDLYMEGTRYDEARQIIIDAVNNGDITMERIDDAVTRIIRVLVLLKNDN
DVLPLTEGT-KVYIMGPACDPRVQCGGWTIGWDIEGVTTILEAFERYS--EDYGIEVVTDPEEADVVILCVGERSYAYGDTEDLELCGMMEENRDAIDEAAALGKPTVT
CIVAGRQVIPDQSDYADWDSVVMCYLPGSE-GKGISDVLCGCGEFSGRLPEPWYGSVDQILT---DE----------CFEEGYGLSY
>A0A2K1IG41_PHYPA
---AATSFPLPCLSVCSYNRTLWNKIGQV
VSTEGRAMGRSTYWSPNINIARDPRWGRTQETPGEDPKLSSGYAVHFVKGLQEGKISACCKHFTAH-DLDRDYDRDHFDSKVTQQDLEDTYNPSFKSCVEGQSSSVMCS
---YNRLNGIPMCTHYELLTLTVRNQWGFDGYIVSDCDAVALIHDYINSEDAVSYVMLAGMDLNCGSTTLVHGLAALD-----------------------LVLLKNEK
NALPWKTHGLKLAVIGHHADTREMLGNYEGYPCK--FVSPLQGFAKVLSDQFYIYAAKEAAAQADAVVLVLGISQAQEKEGRDRDSLLLPGRQMELVSSVVSAGRPVVL
VLLSGSPLDVSFADDPRIQSIIWAGYPGQSGGEAIAEAIFGLVNPGGRLAQSWYYENYTNIDSNMNMR-PYRFTDTPLWEFGHGLSY
>A0A099CWP6_9GAMM
TLREKIGQLMQYNDAGTAQCVDAMKLAADGGMGSMLN-------VVGAERTDKFQRAAVEKSRLHIPLLFGADVIHGYR------TIYPVPLGLASSWDPQLVKDLSRM
AASEATTGGVRWFYSPMVDISRDARWGRSVEGAGEDPYLGAAMARAYIHGYQGDHVAASVKHFAAY--GAAEAGREYNTTDMSDIRLRQVYLPPYKAAIEAGSATVMSA
---FNALNGVPATADPYLLTDILRKEWGFNGFVVSDYTAIMELTHHGIPAEASRKAIDAGTDVDMMSHYYD---TQLPTLLKEHKVSMATIDEAVRRVLRV-VLLKNAK
PVLPLSSNTKTVALIGPLSDSNQMTGAWGGAYRQSDVVTVRQALSARMKKTAGFAAARQAAEKADVVVMALGESGDMSGEAGSRAHLDLPGNQQQLLESIVATGKPVVL
LVFSGRPLVLDWA-AKHVPAIMETWFPGVEAGNAIANVLFGDVSPSGKLPMSFPRAVGQEPL-YYNQFGRYIVPNSALYPFGWGLTY
>A0A5C5FPD7_9BASI
---CTADGPTGINSRYS--TQFPAEVTVGATFDHDLIYARAVA
MGKEYHTLGAHVPLSICIGMGRSVYGGRNWETFSPDPYLTGEAARLSVQGFQEQGVVGLVKHFVGNEQEYLRVGTPRGYFPIDEASLHELYSWPFAEAIRAGSGSFMCA
---YNQVNGSFACENDHLLNTVLKKTLNFHGWVITGAS------HWGAGHDTVNSSLH-GTDFIGWAREDGHLGDALAPFVENGTVPIEVVDDKIIRILT--TLLKNVR

HGLPL-HKPRDLLLVGSSAAGYSSDGYGSGGSPAPYNLDPTAAITARGRKNGTNYFLDTKLAYSNAAVVFVTAM---AREGFDRTDLELLNGGSDLIEYVADRHNDTIV
VITAPGPVDMSRFDHKNVTAILYAYFGGQEGATAIASTLFGDVNPSGKLPFTIARNVSDYPDNYYNGSVNFDQEIEPLFEFGFGKSY
>N2AFG4_9FIRM
TIEEKLGQMNQISPSIELIEMMTDGRISKEEFGRMMAGAKSSVLISDPEVANQLQKIAVEETRLGIPLLFGFDVIHGFR------TVVPIALAEAGTFEMDLIEKTGRI
AAKESRAVGIAWHFAPMVDISRDARWGRISEGPGEDPYLASAFAAARVRGLQNDEVAACLKHYAAY--GACESGRDYNTTGMALSMLHNTYLAPFKAAVEEGAVTVMAA
---FNDLNGIPCTVNKYLLRDILKDMYGFEGFVVSDANAIKECVVHGIETEAAAKAMAAGMDMDMGTWIYS---RKLKEAVETGKADISDIDDAVRRILS-IVLLKNE-
GVLPLEKSA-NISLVGALADPEEVLGSWAIGGRAKDCVTIYQGLQNAGAAELDEAQVRMAVEAGDVIVAVVGELASMSGEASSRADISLPGRQQEMLKMLLESGKPVVA
LLMNGRPLALSWE-KEHLPAMVECWHLGIQMGNAVAHVLFGEVNPSGKLTCTFPAVTGQCPM-YYNAPKTYLAPVEPLYPFGYGLSY
>A0A1Z5SM83_HORWE
--VSRLGFPGICLADGPAGVRGTTF-VNAYPAGIHAAASFNRKLAYERGLY
MGGEFRNKGVDTALSVCVGVGRVATGGRNWEAFGADPYLQGQLGSQMVSGMQL-SVTADVKHFLGNEQVTNKNNVTIPAFDITDHDIHELYMWPFQDAVHAGAGSVMGA
---YQQVNGEYSCESSDLLNDLLKDELNFPGFVVSDWG-----A-----QYTGWPSAASGLDVAMPNSRGKWDGGNLEMSVRNGTLPESRLRDMGRRIV---VLVKND-
GALPL-KTPKILNLYGYDATGNLWVGGGSGGNSPPYVSTPYSALAAKAEMFNASETNPSVNAEADACLVFINDY---ASEGNDRAGVADP-ASDELVTNVAANCSNTVV
VIHNAGQRTLDFAGHPNITAIIMAHLPGQDSGRALVSLLFGETSPTGRLPYTIPYKAEDFGSPVVTPEAPYKFQSNDIYAFGYGLTY
>W2RZ60_9EURO
---FSYATCFPQPYTMGAAFDDQLVRDVAGV
ISTEARRAGVNF-WSPNLNPFRDPRWGRGQEVPSEDVYHTKQYGKAFIRGMQGDKVMATCKHVAAYDLENWHGRVRYGFDTVSSQDLAEYYMQPFQMCVDEKVASLMCS
---YNAINGTPACADPYLMKDIVRDHWDWKGTMVSDCGAVRYASDHHFREEMVTESLKSGIDIDCGYYYP----DYLGSAVRQGLLSEDDINQALIR----VVLLKNG-
GILPLQDRNLTIAAFGSWMNTVEMQGNYQGLAKS--IKSPLEALQALDNAQRSKYEFVQTV-KPDVIVIADGNRLQDAMETVDRDLLEWHGEMSDVVSSLSGLGIP-IV
VINMGEQCDNGPYRDDGVSAVLWAGYGGQAGGEALANILTGKAAPAGRLPVTQYPTEYVYSIDMTDMADPYRYDNAT-LPFGYGLHY
>A0A420WJH9_9PROT
-LDEKLAQVSCVWFGKMEGPSDEPRDVNDSTVVRRMS----ARTPADTVDLVNTQKWMLEETRLGIPTLFHEEGLHGFQGRYA--TAFPQSIALAATFDTELAEDIYSV
TAREIRARGVHHVLSPVVDVALDPRWGRIEETFGEDPFLVSRMGVAAVKGFQGDKVLTTLKHMTGH--GQPESGMNVGPAQISERVLREIFFPPFEAAIEGNAATVMAS
---YNEIDGIPSHANDWLLNDVLRGEWGFDGAVVADYFAISELQVRHGVPEAGALALKSGVDMELPDGTAF---PQLKAMIENGEYDIKYLDQAVRRILEL-VLLKNDD
KLLPLIEDYKRIAVIGPNSD-ITVLGGYSDEPRQ--TISILDGIKEQVGDMARIYQAVATAKTADLIILAIGGDESTSREAWDRNDITLIGQQKELIEALAETGVPIAT
VVISGRPLSLENV-EDKLPSILYAWILGQETGTAVADILFGEVNPGGKMPVTVPRNVGQIPS-FYNHKRRYAGDASPLYPFGFGLSY
>A0A0S6WTQ9_9SPHN
--EEKAGQLVQYFYLGMTSAISVEDAVARGEVGSLLL-------MTDPVEINRLQKIAVEKTRLGIPLLFGFDVIHGFH------TIMPVPLAMAASWDPSVAEKGQAV
AAAEARAAGVNWTFAPMVDIARDARWGRIVEGAGEDPVLGAAMAAAQVRGFQGERILAGPKHFAGY--GASLGGRDYDEVNLSDNELRNVYLPPFKAAIDAGAGNIMAA
---YMQLNGVPATTSSWLLNEVLRKEWGFDGFVVSDANNVSSLVRQGAPEEAAVRAIRSGLDLSMEMPSRTSPMLSLAQSLKDGTLDAKTLDTAVLRLLE--VLLENGK
GVLPLTSKTQSIALIGPFANPHDQLGPWVFPGPKPTGVSVLEGLKAKLGDAAEFDRAVAATKAADVAVLVLGEAQNMAGEAASRASFELPGQQQRLLEEVIATGKPVVV
VLMNARPINLQGA---KPDAVLEAWYPGSEGGPAIANLLTGDAVPGGKLPISWVRDGSQAPF-SYDRMHAWDASNAPLYEFGYGLSY
>A0A010QW43_9PEZI
--RADLVSVFPSGITAAATWDRDMVYRRGVA
LAEEFKGKGGHVILGPSTGMGRAALGGRNWEGFGPDPYLAGVTIENTITGMQSTGVQTCSKHYIAN-EQETQRSNTLPTSNVDDRTIHELYLWPFANAIRAGTTSIMCS
---YNRFNQTYACENSHLLKELLRDELGFQGYTVSDWFA----------THSTADSINAGLDLEMPGAVPGNYGDKVLEAVANGTVTDSRIDDMVRNILT-TVLLKNVN
QTLPLN--PKIIGVFGNDAGGTMIIGGGSGTGRPSYVISPLAAVRAKAAEVLAANDFRGVYPPPEVCLVFQQTF---ASESFDRTSFELDGNSTAVINNVADFCETTVV
VTHSGGVNTMPWANHTKIGAILAAHYPGQESGNSIVDLLWGDIAPSGRLPYTVPRNEEDAPANLTQPVDPYDLDIEPLYEFGFGLTY
>A0A420XNN5_9ACTN
TLQEKLGQLQMQGG-DGAPNDDLVSLARRGMVGTTLG-------VRGAANVNKLQRAALAS-RLGIPLLFAFDVIHGYR------TIFPTPLGESASWDPSLARAAARV
AAVEATAAGIRWTFAPMADVTHDARWGRIVEGAGEDPYLGSAFAVARVRGFQGSNMAACVKHFAAY--GGVESGREYNTVDVSVERLWNTYLPPYHAAVNAGVATVMTS
---FNDISGVPSTGNPELLQDILRGRWAFDGLVVSDYTSVLELVAHGYPADAARLALTAGTDIEMVSTTYH---DNGRELLLSKEITRRQLDDAVRRVLTLVVLLRNEG
GALPLSPGIAKIAVIGPLADGADMIGSWSGDGRAEDSVTVLAGLKAAYPSAPDLAPAVAAAAAADLVVLVVGEPAALSGEASARSDIGLPGHQEQLVETVAAVGKPYVV
VLVTGRPLALPWI-AEHAPALLVAWHPGTEAGNGIADVLLGKVNPSGKLTAEFPRAVGQLPL-YYSHRSTYLVDNSPQFPFGFGLSY
>L7VKQ9_THES1
-----------------------YENLNDKGITGVIKMQRTLEEKASLCSGLDAWNLKSVE-RLGIPSIMVSDGPHGLRKETTDATCFPTAVGLASSWNRELVEKVGAA
LGEECQAEGIAVLLGPGTNIKRSPLCGRNFEYFSEDPYLSSEMAASHIKGVQSRGVGTSLKHFAAN----NQEHRRMSVDAIDERTLREIYLASFEGAVKAKPWTIMCS
---YNRVNGEYASENKFLLTDVLRNEWGFEGIVVSDWGAVNE----------RVKGLEAGLDLEMPSSFGI-GDQKIVEAVKKGELPEEVLDRTVERILNLMVLLKNED
KILPLRKQG-TIAVIGEFAKRPRYQGGGSSHVNPTIMDSPYEEIKKSAGNEKLLEEAKQAALKADVAVIFAGLPEHYECEGYDRQHMRMPESHCTLIEEVAKVQPNVVV
VLCNGSPVEMPWI--DKVKGLLEAYLGGQAMGGAIADLLFGDANPSGKLAETFPKQLSDNPSN---FPDRYDKNMEPLFPFGYGLSY
>Q0CE57_ASPTN
---HLLQDWIMNDTSLSLPAIVQCEGLHGLG--VPNATIFTSPIGLGASWNPELVEQAAQI
IGQEARALGITQVFAPSADLARDPRHGRVEESMSEDPYLAGEMVAHFVQGLWKNKVASIVKHLVAY--GNSEQGINMGPVHGGERELRTTYLPPFRKAIEAGAFTVMTS
---YNSYDGIPSVMNKHLLTDILRGEWDFQYWTTTDYGAPNRLCTAFKMCRDNPIDAEA-ITMKIFPAGQDTEFDSIPDLVNDGTLDIAIVDEAVRRVLR-IVLLENHD
NTLPLKDDIGSIAVIGPFAD-TVNYGDYTMSR--PRGVTPLDGIRSRASQESGIHEATELAKSSDVAVVVVGTWTRSTGEAYDTNDLGLVGAQRLLVQKVAETGVPTIV
VFSSGKPI-VEPWISNATAALLQQFYTSEQGGEALADVLFGKYNPSGRLPLTFPRDVGSLPVDYLDSG-RYV-TGTPLYEFGYGQSY
>A0A3Q9I604_9BACL
TLEEKVGQLVQPFGWQIELTEAFKKQIESGGVGSLYGVLRGLSPEEGAAAVNEIQRYAIEHSRLGIPILIGEECSHGHM-AIGA-TVFPVPLSLGSTWNVELYREMCRA
VARETRSQGGAATYSPVLDVVRDPRWGRTEECFGEDAYLISELAVASVEGLQGQSVATTLKHFVGY--GSSEGGRNAGPVHMGKRELLEVDLLPFKKAVEAGAASIMPA
---YNEIDGIPCTTNHELLQDILRTEWGFEGMVITDCGAINMLAAGHDGMDAAVQAIRAGIDMEMSGEIFG---NHLLEAVRQGKLDEVIIDTAVLRVLD-IILLKNED
QTLPLSKKSGRIAVIGPNADGYNQLGDYTSPQPPANVTTVLGGIRSKLADREGFDYALECARQADTVVMVVGGSSARCGEGIDRMSLKLSGVQLELIQEVYKLGKPVIV
IYINGRPIAE-PWIEDHANAILEAWYPGQEGGHAIADILFGDVNPSGKLTISIPKHVGQLPV-YYNGKSRYLDDSQPRYPFGYGLSY
>A0A433SSR8_ELYCH
---SRLGIPYVWNSNCHRGDQGAKENATAFPQSIGLAASFSHQSVETRGKH
--NDFVRRGVYATFSPVINVVRDPRWGRIQETYGEDPFMSGELAATFLDGLHGDRVTGGCLHLDAY-SGPENIPVSRLSAKVSDYDLYMTYLPGFKRCVQAETFSIMCS
---YNSVNGIPACVNKRLMTDILRKDWKFQGYVVSDEQALEFVISTHKFEDVAAAAVTAGVNLELSADMPQPVFLSIVQAIEQGKLSEAVVRERVK------VLLKNSN
NFLPLPSNYKTVSIIGPMAD---NYDQMFGNLRV-FAKTPLEGLKEIFPSNFSREAVQELVNGTDLVLAVFGTGPAVEAENNDRANLDLPGMQKQVLEEIMEHCAKILV
VLMNAGPLNVTFLVDPRVQAILECFFPGQATGDAIKEVLIGNSSPAGRLPVTWPLHASQIPPNY-SMVRTYRMQSLPLYPFGYGLSY
>A0A540VGT7_9CHLR
TLEEKVAQLTSVWVYELAFSPQKAAAKLGDGIGQITRVAGNAAPRQTAELANTIQRYLVEQTRLGIPALIHEECCSGFMGRGA--TCFPQIIGVASTWEPELVAQMAAV
IRTQMRAVGAHQGLSPVLDIARDPRWGRLEETFGEDPYLTARMGVAYVQALQTDGIVATGKHFLGY--SFTLGGLNWAPAFIPPRELWEVFMFPFEAAIEAGLASMMNA
---YHELDGIPCGASRELLTEILRNQLGFDGLVVSDYMAINQLVDYHHKTHAAHLALQAGIDVELPSRDCY--SDALVEGVRTGAVDPALVDQSVIRVLE-IILLKNEG
DLLPLDRQPGTIAVIGPNGDIRNMLGDYSYPAIYPAMKSVLQAIREKVGETSGFDEAVAVARRADVAIVVVGDKSGLCGEFRDRATLHLPGVQEELVRAVVETGTPTVV
VFINGRPVSSPWI-AAHVPAIVEAWFPGEEGGPAVADVLFGDVNPGGKLPVTVARSVGQVPI-FYAHRSHYVETVAPLFPFGHGLSY
>A0A0B7MX61_9FUNG
-VEEKIGQMTQINQ-DVLNRTAVEYYAKNYYVGSYLNQLANYDAADYAKIIEEIQETLSVNSTFKIPIIYGLDHIHGAHYVAN-TTIFPHGINIAASFNPELAYESASI
TARDTRASGVQWTFAPVLDIPVNKQWPRVFENFGEDPHLSSVMGVASIRGYQGKKVAACMKHFIAY--GAPYSGQDRDSTVVSERTIYDYFVPGFQAAIDAGVATAMES
---YIDINGEPVVSSKKYLRQLLRDQMKFGGMLVTDWQEMENLHTKHMHKEAVRMAIDTSIDMSMVPQDVI-FFESMMDLIKEGRVTMERVDESVARLLQLITLLKNKN
AALPLKDKIKRVLVVGPAGDQGATEDAWHGNVF----YSILDGMQNAVPEDVNMDLVLEKAEDFDAFVVCIGEH--IYSELPNIHDLTLPQGQINNVEKLAGKEKHLIT
VLLEGRPRVLGSV-QDNSDAILQAYLPGPWGGQAIGEVVFGLTNPSGRLPYTYPKYAGDTTLNYWRPA---NDIWDPLYEFGHGLSY
>A0A364KTH7_9EURO
--RLDFPGVCMEDGPNGVN--YNDKNVFPSGVTVAATWDTEFMYQRGLA
LGAEFRDKGINVMLGPVGPLGRSPYDGRTWEGFSPDPYLTGLAMQATIQGVQENGVQTTAKHFIGNEQTTLSNGTQVAAINIGDRALHELYLWPFANAVKAGTSAVMCS
---YNRLNETYACENSDALISILKTELGFEGYVVSDWG----------ATHSAAPSANGGLDLEMPGYTGTTEIPYLPQAIADGSVSIDRLDDMVGRIMT-TVLLKNEG

SILPL-GSYNEIGVFGSSAAGPIAVGGGSGSVRFTTVSAPLDAIKAQASTTAIAANDITFYPVPEICIVFISQF---ATEASDRDSLLASDNSTAVVENVASLCNNTIV
VINAPGPLVLPWVENPNVKAILAAHFPGDQIGNSIVDVLWGNTEPSGRLPYTIPAKTSDPPV--VREPDGYDNRSEPAYEFGFGLGY
>A0A0E9NIL3_SAICN
--RLGIPELCFQDGPAGIRPT--DFSVFPAGITAASTWDRELLLQRGLA
LGAEWRGKGINVALAPVTGLGRNAAGGRFWEGFGADPYLHGAAAYETIIGWQSNGVIATAKHWIAYEQRNTNSGAGYPFTNVSDRTMHELYMWPFAEALRAGAGAVMCS
---YNRINGTQACEDPYSLNHLLKTELDFQGFVMSDWGA----------TYSTIPAVLAGEDVEMPGGDQW-FGQGLIESVNNGSVPETRLNDMVTRLLT--ILLKNTD
RGLPL-TNVKKLAMFGTDAGGTQAVGWGSGASLFPYLVDPLAAINERASQGDSWATINATAHRATHCLVFVQAR---SGEDSDRQNFTLWDNGDNLINTVAANCPNTIV
VEHVVGPVLMEEWENENVTAILNAHLPGQESGNSLVSVLFGDVNPSGKLPYTIAKSADDYPIGEYVNDQVYRFDALNIYEFGFGLSY
>A0A1M6FLV9_9ACTO
-LEEKAGQLIQYF-YQDRLAEQDRDVLAQSEMEGEVRAGRSLLFVRRPEAVNALQRIAVEEGPHGIPLLFGYDVIHGLR------TIMPVPIAMAASWDPAMIEEAQAV
AAREARAVGLSWTFAPMVDIARDPRWGRIVEGAGEDPCLGAAAAAAQVRGFQGERVIAGPKHLAGY--GAARGGRDYEDAEISDNELWNVYLPPFRAAIGAGAGSIMSA
---YMDLGGVPASGNAWLLTDVLREEMGFEGFVVSDANAVRSLTAQHFLPDAASRALTAGLDMEMTMEDAA--FRRLPEAVRAGRVEEGRLDEAVRRMLT---------
PVLPLAEDTPSIAVIGRLADPRDTLGPWVFDEDLDEVTTILAGIRARAGGDAALAEAVRTARGAEAAIVVVGQAQNQIGEKASTSTLALPGRQLELLRAVAATGTPTVA
LVMSGRPLDLRWA-QENLPAIMQVWYPGTRGGEAVAAALFGDIDPAGRLPFTWPRTVGQVPMHSHYRTFQYWEASAPLYPFGHGGSY
>A0A1X2HFP3_SYNRA
--EEKMYQLMQGNIANMIDTDYNLNSSALHQYGTTF--AANMDRDPLARLINETQSYMLNDNRLKIPTIMQSEGVHGYLDVNA--TTFPAALALAGTFNTDLMEKVGDI
IGTEAASLGLHNIFAPVLDLAREPRWGRIEENYGEDPYLTGEMGYAYVKGIQGKRIGAMVKHFVGF--GSPMGGLNIAPVLGGERDMRTLYLPPFKRTIDAGALSIMSA
---YHAYDGIPSAIDKHTLTDILRNEWGYQYFVESDSGAIANLCDTHYDPVAAVKAIEAGNDIEMGGRPMH--YATIPDQIEKGNLKQSTVDEAVRRVLR-ITLLEND-
GTLPISESVESIAVIGPQAN-VMQYSDYTAHGVFERGVTPLAGIQKLVGDKSGFDEAVEAAKKSKVAVVMVGTWTRDTGEHVDANDLRLVGAQMDLIKAVQKTGTPTVV
LLITGKPTAE-PWLKDNVNAILNAFYPGEQSGTAIANILFGKTNPSGRLPISFPTSVGSLPA-FYNYPRPYIGTPVPLWYFGHGKSY
>A0A1B3NKC1_9HYPH
TLEEKVGQLHLSG----RGDGFDIAQVKAGRMGAVMNF-------V-VPAEVLAVQKAVRESRLKIPLIIGLDAVHGFS------TYFPLPLGQAASWNPALIEQAAYW
TGREAAAAGINWTFAPMVDMSRDPRWGRVLEGAGEDVHLASVVAAARTRGYQRGGVATSVKHFVGY--GAGEAGRDYNSTWIPTSQLFDLHLPPFKASFDAGSMTAMAA
---FNALNGMPATAHRGMLTDLLRGQWGFRGFVTSDFGSITELRLHGIDAEAARKALLAGIDMDMMGDVYH---KHLAAEVRAGRVPVKALDEAARRVLRV-MLLKNAG
DILPIRPSVKSVAVIGAMARDPAGLGRRV--------VQPPEALKERLPAFADREAAIRAAAASDLIIAMLGEDCEFMGEGASRTRLDLPGVQQPLLEALVATGKPVVL
VLATGRPLVLTWA-DAHVAAILQTFHGGTEGRTAIADILTGKVNPSGRVPMSFPRSVGQIPV-YYDHLRPIDEANEPLYPFGHGLSY
>A0A2V1D797_9PLEO
--EFSSATSFANPITISAAFDDELVERVGKT
IGVEARAFGLDF-WTPNINPFKDPRWGRGLETPGEDAFRVSQYVKHLLRGMEWAHIIATCKHFASYDLERWEGIVRQKFDQVRMQDLVEYYLPSFRQCADSNVGSIMCA
---YNRVNGTPACADSYMMQTVLREHWGHGNYIVSDCNAVKNIWADHNAAQAAGKAFTAGMDNVCEVSRGS---TDVIGAFNQSLVSEEVIDTSLKR----ITLLKND-
GILPVFKKNQTVAIIGMWANQNRMLGNYFGR--PPYYRSPLWAARQLNISTFNQSAAIEAARSSDTILYFGGIDGSIEAEDLDRTEITWPAPQLSLLSALSKLGKP-II
VVQLGTSLDNSPLDNPSISAIVWAGYPGMYGGPAVFDILTGVKAPAGRLPITQYPAEYAKQVNMTDMTRTYRWYDQAVQEFGYGLGY
>A0A558R463_9SPHN
-LEEKVGQISQ-RFDIMPAMTPLDDNVRKAELGSLLF-------VHEPAVANKYQKIAVEQTRLKIPLLLGYDVIWGMR------TMFPVPIGGAASFDPAGVEQARAI
AASEARALGIHWTFAPMVDIARDPRWGRIVEGAGEDPYLGAAMAAAQVRGFQGDHIVAGPKHFVGY--GASVGGRDYDSAYLSDSELYNVYLPPFAAAINAGAGNIMSA
---YMDLNDVPASANKRLLTDILRGDLGFKGWVVTDASAVHNLVKQGFGADASVRAITAGVDMEMSTAPNA--FSTLVASVRAGKVPMKRIDEAVRRVLM-LVLLKNDG
NALPLAGAQKRVAVIGAMAD---SPGDTTLSLDAVKAVTVFKGISERLSGADELKKAVGLAARSDVVILTLGEKIEMSSEQASRSDLALPGDQRKLLDAVLATGKPVIV
VLMNGRPLDLTGV-YDKVPAILEAWYPGSRGGTAVARALFGDVNPGGKTPVTWPRSVGQVPT-YYARNDPYWAPSTPLIPFGFGLSY
>S0FPI8_RUMCE
TLREKVGQLNQ-RLYGVELSAEFKEEVKYWNGLGVLYGLYGLEGSLAIKAYNLAQKYVVRHSRFGIPMLMSSECPHGHQAL--DGYLLPVNLAMGAAWNPELMASAYGV
CARQMKDLGVNLALVSMLDVLRDPRWGRSEECFSEDPYLCSVLAEAAVKGCQERGVPVVAKHFCAQ--GEGTGGINASAARIGERELREIHLPPAAAVCKAGVKGIMAA
---YNEIDGIPCHGNSRLLQDILRREMNFSGVVMADGTAVDRLDILTGNMRSGALALSSGVDISLWDKGFT----GLEEAVKKGLVSEKLIDRAVLKVLEL-VLLKNE-
GILPLLKKVKSIAVIGPNADIYNQLGDYTPPLRKGEGITLLKGLENLCSDETGIKTAVELVSSSDIVILALGGSSSRCGEGVDCAGLTLPGVQQELARAVFDTGKPVIT
VLIQGRPYAVEEA-AARSRALIAAFYPGPMGGQALAEILLGKVCPSGCLPVSIPRSAGQLPV-YYNHKDAYSMANTPLFPFGFGLSY
>A0A0R1VY77_9LACO
-LKEKIGQLTQFTPQYGREDGELTGIMGDFAVDQVYLDSLSVLNATDRNEVIAMQREHLKKDRLKIPLVFMRDIIHGYR------TTFPIPLGLGATFAPQLVEEVSHV
AGTESAREGIQVTFSPMADLCRDARWGRVMEGTGEDPVLNAQMAAAMVRGYQGARIAACVKHYAGY--GAVLAGRDYNNVDFSRLSLYQDYLPAFQAAIEAGAKLIMPA
---FTLFEGLPATASEYLLKKVLREYLKFDGVAISDWGSVGKLLTMHIQERASELALNAGLDMDMMAGAFV---RGLSDAVAAKKVAVEDVDRAVLRMLNLVVLLKNK-
NVLPISVKS-HIAVTGPLAASQRILGAWSSYGKHDDAVSLYAGLKKQFENPDPISSDPADYANFDVIIVGLGEREDDSGESACKTRIELPEDQVALIRRLQHTGKPIIG
VIFAGRPLALTNV-VPYLDGLLYAWFPGTEGGNALANLISGKAVPEGHLPMTFPRTTGQVPI-YYNEPRNYVCQNSPLYPFGYGLSY
>A0A1J4K6J3_9EUKA
TLEEKIGQLVCPDG-----RFNFTEIFNEQHIGATFFL---------FDDDAKHAQEMARNTRLKIPLIMGIDAIHGNS--FYNGTIFPTQLAASCSWDVEVMKEMAQI
TASEMKYTGTFWTFSPVLCLTRDLRWGRVGESFGEDPYLIGVLADAMITAYQDNGIIATAKHFVGY--GETIGGRDASEGDLSRRKLLSYFTPPFEK-VSSIVSSVMSS
---YQALDGTPAVVNHWLLNETLKEKWGFKGFVVSDYDNVGRLINDQQFVDASLASVKAGNDMFMHTPQFF---GAALEAVNQGKLPISYIDESCRRILRV-VLLKNN-
GILPLESLIQSFAVIGPNADIIIMNGDWSLGSGRNCTVTILDGIKNRMKNEEDIHYAVENVQKSDISVVVIGDRLKYSGEGKSTGTLDLMGNQIELLEKIMETGKKFII
DVISTKPVIIPQHIVDNASAIIHQFSPGMLGGTAFSELIFGDINPSGKLTVSYPVHVGQQPVWY-NQVQHYALTETPMWSFGHGLSY
>A0A2K1IUW5_PHYPA
--EKVTQLVNTASAIPRLSIPAYWWQEGLHGVAHVLPRATSFPLPILTTASFNKDLWNQIGQV
VSTEARADGIATYWSPVINIARDPRWGRIQETSGEDPYTTSAYATHFVQGMQEGKLSACCKHFTAY-DVDNWEGIDRYHFDVTLQDLADTYNPPFQSCVEGRSASLMCS
---YNKVNGVPTCANYDFLENTVRRAWGLNGYIVSDCDSVLVMHESTNTEDAAADALNAGLDLNCGDYLA----SYTEGAVAMGKVNASRVDNAVYNV---IVLLKNDG
NILPLSKNI-NTAVIGPNANTHTMLGNYEGIPCQ--YITPLQGLVKFGSGDDQISSAVSTAAVADAVVLVVGLSQVQESEALDRTSLLLPGYQQTLIDEVAAAGRPVVL
VLMCAGPVDINFANDKRIQSILWVGYPGQSGGQAIAEVIFGAHNPGGKLPMSWYPN---MPD-------SYRYTGEKIYDFGYGLSY
>G3AHM6_SPAPN
--SHATSFPQPILMGGAFNNDLYKQVGNV
IGTEARAFGLDF-YSPNINPFRDARWGRGQEVASESPVLVGNYALNYVQGLQGGQVAATCKHFVGYDMESWNQHSRLGYNAISDQDLADFYLPTFQSCVDAKAAGAMCS
---YNAVNGVPACASEFFLNTVLRDGFDFNGVIHSDCDAIYNVWNPHLLGGAAADAIKAGVDVNCGDTYQ----NNLGYALGNKTINENQIRTSVTR----IALLKND-
GTLPFKQKVRKVAVIGPWANTTQMLGDYAGT--PPYMISPLQGAQSEGFQTSGYTAALNAAKGADAIVYFGGIDNSVENEALDRESLAWPGNQLDLVSKLSGLKKPLVV
LQFGGGQIDDTEINNKNVNAIVYAGYPGQSGGTAIWDILSGKYAPAGRLTTTQYPASYQVPMDMTLRPRQFMYNGEPVYEFGYGLHY
>A0A0Q6UM06_9ACTN
---EKLQQV---------------QLLSDGQITDADARAGSVFSLVDPEKIDHFQRIAVEESRLGIPVLFAYDTIHGYR------TIFPVPLGAASSFDPDVARADAEV
GARESAVQGLKQVYSPMVDVSHEPRWGRIVEGAGEDPYLGSVMGAARVKGAQGSKVVSSVKHYVAY--GQPEGGRDYNTTDMSESRLRNLYLPPFKAAIDAGADTVMCS
---FNSINGVPGCANKYTETDILKKEWGFDGFIESDYTAVAELRACPPGPQAGAAALMAGTDSEMVSTNIR---DYGTELLASRQITMRRLDDAVRRILRVMVLLKNDD
GALPLDPE-RSTALIGPFGDVDDVLGPWSGRGLAPDHVPLVAGLRAASSAGTTIEDAVAAAEAADQVVLALGENAFMSGESNARSELDLPGAQEELIDAVAQTGKPLVV
VLFNGRPLDLSAV-QGKASAILEAWFPGTQGGNAVADVLFGTVNPGGKLPVSFPRRVGTVPY-YYNHERPYRDTCAPLYPFGYGLSY
>A0A5C6CKZ0_9BACT
-LEEKVGQLTQSNGIGGEATGNTKNLVADSALYELIRSGQSILNEINVTTVNEFQRLAVEESRLGIPLIIGRDVIHGFR------TIFPLPLGQAATWNPEMVEQACAI
AAREARSAGVGWTFAPMVDIARDPRWGRIAESFGEDPYLASSLSAASVRGYQGDSIAACVKHFAGY--GAAEGGRDYNATMISPSTMRNVYLPPFQAAVDAGVATLMCG
---FHDVNGIPMSVHKQLLSNVLRGEWGFEGFVVSDWDSIFETIEHGSERAAALAAAQAGVNMEMSSPCYR---KNLTELVTSGQVSETTVDELVKPILRVVVMLKNES
SVLPLKTILKKIAIIGPLADKRDQLGTWIPDGKEADSQTPLAAIRESAKNTQGFAEAVATAEQADIVVLIVGERANISGEARSRATLDLPGAQNELVSTLAKVGKPIIL
IVQAGRPLTIGKQ-IEAVDAVLYSFHAGTMAGPALADLLWGIESPSGKLPVTFPKSVGQIPL-YYNHVNTYIVGPYPLFPFGYGLSY
>A0A5P1FMX0_ASPOF
---VKAATSFPLPINSAAAFNATLWREIGKT
ISTEARAMGFATFWSPNINLVRDPRWGRALETPGECPTTAGIYAINFVRGLQDVKVSACCKHYAAY-DVDYWRTTGSMTVDRTERDMVESYLKPFEMCVEGDVSSVMCS
---YNKINGVPACADGRRMRGTIRDEWDLHGYVVSDCDSIKTIFEKQQAIEATAQVMRAGLDLDCGWYYA----QYLEETVKKGLIKESDVDEAL------MVLLKNKG

DALPLPQKFKRIAIVGPHANTEVMIGNYNGVPCR--YISPIDGLKKYA-------EVDYKCRGCR---CAVGLNLTYEREDWDRTGFDLPGYQNHLIETVANVSKPVVV
VIFSSGTINISSFKSGDVDAIIWAGYPGQEGGQAVADIIFGAYNPGGRLPVTWYPSEYQIPEQFR----PYRYNGTTQFDFGYGLSY
>A0A031JUY3_9SPHN
TIEEKAGQMTCLAD-AIQDVKKLSEEIRKGRVGCLFNG-------IGVAGARKAQEIAVNDSRLGIPLLLAGDVIHGLK------TIFPVPLAEASSFDPVLAQRTARA
MALEATAAGLHLTFAPMADVARDQRWGRVVEGSGEDVTLTALLSAARVRGFQGRSLLACPKHFAAY--GAVAGGLEYGSVDISDETLRETHLPPFGSAFAAGALTTMAA
---FSEINGVPATADRTLLTDILRGEMGFTGFVFSDYTADEELVAHGYDRDAARLAVLAGVDMSMQSGLY---IRYLPDLVKSGAVPMGTVDVAVRRILY-IVLLQND-
GVLPLKGKKQKIALIGPFGDKANLYGPWAFYGDADKGVDVASGLRGAMVDDGGIEEAVKAAKAADVVILALGESQDMSGEAQSRTVIEIPPAQQALADAVAAVKKPTVV
LLRHGRALAIHDG-VANANAVLATWFLGSEAGNAIADVVFGKVDPSGKLPVSFPWESGQEPF-FYDRKSTYATDNSARYPFGHGLSY
>F7PJW4_9EURY
TVEEKVAQLESVPPRWGNINEDNARELLSDSIGHLTRMGGSLEPETAARVTERVQEIAMEESRFDIAAVPHEECLSGYM--GPKGTTYPQGMGIASTWDPELVEGMSTQ
IREQLRAIGTHHALSPLFDVARDPRWGRVEETFGEDPYLVARMGSAFVDGLQGDGISATLKHFAGH--AISEGGKNRSTVQIGEREFREVHLFPFEAAVETDADSVMNA
---YHDVDGVPCTADEWLLTDVLRGEWGFDGNIVSDYFSVRLLKDEHQYYDAAIQALEAGLDVELPQIKAY---QHLPEAVENGDVAEETIDTAVRRVLK-ITLLKNDD
ELLPLEGE-ESVAVLGPKADPSGQLGDYAYAAVNREIVTPLDSLQDRLGATDDIESAAAAAEDADVAVAFVGTRSAISGEGADVTDLELPGVQQKLVEAVNGTDTPLVV
VQVSGKPHSIEWI-DQHVPAVLHAWLPGEEGGNGIADVLLGEHNPSGRLPISIPKDVGQLPV-YYSRRRNVYVDSDPLYSFGHGLSY
>A0A1F5LCV7_9EURO
--FSYATSFATPLLLAAAFDDEMIYQVADT
ISTEARAFGLD-YWTPNVNPYRDPRWGRGSETPGEDPRRIKGYAAHFLRGLGGDKTLTNCKHYAGY-DLETWGGYSRYKANITMQDLVEYYLPPFQQCADSKVDSIMCS
---YNMVNNTPACTNSYLINRVLRQHWNWTAYIVTDCGVVNETVMRTHLAEFSALMFETGIDLVCTGGSP----AAISEAYMKSLLPEKTIDASLRR----MVLLKNN-
GTLPIFMTHPRVALIGVYANTWEMLGSYFGV--PPYYHSPLYAAQQRGLKTGNWTAQIEAAKNADVVIYFGGIDVSTSSELNDRSSVSWPASQLSLIKEVCALGKPCVV
VQLGDQLDDTA-LNNSNVSAILWASYPGQDGGPAIFDVLTGSIPPAGRLPVTQYPTSYQIPMQ----PDRYWYYEDDVLPFGYGLHY
>A0A5B1CHC4_9BACT
TLEEKVAQLNSISIRGVVMKKTITERLN-NGIGQIENTFDPRPPRKSVEQVNKMQQYLIDNTRLKIPALIGSECLHGHAGYN--STVFPVPLAMASSWNPELVNEAFNA
IGIESRVRGSHEAHTPVLDLGRDPRWGRIEESYGEDTYLVSQMALAVVSGLQGGHIVSAPKHFAGY--GQVVGGRNFAATPIETKTLMDDILPPFEVAVIAGAQGMMAS
H---CDVGGVPAHGNRWLLTELLRDQWGFKGMVVSDYMDIKRLEEFHHVQDAARMALIAGMDLDLPDGVAY---QELTAVIKNEPELESYLDQSVSRILRLITLLRNPE
NILPLLDSLDQIAVIGPNAA-SELIGNYTMQ--NDYVVSLLKGITDFASDLPMIEKAVKLAANSGVAIVCVGGDTKSAREAFDRSTLGLLGNQKELVMRVIETGTPTVV
VLMGGRPFSIPEI-AEQPCAILNTFYLGQTNGTAVAKVLFGEVNPSGKLPLSVPRSVGQLPV-YYSQKATYWETSRPLFPFGHGLSY
>A0A6P2C3P3_9ACTN
TLAEKFGQLEMSGPTGGTPGQTLLDEVRAGTVGSVLD-------LVGVSNINQVQQAAL-QSRLHIPVIFGLDVIHGYK------TIFPVPLGEASSWDPAAISRDESI
SASEATADGIKWTFNPMVDISRDPRWGRVVEGAGEDPFLGAAIAAAKVRGYQGSKMAATIKHFGAY--GAVQAGREYASTDMSEQQLRNIYLPPYRAAVDAGAATVMSA
---FTSLNGVPASANPYLLTTILRDEWGFGGTVLSDYQAIQELEVFGYGAQAAQLALTAGVDIEMGVQVPSQFTLYGPDLVKSGKVSMATINNEVRHVLNLMVLLNNNN
GALPLSTSLPSIAVVGPLADPDVPIGYSSSDLNS--VVPVLDGIKTAAPNTSGFGAAVSAAKASAVTVIVAGEPSADSSEASSRSDISLPGQQTALIQAIAATGKPYVV
VLMNGRPLTLGWV-ADNAPALLEAWYPGTEGGNAVADVLFGKVNPGGKLPMSFPRNVGQIPI-SYNELRPYLVANTPQYAFGYGLSY
>A0A0H0XV02_9SPHN
-LDEMAAQLNCPRAADDPAGFEADFPYFAHGIGGVYSASLEAGPEDNARAVMAMQQEVVSRSRFGIPAFVFEECLHGLL--ADGATQFPQAMAMACAFRPDMVRQVFEA
TAKEARSRGSQGCFSPNIDICTDPRWGRSEETWGEDPHVVTVSAKAIVEGLQGARIATSVKHFAGY--GQGIGGRNFAPSHIGPVEMQNVVLPPFRAAIEAGSIGLMAS
---HGEIDGVPAHADTHLLNDVLRDDWGFEGYVVSDWDDVRRIHSLHGEAEAAIMGLRAGVDIELANNGVY---LMLPQLVRDGLLEERYVRRAAERILA-IVLLQNEG
NVLPLSSAVRKMLVVGPNAA-SVHLGGYSPKPFV--GVSALEGLQAYAEQRRLIAEAVATAQDCDVIVMCLGGNESTAREAYDRDDLELIGEQNELAEALLALGKTTVA
VLIHGRPLSP--LLAENCPAILDAFYPGEQGGHAIASILFGDVNPSGKLPVTIVRNVGQLPG-YYYQKRNYVSDSTPLYPFGHGLSY
>A0A402AWY6_9CHLR
TLDEKLAQLGCYWSTAGTFDPEVAAEKMPHGIGQVTRIGAGLHPQESAAFMNELQKIALERTRLGIPIIVHEEATGGFC--HRDATVFPQGIGLAATWNPDLVKQVAEV
IRAQMLAVGARHALAPVLDVARDPRWGRVEETYGEDPILIGAIGTAYVQGLQGQGVAATGKHFLGY--AMSEGGRNWGPVQMGPRELREVYAEPFATVINTGIATIMNS
---YASVDGLPCAGSPAILTDLLRTELGFIGPVVADYASVDMLMNYHRRGEAARLALLAGLDMELPAIDCY--GEPLKAEVEAGRLSQEVVNTAVRRVLQL-ILLTND-
GVLPLSPTIKRVAVIGPGADVPQAGGDYAPGPYFTPHVTPLAGLRAALGQSSGFAQAVEAASAADVAVVVVAGRSGLSGEANDATNLALTGVQPELINALAASGTPLIV
VVLSGRIHTLASV-ADKANALLQLFPAGEEGGNGLADVLLGKVNPSGRLPVSMPRSVGQIPTHVGHRADRYISPTTPLFAFGHGLSY
>D8QYJ3_SELML
--PAATSFPMPIAMAASFNSTLFYSIGEA
VSSEARALGL-TFWSPNVNIFRDPRWGRGQETPGEDPLLASKFASLYVRGLQGGKVSACCKHLTAY-DMDNWKGMDRYHAEVSEQDLVDTYNPPFQSCIDGRVSSVMCS
---YNRVNGVPTCADRSLLTETVRNSWGFNGYIVSDCDALQVLFEDTTAEDAVADSILAGLDLN--CGTFL--GKHAKSALQAGKVTEADLDHAISNLM--VVLLKND-
GSLPLSTALKTVALIGPNANTYTMLGNYEGIPCK--YVSPLQGMQIYNNNGDLVASAVEVATKADAVVLVVGLDQSQERETFDRTSLLLPGMQSQLVSNIANAVCPIVL
VIMSAGPVDISTFDNSRISSVIWIGYPGQSGGAALAHVVFGAYNPGGRLPNTWYHEEFTNVSDM---RRPYRYTGTPLYNFGDGLSY
>F0RST1_SPHGB
TIEEKVAQLVSAWLEIDHRNGNLYQEVLGKGIGQLTRPFGANDPHKQAKAINKLQHYLVTETRLKIPAMLHEECLTGAM--VKGATIFPSALNYGSTWDPALIGRAASA
IGDELRSLGIHQGLAPVLDVARDARWGRLEETFGEDPYLCGVMGIGYVKGLQGASPLATLKHFVGH--SFSEGARNHAPVHCGMREIRNTFALPFEMVVNAHPAAVMPA
---YHDIDGIPCTSNHSLVTDLLKKQWGFDGLIVADYEAIVQLVNDHQMAEAAALAFNAGMDIELPGFTVF--KEGLIEALYRGLVTDEALDQSVLKILQ-LVLLKND-
GTLPLKKGL-KVALIGALADPYAMFGGYAPPVVPVLAKTIKTALQDVLGNVSRIKLACEASRKADVTVLVVGDLAGLVGEGSDAASFTLPGVQEQLMQEVLQTGKPVVV
VLVSGRPYTLDKA-VTDARAILCTWLPGEGGGEAIARTLVGLNNPSGKTPLSFPKSVGSMPY-FYNHTKKVQKQFGTLYPFGHGLSY
>A0A317XSE7_9BASI
---AERLGIPEFCYQDGPAGVR--ASDFTVFPAGVTTGATFNRDLMYRKAKA
IGEEFRAKGVHVALEPVTGLGRSPYQGRNWEGPGSDPYLVGEYAYQTVAGTEDAGVIATSKHFILYEDGLDDRHVSYQSANADDRTLHELYLWPFMNAVRAGTGAIMTV
---YNRVNHTQGTESSYLLNDILKEELGFQGFAVSDWY---SAYDTVNTFNAGLDVIQGGIDGGKRPQTA----AHTVEAVRNGSMSEARLDDAAIR------------
--LPLTKG-QKIGVFGSDAGGTLAVGWGSGGGYFTYLVDPLSALSQRIRSPHDAHYRKQIANQVDAALVFVQAS---SGENVDRFDLELFAEGSKLVQEIASWNNNTIV
VMHNTQQVLIDEWNHPNVTAVIMPHLPGQESGNSLVPVLYGDVSPSGKMPYSMLKRADHYPTWSHNPD------PHVNFDEGLFIDY
>E1R2R1_SEDSS
TIHEKLAQMYSVWFNIVTESPVRFEELLKDGVGEITRPLGPIDARTAVKALNSIQEFLVKGTRLGIPALAHEECLAGLM--AKGATLFPSGISLGALWDEGLVEKIARA
IGDELYSVGSRQGLAPVLDVSRDARWGRTEESMGEDPYLVGTLATAYVRGFQGNRLLATLKHYVGH--SFSEGARNHAPVRMGEDELSDVMLLPFEMAVLAHAASVMPA
---YHDIDGIPMHASLTYLRDILREKWGFDGIIVSDYSGIGQLCHDHRLASAACLAIEAGVDVELPGHECY--KSGALAAIERGDLPVALVDGCVTRVLE-MVLLKND-
GILPLTTG-KKIALIGPLADPLCFFGGYSFPVHH-----ILSSLEDRDEGKSGFPSAIEVARSADVVVLALGDLAGLVGEGSDASSLVLPGVQQELLEELLGLGKPVVL
VLLSGRPYSLKIA-AERCSAILQAWLPGQKGGQAVVDILYGRQNPSGRLPVSIPKAAGAMPF-FYNHKK---SAGTPIYPFGHGLNY
>A0A0L6JSQ8_9FIRM
TLDEKVGQMIQPE-----RHTATADDVKNYYLGSILSGGGNVNTPTGWCDMTDAYQKAAMSTRLQIPFIYGVDAVHGHNNLYGA-TIFPHNIGLGAANDEELVYKIGQI
TAKEVRATGVHWTFAPCIAVPQNEKWGRTYEGFSENTDIVTRLGVAATKGLQGDKILGCIKHFIGD--GATTNGVDASDSVLTEDQIREKYLPPYIEAIKAGARTLMAS
---FSSINGLECHGNKRLLTDILKNELKFDGFVVSDYEAIKDI-DKANFRNCVKESVNAGVDMYMEPRMWKTVITHLKDLVGNGEVPMSRIDDAVTRILRVLVLLKNDN
KVLPLAKSGKKIFVAGKNADLGNQCGGWTITWNITQGTTILQGIKSAVNPTVTYNLNGYGAQGHDVAVVVVGETPYAEVKG-DRTDVALSADDIQTINNVKSAGIPMVV
VLVSGRPMIVTDQ-IKDSAAFVAAWLPGTE-GNGVSDCLFGDYDFSGKLPMSWPSSNAQIPV--NEG-----DGKTPLYQLGYGLK-
>A0A0S7BST2_9CHLR
TLDEKIAQLGSCWFYEKLDMQKVQKRF-SNGIGQITRLTCILPPVQAAQTANLLQKVLLEQTRLGIPAIFHEECNSGSI-ALGA-TIFPQSIGLASTFQPELARLMAAE
IQKQLRAIGVHQGLAPELDVARDPRWGRIEETFGEDPLLISQFGMQYTRGLQGAGIIATGKHFVGH--SLSQGGLNCAPVQVGKRTLWETYLMPFQAAIDAGLASIMNA
---YPELDGEVAAASKNLLTDLLRNQLGFQGLVVSDYEAIIMLHTYHRWAEAAAAAMNAGIDVELPTTKCY--AEDLIHQIEAGIVHMERIDQAVA-----MVLLTNN-
GILPLSKNIKTIAVIGPNADERNLCGAYSYVGDQPIVITPLNAIKEKLPNRSGFPSAIQAAQKADAVILILGDRSGMCGETRDSADLKLPGVQTDLAEAIFTVGKPAVV
VLINGRPLAIPDI-VKRADAILEAWIPGEEGGTAIADILFGDQNPGGKLPITFPRSVGQVPI-FYNNKMKYVESVKPLFPFGHGLSY
>A0A1I4K1N3_9FIRM
TLEEKVAQLGSVGPDKGKFSQQKAEKYLKNGIGQITRIAGALEPEKAAELANQVQKYLAEETRLGIPALIHEECLSGYM--GKGGTTFPQSIGIASSWEPELLKRQTDV
IRKQLRSIGAHLALSPVADVARDLRWGRVEETFGEDPYLVAEMVNAYVAGLQGEGIIATLKHFAGH--SYSEGGRNHAPVNLSERELRETFLFPFEAAITAKAGSVMNA
---YHDIDGIPCAASRQLLTDILRGEWGFDGIVVSDYWSIKMLYNEHKLQEAGIKALSAGLDIELPETECY--GHNLVKAVKEGLISEKIIDQAVSR----MVLLKNES

AILPLSKQIESIALIGPSAATRNLLGDYAYSADAVDIVSIMAGIKAKISSKDGFSQAVKAAQASQVAVVVVGGKSGLTGEHHDRTSLNLPGVQEELVKEIVKTGTPVIV
VFVNGRPLSSQWI-AENAAAILEAWLPGEEGGNGVADILFGDYNPGGKLPVSIPKNVGQLPI-HYNRRRDYVTGNRPLYPFGFGLSY
>A0A1U7LSJ7_NEOID
-------------------------------KGGSFYVGEYLKTIDMYAEAINYGQHVRAVRRHEIDSQNSIDGLGLHGLVMTNATIFTSPIGLGATFNPQLIEKMAEA
IAEEAAVVGINNLFSPNVDLARELRFGRTEETFGEDPYLTGEMAYAYVVGLQRSKVAAMPKHFAAF--SSPQGGLNLAPVPGGERELRTIYLPPFKRAIDAEATMIMSA
------YDGIPSIANHHLLTEILRNEWGYKYAVMCDSGATDLLSSQHGEDCIATISLNSGVDIEMGGGSFN--YKQIPRLVSEGKLEQKAFDDAVRRILS-MILLENN-
GVLPLSSSLKSIALIGPFAN-ANNYGDYVVQGSKYRGITPLDGIQNLIKTQSGFAAAEQLAQEADVAIVFVGTWSRDTGEHVDQSSLDLVGAQADLVRAVSAKAKKLVT
VLTSGTAITE-PWITNVTHALLQTFYPGEQGGAALADILFGLTNPSGKLPISLPRSVGTTPA-FYNYLRPYVNSPVPWYPFGHGLSY
>A0A1C6LN67_9ACTN
TVEEKFGQLQQLTWNPEGQNEQATEAAAEGRLGSVLN-------ITGAKECNALQRHAVEESRLGIPLIFGLDVIHGYL------TTFPVPLAQGASFDPEVVMRDAEV
SAREAASWGVHWTFAPMADVSHEPRWGRVAEGNGEDPYLTAQLAAAKVRGYQGERLAACVKHFVGY--GFPEGGRDYNTVDISERRLRDVALPPFKAAVDAGVATVMAA
---FNTVNGVPAHANPHTLTRILHEELGFDGFVVGDYNGVQELIPHGVGADAARLALGAGVDMEMVSTTYA---EHGKELLEAGKIDQRRLDDAVARILRV-VLLKNDD
RTLPLSKDTPSLAVVGPLGDTKELHGTWAGPGRMFPAVSVLEGIEKAAPGTGGLAEAVAAVEASDAAVVVVGEKAGHSGEAAVRSDIGLPGVQEELIRRVAETGKPFAV
VVLAGRPLALSGV-AEEAPALLYAWHPGIEGGNAVADLLFGDVAPGGKLPATLPRAVGQTAV-YYGHENTYLLPHGPLFPFGYGLSY
>I2FNF0_USTH4
--FTSATQFPQTINLGATFDDDLYQQIASV
IASEVRAYGLNL-YSPLINCFRDPRWGRGQETVGEDPLHMSRFAVSIVHGLQGPTVAATCKHFLAYDLEQYDRGERYQFDAISKQDLSDFHLPQFRACVDGGATTLMTS
---YNAVNNVPPSASKYYLQTLARQAWGLDNYVTSDCDAVANVYDGHRYVEAAAKSINAGTDLDCGATYS----ENLGAALKQKLTDIATI----------ITLLKNLD
STLPIKQKPTKIAIIGPYTNSTSFSGNYAGPAAF--NMTMVHAASQVFPDPSDAQDAVKLTSDADSVVFAGGIDASIERESHDRKDIAWPPNQLRLIHELSDKKSKLVV
VQFGGGQLDGASLSDDAVGALVWAGYPGQSASLAVWDILAGKAVPAGRLPVTQYPASYGLPEM--SLRRTYKYKGVPTYPFGHGLHY
>A0A5C5WAK1_9BACT
TLEEKVAQMVCVWNEKFDPARATEHFGHGHGIGQVGRPGDGATPRDFAELTNAIQRFFVENSRLGIPVLFHDECLHGLV--GRDATSFAQPIGLASTFNPELVRRLYEM
TAREARACGVHQALGPVLDVARDARWGRVEETFGEDPHLVGEMGLAAVRGMQGDRVIATLKHFAAH--GQPESGTNCAPVSISERHLREIFLTPFHKAIEGGAQSVMAS
---YNEIDGVPSHANSWLLRDVLRQEWGFTGTVVSDYYAISELHHREGAAAAATAAVRAGVNIELPEPDCY---LHLVDLVRRGVVDEEEIDELVA-----ITLLKNQS
DTLPLADRLKTIAVIGPNAD-RVMLGGYSGKPKQ--FVTVLKAVEQAVAQLRKIAEAVSIANQADLVIFVGGGNEQTSREAWDRTDLQLVGKQDELVEALHTTGKPIVS
VLFNGRPLAVKDL-ADRSAALLECWYLGQESGTAVADVLFGRCNPSGKLPITIPRSVGHVPA-FYNHRRRYLDDISPLFPFGFGLSY
>A0A137NZN8_CONC2
TLEEKVGQLTQININNDKLNTQLDYYIKQRLAGSFLNNLANAAVPATVRVMNEIQDYIQKNTRLRIPMIYGLDSVHGAN-YIRGATLFPQQIGMGATFNRTAARVWGEI
TAKDTRAVGVHWNFSPLADIAVNKQWGRVYETFGEDPYVAGELGKQVVKGYQGEKVAATMKHFIGY--SSTKSGHDVDGSWMSKRVLDEYFVPPFQALVDSGVATAMES
---YSDIDGDHVAKSKKILVDLLRDQMGFKGALVTDYEQIFKLNFQHHLNDSLVMAMKLGIDVSMVPHNG-QFFDMMVSLVKSGRLPESTITKNAKRVLQLVVLLENRN
KALPLNPSS-KVFVTGPCADLNYLTGGWTFAWR---GTTIAQGLKNIGGSNTSADQIVGAAAASDVNVVCLGETNYAEFIG-NVDDMRLPEGQTDLVKLLSKSNKPLVL
VLSQGRPRSFDNV-VDLPSAILSSFLVGPEGGQAIAEIIYGKVNPSGKLPIVYTNKASLNTINYRRFSD----SYRVQWDFGHGLSY
>A0A231RT81_9BACL
TLKEKVGQLNQ-RMYGELTEAFMEEVAAGGGMGAL----YGITAADSAKVANQIQRYLIEHTRLGIPVMLSEECPHGHQ--ALDGTLTPTNIGIGATWNPQLAEQAYGQ
VAAEIRARGAHLGLVSTLDLLRDPRWGRSEECFSEDPHLTAQFTKAAVHGLQGCKVVAVLKHFCGQ--GAGEGGMNAFPASIGERELREIHLPGMEAGVEAGALGCMAA
---YNEIDGIPCHANRKLLTGILREEWGFDGIVMADGTAVDRLLALTGYESAAALALIAGVDISLWDKAF----STLEQAVLQGKVQVSFIDRAVRRVLQLVVLLKNEN
RLLPLGADLRQIAVIGPNADLYHQLGDYTAIQRPNSGTTVLEGIRELAGDRDGFAEAVELARQSEVAVLVMGGSSARCGEGVDLADLRLGGVQSELIRAIAATGTPIVL
VLIQGRPHAIADV-APDCQAILSGWYPGPEGGRALGEILFGAVNPSGKLAVTIPYSSMSLPA-YYNRKDQYACPRPVLYPFGYGLSY
>A0A1B6Z9V1_9SPHN
-LEEKVGQILQAD-----IASVTPAEVKSYNLGSVLNGGNSVEEWIALADAFWEASTDKTDGGLGVPLLWGTDAVHGHS-NIQSAVIFPHNIGLGAARDPDLLGRIASV
TASEVRATGLDWNFAPTLAVAQDDRWGRTYESYSEDPSIVASYSGAIVKGMQGEKVISTAKHFVGD--GGTENGIDKGDTQGSIDELWALHGAGYPPAISADVQSVMAS
---FSSINGKKMHGYRELLTDKLRGELGFTGFVVGDWD--GHAEIPGCTPTDCVAALNAGVDMYMAPDSWRGLYNSLLVKAKSGELDTARLDEAVLRILTVLVLLKNNK
GILPLNPS-RNILVTGSGANIQQQTGGWTLNWEFVNAETIYEGISKAFKAEVSLSSNGTFESRPDVVVVVFGEEPYAEYRG-DRSDLVFEGENLALIESFKEQDIPVVA
IFLSGRPMWVNPL-LNASDAFVAAWLPGTE-GGGVADVLVGDADFVGRLSFSWPSLGDGNPVNG-------ANAKGALFPFGYGLDY
>A0A1I6LUV7_9EURY
-IREKAGQMTFLALDAQVSEDRARDVVADPGIGGILYGGASFDPEAVASKLNEYQQIATTETDHGLGMVAGIDAVHGNATNE-AAVVFPHNVGMGATWRPDLVRKRAAV
TSRSLRAMGFQWNFSPVADVLYDPRWGRYYEGFHESPSAVSSFVSAAVEGLESSTVASSVKHFAGY--SMPDAGNDRDDARIPLRDLREKVFPPFEAGVEAGAETVMAN
---SGAVNGTPVHASEWLLRTVLRKQMGFEGVVVSDWADIRNLISEHAFEEGVKVVVNAGVDMYMAAESPGQFVDAVETHVQDGEIPEARVDDAVYRILRLMTLLTND-
DVLPF-DGPDTVLVTGPNADPKSQHGGWTLGWTTPPTTTILEGIRERAPPFSEHDAVADAAAAADVVVAVLGEGAYAETDG-DVESLALPQGQRELLSVVAEAAAPTVG
VLVAGRPRGGDRA-FDALDAALMAYYPGSEGGPAVARTLFGDVNPGGRLPFYWPDSTGDVPVASTVR--QITPGDADRFPIGHGETY
>A0A1J0KSY9_9GAMM
TLDEKLGQLNLLDSGDEGANSNVAKLIEEGKVGGILN-------LTSAEKINKAQKIAVEQSRLHIPLLIGLDVIHGYK------TTFPIPLGLSSTWNIDLIEKTARI
AATEASADGINWVYSPMVDISRDPRWGRVAEGSGEDPYLGSLIAKAMVRGYQDNTVMACFKHFALY--GAVEAGRDYNTVDMSKVRMYNEYFPPYKAAVEAGAGSVMAA
---FNDINGVPATEDKWLLTDVLRKEWGFNGFVVTDYTAIPEMVNHGVLQDVSALALKAGVDMDMVGQGFI---KTLKKSLKEEKISIDDINLAVKRILQ--VLLKNQN
NLLPLAKKG-TIAIIGPLGNSVNMAGTWSVSTDQYKSISLLDGVREVIGDKQLLDEAVKIAEKSDVIIAALGESAEMSGESTSRVDIEIPKAQKDLLKALLQTGKPVVL
VLFTGRPLVLVDE-ATTVPAILNVWFPGSEAGLSIADVLFGDVNPSGKLTMSWPRDVGQIPI-YYNHKNTYLVPNDPLYPFGYGLSY
>A0A5E7ZR05_9SPHN
TLEEKAGQLQLMAS-APSFEGQIADAVA-GKLTGVFN-------GNGAEMARRMQTAVMKQSRLKIPLIFAADVIHGHR------TIFPVPVGEAASFEPDLAMRTARA
AAYEAAGAGIDWTFFPMVDIARDQRWGRTMEGAGEDVLVGELFAAARVKGFQGKAVMACIKHFAAY--GAAESGLDYNSVDLSERTLREIYLPPYEAGFAAGAMSGMAS
---FNEINGIPATANKWLMQEVLRDEWKFPGIIVSDYTGDEEMIAAGYGRDAARLAILAGVDMSMQSNLYT---LHLPELVRSGAVPQAVVDRSVRRTLA-IVMLKNDG
DLLPLDKSA-KIALIGPFASQHDLNGPWVVYGDNAQAIDLATGIRNAGARAGGIDAAVAAAQAADIVVLAIGESEGMSGEAQSRADIVLPEPQMALAEAVAKTGKPIVV
VLKNGRALALHGA-VKDAPAILVTWFLGSESGNATADVLFGVYSPAGRLPCSFPYESGQEPY-HYDHKRPWRAPNAALYPFGHGLTY
>A8F439_PSELT
TLDEKVAQLGSVWSYEGNFSNEKAEALLKNGIGQITRPGGNLSAKEVARLINQIQKYLIEQTRLGIPAIMHEECLTGYMGLGA--TNFPQAIAMASTWDPELIEKMTST
IREDMRQMGIHQGLAPVLDVVRDPRWGRTEESFGESAYLVAKMGVSYIIGLQGKGVIATAKHFVGY--GASEGGKNWAPTNIPERELREIFMFPFEAAVEASVMSVMNS
---YSEIDGIPCASSKELFTGVLRKNWGFSGIVVSDYFAIDMLREYHRKKEAAKYALQAGIDVELPKADCY---TTIRELVEQGLISESTVNQATSRVLQ-IVLLKND-
GILPLKKDA-KIALVGPNASVRNLLGDYAYLAHI---KVLLDSVNQTTFNTEGFSEALHAVKNADIAVVVVGDRSGLSGESRSDANLKLPGVQEELVLEIAKCGKPIVL
VLVTGRPYSLKNI-VSRVNAIIEMWLPGEVGGMALVDVLFGKVNPGGKLPISFPRSAGQIPV-YHDVKRSYVELVEPLFSFGHGLSY
>D8SVP2_SELML
--FPGATSFPMPILTAA--SFDAVSTEARA
MH-NYQRAGLT-YWSPNVNIYRDPRWGRGQETPGEDPLLSSKYATFYVRGLQDTKVSACCKHMTAY-DVDNWKGTTRFKFNVTQQDLSDTYNPPFQSCVDAKVSSVMCS
---YNRVNGVPTCADYNLLSATVRSSWNPCSYIVSDCDSLQTFFDNTNAEDVVADALLAGLNLDCGPFLAI----HTQSAITNGKITEANVNQALRYLY--IVLLKNNG
NVLPFKSNIRTVAAIGPHAKTRAMIGNYQGIPCK--YTTPHDGLSAYARVDSLIGSAVSTASQADAVVLFVGLDLNQEAEGKDRTSLLLPGKQQELVTEVTKAAKPAVL
VIFSGGSVDVSFAYNNKVQGILWAGYPGEAGGAAIAQVLFGDHNPGGRLPVTWYPDMNMRPD-----ARTYRYTGQSVYNFGYGKTY
>A0A1B8G8J9_9PEZI
----------------ASFDNTTVYSFNNGQGGGSISF-GNYQNPAAAATLEDVVLQFQKNDRLHVPLINVADSVNGVT-LLN-TTLFPATLSMGQSWNIDLYGKVVQA
MSIENHAVGIHWVLSPELDLAVEPRYGRVGEMYGEDRYHVSRFGVAYVKNMQDTRVATTVKHWV-Y--GSSLGGINEARILGGINDFYNVHSYPYMAVFEANPMALMPS
---YSSYDNVPMTTNIQYTKNVIRDLLKFDGVIISDYAAISQVLSTQHIQAAGLKALAATVDHELGPPNQS-GMEALSILSKNPT-VAKAVREAARRVLTLMVLLKND-
GILPLSSLLLKVAVIGPMAD-IINPGSYAAS-DYSTGSTILSGIKKISSDDTMKAEAVANARNAGLAVVALGSVAQIDGEGFDHANLDFPGPQNELLKAIVETGTPVVL
IVSGGQAFSMEYA-ANATNAIIHTFLQGELGGDVLAEIITGKTNPSGKLTVSIPRLSSAVPINYINSDRKY---QSPVYPFGHGLSY
>A0A1M5MU27_9ACTN
TLAEKLGQLQLVNT----ADLAKAGLGTAGGVGGLFS-------VTDSAVLDAVQHQAVEKTRLGIPLIFGLDVIHGYV------TNFPIPLGTASSWDPEVARTDGRI
SAAEARASGQHWTYAPMMDVTHEPRWGRIAEGNGEDPFLTAAFAAAKVDGYQGSRLAACMKHYVAY--GGAEGGRDYNTVDVSLQRLHNLYLPPFLAAVRAGVATAMAS
---FNTIAGVPAHGSEYAIREVLKERYDFDGFVVSDYTGIQELINHGLGADAAAAGLNAGVDMEMVSTNYV---DFGVALLAAGRVTRREIDDAVRRLLR-MVLLRNED

DVLPFGSGVKTVALVGPLGRTTDLNGTWAGLGPVTPPVTIEAGLQAAGKATSGFAAAVAAARKADAVVLAVGETADMSGEAAARSDIGLPGVQSRLVEAVAAAAKRTAV
VLINGRPLTIADV-LAAAPAVLEAWAPGSQGGNAIADVLTGAVNPGGKLPVSFPRSVGQVPI-YYNHENTYLLPSGPQLEFGFGLSY
>A0A267DV12_9PLAT
---PYQWNTECLSGHGEAGP-ATSFPQAIGLAATFSPELVEAMARA
TGLEVRASYNDYSFAPVINLLRHPYWGRNQETYGEDPHLTGVLSAAFVRGLQGGLAGAGCKHFAAY-SGPEDFPVSRVSFNVPEQDLRQSFLPQFRRCLSAGSFSVMCS
---YNSVNGVPACANKRLLTDVLRRDWNFTGYVVSDEGALEFAVDFHKRSEAAVGALQAGVNLELSPPGCPDQFEHLHEAVAAGRVTRQQL-----------VLLKNAD
DFLPLRLNGKRVGLIGPFADMTLMYGSYAADPMPQYQVPLRSGLARVG-ARRNESALLEAARMSDLLLVAVGTGQLVEAENTDRRNLSLPSGQDRLVRSLAESPPPAVL
LIFSGGPVDIQLAPRSGIRAILWCGFPAQEVGVAVAAVLTGEMSPFASLPFTWYSPDQPLSSNYTLANMTVGSAPRPLYRFGQGLT-
>L8M232_9CYAN
TLPEKVGQMTQITLQAEVDLKKLREAIVKYHVGSILNVHSSALTLGEWQQLIQIQNLATQETRTGIPILYGIDAIHGANYTLEA-TLFPQNLAIAATRNLSLARESAAI
TAYEMRASGIPWNFNPVLDVGRHPLWPRLYETYGEDPYLVSNMGVAYIQGLSGEKVAGCAKHYLGY--SFPLSGKDRTPAWIPERMLRDYFLPPFAEAIAAGVPTVMVN
---SSEINGIPVHSDRNLLTDVLRGELGFQGFVVSDWEDVKNLYQRDRPKEAVYLAVMAGLDMSMVPYDF-SFYNYLIELVQEGRIAESRIDESVRRILHV-TLLKNDQ
DLLPLNKN-QKILVTGPNANRSVLNGGWTYTWYPTSQNTILSALQEKLDAAVNIPEAVTAARNVDVAVVVLGEKTYTETPG-NIDDLALPAAQLQLASAIANTGTPVVL
VLVEGRPRLITPI-VEDAEAILMAYLPGAFGGDAIADVLFGDYNPSGKLPMTYPRSPNDLV--TYDHKETTPNKLNPLFSFGFGLSY
>T1EEJ9_HELRO
---RLGIPYNFDTECLRGVADLNS--TAFPMPIGLAASFSPDLLYEVASA
ISTEVRAIHHNATFAPFMNLAHHPLWGRLQEVFGEDPFLIGQLTKSYVKGLQGNKAIAGCKTVLVHGPENIPSSRFTFDAKVTWRDLQLTFLPHFKDCIEAGALNLMCS
---YNSINGVPACANKSLLTDLIRKEYKFEGFIVSDMDAIENIANGHKYLESAVLAVNAGVDLELHGTANI-SYKLLVEAVEQEKVLYSTILNRTRKL----VLLKNIN
NRLPLEGSIDKLAIIGPMSNMDQIFGGYTPMVVRQFTTTPYDGLKMMVKQTYDQPGVIKAASDADVVVVCLGTGQKIEGEDKDMPDMSLPGLQTQLLRDVVSHASPIIL
LLFTGCPIDLVEFYTQNVGAIMQCFYPAQSTGEALKRVLFKNISPSGRLPYTWPMDLKQVPPDYSMVNRTYRLTSRPLYPFGYGLSY
>A0A179SPW0_9BACI
TLKEKVGQLNQ-KMYGSAFKEQVAKFDSMGALYGLFR-ADNGIKVENSAAVTNMIQKVKENTRLGIPVLFSEECPHGHQ-ALD-STIFPTHIGSGASWNPQLQQMVSKH
VADELHARGGHLGLVSTLDIVRDPRWGRTEECFSEDPFLSSKMTEAVVRGMQGEKVLPVLKHFAAQ--GAGVGGHNSGPALIGERELREIFLPPMKAGVQSGVLACMAA
---YNEIDGVPCHANHHLLTKILREEWDYKGIVMADGTALDRLLLLTGKELAAAYGLKAGVDLSLWDDVYM----EIETAVKSGKIEEQLVDKAVSRVLY-IVLVENKN
KILPISKRLKKIAVIGPNADIYNLLGDYTPPQRRENVVTILDGIKSMVGKKSEFTTVKKMAKEADIVVLALGGTSARCGENIDVANLELGGVQLDLVKEIHSTGTPIIS
VLIQGRPYSI-PLLTQYCEAVLIGWYPGQQGGRAIAEVLFGDVNPNGKLPVSIPRSSMQLPV-YYNYKDSYFMSGRALYPFGYGLSY
>A0A2T1HU26_9HYPH
TLEEKVGQLNLVSGNHPFAPQDVRDAILKGQAGGLFN-------VYGAEYTHSLQEMAVKETRLGIPLLLGFDVLHGYR------TILPIPLGQAASWDMKAIEQGERV
SATEAAAAGVNWIFAPMVDVARDPRWGRVAEGGGESAWLGAQIATARVRGLEGSSVAACVKHFAGN--GATEAGRDYSGLDLSERALREQQLPPFQAAVASRARCVMAA
---FNAVDGVPGVANDRLLRDILRREWGFDGIVVSDFGAISELPVHGVGFDAARIAFQAGTDMDMESRTYV---ASLPLLVREGAVPGGELDEAVRRILQLLVLLKNDR
QTLPFRRDARRVAVIGPLGDGADTLGPWAAHGDPDSTITLKEGIEAVLGGPADIAGAAKLAAQSDIVILALGEHATQSGEAASRASLDLPGDQMALARAVLAVGKPTAV
VLFNGRPLTVEDL-DREAPAILEAWFPGSEGGLAVARTLFGLNEPTGRLPISFPRSVGQIPI-YHDHLRPYILPSTPLYPFGYGLSY
>A0A1I2SBL1_9BACL
TLEEKVGQMTQINVTRPLNEEWMKKVFVDNHVGSILSGGGNPEEWAK--MTNALQRYALQHSRLKIPIIYGVDAVHGHNNVLGA-TIYPHNIGLANSWNPSLVREVYER
TAKEVRATGIHWNFAPGADIARDLRWGRYYETFGEDPLLASEMVGAAVTGLEGDRVAATAKHYVGY--SHPLNGQDRAPAELSLRTLREIFLPSFERAVQDGAETIMVN
---SGSVNGIPVHASPYLLRDVLRKELGFQGVVVSDWEDIIKLHTVHKYKEAIRISINAGVDMSMVPLDAEGFTKNLIELVREKKVSEKRIDEAVSRILA-ITLLKNEK
NLLPLKKDLSTVLVTGPSADPANQMGGWTIGWEMPPAVTLLEGIKGKGSKDKAIRKAVNAAKKADVVIAAVGETPYAEGEG-DTTTAALPPSQAKLIRALKDTGKDVVV
VLVAGRPLVMTET-IESVPAFLMAYLPGTEGGSALADILFGDVSPSGKLASTWPKRIGQLPT-FYNRQG---ASYDPLFPFGYGLSY
>A0A194XN72_9HELO
---ASNFPGPLLMAAAFDDNLIEQVATA
ISIEARAFGAGLDWAPNVNPFRDPRWGRGQETPGEDAFRVSGYTKAFVTGLEGPRGIATCKHLAAY-DLENYRNVTRFTAQVSIQDLADYYTPPFQACADAKAGSIMCS
---YNSVNGIPTCLDPYLLQTVLREHWDWDAYVTTDCFALDVAFDSHNPEQTAADALKAGTDTDCGIFFS----SYLPKALSDGLVAEKDLDRALTRVY--MTLLKNLN
KTLPLTPSAGSIALVGEWSNTTEMLGGYAGAPPF--IHSPLYGLQQVSGIVLDSAPVLAAAESSDIILYVGGIDNTIEAEGLDRVNITWNATQTSLITLLAKLGKPLII
AQSGGGQLDDTDFANPNISSILWIGYPGEDGGVALADVLFGNVAPAGRLPVTMYPASYESPPDMSLRP-NYKFDGAVL-PFGYGLHY
>A0A261Y1M3_9FUNG
-LKQKIGQMSQLNEDKTVNVTAVEYYAKTYYIGSYLNNMANLNAAGYATNIETLSDITVKA--GGLPILYGLDSVHGAHYVAN-ATIFPHGIAQAASFDPSVAYNAAEI
TAKDSRGANIPWTFAPILDLGTNKQWPRIYENFGEDPYLQSVMAAASVRGFQGNKIAACAKHFLGY--GATHSGEDRDSSWIPDRFLYDYYVPPFQAAYDAGAATTMES
---YIDINGEPVVGSTRYLKTLMRDDMGFNGMLVTDWAEIENLYTYHQTASSPLDAVFQGIDMSMIPEDTS-FPELLLQLVQSGKIPESRIDESAGRVLQL-TLTKNNN
TVLPI-KNANKVLVVGPTANQGATVDGWQGAVSDEYFYDILDGIKKAAPSNTTAADVASQAADADYVILCIGEHPYAEAPG-NIHDLTLPAGILTFADDLKAKNQKLVT
ILTQGRPRVIGDV-PTISDAILHTFLPGPWGGIAVGEVLFGITNPSGRLPYSYPQYPGDQTL-VYWKDD--NQHADPLYNFGDGFGY
>A0A2G1PHN3_9ACTO
-WEEKLAQIQV-TF---KMTQEECLDAARSGIGALFW-------PGNAADTNAVQRVAVEETAHGIPLLIGLDVIHGQR------TTFPTPLAMGASFSAAVAQSCAVV
SAAEARSGGVTWTFSPMVDVSRDPRWGRVAEGFGEDPLLTAELGAAMVSGYQHETMVATAKHFIGY--GAAEGGRDYNTVDMSDQRLHSVYLPPFATCVQAGVGSVMAS
---FNTMNGRPVHANRRLLTGILKEELGFTGAIVGDASGVGNLIPHGVLPDAARMSLAAGLDVEMGGHLHDPAARPEHPALLDGDALVARVDDAVRRVLT--VLLTND-
GTLPIAPTARRILLAGPAATHTDHLGAWVQHFAAPPAHSLADALTAALADDAQIAEVAAAAADADLVILALDEPSQLTGEATSRADLHLPGNQAALVHAVAATGTPLAV
VLVAGRPLVVEDW-IEEPGAVLMAWHLGTTAPEVIADVLTGAVNPSGRLPMGLPRHSGQLPADAHENTRPYRLELGPRFRFGHGLSY
>A0A4S8PYZ1_9ACTN
TLEEKCAQLASLWRGVQSIGHSEDEVIA-NGLGQLTRPFGPVDPEAGAARLAALQEKIKANSRFGIAALVHEECLAGFM--AHGATIFPTPLAWGATFDPELIGAVARQ
IGATMRQVGVHQGLAPVLDVVADSRWGRTEETMGEDPYLIGTIGSAYVTGLQESGIIATLKHFAGY--SASHAARNFGPVYLGERQLAETYLTPFEMAVIGRAGSVMAS
---YAANDGIPSHANQRLLTGILRDEWGFEGTVVADYFGVNFLDSLHHRGDAAALALTAGVDVELPNVDCY--GDPLIDAVGTGRLEVDVIDRAAERVLT-IVLLAND-
GILPLAAP-ARIALVGPVAAKAVMLGCYAFPNH----VTLAGALAADLPEDADIAAAVRAAAAAELAIVAVGDRSGLSGEGCDAASHALPGQQGELLDAVLATGTPTVV
VVISGRPYALGAAAEHPAAAIVQAFLPGQEGAGAISAVLTGSAEPEGRLPVGVPARPDSPPATYLGPERRVSVDPTPLFAFGHGLSY
>A0A433D7H7_9FUNG
---GNVPNNTRLAIPALHLQDGPQGVAGRVTNVTAFPSVLTVTAAWDPSLMELFASA
IAVEQRIKGANVMLSPMINIARIPVGGRNFESMGEDPYLAARLVASYVRGVQKNGVMACAKHWANN---NQEHSRMTVGTYIDERTEWEIYYPAFQAAVDADVASVMCA
---YNLVNGTYACENYKLLTVNLKERMGFKYFVMSDWF----------ATHSTVQSVNAGLDQEMPDRNFF-NPGALKQAISQGLVTTSRIDDMVRRIL--TVLLKNRR
GILPLEEKIASIAVIGDAAHNPVVVGGGSGSVHPPHISTPLDGIRSRAGKGGNMALVEDAARKANVVIVVVGAT---SAEGVDRPNLLLPLAQDAMVERVAAANPNTVV
VVYAPGAVLL-PW-KRKVAAIVCGFLPGQEAGDAIAEILFGDVNPSGKLPLTFPLSETQVATQY---PDNYDLKKQPLYPFGHGLSY
>A0A1L9T5R3_9EURO
---EFSYATSFPAPIVLGAAFNDDLVKTVAGI
ISTEARAFGLD-YWTPNINPFKDPRWGRGQETPGEDPLHCSRYVKGFVGGLQGDKVVAACKHLAAY-DLEDWGGVSRFEFDVSAVDLVEYYMPPFRTAVDAKVGAFMCS
---YNALNGVPACADRYLLQTVLREHYGWEGPVTGDCGAVERIQTHHHGPEAAAAALNAGVDLDCGTWLP----TYLGEAQAQGLVSNETLDTALTRLY--TVLLKNDD
RTLPLKRN-GTLALIGPYVNTTELQSNYAGPAHLGY------------TEDGFENALNIAAEADAVIFFGGIDNSIEEESLDRTSIDWPGNQQDLILQLAEVGKPLTV
VQFGGGQVDDSQLSADNIGAIVWVGYPSQAGGTAVFDILTGKVAPAGRLPVTQYPKEYEVPM--TDMNDNYRWYDEAVLPFGYGLHY
>A0A133UC01_9EURY
-LEEKVAQLGSVESDKGEFSPEKAEKALSNGIGQITRIAGGLNPKESAMVANQVQEFLVKNAPHSIPAMTHEECLSGYM--GKGGTTYPQSIGMASTWDPNLMEKITKE
IKKQLKAIGAHLALSPVSDMARDLRWGRVEETFGEDPYLVARMVTSYVKGLQGPGIYATLKHFGGH--SVPEGGRNHSPVNISPRELRENFLPFEATIKAKAGSVMNA
---YHDIDGIPCAASEELLTDILRGEWEFDGIVVSDYFSIGMLYTDHKLQEAGIKAMEAGIDVELPKTNCY--GEKLIEAVENGLISEAVIDEAVRRHLR--VLLKNEG
DLLPLNKNIDSVGVIGPNADTRNLLGDYAYNASAISIISILEGIKKKVSSRDGLEEAVEIAKKSDLAIVVLGGKSGVTGEGNDRTNLQLPGIQKELIKRMYETDVPVVV
ILVNGRPLATKWI-AEHVPAILKTWLPGEEGGNGIADILFGDYCPSGKLPVSIPESVGQLPV-HYRRKRNYVSRNEPLFPFGHGLSY
>F2N9A5_CORGP
---RITNLIQDYVKSHSRWGIPALIVEECPHGHQGL--DGISYPTNIGRGCMFNTDLIREGAHL
MGRELSCMGVDLALVSTLDLARDPRWGRTEECFGEEPYLSAKYSEAIVEGFQGRPVGAVLKHCIAQ--GDAQGGHNSGTVVIGDREFNDVYMPLMRAG-REAVG-IMAA
---YNDIDGVPCHSNTALLTDVLRTNVGFQGIVMADGIALDRLFGPYPISAAAAAALTAGVDMSLWDDAFL----HVDSAIKQNLTSELDLNRAVARVLS-MVLVKNN-

GTLPFDESGKSIAVLGPNADVYSMLGDYTAPQDDSLAATILHELRKISPSDEALDDALACAQASDAVVLCLGGSSERSGENVDVASLSLGGCQMQMAREVAKLGKPMVS
VLVQGRPYDIQEL-EQLSDAVLIAWYPGQSGGAAVARVLTGADNPSGKLSISYPRNASQLPV-YHHQRRSYLEPGSPLHPFGFGLSY
>W9W491_9EURO
--EEKIAQMGGIRRLLATFNRTAWEALYPLQHG-ILSYGSQLNQAQDVLPYANMVREEQLN-SSKVPWITVTDSVNSIY--VPGGTLFPATLSLSTSWNLPLYEEIVAS
IRDENMALGTHWVLSPELDIAKEPRNGRVGEMYGEDVYLVGEFAAQYVKTMQERKVATTVKHFL-Y--GQGSGGVNTASMDGGVNHLYNDLAIPYIRVLKEKPASIMIS
---YSSVDRIPMSMNTALIQDMLRSEMGFTGLIMSDAMGILHLYTESNYKDAAIKALRAGLQLELAPGQP------------------------------IVLLSND-
GFLPQ-ANFSQVALIGPFGD-IIDPGSYAPTTNPEYGRTLRGSLEARMGATAGIQEAVAVAKAAGVAIVSLGSLSVYDGEFYSHASLAFPGNQQQLLDAVLDTGVPTIL
VLNGGQAFVLNNS-TMRCNAILHQFLGGEFSADALVEIITGQVNPSGKLTISMPQADGAFPI-YYDFLDNCLRDGAP-MAFGYGLSY
>A0A1J4JE96_9EUKA
--EEKVNLCGGIDMWHTASVE-RLGVPNIMMSDGPHGLRKQVSEAVCFPAACATSCSFDKDILRKMGQT
LADEMIATDISILLGPDVNIKRSPLCGRNFEYFSEDPFLSTTLSGSLIEGIQSKGVGACIKHFAVN----NQEHRRLVSANVDERALREIYLASFEGAINAKPWAIMCS
---YNRVNGVYASDNEQLLNQILRKEWGYEGFVMSDWGAVND----------RVAGLKAGLDLQMPGPCKW-SIPVVIKAIKDGLLDEKYVDICAERVLN-IVLLKNEN
NVLPLKKT-QKIAFIGGFAKTPRYQGSGSSHINASKVTNAYDSSKVIVGEREKIDQAINLAKSSEVIVIFAGLPDAYESEGYDRAHINMPSNQNILIEEIAKVNNNVIV
ILHNGSPIAM-PW-ASSVSGILECFLGGQAVGEATANILFGITNPSGHLSETYPLRLEDNPSPGHDN--EYRYEKKHMFPFGHGLSY
>A0A4V5PN39_9DELT
TLEEKVGQMNQYIA-PPDPAGKIDPLLAQGLVGSFLF-------VTDASEANALQEKASRA-RLGIPLIFGIDAVHGLAPV-RGATIFPTPIGMAATFDVDLMERFGEV
TAHEMRVTGMHWAFSPVLDVARDPRWGRTAETFGEDPFVVAAMGSALVRGLQGPRVLACLKHFLGP--GLPLGGRNMGPIEVSERALRSTFLPPFQAGVEAGALSVMAA
---YNDVNGVPSHVSEELLTNVLRHEWGFRGFVVSDWEGIEMLHTTHHQKDAIRQAVLAGVDMHMHGEGFA---EPLVELVREGAVGAWRIDEAAGRILRVIVLLRNEQ
DLLPLRKNLKKVLVTGPNADNTALLGDWTAPQPAENVITVLEGIRAAVSPNEAIQRAADEARGAEVAIVVLGENETRGGEGADRADLTLVGRQMDLVRAIVETGTPTVV
VLVNGRPLAIPWI-AANVPAVLEAFEPGLAGGQAVAEVLFGDVSPSGRLPISIPRSVGQLPVHY-DHPRSYVESFEPLYGFGHGLS-
>A0A553F9M5_9GAMM
TLREKAGQMSQFVGLEGLRPADLLAMVEKSEIGSFLH-------VVNIEEANELQKHAMKS-RLGIPLIIGIDAIHGNA-LVRGATVYPSPISAASSFNLDLVKKSSVE
TAKEMRANGSHWTFTPNVDVVRDPRWGRVGETFGEDPFLVAKMGVATVEGLQQTKVIANAKHFVGG--GDSINGLNIAPLDVSERTLRQDYFPPFKEVLDAGVFTVMAA
---HNEVNGVPSHGSKFLLTDVLRGEWDFPGFVVSDWLDVDRLKTLHKHKDAVHLTVDSGMDMNMHGPQFA---GPIIELVEEGRLTEARIDASVKPIL--IVLLKNDN
NVLPL-KNVKNIFVTGPNADAHTTLGDWSLEQPEDNVTTILEGLQQVSGNDEQITEAAKRAKSADVSIVVVGENPLRSGENVARAELDLYGRQLELIKAVHAAGKPVIV
VLINGRPISE-PWLSENVDAIVEAWEPGSFGGQAVAEILYGKVNPSAKMPISVPYSVGHIQS-IYNHKKRYVSPTKNLYEFGFGLSY
>D8RVL3_SELML
---SATSFPQVILTVASFNSSLWNKIAQA
ISIEAIAMGL-TFWSPNINIFRDPRWGRGQETPGEDPLLSSKYAAYFVRGLQEGKVSSCCKHFTAY-DMEKSEGTDCFHAQVTVQDLQDTFDPPFRSCIDGQASGLMCS
---YNRVNGVPSCADYTFLTETVRNSWGFEGYIVSDCDAVALLYEYINAEDAVADVLSAGMDL--NCGTFL--LRHTAAAIEQGKVTEAAVDRALSNVMTVIVLLKNSG
NVLPFRNDLMTIAVIGPSGNTETMLGNYAGVPCQ--YITPFQGLQEYTKGTTLFLAAVRAAENSDAVVIVVGLDKDQEREGLDRTSLLLPGYQQDLVLEVSKVAKPVIL
VVMSGGPIDVTFAGNCKISSVLWVGYPGEAGGKAIARVIFGDHNPAGRLPMTWYPQAFAEHVNMHLRPNTYRYTGENVYEFGHGLSY
>A0A0W0FN73_9AGAR
--GV--RFADFTSFPTGLNTAATFNRTLIRQRGLF
IGLEHRGKGVNVALGPMMNILRVAEAGRNFEGFGADPFLAGEAAYETILGMQQGGVQACAKHFINKTHSEQETARTTSTSNIDDRTQHEIYAHPFLRSVMAGVASVMCS
---YNQVNGTYACENNSTLNGLLKNEFGFQGFVTSDWG---------ATHSTVA-STLSGLDMDMPGSGGPNGGGTLVEAVRNGSVPSSRIQDMATRVF--VVMLKNVN
NALPL-KKPRSLFLAGSDAGGVLGVGWGSGSTYFTYLISPYEAIERRAIEDFNLARAEIMAIGHSAALVFVNAD---SGEDSDRTNLTLWHQGESLIQAVAAQSNNTIV
IVHSVGPLIVESWDHPNITAVLWAGAPGQEAGNSIADVLYGDFNPSGRLPFTIAKRAEDYPAHVVNGSVSYRFDARNIFEFGFGLSY
>A0A242KB74_9ENTE
--SWKLANEVQDYVINHSRLGIPVLLVEECPHGHQGLGS--ISYPTNIGRGNSFNKELIEETARH
MAEELAMKGVHLALVSSLDLSRDPRWGRTEECYGEDPYLAAAFNHAIVNGFQGNQLGVVLKHCIAQ--GDGLGGHNSGAVNIGEREFMEIYY-PLLKSAKNAVG-IMAA
---YNDIDGVPCHTNQRLFEELLRKDIGYQGIVMADGTALDRLKPIYGDEKAAGKALQAGIDLSLWDNTYL----TIGAGIKQAVVQQAALDRAVYRMLS-MTLLKNE-
GVLPLQDIGEKIAVIGPNAHLYNQLGDYTAPQDEDVLDTIFTSIKQAFSHVTHMEKAVELAEKSDKIILVLGGSSARSGENVDVASLALGGKQLALFDCLSKLGKPIVT
LLIQGRPHEIEAI-CQKSDAVVAAWYPGQEGGPAVAQLLAGVVNPSGKLSISYPRSSGQLPV-YHYQRMNYDLPGSALYSFGYGKS-
>E4Q6A9_CALOW
TVEEKVYQLTSVLVKDNQFSEEKAKKAIPHGIGQITRVAGNFTPQQALEAANKIQKFLIENTRLKIPAIIHEESCSGFM--ASKATVFPQSIGVACTFDNELVKEMAKV
IRLQMKAVGAHQALAPLIDVARDARWGRVEETFGEDPYLVANMAVSYVEGIQGKKVIATGKHFVGY--AMSEGGMNWAPVHIPERELREVYLYPFEVAVVAGLKSIMPA
---YHEIDGIPCHANRKLLTEIARNEWGFDGIFVSDYSGVKNILDYHKYEEAAYISLWAGLDIELPRIECF--TEKFIEALKEGKFDMAVVDAAVKRVLE-MVLLKND-
GILPLEKDLKKVAVIGPNANVRNLLGDYSYPAKVVNIKSVYEVIKERIGKKSSFEEAKKAAQGADVVIVVVGDKAGLSGESRDRASLKLPGVQEELIEEIAKVNQNIVV
ILVNGRPVALENI-WQKSKAILEAWFPGEEGAEAIADVIFGKYNPGGKLAISFPRDVGQVPV-YYGHKGKYVMSSKPFLPFGYGLSY
>W3WW73_PESFW
---EKIAQLTGIGGLLVTYNTTLYDQLSSIHQGSISPG-SYLNYASAVPVIKDVIEEFTNNSRLHIPYVNIADSVNGVT-LLGT-TFFPATISMSMSWNLDLFKQAVTA
IRDEMVACGINWVLSPDLDPARDPRHGRVGETYGEDAFLNGEYGITYVETMQESKIATTVKHFLYP---TSVGGINSGSIDTGINNIFNVLAYPYIRVFKTTPASLMPS
---YASIDRVPSHANKGLLQDILRDTLGFKGVILSDADGVSGIYNQHKVYDAGARALEAGVQSVLAIQFPT----GFEEVINTPSL-APQVNEAVTNLLR-IVLLKND-
GLLPLPSTTSNVAVIGPLGD-KIIPGTYAAWTADNHNKTFVDALKGWLGENADIASAVTAATAAEIVILTLGAAT-VDGEGATHGSLTFPGLQEDLLAQVLAVGKPTIL
VISGGQAFELSGT-AQGASAIVHSFLAGEYTGQAVVDILRGLVNPSGKLTISFPNASPVNPI-YYDLLWSWPLTLPALYPFGFGLSY
>A0A4P8XIZ1_9BACL
----------DVELNEGSLEESCIEEIYKHGAGAIQLPFKTDSIETRIKKLNALQDYYVNKTRLGIPVMAQEECLHGHL--AKDATSFPIPIAMASTWDTELIERVYSA
IGKEARVRGGHEAHTPVLDLARDPRWGRTEETYGEDTYLVTRMGVAAVRGLQGTHVVSAPKHLAGY--AQSDGGRNFAPSNIPTRVLRDQILPPFKAVVEAGALGMMPS
---HNEIDGIPCHGNRQLLTEILRDEWGFNGIVVSDYFDASRLDILFHSKEAAVKALKAGLDMDLPGGGCY---TQLLDAIAEDPELEDVLNVTVARILRVITLLKNEG
GLLPLRNEIRKLAVIGPNAH-PICTGSYSTKPNK--GISILDGIIAKTNGEAWIVEAVSVAKESDVAILCVGGNTLTSREAIDRYDLDLPGVQNELVKRIVETGTPTVV
LLINGGPLTINYI-AQHVPAILEGWYLGEETGHAVADVLFGDVNPSGKLPITFPRSVGQLPV-YYSQKFKFAEHDEPLFSFGTGLSY
>A0A3N2DBE8_9MICO
-TREKVGQLNQRLFGWTRTGYVLSELLAHEHWGGIGAVYGNGIAPEAAAEVTRQLQDAVAVSRLAVPALIVEEAPHGHQGLGG--TLLPTTIGQAATFDPEWVAERAAA
VAAELASVGAHVALVSGLDLARDPRWGRSEECFGEDPLLAAELTGATVAAMQGEHVAVVVKHLAAQ--GAAAGGRNAASAVIGRRELHEIHLPPARSAVLADAAGFMSA
---YNDIDGVPCSANRDLLTGLLRQQWGSRGIVMSDMGALDRLAGPAGLVEAGALALNAGVDMSMGDVAFT----LLEEALARGLVNEAAIDLACERVLRLLVLVKND-
GALPLPPGIERVAVIGPNSQVGCLLGDYVPPLPAGDGVSIAEGLVQTLAAPGGIGRAVDLARWAEVVVLALGGSSRRGGEGADLADVRLPGAQRELVEAVAGSGTRIVT
VMVTGRAQGASPA-LELSDALLYAWYPGPDGGRVIAETLIGNDEPAGRMPVSVPRSSAELPV-TYDRRWSYLGPVSPETPFGAGLGY
>A0A4U0TLR0_9PEZI
--RLGYPGMCFNDAGNGVR--SQDGSAFSSGVSVGASWNAMLAYERGLY
MGAEFQRKGINVALGPVVGIGRVAEGGRNWEGFGADPYLDGMLAIPTVQGMQE-SVIACTKHFVAYEQQSTLNPLGLATANVNDKTMHELYLWPFEDAVNAGTGSIMCS
---YNRINGTYSCSNGETLNALLKGELGFNGFVVSDWA---------G-QHDGLPSAQGGLDVVMPSSVYW-DNDQLAKAVQNGTLSRERLENMAQRTL---VLVKNLD
GALPL-KSPRVMSLFGYDATGLLITGGGSGASNAPYISTPFDALSQRAISWNFEQTDPEVEGASDACLVFLNSY---SSEGWDRAGLTDE-QSDNLVLSVASKCGNTMV
FIHNVHIRLVDAWDHPNVTAVMYAHLPGQDAGRALASLIYGEVSPSGRMPYTVAKSASDYGDGSIND--TLARNVTPRYEFGYGLTY
>F0YZW8_9CLOT
TLKEKIGQMNQ-KMHGE-LTEAFAEEVAGDGVGAIYGVFRGIKVRDSVRVANTIQRYIRENTRLGIPVFLSEECPHGHE-ALQ-ATTFPTNIGIGASWNTELYEKVCNI
IARELRARGGHLGLISALDIAMDPRWGRTEECYSEDPFLAAAFCEKAVLGMQGDRVISVLKHFCAQ--GATIGGHNGKATNIGPRELFEIHLPGMKKGAKAGALGCMAA
---YNDIDGVPCHINRSLLTGILREQFGFTGFVMSDGRGVDRAKNITGYESACAAAVHAGVDLNLWNECFL----KLENAVRKNPLLEKDIDAAVLRILE-VVLLENKG
DVLPLGKEIKRIAVIGPNGDVYNQLGDYTQWKEEGEVVTVLQGLRKQAPSKDGFPSAISLAEDADAVVMVLGGSSTRCGEAVDLAELRLGGVQEDLAKEIKKFGKPLIV
VLIEGRPHAVSWM-KENADALLCAWYPGERGGDAVGEILFGRTNPSGRLPVSIPKSSAQLPV-YYNRKETYIESAKPLYPFGYGQSY
>A0A1S1Y3V4_9GAMM
TLEEKVGQMTQIDFSVPVDQAKLDDAIFNHHVGSILNTPTRAQPIEKWRNITQSIRDTAARSRLKIPVIYGIDAIHGAT-YTQNSVLFPQAISMAATFNPELSFKEGEI
VAREVKASGLDWNFSPVMDIGRQPLWPRLWETYGEDVHLAQTLGGAFIKGHQGEKAPTCLKHYVGY--SYPLNGKDRTPAWIGERALREWFLPPFEAGILAGAPSVMIN
---SAEVDGIPGHANYHYLTEILRGEMGFTGFTVSDWEDIIRLHTRDKPREAVKMAVMAGVDMSMVPFNFS-FYDLLLDLAKSGEVPLSRIDEAVARILRVIVLAKNDN

RMLPLSKTA-SILVTGPTANLSVMNGGWTITWYPKQYPTLLQAIQRKTSGEINIEQAVNEARKHDVVVLALGEKTYTETEG-NIDSLALDPVQLQLARAIFEIGKPVVL
VTFGGRPRIITEI-AEKARAVVLGFLPGMEGGAALADILFGDVNPSGKLPISYPRAVNDITP--YDHKEAYENQYRPLYPFGHGLSY
>A0A6H0Y5Y3_9PEZI
--VDRLKIPGFCLADAENGVRQTDF-VNAYPAGISVAASWNKDLAYWRGKY
MGAEFKRKGNHAALGPVVGIGRMAKDGRAFEGFGSDPFLAGKLAAHTIEGLQE-NVMAVVKHLIYNQETMRQEHGATPAISANVDDAHELYLWPFVDAVRADVAAVMSS
---YNRINGTYASQNNKTLNGLLKGELAFPGFVVTDWN-----SQHSGSESAV-----NGLDMAMPNSKGY-WQDALPAFVAAGNLTQERFDDMVTRTLT--VLVKNTN
NALPL-NQPKLLSLFGFDATGILWNGGGSGANHPSALSETYGAIQQRARRWNFLDPTVEVHPLSSACLVFINEL---TSEMFDRVTLAP-SIADAYVLNVASQCNNTIV
VTHNPAIRTVDAWENPNITAVVYAHFPGNDAGESLAQVLYGESSPSGRLPYTVAKNDTDYGS-LENPCTTLKNSITPRFAFGYGLTY
>R4XB27_TAPDE
---------------------NSPAWQRALNLTSLMTNEEKANITTGTGLVARCSGNTSPVARFNIPSLCFQDGPAGVRAVDG-TSAFAAQVNAASTWDIDLIYQQALA
MGAEFRGKGVNVALGPVAGLGRTPYNGRNWEGYGSDPYLHGIAAYHGVRGIQDNGVIATPKHFIAYGIGGPNTNAEQISSDLSERTLRELYLWPFEESLAAKPLAIMCS
---YNRINGTDACADGTLLNDLLKEELEFPGFVVSDWLSV---F----MAGSTNRTMNNGLDLDMPGGEGY-WGSDLVTQVSNGTIAQSRLDDAVTRIL---VLLKNVA
GGLPLKSG-ARLAIFGTDAGGTVALGSGSGSAHFPYLIDPLAAITGAADLAEILNEYDAAMSGADTCLVFVSSF---SGEGYDRTTLKFDNRGDQLIYYVASRCNNTVV
VSHIVGVTNFE-VSHPNVTAILNAGLPGQESGAALVSVLTGNTNPSGKLVYTILQNDNDYPVK-TASSDPYRADAMNLYPFGYGLSY
>A0A1Q2MA35_9GAMM
-TQEKIGQLALRDWGTASDMKAIKQAIREGRVGGFLNV---SFSAVDDEAFAELQRIAVEESPLGIPLLFGQDVIHGYE------TIFPIPLGQAASWNPELIKNGARV
AAQEASADGIRWTFAPMIDISRDPRWGRIAETLGEDPLLTSVLGVAMVEGFQTASLAACGKHFAGY--GAAEGGRDYNSAYIPERLLRDIYLPPFKAGIDAGMQSIMST
---YSTLNDVPGTGSPFLFKQILRDEWGFDGFVVSDWNAVMEMVPHGFAKHAATLAANAGIDMEMHTDTYE---QFFPQLMDEGKFSETQLDTAVANILRV-VLLKNDK
QLLPLKKG-QTVAVIGPLAEAHEQLGTWIYNGDKKYSHTLLPALREMVGDTRGFKSALKAARKADVILFVGGEEAILSGEGHSRGDIRLPGAQADLVAALAETGRPLAM
VLLAGRPLQLDDT-LEQADAVMMAWHPGTMAGPALADVLYGETSPSGRLPLSWPVGAGQIPI-YYNHLRPLDYGHKPLFPFGYGLTY
>A0A4R5TSP5_9GAMM
TLEEKLGQLNQPPGVGPEAMAGNEDQIRRGEIGSFFG-------THGVELTCRLQKIAVEETRLGIPLIFAYDVIHGHR------TLFPVPLGEAASFDVEEVRIAARH
AAVEASAHGIHWVFAPVLDVSRDPRWGRIVEGAGEDPYLGAVLATARVQGFQGDTVLATAKHFVAY--GAADGGRDYDTAEISERTLHEVYLPPFKAAVDAGVQSIMAA
---FNDVGGVPMHAHGGLINGLLRGQWGWDGVLVSDYTGIMELMPHGVRQEAGALGINAGVDIDLVSRIYY---EDLPAAIADGRVSMEQIDEAVRRMLN--VLLENDN
ATLPLSKSLPTLAVIGPLAERQAMLGNWAVAGRQEDVVTPLEGLQAALGERSGFDEAIRAAQQADAVVMFLGEHPDMSAEAHNRTSLDLPGAQEDLALAIAATGKPVVV
VLLNGRPLSIGAL-QGKVGAVLEAWFPGVEGGNAIADVLFGDVNPSGKLPVTFPRNVGQIPIHSHRNTRPYIVPWTPLYPFGHGLSY
>A0A5C6CW83_9BACT
TLEEKVAQMRMFHANLLILSEDVQNRLAQ-GIAGIKNPGEHLTPERAALLNNQLQKYTIENSRLNIPALFVTESYNGVDAHGS--TRFGRPINMAATWNVELTRSIWDA
IGREARLRGMHMCHSPEADIVRDPRFGRMSEAFGEDTYLTTEMIVAAVTGVQGGHIGAVTKHFAGY--GQVLGGTNFAAIEISPRTLRDEIFPPFQAAVRAHTLGIMAS
---HGDINGVASHANPWLLTEVLRGEWGFQGYVVSDSNDIARLHSFMKPEAAVKMAIKAGMNVDLYSDIAF---SLLPKMAKQDARLMKYIDRSVSHVLR-IILLKNKK
KTLPLKEKVGKVALLGPLLNT--KAAFEAAAGKGFKLTNEVRGVPELTKNEQAIENMVSMAQEADVAVLFLGGDEFTAKEAFDRDSIDPVGQQDELMRQVKAVGKPVVV
VLKHRRTLSISVI-AEEADAILDCWDLSEFGDEVLAKMMFGEFSPSGKLPVTVPRSIGQLPFHYSQKEYKYLSKPGPLFAFGHGLSY
>A0A167UWX7_9PEZI
--QDKVGQMGGVRRILLAFNQTSYDALTEYQN-GILGFGNRLNDPARVLQMANQLREDWAN-KSLVPFITVTDTINGPY--VEGGTLFPPTLSVAATFNVDLYGDVVAA
IRDENMALGTHWVLSPELDVPKDPRYGRVGETYGEDPLVVGRFGLKYVDTMQEAKVACTIKHFI-Y--GNPNGGINLASQYGGLNYLYNNLFPPFIQVIEAPPASVMVS
---YTSVDRVPMAVNKYLLQDHLRQNIGFRGVLMSDAGEVPNLYTLSLAETAALRALRAGMQLELAPSDAA--FPRLIDHVNETDV-ADLINQAVRQILE-IVLLQNEN
NTLPLRNGTGKIALLGPFAD-VVVAGTYAASNAT--NKTFGNALRQSLEAASGIAAAVAAARDAGLAIVNLGSLAVQDGELYTHADLGFPGLQQDLLDAVLDTGVPTVL
VITGGQAFALHNRTLSRTGAILHSFLAGEYTADALVEILVGKVNPSGKLPISMPESSGSVPVDYLPSDWTYPTSRAVKYAFGFGLSY
>G4QE37_GLANF
TLDEKLGQLSQVCSPGAHIPDYLAESIRQGRISSVIN-------EVDLNVNNALQRIAVEETRLGIPLLIGRDVIHGFK------TIFPIPLGQAATWSPEIVEKGARI
AAEESSKAGVNWTFAPMIDIARDPRWGRIAESLGEDPHLCSILGAAMVKGFQTDSIAACAKHFAGY--GASESGRDYNTANIPENELRNVYLPPFHQAAKVGAASFMAS
---FSDLNGVPATGNRWLLKQVLREEWNYQGVLVSDWESIKELQVHGLEKDSAYLAAKAGVDMEMASTCY---IDNMAALIAEHQIDEAEVDQMVLNILQ--VLLHNRS
PVLPIRESTKRIAVIGPLANGYEQLGTWIFDGEEKHSVTCLAGLEALANDTQHFDKALDLVNSADVAIVYLGEESILSGEAHSRANIDLPGAQPELIDYLSQSNTPIVL
VVLAGRPLILESL-LDKVDSILYAWHPGTMGGLAIAELLFGEASPSGKLPVSFPRVLGQIPI-YYAQKRPLDTHFSPLYPFGFGLSY
>A0A340YCS6_LIPVE
---EKIGQLRLISISPEMPREKIREEIAAGRIGGTFN-------SRTAPENRPMQDAAMRS-RLKIPMFFAYDTVHGER------TIFPIGLGMAATWDMDAVAKVGRT
AAIEAAADALDMTFAPMVDIARDPRWGRTSEGFGEDTYLTAKIGQVMVRAFQGSSIMAIVKHFALY--GAVEGGRDYNTVDMSLPKMYNDYLPPYRAAIDAGAGGVMVA
---LNSINGVPATSNTWLMNDVLRKEWGFKGVTISDHGAIQELIRHGVGREAAKLAIKAGIDMSMNDTLYG---EELPGLLKAGDVSQAELDQAVREVL--LVLLENRK
QTLPLSKDA-TIALVGPLADPIDMMGSWAADGRPNHSVTVREGLRRAIEGAVLIDEAVKAAQQADVVVAVVGESRGMSHESSSRTSLQVPANQRALIEALKATGKPLVL
VLMNGRPLSIGWE-REQADALLETWFAGTEGGNAIADVLFGDYNPSGKLPITFPRSVGQIPM-YYNHTRIYFEPNGPLYPFGYGLSY
>B8E050_DICTD
TLEEKIAQLQSVFGKEGNFSEEKAEKLLKNGIGQISRVAGGMDPERAVELANKIQKFLKEKTRLGIPAIIHEECLSGFM--AKGATVFPQAIGMASTFEPELIRRVSDV
IRQHMRAANVHQGLSPVLDIPRDPRWGRTEETFGEDPYLVSRMAAEYVKGLQGEGIIATVKHFTAY--GISEGARNLGPAKVGERELREVFLFPFEVAIEGQAGSLMNA
---YHEIDGVPCASSKFLLTKILRWEWGFKGYVVSDYIAIRMLENFHRAKEAAVLALEAGIDIELPSVDCY--GEPLIQAVKEGLISEEVINASVERVLR-IVLLKND-
GILPLSKNIRTVAVIGPNADPRNLHGDYSYTAC----ASILEGIKNKVSAKEGFDEAIEIAKRADVIIAVMGEESGLSGEGNDRTTLELFGIQRDLLRELHKLGKPIVL
VLVNGRPQALKWE-HENLNAILEAWYPGEEGGDAVADVIFGDYNPSGKLPISFPAVTGQVPV-YYNRKFTYVESAKPLYPFGHGLSY
>W2RR05_9EURO
---YATSFPQPITMAAAFDDDLIYAIGDV
ISTEARAFGLD-YWTPNINPFRDPRWGRGQETPGEDTYVVKRYIDNMVTALQGGKIIATCKHYTAYDMEFWQGNTRYGYDKVSPHDMSSYYMQPFVQCADTKVGSIMCS
---YNAINGVPACANGYNIETILRGHWNDENYITSDCTSIQNMYDHHARQQTVAAALNAGVDVDCGYYNPT----WLASAYSQGLFDEATLDTSLLRLY--IVLLKNDN
DILPLSDRNYTILMAGGWINTEQMQGIYAGPART--LVSPWMALQNVSNLWYESPLLVAERLQPDLILWIDATN-EGAEETEDRNTIKWDLMQVDALEMLALTGIPTVA
VHMGEQCDDAAILANDNISALVWAGYPGMLGGQALVNVLLGDAAPAARMPLTQYPTDYHLP--MTDMGRPYKWYDNATIDFGYGLHY
>A0A0J1FYP4_9FIRM
-LEEKINQMLQVTGDFMRENGFTEESVA--QAGSVIG-------LAGADVVKKVQKEYMEKQPHHIPLLFMLDVINGYK------TVFPIPLGQGAAFEPEMSKSCAKA
AAKEAAVSGLHITFAPMVDLVRDARWGRVMESTGEDTYLNCCFSKAMVEGFQGSRVAACIKHFAGY--GAPDAGRDYNTVELSEHTLREFYLPAYQAGIEAGSALVMTS
---FNTIDGVPATGNKWLMRDILREEMGFDGVLISDWAAMEEIIYHGYRLDAAKRSIEAGVDIDMMTGIYS---ERLQELIEKGKVEERLIDEAAMRILEL-VLLKNE-
GALPVEKEQ-KIAFIGPYTDSREILGAWSFIGKSEDAVSIKDAAMEVLDQAEMLNTAIKAAKEADVVIMPLGEHRLQSGEACSRAEIVIPEVQMDLFRKICQVQPNVVV
VLFNGRPLDIREI-SQKAKAVLEVWMPGTEGGHAIMDVLTGEYNPSGKLTMSFPYSVGQVPVHYNEYSRPYLIPNAPLYPFGYGLSY
>A0A175W7P6_9PEZI
--EFSAATQFPQAITTSAAFDDPLIERMGVI
ISTEARAFAAHLDWTPNVNPFRDPRWGRGHETPGEDAFRNKKWADAFIKGMQGPRVVATCKHYAAYDLENSGSTTRFNFNRVSTQDLSEYYLPPFQQCADSKVGSIMCA
---YNAVNGVPACADSYLMDTVLRKHWNDNQYIVSDCDAVYYLGNANGYAAAIGASMEAGCDNMCWATGGT--TPNAAAAFNARQFSQATLDR--------IVLLKND-
GVLPIDGSNSQVAMIGFWANADKMLGGYSGSPR----HDPVTAARSMGINYADTSAAVNAAQRSSVVIFFGGIDNTVEKESQDRTSISWPSGQLNMIQRLAELGKPVIV
VRMGTHVDDT-PLSLPNVKAILWAGYPGQDGGTAIMNLITGKASPAGRLPITVYPSSYTNQAPYTNMARPYRWYKNAVFPFGHGLHY
>A0A428WPZ1_9ACTN
TLTEKIGQLNQ-RLYGAATDQLTAEARRYGGIGAIYGLQRGVTAAEGAELCATIQRAVTAESRLGIPALFVEEVPHGHQ--ALDGTVLPVALAVASTWDPDLYERACRD
VAAEVRARGAHVALVSTLDILRDPRWGRAEETFGEDPYLAAAFTTAAVRGMQGGRLAVVLKHAAGQ--GATVGGRNWAATELGWRELAEIHLPPVRAAAEAGAAGLMAA
---YSEVDGLPVAANRRLLTEVIRGDLGFAGLVMADGTALDRLLRLTGPASAAAMALRAGIDLSLWDEVY----PHLAAAVERGLVAETAIDRAVERVLA--TLLTDD-
GLLPLTGK--RVAVLGPHADVAHALGDYTAPQRPKTGVSIAEALCAAGVRESGIPAAVAMARDADVAVLCLGGSSARAGEGVDLAHLRLGAGQLELLRRTAATGTPVVA
VVVQGRPHVLGEV-LATAGACLAVWYPGPTLGTAVAGVLLGRREARGRLPVSLPRDAASLPV-HYNHRDHYLAPPGPALPFGAGLG-
>A0A512HBQ3_9PROT
-LAEKLGQLNLLTA-SLPIGKERADEIRAGRVGGLLGGLFS-FGVGGPRALREVQEIAVHESRLGIPLLLAYDVIHGHQ------TVFPIPLALSCAWDMAVVETAARW
AAREASAQGLNWVFSPMVDIGRDPRWGRIAEGPGEDPYLGACVARAMVKGLQGDTVMACVKHFVGY--GAAEGGRDYNNADMSPARLREVYLPPFQAAVAAGAGSAMLS
---FLALNGMPSHADAALIEECLGGLLTFDGI--------PEMIAHGLLQTLAERSLRAGVHMDMMGHAYA---TRLEAALAAGRVTQAHIDAACRAVLR--VLLQNKG

DLLPLPRQTLNLAVIGPLADTANILGPWSFQGDPGTAVSVLEGIRQAAGPETLIAEAVAVASAADVVVAVMGEASEMSGEAASRQDLGLPGGQRRLLEALHDTGTPIVL
VVMNGRPLTLSWE-TDHLPALLVAWFGGCEAGAAIAELLFGDRAPSGRLSTTWPRQVGQIPL-SYDHARPYLGPSTPLFPFGFGLGY
>A0A1G7L574_9SPHN
TIEEKAGQLSCFND-EAQGAAAQLADIRAGRIGMLFNG-------YGAQGAIRAQEAALAS-RLRIPLLFAADLIHGCR------TIFPIPLGEAAAFDGDLSRRVARA
VAVEARAAGIHWTFAPVVDVARDQRWGRVAEGAGEDVALNVALAVARVRGFQGAALAATPKHFAGY--AEVRGGMEYGAVDMSDAQLREVVLPPFAAAFRAGAAATMAA
---FTAFNGIPATANRHLLTDILREDLGFTGVCVSDYDADRELIAHGVEADAARLAILAGIDMSMQSGLFQ---RHLPALVASGAVPIAVVDRAVARVLA-IVLLRNEG
AVLPLAPAT-RVALIGPFADRTHLNGPWSFAGRAEDGVDLATAIRPLVVEPGGIDRAVAAARAADVVLLAIGEGADMSGEGNSRVAITVPAAQQALAEAVAATGKPLVI
LLRHGRALALEGA-VRDAPAILATWFLGAQTGHAVADILFGRAEPSGRLPVSFPFATGQQPW-SYDRPRPWRAPDRALFPFGAGLSY
>A0A1J4JN71_9EUKA
TIEEKVGQLVQLPGYAMFMHGPCEQAIKDQHVGSFLW--------VVGENAK-IPINLQKETRLKIPLLFGVDAIHGLS--FIDGTLFPTQLGMAPSWDEDLLKEVAST
TAKEMRYNGTHWTFSPVLCIARDLRWGRVGETFGEDPYLIGRFASAMIKGYQGDKILACAKHYAGY--SETIGGRDASEADLTPRKLLSYFLPQFKKACEAGVGTYMTG
---YQSIDGTPSTANQWLLREILKEEWGFKGFLVTDWYNVGWLVHDQKFEEAAAVAITCGNDMIMATPEFY---EGCLKALKSGKLEEKYVDEACERILR--VLLKND-
NILPLASSLKKAAVVGPNADGAQNGDWAAGTSPRENCKTVLDGVKANFQGTSDFSKAIEYVNDADVTIVVVGDRERYWGESKSTCTLELQGDQIDFLNKIVETGKPFIL
DIISSKPLVIPANIRNNARAIIQQFSPGQMGGQALANIVFGKYNPSGRLTISIPYHVGQQPI-YYNQVQHYAMTQDPCWAFGYGLSY
>A0A1I2VTE1_9EURY
TLEEKAGQLAGTYVGKEKTLEDAEREITEDHIGAVSPFGITLDDPARAAEVNRLQRHAVEETRLGIPLSVPVDAVHGHA-YINGATVFPHNVGMAATRDPTLVERGGEI
TGREARATGATLNYGPTLDVARDQRWGRVFETYGESSYLCGELAAAEARGLRGDTIAATAKHFPAY--GQPERGEDASVVEISESTFRRTFLPAFERVLDEGVDAVMPC
---YNSIDGEPAHGSERFLTDLLRGELGFDGFVTSDWGGVDHLHEDHGRRTSAWQAFTAGLDLVSVGGPDY--AEEVVELVRKEELSERRLDESVRRILELMTLLKND-
GVLPLSESLDDVLVTGPNADLHHQFGGWSVMDDSDQGVTVREGIEDVVGEEADVDAAADAAEDADAAVVVLGEDWYITGDFPTRTQLELPEAQQNLLEAVQATETPTVL
VLVSGRPLSI-PWADENVPGILTAYYPGMDGGTAIAETVFGRHNPSGRLPVSVPRSAGHLPTDYLPHP-THPDSYDPLYPFGHGLSY
>A0A1Y1WEX9_9FUNG
TLEEKIGQMTQIEVGQIDCNGKLNRTASPGNHDGIYQ----WYSAEKFAELTDAVQEIATKKGAKIPVIWGLDSVRGAN-YVKGAVIFPAGIATAATFNPQFAYDAGRV
AAKDTRAAGAHWAFAPVLDIGVNKFWSRTYENFGEDPFLSSQMA------LIRSRVAACMKHFIAY--GNPFDGSDRADRHVADHELLEYYVPSFKAAIDAGSATAMES
---YGAVSGETVMLSNFYLQTLLREHLGFKGFMVTDWGEILSQATIYMPEQAVEGALRTSTDMSMVADDAS--F---------------------------ITLLKNEN
SVLPL-KTSESVLFLGPTLNSSRYMGGGWNEGYQGFADTVLTGIKQVTGSRYDWDGVIAAAKKADKVVIGLGERT--YAENFNIDELTLPQAHIDLVSSSLCWSKDAP-
-V-SSAASLMT------PNAIVNAYLPGSYGGLPIAEILYGKVNPSGRLPYTYPAHSSQASHTIWQASY---VPYKPQWQFGFGLGY
>A0A239A3G8_9PROT
------------------MTLDEKKLLVFGHFGSALK-YKNYLPPKEVRMGSAGYVAGIKR--LGIPPQYLTDAGVGVATQREERTSLPAGIATAATWNPALAEQGGAM
IGREARASGFNVMLAGGINLVREPRNGRNFEYGGEDPWLAATMVAAQIKGIQSNQIISTIKHFALN---AQETGRFVLDARIAEDQARMSDLLAFEIAIEKGPHSVMCA
---YNRYNGPYACESDFLLNQVLKKDWAYPGYVMSDWGAVHSTA----PAANAGLDQESG---YIFDKKPYFGPELLGKALKDGSIPQARLDDMVRRILR-IVLLKNDA
NLLPLAASAKRIAVIGGHADKGVLSGGGSSTVIVYHPSAPLEAIRRRSGDGTDVAAAAKLAAESDLVILFANQW---TGEALDF-PLTLPDGQDALIAAVAKANPKIVV
VLQSGGPVLM-PW-LAQVPAVLEAWYPGTRGGEAIARVLYGEVDATGRLPVTFPQSLDQLPRDGTDKK-PFDKGHKPLFAFGHGLSY
>A0A0W1A3I4_9GAMM
TLAEKLGQLNLLTVGLPVSNQDIDNKIKNGQVGALMN-------AYSPKVVKQLQDLAINNSRLKIPLLFGFDVIHGYR------TVFPIPLAQASSWNPELIKKLAHA
SAAEAAQDGLHWTFSPMVDIVRDPRWGRVMESAGEDPYLAAQIGEAMVKGYQGTSIMACVKHFALY--GGAEAGRDYNTVDMSLPRMYNEYLPPYVAAIKANAKSVMTS
---FNDVNGIPATGNYWLMNKLLRDTYHFDGLLVTDYTAINEMMAHGVDQEVTRLAMNASVDMDMVGEMYV---NQLPKLVNNKSIKEEQIDAAVKRVLR-MVLLKNNN
HILPLNPKQ-KIAFIGPFVKANQQLGEWRAIGRPEEATSLWQALQKDQDAQKLLNEALSIAKKSDVVIAVLGEPFGMSGEAASRTRIGLLPNQSDLLKELKKLDKPLVL
VLMNGRPLTLEWP-HHHVDAIIEAWYGGSEAGPALADILFGRYNPSGKLTMTFPRHVGQIPI-YYNAKRPYIESNLPRYPFGYGLSY
>A0A553FCL0_9GAMM
-IEEKVGQMTQLDVREVVKAEKLKKIIHKYKAGSILNAAGQPMNIEQWGSLISAIQDEALSTEHQIPILYGIDSIHGVTYTAGS-TLFPHNIGLAASLDLELSNQIAKV
TAMETRASGIRWNFDPVLDIGRQPLWSRFEETFGEDPYIVEQMAVGMVKAYEQDAVASTMKHFVGY--SAPDNGKDRTPATISDKDLWEYYLPQFQAAVDAGSSTIMIN
---SASINGVPVHASKHLLQDVLRDKMGFKGLIVTDWEDIIRLHTRHRPREAVKIAIDAGIDMSMVPNEMS-FFEHLTDLVKSGEISEQRINESVAIILRLMTLLKNET
SILPLTKN-DKVLIAGPGANLGPLHGSWSYSWYPESTLTLVEAFQAEVGANYDTDGLAKAAENVDYIVLALGEGAYAESPG-ALDDLTLDKNQIELAKAAALTGKPVIL
VLLQGRPRIVKEI-EPAMKAILLAYRPGSKGAEAIVDTLYGKHNPSGVLPFSYPQFTGDH--STYDRNTNVSNGHKPQWKFGFGLSY
>E0STZ1_IGNAA
-IEEKIAQLISIPLESKKFSVEKAREVLKYGVGEILRIGGRLSPREAVEIYNAIQRFLTRETRLGIPAIVHEESIAGLL--APTATVFPIPLALASTWDPDLVYRVAVA
IRRQIMAIGSRHTLAPVLDLCREPRWGRCEETYGEDSYLAASMGIAYVKGIQGDGVIATGKHFVGH--GVPEGGRNIASIHVGLRELLEIYMYPFEATVEANLLSIMPA
---YHDIDNVPCHANKWLLTDILRGSWGFKGIAVSDYEGVKQLHTIHRCMEAAVKAIKAGVDIEYPSGECF---KQLVEAVRKGLIDEDTINRAVERVLKLIVLLKND-
GILPLKRDIKTIAVIGPNANPWAMLGDYHYDAPSVRIVTVLEAIKSRVSPRSGFGEAIEIAKRADIIIAVMGDRSGLSGEGVDRASLKLPGVQEELLKELASLGKPIIL
VLINGRPLALSSI-LPYVNAIVEAWRPGEEGGNAIADILFGDYSPGGRLPVSLPYDVGQLPI-YYSRKRDYVYPAKPLFPFGYGLSY
>F7PQA1_9EURY
---EKVGQLVGTA--PRETVSGIAEAVTEHHLGAVSPFGHPWETPEECVEVAAIQREARNTTRLGIPVLFYVDADHGHG-FVKGTTVFPHNLGMAATRDPELVERAASV
TATEVAATGGHQNLNPVADVGREARWGRIYETFGESPHLCASMSAAAVRGYQGDSVIATPKHFPAY--SDPVRGEDGSPVDVSEYTLRRVFRPPFEAALDAGAGSIMPA
---YNELNGYPVHGSKEYLTGWLREELDFSGYVVSDWNGINMLHHDHRMEEAVWQATTAGVDVASVGGVEH--AERLLDLLESGDIAESRIDESVRRVL--MTLLQNDD
DVLPLDPDLDSIAVLGPNADLRNQFGGWSTISPEPPGTTIREGIERAVPARVDVDAAREAASASEAAVVVVGETGYRRGEFPTRSELELPAAQRELLGAVRETGTPTVA
VFVAGRPLAMGWT-VEHVPAILFAYLPGSEGGKAVADVLFGAADPGGSLPVSIPRSSGHLPTDYRPHPHPHPETYDPLFPFGHGLSY
>R7TKV4_CAPTE
---ERLGIPYVWITECLAGQVNT--NATAYPQPIGMAASFSEEAHWNANRA
VGKYSTKVGLSC-FSPVINIMRHPLWGRNQETYGEDPLLSGTLAQSFVRGLQGDRANAGCKHFDVHGG-PEDIPVSRFSFDVNMRDWRMTFLPQFKMCVDAGSYSLMCS
---YNRINGIPACANKQLLTDITRDEWGFHGYIVSDSGAISNIKEQHHTVATVVAAIKAGTNLELGGGSNM-YYPKQLDAMKQGLLTEKEIRDNVRP-----VLLKNHN
NLLPIKKQYSKLAIVGPFTNTSELFGTYSSEVNLKFTSTIFEGLSPLGGSGYVRDDVETAVAGADLVIVALGSGQRFESEGNDRAYLDLHGHQLDILKDAVSNGAPVIL
VLINAGPLDITWALDPGVTAILSCGYPAQSTGEALRRSLTMQAAPAGRLQATWPLNLDQVPKDYTMQGRTYRYVGEPLYPFGFGLSY
>V6DQS2_9EURY
TLAEKAGQVTGTWAGTTHDLDDVKEAIDDGHLGFAAPFGWATTPEDAVSAVEELQTYAREETRLGIPLLFSVDAVHGHAYVSG-ATVFPNGLGAAATWDPEGVEAAAAV
TAREVRATGAHQNYGPTVDVGRDARWGRVFETFGESPHLVGALAAAKVRGYQGKRVVATAKHFPAY--GEPERGEDASPVDVSEYKLRNTFVRPFERVLDAGVASVMPS
---YNSINGEPSHGSAAYLDRLLRDELGFDGHVVSDWNGIRHLHEDHRHADGVRQARTAGVDVASVGHTPH--ADRVVELIEDDRLDEATLERAVRRVLRLMTLLQND-
GLLPLSGD-EDVFVGGPNADLVNQLGGWSVGRDE---GVPGKTIREAVADAIDIDEAVRAARDADVAVLALGEGWYITGEWPTRSDLRLPESQRELVRRVHETETPVVG
VLVTGRPLIVDWM-AEHVPAILMAYFPGTEGGVAVAETLFGDNDPSGRLPISVPRDEGDLPQD---ALHPHPDSYDPLFEFGHGLSY
>A0A5C5BFD0_9MICO
-LPELVGQLHQPA---NVDLERDAALLAAGAIGSTLHASGNVRDGVSRDRVDDLQRAAIESSRLGIPLLIARDVIHGHR------TVAPIPLGQAATFDEQVVHDVAAR
AALEASADGLTWTFAPMLDVVDDPRWGRVAESFGESPLLTARLGAAAVRGFQASLVTACAKHYVGY--GLSRGGRDYATAEVGEITLRNRHLVPFRAAVDAGVGTVMAA
---FCDVDGVPMHSHHHLLREVLKGEWGFDGVVVADWNGIGELVEHGVLRDAARLAIEAGVDVDMVSGAYA---AHLAALVEGGEVDLELVRDAARRVLRL-VVLRND-
GMLPLPRPTDTVLLTGAYTRRASLLGTWVLDGDPEQVSAVAPAVVRALGEGAFADRTLRLARDADLTVAFVGEHAARSGEDGSTSDVGLPPGQLEVLRGIAALGSRLVV
VVLTGRPLALGEV-LDLADAVVLAWHPGTEAGEALADVLVRGVPATGRLPMSLPRSVGHLPI-THAE--------------------
>A0A395JNH3_9GAMM
TLAEKAGQMNQYN--GEGDAKNKYEHIRTGMVGSVLN-------ITGTKDVRATQKLAVEGSRLGIPLIFALDVIHGHQ------TVFPIPLAEAASWDLNAIEKSARI
GAIEASARGINWTFAPMVDISRDARWGRVMEGAGEDTFLGQKVAVARVRGFQGDTIAATAKHFAGY--GFGEAGREYNRVDVGTNTLYNMILPPFKASVDAGVASVMNA
---FNTLNGIPATGDVFLQRGILKGAWDFNGLVVSDWGSGKEMMQHGFLKHAAQLGVTAGSDVDMESYMYV---KHLEALVNEGKVDIALVDDAVRRILRLIVLLKNQG
NLLPLSKTSGKIAVIGDFADKNSPLGSWRFGSIDNSAVSLLEGLAKYRDDATGIDEAVALAKKSDVVVMLLGEHGFQSGEGRSRTDLGLPGLQQEMLEAVYAVNPNIVL
LIASGRPLAL-PWAAEHIPSIAATWQLGSQSGNAISEVLFGDYNPSGKLPVSFPYNVGQMPL-YYNRMNTYSSPSEALFPFGHGLSY
>B8E3C1_DICTD
---------------------------KKYPIGELSCALRSMSPRESAEFANEIQKYVLENSEIKIPVLIHDEALHGCMAKGS--TIFPQAIGMASTWNPELIYQVATA
IGKETRSRGIHQVLSPTINIARDPRCGRTEETYGEDPYLASRMAVAYIKGVQEQGVIATPKHFAAN--FVGDGGRDSYPIHFSERLLREVYFPAFKASIEAGALSLMAA
---YNSLDGIPCSSNKWLLTDVLRKEWGFKGYVVSDYFSVLHLMTKHKKAEAARLALEAGLDMELPDSDCF---EEMINLVKGGKLSEETINEAVRRILG-IVLLKNE-

GILPLSKDIGSIAVIGPNAA-VPRLGGYSGYGVK--IVTPLEGIKNKMENKSGFDEAIKIAQKSDVAILFVGNSPETEGEQRDRHNLNLPGVQEELIKEICNTNTPVIV
VLINGSAITMMNW-IDKVQAVIEAWYPGEEGGNAIADVLFGDYNPGGKLPITFPKYSSQLPL-YYNHKRVVDRSPQYLFPFGYGLSY
>F7B5X2_CIOIN
--RLGIPYQWNTECLRGYA-MNGDATCFPQPIGLAATFDQGLIYKLAKT
IALEARAKHNNFTFSPVINILRHPLWGRNQETFGEDPVMSSLMARAYVTGLQGDPATANCKHFAAYGPENIPSSRLSFNANVSIEDLGRTYFPAFRECVHSGAFGIVCS
---YNAINGEPACASSYLQ-TILRDKFNFKGYVSSDESAIEFFDIYFKNLLSAVAAFDAGVDLELTSYGKNNRYSLLNQAVEQGLVTEAALRRSAKRLFR--VLLKND-
GVLPLNHRYENVAIVGPFINSEALTGDYHPNYNLKYFSSPLFAANSLSSSTYNSTHVKEVVTGSDIVLVTLGTGTGIEAEGRDRLSMELPGKQLDMIKDVVYANGPVIV
VLFNAGPLDVSWV-MGNTAAVIACHFSAQMTGEAMLEVLTGIVNPAGRLPNTWPASMQQVPPDYSMHERTYRSTSSPLFPFGYGLSY
>F8DDY5_HALXS
TLREKAGQLSGLFASESYTLDDVEHAVREHAIGSVTPFATSHNSPAVVRIANRLQRIAREETRLGIPLLVPVDAVHGHANVKG-ATVFPHNLGMAATWNPALIRRAARA
TAAEMRATGATMNYSPNADVAREPRWGRTYETYGESPHLVGELAAAEVAGLQNAAVAATVKHFPAY--SAPARGEDAAPVDISPSTLHRVFVPPFDRAIDAGVAAVMPT
---YSAVDGEPAHGSRRYLTSLLRDDLGFDGLVQSDWHGVAFLYDRHRPKEAAAQAVGAGLDVASIGGPEY--AKHLCELVESGRLSEERLDESVRRVLELVVLLDND-
DALPF-NDPDEVLVTGPNADDALCGGWTVADLAADHGTTILEGLSNATDDEINIEAAADAAVGADAAVVVCGENWYVP---NNRTQLRLPDAQRRLLERVADTGTPTVL
VVATGRPLAIPDE-VQVADATLAAFYPGYEAGQAIGEILIGATNPSGRLPISMPRSISQLPL-VHDHRHPHPDAYDPLFAFGHGLSY
>Q8Y3Q3_LISMO
TLAEKCGQLNQ-RMYGQ-ITEKFKEEVTREGIGALYGLFRGVSRKNAGKVANKIQRYVIENTRLGIPVLLAEEVPHGHQALDS--ESYPVNLARAASFNPELQKQVASA
ITEEISEKGVHLALASALDVLRDPRWGRAEECYGEDPYLAAELTAAITEGFQASKIAVILKHFAAQ--GEPIGGHNSGPVSIGVRELREIFLDPMRAGIRSGALGVMAA
---YNEIDGVPCHANKELLTTILREEMGFSGIVMADGCALDRLLKLNPPKKAAKMALEAGVDLSLWDEVF----PFLEESVEKGILDEKIVDDAVSRVLQVICLLKNDF
ETLPLVGEPKKIAVVGPSIDLYNQLGDYTAPQNESECVTVLEGIKNQLPEPDGIQRAEVVAKEADAIVMVLGGSSARAGENVDVADITLPQPQLDLFYAMKRTGKPVIV
VMIQGRPIAIPEI-SLAADAILTAWYPGSLGGTAIAEVLFGHYNPSGKLPVSIPRSSGQIPI-YYNQKYKYFLTGKPLYPFGYGLSY
>A0A0J1G1G5_9FIRM
---------------DNQKNRKEDSMMRAEKVLECRRRAKELVGKMTLEKVSQTVHSAAGIERLGIKAYWWNEALHGVA-RAGVATVFPQAVGMAASFDEEAISTEGRA
MQQEFGDLDIYTFWAPNVNIFRDPRWGRGHETFGEDPYLTSRLGVRFIEGLQGDKTAACAKHFAVH--SGPEDERHSFDAKVNARDLFETYLPAFEACVEGKVEAVMGA
---YNRTNGEPCCGSRTLLTEILRKGWGFEGHVTSDCWAVKDFHEYHKALESAAMAMENGCDLNCGS--L---YLYLTEAVKRGMVTEERLDEAVTNLLS--VLLKNEG
NLLPLKNKINTIGIIGPNANRKALVGNYEGTASR--YYTISEGIQDYVGDNDRLAEVRAVCAHSDVVVACLGLDSSLEGEEGDKPDLALPGLQQDILRTAWESGKPVVL
VLLSGSALAVSWA-DEHIPAIIEGWYPGAMGGKAMASVIFGDYSPEGKLPVTFYRTTEELPDRDYSMERTYRMKQKALYPFGYGLSY
>F0Z224_9CLOT
---------------------------------------GSYNGINDAKTANYLQKIAVEETRLGIPLLFGYDVVHGYR------TIFPIPLAESCAWEPALWEKTARA
AAKEATAGGNHITFAPVVDVAKDARWGRISEGAGEDAYLTSAFGQAKVKGFQGESMAACIKHFAAY--GAAEAGRDYNRVDMSTQRLFEEYLPPFRACIEAGARVVMPA
---FNDINGVPCTASQWLMKELLRKQWGFDGMTISDANAIAECVEHGIRMDAAWQAICAGVDMDMASDCYS---QCLEDLIESGKLDSAILDEAVANILR-MVLLKND-
GILPLKPDV-KLGIVGALADRSEMMGAWAIRGDGNDCISIVDACRAQNKTCVDETEVLRIADECDVILAVVGEYKNQSGEAASRADISLSDTHMKLLHVLKGTGKPVVA
VLFNGRPLAI-PWLKENLSGILEAWHPGVEAGNAVLDILYGCVNPSGKLTTTFPYTSGQCPV-YYSHINTYLTPVEPVYPFGYGLSY
>A0A416ET44_9FIRM
TLREKVGQLNQ-KIYGFEITDIFREEVAYDGVGAIYAPFRGVSAKDSAKVINMLQKYLLEHTRLGIPALISEECPHGHE--ALDGTSIPVNVGIGSTFNPQLYQEAAKC
IAAQIRARGCTLGLVSCLDILQEPRWGRSEESFGEDPYLAARMCENVVRGFQGEKIIAVLKHLCGFG--NPIGAHSGICSNIGEREMREIHLQGMKAGVRAGALGCMAA
---YNDIDGVPCHMNEKLLKDIIRGEYGFQGIVMSDGCAIDMSEAQYGYENAGAAALNAGIDLNLWNQSFL----ALQDAVEHGKVKEDDIDTAVRHVLY-VVLLENKE
QLLPLGKDTGTIAVIGPNAHIYNLLGDFTSWKKEEDVYTILDGIREVFSDREELEAAVKAAEMSDAVILALGGSSARTGEGRDIAELNIGQVQRELAEAIFKVGKPVIV
LNISGRPFSLDWA-K-PANALLQVWYPGEMGGLAVARILAGEVCPSGRLSVSIPKSSGQLPV-YYNHKRNFTMSPEPSYSFGYGLSY
>A0A0R2ESD2_9LACO
TIEEKIGQLVQLSG--EFYNASDISMGPQRKLGQMVDLSGSVLNVAGAKQIHRLQEAYLAKGGHAIPLLFMSDIVYGYK------TVYPIPLGMGASWDPRLIKRAYEN
AAEEAYAGGQQVAFAPMVDLVHDARWGRVLESTGEDPYLNAQFAKAMVGGFQSGGIVSCVKHFAGY--GAVEAGREYNSADMSMSNLYQNYLPSYKAAIQAGAKMVMTS
---LTTLNGVPATADKWLLNDLLRKQWGFTGVVISDYASIYELTQHGFEQDAAYKALDAGVDIDMKSPCYA---NGLKPLLEDGRLSEEKIDAAVWRVLSLMVLLKNER
HLLPLRSGHRRVALIGPYADESKMLGLWAIHGYPKDTITFKHGLSEYLDTKKHHDSAVAAAKRADIIIFAGGEHTLQSGEGAGSRTKLRLPDNQQRLLDELATLGKPIIS
VIISGRPLVLTDV-LADSDAVIQAWFPGIEGGHALADIIFGAYNPSGRLSMSMPAVEGQAPI-YYNHLRPYTAPTGPLFPFGYGLSY
>A0A5N6EL41_9EURO
--TRLGIPKMCFQDGPGGVRPSLGN-TQFPSSVTTAATWDVDLIYARSYA
MGKEFYDMGVHVAMAMVTGLGRSPYGGRNWEGWYADPYGTGIASWYGVKGMMDSGVQTCSKHFGFYEQETYNASEQLPVSNVDDKTSHEIYLWSFAEAVRAGTTHIMCA
---YNCINGTHACANSETNNGLLKGELNFQGAVISDWGGVWGTQGFA----------MGGLDVNMPGRGYGNKDQELYHLVSNGTIAEARLNDMVARVVT-TVLLKNT-
GGLPL-KSPQNIAVIGQDAGGTLSLGGGSGYAWPLNLITPLDAIQAAGLEAETVTGSALPIGPPDVCLVFADRY---MRENMDRNDLSLNTHSEDIIVQTAATCNNTVV
VLHVGGPVIMEAWDNPNVTAVVAPLFPGEQTGPGLVDILWGRVSPSAKLPFTIAKQESDYPPISYDPSTPYRFDTYNIFEFGFGLTY
>A0A086MCX5_9HYPH
TLEEKVGQLNLFSH----GPPLRWEDISEGKAGALLNF-------NSAQDVARAQA-LARQSRLKIPPLFGLDVLHGFR------TQFPLPLGEAAAFSPRISRLASEW
GAREAAYVGVNWTFAPMADLSRDSRWGRIVEGFGEDPYLGSVLTAARVEGFRQGGLAASTKHFAGY--GAPQGGRDYDTTYIPRAEMYDTYLPPFRAAVEAGSASFMAA
---FNALNGEPSTANPWLLTDVLRRQWGFDGFVTSDWVGIGELINHGIGAEAARKAILAGVDMDMMGLLY---IKHLPDEVRAGRVPESVIDESVRRVLR--VLLQNRG
GVLPVPASARSIAVIGPLADPHDQMGPHAARGHKEDSVTILEGIRRRAQGTSGLQAALDAAKQSDVVIAVFGEPQELSGEAASRVRLDLNGKQIEVLEELAKTGKPVAL
VLLGGRPQVLGPV-AERIPAILMAWYPGTEAGPAVADVLFGDVSPSGKLPLTWPRATGQLPL-YYNRLRPYIEAIAPLYPFGWGLSY
>A0A430B320_9ENTE
TLKEKVGQLNQ-RLYGDAFKEEVARWGSIGLIYGVFR-ADGLTKEEALTVSRMIQNYIKEHTRLGIPVFLSEECPHGHQGL--DSTTTPANISSGASWNPELYQQVQEL
VAREIREKGAHLGLISTLDIARDPRWGRTEECFSEDPYLAAQFTLAALAGLQGGHVLAVLKHFAAQ--GSGMGGHNSGPVNIGERELREIHLPPMRAAVAAGAELCMAA
---YNDIDGIPCHGNARLLSSILREEFGFKGAVMADGCALDRLVELSGPAATAAWALESGVDISLWDNVF----PYLEEAVLQGLLSEETLDRAVLRVLRLVVLIKNKH
RQLPLDTAVESIAVIGPNSHIYNQLGDYTPFKHVGRCVTVLQGLQARLAGENGLEEALAAASKAEHLILVLGGSSARSGENIDLADIRLPNVQVELVKKLAQLGKKMTG
ILIQGRPHSLCEI-EPYLDSLLIAGYPGEFGGDAIASIVFGDANPSGKLAMSIPRSSGQLPV-YYNYRDIYFLAGEAAYPFGFGLSY
>A0A1H4CLN6_9RHOB
TVEEKIGQLNLLPAGEAGDATPLAERLDAGLVGAVFG-------VKSRESARAMQERALAGSRHGAPLYFAEDVIHGHR------TVFPLPIALACSWDMALIEETAAH
AAREAAEDGVHQVYAPMVDVSRDPRWGRVAESPGEDPTLAALYAAACVRGLQGARVAACLKHFIAY--GAPQSGRDYDNASLGWAELFEVHAPPFRAGVEAGAASAMVA
---FNAVNRTPMHAHAPLVEGWLRGRLGFEGLVVSDYTGVRELQAHGLRAATVARALHAGVDMDMVGEDYL---AALPALARDG------------------VLLKND-
GVLPLRSGPLTAALVGPLADRANLIGTWSVAGDAAQVRTLREGLEGREGLAEMIAEAVAAARLADIVIAAVGEAKEHAGEASSRLSPDLPAPQRRLIAALAKTGRPLVV
AVCAGRPLALAEV-AEAADALVMAWHGGVEAGPGLADILTGAAEPSARLAAALPAHPGQTPLHAAEPTRPDGGQTDGLYPFGFGLAY
>E3EFF8_PAEPS
-IEEKIGQLIQLTG-DGDMDTVVTGPLKKLGLNTIYN-TGFILNITNPEKIIRLQTDYLEKSVHKIPLLFMADIIYGYR------TIFPIPIAQACSWNYEEIENAASI
AAQECYDEGIHVTFSPMVDMVRDPRWGRVMESPGEDTLLAKKYAQSVVLGIQGSKIAACVKHFAAY--GAPVAGREYNAVDLSEHALREYYLPGYQAAIEAGAKLVMTA
---FNTLNGIPATGNEWLNRDVLRQEMNFDGVLISDYAAIEELIMHGYEASAARLALLAGVDVDMKTAVYA---NQLKDVISDNSYMLELLNEAVYRVLKLVVLLKNEN
NVLPLSKET-KVALMGPYAEENSTLGMWAIKGEQTDTINLKTGLLQLVGSEVLLQEAIRNANEAEVIVLALGESIYQSGEGGSRTNPTLPEPQLRLLHELSLLGKRIVL
IVYSGRPLILTDV-AGKVDAIVQAWYPGTMGGEALANILYGEVNPSGKLAMTFPRSVGQIPV-YYNELNTYIEVNEPLYPFGYGLSY
>V4A1Z7_LOTGI
--SRLGIPYQWKTNSLHGIMG--INATSFPQSVGLAATWSTDAVFQMAEA
TSEELRADGIKERFAPVINIFRDPRWGRNQETYGEDPFLSGMLAQAYVKGLQGDRASAGCKHFDAYGPENIPIHREYFSANVTTRDWRTTFLPAFRYCVEAGTYSLMCS
---YNSINGVPSCANKELLTNVLRGEWGFTGYVVSDGRAIELIESAHHSIDTVAGAVNAGVSLELCGVRKTVNFTQIPDAVKAGKLTEDLVRERVK------VLLKND-
GTLPLKSVYDKIALIGPMADPKLLSGDYAAQPDLKFVYTPKFALGPLANTDYDSHSVQKAVQDVDIVILCIGLGYVLETEGHDRENMELPGKQNQLIQDSIKYSAKVIL
ISFNAGPVNITWAMNPRVSAIIAAFYPGQATGVALYNVLKSNHSQFGRLPYTWYHTCDQVPPDYTMQGRTYKIKQEPLYPFGYGLTY
>A0A1H8FTK1_9BURK
-VDEKVGQLNQVAGADPAKAEDLAAAVRSGRIGSMLN--------IRGAAATRQYQQWAMRSRLKIPLLFALDVIHGYQ------TVFPVPLGETASWDLGLIEQSARI
AAIEASASGIHWTFAPMVDVARDPRWGRVMEGAGEDPWLGAAVARARVLGFQKRAVMATAKHFAGY--GAALAGRDYNTVDMSLQQLHDVYLPPFKAAADAGVASFMNA
---FNTLNGVPATGSRYLQTTLLRRQWGYDGIVVSDWGSVGEMITHGFAAHAAPLALNAGNDIDMESHVYD---KALAAAVRSGAVATPTLDAAVRRVLE--VLLKNHE

RLLPIPPRVRHIAVIGPLARRRDLEGGWLVNSSPQHVVTIAEAIKARAPADAGFAAAEAAALQADLVVLAVGEGWDMTGEDRSRADIGLPGRQNELVQRVARAGKPVAA
VVLGGRPLILNTV-AEHAGAILLAWLPGSEGGNAIGDLLFGHVNPSAKLPITFPRSVGQIPISYAHYMRPYISPNTPLYAFGHGLSY
>A0A2Y9BML5_9FIRM
----------------DKMESKLQELTIEEKVGLL--SGADFWTTKEVKRVN-LPSFMMTDGPNGLRKQVTSSDHLGMNESLPA-TCFPMACALACSWDRELLYEIGKA
LSEECIAADVDILLGPGVNLKRSPLGGRNFEYFSEDPYLTSQLAAKYIKGVQENGTGTSLKHFAAN---NQETRRMVSDSLADERTLRELYLACFESVVEAEPWTVMCS
---YNKLNGTYTSEHDWLLNQVLKKEWQYDGAVVSDWGAVNE----------KVKSVQAGLDLEMPGNQGE-SDRILLEAVKNGEISEDKIDEAVTRILRLMVLLKNEN
KVLPLASG-EKIAVLGELASDIRYQGTGSCKVHPYHVDSPFEEIRVYEPSGLLLEEAEAVVNGCDKVIIFAGYPERAENEGEDKVDMKLPYNQTELIHRIARTHPAVIV
VLCNGSPVEM-PF-SNEAEAILETYLAGEAAGGAAADILFGKKNPSGKLSESFPVKLEDNPSPGMQN--KYDKNIEPLYPFGHGLSY
>A0A5R8Q824_9FIRM
-LDEKVGQINQ-RLYGQ--KVDDDYELTDYFKDEVARFGGGIPHADSLKVARMLQAYVREHTRLGIPLLLSEEVPHGHQ-ALDS-VLYPTNIGMGSTWNPELQMQVSAA
VAAELHYKGVQLGLVSALDVLREPRWGRSEECFSEDPYLASIFTTAVVAGLQRKKTVAVLKHFCAQ--GEPMGGHNTAAAVIGERELREIFLLPMVAGIGAGAKATMAA
---YNEIDGMPCHANKWLLTDVLRDELGFDGFVMADGVALDRLRNDDVGKQAAAFGLEAGVDLSLWDDAYT----HAGESVTSGLLDEAVLDKAVTRILTLIVLLQNN-
EQLPLSKDIKHIAVIGPNANLYNQLGDYTSPQRDDYGVTMFQGIAAAVDGDGGIAAAAELAAKADQVVLVLGGSSTRCGENMDVADLCLPQPQLDLLDAVVAVSKQVVS
VIIQGRPHALSEV-SEVSGSVVCAWYPGPFGGQAVAEVLFGDVNPSGKLPASLPRSSAQLPV-YYNYKDNYSMSGSPLFVFGHGLSY
>A0A0R1NB30_9LACO
---VTGQEAHDLIQLMRDYVVNHSRLGIPPLFSEECPHGHQGLNG--VSYPTNIGKGNSFDTALMTEMAGL
QAKELRNLGINLALVSTLDLAKDPRWGRTEECFGEDPMLSAQFSQAIVNGFQGHHIGVVLKHFIAQ--GEVLGGHNSGSVSMGAREFKEVYT-PLFNAVKNAAG-VMVA
---YNDIDGVPCHANASLL-TSLRHRYQFQGLLMSDGTALDRLAQLYDPKQAIIAALNAGVDLSLWDNVYT----QIADNFDTNM--SAALDRAVSHVLR-ITLLKNDP
AVLPL-NRQKKVLVVGPNANFYNLLGDYTAPQTVEFMTTPLASIRAAFDTAGLIQEAVTKAANADVIIAFLGGSSTRSGENVDLASLALGGAQNQLIQALEKTGKPIVT
VLIEGRPHSLKYV-LPASAAVITAWYPGQQGGNAIVDVLTGKVDPSGRLSMTYPKNSGQLPVYYYQRDKNYDLSGSPEFPFGYGLHY
>A0A2R4X227_9EURY
-LAEQCAQLVGVRPCDGRFDPERAAEAIPHGVGHVARIGGGREPRDAAAFANSVQRFCREETRHGIPAIPHEECLCGYMG--PGGTVVPQAIGMAATFDPDLLRATTDA
IGAELAGIGCRQALSPVLDVGRDPRFGRIEETFGESPALIEVMATAFVDGIQDGALGATVKHFIGH--GTPSGGRSRASVSVGPRDLRAVHARPFETVIAADPDGAMNA
---YNDVDGRPCGGAPELLEGLLRDDLGFDGTVVSDYFSVPQLASVHGDREAARQALTAGIDVELPEADAY---RALEASVESGDLDRSVVRRSCRRVLE---------
GPLPI-DDPDRIAVVGPQADATVLLGDYAYPVDACAVVSPVEGLRRTVDARSGIEEARSVAADADLAIAVVGARSGLAGEGISRSGLGLPGVQPELLDALAAVDTPTVA
VVVAGRPLRV----DAAVDGLVYAWLPGERGGLGLAEALAGT-DPGGRLPVSIPRSAGHVPA-HHARR-PYRGPGDPAHAFGAGESY
>A0A5D0CN98_9BACL
TLEEKVAQLMQLAA-PAEGEGQITGPMKSMGVTEMVRNSGSVLGYAGAEGTIAVQKAHLKKNRLGIPLLFMADIVHGFK------TIFPIPLAIGCSWDLPLAERSAEI
AAREAAVSGVHVTYAPMVDLVRDARWGRVMESTGEDPYLNSELARAFVRGFQGQRVAACVKHFAAY--GLSEGGRDYNTVDLSEWQLREYYLPAYRAALDEGCEMVMTS
---FNTVDGVPASGNTRLMRKLLREEWGFDGVLISDWGAVKELIPHGVEAEAAYKAIQAGVDIEMMTSCYV---HHLPRLVREGRVEEEIIDEAVLRILQL-VLLKNER
GVLPLARGQ-RVALVGPFAQSGDILGPWSWTGSRKDAVRLDTAMKAAAAEEAQWAEAARAAADVDVIVLALGESSEMSGEAGSRADITLPAAQLELLRRMKALGKPVAV
VLFNGRPLDLRGV-YDTADAVLEAWFPGSEGGAAIAALLYGDANPSGRLSMSFPAAVGQIPV-YYNHFSTYLLPNEPLLPFGFGLSY
>A0A267EI12_9PLAT
--LGIPYQWNTECLRGAVKS-GQATSFPQAVGLAATFDPDILSQVGRA
TGLEVRAKHNDTCFAPVINILRHPLWGRNQETYGEDPHLSGVLGAAYVGGLQGNLTGAGCKHFAAYDDSYPESRFGFNALV-SQQDLEQTFLPQFRRCVEAGSVSVMCS
---YNAINGVPACANAQLLRGVLRERWNFTGYVVSDAGALEFAILFHKRLSSAIGSLRAGVNLDLSACPYESCFEQLSVALNTSRISEAELRAAA------LVLLKNLG
GFLPLKRPGLRLGVVGPLSDMSLVYGDYSPNRMPQFEQPLLPALKERFGARQDPAAVSSLIAQSDVLLAVVGTSVDIETEALDRRQLTLPDGQLALLRLLTASGRPVVL
AVVSGAPVGLDEAGNDSVRAVVWCGYPAQTFGPALINLLTGRYSPSAALPFTWPSERAQLPSDYDL-ARNWSGSPPPLFRFGQGLSY
>M1MJF4_9CLOT
TLEEKVGQLVQISPSIEALERNREDEIRQGTLG-------CMGGVHGAEKSNELQKIAVEESRLGIPILFGLDVIHGYR------TIFPIPLAEACSFDIEKIKESARI
AAKEASAAGLHWTFAPMVDISRDPRWGRVAEGAGEDPYLGSVIAKARVEGFQGESILACAKHFAGY--GAPDGGRDYNTVDMSLQTLHDVYLPPFKAAAEAGVGTFMSA
---FNDLNGIPCTVNKYLLTDVLREKFGFNGFVVSDANSIPEVVVHGYNKAASKKALNAGLDMDMSQGTYR---NELPELVKEGDILEEVLDEAVRRVLRVIVLLKNEN
NALPLKKDLKKIAVVGPLAEAAEMLGTWSHTGNPSDVVTIISGIKAAVSTCIDFEGAVRVAKESDVIIAVVGENSDMSGEAASRIDINLPGKQEELLKELRKIGKPLIV
VLINGRPLTI-PWEAENVDALVEAWQLGTQSGNAIADVLFGDYNPSGKLVATFPYSVGQVPI-YYNNPRPYIGPAEPLYPFGFGLSY
>Q0AT45_MARMM
-LEEKIGQLNQVEASADNVLDLLGDDIRAGQVGSIIN-------QVDRDTVLELQRIAREESRLGIPLLVGRDVIHGFK------TVVPLPIGQAASWNPQLVEACARL
ASEEASTVGVNWTFAPMIDVCRDPRWGRIAECLGEDPVLTSVLGAAMVRGFQGASLAACAKHFAGY--GASESGRDYNTTNLPENELRNVHFPPFRAAVEAGVASLMTS
---FSDIDGVPATANSFLLRDVLREEWRYDGLVVSDWDAIQQLCVHGLRDEAAFQAASAGVDMDMVAGAYL---QHLAGLVASGRIELETVDRMVANVLRL-VLLKNEG
RALPLPACLDHLAVIGPLANPAEQLGTWVFDGDPERSVTPLAAIESLAADETAFAEAEAIARNADVVVVFLGEEAILSGEAHCRADIDLPGAQVSLVKRLKAVGKPVIA
VIQAGRPLTLTSV-IDDLDAILFAWHPGSLGGAAIADLLFGRACPSGKLPVSFPKMVGQIPV-YYGHKNTLDAGYEPLYRFGFGLSY
>R7TQQ4_CAPTE
---PIEYITECLRGVR--RENATGFPQALGLAASFSRDLMQRVATA
VSVEVRAFDIQTCFSPVINILRHPLWGRNQETYGEDPYLSGELASQYVSGLQGDRVSAGCKHFDAHGGPDTIPVRKFGFDKIEERDLQMTFLPAFKKCIAAKPYNVMCS
---FNSINGVPSCANKRLLTDVLRAQWGYEGFVVSDDAAVEYIFTEHHFETAAVEAIKSGCNMELVGKFDP-SYWQLTKALNEHLITKDELMENVRPV----VLLKNDR
NFLPLKNSLKTVAVVGPMSNTDGLIGDYSTDTDPSLILTPLHGIKKLAPNDYRATDVAAAVDGAQVVFVALGTGFIVEAENNDRSDIVLPGAQLQLLKDAVANGRPVVL
LLFNGGPLDVTFALTSGIVSIVECFFPAMMTGEAIYRMLINNESPAGRLPLTWPAYLNQVPNTDYTMKRTYRYTEDPLYPFGYGLSY
>A0A3N4IQ33_ASCIM
---PGMCLQDGPVGVR-KTQNATVYPAGVTTAATFDRELMYMRSRA
MGLEYRTKGINVALAPAIALGRSPNAGRNWEGFGPDPYLCGIGGAESVRGLQDEGVIATAKHWVANEQERIENIIGFNSSNVPPRALRELYEWPFIDIIEAGAASVMCA
---YQRVNGTYSCESEEIIKGHLKSDKNFKGFVVTDWFA---ASLASKASTAGTDVVMPG-DIGLMGAAAG--TQAASDGALTGEKGATRLDEAALRV---TVLLKNTN
KALPLTKKIKKIGVFGSDAGGTLAVGWGSGGGTFEYLIDPLQAIEERAAADFDYKKIREVAADKDVCLAFIQSR---SGEGLDRNNLTAWHGGDALIATLTPICDNIVV
VAHTVGPIVVEPWEHPNVKAVLFPHLPGQESGSSLVQTLWGDVNPSGKLPYTLGKKQDDYCCGF-----AYRFAAKGIFEFGFGLSY
>A0A161XRQ5_9EURY
TLEEKVGQMTQMAA-GEISTDDPAETLRKYKPGSLMYL-TSFDPEEVARTSNKLQKAMVNETRLGVPFVYGIDSVRGDNNVAGA-TLFPHNHGVGATWDADKAEEMATV
TSKVMRTTGTHWNFSPVCDIQRDPRWGRFYEGFSEDPFLASQMVASKVRGYEEERTGASVKHFAGY--SAPANGNDRTAALLPYRTFVSSILPSYAAGINAGAETVMVN
---SGSLNGLPAHASKELLTDILRDQLGFDGMVVTDWHDFYRMIKVHGLKEATKLGINAGIDMYMVPAAGIEGQQRLIELVKEGSVSRDRIDDAVTNIL--MTLLTND-
GTLPLDSG-SSILVTGPSADVRNQMGGWALGWTEPPATTVLEGLTDAADSFTNEDDVRRAAECADVVVAVLGEGP-YAEEKGDTDTLALPEAQQRLVEVVSGTDTPTVG
VIMAGRPRGTDVF-D-DLSASMMAYLPGTAAGPAVAATLFGDANPSGRLPFTWPKGTGQIPNNNYPPDNKPQSHETPLFEFGHGLSY
>A0A318XPS1_9FIRM
TLEEKVGQMMQIS-YNTLSAEEAEDWASNKKAGSFLH--------VLGKKAEHLQELA-LSTRLGIPIIFGIDAIHGHG-LMNGSTIFPSQLGMSCSWNPELLEKAGRV
TAREAAADGIHWTFSPVLCIGRDLRWGRINETFGEDSYLIGVLASAIIKGYQGKSILACAKHYLGY--GESTGGKDAYDTEVTCRKIREVFLPPFKKAIEAGCATFMTG
---YQSIDGTPMAINKKLLYDILKQELGFKGFVVTDYNNTGSLVTLQMLDDASKKTVEAGNDMIMATNDFY---ESAIRLVKSGLLSESLIDEAAGRILS-IVLLKNDK
NILPLSTNIKKLAVIGPNADLKAQLGDWTFFTPAFEYYTMLRGISERSRLNQNIEDAVSAAAACDAIIAVIGDCVEQNGEFKDRANLDLSGAQQKLLKALKALGKPLIA
VLVNGKPLCV-PWLQKNADAVVESFNSGILGGKAVAELLYGDFVPSGKLTVSFPYHSGQIPV-YYNQLHSMDAERNALYPFGFGLSY
>A0A0P6W8Z2_9HYPH
TLEEKIGQLDMPSH----GGHYDPARVAAGRTGGVLNF--------GSPQEIAAIRALNAQSRLKIPLIFGLDVIHGYR------TLFPMPLAEAASFDPAVAEQSTHW
AAVESRAAGLNLTFSPVADLARDPRWGRIVEGGGEDPMLAGLFAAARVRGFHSGGIGATLKHFAGY--GAVEGGRDYAPADISEGTLRDRYLPPYRMAVEAGVDVVMTS
---YISLNGTPTTASRRLMSDILRGEWGFPGYVVSDMNAIPEIALLGVNGEAAKLALEAGVDQDMDGEVY---IAELAGLVRDGTLPEAALDRAVARVL---VLLQNKD
DVLPIRPETRSIAVVGALAAQADTLGNNAARAVPAETVTVLDGLRARAPADSNFQAAAAKAAEADLIVAVLGEPQDHSGEGASRASLELPGRQTDLLRRLAATGRPVVV
VLLAGRPLALSEA-VDPAAAILLVWYPGSEGGHAVAETLFGDRDPAGRLPVTFPRHVGQVPI-TYDALRPYLVALGPLFPFGHGLGY
>A0A512INI3_9HYPH
TLEEKAGQLNLVSL----EPGFDPESVRRGEVGAVINF--------SNQNLASNIDGYARTSRLGIPLLISLDIVHGYR------TVFPLPIGMAASFDPDLVRRASAA
AARETLAAGINWSFSPMADVARDLRWGRVVEGLGEDTWLTGQLAAAQVEGFRDGGVASTLKHFAGY--SAVYGGRDYDVTWVSPTELYDTHLPPFRAGIRAGADSVMTA
---LTALNAVPTTADGHLMTQILRREMGFKGVVIADWEAVASLIKHGVGAEATRKAVAAGVDMDMTSGLFV---KHLPEEVRAGRISQASVDESVRRVLRL-VLLKNDR

NLLPIPRTVKRIALVGPFADKWDQVGPHEGNGQVDDVISIRTGLTERAAKRADFPKAIEAAKAADLVVAVIGEGRDQSGEGSSRAYLGMRGLQQDLVQELAATGKPLLL
LVFGGRPVELHEA-IDRAQAAMMIWIPGTEGGPAVAETLFGDFDPSGKMPLSWPRTVGQMPL-TYDRRRPYNETISPRFPFGYGLTY
>E7G6B9_9FIRM
TLDEKVGQLNQ-HLYGQLFKDHVQKFG---GVGAIY---GGVKKEESLQVIEMIQNYLKTETRLKIPALITEECVHGHQGLHS--MMYPANISMGMTWNPDLLKEICQE
VSCELASKGGNLALFTGLDVMRDPRWGRSEECFSEDSYLTSEMTKAAVKGFQLNGVGVILKHLCAQ--GACEGGHNSGAASIGQRELREVFLPPVKAGVLSGAKGVMAA
---YNEIDGIPCHVNQALLTQLLRGEYGFNGIVMADGCALDRLSIMNSIPLMAATALKAGVDLSLWDHVY----PLLGDAVRQGYLDEKVLDRSVKRILKL-VLLKND-
GILPLRKDIKSIAVIGPNAHVMNMLGDYTSFQKAEDVTTLYQGIQQVLGEKADLAEAVELASQSDVVIMALGGSSARCGENVDKASLDLEGLQVELLRKIKQVNQQIVT
VLIQGRPHSIGNI-VNDSRAVLAAWYPGNLGGLAIAQTIFGDHNPSGRLSMSIPQSSMQLPC-YYNGKAKYIMSGKPLYPFGYGLSY
>R7N7J7_9FIRM
-LDEKINQMVQLNADFENGDSITGPMLEMGLTKDQVWQAGSVLGTVGAAKIKKFQKVYMEHQPHHIPLLFMADVINGYR------TIFPMPLAQGCTFDPELVRKMASV
SARETARAGLHLTFSPMSDLARDARWGRVLESTGEDPYLNCCMTSAMVKGYQGEHVAACVKHFAAY--GAPLGGRDYNQVELSERTLREDYLPAYKAGVDAGAQMIMTS
---FNTLGRVPSTANKALMRGILRDEWGFDGVVISDWAAIWELLKHGIEDEAAALAVKAGVDIDMMTEIYA---NHLKKLVEEGKVEEKLIDEAAYRILRL-VLLKND-
GILPLVKKADGIALIGPYVKEKQICGSWSLFWKQSDLVTVEEGIRNKELDEAMMQEAVETAKKAETVILFLGEHYQASGESASQTEIQLPAHQLELLDRVYEVNPQIVV
VTFSGRPLDLRHV-VEKAKAVLHVWFPGTEGGNAIADVLFGDQEPGGRLAMCFPYTVGQVPV-YYSELHTYLAPNHPLYAFGYGLTY
>A0A5C5W5X1_9BACT
-LADKVGQLNQISGGSAAVREKLLSEIRAGRVGSVLG-------ATGAEYTNMLQRVAVEESPHKIPLLIANDVIHGYR------TIFPIPLGEAASWNPALIKTCCQV
AAREARAGGTHWTFAPMVDVCRDPRWGRIAETAGEDTYLGSVIAAARVRGFQQAGVMACAKHFAAY--GGAEGGRDYNTVDISRRTLHEIYLPTFHAAVKAGAGSFMTS
---FNEISGIPATANNYILRDVLRRGWRFNGIVISDYNAIGELIPHGFGAAAAEKAIRAGVDIDMCAFLYE---KHAAALVESGRLPAALLDEAVRRVLA-LVLLRNEG
GLLPIRSGVERIALIGPLAERADLLGTWACIGRAEDVVPLSDGLRKALPNTGGIDEAVAAAKASQVAILAVGESEDMSGEAHSRAEIDLPAPQLELVKAVHAAGVPTVV
VVFTGRPLAI-PWLAENIPAILVAWHPGIEGGNAVADTLIGTYNPGGKLPATFPRSVGQIPL-YYNHKRPYIIDWTPQFPFGFGLSY
>E8RPE5_ASTEC
----------------ERAKAAVAAMTLDEKLTLILSFTDEIKALVNKEHIRGSAGYVPAIPRLGIPAQWQTDASIGVRVQGMARTALPSSLATAASFDPAVTEAGGRM
IAQEARLSGFNTFLAGGANLAREPRNGRNFEYVGEDPWLAGQMASGLINGIQSQHMVSTLKHFAVN---DQESQRTTVNVTISPEAMRQSDLLAFEFILQSNPGSAMCA
---YNLVNGRWACENEYLLNKTLKQDWGFKGYVMADWGAVHSTVDSANYGLDQFTGFPCCGDRQPYFAPI-----NVKAAMEKGDISQKRLDDMAQRVL--LVLLKNEG
NLLPL-SGVKSVAMIGAHADKGVISGGGSSGVPVTYMPSPVEALKADLPKGEDIAAAVALAKSSEVAIVFVTQW---MGEGMDG-TLELKGNQDALVAAVAAANPRTIV
VVQSGGAIFM-PW-KDDVGAILQAFYPGIRGGEAISRLLTGKVNPSGHLPISFPASNDQLPTGFGKPD-GYDKGYTPLFAFGHGLSY
>R4W6C6_9EURY
TLEEKAGQLVGMPLGNGRDVDEAKELVDEHHLGSVAPFGWYWTPDECAEVARELQEYAREETRLGIPLLFNADAVHGHA-YVKGATTFPNGLGMAATWDTELTKRAGEV
TGRELRATGIHQNYGPTCDVGRDPRWGRAHETYGESPYLVKELIGAEVTGLQGESVIATAKHFPAY--GEPRRGEDAAPVEISNSTLRRVFLPSFEAAVEADVWSVMPC
---YNSIDGEAVHGSRRFLTELLREELGFDGHVVSDWGGVDHLQNDHKDEASSRLTRQAGLDVISVGMGRH--AEHTVDLVESGELSEDRIDESVRRVLR-ITLLQNDG
DVLPL-DDPDEIFVGGPNADLIAQIGGWSIMDEE---GVPGKTIREGLESYDDVEAAAEKAAEADVAVVAVGEDWYITGEFPTRTELELPDPQIELVKAIQETGTPTIG
LLVTGRPLAIPWM-AEHLDALMMTYFPGSEGGEVVAEALTGEIEPAGRLPVSIPKSSGHLPTNYLRHPRPHTDSYDPLFEFGHGLGY
>A0A1W6LPU7_9BACT
TLDEKAGQMTQLTLKAKLDENKLEKYIVQEKIGSVLNCGGRALSPEKWLEITSLVQKFAKKTRLQIPVIYGLDSIHGAGYVAGS-TLFPHNIAMAAAGSRQLVQQMAEV
TALETRAAGIRWNFAPVLGVARHPFWPRHYETFGEDPFIASEFAEAYITGLQGDKVLACMKHFLGY--SYPRSGRDRTPAWIPEIQLRELFVPPFRAAVQAGAVTAMIN
---SSEINGRPVHASPFYLKKLLRKDIGFSGFVVSDWADIDNLYTREMQREAVKIGVNAGIDMSMTPFKI-DFKKHLVSLVEAGEVPVQRVDEAVKNIIKVITLLKNES
EILPLKKE-EKILVTGPCSNKSVLNGGWSRTWYPDSQNTILEAVKAEFGRLLDADKAAEKAENCDVILLCAGENTYTEHSG-NIRDYNISLSQRKLAARLSETGKPIIT
ILTQGRPRVVREI-EKVSDAVVMAYLPGVKGADAIADVVSGDVNPSGRLPFSYPKYAGGF--EWYDYKANWSCKVEFQWPFGAGLSY
>A0A172YJV1_9GAMM
TLEEKIGQMTQFTSERPSVRDDYEQDIRAGRVGSVFN-------SFDADFTRRMQRLAVEGSRLGIPLLFGYDVIHGFR------TIFPIPLAMASSWHLEAIEAASRV
AAREAASAGIHWVFTPMVDISRDPRWGRVMEGAGEDPLLGSLVAAAQVRGLQGESVAACVKHYAAY--GAAEAGRDYNGVDISERLLRSVYLPPFKAAVDSGVATLMTA
---FNALDGVPASCNSLLLDQILRREWGFEGLVVTDYTAVMELIHHGVSKDAARLAVNAGVDMDMQDGYFL---DHLAELVAEEAVDGARIDQAVKRILV-MVLLEND-
GLLPLSSELERVALIGPLADHADLLGPWHGDGRAEEVTSVLEALGDRLPDKEAIDAAVREASQAQVAILVLGERESMSGEAASRADIGLPGHQLELALKVIGTGTPTVV
VTMSGRPLVLSEL-APKAQALLHAWWPGTEGAAALVELLFGDHAPCARLPMSFPRAVGQLPL-YYAQARPYLVANTPLYPFGHGLGY
>A0A397NS57_9SPHN
TLEEKAGQLSILGG----DYGDVDDLARRGLLG----GTNGVMPGHDVAAYTRHIQDMAMQSRLKIPVWFMGDVAHGYR------IVMPVPLALAATWDTALVERVHRA
AAIEATSDGVDWTFSPMLDIARDPRWGRVVEGAGEDPYLGSAMAVAQMKGFQGDTMLATAKHFAGY--GAVTAGRDYNAVDLPPRLFRDVYLPPFKAVADAGIGSVMAA
---FNTLDGVPATSSRALLTGTLRDDWGWDGLIVSDYDAVKELSVHGVAAEAARMALRAGIDIDLHSGTYL---EQLPTLVRDGKVPIAEVDAAVRRVLE-MVLLRNT-
GVLPLKKA-GRIAVIGPMADLQRDVQGPMPALVPGEVVTMLAGVRGVAGGEAGISAAVAAAKAADVAVLVLGDSIDMIGEGNSRASIDLPGRQLDLAKAVAATGTPVVA
VVVSGRPLAV-PWLATHADALVFAWLAGDQAGNALGDLLFGDANFSGRLPITVPRSLGQVPL-SYDALRPYVEENTPLFPFGYGLSY
>A0A1E3H1U3_9HYPH
TLAEKIGQLNLVTPGGSVANADVAGKVRAGAIGSIFG-------VKSREAMRAFQDLAME-TRLKIPLMFAEDVIHGYR------TIFPIPLALAASFDMDLVARAARV
SAVEASGMGIDQAYAPMIDVCRDPRWGRIAESPGEDPHLAARFSEALVRGFQGDSVMACLKHFVGY--GAATGGRDYDRADMSPTELHDVYLPPFQAGVAAGAGSIMAA
---FHALNGVPMHTHAGLLRGVLRHAWGFDGPVVADYTGVMELTYHGILAAAAVKGLVGGVDMDMVSEAYV---RHLADAVAEGRLDPALVDEACRRVLA--VLLKNED
GLLPLAQGC-NVALSGPLADSVNMNGTWAVCGDWRDAVPLTAGLGNRCRFEQLIAEAVAQARDADVALAVVGEAREYAGESSSRTDLRLAPGQVALLKALKATGKPVVA
VVMTGRPLVLTDI-VDHCDAILIAWFGGTESGHGIADVLFGAAEPGGRLPATFPWHEGQIPV-FHARLRPE------GYPFGFGLSY
>S6CZX7_9EURY
TVEEKVGQLNQRSVHFEDEGDELEGAIADGEVGSLLN-------VTDLETKRRLQERAVEESRLGIPLLIGYDVIHGYR------TVFPTPLAQAASWNPELAERAERI
AATEASADGHNWTFAPMVDVSRDPRWGRVMEGAGESPVLGSAFARARVRGFQGETVLACAKHFAGY--GAVEGGREYNTVNVSETALRERHLPPFAASVEEGVGSVMNA
---FNVHERIPASGNESLVDGILKGEWDFPGLVVSDWASFREQIQHGTRREAARKAIQAGSDIDMASEVVV---DELADLVRDGDVSEDRLDDAVRRVLTV-VLLENED
DRLPLDPA-DDVAVIGALADAEDALGAWSFEGRSEDVITIRDGLESFLDRDESLEAAVDAATDADVAVAVVGEPADITGEAASRSQIDLPGDQRRLLEALGETDTPVVA
VLMNGRPLAIEWL-AESVPAILDVWHPGVEGGPAVAETLFGEAVPGGHLPMSVPYNEGQIPV-AHDRLRPYLVPNEPLYAFGHGESY
>A0A0A2VY79_BEABA
TTDEKIGQLRLISVGPDNPKEAIREMIKAGQVGAIFN-------TVTRQDIRTMQDQVMQLSRLKIPLFFAYDVVHGQR------TVFPISLGLASSFNLDAVKTVGRI
SAYEAADDGLNMTWAPMVDVSRDPRWGRVSEGFGEDTYLTSIMGRTMVEAMQGKSVMTSVKHFAAY--GAVEGGKEYNTVDMSPQRLFNDYMPPYKAALDAGSGGVMVA
---LNSLNGTPASADSWLLKDLLRDEWKFKGITISDHGAIKELIKHGVPKDAVRIALNSGINMSMSDEYYS---KYLPELVKSGAVPMKELDDATRHVLNVMVLLKNRL
QTLPLKKT-ATIAVIGPLADKRDAMGSWSAAGVAAQSVTPLEGIKNAVGQKAMLDEAVNTAKNADVVVAVVGEAQGMAHEASSRTNLTLPQSQRDLIAALKATGKPLVL
VLMNGRPLALVKE-DQQADALLEAWYGGTEGGNAIADILFGDYNPSGKLPMSFPRSVGQIPT-YYSHLRPYFEANGPLYPFGYGLSY
>A0A511YX56_9CELL
TLEEKVGQLVQMA---NPDVERHADLLTAGAVGSAIVASGNVRDAGTQASHANALQRAALASRLGIPLLVARDVVHGHR------TVFPIPLGQAASFDPDLVRACARV
TAREARADGVRWTFAPMVDVALDPRWGRVAEGYGQDAWLTSLLGAAAVEGFQGGSLAACAKHYVGY--GMAQGGRDYAEVDLGPVTLQNRHLRPFRAAVDAGVATVMSA
---FHTVDRVPMTAHEPLLRDVLRGDLGFAGPVVSDWDAVGEIVRHGLLTGAATLALRAGVDVDMVTGAYA---THAADLVRSGAVDEALLDDAVRRVLA-VVLLRND-
GVLPLRPV-GRVHVTGPFATTEELLGTWTLDGRGEDVVTIADALRERLAAGRFPDASHLAAREADLVIACVGEHPLRTGEANSVASLELPPGQAEVLEGLARVSRTLVV
VVVTGRPLALERL-AHLGSALVLAFHPGTEGGHGIVDVLTGDVPPSGRLPASMPWVTGQVPMHDHLPSRPYRHSDAPQYPFGFGLGY
>D7UX19_LISGR
TLEEKIGQVVQVSG---NVYMEDKQTIDTGPLQDIGFHSDNVNDWRQIKKIQTEYLHKHPK---KIPLLIMADVIYGFR------TINPIPLAQAGSFDFELIQKGATE
TAKESYRNGLHVLFSPMLDLVRDPRWGRVMESPGEDVYVASQYANSIVTGYQGEHVAAGIKHFAAY--GAPEAGREYNSVDMSLLKLRQTYLPPYEAALKANAKLVMTA
---FNLLNGIPATANKFLNRKILREENNFDGVLISDFAAIEELVDHGYKQTAAQKALEAGVDIDMMTSCYA---NELQSLIESDQLPMELLDEAVRRILV--VLLKND-
QQLPL-EKPEKLAVIGPYGTSKLTLGFWASVTSPLDTISLAEGLQQHFPNNELVQEAVEKAKQADKILFTFGENFMESGEGASKAHLDLPEKQLHLLRELKKTGKPIVG
ILYTGRPLVLTEV-ATLFDSLLLVWYPGTMGGIGISNLLTGKANPSGRLAMTFPRAEGQIPI-YYAHHRPYTEANEPLYPFGYGLSY
>D9PYZ6_ACIS3
-LEEAIAQLEAIPFTDGRLSEEKARELVGSGVGIVKRVYGVVRDLAAARELNRLQRFLMER--VGIPAIPEEEGAGGLM--VPQATAFPSPLAMASTFDPGLMLRVAQA
IGRQASRLGVRQLFSPVLDLCLDPRWGRCEETYGEDPKLAAAMGVSYVRGVQSQGVAATLKHFVAY--GASEGGRNGANAPIGYLDLRNLVMVPFEAAVESSPLSVMPS
---YNDVDGVPSHANGLLIDGVLRGEWGFGGFTASDAEALSMLIDTQGREEAALLAITSGVDVENGYAPMRERYWALADMARSGRAPESVIRRAAERVV--LVLLKNE-

GALPLRRDA-RVLLTGPSAAGRALLFDYHLQAEVTRVVSVLEGLRGLGLNDEEVRAAAEATPGHDVVVAVVGEVAGGSGEGIDRDP-VLPECQARLLRELASAGVKLVT
VIVSGRPLAIPDDVVRSSVAILYSFYPGEEGGNAIARALAGLVNPSGRLPVTIPASVGDLPVRYNLRRSSTRRRPSAAFPFGHGLSY
>V3ZQT9_LOTGI
--LGIPFQWWSNCGRGDV-RAGNATSFPESLGLAAAFSPELLFDVTKV
TALETRAFGIHQGFSPNLDIYRDARWGRNEETWGEDPYMAEIYARAFVTSLQGDLVTATCKHYTAS--GGPDSYPENRFTKVSMRDLQMTFLPGFKGCIDAGSYGLLCA
---YNKINGIPACANKETLNDILRTDWGFKGYVISDVAAIENMIFTQHTVDTVAGSINGGCNINAASNSQPNHYYSMVQAVREGKLTEQTVRESMKQ-----VLLKNRG
NFLPMQTKYDNVAIIGPMINPKAQTGNYSPTIMPTYTTTPLETLSKYLAHQYNQTAVIQAVQNSDAIIVCLGLGNDVEQEGNDRRNISLPGHQLDILKDAVAHSTPIVL
IMFNGGAVDLRWPGEDRVTSILEVFYPSQATGKAVYNALVGSLVPAGRLPITWPMSDDQIPPNFTMEGRTYRSPPEPLYPFGYGLSY
>A0A1X7VU49_AMPQE
---PYQWGTECLSGNV-SAGDATSFPMPIGMAASFNYDLLKRVTNA
TAYEVRAKHAAAVWSPVLNIMRDPRWGRNQETYGEDPYLSGYLGQAYVNGLQGNIIAAGCKHFDVHGGPENIPTSRFSFDKVSMRDWRMTFLPQFKACVEAGALSLMCS
---YNRINGVPACANKALLTDILRNEWDFKGYVVSDQGALEFIVIEHHFMKAAADAANAGTCLENIGRKFFNVFEHLVDAVKNNLVSVDTLKNAVSRLF--IVLMKNDD
PFLPITNEVKKACMVGPFSDPEVLFGDYSPTLMRDYVITSLAGLKNANIGNYDSAKVRSACDGVELIIVTAGLSKHLESEGKDLSDINLPGHQLDLMQDAEAASKNVIL
ILFNASPLDIRYATDPRIVGILEAYYPGQTAGKAIANVLTGEYNPSGRLPNTWPASLDQVPGNYTMKERTYRFTQEPLYPFGYGLSY
>A0A4D7QK13_9HYPH
TLAEKVGQLHLTG----RGDSFKPEWVAEGRTGVLMNF-------TQPREVRAVQAQL-ARSRLRIPLLFGLDAINGFA------TYFPQPLGQAATFNARLMEVSAYW
AAREARAVGVNWTFAPMVDITRDARWGRVMEGAGEDVHLGVISAAARVAGYHRGGLATSAKHFIGY--GEAEAGRDYNSVWIPLSKLWDIHIPPFRAAIEAGAFTVMTS
---LSAMNGIPSSADRAMMTDTLKVKLGLRGFIVSDFESIKEMLAHGVGPEVVRKAMLAGVDVDMMAGLYD---RHLADEVRAGRVPQSAVDDAVRRVLRVIILLKND-
QILPLGRQTRSVAVIGGLAT--HQEDWQYSDNAP---------------NPGNTAAALRAAHAADVVVVMLGEDCEQYGEGTSRAHLELPAPQKALLDELIATRKPVIV
IMHTARPFVLTGF-VDRVKAVLQTFHLGTEGRTALAEVMTGRVNPSGKLPMTFPRATGQVPI-YYDQLRPYLEKIEPLFPFGFGLTY
>A0A176U903_9FIRM
-LEEKIGQMVQLTA-NTGPMGSAKIEPEDMKLCGTILG------TSGAAKLKEIQDNAMAAQPHHIPMLFMLDVINGFQ------TIFPIPLAQGCSFEPEIARKGAQI
AAKEAAASGLHVTFAPMADLVRDARWGRVMESPGEDPYMNYCFAKAMTEGFQGENISACLKHFACY--GYPEGGREYDNVELSERTLRQDYLSGYQGAVDGGCRMAMTS
---FNTLNRVPSTANKWLMRKVLREDLGFDGVLISDYSAVEELIPHGIKREAAKLAIEAGVDIDMMSDVYL---HYLKELVTSGEVDETLVDEAVLRILKL-VLLKNEN
KTLPLEQEAADVLFAGPYVDTRAICGAWSFPATYDNIKTVQECIQEHIGTEASIEQTVQAAKSVSKVVLFLGESFRQTGEGASRTCITLPEIQMELLKRVSAVNNNVIV
VLFAGRPIEVALI-EDLAKAVLYVWMPGTEGAAAITDVLFGVKEPTGRLAMSFPNVSSQEPL-YYNRFRPYITEYRPLHSFGYGLSY
>A0A498G1Y2_9EURY
-LESKVAQLGTVRIGSGAFSPRLAREVIPHGVGRVTRVGRGLPPRALAAVVADLQSFLRTETPHGIPAFVREESLCGYAGRKG--ATVPQAIGIASSWDPTLAREVASA
VGDQLRAVGCQLTLSPVADVGVEPRWGRIEETFGEDPALAATMTRHVVDGLADANVDATLKHFVGH--GRPVGGRNARPTASLDEMRDADLVPFRAGIAAGAASVMAA
---YNTVDGVPCHANERLLTDLLREQWGFRGTVVSDGRGIEMLADDYERREAGVRALTAGVDVELPETECF--GDRLVAAVRDGDVDESFVDRALRRHL---VLLDND-
GALPVSADA-TVALVGPNADPRNLLGNYSYAGGV-DIVTPRAALRDRVAAEDDIDAAVTAARDADVAVACVGGQSGIAGEALDRAELGLPGRQTELVRRVAATGTPVVA
VLVSGRPLAVPEV-VDCAAATIAAWLPGQEGGAAIADVLLGT-DPGGRLPVSVPRTVGQLPVAYRQDSSRYVTPGDPLFPFGHGQSY
>A0A660KS62_9ROSI
---SAEDWANMVDGFQSKARESRLGIPLIYGIDAIHGHNNSIRGATIFPHNVGLGATRDADLVRKIGEA
TALEVRATGMHYTFAPCVAVCRDPRWGRCYESYSEDTEIVRKMT-SIVTGLQGKNVIACAKHFVGD--GGTENGKNEGNTISSYEDLERIHMAPYLDCISQGVSTIMAS
---YSSWNGRKLHSDHFLLTEILKEKLGFKGFVVSDWAGLDRLNVPRGYRNCISSAVLAGIDMVMVPYRYELFLKDLISLVKEGRIPIERIDDAVERILRVLVLLKNGK
KFLPLDRNAKRILIAGTHADLGYQCGGWSFKWRITIGTTILDAIKEAMSNEEVPTADTLARRDFSFAILVVGEKPYSETFGDNLEGLVIPKEGVDIISSVTN-NIPTLV
ILISGRPLVLEPWLLEKIDALIAAWLPGSE-GGGIADVVFGDHDFEGQLPVTWFKRVEQLPLHIGV------DSYDPLFPLGFGLTY
>G5IKP4_9CLOT
-LKEKISQLCSIPSIESKVEIENLEEKIAWGIGSFQLPARNFPPEISARINNEIQRRVMEKSRFHIPALIQEECLSGQV--AKGASMFPKPIGLACTFHTELVKQIYEA
VGRETRARGGHQAFTPVLDLGRDPRFGRLEETFGEDTFLTSQMGCAAVKGLQK-NVVSTVKHFAGY--GQCSGGRNFAPSFVGRRELLDEILPPFRAAVEGGAMGVMPS
---HCDVDGIPCHQNRELLDDILRKQWGFKGIVVSDYYDIGRLEHLHGPLEAAMLALKAGVDMDIPDGKTY---QSLENQLLSPD-MMDCIDRAVERVL--LVLLEND-
GILPLERKSLSIAVIGPCAH-PVHFSYYSSAPHI--GVSILEGIRERWEGRKMIRKAADCAKTADLVVLCLGGSAATSREAIDNDNLNLPGHQEELFKKIRKVNPNIVT
VIVSGKPYSNERI-YRESRAVIQCFYAGQETGRAVAALLGGDYNPSGKLCVSVARNVGQLPV-YYSQKRTYLSEYGPLYPFGFGRSY
>A0A1E5KYZ2_9ENTE
---EKIGQLIQLSG-DPKQKLGITQEIVD-RVGSVLN-------VTGAETTRVIQEKYLAKSRHKIPLLFMADIIYGYR------TIFPIPLGLGATWNPELVKKAYKV
TASEARSAGAHVTFAPMVDLVRDARWGRCLESTGEDPVLNADFSKAMVEGFQQEGIASCVKHFAAY--GAGEAGKEYANVDMSERRLRQDYLSGYKAAVDAGCEMVMTA
---FNTYDGIPATGSEFLLKEILREEWGFEGVIISDYAAIQELIAHGVDREASLMAINATTDIDMKTSCYA---KQLEPLIKDQEINPQLVNEAVWRVLKLIVLLQNKD
NILPLAPNKQKILLVGPYGDKQDLIGLWAVHGKTSDVVSLKSSLEKEIVEKEFLKKALAAGEKADVIIFAMGEHTMQSGEAGSRTDITLPTIQKEFIKEMVTLGKKNIL
ISFSGRPLVYTDE-IKQMDAILQAWFPGTEGGNALAEILLGKVNPSGRLSLGFPYHVGQLPM-YYSEFKTYLCPNNPLFPFGYGLSY
>A0A1X7VMN3_AMPQE
---LEKMSQTATNASAIERLDIPAYWWSECLHGLAQSLTSATSFPQVIGLGATFNMSLVLAMGQV
ISTEARAFANNTFFAPNINIYRDPRWGRGQETPGEDPYLTSQYAANFVKGIQEGKAIATCKHYAAY---NLERYLDVRRVNVSDQDLEETYLPAFKACVEGQVGSIMCS
---YNAINGVPNCANDFINNKIARDTWGFEGYIVSDCGAILDIQYKHNTNITVADALKGGCDLNCGHFYE----KYMEDAFDNSTITEEDIDKSLTRLF--IVLLQNKG
SVLPLIVKHSNIAAIGPNADTHIMQGNYHGI--APYLISPLQGFSNLGINTEGFPDAVKAVQGVDAVIAVIGLNNTQEGESHDRTSIALPGHQEDLLLELKAKGTPLIV
VVMSGGSVDLTGV-KDIADAILWAGYPGQSGGQAIAEVIYGKVNPSGRLPVTFYPASYIN-EPYTNMSRVYKYTGTPVFPFGFGLSY
>A0A2T4A960_TRIHA
---GNV-AFNSSTQFPSPMVLGAAFDDQLVHDIATA
VSTEARAFSNHLKWAPNINPYRDPQWGRGQETPGEDPYHVAQYAYNYVVGLQGGKVVSTCKHFAGYDIEDSDGVVRGSYNAISTQDLAEYYLPSFRSCFDAKAGAVMCS
---YNAVNGHPSCANSYMLETVLRDHWGWNSWVTGDCGAVDGVFNQHHDAQGVALALNNGTDLDCGTAYS----SYIASAIQSNYTTEAQLDQALGRL---ITLLKNDD
NILPIRPTGQTVLFVGPWANSVSMFGNYNGVASYNWKVTYSQGLQYVLSNTSQFAAAVSAAQEADVVVYIGGIDEQVEAEAHDRTSIDWPGAQLDLIQQLAAV-KPVVV
VQVGGGQIDDSSLQNGNVKGLLWMGYPGQEFGPGLIDILSGVAAPAGRLPVTQYPADYINQVPMTDQSRPYRWYNGSVIPFGTGLHY
>A0A542DYC3_9MICO
TTEEKAAQLTQVD----NLEPGRDADLLRRGVGSSLYASGNVRDGGVASAVDECQRLAVEGSRLGVPVLFGRDVIHGHR------TVAPIPLGLAATFDVDLLRRVSAL
AAREASTEGVAWTFAPMMDISEEPRWGRVAESLGESPVLAGRLAAAMVEGFQGTTLGATAKHYVGY--GLVQGGRDYDTVTVGENTLRNLHLRPFRDAVDAGVMAVMAA
---FNDVDGVPMHAHRHLLRDVLKGEWGFDGVVVADWNGIGQLVNQGVLRDAARQALLAGVDLDMCSGAYL---DHLPDLVEDGEVPLDLVDDAVRRVLR-MVLVKND-
GLLPLHANIGKIHLAGPFVHGDALLGTWVLDGRGEEVVTPAQAFAERLAAGRFSDVAMSMVREAEVTVAVVGEHRSRSGEDRCISTLELPAGQLEVLEEMAGLGKPLVV
VVHTGRPLELGRV-LELADAVLVAWHPGTEAGHALTDVVFGDVSPSGRLPMTFPRTVGHIPSSSHERPRPIDDRDRQLLTFGYGLTY
>A0A0R1N9E4_9LACO
---------------------------------------NTAGSVFNVTKPDQLRALQKKAIEESPHHIPYLFMADVIYGFR------TIFPIPLAQAGSFDFDKLKKAAQV
TARESYNNGIHCLFSPMLDLSRDPRWGRVLESPGEDVYTAKAYAKSIVQGYQGDRVAACIKHFAGY--GAPEGGREYNTVDMSTQRLFNEYLQPYQAAIDANCQLVMTA
---FNVLNGTPATGNRWLNRDVLRGRFGFSGPLISDYSAISELQSHGMVEESAKTALIAGVDMDMMTSVYA---NALPQLIRQPE-FAKLLDEAVWRLL--VVLLKNE-
DVLPLRKG-KSVALIGPYADSRLTLGFWASVTNPMDTVTLREGLKQSYSDQKLIQLALTAARESDVIVLTLGEQFMESGEAASKAHLALPMREKRLLKALADLHKPIVG
ILYTGRPLVLTDI-VPYLNALLVAWYPGTMGGVGIANVLTGVASPSARLSTSFPRSEGQIPV-YYAQNRPYIEANQPLFGFGAGLSY
>A0A1H7FME3_9FIRM
-LKEKVLQLVQLPGQATGLSDEGASEEEKSLVGSTLG-------IWGAEKIIEIQKKYMDKHPHHIPLLFMLDVIHGHK------TVFPCPLGQGATFDPEIAKTGAEV
QAREAAAEGVHVTFSPMGDLVRDARWGRVLESTGEDPYLNGKMVSAMVEGYQGKHIAACVKHFAAY--GAATSGRDYNSVELSESMLRDQYLPAYAEGIKKGARLVMTS
---FNTLNGIPSSGNTWLMRDILRNEMEFDGVLISDWAAVAEMATWGFLKEAAELAMKAGVDIDMCTESYG---RNLEELLDEGKISKDLLDEAVMRVLTLLVLLKNDY
NALPLVEK--KIAFIGPYSDEKNLHSSWAISCDSKDAVSVKKAAKELYDDKKLFKKAMEVAEWADEIVFCLGEHIGQSGESTSKTDLRIPRAQKKLFEKICKKFKHIIT
LIFCGRPLELENI-PEKSDALMICFRPGTEGGHGIMDVLGGNYSPSGKLSMSFPYSAAQAPLNYNAFSRPYLCPNEARYPFGFGLTY
>A0A1J4JGT0_9EUKA
---ARESRLKIPVLMGIDAIHGHA-FYSGGTVFPTQLGVAQSWDEELIESQGNV
TAFEIRYTGPSWTFSPVLCISRDARWGRVGETFGEDPFLIGKFATALIKGLQGPKVAACAKHFVGY--SETRGGRDSTEADLSYRKLQSYFLPPFEKAVEAKVATFMSG
---YHAIDGTPMTLNDFLLNDVLRGKWGFDGFVVTDYSNIGYLISNQYYVDASAAAVKSGNDMSMAVTDFY---QAALDAVTSGKLDMEFVNQSCRRILELIVMLKND-

GFLPLPTNIQKLAVIGPNADPLSQNGDWSLGTPRNCTITYVDGFTNRLKEKADLEEAIKIVEDSDVTVVVVGDRLVYYGEMRSTGTLELMGGQLELLRRIIETKKKFII
VLMSYKPLIIPDDIIEHASAVLLQFSPGMMGGQALAEAVFGDFSPNGRLTISIPRHAGQIPV-YYNKVHQYVLSTTPRFAFGYGLGY
>B8CZK9_HALOH
-LEEKIGQMFVVEVSTTSGRIDTRKLKGLTNVGSIFSGGSVPNNPATWTEMINNIKQVFND-NSRIPVIYALDAIHGNNKVIGA-PISPHNLGLASTWNPELVERVYGY
TSDSLEAIGVSWNYAPVLDVARDPRWGRTYETFGEDPFLVSVMGAASVRGIQKSRVCATAKHYIGY--SGSENGMDRDPSYIPKRELYEVYYPPFKKAVAEGVKTIMVN
---SGEVNGIPVHVSKWLLNDLLRMELGFSGVIISDYADISKLHDYHMYEEAIIRAVNAGVDMFMEPDNYPGFYRFLIEAVKEGTVSEERINQSVSRILKLIVLLQNKD
NVLPLSREIKSVLVVGNCAEMGNLCGGWTINWDLTTGKTILEAIKEKVAPDIYVKKVLEAASDAEAIIVAIGEEPYAEMMG-DVQNIQLPADQIKLIKALGNTGKPVIT
VLITGRPLAVGPI-LNSTPGLLLSFLPGTEGGNAVADVLFGDYNPSGKLPITIPKYTGQLPL-YYNHKG---VDYDPQFPFGYGLSY
>L0M3W6_ENTBF
TLEEKVGQLVQLSG-ELTVGPKAKLGISQQQV----QCAGSVLNVSGAKKTRHIQEQHLQNSRLKIPLLFMADIVYGYR------TVFPIPLALGCSWSPALIERCCQV
AAKEAVSAGSHVTFAPMADLVRDARWGRCMESTGEDPLLNSRIVSACVRGFQHNGLASCVKHFAGY--GAPVAGRDYNTVELSRQSLFEDYLPPYLAAVQAGCKLVMSA
---FNTIDGVPATANRWLLQDVLRDRWHFDGVTIADYAAVKELIPHGVERHASQLAFNAGLDIDMKSPCYV---NHLAALVNQGVIPEEALNQAVMRVLQLLVLLKNDN
ALLPLKPGT-KVALIGPYADNPDIIGLWAVYGETSHSVTLKQALDEVLAPQTEQARALQYAREADVVLLAMGEHMMQSGEAASRTDIHLPQHQVEFIQAIARVAKKSVL
ILFNGRPLVLSDV-VEHVDAVIEAWFPGSEGGHAIADVLYGKRNPSGRLSMSFPRANGQVPI-YYNALRPYLCPTTPLFPFGFGLSW
>M0MRD9_HALMO
TLSEKVGQLNQLNGTDSVGTVDLDAEIAAGRVGSMLN-------TDGMAARERYQRLAVEESRLGIPLLFGFDVVHGYE------TVFPTPLAEAASWDRETVRANAAA
AAAETAAAGITWTFSPPCDVARDARWGRSMESSGEDPYLAAELTHERVRGYQGTTMLACVKHFVGY--GDVLAGREYNTVDVSETALRERHLPPFEAAVEAGVGSVMTA
---FTDFERVPVGAHKGLLRDLLKDEWGFDGVVVSDWNSVRELLPHGIEREAARAALEAGTDVDMVGHIYS---KELEELVAEGTVAESLLDDAVRRVLE-MVLLEND-
GMLPLTDA-GTVAVGGALATGTDVLGEWRARGDGDDAVPILAGIENAVDAPEKRDELVETVAGADVAVLAVGEPWELSGECSSRTDISLPGDQRAVLEAALETGTPVVT
VILSGRPLAI-PWTAENVPAVLEAWHPGSEGGNAVADVLFGDHSPSGKLPMSFPRTVGQVPL-QYDELRPYVCPNDPLYAFGHGLSY
>R9NAL5_9FIRM
-LREKIGQMIQLTGMY-----FDEEAVLTGTVGEVIRYAGSVLGMDGADKIRAVQEKYMEEHPHHIPLLFMADIIHGYK------TIAPIPLGQACSFHPELVHEAAEY
AAAEASSEGIKATFSPMVDVSKDPRWGRIMESFGEDPFLNGVMGIAMLEGYQGYRIAGCLKHFAGY--GAVNAGKEYNDVELSRRTFMEQYMKPFRMAVYADPAMVMTA
---FHAVDRTPVSKNRELLEEILRGELGFGGTIISDWGAIGQLEEQQVQKEAACAAAEAGVDIDMMSPAYM---SHLEELVEEGRILESAIDRSVWRILQ--VLVKNE-
GILPLDPGQ-KVFWAGPYIDSREFLSRWAIFGDVETIETVLKGRK----IWQKQYAGVEEIREDDTVVLVLGEHEAQSGEAASRAFLDLPERQAELFDMVARRTSHIVT
VMISGRPLDLRRI-AERSEAILLAWRPGTMGAQAIVRLIYGEESPSGKLAAGIPWCTGQIPVSYWDMTHRYMIPNTPLYPFGFGLSY
>R9PTK2_AGAAL
-VEEKVGQMLQLPA----NMPENLDKLESWNVGSYLHC---------TGTMVEELQQRAAKTRLGIPLIFGIDAIHGHC--FENNTVFPTQLALSCAWSKQLSQSMARI
TAIETRACGLHWTFSPVLCVGRDSRWGRINETFGEDPWLIGELAAAAVYGYQGDTILACAKHYVAY--GEATGGRDAYEAEVSPRKLLSLFLPPFEKAVEANVATLMVG
---YQAIDGVPCSANSWLLREVPKNQWGMDGFIVTDWDNIGSLHDKQRLRHAAKIAVESGNDMIMTTPSFY---QHTIDLVKDGELDIALIDDAVSRILK--TLLQNN-
GILPLDASKPKILLCGANADVVAQLGDWSFGSHQKDAITLRQGLQAAADAFGEISSAVNAANNSDIIVACVGDTLSQHGEFHDRADLDLSGQQQAMLEALKATGKPLIV
VFMASKPLTIGWV-KQHADAIVCAFNPGAKGGQALSELLFGELNPSGKLTISFPQHVGQSPV-YYNKYWHYIMPLLPLFSFGEGISY
>A0A0W1RCA5_9EURY
TLHEKAAQLAGTYVGTTRTVEDAAEMVRDHGIGFVTPFGYPHRDSEEVVEANELQRIAREETRLGIPILIPIDAIHGNA-YVEDTTVFPHNMAVAAARDRTLAEQIGTV
TATEVAATGSSLTYGPTCDVARDPRWGRAFETFGESPYLVGELASAKARGIRDADVAAMAKHFPAY--GEPERGEDASPVDKSLSSLYRDFLPPFEKVLDEGVEGIMPS
---YNSINGEPSHGSHYWLTEVLREQLGFDGYVASDWNGINMLYENHRRREAIRQSFTAGVDVHSLGEQGH--VEHIVSLVEDGAIDEAAVDSSVARVLRL-TLLENDD
DCLPFDSNVDNLLVAGPNADLTSQVGGWSLKEDELDGITIKDGIEAQVSDPDDVDAAVAAAKEADAAVVVLGENWYLPTERFNRADLSLPDAQRDLLERILETGTPTAL
VLVSGRPLAL-PWAAENADAILHAYYPGADGGTAVAETLFGDNNPSGKLPISVPRSEGQLPVHNYLP-HPYPGEDEHLWPFGHGLSY
>A0A1I6LBI5_9EURY
TVEEKVGQLLVEPATGDEVVDTVTSNVDRHHLGQASPFGRAPDTPEAMVEVANIQAYAVEETRLGIPLLLISNAVHGNA-YAKGAAVAPHNLGMAATRDPELLEEMASV
TATELAVTGAHQNYDPICDVGREPRWGRVFETFGESPRLCGAMGAAKVRGYQGDTVLATVKHFPAY--GEPVRGEDGAVNELSESTFRRMHLRAHAAAIEAGAEAVMPS
---YAAVDGFPVHGTRKYLGDLL-DGLEFDGFVVSDHHGVRMLHDDHQLRESTLAAVGAGMDAFLDQPAAGTYADALVGLVESGEVDEERLDESVAPVLE-MTLLQNEG
EVLPFDPTVSEVFVAGPNADLDHQFGGWSRGDNE-DGVSIRDGVAAVTGDSRDLDAVARKAASADVAVVAVGESQYITGEFPTRSQLHLPDAQRELVEAVVDSGTPTAV
VLVTGRPLAVADV-ADAAPALLMAYYPGSEGGTAVAETLFGEHNPSGTLPISMPRSAGHLPTRFNRSRDTHPASYDPLFPFGHGLSY
>K9ZYC0_DEIPD
TLTEKIGQLTQINVTRPLSERWLDVVLGEHQVGSLLSGGGPNTPEAWARMTNDLQRYTLTHSRLKIPLIYGVDAVHGHNNVKG-APLFPHNIGLAATFDVNLTRDINAL
TARALRATGISWNFAPVADVGRDPRWGRFYETFGEDPTLTAQLVVASVQGLQGETVAATLKHFIGY--SVPQNGRDRQPAQISRESLQRVHLPPFQAGMKAGAATVMIN
---SGALNGEPAHSSRGLLTELLREELKFPGLAVSDWEDIARLQTVHKYQDAVRQALGAGIDMSMVPNDAPAFTSAVKTLVENGSLPLARVDEAVRRVLA-MTLLKNDS
ELLPL-KGRRSVVVVGRRAVPRSQLGGWSIGWI--PAVTVLDGMKQVLPKTYSEDLSAALPANTDAIVAVVGEAPGAEGEA-DNPGLTLPQEDVALLRRALGSGRPVVA
VLLAGRPLLLPDDVQRGLRALVMAYLPGSEGGRAVADVLYGNTSPSGRLPFTWPKSVSALPMEGAT-----PQNAQALYPFGTGLSY
>A0A5C5WWZ1_9BACT
TLTEKIGQLNLVNPGGSVANSDVHRKLVDGQVGMMFG-------TASLESRCEIQSISVNQTRLGIPMLFACDVIHGYR------TALPLPIALSCTWNLAMIERAAHL
SATEARADGIDLTFGPMADLTRDPRWGRVAEGNGESATLSSWITAAVIRGYQGDRMLACVKHFAGY--GAVDGGREYASVNLGPIELHESHLPPFEAATCAKVAAVMPG
---FHALDRIPVTAHRDLLIGVLRERWNFEGAVISDYTAINELEHHGLLSSAAVAAINAGVDVDMVGESYI---TTLEQSVASGRVDVARIDAACRSVLKL-VLLKNKS
ETLPIGKECRRIALIGPLADRSNLPGTWSVSAIAEECVSLADGLRSTVCDQAMIDEAVAVARDSDVVVLALGEAKEHAGECSSRTDLQLPPPQRLLIDAVADLGVPIVL
LVFAGRPLVLTGI-ADRVNAMLYAWYGGSMAGPGIADVLTGSHVPEGKLTMSLPRSVGQIPV--HHDARPYLEQNDPLYPFGFGLSY
>A0A0N0RTV2_9HYPO
---FSAATAFPAPIHMSAAFDFDLVFSIGEI
ISTEARAFGFASYFTPNINPFRDPRWGRGQETPGEDPFVGSQYALAMVTGLQGQRVIATCKHYAAY---DVENTRFQNDVHPSMQDMSEYFLPAFKKCVDAHAASVMCS
---YNGVDGTPSCANSWLLETVLREHFGFSNYVVTDCDAVDSLVTGLHLSAGAADALNGGTDLDCGSTFG----SNLNSAVQAGLTTEAAITKAVTRLV--MVLLKND-
GTLPITKAPGSVALIGPMCNTTQILGNYAGTPAV--VASPLDAFKSKWQNTSGFAAAIAAAKNADVIFYCGGLDKSVEDEARDRDNIDWPGNQKDLITQLAGLGKKLVV
LQFGGGQVDDSDFKSSGVNSILWTGYPGQEGGRAIFDTVTGASSPAGRLPVTQYPSSYDSVDDMTLR--PYWWYNDAVVPFGYGLHY
>A0A2Z4UDZ3_9FIRM
-LKEKIGQLTQLDASCTGAETELGFSAEDFP------YAGSILGVVGAEQIEKLQKKCMEKQPHHIPVLFMADIINGYR------TVFPIPLALGCSFEPKEAKEVGDV
MAKESAAAGLHVTFAPMVDLVRDARWGRVMESTGEDPYLNAQMAAAMVQGIQGEHLGACTKHFAAY--GAPTAGREYNTVELSERTLREDYLPSYQAAIDAGSAMVMTS
---FNTLDRIPATANQWLMKDVLREEMGFQGVLISDWNAIGELITNGVKKEAARQAIQAGTDMDMMSGCYM---SQLENLVQEGTISEDSIDTAVLRVLELMVLLENKD
HFLPLKKE-EKVAFIGPYAEEKRMLGAWSFFANPEDTVTCREALEEKTENEKMIQEAAEEAAKCEKAVLFLGESCLQSGEGGSRGDITIPEVQKKLLRAVAQANPNLAV
VVLAGRPLDIREI-KEHAKAIVYAWFPGTEGGHAIADILYGDRNPQARLAMSLPWCVSQVPV-FYGEFRHYTMPNQPLYPFGYGLSY
>A0A3N1KR67_9PROT
TIEEKAGQLNL-ETADLYLSDAAEARLRAGRIGGFFN-------MHERALARRIQEIAVHGSRLGIPVLLGYDVVHGYR------TIFPMPLAQAASFDLARIERSERI
AASEAIGDGVNITFSPMLDTSRDPRWGRTAESAGESPWWSAQVAVARLRGFQGESLAACPKHLGAN--GATRAGLDYTGAEIGERELREVHLPPFRAVVT--APCFMAA
---FNTYDQVPAAASPFLLRRILRDEWRSGAVVMSDFGALLELERHGVDAMAARLAIAGGLQLDMASAVYR---DQLPALVRAGRVPEAAVDEAVRHVLRLLVLLQNRG
DRLPLRTDVRRVAVIGPHADPQEMLGSWVGRGAYSRPVSLAAGLRQVLDPPAEQADALAAARAADSVVLALGEPASMSGEASSRTEIDLPGAQNDLAEAVLAVGRPTVA
VLFAGRPLAIERL-SQRADAILLAWFPGTMGGTAVARMLFGLAEPVGRMPMTTPRAVGQIPL-HHERLRPYVRPTTPLYPFGHGLAY
>D7BB48_MEISD
TLEEKLGQMTQVAVSKPLKGELLERYLVQRGIGSVLSGGGPNTPRAWAEMTNAIQRAAVEKGRLGIPLLYGVDAVHGHNNVVGA-TLYPHSLGLAATWNPALVEQVARR
VGQELRATGTLWNFAPVADLGRDPRWGRFYETFGEDPLLAGSLVAATVRGLQAGRVAATLKHFTGY--GQPLGGTDRSPAFLAPRTLQEVWLPPFRAGLEAGALTVMAN
---SGSLNGVPVHASRYLLTDVLRGQMGFKGVVISDWNDIDKLVGDHKFADAVAMSINAGVDVYMVPMEVDRYLQTLKELVEAGRISRARVDEAAGRVL--ITLLENAA
FTLPL-TNVKTLLVTGPAATKTMQMGGWSIDWA--PGATVLEGLQKGAPQPKDARALAAAVRASDAVVVALGEKPYAENEGNNLTG-ELPAEQYKLLRDLKALGKPVVL
VLLAGRPLAFPDDAWLVPKAILMAYLPGSEAGSALADVLFGRHNPSGRLPFTWPKLFGQVPF-TYDRYDIYP-KAEPLYPFGYGLSY
>A0A4Q9VLD7_9HYPH
-LEEKVGQMYMGGW----SPDFDLSEVTSSSIGALSG-------PPDAPTIASIAAT-ARRTRLGIPLLFTQDMIHGYR------TLFPMPLGLAASFDPRVIAAAAEG
TAREAAAQGMHLALGPMVDLSRDPRWGRVVEGPGEDVFLSRLFAEATVRGLAKGGLAATLKHFVGY--GAAEAGREYAATWIPDVQLRDVYLPAFHAGLSAGAPMVMAA
---FNALNGIPVTANRFALTTLLKGEWGFDGFVVSDWDSVSELKAHGLDSDAVALAVNAGLDVEMAGRLFP---RLLPDLVRAGRVPMARIDDAVTRVLRVVVLLKNDA

DRLPLAPP-PRIAVIGAAAAAGDHMGAWGAAGRREDVPLLLDQLRDRLAGTDGFDAAVATAAASDLVIAVLGEPWWMTAEATSRTRLGLPNHQEELLTRLAATGKPIVL
VVIAGRPMVLTET-LPKVAAILWTFSPGTMGGAALADLLLGEASPSARLPMSLPRAVGQVPI-SYDQLRPYLEEASPLFPFGYGLTY
>A0A4R2H0Z2_9HYPH
TLDEKIGQLHMVSADAPSGPPGGLRDLDQGRIGSVLN--------VWGDAAAGLQRKAVEQTRLGLPLLFCLDVVHGFR------TVFPIPLAEAASFSDGIWRETAAA
AAREAAEAGVALTFAPMADVSRDPRWGRICEGGGEDPLVNARMAQAKIAGFQGDRLAATVKHFAAY--GAGVGGREYDSADVSDYAMAADYLPPFRAAVAAGVAALMPA
---FLDIAGAPMTANRALLTDVLRKTWGFGGVTITDYNAVAELIAHGAAEDAATLAFNAGNDVDMVSGLYH---RHLAEAAALGLVALADIDAAALRVLRL-TLLRDPQ
GALPLPRQLARLAVIGALATAADMRGAWAETGDVEAIVTIADGVRAAWPQGDDPQEAAAAARAAGVAILCLGETAAMSGEAAARANPVLPDGQQALLDAVLATGARVIV
LLACGRPLVAPQL-FVADCAVLVIWGLGSEAGRAIADVLSGDASPSGRLPVSWPRAIGQIPV-FYARRRPYALPIAPQFPFGHGLGY
>A0A542LUL6_9BURK
---NMLSLMTVDEKIDALSTDSGVARLGIPSFGSTEGIHGVVQRGDQTTQFPQPPGMGASWDPALVRRAGAV
QSTEARYIGVQMQWGPQADLARDPRWGRSEEVYGEDPFLAGTMAVAFTRGIQGDRGAALLKHFLAN---SNENGRGNSSSDFDERLFREYYAEPFRMGFEGGARGVMAA
---YNAWNGTPMGVHP-LLNSLVIKQWGVD-VVSSDGGAIGNLVKLYKQKEAAVAGLKAGINQYLDTYKD-----ELRAAFKEGAITEAELDAALAR----IVLMKNEQ
HTLPLKTGIKRIAVIGPHAD-SVHWDWYGGI--PPYKVTALDGIRAALGPDNGGGAAERAARAADVAVVLVGNDPTCGREGRDRETLTLA--QEALVRQVLAANPKTVM
VLMSSFPYTINWS-KQHVPAIVQMAHSSQDQGSALAQVLFGDYNPGGKLVATWPASMDQLPPDY---DRTYMARGEPLFPFGHGLSY
>D3R0E2_MAGIU
TLSEKLGQMTQAVGAETGKGEPVEQRIREGKIGSMILV---DEPTALAAKIKHFQKMAVEESRLGIPLLFAQDVIHGFE------TVFPIPLAWSSSFDLELIQGAAEI
AAKEAAACGINYVYSPMVDLVRDPRWGRVAESAGEDPFLGSAIAAALVRGYQGESVAACLKHFLGY--GAAEAGRDYNTVDFSPTTMFNHYLLPFKAGIDAGAASIMAA
---FNVVDGVPVTANYKLLTEILRNKLNFAGVVISDYNGVLELIAHGVEKEAAAKALKAGLDIEMTTNFFT---RFGEELCRASQAITDNVDRAVTRILTLVVLLKND-
SILPIKAE-QKVALIGPFAESKDLCGCWSFSTRKNETT----DLKSGFELNGGLQRAKLLAEKSDLVILALGENDVMSGEACSRMSISLPAVQQELAESLAEIGVPLVL
CLMNGRPLLLNWY-AEHCQAILQCYQLGSQAGLAIAKILIGQVNPSGKLTMSIPYAQGQIPV-YYNHLSTYLGPNHPLYPFGFGLSY
>A0A0P7B566_9HYPO
--LSDLIVASKKEHRLGIPCVHIADAVNGPT--FRGTTLFPSTLSMAMSWNLPLYTEVAEV
LRDELMACGVNWVLSPEVDVARDPRNGRVGEMYGEDPWMNGEYAAAYISTMQEEKVATTIKHYLY---CTSTGGINQASMLGGVNHIYNTLALPYINVMKVAPASVMPS
---YSTIDGIPAHSNSYLLQDILRQQFGYDGVIVSDADAIAMLYLTHKLNDAGIKSLAAGVQLELAIGFPT-AFESLTNQAVEGV--ARNVNEAVSRILN---------
---------------------------------TKVNNTLLDAIKTEFGADTEIDTAVAAAKNAGLAIVALGSIA-VDGEGNTHASLKLPGRQEELLEAITATGVPTIL
VLTGGQAFELHGA-AQDAQAILHGFHSGEETGRALVDIITGRVNPSGKLTITFPARSEVNPV-YYNREWQFPLEKNYIYSFGYGLS-
>A0A1S3JUY6_LINUN
---LGIPYMWTQECLHGVVHARRS-TSFPQSLGLAAAFSPELIYNVSRA
IGMEVRAIHNNHTFSPVVNIMRHPLWGRNQETYGEDPYLTSVYTDHYVRGLQGDYVLASAKHFDAY-SGPEN--SSLYDSEISERDWHLTYLPAFKQCVKSGSLGFMCS
---YNKVNGIPSCVNSKMLDNILRQQWGYKGYVISDMGALEFIISVFSTVDTASASVMAGTNLELPHRLAQDGFMSIPKAVSAGKLTFDIVKEKA-------VLLKNTD
KFLPFQRTFESIAVIGPMANTDQLFGDYSGNIDTSVALNPLQALRTKALYEYNNETILETVSKTQIIFLCLGTGTMVETEGKDRTDLQLPGNQPQLLKDVMANGIPIVL
LLFNAGPLEISAALNDRVPVILECFFPGQATGEALRRVLWPKANPAGRLPNSWPTYLSQVPS-IYNYSRTYRFEGSLQYPFGYGLSY
>A0A1U7P1R4_9DEIO
TLDEKIGQVSMAHFFRGPIAADAGDVFARLLPGATLNGGGQPNTPRGWADALRGLETLGRQNPGGVPAVFGTDAVHGVNNVPGA-ILYPHNLGLGAAFNPALTQELARA
TAADMRAMNMDWDFSPVADLGRDPRWGRFYETFGESPWLVADQITASVTGLQSGGVAATLKHFVAY--GSPGLGRDRANAEISLRALHELYLPPFKAGIRAGAMSVMAN
---SGSVNGVPVHASQNILTDLLRSELGFKGLVVSDWNDIDRLVNTFKLVRATAASVNAGIDLYMVPNDVEKYEAALKEAVEGGLVSQERLDEAALRMLT--TLLEND-
GALPIKTG--RVLVTGPAMDAAIQLGGWSVNWYMPKVSTLATALKASAPADSKREALLKAAANADTIVVALGEAPAAESQ-ANNPNLSLPSGQIGLLRDLLGTGKPVVL
VLMAGRPILLPEDLQNRLPAFVMAYLPGSEGGAALADALYGRAGFPGRLPFTWPGSLGEVGL--TADREGAGDAPLPLYPLGFGLDY
>D3EFJ4_GEOS4
TLAEKIGQMYQTDPGTT------------PMTGPVSERTLSILSATDAETAYEVQKVFLEHNRLKIPLLFMFDIIHGFR------TVFPVPLGLASSWEPKLAEDTSRV
AAAEGAASGINVTFAPMADLVRDPRWGRVMESPGEDPYLNGLMAAAMVRGFQGDTVAACVKHFAAY--GAAEGGRDYNTVDMSEAALRNYYLPAYKAGIDAGAELIMTS
---FNVYDGVPATTNSFLLRKVLREEWGFEGVVISDYTSLWETIFHMSGEDAAKQGLEAGLDIEMISTEYI---SHLEQLVERGEVDVALIDEAVRRILTLMVLLKND-
DILPLKKDIKSVAVIGPFADNGRILGPWSGLGKPDEAVTVKQGLIKKLGNVSAFEAALEAAASSEVVILAMGEEDHMSGEAGSRAYLTLPGVQSKLVEEVLKLGKPTVL
VLFNGRPLELKWY-HEHVPAILEAWFPGTEGGNAVADLLFGDANPSAKLTMSFPYTVGQVPV-YYNCLNTYLIPNAPFYPFGYGLSY
>E7G5H3_9FIRM
TLDEKIGQLVQLNG-NDSNEKVATGPVQKIGIDKIHL-TGSILNTLGAKELKTLQNAYLEKSEKKIPMLFMADIINGFK------TIFPIPLGLGCSFDAELVKKTAQI
AAKEAAVSGIHITFSPMVDMVRDARWGRVMESYGEDNFLNCEYAKAMVEGYQGNTIASCVKHFAGY--GAPLAGKEYNTVELGERSLREAYLPAYKAAIDAGCHLVMTS
---FNTIDGIPVTANKWILKDVLRDEWGFDEVVISDHSAVKELVPHGIYEEAAKLAIEAGCDIDMMTATYS---NHLKDLVESGQIDIQLINESVKRVLKL-VLLKND-
SVLPINQN-QRVAVVGPYASNKNLSGMWSINVDPDKVVTIYDGLKKQGKHDEKIQEAIDIAKESDVVIVAIGEHSHQSGEGGARGDITIPKVQLELLKAIKKLGKPVVT
LVFSGRPLALKDV-AENSDAILQCWFPGTEGGDGIADLVYGKVNPSARLSMTFPYSVGQCPI-YYNHMSTYIIPNDPYYCFGYGLSY
>A0A0B2A325_9MICO
---QKLLQLQIVW----RQDAAERDALVRRGLGSTFW-------PPSAAEADALQRIAVEETELGIPLLIGLDVVHGQF------TIFPTPLAQAASFDPQTAVDDARV
SAIEARSHGVNWTFSPMVDVTRDPRWGRVVEGFGEDVHLSSVFTAAKVRGYQGDSVAACLKHFVAY--GAAEAGRDYNTADVSDRRLRETYLETFRIGVEAGAVTVMAS
---FNSLNGHPMHGNHALLTGVLKREWGFDGVVVGDADGVAQLVDHGVERAAVALALESGIDIVMGGTLLV--DGAGIPLLTPADVSPERIDDAVRRVLRL-VLLTNG-
GSLPLPATG-RILLAGPYARSLDHLGAWVQHFATPADQSLAEALARRLPDDGQIRDLVARADAADLVVLALGEPSRLSGEAASRADITLPDAQRRVIHALADAGARVVV
VLATGRPLVVEDW-VDRVDAVLCTWHLGVEGPAAIAATLSGAVNPGGRVPMTFPRAVGQVPI-HHDHERTY------TFDFGHGLSY
>A0A0P6VM49_9HYPH
TLAEKIGQLTVE----SHGPFGTRERIATGEIGNVINY--------ANAGETGDVQAEAAKSRLGIPLLTGLDVLHGYR------TLFMVPLGQAASFDPELNRRAAEA
IARETTLQGVNWTYAPMVDIGRDPRWGRVVEGAGEDPWLAARMAAARVTGYRAGGLVVTPKHFVGY--GAPAAGLDYAPADMSEATLRDVYLPPFRAAVEAGAQAIMPA
---LSALNGIPASVDSRLLTGILRGEWGFRGFVVSDWGAIDQLGNHGIRAVMARKTFAAGVDMDMASGTYR---TRFPEEVAAGRVSEAAIDAAVRRVLRVIVLLENRR
ETVPIEGR-RKVLLVGALADAREHVGPHAAQYREQDVVTIRRALAERAARERGFGAAVKAARAADVIVAVLGEPGDIAGEAASRADPNLPGRQQALLERLVATGKPVVL
VVMAGRPLIV-PWAAEHVSALLTTWYPGTYGGSRALAEILFGDTAPSGRLPMTWPRHPGQIPI-TYDRLRPYVVDWKPQFAFGHGLS-
>A0A1I5UPQ6_9FIRM
--VSQMIYASSAIPRLGIPEYWWNECLHGVA-RAGIATVFPQAIGMAASFNDKLLYRVACA
ISDEARAKGIYTFWSPNINIFRDPRWGRGQETYGEDPYLTGRMGVAFVKGIQGDKAIATPKHYAVH--SGPEPLRHSFDARVSQKDLRETYLPAFKECVEGKAASIMGA
---YNRTNGEPCCASPTLLQKILRDEWGFDGYVVSDCGAIYDIHAHHKPEESAALAVNAGCDLN--CGRV---FESLVNAVKQGLVSEETIDRAVKRLF--MVLLKNEG
GLLPLRKDLKAIAVIGPNADKKVLLGNYNGTPSK--YVTALEGIRAKVSPTAGFAEALAAAQRADVVIMCLGLSPELEGEEGDKTSLDLPGMQEELLKAVCATGKPVVL
VLFSGSPVSINWA-QENVPAILQAWYPGEEGGTAIADVLFGDYNPGGRLPVTFVKSLDQVPPTDYSMKRTYRMEDEPLYPFGYGLSY
>A0A1J4K0C2_9EUKA
TLKEKLGQMTQSPGLNEVKGNSLYDLIRNGDIGSQIL----IHPNHMIENAKLHQKIAVEESRLGIPLIFCMDVIHGYQ------TIFPIPLAWSCSWNPEQIKKAQQI
AAKEAATVGISLAFSPMVDIAHDARWGRVAESAGEDPFLGSAIARAHVEGLQGNTVCACVKHYLGY--AAAEAGRDYNTCEFSETALRNTYLPPFKAAIDAGCGSLMTA
---FNTINGVPAVGNKWLCKTLLRDELKYDGVVISDYAAVDELIVHGNGKEAAKLSVDATLDIEMSTVNIL---NNGEQLLKEGEITLDQIDDAVRRILT-IVLLKNEE
NALPLKKS-QKVAVIGPFGESKDLLGTWQFS-DFKDVPTIVDGLKAKGIEENGFESALEAAKQADVIILALGESSDMNGEAACRLDISVPAVQFELSQLIVEVRKPVIL
VLTNGRPLLLKWY-QENVTAIVEAWNLGSMTGHAVADVLVGDYNPSGKLTLTFPQFLGQVPL-YYNHYNTYLGWNDPLYTFGYGLSY
>A0A2K9E9P1_9FIRM
TLEEKIGQMCQIDG-----NENAEFWIKERNIGSFLH--------VRGEKAINLQKMATEETRLGIPLLFGIDAIHGHA--FHSGTVFPSQLALSSSWNPQLVEKVGNI
TAKEVSVTGLHWTFSPVICLARDIRWGRVDETFGEDPYLAGKLAAAMIRGYQGESILACAKHFIAY--GETQGARDASESDVSERKLRSIFLPPFKDAVDAGCATFMVA
---YQAIDGVPCSANKWLLKDVLKDELGFEGIVITDWDNVGHMHNLQKMKESCEIAIKAGNDMIMSTPDFF---ENAVGLVKEGVIPESLIDEACSRILE-IVLLKNND
NILPISDKIKKIAVIGPNADVQAQLGDWSFGPDISKIVTILEGIKKRAGEIENIKCAVKIAEKSDIVIAVVGDTIVLNGETRDRSNLDLTGAQEKLLKALKDTGKPLVV
VLINGKPLTIPWI-KENADAIVEAWNPGMEGGNAVASILFGDYNPCGKLSISFPKTVGQQPV-YYNQLWHYAVDAEPLFNFGYGLSY
>A0A4Q7P3A6_9FIRM
-LEDKIGQLVQLTG-EGEIQTGPAEELGIPE--DMVQRTGSILNVTGAEKLKGVQKRYLEQNPSGIPMLFMADVINGFK------TVFPIPLGQGCSFDPGLVEEAAAV
SARESSAAGLHVTFSPMVDLVRDARWGRVMESTGEDVCLNGVMGAAMVRGYQGKTIAACVKHFAAY--GAPEAGREYNMVDLSEKRLREEYLQPYHAAAEAGAALVMTS
---FNTVNGVPATGNRWLLDEILRKEWNYKGVVISDYSAIAELIVHGVGREASRLALECGVDIDMVTDMYA---GHLQDMVESGVISEELLDASVMRVLEL-VLLEND-

GILPLKKEGQKIALIGPAADSAEICGSWSLFWNSADVTTLRTGMEEKTQLSDLIAEAVEAAKKADVVVMTLGESPQQSGEGAARAFLDLPECQKKLFDAVYEVNSRIAV
LVFSGRPLDLRYI-VNRAAALMLVWQPGTEGGAALANLLFGDAVPSGKLSMSVPYCVGQVPV-YYARYNTYQIPNEPLYPFGYGKSY
>E0IBJ9_9BACL
TLEDKVGQMTQFDWGYESEHDLIIELIRQGKVGSIFN-------LSGAAEANELQGLIEQHTELKIPMVIGRDVIHGYR------TVFPIPLAMAAAWNPEVARQTSAA
ASTEALTDGVTWVFAPMIDVSRDPRWGRIAESIGEDPYLTAAYGRAWVEGSQIDATASCPKHFAGY--GMAEAGRDYNTVDLSDRELRDIILPPFQDAVEAGALSIMAS
---FNEINGIPACANEYLLKTILRDEWGFEGVVASDYNALVELIVHGVEEEACEMTVLAGCDMDMHSGIFT---RQLPKLVRAGRVPESVVDDSVRRILA-IVLLQNKE
QVLPLSKAGASIAVIGPLADATDPLGCWALDGRSDEVVTALEGIRQAAAEEEGFEAALEAARSSDVVVMLLGESATMSGESRSRAALDLPGKQRALVEAVAKLGKPIVA
VILSGRPLTFAWL-PEQASAIVQAWHLGVQSGNAIADVLFGDFNPSGRLPVTFPQNVGQIPI-YHYRKKTYISTTEPLYPFGYGLTY
>A0A0R2BYY5_9LACO
---EKIGQLVQLSG-DGPAKELGISPTAVKLVGSVLN-------VAGTQETVRIQKKHLSQHPHQIPLLFMADILYGYK------TIFPSPLGLGATWNPTLIQQAFQA
IGQEAQTGGINVAFGPMVDLVRDARWGRVMESTGEDVYLNSQFARSMVTGLQHGGVAACIKHFAGY--GAVEGGREYNSTNLSPVELYQHYLPAYRAGVKAGAYLVMTS
---LTALNGRPCTANEWLLKKILREKWGFKGVIISDFNSIKELIAHGLEATAAKLALQAGVDIDMKSPCYA---KQLEPLVKNQQLDPQLIDQAAWRVL--VVMLKNEG
DILPLHSQ-QRVAVIGPYADNRMALGMWAVHADYQTTSTLKEGLEKYFATEQMSTQAYKQAAAADVIIFAGGESSLQSGEAGSRTDLHLPANQLKLLQKLAKLDKPIIL
INYSGRPLVLTKL-PVQVKAILQVWYPGTRGGQAIADILVGQVNPSGRLSISFPAAVGQCPI-YYNHLRPYLAPAEPLYPFGYGLSY
>D9STW6_CLOC7
TIREKLGQMYQICIEAAFNLEDDRSLVKKGFAGSVIN-------LVDRNLIQEYQKIAVEESRLGIPLIFARDIIHGFN------LIFPIPLAQAASWNLELAEKAAAI
TAKEATICGIRWTFSPMIDVSRNPKWGRVAEGYGEDAFLVSSFAQAVIRGYQGESLAACSKHFVGY--SATEDGRDYNVTPIPPRELNDVYLPPFLSSVKAGVATIMAG
---FNDLDGIPMSGNKELLKKLLRDEWNFDGVVVSDFNSITEMIFQGFGKEAALKSIESEIDIEMVSLNYM---NFIEKLIEEGYIDTELVNKCVRRILNL-ILLKNNA
SFLPLKDDVQKITVIGPLADKIDQMGCWSMDGDIDRVVTFYESIKSYCLEETIDDVTVEHIRRSDVIVVVGEDSLMNGEKHCRAYLSLPLGQSKMLEKIAEINKNVVT
VVYAGRPLIIKDI-LSNSTSVLYAWHPGSMGGKAVTNILFGKSSPSGRLPISFPRAEGQIPI-YYSHKRPYLINHKPLFPFGYGMSY
>R6B9T8_9FIRM
---EKVGQLNQ-HLYGIIFTQELKDEVKKGGIGTIYGLYRNYETGLSMQAYNQLQKYVIEHSRLGIPFLLSSECPHGHQAL--DGYLLPVNLAVGASFDPALYERAGQV
CGRQLKQMGVDFALVSALDILRDPRWGRSEECYGEDPYLSAELARAIVTGIQKEGVAVVAKHFCAQ--GETTGGVNASAARIGERELWEIHLQAAKACCEAGVKGIMAA
---YNEIDGKFCHANRHLLQDILREQLGFDGVVMADGIAIDQLDIMTGNIRSAALALKAGVDISLWDEGYT----KLEEALKQGFITEKELDQAVLRVLTLVVLLKNE-
NVLPIGRA-EKIALIGPNADIYRQIGDYSPPMDRAGYETLKSGMEKEFGAGHEVGTAVSLAENADIIVLALGGSSSRCGEGMDTARLQLPGNQNELFTAVCALKKPVIS
VVIAGRPYAIPEI-AQDSDALLYAFYPGPMGGKAIAELLSGKYAPSGRLPVSLPRNCGQLPV-YYNHTYPYCMEQGVLYTFGEGMGY
>R7UZ23_CAPTE
---PYQFINECITGVR--WENSTAFPQAIGLGASFSPDLAFNMSQA
IARELRNTEVKSQFTPVINIMRHPLWGRNQETYGEDPWLSGQLSVGFVKGLQGDQASGGCKHFDVH-NGPENIPVSRFDAKVSERDWRMTFLPQFKTCVEAGSINIMCS
---YNRINGVPACANKKLLTDILRKEWGFNGYVISDSGAIENIVYHHKLAEAAADSVKAGCNVELTGATGSGVYFNLLNAVKQNLISEEELRENLK------VLMKNLN
RVLPLKKRFDRLAIIGPFADAETLFGDYIPNWDPKFVSTPYEGLKSLGDDNYDPKAIEKAVKGAQFVFVCLGVGSNLEREGHDRADLDLPGYQLQILKDAESREAPLVL
VLFNAGPVDLTWPLSPEVDGIIECFYPAMGTGKALYQVVTATGVPAARLPSTWPAQLHQVPSTDYNMTHTYRDGGDPLYPFGYGLSY
>A0A2K4ZN91_9FIRM
TVREKVGQLNLDHVSDRSSMEAEAEKVRRGLVGTMLM--------KGREEANYFQKVAVEESRLGIPLLFGFDVIHGHK------TVFPVPLGGASTWDPQMLEEAEEY
AAREAYADGINWIYAPMIDLCRDPRWGRVAEGAGEDPLLGSLIAQAKVRGLQTVYVAACFKHFCGY--GLSQGGRDYEECETSPRTLFMDYMKPYQAAVDAGALSTMSA
---FSVLNGQAVTGSRYYLTEVLRKQFGFRGFVCSDYGAVKELVHHRVRKEAAAQALLAGVDEDMGSHAFA---DYCEALYEEDQRFAEAVDEAVRRILSVIVLLKNQE
KILPLSPS-KKYFLTGPLSDVEEMPGAWAQYHPGTNVISVKMALEEAGLDDSGFAKALKMAEECDEIIYVCGEKAAWSGENRGRVSIDLPKQQYRYLRELKKTGRKIIS
VLMCGRAMSCQEL-AESSDALVLAWHLGTEAGHSIADVLLGSYSPSGRLPITFPCYTGQVPYH---SWRSYKGENGPLYPFGYGLSY
>A0A1R0H2K1_9FUNG
-IEDKVGQMVQVPMWNGLINMTENLMVEKYRVGSVLDSPSNLNSPQRFANFTNTFQHIAITKGSKIPIVNGIDTLRGAG-FVKGATTFTAPVNIGATFNPIHSYNAGRV
GAKDTRSIGVHWTFGPLADINVQKLWSRNYESFGEDPYLIGEMMHAGIKGVQGNRIASTFKHFIGY--SGPLNGRDEEDRYIPYNHLLEYLAPPFQRAIDVGSATGMEA
---YGAVNGQDTIASKRLL--KLKKDLGF---ILLKNEN
NALPLSPG-ENVLFVGPSFNSSYITGAWSIRMYGGYCDTILEGIEGIIGRVSGYDTIVRQARKADKVVFLFGEKP--GTETAIIKTLRMAEDQYNIAKRVIETITSVIL
ILLQNHPYLLGEP-SSISDGIINANLPGAYGGLPVAEALYGKFSPSGRQPYSYPKMDYQAPVVYYTPIW---NEYDPEFAFGQGMGY
>A0A559QQJ4_9ALTE
TLPEKLGQMMQLP----AVDENAEEYIEKYHVGSYLH--------ALDGDITRLKQLNTERSRLGIPLIFGIDAIHGHC--FEDGTVFPVQLALACSWDTALLTEVGKI
TAKEAYGAGIDWTFSPVLCMARDPRWGRTSETFGEDSMLIGELAAAMVDGYQNAPFAACAKHYAAY--GETLGGARDCGDAHVSERNMRNIFLPPFEKVAALGCKTMMAA
---YQSLNGVPCSANTWLMNTVLRDEWRYEGVVVTDWNNCGQVHTVQGMKDAVELCLAASNDIFMTTPTFF---ECAQQLIAEGAISETRIDESVRRVLA--TLVKNG-
GILPLAKGRQKILLVGDNADVLSQLGDWSFIPS-ITHRSSVITLRQALEAQADKVGLLAAAQSADLVIFCGGDALKQHGEFHDRAELDLPGNQNPVFDVLAGSGVPLIS
VLIMSKPHAINSV-MEHSDAVLIAFNPGAKAGCAIRECLFGEFNPSGRLPMSFPRSVGQLPV-YYNQAHAYICDATPLLAFGEGMSY
>A0A2H3IKM7_9EURO
--EEKIGLMGGIRVLLLTFNETTYNSIHELQ-NGILGFRYNLNNALDVLPIANKIRQETSESLLEIPYITVTDSVNSIY--LPGGTLFPSTLSMSAWWNIPLYEQVISA
IRDENVATGVRWVLSPELDVARELRNGRVGEMNL-TFELGGEKKGKVV--LINFHTATTIKHFVYA---SPTGGINTASQNGGLNHLFNDLIQPFVTVIDSQPASVMIS
---YASIDRGPMSANRFMMRTILREKLGFDGVLMSDAEAILHLYTQSKLENAALRALKAGLQLELSPAQPA-AFPTLLSSINST-LVRERIDDAVLKIL--IVLLQND-
GILPLRKGINKTAVIGPFAD-IINPGSYVPNNDRSFGKSLYQSMATAFGSTSGIKAAVQAAKEAELAVLMLGSLSVVSANP------LFDKRTDDLLNEILATGVPTVL
ILSGGQAFVLDDQ-TRQSNSILHSFLGGEYTADSLVEILIGDVNPSNKLTISVPQSTGAIPI-YYNYLD------------------
>A0A4R5DDH3_9ACTN
----------------------AAANLGPDGHGSLSQRQDDASPHAYAESSNAVQRYLAEHTRLGIPALFIGETLHGLVGPAGT-TVFPQAIGLAASWNPELLEQVGLA
TAREARAVGTHVGLSPVLDVCRDPRWGRLEETYGEDPFLVSRLGVAMVRGLQGGGLVATVKHFAGH--GAPQGGRDSAPSGHGREFLREFALPPFEAAVEAGAGGVMAA
---YSDWCYVPCNASHELLTEILRDEWGFGGFVIEDMGAIGFLHTAHATADAAVQALRAGVDQSFASALG----PPVVDAVRAGVLDEEVLDRAVRRVLR-VVLLQND-
GTLPLRADLGTIAVIGPNAG-VAETGDYSGRNP--DLVTPLAGIGARAGTDAGIAAAARIAARADVAVVVVGGSAATSGEIRDAGELDLTGRQDELIRAVHATGTPVVL
VHIGGRPNTMEWA-FDHVAAAVAAWNPGEEGGSALADVLFGDVAPSGKLPVQWPAATAQLPSDYMHTGDEYV--NTPVFPFGHGLSY
>A0A380PMR6_YERPE
TLAEKVGQLCQQPMLDQQHRDDYLAGVRAGRWGSRILADTAPGENVDPCQLNEIQRAAVEHSRLGIPLLFARDVIYGQA------TVLPIPLAQAASWNPELVQQAYRV
IAREAASLGIHWTFAPMLDIARDPRWGRTIETSGEDPWLTAQFAAAVVKGFQGDSLMACAKHFVGY--AAVEGGRDYDTTELSDNTLHNVHLPPFQAAIEAGVGSVMTG
---FCDFGGTPVTAHGELIRGWLKQQQNFDGLVISDWGSIADLTHFGIALRAAELALQAGVDMAMTHEAYE---DKLDQLVLQGRIKEALLDDAVRRVLR-IVLLKNRQ
ALLPITSVPLTLAVIGPHAHQRQHLGSWCLDGDADQVMSIYQSLCAIAGESCFSDEMIECAHRADIVILCTGESHRRTGEARNIAELQLPPGQEELIAAVGRTGKPLVV
IQCTGRPLPS-PATEQYADALLYGWQCGSEAGKAIARIIFGEQVPCGKLPMTVPRSTGQIPI-YYGRKKMYKLADTPLYPFGFGLSY
>V4IV34_9EURY
TLEEKVGQMVG--TPPHEDVAEMESEIRDHHVGSVHFGGTPHNTPEKAEVANAAQRVAVEESRLGVPVFLRAMAEHGHAAVAGS-TVFPQQLGMAATRNPDLAREAASV
AATEMRATGVQSTSSPIGDVARDQRWGRIAETFGESPYLSARMTAAMVEGYQGDSVLAVTKHFPMY--SEGVRGEDTAPNEVSTYTMRRVHVPPYQAGIDAGTGGVMPC
---YNSINGEPVHGSKRILTGLLREELGFEGFVLADYRGAEDLHRAHDLEQSIWQSVSAGMDLLPS--GGSTYTDAIVNLVESGELSERRVEESARRVLR-MTLLKNDD
DVLPLSPDLGEVLVTGPNADAHQHGGWGNVRDPDPMGDTVLDGIEAAVGDAGDVDAAADAAADADAAVVVLGEPDYVADEFPKRTQLTLPDAQLDLAKAVHETGTPTVV
VFVTGRVLATPWV-AEHVPGVLMAYQPGSE-GGAVADVLFGAYNPHGKLPISVPRSEGQVPVNYLPHPRHSHDSYDPLFAYGHGLSY
>A0A428J701_9BACI
TIEEKVGQLAQIDIHFGLDKNKLKTVIVDYGVGSILSGGGDINSPKEWAEMVNEVQKMTEQTRLNIPVLYGVDAVHGHNNVKG-AVIYPHNLAVASTFNVQLAEKEAAL
TSEELAATGINWNFAPVLDVARDPRWGRTYETFGEDPYLVSMMGKHMINGIEASKVAATAKHFIAY--SGTNNGQDRQPADISERTLREIYLPPFEAAFNAGVDTVMVN
---SAEVNGVPVHASKGLLTDLLRDELHFNGVILSDWEDVHKLTDYHQYKQANALAFEAGIDMSMIPMTL-EDIDLLIELVTEGAISEERLDESVQRILTLIILLKND-
DILPLPKDVKSILVVGPTANISRLAGGWTIGWDLTTGSTVLDEIKKKVSPEEERDEIMAVAKDVDVVIAVVGEKPYAEFEG-NTTSLALPDGQSELLATLQSVNENVVM
VTVSGRPLAIEQE-ASMVKGVLWTFLPGTEGGMAIADVLFGDYNPSGKLPITIPKHEGQLPT-YYNDR--VTATYEPLFSFGDGLSY
>A0A0S7BI70_9CHLR
TLKEKVGQMVQADL---SWSENVKQLLREGRIGSLLT-------IQDVRSINEHQHIAVEESRLGIPILVGNDIIHGYR------SIFPIPLALASSWDVDLVEAVAHA
SISEAIAAGTTWNFAPMVDITRDPRWGRIAESAGEDPLLNSKIGAAWVRGYQGYQAAACVKHYAAY--GAVESGKDYNTTDMSERRLREEYLPPYKAAIDAGVKTIMTS
---FNDLNGLPATINPLLLKQILRKEWGFEGVIISDYDSIGELIFHGVHKEAALRSILAGVDIDMMGNAYH---YHLADLVRDGQVPEALLDEAVLRILRLIIILLKNDP

RLLPLHLEGKTIALIGPFADRQSLLGCWSFDGKAGDVETLHEAIERNLPPAADLASAVAVARQADVVLLAVGETDRMSGEAHSRAHLGLPGRQQELVDAVVATGKPVIA
AVFTGRPLAILKL-AQEVPSILLAWHGGTRAAQGLCDVLLGRVNPSAKLNVSFPRSEGQIPV-YYAHKSTYLESNDPLFAFGFGLSY
>A0A1B9GJE5_9TREE
---FPRVGEIDRLGYTGLFYADGAEGIRGASYS-SAFPQALNGAATWDRNLLYRRAVA
IGQEARAKGINVQFGPGVNLMRTPNAGRGFEYNGADPYLAGQAGAQHVQGVQSQGVMSTVRHYIGN---DQETGRRWTSSNMGDRTAHELYLWPFQDAVKAGVASTMCS
---YNGLNGTYSCADPMSLGKWLHEELKFQGWVLSDFGAI----TFGEEI----DAANAGVDTTRVADPPSAPGSALSAAVANNSVSQDRLDDMVHRIVS-IVLLKNSN
DLLPFGGAGAGPGGFGPDSDGNFLGGSGSSYVIPPYQVTLLDALNQQARETVQASQASTGNLAWATCLVDVRQT---CGEGYDRANLTASWGGDQTILSVASQCANTVV
IYSACGPFNTTTWDHPNVTAILNAGGLGQEAGNSLVDVLYGVVNPSARLPYTLAQDISDYPAPDLTI-ETLN--TS-FYNEGLFIDY
>A0A2P6VDP7_9CHLO
--LGVPFIWQYECLHGMKN-MGLGVMYPAPIAWAATFNHQLTSKAATE
IGDEMRAFNASHCFGPHVGIVRDPRWGRLLETYGEDPRLSADMAYAFVTGLQGGKVAATCKHFVGNDMENWKGFTRYNFDKLSADDMRDTFLPPFEACVKANSLAMMCS
---YNGVNGAPSCVNKPLLDGTLRRDMGFKGFVLTDCTALNRIGGPRPRQEASVLALKAGTDMA--CHNYY-------ETLDPDAVAKADIDRAARRVLT-IVLLKNTG
GSLPLVAELGKVAVFGPFADEQSILGNYYSYPNH-GLTTPLEALRAALPPPQNAAADVERCKGADACILFMGTRIKKEGENMDRTSLLLNSNQRKLLQAFIKGGTKIVV
VLVHGGPLDVSELASPRIGAMLTAWVPGEG-AAAIADVLFGTVSPSGRLPVTWYRDAYSRPMDTRMR--AYYKGSATLYPFGYGLSY
>A0A2V3TTN3_9HYPH
TLEEKIGQLTMASGETLGVTPSVAEAIAAGHVGSLLN-------LWGPLSVREVQRLALERSRLKIPLFFSFDVLHGHR------TVFPIPLGEAAAFSPDLWERTARA
AAREAARDGISLTYAPMLDLARDPRWGRMAEGPGEDPWVGSRFAEAKVRGFQGDGVAATAKHIGAY--GAVTAGRDYASIDISERSLRELHLPAFKAAVDAGVAAVMAA
---FPDIAGVPATGNARLLRDILRGEWGFDGIVVSDYGAVAELVAHGVIAEAAALALKAGVDIDMTSKAYP---LGLPVALARGLVSMGEIDAAVSRVLR-IVLLAND-
GVLPLAREAGRIALIGPFAAASEMLGPWTGAGHAAESITIADGLSAALPEGAGIAKAVETARSADVVLLCLGEAADMSGEAASRARVDLPGCQRELAEAVLALGKPVVA
VLFSGRPLIL-PWLVARAAAVVAAWFPGHEAGHAVADVLTGAFNPVGRLPLSWPYDVGQIPI-FYAERRPYLVPTTPQFPFGHGLSY
>A0A150WHT2_BDEBC
TFEEKVGQLSQIAAGATSKEADFITDLKKGRVGSFMN-------VSGTEKTRELQKIAIENTRLKIPLIFGYDVIHGHK------TIFPISLAMSCSWDLKAIENSSRI
GSVEAAADGVHWVFAPAVDISRDPRWGRVFEGVGEDPWWGSKVAVAQVKGIQGATTLACVKHFAAY--GAPTAGREYAAVNVSKRSLLETYLPTYKAAIEAGAATVMTA
---FNDVDGVPATGNKWLFTDLLRKQWKFKGFVVSDFTAVRELIKHGVESEAANLAFNAGVDMEMIGGTYL---NYFKKLVKEKKIKMSTIDTSVRRVLE-MVLLKNDN
QVLPLKRG-GTIALIGPLIHKRNILGNWSLQGDSRDTVSVAAGFTALAGKEAMLKEAVDIAKKSDVIVLTLGESAHMSGEAASRTSIRIPEHQQNLLKALKATGKPVVV
ILFNGRPLCLELE-NSIANALLEAWWPGTEAGHALADVLFGDYNPSGHLVSTFPRNEGQIPI-FYEELRPYISPNDPLFPFGWGLSY
>A0A1V2M6D8_9FIRM
TLEEKVAQLRGFWMRRDDEISIEKCRELIPDGIGHICQFASNAYQADKLAKVIELQDYVKNETKSQIPIMFHEEIICGLA--AREATVTPQMIGMACSFNPALVSQNAKN
AAESIKNLGGYHVLSPMMDVITNANWARAEEGFGEDSYMVSVFSDAFISAVQENGVAATAKHYAGY-----------GVSNQEEDFFINETLFPPFEVAVKSKVATVMPG
---YHKFRDVPASVSTELLTRSLREHLGFDGVIVSDYNAIRNAHTNHKHEEAALMAIKAGIDVDLPAGFNY---AHFVELVKNGQLDESIIDASLERVLK-IVLLTNN-
GILPLQNKETNILVTGPNADCYSLLGDYSWGGNDPKLYTLLGGLEATVNEVTSFNSALEKAKHSDIIIVGVGETRYLCGEGSNRKGIDLPGNQENYINKLVETGKPVIV
VVFGGRPMAISRL-AKKCAAILYAWYPGEEGGNAIAEILLGKTNPTAKLAVTLPNSNQDVPV--CLKD----TNRPQMFPFGFGLSY
>A0A291KE00_BROTH
-VKQKVAQLLQLN--PDRNEEQITGPMTDLHVTEIGL-VGSILGVPDAENARKIQNQHLTTDPNKIPLLFMIDAIHGYK------TIYPIPLAMAGTFNPAIVEETASM
SAYETSTQGHHVTFSPMVDLVRDARWGRVMEASGEDVYVNQEMARAFVRGYQGPKIAACVKHFAGY--GMVEGGREYNTVDLSRKELFQNHLPAYEAALDEGAKLVMSA
---FNLFEGVPVSANTYIFRDILRERLAFEGVTISDWGAVKELVYHGVKEGAAELALKAGIDIEMMSSCY---IEYLEVLVERGEIETAILDEAVLRILTL-VLLKNDE
QTLPLNTEA-KVALVGPFAESDDLLGGWVIFGKRDETPVLATKFKETFTNDAEIAEAVAAAKLSDSVVLAVGETSDESGEASSRSNIQLAAAQQKLIKAVAEVNENITL
VIFNGRPLELTGI-EAYAKAILIAWFPGSEGANALTRVLSGKVAPSAKLTMSFPRAVGQVPI-YYNQLRPYLVANTPLYPFGYGLSY
>A0A559J2C5_9BACL
TLEEKIAQLLQLAA-PADSDGVITGPMKELGITETVRQAGSALGLAGAEKIKHIQQMHLKNNRLGIPLLFMADIIHGFK------TIFPIPLAIGCSWDLELAEKSAEV
AAKEAAVSGVHVTFAPMVDLVRDPRWGRVMESTGEDPYLNSMFARAFVRGFQGDRVAACVKHFAAY--GLAEGGRDYNTVDLSERQLREYYLPAYKAALDEGCEMVMTS
---FNTVDGIPATANSKLMRDLLRDEWGFDGVLISDWAAIKELIPHGVEEEAAYKAIQAGVDIEMMTSTYV---HHMPELIASGKVDEALIDEAVLRVLQL-VLLKNEE
GLLPLQPSS-RIALIGPFAKSSDILGNWSWTGVKDDAVRLDQALLAKVPDEEHYSEALSAAEQADVIVLALGEHSDMSGEGGCRADIKLPQAQLELIARLKQLHKPIVA
VLFNGRPLDLHGV-YDQVDAVLEAWYPGSEGGAAIADILYGDVNPSGRLTMSFPYSVGQVPV-YYNHFNTYLVPNAPLLPFGYGLSY
>A5Z7X3_9FIRM
TLHEKVGQLHQVAPSKHKMLSNHEDEIRKGEIGSFIS-------VMDAETANHYQKIAVEESRLGIPLIFGLDVIHGFK------TMFPIPLAESCSFDDELFEETARV
AAKESAAGGVNWTYAPMVDVARDSRWGRVAEGAGEDTYLASRFSRAKVRGFQGKRIAACVKHFAAY--GAVEGGCDYDTVDMSMPKFFETYYPPYEAAVKEGCASVMMA
---FNDLSGVPCTTNEWLIQDLLRKNLGFKGVVISDANAIKECVNHGTTEDAVKQSIEAGTEMDLGSDLYE---TLLEQMVLDGKVEEKYVDEAVRNILRLIVLLKNDN
KLLPLSKKL-KIAVVGSAASKEQMYGCWSFTGEWENAVTLVDALKKEGYDPFDKEEMMKTVKDADVIIATIEHL--NSGEAESLADITIQGQQLEMLSELKKLEKPIVT
VLFNGRPLAIPEI-VEMSDALVEAWHLGSEAGNAVADVLFGDYNPSARLTMTFPHKSGEYPI-YYNHPNTYMTPQKPLFPFGYGLGY
>Q8Y6F8_LISMO
TLDEKIAQCLQLS--PTNKNAETGPLLQEMKLTDATENAGSVLGSSSALDMIGIQEAYLKTNRLGIPLVFMADVIHGYK------TVFPIPLALGCSFDRETVRVMAEV
SALEATADGHHVTFSPMLDLVRDPRWGRVMESTGEDPFLNSELGKAMVDGYQGDRMAACVKHFAAY--GAAEAGLEYNTVNMSTRELYQNYLPAYHAAIQAGAKLVMTA
---FNVVDGVPATMNKWLNRDVLRGEMDFDGVLISDWGAVAEVINHGTPKEAAQFSMEAGVDLEMMTTCY---IHELKGLIEEGKLSESLLDEAVLRMLH--VLLENKN
RLLPLAKET-KIALVGPLASSPDILGGWNVYGEEKDGINVETGLREVFETDEDKVAVKAAVQNMDVVVLALGEKNEWGGEAGSLATIRLPEAQYELAKFVQTLEKPVVI
TLFNGRPLEVKEL-AESSDALLELWFPGTEAGRVTADLLSGASNPSGKLSMSFPQTTGQIPV-YYNHLRTYLIPNEPFYPFGYGKSY
>A0A2Z2KCG2_9BACL
TLDEKLAQLTQLGPYYGPFKELNLKPQVMKNIGSVLNG-------IGARNVIGLQTRHLQTSRQKIPLLFMADVIHGYR------TILPIPLAMGCSFDLEACERFAEI
AAKESAAAGIHVTFSPMTDLVRDPRWGRVMETSGEDPYLNARVTESMVCGYQGKRIAACVKHFAAY--GAPEGGREYNTVDMSSGVLRDFYLPAYKAAVDAGVSMVMVA
---FNTIDRIPASGNEQLLRGILREEWGFSGVTIADFNSVNELIPHGNGREAAEKSLAAGLDIEMMSTHYL---NHGADLVEQGKLDVALIDEAVVRVLELVVLLKNDH
ETLPLKRGM-KIGLAGPFATSVHVLGGWAGTE-KDPAVSLHTGISNKTSEEDEVEEAYHRLKDCDVILAAVGENQQDTGEGGSKTSLRLSANQEKLIWRLKDTGKKIVT
IVFSGRPLELKPI-LAASDALLQAWFLGSESGNSLADVLFGDYNPSGRLSMSFPYTVGQIPV-YYNAYQTYLCPNDPLFCFGYGLSY
>A0A0R1N2K6_9LACO
TIEEKIGQLLQLSADFQGDNVAITGPLKEAHLDEAALSAGSVLGISGAKTVRQVQNNYLKHSRLGIPLLFMADVVHGYK------TIFPIPLALGASFDPQVMKTASTI
AAKESAAGGIHITFAPMVDLVRDPRWGRVMESTGEDPYLNSVMAKAAVNGFQGTHIAACVKHFAAY--GAPEAGREYNTVDISEWRFREQYLPAYAAAIEAQALLVMTS
---FNTLFGVPATVNQHLMRDIILRNELQFHGVLISDWDAIGEIIHHGVLQHAADLALKAGVDIDMMSFAYA---KYLTKAAVLDTQIEQLINESAQRIL--MVLLKNNH
HTLPLNSA-TAISLVGPAADTGDILGSWSWQGDPEKTETIAAALAKQFSNLSKLDDAVNASRQQEAIIAVLGLPASESGEATSVTDIKLPTEQLMLLRRLAKLNKPLIT
IVITGRPLDLTEV-DALSDAVLLAWFPGSRGGAAIANVLSGKVDPSGRLPMTFPRNVGQVPINAYNTG-RYISANSPLYPFGFGLSY
>A9B3W3_HERA2
TLAEKIGQMRQLHG----TGETQQQLVREGNLGSVLN--------VIDADAHEIQRIAVEESRLGIPLLIGRDVIHGFR------TIFPIPLGQAASFNPQLVREAARI
AAREASASGINWTFAPMIDISRDPRWGRIAESCGEDAYLSSLMGVAMVEGFQGDAIAACAKHYVGY--GASENGRDYNTAWIPEVLLRDVYLAPFKAAADAGVATMMSA
---FHDLNGVPTSGNEFTLRQILKGEWNYDGMVVSDWASVAEMIAHGYLRDAALKGVTAGVDMEMASTSYA---EYLAALVESGALSLDLIDDAVRRVLR--VLLSNQ-
QTLPLNPQQTRVAIVGPLANAADQLGCWVFDGKPEDSQTPLQAIRELLGDQSLFGEAVAAAQTADVVIAFLGEDAGLSGEAHSRAFIDLPGAQLALVDALVATGKPVVA
VVMAGRSLVLGEL-QDKVQAILYAWHPGTMAGPALADLLFGLDNPSGRLPISFPRTVGQVPI-YYNRKNTYLVDHRPLFAFGYGLSY
>A0A060LXM6_9BACI
-TAEKVGQLTQFAGAFQEDSTPVTGRIEFPVDEEMIKQSGSVLGVAGATKLAAIQREHLKKSRLGIPLLFMADVINGYE------TIFPIPLGLGATWEPDLIEKLQTI
SAKEAASAGLHISFAPMADLVRDPRWGRVMESTGEDPYLNSLFAAATVRGLQGERVGACVKHFAAY--GLAEGGRDYNTVDLSERELRDKHLPAYEAALEAGVKLVMTA
---FNTVHSIPATANSALLRDLLREELQFDGVIISDFGAVQELIPHGVEAEAAKKALEAGVDIDMMSLSYA---HSLAALLDKHAIDSTLIDEAVLRVLQLMVLLKNN-
NVLPLTKQA-TVALIGPYADNQDLLGEWSIFGKTDQTTTLKQAVERYYTHDALLTEAIAAMEQSDYLLLALGEGKDRSGEGRSRSSITLAPCQIELARLAKETGKPVIV
ALFNARPLDLTEL-EPYADAILECWHPGSEGAQAAVALLSGEVNPSGKLTMSFPRATGQIPV-YYNAYQTYIIPNEPLYPFGYGLSY
>A0A2R4X4H4_9EURY
TTREKVGQLVGTYVGTHKGPDDVADEIREYGVGSAVPFGVTADPLAAAELANELQEVAIEETRLGIPLVIPVDATHGHAYVLES-TVLPHGLGMGATRRPALARAGASV
TASECRATGASVAYSPTADIAADQRWGRAFETFGETATLAGAFARAKVEGLQGGGVAACVKHFPAY--GQPVGGEDAAPVDVSETTLRERFLPPFEDVMAAAPAVMMPC
---YNAVGVEPAHSSTWMLEDLLRGELGFDGAVFSDWGAIRMLDEDHHHREATYLARQAGVDSASVDGPTH--AESLLDLLEAGELSEQRLDRQVERILA-MTLLDND-

GVLPLDAS-DDVLVTGPNADLVGQCGGWT--WG----TTVREGLDDALDGERDLEAVETAARDADAAVAVVGEGWYIDAEAWRRDDLSLPPAQRRLLDTLFETPTPTAV
VLVSGRPLAVTEA-AERADAVLMAYYPGSTGGTAVAETLLGDVNPGGALPVSVPRSASRVPERIEDRPHPHHDSYDPLYPFGHGESY
>Q5V332_HALMA
--E--AGIPPFRLVDGPLGIRAEGQRATAFPASIATAATFDPDLARQQGAA
MGREAAALGQDALLAPGVNIIRVPHCGRNFEYLSEDPVHAGAVGAGLIDGIQSADVVATVKHFVAN---NQETHRTTVSTEVDERTLRELYLPPFRSAVDAGVGSVMTA
---YNRVNGTHMSDHGRLVGDVLKSEWGFNGYVVSDWYGLESTVGAANPGVAAPGAA---------------EGDPLVEAIDSGEVPAERLDDMVRRVL--TVLLDND-
GVLPLADGA-DVAVIGPNIDEPKLGGGGSSETTPVHSVTPVAGIESRAEGDPSLDDAVDAAAAADVAVVFVRDA---TTEARDRDSLALPGQQDDLVSAVAATNENTVV
VVRSGGPVEL-PW-REDVAAVLEQWYPGQADGDAAAAVLYGDRDPSGRLPVTFAPAERRYPGE------VFDEDADPTYPFGHGHSY
>A0A1H4ULH1_9HYPH
TLSEKLGQLTMLAASLPPGPHNPATMVREGRAGSILN-------TWGAKEIREAQRVALEETRLKIPLFFAVDILHGHR------TIYPIPLAEACAFDRALWLRTAQE
AAEEATRDGIQMTFAPMLDVSRDPRWGRICECAGEDAFINAEYAKAKVIGFQDARLAAVAKHFVAY--GAVTAGRDYAEVDVSQRALHEIYLPPFQAAVEAGVMGIMPS
---FTDIAGVAMTAHKPLLHDLLRKRWGFEGVIISDYNAIAELIPHGVLTDAATQALKAGVDIDMMAGAYE---QGLPVALERGDVSMDEIDRAVRRVLKLIVLLQNRD
AFLPWSQAPQRIAVVGPLADPSELMGAWCMAGEIDETVGILGGMRTGFAGAQAIADAVAVAQAADAVVLCIGESRHISGEAASRTRPAPPDSQLELARAILATGKPVLL
VLVGGRPFILPQWLVDGAQAIIAAWFPGTEGGNAITDIIKGVWNPSARLSISWPVAVGQIPIHYGLRTRPF------LWSFGEGLSY
>A0A1L8QUD7_9ENTE
TIKEKIGQLVQVT--PSFFSEVDGTGIITGLMGDYEIEGESVLGSYDREEIISIQKAYLEKNRLGIPLIFMADVIHGIH------TIFPIPLALASSWNRQVAEEMAEL
SAKECQISGVHLTFSPMVDMMRDPRWGRVMEGTGEDSYLNSEFSKAFVRGYQGERIAACVKHFVGY--GAVEAGREYNHVSIDDLELYQHYLPSFKAAIDQDVKMVMTS
---FNPIKGIPSTGNEYVLKKLLRKQLEFEGTVIADWGAIAQLVTHGVKAQASSMALRAGCDIDMMTNSYY---DYLESEISSGNVSMELLDSAVLRVLSLIVLLKNTD
NILPVTKS-KKIALIGPKAVTQDVLGAWSGYGDPLQAVSLATGLSKEYEQEEEKKQAVQAAKNNEVVILALGESSEESGEAASKGSIELHRSQIELLQTIEKENNQIVT
VLFNGRPLDLREV-EKYSKGMIEAWFLGTQAGHAITNILTGHKNPSGKLPMTFPYSTGQIPI-TYNHMNTYLIPNDPLYPFGYGLSY
>V4B2C5_LOTGI
---LGIPFQWDTECQHGMNEQ--NGTSFMQDIGFGASFSREAINKMYAE
KGYYLDHTGISC-LNPISSLMRDPRWGRSQETYGEDPFLSGQLGQENIRGQHGERTSTTCMWFDVHGENIPESRFSFNAVV-SERDWRTTFLPAFRYCIKGGSDSIMCS
---YNSINGVPTCANKKLLTDILRNEWGFSGYVITDQNAAENIIATHHSIDAAVAAVDAGVNLEIVADYIKNPLKSITDAINQGKLTEDLVRERVK------VLLKND-
GVLPFSTKFNAAALVGPLAD----NKNFRLAADF----RFAAGCSTPACGQYNSSAVKDAVANTDVVFVCFGLGNHLEKENIDRYDLELPGKQQQLLEDVVQYSAKIVL
ITFNANPTNIKWAENNRISAIIAAFYPAQAAGNALRAVMTSSTTPQGRLPYTWYYSADQVPATNYSMERTYRFKEEPLYPFGYGLTY
>A0A2R4X4B7_9EURY
----------------HQTVDDVERMVRSHGLGAVATFGWNGASDVLLETVNRLQRTAIEESRLGIPLLFSVDAVHGHA-YVENATVFPNGLGAAATWDPALVERSASM
TGSEMRATGAVHNYAPVADVARDPRWGRTFETFGESPFLVSEMTAAAVRGYQSGPVAATAKHFPAY--GGSIAAEDAAPADISRDSLQNVYLAPFRRAIAEGVEAIMPA
---YSSVEGHPPHGSRWILQDLLREQLGFDGAVVSDWGGVDHLHKHFTARDSVVRTRRAGMDVESTGGADH--ADRLVALVEAGVLDVETLRASAERVLR----LEND-
GILPVAPTA-DVLVTGPNADPVAQLGGWSVTDDH----TDAVTVRAAFESRVDVDAAARAAADADVAVVVLGEDWYITGEFPTRTHLELPEAQRALLEAVAATDTPTVA
VVVGGRPLILDRE-ADLADALLWAYYPGSDGGPAIRAALTGETSPGGRLPITIPRSLGQIPID---RLRPY-PDYDPLYTLGDGQTY
>A0A4R6GAY3_9MICO
TLAEKLGQLQIVF----RPALEDAAQLVLQGVGSVFW-------PPSAAATNALQRVAVEQTRLGIPLLVGLDVIHGQR------TIAPVPLAQAAAFDPPLVEELASL
AAAEARSGGVNWTFSPMVDISFDPRWGRVVEGFGEDVHLTATMGRAMVRGYQGAAIAATAKHFVAY--GQPEGGRDYDAVDASDHRLRNVHLEPFRAVIEEGVASVMAS
---FNTVAGVPMHANRRLLTGVLKHEWGFDGVVVGDADGVRNLLPHRVLADAVALAYSAGVDVEMGGAASE------LGVEERDAIDVARLDDAVERVLRL-VLLKND-
GTLPL-RAPRRVLLTGPYADSTDHLGAWTQYFRPAIQVEVLPGVGFLSDDGSGIADVVEAAARADVVVVCAGEPSALSGEAASRSDLRLPGRQAELIRAIAGTGIPYVV
VLETGRPLVVADW-IDVAPTVLVAWHGGTEAPAAIVDVLLGEADPAGRLPMSWPRSVGQIPMYAHENTRPYLLDLGPQFAFGHGGGY
>A0A4S2DI38_9CLOT
TLEEKIGQLTQIRTSYGTKSKLNINNNQKWMIGTVLG-------KLDAAMMIEIQKEYLKNNRLGIPLLFMHDIIHGFK------TIFPIPLALSCSWDEGLVEKTARI
SAKEGSSSGYQATFSPMVDIVRDPRWGRVIESYGEDTLLNSLFGAAMVRGYQNNTLISCVKHFAAY--GAAEGGRDYNTVDISEYRLRNEYFPPYYEAIKAGAKLVMAS
---FNVLNGVPSTVNRWLLREVLRNEWMFDGTVISDWGAVKELIPHGVSKDAAELSLKAGIDIEMSTTAYF---EALPELCKDKN-MEKLLDEAVEKVL--MVLLKNS-
GVLPL-ESNKKVALVGPYANNKSILGPWSLDGDLNDVVTIYEGIKNKNIIEEDISKLMNKVTTSDVVILALGEEEKQSGEAGSVSKISLESSQVKLLERMKELNKPVIV
LLVNGRPLDLTNI-INKADAILECWFPGTEGGNSVADILVGDYNPSGRLTMSFPNGVGQIPV-YYNNLRPYLIPNEPLFPFGFGIGY
>A0A094J2F8_9BACT
-LEEKIGQMLQIAPHNFISKSDTKIHGFEYDLGQVFS-AGSVLGIGNAKEQQELQRKYLNDSRLKIPLLFMADIVHGYE------TIFPIPLALSCSWNTDTAFHSARI
SAVEASTAGIQVTFSPMADLSREPRWGRVMEGYGEDPYLLSMFVRAMVKGYQQDNIASCVKHFAGY--GAPIAGLDYNTVDMSRLSFYQTYLGGYEAAIDEGSKMVMAS
---FNTFDGIPATVNQFLMIDVLRNQLNFNGVTITDYDGLNQVIAHRVQREAAIQGVEAKIDIEMASSCYM---KNIEKLIETSIINENDINAAVGRILS--VLLKNES
NILPLSNNS-KVAVVGPYATSKSTNGVWSWKGSINDNISLSEALINQGVDENPLPTDIDMLREIDIVICALGEDIEKTGEAKSIVNLELPLFQVEWIKLAKILKKKVVT
VLYNGRPLVLNNI-D-QSDAILEAWYLGSKSNEALTDLLIGKINPSGKLTMSFPRHEGQIPI-YYNHLRPYLCERTPKYPFGYGLSY
>A0A5R9EFS6_9LACT
TLDEKIGQLVQLTP-DG----PVKEWLDSSRVGSILG-------TQKASQVYNIQKEYLEQSRLKIPLLFMADIIHGYE------TIFPIPLAMASSFDEAIIKEAARL
SAHEGTNAGIHVTFSPNADYVKDARWGRVMETNGEDPILSAALTRAFIEGYQGTSLAACVKHFIGY--GAAQAGRDYNTVDISDIEMYQNYLPAFKAAIDAGVKLVMTS
---FNTIQGIPVSGNKRVIQKTLRQDLNFEGVLISDWASIAELVPHGVGYESAELAFDAGVDIDMMSDSYL---SHLNLIVNAEN--LDKLNESVLRVLN-IVLLKND-
GILPLKNG-QNVGLIGPKATSQDILGAWSWMGQNDKAISLADGLASKSINDEYIEKAKKLAKELDIVIIAVGEKSDESGEGSSLVNIELSRKQDRLIQEISKINAKTIV
IVFSGRPLALSNI-NEEARAIIQAWFPGSEGGNALANILMGDANPQAKLPMSFPRSVGQLPNTYAQMSRPYLELNTPLYPFGHGLSY
>A0A242K0Y1_9ENTE
TLAEKIGQLVQVTPDFT---GPAAEWLKLFQIGSVLG-------THTAEQVYTIQKMYLENSRLNIPLLFMADVIHGYE------TIFPIPLALASSFSSELTEEVARL
SALEATRAGVHVTFSPMADHVMDARWGRVLESNGEDPTLSAELTRGYVRGYQGTRLAACVKHFVGY--GAVQSGRDYNHVDLSAIELYQNYLPAFQAAIDEGVKLVMTS
---FNPINGQLMSVNQSLIKETLRQEMGFEGVVISDWNALEELLAHRVKYQAASEAFQAGTDIDMMSDCYI---NH---LSEFSELDRQELDGAVLRVLTL-VLLKNED
DILPLQKT-QQIALLGPKAASKDILGAWSWIGKTEHAVSLAEGLSSGLPHAEAYQKMKDLAHKNDVILLALGEASEETGEAASKAEIQLSEAQIELVKEISAVNENVVV
VLFNGRPLDLTAI-EPYTKGILEVWFPGSEGGTAIAETLMGEHNPEGKLPMSFPRAVGQLPLSYQAYSRPYMIGNDPLYPFGYGLSY
>A0A328UC58_9BACL
TLQEKIGQLTQLGT---FDEESDGELIEEGMVGSILG-------VRGADTVNELQRIAVEESRLGIPLLFADDVIHGYR------STFPIPLAESSSWNLELLEETAAI
AAREASSDGINWILAPMVDIARDARWGRIAEGAGEDTFLGSAVAAARVRGIQRNHIMACPKHFAAY--GLAEGGRDYNTVDVSETRMRETYFPPFQAALDAGAGTIMAA
---FNEINGVPASGNGWLLKDVLQGEWGFNGVVVSDWESVDELVQHGFREQAGKLAANAGMHMDMHSLIFH---EHLANLIDKGDVKLEVIDDAVRRILRVIVLLKNEN
GTLPLRQGLKKLAVIGPLADGEAQLGCWRGQGRPEDTVSVLAGIQSLAHEAEALEKAVEAAKASEVAVLVLGETADMSGENNSRVSLELPAAQQRLLEAIHATGVPVVL
AVVNGRPLTLEWA-DAHIPAIVNGWQLGIQAGPAIADILFGEASPSGKLTVTFPRHTGQVPI-YYNAKKTYASDIAPLYPFGHGLTY
>A0A0P6X8D2_9CHLR
---KVSQMRNSAEAIDRLGIPAYYWNEGLHGVG-RNGRATVFPQAIGMAATWDTDLIYRVATA
ISTEGRACGNTIIWSPNVNIFRDPRWGRGQETWGEDPFLTGEMGAAFVHGMQGDRTAACAKHYAVH--SGPEDERHTFDANVTRRELFDTYLPAFKKLVEANVEIVMGA
---YNRLYGIPCCASPLLIQEILRDKWGFKGHFVSDCGALADFHQTHGVVESAGVALKAGCDLSCVCTYE-----HLPEAIERGLITEKDIDESLIR----MVLLKNRN
NILPLKKETRDIYVVGPNAALDCLLGSYFGINDH--MVTALEGLSLRAPEANPFDWSLEAAPAADVTIACMGLAPVLEGEEGDRESLSLPAPQVDYIKKLVVRGARIVL
VIFSGSPVVLGEL-EDMVEAIIQVWYPGEEGGKALADVLFGNATPSGKLPITFPASLSQLPADYSMNNRTYRSKETPAFPFGFGLSY
>R6MKF1_9FIRM
--EEKVGQMLQVSY--NTLSKEDYEKYKNLGIGSFLH---------VLGDEADDIKKRAEKTRLGIPPIFGIDAIHGHC-LLNGATVFPSQLAMSSSFNRKLIHDMGKA
TAKEVAADGLDWTFSPVLCIARDLRWGRINETFGEDSYVIGELGKAIIEGYEEDLIIACAKHYIAY--GEATGGRDAYDSEVSERKIREVFLPPFEKAAKVGCGSVMTA
---YGSVDSVPLNANPRMVREILKDEIGFDGFVVSDYENVLNLVTRQFLKDASKLSIEAGNDMSMNTHEFY---DCVSELIENNEIDIKYIDDAVRRILR--VLLKND-
GTLPL-KGNKTIAVIGPNADIRAIYGDWTYFSN----VTIRRGMEEVFGKENYIDDAVKIAEKADVVVCVIGDCLAQNGEYRDRADLELSGYQTELVKRLIDTKKPVIA
VLVNCKPLCISYL-KENCNAIIEDFNGGDFAGLAIAEMIGGKFNPSGKLTISFPRHSAQTPC-YYNQYWHYVLEKGYVYEFGDGLSY
>A0A7K0C063_9ACTN
-----------------ARAQLVVKALTQDEKLGLMAGDDLLGPLKAADDARFRAGTVHGVARLGIPELFMVDAG--SMGVKQGPTALAAGVSLAATFDTGAARRAAAV
VADEAAHRGNDVVLGPAVDIMRTPKGGRTFEAYGEDPLLSSRMGVEWVKTVQKAGLMAEVKHFPAN---NQEANRYRVNAVIDQRALREVYLAPFEAAVQGDAATVMCG
---YNLVNGRPSCSDRTVLGGILRKQWGFKGAVVSDWA---------MAAKNTDASVTNGLDLEMPVGAHYTP-PLLRSVLNSKKITWGDVDQR-------ITLLKNSG

GVLPLTTGT-KVAVIGKAAS-QFRTGIGSMYVKPTAVTTPLQGITARAGAGNDLNAAANAARNAQVVVVVAADA---RAEDADVKDTAL-GDQDALINAVTAANPSTVV
VLQTGGPVLT-PW-AAKTKGIVQAWYAGQHGGAAIARVLYGDTDPGGRLPVTFPAAEKDAPASSALHPNNYDGKITPAFPFGHGLSY
>A0A0R2CAN8_9LACO
--AEKVGQLTQLSG-DGPLQQENLSSADLYRVGSVLG-------VSGFEQVKKIQTNYLRKSRLKIPLLFMADVVHGYR------TIFPIPLALAASFDPQLVRQVSEF
FSREAAAGGIHVTFAPMVDLVRDPRWGRVMEANGEDPFLNSQLAKASVEGIQGQHVAACVKHFAGY--GAVEAGREYNTVDVSEWRLRDQYLSGYQAAIDAGAPLVMTA
---FNTFQGQPATGNYHLMRDILRRELGFQGLLISDWDAIGEMVAHGTLQDAAQQALKAGVDIDMMSMAYL----KLTAQKNPSSKLVKLIDQAAERILN--VLLKNKN
DLLPLAKDC-KIGLVGNYANNPDLLGSWSWQGKTAETPTIKSALQAKFTRQQLQTADLQLLKQQDVIVACLGLSAQQSGEATSMTHPQLPEEQLALLRQLQQLNKPVIT
VLITGRPLVLDPL-VKSSSGLLLLWFPGSRGAAALTQILLGEEEPGGRLPMTFPQDLGQIPL-YYNHYRTYIQVNQPAFCFGYGLG-
>A0A2N6UWX1_9ACTO
-----------------QKIAQLHGAMKTINIYDLVNQGETAEEMEQLVAQIRLERHVKGIEELGIPRFRITNGPVGVGTPSPPATALPMTIGVAASFDTELAYKYGDV
IGEETATLGQHVLEGPAVNLHRTSIAGRNFEYFSEDPYLSGVMGIEVTKAIQSHDIIAMVKHYVLN---DQEDERFRHNIEVDENVFRELYLLPFEMVVDSKVASVMSA
---YNRIRGVYATEYYYSLTTILRDEWGFDGYVQSDFW----------STRSAAPSLNAGLDHEMPDAKWL-NEENIKAALQDTSLEEKTIDRALIR-----VLLRNDD
GLLPVE-PKGTIAIIGTFAGQACQGGGGSSKVDPLYTVDPAPGMEDVLKELSNLDEAKAVAKEADLVVLMAGLV---ATEGADMKNANMFNDQNKMLDELLGLNNKTVV
VMKDSAPVLM-PW-QDKAPTILEVWNQGTEDGHVVADLLFGRINPSGKVPTTYPAREEDT--IYYNHPRHWQQGIKPLFAFGHGLSY
>A0A416EJJ3_9FIRM
-TEEKAGQLNLMPY--MDNTDEILELIREGKAGGILIAATSEIQTAPAAEIWKLKQAALHESRLGIPLLVGKDVLHGHH------TVFPIPLAMAAGWNPELVQRAAET
AALEAAADGINWIFAPMLDIARDPRWGRIIEGFGEDPYLASQFAAASVKGFQRQGMAACAKHFIGY--GASEGGRDYHSAELSDSTLHSVYLPPFRAAVMTGVHTVMAS
---FNDINGEPVTASRRLLTGVLREQLGFEGIVVSDWAAVDQLRQQGARMQAAKAAFLSGVELDMSDGCFLECLPTLIRQEKEAAGLEQRLDQAVYRVLEVMVLLKNLD
GLLPLRKSGGKIAVVGPMAEKMHHLGSWSLDGREEDVISIAEGIRRTVRESPLLDDALSAARQADAVVIVLGESRWRTGEAQSTATISLSPDQELWMECIAAVNKNVIT
VVCAGRPLILKKA-DQYSKAILYAWHSGIQAGMAAAAILFGEVNPSGRLPVTFPRNAGQIPI-YYNHRARYQESGAPMYPFGYGLSY
>D3EAQ6_GEOS4
TLAEKIGQTVQYG----RCEERELKLVAEGKIGSLLN-------VHGPKKINELQRLAVEETRLGIPLLIGDDVIHGFR------TIFPIPLGEASSWDLEGMEKNARI
AAEEAAAEGIRWTFAPMVDVTRDPRWGRIAESTGEDAYLSSLAAAKVSGFQSPTVAACVKHFAGY--GWIEGGRDYDTTDMSERTLRETVLPPFEHGIRAGALSVMSA
---FSELNGVPASGSRYLLRDILKREWGFDGMVVSDWESIEELIYHGYRKDSARKGLNAGVDMDMHSGVYL---DHLEALVQDNPELLQLLDDAVLRILRVIVLLQNRS
GILPLTGKHKKLALIGPLADRHNSMGCWAWKGRDEDVVTVRDAFQSEIAPDGGVERAVQLAKQCDVAVVVVGESEAMTGEHYNVTSITLLPCQERLIRELKQTDTPIVA
VLMNGRPLAT-PWLHEHADAVVEAWHLGTATGLAIVDVLTGKYNPSGRLPVTVPRATGQIPM-YYNRKNTYICDDSPLYPFGYGLSY
>R7I7Q9_9FIRM
-LEEKAAQMVQVPY--TVVGREEALRWAKLGAGSFLH--------VLGDDA-REVQQAALHSRLGIPVLFGIDAIHGHG-LNDHATIFPSQLSCACAWDKDIAREMGEV
TAREVATDGLHWTFSPVLCLGRDTRWGRVDETFGEDPYLAGELGEAIVRGYQGDHILACAKHYIGY--GEAVGARDACDTEMTYRRLRETFLPPFEKAFRAGCATVMTA
---YGSIDGTPFTADEKSMKAILRGDAGFDGFSVTDWDNCHSLLTAQHMPEASVLAAKAGNDMMMTSLGFY---DAAIDAVRSGKLDEAVLDDAVRHILTVITLLKNDA
HTLPL-TSVRRVAVIGANADIRAQYGDWTYFTAVRPYVTIREGIEAIGAQADNIPGAVAACRNADAVVLVVGDVYAQYGETKDRADLALSGRQLELYRELRALGIPLVT
VLLSSKPLAIPEI-AHSTDALVCAFNGGMFGGQAVAEAIFGRLNPSGRLPISFPHHSGQVPV-YYNHLWHYSLPAEPLFTFGEGIGY
>A0A2A5RJ85_9LACT
TLEDKFGQMTQTTGEHPSMEDLGFNAENIYQIGSVLG-------VSSTAVINEIQRTYLKKSRLKIPLLFMHDAIHGYR------TTFPIPLALASSFDRELVKDVAEA
VAEEMRATGLHVNFSPMVDLSRDPRWGRVMEGFGEDSFLAGELGKSMIEGYQGSHVVACLKHFVAY--GAPEAGKDYASVDMSEKEFYGFYARPYEIALSANPRFVMAS
---FNSLNGEPVTASRYLMNDLLREKFGFDGLNISDWGAVGELKNHGVDKEAGDLAMSAGIDIEMLSNTFL---TYGSKILEEKPELLEQIDLAVWKI---TVLLKNNQ
NILPVNRK-DNLILIGPFAKTQDLLGNWACKGKETETISFEQGFKNQNCPETLDEVPDKELKYATQVIITIGEASNWSGEGHSSSDLEIADDQKELIRSLKGMGKKIIA
IGLSGRPLALQSV-IADLDALLWTWYLGNEMGNAVAQLVLGIERPAGRLPMSFPRVSAQVPI-RYNELRPYQIEIGPLFPFGFGLQY
>E6LF07_ENTI1
-IEEKVGQLIQITP-DQAWNLDK---RTLYQVGSVLG----THTREQVEMIQKNYLQRSKH---KIPLLFMADVIHGYE------HIFPIPLALAASFDEKVVEKMAHY
SAKEAADAGIHVTFSPMADHVTDARWGRVLESSGEDPRLAARLTAAYVRGYQGEKIASCVKHFVGY--GAAKGGRDYNEANLSDVELYQNYLPAFQQAIEAGAKFVMTA
---FQSVSGVPMTANRPLIQHVLRELLKFEGVVISDWGAVQELCAHRVSSEAAKLAFQAGIEIEMMSNCYH---EHLEHLVSENG-WEELLDQVVWRILTV-VLLKNEH
QVLPLKK-NEKIILLGSKADSQDVLGAWSWIGKQEMAISLKQGLVDGGNDRLQDAEVVKAIQEADKIIVAIGETSGETGEAASKVHLRVPFEDQLWLDELHKMGKVTIA
VIFAGRPLVLTDI-EPKVDGLVMAWFPGSEAGHILAQLLTGDFDFSGRLPMSFPQNEGQLPLTYQQMERPYLCSNDPLYEFGFGLQY
>R2Q1S1_9ENTE
TLEEKVGQLVQVTP-DGPMLEWTMSNQERYQSGSVLG-------TTEASQVREIQAQYLAQSRHKIPLLFMADVIHGYR------EIFPIPLALASSFDEELVQAVARV
SAKEAAGEGIHVTFSPMADYVKDPRWGRVLESNGEDPLLSQRLTAAYVKGYQGDSLAACVKHFIGY--GAAEGGRDYNTVDFSDLVMYQDYLPSFRSALEAGAKLVMTA
---FNSVRGVPISGNQSLIQQVLREDLAFDGVLISDWAAIQELIAHRVQKEAATMAFKATVDMDMMTSCYQ---RELATIVTEQH-LEHELDEAVWRVLTL-VLLKNE-
QVLPLQKN-EKILLVGTKVATGDLLGAWSCVGDTAQTTSLASAFQTEFPQAEKWPEIQTAALAADKVVIAVGEAGDEAGEAASKTSLRLKPQEVDLIQQLSQLNQHCIG
VIFSGRPLVLTEV-VDHLQGIIAPFFLGSQTSGALAALLSGRRDFSGRLAMGFPRHEGQLPYSYREMSRPYLEENDALYPFGYGLSY
>A0A1I0PAT0_9EURY
---NDRVGVPPLTMVDGPLGVRALGEQATAFPSSIALASSWDPDLAREFGAA
LGREAAAHDQDVVLGPGVNIIRTPHSGRNFEYYSEDPHLAGRMGVGTIEGIQSEGVAATVKHYVAN---NQETNRYEVSADVSERALREIYLPAFRAAVDADVLSVMTA
---YNRVNGVHMSDHEHLLSNVLKDEWGFDGLVVSDWWGTRSAVDAAL---AGLDLEMPGVDLELPGEAD--DGEPLREAVESGAVDESVLDEKIERLLR-TVMLTND-
GTLPLDES-DSIALIGPNADAAKLGGGGSSEVSPVTETSPREGLAERAADGASIDDAVAAAEAADCAVVVAQDD---ATEFKDRDHIELPGEQNELISAVADAADRTVV
VLRTSGPVEL-PW-LDAVDAVLETWYPGQADGEALAAVLFGDDDPSGRLPVTFGRSAADYPTA----AGTFDHDVEPLFPFGHGLSY
>A0A6C0G6B7_9BACL
TLEEKIGQLTQITG-EAEMVETGPEY-ASHVLGGTLYTIGSVIGASSAKFTNLIQSEYLKQSRLKIPLLFMHDAIHGYK------TIFPIPLGLSCSWDENVLETAAAV
TASELRASGIHVNFSPMVDLVRDARWGRVMESFGEDHLLSGNLGRAMIKGYQKIGVAACLKHFAAY--GAGIGGKDYYAVDMSLREFFDYYGKPYEIALQEQPKFVMSS
---FNSFNGTPVTASKRMMKDVLRDRYGFDELVISDWGAVAELQKHRVGREAAELAMNAGIDIEMVSTLYL---EHFETVLAQHPALLADIDAAVMKVLRL-VLLKNDG
DALPLRAAHKRILLVGPFAGTKELLGNWACKGSFDDVVSLADGLKQADPSETLADCPADVLERSDYIIASIGESWTLSGEGHSSVDIGLEASQQALVRAVKATGKPYAC
VCFAGRPLALQDI-ADDMPALLWCWYPGTRAGAAIASLLTGQATPSGKLTMSFPRHSAQNPIHYNEYSRPYQCELGPLFPFGHGLTY
>U4KMV4_9MOLU
TLEEKIGQLIQIA--PEKIDKNVFGPLLDLGISEIFL-TGSVLGIKDAEEMIEVQKTYLEKSRHKIPLMFMADIIHGYK------TIFPVPLAMAAAFNPNLVKKAARI
SSIEAQTAGIHVTFSPMADLTRDPRWGRVVEGFGEDPYLNGVLAASMVQGYQHDNLASCVKHFAGY--GLSEAGRDYNTTDVSRLNLHQYYLTGYKKALDAGARLVMTA
---FNLIEGVPATTNAYLLREVLRDQYQFDGVVISDYDSLKETIEHGTEKDAARQGIIAGLDIEMATAAYF---RNLPALIQENKVDIALIDEAVLRVLEL-VLLKNDG
GTLPLKKEM-RVALLGNYATSKDTIGPWSWHGNPSDNNSLDDVLNGYAASE-AHLLDETSINDSDILVVAIGESRRESGEAHSKTNIKLSNQQERFINDLKQFNKKIVL
VLFNGRPLDLSGV-IDQSNAILETFFLGTCSSEAIAMLLYNEKNPSGKLPMSFPRNVGQVPI-YYNYLRPYLEKNDPLFSFGYGLSY
>A0A3M7P1S5_BRAPC
-----------------------EYAIKQKHVGSILNAPYNIAQSSTWQEAIKTAQNVAQQTKNKIPIIYGIDSIHGAH-YIQESVLFPHAISLAGSFNLDVAKKIAEI
VSIETRAVGIPWNFNPVLDVGRQPVWPRLFETYGEDPYLVGKMGEAFIQGSQGNKVATCLKHYIGY--SLPFNGKDRTPAFIPENMLREVFLPPFEKAIAAGAPTVMVN
---SGEVNGIPGHANYHYLTEILKGELNFSGFVVSDWEDIKRLHYRDKDEEAVRIAVMAGLDMSMVPYDYS-FADYCVNLAKKDAKFSERVDDATRRILKVIVLAQND-
GTLPLSNENKKILVAGPTGNLRVLNGGWSYVWGTQKKTTIFEEVNKLNSNLTNLNETVEKAKNADIIILTIGEDSYCEGFG-NIDNLMLSESQQVLADRLLELNKPVVL
VYIGGRPRIITNI-ARRSKAVLIGFLPGEKGSKAIAEIIFGKYNPNAKLSVSYPLNVNGI--TTYDYK-PMEYDANKLYTFGHGLSY
>A0A0P6WDS3_9HYPH
TLAEKLGQLTMISLGGPVADPATLADVAAGHVGSVLN-------LVGRDR-IATAQTLARSTRLGIPLVFSLDVVHGYR------TIFPVPIGEAAAFDPALWQETAQA
AAAEARREGIHLTFAPMLDIARDPRWGRIVEGPGEDPWLAARFARAKVDGFQGATLAATAKHFVAY--GAAIAGRDYASAAVSAGSLAEVYLPPFRAAVDAGVAAIMPA
---FSDIDGIPMTAHAALIAGRLRHDWGFDGVVISDWDAIGQLVAHGVLAEAAALALNAGVDIDMVSGAYR---CGLPEALERGLVEPETIDAAVLRVLA-TVLLHDRS
GLLPV-GSPRKIAVIGPFGRGADRIGPWAGLGEPDRAPSLPTALRARFPERRGLAGAVAARAADLVLLCLGEPADWSGEAASRAEPGLPGDQAALAEAIFALGRPTVL
ILTGGRPLIA-PAVIDRAGAALMAWFGGSEIAAALARILAGDAAAGGRLPVSWPRSPGQLPVE----RRPYLLPNAPQFPFGHG---
>A0A1Y1WE41_9FUNG
-------------------RTAAEYWINKVKVGSIYDTPGAWYAPQTLANITNTIQELALAKGSRVPVLFGMDSVRGAN-YVKGAAMFPAGIGLAATFQPMYAYEAGRV
AAKDTRASGYQWAFAPSADLNVEKRWSQNYRSFGEDPALLSEMVRYSVRGYQGDRVATCVKHFIGA--SYPFNGKERSTQFIPDNILFEYYLPGFEAALNSGATTLMES
---LSNLNGEALVESSFYLKKLLRDKLQFRGVMLTDYEEVRSQALDFHFTDAVYLTLNTSVDMSAATSDAEFTLDTL-DLVRGGGIHEDRITESVARILQLITLLKNAN

NVLPLKDD-DRVLFIGPHLNSTHLLGGGWIHRE---GDAIYQGLGDTIMSEDILQSIIDIAKQAEKVVIGLGES-NYADDQGDVDDMSLPEPQIELVRRISQAVRPIVA
ILVEGRPRLLKDV-AELADGIVNAYLPGMYGGVPIAEVLYGKISPSGRQPFSYPKHEYQARDTI----WQGMNEYAPQYPFGFGLGY
>A0A4Q2KED3_9FIRM
TLQEKIGQLCQKNIGDNLINEDAYDKIRQGRIGLILQP-----AWNMIDDIREAQRVAVEESRLKVPLLVHSDIIHGFD------TIFPLPIASACSFNTELIRRSAAI
SATEATVAGINVTHAPMLDIARDPRWGRIAEGAGEDAYLAGEIAKAYVKGFQEDCLSATLKHFAGY--GAAEAGRDYNTCELGERTMRNTYLRPFKAGIDAGAKLVMAG
---FHTVDSVPMTANAKYLRKILRKEFTFEGVVISDWCAPYELIAHGIEREAALAAFQGGIDVEMCSDCYD---RFLNELITAGLIDEKTLDAAVLRILM-IVLLKNE-
NILPLKPG-EKIIVTGSRCFDANLLGCWQSSRFITDTITYPQGLEKEGFHHYASTAQELISDDCGTVIVFVGEAAENSGEACSEQDIRILEEDTELLRIAKENDKKTVC
VVSSGRPMILTET-EKYSDAIVYAWYLGHSAGKSLAGILSGRVNPSGKLCVTLPRDMGQIPI-YYNHLRPYIGSSEPLYPFGFGLSY
>A9WCT4_CHLAA
TLEEKIGQLNQPMI----HGLPGLDLLRQGKAGSIINAFGGFDHLNSAEQCNALQRAALES-RLGIPLLFGRDIIHGQR------TVFPIPLAQAASFNPSLVEQINQI
AAREASALGIRWTFAPMLDIARDARWGRIAEGYGEDPLLTSRMAAAAVRGFQGDRLVACAKHYVGY--GAAEGGRDYEQAEISEPTLRDVYLPPFRAAVAAGVGTIMSA
---FLDLNGMPATANRRLLTDVLRNEWGFDGFVVSDWESVGELVQHGIRAHAAALALRAGVDMDMVSGAYL---ETLAENVRCGRVTLAEIDEAVRRILR-MVLLKNER
HLLPL-RDFRRILVAGPFVHTGELFGTWTMDGRAEDAVPLDQAFQAIAPAAAAPDLALSRAHYADAVVLLVGEHPARSGENANVSDLGLPPGQLEWITAMAAIGKPVVL
VVFAGRPLAITRA-VAQAQAVIYAWHPGLEGAAALAEILFGLATPTGRLPVSMPRTTGQAPL-YYAHKRPYVIPTAPLFPFGYGLSY
>A0A4R4BP97_9SPIR
TLQEKIGQMTQIE-----RGSLRPGDISRYFLGSVLSGGGRPNTVQGWQDMIRRYQEEALATRLQIPIIYGVDAVHGHN-NLQNATIFPHNIGLGATGDADLVRRIGKA
TALEMAATGVYWNFAPCIAVGRDPRWGRFYESYGESSALVARLGRAYIEGFKDARPVTTAKHFIGDGGTRGTSKTDTYKIDQDRTYLEEVLFPPYKAAVEAGVRTIMVS
---FSSLNGIKMHAHRELITDVLKKSWGFTGFVVSDWGGIDQIDPD--YSRAVEQGINAGIDMVMVPYDAPRFIDTLEQLVQKRKVPLSRIDDAVRRILRVVVILKNN-
GILPLGSGSGRLFVAGWAADIGIQCGGWTIDWNITAGTTILGALREALPGNAPQSGAKTASDRGELCIVVAGEFPYAEGKG-DTARPELPPREQEALREARTRFKQVIL
VIISGRPLVLDEE-SLSCDAILAAWLPGTE-GAGVVDILLGHVPSTGRLPCAWPRSVEQLPL------DTIQGNEKPLFPVGWGV--
>A0A6G7Z9B1_9FIRM
TLEEKVGQLAQITP-NTGPLHDIGYSIEDNHIGSVLG-------THTKEEVIAIQKAYLEQSRHQIPLLFMADVIHGYK------TIYPIPLALSASWNTDLVERVSQY
AAYEAATQGIHVTFSPMVDTVKDARWGRVMESTGEDVYLNRMMSAAMVKGYQGNAIAACLKHFAGY--GYTEAGREYNKVDISHNELHQFVFPPFKAGIDAGAKVVMTS
---FNLIDGIPATGNQWLLKSILRDQMAFKGVVISDWGSVGEMIPYGVGEAAAHKAINASVDIDMMTNSYL---KNLARIASNDNDVAMKIDEAVLRVLTLIVLLEND-
GSLPLK-TQQKIALVGDKVKTQDVLGAWSWVGRTDESISLYDALVAQD-------IEIVSPVDADVIIAVVGETSEQSGEAASRTSISLSEETEALLDSLRTFKKPVVT
VVYSGRPLDLRRA-KENSNSLVQAWFLGSEAGNALVDVLYGVYNPSGKLTMSFPYNIGQIPI-YYNQTRPYLAPNTPLYPFGYGLSY
>A0A6I2GEK1_9LACT
-LEEKVGQLVQLG--PGPMQAMDMTKTELYQIGSVLG-------THTKEEVKNIQSTYLEHSAHGIPLMFMADVIHGYE------TIFPIPLALASSFDPSIVKEMARL
SAKEATSAGVHVTFSPMADLVRDPRWGRVLESNGEDPLLNATLIKAYVEGYQGDTLAACVKHFIGY--GEAEGGRDYNTVDISDLVLYQNHLPAFKAAIDAGVKLVMTS
---FNTIRGIPATGNRWLLQEVLRRDLNFDGVIISDWASVHELINHRVKRDATFKAANAGVEIDMMTDNYQ---HHLVDLVKEGLISEEFINQAVMHVLQLMVLLENQQ
AILPLQKE-DQLALIGPLAESHDLLGAWSWIGKTEQSVTISEGLLSKTKQMVKDRHDYVKLKYVDKVIVALGEHSEETGEGGSKTEITLPQEQIDLLKEVYKWNQNIIV
VLINGRPLDLTEI-KMYSRAILEAWFPGSEAGNAIADVLYGDVNPSGKLPMSFPRSVGQVPLTY-NMMRAYLNENTALYPFGYGLTY
>A0A7L6N4W5_9BACT
TLKEKIGQLYQAPYFSAFDSSATIQRIKDGRVGSILS-------VHDEKVLYQLQKTAVEESRLGIPLLFAFDVIHGYK------TSFPINLALSNTWDMDLIERISKA
VAFESTKKGLHLTFSPMVDLVRDPRWGRVMESNGEDPYLSSCLAKAYIKGYQGNTIAACAKHFIGY--GLSEAGREYNTVDLSKRVLYNMYLPAFKAAVEANVQMVMTS
---FNTVFDVPSTANKALLKDILREELDFKNVIISDYTSTEEIINHGILKDVAEQCFNAGLEMEMVSESYI---SHLEELVHEGKVKESDIDDSVSRILTLMVLLKNN-
QVLPLKKET-KIMLCGPMIKSQDLIGEWAALTSKDDVVSIFDAFSKDKTLSIIEESGDEAIKNAEVIIMALGEPGNQAGEGNSKTHLHLADEQKAYFDKVYQLNQNIVL
IVFAGRPLIMTDF-ANKVKALIYAYQPGLEAGNALKRLMYGDCSFSGKITMTFPYHQGQIPI-YYNHYRPYICPNEPLYPFGYGLSY
>F4S421_MELLP
---VNGRIHFPAICMQDGPAGLRNVDDLVSAFPAGISVAATWNRKLMRARGVA
MGEEWRAKGAHVYLGPAVDVTRDPRAGRSWEAFGADPYLNGEAAYETVKGVQSQGVQTCVKHLIGY---QQEQYRFTMTSQIDDKTLKELYLKPFQRAIDAGVTCVMCS
---YNKFNGLSACKNPTLTEGILREELGFQGYVVSDWG---ATHDGNWNVKRVNETALAGIDVEMPGGFMLIGYDNLEEAVNENYVTDATIDKMATRFIS--VLLKNSD
DILPLSIP-STIALIGLDAGGTIPVGWGSGTNSLKHVVSPAIAIQDMICKTDDISSAISVAEAAEIALVFVYTG-EIEGNLGDRKNLNLLQNGEELIKAVSAVNNKTIV
VIHSVGSVVMETWDLPGVKAIIMAGLPGEQTGPGIADVLFGKVNPSGRLPYTIAKSEDDFGVKVYPEGNGYRFNQLNTYCFGHGLSY
>A0A369AW13_9FIRM
--TLEEKVSLCHGNTLFKTAGVE-RLGIPPLVTTDGPHGIRQEFKEQTYFCSLIALAATWNDERAYDFGSG
MGQEARARGKDVVLGPGINIMRSPLCGRNFEYLSEDPYLISRIAVAYIKGVQQQDAAACVKHFAAN---NQETNRLSVSVEMDERTLREIYLPGFKACVEGGVFSVMGA
---YNKLRGQYCCHNEYLLKTILKEEWGFDGAVISDWSG---TH-------DTMEAANNGLDIEMGTERPFNEADALVEAVKNGLVEESVVDDKVRRALRLITLLKNEK
GILPLENSIGSIAVIGDNAVKKHASGGMSSEVKALYEISPLEGLKKKLGGDILMEEAVKAAAMSDVAVVFAGLNHDFDTEASDRTDMSLPYRQPELIQRIYDTNPNTIV
VMISGLPVEMEPW-LKNVPALLQAWYCGMEGGNAMAEVLFGDVNPSGKLPVTFPARLEDCSAEF-----PFDNKIEPLFCFGHGLSY
>A0A1W9QH37_9DELT
TLEQKAAQMVM-----GQYGDVSAADVSGEVTGTVFASGSGSSRAGDWASMIDGYITASQSTPNSVPILFGVDAVHGNSKVVGA-VIFPHNIGLGAGKNPKLVERIGEI
TAFEMMATGATWTYAPVLSVAHDKRWGRTYESFSEDPEDVALLGAASVIGLQGRGVIACAKHFAGDGQSTSDTGVDRADVKVDEATMREYGINPYIPSIKAGLGSIMVA
---DTTWNGVNMTGHEQLLTEILKGELGFKGFVSTDWD--AAM--PDQKGPGVIAAINAGVDMLMAANDWKGQRSAIINAAGN-EISQERIDDAARRVLT-LVLLKHEN
NVLPLMQGS-KAWVAGSGANLGRQTGGWTINWDMTEGTTILQGIAKAA-------TVVSTPEEADVAIVVLSESPYARGDVASINT--LPAGDFELLAEARASGKPVVA
VILSGRPVLITDH-LDKADAWVAAWLPGTE-GDGVAEVLFGLYPFKGKLSHTWPRSEEQVTL--KKF----DETYDPLFPYGHGLTY
>A0A387BQ62_9LACT
TIQEKIGQLTQLGG-EQTDNQEITGPIAENKISEAEVYTGSVLGVYGVENIRKIQSDYLEKSRLKIPLLFMADVVHGAR------TIFPIPLGLATSFNPELARKTAEI
AAKEATAAGLHVTFAPMVDLSRDPRWGRVMEGTGEDPFLNGVFAERFVRGFQGDHLAACVKHFAAY--GAVEAGREYNVVDLPENKLRELYLPAYKAAIDAGVALVMTS
---FNTINGVPATGNKWLMKDILRDEWGFDGVVITDYGAVMEQVIWGTAEEAAQKALSATVDIEMMTTAYL---QTLEKLVKTDKKLSVLLDEAVRRVLELIVLLKNDK
KSLPL-KKADKVSFLAPLLDSTDLLGSWSWKGDVTETESILSVLTEKE---------CCPTKEADKLVIFVGEKSAQTGESKSYTNISLTQEAIDLVGQYADGKQEIIL
VVFAGRPLDLSTI-ADRVDSILYAYFPGTMGARAIVDLLYGTQNPSAKLTMSLPRSSGQIPI-YYNQYRPYQCLSTPLYPFGHGLSY
>I2F843_9BACT
TLEEKIGQLVMYG----KYGEAQQKALAEGRIGSFLN------NRGEETQ---MRQNLALDSPTGIPLIMGDDVIHGFR------TIFPIPLALSCSFDLGLIEETCAL
SAREAAMEGINMIFAPMVDISRDPRWGRVAEGAGEDPYLGSEVARARVRGYQRNKTAACAKHYVAY--GAPQGGRDYDGADISERSLREIYLPPFDAAVKAGVMSVMSS
---FNDLNGIPVSGNERAIRGILRGELGFGGVVVSDWESVEELVNHSIGREAARLGFKAGVDIDMNSGVYE---RYLKELVREGKLTVEEIDQAAGRVLKLIVLLKNR-
DLLPLRKGT-KLAVIGKLADRPDMLGCWAGQVEVQESVTVLEGLENLGEEEGGIDRAREIASKVQVVLMVLGETSSMSGENRSRADITIPAAQRRLLRSVMEVNENVVL
VVVSGRPLVLSWE-EANVPAILQLWQPGHQAGNALAEILYGIHNPSGKLTLTFPASVGQIPV-YYNRKNTYIIQDEPLFPFGWGLSY
>A0A6S6QYD9_9HYPH
TLEEKIGQLVMTGI----DSPLGPDAIETGRTGSLISF-------NDAAAI-AEAQAKARNSRLGIPLLVGLDLLHGFR------TLFPVPLAEAASFDPALAARNAEL
AAREAVPAGLNWTFAPMVDVGRDPRWGRIVEGAGEDVRLAMDFAAARARGFRAGGIAPTLKHFAGY--GAVAGGRDYDAVSVSDYELQNLHLPPFRASLGP-MTTVMSA
---LTVTNGIPASSDVGLLRGLLRDQWKFNGVIVSDWGAIDGLVKQGTAQDAVRQAMAAGVDIDMASGFYA---AHLAGEVKAGRIAMSNLDAAVRRVLA--VLLKNEN
QILPLKPDIKKIAVVGPFADAFEQLGPHEARGRPEDAVTLLKGITERAKGDKGFRQAIEAAKSSDLVIAVLGEKREFSGEGASRAFLDFWGRQEDLLEALAATGKPVVL
VIVAGRPLDLRRA-SEIVPSILMAWYPGTEGGNGIADILFGDEAPSAKLPISWPRSVGQSPFSYDTPARPIDENPTPLYPFGHGLTY
>A0A518D915_9BACT
TLAEKVGQMTQADLGGLK----DFRDIATLSLGSVLSGGDEGNAREAWDDTYQQCQRQAMASHLGVPILYGVDAVHGHNNVLGG-VIFPHNIGLGCANDPDLVEQIARL
TALEVRATGIQWTFAPCITIPRDDRWGRTYEGYSEDPRRVAELGAAAVRGLQGGSVLACAKHFVGDGGTSALVGLDQGDTRCDEATLRRIHVAPYPPCIAEGVGTIMPS
---YSSWNGVKCTMHHPLLTDLLKEELGFDGFLISDYDAIDQC--HSDYKTAIGLSINAGIDMAMVSKRYKQYIRLLTELVEEGTVPMARIDDAVRRILRVLVLIKNN-
GVLPIRDSVRHVRVAGAKADMGVQCGGWTIDWVTPGGTTLLQGVREVATGEVTHTVDGRGVEGAEVVIVVVGEAPYAEGVG-DDAELGLPLEDLALIAEAQKSSAPMVL
VLLSGRPIALDDEVIAGADAIVAAWLPGTE-GAGVADILFGAASPTGTLSFTWPHSADQHPINVGDE------KYQPRFPFGHGLRY
>A0A1Z5SL14_HORWE
---QDNENGVRGTDQS-SGFPSQLSIGASWNRSLALERAQF
LGREFKASGANVVLGPVGPIGRIARGGIGRAGFSNDPYLSGQLIAPTVTGIQE-SVIACVKHWLFNEQETNRNGNQAVSSNVDDRTTHELYMWPFQDALAAGAGSVMCS
---YQRANNSYGCQNSKLMNGLLKGELGFQGFVVSDWYAVH----------TGIAANEAGLDMVMPSSSFL-TPESLAEAVNNGSVSAERLTDQATRILA--VLVKNTE

GALPLPSSTHTLNLFGYDAIGTILSGGGSGAVKVAWSISAHDALASRARTHTQFVDQKPTVKAPDACIVMINAQ---SSEGWDRSGIRDE-YSDTLVQNVASQCKNTIV
VIHNAGVRLVDSWENPNVTAVIFAHVPGQMNGEALAAILYGEQSPSGRLPYTHFIQQDITPR----------------FPFGYGLTY
>R9MUP5_9FIRM
TLEEKVAQMVQPE-----QAGITSSDVEQYGFGSVLSGGGSGNRPENWQERVNELKAAALNTRLGIPLLYGIDAVHGNNNVYGA-VIYPHNIGLGATGDLELVERIGEA
AAEEVRAVGIQWTFAPTLGNPQNECWGRTYECFSEDLEEVSKYGRAYIRGFQGEHVLACAKHFVGE--GYTDEGVNQGNISMTQEELESGVIDPYTAALDEGVRTVMVS
---FHSIDGVKCHENKHLITDILKGELGFTGLVVSDYNGIQQLSGV-TYKEQVRQGIDAGIDLFMEVYVWEDFIKYAKELVEEGSISKEQIDDAVRRILRVLVLLKNDK
T--ALLQNAQNIRVCGQKAYLGSQCGGWTISWNITKGTTIIEGIASQIMLTISHDLKGEVLEENDGVIVVFGEGPYVESGG-DRADLKISAADEEMLENLRRQDIPVIG
IIIAGRPVNITEY-MDIFDAVIMAWLPGTE-GEGVADVLFGDFDFTGKLNFTWMKNPEDMDEKFKEG-----NEDKILFVRGFGLD-
>A0A1G4VAB2_9FIRM
TLEEKAYQCVQ-----GEQVNVSVEDVKNTGIGSVLSGGGSYNHPEHWQKRVNELKAAALETRLGIPLLYGIDAVHGNNNIYGA-TIFPHNIGLGAANDPELMEEIAHV
VAREVRAIGVQYSFAPCLANPQNERWGRTYEGFSEKTADVAKLAGPFVAALQGNAVIACAKHYIGE--GYTTEGVNQGNVEMSAAEFDEGVLDPYKACIDNNVLTVMPS
---YNSIDGLKCHENKHLLTDVLKEQLGFKGMVISDYNAIGQC--KGTYDEEVANCMNAGVDMFMEAQEWDRCAQTIIKLVKAGKISEERLDDAVSRILRV-VLLKNGN
GKLAMIKEATNITVCGSGAFIGRQCGGWTISWNITLGTPIVQGIYDEVKEKVSHSVRGELDAASDVIVTVVGENPYAESDG-DVKPSDLKSGDVKLLENIEN-GPLKVL
IVIAGRPIDINDY-VDKYDAVVMAFLPGTE-GEGIADVLFGDYDFTGTLPITWLKDFKKIDE---KH----EPESEILFPYGYGLN-
>A4BA26_9GAMM
TLRQKVGQMTQ-----GEIQHVRPSQAKEYGLGSVLNGGGPFHVLEDWLELADAYWLASTESATGIPLLWGTDAVHGHNNLQG-ATLFPHNIALGATGDLELVRSIAAV
TADQVRASGVDWTFAPTVAIADNPAWGRSYESFSQDADAVFHFAKAVVEGYQQGGILATAKHFIGD--GATRNGVDQGDAWVSEAILRERHAQGFYGALDADVQVIMAS
---FNSWWTKRLHGHEYLLTDVLKKQMGFDGFVISDWNGINDV--YQCLPNSCPQAINAGIDMVMVPTAWKAFIDNTVASVEAGDIPMSRIDDAVRRILRVTVLLKNND
QVLPLNPAG-RYLVTGLAHRIAIQAGGWSLNWS----ATLLDGLREWTGANNGSLQVVNEKLDADAAIVVLSERSYAEGEG-DLTAWQSSAAEKQTLSAIQQRYPEIVT
IVIAGRPLWMNPQ-INVSDAFVMGWLPGTQ-GAGIADLLFGEHPFTGRLPFNWPADDCEGPR----------STRRAAFAVGYG---
>W4V508_9FIRM
---------------------ALERVLIEYGVGFIFYGGTKDNTIEGWRNVCDELKKYTDKTRLKIPVLQGLDAIHGFN-YLSNATIYPHNLGVVSTWDDSLAYRLAKN
VEKELRYAGMNFVFAPNGDIARDPRWSRVYESCGEDPYLAAKMTEQYVKGYQESKLFACAKHFLAY--GESSTGRDREPIDISERTIREIHLPSFRAAVQAGVKMIMLN
---SSSLNGIPVHKNKWIMQKLLREELGFQGLIVSDWADLQKIYTRHMLKSGIIAAINAGIDLNMVPDNL-DIIDIIINAVENGLIPMKRINESVERVLRL-VLLKND-
QVLPISDNHKRILVVGEAADRRHLCGGWTMGWDIQSGDTLLTALRKNSKEAKDEIELSKYKETADMAIMVIAEEP-YAEEGCDLPELCVPQEQLDLLKKLRKNQEKVVC
VLISGRPLVIANI-LELADAIVWCCLPGTQGGTGIADILLGKVNPSGKLPISFPRSSSDLPV-LYNSRN--MTTYNPLFPFGYGLSY
>R7IED0_9FIRM
TVREKIAQMQHPTYLPGKIPSYLNKWCNKEGIGMLLI--RELNSVEAAAVSMNTIQEYAEGSRLGVPVLVSMDSVHGLSYVSG-ATVTGHNLALAATRDEDLVTRLAEI
ARDEHIAIGVRMTLSPEADIASEPRWGRVMETFGEDPDLVTKMVTAQVVAFQNGAIVACMKHFPGA--GPQMEGKDTSPIISSAETL-QIHLKPYYAALEVNIASIMPY
YSVPLELDMESAIGSKATLQDLLRDEMGFEGIIQTDWGMIWAIQEGRGEEVSDEEAILIGITESRVDG----IIDQMEELTNAGKIDEDILTAAATRIVK-MTLLKND-
GILPLQNEKQTILVCGPRAGMDSLVGGWSSAQEG---LTIADAVAAYAGESDNVERIAELAKDADIIIVSVGEPSYQHDPPWGYDTLEITGSQQEILEAAKASGKPMVT
VVTGGRPYILTWC-DENTNAILEAYYPGSQGGIAIAETLFGLNNPTGKTPLQFPRDMDSVRN--QEG-D-VSDLENPLYDYGWGLSY
>A0A194VF47_9PEZI
--RLGIRSLKTTDGPAGVRGATDGTTFIPCGISLAATFDPALIERVGDI
LGAETRSKNSNVLLAPTMNISRSPLGGRNFENFGEDPYLSGIMATSYIKGVQKHGVGACMKHYVAN---DMETRRFNMNQQIDERTLREIYLKPFQMTLAAQPWTAMAA
---YPKINGHHADCSTFLLQDILKKEWGFDGLVMSDWG---------GT-NDTVESILAGTDLEMPGPPI-RYGKALVEAVKAGKVSEKHLNASVMRLLRLIVLLKNS-
GILPLPQKIKKLAIIGPNAKTPTTGGTGSAALRI----------DEQA----LFDEAVAVATEAEIVVLVVGHNNDTEKEGIDRTSLSLPRRTNELVEAICRANANTVV
VTQSASAIAM-PW-AGAPPAIVHAWYQGQENGNALADVLLGHVSPSGKLPVTFPSRLEDHSHD----A-EFDKNIQPLWTFGFGLSY
>A0A1M6SZE5_9FIRM
TLEEKAGQMLQAE-----RNTVKESEVTKLALGSVLSGGGSYPGKNTLSDMIHNIQAAAMKSRLGIPLLYGVDAVHGQN-LIKGAVVYPHNIGLGAANDADLMYQMGAA
VAEEMKLTGTLWNFAPCVAVGQDPRWGRTYESLSSDPKIVSTLSSAYLRGLQEHGVAGTAKHYVADGGTSYGSGLDRGDTDISKSKLFKIHLAPYKQLVKSGVKTVMAS
---FSSYQGTSMHENKYLLSDVLKGELGFKGFVVSDWEAVKDLEG-DSFEEKIAFAVNAGVDMLMEPYDYAKAIDGIVANVNKGTVAMARIDDAVKRILRVLVLLKNKK
NILPLKQG-TTIFVTGPAADMGVQCGGWMSTWKAAEGTTILEGLEEYAEQDLNIITDKDKAGEADVVILAIGEIPYAEYEG-DTRDLSITEENKESIKFAKSLGKPTVA
LIVAGRNVLISNH-VKNWDSIVMCYLPGTQ-GDGIASVLTGETNFTGKLSMPYYKSIKDIGK----------KDAEYLYPLGYGLTY
>R1DJC8_EMIHU
TLREKLGQMMQPDWRTPLTDEQCDRSVGRHCLGSVLGGGGPNEPLAWQAQAAAMQRAALRA-SSGLPLLVCNDSAN-----LRDATLFPHHIGQGCMRDEGLVEELGAL
AARESAACGINWIFSPCAAVALDLRWGRTYESFSEDPALCGRLSAAEVRGIQRCPMAACAKHWVADGGTALGTGLDQGNADCDDAELRATHVAPYLPALAEGVLTVMVS
---YSSLNGVKCHASRALVTGLLKEELGHARLITRDAPRLGLLKEELGFRGVV------GSEERLFGDLTWRRLEELEAAVGRGDVPAERVDDAVRRVLRV-VLLVNEG
GLLPLPAGEGELLVTGIAADLGRQCGGWSLEWAFTTGTTIADAIRAVQPG--ARLAPSAGAASRPVAVVVTGEAPYAEGFG-DVEELRLPPADVRAAVALADAGWAVVL
LLVTGRPLLLPPPLLSRVAAVLVAWLPGTE-GDGVADVLFGRAPATGRLSFSWPHAMDQAAA--AA------RLKGPLFPFGHGLQ-
>A0A4R3VMD8_PELSC
-LRQKIGQMTQPE-----IRAITPDEVRDYAIGTVLNGGGRNATPARWRALSEQFQAAARQATPGVPLLWGTDAVHGHNNVRG-ATLFPHHIALGATRDADLVRDIGRA
TASAVRASGLHWTFAPTLAVVQDVRWGRTYESFGADPALVRRLGQAEVEGLQDGGVLATAKHFIAD--GGTRRGIDQGVSHATATELAQVHGAGYLGALDAGVQTVMAS
---FSSWTDSKMHGNAALLTGVLKDRLAFDGLVVSDWDGIAQ--LPGCTVDDCPAAINAGIDIVMVPFHWKPFIANTVKAVEQGRIPMARIDDAVRRILRVLVLLKNRP
GLLPLRPTA-RVLVVGAAADLPHQSGGWSLTWDFPQGTTLLAALRQRLGGAFDADGSRSDPARFDAVVAVLAESPYAEGEGDNLRHSRRQPQDLALLQRVAGRGAPVVT
LLYSGRPLYVNDL-ANHSDAFIAAWLPGSE-GAGLVDALVAPAGFTGRLPFPWPAGPCQF---------------------------
>A0A0M9UBR3_9CHLR
TLAEKIGQMTLIE-----KNSISPDEVRELAIGGVLSGGGDENSPEAWARMVEAYQQAALETRLGIPILYGVDAVHGHNNVYGA-TIFPHNVGLGAANDAELVARIGRA
TAVEMAATGIYWNYAPGVMVPQDIRWGRTYEGYAERPEHVAALASAFVRGLQSPLVMSTPKHYLGDGGGTGDSGYQIGDTQVDEATMRALHLPPYQAAIEAGAKVIMAS
---YSSWNGEKLHASLYWLTDVLKGELGFDGFVVSDWGAIDQISPD--YYEAVVTAINAGIDMNMVPYDARRFITTLTQAVEAGDVPEARIDDAVRRILRV-VLLKNEG
NVLPLSKDVGHLFVGGLAADMGIQAGGWTIEW----QGTILEGIQAAVSPNKFGNFQGDPSADDAVCIAVVGESPYAEGRG-DSADLRLPTKEIRTLNRMEEACARLVV
VLVSGRPLIVTDH-IDAWDAFVAAWLPGSE-GAGVADVLFGERPFVGRLPYTWPRSVEQVPL------G--RSDEPPLFPYGFGLT-
>A0A2Z7A0Q1_9LAMI
--RLGIPGIDLADSAVGVRTATFQGTLLPSTLGAASSWNPQAAFLYGSV
IGRELRAMGFNMSIGGGVDITREPRNGRNFEYAGEDPLLAGTMTGTLMKGVQAQHLMGDIKHYAFN---DQETGRTTYSADIGKRAARESDLLAFQIAIIAHPSGVMCS
---YNRVNATYACENHWLLTDVLKHDFGFKGWVLSDWG---------GTHSTVKAAL-AGLDQEMPSDRYF--GEPLKQAVLAGKVPMARLDDMDHRILR-MVLLKNEG
HLLPLAASIRSIALIGSHADVGVPSGGGSAQVAIYFPSSPLKYIRKHAPAGTDPAAAAALARKAQVAIVFVNQP---MREGMDRPTLSLPYGQDALVAAVAAANPHTLV
VLETGGPVAM-PW-AGQVQGIVEAWYPGIGGAQALADLLFGKADFSAKLPVTFPRNDDQLPHA-AK---PYKFEEKHLFPFGFGLSY
>B8I1R3_RUMCH
TLEEKVGQMIMAE-----KDYISAHDVKTYGVGCVFAEGGKDNNPDGWRSMIETYKKAANDSRLSIPLLFATDAVHGNN-NMKDTIIYPHNISLGATRNGKLTRQIGAA
VADELKAIGVDWTFSPCVAVSNDIRWGRDYECFSETPDLVTMMATALITELQNKGIIACAKHYVADGAVEFGSGTDRGNTNISTEELKDKYISVYKDAVKSGVKTIMVS
---YSSIKGRKNHSERDLIEYKLKQDIGFQGIVISDYEGVEYL-DGNSLYTKVVNAVNAGIDV-LVEGKRWKEYKCLLEAASQKRVNMDRIDDAVSRVLRVLVLLKNKR
KILPLKK-SDKVAVIGPASDIGVQCGGWTKTWK--WMSTILDGFKEMADKGGLIITDPSRVKEADVVLAVLGEHPYAEGKG-DEKALGLSKENAEVLRIAYQSNKPIVV
ILVSGRPRIITNE-INKWDAMVEAWLPGTE-GRAVAQVIYGENNFKGRLPVSWPKSVEQLPIEKLDN----NEVYDALFQYGFSLKY
>S0FQH7_RUMCE
TLEEKVGQMLQVE-----RRSISSKDIQKFFIGSVLAAGGEKNTMVEWRKLTDEYKSAAVHTRLGIPLLFAIDAVHGNN-NLENTVIYPHNIGLGAGGDSELAGKIAGA
TALELNAAGIDWNFSPCVAVSNDIRWGRSYESFSENPDLVSIMSIPYITNMQKNGVVACAKHYVADGAGTGDSGYDQGNVNINQKDLNDTCLSVYQEAINAGVKSIMVS
---YSSINNEKNHGNKYLLQDRLKDDMGFNGIVISDFEGIHQL-KGGSLYDKVVLAVDAGIDVLMEASQWRECYEALIEAVGNGDIRTERINDAVTRVLRVLVLLKNKD
NIIPFGNN-QNIAVIGPAADIGIQCGGWTKTWRWMSGTTILDGFKELAGKGHKIITDIAKLEEADIIVAVLGEYPYADDDSMDLVNGTALADNEKTLKAAYAAKKPIVV
ILVSGRPRLITDE-IDRWDGLVQAWLPGTE-GGVIARAFYGDAEFTARLPVTWPRNLEQLPITLYKQSDGYN----ALFPYGYGLN-
>A0A4P6PZN7_9ACTN
TLEEKAGQMTQVNH---PNVMDNKEALAEYGIGSLLSGGGAGGTASEWADMYDEYQRAAMKSRLGIPLIYGVDAVHGHSNVEGA-TIFPHNIGLGATRNPWLVRKAQNI
TRKEVLGTGIDWNFAPAVSVPRDDRWGRTYEGFGEVPWLASSMGRASVRGFQGYSVAATAKHYVADGTGEGDYQIDQGNAQMSERELRRIHLPPYRAAIRADVASVMIS
---FSSWNGLKMHQHEYLVNDVLKGELDYDGVVISDWAGVRQVEG-DSYAEKLRKSINAGLDMIMVPNDYKQNIDAIVSEVRAGRISEKRINDAVRRILD--VLLKNDG

NVLPLRKSGKDIVVGGKTADLGYQMGGWSITWDTTEGTTFWEAIQAETEGTRTDVEFVGTETSGDIGIWVGGETPYAEGFG-DDGDLQLSEADTRQLNDICSKTDVCIA
MLVSGRPMIINEE-LKTADAFVASWLPGTE-GAGMTDVVFGHERFRGRLPVTWPSSVDQQPINLGD------HRKDPLFRYGYGL--
>A0A4R4SR02_9ACTN
TLEEKAAQLFVLQIHGLYGADNAAQVMARYRPGGFIYYGENVRDPQQVAAFSNGIQRAAAAQPHRIPATIATDQEGGIVARLQPPTQSPGAMALAAGRRTADARALARI
TGRELRAVGIDQNYAPDADVNVDPANPIGVRSFGSDPGLVASMVTAQIRGYRSAGVTATAKHFPGHGDTTTDSHVGVPQIDHTREEWERLDLPPFRAAIAAGVDSIMTA
HIVVPSLDPSPATLSRPILTGILRERLGYRGVVVTDALDMDGVRAKYGDERIPVLALKAGADVLLKPPVGEDGLAAVVEAVRSGELTERRIDESVYRILA--TLVRNDA
GVLPLRPGARDVLVTGWGVSTTAVLGTEIARRGA--TTTIRQTGAA--PTPAQIDEAVAAAREQDLVVAVTNRA----WDAEDEPGHNGP-GQMDLVKALLATGTP-VV
VVAVRDPYDIAWF--PEADTYLATYSYTAEALRSAAAALFGELNPRGRLPVAIPVRDR--PG-------------TALYPFGHGLSY
>I5AXQ2_EUBCE
-TEQKAAQMVE-----GAFYNVSPEDMKTYDYGSVLSNFSNPSADDWMNTVREYQEGAL-SSEAAIPYIYGQDSVHGVN-YASGCVIFPHNINMGAANDPELMKKYGSL
VGSDIVHTGMLMNFSPCVDAAQDPRWGRTYECYSDDNEMVKNLSVAYAEGLLSEGVVVCAKHFFGGGYTKYGTGENSDLIDRGDAQMSKEQLSVYDGLVKAGVQVIMVS
---HSSLEGTKMHENAKYI-SYLKDDLGFDGFVLSDWDSIENCSG-ADLKENVILCVNAGIDMLMEADNFEECRGYLVEAVEEEAISRERLDDAVTRIIKV--------
-------KGMKVYVSGPAADTGVLCGGWTYLWSP----SILDALKASAKEDFEIITDPKKIDECDLIVLCVGERPYANGDTKDLGELALE-GNKKAIKEAAKSGKPTLT
LIVAGRNVIVDDY-LKDWDSCIMCYLPGSEGGNAIADVLTGDASPEGKLPMPYYSSVKQI---------------------------
>K6YRG7_9ALTE
TLAQKIGQMTQAE-----RSTCTAQDVYQYHLGSVLSAAGRLKDWLEMTDAYWLASMQTDADHLAIPVIYGIDAVHGNN-NVKDAVVFPHNIGLGAGADFDLIEQIAEI
TAKEVCAIGVDWVFSPNLAVAEDYHWGRTYESFSERTDLVCDFAKSMITGLQSAGVLACAKHWIGD--GGTLHGVDQGDTILDWQQLEQIHVRPYYQAIEAGALSIMVS
---FSSWNGEKCHGNRHLLTDILKGNMQFSGILISDMQGIDDLAEDFY--IAVAKGVNAGIDMFMVPGNWKQFIEHLISHVELGTVPIERINDAVRRILSVLVLLKNHD
HVLPLSKNS-RILVTGNSADIGYQCGGFTISWEFPAATSIWQGIQNQATNA--GEITDIDPNQFDVAIVVVGERPYAEGLGDDDNSLELQPQALQTIKTLKVKGIPVVT
ILISGRPLITTSE-ITQSSAFIAAWLPGSE-GDGVADVLYAKAAFSGKLGFSWPDNSQSNID-LEK------QAFDTIYPVGFGLTY
>A0A4Q2A2W5_9FIRM
TLEEKAGQMIQAE-----RSGIQLSEISKYNIGSVLSGGGTKNTPEGWMQLSNRMQKVSRNSSSGIPLIYGIDAVHGHNNVLDA-VIYPHNIGLGAANNPALMHEIGKA
VAKDIKATGIQWNFAPAVSIVQDIRWGRTYESYSEVTGRVSVLGSEYIKGLQGEGVVATTKHFIGDGFTTFGTGEDRGDVTADYQVLLNMYLPAYEQAIASGTKTIMAS
---FNSVNGVKMHGHKTLLTDVLRTQLGFEGVVISDWEAIDGLE--GTLEDRVASAIDAGIDMLMQPFNWKEVYEAILSGVENGKISEDRIDEAVKRILV-VVLLKNE-
EVLPLDSGL-KIYLVGPASDVGIQCGGWTLSWQGEMTADLGTSIKEAFEAAKGGGRLVKDPEEADLVILVIGEKPYAEMNG-DTADLSLDEDNLAAVKEVKKYDLPVVT
IMVAGRPLLVKDH-IGGWDAFVMAWLPGTE-GAGITDVLFGQSPFKGTLPVTWPIENEQASDLFSDY----DLE--HQYKYG-----
>A0A1V1PCC3_9DELT
-LDQKVGQVLQVE-----REFITPEEVKTYHIGSLLSGGGNMEDWIQMNDELWAASMEENERYLAIPVIYGVDAIHGHGNAKG-AVIFPHNIGLGAANDPDLIERIAMT
TARETAATGVDWTFAPTLAVVRNDHWGRTYESYAEVPEIVTSYAARFVKGLQGNNVIACAKHFIAD--GATLHGVNTGDAPINEAELRKIHLPPYLAALKENVLTVMIS
---FSSWNYIKCHANKYLITHLLKEELGFDGIVITDWDGIDYLSDDY--FEAVAIGINAGMDMFMVTERWKLCYHHLKTHIQTGRVAMSRLDDAVRRILRVLVLLKNNK
DILPLNKDA-RIIVAGKSAHRGIQCGGFTIEWSI---VSIWEGISKAAPNSENLTGEDAHLNKHDVGIVVIGEKPYANGEESYGTTLNLCPEDLKTIQNISSKGIPVIV
ILISGRPLVVNKE-LDESEAFVAAWLPGSE-GGGVADVLFGDYDFQGKLSFTWPTYDDDNLN--IGD-D----NYHPLFAYGYGLSY
>A9NG51_ACHLI
TIAEKAGQMVQGERSNNGASGVKPTDVRNLNLGSVLNGGGSSNTTFGWVSMYENMLNASLESSSKIPIIYGVDAVHGHNNLYGA-TIFPHNIGLAAANNKELMKEIGMI
TAYEMGQTGMNMNFSPSIGLIKDKRWGRTYETLGESPDIALNLIPSYIEGIQSYGVIGSAKHFVGDGYTTLDNKLDRGNSTISKEDLETIHFPLYEAAIEAGVKSIMVS
---YSSLNDVRMHENKELITDILKDQMGFKGFVIGDYNGIDDIRA-NTFYERVIKGVNAGIDMLMQPHNFKEVIDAIVRGVEEDRIDIDRINDAVSRILSVMVLLKNNQ
NLLPFNKDL-NLLILGKGSQIGIQSGGWTIDWQ----LTIVDAFKSVT--NGQIYTDINDIDKADQIIIVFSEKPSA--EMMDSLALSLTASNQTLIDIAKQTNKPVIG
LLLSGKPLIIEEV-IPYLDAFVMLFLPGSE-GLGITDVLYGDYNFKGKLPFTWPKSISQSSHENYE-------PSDYRYPFGYGLNY
>A0A4R5GYI3_9ALTE
TLAEKVGQMTQAE-----RNNVTPEDIKKYFLGSVLNGGGSVNRPEDWRAMIDAYQAAALDTRLGIPFIYGTDAVHGHN-NVKDATIFPHNVGLGAMRNPDLMEKIGKA
TAAEVAATGVHWNFAPALCVSRDKRWGRAYECYGEKPEIGVSYSGRYVKGMQESLVLATAKHWVGDGGTTYGTGIDRGDTRVSEQELRDIHIAPYLNAFKQDVGSVMYS
---YSSVNGLKMHENARINNDILKGELGFDGFVISDWQAIEEIAAET-NRERIVKAINAGLDMAMEPEFWREYITDITAAVNDGEIPMARIDDAVTRIL---VVLKNE-
GILPLNKG-SKILVAGSHADIGLQSGGWTIEWDITQGQTILDGIKQHA-SDVTFSKDGSGAAGHDVAVVVVGEKPYAEGAGDDVQPLTLSDEQLATIAKVREAGVPVLV
VLVSGRPLLISDE-LPEWDGLVAAWLPGSE-ADGVADVLFGDFKPKGKLPVSWPSTLEDVNK--NTGD----AGYQPLFDYGYGLT-
>G0GCI8_SPITZ
TIEEKIGQMAMVD----RGYLKSPHDIAEYGLGAILSGGGRRNTPESWKEMVDGFQREALGTRLGIPILYGIDAVHGHNNVHGA-VIFPHNIGLGATGDPELVERIGRA
VAEEVVATGIHWTFAPCVTVPQDERWGRTYEGFGEDPELVARLGAALIRGFQGVTILATAKHFVAD--GGTTGGKDRGDARLTEEELRKVHLRPYVEAVKAGVGSVMVS
---FSSINGVKMHANRDLIQGVLRGELGFDGLIVSDWA--AHTELPGSLEEKLATVINAGVDMIMIPDDYRGFVAAVKSLVEEGVVSRKRIDEAVYRIL--VVLLKNDG
GVLPLKKEGTRILVLGDKADLGVQCGGWTITWRVTEGTTILEAIRKAVSDVTHVRRASQLAQKADVIIVVVGETPYAEMYG-DRQDLSLTREDAELIIHASQTGLPVVV
VLVSGRPRIITDL-LDSMDALLAVWLPGTE-GDGIADVLFGDYAPTGKLPFVWPRSMEVLPL-TIEESH---HPEKALFPYGYGLSY
>G4QEA4_GLANF
TVDEKIGQMTQSV----WHNSVSPEIIQDRKIGSIIHTEGKVSDWV---AKFNTFQAHALKTRLGIPLLIGVDAIHGQN-TFEGAVIFPHNIGMGATRNYDLIRRAAEI
TAIETAGTGFNWTFSPVIAMPEHEHWGRVYEGFSEDANVTTKALIASIQGHQGTTIAATAKHYLGD--GATVGGREGGNAIISEKALRERFLPPYQAAVNHGISAIMVG
---FNSVNGTNMHQNTYLVQDVLKGQLGFDGVVITDWL---GGTRWGEP----HTVINAGIDIAMQPANHDEFMAKLKETVLDGTVSMERIDDAVRRILG-LVLLKSEA
NALPLKA-NESIAVVGEHANSGLQSGGWTMHWSYANSTTILDGIQAFAPEVQYQPMGCTSDTQAEKVVAVVGELPYAEFKG-DSTNLALTVAQQEMIKHCKALGKKVIV
VLISGRAMTVTDT-INQSDAFIAAWLPGSE-GMGIADFLFAANEPVGKLPTSWPKEYADLPL-AQ-------DAENALFPFGFGLS-
>A0A5B9WCA9_9BACT
TLDEKIGQMTQVDF----KAFKDLGDITRLSLGSVLCGGNDDITPAGWAKAQDDCQALALKARLKIPLIFGVDAVHGHNNVDGA-VIFPHNVGLGASRDPSVVEKAARV
TALEMVGTGIRWAFAPCVAVARNERWGRTYESFGERPELAAELGPAAVRGLQGDSVLACVKHFVGD--GGTTNGVDQGNTECDEAALRQIHLPGYVASIKQGAGSIMAS
---YSSWNGKKLHGHKYLVTDLLKGELGFGGFVVSDWAGVDQLSPDF--KAAIDQSINAGVDMVMIPNGPGQKIDKLKELVNEGKVPASRIDDAVRRIL--LVLLKNDR
KVLPLSKSIKKLVVAGPAANIGIQCGGWTIAWVIHGGTTVLAALKEALGPEVVHSADGSGAAGADAAVVVIGERPYAFGDRRGGKDLGLPADDLAALKKVREAGIPVAT
VVFSGRPVLLGPV-LESSDAILAAWLPGTE-GRGIADVLLGDYKPTAKLPHTWPRSMEQIPC---NPED--GAASEALFPFGFGLSY
>A0A1E7JN49_9ACTN
-LKEKIGQLFVVEVYGLYGVSTPAEVVAKYRPGGVIYFDANLQQPRQIAGLSNGLQRAALRTGARIPLLVSIDQEGGSVYRMLEPTQLPGNMALAAARSRGDVRRSSEI
IGTELAAMGINQNYAPVADVNINPDNPIGVRSFGSDPALCSDLVAASVRGYHRGEVSSAAKHFPGHGDTDVDSHTGLPVIKHTREELEKIDLPPFRAAIARGVDTIMTA
HIVVPSLDSSPATMSKPIVTGLLREELGFRGLIVTDALDMGGATEDFPPDVAPVRALKAGCDQLVLAPKADTAHAAVLKAVESGDVPEDRVDASVLRILE-VTLVRNEG
GTLPLSPGARTVLVTGWDTDLARTVGERSGQEAT----ALATGATPGA---TQIEDAVTAAGDHDVTVVLTNAA----ASAKDKG-----AAQADLVKALVKTGKP-VV
AVAVRNAYDIRRF--PGVPACLATFSYGKPSLDSVVRALYGDVNPSGKLPVSIPALDEDE---------------GTLYEFGHGLSY
>HvExoI
TLAEKIGQMTQIE-----RLVATPDVLRDNFIGSLLSGGGKGATAKEWQDMVDGFQKACMSTRLGIPMIYGIDAVHGQNNVYGA-TIFPHNVGLGATRDPYLVKRIGEA
TALEVRATGIQYAFAPCIAVCRDPRWGRCYESYSEDRRIVQSMT-ELIPGLQGDKVAACAKHFVGD--GGTVDGINENNTIINREGLMNIHMPAYKNAMDKGVSTVMIS
---YSSWNGVKMHANQDLVTGYLKDTLKFKGFVISDWEGIDRITTPAGYSYSVKASILAGLDMIMVPNKYQQFISILTGHVNGGVIPMSRIDDAVTRILRVLVLLKNGK
PLLPLPKKAPKILVAGSHADLGYQCGGWTIEWRTTVGTTILEAVKKAVDPAENPDAEFVKSGGFSYAIVAVGEHPYTETKG-DNLNLTIPEPGLSTVQAVCGG-VRCAT
VLISGRPVVVQPL-LAASDALVAAWLPGSE-GQGVTDALFGDFGFTGRLPRTWFKSVDQLPMNVGD------AHYDPLFRLGYGLT-
>D3PUC5_STANL
-LEEKVGQMFTHYAYGLHGVDNAKQLIEKYHLGGIIYFGWNLANPGQVAGLSNGMQKTAMSQGGEIPLLVSTDQETGTVVRLGPPTEWPGNMALGAGRDRGDARDTAAI
AGVELRAVGINQNFAPSGDVNVNPQNPIGVRSYSSDPKLVAKFTGEQVKGYQGRGTSATVKHFPGHGDTHEDSHTDLPRIEHTKEQWKKLDAPPFKAAIKSGVDTVMSA
HIQFPALDASPATLSKPILTGLLREELGFDGVIVTDSLGMQGVRELYTDAEIPVMAIKAGVDQLLMPADLKVAYEAVLAAVKSGEITEKRIDKSVKRLLTL-TLVKNDG
DALPLSKDTGKVFVAGDGANTTSILAKQIGKLGP-----ATEALNTGAPNAATISGAVEKAKQADAVAVVATNTV---------RAH----PAQADLVKALRDSGTK-VV
VVGVKEPYDINRF--PEVDSYVASYGYNTPVLTAVAKVLFGEVDPRGKLPVTIPKADD--PD-------------ATLYEFGHGLSY
>UniRef90_A9NUD1_27_625
TVEEKIGQMTQIE-----RSDATADVMKKYYIGSVLSGGGKASP-ATWINMVDDLQKGAMSTRLQIPMMYGIDAVHGHNNAYGA-TMFPHNIGLGATRDPDLARRIGAA
TALEVRATGIQYTFAPCVAVCRDPRWGRCYESYSEDPKIVKAMT-QIIFGLQGQNVAACAKHFVGD--GGTTNGIDENNTVIDYKGLVNIHMTPYFDAIAKGVSTIMVS
---YSSWNGMKMHANRFLVSEVLKKQLGFKGFVISDWQGIDRITSPPGYSLSVFDGVGAGIDMVMVPENFTNFITELTSQVKGGLISMTRINDAVRRILTVLVLLKNGK

PLLPLDKNAPKILVAGTHPNLGYQCGGWTIEWNSTIGTTILQAIKFAVSPQQNPDANYVKGQGFSYAIVVVGEAPYAEMNG-DNLNLTIPLGGGDTIKNVCSSLKC-LV
ILISGRPLVIEPY-LPLVDAFVAAWLPGTE-GQGVTDVIFGDYGFQGKLPRTWFKSVDQLPMNVGDK------HYDPSFPLGFGL--
>UniRef90_W1PPJ1_6_602
TIAEKVGQMTQIE-----RSVVTLDAMKNQFIGSILNAGGSGASAEEWADMVDGFQRWALESRLSIPVLYGTDAVHGHNNCYGA-TIFPHNIGLGATRDPNLVQKIGEA
TAIEVRATGIPYTFAPCVAVSRDPRWGRCYESYSEDTETVRKMT-TIVQGLQGSNVIACMKHFIGD--GGTKGGINEGNTVGSFDELHSVHLKPFLDCLDQGVCTAMAS
---YSSWNSMMLHSHHFLLTQVLKHQLGFKGFVISDWEGIDRLCQPQGYRFCISASINAGIDMVMVPHDFQKFIGDLTFLVESGEISMTRIDDAVERILRVLVLLKNGK
PFLPLSKNGGRILVGGEHAHLGYQCGGWTITWQITQGTTILEAIKAAVGMEENPTETSFKTQEFSSAIVVVGEKPYAEFLG-DDPKLELSPKAIETIELVCSK-VPTLV
ILLSGRPLIVEPL-IEKMEAFVAAWLPGSE-GAGVADVIFGGYEFHGCLPRTWFKRVDQLPMNVGD------SNYDPLFPFGFGLK-
>UniRef90_W1NE16_9_604
TLAEKIGQMTQIE-----RQVANYSVMKEYAIGSILSGGGQASAAVWVNMVNEFQRGALA-SRLQIPMIYGIDAVHGHNNVYGS-TIFPHNVGLGVTRDPDLLKRIGAA
TALEVRATGIPYTFAPCIAVCRDPRWGRCYESYSEDPEIVEAMT-EIIPGLQGDKVAACAKHFVGD--GGTHNGINENNTIIDRHGLLAIHMAGYYHAIIKGVSTVMVS
---YSSWNGEKMHANRDLVTNFLKKTLHFRGFVISDWQGIDRITSPAGYPYSVHAGVNAGIDMIMVPYNFTEFINDLTEQVNSKSIPMSRIDDAVRRILRVLVLLKNGK
PLLPLDKKAPKILVAGTHADLGYQCGGWTIQWNTTKGTTILTAIKSTVSPEENPSASSLKGQDYDYAVVVVGETPYAETNG-DSMNLTMPEPGPTIIKNVCGSVKC-VV
VVISGRPIVLQPF-LQYIDALVAAWLPGTE-GQGVADNLFGDYPFTGKLARTWFKSVDQLPMNVGDK------HYDPLFPFGFGLT-
>UniRef90_W1NE06_27_621
TLAEKIGQMTQID-----RSVATQDVMKNYSIGSVLSGGGN-ATVEDWVNMVNDFQKGALSSRLQIPMIYGIDAVHGHNNVYGA-TIFPHNIGLGATRDPELAKRIGVA
TALEVRATGIPYVFAPCLAVCRDPRWGRCYESFSENPEIVEAMT-EVIVGLQGGSVAATAKHFVGD--GGTVRGIDENNTVIDYHDLLSIHLRPYYRAIIKGVSSVMVS
---YSSWNGVKMHSNRKLVTGLLKSTLRFRGFVISDWQGIDRITSPPGYMFSVHAGVNAGIDMIMVPYNYTDFINDLTDQVNSKSIPMSRIDDAVRRILRVLVLLKNGK
PLLPVDKKAPKILVAGTHADLGYQCGGWTITWNTTIGTTILSAIRSTVDPQENPDPSSLKANDYSYGIVVVGELPYAEFDG-DSTTLTMIEPGPTIIKNVCASMKC-VV
VVVSGRPIVLEPY-VPYMDALVAAWLPGTE-GQGLADVLFGFYPFSGKLPRTWFKSVNQLPMNVGDK------HYDPLFPFGFSL--
>B9TBT5_RICCO
--PRLGIPHLWETDAGLGVASQVRQATALPSGLNTAATWDVDTAYAGGAM
IGAEARARGFNVMLAGGVNLMRDPRNGRNFEYGGEDPLLAGRMVGAQIRGIQSNHVVSTLKHFALN---DQEIGRTTLNVLISEQAAVTSDLLALQIANEGNPGAVMCA
---YNRVNGVYSCENSWLLNDVLKGDWGFTGWVMSDWGAVH----STVPAANAGLDQQSGMPFDLADYFG----APLKEAVTNGWVPQARLDDMARRVLR-MVLLKNAA
QALPLQRTAKRIAVIGGHADKGVLAGGGSSLVVMYYPSAPLEAIRRRAPGGADRAAAAALARDSDAVVVFATQW---TGEGVDAPDLALPGGQDDLIAAVAAANPKTVV
VLETGGPVTM-PW-LPNVAAVLEAWYPGTSGGDAIAGILFGEVNP---
>UniRef90_A0A176WE76_43_636
TVQEKIGQMTQIE-----RTVATPEVMTQYFIGSVLSGGGEPNAPAAWQDMIDTMQQAALATRLAIPMIYGIDAVHGHNNLYGA-TVFPHNIGLGCSRDPDLVKRIGAA
TALEVRATGIPYAFAPCIATCRDPRWGRCYESYSEDTAVVKTMT-DIILGLQGDKVIGCAKHYVGD--GGTFKGINENDTIVDYDTLYKVHMAPYLDAIAKGVSTIMVS
---YSSWNGERMHANQYLVTQVLKEQLAFRGFIISDWMGVDRLSDPNNYTNSVLKSINAGLDMIMVPFDYEAYISGMLSLVNDGEISMERIDDAVTRILRVLVLLKNGQ
ALLPLKKNATSILVAGSHADIGLQCGGWTISWNTTIGTTVLDAIKAAVSPEKNPAPGFAAQLKPDYAIVVVGEEPYVETYG-DNMELTIPLDGIPTIQNVCAEVKC-LV
IVISGRPLVIEPY-MPQIDALVAAWLPGSE-GQGISDVIFGDYDFVGKLSRTWFRTVDQLPMNFGDA------VYDPLFPFDFGLT-
>UniRef90_A0A2N2MB45_49_637
-LDEKIGQMTQVE-----KNSILPGEVSRYYIGSILSGGGGDDSLEGWVKMVDGLQAAALETPLAIPLIYGVDAVHGHNNVKG-ATIFPHNIGLGATNDPELVEKIGRA
TAEEMLATGISWDFAPVLAVVQDIRWGRTYESYGENTELVTRLGVAYQNGLQAAFVLATPKHYIGDGGTTWASKLDQGDTQMDEARLRELFLPPYQAAVEAGAQSVMVS
---YSSWNGVKMHGHKYLITDVLKGELGFEGFVVSDWAGIDQVDSD--YYTAVVTAINAGVDMNMVPQGYPRYLTVMQQAVEKGDIPMERIDDAVLRILTVLVLLKNDN
ATLPLAKDAGLIFVAGASANIGAQCGGWTIEWNITTGTTILEAIEASASAEVRFDRFGESEQKADVAVVVIGERPYAEGRG-DKENPSLSKSDIELIQRVREQSQRVVV
ILLSGRPLVITEA-LPYADAFVAAWLPGTE-GSGVADVLFGDKPFTGKTPFSWPRSADQLPFDFANLP--ADGCAAPLFPYGYGLD-
>UniRef90_A0A2R6W019_43_631
TLDEKIGQMTQIER-----YVANFDVMKNFSIGSVLSSGGNSSTTEAWQNMVDDLQAGALATRLGIPMLYGIDAIHGHNNVYGA-TIFPHNIGLGCTRDPDLVRRVGAA
TALELRATGIPYTFAPCIAVCRDPRWGRCYESFSEDTSVV-RMMTDVIYGLQGNKVAACAKHYVGD--GGTQRGINSNDTILSYEDLFRIHVAPYVDAIAKGVSTIMLS
---YSSWNGVKMHMNHRLISTLLKQELGFKGFVISDMEGIDFITDIPDYTASVLESINAGLDMIMVPFDYEKFISTLRTLVNTGYISMQRIDDAVTRILRVLVLLKNGK
PFLPLSKNATRVLVAGTHADVGLQCGGWTISWSITKGTTVLDAVKAAVFTEASPTAEVAAKTKADFAIVVVGEQPYAEGAG-DNTNLTIPDEGISTIKNVCSEVKC-LV
ILISGRPLVVEPY-LPLMEAFVAAWLPGTE-GNGVTDVIFGDYDFVGSLSRTWFKSADQLPMNFGDPI--Y----DPLFRFAFGL--
>B9TDB0_RICCO
---------------------------------------NRNALETATYVNAVQKWAVEQTRLGISLFMHEEALHGYV--ARDATSFPQAIGIASSFDPDLTTKIFSV
AAREMRARGANLALAPVVDVAREPRWGRIEETYGEDPYLCGEIGKAAIIGFAGTKVLVTLKHMTGH--GQPESGTNIGPAEVSERTLREEFFPPFEKAIEANVGCVMPS
---YNEIGGVPSHANHWLLHKVLREEWGFKGITVSDYFGINELITRHKPKEAALRAIKAGVDVETPDGLAY---KTLGELVKEKRVSEAEIDTVVRRILTL-VLLKNDK
GLLPLGKKVGKVLLIGTHAK-DTPIGGYSDHPRH--VVSIHDGLQAEAKAARLIADAVAAAKSADTIIMVLGDNEQTSREAWDRESLDLMGQQNDLARAIFALGKPTVV
FLLNGRPLSINLL-AEKADAIIEGWYMGQETGNAAADLLFGRANPGGKLP-----------------------------------
>AUC61062.1
-LKEKIGQLIVVRTTGEANQQQLQTWLSEYNIGGVIL--------LGGSCAEIAQRTKQLNQWAKTPLLVAADIEEGVGQRFTGASWFPPPMALAQIAQDDYAEEMGKI
TAKEALCIGVNWIFAPVVDVNNNPDNPINVRAFGDNPEVVKELSSAFIRGTQSYPILNGAKHFPGHGDTSTDSHLDLPVINHSQARLEKIELVPFQGAIALNVDAIMTA
HLLVSAYDNQPATLSHRILTEELRHNMGFEGLIVTDALIMGGVAKYAPPEKIAVKALQAGADILLMPENPVVAIHSIIEAVEKGEISEHRIDESLQRVSK---------
---F-------------------------

>AFZ46311.1
-LQEKIGQLIVVRTTGEANNQQLQTWLSEYNIGGVIL--------LGGSCAEIAQRTKQLNEWAKTPLLVAADIEEGVGQRFTGASWFPPPMALAQIAKKDCAEEMGKI
TAQEALSIGVNWILAPVVDVNNNPDNPINVRAFGDNPGVVRELSSAFIRGTQYYPILNGAKHFPGHGDTSTDSHLDLPIINHSRTRLEAIELIPFQGAIALGIDAIMTA
HLLVSAYDNQPATLSHAILTKQLREKMGFDGLIVTDALIMGGVAKYAPPEKIAVKALQAGADILLMPENPLVAINSIMEAVQRGEITESRIDESLQRIAK---------
---AE---

>UniRef90_E0Z601_2_481
--------------------------VKKYYIGSVLSGGGKASP-ATWINMVDDLQKGAMSTRLQIPMMYGIDAVHGHNNAYGA-TMFPHNIGLGATRDPDLARRIGAA
TALEVRATGIQYTFAPCVAVCRDPRWGRCYESYSEDPKIVKAMT-QIIFGLQGQNVAACAKHFVGD--GGTTNGIDENNTVIDYKGLVNIHMTPYFDAIAKGVSTIMVS
---YSSWNGMKMHANRFLVSEVLKKQLGFKGFVISDWQGIDRITSPPGYSLSVFDGVGAGIDMVMVPENFTNFITELTSQVKGGLISMTRINDAVRRILTVLVLLKNGK
PLLPLDKNAPKILVAGTHPNLGYQCGGWTIEWNSTIGTTILQAIKFAVSPQQNPDANYVKGQGFSYAIVVVGEAPYAEMNG-DNLNLTIPLGGGDTIKNVCSSLKCLVI
LI---
>UniRef90_W1P4N1_9_377
TLAEKIGQMTQIE-----RLVANYSVMKEYAIGSILSGGGQASAAVWVSMVNEFQRGALAS-RLQIPMIYGIDAVHGHNNVYGS-TIFPHNVGLGVTRDPDLLKRIGAA
TALEVRATGIPYTFAPCIAVCRDPRWGRCYESYSEDPEIVKAMT-EIIPGLQGDKVAACAKHFVGD--GGTHNGINENNTIIDRHGLLSIHMGGYYHAIIKGVSTIMVS
---YSSWNGEKMHANPDLVTNFLKKTLHFRGFVISDWQGIDRITSPAGYPYSVYAGVNAGIDMIMVPNNFTEFINDLTEQVNNKSIPMSRIDDAVRRILRV--------
---E---

>UniRef90_UPI0009BF64A3_53_245
TLAEKIGQMTQID-----RSTANPELLMRLNIGSVLSGGGPKASPAMWADMIDGLQNAALATRLGIPIIYGIDAVHGHNNVYGA-TIFPHNIGLGATSDRDLVRRIGKA
TALEVRATGIPYTFAPCLAVCRDPRWGRCYESYSEDTEVVRSMAD-IILGLQG---

----------------------VLSGGGSIPASPAMWADMIDGLQNAALAR---

>Hv_Exo_II
TLAEKIGQMTQIE-----RENATAEAMSKYFIGSVLSGGGSVPSPAAWQSMVNEMQKGALSTRLGIPMIYGIDAVHGHNNVYKA-TIFPHNVGLGATRDPMLVKRIGEA
TALEVRATGIPYAFAPCIAVCRDPRWGRCYESYSEDPKVVQSMT-TLISGLQGDKVAACAKHYVGD--GGTFMGINENDTIIDAHGLMTIHMPAYYNSIIRGVSTVMTS
---YSSWNGKKMHANHFLVTDFLKNKLKFRGFVISDWQGIDRITSPPGYSYSVEAGVGAGIDMIMVPFAYTEFIDDLTYQVKNNIIPMSRINDAVYRILRVLVLLKNGK

PLLPLPKKAGKILVAGSHADLGNQCGGWTITWDKTAGTTILSAIKSTVDPSENPDSAAVDSGKYDYAIVVVGEPPYAETFG-DNLNLTIPAPGPSVIQNVCKSVR-CVV
VLISGRPLVVEPY-ISAMDAFVAAWLPGSE-GQGVADVLFGDYGFSGKLARTWFKSADQLPMNVGDK------HYDPLFPFGFGLT-
>Zm_Exo_II
TLAEKVGQMTQIE-----RIVASPQALRDYYIGSLLSGGGSVATAAEWVAMVSDFQKACLSTRLGIPMIYGIDAVHGHNNVYGA-TIFPHNVGLGATRDPNLVKRIGAA
TALEVRATGIQYAFAPCIAVCRDPRWGRCYESYSEDHRIVQAMT-ELIPGLQGDKVAACAKHFVGD--GGTQNGINENNTIIDRQGLISIHMPAYLDALRKGFSTVMIS
---YSSWNGLKMHANHNLITGFLKDRLNFQGFTISDWEGIDRVTSPPGYSYSVQASILAGLDMIMVPNNYQNFITILTGHVNSGLIPMSRIDDAVTRILRVLVLLKNGK
PLLPLPKKAARILVAGSHADLGYQCGGWTIEWRTTVGTTVLDAVKAAVDPAESPDAEFVRSGGFSYAIVAVGEHPYTETKG-DSMNLTIPDPGPSTVQTVCAAVR-CVT
VLISGRPVVIQPF-LGAMDAVVAAWLPGTE-GQGVTDVLFGDYGFTGKLPRTWFRSVDQLPMNYGDA------HYDPLFPLGFGLT-
>Nt_Exo
TLEEKIGQMTQIE-----RKVATADVMKQNFIGSVLSGGGSVPAPKASAQVVDEIQKGSLSTRLGIPMIYGIDAVHGHNNVYGA-TIFPHNVGLGVTRDPDLVKRIGAA
TALEVRATGIPYAFAPCIAVCRNPRWGRCYESYSEDHRIVRSM-TEIIPGLQGDKVAACAKHFVGD--GGTLHGVDESNTVISSNSLFSIHMPAYYDSLRKGVATVMVS
---YSSWNGRKMHANRDLVTGFLKDKLKFRGFVISDWQGIDRITDHANYSYSVQAGIMAGIDMIMVPENYREFIDTLTSQVKANIIPMSRIDDAVKRILRVLVLLKNGK
PLLPLPKKAPKILVAGTHAD---NLGYQCGGWTIEWQGTILTAIKKTVDPQQNPDANFVKSNKFSYAIVVVGEVPYAEMFG-DSSNLTIAEPGPSTISNICGSVKC-VV
VVVSGRPVVLEPY-VSKMDALVAAWLPGTE-GQGVADALFGDYGFTGKLARTWFKRVDQLPMNFDDA------HVDPLFPFGFGI--
>Tm_bglu
TLAEKIGQMTQIE-----RKEATPDVISKYFIGSVLSGGGPKASPEAWVDLVNGMQKAALSTRLGIPMIYGIDAVHGHNNVYNA-TIFPHNVGLGVTRDPALIKRIGEA
TALECRATGIPYAFAPCIAVCRDPRWGRCYESYSEDHTIVQAMT-EIIPGLQGDKVAACAKHFVGD--GGTTKGIDENNTVIDSRGLFSIHMPAYHDSIKKGVATVMVS
---YSSWNGLRMHANRDLVTGYLKNKLKFRGFVISDWEGIDRITDPPGYSYSVEAGVGAGIDMIMVPEDFTKFLNELTSQVKKNIIPMSRIDDAVKRILRVLVLLKNGE
PFVPLPKNAKKILVAGSHAD---NLGRQCGGWTIEWQGTILNAIKKTVDPNENPDSNYVKTNSFDYAIVVVGEPPYAEMQG-DSFNLTIPEPGPTTISSVCGAVKC-VV
VVISGRPVVLQPY-VSYMDALVAAWLPGTE-GQGVTDVLFGDYGFTGKLARTWFKTVDQLPMNVGDK------HYDPLFPFGFGLT-
>At_Exo_I
TLPEKIGQMTQIE-----RRVASPSAFTDFFIGSVLNAGGPFEDSSDWADMIDGFQRSALASRLGIPIIYGTDAVHGNNNVYGA-TVFPHNIGLGATRDADLVRRIGAA
TALEVRASGVHWAFSPCVAVLRDPRWGRCYESYGEDPELVCEMT-SLVSGLQGVNVVACVKHFVGD--GGTDKGINEGNTIASYEELEKIHIPPYLKCLAQGVSTVMAS
---YSSWNGTRLHADRFLLTEILKEKLGFKGFLVSDWEGLDRLSEPQGYRYCIKTAVNAGIDMVMVPFKYEQFIQDMTDLVESGEIPMARINDAVERILRV-VLLKNAK
PFLPLDRNAKRILVTGTHADLGYQCGGWTKTWRITIGTTLLDAIKEAVGDKTPSKETLASSEGFSYAIVAVGEPPYAETMG-DNSELRIPFNGTDIVTAVAEI-IPTLV
ILISGRPVVLEPTVLEKTEALVAAWLPGTE-GQGVADVVFGDYDFKGKLPVSWFKHVEHLPLDAHANS------YDPLFPFGFGLN-
>At_Exo_II
TLPEKIGQMTQIE-----RVVTTPPVITDNFIGSVLNGGGEDAKTSDWADMIDGYQNAALASRLGIPIIYGIDAVHGNNNVYGA-TIFPHNIGLGATSLVMLIRRVGAA
TALEVRACGAHWAFAPCVAALRDPRWGRSYESYSEDPDIICELS-SLVSGLQGENVVACAKHFVGD--GGTDKGINEGNTIVSYEELEKIHLAPYLNCLAQGVSTVMAS
---YSSWNGSKLHSDYFLLTELLKQKLGFKGFVISDWEALERLSEPFGYRNCVKISVNAGVDMVMVPFKYEQFIKDLTDLVESGEVTMSRIDDAVERILRVLVLLKNGT
PFLPLDRNVKRILVTGTHADLGYQCGGWTKAWRITIGTTLLDAIKEAVGDKTPSEETLASLQRFSYAIVAVGETPYAETLG-DNSELTIPLNGNDIVTALAEK-IPTLV
VLFSGRPLVLEPLVLEKAEALVAAWLPGTE-GQGMTDVIFGDYDFEGKLPVSWFKRVDQLPLTADA------NSYDPLFPLGFGLNY
>At_Exo_III
TLPEKIGQMTQIE-----RSVASPQVITNSFIGSVQSGGAGPLEDAKDWADMIDGFQRSALASRLGIPIIYGTDAVHGNNNVYGA-TVFPHNIGLGATRDADLVKRIGAA
TALEIRASGVHWTFAPCVAVLGDPRWGRCYESYSEAAKIVCEMSL-LISGLQGENVIACAKHFVGD--GGTEKGLSEGNTITSYEDLEKIHVAPYLNCIAQGVSTVMAS
---FSSWNGSRLHSDYFLLTEVLKQKLGFKGFLVSDWDGLETISEPEGYRNCVKLGINAGIDMVMVPFKYEQFIQDMTDLVESGEIPMARVNDAVERILRVLVLLKNGK
PFLPLDRNAKRILVVGMHAN---DLGNQCGGWRITIGTTLLDSIKAAVGDKTPTKETLASSDGFSYAIVAVGEPPYAEMKG-DNSELTIPFNGNNIITAVAEK-IPTLV
ILFSGRPMVLEPTVLEKTEALVAAWFPGTE-GQGMSDVIFGDYDFKGKLPVSWFKRVDQLPLNAEAN------SYDPLFPLGFGL--
>Dd_bglu
-ITEKIGQMTQLDITTTINETTLAYYAKTYYIGSYLNSPVHINSSVWLDMINTIQTIVIEGSPNKIPMIYGLDSVHGANYVHK-ATLFPHNTGLAATFNIEHATTAAQI
TSKDTVAVGIPWVFAPVLGIGVQPLWSRIYETFGEDPYVASMMGAAAVRGFQGGSAVCTAKHYFGY--SNPTSGKDRTAAWIPERMLRRYFLPSFAEAIGAGAGTIMIN
---SGEVNGVPMHTSYKYLTEVLRGELQFEGVAVTDWQDIEKLVYFHHAEEAILQALDAGI-ICLCHDLL-SQLFSL-EILAAGTVPESRLDLSVRRILNL-TLLLFKN
NILPLTNTIKNVLLTGPSADIRNLNGGWSVHWEFPFGTSILTGLREITNDQTSIDEAVELAQSSDVVVVVIGELPEAETPG-DIYDLSMDPNEVLLLQQLVDTGKPVVL
ILVEARPRILPPDLVYSCAAVLMAYLPGSEGGKPIANILMGNVNPSGRLPLTYPGTTGDIGVPYYHK-YSENGVTTPLFQFGDGLSY
>Tb_bglu
-----------------------------YGIGQITRLGGNLSPRETVRIANQIQKFLIENTRLGIPALIHEESCSGYM--AKGATIFPQTIGVASTWNNEIVEKMASV
IREQMKAVGARQALAPLLDITRDPRWGRTEETFGEDPYLVMRMGVSYIRGLQTEGIVATGKHFVGY--GNSEGGMNWAPAHIPERELREVFLYPFEAAVEAKLSSIMPG
---YHELDGVPCHKSKKLLNDILRKDWGFEGIVVSDYFAISQLYEYHHKKGAAKLALEAGVDVELPSTDYY--GLPLRELIESGEIDIDFVNEAVKRVLK-IVLLKNEN
NLLPLKKDLKSIAVIGPNADIRNMIGDYAYPCI----YTVLQGIKAKVSSKDGFKEAVEIAKQADVAVVVVGDKSGLSGESRDRADLNLPGVQEELIKAIYETGTPVIV
VLINGRPMSISWI-AEKIPAIIEAWLPGEEGGRAVADVIFGDYNPGGKLPISIPQSVGQLPV-YYYHKRSYVLSTKPLYPFGYGLSY
>Te_xyl-ara
TIEEKVAQLNSIWVYEMKFSFDKAKRLMSYGISQITRLGGNLSPRETVRIANQIQKFLIENTRLGIPALIHEESCSGYM--PKGATIFPQTIGVASTWNNEIVEKMASV
IREQMKAVGARQALAPLLDITRDPRWGRTEETFGEDPYLVMRMGVSYIRGLQTEGIVATGKHFVGY--GNSEGGMNWAPAHIPERELREVFLYPFEAAVEPKLSSIMPG
---YHELDGVPCHKSKKLLNDILRKDWGFEGIVVSDYFAISQLYEYHHKKGAAKLALEAGVDVELPSTDYY--GLPLRELIESGEIDIDFVNEAVKRVLK-IVLLKNEN
NLLPLKKDLKSIAVIGPNADIRNMIGDYAYPCI----YTVLQGIKAKASSKDGFKEAVEIAKQADVAVVVVGDKSGLSGESRDRADLNLPGVQEELIKAVYETGTPVIV
VLINGRPMSISWI-AEKIPAIIEAWLPGEEGGRAVADVIFGDYNPGGKLPISIPQSVGQLPV-YYYHKRSYVLSTKPLYPFGYGLSY
>Tn_bglu
---TTAFPVEIMLASTWNRELLEEVGKA
MGEEVREYGVDVLLGPAMNIHRNPLCGRNFEYYSEDPVLSGEMASSFVKGVQSQGVGACIKHFVAN---NQETNRMVVDTIVIERALREIYLRGFEIAVKSKPWSVMSA
---YNKLNGKYCSQNEWLLKKVLREEWGFEGFVMSDWY----------AGDNPVEQLKAGNDLIMPGKAYQVNIEEIMEALKEGKLSEEVLDECVRNILKVVVLLKNE-
EALPLSEN-SKIALFGTGQIETIKGGTGSGDTHPRYAISILEGIKERGLNFLSEKEVHKLAKKNDVAVIVISRI---SGEGYDRGDFYLSDDETDLIKTVSEQGKKVIV
LLNIGSPVEVVSW-RDLVDGILLVWQAGQETGRIVADVLTGRINPSGKLPTTFPRDYSDVPSP--GEPDNYDFGVEPAYEFGYGLSY
>Hv_bxyl
---LRAATSFPQVILTAASFNPHLWYRIGQV
IGTEARAEGL-TFWAPNINVFRDPRWGRGQETPGEDPTMTGKYAAVFVRGVQGYEASACCKHFTAY-DLENWKGVTRFAFKVTEQDLADTYNPPFKSCVDGGASGIMCS
---YNRVNGVPTCADHNLLSKTARGDWSFNGYITSDCDAVAIIHDVQGPEDAVADVLKAGMDVNCGGYIQT----HGVSAYQQGKITGEDIDRALRN----IVLLKNDG
AALPLKSKVSSLAVIGPNGNASLLLGNYFGPPCI--SVTPLQALQGYVKDVSNIGEAVHAAGSADYVVLFMGLDQNQEREEVDRLELGLPGMQESLVNSVAAAKKPVIL
VLLCGGPVDVTFANNPKIGAIVWAGYPGQAGGIAIAQVLFGDHNPGGRLPVTWYPKEFTAPMDMRMRADPYRYKGKTVYNFGYGLSY
>Tb_gln-glc
--VAWSTDVTDGAV-VTANGTVFPQAVGLASTWDPELNQRVGTV
HAQNPVVWGLNL-WAPVVNLLRDPRWGRNEEGYSEDPLLTGAIAIAYGSGIQGDRAAPTLKHYLAN---NNEIRRDTTSSNLPPRVKHEYYEAPFRAAITAGATGVMTA
---YNLVNGRPATVNPDL-NDTVRTWTDRDLLNVTDAGAPNNLVGSQALAEADAAALKAGIDSFTTDETNSAPITAIKTALSQGLLTEQDIDTAVRHIL--MVLLKNER
GTLPLDPG-KKVAVVGPLAD-VLYTDWYSGR--PTYQVTPLDGIRERAASRSGIDDAVAKAKEADVAVVVVGSMPFIGREDHDRTDMNLAEGQEALVKAVFNANPRTVV
VLENSYPTTINWI-DEHVPAILWTTHAGAETGNALADVLYGDVNPAGRLTQTWYLG-GRLPDDIVQRDRTYLFKGTPLYPFGHGLSY
>Nc_bglu
---FNSSTSFPMPLLMAATFDDELIEKVGEV
IGTEGRAFGNAGYWTPNVNPFKDPRWGRGSETPGEDILRIKRYAASMIRGLQGPRVVATCKHYAAN-DFEDWNGSTRHDAKVTLQDLAEYYLSPFQQCADSKVGSIMCS
---YNAVNGVPACANTYLMQTILREHWNWTGYITSDCEAVLDIFANHHNAEGTALAFEAGTDSSCEYESS----SDIPGAWTQGLLEQSTVDRALTRLY--IVLLKND-
QTLPLTDPKSKLAMIGFWANPKTLSGGYSGKPG------------F---NDTWTQAALEAAQDANYILYFGGLDTSAAGETKDRTTINWPEAQLQLIKTLTKLGKP-LV
VVQMGDQLDNTPLATKTVNSILWANWPGQDGGTAVMQILTGLKSPAGRLPVTQYPANYTAAVPMTDMNDRYRWYPTAVQPFGFGLHY
>Rf_GH3
---WSEGLHGVA-RAGTATMFPQTIGMAAMFDDEAVHRAGET
TSREARARDDIYTLWSPNVNIFRDPRWGRGQETYGEDPYLTSCLGVAYAKGLQGDRTAACAKHFAVH--SGPEATRHEFDAKANMKDMTETYIAAFEALVDAKVESVMGA
---YNRVNGEPACASDFVMNKL--EEWGFDGHFVSDCWAIRDFHTNHGAPESAALALKKGCDLNCGNTYL-----HLLAAFNEGLINEEDLRRSCIKLM--MVLLKNN-

GILPLGSKYKTIGVIGPNADVPALEGNYNGKADE--YITFLSGIREAHDGDDRLSEAEIITRTLRCSGSLCWLDATIEGEEGDKNDLRLPESQRKLVKTVMAKGKPVII
VTAAGSAINV----EADCDALIQAWYPGQLGGRALANILFGKVSPSGKLPVTFYEDASKLPDDY-SMKRTYRSEGNILFPFGYGLTY
>HJ_bxyl
--QFEWATSFPMPILTTAALNRTLIHQIADI
ISTQARAFGLDV-YAPNVNGFRSPLWGRGQETPGEDAFLSSAYTYEYITGIQGGKVAATVKHFAGYDLENWNNQSRLGFDAITQQDLSEYYTPQFLAAAYAKSRSLMCA
---YNSVNGVPSCANSFFLQTLLRESWGFPGYVSSDCDAVYNVFNPHDQSSAAASSLRAGTDIDCGQTYPW----HLNESFVAGEVSRGEIERSVTRL---IVLLKND-
GTLPLSKKVRSIALIGPWANTTQMQGNYYGP--APYLISPLEAAKKAGYHTTGFAKAIAAAKKSDAIIYLGGIDNTIEQEGADRTDIAWPGNQLDLIKQLSEVGKPLVV
LQMGGGQVDSSSLSNKKVNSLVWGGYPGQSGGVALFDILSGKRAPAGRLVTTQYPAEYQFPQ----NDRPYIYTGKPVYEFGSGLFY
>Ci_bglu
--------------------------------------AGKVNLTTGTGFLMALVGQTGSALRFGIPRLCLQDGPLGLRNTDHN-TAFPAGISVGATFDKKLMYERGCA
MGEEFRGKGANVHLGPSVGLGRKPRGGRNWEGFGSDPSLQAIAAVETIKGVQSKGVIATIKHLVGNEQEMMTNIVQRASANIDDRTMHELYLWPFAESVRAGVGAVMMA
---YNDVNGSASCQNSKLINGILKDELGFQGFVMTDWY-----AQIGG----VSSA-LAGLDMSMPGDGSVPLASELSRSILNGTVALDRLNDMVTRIV--ITLLKNED
NLLPLPNR--AIKYSEQMPGGVLTMGWGSGTSNLPYLVTPEDAIRNISKNTDKFPNNV-QPGPDDVAIVFVNAD---SGENYDRAQMKLWHNGDELIESAAKKFSNVVV
VVHTVGPIIMEKW-IDLLRSRVSC-LPDFQDKKLEILLLISCSETSVRVAASIYDTESRISDSLINQ--RYRFQKENIYHFGYGLSY
>Sc_bglu
--RFGIPNLCLQDGPLGVR--LTDFTGYPSGMATGATFNKDLFLQRGQA
LGHEFNSKGVHIALGPAVGLGVKARGGRNFEAFGSDPYLQGIAAAATIKGLQENNVMACVKHFIGNEQSKVDPEYDPATANIPDRAMHELYLWPFADSIRAGVGSVMCS
---YNRVNNTYSCENSYMINHLLKEELGFQGFVVSDWAQMSGAYSAI-----------SGLDMSMPGELLGGWGQNLTKAVYNETVPIERLDDMATRIL--IVLLKNEK
NTLPIPNKVRKLLLSGIAAGGALFEGWGSGSVYPKYQVTPFEEISANARKSFDLTQVSTVASDAHMSIVVVSAV---SGEGYDKNNVTLWHNSDNLIKAVAENCANTVV
VITSTGQVDVESFDHPNVTAIVWAGPLGDRSGTAIANILFGNANPSGHLPFTVAKSNDDYPITY-NPPEPYRFEEKNIYAFGYGLSY
>Gg_ave
--VTVFPGGVSAASSWDKDLIYKHGVL
MAEEFRDKGSHVILGPVIGRGRSPYAGRNWEGFSPTSYLAGVMAEQTVKGMQSVGVQACTKHFIGNEQTAVDGKTEAISSNIDDRTMHEAYLWPFYNAVRAGTTSIMCS
---YQRINGSYGCQNSKTLNGLLKTELGFQGFVVSDWA----------ATHSGVASIEAGLDMNMPGPLNFFAGKNITTAVNNGTLSSRRVDEMIERIMT--VLLKNEK
GALPL-KKPMNIGVFGNDAAGTLPLGGGSGTGRYTYVFPPLEDIKARGRSVITSGGLVTIFPVPEVCLVFLKSW---ATEGEDRISLEAQWNAAVVVEKTAVLCNNTIV
VIHGGAPVVMPWRNNPNVTAILAAHMPGQENGHSLVDLVWGDVNPSGKLPYTLADQATDYNKNLVNSTQSFDHNKTPAYEFGFGLSY
>Wa_bglu
---DLDVFPCGMAASSSFNKQLIYDRAVA
IGSEFKGKGADAILGPVYGPMGVKAAGRGWEGHGPDPYLEGVIAYLQTIGIQSQGVVSTAKHLIGNEQAKKDKHAGKNSSEIDDRAMHEIYLWPFAEAVRGGVSSIMCS
---YNKLNGSHACQNSYLLNYLLKEELGFQGFVMTDWGALYSGI----------DAANAGLDMDMPCEAQY-FGGNLTTAVLNGTLPQDRLDDMATRILS-VVLLKNEH
ETLPLREKVKRISILGQAAGGAIGTGYGSGAGTFSYFVTPADGIGARAQQSWNQAAAMDSALYADAAIEVANSV---AGEEIDLNNLTLWHNAVPLIKNISSINNNTIV
IVTSGQQIDLEPFDNENVTAVIYSSYLGQDFGTVLAKVLFGDENPSGKLPFTIAKDVNDYPVKVDVPDDKYFKYNKPVYEFGYGLSY
>HJ_GH3
--ASKISYPSLCLQDGPLGVRYSTGS-TAFTPGVQAASTWDVNLIRERGQF
IGEEVKASGIHVILGPVAGLGKTPQGGRNWEGFGVDPYLTGIAMGQTINGIQSVGVQATAKHYILN---EQELNRETISSNPDDRTLHELYTWPFADAVQANVASVMCS
---YNKVNTTWACEDQYTLQTVLKDQLGFPGYVMTDWN----------AQHTTVQSANSGLDMSMPGTDFNGNGPALTNAVNSNQVPTSRVDDMVTRILA-IVLLKNDA
NILPL-KKPASIAVVGSAAIGALGMGWGSGAVNYPYFVAPYDAINTRASSTDNTSSGASAARGKDVAIVFITAD---SGEGYDRNNLDPWHNGNALVQAVAGANSNVIV
VVHSVGAIILEQIALPQVKAVVWAGLPSQESGNALVDVLWGDVSPSGKLVYTIAKSPNDYNTSG---GDSYKFDDANIYEFGYGLSY
>Cg_bglu
--RLGLPALFETDAGQGVASQVRERTALPSGLSTASTWDPKVAYAGGAM
IGSEARASGFNVMLAGGVNLQREPRNGRNFEYAGEDPLLAGTMIGQAIKGVESNRIISTLKHFVLN---DQETGRNELDARIDKAALRMSDLLAMELALQSDAGSVMCA
---YNRLNGPYTCEHPWLLSEVLKRDWGFRGYVMSDWG----------ATHSTVAAANSGLDQQSGQEFDKSPGGALEEAVKTGAVPQKRLDDMVTRIVR-MVLLKNEG
RLLPLAKTVRTIAVIGGHADAGVLSGGGSSQVVVYYPSSPLRAIQAQAPNGRDPARAARVAAGADVALVFANQW---IGEANDAQTLALPDGQEELITSVAGANGRTVV
VLQTGGPVTM-PW-LARVPAVLEAWYPGTSGGEAIANVLFGAVNPSGHLPATFPQSEQQLPRPKLDGDKNFDKGHKPLFPFGHGLSY
>Se_GH3
--RLGIPQIRGTDGPAGVT-IHQPAIAMPAPVALASAFDDRLAHEYGTV
LGREGRAFEQDIILGPMVNNIRVPQAGRNFETFSEDPLVTARTAAAQIRGIHSQGLMTSAKHYAAN---TQETDRFTIDVDVDQRTLRELELPGFEAAVAAGATSVMCA
---YPKVNGTHACGHRQLLTEILKEQWGFKGWVMSDWTATHATEDLVAG-LDQEMGVEVREDGSLFRGKYL--GEALKKAIREGRIPESALDASVRRILT--VLLRNEG
GVLPLPAAGQDIAVIGPSAQQPKVTGLGSSYVEPDFANAPLDTITQRVGSQREFDAAVERARDSDVAVVFAYDD---GAETADRTSLSLPGTQDKLIDAVASVNPNTVV
VLNTGSSVTM-PW-LDKTRAVLDMWYPGQAGAEATTALLFGDAEPGGRLTQTFPVSQERTPVR---F--PYDEGVDPLFPFGHGLSY
>Ar_bglu
---SLVGGVACFPVAIALGATWDPELIERAGVA
LGGQAKSKGASVLLAPTVNIHRSGLNGRNFECYSEDPALTAACAVAYINGVQSQGVAATIKHFVAN---ESEIERQTMSSDVDERTLREIYLPPFEEAVKAGVKAVMSS
---YNKLNGTYTSENPWLLTKVLREEWGFDGVVMSDWF----------GSHSTAETINAGLDLEMPGPWR-DRGEKLVAAVREGKVKAETVRASARRIL---VLLKND-
GVLPLKSSFDQIAVIGPNAASARVMGGGSARIAAHYTVSPLEGIRAALSNDAGIAEAVETARKSDIVLLLVGREGEWDTEGLDLPDMRLPGRQEELIEAVAETNPNVVV
VLQTGGPIEM-PW-LGKVRAVLQMWYPGQELGNALADVLFGDVEPAGRLPQTFPKALTDNS--AITDDSIYPQDGHVRYAEGIFVGY
>Pb_GH3
--PAGVHIDARKGTNQTFFA-TGFPIGTCLASTWNLDLVEKVGKA
IGNETLEYGCDAILGPGMNLHRNPLCGRNFEYYSEDPIVTGLIGAAMVKGIQSQGVGVSAKHFAVN---SQESDRTRVDERVSQRALRELYLKGFEIMVKSAPWTLMSS
---YNRINGVYSQGNYDLLTKVLRQDWGFKGIVMTDWI--GERAD-----LPISDVVKAGNDLLMPGFPTQ--VNHIIEGVKSGKIDIKDVDRNVRNML--MVLLKNGK
HILPM-KQMSKVALFGVNSYDFMSGGLGSGCVNVPYVVDMVQGLKNAGISEISRRCVEHEVSEAQAAIITIGRQ---AGEGLDRGEFNLSAQEKDMISNVSNQGKPVIV
VINSGSVMETASW-RDLVDAILVAWQPGEEGGNSVVDVLTGKANPSGRLTMTWPIAAVDHPSDYY----NFDFDKPVAYPFGYGLSY
>Km_bglu
--TKK-IERLGIPAVRVSDGPNGIRTKFFDGGCFPNGTGLASTFDRDLLETAGKL
MAKESIAKNAAVILGPTTNMQRGPLGGRGFESFSEDPYLAGMATSSVVKGMQGEGIAATVKHFVCN--DLEDQRFSSNSIV-SERALREIYLEPFRLAVHANPVCIMTA
---YNKVNGDHCSQSKKLLIDILRDEWKWDGMLMSDWF--------GTYT--TAAAIKNGLDIEFPGPTRWRTRALVSHSLNSREITTEDVDDRVRQVLK-IVLLKNKN
NYLT-SKERRQYHVIGPNAKAKTSSGGGSASMNSYYVVSPYEGIVNKLGKDEEIRNAAELAAKHDKAVLIIGLNGEWETEGYDRENMDLPKRTNELVRAVLKANPNTVI
VNQSGTPVEF-PW-LEEANALVQAWYGGNELGNAIADVLYGDVVPNGKLSLSWPFKLQDNPANFKTEF-GYELQRKVAFPFGYGLSY
>Pp_hexo
---------------------ELSELISRYDIGGAILFAENVQNTAQIISLTNALQSAAQQSKSQLPLFIAIDQEGGRVARINRETSFTGNMSIGATYPKQYATKVASA
IGKELNSLGINVNFAPTVDVNSNPNNPINVRSFSENPTVVTKLGLAQVKAFEAAGVLSALKHFPGHGDTHVDSHTGLPRVDHDRDKINQQDLLPFAEIIASPPGMIMTA
HIQYPALDNSPATMSYQIMTQLLRHELGYQGVTVTDALDMAGISDFFNPVDATIETFNAGVDIALMPIAIRNRMAQLADALETNKLNQEQLSSSMARIAK----VKND-
GVLPLRDNAQVVHLIMPDRQKCFALEQALQTYSKNSLTLSCTSLQAY-----DPDIAHDAIKQADMIIAAHASPPQSAVEIGDVARNVQPAALKALLQYGQQQGKK-QL
FISLRAPYEISTF-GPLSNAVLASYAYNVDVNHALAKVILGIAKAEGSLPVTV----------------------------------
>Dc_bglu
------------------DLNAAKTMIADRYVNSFIT-RLSGDNPAQMAEENNKLQQLAEATRLGIPLTISTDSLVGVSVSVGKFSKWPETLGLAAIGDEELVRRFADI
VRQEYRAVGITEALSPQADLATEPRWPRIDGTFGEDPDLTKKMVRGYVTGMQNGSVISIVKHWVGYGAAKWDSHNVYGKYAQFRQNNLQWHIDPFTGAFEAHAAGIMPT
YLRNASWHGKGAGFNRFLLTDLLRGQYGFDGVILSDWLITNDCKGDCLPAERFVKAVNAGVDQFGGVTDS----ALLVQAVQDGKLTEARLDTSVNRILK--VLLQNN-
NLLPLRKG-SRVWLHGIAANAAQEVGFIVVN-------TPEQADVALIR-------THTPYEQPHK-NFFFGSR---HHEGS--LAFRNDPDYQAIVRASAKV--PTLV
TVYMERPAILTNV-VDKTRAVVANFG---VSDSVLLNRLMSGAAYTAKLPFELPSSMSAVRN--QQPDLPY-DSAKPLFPFGYGL--
>Ec_bglu
TVDEKIGQLRLISVGPDNPKEAIREMIKDGQVGAIFN-------TVTRQDIRAMQDQVMELSRLKIPLFFAYDVLHGQR------TVFPISLGLASSFNLDAVKTVGRV
SAYEAADDGLNMTWAPMVDVSRDPRWGRASEGFGEDTYLTSTMGKTMVEAMQGKSVMTSVKHFAAY--GAVEGGKEYNTVDMSPQRLFNDYMPPYKAGLDAGSGAVMVA
---LNSLNGTPATSDSWLLKDVLRDQWGFKGITVSDHGAIKELIKHGTPEDAVRVALKSGINMSMSDEYYS---KYLPGLIKSGKVTMAELDDAARHVLNVLVLLKNRL

ETLPLKKS-ATIAVVGPLADKRDVMGSWSAAGVADQSVTVLTGIKNAVGEQEMIDEAVQTAKQSDVVVAVVGEAQGMAHEASSRTDITIPQSQRDLIAALKATGKPLVL
VLMNGRPLALVKE-DQQADAILETWFAGTEGGNAIADVLFGDYNPSGKLPMSFPRSVGQIPV-YYSHLRPYFEANGALYPFGYGLSY
>Ni_bglu
-------------------TAAQAIIRDQHLNSLIT--RMAIAPADFAAENNRLQGIAAGTRLGIPLTISTDQVLGGASVAASGSQWPETLGFGALNDPALTRRFADL
VRAEYRAVGIQMALSPQADLATEPRWSRINGTFGEDPARVSAQVKAYVQGMQGAGVATVVKHWVGYGAQGYDGHNYYGRFTDFTKGGFDRHVAAFQGAFEAGATGIMPT
YTIQKSLEGKPVSGNKQMLIDLLRGTHKFKGLILSDWAITND--
---EADIALIRLKAPFQTQHEG-DL-DFKEGDAGLTLVRQAAAK-VPVIL
TIYLDRPAILTNI-KPHAATLIGEFG--I-TDAALFDALTGKVAPMGKLPFELPATMAAVRA--QSPA--LPDSADPLYPVGFG---
>St_ac-glc-ami
TLQEKVGQLFVMRVYGEIGVRTAAELIEKYHVGGIIYFAWNTRDPQQIADLSNGIQKAALAQPRGLPLLIATDQEHGIVCRIGKPTLFPGAMAIGAGGSTADARTLGRI
SGAELRAMGVNQDYSPDADVNVNPANPIGVRSFGADPDAVARMVAAQVKGYQGSGVAATAKHFPGHGDTAVDSHTGFPVITHTREQWETLDAVPFRAAIKAGIDSIMTA
HLQFPALDPSPATLSRPILTGILREELGYDGVVITDSLGMEGVRTKYGDDRVPVLALKAGVDQLLNPPSLDVAFHAVLDAVRSGELTEARLDESILRILRL-TLLVNKG
NLLPLRRSHQRILVVGADPD----SPSGTTGPTTGVLAAALNELGFTATAAAVTDQAVAAARDADAVVVATYNV-------------TAGSAQQTLVERLAATGRPVIA
V-AVRNPYDVAQL--PAATAVLAAYSWTDVEVRAAARVVAGRVSPRGTVPVPVQRADD--PE-------------QTLYPIGYGLSY
>Bs_bglu
-------------------DQQLDFLSNDHIRHILV--TRVQSPEVAANWNNNVQAYAERLGLGIPANNSSDPRHGSDTSAGGASMWPESMGLAATFDPAVAREFGEI
ASREYRALGLSTALSPQVDLATDPRWFRFGMTFGEDPRLATDMARAYIDGFQTSSVNAMVKHWPGGGSGEADAHFGYGKYAVYPGNNFEEHLRPFTEGATGEASAVMPY
YTISVGQDPVGNAYNAYLIRDLLRGKYGYDGVVCTDWGI--VVMLKNE-
GILPLPKRQ-TVYIPKRKLP---ADADWMGNPVPPSETYPLDVVRKYF-------DVTDRPADADFALVCIESPRSTAGDPRDVKTAAVA-NEGDLDAVLEMNGKPVVV
SIALSNPAVAAEF-EPAADAILAHFG---VQDQAILDILTGAFEPQALLPFRMPADMTTVEKEDVPHDDVYVDSAGHAYDFAFGLNW
>Ps_ac-glc-ami
TLKQKVGQLVMAGFNGLEASDDARKLITEDHVGGIIYFRRNLAEPAQVAKLSAELQQIAAE-SDNVPLLISIDQEGGMVTRLENGTVVPGNMALGAAGDAELAYEAAHI
IGSELRALGINMNFAPSLDINNNPGNPIGVRSYGGTAELVARLGTEAVRGFQDAGVAATVKHFPGHGDTGEDSHHALPTVPHARERLDRLELAPFREAIARGVDAVMTA
HVLFPAVEPEPATLSSNVIEGLLRGELGYDGVVVTDCLEMNAISKFYGVGEGAVQAVEAGADLILVSHRYERQLDALLAAVESGRISEERIDRSVGRLLA---------
--PDQAALIEALAAKPDVQLI
VASARNPFDINAL--PTVKTFFAAYENTPSAMRALALVLTGQIAVQGTLPA------------------------------------
>Cf_GH3
TLSEKIGQMVQLD-----RTVASAEIMRDYSIGSLLSGGGHKATAQDWTNMVNHFQNGSLSSRLGIPMIYGIDAVHGHNNVYKA-TIFPHNIGLGATRDPELVKRIGAA
TALEVRATGINYVFAPCI-VCRDPRWGRCYESFSEDPKLVEAMT-DIITGLQGDKVAACAKHFVGD--RGTVNGINENNTVIDRHGLLSIHMPGYYHSVIKGVSTIMVS
---YSSFNGKKMHANYDLVTTFLKDTLKFRGFVISDWEGIDRITSPPHYSYSVLAGIQAGIDMVMLPYNHTEFIYILTGFVNNNVIPMSRIDDADGRILRVLVLLKNGE
ALLPLPKKASRILVAGTHAIMGYQCGGWTITQNHTEGTTILNAISAAVDPSENPESDFVNSNNFSYAIVVVGEPPYAETQG-DSLNLTMLEPGPSVINNVCGRVKC-VV
VVVSGRPIVIEPF-VSQMDALVAAWLPGTE-GQGVSDVLFGDYGFTGKLPRTWFKSVDQLPMNVGDA------YYDPLFPFDFGL--
>Aa_bglu
TLPEKIGQMTQIE-----RTVASPTVITDSFIGSVLNAADPFEDAKDWADMIDGFQRSALASRLGIPIIYGIDAIHGNNDVYGS-TIFPHNIGLGATRDEDLVRRIGAA
TALEVRASGAHLTFAPCVAAVRDPRWGRCYESYGEVAKIVCEMT-SVVSGLQGENVVACAKHFAGD--GGTNKGINEGNTILSYKDLNRIHIASFKKCIAQGISTVMVS
---YSSWNGDKLHSHYFLLTEFLKQKLGFKGYINSDWEGLDRLSDPPGYRNCVKIGINAGIDMVMVPFRYKEFIGDLINLVESGEVPMARIDDAVERILRVLVLLKNGN
QFLPLNCNAEKILVVGTHADLGYQCGGWTKTMKITIGTTLLDAIKAAVVEKYPSKETLASGYRFSYAIVAVGEAPYADTKG-DNSELIIPFNGSDIITMVAEK-IPTLA
ILFSGRPMVLEPQVLEKTEALVAAWLPGTEG---
>Es_bglu
TVEEKIGQMTQIH-----RGVSSAAVIKDFFIGSVCNSAGNDVLSVDWAEMIDGFQTAALETRLAIPIIYGLDAVHGNNKFYG-ATIFPHNIGLGATRDEDLARRIGSA
TALEVRASGAHWAFAPCVAVCKDPRWGRCFESYSEDTDVVCNMT-SFVSGLQGTNVVACAKHFVGD--GGTEKGKNEGDTVASYEDLERIHMSPYLKCLAQGVSTVMPS
---YSRWNGSKLHADRFLLTEILKEKLGFKGCLVSDWSGIDKMGEPRGYRECVEAAINAGIDMVMVPYKYEKFINDLTSLVQSGEIPMSRIDDAVERILR-LVLLKNGK
PFLPLDRNAKRVLVAGTHAD---DLGFQCGGMRP--GTTVLDAVKAIVGAEKSPSEETLSGKDFSYAIVVVGEAPYAESRGDDP-EPSIH-FDGAEVMRLVAGKIPTVV
ILMTGRPVILDPTVLDKVEALVAAWLPGTE-GDGIADVVFGDYDFSGKLPISWFRTTEQLPM--NKEAD----GYDPLFPFGFGL--
>Fv_bglu
TLKEKVGQMTQIE-----REVATPFAIKDLSIGSIISGAGKKALSADWADMVDGFQRCALETRLRIPLIYGIDAVHGNNSVYGA-TIFPHNVGLGATRDADLARRIGEA
TALEVRASGIHYTFAPCVAVCRDPRWGRCYESYSEDTEIVRKMT-SIISGLQGKNTIACAKHFVGD--GGTQKGVNEGNTISSYYDLERIHMAPYLDCISQGVSTIMAS
---YSSWNGNRLHADRFLLTEILKGKLGFKGIVISDWDGHETISEPQGYRFSVLSAINAGIDMVMVPFRFKNFVDDLVNLVEHGEIPMSRIDDAVERILRVLVLLKNGK
PFLPLDRKAKRILVTGTHADLGYQCGGWTVTKRFTIGTTILEAIKKAVGHEQLPSTDTLARNDISFAIVAIGEGSYAECKG-DNSELVIPFDGDDIITSVAER-IPTLV
ILISGRPLTVQPSLLDKMDALVSAWLPGSE-GEGIADVIFGDYNFEGKLPVTWFKSVEQLPLDAGSN------SYEPLYPLGFGLT-
>La_unpro
TLKEKIGQMTQIE-----RTVATPSSLIHLSIGSILSCGGENAMSSDWADMVDGFQKSALESRLGIPLIYGIDAVHGNNNVYGA-TIFPHNIGLGATRDADLARRIGAA
TALEVKASGVHYTFAPCVAVCKDPRWGRCFESYSEDTEIVRKMT-SFVSGLQGQNVIACAKHFVGD--GGTYKGMNEGNTALSYEELEKIHMAPYLDCISQGVSTIMAS
---YSSWNGHKLHANNFLLTEILKEKLGFKGFVISDYEGIDRLCDPRGYRYCISSAINAGIDMVMVGVRFEQFIEDLTFLVESGEVPISRIDDAVERILRVLVLLKNGK
PFLPLDRNAKRILVTGTHVDLGYQCGGWTKTGRITIGTTILDAIKAAVGVEQYPSKDTIENNGIVFAIVAVGEVPYAETSG-DNSKLRIPVDGAEIISLVADK-IPTLV
ILISGRPLVLEPWLLEKTEAVVAAWLPGTE-GDGITDVIFGSHDFKGQLPMTWFRRVEQLDH---QPSD--GNSSEALFPLGFGLSY
>Rs_unpro

--------GAHWAFAPCVAAMRDPRWGRSYESYSEDADTICDLT-TLVSGLQGENVVACAKHFVGD--GGTENGTNEGNTIVSFEELERVHLPPYLNCLAQGVSTVMAS
---YSSWNESKLHSDYFLLTELLKQKLGFKGFVISDWEALDRLSEPFGYRHCVKISINAGIDMVMVPFKYEQFIHDLTDLVQSGEVSMARIDDAVERILRVLVLLKNGK
PFLPLDRNAKRILVTGTHAD---DLGYQCGGWRITIGTTLLDAIKAIVGDKNPSEESLASSEAFSYAIVAVGESPYAETMG-DNSELTIPFNGSDIVTTVAER-VPTLM
ILFSGRPMVLEPTVLGKTEAVVSAWLPGSE-GQGMADVIFGDYDFEGKLPVSWFKRVEQLPVNA-DS-DLY----DPLFPLGFGL--
>At_bglu
TIAEKVGQMTQIE-----RSVVTLDAMKNQFIGSILNAGGSGASAEEWADMVDGFQRWALESRLSIPVLYGTDAVHGHNNCYGA-TIFPHNIGLGATRDPNLVQKIGEA
TAIEVRATGIPYTFAPCVAVSRDPRWGRCYESYSEDTETVRKMT-TIVQGLQGSNVIACMKHFIGD--GGTKGGINEGNTVGSFDELHSVHLKPFLDCLDQGVCTAMAS
---YSSWNSMMLHSHHFLLTQVLKHQLGFKGFVISDWEGIDRLCQPQGYRFCISASINAGIDMVMVPHDFQKFIGDLTFLVESGEISMTRIDDAVERILRVLVLLKNGK
PFLPLSKNGGRILVGGEHAHLGYQCGGWTITWQITQGTTILEAIKAAVGMEENPTETSFKTQEFSSAIVVVGEKPYAEFLG-DDPKLELSPKAIETIELVCSK-VPTLV
ILLSGRPLIVEPL-IEKMEAFVAAWLPGSE-GAGVADVIFGGYEFHGCLPRTWFKRVDQLPMNVGD------SNYDPLFPFGFGLK-
>At_unpro_I
TLAEKIGQMTQIE-----RQVANYSVMKEYAIGSILSGGGQASAAVWVNMVNEFQRGALA-SRLQIPMIYGIDAVHGHNNVYGS-TIFPHNVGLGVTRDPDLLKRIGAA
TALEVRATGIPYTFAPCIAVCRDPRWGRCYESYSEDPEIVEAMT-EIIPGLQGDKVAACAKHFVGD--GGTHNGINENNTIIDRHGLLAIHMAGYYHAIIKGVSTVMVS
---YSSWNGEKMHANRDLVTNFLKKTLHFRGFVISDWQGIDRITSPAGYPYSVHAGVNAGIDMIMVPYNFTEFINDLTEQVNSKSIPMSRIDDAVRRILRVLVLLKNGK
PLLPLDKKAPKILVAGTHADLGYQCGGWTIQWNTTKGTTILTAIKSTVSPEENPSASSLKGQDYDYAVVVVGETPYAETNG-DSMNLTMPEPGPTIIKNVCGSVKC-VV
VVISGRPIVLQPF-LQYIDALVAAWLPGTE-GQGVADNLFGDYPFTGKLARTWFKSVDQLPMNVGDK------HYDPLFPFGFGLT-
>At_unpro_II
TLAEKIGQMTQID-----RSVATQDVMKNYSIGSVLSGGGN-ATVEDWVNMVNDFQKGALSSRLQIPMIYGIDAVHGHNNVYGA-TIFPHNIGLGATRDPELAKRIGVA
TALEVRATGIPYVFAPCLAVCRDPRWGRCYESFSENPEIVEAMT-EVIVGLQGGSVAATAKHFVGD--GGTVRGIDENNTVIDYHDLLSIHLRPYYRAIIKGVSSVMVS
---YSSWNGVKMHSNRKLVTGLLKSTLRFRGFVISDWQGIDRITSPPGYMFSVHAGVNAGIDMIMVPYNYTDFINDLTDQVNSKSIPMSRIDDAVRRILRVLVLLKNGK
PLLPVDKKAPKILVAGTHADLGYQCGGWTITWNTTIGTTILSAIRSTVDPQENPDPSSLKANDYSYGIVVVGELPYAEFDG-DSTTLTMIEPGPTIIKNVCASMKC-VV
VVVSGRPIVLEPY-VPYMDALVAAWLPGTE-GQGLADVLFGFYPFSGKLPRTWFKSVNQLPMNVGDK------HYDPLFPFGFSL--
>Ps_bglu_I
-------------------------VKKYYIGSVLSGGGKASP-ATWINMVDDLQKGAMSTRLQIPMMYGIDAVHGHNNAYGA-TMFPHNIGLGATRDPDLARRIGAA
TALEVRATGIQYTFAPCVAVCRDPRWGRCYESYSEDPKIVKAMT-QIIFGLQGQNVAACAKHFVGD--GGTTNGIDENNTVIDYKGLVNIHMTPYFDAIAKGVSTIMVS
---YSSWNGMKMHANRFLVSEVLKKQLGFKGFVISDWQGIDRITSPPGYSLSVFDGVGAGIDMVMVPENFTNFITELTSQVKGGLISMTRINDAVRRILTVLVLLKNGK

PLLPLDKNAPKILVAGTHPNLGYQCGGWTIEWNSTIGTTILQAIKFAVSPQQNPDANYVKGQGFSYAIVVVGEAPYAEMNG-DNLNLTIPLGGGDTIKNVCSSLKCLVI
LI---
>Ps_bglu_II
TVEEKIGQMTQIE-----RSDATADVMKKYYIGSVLSGGGKASP-ATWINMVDDLQKGAMSTRLQIPMMYGIDAVHGHNNAYGA-TMFPHNIGLGATRDPDLARRIGAA
TALEVRATGIQYTFAPCVAVCRDPRWGRCYESYSEDPKIVKAMT-QIIFGLQGQNVAACAKHFVGD--GGTTNGIDENNTVIDYKGLVNIHMTPYFDAIAKGVSTIMVS
---YSSWNGMKMHANRFLVSEVLKKQLGFKGFVISDWQGIDRITSPPGYSLSVFDGVGAGIDMVMVPENFTNFITELTSQVKGGLISMTRINDAVRRILTVLVLLKNGK
PLLPLDKNAPKILVAGTHPNLGYQCGGWTIEWNSTIGTTILQAIKFAVSPQQNPDANYVKGQGFSYAIVVVGEAPYAEMNG-DNLNLTIPLGGGDTIKNVCSSLKC-LV
ILISGRPLVIEPY-LPLVDAFVAAWLPGTE-GQGVTDVIFGDYGFQGKLPRTWFKSVDQLPMNVGDK------HYDPSFPLGFGL--
>Mp_bglu_I
TVQEKIGQMTQIE-----RTVATPEVMTQYFIGSVLSGGGEPNAPAAWQDMIDTMQQAALATRLAIPMIYGIDAVHGHNNLYGA-TVFPHNIGLGCSRDPDLVKRIGAA
TALEVRATGIPYAFAPCIATCRDPRWGRCYESYSEDTAVVKTMT-DIILGLQGDKVIGCAKHYVGD--GGTFKGINENDTIVDYDTLYKVHMAPYLDAIAKGVSTIMVS
---YSSWNGERMHANQYLVTQVLKEQLAFRGFIISDWMGVDRLSDPNNYTNSVLKSINAGLDMIMVPFDYEAYISGMLSLVNDGEISMERIDDAVTRILRVLVLLKNGQ
ALLPLKKNATSILVAGSHADIGLQCGGWTISWNTTIGTTVLDAIKAAVSPEKNPAPGFAAQLKPDYAIVVVGEEPYVETYG-DNMELTIPLDGIPTIQNVCAEVKC-LV
IVISGRPLVIEPY-MPQIDALVAAWLPGSE-GQGISDVIFGDYDFVGKLSRTWFRTVDQLPMNFGDA------VYDPLFPFDFGLT-
>Cs_GH3
-LKEKIGQLIVVRTTGEANQQQLQTWLSEYNIGGVIL--------LGGSCAEIAQRTKQLNQWAKTPLLVAADIEEGVGQRFTGASWFPPPMALAQIAQDDYAEEMGKI
TAKEALCIGVNWIFAPVVDVNNNPDNPINVRAFGDNPEVVKELSSAFIRGTQSYPILNGAKHFPGHGDTSTDSHLDLPVINHSQARLEKIELVPFQGAIALNVDAIMTA
HLLVSAYDNQPATLSHRILTEELRHNMGFEGLIVTDALIMGGVAKYAPPEKIAVKALQAGADILLMPENPVVAIHSIIEAVEKGEISEHRIDESLQRVSK---------
---F-------------------------

>Cs_bglu
-LQEKIGQLIVVRTTGEANNQQLQTWLSEYNIGGVIL--------LGGSCAEIAQRTKQLNEWAKTPLLVAADIEEGVGQRFTGASWFPPPMALAQIAKKDCAEEMGKI
TAQEALSIGVNWILAPVVDVNNNPDNPINVRAFGDNPGVVRELSSAFIRGTQYYPILNGAKHFPGHGDTSTDSHLDLPIINHSRTRLEAIELIPFQGAIALGIDAIMTA
HLLVSAYDNQPATLSHAILTKQLREKMGFDGLIVTDALIMGGVAKYAPPEKIAVKALQAGADILLMPENPLVAINSIMEAVQRGEITESRIDESLQRIAK---------
---AE--

>Cb_bglu
-LDEKIGQMTQVE-----KNSILPGEVSRYYIGSILSGGGGDDSLEGWVKMVDGLQAAALETPLAIPLIYGVDAVHGHNNVKG-ATIFPHNIGLGATNDPELVEKIGRA
TAEEMLATGISWDFAPVLAVVQDIRWGRTYESYGENTELVTRLGVAYQNGLQAAFVLATPKHYIGDGGTTWASKLDQGDTQMDEARLRELFLPPYQAAVEAGAQSVMVS
---YSSWNGVKMHGHKYLITDVLKGELGFEGFVVSDWAGIDQVDSD--YYTAVVTAINAGVDMNMVPQGYPRYLTVMQQAVEKGDIPMERIDDAVLRILTVLVLLKNDN
ATLPLAKDAGLIFVAGASANIGAQCGGWTIEWNITTGTTILEAIEASASAEVRFDRFGESEQKADVAVVVIGERPYAEGRG-DKENPSLSKSDIELIQRVREQSQRVVV
ILLSGRPLVITEA-LPYADAFVAAWLPGTE-GSGVADVLFGDKPFTGKTPFSWPRSADQLPFDFANLP--ADGCAAPLFPYGYGLD-
>Xc_bglu
-VEEKVAQTIQ-----GDIASMTPDDVRKYRIGSVLAGGNKPAEWLKLADAFYEASMDTSKGGNAIPIIFGIDAVHGQSNIVGA-TLFPHNIGLGATRNPDLIKKIGEV
TAAETRVTGMEWTFAPTVAVPQDDRWGRSYEGYSESPDVVASFAGKMVEGVQGVHVISSVKHFVGD--GGTTDGKDQGDTKVSEATMRDIHAAGYPPAIAAGAQTVMAS
---FNSFNGEKMHGNKVMLTDVLKGRMNFGGFVVGDWN--GHGQVKGCTNENCPASFIAGVDMAMASDSWKGIYETELAAVKSGQISMERLDDAVRRILRVLVLLKNQA
GVLPLDPK-KRVLVVGDGAN---DMGKQSGGWTLNWQGTIWEGLNKQITASAELAVDGAYKTKPDVAVVVFGENPYAEFQG-DIATLLYKDSELALLKKFKAEGIPVVA
VFLSGRPLWMNQY-INVADAFVAAWLPGSE-GEGIADVLLRKSDFKGKLSFSWPKTAVQFANNVGQ------KDYDPQFKFGFGLTY
>Cr_GH3

---TPDLRESYLPAFRACVEGRAHSVMCA
---YNKVNGVPACAHPHLLRATLNDSWGFANFVVSDCGAVSDLALTHGLAAAAAAEALTAG-GLSLFCDNAA--AAAVPQAVASGLLAPAVLRAAVRRML---------
-----------LALLGPHADALYYLGTYYGTPSH-PVVTPLAALREALGPSDGLHTCTQAAAAAQVAVVFVGGSSRNESEGLDRGSLRLPGLQEELVRAVARSGVPVVV
VAVAGGPLDLSPLGLQGVAAVLAAPYGGQQAGYALASVLLGTSSPSGRLPATWLYDWY-----------------------------
>Mp_bxyl_I
--KGATSFPMPISLAASFNKSLWNKIGQV
ISTEGRAMGQSTFWSPVINLVRDPRWGRVQETPGEDPYLIGQYSVYFVRGMQEAKTSACCKHYTAY-DLDNWQGVERYDFDVTIQDLADTYNPPFQSCVEGRASCLMCS
---YNKVNGVPTCADPELLKGTVREKWGLRGYIVTDCDSLLVMYDESRPAEAIATAMLAGLDLNCGATIKT----YGASAVQQRLISEADIDRALN-----MVLLKNDE
DTLPLSRHIKTLAIIGPNADKYTMLGNYAGRPCT--YITPLQGLSTYEINDEKIEDALSAAKKADVVILVMGLDQDLERETFDRNSLKLPGKQELLVSSVANVNGPVVL
VLICGGPLDISWANDSRIQSILWMGYPGQAGGLALAQIVFGDRNPVGRLPVTWYPSITDWPMM--NMRHTHRYNGPTVYEFGYGMTY
>Mp_bxyl_II
---GRVKSATSFPQPILTAASFNKELFNKIGQV
ISTEARAMHNETFWAPNINIFRDPRWGRGQETPGEDPYLTSIYAEYFVRGMQEDKTSACCKHFTAY---DIDQWYDVDRAKVTQQDLLDTYNPPFQSCIDGKASSLMCS
---YNRVNGVPTCADYNLLTKLARGTWGFDGYIVSDCDAVQVMYANSRPEEAVAYALKAGMDLNCGDTAS----NFTVEAVHSGLLNTSDIDKAL------IVLLKNDQ
NTLPLADKIRSLAVLGPNANVNTMLGNYAGPPCV--YVTPYLGLAQYVPDSDFIRGAAKVATWTDAVVVVVGLSQDQEREAFDRTSLRLPGQQEELITTVSRVAKPVIL
VLMTGGPVDIGFIDDPKIQSILWVGYPGQAGGQALAQVIFGDRNPGGKLPMSWYPESYTEPMDMHMRPDEYRYSGDVIYRFGEGMSY
>Mp_bglu_II
TLAEKVGQMTQIE-----RGVSNNSVVKDLFIGSVLSGGGAGNTAEAWQDLVDRLQNDALSTRLGIPMIYGIDAVHGHNNVFGA-TIFPHNVGLGCTRDPDLVKRIGVA
TALEVRATGIPYVFAPCIATCRDPRWGRCYESYSESTEVVTSLT-DIILGLQGEKVAACAKHFVGD--GGTTNGINENNTVIDYKGLVDIHMAPYFDSIAKGVSTVMIS
---YTSWNGMKMHANKFLVTQVLKYNLKFKGFVISDWEGIDRITSPAGYTFSIESSINAGLDMIMVPNNYPQFINGLTDLVKGGYINMHRIDDAVTRILRVLVLLKNGK
PLLPLSKTAAKIIVAGSHANLGRQCGGWTITWTTTKGTTILDAIKGAVSPEPYPQPGFAQSQGADFAIVVVGEPPYAETFG-DNLNLTIPEAGIPTIQNVCGEVR-CLV
ILVSGRPLVVEPY-LPVMDAFVAAWLPGSE-GAGVSDVIFGDYDFVGTLARTWFKSVDQLPMNVGDE------KYDPLFPFGYGLK-
>Mp_bglu_III
TLDEKIGQMTQIER-----YVANFDVMKNFSIGSVLSSGGNSSTTEAWQNMVDDLQAGALATRLGIPMLYGIDAIHGHNNVYGA-TIFPHNIGLGCTRDPDLVRRVGAA
TALELRATGIPYTFAPCIAVCRDPRWGRCYESFSEDTSVV-RMMTDVIYGLQGNKVAACAKHYVGD--GGTQRGINSNDTILSYEDLFRIHVAPYVDAIAKGVSTIMLS
---YSSWNGVKMHMNHRLISTLLKQELGFKGFVISDMEGIDFITDIPDYTASVLESINAGLDMIMVPFDYEKFISTLRTLVNTGYISMQRIDDAVTRILRVLVLLKNGK
PFLPLSKNATRVLVAGTHADVGLQCGGWTISWSITKGTTVLDAVKAAVFTEASPTAEVAAKTKADFAIVVVGEQPYAEGAG-DNTNLTIPDEGISTIKNVCSEVKC-LV
ILISGRPLVVEPY-LPLMEAFVAAWLPGTE-GNGVTDVIFGDYDFVGSLSRTWFKSADQLPMNFGDPI--Y----DPLFRFAFGLG-
>Mp_bxyl_III
--KGATSFPMPISLAASFNKSLWNKIGQV
ISTEGRAMNLKQSWSPVINLVRDPRWGRVQETPGEDPYLIGQYSVYFVRGMQEAKTSACCKHYTAY-DLDNWHGIERYYFEVTKQDLADTYNPPFQSCVEGRASCLMCS
---YNKVNGVPTCGDPDLLKGIVRKAWGLRGYIVTDCDSLLVMYDQSRPAEAIATAMLAGLDLNCGSTIK----NHGATAVNQSLISEDDIDRALS-----IVLLKNEE
DSLPLPSQIKTLAIIGPNADKYTMLGNYAGRPCK--YVTPLQGISTYHIDDEKIEDAVSAAKKADAVILVMGLDQDLERETFDRNSLKLPGKQELLVSSVANVSGPVVL
VLMSGGPLDISWATDSKIQSILWMGYSGQAGGLALAQIIFGDRDPVGRLPVTWYPETIDWPMN-MNMRDPHRYNGPTVYEFGYGMSY
>Mp_bxyl_IV
--SGATSFPQPILTASSFNMTLVGQAIST
EGRAMYNLGQSTFWAPNINIFRDPRWGRGQETPGEDPLLTSIYASTFVRAMQEIKTSVCCKHFTAY-DVENWDGIDRYHAEVSDQDLEDTYNRPFQSCVEGQASSMMCS
---YNRVNGVPTCANYNLLTETARNTWGLNGYITSDCDAVELIYSAVNAEDAVAEVLMAGLDLNCGSTAA----NFGQSAVDQGKVNESTIDRAL------IVLLKNDN
NVLPFRGHIQKLALTGPNANTSSMLGNYAGVPCE--YITPLQGLQTYVDLGGLIGAAAAIASSADAVVIVAGIDQTQEQETLDRTSLLLPGQQQSLIETVANSSKPVIL
VIMSGGAVDISFAFNPQIHGILWVGYPGQGGGQALADILFGHRNPGGRLPVTWYPSMTDMNMPN-----TYRYTGETVYSFGDGLSY
>Sf_bglu
TLAEKIGQMTQIE-----RSVANASDIEHYLVGSVLSGGGPNATAFQWNNMTDYFQRSALKTRLGIPLMYGIDAVHGHNNVYGA-TIFPHNIGLGATRDPELAQRIGVA
TALEVRSTGIQYVFAPCLAVCRDPRWGRCYESYSEDPDVVTSMT-TIIDGLQGQKVAATAKHFVGD--GGTTHGIDENNTVISYKGLVDIHMKAYFSAVARGVSTIMVS
---YSSWNGEKMHANRFLLTDVLKGQLGFKGFLISDWEGLDRITTPYDYTYSVFTGIHAGIDMVMVPFDYEGFASNLTKEINIGNIPMSRIDDAVSRILRVLVLLKNGK

```
PLLPLDKNAQKILVVGAHANIGLQCGGWTITWNTTLGTTILQGIQQTVGSKAVPDKNFAKGKGFDYAIVVIGEVPYAEFVG-DNNNLTIPAPGIETVEYTCKAVKC-VV
VIISGRPLVVPPYLLQSMDAFVAAWLPGTE-GQGIADVLYGDYDFTGKLSRTWFKSVDQLPMNVGDD------YYDPLFPFGFGLK-
>Cp_bglu
TLAEKIGQMTQTE-----RSVTNHTNIRDFGIGSILSGGGENATVLQWGNMTNYFQKAAMNTRLAIPLIYGIDAVHGNNNIYGA-TIFPHNVGLGCTRDPELVERIGSA
TALECRATGIQYAFAPCIAVCRDPRWGRCYESYSEDPEIVRDMT-SLIDGLQGRKVAACAKHFVGD--GGTTRGLNGNNTQVSYRELVNIHMKAYKDAVRKGVATVMAS
---YSSWNGVKMHANKFLLTRVLKKELGFKGFIISDYMGIDLITDPPGYTYSVYAGIHAGLDMIMVPFAYEQFIGNLTQMVKSGAIPMSRINDAVTRILRVLVLLKNGK
PLLPLNRNAKRILVTGSHASIGLQCGGWTIKWDITPGTTVLEGIQQAVSPAEKPGKGLFENLGFDYAIVVVGEPPYAETHG-DNLNLTIPLNGPHAITHTCRRV-RCVV
VLMSGRPLVVAPLLH-QMDALVAAWLPGTEAGLGIADVLFGSYDFTGKLARTWFRSVDQLPMNVGDK------HYHPLFPFGYGLT-
>Pp_bglu
TLLEKIGQMTQTE-----RTVTNHTNIREFGLGSILSGGGENASVFQWDNMTNYFQRAAMSSRLQIPINYGIDAVHGNNNIYGA-TIFPHNIGLGCTRDSDLVERIGTA
TALESRATGISYVFAPCIAVCRDPRWGRCYESYSEDPEIVRNMT-SLIDGLQGRRVAACAKHFVGD--GGTTDGINGNNTEVSYDELVNIHMKAYKDAIDKGVTTIMAS
---YSSWNGVKMHANHFLLTKVLKEQLGFKGFIISDYMGIDQITDPPGYTYSVYAGIQAGLDMIMVPFAYDQFIGNLTQMVKSGLIPMSRIDDAVTRILRVLVLLKNGI
RLLPLNRHAKKILVVGSHANIGLQCGGWTIHWDITPGTTVLQGIQQAVSPSERAKKSLIKDQDFDYAVVVVGEPPYAESQG-DNTNLTIPLMGTHAIRNTCRYV-RCVV
VIISGRPLVIEPY-LPMMDALVAAWLPGTEAGLGIADVLFGAYDFTGKLSRTWFRSVDQLPMNVGDK------YYDPLFPFGFGLS-
>Sm_bglu
TLEEKIGQMTQIE-----RENATGSVITKYFIGSVLSGGGSVASAATWAAFVDGLQDGALATRLGIPIIYGIDAVHGHNNVYGA-TIFPHNVGLGSAGDPDLVKRIGAA
TALEVRATGIQYTFAPCVAVCRDPRWGRCFESYSEHPELVKAMT-TIISGLQGEKVAACSKHYVGD--GGTRSGINENNTVGSYKRLVGTHMLPYFDAIDKGVSTVMIS
---YSSWNGIKMHKNRHLITDILKKRLRFKGFVISDWQGIDRITNPAGYTYSVLVSVTAGIDMIMVPYEYTKFIDTLTSLVKQGFISLDRIDDAVRRIL--LVLLKNGK
PLLPLSKTASKILVAGSHANLGNQCGGWTITWNTTLGTTILQGISNTVSKEESPSSSSVKGGGYDFAIVVVGEPPYAETQG-DNLNLTIPQDGANTIESVCSSVKC-LV
ILISGRPLVVAPH-LSSMDALVAAWLPGSE-GQGIADVIFGDYDFQGKSSRTWFKSVEQLPMNY-------GVEYDPLFPFGYGLK-
>Cr_bglu
TLAEKIGQMTQIE-----RLVADPSVIQNYFIGSILSGGGPNASPSAWADMVDGFQTAALSTRLGIPIIYGIDAVHGHNNVYGA-TIFPHNVGLGATRDPNLVQRIGAA
TALEVRATGIPYAFAPCIAVCRDPRWGRCYESYSEDPELVQQMT-QIVFGLQGTNVVACAKHYVGD--GGTTGGIDESNTVATFNELRRIHLPPYYNAIAAGVATIMVS
---YSSWNGVKMSANKFLISTMLKQRLGFQGFTISDWEALDRITSPPDYNYSVNVAINAGLDMVMVPMNYQGFINLMTAQVNAGQISQDRIDDAVRRILRVLVLLKNGK
PMLPLSKNAPKILVAGAHANIGLQCGGWTITWNTTVGTTILEGIKNAVSPLVSPDSGNAANGGYSYAVVVVGEP--TYTEMFDNDNLTIPQDGINLIQNVCSVVE-CAV
ILISGRPLVVEPH-LPLMSAFVAAWLPGSG-GQGVADVLFGDYDFQGKSPRTWFKNVNQLPMNIGDQ------SYDPLFPYNFGL--
```
